# Genome-wide association analysis for feed efficiency in Angus cattle

**DOI:** 10.1111/j.1365-2052.2011.02273.x

**Published:** 2012-08

**Authors:** M M Rolf, J F Taylor, R D Schnabel, S D McKay, M C McClure, S L Northcutt, M S Kerley, R L Weaber

**Affiliations:** * Division of Animal Sciences, University of MissouriColumbia, MO 65211, USA; † American Angus AssociationSaint Joseph, MO 64506, USA

**Keywords:** beef cattle, BovineSNP50, feed efficiency, quantitative trait loci, single nucleotide polymorphism

## Abstract

Estimated breeding values for average daily feed intake (AFI; kg/day), residual feed intake (RFI; kg/day) and average daily gain (ADG; kg/day) were generated using a mixed linear model incorporating genomic relationships for 698 Angus steers genotyped with the Illumina BovineSNP50 assay. Association analyses of estimated breeding values (EBVs) were performed for 41 028 single nucleotide polymorphisms (SNPs), and permutation analysis was used to empirically establish the genome-wide significance threshold (*P* < 0.05) for each trait. SNPs significantly associated with each trait were used in a forward selection algorithm to identify genomic regions putatively harbouring genes with effects on each trait. A total of 53, 66 and 68 SNPs explained 54.12% (24.10%), 62.69% (29.85%) and 55.13% (26.54%) of the additive genetic variation (when accounting for the genomic relationships) in steer breeding values for AFI, RFI and ADG, respectively, within this population. Evaluation by pathway analysis revealed that many of these SNPs are in genomic regions that harbour genes with metabolic functions. The presence of genetic correlations between traits resulted in 13.2% of SNPs selected for AFI and 4.5% of SNPs selected for RFI also being selected for ADG in the analysis of breeding values. While our study identifies panels of SNPs significant for efficiency traits in our population, validation of all SNPs in independent populations will be necessary before commercialization.

## Introduction

Expected progeny differences (EPDs; EPDs are one-half the breeding value of an animal) have enabled beef cattle breeders to make rapid genetic progress in several economically important traits (i.e. AAA 2009) since their inception in 1974 (Willham 1993). However, most EPDs published by breed associations focus on output traits, especially weight traits, at marketing points in the production cycle, as well as meat quality and yield. While a number of breeds have developed evaluations for reproductive, docility and longevity traits, EPDs for input traits are lacking despite the fact that feed is estimated to comprise over 60% of the production cost in calf feeding systems and over 70% in finishing systems (Anderson *et al.* 2005).

The strong, positive phenotypic relationship between growth rate and gross feed efficiency has been exploited for at least the last 30 years through selection for increased growth rate, especially increased yearling weight. However, this approach to increasing feed efficiency has several negative consequences, including an increased mature cow size with a concomitant increase in maintenance energy costs for fast-growing, large-framed cattle (Archer *et al.* 1999; Okine *et al.* 2004). More recently, efforts to increase the metabolic efficiency of cattle have focused on selection to decrease feed intake independent of growth. One approach is to estimate residual feed intake (RFI) as the difference between observed and expected feed intake (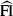), which is usually predicted from the regression of average daily feed intake (AFI) on average daily gain (ADG) and metabolic mid-weight (MMW; mid-weight^0.75^) as:


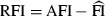



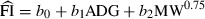


Animals with negative RFI are more efficient than expected given their growth and maintenance requirements (Archer *et al.* 1999). The appeal of this measure of efficiency is that RFI is phenotypically independent of the variables included in the regression, particularly growth rate (Koch *et al.* 1963). However, phenotypic independence does not assure genetic independence, and RFI can be genetically correlated with growth (Kennedy *et al.* 1993). A second approach to the improvement of efficiency is to select for decreased AFI relative to performance via the formulation of a selection index that includes output traits such as weight or growth rate (Herd *et al.* 2003). Selection for AFI and ADG in a selection index is equivalent to selection on RFI and ADG in a selection index, because the index weights would be determined so as to produce identical index values for the same animal; however, the relative economic value of AFI and ADG is inherently more obvious than for RFI (Kennedy *et al.* 1993).

A standardized approach to enable selection for improved feed efficiency has yet to be adopted by the beef industry. The primary limitation has been the inability of the industry to capture sufficient numbers of phenotypes to facilitate effective selection on large numbers of animals. One approach to more efficiently leverage the data already collected is to build genomic selection models and commercialize marker panels that effectively describe variation in efficiency traits with minimal phenotyping. The objectives of this study were to perform genome-wide association analyses to identify areas of the genome associated with AFI, RFI and ADG and to evaluate whether concordance between genomic regions for feed efficiency traits would make selection decisions and selection of markers for small panels more difficult.

## Materials and methods

### Population structure

A sample of 698 commercial Angus steers sired by 100 bulls originating from the Circle A Ranch (Huntsville, Stockton and Iberia, MO, USA) and MFA, Inc. (Thompson and Greenley, MO, USA) were individually fed a commercial feedlot ration using Calan gates (Circle A Ranch in Iberia) or GrowSafe feeding systems (MFA, Inc. fed at the University of Missouri). This population was further described in Rolf *et al.* (2010). Individual feed intake records including AFI and ADG during the feeding trial were used to estimate RFI for each steer within each feeding group. Weights were taken at the beginning, midpoint and end of the feeding trial, and animals were fed in groups of 96 between the years of 1998 and 2005 for an average of 110 days. Blood samples (10 ml) were collected on the last weigh date in vacuum tubes containing 15 mg of EDTA (Covidien) and transported on ice to the University of Missouri. All animal procedures were performed with approval from the University of Missouri Animal Care and Use Committee.

### Data acquisition

Genomic DNA was obtained from blood samples by proteinase-K digestion followed by phenol:chloroform:isoamyl alcohol extraction and ethanol precipitation (Sambrook *et al.* 1989). Data were collected for 54 001 SNP loci using the standard protocol for the Illumina BovineSNP50 BeadChip (Matukumalli *et al.* 2009). Genotypes were called using the Illumina BeadStudio software genotyping module 3.2.32. After filtering for minor allele frequency (MAF) ≥0.05 and call rate ≥95%, 41 028 SNPs remained with an average MAF of 0.28 and an average spacing of 65.73 ± 68.45 kb for 39 484 autosomal and 487 X chromosome loci. SNPs that mapped to unassigned contigs (ChrUn; *n* = 1057) were included in the analysis. Filtering was also applied to eliminate animals with >5% missing genotypes. FastPHASE (Scheet & Stephens 2006) and Btau4.0 positions with the T10-K20 options were used to impute the 0.58% of missing genotypes.

### Dependent variables

Breeding values for AFI, RFI and ADG were estimated using a mixed linear model in which trait observations were adjusted for the fixed effects of pen, year and season and which incorporated a genomic relationship matrix rather than a numerator relationship matrix to account for inferred pedigree structure (Rolf *et al.* 2010). The procedure used to compute the genomic relationship matrix was based on the calibration of allele sharing coefficients to numerator relationship coefficients (VanRaden 2008; Rolf *et al.* 2010). A single trait animal model was fitted using custom FORTRAN code, and variance components were iteratively estimated by restricted maximum likelihood until the heritability estimate had converged from above and below to the third significant figure. Due to the lack of maternal pedigree information, this implementation was shown to be considerably superior to the use of a numerator relationship matrix based upon pedigree data (Rolf *et al.* 2010). Sample statistics, variance component and heritability estimates for AFI, RFI and ADG were reported in Rolf *et al.* (2010), and correlations among estimated breeding values (EBVs) for pairs of traits are shown in Table 1.

**Table 1:** Correlations between estimated breeding values computed using a GRM for *N* = 698 Angus steers. *P*-values are listed below their corresponding correlation coefficients

	RFI	ADG
AFI	0.80	0.53
	<0.0001	<0.0001
RFI		0.04
		0.3113

### Genome-wide association analysis

Genome-wide association (GWA) analysis was performed on EBVs weighted by their corresponding accuracies, and permutation analysis (*N* = 10 000) was utilized to account for multiple hypothesis testing and reduce the reporting of spurious associations by controlling the type I error rate (Churchill & Doerge 1994). EBV weights were established by performing a Cholesky factorization of a diagonal matrix containing the EBV accuracies. To balance the analysis of variance equation, both the left-hand side (EBVs) and the right-hand side (genotypes) were multiplied by the weighting vector, similarly to Morsci *et al.* (2006). Permutation analysis was performed for each trait × model combination to estimate empirical genome-wide *P* < 0.05 thresholds. Code was developed and implemented in matlab (MathWorks, Natick, MA, USA) to perform the GWA analysis in three steps. First, *F*-statistics were formed for each SNP under an additive model to test the effect of each SNP and to identify the set of SNPs determined to be statistically significant for each trait × model combination. Second, this set of candidate SNPs was included in a forward selection analysis which was performed independently for each chromosome. In this analysis, the SNP with the highest *F* statistic was sequentially added to an additive effects model, and an association analysis was performed for the remaining SNPs on the chromosome until no additional SNPs on the chromosome reached the significance threshold determined by the permutation analysis. Finally, after a set of SNPs had been selected for each of the chromosomes by the forward selection algorithm, all selected SNPs were included into a final model to estimate the amount of variance explained by the selected SNPs in the data set (final model *R*^2^ values are reported in Table 2). Due to the limited number of animals available for analysis, the animals were not partitioned into training and validation sets. The logic underlying this analysis is that multiple SNPs within a region of a chromosome harbouring a QTL may provide strong signal, and thus, the total number of genome-wide SNPs associated with a trait provides an overestimate of the number of QTL that create variation in the trait. By sequentially including the SNPs with the strongest association in a forward selection analysis, we are attempting to identify the number and the locations of QTL affecting each trait.

**Table 2:** Numbers of SNPs exceeding the genome-wide *P* < 0.05 significance threshold and included in the final additive model for each analysis and percentages of EBV variance explained by the final suite of SNPs in each model computed using two different methods

Trait	Candidate SNPs	Final model SNPs	% EBV variance^1^	% GBLUP variance^2^
AFI	178	53	54.12	24.10
RFI	281	66	62.69	29.85
ADG	274	68	55.13	26.54

1 EBV variance is the model *R*^2^ from the final analysis which fits all SNPs in a fixed effects analysis.

2 GBLUP variance is the proportion of additive genetic variance explained by fitting all markers in a mixed model including a genomic relationship matrix for residual additive genetic effects.

Because of familial relationships between animals, a genomic BLUP (GBLUP) analysis was used as a secondary measure of the joint variance explained by all markers used in the final models. The GBLUP was implemented in FORTRAN using a similar procedure as described above. Two models were fitted, one which fitted only a fixed mean and the GRM for all animals to estimate the total additive genetic variance and a second that fitted SNP allele substitution effects as covariates along with the GRM to allow estimation of the residual additive genetic variance. The proportional differences between these variance estimates are reported in Table 2.

### Pathway analysis

To characterize the genomic regions identified by the forward selection analysis and to identify candidate genes influencing biological pathways related to the efficiency of growth, a pathway analysis was performed. Because of the more extensive functional annotation of the human genome and because only about 8% of bovine genes have no human othologs (The Bovine Genome Sequencing and Analysis Consortium *et al.* 2009), annotations from the human genome sequence (Feb. 2009) were mapped to the bovine genome sequence (Btau4.0) using the UCSC genome browser. Because of the extent of linkage disequilibrium in the bovine genome (McKay *et al.* 2007), 1-Mb windows (SNP position ± 0.5 Mb) surrounding each forward-selected SNP were queried against human annotations, Genbank accessions were extracted, and a pathway analysis was conducted using the Database for Annotation, Visualization and Integrated Discovery (DAVID) (Dennis *et al.* 2003; Huang *et al.* 2009). Pathways identified using DAVID and the KEGG pathway database (Kanehisa & Goto 2000; Kanehisa *et al.* 2006, 2010) were then researched using KEGG Atlas and summarized into global pathways and corresponding subcategories.

## Results

The correlation between ADG and RFI breeding values was not significant. AFI EBVs were more highly correlated with RFI EBVs than ADG EBVs. The number of significant candidate SNPs (genome-wide *P* < 0.05 correspond to *F* values of: AFI 23.7163; RFI 23.7750; ADG 23.7011) and the number of SNPs included in the final models for each analysis are shown in Table 2. The largest number of candidate SNPs did not always lead to the largest set of SNPs selected to be included in the final model. Table 2 also shows the percentage of variance explained by the full complement of SNPs selected to be included in the final model for each analysis from both the analysis of variance *R*^2^ (% EBV Variance) and GBLUP analyses. Additional information pertaining to the SNPs included in the final model for each trait can be found in Tables S1–S3. The SNPs included in the final model explained a relatively large portion of the variance in EBVs for all traits (AFI, 54.12%; RFI, 62.69%; ADG, 55.13%) when no attempt was made to account for relationships between animals. However, when estimated from the difference in additive genetic variance estimated between models that included the GRM, the amount of variance explained decreased to only 24–30% of the total additive genetic variance being explained by the selected SNPs. Figures S1–S3 show the GWA Manhattan plots for the analysis of EBVs for all feed efficiency traits. Additional information on the single-point associations for all SNPs represented in the Manhattan plots, including significance levels, is in Table S7.

To identify candidate genes influencing feed efficiency traits, 1-Mb regions centred on each forward-selected SNP were examined for concordance between traits (Table 3). From 5 to 45% of the regions detected to harbour QTL were common between pairs of traits. Genes within the 1-Mb regions predicted to harbour QTLs were identified and assembled into pathways using DAVID and the KEGG pathway database for each trait analysis, and the results are shown in Table 4. Many of the identified QTL regions appear to harbour genes whose functions are related to metabolic processes or to growth and the efficiency of energy utilization (100%, 74% and 64% of the pathways for AFI, RFI and ADG, respectively). Tables S4–S6 include more detail pertaining to this analysis including the individual genes found within each pathway. Because our sample was too small to separate into training and validation sets, the QTL regions identified for each trait were compared to the results of Barendse *et al.* (2007), Nkrumah *et al.* (2007) and Sherman *et al.* (2008) to determine whether concordant genomic regions had previously been identified in analyses of independent populations (Table 5). Seventeen SNPs were found to be concordant with previously published QTLs from both Barendse *et al.* (2007) and Nkrumah *et al.* (2007). No SNPs were found to be concordant with the study of Sherman *et al.* (2008).

**Table 3:** Number of concordant QTL regions for AFI, RFI and ADG. Diagonals represent the numbers of forward-selected SNPs for the trait. Off-diagonals represent the number of QTL regions detected to be concordant between traits defined as overlapping QTL regions (forward-selected SNP position ± 0.5 Mb).

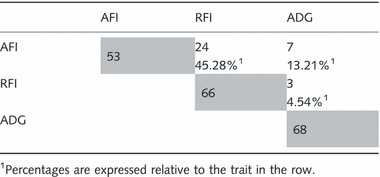

**Table 4:** Summary of pathway analysis from DAVID and the KEGG Pathway Database. Numbers in each trait column are the number of genes within each specific pathway

			Trait		
			
Global pathway	Subpathway	Pathway	ADG	AFI	RFI
Cellular processes	Cell Communication	Adherens junction			1
		Gap junction			2
		Tight junction			3
	Cell growth and death	Apoptosis		1	1
	Cell motility	Regulation of actin cytoskeleton	1		1
	Transport and catabolism	Endocytosis		2	
		Lysosome	3		
		Regulation of autophagy			1
Environmental information processing	Signal transduction	Calcium signalling pathway	1		3
		MAPK signalling pathway			1
		Notch signalling pathway	2		
		Phosphatidylinositol signalling system	1		
		TGF-beta signalling pathway		2	
		Wnt signalling pathway	1		
	Signalling molecules and interaction	Neuroactive ligand–receptor interaction	3		
Genetic information processing	Folding, sorting and degradation	Proteasome		1	
		RNA degradation		1	
		Ubiquitin-mediated proteolysis		2	
	Transcription	Spliceosome	1		
	Translation	Aminoacyl-tRNA biosynthesis		1	
Human Diseases	Cancers	Acute Myeloid Leukaemia			1
		Chronic Myeloid Leukaemia			1
		Pancreatic cancer			1
		Pathways in cancer		2	1
		Prostate cancer			1
		Small cell lung cancer			1
		Thyroid cancer		1	
	Cardiovascular diseases	Viral myocarditis			3
	Infectious diseases	Epithelial cell signalling in *Helicobacter pylori* infection			1
	Metabolic disorders	Type II diabetes mellitus			1
	Neurodegenerative diseases	Alzheimer's disease	2	1	1
		Huntington's disease		1	2
Metabolism	Amino acid metabolism	Phenylalanine metabolism			1
		Tyrosine metabolism			1
	Carbohydrate metabolism	Inositol phosphate metabolism	1		
	Energy metabolism				
	Glycan biosynthesis and metabolism	Keratan Sulphate Biosynthesis			1
		N-Glycan biosynthesis			1
		O-Glycan biosynthesis	2		
	Lipid metabolism	Ether lipid metabolism			1
		Glycerolipid metabolism			1
		Glycerophospholipid metabolism			1
	Metabolism of Terpenoids and Polyketides	Limonene and piene degradation			1
	Nucleotide metabolism	Purine metabolism		1	
		Pyrimidine metabolism		1	
Organismal Systems	Circulatory system	Vascular smooth muscle contraction			3
	Development				
	Endocrine system	Adipocytokine signalling pathway			1
		GnRH signalling pathway			2
		Insulin signalling pathway			1
		Melanogenesis			1
	Immune system	B cell receptor signalling pathway			1
		Chemokine signalling pathway		1	2
		Cytosolic DNA-sensing pathway			1
		Fc gamma R-mediated phagocytosis	1		
		NOD-like receptor signalling pathway			1
		RIG-I-like receptor signalling pathway		1	1
		T cell receptor signalling pathway			1
		Toll-like receptor signalling pathway		1	1
	Nervous system	Long-term depression	1		2
		Long-term potentiation			1
		Neurotrophin signalling pathway	1		1

**Table 5:** Comparison of results from this study to previously published feed efficiency QTL studies. Concordance was established if a forward-selected SNP was within ±0.5 Mb of a previously published QTL position

Trait	SNP ID	Chr	Position (Mb)^1^	Validation position (Mb)^2^
AFI	ss86278343	11	5.660631	5.205392^3^
	ss117964737	14	70.09761	70.53168^4^
	ss86295351	15	61.349658	60.933014^3^
	ss61538007	17	29.240631	28.901^4^
	ss86318895	19	49.810291	49.675^4^
	ss86339752	21	31.529569	31.455^4^
RFI	ss61489474	3	7.649578	7.401859^4^
	ss86274086	5	35.900142	36.02461^4^
	ss86290408	6	105.402482	105.500454^3^
	ss86341687	12	72.395434	72.4973^4^
	ss86295351	15	61.349658	60.933014^3^
	ss61538007	17	29.240631	28.901^4^
	ss86303118	21	30.983757	31.455^4^
ADG	ss86335501	5	33.048112	33.304^4^
	ss86298158	17	12.72016	12.58837^4^
	ss86289007	24	4.017728	4.153065^4^
	ss61547771	24	52.914946	52.746^4^

1 Chromosomal positions are Btau4.0 coordinates

2 Validation positions are taken from the published coordinates (or SNP IDs referenced in publicly accessible databases such as dbSNP) mapped to the Btau4.0 assembly. Where no IDs or genomic positions were given, but a linkage map was referenced, positions correspond to that of the closest marker in the referenced map identified by BLAST and BLAT searches of published primer sequences.

3 Barendse *et al.* (2007);

4 Nkrumah *et al.* (2007).

## Discussion

We identified SNPs that could be included in commercial marker panels for genomic selection or marker-assisted management of feed efficiency traits in Angus cattle (Tables S1–S3). These SNPs explained large amounts of variation (54.12–62.69%) in the feed efficiency-related traits examined in this population. However, these estimates are likely to be biased upward by the selection of the most strongly associated SNPs detected in the sample, which may not necessarily be the most strongly associated with feed efficiency traits in the Angus population. Subsequent joint estimation of the SNP effects using a GBLUP revealed that the proportion of additive genetic variance explained by the selected markers was approximately 50% lower than the anova estimates. Independent validation of SNP associations and of the amount of variation explained by the SNPs will be required prior to their commercial use. In view of the low estimates of heritability found in this sample, we considered the sample size to be too small to attempt a partitioning into training and validation datasets. To validate SNP associations, we compared our results to those from other published GWA studies of feed efficiency (Table 5). Seventeen SNP associations from two independent populations (Barendse *et al.* 2007; Nkrumah *et al.* 2007) were validated using this approach. Association analyses are notorious for spurious associations leading to false positives, so it is imperative that the SNPs that failed to be validated be tested in an independent population to verify that they are not population-specific or spurious associations.

Figures S1–S3 show Manhattan plots for the GWA analyses, and Table 3 contains the number of QTL regions detected by forward selection of significantly associated SNPs that were associated with pairs of traits. Of particular interest is the low concordance between genomic regions harbouring RFI QTL that also harbour ADG QTL (4.5% of RFI regions). On the other hand, 13.2% of the detected QTL regions were concordant for AFI and ADG, indicating that it should be possible to identify QTL for feed intake that are independent of growth and that are associated only with the efficiency of growth. The existence of these QTL indicates that selection for improved feed efficiency could be accomplished without necessarily inducing a correlated response in growth rate or mature size. We identified QTL regions associated with AFI and RFI that were not associated with ADG (AFI: 46; RFI 63). After validation in independent populations, use of associated SNPs within these QTL regions may be useful in reduced marker panel applications for estimating molecular breeding values and genomic-enhanced EPDs. The inclusion of SNPs that have been shown to be associated with feed intake and efficiency, but not growth, will be important to allow selection for increased feed efficiency without inducing correlated responses in growth or mature size.

In addition to large numbers of animals and SNPs evenly distributed throughout the genome, the ability to correct field data for fixed effects is important in livestock GWA studies in order to remove systematic bias and obtain the highest-quality phenotypes for analysis. Even after this adjustment, the estimated heritabilities (AFI 0.14; RFI 0.14; ADG 0.09) for these traits were much lower than literature estimates (AFI 0.45; RFI 0.39; ADG 0.28; MacNeil *et al.* 1991; Arthur *et al.* 2001), possibly due to the relatively small number of sampled animals or possibly because these steers may have been selected to be individually fed based upon their weaning performance. Whether this is the case is unknown, but such selection would also be expected to result in a skewing of allele frequencies for QTL underlying the selected traits that may influence their ability to be detected in the GWA analysis.

To further investigate QTLs in genomic regions which differed from those previously reported, we performed a pathway analysis to identify regions that harbour genes with growth or metabolic functions. After separation into functional groups using KEGG Atlas, categories with importance to growth or metabolism, such as cell growth and death, cancer (aberrant growth of cells) and metabolic disorders, were identified. Other categories such as signal transduction were also considered, because of their involvement in energy transportation or energy-requiring processes (Table 4). Many of the detected QTL regions harbour genes whose functions are related to metabolic processes or to growth and the efficiency of energy utilization, which further supports the integrity of the identified SNPs as candidates for validation. This information also provides candidate genes for the discovery of causal mutations underlying feed efficiency QTL.

This study has identified BovineSNP50 SNPs that are associated with variation in feed efficiency and are validated by previous independent studies. Other associated SNPs that could not be validated by the literature should be tested in independent populations prior to the development of commercial assays. Selection indices incorporating molecular breeding values, for example, for AFI, ADG, carcass traits and mature size, should be used to appropriately select for increased profitability while constraining undesirable correlated responses on mature size.

## Supporting information

**Figure S1** Genome-wide association analysis for AFI.

**Figure S2** Genome-wide association analysis for RFI.

**Figure S3** Genome-wide association analysis for ADG.

**Table S1** ADG SNPs included in the final forward selection model.

**Table S2** AFI SNPs included in the final forward selection model.

**Table S3** RFI SNPs included in the final forward selection model.

**Table S4** Pathway analysis of genomic regions detected as harboring QTLs in the analysis of ADG.

**Table S5** Pathway analysis of genomic regions detected as harboring QTLs in the analysis of AFI.

**Table S6** Pathway analysis of genomic regions detected as harboring QTLs in the analysis of RFI.

**Table S7** Results from single marker association analysis for all SNPs.

As a service to our authors and readers, this journal provides supporting information supplied by the authors. Such materials are peer-reviewed and may be re-organized for online delivery, but are not copy-edited or typeset. Technical support issues arising from supporting information (other than missing files) should be addressed to the authors.
