# Supplementary material for: Genome-wide association analysis for feed efficiency in Angus cattle
Source: Anim Genet. 2012 Aug;43(4):367–74. doi: 10.1111/j.1365-2052.2011.02273.x (PMC3437496; doi:10.1111/j.1365-2052.2011.02273.x)
Supplement: Supplementary file 2 [file age0043-0367-SD2.pdf]

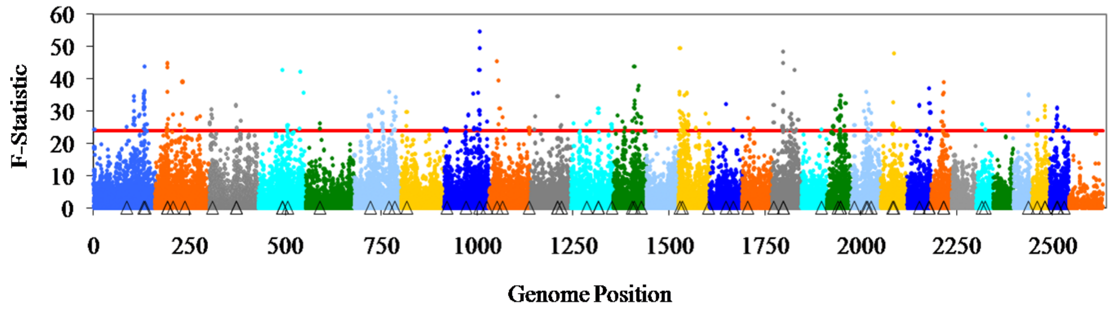

Figure S2: Genome-wide association analysis for RFI. The red line indicates the significance threshold for the analysis (F>23.7750, genome-wide p<0.05) determined by permutation analysis (N = 10,000). Black triangles represent the positions of SNPs included in the final forward selection model.
