# Supplementary material for: Genome-wide association analysis for feed efficiency in Angus cattle
Source: Anim Genet. 2012 Aug;43(4):367–74. doi: 10.1111/j.1365-2052.2011.02273.x (PMC3437496; doi:10.1111/j.1365-2052.2011.02273.x)
Supplement: Supplementary file 3 [file age0043-0367-SD3.pdf]

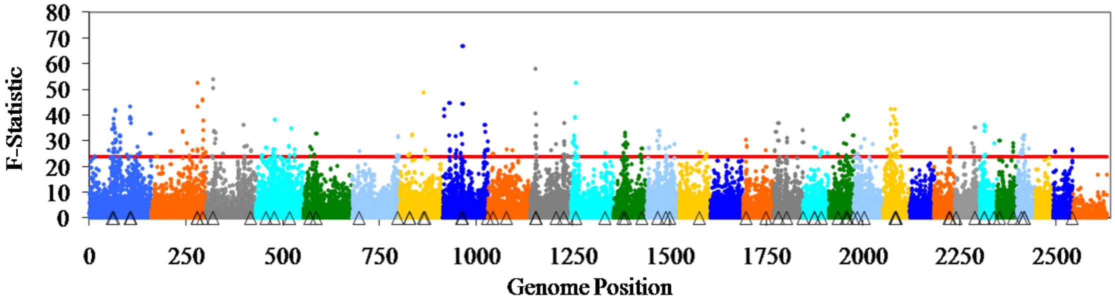

Figure S3: Genome-wide association analysis for ADG. The red line indicates the significance threshold for the analysis ($F>23.7011$, genome-wide $p<0.05$) determined by permutation analysis ($N = 10,000$). Black triangles represent the positions of SNPs included in the final forward-selection model.
