# Supplementary material for: Genome-wide association analysis for feed efficiency in Angus cattle
Source: Anim Genet. 2012 Aug;43(4):367–74. doi: 10.1111/j.1365-2052.2011.02273.x (PMC3437496; doi:10.1111/j.1365-2052.2011.02273.x)
Supplement: Supplementary file 4 [file age0043-0367-SD4.pdf]

Table S1: ADG SNPs included in the final forward selection model. BTA and position denote the chromosome and chromosomal position from the Btau4.0 assembly, respectively. Abs(α) denotes the absolute value of the allele substitution effect. $2pq\alpha^2$ describes the genetic variance for each locus where the allele frequencies are p and q=1-p.

Marker ID	BTA	Position (Mb)	Abs(α)	p	$2pq\alpha^2$
ss86298222	1	59.853273	0.0071	0.1060	0.0000095555
ss63968561	1	63.570304	0.0006	0.5007	0.0000001800
ss61531716	1	105.829263	0.0022	0.3696	0.0000022555
ss65172344	1	108.073679	0.0018	0.3746	0.0000015182
ss86290982	2	118.468144	0.0018	0.4513	0.0000016046
ss86298054	2	118.498847	0.0051	0.4778	0.0000129793
ss86320968	2	132.791346	0.0019	0.5315	0.0000017978
rs29015763	3	18.495143	0.0042	0.7049	0.0000073392
ss86318343	3	115.720543	0.0018	0.4420	0.0000015982
ss64165385	4	27.465876	0.0078	0.4699	0.0000303099
ss86333174	4	48.998895	0.0017	0.0709	0.0000003808
ss86293686	4	88.599955	0.0053	0.5287	0.0000139989
ss61469518	5	16.957757	0.0052	0.8539	0.0000067480
ss86335501	5	33.048111	0.0027	0.3474	0.0000033056
ss61469484	6	18.368731	0.019	0.0559	0.0000380870
ss86307817	6	118.120363	0.006	0.6719	0.0000158719
ss117968894	7	26.449891	0.0046	0.7457	0.0000080252
ss65439566	7	62.064599	0.0106	0.8625	0.0000266562
rs29012520	7	65.978304	0.0004	0.9011	0.0000000285
ss86332207	8	48.680422	0.0027	0.7915	0.0000024057
ss86317861	8	51.089227	0.0068	0.5007	0.0000231200
ss86275977	8	115.607565	0.0093	0.5451	0.0000428927
ss86317257	9	12.902431	0.0049	0.7615	0.0000087223
ss86302682	9	47.588815	0.0036	0.1332	0.0000029934
ss86290351	10	15.067096	0.0045	0.3696	0.0000094366
ss86338016	10	16.437278	0.0039	0.4692	0.0000075761
rs29010337	10	68.029461	0.0046	0.5903	0.0000102352
ss61558932	10	87.622309	0.0032	0.2894	0.0000042116
ss86327397	11	12.784299	0.0069	0.1232	0.0000102865
ss86281383	11	88.002621	0.0061	0.7092	0.0000153490
ss61521197	12	24.477221	0.0033	0.2844	0.0000044324
ss86340783	12	30.270048	0.0013	0.4878	0.0000008445
ss86318494	12	73.030843	0.002	0.6168	0.0000018909
ss86307175	13	28.958202	0.0003	0.4083	0.0000000435
ss86339064	13	50.13714	0.0033	0.8309	0.0000030595
ss86325195	13	59.746078	0.0044	0.7027	0.0000080888
ss86311856	14	52.350311	0.0086	0.1920	0.0000229456

ss63983181	16	7.036933	0.0024	0.1433	0.0000014140
ss86329933	16	58.404713	0.0024	0.5974	0.0000027707
ss86298158	17	12.720105	0.0057	0.1433	0.0000079757
ss62643327	17	34.22045	0.0017	0.6705	0.0000012770
ss86303324	17	76.421554	0.0089	0.1497	0.0000201668
ss117972449	18	29.600313	0.0024	0.6662	0.0000025618
ss86303954	18	47.210624	0.0003	0.5387	0.0000000447
ss105259813	19	24.301922	0.0129	0.0938	0.0000283009
ss86283697	19	45.340598	0.0046	0.2063	0.0000069296
ss117965335	19	48.738154	0.0058	0.6139	0.0000159472
ss117965434	19	63.651366	0.0074	0.5967	0.0000263558
ss62617158	20	8.810833	0.0056	0.6261	0.0000146831
ss61543085	20	26.966402	0.0011	0.6311	0.0000005634
ss86336107	21	30.346882	0.0034	0.3259	0.0000050795
ss64618188	21	32.67505	0.0148	0.0723	0.0000294018
ss62727144	21	34.018769	0.0007	0.2407	0.0000001791
ss117965571	23	39.450899	0.0031	0.6032	0.0000046005
rs29018032	23	41.988126	0.0021	0.8632	0.0000010416
ss86289007	24	4.017729	0.0035	0.1332	0.0000028294
ss61547771	24	52.914945	0.0058	0.4914	0.0000168150
ss117973804	25	12.283689	0.003	0.6318	0.0000041873
ss86284393	25	37.212719	0.0032	0.4828	0.0000051139
ss86312150	26	7.796869	0.0064	0.8016	0.0000130295
ss46526045	26	43.529109	0.0037	0.5315	0.0000068178
ss86290736	27	12.383786	0.005	0.8660	0.0000058005
ss61470138	27	19.790331	0.0006	0.3782	0.0000001693
ss86295347	29	48.856592	<0.0000	0.9033	0.0000000000
ss86320739	Un		0.0015	0.5222	0.0000011228
ss117974825	Un		0.0009	0.4291	0.0000003969
ss63781516	Un		0.0044	0.7808	0.0000066269
ss65169323	Un		0.0071	0.5444	0.0000250061
