# Supplementary material for: Genome-wide association analysis for feed efficiency in Angus cattle
Source: Anim Genet. 2012 Aug;43(4):367–74. doi: 10.1111/j.1365-2052.2011.02273.x (PMC3437496; doi:10.1111/j.1365-2052.2011.02273.x)
Supplement: Supplementary file 5 [file age0043-0367-SD5.pdf]

Table S2: AFI SNPs included in the final forward selection model. BTA and position denote the chromosome and chromosomal position from the Btau4.0 assembly, respectively. Abs(α) denotes the absolute value of the allele substitution effect. $2pq\alpha^2$ describes the genetic variance for each locus where the allele frequencies are p and q=1-p.

Marker ID	BTA	Position (Mb)	Abs(α)	p	$2pq\alpha^2$
ss61531716	1	105.8293	0.0177	0.3696	0.0001458301
ss105242396	1	113.8799	0.0434	0.4219	0.0009207200
ss105254260	2	31.07949	0.0346	0.6870	0.0005137263
ss86316304	2	67.08311	0.0594	0.4011	0.0016969339
rs29015763	3	18.49514	0.0128	0.7049	0.0000676351
ss86341694	3	23.35313	0.0682	0.2600	0.0017904500
ss61517428	3	70.78155	0.0089	0.1447	0.0000195622
ss86331253	4	62.29809	0.0379	0.5595	0.0007065932
ss86297489	6	90.41552	0.0042	0.5215	0.0000089004
ss86336887	6	106.3529	0.0664	0.2085	0.0014558350
ss64249818	7	32.4503	0.0137	0.3789	0.0000884855
ss86335346	8	91.54707	0.0044	0.6934	0.0000082466
rs29022959	8	91.71922	0.016	0.7213	0.0001026578
ss117969435	8	97.33553	0.032	0.8818	0.0002131990
ss61493344	9	78.2669	0.0373	0.6368	0.0006444193
ss86278343	11	5.66063	0.0326	0.0831	0.0001623506
ss86293568	11	39.35181	0.0182	0.4097	0.0001609282
ss117970126	11	40.35811	0.0495	0.1218	0.0005230355
ss86289943	11	78.30539	0.0299	0.7729	0.0003138842
rs29014508	12	23.42551	0.0139	0.3489	0.0000876509
ss86274979	12	32.53872	0.0079	0.8546	0.0000156294
ss61516394	12	48.75502	0.0574	0.3274	0.0014530141
ss65270148	12	61.90864	0.0099	0.1827	0.0000290295
ss86330336	14	14.98921	0.0374	0.3782	0.0006580700
ss117964737	14	70.09761	0.0052	0.4986	0.0000136554
ss86295351	15	61.34966	0.0413	0.5845	0.0008292738
ss86277466	15	78.10219	0.0317	0.9226	0.0001430937
ss117972192	17	4.094041	0.0358	0.6132	0.0006066270
ss61467852	17	27.83249	0.0274	0.6053	0.0003598838
ss61538007	17	29.24063	0.0198	0.7285	0.0001555952
ss117965075	18	52.35454	0.0445	0.0953	0.0003418681
ss61540370	19	29.93895	0.026	0.4362	0.0003327094
ss86328551	19	36.05319	0.0292	0.1060	0.0001618659
ss117965335	19	48.73815	0.0352	0.6139	0.0005873731
ss86318895	19	49.81029	0.0141	0.2299	0.0000702066
ss62644539	20	43.75955	0.007	0.6648	0.0000219648
ss86339752	21	31.52957	0.0415	0.8660	0.0003988280

ss86277953	21	33.92316	0.037	0.8875	0.0002738874
ss61545215	22	25.57142	0.0631	0.6519	0.0018042927
ss61523365	22	31.36968	0.0285	0.4599	0.0004035109
ss61494684	23	32.16787	0.035	0.2894	0.0005038349
ss86341078	23	38.86096	0.0354	0.2615	0.0004845156
rs29019360	24	62.80138	0.0384	0.8116	0.0004508341
ss61550290	27	13.17559	0.0103	0.7744	0.0000372182
ss86301648	28	14.75985	0.0319	0.2550	0.0003862190
ss86331294	28	34.27463	0.0401	0.6375	0.0007439119
ss86329884	29	18.94255	0.0556	0.5723	0.0015132079
ss86291606	29	41.28812	0.0601	0.8438	0.0009528913
ss86323038	29	48.72363	0.0047	0.3739	0.0000102112
ss65170459	Un		0.0024	0.2149	0.0000019275
ss86331954	Un		0.0234	0.7342	0.0002132906
ss86275593	Un		0.0127	0.2701	0.0000639601
ss117974956	Un		0.036	0.5974	0.0006228456
