# Supplementary material for: Genome-wide association analysis for feed efficiency in Angus cattle
Source: Anim Genet. 2012 Aug;43(4):367–74. doi: 10.1111/j.1365-2052.2011.02273.x (PMC3437496; doi:10.1111/j.1365-2052.2011.02273.x)
Supplement: Supplementary file 6 [file age0043-0367-SD6.pdf]

Table S3: RFI SNPs included in the final forward selection model. BTA and position denote the chromosome and chromosomal position from the Btau4.0 assembly, respectively. Abs(α) denotes the absolute value of the allele substitution effect. $2pq\alpha^2$ describes the genetic variance for each locus where the allele frequencies are p and q=1-p.

| Marker ID | BTA | Position (Mb) | Abs(α) | p | $2pq\alpha^2$ |
|---|---|---|---|---|---|
| *ss64260054* | 1 | 85.95453 | 0.0185 | 0.1683 | 0.0000959028 |
| *ss86328736* | 1 | 130.3111 | 0.046 | 0.2221 | 0.0007302880 |
| *ss105301409* | 1 | 133.5587 | 0.0261 | 0.1676 | 0.0001899456 |
| *ss117966494* | 2 | 31.05061 | 0.1186 | 0.3109 | 0.0060264845 |
| *ss105254260* | 2 | 31.07949 | 0.1426 | 0.6870 | 0.0087506812 |
| *ss61484491* | 2 | 45.422 | 0.068 | 0.8474 | 0.0011943462 |
| *ss86293216* | 2 | 76.35988 | 0.0433 | 0.6103 | 0.0008901653 |
| *ss61489474* | 3 | 7.649577 | 0.0084 | 0.2751 | 0.0000281403 |
| *ss64836693* | 3 | 70.03582 | 0.034 | 0.8847 | 0.0002361678 |
| *ss86331253* | 4 | 62.29809 | 0.0213 | 0.5595 | 0.0002242468 |
| *ss64726870* | 4 | 75.92411 | 0.0214 | 0.4312 | 0.0002245856 |
| *ss86274086* | 5 | 35.90014 | 0.0189 | 0.2113 | 0.0001188279 |
| *ss61557043* | 6 | 41.86704 | 0.0289 | 0.4341 | 0.0004112025 |
| *ss86297489* | 6 | 90.41552 | 0.0051 | 0.5215 | 0.0000127277 |
| *ss86290408* | 6 | 105.4025 | 0.0131 | 0.1812 | 0.0000507740 |
| *ss86293830* | 7 | 14.27555 | 0.0066 | 0.3080 | 0.0000187780 |
| *ss86337575* | 8 | 6.769905 | 0.0417 | 0.8009 | 0.0005549146 |
| *ss86339705* | 8 | 56.62185 | 0.0249 | 0.5014 | 0.0003088333 |
| *rs29022959* | 8 | 91.71922 | 0.0251 | 0.7213 | 0.0002524645 |
| *ss86308369* | 8 | 110.8797 | 0.0375 | 0.7385 | 0.0005416445 |
| *ss64696603* | 9 | 20.70463 | 0.0103 | 0.4405 | 0.0000527223 |
| *ss86328884* | 9 | 34.81401 | 0.0474 | 0.0645 | 0.0002705972 |
| *ss61516025* | 9 | 104.334 | 0.0171 | 0.2414 | 0.0001072975 |
| *ss86327686* | 10 | 70.40733 | 0.0269 | 0.6454 | 0.0003312026 |
| *ss61491345* | 10 | 80.49974 | 0.0022 | 0.6354 | 0.0000022528 |
| *ss117970126* | 11 | 40.35811 | 0.0359 | 0.1218 | 0.0002758223 |
| *ss86303685* | 11 | 70.65666 | 0.042 | 0.6418 | 0.0008121868 |
| *ss86336779* | 11 | 105.8802 | 0.0274 | 0.3109 | 0.0003222445 |
| *ss61516394* | 12 | 48.75502 | 0.0084 | 0.3274 | 0.0000310742 |
| *ss61529532* | 12 | 53.97856 | 0.0215 | 0.1519 | 0.0001187438 |
| *ss86341687* | 12 | 72.39544 | 0.0512 | 0.8023 | 0.0008314600 |
| *ss117971065* | 14 | 3.606692 | 0.0037 | 0.5745 | 0.0000065778 |
| *ss86340984* | 14 | 9.990357 | 0.0224 | 0.8188 | 0.0001490423 |
| *ss86300474* | 14 | 78.08375 | 0.03 | 0.4140 | 0.0004353324 |
| *ss62125948* | 15 | 42.20598 | 0.0147 | 0.7034 | 0.0000898396 |
| *ss86295351* | 15 | 61.34966 | 0.0096 | 0.5845 | 0.0000444837 |
| *ss61565643* | 16 | 14.41611 | 0.0071 | 0.8861 | 0.0000102154 |

| | | | | | |
|---|---|---|---|---|---|
| *ss117972192* | 17 | 4.094041 | 0.0347 | 0.6132 | 0.0005725143 |
| *ss61538007* | 17 | 29.24063 | 0.0263 | 0.7285 | 0.0002731933 |
| *ss117972116* | 17 | 29.40406 | 0.0123 | 0.5709 | 0.0000736419 |
| *ss117965075* | 18 | 52.35454 | 0.0371 | 0.0953 | 0.0002375236 |
| *ss86311733* | 19 | 29.25672 | 0.0466 | 0.8782 | 0.0004634874 |
| *ss86329372* | 19 | 35.72306 | 0.0081 | 0.4140 | 0.0000318354 |
| *ss86328551* | 19 | 36.05319 | 0.0233 | 0.1060 | 0.0001031374 |
| *ss65376194* | 20 | 6.746413 | 0.0168 | 0.3617 | 0.0001303928 |
| *ss86314901* | 20 | 37.78467 | 0.022 | 0.5287 | 0.0002415123 |
| *ss61495644* | 20 | 41.21733 | 0.0141 | 0.7622 | 0.0000725756 |
| *ss61511525* | 20 | 50.48725 | 0.0288 | 0.8510 | 0.0002098884 |
| *ss86303118* | 21 | 30.98376 | 0.0234 | 0.3231 | 0.0002394147 |
| *ss86277953* | 21 | 33.92316 | 0.0326 | 0.8875 | 0.0002119006 |
| *ss61523365* | 22 | 31.36968 | 0.0022 | 0.4599 | 0.0000024044 |
| *ss86295370* | 22 | 56.05081 | 0.0246 | 0.5509 | 0.0002997658 |
| *rs29013532* | 22 | 57.36246 | 0.0084 | 0.3181 | 0.0000308784 |
| *ss86316255* | 23 | 32.67781 | 0.0466 | 0.0910 | 0.0003597838 |
| *ss61514878* | 23 | 33.238 | 0.0091 | 0.4986 | 0.0000412229 |
| *ss86330971* | 25 | 14.73887 | 0.0294 | 0.2414 | 0.0003158454 |
| *ss61486991* | 25 | 23.12439 | 0.0172 | 0.6554 | 0.0001341521 |
| *ss86274817* | 27 | 40.08445 | 0.0039 | 0.5193 | 0.0000076717 |
| *ss86321221* | 28 | 14.30796 | 0.0569 | 0.1282 | 0.0007240181 |
| *ss86289932* | 28 | 34.60799 | 0.0468 | 0.8961 | 0.0004068625 |
| *ss86294990* | 29 | 19.5054 | 0.0291 | 0.7958 | 0.0002753421 |
| *ss86328651* | 29 | 21.49747 | 0.0278 | 0.6626 | 0.0003457990 |
| *ss117965798* | 29 | 38.62682 | 0.0296 | 0.2185 | 0.0002981939 |
| *ss86319047* | Un | | 0.001 | 0.4076 | 0.0000005193 |
| *ss65170459* | Un | | 0.0142 | 0.2149 | 0.0000679447 |
| *ss86331954* | Un | | 0.0231 | 0.7342 | 0.0002089518 |
