# Supplementary material for: Genome-wide association analysis for feed efficiency in Angus cattle
Source: Anim Genet. 2012 Aug;43(4):367–74. doi: 10.1111/j.1365-2052.2011.02273.x (PMC3437496; doi:10.1111/j.1365-2052.2011.02273.x)
Supplement: Supplementary file 7 [file age0043-0367-SD7.pdf]

Table S4: Pathway analysis of genomic regions detected as harboring QTLs in the analysis of ADG. Regions were defined as SNP position (from the final model) ±0.5 Mb. Human annotations were mapped to bovine coordinates and genes were identified that spanned this region and analyzed in DAVID using the KEGG Pathway database.

Pathway	Accession	Genes
Alzheimer's disease	NM_000021	presenilin 1
	NM_000447, NM_012486	presenilin 2 (Alzheimer disease 4)
Calcium signaling pathway	J05200, NM_000540	ryanodine receptor 1 (skeletal)
Fc gamma R-mediated phagocytosis	NM_198252, NM_000177	gelsolin (amyloidosis, Finnish type)
Inositol phosphate metabolism	BC062317	inositol polyphosphate-5-phosphatase, 75kDa
Long-term depression	J05200, NM_000540	ryanodine receptor 1 (skeletal)
Lysosome	NM_001283, CR599373	adaptor-related protein complex 1, sigma 1 subunit
	NM_003916	adaptor-related protein complex 1, sigma 2 subunit pseudogene; adaptor-related protein complex 1, sigma 2 subunit
	BC009606, BC021898	adaptor-related protein complex 1, sigma 3 subunit
Neuroactive ligand-receptor interaction	NM_000830	glutamate receptor, ionotropic, kainate 1
	NM_175768, AJ252246, NM_021956, BC037954, BC063814	glutamate receptor, ionotropic, kainate 2
	AJ249210, NM_000831	glutamate receptor, ionotropic, kainate 3
Neurotrophin signaling pathway	NM_000021	presenilin 1
Notch signaling pathway	NM_000021	presenilin 1
	NM_000447, NM_012486	presenilin 2 (Alzheimer disease 4)
O-Glycan biosynthesis	NM_198321, AK023782	UDP-N-acetyl-alpha-D-galactosamine:polypeptide N-acetylgalactosaminyltransferase 10 (GalNAc-T10)
	NM_001034845	UDP-N-acetyl-alpha-D-galactosamine:polypeptide N-acetylgalactosaminyltransferase-like 6
Phosphatidylinositol signaling system	BC062317	inositol polyphosphate-5-phosphatase, 75kDa
Regulation of actin cytoskeleton	NM_198252, NM_000177	gelsolin (amyloidosis, Finnish type)
Spliceosome	NM_004941, BC044586	DEAH (Asp-Glu-Ala-His) box polypeptide 8
Wnt signaling pathway	NM_000021	presenilin 1
