# Supplementary material for: Genome-wide association analysis for feed efficiency in Angus cattle
Source: Anim Genet. 2012 Aug;43(4):367–74. doi: 10.1111/j.1365-2052.2011.02273.x (PMC3437496; doi:10.1111/j.1365-2052.2011.02273.x)
Supplement: Supplementary file 8 [file age0043-0367-SD8.pdf]

Table S5: Pathway analysis of genomic regions detected as harboring QTLs in the analysis of AFI. Regions were defined as SNP position (from the final model) ±0.5 Mb. Human annotations were mapped to bovine coordinates and genes were identified that spanned this region and analyzed in DAVID using the KEGG Pathway database.

Pathway	Accession	Genes
Alzheimer's disease	NM_003824	Fas (TNFRSF6)-associated via death domain
Aminoacyl-tRNA biosynthesis	NM_024678	asparaginyl-tRNA synthetase 2, mitochondrial (putative)
Apoptosis	NM_003824	Fas (TNFRSF6)-associated via death domain
Chemokine signaling pathway	NM_014800, AK126565, AL136787	engulfment and cell motility 1
Endocytosis	NM_022739	SMAD specific E3 ubiquitin protein ligase 2
	NM_020429	SMAD specific E3 ubiquitin protein ligase 1
Huntington's disease	NM_015477	SIN3 homolog A, transcription regulator (yeast)
Pathways in cancer	NM_003824	Fas (TNFRSF6)-associated via death domain
	NM_005436	coiled-coil domain containing 6
Proteasome	NM_002804, BC106920	proteasome (prosome, macropain) 26S subunit, ATPase, 3
Purine metabolism	NM_033109	polyribonucleotide nucleotidyltransferase 1
Pyrimidine metabolism	NM_033109	polyribonucleotide nucleotidyltransferase 1
RIG-I-like receptor signaling pathway	NM_003824	Fas (TNFRSF6)-associated via death domain
RNA degradation	NM_033109	polyribonucleotide nucleotidyltransferase 1
TGF-beta signaling pathway	NM_020429	SMAD specific E3 ubiquitin protein ligase 1
	NM_022739	SMAD specific E3 ubiquitin protein ligase 2
Thyroid cancer	NM_005436	coiled-coil domain containing 6
Toll-like receptor signaling pathway	NM_003824	Fas (TNFRSF6)-associated via death domain
Ubiquitin mediated proteolysis	NM_020429	SMAD specific E3 ubiquitin protein ligase 1
	NM_022739	SMAD specific E3 ubiquitin protein ligase 2
