# Supplementary material for: Genome-wide association analysis for feed efficiency in Angus cattle
Source: Anim Genet. 2012 Aug;43(4):367–74. doi: 10.1111/j.1365-2052.2011.02273.x (PMC3437496; doi:10.1111/j.1365-2052.2011.02273.x)
Supplement: Supplementary file 9 [file age0043-0367-SD9.pdf]

Table S6: Pathway analysis of genomic regions detected as harboring QTLs in the analysis of RFI. Regions were defined as SNP position (from the final model) ±0.5 Mb. Human annotations were mapped to bovine coordinates and genes were identified that spanned this region and analyzed in DAVID using the KEGG Pathway database.

Pathway	Accession	Genes
Acute myeloid leukemia	NM_001556, BC108694	inhibitor of kappa light polypeptide gene enhancer in B-cells, kinase beta
Adherens junction	BC021301, NM_014808	FERM, RhoGEF and pleckstrin domain protein 2
Adipocytokine signaling pathway	NM_001556, BC108694	inhibitor of kappa light polypeptide gene enhancer in B-cells, kinase beta
Alzheimer's disease	NM_002072, BC057777	guanine nucleotide binding protein (G protein), q polypeptide
Apoptosis	NM_001556, BC108694	inhibitor of kappa light polypeptide gene enhancer in B-cells, kinase beta
B cell receptor signaling pathway	NM_001556, BC108694	inhibitor of kappa light polypeptide gene enhancer in B-cells, kinase beta
Calcium signaling pathway	NM_002067	guanine nucleotide binding protein (G protein), alpha 11 (Gq class)
	NM_004297	guanine nucleotide binding protein (G protein), alpha 14
	NM_002072, BC057777	guanine nucleotide binding protein (G protein), q polypeptide
Chemokine signaling pathway	AL136787, NM_014800, AK126565	engulfment and cell motility 1
	NM_001556, BC108694	inhibitor of kappa light polypeptide gene enhancer in B-cells, kinase beta
Chronic myeloid leukemia	NM_001556, BC108694	inhibitor of kappa light polypeptide gene enhancer in B-cells, kinase beta
Cytosolic DNA-sensing pathway	NM_001556, BC108694	inhibitor of kappa light polypeptide gene enhancer in B-cells, kinase beta
Epithelial cell signaling in Helicobacter pylori infection	NM_001556, BC108694	inhibitor of kappa light polypeptide gene enhancer in B-cells, kinase beta
Ether lipid metabolism	AK095284	lysocardiolipin acyltransferase 1
Gap junction	NM_002072, BC057777	guanine nucleotide binding protein (G protein), q polypeptide
	NM_002067	guanine nucleotide binding protein (G protein), alpha 11 (Gq class)
Glycerolipid metabolism	AK095284	lysocardiolipin acyltransferase 1
Glycerophospholipid metabolism	AK095284	lysocardiolipin acyltransferase 1
GnRH signaling pathway	NM_002072, BC057777	guanine nucleotide binding protein (G protein), q polypeptide
	NM_002067	guanine nucleotide binding protein (G protein), alpha 11 (Gq class)
Huntington's disease	NM_002072, BC057777	guanine nucleotide binding protein (G protein), q polypeptide
	NM_015477	SIN3 homolog A, transcription regulator (yeast)
Insulin signaling pathway	NM_001556, BC108694	inhibitor of kappa light polypeptide gene enhancer in B-cells, kinase beta
Keratan sulfate biosynthesis	NM_178155, NM_178157, NM_004480	fucosyltransferase 8 (alpha (1,6) fucosyltransferase)
Limonene and pinene degradation	AK095284	lysocardiolipin acyltransferase 1
Long-term depression	NM_002067	guanine nucleotide binding protein (G protein), alpha 11 (Gq class)
	NM_002072, BC057777	guanine nucleotide binding protein (G protein), q polypeptide
Long-term potentiation	NM_002072, BC057777	guanine nucleotide binding protein (G protein), q polypeptide

MAPK signaling pathway	NM_001556, BC108694	inhibitor of kappa light polypeptide gene enhancer in B-cells, kinase beta
Melanogenesis	NM_002072, BC057777	guanine nucleotide binding protein (G protein), q polypeptide
Neurotrophin signaling pathway	NM_001556, BC108694	inhibitor of kappa light polypeptide gene enhancer in B-cells, kinase beta
N-Glycan biosynthesis	NM_178155, NM_178157, NM_004480	fucosyltransferase 8 (alpha (1,6) fucosyltransferase)
NOD-like receptor signaling pathway	NM_001556, BC108694	inhibitor of kappa light polypeptide gene enhancer in B-cells, kinase beta
Pancreatic cancer	NM_001556, BC108694	inhibitor of kappa light polypeptide gene enhancer in B-cells, kinase beta
Pathways in cancer	NM_001556, BC108694	inhibitor of kappa light polypeptide gene enhancer in B-cells, kinase beta
Phenylalanine metabolism	AK095284	lysocardiolipin acyltransferase 1
Prostate cancer	NM_001556, BC108694	inhibitor of kappa light polypeptide gene enhancer in B-cells, kinase beta
Regulation of actin cytoskeleton	NM_005964, AB210026	myosin, heavy chain 10, non-muscle
	NM_002473	myosin, heavy chain 9, non-muscle
Regulation of autophagy	BC000091, NM_006395	ATG7 autophagy related 7 homolog (S. cerevisiae)
RIG-I-like receptor signaling pathway	NM_001556, BC108694	inhibitor of kappa light polypeptide gene enhancer in B-cells, kinase beta
Small cell lung cancer	NM_001556, BC108694	inhibitor of kappa light polypeptide gene enhancer in B-cells, kinase beta
T cell receptor signaling pathway	NM_001556, BC108694	inhibitor of kappa light polypeptide gene enhancer in B-cells, kinase beta
Tight junction	NM_005964, AB210026	myosin, heavy chain 10, non-muscle
	AY520816, AY520817, NM_002474, NM_022844	myosin, heavy chain 11, smooth muscle
	NM_002473	myosin, heavy chain 9, non-muscle
Toll-like receptor signaling pathway	NM_001556, BC108694	inhibitor of kappa light polypeptide gene enhancer in B-cells, kinase beta
Type II diabetes mellitus	NM_001556, BC108694	inhibitor of kappa light polypeptide gene enhancer in B-cells, kinase beta
Tyrosine metabolism	AK095284	lysocardiolipin acyltransferase 1
Vascular smooth muscle contraction	NM_002067	guanine nucleotide binding protein (G protein), alpha 11 (Gq class)
	NM_002072, BC057777	guanine nucleotide binding protein (G protein), q polypeptide
	AY520816, AY520817, NM_002474, NM_022844	myosin, heavy chain 11, smooth muscle
Viral myocarditis	NM_002473	myosin, heavy chain 9, non-muscle
	NM_005964, AB210026	myosin, heavy chain 10, non-muscle
	AY520816, AY520817, NM_002474, NM_022844	myosin, heavy chain 11, smooth muscle
