# Supplementary material for: Genome-wide association analysis for feed efficiency in Angus cattle
Source: Anim Genet. 2012 Aug;43(4):367–74. doi: 10.1111/j.1365-2052.2011.02273.x (PMC3437496; doi:10.1111/j.1365-2052.2011.02273.x)
Supplement: Supplementary file 11 [file age0043-0367-SD11.doc]

Supplementary Table 1: ADG SNPs included in the final forward selection model. BTA and position denote the chromosome and chromosomal position from the Btau4.0 assembly, respectively. Abs(α) denotes the absolute value of the allele substitution effect. 2pqα^2^ describes the genetic variance for each locus where the allele frequencies are p and q=1-p.

| Marker ID | BTA | Position (Mb) | Abs(α) | p | 2pqα^2^ |
| --- | --- | --- | --- | --- | --- |
| *ss86298222* | 1 | 59.853273 | 0.0071 | 0.1060 | 0.0000095555 |
| *ss63968561* | 1 | 63.570304 | 0.0006 | 0.5007 | 0.0000001800 |
| *ss61531716* | 1 | 105.829263 | 0.0022 | 0.3696 | 0.0000022555 |
| *ss65172344* | 1 | 108.073679 | 0.0018 | 0.3746 | 0.0000015182 |
| *ss86290982* | 2 | 118.468144 | 0.0018 | 0.4513 | 0.0000016046 |
| *ss86298054* | 2 | 118.498847 | 0.0051 | 0.4778 | 0.0000129793 |
| *ss86320968* | 2 | 132.791346 | 0.0019 | 0.5315 | 0.0000017978 |
| *rs29015763* | 3 | 18.495143 | 0.0042 | 0.7049 | 0.0000073392 |
| *ss86318343* | 3 | 115.720543 | 0.0018 | 0.4420 | 0.0000015982 |
| *ss64165385* | 4 | 27.465876 | 0.0078 | 0.4699 | 0.0000303099 |
| *ss86333174* | 4 | 48.998895 | 0.0017 | 0.0709 | 0.0000003808 |
| *ss86293686* | 4 | 88.599955 | 0.0053 | 0.5287 | 0.0000139989 |
| *ss61469518* | 5 | 16.957757 | 0.0052 | 0.8539 | 0.0000067480 |
| *ss86335501* | 5 | 33.048111 | 0.0027 | 0.3474 | 0.0000033056 |
| *ss61469484* | 6 | 18.368731 | 0.019 | 0.0559 | 0.0000380870 |
| *ss86307817* | 6 | 118.120363 | 0.006 | 0.6719 | 0.0000158719 |
| *ss117968894* | 7 | 26.449891 | 0.0046 | 0.7457 | 0.0000080252 |
| *ss65439566* | 7 | 62.064599 | 0.0106 | 0.8625 | 0.0000266562 |
| *rs29012520* | 7 | 65.978304 | 0.0004 | 0.9011 | 0.0000000285 |
| *ss86332207* | 8 | 48.680422 | 0.0027 | 0.7915 | 0.0000024057 |
| *ss86317861* | 8 | 51.089227 | 0.0068 | 0.5007 | 0.0000231200 |
| *ss86275977* | 8 | 115.607565 | 0.0093 | 0.5451 | 0.0000428927 |
| *ss86317257* | 9 | 12.902431 | 0.0049 | 0.7615 | 0.0000087223 |
| *ss86302682* | 9 | 47.588815 | 0.0036 | 0.1332 | 0.0000029934 |
| *ss86290351* | 10 | 15.067096 | 0.0045 | 0.3696 | 0.0000094366 |
| *ss86338016* | 10 | 16.437278 | 0.0039 | 0.4692 | 0.0000075761 |
| *rs29010337* | 10 | 68.029461 | 0.0046 | 0.5903 | 0.0000102352 |
| *ss61558932* | 10 | 87.622309 | 0.0032 | 0.2894 | 0.0000042116 |
| *ss86327397* | 11 | 12.784299 | 0.0069 | 0.1232 | 0.0000102865 |
| *ss86281383* | 11 | 88.002621 | 0.0061 | 0.7092 | 0.0000153490 |
| *ss61521197* | 12 | 24.477221 | 0.0033 | 0.2844 | 0.0000044324 |
| *ss86340783* | 12 | 30.270048 | 0.0013 | 0.4878 | 0.0000008445 |
| *ss86318494* | 12 | 73.030843 | 0.002 | 0.6168 | 0.0000018909 |
| *ss86307175* | 13 | 28.958202 | 0.0003 | 0.4083 | 0.0000000435 |
| *ss86339064* | 13 | 50.13714 | 0.0033 | 0.8309 | 0.0000030595 |
| *ss86325195* | 13 | 59.746078 | 0.0044 | 0.7027 | 0.0000080888 |
| *ss86311856* | 14 | 52.350311 | 0.0086 | 0.1920 | 0.0000229456 |
| *ss63983181* | 16 | 7.036933 | 0.0024 | 0.1433 | 0.0000014140 |
| *ss86329933* | 16 | 58.404713 | 0.0024 | 0.5974 | 0.0000027707 |
| *ss86298158* | 17 | 12.720105 | 0.0057 | 0.1433 | 0.0000079757 |
| *ss62643327* | 17 | 34.22045 | 0.0017 | 0.6705 | 0.0000012770 |
| *ss86303324* | 17 | 76.421554 | 0.0089 | 0.1497 | 0.0000201668 |
| *ss117972449* | 18 | 29.600313 | 0.0024 | 0.6662 | 0.0000025618 |
| *ss86303954* | 18 | 47.210624 | 0.0003 | 0.5387 | 0.0000000447 |
| *ss105259813* | 19 | 24.301922 | 0.0129 | 0.0938 | 0.0000283009 |
| *ss86283697* | 19 | 45.340598 | 0.0046 | 0.2063 | 0.0000069296 |
| *ss117965335* | 19 | 48.738154 | 0.0058 | 0.6139 | 0.0000159472 |
| *ss117965434* | 19 | 63.651366 | 0.0074 | 0.5967 | 0.0000263558 |
| *ss62617158* | 20 | 8.810833 | 0.0056 | 0.6261 | 0.0000146831 |
| *ss61543085* | 20 | 26.966402 | 0.0011 | 0.6311 | 0.0000005634 |
| *ss86336107* | 21 | 30.346882 | 0.0034 | 0.3259 | 0.0000050795 |
| *ss64618188* | 21 | 32.67505 | 0.0148 | 0.0723 | 0.0000294018 |
| *ss62727144* | 21 | 34.018769 | 0.0007 | 0.2407 | 0.0000001791 |
| *ss117965571* | 23 | 39.450899 | 0.0031 | 0.6032 | 0.0000046005 |
| *rs29018032* | 23 | 41.988126 | 0.0021 | 0.8632 | 0.0000010416 |
| *ss86289007* | 24 | 4.017729 | 0.0035 | 0.1332 | 0.0000028294 |
| *ss61547771* | 24 | 52.914945 | 0.0058 | 0.4914 | 0.0000168150 |
| *ss117973804* | 25 | 12.283689 | 0.003 | 0.6318 | 0.0000041873 |
| *ss86284393* | 25 | 37.212719 | 0.0032 | 0.4828 | 0.0000051139 |
| *ss86312150* | 26 | 7.796869 | 0.0064 | 0.8016 | 0.0000130295 |
| *ss46526045* | 26 | 43.529109 | 0.0037 | 0.5315 | 0.0000068178 |
| *ss86290736* | 27 | 12.383786 | 0.005 | 0.8660 | 0.0000058005 |
| *ss61470138* | 27 | 19.790331 | 0.0006 | 0.3782 | 0.0000001693 |
| *ss86295347* | 29 | 48.856592 | <0.0000 | 0.9033 | 0.0000000000 |
| *ss86320739* | Un |  | 0.0015 | 0.5222 | 0.0000011228 |
| *ss117974825* | Un |  | 0.0009 | 0.4291 | 0.0000003969 |
| *ss63781516* | Un |  | 0.0044 | 0.7808 | 0.0000066269 |
| *ss65169323* | Un |  | 0.0071 | 0.5444 | 0.0000250061 |

Supplementary Table 2: AFI SNPs included in the final forward selection model. BTA and position denote the chromosome and chromosomal position from the Btau4.0 assembly, respectively. Abs(α) denotes the absolute value of the allele substitution effect. 2pqα^2^ describes the genetic variance for each locus where the allele frequencies are p and q=1-p.

| Marker ID | BTA | Position (Mb) | Abs(α) | p | 2pqα^2^ |
| --- | --- | --- | --- | --- | --- |
| *ss61531716* | 1 | 105.8293 | 0.0177 | 0.3696 | 0.0001458301 |
| *ss105242396* | 1 | 113.8799 | 0.0434 | 0.4219 | 0.0009207200 |
| *ss105254260* | 2 | 31.07949 | 0.0346 | 0.6870 | 0.0005137263 |
| *ss86316304* | 2 | 67.08311 | 0.0594 | 0.4011 | 0.0016969339 |
| *rs29015763* | 3 | 18.49514 | 0.0128 | 0.7049 | 0.0000676351 |
| *ss86341694* | 3 | 23.35313 | 0.0682 | 0.2600 | 0.0017904500 |
| *ss61517428* | 3 | 70.78155 | 0.0089 | 0.1447 | 0.0000195622 |
| *ss86331253* | 4 | 62.29809 | 0.0379 | 0.5595 | 0.0007065932 |
| *ss86297489* | 6 | 90.41552 | 0.0042 | 0.5215 | 0.0000089004 |
| *ss86336887* | 6 | 106.3529 | 0.0664 | 0.2085 | 0.0014558350 |
| *ss64249818* | 7 | 32.4503 | 0.0137 | 0.3789 | 0.0000884855 |
| *ss86335346* | 8 | 91.54707 | 0.0044 | 0.6934 | 0.0000082466 |
| *rs29022959* | 8 | 91.71922 | 0.016 | 0.7213 | 0.0001026578 |
| *ss117969435* | 8 | 97.33553 | 0.032 | 0.8818 | 0.0002131990 |
| *ss61493344* | 9 | 78.2669 | 0.0373 | 0.6368 | 0.0006444193 |
| *ss86278343* | 11 | 5.66063 | 0.0326 | 0.0831 | 0.0001623506 |
| *ss86293568* | 11 | 39.35181 | 0.0182 | 0.4097 | 0.0001609282 |
| *ss117970126* | 11 | 40.35811 | 0.0495 | 0.1218 | 0.0005230355 |
| *ss86289943* | 11 | 78.30539 | 0.0299 | 0.7729 | 0.0003138842 |
| *rs29014508* | 12 | 23.42551 | 0.0139 | 0.3489 | 0.0000876509 |
| *ss86274979* | 12 | 32.53872 | 0.0079 | 0.8546 | 0.0000156294 |
| *ss61516394* | 12 | 48.75502 | 0.0574 | 0.3274 | 0.0014530141 |
| *ss65270148* | 12 | 61.90864 | 0.0099 | 0.1827 | 0.0000290295 |
| *ss86330336* | 14 | 14.98921 | 0.0374 | 0.3782 | 0.0006580700 |
| *ss117964737* | 14 | 70.09761 | 0.0052 | 0.4986 | 0.0000136554 |
| *ss86295351* | 15 | 61.34966 | 0.0413 | 0.5845 | 0.0008292738 |
| *ss86277466* | 15 | 78.10219 | 0.0317 | 0.9226 | 0.0001430937 |
| *ss117972192* | 17 | 4.094041 | 0.0358 | 0.6132 | 0.0006066270 |
| *ss61467852* | 17 | 27.83249 | 0.0274 | 0.6053 | 0.0003598838 |
| *ss61538007* | 17 | 29.24063 | 0.0198 | 0.7285 | 0.0001555952 |
| *ss117965075* | 18 | 52.35454 | 0.0445 | 0.0953 | 0.0003418681 |
| *ss61540370* | 19 | 29.93895 | 0.026 | 0.4362 | 0.0003327094 |
| *ss86328551* | 19 | 36.05319 | 0.0292 | 0.1060 | 0.0001618659 |
| *ss117965335* | 19 | 48.73815 | 0.0352 | 0.6139 | 0.0005873731 |
| *ss86318895* | 19 | 49.81029 | 0.0141 | 0.2299 | 0.0000702066 |
| *ss62644539* | 20 | 43.75955 | 0.007 | 0.6648 | 0.0000219648 |
| *ss86339752* | 21 | 31.52957 | 0.0415 | 0.8660 | 0.0003988280 |
| *ss86277953* | 21 | 33.92316 | 0.037 | 0.8875 | 0.0002738874 |
| *ss61545215* | 22 | 25.57142 | 0.0631 | 0.6519 | 0.0018042927 |
| *ss61523365* | 22 | 31.36968 | 0.0285 | 0.4599 | 0.0004035109 |
| *ss61494684* | 23 | 32.16787 | 0.035 | 0.2894 | 0.0005038349 |
| *ss86341078* | 23 | 38.86096 | 0.0354 | 0.2615 | 0.0004845156 |
| *rs29019360* | 24 | 62.80138 | 0.0384 | 0.8116 | 0.0004508341 |
| *ss61550290* | 27 | 13.17559 | 0.0103 | 0.7744 | 0.0000372182 |
| *ss86301648* | 28 | 14.75985 | 0.0319 | 0.2550 | 0.0003862190 |
| *ss86331294* | 28 | 34.27463 | 0.0401 | 0.6375 | 0.0007439119 |
| *ss86329884* | 29 | 18.94255 | 0.0556 | 0.5723 | 0.0015132079 |
| *ss86291606* | 29 | 41.28812 | 0.0601 | 0.8438 | 0.0009528913 |
| *ss86323038* | 29 | 48.72363 | 0.0047 | 0.3739 | 0.0000102112 |
| *ss65170459* | Un |  | 0.0024 | 0.2149 | 0.0000019275 |
| *ss86331954* | Un |  | 0.0234 | 0.7342 | 0.0002132906 |
| *ss86275593* | Un |  | 0.0127 | 0.2701 | 0.0000639601 |
| *ss117974956* | Un |  | 0.036 | 0.5974 | 0.0006228456 |

Supplementary Table 3: RFI SNPs included in the final forward selection model. BTA and position denote the chromosome and chromosomal position from the Btau4.0 assembly, respectively. Abs(α) denotes the absolute value of the allele substitution effect. 2pqα^2^ describes the genetic variance for each locus where the allele frequencies are p and q=1-p.

| Marker ID | BTA | Position (Mb) | Abs(α) | p | 2pqα^2^ |
| --- | --- | --- | --- | --- | --- |
| *ss64260054* | 1 | 85.95453 | 0.0185 | 0.1683 | 0.0000959028 |
| *ss86328736* | 1 | 130.3111 | 0.046 | 0.2221 | 0.0007302880 |
| *ss105301409* | 1 | 133.5587 | 0.0261 | 0.1676 | 0.0001899456 |
| *ss117966494* | 2 | 31.05061 | 0.1186 | 0.3109 | 0.0060264845 |
| *ss105254260* | 2 | 31.07949 | 0.1426 | 0.6870 | 0.0087506812 |
| *ss61484491* | 2 | 45.422 | 0.068 | 0.8474 | 0.0011943462 |
| *ss86293216* | 2 | 76.35988 | 0.0433 | 0.6103 | 0.0008901653 |
| *ss61489474* | 3 | 7.649577 | 0.0084 | 0.2751 | 0.0000281403 |
| *ss64836693* | 3 | 70.03582 | 0.034 | 0.8847 | 0.0002361678 |
| *ss86331253* | 4 | 62.29809 | 0.0213 | 0.5595 | 0.0002242468 |
| *ss64726870* | 4 | 75.92411 | 0.0214 | 0.4312 | 0.0002245856 |
| *ss86274086* | 5 | 35.90014 | 0.0189 | 0.2113 | 0.0001188279 |
| *ss61557043* | 6 | 41.86704 | 0.0289 | 0.4341 | 0.0004112025 |
| *ss86297489* | 6 | 90.41552 | 0.0051 | 0.5215 | 0.0000127277 |
| *ss86290408* | 6 | 105.4025 | 0.0131 | 0.1812 | 0.0000507740 |
| *ss86293830* | 7 | 14.27555 | 0.0066 | 0.3080 | 0.0000187780 |
| *ss86337575* | 8 | 6.769905 | 0.0417 | 0.8009 | 0.0005549146 |
| *ss86339705* | 8 | 56.62185 | 0.0249 | 0.5014 | 0.0003088333 |
| *rs29022959* | 8 | 91.71922 | 0.0251 | 0.7213 | 0.0002524645 |
| *ss86308369* | 8 | 110.8797 | 0.0375 | 0.7385 | 0.0005416445 |
| *ss64696603* | 9 | 20.70463 | 0.0103 | 0.4405 | 0.0000527223 |
| *ss86328884* | 9 | 34.81401 | 0.0474 | 0.0645 | 0.0002705972 |
| *ss61516025* | 9 | 104.334 | 0.0171 | 0.2414 | 0.0001072975 |
| *ss86327686* | 10 | 70.40733 | 0.0269 | 0.6454 | 0.0003312026 |
| *ss61491345* | 10 | 80.49974 | 0.0022 | 0.6354 | 0.0000022528 |
| *ss117970126* | 11 | 40.35811 | 0.0359 | 0.1218 | 0.0002758223 |
| *ss86303685* | 11 | 70.65666 | 0.042 | 0.6418 | 0.0008121868 |
| *ss86336779* | 11 | 105.8802 | 0.0274 | 0.3109 | 0.0003222445 |
| *ss61516394* | 12 | 48.75502 | 0.0084 | 0.3274 | 0.0000310742 |
| *ss61529532* | 12 | 53.97856 | 0.0215 | 0.1519 | 0.0001187438 |
| *ss86341687* | 12 | 72.39544 | 0.0512 | 0.8023 | 0.0008314600 |
| *ss117971065* | 14 | 3.606692 | 0.0037 | 0.5745 | 0.0000065778 |
| *ss86340984* | 14 | 9.990357 | 0.0224 | 0.8188 | 0.0001490423 |
| *ss86300474* | 14 | 78.08375 | 0.03 | 0.4140 | 0.0004353324 |
| *ss62125948* | 15 | 42.20598 | 0.0147 | 0.7034 | 0.0000898396 |
| *ss86295351* | 15 | 61.34966 | 0.0096 | 0.5845 | 0.0000444837 |
| *ss61565643* | 16 | 14.41611 | 0.0071 | 0.8861 | 0.0000102154 |
| *ss117972192* | 17 | 4.094041 | 0.0347 | 0.6132 | 0.0005725143 |
| *ss61538007* | 17 | 29.24063 | 0.0263 | 0.7285 | 0.0002731933 |
| *ss117972116* | 17 | 29.40406 | 0.0123 | 0.5709 | 0.0000736419 |
| *ss117965075* | 18 | 52.35454 | 0.0371 | 0.0953 | 0.0002375236 |
| *ss86311733* | 19 | 29.25672 | 0.0466 | 0.8782 | 0.0004634874 |
| *ss86329372* | 19 | 35.72306 | 0.0081 | 0.4140 | 0.0000318354 |
| *ss86328551* | 19 | 36.05319 | 0.0233 | 0.1060 | 0.0001031374 |
| *ss65376194* | 20 | 6.746413 | 0.0168 | 0.3617 | 0.0001303928 |
| *ss86314901* | 20 | 37.78467 | 0.022 | 0.5287 | 0.0002415123 |
| *ss61495644* | 20 | 41.21733 | 0.0141 | 0.7622 | 0.0000725756 |
| *ss61511525* | 20 | 50.48725 | 0.0288 | 0.8510 | 0.0002098884 |
| *ss86303118* | 21 | 30.98376 | 0.0234 | 0.3231 | 0.0002394147 |
| *ss86277953* | 21 | 33.92316 | 0.0326 | 0.8875 | 0.0002119006 |
| *ss61523365* | 22 | 31.36968 | 0.0022 | 0.4599 | 0.0000024044 |
| *ss86295370* | 22 | 56.05081 | 0.0246 | 0.5509 | 0.0002997658 |
| *rs29013532* | 22 | 57.36246 | 0.0084 | 0.3181 | 0.0000308784 |
| *ss86316255* | 23 | 32.67781 | 0.0466 | 0.0910 | 0.0003597838 |
| *ss61514878* | 23 | 33.238 | 0.0091 | 0.4986 | 0.0000412229 |
| *ss86330971* | 25 | 14.73887 | 0.0294 | 0.2414 | 0.0003158454 |
| *ss61486991* | 25 | 23.12439 | 0.0172 | 0.6554 | 0.0001341521 |
| *ss86274817* | 27 | 40.08445 | 0.0039 | 0.5193 | 0.0000076717 |
| *ss86321221* | 28 | 14.30796 | 0.0569 | 0.1282 | 0.0007240181 |
| *ss86289932* | 28 | 34.60799 | 0.0468 | 0.8961 | 0.0004068625 |
| *ss86294990* | 29 | 19.5054 | 0.0291 | 0.7958 | 0.0002753421 |
| *ss86328651* | 29 | 21.49747 | 0.0278 | 0.6626 | 0.0003457990 |
| *ss117965798* | 29 | 38.62682 | 0.0296 | 0.2185 | 0.0002981939 |
| *ss86319047* | Un |  | 0.001 | 0.4076 | 0.0000005193 |
| *ss65170459* | Un |  | 0.0142 | 0.2149 | 0.0000679447 |
| *ss86331954* | Un |  | 0.0231 | 0.7342 | 0.0002089518 |

Supplementary Table 4: Pathway analysis of genomic regions detected as harboring QTLs in the analysis of ADG. Regions were defined as SNP position (from the final model) ±0.5 Mb. Human annotations were mapped to bovine coordinates and genes were identified that spanned this region and analyzed in DAVID using the KEGG Pathway database.

| Pathway | Accession | Genes |
| --- | --- | --- |
| Alzheimer's disease | NM_000021 | presenilin 1 |
|  | NM_000447, NM_012486 | presenilin 2 (Alzheimer disease 4) |
| Calcium signaling pathway | J05200, NM_000540 | ryanodine receptor 1 (skeletal) |
| Fc gamma R-mediated phagocytosis | NM_198252, NM_000177 | gelsolin (amyloidosis, Finnish type) |
| Inositol phosphate metabolism | BC062317 | inositol polyphosphate-5-phosphatase, 75kDa |
| Long-term depression | J05200, NM_000540 | ryanodine receptor 1 (skeletal) |
| Lysosome | NM_001283, CR599373 | adaptor-related protein complex 1, sigma 1 subunit |
|  | NM_003916 | adaptor-related protein complex 1, sigma 2 subunit pseudogene; adaptor-related protein complex 1, sigma 2 subunit |
|  | BC009606, BC021898 | adaptor-related protein complex 1, sigma 3 subunit |
| Neuroactive ligand-receptor interaction | NM_000830 | glutamate receptor, ionotropic, kainate 1 |
|  | NM_175768, AJ252246, NM_021956, BC037954, BC063814 | glutamate receptor, ionotropic, kainate 2 |
|  | AJ249210, NM_000831 | glutamate receptor, ionotropic, kainate 3 |
| Neurotrophin signaling pathway | NM_000021 | presenilin 1 |
| Notch signaling pathway | NM_000021 | presenilin 1 |
|  | NM_000447, NM_012486 | presenilin 2 (Alzheimer disease 4) |
| O-Glycan biosynthesis | NM_198321, AK023782 | UDP-N-acetyl-alpha-D-galactosamine:polypeptide N-acetylgalactosaminyltransferase 10 (GalNAc-T10) |
|  | NM_001034845 | UDP-N-acetyl-alpha-D-galactosamine:polypeptide N-acetylgalactosaminyltransferase-like 6 |
| Phosphatidylinositol signaling system | BC062317 | inositol polyphosphate-5-phosphatase, 75kDa |
| Regulation of actin cytoskeleton | NM_198252, NM_000177 | gelsolin (amyloidosis, Finnish type) |
| Spliceosome | NM_004941, BC044586 | DEAH (Asp-Glu-Ala-His) box polypeptide 8 |
| Wnt signaling pathway | NM_000021 | presenilin 1 |

Supplementary Table 5: Pathway analysis of genomic regions detected as harboring QTLs in the analysis of AFI. Regions were defined as SNP position (from the final model) ±0.5 Mb. Human annotations were mapped to bovine coordinates and genes were identified that spanned this region and analyzed in DAVID using the KEGG Pathway database.

| Pathway | Accession | Genes |
| --- | --- | --- |
| Alzheimer's disease | NM_003824 | Fas (TNFRSF6)-associated via death domain |
| Aminoacyl-tRNA biosynthesis | NM_024678 | asparaginyl-tRNA synthetase 2, mitochondrial (putative) |
| Apoptosis | NM_003824 | Fas (TNFRSF6)-associated via death domain |
| Chemokine signaling pathway | NM_014800, AK126565, AL136787 | engulfment and cell motility 1 |
| Endocytosis | NM_022739 | SMAD specific E3 ubiquitin protein ligase 2 |
|  | NM_020429 | SMAD specific E3 ubiquitin protein ligase 1 |
| Huntington's disease | NM_015477 | SIN3 homolog A, transcription regulator (yeast) |
| Pathways in cancer | NM_003824 | Fas (TNFRSF6)-associated via death domain |
|  | NM_005436 | coiled-coil domain containing 6 |
| Proteasome | NM_002804, BC106920 | proteasome (prosome, macropain) 26S subunit, ATPase, 3 |
| Purine metabolism | NM_033109 | polyribonucleotide nucleotidyltransferase 1 |
| Pyrimidine metabolism | NM_033109 | polyribonucleotide nucleotidyltransferase 1 |
| RIG-I-like receptor signaling pathway | NM_003824 | Fas (TNFRSF6)-associated via death domain |
| RNA degradation | NM_033109 | polyribonucleotide nucleotidyltransferase 1 |
| TGF-beta signaling pathway | NM_020429 | SMAD specific E3 ubiquitin protein ligase 1 |
|  | NM_022739 | SMAD specific E3 ubiquitin protein ligase 2 |
| Thyroid cancer | NM_005436 | coiled-coil domain containing 6 |
| Toll-like receptor signaling pathway | NM_003824 | Fas (TNFRSF6)-associated via death domain |
| Ubiquitin mediated proteolysis | NM_020429 | SMAD specific E3 ubiquitin protein ligase 1 |
|  | NM_022739 | SMAD specific E3 ubiquitin protein ligase 2 |

Supplementary Table 6: Pathway analysis of genomic regions detected as harboring QTLs in the analysis of RFI. Regions were defined as SNP position (from the final model) ±0.5 Mb. Human annotations were mapped to bovine coordinates and genes were identified that spanned this region and analyzed in DAVID using the KEGG Pathway database.

| Pathway | Accession | Genes |
| --- | --- | --- |
| Acute myeloid leukemia | NM_001556, BC108694 | inhibitor of kappa light polypeptide gene enhancer in B-cells, kinase beta |
| Adherens junction | BC021301, NM_014808 | FERM, RhoGEF and pleckstrin domain protein 2 |
| Adipocytokine signaling pathway | NM_001556, BC108694 | inhibitor of kappa light polypeptide gene enhancer in B-cells, kinase beta |
| Alzheimer's disease | NM_002072, BC057777 | guanine nucleotide binding protein (G protein), q polypeptide |
| Apoptosis | NM_001556, BC108694 | inhibitor of kappa light polypeptide gene enhancer in B-cells, kinase beta |
| B cell receptor signaling pathway | NM_001556, BC108694 | inhibitor of kappa light polypeptide gene enhancer in B-cells, kinase beta |
| Calcium signaling pathway | NM_002067 | guanine nucleotide binding protein (G protein), alpha 11 (Gq class) |
|  | NM_004297 | guanine nucleotide binding protein (G protein), alpha 14 |
|  | NM_002072, BC057777 | guanine nucleotide binding protein (G protein), q polypeptide |
| Chemokine signaling pathway | AL136787, NM_014800, AK126565 | engulfment and cell motility 1 |
|  | NM_001556, BC108694 | inhibitor of kappa light polypeptide gene enhancer in B-cells, kinase beta |
| Chronic myeloid leukemia | NM_001556, BC108694 | inhibitor of kappa light polypeptide gene enhancer in B-cells, kinase beta |
| Cytosolic DNA-sensing pathway | NM_001556, BC108694 | inhibitor of kappa light polypeptide gene enhancer in B-cells, kinase beta |
| Epithelial cell signaling in Helicobacter pylori infection | NM_001556, BC108694 | inhibitor of kappa light polypeptide gene enhancer in B-cells, kinase beta |
| Ether lipid metabolism | AK095284 | lysocardiolipin acyltransferase 1 |
| Gap junction | NM_002072, BC057777 | guanine nucleotide binding protein (G protein), q polypeptide |
|  | NM_002067 | guanine nucleotide binding protein (G protein), alpha 11 (Gq class) |
| Glycerolipid metabolism | AK095284 | lysocardiolipin acyltransferase 1 |
| Glycerophospholipid metabolism | AK095284 | lysocardiolipin acyltransferase 1 |
| GnRH signaling pathway | NM_002072, BC057777 | guanine nucleotide binding protein (G protein), q polypeptide |
|  | NM_002067 | guanine nucleotide binding protein (G protein), alpha 11 (Gq class) |
| Huntington's disease | NM_002072, BC057777 | guanine nucleotide binding protein (G protein), q polypeptide |
|  | NM_015477 | SIN3 homolog A, transcription regulator (yeast) |
| Insulin signaling pathway | NM_001556, BC108694 | inhibitor of kappa light polypeptide gene enhancer in B-cells, kinase beta |
| Keratan sulfate biosynthesis | NM_178155, NM_178157, NM_004480 | fucosyltransferase 8 (alpha (1,6) fucosyltransferase) |
| Limonene and pinene degradation | AK095284 | lysocardiolipin acyltransferase 1 |
| Long-term depression | NM_002067 | guanine nucleotide binding protein (G protein), alpha 11 (Gq class) |
|  | NM_002072, BC057777 | guanine nucleotide binding protein (G protein), q polypeptide |
| Long-term potentiation | NM_002072, BC057777 | guanine nucleotide binding protein (G protein), q polypeptide |
| MAPK signaling pathway | NM_001556, BC108694 | inhibitor of kappa light polypeptide gene enhancer in B-cells, kinase beta |
| Melanogenesis | NM_002072, BC057777 | guanine nucleotide binding protein (G protein), q polypeptide |
| Neurotrophin signaling pathway | NM_001556, BC108694 | inhibitor of kappa light polypeptide gene enhancer in B-cells, kinase beta |
| N-Glycan biosynthesis | NM_178155, NM_178157, NM_004480 | fucosyltransferase 8 (alpha (1,6) fucosyltransferase) |
| NOD-like receptor signaling pathway | NM_001556, BC108694 | inhibitor of kappa light polypeptide gene enhancer in B-cells, kinase beta |
| Pancreatic cancer | NM_001556, BC108694 | inhibitor of kappa light polypeptide gene enhancer in B-cells, kinase beta |
| Pathways in cancer | NM_001556, BC108694 | inhibitor of kappa light polypeptide gene enhancer in B-cells, kinase beta |
| Phenylalanine metabolism | AK095284 | lysocardiolipin acyltransferase 1 |
| Prostate cancer | NM_001556, BC108694 | inhibitor of kappa light polypeptide gene enhancer in B-cells, kinase beta |
| Regulation of actin cytoskeleton | NM_005964, AB210026 | myosin, heavy chain 10, non-muscle |
|  | NM_002473 | myosin, heavy chain 9, non-muscle |
| Regulation of autophagy | BC000091, NM_006395 | ATG7 autophagy related 7 homolog (S. cerevisiae) |
| RIG-I-like receptor signaling pathway | NM_001556, BC108694 | inhibitor of kappa light polypeptide gene enhancer in B-cells, kinase beta |
| Small cell lung cancer | NM_001556, BC108694 | inhibitor of kappa light polypeptide gene enhancer in B-cells, kinase beta |
| T cell receptor signaling pathway | NM_001556, BC108694 | inhibitor of kappa light polypeptide gene enhancer in B-cells, kinase beta |
| Tight junction | NM_005964, AB210026 | myosin, heavy chain 10, non-muscle |
|  | AY520816, AY520817, NM_002474, NM_022844 | myosin, heavy chain 11, smooth muscle |
|  | NM_002473 | myosin, heavy chain 9, non-muscle |
| Toll-like receptor signaling pathway | NM_001556, BC108694 | inhibitor of kappa light polypeptide gene enhancer in B-cells, kinase beta |
| Type II diabetes mellitus | NM_001556, BC108694 | inhibitor of kappa light polypeptide gene enhancer in B-cells, kinase beta |
| Tyrosine metabolism | AK095284 | lysocardiolipin acyltransferase 1 |
| Vascular smooth muscle contraction | NM_002067 | guanine nucleotide binding protein (G protein), alpha 11 (Gq class) |
|  | NM_002072, BC057777 | guanine nucleotide binding protein (G protein), q polypeptide |
|  | AY520816, AY520817, NM_002474, NM_022844 | myosin, heavy chain 11, smooth muscle |
| Viral myocarditis | NM_002473 | myosin, heavy chain 9, non-muscle |
|  | NM_005964, AB210026 | myosin, heavy chain 10, non-muscle |
|  | AY520816, AY520817, NM_002474, NM_022844 | myosin, heavy chain 11, smooth muscle |
