# Supplementary material for: Genome-wide association analysis for feed efficiency in Angus cattle
Source: Anim Genet. 2012 Aug;43(4):367–74. doi: 10.1111/j.1365-2052.2011.02273.x (PMC3437496; doi:10.1111/j.1365-2052.2011.02273.x)
Supplement: Supplementary file 10 [file age0043-0367-SD10.pdf]

ss_id	rs_id	Chr	Chr Pos	FreqA	AFI F	AFI p	AFI α	AFI 2pqα
ss61512987	*rs41609588*	1	0.113642	0.1619	1.9159	0.166748	0.0352	0.0095388163
ss86310701		1	0.244699	0.3059	0.0138	0.906678	0.0024	0.0009989882
ss117966399		1	0.369419	0.1225	0.2923	0.588904	0.0150	0.0032179260
ss86329363		1	0.447278	0.1676	2.1861	0.139715	0.0357	0.0099759744
	rs29015852	1	0.487654	0.3961	5.6394	0.017831	0.0445	0.0212686356
ss86296752		1	0.524507	0.2966	0.0933	0.760099	0.0063	0.0026386449
ss86336721		1	0.562285	0.3961	5.6394	0.017831	0.0445	0.0212686356
ss86295506		1	0.68196	0.6282	0.4520	0.501633	0.0125	0.0058361813
ss86289560		1	0.772654	0.1383	0.0602	0.806299	0.0064	0.0015160341
ss86329883		1	0.807571	0.6956	0.7052	0.401317	0.0168	0.0071039093
ss86288779		1	0.838468	0.2206	1.5155	0.218720	0.0273	0.0093796137
ss105250851		1	0.91363	0.1375	0.0755	0.783590	0.0072	0.0016986297
ss61542612	*rs41635940*	1	1.008611	0.7744	1.6501	0.199368	0.0277	0.0096706717
ss105238835		1	1.097491	0.7278	0.1260	0.722766	0.0073	0.0028851197
ss46526150	*rs41255293*	1	1.170768	0.2944	0.3657	0.545528	0.0120	0.0049843326
ss86340900		1	1.196423	0.4685	14.6384	0.000142	0.0689	0.0343066900
ss61481704	*rs41580909*	1	1.324142	0.7958	8.6654	0.003351	0.0661	0.0214736877
ss86302482		1	1.354281	0.2042	8.6654	0.003351	0.0661	0.0214736877
ss86299572		1	1.393699	0.8496	3.9073	0.048472	0.0508	0.0129898928
ss86294596		1	1.479888	0.8768	3.8781	0.049317	0.0557	0.0120277070
ss86305189		1	1.857164	0.7844	0.3706	0.542878	0.0134	0.0045476734
ss61522896	*rs41622765*	1	1.936699	0.3933	7.5638	0.006110	0.0511	0.0243927548
ss86315396		1	2.019349	0.3811	8.1686	0.004390	0.0535	0.0252216595
ss86333800		1	2.098937	0.4212	11.3293	0.000805	0.0604	0.0294584003
ss86338703		1	2.121462	0.7550	4.6914	0.030653	0.0447	0.0165443028
ss86300385		1	2.202188	0.2221	2.6295	0.105350	0.0344	0.0118817493
ss86298198		1	2.223443	0.2221	2.6295	0.105350	0.0344	0.0118817493
ss86330583		1	2.270723	0.7507	4.5815	0.032666	0.0438	0.0163827627
ss86313348		1	2.333175	0.3016	0.2205	0.638791	0.0093	0.0039325909
ss86321979		1	2.391305	0.1676	1.9166	0.166670	0.0342	0.0095481851
ss86319636		1	2.45366	0.4807	0.8541	0.355726	0.0170	0.0084840662
ss86288974		1	2.522908	0.6153	1.5815	0.208969	0.0241	0.0114020585
ss86328148		1	2.558855	0.3625	0.0074	0.931661	0.0016	0.0007382844
ss65125915	*rs43207817*	1	2.58076	0.4835	7.9989	0.004815	0.0544	0.0271884535
ss86289121		1	2.68838	0.1218	0.1136	0.736214	0.0092	0.0019667695
ss65125602	*rs43213404*	1	2.741613	0.4076	1.3533	0.245102	0.0215	0.0103888232
ss86285875		1	2.775727	0.6139	0.0229	0.879631	0.0028	0.0013110949
ss86290613		1	2.809423	0.8918	0.0009	0.976440	0.0008	0.0001630737
ss65095830	*rs43180934*	1	2.877872	0.6905	1.0612	0.303303	0.0209	0.0089255265
ss65125377	*rs43211381*	1	2.914488	0.3087	1.0727	0.300702	0.0210	0.0089589598
ss86313471		1	2.977385	0.1146	0.0087	0.925626	0.0026	0.0005365776
ss86304200		1	3.015077	0.2199	0.7712	0.380139	0.0190	0.0065213072
	rs29012843	1	3.083368	0.5917	1.7664	0.184262	0.0244	0.0117865762
ss86339423		1	3.176765	0.1089	4.7432	0.029749	0.0654	0.0126899790
ss86307533		1	3.211002	0.4047	14.3036	0.000169	0.0722	0.0347917116
ss61479792	*rs41582551*	1	3.239885	0.2106	0.7722	0.379842	0.0195	0.0064893009

ss86318526		1	3.268724	0.0981	2.1322	0.144682	0.0447	0.0079139802
ss61480029	*rs41584384*	1	3.294592	0.0630	5.9479	0.014984	0.0939	0.0110940544
ss86300039		1	3.408359	0.3582	0.9235	0.336887	0.0186	0.0085379742
ss86301529		1	3.434514	0.2837	5.2299	0.022501	0.0475	0.0192900195
	rs29024375	1	3.467665	0.4678	6.8569	0.009022	0.0475	0.0236333342
ss86334800		1	3.517249	0.0774	0.2720	0.602172	0.0180	0.0025708782
ss61480294	*rs41577048*	1	3.555007	0.9226	0.2720	0.602172	0.0180	0.0025708782
ss86311110		1	3.654143	0.3496	2.9581	0.085892	0.0342	0.0155557766
ss65127461	*rs43212751*	1	3.707569	0.2357	0.1927	0.660812	0.0095	0.0034215746
ss65127265	*rs43211655*	1	3.775845	0.4807	3.5406	0.060299	0.0355	0.0177114138
ss105241323		1	3.816	0.6948	0.6355	0.425617	0.0162	0.0068662990
ss61534748	*rs41628897*	1	3.848026	0.1748	1.0088	0.315550	0.0248	0.0071564466
ss61534229	*rs41633082*	1	3.871067	0.6017	2.3167	0.128445	0.0279	0.0133754973
ss61534216	*rs41633069*	1	3.895811	0.6003	2.7339	0.098693	0.0304	0.0145665544
ss65126986	*rs43209887*	1	3.923164	0.8095	0.3579	0.549873	0.0141	0.0043429027
ss65119597	*rs43204112*	1	4.149603	0.5989	8.0011	0.004810	0.0515	0.0247516032
ss65011705	*rs43099270*	1	4.284069	0.5587	0.0252	0.873814	0.0028	0.0014024387
ss61512686	*rs41614489*	1	4.477888	0.2006	3.1992	0.074107	0.0424	0.0136066401
ss63697380	*rs42317790*	1	4.525407	0.9169	0.4478	0.503608	0.0222	0.0033890277
	rs29021339	1	4.639722	0.1361	3.1607	0.075868	0.0485	0.0114094321
	rs29022048	1	4.696815	0.6490	2.6681	0.102832	0.0311	0.0141898597
ss63696456	*rs42314082*	1	4.744613	0.9062	0.0330	0.855860	0.0058	0.0009857480
ss86339225		1	4.827062	0.2256	0.0350	0.851583	0.0041	0.0014316527
ss63696000	*rs42318929*	1	4.850611	0.4878	0.4507	0.502237	0.0124	0.0061973753
ss63695853	*rs42318880*	1	4.874582	0.5537	1.7328	0.188491	0.0242	0.0119457609
ss61512673	*rs41614477*	1	4.927997	0.4262	1.9160	0.166746	0.0254	0.0124466183
ss86336516		1	4.951465	0.7672	2.6927	0.101263	0.0358	0.0128040219
ss64746472	*rs42845302*	1	5.09884	0.7701	0.1229	0.726016	0.0076	0.0027027364
ss65128280	*rs43213168*	1	5.146395	0.2514	0.1621	0.687364	0.0085	0.0032034504
ss86334106		1	5.191117	0.7593	0.2467	0.619564	0.0107	0.0038950155
ss61499586		1	5.222501	0.3453	0.0420	0.837764	0.0040	0.0017881631
ss61523849	*rs41623606*	1	5.249214	0.3453	0.0420	0.837764	0.0040	0.0017881631
ss65128067	*rs43211556*	1	5.273271	0.8610	3.0278	0.082290	0.0458	0.0109719058
ss65128012	*rs43210901*	1	5.303426	0.4097	0.1753	0.675559	0.0078	0.0037771912
ss61535885	*rs41630314*	1	5.372373	0.7242	0.1392	0.709220	0.0076	0.0030517841
ss65127950	*rs43210840*	1	5.395582	0.3768	0.4734	0.491638	0.0129	0.0060699556
ss61522903	*rs41622772*	1	5.417033	0.5552	2.1222	0.145627	0.0272	0.0134178376
ss61480774	*rs41580510*	1	5.485774	0.5201	0.5247	0.469095	0.0137	0.0068199650
ss86336741		1	5.524766	0.5666	0.1456	0.702919	0.0072	0.0035219267
ss61480767	*rs41580503*	1	5.586311	0.1146	0.4312	0.511625	0.0184	0.0037397462
ss64519712	*rs42677546*	1	5.773944	0.3804	0.0875	0.767429	0.0057	0.0026972132
ss86335879		1	5.830837	0.1812	3.7986	0.051698	0.0465	0.0137855278
ss86302112		1	5.856709	0.8367	3.9803	0.046424	0.0505	0.0138135375
ss64375229	*rs42612145*	1	5.888292	0.7199	3.2590	0.071464	0.0379	0.0152698275
ss86336967		1	5.91607	0.2421	0.4628	0.496548	0.0149	0.0054543723
ss64375489	*rs42613075*	1	5.973404	0.4685	1.6765	0.195819	0.0236	0.0117505521
ss46526166	*rs41255308*	1	6.046098	0.4277	0.3208	0.571334	0.0107	0.0052482743

ss61508668	*rs41605828*	1	6.077725	0.4248	0.4445	0.505164	0.0127	0.0061826622
ss61467737	*rs41571862*	1	6.219143	0.6003	0.0025	0.960172	0.0009	0.0004544413
ss86335646		1	6.301299	0.8295	0.0118	0.913627	0.0026	0.0007492795
ss117966392		1	6.350259	0.2271	0.6502	0.420300	0.0177	0.0062181592
ss117963551		1	6.410344	0.6010	0.9286	0.335555	0.0177	0.0085036836
ss61483023	*rs41583696*	1	6.443778	0.5587	0.3433	0.558118	0.0107	0.0052608841
ss61483025	*rs41583698*	1	6.481567	0.0552	0.0014	0.969759	0.0016	0.0001638759
ss61540450	*rs41643591*	1	6.539424	0.4341	0.0593	0.807632	0.0046	0.0022384085
ss86340843		1	6.559946	0.1003	2.3029	0.129583	0.0460	0.0083093698
ss61483259	*rs41576336*	1	6.590363	0.5301	9.3697	0.002291	0.0563	0.0280729080
ss61483542	*rs41578709*	1	6.722802	0.3503	1.1489	0.284151	0.0207	0.0094335995
ss86326514		1	6.747618	0.4277	6.7289	0.009686	0.0478	0.0234010620
ss61540987	*rs41639125*	1	6.78311	0.4542	6.5197	0.010881	0.0464	0.0230191702
ss61483784	*rs41581949*	1	6.868187	0.9348	5.3586	0.020910	0.0858	0.0104587985
ss61507178	*rs41603577*	1	6.914656	0.2801	9.0694	0.002693	0.0615	0.0247844141
ss86292125		1	6.936639	0.6189	0.8470	0.357718	0.0175	0.0082463660
ss86288858		1	7.106053	0.4986	0.9119	0.339940	0.0179	0.0089607996
ss86339941		1	7.237454	0.2758	7.1708	0.007585	0.0541	0.0216025222
ss61532196	*rs41633309*	1	7.298269	0.4821	0.4192	0.517546	0.0118	0.0058736536
ss61511952	*rs41609666*	1	7.378097	0.1590	2.0812	0.149572	0.0366	0.0097955586
ss61543192	*rs41639307*	1	7.526893	0.9441	0.0067	0.934775	0.0033	0.0003468652
ss61502401	*rs41606310*	1	7.569168	0.9362	4.0275	0.045151	0.0744	0.0088765549
ss61537877	*rs41635667*	1	7.608504	0.7020	8.6597	0.003362	0.0593	0.0248250510
ss61519664	*rs41616911*	1	7.631043	0.8489	0.0004	0.984091	0.0005	0.0001284525
ss86337705		1	7.658696	0.6003	5.4164	0.020235	0.0440	0.0210921550
ss117966073		1	7.686125	0.1282	8.7830	0.003144	0.0809	0.0180858757
ss65052692	*rs43140120*	1	7.80882	0.8911	0.6025	0.437890	0.0229	0.0044351815
ss86293484		1	7.846962	0.1741	0.4453	0.504804	0.0157	0.0045197375
ss86289866		1	7.959111	0.2872	2.0843	0.149267	0.0283	0.0115826532
ss117963534		1	8.015128	0.7063	0.1349	0.713497	0.0074	0.0030844551
ss105260155		1	8.044612	0.4119	0.9035	0.342171	0.0174	0.0084201336
ss61498915	*rs43707416*	1	8.085853	0.4341	0.7846	0.376041	0.0166	0.0081719008
ss86322629		1	8.134281	0.5659	0.6433	0.422787	0.0149	0.0073267395
	rs29013987	1	8.157757	0.5122	1.1072	0.293063	0.0195	0.0097671689
ss86336235		1	8.264036	0.1748	0.1089	0.741533	0.0079	0.0022906375
ss61511689	*rs41614399*	1	8.294019	0.5559	8.9342	0.002897	0.0552	0.0272316395
ss105254853		1	8.353754	0.7808	4.7237	0.030086	0.0469	0.0160454427
ss61475241	*rs41572727*	1	8.541121	0.2364	3.0099	0.083199	0.0376	0.0135828601
	rs29024165	1	8.748047	0.1418	0.1144	0.735276	0.0088	0.0021526542
ss61522777	*rs41572726*	1	8.885096	0.5910	1.0761	0.299939	0.0187	0.0090574645
ss46526282	*rs41255421*	1	8.949726	0.3009	5.1707	0.023275	0.0456	0.0191719000
	rs29015773	1	8.982861	0.3489	9.2276	0.002473	0.0585	0.0265976379
ss86341785		1	9.004207	0.3503	7.8885	0.005115	0.0543	0.0246991247
ss86288486		1	9.0989	0.4900	0.2840	0.594249	0.0099	0.0049352211
ss61537436	*rs41640533*	1	9.306679	0.5201	0.0000	0.996540	0.0001	0.0000400672
ss61537420		1	9.35849	0.3059	1.0685	0.301652	0.0205	0.0087193997
ss61536880		1	9.387585	0.4119	0.0245	0.875585	0.0029	0.0014181536

ss86284510		1	9.467429	0.9026	8.3317	0.004017	0.0873	0.0153604902
ss86340246		1	9.575773	0.7744	17.2591	0.000037	0.0886	0.0309668239
ss61536385	*rs41634509*	1	9.741578	0.7321	0.0120	0.912904	0.0023	0.0008929313
ss61543832	*rs41638135*	1	9.772389	0.7844	0.0036	0.951867	0.0013	0.0004498127
ss86274002		1	9.873496	0.3489	0.1747	0.676119	0.0081	0.0036793121
ss86323630		1	9.894291	0.4226	0.0029	0.957367	0.0010	0.0004733837
ss86274716		1	9.915779	0.7744	1.4608	0.227214	0.0264	0.0092243809
ss61477416	*rs41576751*	1	9.955276	0.0824	9.2373	0.002460	0.1006	0.0152098432
	rs29022825	1	10.029277	0.6784	0.6510	0.420033	0.0158	0.0068906384
ss61484583	*rs41580327*	1	10.075526	0.7421	1.0134	0.314450	0.0210	0.0080513757
ss61484579	*rs41580323*	1	10.098308	0.6533	0.8645	0.352800	0.0178	0.0080674510
ss86318023		1	10.122356	0.7994	4.4529	0.035198	0.0481	0.0154196615
ss61526699	*rs41625585*	1	10.143921	0.2328	0.5023	0.478748	0.0150	0.0053555223
ss117966158		1	10.245715	0.6160	0.5316	0.466172	0.0138	0.0065413193
ss86337292		1	10.387988	0.4764	0.0098	0.921283	0.0019	0.0009340228
ss86341378		1	10.447569	0.4678	0.1224	0.726584	0.0065	0.0032359845
ss61524333	*rs41619783*	1	10.680023	0.3703	0.3246	0.569069	0.0111	0.0051962199
	rs29019235	1	10.75379	0.8044	3.7020	0.054755	0.0445	0.0140099364
ss86308138		1	10.871815	0.9033	0.2921	0.589041	0.0174	0.0030319216
ss61525542	*rs41621754*	1	10.943316	0.5616	0.0761	0.782757	0.0050	0.0024409722
ss86315185		1	11.021075	0.6562	6.0829	0.013889	0.0467	0.0210686048
	rs29009637	1	11.260939	0.3768	0.0186	0.891526	0.0026	0.0012089200
ss61507561	*rs41607853*	1	11.324871	0.2414	2.3430	0.126298	0.0332	0.0121527409
	rs29027263	1	11.478452	0.4076	0.7826	0.376663	0.0164	0.0079351912
ss105239548		1	11.862359	0.1311	5.0691	0.024667	0.0605	0.0137891217
ss61485622		1	11.914097	0.1640	5.2429	0.022335	0.0582	0.0159683618
	rs29015665	1	11.959753	0.6977	0.0865	0.768821	0.0059	0.0024908447
ss61545435	*rs41643380*	1	11.98171	0.3259	0.0508	0.821671	0.0045	0.0019571687
ss86336383		1	12.214278	0.1490	7.0118	0.008281	0.0700	0.0177491103
ss65129930	*rs43213915*	1	12.265746	0.1597	4.3281	0.037853	0.0529	0.0142012219
ss105240292		1	12.506327	0.8259	6.7358	0.009649	0.0641	0.0184333265
	rs29019231	1	12.544191	0.1812	5.8710	0.015646	0.0591	0.0175446747
ss61494668	*rs41589357*	1	12.741359	0.5136	0.9180	0.338327	0.0175	0.0087475156
ss105236745		1	12.959937	0.5709	5.7390	0.016855	0.0435	0.0213079697
ss105300520		1	13.137364	0.8381	0.7309	0.392894	0.0213	0.0057798775
ss61566561	*rs41665389*	1	13.159988	0.8653	3.5140	0.061270	0.0495	0.0115301174
ss61505139	*rs41602904*	1	13.22716	0.4785	1.1696	0.279852	0.0196	0.0097589225
ss86336576		1	13.447813	0.0903	5.0678	0.024685	0.0707	0.0116127373
ss86300425		1	14.28437	0.2586	6.7669	0.009484	0.0535	0.0205313230
ss117966132		1	14.321529	0.6282	13.7671	0.000223	0.0689	0.0321832547
ss105241001		1	14.360343	0.8453	8.1428	0.004452	0.0722	0.0188961573
ss86334397		1	14.5484	0.4277	2.0070	0.157024	0.0264	0.0129382514
	rs29013338	1	14.765724	0.9262	0.3408	0.559546	0.0205	0.0028025028
ss86334527		1	14.816302	0.5509	0.1683	0.681771	0.0074	0.0036479078
ss61531273	*rs41633932*	1	14.841104	0.8431	7.0512	0.008102	0.0660	0.0174668917
ss61859589	*rs41671573*	1	14.875612	0.6433	1.7251	0.189474	0.0247	0.0113371483
ss61568596	*rs41665284*	1	14.989866	0.1834	1.8201	0.177745	0.0316	0.0094571771

ss61473640	*rs41573056*	1	15.157456	0.6755	0.7232	0.395386	0.0169	0.0073899647
ss61519181	*rs41612434*	1	15.189918	0.1053	0.1264	0.722348	0.0104	0.0019676240
ss63848004	*rs42386288*	1	15.241613	0.3711	3.0819	0.079608	0.0336	0.0156847712
ss86308312		1	15.281557	0.8954	4.8630	0.027765	0.0666	0.0124675340
ss86332902		1	15.440305	0.5967	0.3871	0.534049	0.0117	0.0056266808
ss105238921		1	15.46415	0.6669	6.3015	0.012289	0.0493	0.0219031318
ss61519191	*rs41612444*	1	15.489904	0.4162	0.5056	0.477283	0.0132	0.0064170222
ss63848838	*rs42380309*	1	15.639891	0.9441	1.9506	0.162962	0.0567	0.0059863808
	rs29017514	1	15.956986	0.1261	8.9042	0.002945	0.0823	0.0181415053
ss63785892	*rs42346807*	1	16.027989	0.5974	0.8630	0.353231	0.0179	0.0086320822
ss62043931	*rs41735645*	1	16.068792	0.5143	0.4593	0.498200	0.0125	0.0062263289
ss86325921		1	16.100121	0.2722	0.6557	0.418370	0.0171	0.0067661946
ss86328392		1	16.219632	0.8388	0.8228	0.364665	0.0229	0.0061858612
ss61475803	*rs41577373*	1	16.276348	0.6533	0.0816	0.775263	0.0055	0.0024688652
ss65014448	*rs43108390*	1	16.316415	0.6791	0.2478	0.618808	0.0099	0.0043198833
ss86303567		1	16.360595	0.8059	0.0412	0.839162	0.0047	0.0014576220
ss117963529		1	16.400013	0.3489	0.0024	0.960705	0.0010	0.0004424180
ss63468920	*rs42237543*	1	16.443758	0.1676	3.7644	0.052759	0.0487	0.0135777795
ss86280744		1	16.472909	0.7042	0.0067	0.934887	0.0017	0.0006981959
ss86299876		1	16.511615	0.4685	3.4740	0.062763	0.0342	0.0170526809
ss63468770	*rs42243991*	1	16.597222	0.0989	4.2447	0.039744	0.0633	0.0112790475
ss63468692	*rs42243965*	1	16.642719	0.0874	3.1322	0.077198	0.0568	0.0090530869
ss63468122	*rs42242577*	1	16.667455	0.9105	3.5314	0.060635	0.0597	0.0097405352
ss86322744		1	16.740527	0.6934	2.0442	0.153235	0.0293	0.0124622861
ss63468008	*rs42242539*	1	16.769382	0.1426	4.4671	0.034909	0.0580	0.0141703772
ss64888650	*rs42984454*	1	16.80175	0.4312	6.1635	0.013276	0.0461	0.0225907460
ss64888702	*rs42984506*	1	16.828641	0.9212	1.7197	0.190163	0.0445	0.0064616460
	rs29019581	1	16.957941	0.6605	1.6399	0.200762	0.0251	0.0112693920
ss61521719	*rs41621019*	1	16.985643	0.8044	0.6328	0.426618	0.0181	0.0057045759
ss62576397	*rs41923266*	1	17.01236	0.4850	2.0206	0.155631	0.0260	0.0129893778
ss62576607	*rs41924236*	1	17.106221	0.6483	0.2906	0.589982	0.0106	0.0048441218
ss86281109		1	17.145974	0.6225	0.1956	0.658460	0.0083	0.0039186595
ss61521801	*rs41621900*	1	17.178059	0.3589	0.0308	0.860699	0.0034	0.0015585246
	rs29009793	1	17.201725	0.5723	1.7053	0.192030	0.0246	0.0120493209
ss61518195	*rs41612163*	1	17.325122	0.2901	4.6160	0.032020	0.0432	0.0177989041
	rs29011587	1	17.361054	0.5723	2.1923	0.139153	0.0275	0.0134769849
ss61516693	*rs41617994*	1	17.454663	0.8331	0.0327	0.856521	0.0045	0.0012469734
ss61518227	*rs41612192*	1	17.516394	0.2342	2.0970	0.148043	0.0308	0.0110511187
ss61466634	*rs41571286*	1	17.549129	0.4864	1.6525	0.199051	0.0237	0.0118435197
ss86340204		1	17.616387	0.6160	0.1032	0.748090	0.0061	0.0028959368
ss61552393	*rs43710749*	1	17.699247	0.6325	6.2521	0.012634	0.0471	0.0218990383
ss86334446		1	17.812948	0.7393	0.0681	0.794160	0.0054	0.0020801497
ss86294724		1	17.92163	0.0573	3.5899	0.058545	0.0750	0.0081076366
ss65130700	*rs43223333*	1	17.9811	0.8345	2.4063	0.121303	0.0387	0.0106790295
ss117966257		1	18.042777	0.4484	0.7130	0.398755	0.0161	0.0079399498
ss86328418		1	18.195202	0.2464	8.5958	0.003480	0.0634	0.0235287522
ss64422278	*rs42629600*	1	18.221165	0.2450	8.3147	0.004054	0.0626	0.0231443009

ss64422404	rs42630163	1	18.244759	0.2163	1.6202	0.203482	0.0287	0.0097328825
ss64422484	rs42630203	1	18.291729	0.7321	10.4680	0.001272	0.0680	0.0266756061
ss64422622	rs42630971	1	18.323377	0.7593	5.7091	0.017143	0.0521	0.0190381296
ss64948173	rs43045260	1	18.362369	0.7607	5.9137	0.015275	0.0530	0.0192869306
	rs29009870	1	18.383578	0.2321	6.1312	0.013518	0.0540	0.0192500423
ss61547049	rs41643682	1	18.426961	0.6039	7.4525	0.006495	0.0504	0.0241052244
ss38328132	rs29020446	1	18.449957	0.3374	3.2027	0.073950	0.0347	0.0154952219
ss64906637	rs42999739	1	18.662822	0.8216	8.5188	0.003628	0.0700	0.0205032250
ss64083329	rs42469069	1	18.7026	0.2428	10.5177	0.001239	0.0698	0.0256701927
ss117966449		1	18.771895	0.6146	11.0848	0.000916	0.0641	0.0303614499
ss61530321	rs41633399	1	18.918773	0.6203	11.4866	0.000741	0.0640	0.0301249964
ss86338922		1	18.953352	0.2443	6.8414	0.009100	0.0585	0.0215926201
ss86339602		1	19.148985	0.1132	0.1792	0.672159	0.0125	0.0025112476
ss61570352	rs41666815	1	19.17935	0.3539	6.2925	0.012352	0.0487	0.0222515850
ss86274921		1	19.345603	0.4599	2.9362	0.087060	0.0318	0.0157795818
ss86289776		1	19.385765	0.4599	2.9362	0.087060	0.0318	0.0157795818
ss61465563	rs41566808	1	19.640293	0.5989	5.4621	0.019716	0.0443	0.0212808947
ss61522026		1	19.802294	0.9269	0.1280	0.720594	0.0129	0.0017470180
ss118418281		1	19.851471	0.2206	9.0539	0.002716	0.0657	0.0225960655
ss117963490		1	19.877646	0.4269	5.6403	0.017823	0.0436	0.0213306968
ss105237541		1	19.919636	0.4384	5.8854	0.015520	0.0441	0.0217372877
ss118418284		1	19.952844	0.4169	6.4594	0.011252	0.0465	0.0226059791
ss105237529		1	19.988097	0.5852	6.0841	0.013880	0.0451	0.0219158860
ss86295604		1	20.038994	0.7951	1.6964	0.193187	0.0305	0.0099355387
ss61508468	rs41604738	1	20.064587	0.4835	0.1298	0.718718	0.0069	0.0034421756
ss61537390		1	20.265533	0.7242	1.2250	0.268765	0.0236	0.0094278050
ss86340805		1	20.294178	0.2077	4.5593	0.033089	0.0497	0.0163607855
ss61508465	rs41604735	1	20.373074	0.2393	0.7736	0.379400	0.0195	0.0070905654
ss105241769		1	20.399663	0.3524	1.4315	0.231925	0.0236	0.0107751382
ss86339310		1	20.421151	0.6626	2.2214	0.136565	0.0293	0.0131021161
ss117966255		1	20.560762	0.7407	0.8475	0.357588	0.0197	0.0075673254
ss117966265		1	20.637773	0.5150	6.6086	0.010356	0.0487	0.0243479159
	rs29023743	1	20.722666	0.3567	0.9272	0.335924	0.0197	0.0090511048
	rs29018413	1	20.830495	0.2529	0.2531	0.615056	0.0113	0.0042521328
	rs29010295	1	20.870761	0.5344	4.3672	0.037000	0.0407	0.0202426976
ss117966174		1	20.912267	0.4628	0.1886	0.664237	0.0080	0.0039898003
ss86299184		1	20.933176	0.7586	0.0443	0.833449	0.0047	0.0017120258
ss117963464		1	21.011529	0.1963	0.3877	0.533740	0.0144	0.0045543232
ss117963566		1	21.044559	0.2393	0.0181	0.893162	0.0030	0.0010911252
ss61530096	rs41631792	1	21.093245	0.7808	0.3135	0.575690	0.0122	0.0041817940
ss86320919		1	21.224196	0.6519	2.6620	0.103222	0.0321	0.0145678393
ss61529419	rs41625140	1	21.300411	0.4019	3.2976	0.069810	0.0356	0.0170959394
ss86332554		1	21.412633	0.7013	2.4649	0.116872	0.0322	0.0134735364
	rs29027390	1	21.491227	0.7787	0.3211	0.571123	0.0132	0.0045483740
ss64062005	rs42453650	1	21.530312	0.2944	0.0335	0.854769	0.0038	0.0015803103
ss63819045	rs42366516	1	21.595039	0.1261	0.4079	0.523229	0.0185	0.0040743332
ss63818797	rs42365792	1	21.658255	0.3632	0.0859	0.769598	0.0057	0.0026310957

ss63819105	*rs42367046*	1	21.693775	0.5215	0.0086	0.926039	0.0017	0.0008424694
ss63564364	*rs42273209*	1	21.801171	0.3188	1.4123	0.235088	0.0230	0.0100015257
ss63564400	*rs42273221*	1	21.837258	0.0931	2.2548	0.133656	0.0480	0.0081006779
ss63564430	*rs42273731*	1	21.863741	0.4456	6.8698	0.008958	0.0492	0.0243205720
ss61563912	*rs41665076*	1	21.892256	0.5802	2.3967	0.122049	0.0292	0.0142334515
ss63564514	*rs42273759*	1	21.919793	0.3517	1.5626	0.211710	0.0242	0.0110312175
	rs29026807	1	22.034496	0.3532	2.2575	0.133422	0.0293	0.0133771571
ss63564601	*rs42273787*	1	22.063411	0.3395	3.7224	0.054093	0.0377	0.0169268376
ss86281358		1	22.126711	0.5946	4.5448	0.033369	0.0406	0.0195781402
ss61564220		1	22.160248	0.4040	4.5555	0.033161	0.0406	0.0195424389
ss117963557		1	22.184889	0.4269	5.3410	0.021121	0.0440	0.0215427157
ss105241835		1	22.223201	0.2199	0.9475	0.330683	0.0220	0.0075620031
ss117966171		1	22.253339	0.8961	0.1065	0.744230	0.0102	0.0018899091
ss86341359		1	22.292406	0.6748	0.2070	0.649249	0.0092	0.0040230231
ss65135012	*rs43218341*	1	22.335556	0.5473	7.1970	0.007476	0.0501	0.0248495050
ss61548179	*rs41646592*	1	22.37239	0.6125	2.4134	0.120752	0.0298	0.0141347016
ss65135213	*rs43219642*	1	22.410017	0.5860	1.7556	0.185605	0.0249	0.0120783318
ss86340422		1	22.448596	0.5580	4.7602	0.029459	0.0417	0.0205762439
ss65135358	*rs43219987*	1	22.838393	0.5903	1.7600	0.185058	0.0249	0.0120435793
ss64686703	*rs42781274*	1	22.921098	0.8496	6.5058	0.010965	0.0665	0.0169873799
ss65040729	*rs43131664*	1	22.96185	0.8116	5.7828	0.016443	0.0559	0.0170863273
ss64524436	*rs42677996*	1	23.220898	0.4004	2.2594	0.133256	0.0277	0.0133072596
ss86327885		1	23.317323	0.3403	0.8586	0.354441	0.0183	0.0082029718
ss86321749		1	23.503625	0.2665	0.0506	0.822169	0.0047	0.0018335797
ss63675957	*rs42309386*	1	23.55125	0.3145	2.2596	0.133242	0.0302	0.0130156961
ss63675399	*rs42307601*	1	23.644532	0.4234	0.1346	0.713844	0.0068	0.0033004962
ss63675504	*rs42308435*	1	23.694843	0.4799	0.1196	0.729616	0.0064	0.0031701572
ss86292289		1	23.728417	0.0996	7.8884	0.005115	0.0880	0.0157776479
ss117966339		1	23.76305	0.2249	0.2874	0.592034	0.0119	0.0041614530
	rs29017639	1	23.939833	0.2536	2.4144	0.120680	0.0322	0.0121930022
ss61542627	*rs41635955*	1	23.970355	0.7213	0.3112	0.577148	0.0117	0.0046884803
ss61484341	*rs41578194*	1	24.000231	0.8374	1.6814	0.195163	0.0320	0.0087216239
ss65134311	*rs43222739*	1	24.039671	0.2908	0.5925	0.441696	0.0159	0.0065437190
ss61542633	*rs41635960*	1	24.118522	0.7815	0.0211	0.884468	0.0033	0.0011113207
ss86314294		1	24.145529	0.7951	0.4355	0.509518	0.0146	0.0047686626
ss65134757	*rs43227184*	1	24.167339	0.7443	0.7099	0.399751	0.0173	0.0066011458
ss105241113		1	24.189629	0.2099	1.3462	0.246346	0.0263	0.0087301375
ss65134815	*rs43228241*	1	24.232501	0.8073	1.6101	0.204901	0.0294	0.0091604359
ss86318974		1	24.275183	0.3732	0.7259	0.394523	0.0160	0.0074639932
ss105236938		1	24.368192	0.1074	0.4089	0.522738	0.0190	0.0036471020
ss65138355	*rs43223578*	1	24.411506	0.8890	1.6367	0.201208	0.0371	0.0073218995
	rs29014870	1	24.483632	0.9255	5.4787	0.019531	0.0825	0.0113744986
ss61542586	*rs41646101*	1	24.574639	0.9262	1.5153	0.218746	0.0446	0.0060910329
	rs29016259	1	24.611233	0.9277	1.4403	0.230505	0.0438	0.0058787141
ss61509073	*rs41601537*	1	24.657857	0.2966	4.7692	0.029307	0.0433	0.0180843962
ss61537397	*rs41639694*	1	24.746917	0.8789	7.9952	0.004825	0.0790	0.0168148524
	rs29022780	1	24.769055	0.5480	5.7242	0.016997	0.0454	0.0224831158

ss86293562		1	24.797857	0.8338	3.7345	0.053705	0.0469	0.0129878268
ss65132173	*rs43217804*	1	24.853103	0.5344	9.9233	0.001702	0.0582	0.0289699580
ss117963512		1	24.908255	0.3345	2.8665	0.090889	0.0325	0.0144500166
ss86332970		1	24.935985	0.1920	0.2020	0.653284	0.0106	0.0032746566
ss64992692	*rs43086367*	1	24.996033	0.4284	0.3776	0.539096	0.0116	0.0056798050
ss61542599	*rs41646113*	1	25.018717	0.1146	4.5137	0.033977	0.0636	0.0129048358
ss61542604	*rs41635932*	1	25.071646	0.2142	1.5374	0.215418	0.0280	0.0094412862
ss61542596	*rs41646110*	1	25.129232	0.4534	0.1174	0.731965	0.0064	0.0031709844
ss105299265		1	25.183429	0.1411	1.7362	0.188051	0.0355	0.0085940123
ss86340554		1	25.215049	0.6383	0.7616	0.383121	0.0171	0.0079095667
ss65134052	*rs43219083*	1	25.366412	0.7994	0.0199	0.887869	0.0032	0.0010349266
ss86312410		1	25.408457	0.3481	2.5430	0.111241	0.0305	0.0138383747
ss86332194		1	25.603107	0.3138	1.3123	0.252379	0.0232	0.0100025613
ss61542613	*rs41635941*	1	25.630994	0.2099	5.5252	0.019023	0.0516	0.0171109781
ss105239007		1	25.652143	0.3832	6.5034	0.010980	0.0485	0.0229344705
ss86341407		1	25.684793	0.7908	3.5442	0.060172	0.0421	0.0139213957
ss61542611	*rs41635939*	1	25.789603	0.6067	7.3569	0.006846	0.0511	0.0243827458
ss86322592		1	25.865178	0.3410	6.5849	0.010493	0.0491	0.0220778640
ss65133561	*rs43223792*	1	25.945613	0.0451	2.3441	0.126212	0.0680	0.0058586061
ss65133522	*rs43223753*	1	26.009921	0.0766	0.0216	0.883135	0.0052	0.0007424548
ss86300221		1	26.048845	0.5960	0.0199	0.887753	0.0027	0.0012761843
ss86304407		1	26.399729	0.7049	1.1658	0.280634	0.0225	0.0093473217
ss86329426		1	26.49257	0.5974	1.3510	0.245498	0.0217	0.0104607017
ss86281869		1	26.518093	0.7794	0.0044	0.947252	0.0015	0.0005168985
ss64366153	*rs42604233*	1	26.604937	0.7414	0.1448	0.703665	0.0079	0.0030374991
	rs29016891	1	26.710109	0.0723	0.9054	0.341656	0.0347	0.0046642394
ss61467028	*rs41565264*	1	26.758615	0.9262	1.2980	0.254977	0.0413	0.0056396877
	rs29013246	1	26.844053	0.7887	0.8759	0.349648	0.0207	0.0069051419
ss86291188		1	26.876168	0.6132	0.2409	0.623683	0.0093	0.0044345409
ss61479037	*rs41579905*	1	26.91248	0.1691	2.4148	0.120649	0.0385	0.0108278540
ss61522795	*rs41622064*	1	27.204282	0.8832	0.1044	0.746749	0.0089	0.0018297503
ss63929739	*rs42338726*	1	27.248547	0.1053	1.4168	0.234331	0.0367	0.0069122669
ss64466644	*rs42648975*	1	27.314803	0.5437	1.6466	0.199853	0.0237	0.0117415807
ss63929325	*rs42336619*	1	27.390503	0.8446	5.4253	0.020133	0.0587	0.0154089848
ss117975265		1	27.415115	0.3532	0.3837	0.535810	0.0118	0.0054135322
ss61506365	*rs41605379*	1	27.482861	0.3890	0.0224	0.881087	0.0028	0.0013334118
	rs29013556	1	27.579796	0.4226	1.6465	0.199861	0.0244	0.0119034474
ss61507625	*rs41607913*	1	27.623092	0.6927	1.6204	0.203468	0.0267	0.0113668584
ss86341236		1	27.65867	0.8503	1.9464	0.163417	0.0366	0.0093082958
ss117966276		1	27.732833	0.7020	0.0076	0.930379	0.0018	0.0007524294
ss65135507	*rs43221236*	1	27.81839	0.2077	0.1080	0.742574	0.0075	0.0024813007
ss86295535		1	27.854013	0.9033	2.0227	0.155414	0.0455	0.0079530993
ss65135776	*rs43222405*	1	27.893559	0.9033	1.9031	0.168175	0.0442	0.0077146866
ss61548201	*rs41646614*	1	28.006219	0.5881	0.5808	0.446249	0.0140	0.0068063657
ss61486868	*rs41586446*	1	28.036958	0.4183	1.1605	0.281732	0.0201	0.0097785653
ss61548202	*rs41646615*	1	28.244438	0.6103	3.4173	0.064939	0.0354	0.0168369666
ss63968679	*rs42412574*	1	28.538947	0.3775	1.2202	0.269710	0.0212	0.0099654529

ss63968835	*rs42413552*	1	28.564086	0.0709	0.2513	0.616342	0.0183	0.0024054776
ss63969055	*rs42414262*	1	28.59399	0.1196	3.8658	0.049676	0.0560	0.0118034286
ss63969735	*rs42408102*	1	28.769749	0.9312	0.0373	0.846860	0.0071	0.0009126763
ss63969781	*rs42408125*	1	28.82376	0.0888	1.0414	0.307840	0.0332	0.0053660475
ss64065929	*rs42454611*	1	28.90024	0.0236	4.8928	0.027293	0.1342	0.0061943850
ss64066071	*rs42455482*	1	28.926714	0.4477	0.0001	0.992065	0.0002	0.0000892385
ss65136431	*rs43223057*	1	29.303973	0.5774	1.7350	0.188210	0.0250	0.0122061479
ss65136409	*rs43223035*	1	29.33036	0.3589	2.0010	0.157641	0.0274	0.0125967006
ss105240450		1	29.353911	0.4706	3.0077	0.083314	0.0330	0.0164239045
ss86276602		1	29.40629	0.6311	1.5397	0.215082	0.0238	0.0110827685
ss105241197		1	29.604866	0.1282	0.0909	0.763122	0.0083	0.0018496826
ss86341264		1	29.642352	0.5910	1.3208	0.250845	0.0221	0.0106642620
ss105241200		1	29.675491	0.2285	0.2005	0.654466	0.0098	0.0034699211
ss61548206	*rs41646619*	1	29.700549	0.3553	0.0304	0.861528	0.0035	0.0015816209
ss65136706	*rs43225332*	1	29.712571	0.3532	0.0273	0.868783	0.0033	0.0014906022
ss65136729	*rs43225355*	1	29.739311	0.4148	0.0186	0.891466	0.0026	0.0012419466
ss64752215	*rs42847849*	1	29.78374	0.6605	0.0104	0.918888	0.0020	0.0009062209
ss64752242	*rs42847876*	1	29.808261	0.5824	0.0943	0.758930	0.0058	0.0027999634
ss86335463		1	29.830061	0.6168	0.8780	0.349066	0.0182	0.0086067580
ss86333573		1	29.864863	0.2837	0.0000	0.995396	0.0001	0.0000483259
ss86305097		1	29.885948	0.7170	1.2105	0.271606	0.0228	0.0092396328
ss65136841	*rs43226767*	1	29.929917	0.8883	0.0739	0.785772	0.0079	0.0015746136
ss61548211	*rs41646624*	1	29.975903	0.6332	1.5539	0.212975	0.0242	0.0112225443
ss65136908	*rs43219334*	1	30.006608	0.7070	0.5492	0.458909	0.0151	0.0062467542
ss105236910		1	30.036543	0.7170	1.3327	0.248718	0.0243	0.0098717516
ss86337187		1	30.155665	0.1748	0.0279	0.867461	0.0041	0.0011879605
ss86332603		1	30.188209	0.7042	0.1033	0.748029	0.0064	0.0026784675
ss86336613		1	30.242626	0.4499	0.0516	0.820346	0.0041	0.0020521059
ss65137376	*rs43224102*	1	30.358495	0.0960	4.4582	0.035089	0.0666	0.0115624787
ss65063768	*rs43153720*	1	30.429565	0.6433	5.5736	0.018508	0.0457	0.0209699290
ss65137466	*rs43225192*	1	30.515559	0.0279	3.2294	0.072762	0.1010	0.0054848585
ss61502983		1	30.619466	0.4792	5.1025	0.024200	0.0420	0.0209664202
ss65137651	*rs43227777*	1	30.660886	0.9778	6.5827	0.010506	0.1600	0.0069497227
ss65137741	*rs43228767*	1	30.729603	0.1913	0.2100	0.646932	0.0110	0.0034000853
ss86305463		1	30.828462	0.2192	5.8031	0.016256	0.0547	0.0187192078
ss105237617		1	30.843173	0.1755	0.7821	0.376798	0.0213	0.0061752129
ss86317184		1	30.878713	0.6633	0.2053	0.650643	0.0090	0.0040161139
ss61526032	*rs41624026*	1	30.905686	0.4821	0.1650	0.684676	0.0073	0.0036484041
ss64004814	*rs42426104*	1	30.970701	0.8087	0.2618	0.609046	0.0120	0.0037140575
ss64004696	*rs42426045*	1	31.017188	0.1934	0.9881	0.320561	0.0228	0.0070990307
ss61563358	*rs41660832*	1	31.039668	0.4878	0.0868	0.768335	0.0054	0.0026755985
ss61538656		1	31.059822	0.6913	0.3136	0.575687	0.0110	0.0047039558
ss61538659	*rs41634839*	1	31.179907	0.6748	0.0512	0.821069	0.0045	0.0019567919
ss65138175	*rs43221498*	1	31.301533	0.4484	7.9924	0.004832	0.0530	0.0262382351
ss86329867		1	31.327686	0.1003	0.0005	0.981937	0.0007	0.0001234028
ss61517671	*rs41618035*	1	31.51418	0.4377	6.7592	0.009525	0.0493	0.0242482694
ss64671833	*rs42764945*	1	31.547313	0.4398	1.1163	0.291076	0.0193	0.0095311476

ss64671794	*rs42764106*	1	31.580518	0.8990	1.1864	0.276428	0.0344	0.0062446554
ss64069477	*rs42461272*	1	31.800715	0.8825	1.7425	0.187257	0.0387	0.0080205572
ss86284486		1	32.023797	0.0924	2.1596	0.142130	0.0483	0.0080953310
ss64859916	*rs42953949*	1	32.110862	0.2529	2.6742	0.102439	0.0352	0.0132849162
ss86311084		1	32.192771	0.8617	3.2681	0.071071	0.0484	0.0115207210
ss64675681	*rs42771270*	1	32.26759	0.4527	1.2472	0.264476	0.0207	0.0102377655
ss64971617	*rs43060141*	1	32.308256	0.4585	1.1835	0.277021	0.0201	0.0099846438
ss86294493		1	32.33409	0.4706	1.3198	0.251027	0.0211	0.0104956908
ss64772905	*rs42868678*	1	32.364123	0.4728	0.6623	0.416043	0.0149	0.0074494190
ss65033825	*rs43123606*	1	32.423821	0.5365	1.7517	0.186097	0.0245	0.0121909252
ss65138565	*rs43225084*	1	32.491089	0.4105	0.4133	0.520495	0.0115	0.0055713364
ss61553526	*rs43712972*	1	32.539929	0.3997	0.7819	0.376866	0.0159	0.0076078560
ss65138649	*rs43226468*	1	32.582467	0.5380	0.8400	0.359721	0.0169	0.0083962312
ss86274548		1	32.61923	0.4585	0.3778	0.539001	0.0112	0.0055796192
ss65138694	*rs43226513*	1	32.641848	0.2149	5.8669	0.015683	0.0525	0.0177089715
ss65138781	*rs43227700*	1	32.712009	0.5415	0.7559	0.384901	0.0161	0.0080050625
ss65138808	*rs43227727*	1	32.75562	0.2149	5.4710	0.019617	0.0517	0.0174588070
ss61553530	*rs43712976*	1	32.846884	0.8782	0.4708	0.492826	0.0195	0.0041694803
ss61489501	*rs41585124*	1	32.878144	0.5301	0.0547	0.815080	0.0044	0.0021775733
ss65138967	*rs43220788*	1	32.949575	0.5523	0.0612	0.804691	0.0046	0.0022724435
ss65139003	*rs43220824*	1	32.987753	0.4513	3.7968	0.051752	0.0363	0.0179915314
ss86311406		1	33.014524	0.7830	4.2218	0.040280	0.0458	0.0155675871
ss65139131	*rs43223452*	1	33.047886	0.4542	4.2999	0.038483	0.0388	0.0192455886
ss65139210	*rs43224231*	1	33.095596	0.6261	8.8508	0.003031	0.0556	0.0260523457
ss65139383	*rs43225704*	1	33.136014	0.6547	5.3124	0.021467	0.0440	0.0199054309
ss61553533	*rs43712979*	1	33.178038	0.4334	1.3287	0.249430	0.0213	0.0104701811
ss65139445	*rs43226666*	1	33.216183	0.4506	0.2927	0.588669	0.0096	0.0047677780
ss65139729	*rs43229429*	1	33.502427	0.3460	0.0434	0.835004	0.0040	0.0018092226
ss86280141		1	33.558636	0.7249	0.0030	0.956075	0.0011	0.0004438628
ss65140927	*rs43229627*	1	33.580543	0.7350	0.0193	0.889652	0.0028	0.0010972019
ss61553552	*rs43712998*	1	33.637325	0.4799	0.0064	0.936134	0.0014	0.0007175714
ss65140242	*rs43226542*	1	33.744104	0.1870	4.4434	0.035394	0.0493	0.0149825927
ss65140339	*rs43227039*	1	33.771636	0.6390	0.1639	0.685742	0.0079	0.0036461385
ss61553556	*rs43713002*	1	33.799178	0.4491	0.3829	0.536233	0.0115	0.0057103852
ss61553558	*rs43713004*	1	33.828832	0.6734	0.9623	0.326945	0.0196	0.0086268652
ss61489517	*rs41585840*	1	33.849426	0.9162	0.1447	0.703768	0.0122	0.0018688614
ss62985582	*rs42066935*	1	34.054463	0.8625	1.5079	0.219871	0.0331	0.0078417076
ss62985798	*rs42067007*	1	34.0858	0.5852	0.1178	0.731563	0.0064	0.0030842391
ss86295334		1	34.194844	0.4169	0.1965	0.657711	0.0081	0.0039559675
ss86320628		1	34.217266	0.6361	0.9036	0.342145	0.0184	0.0085301498
ss61565463	*rs41660815*	1	34.242602	0.5723	0.5483	0.459247	0.0136	0.0066371282
ss61565469	*rs41660821*	1	34.300519	0.2185	0.1921	0.661335	0.0096	0.0032924679
ss117966390		1	34.347535	0.5337	3.9166	0.048205	0.0371	0.0184486350
ss86338872		1	34.407164	0.2314	0.0434	0.834950	0.0045	0.0016137864
ss63054450	*rs42092526*	1	34.512156	0.2013	3.6255	0.057311	0.0434	0.0139584205
	rs29027574	1	34.639391	0.5716	0.6667	0.414473	0.0154	0.0075313879
ss63054650	*rs42086427*	1	34.679429	0.2622	0.2615	0.609283	0.0105	0.0040815584

ss63054893	*rs42088249*	1	34.747729	0.5072	0.3970	0.528827	0.0116	0.0057901264
ss63055164	*rs42088877*	1	34.780065	0.2271	0.0000	0.999800	0.0000	0.0000019285
ss61564367	*rs41660538*	1	34.825272	0.2787	1.8745	0.171405	0.0284	0.0114150322
ss86326578		1	34.846249	0.1039	0.0273	0.868886	0.0049	0.0009106952
ss64981639	*rs43076511*	1	34.927493	0.8023	3.5431	0.060210	0.0454	0.0144058657
ss61549024	*rs41646436*	1	34.98755	0.6798	8.7072	0.003276	0.0574	0.0249797292
ss63820790	*rs42368386*	1	35.047499	0.3009	4.2338	0.040000	0.0412	0.0173263553
ss61507939	*rs41599929*	1	35.080818	0.8646	1.0253	0.311616	0.0276	0.0064557273
ss61530899	*rs41630064*	1	35.107692	0.6798	0.5444	0.460879	0.0144	0.0062719830
ss64907386	*rs42996666*	1	35.130955	0.4262	1.0993	0.294791	0.0195	0.0095343550
ss117963498		1	35.152844	0.3517	0.0424	0.836859	0.0039	0.0017812606
ss63820438	*rs42366110*	1	35.197091	0.1311	1.0342	0.309528	0.0271	0.0061651072
ss105238338		1	35.238665	0.8052	0.8627	0.353294	0.0210	0.0065851503
ss65141279	*rs43226378*	1	35.262097	0.4076	0.0197	0.888518	0.0026	0.0012458671
ss61492102	*rs41594257*	1	35.285632	0.8539	0.0687	0.793246	0.0068	0.0016951040
ss61492103	*rs41594258*	1	35.309775	0.1390	0.0111	0.916200	0.0028	0.0006727337
ss61517760	*rs41618123*	1	35.331614	0.6418	0.1582	0.690895	0.0078	0.0036056851
ss63820310	*rs42366046*	1	35.360433	0.7579	1.3470	0.246199	0.0248	0.0090917568
ss63820252	*rs42365017*	1	35.381802	0.5845	0.1350	0.713438	0.0070	0.0033884523
ss63820172	*rs42364977*	1	35.425847	0.1125	0.3585	0.549547	0.0176	0.0035061351
ss117966070		1	35.480869	0.5107	2.8749	0.090420	0.0313	0.0156494108
ss86331213		1	35.610744	0.4026	1.3946	0.238032	0.0221	0.0106143628
ss65140981	*rs43224981*	1	35.676937	0.4620	1.5625	0.211720	0.0225	0.0111895251
ss86325186		1	35.713131	0.3976	0.1372	0.711145	0.0066	0.0031810009
ss117963056		1	35.756434	0.5881	0.0069	0.933704	0.0015	0.0007367718
ss117974875		1	35.881278	0.2278	0.0053	0.942069	0.0016	0.0005485879
ss65141177	*rs43225776*	1	36.085241	0.1340	0.6657	0.414820	0.0216	0.0050137529
ss65141185	*rs43225784*	1	36.139751	0.2994	2.0130	0.156404	0.0274	0.0114787697
ss86290956		1	36.174882	0.9169	0.2811	0.596136	0.0175	0.0026644062
ss117963022		1	36.37735	0.4685	0.1137	0.736085	0.0059	0.0029545394
ss61510785	*rs41607005*	1	36.404482	0.8782	2.7532	0.097511	0.0471	0.0100834681
ss105317409		1	36.507776	0.7557	5.9199	0.015222	0.0511	0.0188682611
ss65141436	*rs43227235*	1	36.632292	0.4878	0.0592	0.807758	0.0042	0.0021217975
ss65141461	*rs43227260*	1	36.66096	0.6569	1.3447	0.246612	0.0216	0.0097551199
ss61558884	*rs41654534*	1	36.730939	0.9140	1.7757	0.183116	0.0442	0.0069383802
ss86338505		1	36.761877	0.2758	1.1180	0.290707	0.0211	0.0084122369
ss61558883	*rs41654533*	1	36.790108	0.7314	0.5200	0.471077	0.0147	0.0057932865
ss65142265	*rs43228159*	1	36.8319	0.4097	1.3469	0.246215	0.0219	0.0106076143
ss86340576		1	36.8751	0.5122	0.2236	0.636426	0.0086	0.0042844938
ss105279922		1	36.897971	0.5107	0.1823	0.669538	0.0077	0.0038593974
	rs29009759	1	36.925179	0.7872	0.4080	0.523194	0.0141	0.0047234141
ss61558880	*rs41661324*	1	36.958448	0.0466	0.5620	0.453696	0.0329	0.0029234178
ss65141975	*rs43224874*	1	37.028006	0.9198	0.0016	0.968591	0.0014	0.0002017027
ss105237011		1	37.055584	0.9448	0.3617	0.547787	0.0233	0.0024289326
ss86341532		1	37.086738	0.3481	0.1299	0.718666	0.0071	0.0032031307
ss65141700	*rs41257617*	1	37.140939	0.7142	0.5527	0.457480	0.0153	0.0062586911
ss117966443		1	37.243056	0.7708	1.1720	0.279376	0.0243	0.0085838627

ss61478505	*rs41576098*	1	37.438185	0.3481	4.9320	0.026684	0.0432	0.0196181061
ss86331462		1	37.479119	0.7099	0.4202	0.517029	0.0132	0.0054419773
ss61564676	*rs41662840*	1	37.667472	0.6426	0.3246	0.569021	0.0112	0.0051329095
ss117963025		1	37.830245	0.5573	2.3842	0.123022	0.0300	0.0147978430
ss61518088	*rs41611257*	1	37.861523	0.7550	0.2027	0.652669	0.0097	0.0035936255
ss117963028		1	37.890858	0.1583	0.7515	0.386315	0.0223	0.0059481774
ss105237735		1	37.933312	0.3087	0.1999	0.654972	0.0089	0.0038054365
ss61465865	*rs41565131*	1	37.962259	0.6905	1.1840	0.276916	0.0217	0.0092661287
ss61473175	*rs41567595*	1	38.022101	0.2557	0.1596	0.689624	0.0086	0.0032664762
ss86314885		1	38.083012	0.1139	10.1122	0.001538	0.0942	0.0190211955
ss61475204	*rs41572691*	1	38.119282	0.7758	21.4935	0.000004	0.1032	0.0359078180
ss86323797		1	38.24598	0.1383	9.0302	0.002751	0.0809	0.0192787666
ss61564349	*rs41660220*	1	38.277858	0.2185	16.9071	0.000044	0.0928	0.0317062453
ss105240497		1	38.298217	0.1254	6.2424	0.012702	0.0697	0.0152904876
ss61568457	*rs41664554*	1	38.327652	0.2923	0.0004	0.984834	0.0004	0.0001594640
ss61505698	*rs41608833*	1	38.372695	0.0938	0.0424	0.836981	0.0066	0.0011253164
ss86291978		1	38.486179	0.3617	5.2090	0.022771	0.0441	0.0203836175
ss86283358		1	38.620893	0.2543	7.0641	0.008045	0.0562	0.0212957263
ss86332790		1	38.806545	0.5745	0.0039	0.950474	0.0012	0.0005721499
ss86338915		1	38.952267	0.8954	3.6719	0.055746	0.0560	0.0104796293
ss86332733		1	39.186974	0.9069	0.7009	0.402776	0.0265	0.0044820540
ss86329679		1	39.307649	0.1397	6.3118	0.012219	0.0661	0.0158758218
ss86316173		1	39.384792	0.2543	1.4281	0.232477	0.0255	0.0096858202
ss61475939	*rs41578404*	1	39.429582	0.6841	6.7839	0.009395	0.0506	0.0218854016
ss61564232	*rs41659405*	1	39.454544	0.8582	5.9752	0.014756	0.0656	0.0159660083
ss61499736	*rs41594722*	1	39.568802	0.9821	10.3783	0.001334	0.2054	0.0072249809
ss86328662		1	39.6922	0.7479	0.1468	0.701720	0.0081	0.0030362390
ss38329827	*rs29027438*	1	39.716032	0.1196	0.5329	0.465641	0.0217	0.0045794203
ss61472653	*rs41572876*	1	39.882376	0.2120	8.9433	0.002883	0.0686	0.0229125450
ss61474636	*rs41571631*	1	39.933731	0.7550	0.0403	0.840911	0.0044	0.0016289930
ss86308787		1	39.976416	0.6956	4.6220	0.031908	0.0438	0.0185589649
ss61568319	*rs41663117*	1	40.050487	0.1347	1.1042	0.293710	0.0284	0.0066238794
ss105307553		1	40.120711	0.4979	16.8602	0.000045	0.0754	0.0377081158
ss86314786		1	40.150878	0.1182	14.8566	0.000127	0.1108	0.0231021914
ss105237744		1	40.30054	0.1562	13.5623	0.000249	0.0957	0.0252290353
ss61558902	*rs41654552*	1	40.327197	0.5659	9.6761	0.001943	0.0573	0.0281325826
ss105238445		1	40.407179	0.3768	5.2233	0.022586	0.0425	0.0199487772
ss117966227		1	40.566715	0.8403	4.0581	0.044343	0.0507	0.0136150626
ss64827629	*rs42922130*	1	40.604385	0.6676	0.0003	0.985924	0.0004	0.0001572334
ss86294161		1	40.638044	0.7514	2.2013	0.138344	0.0330	0.0123220070
ss61530700	*rs41627671*	1	40.661663	0.8754	7.6060	0.005970	0.0765	0.0166875103
ss86285311		1	40.691263	0.2579	3.0015	0.083629	0.0381	0.0145739548
ss65143035	*rs43226230*	1	40.719347	0.4893	0.0309	0.860604	0.0033	0.0016638000
ss61563380	*rs41660854*	1	40.758983	0.4936	1.4976	0.221454	0.0227	0.0113705441
ss105239127		1	40.878676	0.9319	0.0707	0.790403	0.0098	0.0012484029
ss64823435	*rs42919463*	1	40.904126	0.7314	1.2758	0.259064	0.0239	0.0093953260
ss86299361		1	40.931077	0.4097	2.5100	0.113583	0.0307	0.0148375006

ss65142706	*rs43231497*	1	41.020598	0.6605	0.5181	0.471884	0.0142	0.0063598729
ss65142683	*rs43231474*	1	41.042735	0.6791	0.4362	0.509158	0.0132	0.0057631567
ss63898388	*rs41971077*	1	41.168633	0.5817	0.2358	0.627420	0.0093	0.0045068551
ss61521305	*rs41618224*	1	41.201222	0.2385	0.0394	0.842659	0.0044	0.0016042481
ss63898602	*rs41972284*	1	41.272857	0.6275	0.0132	0.908559	0.0022	0.0010354476
ss61474591	*rs41571086*	1	41.30613	0.8976	0.1836	0.668456	0.0132	0.0024359323
ss86316540		1	41.342674	0.3703	2.4884	0.115145	0.0292	0.0136399331
ss105239151		1	41.395717	0.1884	4.4835	0.034578	0.0492	0.0150529841
ss86341243		1	41.437044	0.4205	2.3785	0.123467	0.0295	0.0143811218
ss86317927		1	41.459257	0.0845	2.2481	0.134228	0.0504	0.0077995443
ss63899230	*rs41965198*	1	41.489565	0.5838	2.0962	0.148112	0.0279	0.0135413284
ss61521307	*rs41618226*	1	41.516047	0.7178	0.7526	0.385964	0.0183	0.0073946882
ss86341778		1	41.564116	0.3338	3.1531	0.076218	0.0350	0.0155763465
ss64968418	*rs43064657*	1	41.600354	0.4692	0.4081	0.523152	0.0117	0.0058510847
ss61473171	*rs41567591*	1	41.630234	0.1132	1.8681	0.172130	0.0374	0.0075070115
ss86299858		1	41.675181	0.9405	0.5695	0.450731	0.0288	0.0032227246
ss64321832	*rs42587534*	1	41.72274	0.4176	1.1612	0.281580	0.0200	0.0097272749
ss117966396		1	41.793197	0.4821	0.0673	0.795315	0.0048	0.0024117337
ss61563861	*rs41664126*	1	41.828459	0.1103	1.3590	0.244118	0.0325	0.0063829646
ss86332260		1	41.888631	0.1440	3.4717	0.062848	0.0464	0.0114290001
ss64509286	*rs42671116*	1	42.047523	0.2593	3.8419	0.050386	0.0405	0.0155680566
ss64509404	*rs42671475*	1	42.082815	0.1483	6.1958	0.013038	0.0638	0.0161220637
ss86335395		1	42.106877	0.8517	6.1958	0.013038	0.0638	0.0161220637
ss86338123		1	42.131682	0.5301	13.6178	0.000242	0.0680	0.0339013524
ss63622908	*rs42295158*	1	42.301092	0.0595	0.3308	0.565351	0.0220	0.0024569891
ss61496351	*rs41598302*	1	42.337111	0.5759	1.2574	0.262531	0.0211	0.0102828328
ss63623082	*rs42295216*	1	42.367798	0.1397	1.6840	0.194821	0.0340	0.0081721939
ss117966109		1	42.389699	0.6411	0.1431	0.705334	0.0074	0.0034086593
ss61567680	*rs41664185*	1	42.488246	0.7772	2.5041	0.114005	0.0350	0.0121122310
ss61496352	*rs41598303*	1	42.548043	0.7407	0.0049	0.943945	0.0015	0.0005620841
ss61567683	*rs41664188*	1	42.612042	0.7772	0.0101	0.919980	0.0022	0.0007609800
ss61532711	*rs41626802*	1	42.676544	0.4785	0.4487	0.503173	0.0124	0.0062010939
ss61531661	*rs41628114*	1	42.714936	0.1225	1.6118	0.204661	0.0360	0.0077375017
ss117966366		1	42.738902	0.4198	2.0441	0.153250	0.0269	0.0130839900
ss64865493	*rs42956975*	1	42.763139	0.7550	1.5887	0.207939	0.0272	0.0100616306
ss61466364	*rs41568119*	1	42.787965	0.5287	1.2081	0.272083	0.0205	0.0101936946
ss63624483	*rs42290796*	1	42.813944	0.7772	2.2938	0.130344	0.0342	0.0118324094
ss86299697		1	42.875764	0.4019	1.4376	0.230936	0.0229	0.0109951755
	rs29019617	1	42.906017	0.2056	3.0424	0.081558	0.0405	0.0132253963
ss86309988		1	42.937352	0.5824	1.4316	0.231918	0.0226	0.0110095538
ss86341467		1	43.030542	0.3954	0.3227	0.570171	0.0109	0.0052265650
ss86294850		1	43.053683	0.5845	1.4961	0.221693	0.0234	0.0113436866
	rs29011297	1	43.082467	0.7192	0.2198	0.639336	0.0097	0.0039121289
ss61522921	*rs41622790*	1	43.137145	0.1791	0.6015	0.438262	0.0186	0.0054701133
ss65143565	*rs43232559*	1	43.173391	0.9241	0.9666	0.325875	0.0328	0.0045976413
ss105241305		1	43.216667	0.1519	1.2359	0.266637	0.0290	0.0074794450
ss86315871		1	43.259209	0.2887	0.0822	0.774444	0.0060	0.0024725109

ss117966452		1	43.280023	0.8023	4.1046	0.043148		0.0476	0.0150884393
ss65143771	*rs43234665*	1	43.303024	0.7830	0.7373	0.390812		0.0194	0.0066057663
ss61564513	*rs41661483*	1	43.391348	0.7822	0.3581	0.549730		0.0135	0.0046148199
ss64726487	*rs42821384*	1	43.461071	0.3259	4.6978	0.030540		0.0411	0.0180404064
ss86287140		1	43.483581	0.7543	1.8621	0.172828		0.0290	0.0107537658
ss64971059	*rs43065183*	1	43.522084	0.2529	4.2864	0.038787		0.0437	0.0165054278
ss61471354	*rs41569323*	1	43.622399	0.4105	0.4908	0.483806		0.0132	0.0063960962
ss86311107		1	43.642684	0.8489	10.4129	0.001310		0.0794	0.0203649666
ss86306407		1	43.67472	0.8481	13.8751	0.000211		0.0913	0.0235123135
ss86323154		1	43.728415	0.6719	3.7313	0.053807		0.0377	0.0166327102
ss61531094	*rs41632057*	1	43.760388	0.1597	9.3948	0.002260		0.0742	0.0199105528
ss65143857	*rs43235851*	1	43.817826	0.7400	0.6386	0.424481		0.0166	0.0063940756
ss86313477		1	43.866603	0.8044	0.0158	0.900079		0.0029	0.0009035661
	rs29010070	1	43.944467	0.8352	8.3665	0.003941		0.0711	0.0195696742
ss65143978	*rs43229073*	1	44.064307	0.2278	1.1343	0.287237		0.0240	0.0084450749
ss86319475		1	44.092636	0.2385	0.0402	0.841097		0.0045	0.0016187200
ss65144042	*rs43229736*	1	44.114981	0.1569	0.4714	0.492577		0.0174	0.0046022129
ss86299767		1	44.192991	0.9470	4.9808	0.025948		0.0938	0.0094218336
	rs29018366	1	44.240246	0.1010	3.7257	0.053987		0.0588	0.0106776439
ss105242018		1	44.310182	0.1590	4.3236	0.037953		0.0530	0.0141731829
ss61564526	*rs41661496*	1	44.486455	0.5580	0.3964	0.529136		0.0119	0.0058679813
ss86319701		1	44.538807	0.6834	2.1795	0.140309		0.0298	0.0128990366
	rs29013984	1	44.640563	0.6913	3.8995	0.048695		0.0400	0.0170920630
ss61527574	*rs41625031*	1	44.66757	0.3832	2.5683	0.109476		0.0309	0.0146200923
ss61494841	*rs41590428*	1	44.68927	0.1805	0.1497	0.698958		0.0095	0.0028216781
ss65144536	*rs43233430*	1	44.713675	0.2299	7.6463	0.005840		0.0611	0.0216268138
ss61564535	*rs41661505*	1	44.739865	0.2299	7.6463	0.005840		0.0611	0.0216268138
ss61494847	*rs41591434*	1	44.861513	0.5143	0.2915	0.589427		0.0100	0.0049775182
ss86333104		1	44.894257	0.7787	1.5714	0.210423		0.0278	0.0095947641
ss86326953		1	44.931182	0.3016	8.5686	0.003532		0.0589	0.0247951510
ss86334904		1	44.961871	0.3732	0.4137	0.520303		0.0122	0.0057104543
ss65285871	*rs43373834*	1	44.99084	0.7607	2.8286	0.093046		0.0368	0.0133938327
ss86336725		1	45.063751	0.2951	0.9594	0.327679		0.0201	0.0083803483
ss86327189		1	45.088246	0.3066	1.8994	0.168592		0.0280	0.0118880897
ss61564541	*rs41661511*	1	45.128701	0.7758	1.2174	0.270246		0.0239	0.0083098130
ss117963569		1	45.151263	0.3961	0.6757	0.411368		0.0155	0.0074076944
ss86338328		1	45.25496	0.1332	1.3314	0.248952		0.0313	0.0072378824
ss86339453		1	45.326137	0.4649	5.6735	0.017490		0.0430	0.0213725364
	rs29025772	1	45.382464	0.3775	9.4358	0.002211		0.0600	0.0281887208
ss61564549	*rs41661519*	1	45.412401	0.9556	3.6856	0.055292		0.0845	0.0071691286
ss86288219		1	45.512563	0.8216	2.9159	0.088155		0.0412	0.0120760994
ss65145519	*rs43232409*	1	45.536994	0.8352	2.0011	0.157634		0.0354	0.0097395309
ss65145529	*rs43232419*	1	45.647138	0.3173	5.3718	0.020754		0.0459	0.0198867847
ss61477193	*rs41575440*	1	45.986516	0.4004	8.2318	0.004241		0.0543	0.0260727022
ss86305829		1	46.151959	0.0638	0.1984	0.656154		0.0168	0.0020008527
ss86323406		1	46.199933	0.2765	4.1412	0.042230		0.0418	0.0167405157
ss61496275	*rs41597527*	1	46.229321	0.2328	0.2691	0.604116		0.0113	0.0040438294

ss64321334	*rs42584386*	1	46.291678	0.9126	2.4887	0.115124	0.0514	0.0082058087
ss64321396	*rs42584417*	1	46.323	0.2314	0.3239	0.569454	0.0126	0.0044724834
ss86298363		1	46.346236	0.7013	3.3317	0.068384	0.0364	0.0152450320
ss86336731		1	46.368854	0.4620	0.6559	0.418282	0.0149	0.0074036473
ss61514172	*rs41610345*	1	46.391244	0.7156	0.1420	0.706411	0.0076	0.0031090469
ss86338958		1	46.434313	0.4577	3.0974	0.078855	0.0320	0.0159040492
ss65601492	*rs43682200*	1	46.459715	0.6669	0.0936	0.759726	0.0061	0.0026902603
ss65601480	*rs43682189*	1	46.488917	0.3754	2.9661	0.085472	0.0328	0.0153712867
ss65601453	*rs43682162*	1	46.536856	0.1812	0.0054	0.941313	0.0018	0.0005208957
ss86319004		1	46.565191	0.1848	6.1494	0.013381	0.0589	0.0177493567
ss64799303	*rs42899845*	1	46.591594	0.1691	0.1695	0.680674	0.0101	0.0028322569
ss61495000	*rs41592484*	1	46.627511	0.6626	5.5399	0.018864	0.0457	0.0204155999
ss86288239		1	46.663777	0.1182	5.7271	0.016969	0.0656	0.0136670768
ss64320944	*rs42582291*	1	46.68984	0.3467	4.5510	0.033248	0.0419	0.0190028117
ss64320914	*rs42582276*	1	46.72096	0.5609	7.7662	0.005468	0.0505	0.0248918128
ss86277249		1	46.801013	0.9284	2.8018	0.094607	0.0574	0.0076385444
	rs29016414	1	46.881368	0.0723	1.3111	0.252598	0.0392	0.0052615589
ss61567519	*rs41663226*	1	46.901459	0.1139	0.8724	0.350626	0.0271	0.0054603456
ss64803210	*rs42896423*	1	46.955851	0.1941	2.9411	0.086797	0.0401	0.0125509144
	rs29023125	1	47.016799	0.9277	1.3111	0.252598	0.0392	0.0052615589
ss61564603		1	47.04523	0.2307	0.7168	0.397493	0.0187	0.0066296315
ss61494867	*rs41591454*	1	47.065261	0.6712	3.6137	0.057717	0.0382	0.0168709335
ss46526251	*rs41255390*	1	47.121527	0.1297	1.6354	0.201379	0.0367	0.0082737820
ss117963492		1	47.153851	0.6712	3.6137	0.057717	0.0382	0.0168709335
ss65591692	*rs43671733*	1	47.187443	0.0723	1.2442	0.265050	0.0382	0.0051253869
ss65591681	*rs43671022*	1	47.220241	0.2342	1.3341	0.248474	0.0254	0.0091203491
ss65591671	*rs43671012*	1	47.270174	0.0702	1.7583	0.185267	0.0480	0.0062608088
ss65591658	*rs43670999*	1	47.307922	0.0795	3.3249	0.068668	0.0628	0.0091912664
ss86340057		1	47.347125	0.6504	0.4341	0.510178	0.0127	0.0057928715
ss86297409		1	47.372647	0.2801	0.0354	0.850719	0.0039	0.0015780574
ss65591624	*rs43670966*	1	47.400089	0.0745	2.2503	0.134040	0.0530	0.0073088197
ss65591615	*rs43670957*	1	47.427314	0.6297	0.0413	0.838948	0.0039	0.0018030664
ss61564599	*rs41662169*	1	47.466444	0.6361	0.1589	0.690290	0.0077	0.0035620465
ss64615912	*rs42723377*	1	47.53676	0.6254	0.0069	0.933673	0.0016	0.0007452562
ss86299634		1	47.565937	0.7249	0.0004	0.983840	0.0004	0.0001656779
ss61472541	*rs41571566*	1	47.694704	0.3460	0.0002	0.990046	0.0002	0.0001120804
ss61516428	*rs41615838*	1	47.778312	0.5645	0.5325	0.465798	0.0135	0.0066403762
	rs29018839	1	47.904413	0.6640	10.8244	0.001052	0.0637	0.0284208696
ss86298555		1	47.924986	0.6977	0.3628	0.547126	0.0120	0.0050517501
ss86323743		1	48.053651	0.2679	0.0022	0.962381	0.0010	0.0003844641
ss61569511	*rs41667890*	1	48.097917	0.9062	6.1299	0.013528	0.0773	0.0131409674
ss61505821	*rs41599954*	1	48.122471	0.1067	5.8596	0.015747	0.0703	0.0134029408
ss105251701		1	48.191476	0.2937	6.3250	0.012129	0.0524	0.0217218098
	rs29012753	1	48.219435	0.5229	6.6300	0.010233	0.0478	0.0238384521
ss65104660	*rs43192760*	1	48.248142	0.4011	14.4574	0.000156	0.0726	0.0348935249
ss64720093	*rs42813614*	1	48.281401	0.2457	5.4023	0.020397	0.0499	0.0185134567
ss64542922	*rs42688785*	1	48.327003	0.5337	0.4901	0.484106	0.0131	0.0065331517

ss61471396	*rs41570065*	1	48.35495	0.5115	3.3652	0.067012	0.0338	0.0169112697
ss64983782	*rs43081412*	1	48.471746	0.4606	4.4517	0.035222	0.0399	0.0198353982
ss61473123	*rs41567544*	1	48.54774	0.7500	11.4758	0.000745	0.0728	0.0273092235
ss63803448	*rs42362949*	1	48.640548	0.4857	4.0410	0.044792	0.0372	0.0186073852
ss61524141	*rs41625686*	1	48.665089	0.5480	5.4165	0.020233	0.0423	0.0209609229
	rs29024905	1	48.689181	0.2944	0.1278	0.720866	0.0072	0.0029749103
ss63803614	*rs42363029*	1	48.771337	0.4069	0.1053	0.745684	0.0061	0.0029232506
ss117966210		1	48.799607	0.8360	0.0007	0.978253	0.0007	0.0001890579
ss86334699		1	48.853132	0.4857	6.3921	0.011683	0.0467	0.0233394804
ss86275064		1	48.889678	0.7479	2.7293	0.098973	0.0360	0.0135902263
ss86304371		1	48.933291	0.7765	4.2816	0.038895	0.0468	0.0162376701
ss105243316		1	48.958005	0.2299	1.2821	0.257895	0.0252	0.0089171085
ss61467072	*rs41565305*	1	49.022709	0.8102	0.1512	0.697532	0.0096	0.0029525316
ss86281719		1	49.2212	0.2722	1.9438	0.163698	0.0287	0.0113572253
	rs29024583	1	49.302477	0.5372	2.1476	0.143245	0.0270	0.0134417833
ss86298472		1	49.403484	0.4348	0.6769	0.410943	0.0151	0.0074448499
ss86317301		1	49.481803	0.3166	0.0377	0.846119	0.0039	0.0016957199
ss64663284	*rs42759062*	1	49.589647	0.8768	13.2608	0.000291	0.0990	0.0213866630
ss63656634	*rs42304080*	1	49.614529	0.8467	0.0126	0.910584	0.0030	0.0007826271
ss63656334	*rs42303280*	1	49.690525	0.3460	0.0200	0.887690	0.0028	0.0012826952
ss61518722	*rs41617274*	1	49.754204	0.6633	1.6022	0.206019	0.0250	0.0111584698
ss86279903		1	49.858607	0.3954	0.4498	0.502652	0.0126	0.0060290358
ss63803430	*rs42362940*	1	49.875245	0.8854	0.6796	0.410017	0.0235	0.0047731476
ss61518709	*rs41617261*	1	49.925039	0.2958	0.7405	0.389794	0.0175	0.0072991741
ss63448381	*rs42234990*	1	49.971484	0.6024	0.0186	0.891528	0.0026	0.0012281410
ss63448075	*rs42233888*	1	50.008816	0.3976	0.2084	0.648163	0.0086	0.0041096229
ss61471104	*rs41567176*	1	50.169471	0.9047	0.2952	0.587060	0.0160	0.0027637151
ss63447265	*rs42240515*	1	50.222674	0.1282	3.6492	0.056506	0.0533	0.0119136956
ss61518695	*rs41617247*	1	50.261396	0.8689	2.9875	0.084353	0.0479	0.0109190369
ss105241578		1	50.282113	0.8438	0.2165	0.641850	0.0113	0.0029881349
ss65256754	*rs43344446*	1	50.319323	0.9119	0.9924	0.319497	0.0330	0.0053073117
ss61519660	*rs41616907*	1	50.373059	0.2493	0.0113	0.915541	0.0022	0.0008321800
ss61512070	*rs41610080*	1	50.441312	0.1870	3.0203	0.082668	0.0398	0.0120886307
ss65146369	*rs43234048*	1	50.531108	0.0974	2.3065	0.129289	0.0478	0.0084004466
ss65146414	*rs43234093*	1	50.555211	0.9026	2.3065	0.129289	0.0478	0.0084004466
ss63711264	*rs42327967*	1	50.586006	0.9026	2.3065	0.129289	0.0478	0.0084004466
ss63711231	*rs42327956*	1	50.631826	0.1884	4.4036	0.036222	0.0496	0.0151728481
ss61470665	*rs41572341*	1	50.69514	0.2307	6.5108	0.010935	0.0551	0.0195683128
ss117966270		1	50.74265	0.8582	8.3869	0.003898	0.0736	0.0179183391
ss63710745	*rs42326004*	1	50.801119	0.2342	0.2863	0.592759	0.0115	0.0041219400
	rs29012790	1	50.853398	0.1841	1.4733	0.225244	0.0289	0.0086898496
ss65146624	*rs43235803*	1	50.937218	0.4893	3.1124	0.078137	0.0330	0.0165157895
ss65146648	*rs43235827*	1	50.97871	0.9105	0.1137	0.736080	0.0106	0.0017335545
ss61470119	*rs41567114*	1	51.00614	0.5158	2.3896	0.122602	0.0287	0.0143238992
ss86332464		1	51.124342	0.0716	0.2928	0.588614	0.0188	0.0025059998
ss86331292		1	51.163887	0.2536	2.2584	0.133343	0.0327	0.0123919700
ss86320435		1	51.300175	0.0759	0.0893	0.765207	0.0099	0.0013844891

ss86274201		1	51.34236	0.0860	2.4752	0.116109		0.0531	0.0083502945
ss61568881	*rs41667262*	1	51.389105	0.3725	0.0019	0.965199		0.0008	0.0003930441
ss86337620		1	51.460268	0.0967	3.4040	0.065462		0.0567	0.0099045446
ss86282551		1	51.506987	0.2600	1.2855	0.257265		0.0242	0.0092981029
ss64679882	*rs42774937*	1	51.534244	0.3016	0.3145	0.575111		0.0113	0.0047466663
ss64004398	*rs42424497*	1	51.702373	0.1640	4.8539	0.027911		0.0504	0.0138190728
ss86310054		1	51.737681	0.6991	0.8461	0.357990		0.0187	0.0078815432
ss64004670	*rs42426032*	1	51.793142	0.8517	0.0132	0.908536		0.0030	0.0007454396
ss117963526		1	51.859933	0.4176	3.2658	0.071170		0.0343	0.0166958719
	rs29027151	1	51.933754	0.9448	0.1880	0.664719		0.0175	0.0018214066
ss65147862	*rs43236036*	1	51.972286	0.6841	0.1088	0.741648		0.0066	0.0028736360
	rs29022483	1	52.01299	0.6655	0.4154	0.519439		0.0127	0.0056326476
ss65147664	*rs43234743*	1	52.066861	0.1103	4.7459	0.029704		0.0636	0.0124815817
ss61505886	*rs41600017*	1	52.172542	0.4828	0.6190	0.431700		0.0142	0.0070869247
ss86315945		1	52.217229	0.5201	0.0108	0.917082		0.0019	0.0009402817
ss86292320		1	52.272441	0.3023	2.0762	0.150059		0.0279	0.0117530417
ss61531213	*rs41633172*	1	52.328173	0.4434	4.9960	0.025723		0.0414	0.0204493710
ss117966103		1	52.388164	0.2550	1.8003	0.180118		0.0276	0.0105042121
ss117966055		1	52.539817	0.5079	5.0572	0.024836		0.0425	0.0212669519
ss86298416		1	52.561031	0.4155	0.9607	0.327353		0.0180	0.0087513837
ss64761135	*rs42854774*	1	52.582865	0.7636	3.0281	0.082274		0.0379	0.0136708111
ss117966437		1	52.687802	0.6139	0.0565	0.812175		0.0045	0.0021376058
ss105244421		1	52.757943	0.8288	2.3922	0.122400		0.0389	0.0110381277
ss86335826		1	52.795446	0.1948	2.3096	0.129032		0.0351	0.0110133694
ss61470126	*rs41567121*	1	52.827029	0.5544	1.6472	0.199774		0.0233	0.0114949646
ss105250813		1	52.847366	0.5215	0.6680	0.414026		0.0149	0.0074403346
ss61512091	*rs41610101*	1	52.870294	0.4291	0.4190	0.517638		0.0120	0.0058867117
ss61522929	*rs41622798*	1	52.898767	0.4327	0.0400	0.841511		0.0037	0.0018166647
ss61522930	*rs41622799*	1	52.952325	0.3546	0.0016	0.967694		0.0008	0.0003534171
ss86319687		1	52.984946	0.5079	0.6715	0.412821		0.0150	0.0074848901
ss65150259	*rs43234157*	1	53.025392	0.5817	3.0354	0.081908		0.0327	0.0159147497
ss86306806		1	53.102123	0.4792	0.6495	0.420575		0.0148	0.0074074139
ss63610026	*rs42290710*	1	53.313134	0.8202	0.1755	0.675439		0.0103	0.0030255858
ss61495935	*rs41594493*	1	53.341464	0.2335	0.0460	0.830215		0.0047	0.0016896976
ss61566777	*rs41667398*	1	53.42403	0.4542	0.0744	0.785092		0.0051	0.0025201185
ss63609408	*rs42289209*	1	53.451901	0.0766	2.7596	0.097120		0.0579	0.0081997526
ss61566780	*rs41667401*	1	53.484914	0.1612	0.4309	0.511751		0.0166	0.0044799764
ss61469636	*rs41572133*	1	53.549172	0.1970	0.1354	0.713012		0.0085	0.0026893106
ss61477302	*rs41575847*	1	53.598731	0.3754	0.0519	0.819854		0.0043	0.0019951166
ss61507486	*rs41606679*	1	53.671558	0.5365	0.6245	0.429663		0.0151	0.0075064806
ss64028889	*rs42438803*	1	53.723264	0.7378	0.3170	0.573584		0.0120	0.0046577093
ss86316695		1	53.793026	0.1576	3.8430	0.050353		0.0498	0.0132343662
ss61466702		1	53.822907	0.5867	0.4385	0.508081		0.0121	0.0058843742
ss86325000		1	53.880017	0.7070	1.8870	0.169986		0.0279	0.0115726675
ss64029087	*rs42439402*	1	53.907613	0.1468	3.2830	0.070431		0.0473	0.0118530595
ss64729840	*rs42830001*	1	53.936173	0.3911	0.0950	0.757957		0.0058	0.0027729087
ss64729850	*rs42830011*	1	53.960828	0.8539	2.7252	0.099225		0.0434	0.0108377829

ss86341599		1	54.006177	0.3875	0.0539	0.816554	0.0044	0.0020905477
ss86335207		1	54.185146	0.8567	0.9101	0.340429	0.0249	0.0061050259
ss86337179		1	54.219097	0.4456	0.0026	0.959153	0.0009	0.0004645922
ss61475098	*rs41571491*	1	54.252126	0.8403	7.8277	0.005287	0.0685	0.0183941662
ss86300387		1	54.274662	0.8582	7.8719	0.005161	0.0718	0.0174678115
ss65341285	*rs43422840*	1	54.317874	0.0774	1.5835	0.208686	0.0430	0.0061391372
ss117963539		1	54.386908	0.7615	0.7978	0.372056	0.0191	0.0069240426
ss86316738		1	54.407044	0.2056	0.3403	0.559822	0.0133	0.0043366734
ss86339185		1	54.436598	0.8238	0.0768	0.781748	0.0068	0.0019619060
ss61517298	*rs41613887*	1	54.461363	0.1332	1.1146	0.291458	0.0285	0.0065713685
ss61465830	*rs41569974*	1	54.514534	0.4878	0.1036	0.747691	0.0059	0.0029455310
ss65150842	*rs43239430*	1	54.591712	0.6848	0.6443	0.422447	0.0159	0.0068494012
ss86288001		1	54.638743	0.6203	0.0290	0.864899	0.0032	0.0015222446
ss65151009	*rs43241897*	1	54.667969	0.1046	0.1962	0.657911	0.0131	0.0024506190
	rs29023044	1	54.695761	0.6053	0.1790	0.672366	0.0081	0.0038526727
ss105247656		1	54.717464	0.8274	1.2724	0.259703	0.0272	0.0077838977
ss61569689	*rs41669553*	1	54.73819	0.4105	3.3585	0.067284	0.0349	0.0168931452
	rs29023662	1	54.775124	0.1605	8.0265	0.004743	0.0735	0.0198102892
	rs29019635	1	54.801752	0.3883	1.1590	0.282040	0.0207	0.0098506251
ss64923573	*rs43020781*	1	54.915237	0.8632	0.9775	0.323161	0.0258	0.0061030161
ss86279792		1	54.993939	0.7643	5.1206	0.023950	0.0508	0.0183133341
ss61517267	*rs41613861*	1	55.068452	0.8503	0.5739	0.448953	0.0192	0.0048797403
ss86325009		1	55.091201	0.6691	0.1567	0.692357	0.0079	0.0034962854
ss86305181		1	55.117569	0.3825	1.5160	0.218647	0.0236	0.0111599085
ss61472799	*rs41574018*	1	55.175718	0.2808	0.4168	0.518752	0.0133	0.0053579405
ss61472800	*rs41574019*	1	55.206939	0.3016	0.2246	0.635738	0.0096	0.0040649602
ss65149392	*rs43236439*	1	55.229661	0.0695	1.4602	0.227315	0.0430	0.0055629169
ss65149454	*rs43236501*	1	55.394737	0.8789	1.1660	0.280602	0.0314	0.0066823169
ss61517284	*rs41613873*	1	55.513136	0.3252	0.0261	0.871623	0.0033	0.0014352412
ss117966126		1	55.673836	0.5788	0.0122	0.912110	0.0020	0.0009992699
ss117966202		1	55.694419	0.6877	0.1318	0.716709	0.0072	0.0030866461
ss86335520		1	56.015336	0.5759	3.6629	0.056046	0.0353	0.0172348916
ss117966319		1	56.041804	0.7701	9.1598	0.002565	0.0666	0.0235933492
ss86315731		1	56.063826	0.3123	1.1162	0.291110	0.0212	0.0090890232
ss61495936	*rs41594494*	1	56.188979	0.7400	0.0764	0.782359	0.0059	0.0022867486
ss117966347		1	56.254239	0.7643	1.2950	0.255512	0.0248	0.0089336826
ss86303260		1	56.343907	0.8961	0.0089	0.924712	0.0029	0.0005424781
ss86339433		1	56.552777	0.7729	0.4566	0.499449	0.0154	0.0053974555
ss86317746		1	56.765215	0.9112	0.0148	0.903315	0.0039	0.0006393042
ss61478283	*rs41575389*	1	56.809585	0.0645	0.4396	0.507515	0.0248	0.0029912728
ss86328794		1	56.871053	0.8933	0.3434	0.558071	0.0177	0.0033817188
ss86305129		1	56.906467	0.8940	0.2697	0.603680	0.0158	0.0029863997
ss61530142	*rs41631827*	1	56.928253	0.8947	0.2361	0.627195	0.0148	0.0027835519
ss61478286	*rs41575392*	1	56.964509	0.1067	0.2907	0.589975	0.0163	0.0031114433
ss86339056		1	57.09755	0.7034	5.9516	0.014953	0.0502	0.0209626205
ss86303425		1	57.143017	0.2966	5.7112	0.017122	0.0490	0.0204549244
ss86289454		1	57.180148	0.4928	7.0378	0.008162	0.0493	0.0246427018

ss86307790		1	57.260972	0.9534	2.9155	0.088178	0.0727	0.0064523075
ss86282421		1	57.330357	0.6168	0.8653	0.352570	0.0180	0.0085238840
ss61478260	*rs41575366*	1	57.421048	0.5781	0.4977	0.480768	0.0133	0.0065013822
ss86273594		1	57.450474	0.7436	5.3529	0.020979	0.0509	0.0194296617
ss117963521		1	57.47164	0.5258	1.4086	0.235693	0.0219	0.0109305561
ss86314501		1	57.512973	0.6547	7.5799	0.006056	0.0540	0.0244105564
	rs29019616	1	57.616025	0.5308	2.4516	0.117859	0.0297	0.0147844057
ss61478182	*rs41574696*	1	57.702385	0.4613	2.1446	0.143525	0.0277	0.0137617355
ss61478188	*rs41574702*	1	57.774377	0.6175	2.7314	0.098846	0.0316	0.0149414702
ss117966117		1	57.798609	0.8489	1.4676	0.226143	0.0323	0.0082866753
ss86280841		1	57.913624	0.4570	0.0706	0.790563	0.0050	0.0024721828
ss61478189	*rs41574703*	1	57.95103	0.3510	2.9054	0.088732	0.0329	0.0149705637
ss86331322		1	57.98282	0.8481	2.3901	0.122563	0.0412	0.0106009947
ss46527080	*rs41255623*	1	58.042638	0.6934	17.5339	0.000032	0.0822	0.0349343170
ss61478192	*rs41574706*	1	58.102775	0.3016	19.2043	0.000014	0.0860	0.0362267725
ss86339163		1	58.180914	0.8453	0.7570	0.384568	0.0229	0.0060016676
ss86340931		1	58.202087	0.0788	10.7178	0.001114	0.1114	0.0161732947
ss86317645		1	58.229631	0.1533	0.9886	0.320429	0.0264	0.0068622046
ss61530181	*rs41632565*	1	58.47808	0.8438	1.1382	0.286394	0.0281	0.0073946672
ss86336337		1	58.773522	0.1519	1.0450	0.307022	0.0271	0.0069739642
ss86313052		1	58.815378	0.5781	0.0566	0.812008	0.0045	0.0021942740
ss86294250		1	59.075284	0.3947	3.5079	0.061494	0.0360	0.0172135606
ss117974889		1	59.101517	0.5924	1.2458	0.264744	0.0206	0.0099394914
ss117974886		1	59.128232	0.3596	0.1571	0.691998	0.0079	0.0036308730
ss86330064		1	59.166286	0.6554	5.9051	0.015349	0.0452	0.0204153370
	rs29010381	1	59.323901	0.2579	9.2391	0.002458	0.0643	0.0246008601
ss61511908	*rs41616212*	1	59.374583	0.2479	0.3775	0.539133	0.0127	0.0047406384
ss61496709	*rs41592061*	1	59.460639	0.3309	3.9990	0.045916	0.0381	0.0168695296
ss61568362	*rs41663660*	1	59.607414	0.2615	3.3511	0.067588	0.0391	0.0150810987
ss61479775	*rs41582534*	1	59.664885	0.5953	0.3187	0.572599	0.0107	0.0051351209
ss61568892	*rs41667273*	1	59.71897	0.9549	1.0219	0.312419	0.0449	0.0038729583
ss117963476		1	59.799328	0.3961	6.3549	0.011928	0.0480	0.0229725364
ss117963478		1	59.825125	0.4592	7.5463	0.006169	0.0521	0.0258634993
ss86298222		1	59.853274	0.1060	2.0510	0.152558	0.0434	0.0082206664
ss65152391	*rs43237971*	1	59.951163	0.9183	1.0859	0.297754	0.0352	0.0052784858
ss64884762	*rs42973922*	1	59.981024	0.7973	0.4291	0.512671	0.0157	0.0050678067
ss65152492	*rs43238971*	1	60.037926	0.6626	0.1798	0.671646	0.0085	0.0037928686
ss61479776	*rs41582535*	1	60.0731	0.2987	5.3918	0.020520	0.0475	0.0199168284
ss86325115		1	60.09975	0.7658	2.0082	0.156896	0.0315	0.0112911734
	rs29027437	1	60.152426	0.3589	0.0191	0.890216	0.0027	0.0012417322
ss65152637	*rs43239615*	1	60.178481	0.3789	0.0834	0.772780	0.0056	0.0026196333
ss65152660	*rs43240138*	1	60.205898	0.5086	0.0048	0.944910	0.0013	0.0006420197
ss61533227	*rs41630790*	1	60.357503	0.3223	2.9220	0.087827	0.0350	0.0152964682
ss65152901	*rs43241979*	1	60.394453	0.3818	2.6424	0.104496	0.0316	0.0149304855
ss65152848	*rs43241226*	1	60.513895	0.0953	0.8226	0.364743	0.0285	0.0049132188
ss65152801	*rs43241179*	1	60.548242	0.8968	1.6549	0.198715	0.0393	0.0072787618
ss61499516	*rs43710703*	1	60.572493	0.2278	2.6014	0.107225	0.0353	0.0124190533

ss61479782	*rs41582541*	1	60.619267	0.3259	5.4989	0.019309	0.0474	0.0208268791
ss86336402		1	60.663284	0.7665	2.5442	0.111154	0.0353	0.0126309338
ss61508045	*rs41601432*	1	60.686366	0.1762	1.8656	0.172417	0.0337	0.0097952319
ss65153191	*rs43237670*	1	60.751595	0.9198	1.4288	0.232372	0.0407	0.0060043340
ss61479784	*rs41582543*	1	60.809665	0.2436	0.0846	0.771266	0.0060	0.0022286530
	rs29014975	1	60.842756	0.2450	5.2076	0.022789	0.0502	0.0185851463
ss86311714		1	60.8659	0.5451	1.4283	0.232450	0.0213	0.0105665384
ss86336152		1	60.908248	0.4470	0.1571	0.691987	0.0073	0.0036269165
ss64225230	*rs42541634*	1	60.991916	0.1282	0.1974	0.656953	0.0121	0.0027123050
ss65024617	*rs43114735*	1	61.182531	0.1748	0.3878	0.533675	0.0151	0.0043691551
ss61528709	*rs41628641*	1	61.216644	0.2407	0.0271	0.869191	0.0035	0.0012970327
ss86339068		1	61.245263	0.2643	1.3746	0.241427	0.0243	0.0094499765
ss117963541		1	61.282581	0.3302	0.0730	0.787040	0.0053	0.0023607838
ss117963518		1	61.309669	0.0652	0.6307	0.427369	0.0289	0.0035239846
ss86294257		1	61.351329	0.6862	0.1363	0.712099	0.0077	0.0033096281
ss46526391	*rs41255529*	1	61.37741	0.3546	0.8065	0.369469	0.0178	0.0081442379
ss86294004		1	61.404517	0.3367	0.0003	0.985261	0.0004	0.0001638074
ss65153920	*rs43243495*	1	61.446735	0.8388	0.9770	0.323279	0.0245	0.0066331040
ss61479790	*rs41582549*	1	61.489778	0.4312	0.1050	0.746013	0.0063	0.0030709910
ss61533258	*rs41630820*	1	61.523008	0.6433	1.2868	0.257038	0.0223	0.0102250561
ss86287861		1	61.576493	0.2450	0.8213	0.365104	0.0203	0.0075092271
ss117966409		1	61.609955	0.4986	5.5029	0.019265	0.0427	0.0213568427
ss86326973		1	61.648932	0.6999	0.6103	0.434943	0.0158	0.0066186726
ss86324603		1	61.707544	0.2113	1.4023	0.236742	0.0269	0.0089510356
ss61525318	*rs41620345*	1	61.749988	0.0903	4.1047	0.043146	0.0664	0.0109121941
ss65021416	*rs43113585*	1	61.781774	0.1583	4.0522	0.044499	0.0516	0.0137483178
	rs29016420	1	61.816725	0.2586	2.8556	0.091504	0.0345	0.0132306994
ss105246696		1	61.960503	0.2629	0.9365	0.333521	0.0206	0.0080030138
ss86291663		1	61.980382	0.7371	0.9365	0.333521	0.0206	0.0080030138
ss61480027		1	62.018974	0.8080	0.3009	0.583514	0.0129	0.0039985983
ss65155380	*rs43239756*	1	62.041329	0.9019	2.5446	0.111122	0.0500	0.0088441338
ss61495499	*rs41597969*	1	62.088777	0.9019	2.5446	0.111122	0.0500	0.0088441338
ss65155213	*rs43238189*	1	62.200612	0.2049	6.1893	0.013086	0.0549	0.0178951318
ss61508103	*rs41601490*	1	62.229404	0.3574	0.3065	0.580022	0.0105	0.0048275197
ss61533263	*rs41630825*	1	62.290491	0.1232	2.2310	0.135721	0.0418	0.0090218849
ss65154953	*rs43245227*	1	62.319107	0.1526	4.9340	0.026654	0.0569	0.0147030457
ss86328156		1	62.347548	0.0659	8.5030	0.003660	0.1041	0.0128148809
	rs29018897	1	62.453902	0.5602	5.7426	0.016821	0.0463	0.0228217449
ss86294598		1	62.561108	0.5974	0.7135	0.398580	0.0157	0.0075283265
ss61513853		1	62.700888	0.1583	12.4888	0.000436	0.0908	0.0241996952
ss86288046		1	62.745876	0.6468	11.0810	0.000918	0.0641	0.0292662369
ss61513391	*rs41612071*	1	62.903762	0.3782	1.9478	0.163269	0.0270	0.0126976881
ss61467252	*rs41566183*	1	62.988708	0.5609	5.5783	0.018459	0.0456	0.0224428172
ss61533745	*rs41629108*	1	63.057542	0.1884	0.0213	0.884056	0.0034	0.0010468151
ss65155713	*rs43242089*	1	63.103376	0.5351	0.6182	0.431995	0.0149	0.0074272491
ss65155784	*rs43242760*	1	63.134249	0.3288	3.3305	0.068436	0.0367	0.0161889455
ss86293031		1	63.165653	0.3001	0.0719	0.788638	0.0056	0.0023472569

ss86332925		1	63.201432	0.4950	0.1440	0.704412	0.0071	0.0035677098
ss86336760		1	63.260592	0.2292	0.0130	0.909135	0.0026	0.0009032627
ss86338540		1	63.290921	0.7163	1.0443	0.307171	0.0216	0.0087863110
ss86331246		1	63.317735	0.3725	7.3932	0.006710	0.0525	0.0245634715
ss64683294	*rs42779775*	1	63.353671	0.8152	5.5527	0.018728	0.0566	0.0170446593
ss86306415		1	63.390653	0.4484	9.5835	0.002042	0.0568	0.0280847671
ss64412128	*rs42624841*	1	63.419414	0.2672	9.5146	0.002119	0.0659	0.0258251683
ss86313070		1	63.451323	0.1268	5.7212	0.017025	0.0660	0.0146195339
ss63968169	*rs42409620*	1	63.491319	0.4656	0.8708	0.351058	0.0179	0.0088986163
ss86338870		1	63.528235	0.3288	1.1259	0.289029	0.0208	0.0091694935
ss63968561	*rs42411615*	1	63.570305	0.5007	5.0238	0.025315	0.0407	0.0203546956
	rs29016002	1	63.611306	0.4499	8.7064	0.003278	0.0564	0.0279376258
ss61466486	*rs41570239*	1	63.668302	0.6103	0.0009	0.976104	0.0006	0.0002712231
ss86325441		1	63.7461	0.4842	0.2463	0.619841	0.0091	0.0045553847
ss61467253	*rs41566184*	1	63.770113	0.7142	7.9619	0.004913	0.0584	0.0238398680
ss38324116	*rs29009624*	1	63.793608	0.3245	2.3864	0.122848	0.0309	0.0135548939
ss65156028	*rs43243803*	1	63.816913	0.1540	0.5675	0.451496	0.0191	0.0049781898
ss61498232	*rs43709934*	1	63.872789	0.2980	12.3430	0.000471	0.0711	0.0297386115
ss61472519	*rs41571544*	1	63.920663	0.9499	0.0252	0.873799	0.0066	0.0006259585
ss61512958	*rs41609561*	1	63.984669	0.1340	2.5733	0.109133	0.0429	0.0099591562
ss86338054		1	64.054879	0.8646	2.1654	0.141601	0.0390	0.0091394809
ss61524032	*rs41624979*	1	64.091422	0.7865	0.5626	0.453451	0.0166	0.0055576749
ss86279693		1	64.147284	0.6956	0.5074	0.476512	0.0142	0.0059944395
ss61514367	*rs41611740*	1	64.173388	0.1390	0.0402	0.841106	0.0053	0.0012667284
ss86329122		1	64.297826	0.5272	0.9684	0.325433	0.0179	0.0089352046
ss86305570		1	64.378197	0.4284	0.8396	0.359821	0.0176	0.0086308140
ss62256254	*rs41806014*	1	64.424192	0.2400	0.0138	0.906484	0.0025	0.0009265550
ss62255753	*rs41805548*	1	64.493166	0.6870	0.7371	0.390889	0.0170	0.0072915781
ss117966299		1	64.554511	0.7400	12.5085	0.000432	0.0736	0.0283100958
ss64705675	*rs42803894*	1	64.647196	0.3532	1.7734	0.183393	0.0256	0.0116780602
ss62255582	*rs41804694*	1	64.670141	0.5408	1.5300	0.216537	0.0229	0.0113923635
ss61566268	*rs41662310*	1	64.787542	0.4599	0.1204	0.728740	0.0065	0.0032446084
ss86307028		1	64.82509	0.6268	2.2762	0.131829	0.0293	0.0137098568
ss117963560		1	64.972045	0.4549	8.7673	0.003171	0.0562	0.0278552182
ss65574238	*rs43653256*	1	65.008552	0.4570	6.4938	0.011039	0.0483	0.0239745726
ss86301890		1	65.146606	0.3532	6.4446	0.011345	0.0488	0.0223038490
ss65157336	*rs43242686*	1	65.167828	0.3503	1.8549	0.173658	0.0264	0.0120376729
ss65157413	*rs43243263*	1	65.244082	0.5057	0.7033	0.401974	0.0156	0.0077906360
ss86320188		1	65.287593	0.1160	0.9932	0.319303	0.0287	0.0058857650
ss86339635		1	65.331988	0.1970	6.6948	0.009871	0.0635	0.0200842104
ss65151094	*rs43233287*	1	65.36674	0.3438	1.4062	0.236086	0.0230	0.0103720374
ss117966308		1	65.401554	0.1755	1.3315	0.248934	0.0277	0.0080251811
ss86297322		1	65.515987	0.6633	16.5174	0.000054	0.0808	0.0360972012
ss65151304	*rs43235097*	1	65.537491	0.3138	11.0261	0.000945	0.0668	0.0287546843
ss65151313	*rs43235106*	1	65.560286	0.6182	0.7000	0.403072	0.0160	0.0075638375
ss117966079		1	65.60654	0.3016	2.5741	0.109079	0.0320	0.0134989677
ss61524616	*rs41622147*	1	65.638343	0.8639	0.0433	0.835132	0.0057	0.0013294298

ss86341192		1	65.661818	0.2930	0.5463	0.460087	0.0152	0.0062778100
ss86274339		1	65.687831	0.1311	0.0158	0.900045	0.0034	0.0007796611
ss65151406	*rs43235999*	1	65.712985	0.7221	0.0913	0.762625	0.0062	0.0024996003
ss65151458	*rs43236849*	1	65.774963	0.3338	2.5950	0.107654	0.0316	0.0140627333
ss86284784		1	65.846476	0.8324	5.5822	0.018418	0.0580	0.0161720083
ss65151555	*rs43237846*	1	65.869153	0.3231	3.4527	0.063569	0.0366	0.0159913131
ss65151589	*rs43237880*	1	65.901953	0.1562	0.1717	0.678705	0.0107	0.0028132799
ss65151662	*rs43238752*	1	65.930952	0.2958	13.8413	0.000215	0.0738	0.0307522895
ss61524618	*rs41575644*	1	65.95739	0.7056	13.6928	0.000232	0.0734	0.0304894986
ss117966330		1	66.014815	0.8245	10.0506	0.001590	0.0753	0.0217971466
	rs29014781	1	66.058911	0.8195	9.2649	0.002424	0.0750	0.0221847673
ss61475572	*rs41575645*	1	66.098175	0.8238	8.7498	0.003201	0.0732	0.0212514414
ss86313851		1	66.33048	0.9162	0.1263	0.722444	0.0123	0.0018825845
ss65151840	*rs43239719*	1	66.385196	0.5193	2.8333	0.092775	0.0316	0.0157591419
ss65083578	*rs43168866*	1	66.410103	0.6067	0.8606	0.353894	0.0177	0.0084454499
ss86291952		1	66.448326	0.9040	1.3300	0.249196	0.0361	0.0062711051
ss86287891		1	66.574543	0.1913	17.4172	0.000034	0.0988	0.0305601548
ss86337234		1	66.596156	0.5881	9.2424	0.002453	0.0570	0.0276323248
ss86320655		1	66.642631	0.5451	0.4289	0.512742	0.0125	0.0062187675
ss61475575	*rs41575648*	1	66.684196	0.5380	0.2021	0.653208	0.0087	0.0043429790
ss105246055		1	66.706737	0.0509	0.8312	0.362229	0.0385	0.0037182791
ss105246057		1	66.7972	0.8274	0.0801	0.777256	0.0070	0.0019988462
ss65151895	*rs43240574*	1	66.82196	0.5666	0.7509	0.386504	0.0165	0.0080952661
ss86340622		1	66.851553	0.4670	1.1306	0.288009	0.0196	0.0097801039
ss86292706		1	66.903183	0.4656	1.1063	0.293252	0.0194	0.0096777560
ss61534220	*rs41633073*	1	66.936405	0.2930	0.2878	0.591777	0.0110	0.0045455391
ss61534219	*rs41633072*	1	66.971785	0.2901	0.0001	0.991923	0.0002	0.0000855374
ss86313185		1	67.055261	0.2901	0.0001	0.991923	0.0002	0.0000855374
ss86297710		1	67.081775	0.7099	0.0001	0.991923	0.0002	0.0000855374
ss61534214	*rs43741403*	1	67.119589	0.3059	0.1566	0.692397	0.0080	0.0034107114
ss86339764		1	67.147488	0.5745	3.0577	0.080795	0.0335	0.0163641387
ss86325330		1	67.173005	0.1032	4.5429	0.033404	0.0642	0.0118809362
ss46526276	*rs41255415*	1	67.210808	0.3238	8.6818	0.003322	0.0599	0.0262476412
ss86322599		1	67.249054	0.2529	5.1383	0.023709	0.0489	0.0184937301
ss65156920	*rs43244070*	1	67.551498	0.7314	0.2983	0.585133	0.0112	0.0043827573
ss65156894	*rs43244044*	1	67.583534	0.9441	4.4838	0.034571	0.0847	0.0089390468
ss65157432	*rs43243282*	1	67.627872	0.8804	10.0810	0.001564	0.0901	0.0189768399
ss86302008		1	67.680631	0.8926	6.7462	0.009593	0.0769	0.0147588934
ss86306999		1	67.755813	0.4427	0.2125	0.644945	0.0086	0.0042206200
ss65150353	*rs43235351*	1	67.779829	0.3352	8.5486	0.003570	0.0579	0.0258271970
ss65150367	*rs43235365*	1	67.801353	0.4799	35.3887	0.000000	0.1080	0.0539216330
ss61533230	*rs41630793*	1	67.885083	0.0838	0.0270	0.869605	0.0055	0.0008444829
ss38327238	*rs29019349*	1	67.947813	0.1662	5.7223	0.017015	0.0594	0.0164486054
ss65152958	*rs43242536*	1	67.97689	0.0852	0.0137	0.906688	0.0039	0.0006086596
ss117963537		1	68.188517	0.4986	21.7535	0.000004	0.0832	0.0416057737
ss65156451	*rs43241623*	1	68.220457	0.3739	0.9617	0.327110	0.0184	0.0086092793
ss65156446	*rs43241618*	1	68.2504	0.1884	2.3030	0.129581	0.0362	0.0110617645

ss65156409	*rs43241581*	1	68.27948	0.4771	3.9252	0.047961	0.0359	0.0178929336
ss65156399	*rs43241571*	1	68.300741	0.7858	0.0745	0.785005	0.0061	0.0020680738
	rs29018937	1	68.371872	0.7307	1.9206	0.166230	0.0291	0.0114634554
ss61508180	*rs41602465*	1	68.396136	0.7830	0.0013	0.971424	0.0008	0.0002751258
ss86340463		1	68.456231	0.8166	0.3895	0.532769	0.0149	0.0044564015
ss86341364		1	68.486532	0.0845	9.6674	0.001952	0.1020	0.0157911255
ss61533238	*rs41630801*	1	68.51027	0.4986	0.5007	0.479429	0.0134	0.0067138324
ss86335565		1	68.553122	0.6060	2.0463	0.153029	0.0271	0.0129233041
ss86336133		1	68.62327	0.1017	7.0191	0.008248	0.0792	0.0144790691
ss64753772	*rs42845689*	1	68.644961	0.1913	0.9154	0.339010	0.0235	0.0072546028
	rs29024892	1	68.666223	0.1927	1.1491	0.284106	0.0262	0.0081640169
ss61534762	*rs41628909*	1	68.709077	0.7629	1.1701	0.279761	0.0239	0.0086613888
ss86318041		1	68.762711	0.7221	1.0334	0.309713	0.0216	0.0086866334
ss86301034		1	68.801602	0.5688	9.8646	0.001756	0.0583	0.0286193703
ss86315883		1	68.85569	0.6046	0.5696	0.450667	0.0144	0.0068831219
ss105264089		1	68.876073	0.5774	1.0079	0.315752	0.0184	0.0089982442
ss61480548	*rs41579089*	1	68.905838	0.8066	9.4587	0.002184	0.0711	0.0221892106
ss86339073		1	68.959338	0.4785	4.3277	0.037862	0.0387	0.0193369601
ss86337347		1	69.009178	0.0981	0.1199	0.729209	0.0111	0.0019601003
ss61480546	*rs41579087*	1	69.034902	0.4964	3.7145	0.054348	0.0356	0.0178011472
ss86302873		1	69.081746	0.8309	3.3963	0.065768	0.0462	0.0129893285
ss61480542	*rs41579083*	1	69.106533	0.2299	18.5249	0.000019	0.0933	0.0330407353
ss86286166		1	69.380688	0.6784	3.3574	0.067332	0.0354	0.0154692282
ss86318032		1	69.443399	0.2787	3.0501	0.081174	0.0361	0.0145325812
	rs29025402	1	69.472454	0.6956	5.0326	0.025188	0.0446	0.0189019032
ss86316685		1	69.503256	0.8030	12.7653	0.000377	0.0837	0.0264835101
ss86302157		1	69.528622	0.7772	15.4965	0.000091	0.0866	0.0299816428
ss86331779		1	69.551738	0.1977	11.8305	0.000617	0.0803	0.0254635986
ss61507062	*rs41602364*	1	69.576517	0.1468	1.6972	0.193089	0.0337	0.0084496610
	rs29023069	1	69.643959	0.3782	22.6127	0.000002	0.0905	0.0425749876
ss86339134		1	69.707257	0.3854	14.3424	0.000166	0.0718	0.0340108866
ss86341485		1	69.789279	0.4871	1.2812	0.258075	0.0215	0.0107649611
ss86285524		1	69.815898	0.3166	7.8501	0.005223	0.0551	0.0238545954
ss86340613		1	69.848816	0.6827	7.8042	0.005356	0.0548	0.0237502466
ss86335828		1	69.963398	0.1426	11.3542	0.000794	0.0888	0.0217026063
ss86289762		1	70.012034	0.4377	1.8450	0.174802	0.0249	0.0122379786
ss61522942	*rs41622811*	1	70.05187	0.2794	1.6132	0.204464	0.0255	0.0102672855
ss86286670		1	70.095337	0.6948	4.4710	0.034830	0.0420	0.0177971097
ss86328166		1	70.168783	0.5609	0.0001	0.992937	0.0002	0.0000812423
ss61534196	*rs41633052*	1	70.196076	0.2049	0.2692	0.604007	0.0120	0.0039250261
ss86313603		1	70.234225	0.5931	9.3407	0.002327	0.0576	0.0278103408
	rs29019798	1	70.265609	0.5759	7.1731	0.007575	0.0499	0.0243951735
ss86293415		1	70.443282	0.5444	0.0635	0.801128	0.0047	0.0023069445
ss86339497		1	70.48104	0.4577	0.0063	0.937002	0.0015	0.0007234997
ss86334411		1	70.520163	0.1096	12.7183	0.000387	0.1060	0.0206861842
ss86288454		1	70.653973	0.9327	6.2307	0.012785	0.0935	0.0117449792
ss86341072		1	70.738581	0.2708	4.8099	0.028626	0.0456	0.0179908479

ss86336694		1	70.771471	0.5795	11.6343	0.000685		0.0656	0.0319939444
ss86332746		1	70.985722	0.6282	10.8803	0.001021		0.0618	0.0288652582
ss61533757	*rs41629120*	1	71.055014	0.4964	11.4087	0.000772		0.0628	0.0314095092
ss61533753	*rs41629116*	1	71.100927	0.9312	0.8777	0.349148		0.0345	0.0044171989
ss61522937	*rs41622806*	1	71.127524	0.6848	2.5599	0.110062		0.0316	0.0136332492
ss61508107	*rs41601494*	1	71.169378	0.9319	1.1469	0.284566		0.0396	0.0050204740
ss65156044	*rs43243818*	1	71.234296	0.3202	2.6727	0.102534		0.0318	0.0138648889
ss61476013	*rs41579376*	1	71.357246	0.2185	3.7735	0.052473		0.0437	0.0149087978
ss46526952	*rs41257398*	1	71.432874	0.2178	3.6049	0.058022		0.0427	0.0145421098
ss61562666	*rs41663962*	1	71.497171	0.0738	1.9712	0.160771		0.0497	0.0067954162
ss61534764	*rs41628911*	1	71.563555	0.6454	13.0441	0.000326		0.0711	0.0325600884
ss86310197		1	71.585336	0.1182	2.5685	0.109468		0.0466	0.0097044270
ss86339086		1	71.660197	0.5516	8.5802	0.003510		0.0539	0.0266390847
ss86283282		1	71.699084	0.5530	8.9792	0.002828		0.0554	0.0273984800
ss86325676		1	71.77815	0.1576	1.1866	0.276389		0.0282	0.0074845787
ss86313057		1	71.812294	0.7013	0.0000	0.999402		0.0000	0.0000066060
ss105263683		1	71.898007	0.4255	0.0478	0.826961		0.0040	0.0019719784
	rs29015962	1	71.919665	0.2278	1.3093	0.252910		0.0244	0.0085697586
ss86329302		1	71.979458	0.0523	0.1395	0.708886		0.0156	0.0015482327
ss61480552	*rs41579093*	1	72.076618	0.7729	0.1959	0.658193		0.0095	0.0033240506
ss86279905		1	72.115872	0.2593	1.3519	0.245347		0.0241	0.0092722079
ss61480553	*rs41579094*	1	72.224768	0.4993	0.6603	0.416724		0.0146	0.0072964307
ss86286948		1	72.354343	0.5415	0.0399	0.841790		0.0037	0.0018579874
ss86317117		1	72.377125	0.8689	1.4091	0.235614		0.0321	0.0073171678
ss61534777	*rs41628924*	1	72.412086	0.5344	0.4176	0.518344		0.0123	0.0060976304
ss61534770	*rs41628917*	1	72.472613	0.5795	8.3165	0.004050		0.0536	0.0261391732
ss61508233	*rs41602517*	1	72.502801	0.2443	2.9899	0.084229		0.0377	0.0139290729
	rs29025422	1	72.536977	0.1991	8.4433	0.003780		0.0673	0.0214788508
ss86335918		1	72.565212	0.2500	1.4353	0.231304		0.0261	0.0097745841
ss86279358		1	72.587546	0.0602	0.4184	0.517975		0.0245	0.0027665545
ss86321025		1	72.673466	0.9219	0.1699	0.680359		0.0145	0.0020836697
ss61565032	*rs41657696*	1	72.776086	0.0781	0.1699	0.680359		0.0145	0.0020836697
ss86341426		1	72.956334	0.5903	0.1466	0.701968		0.0073	0.0035290465
ss86281640		1	73.087573	0.1440	4.7667	0.029349		0.0582	0.0143449017
ss86308247		1	73.119196	0.5595	0.0036	0.952130		0.0011	0.0005355421
ss86276175		1	73.154505	0.4670	0.5963	0.440259		0.0143	0.0070940678
ss86340583		1	73.20315	0.7264	14.0381	0.000194		0.0799	0.0317421094
ss61535325	*rs41633660*	1	73.249867	0.1791	16.7432	0.000048		0.0961	0.0282492602
ss61535323	*rs41633658*	1	73.314637	0.7049	8.5571	0.003554		0.0609	0.0253497425
ss86302886		1	73.357205	0.7228	3.8757	0.049385		0.0411	0.0164867940
ss86334054		1	73.451903	0.4155	0.1598	0.689495		0.0075	0.0036573232
ss86341435		1	73.476448	0.4097	0.2668	0.605655		0.0098	0.0047270119
ss86324271		1	73.562297	0.4119	0.1647	0.684964		0.0076	0.0036973862
ss86340772		1	73.596493	0.5917	3.4437	0.063916		0.0346	0.0167224486
ss86330967		1	73.624954	0.3990	13.8961	0.000209		0.0724	0.0347082207
ss86290159		1	73.659512	0.4656	0.4361	0.509221		0.0121	0.0060453041
ss86319973		1	73.730135	0.2880	4.8520	0.027941		0.0442	0.0181323183

ss61534784	*rs41629731*	1	73.786758	0.4427	19.9334	0.000009	0.0805	0.0397350779
ss86326563		1	74.016192	0.2880	0.3594	0.549030	0.0124	0.0050725762
ss61534782	*rs41628929*	1	74.099263	0.4441	8.5661	0.003536	0.0542	0.0267753836
ss61534780	*rs41628927*	1	74.129178	0.8775	2.7924	0.095160	0.0480	0.0103257236
ss117966406		1	74.20885	0.2593	10.4176	0.001307	0.0684	0.0262798005
ss86337840		1	74.505016	0.7507	0.0265	0.870712	0.0034	0.0012709842
ss86284717		1	74.569739	0.3689	1.0159	0.313853	0.0188	0.0087310951
ss61535332	*rs41633667*	1	74.635915	0.3911	1.4970	0.221541	0.0225	0.0107340184
ss86336965		1	74.760161	0.1884	1.1960	0.274494	0.0258	0.0078997903
ss86284212		1	74.797121	0.1476	1.7825	0.182283	0.0346	0.0087147869
ss86323568		1	74.825741	0.1390	0.1408	0.707585	0.0104	0.0024906200
ss86306988		1	74.957978	0.3231	1.6603	0.197990	0.0249	0.0109078706
ss105265054		1	74.979241	0.9728	0.8113	0.368050	0.0513	0.0027173699
ss61480772	*rs41580508*	1	75.042636	0.6791	1.1223	0.289800	0.0209	0.0091024499
ss117963506		1	75.104988	0.3711	0.1628	0.686712	0.0077	0.0035748662
ss86339892		1	75.135242	0.4749	3.7723	0.052512	0.0355	0.0177192652
ss86294423		1	75.221451	0.1067	11.5035	0.000734	0.1018	0.0194032959
ss86319145		1	75.24629	0.1010	18.7894	0.000017	0.1318	0.0239306095
ss61535865	*rs41630294*	1	75.390599	0.1655	13.2280	0.000296	0.0917	0.0253263450
ss61535870	*rs41630299*	1	75.559938	0.7206	0.3114	0.577006	0.0117	0.0047079334
ss86310009		1	75.66813	0.5852	0.6137	0.433652	0.0148	0.0071631197
ss86321139		1	75.815672	0.2070	0.6538	0.419026	0.0187	0.0061463660
ss86311633		1	76.074458	0.3288	0.0022	0.962740	0.0009	0.0004057820
ss86312351		1	76.099438	0.2844	0.4489	0.503073	0.0135	0.0055124535
ss61519787	*rs41617926*	1	76.155226	0.8317	0.8871	0.346587	0.0228	0.0063900335
ss86329547		1	76.229188	0.7221	0.1522	0.696587	0.0079	0.0031796140
ss61525553	*rs41621765*	1	76.291521	0.6812	0.2570	0.612330	0.0102	0.0044375296
ss117966412		1	76.362569	0.9011	7.9611	0.004916	0.0886	0.0157921610
ss117966155		1	76.434293	0.8381	3.6212	0.057460	0.0459	0.0124488964
ss86338773		1	76.505505	0.6812	1.1062	0.293272	0.0206	0.0089683752
ss86308119		1	76.527161	0.3123	3.2669	0.071123	0.0358	0.0153590889
ss86315519		1	76.55654	0.6218	2.1894	0.139412	0.0279	0.0131377808
ss105259413		1	76.629388	0.3503	1.2143	0.270864	0.0216	0.0098312342
ss61505921	*rs41600452*	1	76.732789	0.0838	2.5966	0.107548	0.0533	0.0081912537
	rs29012834	1	76.817711	0.2887	0.0212	0.884218	0.0029	0.0012007632
ss86293972		1	77.18878	0.2908	0.3274	0.567350	0.0114	0.0047098899
ss61531718	*rs41629270*	1	77.235779	0.1884	12.0845	0.000540	0.0812	0.0248389103
ss61479024	*rs41579892*	1	77.26918	0.8446	0.2072	0.649106	0.0115	0.0030075464
ss86341784		1	77.317385	0.8610	3.0037	0.083517	0.0465	0.0111321650
ss86296452		1	77.415588	0.7321	12.8270	0.000365	0.0742	0.0291219831
ss105259423		1	77.469637	0.7163	17.5650	0.000031	0.0860	0.0349635207
ss61520450	*rs41614575*	1	77.503532	0.8847	10.4906	0.001257	0.0943	0.0192453189
ss86274911		1	77.643198	0.6755	1.8415	0.175219	0.0262	0.0114975333
ss61496363	*rs41598314*	1	77.706623	0.5394	0.1783	0.672967	0.0077	0.0038152232
ss61567714	*rs41664218*	1	77.735937	0.3890	0.5009	0.479349	0.0134	0.0063626964
ss105259418		1	77.789354	0.3653	2.0684	0.150833	0.0276	0.0127878527
ss61526353	*rs41623050*	1	77.874001	0.1726	12.3660	0.000466	0.0840	0.0239903084

ss61508326	*rs41603203*	1	77.938518	0.3295	5.2078	0.022787		0.0434	0.0191871799
ss86339066		1	77.994924	0.6361	0.1627	0.686796		0.0076	0.0035395607
ss61481014	*rs41582637*	1	78.205573	0.3274	1.8191	0.177861		0.0254	0.0111756241
ss61481015	*rs41582638*	1	78.245999	0.3274	0.2067	0.649499		0.0087	0.0038271518
ss117966384		1	78.280008	0.4850	0.5842	0.444932		0.0139	0.0069429666
ss86318501		1	78.32067	0.4183	0.3130	0.576004		0.0102	0.0049590583
ss86323031		1	78.441351	0.8159	2.4751	0.116113		0.0367	0.0110226798
ss61535878	*rs41630307*	1	78.482029	0.5509	5.8096	0.016197		0.0451	0.0223008206
ss105262943		1	78.556254	0.4398	41.8627	0.000000		0.1158	0.0570641936
ss86310983		1	78.60347	0.3059	11.0593	0.000929		0.0643	0.0273165107
ss86306740		1	78.66828	0.9219	4.2291	0.040109		0.0713	0.0102623198
ss61535882	*rs41630311*	1	78.966222	0.7880	12.7748	0.000376		0.0814	0.0271857548
	rs29009958	1	79.110246	0.1340	4.4862	0.034524		0.0576	0.0133687462
ss86286585		1	79.173536	0.2908	16.9886	0.000042		0.0821	0.0338505546
ss61518249	*rs41612214*	1	79.595901	0.5494	17.6350	0.000030		0.0756	0.0374313880
ss61518245	*rs41612210*	1	79.657036	0.3789	5.4378	0.019990		0.0442	0.0207907571
ss86290355		1	79.783568	0.8546	3.1952	0.074289		0.0468	0.0116292233
ss61501443	*rs41607668*	1	79.908851	0.8711	3.3331	0.068328		0.0506	0.0113552938
ss105259439		1	79.937591	0.6125	2.5188	0.112951		0.0305	0.0144777064
ss61536343	*rs41634467*	1	79.972528	0.1691	13.2784	0.000288		0.0888	0.0249454462
ss61536342	*rs41634466*	1	80.026845	0.1913	4.4838	0.034572		0.0483	0.0149553643
ss65159127	*rs43244750*	1	80.050487	0.2736	12.2049	0.000507		0.0714	0.0283996623
ss86276944		1	80.110091	0.2572	12.9834	0.000337		0.0744	0.0284434467
ss86338192		1	80.132372	0.6168	23.1206	0.000002		0.0885	0.0418427633
ss105261559		1	80.155227	0.3861	21.4523	0.000004		0.0851	0.0403498753
ss65158984	*rs43252102*	1	80.180424	0.2034	23.1620	0.000002		0.1042	0.0337699649
ss61481021	*rs41582644*	1	80.204226	0.8489	0.3581	0.549777		0.0153	0.0039245658
ss65158933	*rs43252051*	1	80.254378	0.8166	0.5548	0.456610		0.0175	0.0052541538
ss61508328	*rs41603205*	1	80.288508	0.0974	4.6798	0.030860		0.0679	0.0119427578
ss61518357	*rs41613221*	1	80.314815	0.4183	4.2018	0.040756		0.0379	0.0184354462
ss65158836	*rs43250959*	1	80.362916	0.3653	1.1787	0.278000		0.0208	0.0096331948
ss86338828		1	80.419712	0.4807	0.7903	0.374304		0.0163	0.0081302443
ss86329338		1	80.469987	0.4527	3.7663	0.052700		0.0355	0.0175681699
ss65158537	*rs43247266*	1	80.645629	0.4835	1.6469	0.199804		0.0246	0.0122884571
ss61535891	*rs41630320*	1	80.812353	0.7915	1.0711	0.301048		0.0235	0.0077623775
ss65158445	*rs43245775*	1	80.854329	0.4570	2.7901	0.095298		0.0315	0.0156370550
ss61481018	*rs41582641*	1	80.88389	0.4248	0.2075	0.648903		0.0085	0.0041479702
ss86286874		1	80.963024	0.4577	1.7968	0.180533		0.0253	0.0125814614
ss86298346		1	81.022762	0.6612	1.6681	0.196937		0.0258	0.0115390940
ss86336994		1	81.062375	0.3360	0.6233	0.430097		0.0154	0.0068648465
ss61570331	*rs41666794*	1	81.085496	0.5201	0.0281	0.866971		0.0031	0.0015573576
ss86339823		1	81.142207	0.1032	0.1916	0.661708		0.0130	0.0024021184
ss65160259	*rs43245574*	1	81.19906	0.8968	0.1916	0.661708		0.0130	0.0024021184
ss86330710		1	81.242738	0.4506	3.4275	0.064543		0.0345	0.0170765538
ss61536396	*rs41634520*	1	81.283582	0.4255	0.0023	0.961386		0.0009	0.0004528343
ss61536845	*rs41635167*	1	81.318446	0.0752	0.8824	0.347874		0.0331	0.0046037556
ss86280870		1	81.338913	0.8990	2.9102	0.088468		0.0516	0.0093655381

ss86307670		1	81.376956	0.5666	0.2626	0.608511		0.0098	0.0048229292
ss86284959		1	81.448073	0.2650	0.1755	0.675432		0.0087	0.0033933827
ss65160378	*rs43246393*	1	81.527609	0.1676	0.0169	0.896596		0.0031	0.0008671295
ss61562351	*rs41660452*	1	81.639876	0.3374	1.4086	0.235701		0.0227	0.0101649929
ss86316456		1	81.761527	0.7436	0.8060	0.369625		0.0192	0.0073228194
ss86274934		1	81.829875	0.5344	3.5578	0.059682		0.0345	0.0171863677
ss86314628		1	81.856895	0.0874	5.4500	0.019852		0.0740	0.0117981124
ss86310174		1	81.879312	0.3918	0.1229	0.726058		0.0067	0.0032102940
ss61536855	*rs41635177*	1	82.099085	0.5107	3.2311	0.072685		0.0336	0.0167857985
ss86281729		1	82.120062	0.2765	5.4891	0.019416		0.0501	0.0200409500
ss86312025		1	82.201456	0.6547	3.3951	0.065818		0.0358	0.0162023366
ss61536369	*rs41634493*	1	82.298892	0.6046	12.6398	0.000403		0.0671	0.0320720711
ss61536365	*rs41634489*	1	82.377934	0.2493	1.2533	0.263309		0.0239	0.0089611216
ss117963531		1	82.400005	0.3689	4.0363	0.044918		0.0397	0.0184877069
ss61536847	*rs41635169*	1	82.442199	0.4785	3.1851	0.074747		0.0333	0.0165993616
ss86326397		1	82.490786	0.7400	0.0978	0.754524		0.0066	0.0025478840
ss61537448	*rs41640545*	1	82.521418	0.2271	0.5106	0.475108		0.0165	0.0057811560
ss61537445	*rs41640542*	1	82.553338	0.6648	6.1775	0.013172		0.0508	0.0226573753
	rs29012500	1	82.627068	0.9198	5.1731	0.023242		0.0772	0.0113893392
ss86309089		1	82.655036	0.4391	0.0005	0.982715		0.0004	0.0002071684
ss86339204		1	82.740462	0.2801	3.5288	0.060728		0.0402	0.0162300168
ss61537427	*rs41639724*	1	82.768682	0.2794	3.3712	0.066771		0.0393	0.0158426612
	rs29021199	1	82.797926	0.2794	3.3712	0.066771		0.0393	0.0158426612
ss61537428	*rs41639725*	1	82.822088	0.0723	3.2380	0.072382		0.0635	0.0085228607
ss86307360		1	82.843582	0.7213	3.4875	0.062253		0.0402	0.0161438006
ss61501595	*rs41608610*	1	82.871326	0.7199	3.4910	0.062122		0.0400	0.0161467776
ss86334755		1	82.904951	0.1970	1.3247	0.250153		0.0268	0.0084793859
ss86337625		1	82.938827	0.7271	2.2623	0.133011		0.0327	0.0129876244
ss86301461		1	82.984086	0.4642	9.9538	0.001674		0.0600	0.0298676216
ss61537441	*rs41640538*	1	83.072761	0.8481	0.0495	0.823957		0.0058	0.0014862470
ss86287067		1	83.14165	0.8539	0.5580	0.455309		0.0193	0.0048046144
ss61507058	*rs41602360*	1	83.367805	0.4936	5.5209	0.019069		0.0434	0.0217070750
ss46526210	*rs41255350*	1	83.459606	0.3331	0.3955	0.529627		0.0126	0.0055830498
ss86292310		1	83.499181	0.1447	3.0184	0.082765		0.0465	0.0114999058
ss86280665		1	83.575092	0.4269	1.9351	0.164651		0.0270	0.0132012306
ss63772975	*rs42344261*	1	83.718412	0.4169	2.6598	0.103363		0.0326	0.0158424219
ss61570060	*rs41672105*	1	83.751111	0.4026	1.5378	0.215357		0.0230	0.0110417434
ss65160007	*rs43250826*	1	83.793212	0.3238	4.8906	0.027327		0.0444	0.0194217944
ss61484424	*rs41579176*	1	83.842132	0.3259	6.9197	0.008714		0.0519	0.0227955230
ss86336758		1	83.915932	0.2479	5.3667	0.020814		0.0504	0.0188060937
ss86327992		1	83.964513	0.6762	3.7816	0.052222		0.0380	0.0166540775
ss61505297	*rs41604955*	1	83.986617	0.4986	0.0970	0.755517		0.0059	0.0029345626
ss86340333		1	84.010214	0.6891	1.8311	0.176434		0.0281	0.0120356230
ss63394027	*rs42215216*	1	84.071804	0.2385	3.1295	0.077326		0.0385	0.0139784472
ss61536879	*rs41635201*	1	84.119387	0.0974	2.4541	0.117676		0.0492	0.0086609024
ss61536886	*rs41635208*	1	84.213943	0.9248	0.3190	0.572401		0.0197	0.0027364738
ss86338294		1	84.234969	0.9241	0.2903	0.590230		0.0187	0.0026237951

ss86298985		1	84.289929	0.3990	0.0131	0.909054	0.0022	0.0010569231
ss86329397		1	84.314502	0.4713	0.1679	0.682084	0.0076	0.0037999291
ss86282770		1	84.339045	0.2077	0.5873	0.443743	0.0174	0.0057136829
ss86317649		1	84.36543	0.5630	0.0353	0.850936	0.0035	0.0017403730
ss61537408	*rs41639705*	1	84.394164	0.4298	0.0000	0.996001	0.0001	0.0000463990
ss86299947		1	84.415672	0.5688	0.0150	0.902565	0.0023	0.0011330474
ss65161449	*rs43248063*	1	84.438165	0.9183	7.1913	0.007500	0.0911	0.0136658543
ss61537418	*rs41639715*	1	84.461726	0.2951	3.0922	0.079108	0.0356	0.0148001108
ss86308120		1	84.528382	0.7285	2.7116	0.100073	0.0343	0.0135659295
ss65160589	*rs43248004*	1	84.578824	0.2808	3.2429	0.072165	0.0372	0.0150143234
ss86328397		1	84.630641	0.3732	2.8843	0.089895	0.0328	0.0153686831
ss86306123		1	84.664648	0.4377	1.8862	0.170078	0.0257	0.0126708113
ss86295432		1	84.722208	0.0960	2.2794	0.131560	0.0465	0.0080679710
ss117966112		1	84.809474	0.0802	7.9410	0.004970	0.0973	0.0143620999
ss86339392		1	85.16143	0.8016	1.2323	0.267341	0.0253	0.0080586747
ss61481490	*rs41578805*	1	85.182073	0.7973	1.1793	0.277881	0.0246	0.0079541464
ss86283985		1	85.244394	0.1748	0.3155	0.574481	0.0136	0.0039172477
ss61508407	*rs41603780*	1	85.264719	0.7973	1.1793	0.277881	0.0246	0.0079541464
ss61536859	*rs41635181*	1	85.439534	0.7414	11.9602	0.000577	0.0698	0.0267768438
	rs29016842	1	85.503594	0.4040	5.6428	0.017797	0.0437	0.0210313113
	rs29026905	1	85.6426	0.8517	0.0094	0.922980	0.0025	0.0006266818
ss86335367		1	85.694937	0.9033	0.0775	0.780856	0.0084	0.0014726291
ss64719895	*rs42820814*	1	85.798175	0.1511	2.9962	0.083903	0.0437	0.0112022428
ss64719871	*rs42820790*	1	85.819756	0.8625	4.2561	0.039480	0.0537	0.0127483892
ss64260054	*rs42548511*	1	85.95453	0.1683	10.7488	0.001095	0.0796	0.0222907857
ss86289851		1	85.988055	0.7708	10.6301	0.001167	0.0711	0.0251288029
ss64260184	*rs42549376*	1	86.052864	0.9463	4.0688	0.044066	0.0820	0.0083325002
ss61566209	*rs41662251*	1	86.078937	0.1683	1.7945	0.180818	0.0327	0.0091579143
ss117966446		1	86.222951	0.2299	0.0917	0.762058	0.0064	0.0022824476
ss61481734	*rs41581439*	1	86.277311	0.1261	1.0922	0.296341	0.0283	0.0062275687
ss65162397	*rs43246311*	1	86.371827	0.1748	0.3910	0.531998	0.0150	0.0043396674
	rs29024815	1	86.748746	0.3961	4.8046	0.028715	0.0402	0.0192497835
ss117966358		1	86.979061	0.4362	3.2180	0.073268	0.0316	0.0155512473
ss86339905		1	87.122551	0.1103	1.0454	0.306921	0.0300	0.0058807721
ss61508527	*rs41604794*	1	87.181372	0.3897	1.8218	0.177542	0.0256	0.0121534726
ss61482029	*rs41583530*	1	87.235428	0.5860	3.5200	0.061052	0.0351	0.0170442098
ss86284720		1	87.338372	0.8231	2.7782	0.096003	0.0403	0.0117483888
ss65162670	*rs43248683*	1	87.386242	0.7658	0.3145	0.575095	0.0123	0.0044185825
ss65161324	*rs43247039*	1	87.457262	0.5372	1.5347	0.215826	0.0235	0.0116750995
ss86288932		1	87.485098	0.2350	0.3317	0.564847	0.0126	0.0045464156
ss65161248	*rs43245663*	1	87.563646	0.6461	1.9755	0.160313	0.0273	0.0124820473
ss105262438		1	87.774882	0.2163	1.1738	0.278998	0.0248	0.0083980872
ss61481502	*rs41578817*	1	87.845375	0.3109	3.7567	0.053000	0.0387	0.0165868051
ss61481500	*rs41578815*	1	87.872449	0.5308	0.8045	0.370048	0.0170	0.0084848793
ss65161225	*rs43245640*	1	87.904074	0.2350	5.9824	0.014696	0.0526	0.0189234986
ss65161205	*rs43244720*	1	87.970511	0.5193	5.3563	0.020938	0.0427	0.0212990032
ss86341156		1	88.023753	0.4828	3.7712	0.052545	0.0359	0.0179168761

ss86301450		1	88.181051	0.3417	3.2450	0.072074		0.0345	0.0155167705
ss86313031		1	88.204957	0.3417	3.2450	0.072074		0.0345	0.0155167705
ss65160923	*rs43251938*	1	88.245891	0.4520	2.7342	0.098670		0.0302	0.0149805658
ss86341483		1	88.292011	0.4477	0.0823	0.774276		0.0052	0.0025883426
ss65161024	*rs43252439*	1	88.314986	0.4857	2.2251	0.136237		0.0276	0.0137895738
ss61536865	*rs41635187*	1	88.336774	0.4133	0.1874	0.665214		0.0080	0.0038895266
ss61536864	*rs41635186*	1	88.357867	0.6347	8.0162	0.004770		0.0527	0.0244559637
ss61508408	*rs41603781*	1	88.395149	0.8503	10.0484	0.001592		0.0825	0.0210054451
ss105295110		1	88.52676	0.1805	2.2686	0.132477		0.0353	0.0104480379
	rs29027507	1	88.603117	0.0688	7.4101	0.006648		0.0998	0.0127873113
ss61537886	*rs41635676*	1	88.650927	0.7550	0.4401	0.507275		0.0143	0.0052808875
ss86307016		1	88.871763	0.1304	0.8901	0.345790		0.0253	0.0057388466
ss105261235		1	88.892609	0.5036	6.8142	0.009238		0.0490	0.0245034876
	rs29021588	1	89.041799	0.5358	11.8923	0.000598		0.0639	0.0317974157
ss65162812	*rs43249325*	1	89.082974	0.4126	2.4026	0.121587		0.0283	0.0137110621
ss61478225	*rs41575337*	1	89.161164	0.1769	0.0481	0.826475		0.0053	0.0015425593
ss65162810	*rs43249323*	1	89.22164	0.4993	0.1105	0.739661		0.0060	0.0029795485
ss63985769	*rs42420812*	1	89.320365	0.9341	1.7055	0.192007		0.0489	0.0060184060
ss64874675	*rs42967263*	1	89.347107	0.1762	3.1950	0.074297		0.0431	0.0125207567
ss61529968	*rs41630469*	1	89.430809	0.9448	0.1709	0.679416		0.0171	0.0017854653
ss61524439	*rs41620681*	1	89.458551	0.8152	4.9110	0.027008		0.0550	0.0165589050
ss86286921		1	89.509672	0.4885	8.1717	0.004382		0.0531	0.0265249056
ss61513988	*rs41609768*	1	89.611099	0.1060	0.0756	0.783442		0.0081	0.0015367131
ss61471173	*rs41568144*	1	89.646907	0.1340	5.0743	0.024594		0.0602	0.0139690090
	rs29016036	1	89.677286	0.5150	0.7900	0.374410		0.0169	0.0084444235
ss86338266		1	89.710443	0.4133	1.4550	0.228130		0.0236	0.0114216209
	rs29009859	1	89.733516	0.1168	4.0183	0.045396		0.0568	0.0117149855
ss61514272	*rs41610645*	1	89.771787	0.1791	1.7640	0.184557		0.0326	0.0095817183
ss65163538	*rs43248805*	1	90.015794	0.4291	0.0401	0.841270		0.0039	0.0018902874
ss64854528	*rs42946198*	1	90.093495	0.5100	0.9569	0.328299		0.0186	0.0093119288
ss65163477	*rs43248747*	1	90.156639	0.7106	0.1422	0.706210		0.0080	0.0032966896
ss86338956		1	90.183076	0.2564	0.4534	0.500950		0.0147	0.0056151623
	rs29010500	1	90.233259	0.6411	0.0028	0.957790		0.0010	0.0004734836
ss61537912	*rs41635702*	1	90.33302	0.3388	0.3086	0.578707		0.0111	0.0049583805
ss61537897	*rs41635687*	1	90.411585	0.4670	0.8303	0.362489		0.0177	0.0087950269
	rs29026448	1	90.514214	0.5752	0.2964	0.586351		0.0104	0.0050907124
ss63790608	*rs42353559*	1	90.66347	0.2636	0.0311	0.859990		0.0038	0.0014842877
ss61472468	*rs41570902*	1	90.760335	0.5960	0.3948	0.529988		0.0122	0.0058628857
ss63790522	*rs42352716*	1	90.792114	0.3868	0.7502	0.386704		0.0169	0.0080378295
ss61524127	*rs41625673*	1	90.839077	0.9169	1.3948	0.237999		0.0396	0.0060385978
ss63791402	*rs42357756*	1	90.880213	0.7185	2.7945	0.095038		0.0343	0.0138867054
ss86324322		1	90.9212	0.6519	3.4035	0.065483		0.0364	0.0165275431
ss63791740	*rs42359025*	1	90.954551	0.9083	1.9045	0.168014		0.0439	0.0073177053
ss117966455		1	90.985132	0.9434	4.1264	0.042598		0.0788	0.0084162231
ss61498216	*rs43708918*	1	91.027727	0.7701	0.0544	0.815708		0.0052	0.0018378277
ss63792262	*rs42351089*	1	91.07191	0.0552	3.2733	0.070848		0.0709	0.0073946018
ss63792436	*rs42351876*	1	91.104639	0.9019	0.0026	0.959096		0.0016	0.0002829540

ss64200274	*rs42525155*	1	91.406645	0.1483	0.0833	0.772997	0.0076	0.0019296699
ss86293068		1	91.440773	0.0623	0.3462	0.556478	0.0228	0.0026699987
ss61538420	*rs41641200*	1	91.478127	0.1347	6.0008	0.014545	0.0653	0.0152158909
ss86294272		1	91.501098	0.0852	16.6621	0.000050	0.1342	0.0209266414
ss86319735		1	91.625391	0.7844	10.8724	0.001026	0.0735	0.0248598599
ss86337783		1	91.721439	0.4198	6.4052	0.011598	0.0478	0.0233082162
ss65165257	*rs43246622*	1	91.761868	0.8818	9.4219	0.002227	0.0869	0.0181239932
ss61538418	*rs41641198*	1	91.802143	0.1741	1.0886	0.297146	0.0251	0.0072297264
ss61482318	*rs41576315*	1	91.848224	0.3489	0.6580	0.417536	0.0162	0.0073444187
ss86315157		1	91.916661	0.3782	0.3860	0.534625	0.0122	0.0057389998
ss65165029	*rs43250493*	1	91.952264	0.4756	2.0012	0.157623	0.0269	0.0134088199
ss61538410	*rs41641190*	1	91.985875	0.0716	0.0347	0.852253	0.0068	0.0009087738
ss86308858		1	92.039958	0.0573	3.2815	0.070494	0.0709	0.0076562751
ss86303793		1	92.060017	0.8195	0.2933	0.588277	0.0129	0.0038186653
ss61538409	*rs41641189*	1	92.111007	0.7092	0.9111	0.340162	0.0199	0.0082081824
ss61482312	*rs41576309*	1	92.19814	0.2550	1.1557	0.282739	0.0233	0.0088656371
ss86278790		1	92.272317	0.6798	3.7559	0.053025	0.0381	0.0165974370
ss86286316		1	92.297145	0.3725	1.8806	0.170712	0.0264	0.0123629183
ss105264530		1	92.325176	0.7987	1.3757	0.241244	0.0271	0.0087213687
ss61525978	*rs41623976*	1	92.348088	0.1454	0.9296	0.335295	0.0258	0.0064054872
	rs29019866	1	92.433977	0.2564	3.1111	0.078197	0.0387	0.0147519958
ss61508606	*rs41605771*	1	92.476463	0.6712	7.1678	0.007597	0.0531	0.0234266740
ss117966281		1	92.568842	0.1605	0.0149	0.903040	0.0031	0.0008284894
ss61567173	*rs41660693*	1	92.589739	0.6712	1.7957	0.180671	0.0273	0.0120652116
ss61511724	*rs41615034*	1	92.627088	0.8653	1.4858	0.223289	0.0330	0.0076893305
	rs29010311	1	92.669976	0.2751	0.7654	0.381933	0.0180	0.0071673420
ss61496105	*rs41596559*	1	92.700621	0.3252	1.3845	0.239741	0.0242	0.0106038559
ss61500354	*rs41598608*	1	92.735975	0.2572	3.8230	0.050953	0.0417	0.0159150015
ss61529264	*rs41633772*	1	92.762692	0.6461	2.3072	0.129229	0.0309	0.0141130646
ss61529267	*rs41633775*	1	92.786888	0.6583	1.5700	0.210634	0.0254	0.0114470490
	rs29010538	1	92.823719	0.5580	0.0180	0.893202	0.0025	0.0012519944
ss64061243	*rs42460562*	1	92.965211	0.8904	0.1356	0.712779	0.0110	0.0021397365
ss86290885		1	93.00775	0.7858	1.7279	0.189114	0.0289	0.0097397794
ss64862775	*rs42957786*	1	93.154369	0.7292	0.3313	0.565073	0.0117	0.0046049423
ss61477867	*rs41580687*	1	93.263037	0.7471	0.6145	0.433367	0.0162	0.0061386108
ss61477865	*rs41580685*	1	93.283601	0.3102	2.9744	0.085034	0.0342	0.0146323470
ss86297976		1	93.311608	0.5401	0.1776	0.673549	0.0079	0.0039481823
ss64082495	*rs42465953*	1	93.418567	0.3725	0.0008	0.977162	0.0006	0.0002584898
ss105265431		1	93.450163	0.3732	4.4831	0.034585	0.0399	0.0186732916
ss105265430		1	93.479597	0.3617	5.0177	0.025405	0.0424	0.0195646370
ss64082253	*rs42465232*	1	93.524609	0.5079	0.1913	0.661961	0.0082	0.0041066212
ss86323079		1	93.572606	0.6862	5.3930	0.020505	0.0464	0.0199975591
ss64780263	*rs42871690*	1	93.599282	0.2292	4.5018	0.034212	0.0468	0.0165336366
ss86285031		1	93.721559	0.1683	1.5838	0.208634	0.0314	0.0087995064
ss86337313		1	93.782655	0.8317	1.5838	0.208634	0.0314	0.0087995064
ss117966377		1	93.805922	0.8660	5.4014	0.020407	0.0648	0.0150437232
ss64060375	*rs42455329*	1	93.854411	0.9119	2.3046	0.129448	0.0499	0.0080105215

ss64765844	*rs42864406*	1	93.887502	0.3761	0.0019	0.965354	0.0008	0.0003932930
ss61521463	*rs41619569*	1	93.920926	0.1934	2.3929	0.122343	0.0361	0.0112667963
ss61518305	*rs41613169*	1	93.945351	0.4885	1.1252	0.289167	0.0199	0.0099511141
ss61477935	*rs41581855*	1	93.993442	0.8689	1.5084	0.219802	0.0342	0.0077850421
ss64060115	*rs42454500*	1	94.052771	0.8080	1.2804	0.258211	0.0272	0.0084367501
ss63820980	*rs42369181*	1	94.078581	0.8940	2.2558	0.133568	0.0458	0.0086857193
ss61473011	*rs41566432*	1	94.181809	0.3245	0.0979	0.754470	0.0061	0.0026621006
ss61517777	*rs41619040*	1	94.216216	0.6189	0.3605	0.548406	0.0113	0.0053186995
ss86280345		1	94.303131	0.8331	0.1571	0.691962	0.0098	0.0027170500
ss117966183		1	94.33648	0.1662	0.1980	0.656510	0.0110	0.0030420244
	rs29019397	1	94.369685	0.7221	0.3156	0.574441	0.0115	0.0046204367
ss86324636		1	94.409184	0.6168	0.7693	0.380721	0.0163	0.0077169354
	rs29015706	1	94.470192	0.0881	2.5594	0.110093	0.0525	0.0084394801
ss61466511	*rs41570264*	1	94.653902	0.1397	3.5544	0.059803	0.0501	0.0120485068
	rs29016796	1	94.697346	0.1991	3.0852	0.079449	0.0403	0.0128414505
ss117966082		1	94.850835	0.5630	3.2579	0.071513	0.0336	0.0165357134
ss61538937	*rs41635504*	1	94.93108	0.5766	0.5191	0.471479	0.0135	0.0066027525
	rs29027508	1	94.958244	0.7206	1.4957	0.221741	0.0248	0.0099874330
ss61467677	*rs41570602*	1	95.08234	0.7801	0.2404	0.624038	0.0108	0.0037115641
ss86275322		1	95.148545	0.1583	1.7172	0.190484	0.0331	0.0088279336
ss61484063	*rs41583914*	1	95.245457	0.0530	0.3647	0.546124	0.0247	0.0024844950
ss86280743		1	95.279844	0.6819	1.9846	0.159349	0.0277	0.0120346658
ss61541968	*rs41639495*	1	95.317236	0.6676	3.0992	0.078771	0.0342	0.0151750715
	rs29013410	1	95.456256	0.2872	1.0337	0.309637	0.0204	0.0083434721
ss61484047	*rs41583899*	1	95.518231	0.7077	4.3172	0.038095	0.0411	0.0170020462
ss105265398		1	95.61657	0.0544	0.4639	0.496036	0.0269	0.0027674020
ss105265396		1	95.653468	0.1834	0.0039	0.950185	0.0015	0.0004535166
ss61484046	*rs41583898*	1	95.690573	0.2328	0.4998	0.479840	0.0159	0.0056828629
	rs29009621	1	95.777552	0.6848	2.3283	0.127498	0.0304	0.0131114171
ss61509140	*rs41601600*	1	95.898816	0.4907	1.2807	0.258162	0.0209	0.0104430210
ss62608709	*rs41930908*	1	95.930224	0.9434	0.9200	0.337806	0.0373	0.0039795616
ss117966137		1	95.972034	0.1218	11.9842	0.000569	0.0977	0.0208914670
ss63477596	*rs42244826*	1	96.000461	0.2443	1.4675	0.226152	0.0262	0.0096718227
ss86317537		1	96.129507	0.4549	0.0227	0.880324	0.0028	0.0013817180
ss86334953		1	96.15147	0.3309	0.0654	0.798206	0.0051	0.0022591099
ss86288878		1	96.182881	0.7600	0.0552	0.814237	0.0051	0.0018764172
ss63476801	*rs42243461*	1	96.228588	0.8001	2.9790	0.084796	0.0402	0.0128484979
ss86310041		1	96.288959	0.7908	2.6618	0.103239	0.0365	0.0120745455
ss64810213	*rs42910143*	1	96.315405	0.4749	0.5108	0.475030	0.0137	0.0068214234
ss63476669	*rs42243124*	1	96.337881	0.4491	0.4341	0.510192	0.0126	0.0062305500
ss63476582	*rs42243095*	1	96.379264	0.4649	1.3485	0.245931	0.0222	0.0110368099
ss63476558	*rs42243087*	1	96.408611	0.8682	6.0522	0.014131	0.0672	0.0153737864
ss63476519	*rs42243074*	1	96.436595	0.5236	1.7367	0.187990	0.0248	0.0123537984
ss64581692	*rs42707742*	1	96.646194	0.4427	7.4027	0.006675	0.0512	0.0252452456
ss86337300		1	96.692592	0.7156	13.6860	0.000233	0.0776	0.0315970476
	rs29016980	1	96.74425	0.8610	10.0697	0.001574	0.0858	0.0205440417
ss86314254		1	96.772613	0.2056	1.3358	0.248166	0.0267	0.0087341802

	rs29021342	1	96.826809	0.9026	1.6667	0.197123	0.0406	0.0071381516
ss61521580	*rs41620483*	1	96.957947	0.4226	6.5007	0.010996	0.0506	0.0246823437
ss105265421		1	97.0349	0.9405	4.8496	0.027979	0.0858	0.0096010933
ss61475479	*rs41575053*	1	97.068956	0.2242	5.1793	0.023160	0.0508	0.0176782959
ss86335092		1	97.100732	0.7514	2.9438	0.086651	0.0373	0.0139305582
ss86339213		1	97.169258	0.2192	4.0097	0.045627	0.0472	0.0161724465
ss86282474		1	97.197023	0.5014	13.3487	0.000278	0.0710	0.0355242739
ss86336652		1	97.518815	0.1934	9.2447	0.002450	0.0737	0.0229930581
ss38327071	*rs29019186*	1	97.545394	0.2106	0.8913	0.345453	0.0221	0.0073487571
ss61539901	*rs41638856*	1	97.601882	0.9441	1.1631	0.281188	0.0432	0.0045621372
ss61539903	*rs41638858*	1	97.648247	0.8066	2.1980	0.138639	0.0346	0.0108015787
ss61483006	*rs41583680*	1	97.674275	0.1454	5.8663	0.015688	0.0637	0.0158298018
	rs29015093	1	97.730993	0.3940	0.9005	0.342979	0.0186	0.0088997779
ss61539910	*rs41638865*	1	97.953338	0.7400	1.7853	0.181938	0.0287	0.0110290335
ss86334967		1	97.989629	0.2357	4.5258	0.033738	0.0479	0.0172475508
ss61539916	*rs41638871*	1	98.159158	0.7801	0.3636	0.546685	0.0134	0.0046043767
ss86320685		1	98.273352	0.5738	5.0626	0.024759	0.0427	0.0208849960
ss61539933	*rs41638888*	1	98.423737	0.4126	3.1573	0.076026	0.0339	0.0164345065
ss61482754	*rs41581638*	1	98.602705	0.7464	0.6627	0.415903	0.0169	0.0063981464
ss86326522		1	98.639695	0.8367	1.3605	0.243845	0.0284	0.0077592256
ss117963548		1	98.674391	0.8116	0.6560	0.418251	0.0191	0.0058352087
ss61539406	*rs41638957*	1	98.763466	0.1110	8.5298	0.003607	0.0844	0.0166578678
ss86290324		1	98.787467	0.7056	0.4155	0.519398	0.0129	0.0053583870
ss105259657		1	98.826238	0.2944	0.4155	0.519398	0.0129	0.0053583870
ss61482772	*rs41581656*	1	98.853038	0.7543	11.4005	0.000775	0.0706	0.0261692850
	rs29023726	1	98.876945	0.7801	8.1583	0.004414	0.0630	0.0216031428
ss86340551		1	98.906855	0.7686	1.8751	0.171338	0.0291	0.0103619958
ss61482773	*rs41581657*	1	98.94463	0.8130	4.3483	0.037409	0.0498	0.0151305885
ss86325070		1	98.970688	0.2908	0.5895	0.442884	0.0154	0.0063483942
ss86326646		1	99.00223	0.8517	0.2133	0.644329	0.0120	0.0030272979
ss61539417	*rs41638968*	1	99.075511	0.2414	1.8927	0.169337	0.0298	0.0109071467
ss86340629		1	99.110444	0.4993	0.0176	0.894583	0.0025	0.0012515991
ss86323957		1	99.212528	0.8926	8.2199	0.004269	0.0861	0.0165110851
ss61482778	*rs41581662*	1	99.456799	0.5903	0.2540	0.614419	0.0093	0.0045098810
ss61467740	*rs41571865*	1	99.506677	0.1232	1.1258	0.289044	0.0304	0.0065777182
ss86302766		1	99.607723	0.1726	0.4735	0.491622	0.0167	0.0047626812
ss86283737		1	99.686394	0.2615	8.7946	0.003125	0.0596	0.0230125282
ss86314300		1	99.752008	0.6060	4.8394	0.028145	0.0411	0.0196092105
	rs29011049	1	99.793771	0.1726	27.2317	0.000000	0.1232	0.0352079896
ss61522955	*rs41622824*	1	99.829786	0.4176	3.6489	0.056517	0.0351	0.0170793190
ss61482786	*rs43744169*	1	99.914084	0.8682	2.4446	0.118383	0.0434	0.0099304700
ss61482787	*rs41581670*	1	99.940813	0.6476	2.8891	0.089627	0.0332	0.0151595056
	rs29019531	1	100.026597	0.3940	9.0915	0.002661	0.0573	0.0273539259
ss65166625	*rs43254089*	1	100.161834	0.1046	2.2671	0.132600	0.0462	0.0086550039
	rs29025486	1	100.185633	0.1132	2.2445	0.134541	0.0448	0.0089898002
ss65166758	*rs43255022*	1	100.261282	0.4484	3.9508	0.047241	0.0370	0.0183123237
ss65166895	*rs43256659*	1	100.339278	0.4448	3.4629	0.063182	0.0345	0.0170264479

ss61539877	*rs41638832*	1	100.434164	0.8854	0.3359	0.562415	0.0169	0.0034314613
ss61539878	*rs41638833*	1	100.459885	0.2156	3.3938	0.065869	0.0424	0.0143262802
ss61539879	*rs41638834*	1	100.481932	0.5609	4.1537	0.041922	0.0370	0.0182424877
ss61539880	*rs41638835*	1	100.547473	0.8231	16.1257	0.000066	0.0999	0.0291073927
ss61508775	*rs41607232*	1	100.57467	0.7385	1.2494	0.264060	0.0243	0.0093896206
ss61539892	*rs41638847*	1	100.674986	0.5702	1.5255	0.217205	0.0230	0.0112898472
ss61483000	*rs41583674*	1	100.754012	0.5695	1.2992	0.254751	0.0215	0.0105330610
ss64336634	*rs42586578*	1	100.906055	0.5860	0.0047	0.945188	0.0013	0.0006272177
ss64336562	*rs42586542*	1	100.935002	0.4499	2.0335	0.154318	0.0266	0.0131814791
ss61567850	*rs41665648*	1	100.970946	0.6239	0.0491	0.824734	0.0043	0.0020263437
	rs29022894	1	101.001399	0.6762	0.8415	0.359276	0.0180	0.0078729034
ss64849183	*rs42940101*	1	101.09137	0.8718	0.0012	0.972096	0.0010	0.0002218937
ss105261920		1	101.116383	0.2765	0.0016	0.967842	0.0009	0.0003515440
ss61478035	*rs41582753*	1	101.161219	0.6719	1.9853	0.159283	0.0292	0.0128855167
ss61511825	*rs41615129*	1	101.205835	0.4563	0.1311	0.717416	0.0069	0.0034074044
ss86303063		1	101.253497	0.4871	0.7400	0.389951	0.0162	0.0080848248
ss61477836	*rs41580656*	1	101.333383	0.1683	0.1630	0.686498	0.0099	0.0027782203
ss64535046	*rs42683375*	1	101.468705	0.5279	0.8314	0.362195	0.0168	0.0083783649
ss64535278	*rs42684186*	1	101.525563	0.1289	13.5702	0.000248	0.1047	0.0235172759
ss65168854	*rs43256310*	1	101.560118	0.3431	2.2948	0.130258	0.0287	0.0129395505
ss61540463	*rs41643602*	1	101.584563	0.4262	8.9019	0.002948	0.0576	0.0281933631
	rs29020368	1	101.604608	0.5659	7.5130	0.006283	0.0523	0.0257185347
ss61540462	*rs41643601*	1	101.656858	0.2221	4.3767	0.036796	0.0473	0.0163462274
ss105261012		1	101.714381	0.8195	7.9321	0.004994	0.0660	0.0195397953
ss65168615	*rs43255173*	1	101.735867	0.1074	2.5544	0.110441	0.0482	0.0092433028
ss61508831	*rs41607285*	1	101.76355	0.0731	5.9477	0.014986	0.0866	0.0117245151
ss61483252	*rs41585823*	1	101.800482	0.4341	2.2409	0.134854	0.0281	0.0138169262
ss61483245	*rs41585816*	1	101.861685	0.6984	5.4882	0.019426	0.0482	0.0202961539
ss86299185		1	101.988988	0.5057	1.4754	0.224904	0.0228	0.0114078194
ss65168116	*rs43258773*	1	102.073416	0.1905	10.4400	0.001291	0.0762	0.0235022182
ss86297518		1	102.121833	0.1010	10.3723	0.001339	0.0970	0.0176187834
ss65168005	*rs43258062*	1	102.204001	0.0788	3.0015	0.083632	0.0587	0.0085197934
ss65167864	*rs43255821*	1	102.280845	0.3883	6.2892	0.012374	0.0497	0.0235928388
ss61540447	*rs41643588*	1	102.365561	0.2063	17.0622	0.000041	0.0960	0.0314445243
ss65167816	*rs43255773*	1	102.417281	0.1282	0.2692	0.604035	0.0142	0.0031804732
ss64649880	*rs42742620*	1	102.440408	0.8711	0.3270	0.567599	0.0157	0.0035166237
ss64649760	*rs42742505*	1	102.480517	0.8016	0.5973	0.439882	0.0181	0.0057677872
ss61516643	*rs41617944*	1	102.515261	0.1411	10.5703	0.001205	0.0863	0.0209290499
ss65167763	*rs43254920*	1	102.542345	0.1662	0.2911	0.589705	0.0134	0.0037067902
ss86292508		1	102.635377	0.1827	4.3386	0.037622	0.0496	0.0147963003
ss63867900	*rs42391114*	1	102.721656	0.1297	1.2979	0.254983	0.0314	0.0070834302
ss63867648	*rs42390288*	1	102.790745	0.1805	0.0006	0.979789	0.0006	0.0001840543
ss63867514	*rs42389621*	1	102.828369	0.7479	0.0113	0.915361	0.0023	0.0008833386
ss63867284	*rs42388806*	1	102.875006	0.1361	7.0077	0.008300	0.0721	0.0169658322
ss61520099		1	103.004836	0.6275	2.0531	0.152349	0.0279	0.0130464137
ss117966351		1	103.025506	0.5845	4.5718	0.032849	0.0408	0.0198325550
ss63867094	*rs42387812*	1	103.059782	0.9491	2.3851	0.122952	0.0652	0.0062938354

ss61476625	*rs41577779*	1	103.095845	0.4033	3.3711	0.066777		0.0344	0.0165465472
ss61506812	*rs41608306*	1	103.167664	0.5924	0.3281	0.566966		0.0109	0.0052802011
ss61517013	*rs41621210*	1	103.229116	0.6913	2.4089	0.121104		0.0313	0.0133501894
ss61517016		1	103.275172	0.5587	0.5425	0.461636		0.0141	0.0069383020
ss63626415	*rs42295315*	1	103.356064	0.2335	8.0045	0.004800		0.0606	0.0217112187
ss65012451	*rs43103300*	1	103.377348	0.2479	8.8086	0.003101		0.0616	0.0229611969
		1	103.402662	0.3653	3.7844	0.052133		0.0381	0.0176613870
	rs29026147	1	103.459112	0.5802	9.5586	0.002070		0.0584	0.0284714585
ss63626289	*rs42295273*	1	103.501423	0.3109	0.7418	0.389369		0.0171	0.0073139098
ss61567732	*rs41665136*	1	103.536855	0.9112	0.3161	0.574116		0.0180	0.0029069394
	rs29020933	1	103.577743	0.4613	0.0004	0.984088		0.0004	0.0001807927
ss61567727	*rs41665131*	1	103.600904	0.5365	0.5406	0.462418		0.0134	0.0066558690
ss61496389		1	103.637986	0.3345	0.7050	0.401384		0.0161	0.0071547451
ss63625404	*rs42293088*	1	103.684112	0.4893	0.7930	0.373506		0.0168	0.0083993038
ss63625293	*rs42293051*	1	103.709257	0.8317	7.3807	0.006757		0.0663	0.0185550407
ss63624894	*rs42291618*	1	103.737793	0.8639	3.9298	0.047831		0.0541	0.0127280764
ss117975115		1	103.842184	0.8317	4.9879	0.025842		0.0571	0.0159853603
ss117975116		1	103.924192	0.5014	0.3914	0.531786		0.0117	0.0058289760
ss64702504	*rs42797539*	1	103.987161	0.0874	0.0537	0.816776		0.0077	0.0012303497
ss64827910	*rs42918612*	1	104.031791	0.0559	0.5557	0.456238		0.0303	0.0031966865
ss62702145	*rs41968322*	1	104.120823	0.9448	0.3995	0.527577		0.0258	0.0026923516
ss62701998	*rs41968279*	1	104.142428	0.0587	0.4577	0.498906		0.0269	0.0029773854
ss62701872	*rs41968237*	1	104.164419	0.9448	0.3995	0.527577		0.0258	0.0026923516
ss61543973	*rs41639575*	1	104.238167	0.1956	1.8149	0.178364		0.0311	0.0097868895
ss86297974		1	104.28853	0.1848	0.8707	0.351074		0.0221	0.0066672754
ss65171513	*rs43257460*	1	104.370684	0.1898	0.0303	0.861782		0.0042	0.0012795695
ss86308545		1	104.402766	0.6905	2.1445	0.143529		0.0305	0.0130221836
ss61540930	*rs41639071*	1	104.491235	0.3016	6.9262	0.008682		0.0537	0.0226154317
ss86284790		1	104.535802	0.7242	0.8479	0.357452		0.0199	0.0079611028
ss105275357		1	104.563153	0.7192	1.1006	0.294500		0.0225	0.0090711275
ss64775302	*rs42870862*	1	104.601642	0.7242	0.8479	0.357452		0.0199	0.0079611028
ss65008238	*rs43104499*	1	104.637962	0.7221	1.1473	0.284488		0.0229	0.0092019921
ss61483262	*rs41576339*	1	104.670913	0.4226	0.5391	0.463034		0.0144	0.0070262167
ss65171100	*rs43259751*	1	104.72991	0.4986	0.5714	0.449941		0.0145	0.0072499016
ss61540492	*rs41644831*	1	104.766981	0.5007	0.5562	0.456050		0.0143	0.0071641603
ss65170916	*rs43258367*	1	104.804026	0.8718	1.4869	0.223103		0.0331	0.0074007556
ss65170894	*rs43258348*	1	104.857838	0.0709	0.0662	0.797028		0.0090	0.0011843413
ss65170815	*rs43257669*	1	104.888331	0.9248	0.0031	0.955834		0.0019	0.0002609524
ss86306448		1	104.922159	0.4542	0.5952	0.440690		0.0145	0.0071739853
ss65131565	*rs43221199*	1	105.164598	0.1110	0.0542	0.815993		0.0068	0.0013500565
ss65131124	*rs43217858*	1	105.349689	0.8152	0.6443	0.422425		0.0188	0.0056580996
ss61481711	*rs41580916*	1	105.441846	0.8166	0.4775	0.489804		0.0162	0.0048481073
ss61537364	*rs41639661*	1	105.499699	0.6440	1.2258	0.268617		0.0217	0.0099635477
ss65170726	*rs43256880*	1	105.550684	0.3782	1.5004	0.221026		0.0241	0.0113419284
ss65170755	*rs43256909*	1	105.600777	0.4062	20.7358	0.000006		0.0868	0.0418709498
ss65088037	*rs43174752*	1	105.688295	0.3746	1.8438	0.174941		0.0261	0.0122430755
ss65169998	*rs43259154*	1	105.78139	0.3524	2.2982	0.129978		0.0297	0.0135674582

ss61531716	*rs41629268*	1	105.829264	0.3696	41.9605	0.000000	0.1255	0.0584831443
ss64848121	*rs42940149*	1	105.918418	0.6132	37.9630	0.000000	0.1172	0.0555852006
ss64558112	*rs42698829*	1	106.014837	0.3897	37.6324	0.000000	0.1156	0.0550026752
ss64557900	*rs42697824*	1	106.079524	0.2679	34.2926	0.000000	0.1218	0.0477880107
ss61531203	*rs41633163*	1	106.204208	0.3875	38.1800	0.000000	0.1172	0.0556472115
ss65022303	*rs43113331*	1	106.237649	0.6125	38.1800	0.000000	0.1172	0.0556472115
ss64563276	*rs42699492*	1	106.271502	0.1160	0.0351	0.851499	0.0054	0.0011133470
ss61537376		1	106.29862	0.2428	0.3590	0.549284	0.0131	0.0048027150
ss65109732	*rs43197096*	1	106.367469	0.0953	0.0121	0.912266	0.0034	0.0005834604
ss65169112	*rs43257968*	1	106.46996	0.1383	0.0659	0.797533	0.0070	0.0016575646
ss65168892	*rs43256448*	1	106.581506	0.2457	2.2868	0.130933	0.0332	0.0122905746
ss61508834	*rs41607288*	1	106.618401	0.5279	0.0024	0.960772	0.0009	0.0004618240
ss61483255	*rs41576332*	1	106.683503	0.8904	0.9514	0.329705	0.0286	0.0055916170
ss61568394	*rs41663692*	1	106.738822	0.6963	0.9439	0.331621	0.0196	0.0082890070
ss86302537		1	106.837146	0.4219	3.7475	0.053292	0.0368	0.0179283252
ss86337507		1	106.943463	0.7443	18.1337	0.000023	0.0909	0.0345901963
		1	106.965027	0.1913	5.1292	0.023834	0.0536	0.0165773731
ss65173017	*rs43263261*	1	107.216857	0.8066	11.8700	0.000605	0.0819	0.0255625511
ss65172971	*rs43262015*	1	107.238265	0.4885	0.1261	0.722612	0.0067	0.0033418273
ss65172949	*rs43261993*	1	107.267244	0.8080	11.7816	0.000634	0.0821	0.0254804896
	rs29011752	1	107.291206	0.4885	0.1261	0.722612	0.0067	0.0033418273
	rs29012506	1	107.399663	0.5007	21.8665	0.000004	0.0862	0.0430806360
ss61567522	*rs41663229*	1	107.442325	0.2607	1.2070	0.272302	0.0237	0.0091536477
ss117966199		1	107.487718	0.8854	1.0227	0.312231	0.0299	0.0060690001
ss61483555	*rs41578722*	1	107.547213	0.6476	26.5033	0.000000	0.0984	0.0449149681
ss61567530	*rs41663537*	1	107.630272	0.2457	2.9980	0.083809	0.0371	0.0137594956
ss61540998	*rs41640336*	1	107.653497	0.7500	2.9012	0.088962	0.0365	0.0136919159
ss65172834	*rs43261179*	1	107.677595	0.7371	2.1680	0.141362	0.0304	0.0117766270
ss65172745	*rs43260190*	1	107.743804	0.4133	0.2085	0.648119	0.0085	0.0041176520
ss65172692	*rs43260137*	1	107.76986	0.5903	0.1451	0.703352	0.0071	0.0034213455
ss61540974	*rs41578721*	1	107.803284	0.4083	0.2212	0.638271	0.0087	0.0042228954
ss65172659	*rs43259704*	1	107.837379	0.1834	0.5764	0.447971	0.0183	0.0054848189
ss86338825		1	107.858786	0.4771	0.3263	0.568035	0.0105	0.0052393057
ss65172529	*rs43258874*	1	107.916846	0.8933	0.8071	0.369277	0.0266	0.0050724933
ss61540971		1	108.045326	0.5358	15.6922	0.000082	0.0726	0.0361171628
ss65172344	*rs43256591*	1	108.073678	0.3746	37.5848	0.000000	0.1164	0.0545369840
ss61540970	*rs41639109*	1	108.137412	0.6934	13.9652	0.000201	0.0764	0.0324743949
ss65172247	*rs43255695*	1	108.181536	0.4040	13.4725	0.000261	0.0693	0.0333908774
ss86338785		1	108.205447	0.4635	17.2379	0.000037	0.0761	0.0378228619
ss117966313		1	108.250419	0.4513	6.9832	0.008413	0.0481	0.0238214036
ss65171854	*rs43260098*	1	108.290501	0.5788	3.7702	0.052575	0.0360	0.0175494989
ss65171775	*rs43259319*	1	108.316537	0.5781	3.6634	0.056029	0.0355	0.0173250346
ss117963509		1	108.365652	0.5731	4.3311	0.037786	0.0387	0.0189139471
	rs29022224	1	108.416178	0.1977	5.8149	0.016149	0.0547	0.0173602095
ss65173270	*rs43257188*	1	108.511195	0.7772	9.9547	0.001673	0.0718	0.0248626736
ss65173285	*rs43257203*	1	108.549474	0.7772	8.5263	0.003614	0.0665	0.0230440626
ss65364546	*rs43449923*	1	108.622399	0.1927	11.2087	0.000858	0.0807	0.0251122100

ss86294577		1	108.652356	0.1956	10.9126	0.001004		0.0795	0.0250039388
ss65173863	*rs43263479*	1	108.863906	0.5043	14.7518	0.000134		0.0719	0.0359241397
ss61476352	*rs41575613*	1	108.937104	0.1046	1.5052	0.220287		0.0376	0.0070494695
ss61523818	*rs41623577*	1	108.967416	0.8188	13.7324	0.000228		0.0897	0.0266229473
ss65169843	*rs43257799*	1	109.028053	0.5272	0.1467	0.701790		0.0074	0.0037115902
ss117966387		1	109.08758	0.4921	2.5710	0.109295		0.0307	0.0153622655
ss105259680		1	109.108004	0.1855	10.8164	0.001057		0.0802	0.0242314758
ss86273774		1	109.132511	0.2858	0.4372	0.508710		0.0137	0.0056117025
ss86336803		1	109.182737	0.3718	11.0371	0.000940		0.0630	0.0294165844
ss86337776		1	109.215863	0.3238	9.0587	0.002709		0.0610	0.0267267822
ss65169478	*rs43255435*	1	109.252871	0.1683	0.0422	0.837319		0.0052	0.0014688062
ss61540465	*rs41643604*	1	109.285801	0.5881	7.2715	0.007175		0.0500	0.0242220042
ss61483562	*rs41578729*	1	109.329096	0.5795	12.4575	0.000444		0.0652	0.0317752308
ss65173564	*rs43260382*	1	109.386042	0.6168	6.3612	0.011886		0.0473	0.0223787802
ss65173712	*rs43261328*	1	109.481574	0.5029	1.6072	0.205315		0.0236	0.0118188212
ss64037158	*rs42443533*	1	109.547033	0.0860	1.4597	0.227387		0.0405	0.0063576299
ss61524405	*rs41620647*	1	109.569254	0.5043	6.2994	0.012304		0.0469	0.0234425239
ss86339512		1	109.608522	0.0981	0.1849	0.667287		0.0135	0.0023932759
ss61522637	*rs41620808*	1	109.643162	0.3245	1.5562	0.212648		0.0248	0.0108623070
ss61522635		1	109.682945	0.4577	0.2476	0.618942		0.0092	0.0045590176
ss86338626		1	109.717258	0.2228	3.7150	0.054331		0.0435	0.0150623937
ss86285022		1	109.800213	0.1991	4.2234	0.040243		0.0480	0.0153015034
ss61522638	*rs41620809*	1	109.836862	0.9119	0.6136	0.433713		0.0260	0.0041805175
ss61507051	*rs41602353*	1	109.87196	0.1096	3.2623	0.071321		0.0541	0.0105571640
ss117966342		1	109.917172	0.8875	3.4576	0.063382		0.0548	0.0109432294
ss63925182	*rs42283451*	1	109.945203	0.8868	3.1603	0.075887		0.0523	0.0105010721
ss64977352	*rs43070837*	1	109.96951	0.5530	17.3454	0.000035		0.0779	0.0384913853
ss61506390	*rs41605402*	1	110.016501	0.5344	20.2257	0.000008		0.0853	0.0424698855
ss86307480		1	110.03662	0.4936	13.5837	0.000246		0.0692	0.0345837784
ss65024015	*rs43111834*	1	110.073711	0.4914	12.4203	0.000452		0.0663	0.0331443783
ss61531010	*rs41631073*	1	110.100385	0.0351	2.8950	0.089301		0.0828	0.0056068054
ss86336502		1	110.193984	0.6999	9.2769	0.002408		0.0605	0.0254156533
ss86273989		1	110.215354	0.4405	0.1426	0.705783		0.0071	0.0035005156
ss86282291		1	110.246342	0.2758	5.2436	0.022326		0.0477	0.0190705205
ss63801846	*rs42355462*	1	110.27861	0.5129	9.2837	0.002400		0.0571	0.0285292826
ss61475518	*rs41575091*	1	110.308505	0.6662	0.4106	0.521883		0.0128	0.0057042012
ss63801746	*rs42360611*	1	110.353592	0.4348	1.3031	0.254035		0.0217	0.0106900735
ss86285948		1	110.40402	0.4441	0.8644	0.352842		0.0178	0.0087773627
ss86339126		1	110.424874	0.4398	0.9305	0.335077		0.0184	0.0090536700
ss61511965	*rs41609675*	1	110.46203	0.8388	2.2315	0.135674		0.0387	0.0104623828
ss86297438		1	110.51498	0.5322	0.7411	0.389614		0.0161	0.0080341089
ss86341492		1	110.53718	0.9362	1.0487	0.306161		0.0381	0.0045440674
	rs29014005	1	110.567888	0.8804	2.7798	0.095913		0.0501	0.0105437262
ss86323579		1	110.647651	0.2178	0.7223	0.395686		0.0199	0.0067900809
ss86299609		1	110.67961	0.9205	3.4216	0.064772		0.0631	0.0092326865
ss86340385		1	110.704594	0.8173	17.9135	0.000026		0.1023	0.0305325903
ss61517816	*rs41619079*	1	110.747606	0.2163	21.3931	0.000004		0.1027	0.0348169504

ss61517814	*rs41619077*	1	110.87222	0.6483	0.7093	0.399969	0.0163	0.0074292261
	rs29019282	1	110.940742	0.1375	0.5917	0.442019	0.0213	0.0050455712
ss61514385	*rs41611758*	1	111.058353	0.2228	1.7240	0.189609	0.0288	0.0099698716
ss64536422	*rs42682258*	1	111.091441	0.2228	1.7240	0.189609	0.0288	0.0099698716
ss64536440	*rs42682267*	1	111.113819	0.0602	8.0852	0.004594	0.1115	0.0126100688
ss65130418	*rs43219754*	1	111.322944	0.1390	2.6993	0.100847	0.0455	0.0108910936
ss86282828		1	111.60852	0.3789	4.4255	0.035764	0.0407	0.0191367496
ss105242387		1	111.631004	0.7880	10.7092	0.001119	0.0769	0.0256813501
ss63716076	*rs42322961*	1	111.745803	0.0795	18.2817	0.000022	0.1437	0.0210387250
ss105242389		1	111.804662	0.3725	4.1592	0.041786	0.0399	0.0186567525
ss63716220	*rs42323009*	1	111.830745	0.1246	2.7530	0.097522	0.0481	0.0105030306
ss61506008	*rs41601833*	1	111.891138	0.6805	5.9092	0.015314	0.0476	0.0206881512
ss86300741		1	111.918114	0.5057	4.2766	0.039010	0.0393	0.0196313958
ss61513273		1	111.957695	0.0666	3.7547	0.053062	0.0713	0.0088724006
ss61513279	*rs41611562*	1	112.000088	0.8331	7.0770	0.007988	0.0659	0.0183226094
ss61513282	*rs41611565*	1	112.036864	0.0723	11.6048	0.000696	0.1208	0.0162183235
ss63717030	*rs42324979*	1	112.059305	0.0903	0.0399	0.841723	0.0065	0.0010601103
ss86321784		1	112.095726	0.9277	10.4707	0.001270	0.1148	0.0154147371
ss86288192		1	112.143241	0.3274	1.2872	0.256951	0.0230	0.0101207649
ss61513278	*rs41611561*	1	112.234095	0.9090	0.2568	0.612506	0.0163	0.0027006726
ss86339175		1	112.257304	0.5817	1.8418	0.175182	0.0258	0.0125551907
ss86341222		1	112.319361	0.1691	5.8330	0.015985	0.0583	0.0163921579
ss61568072	*rs41661378*	1	112.52828	0.9585	0.0317	0.858831	0.0082	0.0006543938
ss63636151	*rs42296756*	1	112.552839	0.1103	10.6838	0.001134	0.0957	0.0187873672
ss86291804		1	112.661372	0.4943	0.3963	0.529228	0.0120	0.0059761261
ss86301375		1	112.691946	0.4957	0.8237	0.364424	0.0172	0.0086125236
ss86340569		1	112.798672	0.2937	1.7923	0.181079	0.0270	0.0111961885
ss86296970		1	112.847407	0.1490	4.2097	0.040567	0.0530	0.0134490796
ss86320086		1	112.880131	0.0702	11.6367	0.000684	0.1224	0.0159748113
ss86336327		1	113.130882	0.8173	1.1308	0.287977	0.0266	0.0079382069
ss65071112	*rs43165194*	1	113.223242	0.3403	0.0255	0.873180	0.0031	0.0014077157
ss86334289		1	113.265667	0.7278	4.2592	0.039408	0.0438	0.0173489031
ss86335400		1	113.307506	0.9255	9.8683	0.001753	0.1103	0.0152093846
ss65173740	*rs43262256*	1	113.338242	0.2744	4.2777	0.038985	0.0434	0.0172829048
ss86312505		1	113.395008	0.2643	7.6023	0.005982	0.0582	0.0226536326
ss61473861	*rs41574371*	1	113.475561	0.9026	13.2858	0.000287	0.1125	0.0197913298
ss105297176		1	113.500011	0.0924	16.9857	0.000042	0.1293	0.0216802969
ss63857788	*rs42388649*	1	113.521537	0.6375	5.8571	0.015770	0.0459	0.0212338479
ss61536598	*rs41632723*	1	113.786292	0.4441	15.9354	0.000073	0.0749	0.0370011861
ss86315196		1	113.826464	0.6325	5.5116	0.019170	0.0456	0.0211943003
ss105242396		1	113.879898	0.4219	23.8643	0.000001	0.0914	0.0445645481
ss86318478		1	114.010037	0.7607	0.6898	0.406524	0.0177	0.0064272714
ss117963544		1	114.072353	0.3410	0.0003	0.985534	0.0004	0.0001582692
ss65174190	*rs43266806*	1	114.096268	0.3403	0.0021	0.963548	0.0009	0.0003995364
ss62517829	*rs41897688*	1	114.148558	0.0602	0.8221	0.364872	0.0362	0.0040924553
ss62517784	*rs41897673*	1	114.188685	0.7221	0.1245	0.724322	0.0076	0.0030386660
ss65174205	*rs43266821*	1	114.246014	0.2729	15.6443	0.000084	0.0828	0.0328526247

ss86335115		1	114.280345	0.2507	12.0800	0.000541	0.0756	0.0284192445
ss61467890	*rs41573010*	1	114.412542	0.2529	2.2522	0.133877	0.0323	0.0122075260
ss86303810		1	114.523869	0.6375	10.9488	0.000985	0.0627	0.0289833136
ss86338315		1	114.544237	0.0501	1.2905	0.256346	0.0491	0.0046732553
ss86298296		1	114.569286	0.3252	1.1569	0.282476	0.0212	0.0092967635
ss61483793		1	114.6704	0.7407	6.1996	0.013010	0.0538	0.0206508612
ss86338265		1	114.747946	0.0587	0.0211	0.884449	0.0056	0.0006162929
ss105297276		1	114.796882	0.5630	10.5586	0.001212	0.0590	0.0290452079
	rs29023119	1	114.87133	0.4907	0.0020	0.964102	0.0008	0.0004117439
	rs29019540	1	114.943931	0.4477	3.1364	0.076998	0.0320	0.0158365981
ss61564430	*rs41660601*	1	115.085095	0.7428	8.0583	0.004662	0.0591	0.0225979119
ss63391759	*rs42218659*	1	115.122127	0.2185	3.7337	0.053730	0.0440	0.0150246133
ss86304307		1	115.16286	0.2285	0.2492	0.617797	0.0109	0.0038468873
ss63391717	*rs42218645*	1	115.201744	0.4871	4.2426	0.039792	0.0380	0.0189955904
ss61523840	*rs41623597*	1	115.27915	0.5064	2.9230	0.087770	0.0316	0.0157984036
ss86283341		1	115.311865	0.1039	1.2317	0.267451	0.0329	0.0061154450
ss63391193	*rs42215609*	1	115.36331	0.5530	0.6857	0.407910	0.0151	0.0074779132
ss86285322		1	115.391395	0.3367	2.1262	0.145248	0.0278	0.0124344024
	rs29025571	1	115.442372	0.6633	2.1262	0.145248	0.0278	0.0124344024
ss63390705	*rs42223561*	1	115.542123	0.3954	2.0373	0.153926	0.0274	0.0130807738
ss86299163		1	115.696833	0.2013	7.2130	0.007410	0.0627	0.0201679526
ss61564446	*rs41660617*	1	115.727044	0.3274	5.7151	0.017084	0.0471	0.0207626588
ss64667759	*rs42762584*	1	115.795296	0.1211	0.3288	0.566562	0.0160	0.0034081244
ss63389799	*rs42218212*	1	115.97576	0.0766	7.4225	0.006603	0.0968	0.0137070368
ss61564427	*rs41660598*	1	116.178359	0.3768	0.5722	0.449661	0.0148	0.0069566599
ss61496069	*rs41595625*	1	116.337327	0.4921	8.3909	0.003889	0.0549	0.0274490908
ss86292398		1	116.445186	0.5050	7.7308	0.005575	0.0524	0.0261992190
ss65174396	*rs43258512*	1	116.476339	0.8976	0.8715	0.350862	0.0276	0.0050712846
ss86307161		1	116.501524	0.1168	1.7096	0.191463	0.0371	0.0076487615
ss86323035		1	116.577669	0.3309	0.3804	0.537613	0.0120	0.0052928791
ss86287160		1	116.611633	0.3374	0.2340	0.628750	0.0093	0.0041742115
ss64311428	*rs42575643*	1	116.719177	0.1468	2.7746	0.096222	0.0428	0.0107242534
ss86317083		1	116.758709	0.3288	2.1508	0.142949	0.0288	0.0127246831
ss61567351	*rs41662567*	1	116.795837	0.5408	0.4182	0.518055	0.0119	0.0059257914
ss117963470		1	116.820291	0.4728	2.9081	0.088583	0.0310	0.0154534564
ss64761632	*rs42859069*	1	116.869213	0.0953	1.0969	0.295316	0.0321	0.0055422017
ss86298154		1	116.951612	0.6977	2.9897	0.084241	0.0339	0.0143125161
ss61541505	*rs41645038*	1	117.019092	0.6325	0.0781	0.779921	0.0052	0.0024262283
	rs29016851	1	117.145831	0.7894	8.1103	0.004531	0.0629	0.0209112710
ss61570066	*rs41664832*	1	117.242906	0.7170	2.2083	0.137718	0.0299	0.0121462736
ss86288439		1	117.272466	0.0967	16.1556	0.000065	0.1224	0.0213841330
ss64548974	*rs42691194*	1	117.318988	0.4448	4.0639	0.044192	0.0371	0.0183484499
ss65056601	*rs43146027*	1	117.526929	0.8725	14.1022	0.000188	0.1044	0.0232206942
ss61514574	*rs41614035*	1	117.582503	0.3582	2.5375	0.111622	0.0311	0.0142889681
ss61495046	*rs41592529*	1	117.659662	0.1074	4.8749	0.027576	0.0640	0.0122686317
ss65175500	*rs43258621*	1	117.804167	0.3797	8.6315	0.003413	0.0546	0.0256993238
ss61502248	*rs41605459*	1	117.879706	0.6340	3.3800	0.066417	0.0344	0.0159489939

ss65062608	*rs43156801*	1	117.935281	0.7221	3.5362	0.060458	0.0393	0.0157775821
ss64640983	*rs42743786*	1	117.960567	0.4434	0.9627	0.326838	0.0184	0.0090743712
ss61541928	*rs41639459*	1	118.134338	0.2994	0.0002	0.989825	0.0003	0.0001051740
ss86320179		1	118.23294	0.4355	8.6478	0.003383	0.0555	0.0272753789
ss61476883	*rs41580133*	1	118.270529	0.6196	7.2150	0.007402	0.0506	0.0238687010
	rs29019947	1	118.304465	0.9219	0.0544	0.815659	0.0080	0.0011560133
ss64009798	*rs42432973*	1	118.365634	0.4427	0.6704	0.413186	0.0152	0.0074796559
ss64009770	*rs42432959*	1	118.393694	0.5136	0.0503	0.822607	0.0042	0.0021102666
ss86278125		1	118.422822	0.3617	0.9950	0.318882	0.0194	0.0089354540
ss64659201	*rs42750853*	1	118.460091	0.5681	18.4578	0.000020	0.0778	0.0381929423
ss61506398	*rs41605410*	1	118.496249	0.1999	0.0193	0.889556	0.0031	0.0009937648
ss64395232	*rs42618761*	1	118.584676	0.1870	0.0617	0.803860	0.0057	0.0017190290
ss61497091	*rs41594021*	1	118.619266	0.0917	15.8601	0.000075	0.1246	0.0207574154
ss117963473		1	118.663239	0.8188	34.9105	0.000000	0.1407	0.0417650741
ss61569216	*rs41665600*	1	118.692413	0.1017	20.1417	0.000008	0.1332	0.0243383181
ss65175744	*rs43261465*	1	118.767793	0.3166	0.4423	0.506237	0.0133	0.0057739071
ss86323251		1	118.800644	0.1848	1.5658	0.211244	0.0295	0.0088987002
ss61541961	*rs41583907*	1	118.861849	0.5895	2.2953	0.130218	0.0289	0.0139669009
ss61541959	*rs41639487*	1	118.891283	0.2550	5.1804	0.023146	0.0468	0.0177699407
ss65176096	*rs43264910*	1	118.923301	0.0953	3.2221	0.073085	0.0550	0.0094779785
ss86316868		1	118.986155	0.5322	2.7028	0.100624	0.0299	0.0148886291
ss65176133	*rs43265947*	1	119.24211	0.1196	9.9127	0.001712	0.0894	0.0188396930
ss61509040	*rs41601104*	1	119.392788	0.5666	1.2462	0.264656	0.0206	0.0101198005
ss61484344	*rs41578197*	1	119.440108	0.7679	0.9557	0.328615	0.0210	0.0074779770
ss65176723	*rs43262828*	1	119.46789	0.1361	6.2232	0.012840	0.0672	0.0158085736
ss65176696	*rs43262801*	1	119.501868	0.8696	7.7036	0.005659	0.0756	0.0171404014
ss65176680	*rs43262785*	1	119.530318	0.7443	4.1765	0.041364	0.0437	0.0166348139
ss61497499	*rs41596925*	1	119.572421	0.3195	0.0720	0.788546	0.0054	0.0023352095
ss86315616		1	119.610082	0.1325	23.3546	0.000002	0.1287	0.0295950770
ss65176410	*rs43260018*	1	119.673817	0.9284	0.2441	0.621382	0.0180	0.0023879294
ss61541966	*rs41639493*	1	119.695611	0.7221	0.6249	0.429504	0.0163	0.0065511795
ss65176516	*rs43260623*	1	119.71874	0.9284	0.2441	0.621382	0.0180	0.0023879294
ss86334877		1	119.740644	0.7271	0.3594	0.549057	0.0126	0.0049954118
ss65175757	*rs43261478*	1	119.835791	0.5817	6.5914	0.010455	0.0485	0.0235870272
ss64485710	*rs42660905*	1	119.897273	0.0716	15.3655	0.000097	0.1408	0.0187235349
ss64485598	*rs42660849*	1	119.935083	0.5523	7.2330	0.007329	0.0512	0.0253372130
ss61485283	*rs41585307*	1	120.135284	0.2249	1.0746	0.300263	0.0235	0.0081990369
ss86290300		1	120.24929	0.3066	15.2276	0.000105	0.0772	0.0328040553
ss86295730		1	120.277405	0.1053	5.1820	0.023125	0.0679	0.0128011259
ss61544706	*rs41637805*	1	120.372216	0.1125	1.6647	0.197396	0.0378	0.0075470989
ss65029012	*rs43120098*	1	120.396682	0.3675	0.3420	0.558892	0.0111	0.0051489959
ss86336916		1	120.418673	0.1934	12.7586	0.000379	0.0815	0.0254157601
ss64894442	*rs42986744*	1	120.443788	0.3474	3.6461	0.056611	0.0359	0.0162635363
ss86340544		1	120.468145	0.3252	0.1681	0.681933	0.0080	0.0034948131
ss65176946	*rs43265648*	1	120.541653	0.2887	5.3829	0.020623	0.0466	0.0191438188
ss86284768		1	120.575773	0.6734	13.7981	0.000220	0.0730	0.0321202406
ss86339381		1	120.714933	0.0688	0.8996	0.343215	0.0349	0.0044756421

ss86321184		1	120.738404	0.7371	19.3611	0.000013	0.0917	0.0355417253
ss65177035	*rs43266535*	1	120.794848	0.7636	7.8248	0.005296	0.0615	0.0221943419
ss64960060	*rs43051807*	1	120.841265	0.2708	7.3705	0.006795	0.0568	0.0224345078
ss65177629	*rs43263227*	1	120.87895	0.9226	29.1454	0.000000	0.1828	0.0260906800
ss61542669	*rs41635996*	1	120.914953	0.2471	2.4748	0.116139	0.0336	0.0125083284
ss86280063		1	120.937312	0.8266	3.5374	0.060417	0.0456	0.0130638911
ss105304466		1	120.983738	0.5752	1.2367	0.266482	0.0204	0.0099452016
ss65177446	*rs43262044*	1	121.00904	0.7486	4.0319	0.045035	0.0428	0.0161058563
ss117966381		1	121.159769	0.5723	0.0089	0.924953	0.0018	0.0008861466
ss105304387		1	121.312883	0.3782	0.0977	0.754681	0.0057	0.0026819589
ss117966097		1	121.339548	0.8309	7.4722	0.006425	0.0676	0.0189850238
ss61494206	*rs41594412*	1	121.366206	0.4678	13.2410	0.000294	0.0661	0.0329193401
ss65176334	*rs43259942*	1	121.510242	0.2063	14.7634	0.000133	0.0882	0.0288931534
ss64349061	*rs42596239*	1	121.532195	0.8245	14.3027	0.000169	0.0911	0.0263693745
ss86329455		1	121.607486	0.8138	8.1644	0.004400	0.0664	0.0201236651
ss65176358	*rs43259966*	1	121.662882	0.2787	2.7429	0.098141	0.0344	0.0138461848
ss65177843	*rs43266641*	1	121.769775	0.2822	1.4933	0.222124	0.0252	0.0102235455
ss105239551		1	121.811393	0.4649	10.5172	0.001239	0.0606	0.0301317193
ss61493410	*rs41594942*	1	121.969703	0.6705	1.6394	0.200830	0.0258	0.0113877688
ss61484351	*rs41578204*	1	122.483958	0.4577	3.7277	0.053923	0.0362	0.0179493645
ss64920982	*rs43010529*	1	122.909909	0.4850	0.1126	0.737297	0.0064	0.0031959601
ss63567001	*rs42272883*	1	122.957014	0.1819	0.0725	0.787784	0.0064	0.0018926690
ss63566794	*rs42271814*	1	123.009475	0.1755	0.0273	0.868884	0.0040	0.0011514232
ss117974853		1	123.04193	0.2744	0.0153	0.901719	0.0026	0.0010255513
ss86336127		1	123.116252	0.4663	0.2881	0.591600	0.0099	0.0049380301
ss117974856		1	123.143441	0.1347	17.7057	0.000029	0.1127	0.0262556894
ss64810381	*rs42911610*	1	123.290078	0.7930	1.7855	0.181916	0.0302	0.0099217191
ss61509083	*rs41601545*	1	123.423499	0.6504	0.0292	0.864398	0.0032	0.0014643585
ss61484354	*rs41578207*	1	123.444392	0.6676	0.0965	0.756127	0.0061	0.0026941662
ss65178569	*rs43262568*	1	123.519598	0.7321	8.1880	0.004343	0.0561	0.0220071044
ss61525441	*rs41621057*	1	123.61035	0.8653	1.1366	0.286746	0.0281	0.0065528828
ss86286835		1	123.679885	0.3331	4.5576	0.033121	0.0414	0.0183791959
ss61542683	*rs41636008*	1	123.727694	0.8453	0.0007	0.978765	0.0007	0.0001766476
ss65178278	*rs43269074*	1	123.858736	0.8768	0.1901	0.662991	0.0122	0.0026271612
ss65178817	*rs43263816*	1	123.88883	0.4620	5.4169	0.020228	0.0418	0.0207657495
ss61484361	*rs41578214*	1	123.918204	0.5881	0.7342	0.391816	0.0159	0.0077076112
ss61542694	*rs41636018*	1	124.083398	0.2887	0.7511	0.386430	0.0170	0.0069983077
ss65178678	*rs43263077*	1	124.10538	0.2142	4.7151	0.030235	0.0477	0.0160570093
ss86298836		1	124.129919	0.5236	4.1835	0.041195	0.0372	0.0185541060
ss105240261		1	124.198464	0.7937	0.1482	0.700343	0.0087	0.0028590613
ss86327895		1	124.25336	0.6533	0.0152	0.901890	0.0024	0.0011005560
ss64162853	*rs42502557*	1	124.280496	0.1160	0.1532	0.695595	0.0115	0.0023679510
	rs29020824	1	124.339372	0.5860	0.9126	0.339755	0.0184	0.0089047014
ss86317971		1	124.454759	0.1024	0.0889	0.765641	0.0093	0.0017130536
ss64712560	*rs42810757*	1	124.477746	0.1010	0.0074	0.931700	0.0026	0.0004681128
ss64712520	*rs42809517*	1	124.499732	0.8818	0.9517	0.329632	0.0277	0.0057765133
ss64712479	*rs42809476*	1	124.523168	0.3087	0.9048	0.341832	0.0189	0.0080572781

ss86323467		1	124.554177	0.2966	0.1531	0.695700	0.0079	0.0032968162
ss65178869	*rs43264168*	1	124.658903	0.1554	27.9241	0.000000	0.1330	0.0349331448
ss65178855	*rs43264154*	1	124.695554	0.3754	13.9985	0.000198	0.0704	0.0330049335
ss65180237	*rs43266936*	1	124.726924	0.1791	11.8649	0.000606	0.0819	0.0240904012
ss117963554		1	124.766306	0.8159	24.9744	0.000001	0.1154	0.0346727968
ss65180146	*rs43266445*	1	124.79792	0.1984	23.6636	0.000001	0.1097	0.0349051641
ss65179949	*rs43265048*	1	124.844876	0.1991	22.5158	0.000003	0.1075	0.0343016972
ss105240966		1	124.989231	0.6132	12.4408	0.000448	0.0655	0.0310849523
ss61543181	*rs41639296*	1	125.015814	0.3016	12.0230	0.000558	0.0689	0.0290087597
ss105240957		1	125.102104	0.3653	0.3159	0.574285	0.0108	0.0050126141
ss65179829	*rs43263928*	1	125.138085	0.2937	0.1890	0.663903	0.0087	0.0036285581
ss86303432		1	125.168156	0.7751	10.0758	0.001569	0.0693	0.0241600672
ss64356411	*rs42602615*	1	125.218595	0.4155	0.0775	0.780759	0.0051	0.0024767900
ss61496667	*rs41591319*	1	125.264325	0.2264	10.7721	0.001082	0.0716	0.0250704552
ss61522242	*rs41617727*	1	125.330706	0.5910	3.0431	0.081523	0.0330	0.0159348924
ss86274007		1	125.411898	0.3424	10.9735	0.000972	0.0650	0.0292785808
ss61477377	*rs41575914*	1	125.572319	0.1375	13.0389	0.000327	0.0958	0.0227383946
ss61528052	*rs41629193*	1	125.705517	0.7342	0.8048	0.369976	0.0190	0.0074335728
ss117966305		1	125.796501	0.1067	1.2480	0.264323	0.0319	0.0060910734
ss61528056	*rs41629197*	1	125.844524	0.0845	1.8580	0.173294	0.0451	0.0069816233
ss61519268	*rs41612521*	1	125.911737	0.9047	2.8410	0.092337	0.0518	0.0089244116
ss61466455	*rs41569108*	1	126.070215	0.9506	0.0730	0.787047	0.0114	0.0010720810
ss65180267	*rs43266963*	1	126.163013	0.6705	0.3836	0.535875	0.0120	0.0053212670
ss61520882	*rs41618401*	1	126.236415	0.3890	1.1719	0.279386	0.0205	0.0097238986
ss61512263	*rs41610871*	1	126.251225	0.5415	4.3510	0.037351	0.0392	0.0194827886
ss117966193		1	126.29574	0.2830	20.6449	0.000007	0.0893	0.0362448480
	rs29022289	1	126.365382	0.3782	3.6866	0.055261	0.0384	0.0180802752
ss64207118	*rs42529951*	1	126.392829	0.1404	7.6704	0.005763	0.0758	0.0182948204
ss64207444	*rs42530614*	1	126.422013	0.7736	0.1656	0.684197	0.0091	0.0032008924
ss61511947	*rs41609661*	1	126.460904	0.6862	13.1083	0.000315	0.0730	0.0314397844
	rs29020074	1	126.527141	0.6325	2.4944	0.114704	0.0313	0.0145673464
ss86293776		1	126.583646	0.2020	13.3919	0.000272	0.0854	0.0275407206
ss86338442		1	126.796504	0.3474	1.4994	0.221182	0.0239	0.0108447407
ss64136969	*rs42492559*	1	126.971873	0.4026	2.1453	0.143462	0.0276	0.0132524037
ss64137311	*rs42493530*	1	127.056007	0.5559	3.9575	0.047055	0.0372	0.0183807943
ss64137449	*rs42494297*	1	127.110233	0.1110	15.0302	0.000116	0.1083	0.0213849017
ss86319907		1	127.157376	0.4563	0.0908	0.763279	0.0057	0.0028110950
ss61522627	*rs41620798*	1	127.444859	0.6024	17.2244	0.000037	0.0768	0.0367719581
ss86304677		1	127.507518	0.2142	13.4288	0.000267	0.0796	0.0267881657
ss65185081	*rs43274686*	1	127.594335	0.7636	12.4487	0.000446	0.0745	0.0268808742
ss65184952	*rs43273660*	1	127.624398	0.5272	3.3680	0.066901	0.0341	0.0169967308
ss86327948		1	127.680108	0.3653	6.8356	0.009129	0.0510	0.0236395534
ss86336869		1	127.725447	0.5050	5.7356	0.016888	0.0446	0.0223193900
ss86321265		1	127.770063	0.5580	5.1205	0.023952	0.0424	0.0209242555
ss86330123		1	127.803933	0.3066	1.0513	0.305558	0.0207	0.0088068531
ss61543191	*rs41639306*	1	127.893558	0.3145	6.9671	0.008488	0.0515	0.0221901593
ss61543188	*rs41639303*	1	127.923587	0.6712	1.5568	0.212550	0.0256	0.0112974906

ss86309543		1	127.975919	0.7593	0.5645	0.452696	0.0163	0.0059687498
ss105304710		1	128.008878	0.4062	7.9787	0.004869	0.0535	0.0258292252
ss86304706		1	128.19137	0.9198	0.0620	0.803478	0.0086	0.0012636045
ss61543224	*rs41639330*	1	128.294158	0.1898	1.5054	0.220264	0.0277	0.0085210904
ss64951804	*rs43043730*	1	128.338198	0.1640	1.2410	0.265654	0.0274	0.0075144197
	rs29027485	1	128.421097	0.1848	1.3189	0.251185	0.0272	0.0082091975
ss65182066	*rs43271641*	1	128.449387	0.1082	0.4355	0.509505	0.0202	0.0038937099
ss64341161	*rs42590207*	1	128.490068	0.3037	3.2848	0.070353	0.0348	0.0147160792
ss61543774	*rs41637080*	1	128.51163	0.6963	3.2848	0.070353	0.0348	0.0147160792
ss86285692		1	128.564263	0.6082	3.9600	0.046986	0.0361	0.0172196295
ss61567958	*rs41666456*	1	128.593624	0.3918	3.9600	0.046986	0.0361	0.0172196295
ss65180359	*rs43261058*	1	128.625913	0.3037	2.7239	0.099307	0.0317	0.0134056803
ss61509122	*rs41601582*	1	128.65303	0.3926	2.5677	0.109522	0.0292	0.0139429836
ss65180396	*rs43261094*	1	128.680931	0.3926	2.5677	0.109522	0.0292	0.0139429836
ss65180422	*rs43261120*	1	128.725282	0.3926	2.5677	0.109522	0.0292	0.0139429836
ss65180441	*rs43262139*	1	128.749077	0.6963	2.7239	0.099307	0.0317	0.0134056803
ss105241728		1	128.817538	0.6956	2.7342	0.098672	0.0318	0.0134525907
ss61484575	*rs41580322*	1	128.88223	0.2958	2.7772	0.096063	0.0334	0.0139350343
ss86281997		1	129.147033	0.3195	4.1193	0.042777	0.0380	0.0165079587
ss86341171		1	129.180905	0.7937	3.2433	0.072149	0.0414	0.0135684817
ss105242415		1	129.242123	0.1905	5.5148	0.019136	0.0545	0.0168199899
ss61527961	*rs41627902*	1	129.270726	0.5702	0.8629	0.353246	0.0165	0.0080944989
ss86323097		1	129.527489	0.7006	1.3587	0.244164	0.0236	0.0099051327
ss65180790	*rs43265267*	1	129.578765	0.3173	4.3427	0.037533	0.0420	0.0181831512
ss65180820	*rs43265297*	1	129.603986	0.5444	3.7985	0.051701	0.0354	0.0175403937
ss86308471		1	129.62648	0.7994	0.4278	0.513294	0.0148	0.0047424625
ss86340988		1	129.653112	0.3639	0.0518	0.820062	0.0044	0.0020267098
ss86341223		1	129.677785	0.6877	0.8012	0.371030	0.0177	0.0075828643
ss86339933		1	129.728771	0.4599	0.3012	0.583277	0.0098	0.0048743026
ss86290858		1	129.791245	0.1461	0.5192	0.471421	0.0189	0.0047230782
ss65181146	*rs43268022*	1	129.940917	0.7679	0.1192	0.729982	0.0074	0.0026266858
ss61543210	*rs41639316*	1	129.964919	0.3926	0.8406	0.359540	0.0176	0.0084128403
ss86335677		1	130.00917	0.2299	0.1564	0.692609	0.0085	0.0030013016
ss86274593		1	130.052319	0.5000	3.8343	0.050612	0.0366	0.0183069671
ss86312645		1	130.073477	0.4828	2.8221	0.093421	0.0316	0.0157693804
ss61543215	*rs41639321*	1	130.097032	0.2514	3.5348	0.060510	0.0404	0.0151953318
ss86335232		1	130.117808	0.3295	1.5600	0.212081	0.0253	0.0111909982
ss65181357	*rs43265135*	1	130.147294	0.7178	1.0574	0.304170	0.0216	0.0087458743
ss86278214		1	130.172215	0.0989	0.1648	0.684897	0.0127	0.0022632751
ss65181458	*rs43266036*	1	130.216267	0.1748	12.7005	0.000391	0.0905	0.0261079741
ss86340956		1	130.258924	0.8625	21.7189	0.000004	0.1260	0.0298955465
ss86328736		1	130.311121	0.2221	14.9087	0.000123	0.0826	0.0285327900
ss86334077		1	130.335062	0.4699	5.1687	0.023302	0.0422	0.0210142817
ss65181866	*rs43269642*	1	130.356322	0.2013	0.4788	0.489218	0.0155	0.0049957770
ss65081617	*rs43166563*	1	130.378726	0.1791	0.3279	0.567106	0.0144	0.0042228115
ss86338673		1	130.41418	0.2808	8.6257	0.003424	0.0598	0.0241604070
ss61484363	*rs41578216*	1	130.456169	0.4613	4.7293	0.029989	0.0411	0.0204185217

ss65179382	*rs43260781*	1	130.534726	0.2514	0.3708	0.542790	0.0129	0.0048550618
ss86329424		1	130.568658	0.4441	7.1703	0.007587	0.0503	0.0248327421
ss61542714	*rs41636438*	1	130.593854	0.1117	19.5228	0.000012	0.1299	0.0257943178
ss86336369		1	130.615957	0.5029	3.8066	0.051453	0.0371	0.0185283539
ss65179757	*rs43263856*	1	130.651646	0.0874	21.2396	0.000005	0.1484	0.0236751257
ss86294471		1	130.730865	0.1533	2.3600	0.124936	0.0389	0.0100946158
ss86316338		1	130.847516	0.1698	2.9351	0.087117	0.0415	0.0116861889
ss86308452		1	130.868791	0.4936	12.9342	0.000345	0.0634	0.0317038457
ss86311047		1	130.903403	0.3474	0.6052	0.436880	0.0150	0.0068215445
	rs29022778	1	130.937338	0.6297	5.3275	0.021284	0.0429	0.0200236237
ss61564397	*rs41660568*	1	130.96563	0.9334	29.8563	0.000000	0.1932	0.0240274569
ss86317385		1	130.997444	0.8130	38.7062	0.000000	0.1453	0.0441864344
ss64762434	*rs42855951*	1	131.229708	0.5158	7.7630	0.005478	0.0489	0.0244096649
ss86289765		1	131.261697	0.1325	1.8745	0.171401	0.0372	0.0085514729
ss61505266	*rs41604024*	1	131.319208	0.5602	0.4742	0.491270	0.0126	0.0062025667
ss86339249		1	131.400396	0.1117	38.4981	0.000000	0.1776	0.0352608435
ss61523866	*rs41623623*	1	131.45667	0.1375	38.3455	0.000000	0.1610	0.0381994074
ss64680021	*rs42775876*	1	131.505562	0.8152	38.4621	0.000000	0.1409	0.0424629992
ss61495485	*rs41597955*	1	131.552032	0.8166	38.2183	0.000000	0.1407	0.0421363558
ss117966424		1	131.657377	0.8102	38.8796	0.000000	0.1398	0.0430022495
ss86324032		1	131.745799	0.1519	5.2474	0.022277	0.0599	0.0154427184
ss86304424		1	131.846797	0.4420	2.3837	0.123062	0.0304	0.0149905925
ss86304715		1	131.867214	0.5738	3.8574	0.049925	0.0364	0.0177816846
ss86337247		1	131.897226	0.6182	18.8675	0.000016	0.0816	0.0385302295
ss61543778	*rs41637083*	1	131.939497	0.4305	1.6047	0.205666	0.0236	0.0115841357
	rs29019405	1	131.991276	0.1254	41.0733	0.000000	0.1779	0.0390107673
ss86287276		1	132.074377	0.2034	26.5173	0.000000	0.1154	0.0373951335
ss86336373		1	132.110176	0.4191	16.7828	0.000047	0.0752	0.0365937777
ss65182608	*rs43268082*	1	132.160169	0.3890	1.2294	0.267899	0.0205	0.0097252306
ss86341561		1	132.204201	0.3818	1.6414	0.200564	0.0237	0.0111912610
ss61544710	*rs41637809*	1	132.301206	0.2006	27.9521	0.000000	0.1180	0.0378569723
ss105290965		1	132.341881	0.3438	13.1791	0.000304	0.0685	0.0309167321
ss86339523		1	132.379423	0.3761	13.9575	0.000202	0.0713	0.0334518720
ss65182862	*rs43271134*	1	132.41119	0.6970	2.7291	0.098983	0.0329	0.0138851263
ss61543809	*rs41637112*	1	132.497309	0.2285	26.6763	0.000000	0.1112	0.0392224948
ss65182930	*rs43271202*	1	132.559271	0.7120	4.5272	0.033711	0.0432	0.0177062275
ss86315075		1	132.622087	0.4298	8.3012	0.004084	0.0533	0.0261372834
ss65183329	*rs43275292*	1	132.688607	0.1791	28.7003	0.000000	0.1273	0.0374155004
ss65183374	*rs43268344*	1	132.709919	0.0946	0.3633	0.546901	0.0195	0.0033376516
ss86308916		1	132.760055	0.2787	26.5636	0.000000	0.1028	0.0413315489
ss86292166		1	132.845507	0.2722	25.3283	0.000001	0.0995	0.0394183909
ss86306961		1	132.886246	0.6239	14.3161	0.000168	0.0706	0.0331365429
ss61543820	*rs41637123*	1	132.991762	0.8209	0.5378	0.463593	0.0177	0.0051954547
ss86317098		1	133.029318	0.0638	3.2399	0.072299	0.0674	0.0080487824
ss65183615	*rs43270484*	1	133.05052	0.8317	4.2294	0.040101	0.0502	0.0140684745
ss86290467		1	133.096674	0.7844	7.2970	0.007075	0.0616	0.0208474194
ss86312264		1	133.166436	0.8037	0.0000	0.995403	0.0001	0.0000422315

ss61543830	*rs41638133*	1	133.259853	0.8847	19.5050	0.000012	0.1267	0.0258538268
ss86308833		1	133.332715	0.3030	12.6186	0.000408	0.0714	0.0301475120
ss61543831	*rs41638134*	1	133.390969	0.3195	21.3696	0.000005	0.0886	0.0385430753
ss86330784		1	133.424899	0.8689	35.3690	0.000000	0.1563	0.0356127396
ss86287434		1	133.499406	0.7149	0.6366	0.425221	0.0162	0.0066209759
ss61484881	*rs41582213*	1	133.521692	0.7135	0.1727	0.677814	0.0085	0.0034624245
ss105301409		1	133.558741	0.1676	29.3825	0.000000	0.1277	0.0356249264
ss61485375	*rs41581391*	1	133.649773	0.4262	15.2305	0.000104	0.0733	0.0358350663
ss86275255		1	133.6987	0.2744	24.0013	0.000001	0.0990	0.0394359818
ss61509223	*rs41602583*	1	133.720707	0.3181	0.8269	0.363479	0.0178	0.0077045232
ss86309409		1	133.765688	0.7672	0.5153	0.473087	0.0165	0.0058764113
ss117966404		1	133.882163	0.7915	0.3707	0.542830	0.0139	0.0045710224
ss86339053		1	133.938434	0.7271	22.6828	0.000002	0.0965	0.0383057981
ss105236743		1	133.980973	0.2450	0.9999	0.317684	0.0220	0.0081564196
ss65183771	*rs43272238*	1	134.025806	0.1884	0.0387	0.844123	0.0048	0.0014728468
ss86291187		1	134.088757	0.4155	7.0839	0.007958	0.0488	0.0236818589
ss65183829	*rs43272296*	1	134.111438	0.3095	0.0363	0.848936	0.0039	0.0016496227
ss86295521		1	134.139302	0.5838	8.8683	0.003002	0.0547	0.0265731154
ss86335334		1	134.258377	0.4291	0.0956	0.757326	0.0058	0.0028504670
ss86298512		1	134.280051	0.4198	0.0028	0.957718	0.0010	0.0004813040
ss65185443	*rs43268844*	1	134.316657	0.3911	2.1122	0.146584	0.0282	0.0134516185
ss61506548	*rs41607057*	1	134.419503	0.3711	0.1253	0.723465	0.0069	0.0032143473
ss65185517	*rs43268918*	1	134.484755	0.0559	2.7845	0.095628	0.0687	0.0072455455
ss63973210	*rs42409034*	1	134.659982	0.6777	7.8791	0.005141	0.0561	0.0245184625
ss61476222	*rs41575285*	1	134.757616	0.3711	0.3720	0.542134	0.0114	0.0053395682
ss63828660	*rs42370682*	1	134.828444	0.0831	0.8283	0.363074	0.0318	0.0048419112
ss63828720	*rs42370712*	1	134.869152	0.0795	0.4022	0.526148	0.0225	0.0033000907
ss63828824	*rs42371764*	1	134.900836	0.9169	0.8283	0.363074	0.0318	0.0048419112
ss64979769	*rs43067163*	1	134.977005	0.9140	2.3341	0.127026	0.0526	0.0082609615
ss65185752	*rs43271751*	1	135.008984	0.2980	0.0040	0.949584	0.0013	0.0005271298
ss105236759		1	135.081107	0.5451	0.0088	0.925237	0.0017	0.0008353891
ss86337212		1	135.119864	0.4520	0.0132	0.908713	0.0021	0.0010208694
ss86338483		1	135.140555	0.1798	0.8482	0.357380	0.0229	0.0067624178
ss117966128		1	135.17608	0.6289	0.1409	0.707510	0.0071	0.0032906207
ss61544878	*rs41639348*	1	135.2195	0.6067	0.2676	0.605142	0.0096	0.0045849173
ss38333934	*rs29017627*	1	135.258763	0.2557	0.6993	0.403311	0.0176	0.0066929677
ss61544879	*rs41639349*	1	135.45775	0.3517	0.1789	0.672444	0.0080	0.0036316408
ss61505836	*rs41599969*	1	135.532554	0.3496	3.9470	0.047346	0.0387	0.0176038528
ss64659102	*rs42759854*	1	135.622826	0.3446	0.5075	0.476462	0.0134	0.0060559864
ss86331750		1	135.655864	0.4054	0.4052	0.524636	0.0119	0.0057201931
ss61529952	*rs41630453*	1	135.771215	0.8696	0.1359	0.712542	0.0102	0.0023052674
ss64806845	*rs42898322*	1	135.864178	0.6468	8.4165	0.003836	0.0557	0.0254268671
ss86337861		1	135.915394	0.6526	9.1276	0.002610	0.0580	0.0263178390
ss86300764		1	135.988521	0.5573	0.0307	0.860884	0.0032	0.0015804362
ss86295027		1	136.027886	0.8044	4.6627	0.031166	0.0493	0.0154977498
ss65186252	*rs43277348*	1	136.080818	0.3847	0.0872	0.767896	0.0057	0.0026876252
ss86284925		1	136.11176	0.0308	2.2471	0.134317	0.0806	0.0048119587

ss105237451		1	136.137382	0.0745	2.1562	0.142451	0.0524	0.0072285871
ss86304579		1	136.236469	0.1383	0.1697	0.680508	0.0110	0.0026323462
ss86285232		1	136.258142	0.4699	0.2153	0.642785	0.0090	0.0044812500
ss117966421		1	136.344019	0.1060	2.4741	0.116188	0.0484	0.0091759075
ss86275125		1	136.401655	0.1024	0.1885	0.664322	0.0134	0.0024646549
ss86318256		1	136.440804	0.8847	0.0024	0.960747	0.0014	0.0002849271
ss86338186		1	136.4992	0.3453	0.0202	0.886998	0.0026	0.0011943429
ss86309292		1	136.573912	0.7744	13.4860	0.000259	0.0805	0.0281360578
ss86285989		1	136.695554	0.5745	0.7581	0.384221	0.0162	0.0079418904
ss86304578		1	136.72116	0.7787	0.7567	0.384658	0.0189	0.0064997328
ss86310616		1	136.742668	0.7364	3.6978	0.054894	0.0392	0.0152314582
ss86332038		1	136.781568	0.2085	1.6847	0.194728	0.0286	0.0094460504
ss86295275		1	136.831204	0.4742	1.9632	0.161621	0.0256	0.0127832929
ss64107799	rs42482108	1	136.852565	0.3395	3.4443	0.063894	0.0370	0.0166164991
ss86303138		1	137.000996	0.4556	0.9376	0.333230	0.0186	0.0092130729
ss86338215		1	137.063131	0.8202	0.0018	0.966363	0.0010	0.0002871828
ss61545434	rs41643379	1	137.113366	0.7744	1.6784	0.195573	0.0281	0.0098330492
ss65188075	rs43272864	1	137.199869	0.2493	0.0729	0.787255	0.0056	0.0021054084
ss86276329		1	137.248123	0.5337	0.6683	0.413915	0.0151	0.0075193775
ss86336214		1	137.28271	0.5602	13.2168	0.000298	0.0677	0.0333610058
ss86289615		1	137.331935	0.7536	0.5367	0.464037	0.0159	0.0058921730
ss86336813		1	137.367956	0.9334	1.9393	0.164189	0.0520	0.0064668581
ss86327491		1	137.393923	0.8130	0.1073	0.743313	0.0078	0.0023760855
ss86317610		1	137.440693	0.3460	8.9592	0.002859	0.0567	0.0256814559
ss105237458		1	137.464141	0.5788	5.0142	0.025456	0.0415	0.0202375302
ss61544894	rs41639364	1	137.499967	0.4226	4.8325	0.028257	0.0408	0.0199206976
ss61485380	rs41581396	1	137.531828	0.6003	5.4269	0.020114	0.0443	0.0212761349
ss65188020	rs43272109	1	137.595626	0.0494	0.4106	0.521853	0.0271	0.0025469697
ss61485379	rs41581395	1	137.680052	0.8976	0.3481	0.555371	0.0182	0.0033508993
ss86329359		1	137.749528	0.6168	4.6498	0.031399	0.0396	0.0187361245
ss86325178		1	137.904261	0.6963	4.7428	0.029755	0.0425	0.0179693894
ss86286168		1	137.924385	0.4183	6.3567	0.011916	0.0467	0.0227179369
ss86296409		1	137.980069	0.4771	5.4730	0.019594	0.0422	0.0210740602
ss86323681		1	138.007562	0.0559	1.0256	0.311543	0.0407	0.0042898861
ss86338482		1	138.070691	0.2794	26.3042	0.000000	0.1043	0.0419769539
ss86321239		1	138.119456	0.8352	0.2274	0.633574	0.0118	0.0032474466
ss86308259		1	138.165925	0.1748	0.1093	0.741053	0.0080	0.0022975989
ss86298877		1	138.214555	0.3238	2.3937	0.122281	0.0304	0.0133298905
ss65163922	rs43253484	1	138.254741	0.3188	2.4316	0.119363	0.0307	0.0133260152
ss86290999		1	138.357697	0.1003	0.0107	0.917810	0.0031	0.0005631952
ss61537926	rs41635716	1	138.392971	0.9269	2.5257	0.112460	0.0566	0.0076667138
ss61522954	rs41622823	1	138.419898	0.3037	4.4245	0.035785	0.0437	0.0184777642
ss65163777	rs43252339	1	138.457514	0.3066	9.6161	0.002007	0.0607	0.0258229603
ss86298317		1	138.48023	0.8782	0.5814	0.446022	0.0213	0.0045498354
	rs29021130	1	138.52683	0.8030	2.1158	0.146238	0.0340	0.0107493563
ss86336105		1	138.561549	0.1297	2.7863	0.095526	0.0463	0.0104444258
ss105240989		1	138.602576	0.2865	0.3200	0.571777	0.0117	0.0047902713

ss105240990		1	138.635746	0.4026	0.4553	0.500046	0.0127	0.0060871869
ss86338048		1	138.66165	0.6132	0.8375	0.360416	0.0171	0.0081143899
ss61522953	*rs41622822*	1	138.702884	0.8295	0.4028	0.525852	0.0154	0.0043499923
ss61562029	*rs41657135*	1	138.782443	0.3102	0.1782	0.673019	0.0081	0.0034783167
ss86293375		1	138.836143	0.5430	2.2682	0.132507	0.0278	0.0137844291
ss65530165	*rs43611067*	1	138.938899	0.4907	5.0410	0.025067	0.0413	0.0206583023
	rs29024603	1	139.258263	0.7414	12.9254	0.000347	0.0725	0.0278039639
ss117966374		1	139.293827	0.1583	4.6081	0.032167	0.0533	0.0142061081
ss86289725		1	139.357739	0.3789	4.0007	0.045870	0.0372	0.0175007131
ss105285844		1	139.476366	0.6683	3.9867	0.046250	0.0389	0.0172560696
ss86308122		1	139.510073	0.2199	0.3994	0.527620	0.0141	0.0048322214
ss86323239		1	139.548621	0.1368	14.1972	0.000179	0.0989	0.0233609497
ss86306737		1	139.640353	0.6583	20.2941	0.000008	0.0864	0.0388663020
ss64790436	*rs42883237*	1	139.707352	0.2686	19.3563	0.000013	0.0893	0.0351001290
ss64795003	*rs42889208*	1	139.807114	0.7973	2.0966	0.148074	0.0333	0.0107590378
ss64008728	*rs42428138*	1	139.845868	0.8302	1.4523	0.228564	0.0296	0.0083441627
ss61524374	*rs41619822*	1	139.935236	0.2027	1.4777	0.224543	0.0277	0.0089500329
ss86332461		1	139.982306	0.2915	5.1136	0.024046	0.0466	0.0192437415
ss64008994	*rs42428771*	1	140.036553	0.8173	2.0983	0.147912	0.0352	0.0104960644
ss64009134	*rs42429341*	1	140.060872	0.7787	5.4212	0.020179	0.0494	0.0170243422
ss86318277		1	140.082293	0.7672	3.0851	0.079453	0.0386	0.0137896176
ss61527341	*rs41622705*	1	140.109734	0.0974	19.3024	0.000013	0.1321	0.0232297736
ss61527348	*rs41622712*	1	140.169251	0.1554	0.2740	0.600840	0.0138	0.0036188072
ss61562427	*rs41660526*	1	140.191583	0.2077	1.6893	0.194120	0.0303	0.0099599293
ss65250653	*rs43338878*	1	140.220153	0.1411	1.5961	0.206881	0.0340	0.0082503726
ss61489053	*rs41589492*	1	140.254183	0.8553	0.2840	0.594277	0.0142	0.0035137026
ss86337557		1	140.401581	0.1447	2.3537	0.125442	0.0410	0.0101604987
ss61512210	*rs41610520*	1	140.495455	0.1268	2.0721	0.150465	0.0407	0.0090159050
ss86340491		1	140.530805	0.1289	0.6548	0.418665	0.0228	0.0051218065
ss86312306		1	140.706547	0.2443	0.0345	0.852666	0.0040	0.0014786949
ss86305401		1	140.82255	0.2128	14.3933	0.000161	0.0828	0.0277402020
ss86306650		1	140.847627	0.3861	1.0316	0.310142	0.0192	0.0090900917
ss117963486		1	140.876202	0.1132	0.4391	0.507753	0.0200	0.0040097043
ss65195114	*rs43282740*	1	141.061623	0.5337	0.0273	0.868842	0.0031	0.0015214172
ss65195052	*rs43281678*	1	141.086853	0.1218	0.3397	0.560177	0.0166	0.0035448993
ss65194931	*rs43280557*	1	141.186328	0.4026	0.0157	0.900397	0.0024	0.0011480524
ss61547063	*rs41643696*	1	141.260739	0.3195	9.6196	0.002003	0.0604	0.0262735620
ss86304472		1	141.310419	0.6984	9.7680	0.001850	0.0615	0.0259098462
ss86326983		1	141.355474	0.0752	0.5471	0.459767	0.0253	0.0035178550
ss61485639	*rs41584543*	1	141.382338	0.9585	0.4594	0.498128	0.0313	0.0024919405
ss86316558		1	141.468989	0.1798	0.1799	0.671610	0.0099	0.0029176438
ss86335237		1	141.5104	0.7830	0.2491	0.617878	0.0108	0.0036743601
ss61545442	*rs41643387*	1	141.618694	0.4255	0.0778	0.780345	0.0053	0.0026149472
ss86282286		1	141.653948	0.8596	0.1264	0.722320	0.0097	0.0023488798
ss61516574	*rs41616977*	1	141.703833	0.4212	0.0246	0.875458	0.0030	0.0014623658
ss86283471		1	141.740045	0.5802	0.1564	0.692630	0.0077	0.0037376348
ss86340796		1	141.989336	0.3116	7.8980	0.005088	0.0575	0.0246538065

ss61545450	*rs41643395*	1	142.223661	0.6289	17.4652	0.000033	0.0786	0.0366871749
ss65191466	*rs43275844*	1	142.238759	0.2350	0.6755	0.411423	0.0183	0.0065757825
ss86336776		1	142.283845	0.1834	0.4742	0.491283	0.0165	0.0049451239
ss61519225	*rs41612478*	1	142.441349	0.6246	0.1032	0.748090	0.0060	0.0028133480
ss117963504		1	142.495434	0.2744	0.0043	0.947511	0.0014	0.0005375679
ss86321727		1	142.620597	0.3431	11.1975	0.000863	0.0633	0.0285131264
ss86298401		1	142.642593	0.5910	6.7049	0.009816	0.0485	0.0234543602
ss63850028	*rs42384304*	1	142.666938	0.6426	13.3099	0.000284	0.0720	0.0330964255
ss86289175		1	142.695856	0.8718	0.1482	0.700350	0.0109	0.0024462519
ss86284635		1	142.735792	0.6039	0.0716	0.789163	0.0051	0.0024374594
ss65191259	*rs43279634*	1	142.858811	0.4742	6.7430	0.009610	0.0480	0.0239283369
ss117963483		1	142.882662	0.8102	0.6375	0.424890	0.0192	0.0059142134
ss64967581	*rs43057535*	1	142.970402	0.1160	0.8378	0.360354	0.0262	0.0053807124
ss64971179	*rs43066203*	1	142.99662	0.1046	1.1883	0.276055	0.0327	0.0061250997
ss64860889	*rs42956310*	1	143.02666	0.3295	0.2581	0.611619	0.0099	0.0043926679
ss86335015		1	143.049918	0.3073	4.7909	0.028943	0.0430	0.0183224941
ss86337656		1	143.287161	0.7715	0.3057	0.580480	0.0120	0.0042364842
ss86312073		1	143.370558	0.2650	5.0235	0.025319	0.0464	0.0180795341
ss86313458		1	143.390796	0.5903	8.8939	0.002961	0.0537	0.0259576582
ss86281472		1	143.579977	0.3138	2.4462	0.118264	0.0309	0.0133016858
ss61566809	*rs41667429*	1	143.611781	0.1153	17.2680	0.000037	0.1154	0.0235526536
ss86292529		1	143.642806	0.5473	2.0507	0.152584	0.0268	0.0132973233
ss64728461	*rs42823060*	1	143.688546	0.1304	17.4408	0.000033	0.1122	0.0254424365
ss86312673		1	143.748148	0.2521	22.2397	0.000003	0.1005	0.0379030696
ss86298544		1	143.775482	0.3030	5.1196	0.023964	0.0465	0.0196304725
ss86341652		1	143.801372	0.5050	3.8596	0.049860	0.0367	0.0183560033
ss86327190		1	143.846112	0.1189	0.6384	0.424572	0.0228	0.0047677227
ss65190807	*rs43275396*	1	143.894063	0.6519	6.5628	0.010623	0.0488	0.0221566362
ss65190854	*rs43275943*	1	143.915489	0.5115	2.8035	0.094506	0.0304	0.0151874432
ss86341036		1	144.105011	0.3725	3.6596	0.056158	0.0362	0.0169090106
ss61545980	*rs41639230*	1	144.161248	0.6375	4.4972	0.034303	0.0404	0.0186668581
ss86340662		1	144.230611	0.6569	14.2289	0.000176	0.0718	0.0323796776
ss86339012		1	144.271781	0.7457	6.2098	0.012936	0.0519	0.0196918752
ss86289764		1	144.378178	0.6855	15.9384	0.000072	0.0779	0.0336076624
ss86340327		1	144.405313	0.9040	1.1042	0.293717	0.0327	0.0056705705
ss86335695		1	144.433593	0.5301	13.9917	0.000199	0.0691	0.0344040155
ss86340025		1	144.479372	0.5229	6.1916	0.013068	0.0453	0.0226207161
ss61546530	*rs41643465*	1	144.516797	0.8374	21.5873	0.000004	0.1140	0.0310362811
ss86339312		1	144.559051	0.2371	0.8605	0.353907	0.0204	0.0073959245
ss117963481		1	144.626795	0.4319	5.1902	0.023016	0.0419	0.0205569138
ss86315657		1	144.647921	0.3188	3.8054	0.051490	0.0397	0.0172439356
ss86335356		1	144.752234	0.1404	0.0390	0.843426	0.0054	0.0013006498
ss86323132		1	144.80215	0.8481	12.7026	0.000390	0.0900	0.0231821413
ss86320337		1	144.952299	0.7550	1.1263	0.288934	0.0231	0.0085580181
ss61545957	*rs41639207*	1	145.006899	0.2393	0.1115	0.738502	0.0076	0.0027587584
ss105241710		1	145.047458	0.4943	2.1992	0.138538	0.0273	0.0136610616
ss86328895		1	145.077997	0.2027	0.0298	0.862926	0.0040	0.0012848254

ss86321686		1	145.147732	0.3238	0.1768	0.674227	0.0084	0.0036819843
ss86340179		1	145.192864	0.3976	0.5212	0.470587	0.0141	0.0067518622
ss86337311		1	145.233055	0.1282	22.1840	0.000003	0.1250	0.0279366963
ss86283522		1	145.259158	0.6368	5.1635	0.023371	0.0448	0.0207028037
ss86334645		1	145.279395	0.5014	0.4573	0.499119	0.0128	0.0064127650
ss86340404		1	145.306409	0.5308	5.9478	0.014984	0.0446	0.0222314517
ss65191955	*rs43278831*	1	145.349801	0.5595	4.3976	0.036350	0.0382	0.0188255552
ss65192010	*rs43278886*	1	145.372761	0.8539	0.0698	0.791763	0.0070	0.0017385103
ss86336507		1	145.436566	0.1920	1.8260	0.177044	0.0322	0.0099786525
ss86320199		1	145.501111	0.6777	1.6377	0.201065	0.0254	0.0110922743
ss86310244		1	145.522082	0.7958	4.7685	0.029319	0.0512	0.0166292043
ss65192396	*rs43281569*	1	145.577675	0.1117	0.0001	0.994022	0.0002	0.0000440359
ss86339121		1	145.675616	0.2163	23.6948	0.000001	0.1067	0.0361863735
ss86315307		1	145.710028	0.1547	0.0208	0.885391	0.0037	0.0009777926
ss86302054		1	145.745292	0.1003	3.6881	0.055212	0.0574	0.0103636342
ss86321704		1	145.772707	0.2787	0.6106	0.434822	0.0160	0.0064334398
ss86298922		1	145.800119	0.0394	3.3849	0.066221	0.0867	0.0065587293
ss86328364		1	145.824635	0.2758	0.5007	0.479436	0.0145	0.0057852322
ss63163129	*rs42140284*	1	145.886528	0.8804	1.0297	0.310579	0.0296	0.0062407363
ss86341690		1	145.913676	0.2092	2.5686	0.109461	0.0363	0.0120206167
ss61485900	*rs41586699*	1	145.95415	0.8130	1.5157	0.218693	0.0297	0.0090401629
ss86335606		1	146.022343	0.6454	0.5384	0.463334	0.0146	0.0066942980
ss86331118		1	146.049638	0.3517	0.6323	0.426795	0.0158	0.0072081898
ss86313080		1	146.075064	0.7113	2.6487	0.104085	0.0346	0.0142238784
ss86320916		1	146.14279	0.9434	0.7980	0.371986	0.0367	0.0039133873
ss86291561		1	146.164292	0.6870	0.5302	0.466765	0.0148	0.0063613261
ss86301146		1	146.249469	0.7436	0.1024	0.749024	0.0073	0.0027960900
ss117966434		1	146.320537	0.1927	0.0049	0.944469	0.0016	0.0005113109
ss86314515		1	146.340622	0.0552	0.1329	0.715572	0.0149	0.0015544270
ss86326641		1	146.377857	0.8854	2.6710	0.102645	0.0476	0.0096528135
ss86284723		1	146.387649	0.2221	0.0774	0.780991	0.0063	0.0021716818
ss86287278		1	146.433082	0.4348	1.3985	0.237374	0.0230	0.0113189537
ss86338734		1	146.508751	0.8302	0.0445	0.832955	0.0054	0.0015172206
ss105241700		1	146.60884	0.4670	3.3918	0.065948	0.0344	0.0171470197
ss86298938		1	146.630818	0.4319	0.0276	0.868041	0.0031	0.0015434422
ss86317547		1	146.6661	0.3252	0.1135	0.736292	0.0069	0.0030240462
ss86337203		1	146.756999	0.5050	0.0097	0.921650	0.0018	0.0009223680
ss46527085	*rs41255628*	1	146.829619	0.6504	1.4835	0.223643	0.0254	0.0115643625
ss86321087		1	146.933478	0.1375	4.2468	0.039694	0.0554	0.0131355980
ss86339805		1	146.955288	0.1633	0.7295	0.393331	0.0217	0.0059289141
ss64678575	*rs42771735*	1	146.980055	0.0946	0.0567	0.811814	0.0076	0.0012989542
ss86306455		1	147.021	0.7607	1.0242	0.311875	0.0224	0.0081499297
ss86333308		1	147.07525	0.2658	0.0116	0.914353	0.0024	0.0009185530
ss86285231		1	147.096213	0.3324	0.2126	0.644879	0.0094	0.0041536893
ss86292871		1	147.12746	0.1010	0.1344	0.713984	0.0109	0.0019741607
ss86314680		1	147.166578	0.7486	12.4581	0.000444	0.0772	0.0290649260
ss86304846		1	147.197898	0.2944	3.0372	0.081815	0.0352	0.0146160751

ss86336787		1	147.224999	0.5544	0.8913	0.345465	0.0183	0.0090181209
ss86323771		1	147.29152	0.0845	0.0737	0.786090	0.0092	0.0014299664
ss86334102		1	147.319319	0.1483	15.1652	0.000108	0.1005	0.0253924384
ss86322201		1	147.355781	0.6153	9.6276	0.001994	0.0591	0.0279752225
ss86337848		1	147.382789	0.6712	9.3755	0.002284	0.0585	0.0258114531
ss86336317		1	147.411133	0.8725	3.3258	0.068629	0.0514	0.0114464642
ss86318026		1	147.454173	0.7235	0.7349	0.391582	0.0178	0.0071368975
ss86306327		1	147.524969	0.4370	2.7894	0.095338	0.0311	0.0152831893
ss86330331		1	147.551139	0.4642	1.0342	0.309519	0.0190	0.0094678412
ss46526105	*rs41255249*	1	147.670375	0.7443	14.9204	0.000123	0.0807	0.0307271602
ss46526129	*rs41255272*	1	147.707816	0.7135	2.2227	0.136444	0.0308	0.0126032664
ss86274152		1	147.773269	0.2271	2.8634	0.091064	0.0399	0.0139929047
ss86284116		1	147.795528	0.6332	0.0383	0.844892	0.0038	0.0017684180
ss61546552	*rs41643486*	1	147.821461	0.7063	0.1155	0.734125	0.0070	0.0029079279
ss61546554	*rs41643488*	1	147.842221	0.5623	0.4347	0.509917	0.0125	0.0061330648
ss86302068		1	147.868234	0.5580	0.4925	0.483042	0.0133	0.0065653409
ss86330476		1	147.896561	0.3510	11.1366	0.000891	0.0660	0.0300580973
ss86316168		1	147.94441	0.4864	20.8317	0.000006	0.0842	0.0420540810
ss61506262	*rs41604478*	1	147.984681	0.1211	0.0123	0.911550	0.0031	0.0006685754
ss86340665		1	148.005087	0.0645	1.1454	0.284881	0.0396	0.0047745637
ss86337886		1	148.032391	0.8095	0.0004	0.984415	0.0005	0.0001416478
ss61526964	*rs41628433*	1	148.224422	0.4456	2.5391	0.111511	0.0297	0.0146763704
ss86309699		1	148.288799	0.9405	0.6283	0.428258	0.0310	0.0034621985
ss61476732	*rs41578283*	1	148.322768	0.3489	1.1730	0.279167	0.0211	0.0095742382
ss61476730	*rs41578281*	1	148.343172	0.8768	0.4277	0.513316	0.0183	0.0039492677
ss64000072	*rs42429246*	1	148.402823	0.8195	5.9056	0.015345	0.0568	0.0168007253
ss61528661	*rs41627294*	1	148.462983	0.6304	4.7948	0.028877	0.0432	0.0201469411
ss86301811		1	148.484066	0.2973	3.2045	0.073873	0.0362	0.0151142405
ss86333524		1	148.50778	0.4728	4.4668	0.034915	0.0397	0.0198086105
ss86299927		1	148.531945	0.3245	5.5918	0.018318	0.0454	0.0199246609
ss86295782		1	148.57001	0.1096	0.2073	0.648998	0.0137	0.0026676602
ss86333239		1	148.6771	0.1003	0.5362	0.464255	0.0232	0.0041858238
ss86340087		1	148.698038	0.2228	3.7747	0.052436	0.0429	0.0148595362
ss86299742		1	148.741456	0.8431	7.6565	0.005807	0.0706	0.0186754279
ss61522035	*rs41623028*	1	148.765346	0.2686	2.8259	0.093203	0.0349	0.0137284028
ss86283217		1	148.770164	0.8181	3.1457	0.076563	0.0417	0.0124223470
ss86313410		1	148.854021	0.5953	0.4227	0.515813	0.0126	0.0060942486
ss86337531		1	148.8864	0.8668	3.2102	0.073614	0.0483	0.0111554902
ss86324926		1	148.911816	0.1691	0.1878	0.664924	0.0110	0.0030875159
ss86335539		1	148.951348	0.7085	2.3869	0.122810	0.0314	0.0129620448
ss61539937	*rs41638892*	1	148.97641	0.6812	3.1158	0.077974	0.0354	0.0153691120
ss65167549	*rs43253207*	1	149.000569	0.1769	0.4726	0.492037	0.0168	0.0048847458
ss86341024		1	149.153072	0.2966	0.7987	0.371779	0.0184	0.0076747814
ss86319126		1	149.18984	0.9076	11.5088	0.000732	0.1037	0.0174021749
ss64778695	*rs42875030*	1	149.211909	0.2314	14.2648	0.000172	0.0820	0.0291613010
ss86274045		1	149.295821	0.6354	1.6560	0.198568	0.0253	0.0117026239
ss117963563		1	149.326578	0.6834	2.2256	0.136191	0.0295	0.0127535294

ss86338743		1	149.363846	0.8610	7.2538	0.007245	0.0682	0.0163310494
ss86316795		1	149.402856	0.4047	0.0007	0.979261	0.0005	0.0002352147
ss86322824		1	149.650627	0.3374	8.5589	0.003550	0.0562	0.0251128871
ss86290638		1	149.697044	0.2514	0.0674	0.795255	0.0055	0.0020570251
ss86319617		1	149.767231	0.2830	0.4880	0.485049	0.0141	0.0057300898
ss86302895		1	149.809093	0.2514	0.2428	0.622313	0.0108	0.0040498018
ss61468330	*rs41567450*	1	149.865183	0.2830	1.3266	0.249807	0.0241	0.0097990679
ss86284407		1	149.983557	0.8052	4.8149	0.028545	0.0508	0.0159404122
ss86328743		1	150.055732	0.7400	2.9123	0.088355	0.0356	0.0137165374
ss86290106		1	150.0939	0.5408	0.5110	0.474960	0.0135	0.0067046559
ss86297683		1	150.115092	0.7221	0.0279	0.867440	0.0034	0.0013784855
ss86303117		1	150.140587	0.1304	2.6317	0.105206	0.0441	0.0099905529
ss86331077		1	150.188943	0.4742	0.3355	0.562620	0.0108	0.0053909966
ss86273472		1	150.241288	0.0967	2.2850	0.131085	0.0472	0.0082509471
ss86328276		1	150.264958	0.5960	2.0316	0.154512	0.0269	0.0129566098
ss86333522		1	150.338087	0.4348	0.4190	0.517664	0.0122	0.0060076422
ss61515203	*rs41612861*	1	150.362015	0.6368	2.1777	0.140479	0.0286	0.0132326514
ss64991024	*rs43086514*	1	150.396166	0.5630	2.6509	0.103942	0.0309	0.0152049283
ss86291704		1	150.417816	0.5874	1.4071	0.235949	0.0227	0.0110126666
ss65194051	*rs43279180*	1	150.546404	0.6755	0.4871	0.485474	0.0136	0.0059598467
ss65194030	*rs43279159*	1	150.615823	0.0831	1.0899	0.296857	0.0351	0.0053466862
ss65193941	*rs43278571*	1	150.686877	0.1297	0.4585	0.498564	0.0189	0.0042707016
ss65193873	*rs43277803*	1	150.72879	0.9047	3.7397	0.053539	0.0612	0.0105454349
ss105287609		1	150.807422	0.5358	4.0686	0.044070	0.0372	0.0184908364
ss86335566		1	150.892465	0.4191	0.2924	0.588836	0.0106	0.0051636846
ss86336476		1	151.286555	0.5974	1.9961	0.158149	0.0267	0.0128323829
ss117966151		1	151.456809	0.4484	3.4143	0.065056	0.0347	0.0171552769
ss61547048	*rs41643681*	1	151.495784	0.5444	3.3410	0.068001	0.0348	0.0172679647
ss86290810		1	151.530117	0.0702	1.3982	0.237425	0.0433	0.0056482319
ss86309411		1	151.562634	0.5960	0.3904	0.532273	0.0120	0.0057997804
ss86314568		1	151.698382	0.1282	2.4893	0.115081	0.0428	0.0095758797
ss86299501		1	151.725393	0.8761	0.0245	0.875780	0.0045	0.0009687737
ss86339887		1	151.74924	0.0817	0.3076	0.579310	0.0193	0.0028993012
ss86335296		1	151.773368	0.1970	0.0133	0.908220	0.0026	0.0008249694
ss86329022		1	151.811202	0.1082	0.0221	0.881811	0.0045	0.0008729419
	rs29027916	1	151.921264	0.7787	0.9497	0.330140	0.0224	0.0077318985
ss86304275		1	151.95522	0.1225	0.0002	0.988201	0.0004	0.0000917136
ss86290719		1	152.035402	0.6984	2.6921	0.101300	0.0344	0.0145108266
ss86302264		1	152.066435	0.7994	2.5907	0.107949	0.0386	0.0123855108
ss86322909		1	152.125151	0.1834	2.2631	0.132944	0.0376	0.0112483477
ss86275911		1	152.153086	0.7973	2.4706	0.116448	0.0377	0.0121761364
ss86340405		1	152.228421	0.9341	2.6016	0.107211	0.0597	0.0073454549
ss86340497		1	152.572439	0.3145	0.1938	0.659886	0.0088	0.0037950129
ss86301285		1	152.795757	0.1762	0.6856	0.407948	0.0202	0.0058588164
ss86287700		1	152.817113	0.1734	0.5107	0.475081	0.0175	0.0050063772
ss86341165		1	152.850441	0.8854	0.9345	0.334035	0.0280	0.0056835661
ss117966427		1	153.015008	0.1375	0.6008	0.438539	0.0214	0.0050843971

ss86326533		1	153.17268	0.1991	3.0678	0.080296	0.0409	0.0130476581
ss61520600	*rs41615623*	1	153.203876	0.8646	2.0591	0.151747	0.0400	0.0093629106
ss86323226		1	153.2365	0.6805	1.9723	0.160646	0.0281	0.0122101350
ss86306510		1	153.276152	0.4728	1.1140	0.291579	0.0200	0.0099729980
ss63880716	*rs41706473*	1	153.301035	0.8453	0.0467	0.828910	0.0055	0.0014477156
ss86336917		1	153.350947	0.3897	0.0630	0.801843	0.0049	0.0023253099
ss86339199		1	153.378594	0.5136	0.0004	0.984379	0.0004	0.0001801821
ss61520596	*rs41615619*	1	153.421176	0.2414	1.6130	0.204499	0.0282	0.0103407715
ss61474232	*rs41568244*	1	153.454845	0.1168	0.1388	0.709617	0.0109	0.0022454259
ss86338552		1	153.489283	0.2937	0.1765	0.674493	0.0090	0.0037236009
	rs29017440	1	153.517161	0.3223	0.0801	0.777273	0.0056	0.0024280333
ss61520601	*rs41615624*	1	153.540792	0.1361	0.4209	0.516696	0.0178	0.0041757873
ss61522810	*rs41622079*	1	153.588453	0.1225	0.4717	0.492427	0.0198	0.0042533860
	rs29024220	1	153.608601	0.7851	0.2343	0.628540	0.0110	0.0037043293
ss61525103	*rs41625329*	1	153.670496	0.7872	0.9053	0.341705	0.0214	0.0071798597
ss117966440		1	153.870569	0.4162	1.4760	0.224806	0.0237	0.0115113851
ss65195167	*rs43282793*	1	153.905884	0.6139	0.0803	0.776914	0.0055	0.0026273647
ss61547075	*rs41643708*	1	153.966569	0.4090	0.1959	0.658166	0.0085	0.0041039345
ss117962564		1	154.026607	0.4527	0.0646	0.799473	0.0049	0.0024041819
	rs29026782	1	154.053554	0.5308	0.3961	0.529303	0.0121	0.0060145513
ss61547597	*rs41646716*	1	154.090029	0.1970	0.9328	0.334462	0.0227	0.0071821891
ss61522968	*rs41623436*	1	154.125189	0.2385	0.3214	0.570928	0.0125	0.0045249010
ss65195926	*rs43282853*	1	154.146603	0.2830	3.9127	0.048317	0.0405	0.0164454975
ss86338760		1	154.222249	0.6375	0.9753	0.323702	0.0193	0.0089423206
ss61529443	*rs41625164*	1	154.312109	0.3281	0.0255	0.873211	0.0031	0.0013690281
ss86296581		1	154.351347	0.1246	3.9342	0.047706	0.0551	0.0120190417
ss86325062		1	154.394122	0.1160	2.8869	0.089751	0.0486	0.0099651912
ss61547601	*rs41646720*	1	154.425338	0.1769	5.9085	0.015320	0.0584	0.0170193624
ss65196108	*rs43285134*	1	154.46111	0.3961	0.1355	0.712858	0.0072	0.0034532804
ss61547602	*rs41646721*	1	154.492953	0.8467	1.5891	0.207879	0.0324	0.0084171513
ss86289999		1	154.551516	0.4441	7.3883	0.006728	0.0516	0.0254814363
ss86321169		1	154.573212	0.3968	0.8445	0.358421	0.0176	0.0084492947
ss46526524	*rs41256984*	1	154.612126	0.0781	0.0199	0.887786	0.0050	0.0007131524
ss86331890		1	154.655767	0.8496	0.7217	0.395869	0.0229	0.0058551352
ss86308356		1	154.688915	0.0831	0.1118	0.738155	0.0111	0.0016932076
ss86282482		1	154.731272	0.0960	0.0845	0.771435	0.0090	0.0015680410
ss86294074		1	154.759226	0.3976	0.6307	0.427364	0.0153	0.0073050565
ss86329217		1	154.783579	0.8138	1.9400	0.164118	0.0346	0.0105029667
ss86321891		1	154.819792	0.5623	0.0000	0.999978	0.0000	0.0000002566
ss86302037		1	154.903343	0.5215	1.1370	0.286649	0.0197	0.0098391498
ss86330947		1	154.955228	0.2020	1.2033	0.273045	0.0257	0.0082744676
ss105238943		1	155.056509	0.8718	0.1142	0.735524	0.0098	0.0021881542
ss86287185		1	155.078137	0.2385	0.0513	0.820887	0.0050	0.0018228355
ss86329141		1	155.120957	0.7106	0.3882	0.533445	0.0130	0.0053538273
ss86340543		1	155.151875	0.6046	1.3118	0.252468	0.0215	0.0102665634
ss61471012	*rs41566585*	1	155.302655	0.3696	0.1875	0.665106	0.0083	0.0038589038
ss86311339		1	155.346627	0.1741	4.6543	0.031317	0.0516	0.0148446470

ss86278585		1	155.416072	0.3782	2.2787	0.131617	0.0288	0.0135244686
ss64704255	*rs42800089*	1	155.451307	0.4520	0.0528	0.818360	0.0042	0.0020558844
ss63262699	*rs42165137*	1	155.503791	0.0845	1.4229	0.233340	0.0406	0.0062795825
ss64677943	*rs42774205*	1	155.562462	0.9162	1.9377	0.164362	0.0475	0.0072924179
ss86340476		1	155.587367	0.4993	0.0000	0.998462	0.0000	0.0000179159
ss61551244	*rs41648924*	1	155.664424	0.7436	8.8294	0.003066	0.0615	0.0234667178
ss86332770		1	155.701301	0.1440	1.2254	0.268687	0.0291	0.0071793717
ss61547614	*rs41647233*	1	155.760349	0.7672	1.1450	0.284974	0.0236	0.0084216876
ss86341125		1	155.785809	0.4319	0.0048	0.945054	0.0013	0.0006355961
ss86330606		1	155.816822	0.2113	0.3284	0.566777	0.0131	0.0043541206
ss86325416		1	155.842601	0.4090	1.5555	0.212746	0.0236	0.0114037694
ss86274069		1	155.865313	0.2099	3.0600	0.080680	0.0394	0.0130558952
ss86321922		1	155.889669	0.0931	0.7835	0.376365	0.0285	0.0048205336
ss86309975		1	155.941811	0.1232	0.7398	0.390021	0.0239	0.0051582589
ss86341123		1	155.991205	0.5014	0.9996	0.317752	0.0189	0.0094647297
ss117966100		1	156.106965	0.2371	0.2022	0.653068	0.0098	0.0035581488
ss64369065	*rs42609964*	1	156.134713	0.5523	0.7115	0.399222	0.0161	0.0079822752
ss86329115		1	156.161612	0.5036	1.5991	0.206460	0.0241	0.0120252150
ss86314534		1	156.219382	0.0387	1.5357	0.215671	0.0601	0.0044666748
ss86292225		1	156.259623	0.5165	1.9354	0.164615	0.0265	0.0132142557
ss61469971	*rs41565968*	1	156.282211	0.5165	1.9354	0.164615	0.0265	0.0132142557
ss61486642	*rs41585131*	1	156.355591	0.2486	2.0684	0.150833	0.0307	0.0114529568
ss61509552	*rs41604901*	1	156.390945	0.7872	0.4756	0.490667	0.0158	0.0052884839
ss86288175		1	156.457927	0.6991	9.5413	0.002089	0.0618	0.0259966790
ss65197227	*rs43286253*	1	156.510437	0.3539	1.3889	0.238988	0.0240	0.0109860266
ss86294317		1	156.589825	0.4721	0.0359	0.849695	0.0035	0.0017556119
ss65197152	*rs43285378*	1	156.613986	0.5294	2.9078	0.088601	0.0315	0.0156961816
ss86308286		1	156.643382	0.0731	0.7604	0.383507	0.0314	0.0042567560
ss86306069		1	156.70991	0.1110	1.9501	0.163026	0.0402	0.0079431655
ss86310071		1	156.746565	0.4692	0.8578	0.354681	0.0176	0.0087581476
	rs29022938	1	156.806951	0.1390	0.0000	0.995221	0.0002	0.0000390340
ss86300448		1	156.888631	0.1619	4.9663	0.026164	0.0558	0.0151542461
ss61465386	*rs41565735*	1	156.913403	0.3066	3.5372	0.060425	0.0382	0.0162400958
ss86276529		1	156.988168	0.8181	2.5310	0.112086	0.0377	0.0112240661
ss86316513		1	157.085204	0.4936	0.3111	0.577170	0.0103	0.0051416067
ss86289015		1	157.105609	0.0888	0.6215	0.430741	0.0259	0.0041920073
ss86273704		1	157.127466	0.2156	0.0040	0.949518	0.0015	0.0005053405
ss86314205		1	157.214772	0.4900	3.8475	0.050217	0.0365	0.0182596111
ss86324300		1	157.253388	0.3431	0.0080	0.928717	0.0018	0.0007915524
ss86333177		1	157.276473	0.5924	2.1503	0.142994	0.0279	0.0134900197
ss86291056		1	157.340819	0.8188	0.3155	0.574508	0.0138	0.0041042467
ss86331938		1	157.372291	0.8274	3.9487	0.047298	0.0473	0.0135244650
ss86292024		1	157.392714	0.7335	0.6165	0.432622	0.0166	0.0064941573
	rs29015966	1	157.448706	0.4685	0.0458	0.830559	0.0040	0.0019736913
ss61512104	*rs41610114*	1	157.487276	0.3532	0.0032	0.954897	0.0011	0.0005071289
ss61505891	*rs41600022*	1	157.520046	0.6626	0.1216	0.727376	0.0071	0.0031890051
ss46526066	*rs41255213*	1	157.541725	0.1254	2.9216	0.087847	0.0457	0.0100279511

ss61518919	*rs41618669*	1	157.571776	0.2822	2.0471	0.152950	0.0287	0.0116423242
ss86335079		1	157.630892	0.2471	2.8285	0.093051	0.0365	0.0135931231
ss65148143	*rs43232718*	1	157.661921	0.1125	3.5438	0.060185	0.0547	0.0109251025
ss65148195	*rs43233970*	1	157.758037	0.1691	1.1902	0.275659	0.0276	0.0077425243
ss86292556		1	157.793121	0.7801	2.1420	0.143765	0.0340	0.0116659819
ss105240345		1	157.851579	0.8403	0.2581	0.611561	0.0130	0.0034896769
ss61524556	*rs41621593*	1	157.873825	0.1597	0.2581	0.611561	0.0130	0.0034896769
ss65198448	*rs43288865*	1	157.908805	0.1712	1.0903	0.296772	0.0260	0.0073911522
ss65148398	*rs43236373*	1	158.137015	0.2872	0.4215	0.516401	0.0134	0.0054911214
ss86333316		1	158.175656	0.3524	1.5000	0.221080	0.0237	0.0108241510
ss61522924	*rs41622793*	1	158.260956	0.0759	0.7122	0.399018	0.0287	0.0040301136
ss65198082	*rs43284202*	1	158.379036	0.1605	0.4896	0.484342	0.0176	0.0047299959
	rs29016284	1	158.406481	0.7163	0.0138	0.906477	0.0025	0.0010019372
ss86287770		1	158.45142	0.5387	0.5438	0.461114	0.0142	0.0070402848
ss64841077	*rs42934160*	1	158.489039	0.9255	0.1620	0.687442	0.0145	0.0020056339
ss86337394		1	158.521306	0.2615	0.3516	0.553386	0.0127	0.0049144003
ss86315502		1	158.555842	0.4606	0.6969	0.404111	0.0160	0.0079556693
ss61507110	*rs41602411*	1	158.588253	0.2092	0.1204	0.728733	0.0079	0.0026198593
ss86318684		1	158.628292	0.4542	0.3687	0.543938	0.0115	0.0056771860
ss65198312	*rs43286429*	1	158.706316	0.8861	0.3023	0.582593	0.0157	0.0031697063
ss86332417		1	158.761704	0.8145	0.1908	0.662412	0.0105	0.0031726070
ss65505303	*rs43585687*	1	158.796318	0.5222	6.6290	0.010239	0.0470	0.0234484567
ss86292029		1	158.831291	0.8911	3.3932	0.065891	0.0545	0.0105751742
ss86326852		1	158.855122	0.1999	0.1852	0.667102	0.0101	0.0032156019
ss86316650		1	158.94011	0.7951	0.2475	0.619020	0.0115	0.0037584541
ss86304736		1	158.992591	0.8682	0.0949	0.758124	0.0085	0.0019479242
ss86308015		1	159.015273	0.5186	5.2069	0.022798	0.0420	0.0209697465
ss61524564	*rs41621600*	1	159.055177	0.6941	3.1207	0.077741	0.0346	0.0146831129
ss65199125	*rs43285242*	1	159.100253	0.6819	4.2038	0.040707	0.0403	0.0174996043
ss61524567	*rs41621603*	1	159.144013	0.1884	0.4669	0.494661	0.0162	0.0049388533
ss86284888		1	159.167781	0.1676	2.9373	0.086999	0.0431	0.0120170376
ss105242442		1	159.208319	0.9262	0.7324	0.392392	0.0297	0.0040639327
ss86326473		1	159.23197	0.2987	0.1605	0.688804	0.0080	0.0033370055
ss64651205	*rs42746341*	1	159.270079	0.6877	2.1805	0.140221	0.0304	0.0130419886
ss64651191	*rs42745627*	1	159.309201	0.7865	0.1332	0.715292	0.0083	0.0027786054
ss61524569	*rs41621605*	1	159.468528	0.1956	0.6390	0.424353	0.0184	0.0057763917
ss65199735	*rs43282452*	1	159.495208	0.6633	1.9017	0.168326	0.0270	0.0120703996
ss61466392	*rs41569046*	1	159.595033	0.8567	1.9978	0.157980	0.0381	0.0093418803
ss86295630		1	159.62263	0.3732	0.8642	0.352878	0.0179	0.0083854333
ss61478765	*rs41577946*	1	159.684792	0.3574	9.6847	0.001934	0.0591	0.0271328896
ss86317744		1	159.738524	0.7443	5.2705	0.021987	0.0477	0.0181721234
ss86301649		1	159.767157	0.3145	3.6605	0.056127	0.0385	0.0166156972
ss86340250		1	159.874831	0.5115	3.2822	0.070465	0.0340	0.0169896093
ss65200489	*rs43290203*	1	159.949422	0.5387	4.9552	0.026332	0.0423	0.0210314183
ss65200563	*rs43290577*	1	159.975503	0.0766	0.7623	0.382914	0.0306	0.0043269608
ss65200647	*rs43282562*	1	159.997049	0.6676	3.2656	0.071177	0.0361	0.0160216391
ss61515907	*rs41617748*	1	160.114501	0.6189	1.0185	0.313226	0.0189	0.0088994280

ss86274540		1	160.152518	0.9004	0.0861	0.769226		0.0093	0.0016710051
ss86296731		1	160.186914	0.8718	2.7234	0.099337		0.0449	0.0100380289
ss86297066		1	160.220176	0.1999	0.0355	0.850547		0.0043	0.0013689935
ss86307234		1	160.252899	0.2271	0.5128	0.474161		0.0156	0.0054824329
ss86321601		1	160.279855	0.3725	1.0822	0.298566		0.0199	0.0092795703
ss86339821		1	160.385108	0.4678	0.2535	0.614748		0.0092	0.0045949432
	rs29024564	1	160.428313	0.4198	0.8037	0.370294		0.0166	0.0080620919
ss65200828	*rs43283639*	1	160.447283	0.2923	0.3069	0.579781		0.0109	0.0045133875
ss86292886		1	160.501199	0.8574	1.9017	0.168332		0.0353	0.0086388023
ss86301832		1	160.566262	0.3968	0.4758	0.490540		0.0127	0.0060949420
ss61524574	*rs41621610*	1	160.590619	0.9183	1.3685	0.242471		0.0400	0.0059973895
ss86329934		1	160.620864	0.1311	5.7391	0.016855		0.0652	0.0148505300
ss61562828	*rs41665018*	1	160.640866	0.0924	1.5698	0.210657		0.0402	0.0067399247
ss86278533		1	160.690162	0.7206	0.6121	0.434263		0.0156	0.0062827275
ss86303675		1	160.749268	0.4162	4.4939	0.034369		0.0393	0.0190771226
ss86324522		1	160.772959	0.1383	3.5208	0.061020		0.0510	0.0121523232
ss86331037		1	160.827405	0.7830	9.0322	0.002748		0.0669	0.0227377253
ss61471704	*rs41572453*	1	160.848631	0.3603	0.1100	0.740204		0.0065	0.0029758652
ss64569332	*rs42701437*	1	160.887459	0.5000	1.6874	0.194374		0.0239	0.0119699294
ss86321690		1	160.952611	0.1304	1.7679	0.184081		0.0354	0.0080209257
ss86313745		1	160.97658	0.0638	0.3792	0.538230		0.0229	0.0027280277
ss61501016	*rs41602958*	1	161.021444	0.7486	0.0003	0.985867		0.0004	0.0001416579
ss86307836		1	161.062329	0.5057	1.0431	0.307462		0.0191	0.0095517333
ss86332648		2	0.0351	0.8317	0.2193	0.639714		0.0115	0.0032077889
ss64807001	*rs42900661*	2	0.191748	0.5989	0.1381	0.710315		0.0073	0.0034992991
ss86294932		2	0.217606	0.9570	6.4994	0.011005		0.1173	0.0096503421
ss65201481	*rs43287979*	2	0.240833	0.7830	1.0511	0.305607		0.0232	0.0078801117
ss117966782		2	0.265146	0.2937	0.2220	0.637678		0.0096	0.0039881178
ss86339045		2	0.513819	0.4771	0.6612	0.416411		0.0151	0.0075458804
ss61517065	*rs41611370*	2	0.582804	0.5451	0.9632	0.326735		0.0181	0.0089704140
ss63806880	*rs42361465*	2	0.612925	0.8431	1.0264	0.311350		0.0257	0.0067951190
ss86292875		2	0.643389	0.4599	1.4300	0.232169		0.0223	0.0110898746
ss63806818	*rs42361434*	2	0.66614	0.0917	0.0979	0.754498		0.0101	0.0016762019
ss86332309		2	0.692786	0.6383	0.7583	0.384162		0.0175	0.0080882065
ss117966506		2	0.730619	0.5258	6.3350	0.012061		0.0465	0.0231739079
ss86286290		2	0.782503	0.3052	0.4862	0.485869		0.0141	0.0059679650
ss61517077	*rs41611382*	2	0.81975	0.5430	0.1231	0.725854		0.0065	0.0032323226
ss86304917		2	0.862583	0.3059	0.6241	0.429809		0.0160	0.0067740572
ss61517063	*rs41611368*	2	0.883341	0.7600	3.0486	0.081246		0.0380	0.0138556266
	rs29024554	2	0.943569	0.4097	0.2449	0.620831		0.0092	0.0044690274
ss105274143		2	0.967245	0.2299	1.6169	0.203949		0.0270	0.0095762322
ss86273503		2	1.007391	0.4850	0.0542	0.815947		0.0044	0.0022118718
ss46526613	*rs41257069*	2	1.03048	0.6275	2.0666	0.151006		0.0277	0.0129419142
ss86339776		2	1.076041	0.6318	0.6995	0.403236		0.0160	0.0074479532
ss117963613		2	1.103143	0.8238	7.9965	0.004822		0.0700	0.0203214547
ss86340519		2	1.148542	0.3732	2.7885	0.095392		0.0314	0.0147098273
ss65201636	*rs43282638*	2	1.200474	0.4327	1.4019	0.236806		0.0225	0.0110457212

ss65201579	*rs43288576*	2	1.221307	0.5638	2.9783	0.084832	0.0332	0.0163085558
ss86320116		2	1.252338	0.4463	2.6021	0.107174	0.0307	0.0151929068
ss65201466	*rs43287965*	2	1.283798	0.6834	0.0279	0.867425	0.0034	0.0014603214
ss86285597		2	1.309839	0.3173	0.1654	0.684330	0.0082	0.0035506682
ss86298454		2	1.352094	0.6805	0.1667	0.683208	0.0082	0.0035682634
ss64728056	*rs42827739*	2	1.377407	0.4305	0.0692	0.792514	0.0051	0.0024841362
ss86290695		2	1.439832	0.3295	2.7974	0.094870	0.0333	0.0147335582
ss86339778		2	1.501294	0.2479	0.8739	0.350197	0.0202	0.0075222784
ss61506206	*rs41603424*	2	1.525206	0.3854	1.2200	0.269743	0.0211	0.0100081818
ss86283223		2	1.550331	0.9628	0.1747	0.676086	0.0206	0.0014801996
ss86289687		2	1.82271	0.1798	8.1864	0.004347	0.0672	0.0198111033
ss61541979	*rs41639506*	2	1.854042	0.4011	1.2903	0.256379	0.0219	0.0105264037
ss61493051	*rs41591798*	2	1.892028	0.6146	2.2515	0.133938	0.0287	0.0135764646
ss86302312		2	1.922119	0.1583	6.5415	0.010750	0.0659	0.0175590649
ss86312674		2	1.952252	0.6017	3.4802	0.062529	0.0356	0.0170849770
ss86340044		2	2.008109	0.8016	1.3703	0.242155	0.0270	0.0085843870
ss86334403		2	2.193417	0.1884	6.9668	0.008490	0.0628	0.0192085319
ss86337010		2	2.241157	0.2299	0.3864	0.534401	0.0141	0.0050104118
ss86302636		2	2.271204	0.1999	2.0839	0.149311	0.0346	0.0110733548
ss86305378		2	2.35263	0.3001	0.3907	0.532124	0.0128	0.0053848185
ss86338135		2	2.687252	0.4427	0.0748	0.784613	0.0050	0.0024866844
ss61511612	*rs41613523*	2	2.874375	0.8066	1.8888	0.169778	0.0319	0.0099548587
ss86330023		2	2.920608	0.1934	1.7192	0.190229	0.0303	0.0094574191
ss86341553		2	3.006913	0.1017	7.8050	0.005354	0.0854	0.0155973962
ss86311687		2	3.029342	0.1032	7.8028	0.005360	0.0843	0.0155989491
ss86339430		2	3.107342	0.2278	3.5860	0.058683	0.0414	0.0145735611
ss86313845		2	3.212952	0.2529	0.0021	0.963314	0.0010	0.0003703402
ss86297853		2	3.233858	0.6232	0.0908	0.763204	0.0059	0.0027688177
ss86283853		2	3.25863	0.9592	2.0799	0.149696	0.0682	0.0053432898
ss86291691		2	3.303349	0.4119	2.1766	0.140576	0.0279	0.0135027401
ss105271634		2	3.469688	0.4599	5.6912	0.017317	0.0439	0.0218047828
ss86289352		2	3.51158	0.0910	1.7985	0.180337	0.0432	0.0071371404
ss61566021	*rs41659668*	2	3.604635	0.1562	0.0155	0.901074	0.0031	0.0008163818
ss86292796		2	3.724223	0.4133	0.4383	0.508151	0.0127	0.0061542226
ss86277193		2	3.750891	0.7507	0.2212	0.638289	0.0104	0.0038747319
ss61518394	*rs41614151*	2	3.843351	0.3216	0.2043	0.651399	0.0090	0.0039208873
ss86295461		2	3.871116	0.6920	2.3093	0.129055	0.0315	0.0134414353
ss86332815		2	3.903006	0.0996	0.1206	0.728494	0.0108	0.0019454881
ss86322131		2	3.943247	0.2622	1.0637	0.302722	0.0220	0.0085273008
ss86331347		2	3.983055	0.6375	0.0176	0.894482	0.0027	0.0012290852
ss86293775		2	4.033678	0.6497	0.2599	0.610356	0.0098	0.0044547563
ss86289918		2	4.065885	0.3410	2.2794	0.131558	0.0298	0.0134034991
ss61563023	*rs41657820*	2	4.138821	0.5036	1.2920	0.256062	0.0214	0.0106999895
ss61518389	*rs41614146*	2	4.168811	0.0903	0.1355	0.712958	0.0111	0.0018158196
ss86336883		2	4.206765	0.2980	0.1009	0.750894	0.0065	0.0027143650
ss86329886		2	4.247772	0.3746	0.2093	0.647431	0.0087	0.0040848245
ss86323462		2	4.283358	0.4377	0.2808	0.596360	0.0097	0.0047724158

ss61523273	*rs41619945*	2	4.320685	0.1361	1.8653	0.172450		0.0366	0.0086179714
ss105261279		2	4.344035	0.5774	0.3597	0.548888		0.0116	0.0056382525
ss65202204	*rs43288103*	2	4.499996	0.9298	12.2031	0.000507		0.1242	0.0162169553
ss65202137	*rs43288036*	2	4.555873	0.0702	12.2031	0.000507		0.1242	0.0162169553
ss61541295	*rs41643132*	2	4.583359	0.8890	12.3587	0.000467		0.1021	0.0201494026
ss64807470	*rs42904827*	2	4.61649	0.7543	0.9711	0.324748		0.0209	0.0077362952
ss64807421	*rs42904778*	2	4.639097	0.7443	2.3854	0.122928		0.0324	0.0123436866
ss46526222	*rs41255361*	2	4.660752	0.4262	0.1940	0.659771		0.0081	0.0039563481
ss65201975	*rs43285975*	2	4.681598	0.7600	1.2540	0.263173		0.0245	0.0089326913
ss64757433	*rs42855482*	2	4.771835	0.7787	1.8993	0.168596		0.0309	0.0106407029
ss65203170	*rs43287050*	2	4.913636	0.6032	1.8115	0.178766		0.0253	0.0121320215
ss86290722		2	4.963099	0.1956	0.0230	0.879534		0.0035	0.0010925624
ss65203213	*rs43287093*	2	4.983158	0.1168	0.3740	0.541014		0.0176	0.0036343063
ss86323551		2	5.017643	0.4083	0.0949	0.758128		0.0057	0.0027628327
ss61541788	*rs41637221*	2	5.091208	0.5236	2.6991	0.100858		0.0304	0.0151530505
ss86338935		2	5.112946	0.0322	1.0039	0.316720		0.0505	0.0031522460
ss61496811	*rs41592756*	2	5.156156	0.8388	3.5264	0.060816		0.0465	0.0125755464
	rs29012920	2	5.178696	0.5766	0.0015	0.968736		0.0007	0.0003611387
ss86340194		2	5.237005	0.3603	4.4695	0.034861		0.0401	0.0184730924
ss86289261		2	5.334097	0.7693	9.0047	0.002789		0.0646	0.0229178340
ss86328437		2	5.385944	0.8524	0.1312	0.717347		0.0095	0.0023850025
ss86285846		2	5.417403	0.3496	2.3557	0.125278		0.0294	0.0133894634
ss86341647		2	5.550332	0.4878	3.1508	0.076327		0.0339	0.0169368418
ss86290936		2	5.614811	0.7822	13.0131	0.000331		0.0813	0.0276847355
ss86320927		2	5.635267	0.1705	0.1608	0.688500		0.0099	0.0028084079
ss86277114		2	5.683821	0.5115	2.1195	0.145885		0.0281	0.0140432275
	rs29021061	2	5.735609	0.2980	0.3634	0.546795		0.0122	0.0050940706
ss86333366		2	5.809993	0.7758	0.5698	0.450581		0.0171	0.0059469654
ss86276190		2	5.874037	0.5860	3.8582	0.049900		0.0367	0.0178112382
ss117966897		2	5.91353	0.5824	4.3567	0.037226		0.0388	0.0188921863
	rs29011261	2	5.937141	0.2908	1.6849	0.194701		0.0267	0.0110160366
ss86287493		2	6.029976	0.2808	1.0022	0.317123		0.0201	0.0081178835
ss105253549		2	6.053019	0.6726	3.5776	0.058978		0.0365	0.0160606121
ss105256184		2	6.075489	0.6726	3.5776	0.058978		0.0365	0.0160606121
ss105253071		2	6.09595	0.0931	8.0435	0.004700		0.0885	0.0149543739
ss86309406		2	6.116319	0.1519	0.7563	0.384790		0.0224	0.0057810246
ss86324367		2	6.26108	0.1032	0.0028	0.957723		0.0017	0.0003130311
	rs29010906	2	6.363054	0.4226	0.7518	0.386204		0.0162	0.0078898508
ss86289769		2	6.390118	0.8138	0.1165	0.732994		0.0079	0.0023978365
ss86286355		2	6.470682	0.1117	1.5101	0.219544		0.0357	0.0070789730
ss86326702		2	6.502337	0.1819	2.3879	0.122733		0.0367	0.0109384840
ss86286053		2	6.99675	0.3403	1.6583	0.198266		0.0245	0.0110210634
ss86337493		2	7.025774	0.6755	3.6209	0.057469		0.0380	0.0166781571
ss86337894		2	7.107333	0.8395	0.0866	0.768661		0.0075	0.0020223233
	rs29023584	2	7.136739	0.0903	3.7982	0.051710		0.0633	0.0103924725
ss61541841	*rs41638273*	2	7.164003	0.9398	2.1819	0.140091		0.0582	0.0065813082
ss61532639	*rs41626743*	2	7.229722	0.4599	1.1169	0.290962		0.0194	0.0096228795

ss86312355		2	7.441108	0.8582	6.0017	0.014537	0.0639	0.0155468725
	rs29018577	2	7.480738	0.3181	0.5438	0.461095	0.0146	0.0063303879
ss61541631	*rs41636170*	2	7.520464	0.8883	4.0315	0.045044	0.0597	0.0118530459
ss86321482		2	7.564438	0.6289	0.0409	0.839733	0.0039	0.0018099270
ss86288776		2	7.643293	0.2285	2.7162	0.099785	0.0368	0.0129833970
	rs29017456	2	7.664939	0.7937	7.8925	0.005103	0.0650	0.0212848966
ss46527095	*rs41255638*	2	7.744686	0.7371	4.5055	0.034139	0.0450	0.0174448899
ss65202862	*rs43285042*	2	7.914705	0.6010	3.0815	0.079628	0.0340	0.0163130370
ss64992874	*rs43088149*	2	7.936126	0.5344	4.1672	0.041590	0.0373	0.0185837786
ss86338738		2	8.065961	0.7199	0.5253	0.468819	0.0150	0.0060590858
ss64735548	*rs42832199*	2	8.160688	0.3403	0.1386	0.709775	0.0075	0.0033714504
ss61478650	*rs41577233*	2	8.184569	0.6562	0.0101	0.920035	0.0020	0.0009166960
ss65203098	*rs43286478*	2	8.263018	0.8897	1.2872	0.256947	0.0329	0.0064649019
ss117963644		2	8.289738	0.6483	1.3567	0.244508	0.0233	0.0106032538
ss61562445	*rs41661744*	2	8.385956	0.6447	0.0315	0.859286	0.0035	0.0015945255
ss61511126	*rs41610251*	2	8.47994	0.7342	6.5246	0.010851	0.0553	0.0215895743
ss61493738	*rs41590264*	2	8.566611	0.5716	1.2032	0.273069	0.0204	0.0099809254
ss63549024	*rs42269671*	2	8.696446	0.4312	9.2217	0.002481	0.0556	0.0272931868
ss64946190	*rs43043879*	2	8.730899	0.2092	9.5201	0.002113	0.0690	0.0228172739
ss64946201	*rs43043890*	2	8.754837	0.5867	5.7041	0.017191	0.0447	0.0216597157
ss86303971		2	8.780974	0.9670	0.0123	0.911737	0.0058	0.0003689838
ss61493736	*rs41590262*	2	8.815405	0.6404	3.9340	0.047712	0.0378	0.0174273616
ss64268590	*rs43773236*	2	8.848489	0.2915	2.2488	0.134174	0.0304	0.0125702651
ss86293534		2	8.881566	0.6189	2.0152	0.156178	0.0267	0.0125928417
ss64268392	*rs43773490*	2	8.902713	0.2471	4.4632	0.034988	0.0474	0.0176350472
ss64268262	*rs43772412*	2	8.948425	0.1984	0.0278	0.867551	0.0039	0.0012311184
ss61531221	*rs41633180*	2	8.97194	0.5287	3.3996	0.065635	0.0342	0.0170515434
ss65203408	*rs43288488*	2	8.993804	0.1777	0.0054	0.941682	0.0016	0.0004675149
ss65203384	*rs43288464*	2	9.017718	0.3252	1.6787	0.195531	0.0251	0.0110117303
ss65087333	*rs43179850*	2	9.051445	0.1483	0.4393	0.507682	0.0177	0.0044786214
ss86291502		2	9.28846	0.6784	10.4326	0.001296	0.0627	0.0273717565
ss61506143	*rs41603362*	2	9.337707	0.4334	1.8746	0.171388	0.0256	0.0125769679
ss62866233	*rs42025573*	2	9.433765	0.6511	0.6736	0.412088	0.0162	0.0073404597
ss62866347	*rs42025610*	2	9.454759	0.3510	0.5783	0.447246	0.0150	0.0068362135
ss62866317	*rs42025600*	2	9.483187	0.5000	1.5588	0.212259	0.0229	0.0114656652
ss65108457	*rs43195774*	2	9.505404	0.6734	3.4058	0.065392	0.0357	0.0156889809
ss65203456	*rs43289236*	2	9.529273	0.3145	1.7411	0.187429	0.0267	0.0114908545
ss65203468	*rs43289248*	2	9.553018	0.5029	0.9902	0.320041	0.0187	0.0093443264
ss61484011	*rs41583863*	2	9.590181	0.3897	0.3212	0.571093	0.0108	0.0051219050
ss65203428	*rs43288508*	2	9.664438	0.3195	0.4820	0.487750	0.0143	0.0062012266
ss65203539	*rs43289319*	2	9.728844	0.0888	3.3108	0.069255	0.0600	0.0097195393
ss86340765		2	9.767528	0.6175	0.8124	0.367734	0.0169	0.0079684818
	rs29013415	2	9.810426	0.8560	1.2144	0.270849	0.0297	0.0073125439
ss65203647	*rs43289727*	2	9.851529	0.6297	0.3282	0.566896	0.0111	0.0051946091
ss63963451	*rs42405361*	2	9.958808	0.3166	0.0295	0.863673	0.0035	0.0015138121
ss65031843	*rs43128174*	2	9.993855	0.6368	4.5350	0.033559	0.0400	0.0185230051
ss65203722	*rs43287402*	2	10.020185	0.3632	4.5350	0.033559	0.0400	0.0185230051

ss117966497		2	10.09261	0.3904	0.0591	0.808044	0.0046	0.0021784922
ss86337153		2	10.34818	0.5294	0.1640	0.685600	0.0077	0.0038244915
	rs29015637	2	10.373211	0.1354	1.9726	0.160622	0.0389	0.0091115592
ss61467940	*rs41564963*	2	10.42978	0.4699	0.5331	0.465533	0.0130	0.0064882869
ss61516700	*rs41618001*	2	10.561002	0.5630	0.0657	0.797767	0.0047	0.0023297996
ss61472590	*rs41571613*	2	10.583794	0.7156	0.0004	0.983784	0.0004	0.0001783544
ss61478906		2	10.659905	0.9241	0.9335	0.334302	0.0336	0.0047114235
ss61540367	*rs41642713*	2	10.76038	0.5946	1.2714	0.259902	0.0211	0.0101784701
ss86297649		2	10.785338	0.2400	3.8873	0.049048	0.0418	0.0152453220
ss61473746	*rs41574062*	2	10.85095	0.7887	0.3399	0.560096	0.0130	0.0043339122
ss86300716		2	11.005019	0.5838	0.1120	0.738009	0.0061	0.0029709536
ss61507619	*rs41607908*	2	11.032432	0.7414	1.1728	0.279196	0.0227	0.0086940364
ss61528725	*rs41628655*	2	11.060395	0.4097	0.9364	0.333541	0.0178	0.0086135880
ss86284129		2	11.722234	0.2550	1.7203	0.190081	0.0265	0.0100574336
ss86296049		2	11.74834	0.7436	1.4378	0.230909	0.0242	0.0092305133
ss86321159		2	11.879297	0.9148	1.1697	0.279841	0.0353	0.0055004452
ss61472709	*rs41573932*	2	12.030067	0.2085	1.6574	0.198381	0.0287	0.0094780869
ss61484233	*rs41585680*	2	12.087589	0.2299	1.8292	0.176665	0.0295	0.0104357268
ss61484222	*rs41585669*	2	12.110271	0.6497	1.6061	0.205467	0.0244	0.0111127967
ss61484209	*rs41585656*	2	12.165536	0.7292	0.2845	0.593906	0.0111	0.0043744722
ss86292443		2	12.213338	0.5795	0.2181	0.640626	0.0090	0.0043898892
ss61542394	*rs41644214*	2	12.291213	0.8739	2.0418	0.153476	0.0388	0.0085595177
ss86331708		2	12.406643	0.5917	3.4600	0.063293	0.0350	0.0169318774
ss61542264	*rs41643286*	2	12.426923	0.6089	1.3529	0.245167	0.0222	0.0105721457
ss61479127	*rs41580394*	2	12.523554	0.1834	0.7475	0.387556	0.0205	0.0061289416
ss61473672	*rs41573088*	2	12.587408	0.6934	1.5065	0.220086	0.0236	0.0100434667
ss105254777		2	12.637006	0.1662	0.0118	0.913525	0.0027	0.0007510380
ss61506461	*rs41606071*	2	12.764123	0.4097	6.5150	0.010909	0.0477	0.0230542570
ss117966843		2	12.801682	0.6254	0.0094	0.922697	0.0019	0.0008829733
ss61517389	*rs41614867*	2	12.990403	0.8990	5.1033	0.024189	0.0694	0.0126058010
ss117966764		2	13.045886	0.1748	5.1060	0.024152	0.0550	0.0158727114
ss64825581	*rs42923902*	2	13.076787	0.2471	4.8171	0.028508	0.0482	0.0179414947
ss105263926		2	13.136277	0.2894	0.0130	0.909215	0.0023	0.0009541166
ss86280157		2	13.219799	0.2464	1.6434	0.200287	0.0276	0.0102339842
ss61466641	*rs41571293*	2	13.295993	0.9054	6.8017	0.009303	0.0813	0.0139183114
ss61566406	*rs41664246*	2	13.41939	0.8231	3.2074	0.073740	0.0434	0.0126495778
ss105254788		2	13.50918	0.8639	0.9233	0.336934	0.0268	0.0063053082
ss61527333	*rs41622697*	2	13.533477	0.3159	1.6247	0.202864	0.0256	0.0110691202
ss105260438		2	13.584794	0.4850	11.2848	0.000824	0.0604	0.0301480769
ss86326891		2	13.616391	0.6941	0.0858	0.769712	0.0058	0.0024675258
ss105260440		2	13.650485	0.3725	7.7411	0.005544	0.0527	0.0246521180
ss61527315	*rs43743867*	2	13.682596	0.3331	17.6598	0.000030	0.0820	0.0364132207
ss86319201		2	13.718482	0.5000	3.0489	0.081233	0.0325	0.0162308497
ss46526475	*rs41256938*	2	13.762689	0.7665	8.6724	0.003338	0.0674	0.0241288797
	rs29013687	2	13.921054	0.3940	8.2633	0.004169	0.0553	0.0263881035
	rs29012413	2	13.941816	0.3968	4.4636	0.034979	0.0404	0.0193179907
ss61542305	*rs41643327*	2	14.097645	0.4721	1.4825	0.223791	0.0223	0.0111192100

ss86286906		2	14.156827	0.4062	13.3607	0.000276		0.0678	0.0327267359
	rs29018046	2	14.210087	0.3317	2.8373	0.092549		0.0328	0.0145607146
ss86318772		2	14.277681	0.5867	1.0701	0.301277		0.0191	0.0092736453
ss86304971		2	14.335739	0.6103	13.6072	0.000243		0.0690	0.0328284038
ss105261482		2	14.563746	0.2923	1.6414	0.200565		0.0271	0.0112049877
ss61496822	*rs41592766*	2	14.701653	0.6812	9.6131	0.002010		0.0631	0.0274109493
ss86336557		2	14.754414	0.1096	0.2936	0.588080		0.0158	0.0030828841
ss61511927	*rs41609642*	2	14.778112	0.5666	6.7323	0.009668		0.0490	0.0240803975
ss86328527		2	14.82177	0.1547	22.7446	0.000002		0.1164	0.0304537602
ss63648529	*rs42301357*	2	14.846418	0.7099	7.9559	0.004930		0.0587	0.0241629173
	rs29012658	2	14.869773	0.5222	19.8194	0.000010		0.0824	0.0411367503
	rs29010223	2	14.916341	0.4771	0.0464	0.829488		0.0039	0.0019272911
ss105262181		2	14.945397	0.3689	3.3547	0.067442		0.0345	0.0160495358
ss61473397	*rs41569815*	2	14.985891	0.0458	0.0055	0.940895		0.0033	0.0002869421
ss117966791		2	15.082193	0.7579	8.9253	0.002911		0.0624	0.0229087264
ss63472187	*rs42238130*	2	15.133598	0.5946	0.9017	0.342647		0.0174	0.0084095128
ss86276034		2	15.178676	0.3711	0.2288	0.632583		0.0089	0.0041345141
ss63471929	*rs42238044*	2	15.217859	0.5903	6.0037	0.014521		0.0452	0.0218736698
	rs29023429	2	15.2773	0.7285	0.7318	0.392596		0.0179	0.0070927031
ss86314757		2	15.346346	0.6218	0.8089	0.368756		0.0172	0.0081031307
ss86308813		2	15.370081	0.3066	1.2425	0.265373		0.0224	0.0095123123
ss61474915	*rs41573501*	2	15.399602	0.4033	0.6235	0.430035		0.0148	0.0071383375
ss63470888	*rs42242397*	2	15.4211	0.5838	0.5721	0.449671		0.0141	0.0068408643
ss63470684	*rs42241429*	2	15.457642	0.4191	0.7021	0.402366		0.0157	0.0076227512
ss86313045		2	15.522415	0.9126	0.2170	0.641455		0.0157	0.0024989407
ss63470078	*rs42239927*	2	15.576613	0.7457	7.2401	0.007300		0.0566	0.0214526977
ss63469844	*rs42239850*	2	15.708505	0.9506	0.2714	0.602549		0.0227	0.0021307126
ss63469613	*rs42239273*	2	15.798248	0.5451	0.0755	0.783590		0.0052	0.0025627986
ss86334390		2	15.821188	0.7006	1.4864	0.223192		0.0252	0.0105581121
ss64599266	*rs42721226*	2	15.969666	0.8818	0.9298	0.335252		0.0271	0.0056501866
ss64599318	*rs42722352*	2	16.000786	0.5989	4.2663	0.039244		0.0396	0.0190353433
ss64885364	*rs42979624*	2	16.039265	0.1046	1.2661	0.260886		0.0346	0.0064805313
ss61564791	*rs41663754*	2	16.069902	0.1089	1.4182	0.234114		0.0361	0.0070131883
ss64218614	*rs42533759*	2	16.110744	0.3288	0.0016	0.968097		0.0008	0.0003705110
ss86301607		2	16.166342	0.4277	0.0217	0.883046		0.0028	0.0013586089
ss64862413	*rs42954224*	2	16.41232	0.2550	0.0271	0.869211		0.0036	0.0013832134
ss61478501	*rs41576094*	2	16.470703	0.1991	1.2117	0.271380		0.0255	0.0081184287
ss86338628		2	16.491571	0.7063	0.1477	0.700852		0.0077	0.0032078308
ss65204982	*rs43291061*	2	16.525404	0.1870	1.4862	0.223217		0.0293	0.0089125012
ss86330425		2	16.561482	0.9076	0.0246	0.875393		0.0051	0.0008504445
ss61500695	*rs41599450*	2	16.656832	0.9305	1.6381	0.201012		0.0481	0.0062176096
ss61517051	*rs41611356*	2	16.736404	0.4105	1.9672	0.161195		0.0260	0.0125760555
ss64124525	*rs42483474*	2	16.852287	0.8718	4.5916	0.032474		0.0587	0.0131222621
ss61531793	*rs41630342*	2	16.873683	0.1074	4.9462	0.026468		0.0660	0.0126550777
ss61531215	*rs41633174*	2	16.90382	0.2665	3.1852	0.074743		0.0378	0.0147699264
ss64968768	*rs43058109*	2	17.060623	0.4183	0.7690	0.380832		0.0163	0.0079240596
ss64809650	*rs42902881*	2	17.101058	0.3890	3.5876	0.058627		0.0369	0.0175288914

ss64869803	*rs42965357*	2	17.135111	0.2744	3.2045	0.073871	0.0374	0.0148956866
	rs29020884	2	17.173049	0.5473	2.6261	0.105572	0.0316	0.0156455809
ss65205863	*rs43290941*	2	17.26	0.4656	0.0484	0.825883	0.0042	0.0021039687
ss61568652	*rs41665738*	2	17.291249	0.3639	0.0376	0.846348	0.0037	0.0017197399
ss61505734	*rs41608869*	2	17.321843	0.2443	0.1781	0.673105	0.0092	0.0034042754
ss86339675		2	17.493513	0.0688	3.0497	0.081194	0.0651	0.0083331924
ss61542870	*rs41636993*	2	17.551645	0.2393	1.5488	0.213726	0.0265	0.0096558129
ss86314679		2	17.716352	0.6655	5.2094	0.022766	0.0439	0.0195540349
ss86341194		2	17.791238	0.1669	1.4846	0.223473	0.0301	0.0083599432
	rs29013591	2	17.854128	0.1132	0.6731	0.412247	0.0241	0.0048469386
ss38326487	*rs29014795*	2	17.931827	0.2471	2.8164	0.093755	0.0353	0.0131192649
ss86301888		2	18.057166	0.3223	0.7953	0.372821	0.0181	0.0079088274
ss86301355		2	18.170941	0.5716	0.0560	0.812934	0.0044	0.0021496929
ss65069076	*rs43157675*	2	18.212187	0.0860	0.0008	0.978029	0.0009	0.0001410315
ss117975173		2	18.257291	0.0731	0.7641	0.382345	0.0311	0.0042185164
ss65206215	*rs43292493*	2	18.293027	0.1991	0.4066	0.523914	0.0146	0.0046672012
ss61502365	*rs41606275*	2	18.342153	0.8016	0.3643	0.546324	0.0139	0.0044082986
ss64517618	*rs42677697*	2	18.389193	0.0946	1.2500	0.263930	0.0352	0.0060262368
ss64517928	*rs42679152*	2	18.48698	0.4477	0.5729	0.449357	0.0139	0.0068974367
ss61484393	*rs41579146*	2	18.531691	0.8381	7.7006	0.005668	0.0706	0.0191713596
ss61467993	*rs41565016*	2	18.599754	0.4019	3.6869	0.055251	0.0362	0.0173908495
ss61542820	*rs41636943*	2	18.665031	0.1426	0.2231	0.636822	0.0126	0.0030814932
ss61542829	*rs41636952*	2	18.69359	0.6533	0.6393	0.424239	0.0166	0.0075059238
ss65205427	*rs43294605*	2	18.721368	0.3696	0.3885	0.533279	0.0119	0.0055633628
ss86335990		2	18.754377	0.7077	3.2012	0.074018	0.0374	0.0154765215
ss61542838	*rs41636961*	2	18.791279	0.3302	0.4238	0.515281	0.0135	0.0059728981
ss117966826		2	18.824551	0.4355	0.4127	0.520795	0.0118	0.0058191403
ss65205573	*rs43296651*	2	18.849986	0.6239	0.8228	0.364689	0.0173	0.0081305068
	rs29022477	2	18.931834	0.4377	2.9100	0.088478	0.0315	0.0155015199
ss65205688	*rs43289867*	2	18.964971	0.0996	1.0479	0.306352	0.0328	0.0058773725
ss86274672		2	19.053041	0.5315	0.6108	0.434759	0.0145	0.0072193994
ss105263466		2	19.105153	0.4799	1.4597	0.227383	0.0221	0.0110476280
ss86315379		2	19.12618	0.5344	0.4028	0.525864	0.0119	0.0059131074
	rs29020795	2	19.202356	0.4176	0.7278	0.393898	0.0158	0.0076651122
ss65190194	*rs43278780*	2	19.229951	0.2815	2.4396	0.118758	0.0329	0.0133218434
ss61509328	*rs41603483*	2	19.2559	0.3223	0.4052	0.524607	0.0129	0.0056384130
ss65190378	*rs43279563*	2	19.315116	0.3001	0.4174	0.518430	0.0130	0.0054672404
ss61485892	*rs41586691*	2	19.455276	0.1476	0.1558	0.693219	0.0103	0.0025984881
ss65190559	*rs43273549*	2	19.481404	0.1196	0.9579	0.328060	0.0281	0.0059292268
ss65206425	*rs43293795*	2	19.555268	0.3847	0.0167	0.897318	0.0025	0.0011614077
ss86330199		2	19.681411	0.7436	0.0050	0.943859	0.0015	0.0005706367
ss86321226		2	19.710623	0.2758	0.1737	0.676960	0.0088	0.0035065966
ss63554025	*rs42267687*	2	19.731355	0.3195	0.3644	0.546270	0.0121	0.0052532324
ss86310789		2	19.801474	0.4491	0.0118	0.913634	0.0020	0.0010098661
ss61494015	*rs41592225*	2	19.832559	0.6970	0.0131	0.908784	0.0023	0.0009809769
ss63554433	*rs42268208*	2	19.91087	0.8159	0.7911	0.374071	0.0209	0.0062796069
ss63554463	*rs42268218*	2	19.938235	0.8768	0.2945	0.587499	0.0150	0.0032387304

ss61563042	*rs41658439*	2	19.963403	0.0666	7.5101	0.006293	0.0995	0.0123767746
ss86318716		2	20.044858	0.4807	0.5485	0.459168	0.0140	0.0069814848
ss86340253		2	20.076075	0.0616	0.0574	0.810668	0.0094	0.0010824265
	rs29014139	2	20.235088	0.3825	0.1421	0.706282	0.0073	0.0034268028
ss105263449		2	20.312917	0.4964	0.6774	0.410763	0.0155	0.0077286135
ss64509206	*rs42671076*	2	20.347437	0.1032	0.8195	0.365635	0.0279	0.0051651323
ss64509178	*rs42671062*	2	20.367577	0.8854	1.5775	0.209543	0.0371	0.0075339174
ss86310733		2	20.391165	0.6196	1.0468	0.306591	0.0200	0.0094493596
ss63554961	*rs42270183*	2	20.504697	0.6748	0.2503	0.616993	0.0100	0.0043859060
ss86309669		2	20.549824	0.0852	0.9341	0.334136	0.0328	0.0051117223
ss61494342	*rs41596643*	2	20.648848	0.7923	0.9352	0.333855	0.0234	0.0076895917
	rs29020690	2	20.7103	0.7844	0.2613	0.609375	0.0118	0.0039905903
ss65207387	*rs43297742*	2	20.737588	0.9491	0.1647	0.684959	0.0174	0.0016833373
ss65207287	*rs43296742*	2	20.813843	0.8761	1.6723	0.196381	0.0359	0.0077858974
ss61508930	*rs41600398*	2	20.848885	0.2364	3.3303	0.068443	0.0403	0.0145373545
	rs29022995	2	20.978361	0.9549	2.0641	0.151258	0.0639	0.0055080202
ss86339046		2	21.00037	0.1440	4.8249	0.028380	0.0567	0.0139651135
ss86339183		2	21.022389	0.7973	8.5626	0.003543	0.0664	0.0214571963
ss38334588	*rs29024488*	2	21.084997	0.0931	0.3368	0.561855	0.0178	0.0030099222
ss105263439		2	21.127634	0.1268	3.5605	0.059585	0.0533	0.0117982904
ss65206939	*rs43292394*	2	21.197106	0.2507	0.2923	0.588902	0.0115	0.0043350534
ss61508929	*rs41600397*	2	21.269246	0.4828	2.1930	0.139088	0.0276	0.0138085482
ss86323046		2	21.301375	0.2779	0.0685	0.793602	0.0054	0.0021694508
ss65207039	*rs43293394*	2	21.328418	0.1963	0.7258	0.394540	0.0201	0.0063490789
ss86336145		2	21.388968	0.2543	4.9242	0.026804	0.0469	0.0178032640
ss65211014	*rs43294756*	2	21.525506	0.6511	0.4894	0.484413	0.0134	0.0060874283
ss117966901		2	21.560141	0.5415	0.0029	0.956944	0.0010	0.0005027529
ss65207691	*rs43290746*	2	21.649588	0.0817	4.2148	0.040446	0.0686	0.0102935586
ss61508935	*rs41600403*	2	21.706967	0.9248	5.7739	0.016527	0.0827	0.0115018036
	rs29024697	2	21.744159	0.0824	3.1670	0.075577	0.0594	0.0089739358
ss61502087	*rs41603101*	2	21.781367	0.0824	5.0404	0.025076	0.0754	0.0114067941
ss63721586	*rs42322784*	2	21.811132	0.3639	0.4324	0.511051	0.0125	0.0057733472
ss117963571		2	21.841023	0.2894	1.2142	0.270875	0.0222	0.0091366893
ss61474306	*rs41568316*	2	21.873143	0.8446	0.7364	0.391124	0.0222	0.0058162557
ss64746000	*rs42841932*	2	21.894376	0.7794	1.8107	0.178858	0.0299	0.0102927001
ss86333971		2	21.928175	0.9155	0.3263	0.568056	0.0190	0.0029481107
ss86340937		2	21.961007	0.1719	1.6573	0.198391	0.0323	0.0091828582
ss63721415	*rs42322327*	2	21.99018	0.5681	0.8790	0.348796	0.0175	0.0085949784
ss86307188		2	22.01527	0.5501	3.7574	0.052980	0.0363	0.0179722419
ss86336863		2	22.046641	0.3553	2.1556	0.142504	0.0299	0.0137089559
ss86317068		2	22.068494	0.4792	5.5176	0.019105	0.0449	0.0224024032
ss64800080	*rs42897507*	2	22.130368	0.1905	1.3902	0.238774	0.0277	0.0085417736
ss86312183		2	22.150542	0.0874	2.7299	0.098937	0.0545	0.0086938934
ss61520389		2	22.200874	0.3288	0.0205	0.886204	0.0029	0.0012996409
ss64735921	*rs42835857*	2	22.230389	0.1067	0.3053	0.580736	0.0165	0.0031421741
ss61541279	*rs41642316*	2	22.282895	0.8632	0.1879	0.664780	0.0116	0.0027318714
ss65209560	*rs43300500*	2	22.308433	0.5186	4.8783	0.027522	0.0419	0.0209369784

ss65213552	*rs43300345*	2	22.348417	0.2077	8.1473	0.004441		0.0636	0.0209192016
ss61541278	*rs41642315*	2	22.381259	0.5437	4.6812	0.030834		0.0413	0.0204938631
ss86290451		2	22.556139	0.3854	0.2714	0.602558		0.0101	0.0047774170
ss61541266	*rs41642303*	2	22.690073	0.5494	2.8040	0.094478		0.0323	0.0159904290
ss86305964		2	22.727721	0.4105	1.9441	0.163667		0.0267	0.0129404119
ss105258160		2	22.753496	0.5458	1.3327	0.248716		0.0218	0.0107968920
ss65209073	*rs29004009*	2	22.88672	0.5587	2.9442	0.086632		0.0327	0.0161112818
ss61483685	*rs41580951*	2	22.937267	0.3259	3.4727	0.062809		0.0383	0.0168331091
ss65208957	*rs43294104*	2	22.966997	0.7973	1.7452	0.186911		0.0308	0.0099437411
ss65208903	*rs43294050*	2	22.987198	0.1791	1.4621	0.227014		0.0293	0.0086192197
ss65208950	*rs43294097*	2	23.009489	0.5924	4.1150	0.042885		0.0382	0.0184370360
ss65208826	*rs43292973*	2	23.064597	0.3897	0.6907	0.406217		0.0156	0.0074015662
ss65208801	*rs43292948*	2	23.10706	0.8904	0.4521	0.501539		0.0205	0.0040032609
ss61541260	*rs41642297*	2	23.235237	0.7185	0.6728	0.412343		0.0167	0.0067641147
ss65208509	*rs43298451*	2	23.310209	0.3102	0.5908	0.442377		0.0153	0.0065395465
ss65208476	*rs43297418*	2	23.352853	0.6941	0.2955	0.586915		0.0108	0.0045793849
ss86289528		2	23.474879	0.8546	0.1566	0.692417		0.0103	0.0025676041
ss64711372	*rs42806471*	2	23.792091	0.6905	0.0304	0.861549		0.0037	0.0015686147
ss86335562		2	23.822655	0.3768	2.6145	0.106343		0.0315	0.0147775633
ss86320567		2	23.87754	0.6160	0.0051	0.942871		0.0013	0.0006363306
ss62469160	*rs41887008*	2	23.937018	0.7665	0.0623	0.802984		0.0056	0.0019874106
ss64485420	*rs42660260*	2	23.987983	0.2228	0.0670	0.795834		0.0058	0.0020234671
ss64485350	*rs42659225*	2	24.015633	0.2206	0.0895	0.764960		0.0068	0.0023330001
ss64485256	*rs42659179*	2	24.041804	0.8560	0.3370	0.561774		0.0159	0.0039077970
ss64485136	*rs42658419*	2	24.063252	0.7135	6.1103	0.013678		0.0511	0.0208905023
	rs29026677	2	24.11181	0.1597	1.9166	0.166670		0.0347	0.0093176210
ss65209669	*rs43301308*	2	24.138052	0.4004	1.3438	0.246764		0.0218	0.0104496969
ss65209704	*rs43291849*	2	24.230371	0.4534	0.0888	0.765789		0.0054	0.0026947043
ss65209788	*rs43292533*	2	24.25089	0.3825	1.0180	0.313334		0.0187	0.0088503773
ss61483691	*rs41580957*	2	24.292139	0.1999	1.4785	0.224427		0.0284	0.0090868397
ss65210043	*rs43293986*	2	24.360937	0.4298	0.9752	0.323739		0.0179	0.0087929553
ss86305188		2	24.386458	0.6999	7.8778	0.005144		0.0554	0.0232660643
	rs29027446	2	24.418477	0.4513	9.1311	0.002605		0.0558	0.0276450647
ss86330302		2	24.465562	0.6354	7.4966	0.006340		0.0513	0.0237593689
ss86311598		2	24.573948	0.8524	2.6295	0.105348		0.0412	0.0103569469
ss86338364		2	24.61113	0.1461	1.9694	0.160957		0.0361	0.0090055834
ss86339265		2	24.659199	0.3811	4.9765	0.026012		0.0439	0.0207272731
ss61508942	*rs41600410*	2	24.691213	0.1540	1.3513	0.245446		0.0294	0.0076535078
	rs29021257	2	24.794614	0.2550	0.0262	0.871405		0.0034	0.0012763175
ss61541315	*rs41643150*	2	24.932967	0.1332	1.2781	0.258648		0.0299	0.0068950580
ss65211121	*rs43295863*	2	24.962747	0.3367	0.7716	0.380022		0.0175	0.0078096129
ss61541313	*rs41643148*	2	24.991102	0.6418	0.0381	0.845258		0.0039	0.0017796742
ss65211028	*rs43294770*	2	25.014442	0.7894	3.2870	0.070259		0.0426	0.0141661449
ss86300025		2	25.036141	0.1755	5.3150	0.021436		0.0560	0.0162157222
ss86325076		2	25.093658	0.1483	2.0021	0.157526		0.0366	0.0092343088
ss86329440		2	25.13038	0.7507	1.9329	0.164886		0.0306	0.0114431238
ss86291247		2	25.174431	0.0860	0.1903	0.662818		0.0146	0.0022953077

	rs29015710	2	25.244145	0.4900	0.2473	0.619163	0.0091	0.0045467939
	rs29012418	2	25.316609	0.4635	3.3487	0.067688	0.0339	0.0168634356
ss65211714	*rs43301655*	2	25.340314	0.5265	0.3271	0.567544	0.0108	0.0053709165
ss86287045		2	25.364609	0.4327	1.1595	0.281937	0.0197	0.0096480196
ss65211869	*rs43293111*	2	25.388682	0.5415	1.4616	0.227081	0.0219	0.0108715104
ss65211894	*rs43294136*	2	25.435739	0.3209	12.2614	0.000492	0.0690	0.0300775344
ss65211913	*rs43294155*	2	25.458878	0.3109	8.1413	0.004455	0.0563	0.0241128676
ss65211973	*rs43294215*	2	25.49413	0.6905	2.5703	0.109340	0.0321	0.0137342071
ss117966468		2	25.532653	0.6010	1.4177	0.234186	0.0229	0.0110052826
ss86327619		2	25.572322	0.3009	4.4482	0.035294	0.0427	0.0179712137
ss65208041	*rs43293483*	2	25.599756	0.6540	0.9695	0.325152	0.0194	0.0087632252
ss61541246	*rs41642283*	2	25.634966	0.2958	2.3398	0.126558	0.0307	0.0127816389
ss86337755		2	25.680508	0.6948	2.8714	0.090613	0.0339	0.0143854892
ss65208171	*rs43294413*	2	25.702375	0.3345	8.8877	0.002971	0.0567	0.0252628555
ss61483678	*rs41580944*	2	25.734542	0.6218	1.4367	0.231081	0.0229	0.0107657596
ss86315213		2	25.784986	0.6168	1.6384	0.200978	0.0244	0.0115471839
ss61541251	*rs41642288*	2	25.816052	0.1196	0.1044	0.746698	0.0091	0.0019267889
	rs29012194	2	26.095593	0.1920	0.2049	0.650970	0.0108	0.0033605458
ss65210115	*rs43294658*	2	26.175812	0.4821	15.6217	0.000085	0.0737	0.0367907148
ss61483692	*rs41580958*	2	26.217629	0.3145	13.7760	0.000222	0.0715	0.0308145810
ss86331567		2	26.258342	0.8417	7.5078	0.006301	0.0659	0.0175544869
ss117966811		2	26.292765	0.9234	5.1396	0.023692	0.0767	0.0108631464
ss86313796		2	26.379855	0.8360	0.0372	0.847199	0.0048	0.0013148458
ss61483697	*rs41580963*	2	26.427913	0.3840	5.2981	0.021643	0.0426	0.0201670161
ss105261280		2	26.456565	0.4628	8.9132	0.002930	0.0551	0.0274075124
ss86291474		2	26.477012	0.5365	8.6629	0.003356	0.0543	0.0269823213
ss61541296	*rs41643133*	2	26.504946	0.2958	4.0007	0.045870	0.0404	0.0168276392
ss61523284	*rs41619956*	2	26.529986	0.4097	0.0067	0.935006	0.0016	0.0007610866
ss86297343		2	26.58003	0.3754	1.6357	0.201341	0.0250	0.0117101164
ss86338398		2	26.61821	0.3052	0.0533	0.817421	0.0045	0.0019170794
ss65210741	*rs43292086*	2	26.64192	0.3668	1.3650	0.243072	0.0221	0.0102751782
ss117966755		2	26.67694	0.7679	0.5028	0.478500	0.0157	0.0055894971
ss86310366		2	26.853841	0.2393	0.0497	0.823633	0.0050	0.0018055171
ss61507709	*rs41608694*	2	26.886068	0.5630	0.1446	0.703840	0.0073	0.0035814633
ss65212160	*rs43296401*	2	26.936941	0.1497	4.3028	0.038418	0.0558	0.0142150253
ss86317732		2	26.957453	0.7908	4.4148	0.035987	0.0472	0.0156070781
ss61541359	*rs41643194*	2	26.97935	0.1605	14.3471	0.000165	0.0923	0.0248559073
ss86307276		2	27.008381	0.7744	3.3120	0.069206	0.0405	0.0141575039
ss61541422	*rs41644055*	2	27.031147	0.2672	8.2324	0.004240	0.0611	0.0239459076
ss65215842	*rs43298653*	2	27.053902	0.7830	14.6232	0.000143	0.0841	0.0285741752
ss65215713	*rs43305321*	2	27.107785	0.5079	0.3941	0.530381	0.0117	0.0058532529
ss61541420	*rs41644053*	2	27.178252	0.3582	2.4436	0.118457	0.0302	0.0138633255
ss65215672	*rs43305280*	2	27.20535	0.1383	6.7981	0.009321	0.0691	0.0164613449
ss117966866		2	27.273587	0.2221	7.7525	0.005509	0.0611	0.0211111099
ss46526812	*rs41257263*	2	27.338225	0.0795	3.2028	0.073945	0.0599	0.0087672118
ss61518691	*rs41617243*	2	27.529203	0.1368	10.6928	0.001129	0.0878	0.0207435003
ss86293077		2	27.568718	0.2586	3.0831	0.079549	0.0374	0.0143342571

ss65213153	*rs43296564*	2	27.651438	0.0953	2.9576	0.085922	0.0564	0.0097160300
ss65213428	*rs43299131*	2	27.711954	0.4456	1.5499	0.213571	0.0238	0.0117579173
ss86285088		2	27.785383	0.4971	0.3775	0.539147	0.0113	0.0056420335
ss86281772		2	27.900411	0.2937	0.0148	0.903326	0.0025	0.0010458712
ss86289906		2	27.939192	0.8295	1.4745	0.225054	0.0293	0.0082749682
ss64741713	*rs42835390*	2	27.967802	0.4563	0.1028	0.748551	0.0059	0.0029350898
ss86288139		2	28.025834	0.4076	0.1616	0.687848	0.0075	0.0036108489
ss117963601		2	28.058809	0.4191	0.6307	0.427369	0.0149	0.0072726462
ss61467876	*rs41572996*	2	28.122053	0.3188	0.3633	0.546871	0.0123	0.0053361753
ss65215502	*rs43303711*	2	28.144488	0.7772	0.0953	0.757679	0.0070	0.0024352370
ss65215496	*rs43303705*	2	28.201467	0.6225	0.5061	0.477067	0.0139	0.0065360062
ss61467875	*rs41572995*	2	28.252001	0.0924	4.3529	0.037309	0.0650	0.0108986973
ss65215341	*rs43302750*	2	28.29023	0.7264	10.4477	0.001286	0.0686	0.0272761472
	rs29014793	2	28.328099	0.3574	1.8524	0.173945	0.0270	0.0124066092
ss86324899		2	28.387866	0.5888	7.9480	0.004951	0.0540	0.0261531000
ss65215255	*rs43301566*	2	28.418146	0.6383	10.9387	0.000990	0.0658	0.0303674265
ss61483758	*rs41581023*	2	28.584396	0.8138	2.3675	0.124339	0.0374	0.0113261869
ss65215082	*rs43299393*	2	28.606629	0.6347	11.4745	0.000745	0.0669	0.0310119077
ss65215020	*rs43299331*	2	28.66809	0.5000	0.2367	0.626734	0.0090	0.0044776607
ss86278808		2	28.699648	0.4735	4.2524	0.039566	0.0375	0.0187183563
ss61502157	*rs41604269*	2	28.746311	0.2536	2.5814	0.108581	0.0352	0.0133105065
ss86307554		2	28.77827	0.2235	0.3843	0.535535	0.0137	0.0047540793
ss61541405	*rs41644038*	2	28.804682	0.7600	0.0969	0.755700	0.0066	0.0024184329
ss65214629	*rs43302839*	2	28.833939	0.1504	1.5052	0.220295	0.0319	0.0081528889
ss86293810		2	28.854903	0.3044	1.2918	0.256116	0.0229	0.0096777087
ss65214588	*rs43302298*	2	28.89004	0.5451	2.8813	0.090061	0.0325	0.0160999198
ss61541402	*rs41644035*	2	28.922494	0.6590	1.1162	0.291108	0.0207	0.0093203998
ss86307798		2	28.976014	0.6984	0.6391	0.424318	0.0165	0.0069368520
ss86300937		2	29.014103	0.0867	0.5234	0.469620	0.0237	0.0037558950
ss86336663		2	29.046281	0.2271	0.2187	0.640182	0.0104	0.0036570470
ss61541400	*rs41644033*	2	29.070028	0.7185	10.7270	0.001108	0.0656	0.0265477921
ss61483751	*rs41581016*	2	29.103512	0.5809	7.2677	0.007190	0.0495	0.0240980262
ss86338536		2	29.154674	0.5888	4.8016	0.028764	0.0412	0.0199508070
	rs29026016	2	29.192133	0.9298	0.1145	0.735132	0.0122	0.0015975226
ss65214164	*rs43299775*	2	29.219372	0.1948	9.4702	0.002170	0.0711	0.0223136889
	rs29026473	2	29.283744	0.7414	1.5646	0.211419	0.0261	0.0099965594
ss61521409	*rs41618615*	2	29.325055	0.5086	0.0022	0.962200	0.0009	0.0004321998
ss63638938	*rs42296575*	2	29.369337	0.2163	0.7950	0.372915	0.0196	0.0066481224
ss86328449		2	29.413883	0.4771	0.0116	0.914226	0.0020	0.0009801396
ss63639628	*rs42297800*	2	29.438357	0.6039	1.8509	0.174116	0.0261	0.0124755274
	rs29022835	2	29.478444	0.2328	2.9438	0.086654	0.0385	0.0137463226
ss105258895		2	29.540904	0.1640	3.2191	0.073217	0.0453	0.0124340032
ss61496623	*rs41591276*	2	29.597896	0.8238	1.2796	0.258371	0.0272	0.0078852221
ss61568170	*rs41662072*	2	29.634458	0.4405	0.2125	0.644922	0.0087	0.0043061962
ss61496625	*rs41591278*	2	29.661843	0.3589	10.2675	0.001416	0.0620	0.0285412936
	rs29026680	2	29.692101	0.4076	0.0827	0.773733	0.0054	0.0026078218
ss86308249		2	29.767064	0.4864	0.0278	0.867660	0.0031	0.0015289402

ss105257824		2	29.78782	0.5473	0.4222	0.516051	0.0121	0.0059861471
ss64384861	*rs42610185*	2	29.815007	0.9241	8.0319	0.004729	0.1011	0.0141908786
ss61496970	*rs41593604*	2	29.844102	0.2128	1.2736	0.259476	0.0261	0.0087274231
ss64384819	*rs42610164*	2	29.871688	0.2221	1.1845	0.276822	0.0238	0.0082220157
ss61515566	*rs41615219*	2	29.943217	0.1719	5.6363	0.017862	0.0560	0.0159397955
ss61568173	*rs41662075*	2	29.975865	0.6741	3.4163	0.064980	0.0362	0.0159012862
ss86330363		2	30.004094	0.6311	0.0344	0.852817	0.0037	0.0017121787
ss86334404		2	30.041448	0.3653	0.0000	0.997371	0.0001	0.0000303604
ss61564302	*rs41660173*	2	30.084206	0.3625	8.4108	0.003848	0.0572	0.0264226363
ss38331592	*rs29025699*	2	30.171899	0.1891	0.1493	0.699322	0.0092	0.0028099360
ss65003050	*rs43095753*	2	30.197475	0.3395	4.2228	0.040256	0.0392	0.0175878551
ss61532599	*rs41626505*	2	30.223011	0.5380	1.3422	0.247039	0.0218	0.0108568155
ss61479423	*rs41578486*	2	30.25798	0.8059	0.0419	0.837841	0.0048	0.0015037970
ss86335951		2	30.289849	0.4484	0.0828	0.773658	0.0055	0.0027312653
ss86283762		2	30.317827	0.3653	2.5832	0.108455	0.0301	0.0139720167
ss61500825	*rs41600874*	2	30.353152	0.5387	3.7639	0.052773	0.0368	0.0182873816
ss61467123	*rs41565554*	2	30.388491	0.4542	1.6640	0.197493	0.0244	0.0120973626
ss61467124	*rs41565555*	2	30.481243	0.6569	3.7761	0.052392	0.0375	0.0168847575
ss64994397	*rs43084543*	2	30.5056	0.4620	8.3077	0.004069	0.0536	0.0266686147
	rs29016992	2	30.526227	0.5337	16.7281	0.000048	0.0747	0.0371856731
ss105257815		2	30.551139	0.5351	3.4920	0.062085	0.0343	0.0170647263
ss64834071	*rs42926930*	2	30.598831	0.7514	13.4735	0.000260	0.0781	0.0291924162
ss61507260	*rs41604658*	2	30.624251	0.1705	7.0668	0.008033	0.0643	0.0181921842
ss63885098	*rs41807745*	2	30.699457	0.2615	0.3107	0.577458	0.0123	0.0047453239
ss105261285		2	30.758551	0.3682	1.2352	0.266773	0.0220	0.0102575308
ss63365699	*rs42209697*	2	30.784775	0.3539	2.5727	0.109174	0.0309	0.0141109805
ss86324982		2	30.809232	0.5301	0.1163	0.733166	0.0063	0.0031403333
ss86341117		2	30.838734	0.5029	0.0000	0.999365	0.0000	0.0000077326
ss86292491		2	30.863718	0.4370	1.7099	0.191433	0.0247	0.0121294120
ss86294482		2	30.892143	0.5064	0.0021	0.963391	0.0008	0.0004174474
ss61483766	*rs41581931*	2	30.9266	0.1970	2.8230	0.093373	0.0374	0.0118227503
ss86302172		2	30.955235	0.1963	2.6859	0.101695	0.0366	0.0115553923
ss105255664		2	31.01687	0.2830	11.3836	0.000782	0.0701	0.0284556041
ss117966494		2	31.050611	0.3109	31.2752	0.000000	0.1132	0.0485100540
ss105254260		2	31.079489	0.6870	33.2892	0.000000	0.1164	0.0500561632
	rs29012101	2	31.10609	0.4112	0.0187	0.891202	0.0027	0.0012936872
ss105256630		2	31.145929	0.2128	0.9711	0.324755	0.0224	0.0075021099
ss64171473	*rs42506054*	2	31.211043	0.1999	4.6592	0.031228	0.0500	0.0160016773
ss105257313		2	31.256176	0.5107	0.2325	0.629839	0.0088	0.0044108083
ss105258632		2	31.317703	0.1927	31.6441	0.000000	0.1311	0.0408010928
ss86333517		2	31.350934	0.5122	2.9913	0.084157	0.0323	0.0161598451
ss61563147	*rs41659239*	2	31.376042	0.2493	0.0385	0.844520	0.0043	0.0016240177
ss105260598		2	31.405766	0.2385	0.2775	0.598509	0.0115	0.0041733838
ss61520497	*rs41614621*	2	31.430145	0.6891	4.6030	0.032261	0.0424	0.0181799610
ss105259321		2	31.453452	0.0666	6.1557	0.013334	0.0913	0.0113513191
ss86291515		2	31.477764	0.0895	0.0225	0.880736	0.0048	0.0007860221
ss105256040		2	31.559155	0.7830	1.5499	0.213573	0.0272	0.0092277063

ss105257401		2	31.583468	0.4799	0.4461	0.504418	0.0130	0.0064717096
ss105258029		2	31.607615	0.8138	2.7959	0.094956	0.0399	0.0121033452
ss117966659		2	31.628569	0.4656	0.8549	0.355491	0.0175	0.0086973674
ss105258055		2	31.652459	0.1941	2.7202	0.099534	0.0390	0.0121882927
ss61569254	*rs41666338*	2	31.721776	0.6519	1.2891	0.256605	0.0225	0.0101902208
ss117966886		2	31.773764	0.3181	1.1603	0.281767	0.0220	0.0095641563
ss105256723		2	31.875407	0.6884	0.0001	0.992235	0.0002	0.0000865209
ss105258734		2	31.936465	0.3832	14.7728	0.000132	0.0732	0.0346215189
ss105261392		2	31.965974	0.9212	3.5016	0.061729	0.0628	0.0091147863
ss105262031		2	31.996369	0.7292	12.9084	0.000350	0.0750	0.0296347790
ss105257406		2	32.026428	0.2966	4.4084	0.036123	0.0422	0.0176151852
ss105257448		2	32.057606	0.8281	5.4993	0.019304	0.0585	0.0166471755
ss117966892		2	32.088154	0.5186	5.9723	0.014780	0.0451	0.0225413775
ss61498830	*rs43707331*	2	32.14257	0.6920	2.6217	0.105863	0.0322	0.0137435530
ss105299964		2	32.187768	0.5716	6.1444	0.013418	0.0475	0.0232432749
ss105260032		2	32.21109	0.9191	0.2433	0.621956	0.0157	0.0023410714
ss86341634		2	32.232984	0.4706	0.0372	0.847033	0.0036	0.0017817492
ss86338190		2	32.273417	0.5287	0.0238	0.877365	0.0029	0.0014273181
ss105258101		2	32.358778	0.0709	0.8934	0.344896	0.0340	0.0044869325
ss117966592		2	32.411811	0.6454	6.0138	0.014439	0.0466	0.0213162297
ss64961112	*rs43052351*	2	32.44574	0.6705	12.6270	0.000406	0.0688	0.0304186316
ss61541435	*rs41644068*	2	32.478661	0.8324	0.0645	0.799592	0.0062	0.0017180149
	rs29026993	2	32.515385	0.7077	3.2996	0.069725	0.0376	0.0155439598
ss61483774	*rs41581939*	2	32.54858	0.1841	4.2056	0.040665	0.0494	0.0148296552
ss65216712	*rs43303920*	2	32.592374	0.8030	5.2782	0.021890	0.0522	0.0165223007
ss64542482	*rs42687066*	2	32.61589	0.7514	5.9792	0.014722	0.0518	0.0193671222
ss86310615		2	32.6403	0.5580	2.4931	0.114801	0.0291	0.0143528919
ss61514452	*rs41611823*	2	32.676654	0.3983	0.2233	0.636650	0.0089	0.0042680424
ss86292006		2	32.738673	0.9219	7.7566	0.005497	0.0934	0.0134515566
ss65217634	*rs43306643*	2	32.81525	0.3266	0.0047	0.945592	0.0013	0.0005650760
ss65217624	*rs43306633*	2	32.87243	0.5716	8.8812	0.002982	0.0569	0.0278608318
ss86302337		2	32.91673	0.1991	5.4454	0.019904	0.0551	0.0175785367
ss65217606	*rs43305615*	2	33.00097	0.5659	0.8884	0.346238	0.0176	0.0086544490
ss61541509	*rs41645041*	2	33.028756	0.3983	0.6433	0.422775	0.0153	0.0073406753
ss65217455	*rs43304664*	2	33.054899	0.4155	6.9005	0.008807	0.0500	0.0243029036
ss65217401	*rs43304010*	2	33.077598	0.4133	6.0496	0.014152	0.0467	0.0226650591
ss65217432	*rs43304641*	2	33.101143	0.3281	0.0272	0.869111	0.0034	0.0014808695
ss86283959		2	33.159313	0.9155	0.0170	0.896157	0.0043	0.0006611069
ss86308417		2	33.211487	0.3152	0.0003	0.986476	0.0003	0.0001476539
ss65217215	*rs43302224*	2	33.250946	0.6024	1.6857	0.194600	0.0246	0.0117833431
ss105274425		2	33.300874	0.4964	8.0748	0.004620	0.0535	0.0267605311
ss61483801	*rs41581963*	2	33.321372	0.4133	1.5212	0.217856	0.0234	0.0113588211
ss86279549		2	33.429413	0.1526	2.0454	0.153111	0.0377	0.0097464908
ss86281776		2	33.501109	0.8732	1.5156	0.218696	0.0348	0.0077072335
ss64498068	*rs42667660*	2	33.538423	0.1426	2.2904	0.130630	0.0411	0.0100418578
ss61483780	*rs41581945*	2	33.65565	0.0960	0.2674	0.605250	0.0166	0.0028841051
ss65216897	*rs43298806*	2	33.676206	0.8782	0.0398	0.842002	0.0056	0.0012051054

ss61483779	*rs41581944*	2	33.698675	0.2221	12.0117	0.000561	0.0758	0.0261815041
ss86339610		2	33.743659	0.4219	4.7125	0.030281	0.0414	0.0201810116
ss61541440	*rs41644073*	2	33.790226	0.5201	0.5995	0.439022	0.0149	0.0074442936
ss61541439	*rs41644072*	2	33.853647	0.4334	0.6824	0.409050	0.0159	0.0077882409
ss65216810	*rs43304414*	2	33.883714	0.2615	8.4095	0.003850	0.0624	0.0241002258
ss65216792	*rs43304396*	2	33.933757	0.7880	11.9185	0.000589	0.0777	0.0259654001
ss65218732	*rs43309340*	2	33.966779	0.2428	2.2862	0.130982	0.0334	0.0122776297
ss86297556		2	34.013813	0.0552	2.0163	0.156066	0.0571	0.0059481588
ss86301852		2	34.068129	0.5731	0.4803	0.488531	0.0128	0.0062443028
ss65218520	*rs43306128*	2	34.122324	0.3080	0.1245	0.724269	0.0071	0.0030084852
ss65218437	*rs43306045*	2	34.252811	0.3152	0.0030	0.956025	0.0011	0.0004720527
ss65218430	*rs43306038*	2	34.281719	0.2521	0.0100	0.920190	0.0022	0.0008137154
ss65218344	*rs43304953*	2	34.345939	0.6619	0.0673	0.795376	0.0051	0.0022723708
ss65218311	*rs43304120*	2	34.373169	0.1669	0.7797	0.377541	0.0222	0.0061743909
ss86321229		2	34.401741	0.3911	0.9383	0.333047	0.0184	0.0087848825
ss105272036		2	34.438427	0.3868	0.5225	0.470001	0.0139	0.0066165428
ss65217994	*rs43302003*	2	34.469833	0.8395	0.3530	0.552610	0.0151	0.0040597177
ss65217917	*rs43301226*	2	34.533425	0.5344	0.1316	0.716920	0.0066	0.0032940964
ss65217889	*rs43301198*	2	34.61541	0.7221	3.5864	0.058669	0.0392	0.0157163187
ss86337120		2	34.720067	0.3052	0.0722	0.788308	0.0055	0.0023285777
ss86296900		2	34.817149	0.2744	1.6164	0.204012	0.0264	0.0105251869
ss65217854	*rs43301163*	2	34.839746	0.8797	5.6528	0.017697	0.0676	0.0143195467
ss86299141		2	35.010648	0.4062	10.6111	0.001179	0.0618	0.0298044802
ss65212586	*rs43300008*	2	35.031836	0.4592	12.2890	0.000485	0.0660	0.0327741173
ss86296658		2	35.081505	0.3567	3.7319	0.053788	0.0356	0.0163443836
ss65212715	*rs43302034*	2	35.149654	0.8897	1.6016	0.206093	0.0362	0.0071039715
ss65212773	*rs43302092*	2	35.183998	0.0845	2.1999	0.138472	0.0493	0.0076363794
ss65212830	*rs43293152*	2	35.260055	0.4921	2.1146	0.146353	0.0271	0.0135664394
ss65212839	*rs43293161*	2	35.323344	0.1418	0.7020	0.402408	0.0224	0.0054512259
ss64913299	*rs43006513*	2	35.394267	0.6218	2.0334	0.154323	0.0267	0.0125522251
ss86335932		2	35.416305	0.5652	1.9538	0.162618	0.0258	0.0126712069
ss65216730	*rs43304338*	2	35.444552	0.5265	1.2520	0.263557	0.0208	0.0103766570
ss86294940		2	35.515941	0.3460	1.4518	0.228648	0.0236	0.0106636917
ss117963618		2	35.55283	0.3553	4.1843	0.041174	0.0386	0.0176876317
ss117966604		2	35.588096	0.6870	0.0182	0.892775	0.0027	0.0011439035
ss86294529		2	35.609481	0.2894	1.3795	0.240591	0.0242	0.0099716765
ss63140527	*rs42128371*	2	35.711188	0.6805	6.0986	0.013768	0.0484	0.0210417662
ss61487800	*rs41586265*	2	35.740866	0.5172	9.0645	0.002700	0.0549	0.0273993284
ss105258601		2	35.949562	0.8596	0.4592	0.498203	0.0182	0.0043821951
ss86332556		2	36.035874	0.8496	0.1698	0.680453	0.0109	0.0027739001
ss61496414	*rs41598664*	2	36.074928	0.1533	0.2258	0.634771	0.0125	0.0032336829
ss61523952	*rs41624307*	2	36.153525	0.0845	0.0011	0.973711	0.0011	0.0001672415
ss65213533	*rs43300334*	2	36.295801	0.7894	0.0025	0.959794	0.0012	0.0003853653
ss61541382	*rs41643216*	2	36.350582	0.8832	1.4349	0.231372	0.0338	0.0069650041
	rs29025790	2	36.426982	0.7615	2.2810	0.131421	0.0328	0.0119161641
ss61478413	*rs41575812*	2	36.736942	0.8804	0.1592	0.689987	0.0117	0.0024733239
ss61466169	*rs41565930*	2	36.757251	0.8195	1.6098	0.204947	0.0304	0.0090029682

ss65419218	*rs43501901*	2	36.905025	0.1003	0.5224	0.470063	0.0218	0.0039320937
ss65419190	*rs43501873*	2	36.929856	0.8832	0.6857	0.407928	0.0236	0.0048763717
ss63890854	*rs41867312*	2	36.968195	0.8961	0.5041	0.477959	0.0210	0.0039140472
ss86317455		2	37.040275	0.7772	0.0009	0.975967	0.0007	0.0002393850
ss105264785		2	37.078459	0.1297	0.1812	0.670472	0.0121	0.0027347187
ss61565335	*rs41660090*	2	37.135212	0.8367	1.0290	0.310742	0.0262	0.0071580073
ss86319461		2	37.219523	0.0981	0.0375	0.846525	0.0060	0.0010589820
ss61495227	*rs41594809*	2	37.284607	0.5681	4.8309	0.028282	0.0413	0.0202564276
ss86339792		2	37.369468	0.6125	1.8318	0.176359	0.0263	0.0124665143
ss117966565		2	37.393308	0.5344	2.0715	0.150525	0.0284	0.0141260047
ss86289191		2	37.469796	0.7400	2.7616	0.097004	0.0351	0.0135025424
ss86339949		2	37.532364	0.8775	1.6329	0.201725	0.0365	0.0078400967
ss61508981	*rs41601048*	2	37.581123	0.6597	0.0522	0.819276	0.0044	0.0019916474
ss61500248	*rs41598403*	2	37.659599	0.1640	0.0115	0.914548	0.0027	0.0007318678
ss86331593		2	37.929961	0.3095	0.7267	0.394247	0.0168	0.0071670769
ss105258556		2	38.165768	0.1655	0.7725	0.379754	0.0220	0.0060842857
	rs29018633	2	38.241364	0.1948	0.2608	0.609712	0.0120	0.0037736473
ss61546103	*rs41640750*	2	38.338458	0.8066	0.0468	0.828861	0.0051	0.0015898027
ss117966577		2	38.505055	0.0738	3.2140	0.073445	0.0636	0.0086893480
ss61541195	*rs41642232*	2	38.53084	0.9269	3.1882	0.074608	0.0636	0.0086089776
ss86273461		2	38.577906	0.0580	1.7970	0.180512	0.0529	0.0057852689
	rs29019574	2	38.762005	0.9119	0.0518	0.819982	0.0075	0.0012048126
	rs29024139	2	38.842037	0.3990	3.3554	0.067414	0.0337	0.0161602257
ss86338793		2	38.881823	0.4878	1.2387	0.266108	0.0205	0.0102203489
ss86288122		2	39.03247	0.8682	0.0177	0.894145	0.0036	0.0008351436
ss61562462	*rs41661761*	2	39.09875	0.9076	1.4831	0.223707	0.0393	0.0065954614
ss86320881		2	39.158762	0.8617	0.0033	0.954060	0.0016	0.0003695150
ss86341791		2	39.199348	0.0666	1.0309	0.310308	0.0380	0.0047197272
ss61528537	*rs41626579*	2	39.301914	0.9119	1.6140	0.204349	0.0422	0.0067812943
ss61524420	*rs41620662*	2	39.59849	0.1956	0.8141	0.367223	0.0209	0.0065623201
ss86320022		2	39.626553	0.4219	1.4102	0.235429	0.0218	0.0106114015
ss61483840	*rs41582002*	2	39.713187	0.8582	0.0299	0.862694	0.0047	0.0011421161
ss86309040		2	39.733383	0.0530	0.1389	0.709500	0.0153	0.0015368744
ss61541579	*rs41645110*	2	39.822574	0.5630	0.0015	0.968704	0.0007	0.0003606076
ss86292818		2	39.844697	0.0924	1.0819	0.298639	0.0333	0.0055892254
ss86291412		2	39.885395	0.2213	0.4123	0.521006	0.0142	0.0048847373
ss117963641		2	39.991756	0.2901	0.1572	0.691894	0.0080	0.0032947831
ss61541543	*rs41645075*	2	40.382567	0.3130	2.0433	0.153324	0.0279	0.0119920584
ss61541555	*rs41645086*	2	40.405109	0.6433	0.8593	0.354267	0.0178	0.0081624002
	rs29015860	2	40.439269	0.3689	0.8684	0.351716	0.0178	0.0082713963
ss86295646		2	40.490963	0.9234	0.0566	0.811974	0.0083	0.0011680613
ss65220196	*rs43306402*	2	40.514461	0.0695	0.8074	0.369187	0.0327	0.0042298118
ss86297730		2	40.543041	0.0838	1.0876	0.297374	0.0353	0.0054234337
ss117966773		2	40.671382	0.4577	0.0664	0.796670	0.0048	0.0023645223
ss86290397		2	40.696798	0.5380	1.7057	0.191973	0.0241	0.0119683827
ss105255700		2	40.726084	0.4549	0.0249	0.874591	0.0029	0.0014443749
ss86333220		2	40.796451	0.5938	0.8406	0.359531	0.0174	0.0083940971

ss86341382		2	40.959609	0.4606	6.8879	0.008868		0.0474	0.0235531276
ss61473860	*rs41574370*	2	41.012741	0.6476	15.5495	0.000089		0.0767	0.0350287562
ss86335242		2	41.080035	0.4076	7.4464	0.006517		0.0507	0.0244817850
ss63382353	*rs42211818*	2	41.147383	0.4062	4.0766	0.043863		0.0373	0.0180030664
ss86336820		2	41.304216	0.5695	3.6344	0.057010		0.0355	0.0174309560
ss117966884		2	41.325781	0.6640	2.4361	0.119027		0.0298	0.0133121765
	rs29010162	2	41.474655	0.3087	0.4432	0.505815		0.0134	0.0057072113
ss63382271	*rs42211777*	2	41.630762	0.6726	0.1207	0.728373		0.0069	0.0030597441
ss63382243	*rs42211763*	2	41.656122	0.1440	3.3942	0.065853		0.0481	0.0118543149
ss63382181	*rs42211732*	2	41.709421	0.5072	0.5269	0.468161		0.0133	0.0066273558
ss86301102		2	41.832027	0.3338	2.6479	0.104143		0.0320	0.0142407452
ss86281606		2	41.858808	0.4004	1.8636	0.172653		0.0254	0.0121769715
ss105270603		2	41.97186	0.8095	1.3539	0.244996		0.0272	0.0084055064
ss61519017	*rs41619655*	2	42.004976	0.8804	0.8065	0.369478		0.0262	0.0055173645
ss38330113	*rs29021628*	2	42.132665	0.2163	0.4813	0.488070		0.0156	0.0052878145
ss61518979	*rs41618722*	2	42.191064	0.0738	1.4890	0.222778		0.0423	0.0057856177
ss64666484	*rs42760526*	2	42.512442	0.6354	1.2752	0.259186		0.0216	0.0100146711
ss86284553		2	42.543154	0.7156	1.0583	0.303967		0.0209	0.0084887326
ss86333018		2	42.669108	0.2658	0.2877	0.591851		0.0112	0.0043553552
ss61530100	*rs41631796*	2	42.701848	0.4585	2.0046	0.157267		0.0259	0.0128839544
ss64080785	*rs42466998*	2	42.770712	0.5752	4.0461	0.044658		0.0369	0.0180537355
ss61502170	*rs41604282*	2	42.831813	0.5193	1.8923	0.169386		0.0255	0.0127215878
ss64887875	*rs42977135*	2	42.900354	0.5021	12.5227	0.000429		0.0642	0.0320763008
ss64900216	*rs42997673*	2	42.936348	0.4971	12.5880	0.000414		0.0644	0.0322050846
ss86286014		2	42.963388	0.9212	12.7663	0.000377		0.1212	0.0175961197
ss105255445		2	43.026928	0.3961	1.0440	0.307237		0.0193	0.0092523367
ss65221118	*rs43305418*	2	43.072293	0.3840	0.0617	0.803885		0.0046	0.0021993120
ss61541597	*rs41636137*	2	43.141034	0.7120	1.9099	0.167415		0.0271	0.0111143340
ss61483846	*rs41582008*	2	43.164686	0.5064	0.7748	0.379052		0.0163	0.0081722256
ss105257248		2	43.184792	0.4921	0.8767	0.349436		0.0174	0.0087179699
ss61467904	*rs41573024*	2	43.208247	0.5974	0.0177	0.894251		0.0025	0.0012225193
ss61511487	*rs41612801*	2	43.229117	0.4069	0.0000	0.994380		0.0001	0.0000653744
	rs29022905	2	43.26836	0.8403	3.3480	0.067715		0.0472	0.0126604923
ss61467906	*rs41573026*	2	43.294132	0.1734	3.9365	0.047641		0.0502	0.0143882774
ss65221253	*rs43307553*	2	43.342543	0.7085	0.1694	0.680792		0.0086	0.0035556017
ss65221265	*rs43307565*	2	43.363261	0.6920	0.0209	0.885018		0.0030	0.0012910439
ss61541604	*rs41636144*	2	43.398479	0.7486	4.9877	0.025845		0.0474	0.0178330157
ss61541622	*rs41636161*	2	43.506577	0.7378	3.4513	0.063626		0.0387	0.0149892532
ss64895510	*rs42987895*	2	43.558749	0.9405	6.0906	0.013829		0.0974	0.0108923528
ss65221385	*rs43308485*	2	43.608228	0.1798	1.8547	0.173684		0.0323	0.0095228183
ss86278349		2	43.726616	0.1297	1.9544	0.162559		0.0385	0.0086951247
ss63883110	*rs41755269*	2	43.757666	0.4592	2.6461	0.104259		0.0302	0.0149979446
ss63883022	*rs41754725*	2	43.784855	0.7099	0.0368	0.847967		0.0038	0.0015701594
ss63883004	*rs41754716*	2	43.818479	0.6297	13.8492	0.000214		0.0737	0.0343758680
ss63882898	*rs41754663*	2	43.845916	0.1218	2.3509	0.125668		0.0424	0.0090697732
ss63882794	*rs41760904*	2	43.875346	0.6196	0.5688	0.450973		0.0141	0.0066616074
ss86338955		2	44.160899	0.2271	1.0001	0.317641		0.0225	0.0078977491

ss86336301		2	44.194706	0.3281	7.5475	0.006165	0.0532	0.0234650106
ss86289631		2	44.238234	0.3116	1.4829	0.223727	0.0236	0.0101301391
ss63892992	*rs41911587*	2	44.321413	0.5967	2.3570	0.125178	0.0285	0.0137366711
ss61526969	*rs41628437*	2	44.935717	0.5946	3.0041	0.083499	0.0328	0.0157918387
ss64855775	*rs42950532*	2	44.962739	0.5752	2.4628	0.117023	0.0290	0.0141632823
ss63892932	*rs41911557*	2	44.996947	0.2779	1.1092	0.292615	0.0216	0.0086526573
ss63892878	*rs41863730*	2	45.017736	0.7042	0.6278	0.428430	0.0158	0.0065940969
ss61478787	*rs41577967*	2	45.222357	0.0494	0.2741	0.600738	0.0224	0.0021067809
ss86335563		2	45.381901	0.3567	5.4201	0.020191	0.0438	0.0201137931
ss61484491		2	45.421999	0.8474	12.0711	0.000544	0.0874	0.0225921460
ss86305386		2	45.442434	0.3897	3.7273	0.053936	0.0366	0.0174142044
ss86321660		2	45.474351	0.4534	4.3499	0.037374	0.0383	0.0189862959
ss86339952		2	45.49896	0.3539	1.5668	0.211091	0.0244	0.0111695962
ss86309218		2	45.924861	0.5573	4.5118	0.034013	0.0392	0.0193505389
ss86311255		2	45.987142	0.6655	0.8764	0.349521	0.0181	0.0080601874
ss117963647		2	46.135198	0.6361	0.9023	0.342504	0.0183	0.0084591845
ss61508990	*rs41601055*	2	46.193656	0.1999	1.2133	0.271052	0.0250	0.0079953904
ss86294750		2	46.213857	0.5308	0.9790	0.322780	0.0181	0.0090045118
ss86323692		2	46.283875	0.6468	2.5072	0.113780	0.0299	0.0136400070
ss86299569		2	46.474767	0.5179	0.7322	0.392455	0.0152	0.0075928513
ss86320605		2	46.559451	0.9291	0.7013	0.402634	0.0302	0.0039781648
ss46526681	*rs41257135*	2	46.805342	0.5788	1.9356	0.164596	0.0261	0.0127433716
ss86309332		2	46.869911	0.7579	1.0195	0.312990	0.0218	0.0080018212
ss61484479	*rs41579230*	2	46.99417	0.3295	1.2190	0.269947	0.0217	0.0095870981
	rs29025329	2	46.996896	0.6669	0.9584	0.327940	0.0193	0.0085679516
ss61515489	*rs41615145*	2	47.074716	0.5752	0.3219	0.570639	0.0108	0.0052668553
ss61515487	*rs41615143*	2	47.213028	0.1282	2.5222	0.112711	0.0441	0.0098512812
ss86296474		2	47.27176	0.3302	3.6445	0.056665	0.0375	0.0166044716
ss86321147		2	47.358198	0.1289	3.2607	0.071392	0.0515	0.0115658858
ss86326871		2	47.442045	0.4463	1.0492	0.306048	0.0184	0.0091112796
ss86322096		2	47.523313	0.4420	1.5683	0.210876	0.0226	0.0111579689
	rs29021231	2	47.702697	0.7636	0.1370	0.711438	0.0081	0.0029324934
ss61483859	*rs41582021*	2	47.722699	0.1024	2.4013	0.121686	0.0478	0.0087930875
ss117966568		2	47.768673	0.3517	1.6483	0.199614	0.0245	0.0111545984
ss86298380		2	47.792562	0.7328	1.0033	0.316855	0.0213	0.0083409764
	rs29011466	2	47.797827	0.4585	4.7426	0.029760	0.0398	0.0197465692
ss117975285		2	47.870029	0.5946	1.7447	0.186983	0.0243	0.0117075892
ss105270607		2	48.403116	0.4162	0.1839	0.668162	0.0080	0.0039028234
ss61495506	*rs41597976*	2	48.427236	0.8639	1.4888	0.222810	0.0340	0.0079975763
ss117966824		2	48.495348	0.4413	1.0219	0.312414	0.0187	0.0092028226
ss86309874		2	48.557519	0.6934	0.3821	0.536673	0.0125	0.0053060506
ss61541689	*rs41636227*	2	48.587808	0.8467	2.3288	0.127451	0.0397	0.0103161507
ss86311692		2	48.616148	0.9090	0.4640	0.495973	0.0226	0.0037335000
ss86300444		2	48.720147	0.2407	3.5170	0.061161	0.0407	0.0148607925
	rs29011345	2	48.820056	0.2872	0.0006	0.980146	0.0005	0.0002084284
ss61541679	*rs41636217*	2	48.899557	0.4248	0.2150	0.643001	0.0089	0.0043366926
ss86340486		2	48.948042	0.1719	0.6952	0.404673	0.0208	0.0059114357

ss61520998	*rs41619017*	2	49.066463	0.1275	0.3783	0.538733	0.0170	0.0037826289
ss86333579		2	49.200214	0.3023	0.6501	0.420341	0.0159	0.0066956449
ss86278882		2	49.230488	0.5473	0.0546	0.815230	0.0043	0.0021284569
ss117966835		2	49.287569	0.2285	1.9754	0.160322	0.0305	0.0107711079
ss61541665	*rs41636204*	2	49.316996	0.3474	1.2466	0.264590	0.0217	0.0098198069
ss86340329		2	49.352805	0.6755	0.1412	0.707217	0.0074	0.0032512870
ss86307124		2	49.403543	0.1132	1.6776	0.195679	0.0384	0.0077127836
ss105277002		2	49.454048	0.3066	0.0522	0.819301	0.0047	0.0019824423
ss86313293		2	49.475639	0.1891	2.5581	0.110182	0.0376	0.0115225180
ss61541697	*rs41637133*	2	49.500516	0.8116	1.7383	0.187793	0.0310	0.0094845905
ss86288609		2	49.52372	0.2923	1.0622	0.303066	0.0209	0.0086553103
ss105255478		2	49.557875	0.1354	0.1646	0.685090	0.0107	0.0025080178
ss61541641	*rs41636180*	2	49.852853	0.3288	0.1576	0.691459	0.0081	0.0035602568
ss86328856		2	49.874953	0.3997	1.7903	0.181321	0.0265	0.0127327252
ss61570398	*rs41667459*	2	49.965996	0.2235	0.3897	0.532665	0.0140	0.0048683251
ss86332981		2	50.235033	0.2586	0.0001	0.992360	0.0002	0.0000774243
ss86313151		2	50.310995	0.9499	7.4423	0.006532	0.1122	0.0106862887
ss61541647	*rs41636186*	2	50.333853	0.1533	7.7248	0.005594	0.0711	0.0184696669
ss105263586		2	50.37598	0.4377	2.5534	0.110513	0.0296	0.0145946538
ss86340365		2	50.447902	0.0702	0.0959	0.756897	0.0112	0.0014626852
ss117963639		2	50.48535	0.4491	3.3574	0.067329	0.0335	0.0165984102
ss61541658	*rs41636197*	2	50.531535	0.8438	5.6401	0.017824	0.0606	0.0159776908
ss38336734	*rs29023443*	2	50.999176	0.8001	6.6192	0.010294	0.0589	0.0188314450
ss61483877	*rs41582933*	2	51.028979	0.2385	4.6211	0.031925	0.0466	0.0169444119
	rs29019993	2	51.090803	0.8625	4.6574	0.031262	0.0576	0.0136633141
ss86336004		2	51.145679	0.1956	1.3043	0.253813	0.0271	0.0085382126
ss61522712	*rs41621483*	2	51.287243	0.2607	3.9459	0.047378	0.0421	0.0162439804
ss86319987		2	51.919825	0.3911	9.0054	0.002788	0.0563	0.0268021190
ss61475212	*rs41572699*	2	52.026892	0.1970	0.1419	0.706550	0.0087	0.0027369252
ss86341744		2	52.055632	0.1691	0.3225	0.570308	0.0139	0.0039016395
ss61483892	*rs41582945*	2	52.193862	0.6468	4.9003	0.027176	0.0410	0.0187472656
ss61509007	*rs41601072*	2	52.25476	0.8761	0.9687	0.325342	0.0275	0.0059621632
ss61509002	*rs41601067*	2	52.289716	0.2142	7.1368	0.007728	0.0587	0.0197637582
ss61483890	*rs41582943*	2	52.348153	0.0473	0.0163	0.898510	0.0057	0.0005100743
ss65223164	*rs43306221*	2	52.387332	0.3073	10.5255	0.001234	0.0656	0.0279270146
ss61541740	*rs41637173*	2	52.493503	0.6569	13.5936	0.000245	0.0710	0.0320055234
ss65223044	*rs43305501*	2	52.6317	0.2758	3.3650	0.067023	0.0379	0.0151560013
ss61541732	*rs41637166*	2	52.720969	0.2328	0.1225	0.726405	0.0077	0.0027557963
ss105264242		2	52.813363	0.6769	7.3426	0.006900	0.0513	0.0224324369
ss64738184	*rs42838818*	2	52.925405	0.3317	0.0255	0.873269	0.0031	0.0013941517
ss86330101		2	52.948557	0.5272	1.6920	0.193768	0.0243	0.0121190095
ss64759257	*rs42853311*	2	53.023212	0.6862	0.0046	0.945705	0.0014	0.0005937078
ss65221948	*rs43305105*	2	53.116612	0.5079	0.8144	0.367143	0.0169	0.0084556811
ss61541702	*rs41637137*	2	53.233831	0.3438	0.9345	0.334025	0.0191	0.0085965604
ss61541703	*rs41637138*	2	53.268661	0.3460	0.5742	0.448848	0.0148	0.0066950019
ss65222336	*rs43309692*	2	53.307188	0.3546	0.2490	0.617969	0.0096	0.0044113579
ss65222354	*rs43309710*	2	53.333719	0.1862	2.9754	0.084985	0.0393	0.0118989664

ss65222430	*rs43310586*	2	53.38042	0.2006	2.6126	0.106474	0.0360	0.0115330314
ss86341271		2	53.678652	0.6454	0.2789	0.597615	0.0101	0.0046091992
ss65222475	*rs43310630*	2	53.754324	0.8324	0.4298	0.512323	0.0165	0.0046064499
ss61541712	*rs41637147*	2	53.830446	0.8231	4.3404	0.037583	0.0499	0.0145322038
ss105265587		2	53.980758	0.6261	1.5736	0.210102	0.0238	0.0111380952
ss86336571		2	54.018018	0.7163	0.2765	0.599182	0.0110	0.0044790930
	rs29022902	2	54.06498	0.7321	3.9371	0.047626	0.0417	0.0163762304
ss65222660	*rs43312514*	2	54.110159	0.2658	4.4403	0.035458	0.0445	0.0173555083
ss61541723	*rs41637157*	2	54.196846	0.5981	1.4343	0.231466	0.0224	0.0107782760
ss65222839	*rs43313793*	2	54.236611	0.6927	2.2064	0.137889	0.0301	0.0128151772
ss65222862	*rs43313816*	2	54.257523	0.3044	2.1891	0.139445	0.0299	0.0126802547
ss86329049		2	54.282944	0.7128	0.0099	0.920608	0.0020	0.0008367609
ss86337427		2	54.304163	0.3238	3.6790	0.055510	0.0381	0.0166769508
ss61508998	*rs41601063*	2	54.357046	0.4828	0.0327	0.856530	0.0034	0.0016907753
ss61541726	*rs41637160*	2	54.442166	0.7142	0.0155	0.900902	0.0025	0.0010324340
ss61541725	*rs41637159*	2	54.466803	0.2235	2.7401	0.098310	0.0364	0.0126291322
ss65222988	*rs43305445*	2	54.520009	0.1426	4.2312	0.040059	0.0529	0.0129344369
ss61509000	*rs41601065*	2	54.544859	0.8947	4.5199	0.033853	0.0660	0.0124335642
ss65222940	*rs43304797*	2	54.577243	0.8181	0.4454	0.504740	0.0158	0.0047017634
ss65222931	*rs43304788*	2	54.604126	0.1597	1.6007	0.206226	0.0323	0.0086617113
ss65222905	*rs43304762*	2	54.637196	0.8403	1.6007	0.206226	0.0323	0.0086617113
	rs29025559	2	54.68963	0.7844	0.9748	0.323826	0.0223	0.0075277757
ss63932230	*rs42392928*	2	54.720203	0.1218	4.1358	0.042365	0.0585	0.0125195514
ss61475274	*rs41573759*	2	54.752584	0.6877	3.2418	0.072213	0.0358	0.0153647953
ss61522841	*rs41622110*	2	54.772881	0.8603	1.3027	0.254109	0.0308	0.0074119603
ss63932058	*rs42392842*	2	54.832957	0.2242	0.4593	0.498159	0.0148	0.0051407013
ss86295948		2	54.918839	0.7665	0.1516	0.697139	0.0087	0.0031176769
ss63931712	*rs42390469*	2	55.040367	0.8259	0.2729	0.601562	0.0131	0.0037705953
ss61525076	*rs41625303*	2	55.106601	0.5523	10.0682	0.001575	0.0613	0.0303127075
ss64643748	*rs42738852*	2	55.127279	0.2507	1.6559	0.198581	0.0281	0.0105635545
ss61465758	*rs41569002*	2	55.156876	0.3467	9.2730	0.002413	0.0596	0.0270121911
ss61516458	*rs41615866*	2	55.189145	0.0903	0.1825	0.669354	0.0138	0.0022708493
ss61475796	*rs41577366*	2	55.226668	0.2235	3.4483	0.063739	0.0417	0.0144747728
ss117966798		2	55.347853	0.2966	3.0860	0.079407	0.0358	0.0149505958
ss63946415	*rs42400983*	2	55.393279	0.6375	0.6871	0.407449	0.0161	0.0074296040
ss86280432		2	55.50548	0.5315	0.6645	0.415266	0.0156	0.0077829604
ss86290119		2	55.52595	0.6884	3.7970	0.051746	0.0393	0.0168446788
ss64516810	*rs42674303*	2	55.568155	0.1734	3.7397	0.053540	0.0461	0.0132217075
ss64222180	*rs42532809*	2	55.589622	0.8926	6.6888	0.009904	0.0777	0.0149124545
ss61495027	*rs41592510*	2	55.61478	0.4362	0.4285	0.512938	0.0126	0.0062101069
ss64222464	*rs42534351*	2	55.638949	0.0609	3.1875	0.074637	0.0699	0.0079977794
ss61564977	*rs41657643*	2	55.716694	0.0716	0.0550	0.814699	0.0085	0.0011311546
ss64222336	*rs42533487*	2	55.751116	0.3274	0.6592	0.417126	0.0161	0.0071043087
ss64222294	*rs42533466*	2	55.782448	0.2600	2.1180	0.146028	0.0313	0.0120579074
ss64222194	*rs42532816*	2	55.833877	0.1325	2.1583	0.142256	0.0407	0.0093486701
ss64179039	*rs42518312*	2	56.022349	0.6547	6.0452	0.014186	0.0495	0.0223614749
ss64179091	*rs42509638*	2	56.098625	0.3503	7.2291	0.007345	0.0539	0.0245447775

ss64179167	*rs42509675*	2	56.122993	0.3711	6.4549	0.011280		0.0495	0.0231244930
ss86338053		2	56.143759	0.9126	0.3343	0.563315		0.0187	0.0029878332
ss64179279	*rs42510431*	2	56.242165	0.8933	2.7479	0.097836		0.0497	0.0094822621
	rs29020790	2	56.269394	0.7128	1.0308	0.310317		0.0213	0.0087332963
ss61526107	*rs41624899*	2	56.291807	0.6418	0.1681	0.681954		0.0081	0.0037385280
ss86336868		2	56.348034	0.1218	7.9462	0.004956		0.0791	0.0169249855
ss61495020	*rs41592503*	2	56.459455	0.9319	0.0166	0.897660		0.0048	0.0006048647
ss64639506	*rs42736712*	2	56.611524	0.5265	2.4126	0.120815		0.0298	0.0148404467
ss61472355	*rs41569893*	2	56.647106	0.2779	11.7478	0.000645		0.0722	0.0289786697
ss61494596	*rs41588086*	2	56.704457	0.5838	1.8105	0.178882		0.0266	0.0129476555
ss64793388	*rs42883957*	2	56.721816	0.6089	10.1298	0.001524		0.0613	0.0292100538
ss117966845		2	56.76163	0.4026	2.7887	0.095384		0.0316	0.0151903494
ss86318400		2	56.790863	0.5380	9.3635	0.002299		0.0581	0.0288802288
	rs29025817	2	56.831558	0.0516	0.4028	0.525835		0.0263	0.0025748530
ss64181217	*rs42511576*	2	56.878351	0.4004	0.0001	0.992649		0.0002	0.0000824724
ss117963631		2	56.908137	0.3116	0.3610	0.548145		0.0120	0.0051383289
	rs29011513	2	56.928322	0.2607	5.6279	0.017948		0.0513	0.0197655579
ss65307255	*rs43394893*	2	56.950022	0.1948	0.1423	0.706154		0.0088	0.0027519249
ss65307235	*rs43394873*	2	56.976468	0.6089	16.2200	0.000063		0.0771	0.0367320819
ss61554208	*rs41651155*	2	57.038371	0.6984	3.7706	0.052565		0.0386	0.0162521578
ss117966681		2	57.105487	0.4427	0.7882	0.374940		0.0164	0.0080762559
ss63986515	*rs42418154*	2	57.174981	0.5394	5.9168	0.015248		0.0456	0.0226400358
ss63985893	*rs42415055*	2	57.254628	0.4534	1.6046	0.205677		0.0242	0.0119977210
ss61470678	*rs41572354*	2	57.279353	0.0795	0.9530	0.329291		0.0343	0.0050208187
ss61513048	*rs41609846*	2	57.335961	0.3868	1.0582	0.303995		0.0201	0.0095556105
ss61513047	*rs41609845*	2	57.362656	0.2930	0.6604	0.416709		0.0169	0.0069879875
ss63711834	*rs42320860*	2	57.418789	0.2070	0.9528	0.329348		0.0226	0.0074275512
ss86341559		2	57.444038	0.6569	0.1044	0.746705		0.0065	0.0029362115
ss61470677	*rs41572353*	2	57.476443	0.7872	0.7306	0.392982		0.0201	0.0067304825
ss63711579	*rs42320075*	2	57.637484	0.0802	3.1448	0.076606		0.0608	0.0089794976
ss61513065	*rs41609863*	2	57.670538	0.2729	0.0465	0.829308		0.0046	0.0018306450
ss63711438	*rs42328025*	2	57.696863	0.3574	0.2737	0.600999		0.0102	0.0046690011
ss61513044	*rs41609842*	2	57.719731	0.3216	4.1750	0.041400		0.0396	0.0173019745
ss63711354	*rs42327997*	2	57.741282	0.1547	0.2163	0.642018		0.0117	0.0030652030
ss64909453	*rs42999674*	2	57.761347	0.1218	0.0637	0.800790		0.0072	0.0015450567
ss61499491	*rs43710679*	2	57.796573	0.6590	0.0440	0.833955		0.0043	0.0019261575
ss64187157	*rs42520627*	2	57.821156	0.2285	4.9155	0.026938		0.0486	0.0171515213
ss61563748	*rs41663417*	2	57.843142	0.3059	4.2741	0.039067		0.0414	0.0175935400
ss86297480		2	57.885744	0.1590	5.3223	0.021347		0.0585	0.0156351458
ss64771163	*rs42862169*	2	57.916273	0.7736	2.0022	0.157523		0.0319	0.0111752567
ss117966821		2	57.983537	0.5279	5.3025	0.021589		0.0442	0.0220073620
ss61570651	*rs41670205*	2	58.018846	0.4463	6.4240	0.011477		0.0481	0.0237645275
ss65477148	*rs43559481*	2	58.039478	0.7973	0.0577	0.810310		0.0057	0.0018379181
ss61492703	*rs41589853*	2	58.063627	0.5981	4.4614	0.035026		0.0400	0.0192262519
ss65477011	*rs43558644*	2	58.092926	0.5007	2.7694	0.096533		0.0317	0.0158448528
ss63698115	*rs42320735*	2	58.11407	0.7973	0.0577	0.810310		0.0057	0.0018379181
ss86292569		2	58.134609	0.7966	0.0811	0.775951		0.0067	0.0021730838

ss63698370	*rs42320820*	2	58.163682	0.4004	5.5366	0.018901		0.0444	0.0213401404
	rs29015781	2	58.185444	0.0910	0.5860	0.444240		0.0242	0.0040106824
ss117966663		2	58.250628	0.6125	3.2102	0.073617		0.0344	0.0163118506
ss61512721	*rs41614523*	2	58.366503	0.2586	6.6966	0.009861		0.0547	0.0209598245
ss61465442	*rs41565791*	2	58.421289	0.8188	2.4266	0.119748		0.0372	0.0110381059
	rs29019912	2	58.451221	0.5888	6.1940	0.013051		0.0468	0.0226645578
ss86283415		2	58.527215	0.6590	0.1001	0.751751		0.0062	0.0027830622
ss61515101	*rs41611962*	2	58.548041	0.5759	4.7629	0.029412		0.0410	0.0200501804
ss64576190	*rs42708826*	2	58.580086	0.4248	2.6408	0.104606		0.0298	0.0145861448
ss61515097	*rs41611958*	2	58.626068	0.1741	2.2038	0.138125		0.0367	0.0105516707
ss61470500	*rs41570485*	2	58.72267	0.5910	3.0171	0.082833		0.0319	0.0154416316
ss61512724	*rs41614526*	2	58.759	0.2543	1.6035	0.205836		0.0267	0.0101158423
ss61512728	*rs41614530*	2	58.785905	0.4542	1.3756	0.241247		0.0215	0.0106514460
ss61465443	*rs41565792*	2	58.818593	0.5867	3.8419	0.050387		0.0361	0.0175188459
ss117966671		2	58.84274	0.4735	9.9314	0.001695		0.0585	0.0291464849
ss64835961	*rs42927614*	2	58.915746	0.4928	2.6448	0.104342		0.0298	0.0148820036
ss63654165	*rs42300442*	2	59.14527	0.6275	10.4301	0.001298		0.0613	0.0286737441
ss63654102	*rs42300121*	2	59.226518	0.6683	3.6826	0.055392		0.0375	0.0166391016
ss63653464	*rs42303892*	2	59.276345	0.8947	0.0239	0.877126		0.0046	0.0008681737
ss63653365	*rs42303859*	2	59.345688	0.0645	1.2799	0.258299		0.0414	0.0049915558
ss65459286	*rs43538128*	2	59.428328	0.0867	2.8934	0.089389		0.0561	0.0088865845
ss61526613	*rs41624599*	2	59.718306	0.3875	1.4779	0.224518		0.0237	0.0112344020
ss61473443	*rs41570661*	2	59.798987	0.6361	0.0569	0.811481		0.0048	0.0022002743
ss65224016	*rs43305873*	2	59.832175	0.4269	0.0385	0.844542		0.0036	0.0017526420
ss65223857	*rs43312111*	2	59.901005	0.4284	0.0012	0.972035		0.0006	0.0003132893
ss86291019		2	59.932526	0.8761	1.0954	0.295646		0.0290	0.0063075355
ss117966571		2	59.96753	0.2464	2.1386	0.144085		0.0313	0.0116419726
	rs29021692	2	60.07131	0.7393	19.7298	0.000010		0.0979	0.0377428950
ss61483895	*rs41582948*	2	60.115778	0.1683	6.3332	0.012074		0.0643	0.0180113142
ss65223472	*rs43308427*	2	60.14319	0.4871	0.2183	0.640481		0.0090	0.0045120678
ss61541742	*rs41637175*	2	60.178845	0.7758	3.6144	0.057695		0.0425	0.0147896139
ss65223312	*rs43307468*	2	60.217351	0.8109	0.4020	0.526273		0.0151	0.0046422485
	rs29024293	2	60.249494	0.2872	1.1137	0.291649		0.0226	0.0092360765
ss86322170		2	60.288883	0.7407	0.6676	0.414155		0.0185	0.0071206531
ss86338239		2	60.434577	0.4126	12.5420	0.000424		0.0691	0.0335119014
ss63793428	*rs29020674*	2	60.485597	0.0451	0.5906	0.442446		0.0342	0.0029481007
ss86341583		2	60.521064	0.6898	0.1814	0.670305		0.0085	0.0036578986
ss63793004	*rs42354159*	2	60.803066	0.1275	1.0786	0.299361		0.0289	0.0064382111
ss63792682	*rs42352599*	2	60.844511	0.6984	0.2123	0.645128		0.0096	0.0040338138
ss86303018		2	61.020129	0.8145	0.7553	0.385113		0.0205	0.0062043546
ss86340917		2	61.047936	0.0659	0.1851	0.667148		0.0160	0.0019642900
ss86320833		2	61.074008	0.1146	4.2296	0.040097		0.0594	0.0120458973
ss86340229		2	61.108766	0.7722	0.6685	0.413860		0.0187	0.0065822347
ss86313731		2	61.142844	0.6540	0.0382	0.845012		0.0038	0.0017236778
ss86339412		2	61.165821	0.3739	1.0128	0.314575		0.0194	0.0090840652
ss86288785		2	61.311966	0.5136	1.8846	0.170252		0.0256	0.0127905108
ss86296677		2	61.390455	0.9499	2.3830	0.123119		0.0648	0.0061694999

ss61477446		2	61.541676	0.4377	6.3312	0.012087	0.0483	0.0237506339
ss61529031	*rs41631942*	2	61.579008	0.5996	2.9022	0.088905	0.0325	0.0155856539
ss61529034	*rs41631945*	2	61.621314	0.6354	0.5767	0.447866	0.0147	0.0067964795
ss86321230		2	61.672718	0.1519	0.0151	0.902290	0.0032	0.0008324989
ss86341411		2	61.746771	0.6024	10.3282	0.001371	0.0621	0.0297644544
ss117966751		2	61.839493	0.1705	6.6088	0.010355	0.0634	0.0179372783
ss64055735	*rs42452794*	2	61.863325	0.7357	4.5809	0.032677	0.0445	0.0173205823
ss64120491	*rs42483763*	2	61.919312	0.2020	1.1046	0.293633	0.0244	0.0078637415
ss61564158	*rs41659333*	2	62.039835	0.0759	3.8078	0.051416	0.0699	0.0098026091
ss64918747	*rs43009207*	2	62.06223	0.1762	2.7075	0.100326	0.0408	0.0118406485
ss64203140	*rs42530075*	2	62.0888	0.8410	1.9875	0.159052	0.0364	0.0097459214
ss61478968	*rs41579840*	2	62.137308	0.2493	0.0016	0.967871	0.0009	0.0003309105
ss86275838		2	62.169964	0.8030	0.2860	0.592975	0.0125	0.0039562527
ss86304122		2	62.19439	0.0967	12.6094	0.000410	0.1103	0.0192627979
ss64813306	*rs42910364*	2	62.26975	0.7471	0.0036	0.952237	0.0013	0.0004948443
ss61525448	*rs41621063*	2	62.293568	0.2128	2.7346	0.098647	0.0380	0.0127234872
	rs29017559	2	62.324131	0.1203	15.2643	0.000103	0.1097	0.0232301287
ss64669890	*rs42766559*	2	62.364408	0.1239	14.6134	0.000144	0.1065	0.0231247590
ss64823657	*rs42921582*	2	62.402577	0.1454	13.6172	0.000242	0.0967	0.0240397695
ss86316829		2	62.559414	0.1805	20.0893	0.000009	0.1049	0.0310236707
ss65224320	*rs43307673*	2	62.689753	0.8539	5.1363	0.023737	0.0600	0.0149725503
ss86287059		2	62.751115	0.1970	4.6515	0.031368	0.0511	0.0161533840
ss65224048	*rs43305905*	2	62.786375	0.1963	4.7300	0.029977	0.0515	0.0162481960
ss61483906	*rs41582958*	2	62.81	0.8947	0.3227	0.570184	0.0174	0.0032851799
ss86316557		2	62.843802	0.6640	0.3869	0.534163	0.0121	0.0053977571
ss86308140		2	62.895962	0.6963	0.2242	0.636024	0.0096	0.0040589081
ss86338619		2	62.929794	0.4749	3.3480	0.067716	0.0342	0.0170362235
ss86337617		2	63.023009	0.6332	9.1122	0.002632	0.0570	0.0264544015
ss86314096		2	63.058132	0.4943	1.3672	0.242697	0.0207	0.0103374885
ss63430444	*rs42226330*	2	63.146405	0.8410	2.2204	0.136652	0.0369	0.0098616707
ss63430738	*rs42227428*	2	63.187546	0.1225	3.0984	0.078807	0.0489	0.0105057262
ss86293598		2	63.385853	0.2514	1.7445	0.187002	0.0291	0.0109496658
ss86326486		2	63.471875	0.0738	5.1480	0.023579	0.0802	0.0109575498
ss61465660	*rs41568005*	2	63.593895	0.8388	4.6359	0.031653	0.0538	0.0145393515
ss86295998		2	63.623563	0.8689	2.1571	0.142363	0.0393	0.0089536048
ss86324908		2	63.655052	0.4377	2.3816	0.123227	0.0290	0.0142909941
ss86339564		2	63.687699	0.8739	0.0480	0.826708	0.0063	0.0013889587
ss86281074		2	63.739827	0.9262	12.3979	0.000458	0.1213	0.0165735932
ss86298549		2	63.779377	0.2149	11.1667	0.000877	0.0755	0.0254790621
ss86326539		2	63.860451	0.7908	12.4136	0.000454	0.0816	0.0269868709
	rs29011172	2	63.94767	0.1067	6.6439	0.010154	0.0794	0.0151487355
ss86339595		2	63.998174	0.4305	24.2068	0.000001	0.0894	0.0438455952
ss86335891		2	64.044332	0.8911	5.6465	0.017760	0.0728	0.0141236813
	rs29015037	2	64.120219	0.2880	0.9040	0.342044	0.0194	0.0079659445
ss86291906		2	64.159905	0.1819	2.0472	0.152936	0.0351	0.0104390715
ss61485821	*rs41586020*	2	64.218857	0.2693	0.3611	0.548117	0.0125	0.0049142090
ss86340901		2	64.2449	0.2844	0.0569	0.811532	0.0050	0.0020178861

ss61469568	*rs41571167*	2	64.266248	0.5451	0.7320	0.392517	0.0158	0.0078502218
ss86295467		2	64.296023	0.9112	9.8880	0.001734	0.0990	0.0160179998
ss86331677		2	64.455007	0.7350	2.4324	0.119307	0.0325	0.0126476047
ss86323092		2	64.525879	0.1669	0.0000	0.999785	0.0000	0.0000018813
ss86290479		2	64.947791	0.6748	11.9566	0.000578	0.0666	0.0292106440
ss61519990	*rs41620314*	2	65.054366	0.8797	0.4639	0.496025	0.0197	0.0041749272
ss86297260		2	65.109734	0.5860	0.4918	0.483348	0.0127	0.0061599141
ss61541799	*rs41638232*	2	65.462684	0.3625	7.1099	0.007844	0.0489	0.0226039134
ss86287538		2	65.499051	0.6390	14.8465	0.000127	0.0728	0.0335928901
ss86334225		2	65.525412	0.3646	13.2689	0.000290	0.0694	0.0321434314
ss86309355		2	65.562802	0.7206	0.1530	0.695791	0.0078	0.0031376034
ss61541806	*rs41638239*	2	65.624076	0.2292	4.3369	0.037660	0.0459	0.0162257091
ss61483941	*rs41582993*	2	65.649421	0.2013	6.1836	0.013127	0.0573	0.0184158654
ss61483943	*rs41582995*	2	65.737667	0.5989	1.6037	0.205801	0.0239	0.0114867913
ss61541807	*rs41638240*	2	65.784471	0.6812	0.1435	0.704944	0.0072	0.0031229966
ss86287664		2	65.812459	0.1562	1.2294	0.267904	0.0283	0.0074497943
ss86340241		2	65.854003	0.4570	5.9391	0.015058	0.0432	0.0214388763
ss86333648		2	65.960162	0.4119	1.1322	0.287677	0.0197	0.0095370629
ss86333605		2	65.983645	0.1196	10.0301	0.001607	0.0870	0.0183222573
ss117966509		2	66.017452	0.4362	1.3488	0.245883	0.0210	0.0103406605
ss86307884		2	66.040482	0.3732	1.6634	0.197577	0.0243	0.0113820847
	rs29010456	2	66.096301	0.3431	0.5832	0.445303	0.0146	0.0065929951
ss86291388		2	66.117523	0.3059	0.8025	0.370669	0.0184	0.0078130798
ss86330147		2	66.145048	0.5881	2.6737	0.102470	0.0298	0.0144144999
ss61541812	*rs41638245*	2	66.201456	0.3016	0.3818	0.536852	0.0125	0.0052757650
ss86341319		2	66.236339	0.3037	0.4895	0.484366	0.0141	0.0059671238
	rs29010903	2	66.266589	0.3130	0.4463	0.504311	0.0131	0.0056387242
ss61467919	*rs41564942*	2	66.379943	0.7307	0.0631	0.801789	0.0053	0.0020776144
ss86300184		2	66.464867	0.7163	2.1325	0.144659	0.0301	0.0122221959
	rs29027494	2	66.587165	0.6074	4.2185	0.040358	0.0397	0.0189469826
ss61541815	*rs41638248*	2	66.624237	0.5337	1.0331	0.309792	0.0192	0.0095768645
ss86336228		2	66.654373	0.3940	0.0783	0.779714	0.0054	0.0025881469
ss86340290		2	66.702277	0.3446	3.5774	0.058986	0.0369	0.0166878283
ss117966653		2	66.743728	0.3381	0.5642	0.452836	0.0146	0.0065480951
ss61541823	*rs41638256*	2	66.863071	0.6619	0.5642	0.452836	0.0146	0.0065480951
ss61527393	*rs41623355*	2	67.057934	0.6160	9.0194	0.002767	0.0575	0.0272217152
ss86316304		2	67.083105	0.4011	27.2106	0.000000	0.0997	0.0478816807
ss61477460	*rs41576795*	2	67.184727	0.7464	14.9432	0.000121	0.0808	0.0306062528
ss61527294	*rs41622659*	2	67.231613	0.7744	13.7039	0.000231	0.0818	0.0285849590
ss86331093		2	67.255212	0.6404	2.6699	0.102717	0.0320	0.0147329583
ss86321904		2	67.279288	0.7421	0.3945	0.530172	0.0137	0.0052453037
ss61523294	*rs41619965*	2	67.342171	0.1884	5.0015	0.025641	0.0535	0.0163561870
ss86313264		2	67.366016	0.4957	10.4301	0.001298	0.0580	0.0290115952
ss117966632		2	67.408261	0.5208	8.1950	0.004327	0.0521	0.0259840254
ss61485350	*rs41581369*	2	67.42979	0.4792	8.1950	0.004327	0.0521	0.0259840254
ss61468125	*rs41565647*	2	67.451124	0.3252	10.1875	0.001478	0.0627	0.0275192061
ss86320554		2	67.665986	0.0652	4.8951	0.027257	0.0841	0.0102447901

ss61541832	*rs41638264*	2	67.882036	0.6103	1.3917	0.238517	0.0220	0.0104879877
ss86341326		2	67.968261	0.5000	8.5589	0.003550	0.0537	0.0268624575
ss86301441		2	67.981465	0.4635	4.3656	0.037033	0.0383	0.0190357773
ss117963582		2	68.006158	0.5415	4.7426	0.029760	0.0398	0.0197465692
ss117966888		2	68.074186	0.3746	5.0747	0.024588	0.0420	0.0196779636
ss86298279		2	68.112476	0.1777	3.7082	0.054553	0.0465	0.0136001651
ss86285808		2	68.188569	0.6712	12.3942	0.000459	0.0677	0.0298888866
ss86298138		2	68.219182	0.1354	13.2567	0.000292	0.0953	0.0223036416
	rs29024553	2	68.241373	0.3044	0.7893	0.374617	0.0177	0.0074973881
ss61541837	*rs41638269*	2	68.284576	0.5566	1.0123	0.314700	0.0193	0.0095263372
ss86296518		2	68.355014	0.6554	4.6012	0.032295	0.0413	0.0186611906
ss86339390		2	68.375425	0.6074	7.2154	0.007401	0.0495	0.0235838290
ss86281321		2	68.409302	0.6576	5.1558	0.023473	0.0439	0.0197803525
ss86302183		2	68.429675	0.3947	7.1175	0.007811	0.0489	0.0233444710
ss86286153		2	68.453742	0.3417	5.1626	0.023382	0.0439	0.0197652757
ss86285785		2	68.492186	0.3961	6.3854	0.011726	0.0465	0.0222659123
ss61483990	*rs41583842*	2	68.515035	0.3954	6.6755	0.009977	0.0475	0.0227321964
ss86326898		2	68.540511	0.1404	5.8760	0.015602	0.0635	0.0153336085
ss61541849	*rs41638281*	2	68.641435	0.6626	7.4159	0.006627	0.0529	0.0236640977
ss86331630		2	68.885099	0.0630	0.0480	0.826607	0.0085	0.0010001706
ss86335759		2	68.908329	0.9212	1.0316	0.310126	0.0338	0.0049049551
ss86310606		2	69.008532	0.0960	4.8494	0.027984	0.0695	0.0120557296
ss61532155	*rs41633274*	2	69.069975	0.5788	1.4280	0.232501	0.0222	0.0108083492
ss61479207	*rs41576178*	2	69.106818	0.4706	0.0542	0.815986	0.0042	0.0021139671
ss86337037		2	69.262611	0.4434	17.9577	0.000026	0.0786	0.0387936367
	rs29017490	2	69.408877	0.7665	7.9989	0.004815	0.0611	0.0218744160
ss61532167	*rs41633286*	2	69.463699	0.4721	4.6631	0.031158	0.0406	0.0202265099
ss86339741		2	69.522159	0.5860	0.0049	0.944237	0.0013	0.0006522685
ss86301810		2	69.664459	0.1311	6.2519	0.012635	0.0664	0.0151270773
ss86282813		2	69.749824	0.2185	2.5783	0.108789	0.0365	0.0124652465
ss86290421		2	69.792716	0.7801	3.3110	0.069248	0.0418	0.0143362965
	rs29010833	2	69.818302	0.4835	0.1746	0.676218	0.0077	0.0038673656
	rs29011280	2	69.933796	0.4040	18.2975	0.000022	0.0791	0.0381100286
ss86317529		2	69.96463	0.4019	16.5529	0.000053	0.0750	0.0360382805
ss86301037		2	69.987499	0.1554	0.9684	0.325415	0.0249	0.0065302894
ss86290017		2	70.009715	0.2536	2.8451	0.092103	0.0355	0.0134555512
ss64063517	*rs42457904*	2	70.063884	0.2944	0.2804	0.596581	0.0107	0.0044276322
ss64063423	*rs42457857*	2	70.098921	0.5616	0.8533	0.355935	0.0171	0.0084398258
ss64063247	*rs42457169*	2	70.147589	0.2894	0.0018	0.966508	0.0009	0.0003549454
ss61500356	*rs41598610*	2	70.180384	0.8761	4.6036	0.032251	0.0614	0.0133350021
ss86336697		2	70.226407	0.4756	16.8425	0.000045	0.0768	0.0382872284
ss61466843	*rs41573383*	2	70.268963	0.4936	0.0167	0.897241	0.0024	0.0012031078
ss64062781	*rs42456036*	2	70.294798	0.2722	16.5709	0.000052	0.0850	0.0336886878
ss86325720		2	70.320764	0.4305	5.9168	0.015248	0.0480	0.0235131781
ss64748007	*rs42844441*	2	70.475936	0.7242	0.1203	0.728830	0.0073	0.0029268247
ss86290498		2	70.508564	0.2292	12.9947	0.000335	0.0813	0.0287155345
ss64969407	*rs43063137*	2	70.538092	0.2808	0.0626	0.802470	0.0053	0.0021220767

ss61473655	*rs41573071*	2	70.583896	0.3703	7.5725	0.006081	0.0534	0.0249158498
ss86336320		2	70.660556	0.7636	15.6469	0.000084	0.0877	0.0316458476
ss64602104	*rs42716746*	2	70.726543	0.2987	2.1543	0.142623	0.0309	0.0129666751
ss64672063	*rs42767362*	2	70.838276	0.0946	0.1590	0.690168	0.0123	0.0021077125
ss61526592	*rs41624578*	2	70.885449	0.2242	0.4529	0.501206	0.0155	0.0053786437
ss63992211	*rs42416266*	2	70.962445	0.1297	6.1236	0.013576	0.0687	0.0155080087
ss63992157	*rs42416239*	2	71.012672	0.4119	0.0004	0.984668	0.0004	0.0001760713
ss61526600	*rs41624586*	2	71.046513	0.1934	1.6774	0.195706	0.0322	0.0100408894
ss86310400		2	71.077343	0.3023	0.0000	0.998727	0.0000	0.0000137242
ss61526601	*rs41624587*	2	71.105647	0.6211	0.0311	0.860071	0.0034	0.0016043775
ss61531853	*rs41630393*	2	71.152803	0.0458	11.1560	0.000882	0.1467	0.0128329266
ss63992331	*rs42416326*	2	71.188152	0.6325	0.1901	0.662962	0.0084	0.0038977180
ss61541861	*rs41638293*	2	71.262694	0.5824	2.0732	0.150353	0.0272	0.0132460756
ss61476301	*rs41575562*	2	71.290852	0.7185	5.6875	0.017353	0.0492	0.0199014525
ss61566642	*rs41666568*	2	71.351612	0.3395	3.2705	0.070968	0.0347	0.0155652427
ss61470615	*rs41571394*	2	71.418874	0.2579	4.7527	0.029586	0.0449	0.0171846219
ss64472788	*rs42653038*	2	71.449761	0.2564	0.7984	0.371880	0.0188	0.0071742989
ss64101845	*rs42471251*	2	71.482689	0.6755	4.6433	0.031518	0.0427	0.0187039658
ss64958042	*rs43048998*	2	71.513214	0.4842	2.2818	0.131351	0.0284	0.0141769357
ss61531001	*rs41631064*	2	71.545225	0.0981	5.0128	0.025476	0.0701	0.0124014259
ss64472736	*rs42652212*	2	71.575538	0.6597	5.0963	0.024286	0.0449	0.0201505873
ss61512885	*rs41616286*	2	71.59688	0.6676	0.2400	0.624384	0.0094	0.0041779136
ss61530997	*rs41631060*	2	71.637774	0.8446	1.4495	0.229017	0.0312	0.0082014318
ss86277685		2	71.664678	0.6927	4.1913	0.041008	0.0412	0.0175446366
ss61474042	*rs41566654*	2	71.717547	0.7643	5.0475	0.024975	0.0495	0.0178479474
ss105268936		2	71.743548	0.9269	6.5988	0.010412	0.0910	0.0123312978
ss117966784		2	71.769071	0.9248	5.4297	0.020082	0.0817	0.0113694323
ss64848734	*rs42947060*	2	71.797478	0.0602	8.4933	0.003679	0.1128	0.0127527130
	rs29019573	2	71.826	0.2113	3.0306	0.082148	0.0392	0.0130712733
ss61510147	*rs41601692*	2	72.289433	0.3675	7.6966	0.005681	0.0540	0.0250838472
ss65245075	*rs43326323*	2	72.312665	0.1261	3.5175	0.061143	0.0530	0.0116879793
ss65245084	*rs43327132*	2	72.337961	0.2120	3.0414	0.081606	0.0405	0.0135170991
ss65245094	*rs43327142*	2	72.362724	0.9499	10.7849	0.001075	0.1367	0.0130261731
ss61484016	*rs41583868*	2	72.394682	0.1497	0.3316	0.564928	0.0153	0.0038869895
ss86284772		2	72.467761	0.5681	1.3667	0.242777	0.0221	0.0108506558
	rs29017351	2	72.527749	0.1547	7.0133	0.008274	0.0675	0.0176601553
ss65245442	*rs43330487*	2	72.573878	0.2765	0.1820	0.669824	0.0087	0.0034855553
ss86310870		2	72.596097	0.4592	0.9152	0.339058	0.0177	0.0087817083
ss86320403		2	72.620837	0.5666	1.2461	0.264681	0.0210	0.0103310558
ss61489410	*rs41585038*	2	72.65889	0.5659	1.2493	0.264080	0.0211	0.0103614704
ss86337364		2	72.709978	0.3596	3.1713	0.075378	0.0340	0.0156769670
ss65245649	*rs43332694*	2	72.730729	0.3625	2.9666	0.085446	0.0329	0.0152135704
ss64620356	*rs42726892*	2	72.809516	0.7772	0.8354	0.361041	0.0202	0.0069863233
ss61477319	*rs41575864*	2	72.889808	0.6719	1.9305	0.165151	0.0273	0.0120397340
ss117966711		2	72.911422	0.7457	2.7059	0.100427	0.0349	0.0132261727
ss86294956		2	73.19235	0.9241	0.0044	0.947357	0.0024	0.0003318652
ss86300187		2	73.291459	0.4914	0.0198	0.888195	0.0026	0.0013085294

ss61541862	*rs41638294*	2	73.314772	0.7643	3.3478	0.067724	0.0402	0.0144921630
ss61467745	*rs41571870*	2	73.434414	0.8288	0.3535	0.552354	0.0145	0.0041203628
ss86325656		2	73.477445	0.6211	9.5767	0.002050	0.0601	0.0282810365
ss61512315	*rs41610923*	2	73.581086	0.8066	9.1826	0.002534	0.0710	0.0221472997
ss64443718	*rs42641880*	2	73.603908	0.8216	7.6428	0.005851	0.0657	0.0192596638
ss86339016		2	73.653376	0.1963	9.4277	0.002221	0.0714	0.0225333526
ss64792028	*rs42889305*	2	73.735717	0.1046	0.9424	0.331989	0.0301	0.0056310432
ss86313524		2	73.773944	0.5036	6.1671	0.013249	0.0465	0.0232276484
ss61541860	*rs41638292*	2	73.808804	0.6905	1.8036	0.179710	0.0270	0.0115216880
ss117966861		2	73.940063	0.4542	3.2366	0.072441	0.0336	0.0166704713
ss86313247		2	74.023817	0.2872	2.7038	0.100561	0.0335	0.0137349157
ss61541866	*rs41638298*	2	74.160665	0.5136	0.0139	0.906074	0.0022	0.0011033197
ss86298183		2	74.189139	0.0437	0.1555	0.693463	0.0181	0.0015141824
ss61541865	*rs41638297*	2	74.21425	0.0437	0.1555	0.693463	0.0181	0.0015141824
ss86327401		2	74.267441	0.2765	7.9131	0.005046	0.0582	0.0232714427
ss86274992		2	74.336966	0.1719	0.2367	0.626753	0.0120	0.0034091799
ss86317856		2	74.48256	0.4964	1.3163	0.251643	0.0211	0.0105317150
ss86292644		2	74.509813	0.8173	1.4047	0.236347	0.0283	0.0084380861
ss86340398		2	74.563877	0.8195	1.4205	0.233732	0.0286	0.0084686131
ss61484010	*rs41583862*	2	74.617146	0.1827	1.6532	0.198955	0.0307	0.0091533568
ss86331989		2	74.662176	0.1891	1.7207	0.190033	0.0308	0.0094514763
ss86330840		2	74.692652	0.1855	1.6274	0.202488	0.0303	0.0091709163
ss86296125		2	74.749692	0.7529	6.4783	0.011135	0.0539	0.0200461999
ss86273569		2	74.778481	0.3797	2.2440	0.134586	0.0291	0.0137146329
ss86288956		2	74.858654	0.7106	1.8218	0.177538	0.0283	0.0116223956
ss86306729		2	74.927107	0.4477	0.0373	0.846934	0.0036	0.0018004033
ss86288491		2	74.983776	0.4957	0.3841	0.535600	0.0116	0.0057865470
ss86291928		2	75.00447	0.5509	1.1799	0.277743	0.0205	0.0101493930
ss86333651		2	75.237934	0.3130	1.1246	0.289302	0.0210	0.0090171231
ss61484009	*rs41583861*	2	75.288926	0.5516	1.3592	0.244074	0.0220	0.0108779099
ss86315675		2	75.443117	0.7615	0.0282	0.866757	0.0037	0.0013269615
ss86278076		2	75.521554	0.2357	0.0082	0.927861	0.0020	0.0007102559
ss86284271		2	75.657529	0.0738	0.8505	0.356742	0.0334	0.0045685882
ss86340369		2	75.682987	0.5953	0.4817	0.487878	0.0132	0.0063680640
ss86337405		2	75.705825	0.5953	0.4817	0.487878	0.0132	0.0063680640
ss86324833		2	75.76286	0.4040	6.6626	0.010049	0.0474	0.0228086536
	rs29021995	2	75.801928	0.7464	1.1707	0.279639	0.0223	0.0084407943
ss86316201		2	75.822876	0.0752	4.2628	0.039325	0.0718	0.0099836871
ss86316479		2	75.86123	0.6848	3.8729	0.049468	0.0389	0.0167771375
ss86293992		2	75.885464	0.0752	4.6890	0.030696	0.0752	0.0104668345
ss86328083		2	76.074734	0.4076	0.3824	0.536498	0.0117	0.0056403256
ss86310726		2	76.164959	0.0960	1.4742	0.225092	0.0381	0.0066044079
ss86277753		2	76.205027	0.7393	0.0017	0.967323	0.0009	0.0003302091
ss86312361		2	76.290402	0.4549	13.7673	0.000223	0.0681	0.0337932873
ss86279378		2	76.334239	0.3904	14.8396	0.000128	0.0722	0.0343868638
ss86293216		2	76.359876	0.6103	16.5368	0.000053	0.0761	0.0361896171
ss86311572		2	76.399248	0.5072	12.0373	0.000554	0.0631	0.0315405621

ss86319904		2	76.604372	0.4047	12.6080	0.000410	0.0649	0.0312795534
ss86300144		2	76.846924	0.8510	8.6906	0.003306	0.0774	0.0196207864
ss86328905		2	76.903632	0.9176	22.2596	0.000003	0.1551	0.0234413639
ss117966771		2	76.981767	0.0924	3.7742	0.052451	0.0601	0.0100747711
ss86290645		2	77.207676	0.5122	13.9517	0.000203	0.0676	0.0337938106
ss86291713		2	77.260266	0.0702	4.7941	0.028888	0.0790	0.0103087044
ss86310409		2	77.298887	0.7436	0.0092	0.923533	0.0021	0.0007922350
ss86341644		2	77.326858	0.2679	0.8184	0.365950	0.0183	0.0071739128
ss117966501		2	77.402264	0.7400	1.6278	0.202431	0.0259	0.0099790406
ss86338480		2	77.425492	0.9269	0.0165	0.897900	0.0045	0.0006127976
ss86277963		2	77.500953	0.3116	3.4193	0.064862	0.0369	0.0158496426
ss86338759		2	77.544444	0.6519	0.4246	0.514876	0.0130	0.0059055488
ss86290288		2	77.565814	0.8668	4.2541	0.039527	0.0567	0.0130887073
ss86306484		2	77.600015	0.8102	0.0001	0.991891	0.0002	0.0000721988
ss117966524		2	77.63395	0.1927	0.0086	0.926326	0.0021	0.0006568932
ss86334285		2	77.76129	0.2357	0.9855	0.321182	0.0213	0.0076668572
ss61498229	*rs43709931*	2	78.009237	0.7178	0.5982	0.439512	0.0161	0.0065345615
ss86281107		2	78.151464	0.6168	0.2500	0.617202	0.0100	0.0047109350
ss86295323		2	78.277552	0.4850	0.7799	0.377478	0.0162	0.0080774867
ss117966834		2	78.322513	0.8782	0.8383	0.360193	0.0259	0.0055302554
ss117966767		2	78.399537	0.8617	1.4752	0.224936	0.0328	0.0078259919
ss61466043	*rs41565506*	2	78.588031	0.3703	0.4372	0.508683	0.0134	0.0062628192
ss86299505		2	78.681035	0.5509	5.5594	0.018657	0.0444	0.0219478335
ss61568149	*rs41662054*	2	78.895851	0.5602	5.5897	0.018340	0.0437	0.0215563846
ss86303199		2	79.076533	0.7027	5.8317	0.015997	0.0485	0.0202649743
ss61568156	*rs41662061*	2	79.248642	0.7407	0.2425	0.622536	0.0104	0.0039976867
ss117966527		2	79.510433	0.2256	1.2947	0.255576	0.0252	0.0087945198
ss86300058		2	79.557944	0.6698	0.8985	0.343524	0.0188	0.0083088321
	rs29018861	2	79.635503	0.2650	1.3174	0.251458	0.0245	0.0095306418
ss86334679		2	79.69137	0.2658	1.6021	0.206030	0.0271	0.0105634284
ss117963586		2	79.730014	0.3317	5.5078	0.019211	0.0466	0.0206437936
ss63406096	*rs42220527*	2	79.75696	0.1146	0.5102	0.475304	0.0213	0.0043171529
ss61527758	*rs41626002*	2	79.783049	0.7421	1.2380	0.266235	0.0230	0.0088172144
ss86308602		2	79.827895	0.3532	8.0233	0.004752	0.0564	0.0257564829
ss64024619	*rs42432716*	2	79.850194	0.5466	7.7325	0.005570	0.0533	0.0264426004
ss86330823		2	79.943942	0.1870	0.0218	0.882731	0.0036	0.0010898094
ss65080564	*rs43166122*	2	79.981271	0.3360	2.8891	0.089627	0.0340	0.0151885990
ss61476988	*rs41580736*	2	80.018457	0.2414	1.3061	0.253490	0.0254	0.0092941956
ss86321406		2	80.059142	0.7787	2.4935	0.114771	0.0358	0.0123333648
ss61527756	*rs41626000*	2	80.097532	0.2077	4.7435	0.029745	0.0500	0.0164637493
ss64024073	*rs42439541*	2	80.118827	0.7034	0.0220	0.882128	0.0029	0.0012073440
ss63029787	*rs42076238*	2	80.193482	0.3030	0.0567	0.811898	0.0046	0.0019613188
ss63029593	*rs42075641*	2	80.222971	0.0874	1.3558	0.244659	0.0384	0.0061220098
	rs29021731	2	80.279182	0.1648	0.5492	0.458897	0.0190	0.0052223038
ss86313260		2	80.315321	0.6905	0.5982	0.439518	0.0151	0.0064617642
ss86314008		2	80.346573	0.4828	3.7107	0.054473	0.0362	0.0180774331
ss65549062	*rs43627136*	2	80.465812	0.3102	2.0838	0.149318	0.0295	0.0126129160

ss86339014		2	80.493909	0.7421	1.3045	0.253777	0.0241	0.0092314710
ss61489752	*rs41586870*	2	80.533423	0.3066	2.0963	0.148111	0.0296	0.0125895222
ss65549125	*rs43627198*	2	80.577445	0.4570	7.3182	0.006993	0.0493	0.0244572388
ss61541882	*rs41638314*	2	80.591622	0.2758	6.6581	0.010075	0.0526	0.0209921324
ss86323741		2	80.655378	0.7314	3.4024	0.065526	0.0378	0.0148474714
ss65227258	*rs43311892*	2	80.678028	0.5652	6.4870	0.011081	0.0460	0.0226333895
ss86337269		2	80.700644	0.2908	0.8478	0.357501	0.0186	0.0076712478
ss65227058	*rs43310192*	2	80.763206	0.2994	8.8313	0.003063	0.0608	0.0255175192
	rs29020755	2	80.814826	0.4678	1.0440	0.307256	0.0190	0.0094365443
ss65022432	*rs43114460*	2	80.857635	0.8840	0.0000	0.997982	0.0001	0.0000153868
ss61531965	*rs41631503*	2	80.881624	0.1160	0.0000	0.997982	0.0001	0.0000153868
ss86295751		2	80.974131	0.8797	0.0766	0.782087	0.0080	0.0017038718
ss61484033	*rs41583885*	2	81.010607	0.8818	0.0757	0.783299	0.0080	0.0016773760
ss61509029	*rs41601093*	2	81.107937	0.2443	1.2860	0.257179	0.0242	0.0089177535
ss65226458	*rs43312598*	2	81.180167	0.1383	5.3559	0.020943	0.0643	0.0153295966
ss61512006	*rs41609716*	2	81.219099	0.2421	5.3758	0.020707	0.0500	0.0183510036
ss61569455	*rs41667836*	2	81.251474	0.8087	0.4914	0.483560	0.0164	0.0050636249
ss61569457	*rs41667838*	2	81.288597	0.8030	0.5815	0.445967	0.0177	0.0055848973
ss86318401		2	81.454191	0.3775	0.0954	0.757546	0.0058	0.0027282697
ss64431472	*rs42635530*	2	81.485039	0.3761	0.0874	0.767617	0.0055	0.0026042550
ss61569953	*rs41671202*	2	81.519018	0.6239	0.0874	0.767617	0.0055	0.0026042550
ss61470959	*rs41566533*	2	81.596136	0.3331	9.1622	0.002562	0.0583	0.0259093372
ss61470958	*rs41566532*	2	81.627377	0.9234	7.3931	0.006711	0.0957	0.0135459649
ss64497580	*rs42665716*	2	81.679349	0.5150	3.1137	0.078076	0.0327	0.0163370622
ss64738209	*rs42830643*	2	81.703277	0.1755	4.1611	0.041739	0.0489	0.0141518244
ss117966607		2	81.725075	0.6010	0.0154	0.901267	0.0023	0.0010964046
ss61496179	*rs41597432*	2	81.7567	0.3976	0.0573	0.810897	0.0044	0.0021196259
ss64961660	*rs43049698*	2	81.79024	0.6010	0.0154	0.901267	0.0023	0.0010964046
ss64785332	*rs42879427*	2	81.831063	0.6010	0.0154	0.901267	0.0023	0.0010964046
ss64785305	*rs42879400*	2	81.861373	0.5337	0.1253	0.723468	0.0065	0.0032562881
ss61474108	*rs41566720*	2	81.883501	0.5430	0.1477	0.700903	0.0071	0.0035235154
ss64979548	*rs43072892*	2	81.961483	0.6655	3.8937	0.048864	0.0393	0.0175054625
ss65227522	*rs43314456*	2	81.983989	0.5602	15.0968	0.000112	0.0733	0.0361113602
ss86302814		2	82.009168	0.4391	15.0077	0.000117	0.0730	0.0359485959
ss65227610	*rs43315444*	2	82.032203	0.3381	4.2756	0.039031	0.0410	0.0183649965
ss61502238	*rs41605449*	2	82.0558	0.6619	4.5268	0.033720	0.0421	0.0188321778
ss64877365	*rs42973444*	2	82.120283	0.6755	5.8057	0.016233	0.0488	0.0214107860
ss64877466	*rs42968246*	2	82.154684	0.9398	3.3735	0.066680	0.0721	0.0081597479
ss65227664	*rs43315498*	2	82.178085	0.6067	0.4926	0.483020	0.0132	0.0062828080
ss65227702	*rs43316036*	2	82.230778	0.2299	3.7692	0.052607	0.0430	0.0152229745
ss86304593		2	82.267986	0.3281	5.5004	0.019292	0.0472	0.0207897155
ss64145887	*rs42497832*	2	82.300196	0.6705	5.4871	0.019438	0.0471	0.0208302100
ss64145851	*rs42497114*	2	82.321653	0.3252	6.1495	0.013380	0.0499	0.0219048466
	rs29023283	2	82.369627	0.6648	4.7803	0.029119	0.0435	0.0193824880
ss86338271		2	82.549251	0.4441	15.8639	0.000075	0.0765	0.0377510624
ss61500767	*rs41599520*	2	82.573891	0.3489	15.0755	0.000113	0.0747	0.0339377307
ss64826728	*rs42926940*	2	82.634372	0.7199	3.5791	0.058926	0.0400	0.0161247090

ss117963579		2	82.703468	0.4893	0.2878	0.591790	0.0100	0.0049921436
ss86338389		2	82.724419	0.5330	0.0814	0.775457	0.0052	0.0025857795
ss86327993		2	82.772106	0.4019	1.2944	0.255627	0.0214	0.0102966752
ss86339800		2	82.803279	0.0738	1.6165	0.204006	0.0460	0.0062874916
ss86336288		2	82.84169	0.3682	0.0141	0.905394	0.0023	0.0010593554
ss117966789		2	82.865615	0.3668	0.0169	0.896603	0.0025	0.0011615635
ss86326414		2	82.933273	0.6562	2.0948	0.148248	0.0280	0.0126566608
ss86295399		2	82.959551	0.4298	2.3507	0.125684	0.0285	0.0139537616
ss61514601	*rs41614062*	2	83.013169	0.3567	2.5723	0.109201	0.0303	0.0138846458
ss86340193		2	83.037166	0.3574	2.7541	0.097458	0.0313	0.0143873316
ss86336801		2	83.072766	0.3317	1.6737	0.196197	0.0251	0.0111250905
ss61562700	*rs41663995*	2	83.335056	0.6003	0.1402	0.708243	0.0069	0.0033200971
ss86325216		2	83.382632	0.5201	0.1792	0.672218	0.0077	0.0038385372
ss117966856		2	83.412866	0.5179	0.1813	0.670373	0.0077	0.0038680242
ss61511174	*rs41610299*	2	83.490952	0.5193	0.3012	0.583314	0.0100	0.0049847304
ss86315147		2	83.588442	0.4241	2.6791	0.102127	0.0318	0.0155228411
ss86341054		2	83.640591	0.8360	1.4366	0.231093	0.0296	0.0081273244
ss86314633		2	83.677076	0.2514	0.9137	0.339465	0.0200	0.0075267429
ss61562702	*rs41663997*	2	83.729115	0.6239	5.9887	0.014644	0.0462	0.0216728372
ss61493844	*rs41591067*	2	83.750114	0.3374	7.6267	0.005903	0.0531	0.0237562742
ss61493845	*rs41591068*	2	83.792123	0.7049	0.0677	0.794752	0.0053	0.0021905152
ss117966828		2	83.842903	0.7256	5.2366	0.022416	0.0461	0.0183592569
ss117966818		2	83.879822	0.7149	1.4161	0.234450	0.0255	0.0104108982
ss46526243	*rs41255382*	2	83.913946	0.7471	9.8146	0.001804	0.0660	0.0249409146
ss86303621		2	83.971255	0.1741	6.2014	0.012997	0.0619	0.0177883113
ss61562725	*rs41664019*	2	84.004687	0.3295	0.4598	0.497955	0.0131	0.0057688398
ss61522003	*rs41622996*	2	84.09622	0.6898	1.7980	0.180390	0.0271	0.0115804305
ss61567281	*rs41661697*	2	84.120631	0.5573	0.3675	0.544542	0.0112	0.0055299427
ss86285351		2	84.155337	0.4427	0.3760	0.539930	0.0113	0.0055632309
ss61523927	*rs41624283*	2	84.23438	0.5566	0.4871	0.485478	0.0128	0.0063361849
ss117966881		2	84.405513	0.6397	6.0293	0.014314	0.0474	0.0218576679
ss86324755		2	84.487412	0.2693	1.0440	0.307243	0.0215	0.0084490652
ss61541918	*rs41639449*	2	84.56996	0.3367	1.7233	0.189699	0.0255	0.0113761711
ss61541909	*rs41639441*	2	84.65044	0.0573	0.9556	0.328643	0.0387	0.0041860084
ss61523297	*rs41619967*	2	84.677049	0.6848	3.8581	0.049904	0.0389	0.0168030379
ss61530021	*rs41630518*	2	84.753766	0.2837	0.8917	0.345338	0.0196	0.0079624719
ss117966800		2	84.885301	0.4054	0.1188	0.730496	0.0068	0.0032788094
ss61469617	*rs41571214*	2	85.079752	0.6956	0.9896	0.320183	0.0197	0.0083460807
ss61527642	*rs41625098*	2	85.237589	0.9155	0.3357	0.562535	0.0197	0.0030421795
ss61514976	*rs41611439*	2	85.473318	0.0838	0.3413	0.559246	0.0199	0.0030540831
ss61514979	*rs41611442*	2	85.565714	0.1053	0.7020	0.402403	0.0252	0.0047550403
ss61514982	*rs41611445*	2	85.589866	0.1289	4.6666	0.031095	0.0587	0.0131786027
ss61509043	*rs41601107*	2	86.309128	0.4499	6.8955	0.008831	0.0484	0.0239480348
ss38327001	*rs29015299*	2	86.330389	0.6196	0.1033	0.748054	0.0062	0.0029340735
ss61541969	*rs41639496*	2	86.373799	0.1956	3.4671	0.063020	0.0428	0.0134604456
ss61542003	*rs41639530*	2	86.55223	0.6777	7.4094	0.006651	0.0534	0.0233204761
ss61542008	*rs41640435*	2	86.572887	0.3245	8.9957	0.002803	0.0588	0.0257838984

ss117975306		2	86.610607	0.4670	3.5986	0.058241	0.0337	0.0167813314
ss86335374		2	86.763945	0.3546	0.0165	0.897726	0.0025	0.0011412213
ss61479631	*rs41580594*	2	86.882774	0.7693	4.7042	0.030426	0.0483	0.0171374811
ss63373047	*rs42206466*	2	86.906672	0.9269	0.3319	0.564752	0.0212	0.0028721860
ss86285489		2	87.116438	0.4993	0.2947	0.587401	0.0104	0.0052072056
ss61473710	*rs41573126*	2	87.325296	0.5831	4.9221	0.026838	0.0427	0.0207761172
ss61519316	*rs41613569*	2	87.432082	0.5831	0.1981	0.656367	0.0085	0.0041153663
ss61519314		2	87.460779	0.7636	0.0592	0.807820	0.0055	0.0019713720
ss117966868		2	87.628042	0.4047	0.8615	0.353652	0.0176	0.0084579372
ss117966639		2	87.677142	0.6196	3.7799	0.052274	0.0376	0.0177373233
ss61526735	*rs41625621*	2	87.743833	0.4670	3.0629	0.080540	0.0334	0.0166109549
ss86329199		2	87.880854	0.6189	5.6418	0.017808	0.0459	0.0216585565
	rs29026510	2	88.322262	0.5380	5.1295	0.023830	0.0427	0.0212074796
ss86335950		2	88.378268	0.7586	19.2417	0.000013	0.0932	0.0341309743
ss86317353		2	88.476615	0.0587	3.9914	0.046122	0.0804	0.0088959791
ss61483994	*rs41583846*	2	88.598931	0.5351	3.3013	0.069657	0.0330	0.0164023986
ss61542077	*rs41640502*	2	89.005464	0.0609	2.6096	0.106669	0.0633	0.0072349712
ss86296932		2	89.029477	0.1433	4.6464	0.031461	0.0566	0.0138909574
ss117966839		2	89.062659	0.1490	23.7033	0.000001	0.1240	0.0314356649
ss86339881		2	89.101819	0.1612	0.1156	0.733942	0.0086	0.0023387764
ss61542079	*rs41640504*	2	89.133232	0.8754	0.0116	0.914238	0.0030	0.0006589464
ss86319340		2	89.163561	0.1368	14.2183	0.000177	0.1006	0.0237633560
ss61509047	*rs41601111*	2	89.331627	0.5100	3.3327	0.068344	0.0335	0.0167543229
ss86340068		2	89.491555	0.0530	3.6904	0.055135	0.0797	0.0080003530
ss61559241	*rs41656086*	2	89.821803	0.8360	1.0547	0.304783	0.0252	0.0069212269
ss86316413		2	90.073392	0.2307	0.2828	0.595010	0.0115	0.0040929055
ss61542034	*rs41640459*	2	90.107658	0.2106	8.0000	0.004812	0.0631	0.0209777787
ss61483265	*rs41576342*	2	90.269911	0.3517	0.0097	0.921633	0.0019	0.0008669330
ss61529263	*rs41633771*	2	90.512024	0.5330	0.7322	0.392479	0.0158	0.0078872986
ss61494978	*rs41592462*	2	90.595293	0.1999	3.4074	0.065329	0.0428	0.0136777442
ss61494975	*rs41592459*	2	90.639724	0.1440	0.2799	0.596927	0.0143	0.0035275676
ss61474455	*rs41570157*	2	90.716864	0.7973	1.0826	0.298485	0.0245	0.0079294839
ss117966896		2	90.766798	0.1712	6.9493	0.008572	0.0667	0.0189188105
ss86286219		2	90.811821	0.2414	0.0121	0.912523	0.0024	0.0008932087
ss86320759		2	90.915444	0.7443	8.4054	0.003859	0.0594	0.0225943757
ss86334138		2	90.939953	0.2364	6.1505	0.013373	0.0568	0.0205060650
ss61520660	*rs41616583*	2	91.61925	0.7808	0.4677	0.494292	0.0156	0.0053462656
ss86304844		2	91.654003	0.0308	10.9152	0.001003	0.1765	0.0105365753
ss86322690		2	91.716733	0.7249	0.0860	0.769401	0.0061	0.0024433569
ss61520662	*rs41616585*	2	91.745584	0.7629	10.9448	0.000987	0.0685	0.0247890342
ss86325101		2	91.815686	0.3653	7.0057	0.008309	0.0492	0.0227945820
ss86336562		2	92.032671	0.8223	4.1673	0.041587	0.0500	0.0146234045
	rs29015159	2	92.075537	0.6239	6.3323	0.012079	0.0485	0.0227391655
ss117966854		2	92.309639	0.9262	4.9030	0.027133	0.0791	0.0108072154
ss86300982		2	92.385023	0.2192	2.4146	0.120664	0.0360	0.0123354382
ss117966822		2	92.419325	0.4054	0.5134	0.473919	0.0134	0.0064526588
ss86340983		2	92.455936	0.3711	0.5545	0.456748	0.0141	0.0065956339

ss86334753		2	92.482963	0.9377	0.3599	0.548774	0.0230	0.0026900673
ss61542067	*rs41640492*	2	92.57295	0.4298	0.0871	0.767953	0.0056	0.0027605105
ss61495928	*rs41594486*	2	92.801019	0.2493	0.0490	0.824868	0.0049	0.0018391822
ss86321720		2	92.885428	0.7242	0.0467	0.829015	0.0047	0.0018755576
ss117966894		2	92.966541	0.4434	2.0671	0.150961	0.0265	0.0130629370
ss86318293		2	93.116526	0.8252	0.3247	0.568970	0.0139	0.0040139793
ss86335868		2	93.251972	0.2385	1.2106	0.271590	0.0241	0.0087676192
ss61512120	*rs41610130*	2	93.320533	0.4527	2.9166	0.088116	0.0305	0.0151299972
ss86289118		2	93.354567	0.6032	0.5729	0.449358	0.0141	0.0067399480
ss86288321		2	93.460449	0.9090	3.6732	0.055702	0.0628	0.0103895001
ss86332558		2	93.510481	0.9026	4.1415	0.042223	0.0645	0.0113344146
ss86341371		2	93.586145	0.5946	0.9855	0.321191	0.0188	0.0090469432
ss86303606		2	93.630367	0.8030	1.1089	0.292688	0.0246	0.0077911849
ss86331355		2	93.655203	0.8911	2.4349	0.119118	0.0461	0.0089518210
ss86340284		2	93.679101	0.2421	3.3405	0.068024	0.0387	0.0141882928
ss86326904		2	93.723068	0.6282	0.4793	0.488988	0.0134	0.0062417522
ss86336299		2	93.757866	0.5702	1.7468	0.186713	0.0249	0.0122218369
ss86339951		2	93.809308	0.3295	11.6841	0.000667	0.0669	0.0295510004
ss61497657	*rs43703384*	2	93.963499	0.6569	0.7095	0.399895	0.0167	0.0075471266
ss61521886	*rs41622384*	2	93.990663	0.6762	0.0650	0.798841	0.0052	0.0022554757
ss86330289		2	94.132348	0.5888	5.8208	0.016095	0.0453	0.0219268605
ss86338665		2	94.20036	0.5029	3.7377	0.053604	0.0355	0.0177602760
ss86341614		2	94.230525	0.1218	4.9542	0.026347	0.0631	0.0134900331
ss86322796		2	94.256196	0.2486	0.6349	0.425838	0.0170	0.0063521646
ss61484084	*rs41584635*	2	94.403001	0.9362	0.0363	0.848993	0.0074	0.0008858556
ss61497001	*rs41593932*	2	94.65296	0.5244	0.1999	0.654905	0.0082	0.0040798461
ss86297123		2	94.97916	0.1354	14.0920	0.000189	0.1017	0.0238039870
ss86336347		2	95.019016	0.9534	2.0526	0.152391	0.0639	0.0056698096
ss86280212		2	95.069809	0.0953	0.1281	0.720559	0.0114	0.0019575294
ss86319799		2	95.23376	0.9513	1.0496	0.305956	0.0434	0.0040256098
ss86311197		2	95.288641	0.9262	2.1363	0.144304	0.0502	0.0068659935
ss86323076		2	95.445519	0.3453	0.8022	0.370754	0.0173	0.0078357625
ss61568889	*rs41667270*	2	95.621274	0.3524	1.0147	0.314132	0.0197	0.0089774526
ss117966857		2	95.762271	0.8696	0.7386	0.390417	0.0233	0.0052927335
ss117966786		2	95.823711	0.6368	1.2560	0.262796	0.0216	0.0100058027
ss61529085	*rs41631995*	2	96.422489	0.5695	6.5156	0.010906	0.0467	0.0229034650
ss86287659		2	96.45796	0.1569	8.6671	0.003348	0.0723	0.0191130583
ss61466811	*rs41573352*	2	96.50513	0.0766	0.1098	0.740422	0.0114	0.0016073024
ss61529086	*rs41631996*	2	96.553379	0.0967	0.0452	0.831651	0.0067	0.0011629085
ss61542092	*rs41640516*	2	96.751076	0.8782	2.8988	0.089095	0.0500	0.0106981047
ss86281428		2	96.811087	0.4341	0.0319	0.858299	0.0034	0.0016848454
ss61542083	*rs41640507*	2	96.890597	0.1461	0.5171	0.472303	0.0192	0.0047910389
ss117966852		2	96.971208	0.6232	6.9854	0.008403	0.0503	0.0236044824
ss61529200	*rs41633009*	2	97.061784	0.1934	1.0405	0.308070	0.0246	0.0076770136
ss86316097		2	97.168263	0.2042	2.6511	0.103930	0.0379	0.0123139574
ss86282252		2	97.248833	0.6626	3.9782	0.046482	0.0394	0.0176354693
ss86322953		2	97.280973	0.5007	0.4452	0.504858	0.0122	0.0061015687

ss117966635		2	97.364883	0.3044	0.6181	0.432039	0.0158	0.0067063443
ss86313790		2	97.398003	0.3438	3.0384	0.081755	0.0346	0.0156180064
ss86324577		2	97.424735	0.2006	2.5765	0.108912	0.0382	0.0122462855
ss86277378		2	97.477692	0.4004	13.5531	0.000250	0.0675	0.0324322738
ss86296838		2	97.512822	0.4728	8.3687	0.003937	0.0515	0.0256492367
ss86279590		2	97.540835	0.2500	1.1368	0.286691	0.0228	0.0085340901
ss86277420		2	97.610479	0.8904	0.2262	0.634472	0.0142	0.0027654124
ss61519909	*rs41620240*	2	97.692692	0.1254	8.4965	0.003673	0.0805	0.0176497253
ss86336573		2	97.721711	0.5150	0.3028	0.582322	0.0102	0.0050868638
ss117966462		2	97.754555	0.4105	0.1394	0.708943	0.0064	0.0030936274
ss86304629		2	97.836175	0.2400	0.2989	0.584729	0.0118	0.0043013973
ss117975379		2	98.025351	0.8904	0.1083	0.742130	0.0100	0.0019551630
ss86285497		2	98.134048	0.6125	3.6733	0.055699	0.0371	0.0175922994
ss117974904		2	98.163576	0.6046	20.3372	0.000008	0.0816	0.0390336959
ss86313529		2	98.188032	0.3947	20.5456	0.000007	0.0822	0.0392792288
ss86333426		2	98.444258	0.4756	7.7787	0.005431	0.0519	0.0259052851
ss86295473		2	98.519637	0.7314	2.7936	0.095093	0.0349	0.0137324001
ss117966846		2	98.67861	0.8976	1.0290	0.310737	0.0316	0.0058160479
ss61512668	*rs41614472*	2	98.83821	0.2092	5.6879	0.017349	0.0545	0.0180220751
	rs29025245	2	98.897412	0.6805	7.5998	0.005990	0.0544	0.0236706410
ss86305283		2	98.935087	0.7980	4.8145	0.028550	0.0527	0.0170055645
ss86295522		2	98.989284	0.5552	5.6333	0.017893	0.0443	0.0218960447
ss86286045		2	99.062708	0.9384	0.0019	0.965351	0.0017	0.0001961955
ss86319977		2	99.11546	0.3546	13.5919	0.000245	0.0701	0.0321052084
ss61542104	*rs41640528*	2	99.154979	0.6920	3.1307	0.077268	0.0355	0.0151157988
ss61525812	*rs41622515*	2	99.248005	0.5136	0.8456	0.358123	0.0175	0.0087382226
ss86337552		2	99.406944	0.3976	0.0822	0.774361	0.0054	0.0025760067
	rs29023487	2	99.511318	0.2199	1.8070	0.179309	0.0307	0.0105220057
ss61542152	*rs41641875*	2	99.714541	0.1762	1.2536	0.263253	0.0276	0.0080047015
ss61542148	*rs41641871*	2	99.737044	0.3474	1.3709	0.242066	0.0229	0.0103831195
ss61542145	*rs41641868*	2	99.766432	0.0953	0.9214	0.337454	0.0299	0.0051625545
ss86319733		2	99.847367	0.3933	0.3226	0.570230	0.0107	0.0051271284
ss86289470		2	99.893316	0.8309	0.7829	0.376569	0.0220	0.0061691169
ss61542223	*rs41643245*	2	100.11097	0.9076	0.8984	0.343528	0.0307	0.0051436906
ss117966476		2	100.228431	0.1003	0.5355	0.464546	0.0226	0.0040779180
ss61509051	*rs41601115*	2	100.362436	0.2908	2.3828	0.123135	0.0319	0.0131394425
ss117966795		2	100.405368	0.6239	1.9894	0.158853	0.0262	0.0122733606
ss86284419		2	100.514495	0.7128	2.6835	0.101845	0.0341	0.0139705684
ss117966722		2	100.537204	0.1590	12.8431	0.000362	0.0862	0.0230626649
ss86332197		2	100.67793	0.5838	6.8683	0.008966	0.0469	0.0227851509
ss86303381		2	100.705749	0.2128	2.5920	0.107860	0.0376	0.0126049742
ss61542348	*rs41644170*	2	100.819026	0.2407	0.2026	0.652782	0.0094	0.0034368248
ss86325156		2	101.047483	0.4721	11.0067	0.000955	0.0613	0.0305501949
ss86330128		2	101.069301	0.6225	8.6963	0.003295	0.0557	0.0261990252
ss61484173	*rs41584722*	2	101.230934	0.6877	0.1927	0.660851	0.0085	0.0036473589
ss86314478		2	101.272741	0.8080	1.6138	0.204379	0.0293	0.0090959653
ss86274618		2	101.392052	0.6418	0.4238	0.515267	0.0125	0.0057679368

ss86291408		2	101.524612	0.1999	0.9558	0.328585	0.0224	0.0071623831
ss61542129	*rs41641852*	2	101.559183	0.0903	0.6570	0.417915	0.0264	0.0043374586
ss117966890		2	101.605756	0.3080	0.2828	0.595023	0.0101	0.0043262036
ss117963620		2	101.655571	0.4054	0.9234	0.336911	0.0171	0.0082552274
ss61484098	*rs41584649*	2	101.680924	0.5115	2.0808	0.149608	0.0263	0.0131515606
ss86333809		2	101.815166	0.3424	3.2666	0.071136	0.0358	0.0161337069
ss86340239		2	101.883039	0.2830	0.0047	0.945522	0.0014	0.0005856707
ss86338654		2	102.085391	0.6182	0.5983	0.439479	0.0142	0.0067150897
ss86329169		2	102.177746	0.4735	4.9762	0.026016	0.0419	0.0209132170
ss61502273	*rs41605484*	2	102.563958	0.5809	0.0116	0.914430	0.0019	0.0009405605
ss86336156		2	102.666902	0.3345	0.3075	0.579392	0.0107	0.0047485154
ss61542138	*rs41641861*	2	102.736691	0.2729	1.6599	0.198045	0.0255	0.0101104433
ss86322548		2	102.810749	0.8202	3.2327	0.072613	0.0446	0.0131426808
ss86327496		2	102.897432	0.4663	7.0056	0.008309	0.0495	0.0246518190
ss117966870		2	102.930478	0.5186	1.3826	0.240062	0.0210	0.0104770359
ss86293613		2	102.954083	0.7607	3.5216	0.060994	0.0396	0.0144025060
ss86274646		2	103.004093	0.6454	4.8543	0.027904	0.0433	0.0198005541
ss117966832		2	103.202764	0.5337	1.5398	0.215069	0.0223	0.0111151483
ss86298587		2	103.246909	0.2801	0.9180	0.338326	0.0202	0.0081280748
ss86302563		2	103.278762	0.4219	0.0974	0.755041	0.0059	0.0028907461
ss86330723		2	103.335636	0.6662	0.3738	0.541145	0.0121	0.0053800728
ss86329177		2	103.423638	0.4943	16.8644	0.000045	0.0741	0.0370496335
ss86327115		2	103.515098	0.1612	0.3833	0.536057	0.0150	0.0040680567
ss117963584		2	103.573252	0.6182	14.0715	0.000191	0.0704	0.0332413487
ss86292899		2	103.761038	0.3603	0.5317	0.466153	0.0139	0.0063966847
ss86302871		2	104.189727	0.2299	7.9860	0.004849	0.0605	0.0214170939
ss86338515		2	104.389326	0.1583	7.0982	0.007895	0.0661	0.0176073287
ss61493663	*rs41589291*	2	104.64427	0.8438	0.5964	0.440218	0.0194	0.0051185486
ss38327907	*rs29020007*	2	105.044811	0.5552	6.5485	0.010707	0.0462	0.0228368384
	rs29014567	2	105.099933	0.4928	1.7787	0.182748	0.0245	0.0122554823
ss86331726		2	105.16515	0.4234	4.8345	0.028225	0.0413	0.0201594669
ss86334041		2	105.230174	0.3546	1.2729	0.259607	0.0217	0.0099169223
ss61522862	*rs41622731*	2	105.251561	0.8302	3.4738	0.062768	0.0443	0.0124931958
ss86286478		2	105.676779	0.1311	6.7307	0.009676	0.0706	0.0160759876
ss61475297	*rs41573777*	2	105.726486	0.6418	0.3706	0.542885	0.0114	0.0052188690
ss86340993		2	105.865904	0.2357	1.2001	0.273688	0.0239	0.0086027452
ss86304109		2	106.030953	0.5530	9.2244	0.002477	0.0536	0.0265213459
ss86312859		2	106.082927	0.5423	2.8157	0.093796	0.0315	0.0156359985
ss61515171	*rs41612831*	2	106.18544	0.2901	0.1267	0.722036	0.0072	0.0029474316
ss61478813	*rs41577989*	2	106.248615	0.2966	6.3113	0.012223	0.0495	0.0206359337
ss86300856		2	106.291562	0.3066	1.2695	0.260242	0.0223	0.0094877114
ss86334089		2	106.490194	0.7514	1.0557	0.304548	0.0220	0.0082063375
ss86290949		2	106.715006	0.2994	3.0062	0.083388	0.0353	0.0147951442
ss117966875		2	106.93631	0.1311	2.8192	0.093592	0.0473	0.0107643033
ss61526999	*rs41628461*	2	107.333515	0.6182	0.4416	0.506595	0.0126	0.0059447648
ss61542156	*rs41641879*	2	107.470592	0.8130	6.2654	0.012540	0.0596	0.0181259011
ss61467948	*rs41564971*	2	107.60407	0.1977	0.0011	0.973235	0.0008	0.0002462168

ss61530835	*rs41628800*	2	107.645561	0.8596	0.3261	0.568153		0.0155	0.0037379181
ss61542163	*rs41641886*	2	107.679214	0.4606	7.8370	0.005261		0.0509	0.0252692488
ss86313093		2	107.856125	0.1683	14.2913	0.000170		0.0903	0.0252788354
ss61542164	*rs41641887*	2	107.952012	0.3818	5.0628	0.024757		0.0426	0.0201078440
ss61473192	*rs41567611*	2	108.025806	0.9599	6.0501	0.014147		0.1170	0.0090075582
ss86278354		2	108.094094	0.2206	8.3521	0.003972		0.0637	0.0218960648
ss86302370		2	108.236811	0.1175	1.7293	0.188931		0.0367	0.0076166791
ss86337173		2	108.276539	0.1562	7.1555	0.007649		0.0692	0.0182269766
ss86332000		2	108.426462	0.1669	2.5766	0.108908		0.0394	0.0109477803
ss61467955	*rs41564978*	2	108.556922	0.6468	1.3609	0.243787		0.0224	0.0102292102
ss61484129	*rs41584680*	2	108.61098	0.8245	2.1759	0.140638		0.0353	0.0102197100
ss86340298		2	108.647438	0.6261	3.6804	0.055463		0.0373	0.0174693923
ss86303585		2	108.670605	0.2572	3.9930	0.046077		0.0430	0.0164245613
ss86290673		2	108.723589	0.2693	1.9871	0.159087		0.0291	0.0114709950
ss86339957		2	108.756246	0.5766	1.8000	0.180148		0.0250	0.0122176143
ss61467949	*rs41564972*	2	108.781786	0.8066	15.6420	0.000084		0.0897	0.0279953903
ss61484108	*rs41584659*	2	108.823151	0.9054	0.0934	0.759967		0.0099	0.0016959363
ss117963035		2	108.854239	0.1884	8.7448	0.003210		0.0720	0.0220168211
ss86286769		2	109.131559	0.4599	0.0086	0.926141		0.0017	0.0008281870
ss86332841		2	109.195676	0.5107	3.1898	0.074534		0.0323	0.0161399826
ss86316781		2	109.216736	0.4986	3.9905	0.046147		0.0363	0.0181645466
ss61542382	*rs41644202*	2	109.265844	0.7937	0.5473	0.459662		0.0172	0.0056422691
ss86286066		2	109.341279	0.7120	2.4131	0.120776		0.0317	0.0129972826
ss86338803		2	109.425997	0.0824	7.3012	0.007059		0.0914	0.0138193279
ss61542356	*rs41644177*	2	109.956379	0.3861	0.1330	0.715500		0.0068	0.0032341857
ss86299181		2	109.989489	0.3947	4.2458	0.039717		0.0396	0.0189419915
ss61467963	*rs41564986*	2	110.085856	0.7307	3.5705	0.059232		0.0398	0.0156633478
ss86339131		2	110.149194	0.6440	4.5459	0.033348		0.0411	0.0188645278
ss86312466		2	110.209786	0.3059	0.4582	0.498691		0.0136	0.0057780109
ss86313493		2	110.233944	0.2693	1.8454	0.174754		0.0284	0.0111920219
ss65232092	*rs43314908*	2	110.290559	0.2822	11.3652	0.000790		0.0694	0.0281010712
ss117963626		2	110.315909	0.6554	6.6814	0.009945		0.0480	0.0216895935
ss117963598		2	110.348989	0.8775	2.7319	0.098815		0.0481	0.0103374780
ss61484185	*rs41585634*	2	110.375135	0.0752	5.4072	0.020341		0.0834	0.0115981586
ss86290938		2	110.412732	0.0759	6.2039	0.012979		0.0889	0.0124776451
ss86295056		2	110.433737	0.7199	7.4015	0.006680		0.0568	0.0229136143
ss86325415		2	110.454904	0.1812	4.4874	0.034500		0.0504	0.0149720012
ss61484199	*rs41585646*	2	110.631696	0.0802	0.0041	0.949228		0.0022	0.0003231489
ss61484200	*rs41585647*	2	110.66369	0.0838	1.4362	0.231157		0.0408	0.0062727858
ss65232961	*rs43313873*	2	110.755134	0.3582	0.0013	0.971714		0.0007	0.0003133287
ss86331640		2	110.782975	0.7658	1.5301	0.216517		0.0275	0.0098561631
ss117966756		2	110.847873	0.6999	2.2576	0.133414		0.0304	0.0127751808
ss117966807		2	110.965849	0.2736	1.1141	0.291568		0.0222	0.0088085665
ss86338592		2	110.997722	0.7264	1.1141	0.291568		0.0222	0.0088085665
ss86326532		2	111.058281	0.6633	0.0960	0.756829		0.0062	0.0027601632
ss61542402	*rs41644222*	2	111.08936	0.1547	0.0111	0.916120		0.0027	0.0007055276
ss65232974	*rs43313886*	2	111.112447	0.8446	0.0002	0.989469		0.0003	0.0000886531

ss65233005	*rs43313917*	2	111.134961	0.8926	0.7766	0.378500		0.0264	0.0050700498
ss65233038	*rs43315150*	2	111.158169	0.1196	0.6046	0.437101		0.0223	0.0047073890
ss86339575		2	111.184878	0.1676	3.2422	0.072198		0.0452	0.0126158090
ss86309153		2	111.208687	0.4699	0.5213	0.470530		0.0135	0.0067499116
ss86337641		2	111.235373	0.4964	0.4718	0.492407		0.0127	0.0063715134
ss61523301	*rs41619971*	2	111.264655	0.2350	0.0337	0.854401		0.0041	0.0014644480
ss86280185		2	111.296919	0.1117	1.1323	0.287662		0.0315	0.0062461915
ss117963615		2	111.337738	0.8782	0.0229	0.879728		0.0044	0.0009329889
ss86291653		2	111.361389	0.1239	0.2107	0.646339		0.0131	0.0028364876
ss61542411	*rs41644230*	2	111.385309	0.9255	0.9656	0.326110		0.0348	0.0048012336
ss86336237		2	111.426871	0.4628	2.0091	0.156810		0.0275	0.0136697363
ss86313762		2	111.478005	0.1232	0.1094	0.740960		0.0094	0.0020246411
ss65233393	*rs43318503*	2	111.499935	0.0709	0.5003	0.479590		0.0254	0.0033528488
ss65233452	*rs43319460*	2	111.560197	0.7077	0.1032	0.748142		0.0065	0.0027053553
ss86320492		2	111.709743	0.6734	7.1586	0.007636		0.0539	0.0237089597
ss61467966	*rs41564989*	2	111.736094	0.1461	11.2696	0.000831		0.0873	0.0217894494
ss46526654	*rs41257109*	2	111.798062	0.5444	2.7381	0.098431		0.0316	0.0156693102
ss86329573		2	111.946238	0.0666	1.0316	0.310137		0.0367	0.0045600314
ss65232346	*rs43317958*	2	112.019423	0.6203	0.3360	0.562364		0.0110	0.0051614437
ss86334961		2	112.046569	0.8102	0.9556	0.328642		0.0237	0.0072763546
ss86291008		2	112.07635	0.0666	2.6587	0.103436		0.0616	0.0076575004
ss86312718		2	112.138612	0.2335	4.1234	0.042674		0.0441	0.0157877799
ss65233716	*rs43321720*	2	112.161377	0.0802	5.9611	0.014873		0.0837	0.0123458484
ss86303179		2	112.188758	0.9169	6.7218	0.009724		0.0876	0.0133516839
ss65233657	*rs43321661*	2	112.209855	0.1712	8.4082	0.003853		0.0714	0.0202666272
ss86327691		2	112.260394	0.7321	1.6620	0.197762		0.0266	0.0104365593
ss86303543		2	112.280646	0.2693	2.5393	0.111500		0.0337	0.0132680917
	rs29025784	2	112.396634	0.7521	9.8211	0.001798		0.0678	0.0252656456
ss65233604	*rs43320510*	2	112.427042	0.1819	5.3957	0.020474		0.0551	0.0163909738
ss86340674		2	112.534489	0.5559	0.1473	0.701222		0.0071	0.0034971766
ss86340120		2	112.566029	0.8001	1.0237	0.311987		0.0233	0.0074362221
ss61469972	*rs41565969*	2	112.58653	0.1024	0.5283	0.467570		0.0220	0.0040477482
ss86311405		2	112.611834	0.4534	1.2973	0.255098		0.0212	0.0105243963
ss86308738		2	112.633455	0.5458	1.3715	0.241959		0.0219	0.0108372994
ss64012970	*rs42431948*	2	112.666908	0.2815	3.6667	0.055919		0.0390	0.0157655091
ss64012830	*rs42431478*	2	112.736343	0.5602	0.0934	0.760004		0.0058	0.0028719632
ss64012798	*rs42431462*	2	112.757656	0.6913	2.2761	0.131836		0.0302	0.0128902608
ss61542420	*rs41645139*	2	112.868444	0.2464	0.7440	0.388677		0.0181	0.0067228203
ss61509065	*rs41601129*	2	112.955955	0.8696	2.7297	0.098951		0.0457	0.0103518832
ss61542417		2	112.990362	0.5781	0.8871	0.346584		0.0181	0.0088340721
ss86325254		2	113.031963	0.1282	2.9283	0.087484		0.0478	0.0106892351
ss65233800	*rs43322304*	2	113.109388	0.2414	10.6628	0.001147		0.0725	0.0265486799
ss65235128	*rs43318635*	2	113.292089	0.5824	0.3833	0.536021		0.0117	0.0056890852
ss65235114	*rs43317722*	2	113.353897	0.6261	2.1821	0.140074		0.0285	0.0133514622
ss65235105	*rs43317713*	2	113.379748	0.5236	0.0015	0.968874		0.0007	0.0003706298
ss86308108		2	113.40016	0.8059	3.3515	0.067573		0.0428	0.0134047995
ss65235053	*rs43317661*	2	113.429066	0.5602	4.5660	0.032961		0.0402	0.0198208917

ss65234981	*rs43316689*	2	113.453815	0.4033	7.6211	0.005921	0.0523	0.0251506227
ss61542454	*rs41645172*	2	113.572129	0.7514	3.1076	0.078365	0.0374	0.0139712964
ss117966859		2	113.605901	0.2464	3.7199	0.054173	0.0409	0.0151787466
	rs29010055	2	113.632778	0.3832	0.4002	0.527183	0.0124	0.0058470292
ss86336532		2	113.667052	0.8668	1.8801	0.170762	0.0373	0.0086170816
ss65234790	*rs43323794*	2	113.704008	0.9069	3.0476	0.081297	0.0557	0.0094142483
ss61484240	*rs41585687*	2	113.738823	0.6819	1.1128	0.291846	0.0209	0.0090622248
ss65234749	*rs43323753*	2	113.763038	0.3166	8.9024	0.002948	0.0592	0.0256224749
ss61509068	*rs41601532*	2	113.801058	0.3832	0.0390	0.843512	0.0038	0.0018129374
ss65234699	*rs43322903*	2	113.82536	0.5809	0.1308	0.717680	0.0069	0.0033588650
ss86336797		2	113.849262	0.7736	1.9659	0.161331	0.0327	0.0114542131
ss61542441	*rs41645160*	2	113.891546	0.4785	1.3141	0.252039	0.0216	0.0107638858
	rs29018764	2	113.931093	0.5050	0.5127	0.474209	0.0132	0.0066041719
	rs29018607	2	113.984724	0.5000	2.0206	0.155631	0.0263	0.0131604021
ss65234372	*rs43319879*	2	114.056226	0.7170	8.1503	0.004433	0.0569	0.0230690614
ss61502281	*rs41605492*	2	114.077243	0.1203	0.2583	0.611478	0.0142	0.0030093292
ss61542432	*rs41645151*	2	114.101456	0.2980	4.1317	0.042467	0.0407	0.0170466056
ss65234315	*rs43318822*	2	114.123366	0.2973	7.4800	0.006398	0.0569	0.0237925914
ss65234299	*rs43318806*	2	114.144818	0.2077	8.3424	0.003993	0.0660	0.0217213000
ss65234141	*rs43317748*	2	114.273018	0.2629	2.3983	0.121921	0.0320	0.0124019234
ss86284733		2	114.303562	0.1254	0.9313	0.334862	0.0272	0.0059641704
ss63724085	*rs42321997*	2	114.396466	0.7407	0.2369	0.626591	0.0102	0.0039176841
ss63725459	*rs42326252*	2	114.437507	0.6340	1.5615	0.211868	0.0243	0.0112821557
ss63725315	*rs42325605*	2	114.481626	0.3044	0.3014	0.583194	0.0113	0.0048065834
ss63725123	*rs42325541*	2	114.506888	0.5817	3.5857	0.058693	0.0349	0.0169661439
ss86320750		2	114.553299	0.8166	0.2422	0.622776	0.0119	0.0035702665
ss86289373		2	114.592087	0.5021	8.9979	0.002799	0.0552	0.0276039852
ss117966553		2	114.635694	0.5322	7.6219	0.005918	0.0514	0.0255701390
ss86307573		2	114.889415	0.4062	6.6691	0.010013	0.0473	0.0228378606
ss63980409	*rs42415872*	2	114.968298	0.3181	10.5418	0.001223	0.0634	0.0274919538
ss117966883		2	115.024544	0.4821	5.7377	0.016868	0.0435	0.0217432709
ss105254747		2	115.049292	0.4828	5.9504	0.014963	0.0439	0.0219277041
ss117963592		2	115.073914	0.2070	0.0225	0.880689	0.0035	0.0011468500
ss86309384		2	115.12328	0.7830	0.4081	0.523139	0.0145	0.0049179182
ss117966872		2	115.228932	0.3102	0.0010	0.974413	0.0006	0.0002765644
ss86338410		2	115.271152	0.6877	0.0000	0.994398	0.0001	0.0000602843
ss86278583		2	115.386442	0.8675	0.5550	0.456516	0.0205	0.0047187166
ss117963038		2	115.589573	0.1332	0.9520	0.329547	0.0268	0.0061972850
ss61990256	*rs41718762*	2	115.648223	0.3173	0.2865	0.592626	0.0107	0.0046534247
ss61534693	*rs41628842*	2	115.681958	0.6433	9.0333	0.002746	0.0598	0.0274337935
ss117963604		2	115.715356	0.2665	10.1523	0.001506	0.0664	0.0259402220
ss61480504	*rs41579048*	2	115.742594	0.8875	0.9476	0.330667	0.0290	0.0057829123
ss61989758	*rs41716596*	2	115.796375	0.0860	4.4620	0.035012	0.0712	0.0111884630
ss117966530		2	115.880174	0.6590	2.3784	0.123475	0.0302	0.0135682507
ss65550276	*rs43629326*	2	115.907052	0.9126	3.1112	0.078193	0.0586	0.0093495285
ss86299632		2	116.06319	0.8188	0.7321	0.392484	0.0210	0.0062200465
ss65235581	*rs43322687*	2	116.092096	0.8639	0.8172	0.366306	0.0249	0.0058502668

ss65235893	*rs43325193*	2	116.176281	0.0946	0.8125	0.367708	0.0284	0.0048628967
ss61542474	*rs41645191*	2	116.19972	0.1726	5.6326	0.017900	0.0572	0.0163541476
ss86333197		2	116.243968	0.3381	0.5668	0.451790	0.0146	0.0065466593
ss61531379	*rs41626041*	2	116.276141	0.3933	2.9130	0.088314	0.0319	0.0152269766
ss61567016	*rs41659838*	2	116.296776	0.4706	4.8107	0.028614	0.0403	0.0200668524
ss61542482	*rs41645199*	2	116.391872	0.2851	0.4279	0.513230	0.0134	0.0054678317
ss86273694		2	116.462488	0.1633	0.7854	0.375787	0.0221	0.0060272651
ss65236474	*rs41256296*	2	116.514608	0.1963	6.3178	0.012178	0.0565	0.0178386640
ss86293200		2	116.542695	0.3890	1.2298	0.267825	0.0204	0.0096934354
ss86274321		2	116.577788	0.5272	0.5760	0.448122	0.0137	0.0068212895
ss46526950	*rs41257396*	2	116.628362	0.8446	3.0554	0.080912	0.0437	0.0114688136
ss86286350		2	116.687684	0.4570	2.3773	0.123568	0.0281	0.0139237406
ss65236834	*rs43321227*	2	116.713948	0.2729	1.1715	0.279475	0.0232	0.0092256344
ss61542484	*rs41645201*	2	116.748865	0.2364	1.9196	0.166348	0.0301	0.0108490068
ss86284964		2	116.797851	0.1905	7.6244	0.005910	0.0625	0.0192825723
ss65237091	*rs43320289*	2	116.824608	0.5501	0.2794	0.597257	0.0099	0.0049161687
ss86318216		2	116.893897	0.1683	2.5029	0.114093	0.0395	0.0110656214
ss65237120	*rs43320318*	2	116.935081	0.0860	1.6954	0.193328	0.0420	0.0066022504
ss86332502		2	117.080477	0.4735	0.3080	0.579085	0.0105	0.0052324838
ss61519960	*rs41620284*	2	117.183204	0.4269	4.0314	0.045047	0.0371	0.0181600571
ss61519962	*rs41620286*	2	117.205206	0.4269	4.2721	0.039111	0.0383	0.0187433242
ss105263239		2	117.246368	0.5695	0.0327	0.856652	0.0034	0.0016651573
ss86335770		2	117.341937	0.8818	7.0980	0.007895	0.0750	0.0156336798
ss86298065		2	117.378824	0.3245	0.0103	0.919078	0.0020	0.0008899480
ss86274088		2	117.422437	0.1060	7.7121	0.005633	0.0814	0.0154323191
ss86302881		2	117.446981	0.8718	8.0146	0.004774	0.0778	0.0174030996
ss117963576		2	117.480046	0.2958	0.0126	0.910691	0.0024	0.0009822903
ss86298024		2	117.513325	0.6476	1.9920	0.158575	0.0275	0.0125542077
	rs29019168	2	117.662338	0.7049	0.0064	0.936498	0.0017	0.0007030362
ss63652036	*rs42301516*	2	117.709717	0.5509	2.6113	0.106559	0.0302	0.0149631598
ss61568829	*rs41666711*	2	117.757197	0.6032	3.6471	0.056577	0.0353	0.0168885178
ss61505741	*rs41608876*	2	117.783712	0.1920	0.5079	0.476285	0.0171	0.0053113911
ss86304423		2	117.84942	0.3166	6.4428	0.011357	0.0516	0.0223129350
	rs29019665	2	117.878756	0.6755	8.5465	0.003574	0.0592	0.0259725684
ss64712049	*rs42805646*	2	117.90265	0.3238	8.4548	0.003757	0.0589	0.0257926095
ss61473742	*rs41574058*	2	117.923887	0.0752	1.4678	0.226098	0.0436	0.0060618479
ss65007272	*rs43103216*	2	117.954413	0.9277	2.0453	0.153122	0.0522	0.0070098153
	rs29027121	2	117.977208	0.3539	1.5699	0.210644	0.0242	0.0110606444
ss86299733		2	118.007325	0.2049	0.4408	0.506953	0.0150	0.0048969131
ss64001414	*rs42425010*	2	118.053302	0.5881	3.7015	0.054772	0.0366	0.0177179424
ss86337655		2	118.074074	0.1676	0.4053	0.524596	0.0160	0.0044618454
ss105263245		2	118.107711	0.6325	0.1400	0.708415	0.0071	0.0032832987
ss61527057	*rs41628517*	2	118.141998	0.8782	0.0266	0.870551	0.0046	0.0009836534
ss86302832		2	118.170579	0.8782	0.0266	0.870551	0.0046	0.0009836534
ss86306550		2	118.203477	0.7564	0.1577	0.691384	0.0083	0.0030661694
ss61527062	*rs41628521*	2	118.232623	0.1053	4.5535	0.033200	0.0628	0.0118350293
ss117966465		2	118.264372	0.6633	0.0484	0.825893	0.0044	0.0019570983

ss86290582		2	118.303895	0.9234	5.5158	0.019125	0.0819	0.0115929892
ss61511975	*rs41609685*	2	118.332621	0.6390	0.3854	0.534916	0.0120	0.0055485095
ss86274686		2	118.357634	0.8403	2.9668	0.085435	0.0432	0.0115891914
ss61497094	*rs41594024*	2	118.427258	0.3374	1.9157	0.166770	0.0273	0.0121977647
ss86290982		2	118.468145	0.4513	7.9834	0.004856	0.0530	0.0262712150
ss86298054		2	118.498848	0.4778	17.5549	0.000032	0.0808	0.0403209484
ss61493088	*rs41592634*	2	118.562717	0.1533	1.7879	0.181617	0.0348	0.0090460842
ss61472950	*rs41575169*	2	118.617225	0.3746	4.0769	0.043856	0.0396	0.0185597862
ss61472961	*rs41575180*	2	118.640109	0.1676	0.9529	0.329333	0.0237	0.0066077785
ss61530348	*rs41624633*	2	118.685524	0.4176	0.1092	0.741191	0.0063	0.0030629375
ss86336185		2	118.720371	0.9384	4.5447	0.033371	0.0810	0.0093659470
ss64084689	*rs42466843*	2	118.775917	0.7020	9.1907	0.002523	0.0631	0.0263904949
ss64084739	*rs42466868*	2	118.803647	0.9026	0.5888	0.443125	0.0238	0.0041795446
ss64084653	*rs42466325*	2	118.85703	0.7872	0.1024	0.749008	0.0070	0.0023607945
ss86275135		2	118.886838	0.8868	11.4557	0.000753	0.0976	0.0195931606
ss61473899	*rs41574408*	2	118.955564	0.1583	0.7246	0.394928	0.0215	0.0057221199
		2	118.990927	0.5824	0.1522	0.696561	0.0073	0.0035658867
ss61473901	*rs41574410*	2	119.028634	0.4155	0.0022	0.962791	0.0009	0.0004186893
ss86301705		2	119.086979	0.4341	0.0540	0.816320	0.0043	0.0021019914
ss86325465		2	119.235993	0.5831	0.5380	0.463522	0.0136	0.0066044815
ss61532235	*rs41634246*	2	119.309504	0.1332	2.1491	0.143107	0.0392	0.0090606651
ss61514897	*rs41617152*	2	119.359941	0.5809	0.0432	0.835397	0.0039	0.0018876146
ss86274115		2	119.496312	0.2701	2.5867	0.108221	0.0333	0.0131261963
ss86317434		2	119.552373	0.3016	3.5040	0.061640	0.0386	0.0162416944
ss86330232		2	119.573259	0.4635	2.7558	0.097355	0.0312	0.0154919723
ss117963589		2	119.698804	0.6963	5.2018	0.022864	0.0480	0.0202821424
ss61544349	*rs41643747*	2	119.895762	0.1734	2.3694	0.124190	0.0367	0.0105138499
ss117963637		2	119.918132	0.1483	2.4824	0.115583	0.0407	0.0102824549
ss86335588		2	119.962497	0.4821	0.2076	0.648815	0.0084	0.0041762728
ss61542501	*rs41645218*	2	120.168469	0.4284	4.4578	0.035097	0.0396	0.0193841219
ss65237777	*rs43327273*	2	120.189318	0.4234	0.3076	0.579317	0.0101	0.0049463023
ss61542506	*rs41645223*	2	120.267155	0.9362	0.0476	0.827280	0.0083	0.0009913914
ss61484278	*rs41578131*	2	120.340093	0.3689	0.1424	0.706008	0.0074	0.0034309660
ss86314103		2	120.424437	0.9384	1.9467	0.163393	0.0551	0.0063715880
ss86331956		2	120.464241	0.5093	0.3551	0.551451	0.0109	0.0054723934
	rs29009608	2	120.518404	0.5924	0.0016	0.968392	0.0008	0.0003644334
ss61515248	*rs41612906*	2	120.554693	0.6884	0.6565	0.418061	0.0167	0.0071839714
ss86321954		2	120.628723	0.8223	12.9390	0.000345	0.0852	0.0249061669
ss86292553		2	120.661212	0.4678	0.0311	0.860072	0.0033	0.0016453347
ss61484288	*rs41578141*	2	120.709811	0.4348	0.0028	0.957896	0.0010	0.0004884551
ss86305163		2	120.769798	0.8574	1.3819	0.240174	0.0310	0.0075765898
ss86278423		2	120.799058	0.7450	7.5715	0.006084	0.0581	0.0220675893
ss61513550	*rs41613018*	2	120.833843	0.9040	2.9721	0.085157	0.0531	0.0092198464
ss86335872		2	120.857158	0.1146	0.7174	0.397278	0.0247	0.0050130243
ss61507219	*rs41579391*	2	120.986557	0.4105	1.3375	0.247880	0.0222	0.0107342789
ss61496322	*rs41598273*	2	121.06897	0.1160	2.0621	0.151453	0.0429	0.0087933497
	rs29020417	2	121.092177	0.9305	1.0678	0.301793	0.0384	0.0049621802

ss105254780		2	121.117976	0.8181	0.0990	0.753073	0.0077	0.0022880623
ss61466622	*rs41571274*	2	121.279607	0.9148	0.0245	0.875666	0.0053	0.0008278179
ss61476833	*rs41579284*	2	121.321453	0.3761	0.1571	0.691974	0.0076	0.0035757302
ss61527233		2	121.3507	0.0881	1.4498	0.228971	0.0389	0.0062487063
ss86301556		2	121.425825	0.4964	0.2786	0.597770	0.0098	0.0049126344
ss61476831	*rs41579282*	2	121.46102	0.4334	2.5805	0.108643	0.0296	0.0145403494
ss86297381		2	122.11351	0.2135	0.1452	0.703305	0.0085	0.0028507618
ss61542535	*rs41646050*	2	122.164209	0.1454	4.0856	0.043631	0.0539	0.0133961821
ss86338449		2	122.194521	0.3868	0.1046	0.746481	0.0062	0.0029510839
ss86323478		2	122.294602	0.1519	1.2964	0.255257	0.0286	0.0073723508
ss86316232		2	122.338918	0.2894	2.9022	0.088904	0.0339	0.0139239648
ss86340702		2	122.364401	0.4448	1.8504	0.174177	0.0246	0.0121344751
ss105251607		2	122.406133	0.5552	1.8504	0.174177	0.0246	0.0121344751
ss61484304	*rs41578157*	2	122.440316	0.7643	3.8274	0.050822	0.0422	0.0152152601
ss86334438		2	122.503441	0.3503	7.2789	0.007146	0.0509	0.0231841284
ss86339790		2	122.54911	0.8109	2.4213	0.120153	0.0361	0.0110568037
	rs29011523	2	122.576369	0.2815	6.3229	0.012144	0.0518	0.0209712329
	rs29013679	2	122.730627	0.4212	0.2804	0.596603	0.0099	0.0048491766
ss86326783		2	122.853612	0.6984	0.9158	0.338917	0.0193	0.0081337811
ss86321083		2	122.878976	0.3388	0.0634	0.801292	0.0050	0.0022427492
ss86325569		2	122.905177	0.1189	0.0380	0.845496	0.0055	0.0011608792
ss86328344		2	122.960704	0.3438	0.9502	0.330001	0.0190	0.0085941168
	rs29026778	2	122.99567	0.2206	3.0966	0.078895	0.0385	0.0132500608
ss86320147		2	123.209779	0.2715	0.6769	0.410951	0.0170	0.0067426373
ss86341267		2	123.371915	0.5158	0.4202	0.517062	0.0119	0.0059513923
ss61507373	*rs41605667*	2	123.400703	0.5630	1.4231	0.233295	0.0220	0.0108415079
ss86292649		2	123.462478	0.4620	2.3381	0.126695	0.0280	0.0139072104
ss86297040		2	123.499836	0.4606	0.4198	0.517258	0.0121	0.0059928801
ss86302710		2	123.526061	0.7278	0.1198	0.729394	0.0072	0.0028705053
ss61542755	*rs41636478*	2	123.657396	0.7500	4.6819	0.030822	0.0450	0.0168742922
ss86338458		2	123.70595	0.6361	4.0279	0.045139	0.0380	0.0175947470
ss61484383	*rs41579136*	2	123.727853	0.2142	6.0935	0.013807	0.0542	0.0182462315
ss61542579	*rs41646094*	2	123.815849	0.8195	0.4039	0.525299	0.0151	0.0044664519
ss61542717	*rs41636441*	2	123.898739	0.4176	1.0264	0.311358	0.0191	0.0092981872
ss86331247		2	124.020338	0.4685	0.3455	0.556846	0.0109	0.0054288179
ss61509093	*rs41601555*	2	124.130278	0.8087	0.3499	0.554377	0.0141	0.0043497172
ss86320642		2	124.216287	0.2142	0.0264	0.870913	0.0036	0.0012089646
ss86335534		2	124.293575	0.3532	0.1949	0.659036	0.0084	0.0038573062
ss86313323		2	124.32998	0.3474	0.0070	0.933537	0.0016	0.0007163143
ss61478994	*rs41579865*	2	124.480807	0.8345	1.4890	0.222787	0.0304	0.0083976938
ss117966877		2	124.585179	0.8374	1.9102	0.167387	0.0346	0.0094112835
ss61542570	*rs41646085*	2	124.617273	0.4155	4.4415	0.035432	0.0388	0.0188690041
ss61542567	*rs41646082*	2	124.643625	0.2600	0.0932	0.760283	0.0063	0.0024284586
ss86286415		2	124.676202	0.8209	1.6120	0.204634	0.0304	0.0089265101
ss86336992		2	124.710942	0.8166	0.8238	0.364393	0.0216	0.0064788706
ss86302466		2	124.741357	0.8374	0.6013	0.438340	0.0188	0.0051176537
ss86337764		2	124.785385	0.7364	1.0868	0.297538	0.0217	0.0084102181

ss86314333		2	124.829244	0.2600	1.2350	0.266821	0.0231	0.0088813942
ss117963623		2	124.856725	0.6705	3.5748	0.059079	0.0360	0.0158995068
ss86322876		2	125.004433	0.2658	1.9636	0.161579	0.0287	0.0111835743
ss86333936		2	125.061997	0.2128	2.2792	0.131574	0.0344	0.0115373683
ss86308266		2	125.085918	0.2564	0.2486	0.618251	0.0105	0.0040075750
ss117966677		2	125.201301	0.3102	0.2505	0.616907	0.0101	0.0043091852
ss86295693		2	125.382334	0.2228	0.9428	0.331900	0.0220	0.0076347503
ss61476302	*rs41575563*	2	125.408764	0.3990	0.2555	0.613370	0.0095	0.0045783573
ss61542753	*rs41636477*	2	125.453516	0.2342	1.2025	0.273211	0.0245	0.0087847443
ss86292432		2	125.84346	0.3438	0.5792	0.446880	0.0154	0.0069570515
ss86289625		2	125.894203	0.0845	0.0641	0.800201	0.0085	0.0013215249
ss61472137	*rs41567677*	2	125.916751	0.1189	0.2535	0.614797	0.0144	0.0030218077
ss86332047		2	125.942689	0.9448	0.6595	0.417003	0.0337	0.0035088074
ss38325723	*rs29011432*	2	125.985733	0.9563	0.0366	0.848307	0.0088	0.0007347247
	rs29027359	2	126.1416	0.0874	0.0063	0.936863	0.0026	0.0004129266
ss46526209	*rs41255349*	2	126.210851	0.3639	3.2835	0.070412	0.0346	0.0160092588
ss61484402	*rs41579155*	2	126.342428	0.8087	0.3670	0.544854	0.0142	0.0044069540
ss86296742		2	126.427545	0.2572	3.9317	0.047778	0.0432	0.0164872345
ss86330831		2	126.58295	0.3596	4.4830	0.034587	0.0414	0.0190790231
ss86302999		2	126.622213	0.0480	1.8065	0.179370	0.0573	0.0052396962
ss86327014		2	126.657038	0.1289	0.2614	0.609327	0.0141	0.0031712871
ss117966874		2	126.677801	0.2543	0.0112	0.915754	0.0022	0.0008495759
ss86327783		2	126.704973	0.8295	3.4340	0.064289	0.0468	0.0132342967
ss86305122		2	126.726985	0.0516	4.5748	0.032793	0.0911	0.0089106574
ss86333052		2	126.761889	0.8331	7.6421	0.005853	0.0674	0.0187486752
ss61542797	*rs41636520*	2	126.793966	0.4248	8.3276	0.004026	0.0535	0.0261209272
ss86338345		2	126.852879	0.6268	0.3310	0.565275	0.0109	0.0050902845
ss86301176		2	126.895289	0.6891	0.2525	0.615451	0.0098	0.0042034887
ss86337566		2	126.995306	0.3102	0.0023	0.961522	0.0009	0.0004035429
ss86316108		2	127.048881	0.7156	2.6494	0.104044	0.0339	0.0138130406
ss86326518		2	127.078577	0.4004	0.4495	0.502817	0.0127	0.0061186777
ss86299503		2	127.151023	0.3360	3.3846	0.066234	0.0364	0.0162294126
ss86340336		2	127.172772	0.4327	1.8848	0.170234	0.0260	0.0127400685
ss61542786	*rs41636509*	2	127.202756	0.6597	0.4112	0.521560	0.0122	0.0054642251
ss86311731		2	127.229859	0.1526	0.1152	0.734415	0.0085	0.0022048237
ss105254768		2	127.263786	0.5430	1.2415	0.265558	0.0202	0.0100313238
ss86313571		2	127.324654	0.1791	2.8941	0.089352	0.0403	0.0118616047
ss86292145		2	127.353273	0.4850	1.2679	0.260540	0.0203	0.0101614658
ss86293987		2	127.414822	0.3095	2.0666	0.151009	0.0274	0.0117203005
ss86285154		2	127.474015	0.5752	0.1062	0.744562	0.0060	0.0029538146
ss117963594		2	127.508329	0.4040	8.6407	0.003396	0.0531	0.0255881362
ss86292908		2	127.531138	0.5423	4.0639	0.044192	0.0362	0.0179672305
ss86310438		2	127.559619	0.0989	0.2687	0.604365	0.0159	0.0028250586
ss86289094		2	127.594125	0.6032	2.5749	0.109021	0.0306	0.0146720398
ss86292824		2	127.63878	0.4642	2.4694	0.116535	0.0281	0.0139599391
ss61542811	*rs41636934*	2	127.692028	0.7149	1.2082	0.272065	0.0219	0.0089260826
ss86320887		2	127.831357	0.3102	0.8303	0.362515	0.0176	0.0075362421

ss61480082	*rs41585336*	2	127.937482	0.1125	0.1552	0.693693		0.0117	0.0023405219
ss86304679		2	128.104238	0.2314	1.9303	0.165168		0.0286	0.0101627755
ss86310307		2	128.161004	0.3489	6.7585	0.009528		0.0515	0.0233778075
ss86313548		2	128.256986	0.4355	3.7312	0.053811		0.0354	0.0174059700
ss117966687		2	128.288954	0.3660	1.0876	0.297361		0.0192	0.0089328718
ss86295922		2	128.342763	0.4284	1.5580	0.212385		0.0228	0.0111708615
ss86336650		2	128.387239	0.2471	0.2601	0.610244		0.0110	0.0040756027
ss86294797		2	128.428438	0.7042	5.1070	0.024138		0.0444	0.0185085580
ss86340444		2	128.466579	0.1039	1.4570	0.227815		0.0371	0.0069028253
ss61523307	*rs41619977*	2	128.50888	0.6383	0.2974	0.585672		0.0106	0.0049105550
ss61523309		2	128.679717	0.2572	0.9565	0.328413		0.0207	0.0078909340
ss86304376		2	128.758777	0.1053	0.3784	0.538661		0.0184	0.0034708542
ss86282731		2	128.829278	0.6289	0.1857	0.666665		0.0083	0.0038857609
ss86337589		2	128.962024	0.6418	0.0840	0.772095		0.0055	0.0025505683
ss61542823	*rs41636946*	2	128.98729	0.8610	1.0110	0.315024		0.0268	0.0064168710
ss86277866		2	129.008833	0.2679	0.3078	0.579242		0.0112	0.0044060735
ss105255458		2	129.080262	0.6741	0.0495	0.824033		0.0043	0.0018998440
ss86322302		2	129.167684	0.5444	7.7540	0.005505		0.0511	0.0253266068
	rs29024663	2	129.214484	0.8066	4.4769	0.034711		0.0487	0.0151895472
ss46526471	*rs41256934*	2	129.235649	0.6046	2.0596	0.151703		0.0280	0.0133878531
ss86335662		2	129.269182	0.2923	1.7138	0.190924		0.0266	0.0110092085
	rs29020940	2	129.308355	0.5279	8.8049	0.003107		0.0545	0.0271792818
ss86336051		2	129.364891	0.7278	0.0072	0.932380		0.0017	0.0006893089
ss86309575		2	129.390724	0.1705	0.0243	0.876255		0.0037	0.0010599030
ss61484415	*rs41579167*	2	129.419061	0.6848	2.5876	0.108156		0.0312	0.0134706108
ss61484411	*rs41579163*	2	129.445573	0.8195	0.0017	0.967163		0.0010	0.0003088968
ss38331317	*rs29025431*	2	129.578916	0.4986	3.2838	0.070399		0.0329	0.0164340841
ss86306419		2	129.618579	0.9405	0.0260	0.871935		0.0064	0.0007144468
ss86340834		2	129.672569	0.1891	0.6186	0.431827		0.0180	0.0055105178
ss86341048		2	129.702918	0.6067	0.0013	0.970743		0.0007	0.0003436531
ss86314466		2	129.740746	0.1970	0.1276	0.720992		0.0081	0.0025642848
ss61542819	*rs41636942*	2	129.772768	0.8825	0.3651	0.545873		0.0176	0.0036490846
ss86337876		2	129.864242	0.8037	0.1282	0.720363		0.0081	0.0025645625
ss86338841		2	129.88505	0.6225	4.1161	0.042858		0.0375	0.0176224745
ss86318692		2	129.955452	0.1805	0.0994	0.752586		0.0076	0.0022607157
ss86302988		2	130.01527	0.1196	13.4489	0.000264		0.1037	0.0218451424
ss65240024	*rs43323909*	2	130.055151	0.8295	0.0596	0.807259		0.0060	0.0016860611
ss117963634		2	130.326976	0.8653	1.8934	0.169262		0.0366	0.0085290891
ss86274537		2	130.394179	0.8288	6.3171	0.012183		0.0596	0.0169272929
ss86321679		2	130.438552	0.0516	0.0529	0.818213		0.0095	0.0009326342
ss117966544		2	130.46279	0.8073	1.0782	0.299460		0.0239	0.0074388828
ss86295200		2	130.490128	0.5229	0.6638	0.415517		0.0153	0.0076349372
ss86337533		2	130.62815	0.8467	0.0953	0.757617		0.0078	0.0020327358
ss86305688		2	130.717728	0.4148	0.7841	0.376209		0.0164	0.0079504600
ss86313988		2	130.824576	0.0537	0.3090	0.578469		0.0221	0.0022441486
ss86339539		2	130.863224	0.6726	1.9900	0.158786		0.0271	0.0119173403
ss86307752		2	130.891402	0.6139	2.4633	0.116988		0.0295	0.0139706924

ss86339841		2	130.979447	0.9241	0.7021	0.402381		0.0297	0.0041681539
ss86335425		2	131.044486	0.1920	0.5272	0.468034		0.0169	0.0052470625
ss86325404		2	131.080917	0.8181	0.0025	0.960348		0.0012	0.0003513476
ss105260461		2	131.142175	0.3324	0.1420	0.706446		0.0073	0.0032549515
ss117975271		2	131.195034	0.9183	0.5686	0.451069		0.0254	0.0038163998
ss86305344		2	131.219622	0.3639	0.9050	0.341768		0.0185	0.0085415384
ss86323324		2	131.280873	0.4957	0.0048	0.944651		0.0012	0.0006214952
ss86322079		2	131.317785	0.7443	0.3018	0.582907		0.0117	0.0044630474
ss86339577		2	131.449289	0.7400	5.6161	0.018068		0.0491	0.0189078657
ss105260774		2	131.480672	0.3625	6.4036	0.011608		0.0477	0.0220317553
ss86298710		2	131.501011	0.4871	4.8948	0.027262		0.0412	0.0205930735
ss86341244		2	131.588874	0.1719	2.2183	0.136840		0.0366	0.0104227796
ss86315553		2	131.69024	0.3438	0.3207	0.571358		0.0109	0.0049218037
ss86288660		2	131.74609	0.6877	9.6043	0.002019		0.0603	0.0258988683
ss86310498		2	131.782872	0.2987	0.3478	0.555579		0.0117	0.0048880492
ss86329900		2	131.95746	0.3503	1.8632	0.172701		0.0258	0.0117226320
ss86285781		2	132.059302	0.2586	0.2666	0.605758		0.0106	0.0040733472
ss86327810		2	132.210344	0.7822	0.5201	0.471025		0.0160	0.0054627918
ss61467172	*rs41565603*	2	132.244416	0.5516	3.3146	0.069094		0.0344	0.0170212294
ss86323085		2	132.265351	0.0616	0.2710	0.602836		0.0199	0.0022976857
ss86275918		2	132.291338	0.1683	1.0645	0.302548		0.0265	0.0074262617
ss86340584		2	132.335652	0.7128	3.1773	0.075103		0.0390	0.0159803578
ss86338090		2	132.387247	0.5136	9.9355	0.001691		0.0588	0.0293625569
ss86294666		2	132.408866	0.8453	3.9269	0.047912		0.0514	0.0134329917
ss86294698		2	132.442463	0.2894	4.2544	0.039519		0.0417	0.0171528082
ss86334478		2	132.476557	0.8295	0.0826	0.773891		0.0072	0.0020397148
ss86298707		2	132.504979	0.4162	2.1950	0.138913		0.0275	0.0133418915
ss61533029	*rs41629000*	2	132.707748	0.4105	10.7571	0.001091		0.0617	0.0298445989
ss86335919		2	132.731274	0.5960	8.8789	0.002985		0.0567	0.0272934635
ss86293018		2	132.765641	0.6332	13.0971	0.000317		0.0681	0.0316219342
ss86320968		2	132.791347	0.5315	8.6646	0.003353		0.0545	0.0271463947
ss86321326		2	132.842155	0.8352	0.5018	0.478961		0.0180	0.0049408736
ss86308575		2	132.871189	0.2715	8.7467	0.003207		0.0620	0.0245091794
ss86338754		2	133.007841	0.1218	1.2444	0.265003		0.0317	0.0067827506
	rs29012608	2	133.058385	0.5659	5.2038	0.022839		0.0421	0.0206649302
	rs29019426	2	133.195302	0.2779	1.3906	0.238703		0.0248	0.0099669619
ss86294385		2	133.234786	0.4262	5.5980	0.018254		0.0446	0.0217997710
ss86293518		2	133.26971	0.3668	3.4630	0.063179		0.0359	0.0166630012
ss61542888	*rs41637011*	2	133.361283	0.3352	0.5368	0.464020		0.0146	0.0065296071
ss86339782		2	133.401204	0.9162	0.5110	0.474946		0.0240	0.0036794554
ss86340679		2	133.454711	0.3632	0.0458	0.830527		0.0041	0.0018983780
ss86319600		2	133.522698	0.4835	0.9340	0.334173		0.0182	0.0090976569
ss86340401		2	133.583088	0.4434	3.1288	0.077360		0.0333	0.0164556936
ss86336891		2	133.613126	0.5043	4.4674	0.034904		0.0398	0.0199083737
ss86336427		2	133.688957	0.4592	0.3479	0.555504		0.0109	0.0054048988
ss86313580		2	133.769183	0.1691	0.0067	0.934844		0.0020	0.0005586535
ss86328292		2	133.789399	0.5387	0.0542	0.816061		0.0043	0.0021446545

ss86330236		2	133.872089	0.3403	0.8103	0.368351	0.0178	0.0080101081
ss61542901	*rs41637024*	2	133.894183	0.5100	0.0060	0.938257	0.0014	0.0007179468
ss86320375		2	133.917411	0.4713	0.1069	0.743818	0.0061	0.0030275624
ss61484449	*rs41579201*	2	133.982375	0.6999	0.0825	0.774061	0.0057	0.0024008929
ss86315136		2	134.027686	0.6082	4.1858	0.041138	0.0386	0.0183962402
ss86337519		2	134.059896	0.5208	12.3987	0.000458	0.0648	0.0323414760
ss61484450	*rs41579202*	2	134.199188	0.1132	8.4611	0.003744	0.0853	0.0171230531
ss86300519		2	134.293576	0.5544	5.5698	0.018548	0.0447	0.0220919048
ss86286519		2	134.352484	0.7292	3.1297	0.077314	0.0377	0.0148856179
ss86304037		2	134.495781	0.9241	2.1770	0.140543	0.0507	0.0071089741
ss86336589		2	134.636107	0.6683	0.9404	0.332504	0.0192	0.0084920692
ss86335030		2	134.666758	0.6784	0.1968	0.657495	0.0088	0.0038537338
ss86338371		2	134.729836	0.7256	1.7986	0.180316	0.0287	0.0114294999
ss86296992		2	134.761842	0.2185	1.9426	0.163830	0.0312	0.0106494566
ss86302880		2	134.790383	0.3517	7.0945	0.007911	0.0516	0.0235477409
ss86293038		2	134.844418	0.5931	1.8924	0.169379	0.0259	0.0125111054
ss86337204		2	134.893756	0.7307	3.0479	0.081285	0.0370	0.0145743860
ss86284795		2	134.918981	0.8639	0.1852	0.667081	0.0119	0.0027881426
ss86284348		2	134.965246	0.4835	4.9543	0.026345	0.0410	0.0205017514
ss86325401		2	135.039795	0.1225	0.9503	0.329970	0.0279	0.0059880812
ss86290463		2	135.075825	0.1203	2.3894	0.122616	0.0431	0.0091299842
ss61542917	*rs41637440*	2	135.121889	0.6017	0.1463	0.702229	0.0073	0.0035178172
ss86328871		2	135.148527	0.6046	0.1492	0.699381	0.0075	0.0035745459
ss86320978		2	135.184299	0.6046	0.1492	0.699381	0.0075	0.0035745459
ss117963573		2	135.226321	0.3195	0.0157	0.900349	0.0025	0.0011045580
ss65110417	*rs43197278*	2	135.249631	0.3775	0.3071	0.579617	0.0107	0.0050122930
ss65241846	*rs43328895*	2	135.270523	0.3080	3.6781	0.055541	0.0384	0.0163664885
ss86338111		2	135.33578	0.6383	0.7165	0.397574	0.0163	0.0075481936
ss86294352		2	135.379063	0.4384	1.8533	0.173842	0.0253	0.0124382451
ss86288075		2	135.414341	0.5501	4.1332	0.042429	0.0376	0.0185900538
ss86326092		2	135.570789	0.2178	0.5239	0.469439	0.0170	0.0057781253
ss86307061		2	135.609164	0.2514	0.3184	0.572773	0.0116	0.0043719036
ss63860260	*rs42392149*	2	135.629696	0.6275	0.1981	0.656429	0.0088	0.0041100673
ss61519815	*rs41618854*	2	135.680207	0.4291	0.8065	0.369454	0.0178	0.0087352093
ss61519821	*rs41618860*	2	135.720865	0.7113	6.1441	0.013421	0.0498	0.0204383207
ss64994565	*rs43085211*	2	135.75196	0.6153	2.1166	0.146161	0.0277	0.0131041178
ss86321108		2	135.776021	0.3145	1.4804	0.224124	0.0248	0.0106841789
ss86341230		2	135.810007	0.6447	2.2791	0.131580	0.0298	0.0136348482
ss86274725		2	135.837462	0.3238	8.0860	0.004592	0.0558	0.0244250380
ss86313478		2	135.897721	0.3883	0.9478	0.330623	0.0191	0.0090671684
ss61484463	*rs41579215*	2	135.962795	0.7486	0.4222	0.516039	0.0136	0.0051126567
ss61542929		2	136.03674	0.5716	6.5768	0.010540	0.0471	0.0230546644
ss86340876		2	136.291626	0.9398	11.4077	0.000772	0.1286	0.0145488381
ss65242187	*rs43326840*	2	136.341159	0.3832	0.0397	0.842087	0.0039	0.0018413610
ss62369003	*rs41852294*	2	136.38108	0.8324	0.3061	0.580253	0.0137	0.0038251034
ss86339807		2	136.424421	0.3854	0.4436	0.505591	0.0133	0.0062861878
ss86290392		2	136.467884	0.6239	1.1791	0.277911	0.0203	0.0095216447

ss86321360		2	136.533208	0.4764	0.5666	0.451856		0.0136	0.0068068759
ss86319718		2	136.625095	0.2994	1.2162	0.270485		0.0226	0.0094610578
ss86332004		2	136.680529	0.2242	0.0889	0.765628		0.0065	0.0022600202
ss86285686		2	136.766495	0.1762	0.6050	0.436957		0.0191	0.0055381359
ss86275049		2	136.946255	0.2006	0.1319	0.716625		0.0084	0.0027076196
ss64752545	*rs42850477*	2	136.970604	0.2077	0.0375	0.846460		0.0044	0.0014567283
ss86340663		2	137.001335	0.6354	2.2915	0.130540		0.0287	0.0133112848
ss86339416		2	137.038076	0.4033	0.8364	0.360740		0.0173	0.0083454753
ss86285137		2	137.073322	0.5595	0.2566	0.612628		0.0094	0.0046482620
ss105261409		2	137.116058	0.3961	0.3781	0.538797		0.0119	0.0056793898
ss86320424		2	137.171087	0.3424	0.1028	0.748611		0.0063	0.0028236020
ss86338964		2	137.195726	0.6497	0.4000	0.527289		0.0119	0.0053996830
ss86323980		2	137.217212	0.8030	0.7645	0.382213		0.0210	0.0066579902
ss86286381		2	137.256834	0.3281	0.0294	0.863844		0.0033	0.0014754640
ss86338333		2	137.307257	0.1605	0.1853	0.667031		0.0113	0.0030416359
ss86307009		2	137.360384	0.5365	2.5166	0.113104		0.0308	0.0153276654
ss64680173	*rs42776828*	2	137.385204	0.1547	0.0068	0.934164		0.0021	0.0005507300
ss64220130	*rs42532688*	2	137.508144	0.1683	0.0901	0.764088		0.0075	0.0020923978
ss86293553		2	137.539568	0.2020	0.0450	0.832007		0.0051	0.0016425753
ss86319165		2	137.603672	0.3517	0.9059	0.341542		0.0188	0.0085916687
ss86337414		2	137.634388	0.3868	0.2292	0.632285		0.0090	0.0042474804
ss86289241		2	137.654941	0.6483	1.4534	0.228393		0.0237	0.0108265524
ss86298881		2	137.692159	0.9112	0.0059	0.938859		0.0025	0.0004039139
ss86336559		2	137.779519	0.0817	1.9958	0.158186		0.0470	0.0070424324
ss65231620	*rs43321737*	2	137.799962	0.1304	0.6715	0.412825		0.0234	0.0053162404
ss86337323		2	137.851764	0.5208	0.4438	0.505496		0.0125	0.0062355734
ss86303212		2	137.876529	0.7901	3.4797	0.062547		0.0432	0.0143341417
ss86296543		2	137.896728	0.1032	3.9053	0.048530		0.0593	0.0109798778
ss86338015		2	137.924405	0.3109	5.3355	0.021187		0.0466	0.0199753811
ss86338965		2	137.949517	0.6669	2.4331	0.119249		0.0309	0.0137198235
ss86303217		2	137.976078	0.3718	6.4521	0.011298		0.0488	0.0227856819
ss86297299		2	138.018047	0.5372	2.6926	0.101265		0.0309	0.0153737325
ss86322326		2	138.092598	0.8123	3.7049	0.054659		0.0457	0.0139207758
ss117966678		2	138.137067	0.2428	6.5327	0.010802		0.0553	0.0203344667
ss86324110		2	138.200095	0.1698	0.2558	0.613151		0.0124	0.0035032719
ss86303518		2	138.247988	0.1347	1.4185	0.234055		0.0325	0.0075665624
ss86299710		2	138.283629	0.2693	4.0935	0.043430		0.0414	0.0163025629
	rs29014082	2	138.317818	0.3231	1.2981	0.254944		0.0233	0.0102095664
ss86274704		2	138.345708	0.4319	1.3276	0.249635		0.0218	0.0106910721
ss86325947		2	138.367827	0.6189	0.0391	0.843302		0.0038	0.0017874055
ss86316635		2	138.38981	0.3861	0.0187	0.891361		0.0027	0.0012683741
ss86291420		2	138.421341	0.0946	0.8463	0.357925		0.0278	0.0047596440
ss86304298		2	138.453345	0.4441	0.0503	0.822558		0.0042	0.0020694066
ss86337710		2	138.475645	0.3116	0.0163	0.898569		0.0026	0.0011009468
ss61484154	*rs41584705*	2	138.498694	0.5351	1.2094	0.271837		0.0203	0.0100952807
ss86340041		2	138.520239	0.5544	0.1344	0.714019		0.0068	0.0033412449
ss86297798		2	138.588187	0.3037	0.0657	0.797775		0.0051	0.0021376122

ss61542280	*rs41643302*	2	138.625881	0.5079	0.0677	0.794850	0.0048	0.0023940972
ss86312723		2	138.667975	0.3467	4.9604	0.026254	0.0430	0.0194631196
ss86309231		2	138.701505	0.3209	0.0712	0.789655	0.0052	0.0022705169
ss86340933		2	138.73893	0.3467	9.9542	0.001674	0.0596	0.0269918785
ss86293267		2	138.827573	0.1963	3.6093	0.057871	0.0433	0.0136683752
ss86297742		2	138.887828	0.6834	2.8870	0.089746	0.0340	0.0147066980
ss86316613		2	139.004087	0.5201	0.6490	0.420747	0.0152	0.0076121991
ss86323300		2	139.108293	0.1039	0.0609	0.805078	0.0074	0.0013833447
ss86336877		2	139.133717	0.3861	2.6804	0.102043	0.0314	0.0148674104
ss61544273	*rs41642971*	2	139.166009	0.3811	0.0000	0.997466	0.0001	0.0000285937
ss86317313		2	139.19475	0.6053	3.0557	0.080896	0.0329	0.0157348013
ss86286362		2	139.222087	0.8825	2.2355	0.135328	0.0438	0.0090879990
ss61542259	*rs41643281*	2	139.251135	0.2779	0.8310	0.362289	0.0182	0.0073078343
ss86297852		2	139.274356	0.5387	0.2048	0.651052	0.0085	0.0042128687
ss86309726		2	139.352253	0.4713	3.4413	0.064008	0.0343	0.0170925441
ss86293652		2	139.372355	0.4334	3.0691	0.080234	0.0325	0.0159404124
ss86274503		2	139.408531	0.3524	0.1912	0.662086	0.0089	0.0040538135
ss86333466		2	139.429929	0.8030	0.4024	0.526051	0.0148	0.0046898267
ss86339929		2	139.493014	0.6261	0.0307	0.861073	0.0034	0.0015865789
ss61530688	*rs41627659*	2	139.516885	0.7794	14.8218	0.000129	0.0856	0.0294273273
ss64095889	*rs42467613*	2	139.55134	0.6160	0.0176	0.894349	0.0025	0.0011838672
ss86329004		2	139.576495	0.5208	0.1479	0.700623	0.0070	0.0035016399
ss86327551		2	139.624425	0.7808	8.8437	0.003043	0.0655	0.0224269154
ss86320951		2	139.665915	0.1941	0.1707	0.679590	0.0096	0.0030046130
ss86336471		2	139.700777	0.7794	3.7246	0.054023	0.0437	0.0150418961
ss86289317		2	139.727814	0.2221	0.4892	0.484518	0.0159	0.0054863502
ss86299240		2	139.757788	0.5580	2.1411	0.143850	0.0276	0.0136056647
ss86297444		2	139.783052	0.5695	1.7880	0.181604	0.0251	0.0122834224
ss86283753		2	139.808623	0.2858	0.5086	0.475965	0.0151	0.0061479343
ss86307880		2	139.836776	0.4398	1.5579	0.212387	0.0233	0.0114869541
ss64132283	*rs42486527*	2	139.864611	0.1977	0.7555	0.385037	0.0204	0.0064855739
ss86297972		2	139.892519	0.1418	0.0042	0.948287	0.0017	0.0004204261
ss117966879		2	139.942812	0.1898	18.1429	0.000023	0.0990	0.0304618723
ss86273935		2	139.963958	0.7027	0.2184	0.640371	0.0096	0.0040182807
ss86296590		2	139.987691	0.1683	1.4836	0.223620	0.0293	0.0082122149
ss86278071		2	140.010696	0.1411	2.4967	0.114538	0.0425	0.0103100506
ss86335745		2	140.030965	0.5573	4.6009	0.032301	0.0401	0.0197777973
ss86293981		2	140.090279	0.2758	3.2828	0.070441	0.0383	0.0153021879
ss61532025	*rs41632459*	2	140.129976	0.2557	0.2147	0.643233	0.0098	0.0037456606
ss86320970		2	140.186752	0.1655	2.2302	0.135787	0.0364	0.0100400991
ss86335445		2	140.213621	0.1583	0.1577	0.691450	0.0101	0.0026917532
ss86337290		2	140.250152	0.6734	0.0717	0.788936	0.0054	0.0023673840
ss86333178		2	140.273143	0.3725	0.4363	0.509130	0.0131	0.0061413815
	rs29027154	2	140.307484	0.6605	0.4071	0.523660	0.0128	0.0057388906
ss86327482		2	140.331355	0.5587	0.2973	0.585766	0.0103	0.0050976058
ss86340527		2	140.38905	0.4054	0.0307	0.860998	0.0033	0.0016066087
ss61484454		2	140.506974	0.6540	0.8699	0.351309	0.0184	0.0083459388

ss86326278		2	140.579392	0.6017	2.2456	0.134447		0.0293	0.0140423556
ss65241350	*rs43326106*	2	140.60318	0.1003	0.0030	0.956346		0.0017	0.0003029264
ss86339699		2	140.625558	0.5860	0.0002	0.988643		0.0003	0.0001343117
ss86304961		2	140.672838	0.1769	5.3423	0.021106		0.0568	0.0165289182
ss61530985	*rs41631048*	3	0.783652	0.2650	1.3247	0.250138		0.0245	0.0095334094
ss86287051		3	0.830269	0.3238	0.1405	0.707910		0.0077	0.0033531422
ss86341522		3	0.851957	0.6683	0.7900	0.374421		0.0181	0.0080314697
	rs29010435	3	1.07157	0.3317	3.0720	0.080094		0.0359	0.0159289122
ss86294040		3	1.103724	0.4778	6.9350	0.008640		0.0493	0.0246160342
ss61553761	*rs43715303*	3	1.164761	0.7815	0.0040	0.949622		0.0014	0.0004909251
ss61553400	*rs43712149*	3	1.242989	0.7228	0.6814	0.409371		0.0173	0.0069431207
ss86297979		3	1.322025	0.7930	0.0192	0.889914		0.0032	0.0010542935
ss117967140		3	1.430154	0.7765	0.3071	0.579664		0.0127	0.0044158028
ss86337542		3	1.480363	0.7865	6.1807	0.013149		0.0591	0.0198533601
ss61553052		3	1.517811	0.1640	0.2091	0.647625		0.0119	0.0032518311
ss86328405		3	1.539202	0.5365	0.0475	0.827515		0.0040	0.0020036426
ss61552845	*rs43707200*	3	1.562409	0.9176	0.0008	0.977111		0.0010	0.0001506189
ss86335306		3	1.594643	0.3596	1.4296	0.232231		0.0230	0.0106143154
ss61468714	*rs41570825*	3	1.618717	0.4742	0.9004	0.342997		0.0176	0.0087738351
ss86335064		3	1.647714	0.9176	2.0973	0.148012		0.0488	0.0073776884
ss86336300		3	1.694778	0.2693	0.3397	0.560165		0.0120	0.0047406965
ss86331778		3	1.737536	0.5931	0.2734	0.601202		0.0101	0.0048680170
ss117963675		3	1.758974	0.5587	11.6144	0.000692		0.0624	0.0307815861
ss86301348		3	1.781964	0.4348	11.3084	0.000814		0.0620	0.0304967161
ss86288564		3	1.824721	0.6676	6.3637	0.011869		0.0496	0.0220257280
ss117967013		3	2.054174	0.1304	0.9483	0.330496		0.0263	0.0059651419
ss61552531	*rs43710983*	3	2.126548	0.4413	1.1023	0.294130		0.0200	0.0098834768
ss86337671		3	2.166798	0.8188	1.7898	0.181387		0.0325	0.0096482946
ss86312631		3	2.230466	0.6963	0.0180	0.893215		0.0027	0.0011339565
ss86286210		3	2.281391	0.7085	2.4895	0.115065		0.0329	0.0135909319
ss86288120		3	2.32312	0.3603	1.0925	0.296288		0.0200	0.0091969625
ss61468699	*rs41570810*	3	2.454658	0.7321	0.0125	0.910907		0.0024	0.0009226378
ss86274159		3	2.47596	0.7751	0.5758	0.448228		0.0171	0.0059631155
ss86334884		3	2.562456	0.7908	1.2876	0.256881		0.0259	0.0085788875
ss86333513		3	2.642983	0.2156	1.0352	0.309296		0.0229	0.0077426196
ss86334582		3	2.676717	0.3309	4.5116	0.034018		0.0418	0.0185015484
ss61524121	*rs41625668*	3	2.722673	0.6934	2.0069	0.157027		0.0284	0.0120644168
ss61516232	*rs41613350*	3	2.751898	0.1791	3.9870	0.046241		0.0475	0.0139797128
ss61516233	*rs41613351*	3	2.804519	0.9312	8.3159	0.004051		0.1069	0.0136922383
ss61472333		3	2.9967	0.3861	0.2906	0.589986		0.0105	0.0049895419
ss61516236	*rs41613354*	3	3.056576	0.6404	0.0134	0.907853		0.0023	0.0010413794
ss86295935		3	3.117272	0.3818	0.0751	0.784127		0.0053	0.0025217852
ss86336794		3	3.290124	0.5064	3.8505	0.050129		0.0358	0.0178776374
ss86338839		3	3.314581	0.4907	0.0990	0.753129		0.0058	0.0029033487
ss86337794		3	3.350883	0.3603	4.1083	0.043054		0.0385	0.0177361105
ss86312135		3	3.403598	0.6791	2.1397	0.143984		0.0303	0.0132116444
ss117962782		3	3.425522	0.3202	2.2942	0.130311		0.0316	0.0137543958

ss86321942		3	3.513944	0.2034	1.5062	0.220134		0.0287	0.0092870368
ss86329954		3	3.575551	0.4606	1.1524	0.283427		0.0202	0.0100426103
ss86295172		3	3.683167	0.4979	2.4566	0.117484		0.0290	0.0144978028
ss86312534		3	3.715521	0.2457	1.8000	0.180149		0.0288	0.0106788400
ss117967004		3	3.897428	0.4613	0.0138	0.906359		0.0022	0.0010944138
ss86287033		3	3.962473	0.1476	10.3404	0.001362		0.0817	0.0205607329
ss86285432		3	3.987323	0.2242	0.0128	0.909979		0.0025	0.0008531586
ss117962770		3	4.064094	0.2256	0.2853	0.593396		0.0119	0.0041473786
ss86339011		3	4.090963	0.1554	0.3542	0.551966		0.0154	0.0040386035
ss86331212		3	4.138641	0.3926	2.3264	0.127649		0.0286	0.0136366238
ss86336336		3	4.16558	0.3467	6.0151	0.014429		0.0477	0.0216168323
ss86301682		3	4.203995	0.7092	1.8104	0.178894		0.0273	0.0112478126
ss61552881	*rs43707936*	3	4.233403	0.6884	3.8378	0.050508		0.0391	0.0167613725
ss61523537	*rs41621703*	3	4.294079	0.2471	4.7848	0.029044		0.0466	0.0173526654
ss86297640		3	4.346123	0.1712	1.3692	0.242353		0.0292	0.0082753822
ss86324869		3	4.39293	0.5523	0.0143	0.904768		0.0022	0.0010814979
ss61474109	*rs41566721*	3	4.435621	0.4019	0.6824	0.409058		0.0154	0.0074261344
ss86329253		3	4.525457	0.4183	6.1357	0.013485		0.0465	0.0226227734
ss63828126	*rs42367515*	3	4.710036	0.1160	7.3016	0.007057		0.0781	0.0160150866
ss61524308	*rs41619760*	3	4.863972	0.8188	0.4070	0.523698		0.0155	0.0046119611
ss64786860	*rs42885366*	3	4.887774	0.8718	2.5174	0.113051		0.0438	0.0097828083
ss86317958		3	4.911461	0.3489	0.5412	0.462180		0.0142	0.0064622545
ss63827692	*rs42372510*	3	4.931652	0.3517	9.9367	0.001690		0.0596	0.0271613552
ss61518029	*rs41621392*	3	4.958546	0.6762	6.4858	0.011088		0.0498	0.0217921212
ss86286004		3	4.98151	0.6175	4.8887	0.027357		0.0423	0.0200043386
ss63827468	*rs42371699*	3	5.037016	0.5523	1.0638	0.302705		0.0195	0.0096264075
ss117967146		3	5.061437	0.2851	0.2722	0.602047		0.0106	0.0043139652
ss86339962		3	5.111887	0.4649	1.5123	0.219204		0.0225	0.0111769050
ss61518036		3	5.148447	0.6397	2.6965	0.101019		0.0311	0.0143296339
ss86283289		3	5.177055	0.2156	2.1560	0.142463		0.0323	0.0109316129
ss64477212	*rs42657029*	3	5.270355	0.8395	0.3878	0.533683		0.0157	0.0042398394
ss64895752	*rs42989922*	3	5.307453	0.7844	11.3366	0.000802		0.0756	0.0255706568
ss61520160	*rs41611090*	3	5.332915	0.5860	0.1030	0.748384		0.0063	0.0030690088
ss61506789		3	5.369762	0.4069	6.9303	0.008663		0.0506	0.0244344668
ss61515365	*rs41613721*	3	5.44201	0.1984	13.2416	0.000294		0.0846	0.0268964856
ss86328846		3	5.539104	0.8424	14.6193	0.000143		0.0963	0.0255690782
ss86311181		3	5.572041	0.5215	2.5084	0.113696		0.0297	0.0148156032
ss86337887		3	5.63281	0.1827	13.7018	0.000231		0.0849	0.0253529407
ss117962967		3	5.677663	0.8080	11.8335	0.000616		0.0779	0.0241582100
ss86340273		3	5.763387	0.0996	0.0000	0.998367		0.0001	0.0000113778
ss61532192	*rs41633305*	3	5.870544	0.4291	2.2918	0.130514		0.0286	0.0140127582
ss63867960	*rs42391944*	3	5.946006	0.8338	0.8719	0.350747		0.0240	0.0066602710
ss64763404	*rs42859507*	3	6.105593	0.9635	0.0993	0.752760		0.0154	0.0010825673
ss86299547		3	6.148361	0.8009	0.0557	0.813416		0.0054	0.0017154844
ss86311724		3	6.247497	0.2593	1.0648	0.302477		0.0223	0.0085657600
ss38337028	*rs29023733*	3	6.301348	0.7579	1.5896	0.207804		0.0277	0.0101574600
	rs29027025	3	6.402261	0.2708	0.0733	0.786730		0.0058	0.0023058407

ss64074183	*rs42458820*	3	6.444053	0.2350	0.8140	0.367245	0.0193	0.0069503422
ss61479338	*rs41577207*	3	6.480556	0.7400	0.3671	0.544811	0.0128	0.0049411649
ss61478196	*rs43744097*	3	6.553149	0.7851	0.0645	0.799589	0.0057	0.0019137418
ss64074545	*rs42461078*	3	6.580371	0.2486	0.0396	0.842400	0.0044	0.0016295903
ss61507448	*rs41606642*	3	6.606823	0.3474	2.0782	0.149865	0.0276	0.0124985194
ss61489396	*rs41592618*	3	6.661025	0.2844	0.5109	0.474995	0.0152	0.0061682560
ss61553368	*rs43711417*	3	6.744509	0.1562	0.5318	0.466079	0.0181	0.0047823013
ss64064263	*rs42456176*	3	6.856019	0.4241	0.2116	0.645627	0.0086	0.0041894692
ss64064319	*rs42456204*	3	6.885989	0.4979	0.6131	0.433873	0.0146	0.0073029966
ss86327524		3	6.912399	0.4742	0.6111	0.434641	0.0146	0.0072801193
ss86336669		3	6.950127	0.7256	0.2213	0.638203	0.0095	0.0037771992
ss64695833	*rs42790679*	3	6.985014	0.6691	3.3491	0.067669	0.0363	0.0160722367
ss64695893	*rs42791039*	3	7.009487	0.4871	0.0319	0.858243	0.0034	0.0016930229
ss65419566	*rs43504549*	3	7.030974	0.4592	0.1945	0.659364	0.0080	0.0039823838
ss65419647	*rs43499732*	3	7.067935	0.6827	0.7931	0.373466	0.0182	0.0078663484
ss117966994		3	7.098276	0.1569	0.1735	0.677140	0.0107	0.0028221624
ss61472899	*rs41574618*	3	7.13378	0.4248	6.0868	0.013860	0.0460	0.0224837848
ss86338833		3	7.210717	0.3173	1.5352	0.215745	0.0246	0.0106520043
ss61553420	*rs43712168*	3	7.259952	0.5623	0.8251	0.364014	0.0171	0.0084002599
ss61553652	*rs43714196*	3	7.311099	0.9004	0.1153	0.734265	0.0107	0.0019198831
ss86341730		3	7.362256	0.8446	0.1602	0.689120	0.0104	0.0027335705
ss86325978		3	7.409645	0.5079	0.9097	0.340521	0.0175	0.0087416266
ss46526189	*rs41255331*	3	7.498254	0.4019	4.0373	0.044892	0.0369	0.0177599680
ss61553518	*rs43712964*	3	7.571091	0.1855	0.8359	0.360898	0.0216	0.0065246147
ss61553516	*rs43712962*	3	7.597799	0.3023	0.1801	0.671378	0.0086	0.0036255471
ss61489474	*rs41585097*	3	7.649578	0.2751	6.1111	0.013672	0.0502	0.0200252844
ss117963661		3	7.703977	0.9133	0.0013	0.971039	0.0012	0.0001870189
ss86340582		3	7.745297	0.4011	3.7315	0.053803	0.0371	0.0178301560
ss86334965		3	7.83089	0.7092	0.1355	0.712911	0.0075	0.0030967235
ss86333142		3	7.968824	0.4857	0.2547	0.613958	0.0096	0.0047896271
ss61494308	*rs41595711*	3	7.989134	0.7951	2.4198	0.120263	0.0346	0.0112839522
ss86324790		3	8.014476	0.2722	0.0579	0.809905	0.0050	0.0019899534
ss117967012		3	8.043398	0.5064	5.1827	0.023115	0.0416	0.0208028533
ss86277693		3	8.176132	0.2901	0.1854	0.666881	0.0088	0.0036132613
ss86290205		3	8.198277	0.1762	0.6655	0.414889	0.0199	0.0057767456
ss117967053		3	8.237421	0.4033	9.5442	0.002086	0.0564	0.0271366088
ss61510035	*rs41601182*	3	8.435318	0.2020	5.8129	0.016167	0.0558	0.0179795157
ss38331829	*rs29025933*	3	8.521639	0.8718	0.0006	0.981110	0.0007	0.0001474164
ss86317150		3	8.558755	0.2715	0.0578	0.810043	0.0049	0.0019208373
ss86340800		3	8.598511	0.3274	3.5182	0.061117	0.0361	0.0158904666
ss117962685		3	8.64933	0.1934	3.3488	0.067683	0.0427	0.0133126751
ss117962836		3	8.742286	0.3668	15.4162	0.000095	0.0760	0.0352918306
ss117962747		3	9.159238	0.6855	1.1339	0.287324	0.0203	0.0087719038
ss86303366		3	9.25457	0.3130	1.6683	0.196921	0.0250	0.0107659381
	rs29027882	3	9.300477	0.2894	5.9353	0.015090	0.0489	0.0201039597
ss86293935		3	9.414912	0.3209	1.3694	0.242310	0.0234	0.0102176039
	rs29016221	3	9.439254	0.8904	9.0238	0.002761	0.0903	0.0176254917

ss86316782		3	9.51117	0.5824	4.8714	0.027630		0.0406	0.0197347828
ss86326930		3	9.560676	0.8617	0.0001	0.990955		0.0003	0.0000736841
ss86320369		3	9.591429	0.5072	0.2916	0.589390		0.0098	0.0048862418
ss61566716	*rs41667341*	3	9.772744	0.3016	19.5191	0.000012		0.0861	0.0362501504
ss61567394	*rs41662609*	3	9.887417	0.3596	6.9314	0.008658		0.0497	0.0228724971
ss86341650		3	10.316149	0.2937	17.1353	0.000039		0.0818	0.0339215013
ss86287231		3	10.383301	0.2758	24.4610	0.000001		0.0984	0.0392875525
ss117962654		3	10.434204	0.7063	19.5089	0.000012		0.0868	0.0360102820
ss86303320		3	10.466381	0.2421	2.9054	0.088727		0.0355	0.0130284912
ss86326086		3	10.513742	0.4434	1.9578	0.162194		0.0254	0.0125349402
ss86274434		3	10.541366	0.7837	3.5908	0.058516		0.0416	0.0141052156
ss86338768		3	10.606176	0.6805	4.8062	0.028687		0.0429	0.0186755932
ss86339467		3	10.643374	0.2120	0.0008	0.976888		0.0007	0.0002207332
ss86340586		3	10.665426	0.7858	5.1677	0.023314		0.0508	0.0171084800
ss86285838		3	10.692015	0.9019	0.0216	0.883122		0.0046	0.0008110579
ss86299316		3	10.836472	0.4499	6.3922	0.011682		0.0470	0.0232393662
ss86304157		3	10.856674	0.7858	0.5716	0.449890		0.0175	0.0059058427
ss86335901		3	10.877235	0.6268	6.6579	0.010075		0.0494	0.0231149494
ss86292240		3	10.916641	0.1476	1.0831	0.298364		0.0272	0.0068520768
ss86339756		3	10.999148	0.1368	1.7436	0.187118		0.0354	0.0083568019
ss86333793		3	11.052308	0.0881	1.5801	0.209162		0.0401	0.0064364511
ss61503741	*rs41599733*	3	11.095682	0.5251	17.5149	0.000032		0.0765	0.0381491444
ss61520877	*rs41618396*	3	11.175629	0.6819	1.8992	0.168616		0.0275	0.0119428435
ss61520870	*rs41618389*	3	11.207588	0.3983	4.6540	0.031323		0.0394	0.0189055983
ss86301098		3	11.276354	0.1855	3.1555	0.076110		0.0417	0.0125967435
ss86274798		3	11.316	0.1554	4.7450	0.029719		0.0537	0.0140916490
ss86274279		3	11.369783	0.7808	8.9982	0.002799		0.0646	0.0221023133
ss86335657		3	11.426261	0.5537	3.0575	0.080804		0.0328	0.0161889305
ss86324340		3	11.470013	0.2042	15.0389	0.000115		0.0850	0.0276255126
ss61466142	*rs41565903*	3	11.491138	0.4864	0.5139	0.473706		0.0132	0.0066064318
ss86309487		3	11.511276	0.1540	1.6273	0.202508		0.0327	0.0085114153
ss86304164		3	11.564219	0.0752	0.0178	0.893776		0.0047	0.0006562157
ss86318009		3	11.597249	0.8689	1.0598	0.303622		0.0285	0.0064940632
ss86324399		3	11.890639	0.6748	5.0357	0.025143		0.0440	0.0193114953
ss86330763		3	11.961227	0.0967	0.8461	0.357990		0.0283	0.0049394076
ss61475074	*rs41571467*	3	11.98552	0.3524	13.2510	0.000293		0.0686	0.0312986780
ss86321613		3	12.190029	0.3660	3.5305	0.060666		0.0357	0.0165745872
ss61474963	*rs41574149*	3	12.273922	0.6741	14.6688	0.000140		0.0748	0.0328537144
ss61478376	*rs41575776*	3	12.394094	0.3052	0.1076	0.742989		0.0063	0.0026718133
ss61478372	*rs41575772*	3	12.41764	0.2042	2.8698	0.090701		0.0385	0.0125230146
ss86334835		3	12.6341	0.3338	0.0763	0.782521		0.0053	0.0023480015
ss86328970		3	12.677939	0.3338	0.1812	0.670507		0.0082	0.0036305718
ss86288973		3	12.903718	0.2507	2.5919	0.107865		0.0351	0.0131946351
ss86316669		3	13.515249	0.7149	0.1552	0.693759		0.0077	0.0031495388
	rs29012865	3	13.542667	0.7170	0.6372	0.425009		0.0157	0.0063693600
ss86302754		3	13.63832	0.7794	5.4865	0.019445		0.0519	0.0178426131
ss86285582		3	13.910519	0.2887	0.3370	0.561759		0.0118	0.0048404875

	rs29016987	3	14.026624	0.4828	0.9563	0.328472	0.0178	0.0089001863
ss86319266		3	14.120856	0.5788	0.7581	0.384216	0.0161	0.0078544033
ss86334928		3	14.178813	0.7665	5.1569	0.023458	0.0497	0.0177946183
ss86340255		3	14.250682	0.3782	11.6076	0.000695	0.0642	0.0301943224
ss86330567		3	14.284628	0.3317	0.3771	0.539376	0.0122	0.0054073429
ss86327308		3	14.313244	0.3890	8.7505	0.003200	0.0549	0.0260805758
ss86333838		3	14.35281	0.9119	5.9677	0.014818	0.0774	0.0124425747
ss86305937		3	14.418593	0.8775	2.1337	0.144547	0.0414	0.0088986117
ss86278527		3	14.589463	0.1819	7.4811	0.006394	0.0654	0.0194560837
ss86273812		3	14.779898	0.7708	2.9596	0.085815	0.0374	0.0132264292
ss86301573		3	14.946816	0.5287	0.7417	0.389418	0.0159	0.0079354914
ss86283833		3	15.068305	0.3266	4.1799	0.041281	0.0388	0.0170556668
ss86337259		3	15.114778	0.2213	8.2997	0.004087	0.0631	0.0217620112
	rs29010859	3	15.190305	0.6404	19.9007	0.000010	0.0889	0.0409484285
ss86274753		3	15.349187	0.1626	1.2456	0.264771	0.0272	0.0074059252
ss86301480		3	15.437177	0.2063	22.6902	0.000002	0.1071	0.0350886950
ss117963699		3	15.459129	0.8890	0.0216	0.883217	0.0043	0.0008480980
ss86305393		3	15.480732	0.6712	5.7715	0.016550	0.0458	0.0201966443
ss46526153	*rs41255296*	3	15.513381	0.5380	3.1220	0.077682	0.0340	0.0169168834
ss86304013		3	15.624902	0.2607	5.2904	0.021738	0.0480	0.0185217721
ss61488827	*rs41587368*	3	15.679302	0.3431	0.1919	0.661472	0.0082	0.0037028154
ss86304033		3	15.72774	0.8367	2.1775	0.140498	0.0359	0.0098240256
ss86289884		3	15.874036	0.6991	7.4980	0.006335	0.0545	0.0229191965
ss86327323		3	15.911824	0.1160	5.4882	0.019426	0.0668	0.0137128900
ss86315112		3	15.957682	0.8818	1.5920	0.207463	0.0343	0.0071549694
ss46526101	*rs41255245*	3	15.979258	0.8696	2.8864	0.089778	0.0447	0.0101408629
ss86338546		3	16.009461	0.1153	1.0761	0.299941	0.0292	0.0059499855
ss117962610		3	16.046038	0.7228	2.7933	0.095110	0.0349	0.0139776835
ss86300263		3	16.073321	0.7686	2.8142	0.093882	0.0367	0.0130359067
ss61552164	*rs43708421*	3	16.09345	0.8725	3.0890	0.079263	0.0483	0.0107406508
	rs29011589	3	16.167104	0.6261	5.4103	0.020305	0.0454	0.0212568487
ss86325581		3	16.201159	0.7178	24.7308	0.000001	0.1008	0.0408575818
ss61552167	*rs43708424*	3	16.221715	0.8266	6.6919	0.009887	0.0621	0.0177932160
ss86331066		3	16.275382	0.8266	5.5750	0.018493	0.0567	0.0162623501
ss86317228		3	16.430468	0.7758	1.3442	0.246684	0.0257	0.0089377147
ss86341393		3	16.641799	0.8130	0.0780	0.780096	0.0069	0.0021124741
ss86314533		3	16.6645	0.9105	14.1056	0.000187	0.1195	0.0194814523
ss86321188		3	16.744495	0.1848	0.5689	0.450932	0.0189	0.0056923019
ss86284345		3	16.765381	0.8116	0.1641	0.685561	0.0100	0.0030604216
ss86340570		3	16.791053	0.1404	0.1913	0.661993	0.0118	0.0028586352
ss61510211	*rs41602655*	3	16.823582	0.7708	0.0020	0.964326	0.0010	0.0003479618
ss86294263		3	16.850664	0.4040	2.1177	0.146053	0.0275	0.0132373556
ss86328419		3	17.119072	0.4885	0.8587	0.354413	0.0173	0.0086216909
ss86328559		3	17.230698	0.5874	10.9894	0.000964	0.0586	0.0284011067
ss86321294		3	17.276447	0.3152	12.2120	0.000505	0.0709	0.0306123177
ss61553881	*rs41648129*	3	17.398313	0.3109	0.0029	0.957257	0.0010	0.0004478109
ss86320758		3	17.423094	0.2063	0.3426	0.558547	0.0136	0.0044375189

ss86275706		3	17.47751	0.3338	1.1190	0.290508	0.0214	0.0095086459
ss86322846		3	17.505391	0.3933	2.2626	0.132985	0.0281	0.0134051937
ss86279330		3	17.568603	0.8603	1.4987	0.221292	0.0302	0.0072622077
ss86321255		3	17.604942	0.0895	0.3130	0.576044	0.0180	0.0029286761
ss86293333		3	17.702102	0.9255	1.4376	0.230940	0.0420	0.0057915716
ss86332523		3	17.725635	0.6261	12.9039	0.000351	0.0657	0.0307794093
ss117966908		3	17.769827	0.1583	4.4577	0.035100	0.0532	0.0141850338
ss86318221		3	17.859167	0.8467	0.0398	0.842007	0.0052	0.0013573237
ss86327483		3	17.88245	0.2772	25.9482	0.000000	0.1011	0.0404995186
ss61553728	*rs43715270*	3	18.050429	0.8761	0.0005	0.982261	0.0006	0.0001365050
ss61553726	*rs43715268*	3	18.070495	0.4900	5.3497	0.021017	0.0423	0.0211413980
ss86275087		3	18.110134	0.4799	5.7474	0.016775	0.0436	0.0217627072
	rs29010816	3	18.218646	0.4936	2.3910	0.122488	0.0273	0.0136659763
ss86306054		3	18.318316	0.0308	2.0433	0.153324	0.0768	0.0045875303
ss61510016	*rs41601163*	3	18.338694	0.8001	1.3435	0.246815	0.0266	0.0085155475
	rs29015763	3	18.495144	0.7049	28.8448	0.000000	0.1056	0.0439500567
ss61488954	*rs41588495*	3	18.530411	0.8711	6.7028	0.009827	0.0714	0.0160486528
ss61552453	*rs43710808*	3	18.576297	0.1648	15.2743	0.000102	0.0946	0.0260374578
ss61552503		3	18.61457	0.8789	2.4887	0.115123	0.0445	0.0094720278
ss61471094	*rs41567166*	3	18.653445	0.2378	16.1908	0.000064	0.0878	0.0318278279
ss86291527		3	18.788654	0.9348	0.3617	0.547738	0.0224	0.0027288395
ss117966903		3	19.078902	0.4484	6.3014	0.012290	0.0465	0.0229981445
ss117962964		3	19.377134	0.2486	5.3537	0.020969	0.0488	0.0182385576
ss117975005		3	19.455354	0.6261	7.4377	0.006548	0.0524	0.0245188776
ss61488866	*rs41587407*	3	19.967708	0.5595	7.0035	0.008319	0.0497	0.0244953182
ss86332121		3	20.000611	0.3861	6.6517	0.010110	0.0471	0.0223234663
ss86280613		3	20.042272	0.5917	11.3503	0.000796	0.0624	0.0301476916
ss61552245	*rs43709202*	3	20.100357	0.4907	6.3132	0.012209	0.0462	0.0230847789
ss86309198		3	20.13043	0.1268	0.6252	0.429388	0.0221	0.0048896129
ss86329240		3	20.159585	0.4054	5.0280	0.025255	0.0413	0.0199151841
	rs29010082	3	20.291135	0.7593	28.1386	0.000000	0.1160	0.0423923930
ss86282864		3	20.421497	0.2020	23.5441	0.000002	0.1128	0.0363591467
ss117962714		3	20.519555	0.3840	0.5761	0.448113	0.0146	0.0068863683
ss86301142		3	20.564446	0.2987	5.1255	0.023884	0.0447	0.0187290955
ss61552257	*rs43709214*	3	20.664803	0.4327	9.8722	0.001749	0.0591	0.0290043457
ss117962753		3	20.719615	0.7350	20.2590	0.000008	0.0945	0.0368079515
ss86285964		3	20.781916	0.3381	0.0524	0.819069	0.0046	0.0020438088
ss86279459		3	20.809916	0.2937	18.8381	0.000016	0.0880	0.0365009857
ss86341092		3	20.847926	0.6340	13.6006	0.000244	0.0684	0.0317255045
ss86289254		3	20.909791	0.3646	0.0468	0.828728	0.0042	0.0019282509
ss61488880	*rs41587421*	3	21.00724	0.7564	1.5869	0.208196	0.0278	0.0102295833
ss86282202		3	21.137308	0.4921	2.2739	0.132019	0.0277	0.0138464228
ss61552279	*rs43709835*	3	21.163507	0.7514	7.0308	0.008194	0.0567	0.0211811061
ss86291838		3	21.289296	0.4577	2.3124	0.128796	0.0287	0.0142459376
ss117963685		3	21.490082	0.2600	0.3020	0.582826	0.0120	0.0046221883
ss117967102		3	21.525128	0.2600	0.3020	0.582826	0.0120	0.0046221883
ss86331754		3	21.702166	0.3424	3.0940	0.079022	0.0352	0.0158445288

ss61552295	*rs43709851*	3	21.973566	0.3503	0.2092	0.647549	0.0090	0.0041021557
ss86291898		3	21.995117	0.2278	9.8954	0.001727	0.0711	0.0250015471
ss117962856		3	22.068687	0.3252	15.5176	0.000090	0.0765	0.0335540681
ss117967123		3	22.224518	0.2178	5.1362	0.023738	0.0508	0.0173024155
ss46527060	*rs41255603*	3	22.266087	0.7744	3.1235	0.077610	0.0391	0.0136477827
ss86295803		3	22.405204	0.5143	0.6557	0.418358	0.0149	0.0074374769
ss86335876		3	22.523748	0.8797	3.5533	0.059843	0.0526	0.0111300379
ss86329461		3	22.977078	0.4663	3.7793	0.052293	0.0357	0.0177449627
ss86341694		3	23.353135	0.2600	25.7543	0.000000	0.1064	0.0409413323
ss86335810		3	23.40555	0.5100	2.8345	0.092710	0.0303	0.0151328228
ss86304918		3	23.448559	0.6239	2.8550	0.091541	0.0328	0.0153838186
ss86312549		3	23.483333	0.5967	3.8694	0.049570	0.0362	0.0174243241
ss117962803		3	23.541839	0.2708	12.2180	0.000503	0.0714	0.0281851426
ss61488867	*rs41587408*	3	23.563372	0.1297	0.0400	0.841570	0.0056	0.0012613935
ss86327855		3	23.635154	0.3775	12.0186	0.000559	0.0648	0.0304552756
ss86303514		3	23.690418	0.6096	1.5735	0.210117	0.0241	0.0114783773
ss117967093		3	23.786087	0.7099	18.3228	0.000021	0.0882	0.0363324157
ss86339275		3	23.900198	0.2980	20.3866	0.000007	0.0919	0.0384586564
ss117962800		3	23.933163	0.2350	17.4749	0.000033	0.0903	0.0324650039
ss86334501		3	23.959923	0.1440	6.7250	0.009707	0.0687	0.0169376934
ss86332374		3	24.018818	0.6196	4.5366	0.033527	0.0406	0.0191525998
ss86287295		3	24.038997	0.5681	2.2907	0.130604	0.0283	0.0138977683
	rs29019824	3	24.109922	0.1862	0.7047	0.401494	0.0198	0.0060058345
ss117962624		3	24.238044	0.8216	8.9804	0.002826	0.0724	0.0212266029
ss61552348	*rs43709904*	3	24.258942	0.1891	7.5106	0.006291	0.0650	0.0199409905
ss86309878		3	24.301872	0.4255	3.1636	0.075735	0.0333	0.0162746618
	rs29022370	3	24.374863	0.8890	1.5786	0.209388	0.0378	0.0074593948
ss117967042		3	24.39561	0.6189	22.7804	0.000002	0.0893	0.0421302475
ss86307074		3	24.418803	0.5752	3.0581	0.080774	0.0327	0.0159754172
ss86306287		3	24.450578	0.2808	12.5996	0.000412	0.0719	0.0290455721
ss86337899		3	24.568248	0.6812	3.2606	0.071395	0.0435	0.0188808021
ss86308145		3	24.6568	0.2708	7.0321	0.008189	0.0534	0.0210697636
ss86306088		3	24.685741	0.5415	0.8106	0.368240	0.0167	0.0083039319
ss86308308		3	24.708594	0.9155	0.3639	0.546545	0.0203	0.0031453898
ss117966949		3	24.739952	0.2199	3.4628	0.063184	0.0418	0.0143401198
ss61552300	*rs43709856*	3	24.817743	0.7880	0.6470	0.421449	0.0184	0.0061500691
ss117962670		3	25.030785	0.1139	0.1522	0.696548	0.0115	0.0023193639
ss61488892	*rs41588433*	3	25.071775	0.1254	0.0834	0.772854	0.0083	0.0018099124
ss61552310	*rs43709866*	3	25.096512	0.4362	2.7876	0.095449	0.0310	0.0152457130
ss86307447		3	25.30501	0.3309	6.3183	0.012174	0.0491	0.0217528920
ss117966910		3	25.354125	0.8123	4.7280	0.030011	0.0517	0.0157641008
ss61552322	*rs43709878*	3	25.405073	0.3968	1.6404	0.200697	0.0239	0.0114505189
ss117962664		3	25.514569	0.7493	6.5875	0.010478	0.0549	0.0206430323
ss46526193	*rs41255335*	3	25.578468	0.8589	6.7885	0.009371	0.0694	0.0168328470
ss86318349		3	25.61683	0.8367	3.8744	0.049425	0.0494	0.0135014344
ss61552349	*rs43709905*	3	25.670442	0.1074	7.3888	0.006726	0.0811	0.0155550828
ss86276367		3	25.987677	0.1297	3.3300	0.068457	0.0499	0.0112536580

ss117962646		3	26.0557	0.5530	0.6376	0.424838	0.0149	0.0073447894
ss117962830		3	26.122117	0.6318	6.7390	0.009632	0.0503	0.0233956180
ss86336755		3	26.198155	0.6211	4.8771	0.027541	0.0421	0.0198337424
ss117962768		3	26.245691	0.1318	0.0048	0.944941	0.0019	0.0004256395
ss117966945		3	26.331034	0.8789	0.7043	0.401624	0.0244	0.0051862632
ss86329892		3	26.521096	0.7507	0.0002	0.989002	0.0003	0.0001103797
ss117967088		3	26.589134	0.7321	12.1827	0.000513	0.0724	0.0284064050
	rs29018102	3	27.014669	0.2622	0.8215	0.365043	0.0188	0.0072674535
ss61516304	*rs41613420*	3	27.073081	0.6698	0.0275	0.868436	0.0032	0.0014158380
ss86338636		3	27.283056	0.1146	1.0094	0.315394	0.0293	0.0059393476
	rs29010328	3	27.364376	0.9083	1.3193	0.251112	0.0372	0.0061940624
ss61542466		3	27.411733	0.7665	6.8472	0.009071	0.0564	0.0202038759
ss38326034	*rs29011738*	3	27.536184	0.5236	0.1528	0.696039	0.0070	0.0034969646
ss86313234		3	27.568433	0.1175	0.0640	0.800393	0.0073	0.0015211400
ss86340024		3	27.648606	0.2292	5.5659	0.018589	0.0511	0.0180740752
ss86338455		3	27.724312	0.1132	0.7270	0.394155	0.0255	0.0051204903
ss86306757		3	27.76088	0.7901	0.5630	0.453303	0.0172	0.0057123031
ss86340406		3	27.845242	0.2507	14.0217	0.000196	0.0824	0.0309669123
ss61552374	*rs43709930*	3	27.987973	0.8825	1.2105	0.271608	0.0319	0.0066082820
ss86302394		3	28.245269	0.2722	1.2160	0.270524	0.0232	0.0091751822
ss86334418		3	28.27838	0.1397	1.8964	0.168929	0.0364	0.0087558147
ss61562154	*rs41658158*	3	28.416733	0.1648	3.0747	0.079961	0.0431	0.0118544335
ss86336818		3	28.449959	0.6662	2.2366	0.135233	0.0290	0.0128778069
ss117962643		3	28.476529	0.8145	1.5721	0.210322	0.0286	0.0086408603
ss117963702		3	28.510732	0.3166	0.6582	0.417475	0.0164	0.0071122510
ss61552382	*rs43710738*	3	28.591798	0.6046	1.1782	0.278095	0.0204	0.0097576544
ss86336825		3	28.627702	0.5451	0.0696	0.791976	0.0048	0.0023970998
ss86311737		3	28.650334	0.1748	2.3479	0.125902	0.0373	0.0107511936
ss86335556		3	28.671142	0.4463	0.5266	0.468288	0.0133	0.0065542791
ss86294341		3	28.729382	0.7944	2.9642	0.085571	0.0392	0.0128010223
ss86329478		3	28.775887	0.4857	0.2152	0.642903	0.0085	0.0042574982
	rs29011537	3	28.822646	0.0838	0.0950	0.758017	0.0106	0.0016312744
ss86336177		3	28.917938	0.3589	3.9413	0.047505	0.0376	0.0172970320
ss117963697		3	28.950467	0.5552	4.9437	0.026506	0.0404	0.0199415193
ss86304938		3	28.970654	0.4219	0.0559	0.813116	0.0044	0.0021523996
ss86325712		3	29.179683	0.0723	1.1261	0.288972	0.0375	0.0050357409
ss86336254		3	29.235407	0.3926	0.0038	0.950569	0.0012	0.0005580796
ss117966920		3	29.269845	0.2099	0.6835	0.408669	0.0188	0.0062269228
ss86320287		3	29.292585	0.2077	1.2008	0.273534	0.0249	0.0081954437
ss86274528		3	29.33501	0.8861	1.7124	0.191102	0.0372	0.0075002648
ss86303878		3	29.447164	0.0802	6.6847	0.009926	0.0868	0.0128066763
ss86336659		3	29.484239	0.7980	5.2634	0.022076	0.0544	0.0175240715
ss86325193		3	29.538733	0.6397	0.0139	0.906042	0.0024	0.0011027701
ss86285346		3	29.583414	0.8804	0.1620	0.687487	0.0115	0.0024233569
ss117966981		3	29.634183	0.8489	0.0940	0.759214	0.0080	0.0020596554
ss86303287		3	29.673627	0.0380	0.3202	0.571650	0.0272	0.0019853341
ss61552425	*rs43710781*	3	29.767916	0.8732	0.0571	0.811234	0.0063	0.0013975152

ss61552410	*rs43710766*	3	29.868906	0.3474	2.9449	0.086596	0.0339	0.0153923765
	rs29022505	3	29.921989	0.6798	3.5755	0.059052	0.0376	0.0163526435
ss86320795		3	29.967674	0.7557	8.3851	0.003902	0.0615	0.0227151743
ss86335639		3	30.00957	0.3825	6.8243	0.009187	0.0496	0.0234126527
ss86305674		3	30.065979	0.3252	6.6167	0.010309	0.0512	0.0224933080
ss86318640		3	30.086445	0.1354	0.2236	0.636425	0.0131	0.0030572127
ss86339364		3	30.231327	0.2607	0.1769	0.674203	0.0087	0.0033530319
ss86307412		3	30.42741	0.3503	4.3673	0.036998	0.0399	0.0181437863
ss117962755		3	30.453423	0.6705	6.5250	0.010849	0.0492	0.0217506963
ss86338373		3	30.517801	0.6784	6.8071	0.009275	0.0503	0.0219603495
ss86313359		3	30.55271	0.2235	7.8402	0.005251	0.0612	0.0212253371
ss117966943		3	30.821638	0.0623	0.1339	0.714568	0.0139	0.0016188057
ss86338218		3	31.193366	0.3281	4.6803	0.030850	0.0433	0.0190938850
	rs29021049	3	31.31746	0.4936	0.1464	0.702103	0.0071	0.0035402516
ss86304394		3	31.458232	0.4169	0.3505	0.554022	0.0111	0.0054120018
ss86336970		3	31.493392	0.2350	1.3748	0.241384	0.0259	0.0093055678
ss86339959		3	31.525914	0.5702	0.0049	0.944321	0.0013	0.0006449628
ss61552545	*rs43710997*	3	31.576358	0.7665	0.5736	0.449080	0.0170	0.0060921474
	rs29027608	3	31.647313	0.2328	0.0650	0.798826	0.0055	0.0019710644
ss86328618		3	31.662249	0.6941	0.3649	0.546012	0.0120	0.0050871784
ss86281359		3	31.693744	0.6476	0.6115	0.434472	0.0155	0.0070541137
ss86336884		3	31.741384	0.8102	0.0156	0.900649	0.0030	0.0009244803
ss86334278		3	31.797171	0.2658	0.3388	0.560719	0.0127	0.0049484369
ss86309302		3	31.819882	0.1691	1.1018	0.294239	0.0262	0.0073659360
ss86301308		3	31.841378	0.9577	0.9220	0.337293	0.0426	0.0034473873
	rs29020744	3	31.885701	0.1669	1.1901	0.275679	0.0273	0.0075965696
ss117962850		3	31.942822	0.1554	0.6995	0.403253	0.0222	0.0058384527
ss117962744		3	32.165811	0.8675	0.8062	0.369559	0.0238	0.0054834113
ss61552452	*rs43710807*	3	32.241262	0.7364	10.3390	0.001363	0.0689	0.0267655785
ss86305090		3	32.276645	0.6705	1.8294	0.176633	0.0270	0.0119395001
ss86335236		3	32.408065	0.3395	0.1318	0.716705	0.0071	0.0031880657
ss86317163		3	32.428468	0.7249	2.6505	0.103969	0.0349	0.0139020949
ss61488992	*rs41589433*	3	32.449266	0.4198	2.9911	0.084169	0.0329	0.0160213481
ss86300277		3	32.498386	0.6139	2.9314	0.087316	0.0341	0.0161449606
ss117963705		3	32.62488	0.7822	0.0054	0.941641	0.0016	0.0005563649
ss86323473		3	32.655139	0.0681	0.0010	0.974194	0.0012	0.0001505809
ss61564910	*rs41664672*	3	32.71576	0.9413	0.8177	0.366155	0.0356	0.0039342499
ss61494983	*rs41592467*	3	32.78497	0.8123	3.3496	0.067647	0.0415	0.0126501645
ss61494988	*rs41592472*	3	32.807639	0.7264	0.0693	0.792436	0.0054	0.0021378358
ss61564878	*rs41664640*	3	32.854057	0.2665	0.5026	0.478580	0.0150	0.0058820453
ss61564882	*rs41664644*	3	32.874729	0.5888	0.0867	0.768448	0.0055	0.0026529843
ss86339359		3	33.023094	0.8216	0.8116	0.367971	0.0220	0.0064611121
ss86316452		3	33.128963	0.4126	3.8045	0.051517	0.0386	0.0187190872
ss86285086		3	33.15945	0.4685	5.7517	0.016735	0.0472	0.0235282525
ss86337385		3	33.224907	0.1554	2.4951	0.114654	0.0409	0.0107451740
ss117967028		3	33.293579	0.9284	2.1723	0.140969	0.0534	0.0071007238
ss65249252	*rs43332280*	3	33.334174	0.9011	0.0300	0.862618	0.0055	0.0009732558

ss117962841		3	33.358819	0.4234	0.0080	0.928871	0.0017	0.0008382354
ss61512422	*rs41611728*	3	33.381002	0.5709	0.1461	0.702369	0.0074	0.0036096550
ss86303283		3	33.430283	0.1612	0.0127	0.910393	0.0028	0.0007686692
ss86340974		3	33.460651	0.6282	0.5560	0.456140	0.0140	0.0065325829
ss86338017		3	33.497831	0.2772	2.3235	0.127888	0.0321	0.0128526713
ss86284418		3	33.534782	0.2213	0.0875	0.767423	0.0068	0.0023554151
ss86296394		3	33.555322	0.1662	0.1182	0.731137	0.0086	0.0023895373
ss86287184		3	33.587267	0.2092	1.6078	0.205227	0.0293	0.0097039157
ss61552567	*rs43711019*	3	33.634546	0.7479	0.3271	0.567578	0.0121	0.0045557685
ss86298993		3	33.670691	0.2421	0.0015	0.968934	0.0008	0.0003068523
ss61489023	*rs41589464*	3	33.70029	0.2414	0.0060	0.938528	0.0017	0.0006064327
ss86309875		3	33.746652	0.1798	0.3029	0.582243	0.0129	0.0037979346
ss86330815		3	33.80345	0.9434	4.3069	0.038324	0.0825	0.0088133777
ss117962874		3	33.837443	0.1082	0.8563	0.355090	0.0275	0.0052987819
ss86340177		3	33.865979	0.5344	0.7811	0.377113	0.0167	0.0083329069
ss86306057		3	33.942046	0.3453	1.6247	0.202857	0.0250	0.0112865548
ss117967161		3	33.963385	0.5831	0.5174	0.472202	0.0135	0.0065672674
ss86286319		3	34.022397	0.3037	0.7367	0.391012	0.0173	0.0073126481
ss86296640		3	34.073884	0.2457	3.1525	0.076248	0.0378	0.0140015257
ss117967047		3	34.095513	0.9040	0.2466	0.619602	0.0153	0.0026618941
ss86316485		3	34.144303	0.5007	2.0171	0.155982	0.0265	0.0132377295
	rs29018416	3	34.165561	0.4004	0.4997	0.479867	0.0137	0.0065645478
ss86279062		3	34.19057	0.6017	0.7635	0.382542	0.0169	0.0080768904
ss65249264	*rs43332292*	3	34.346983	0.8259	0.0049	0.944408	0.0017	0.0004833211
ss117962974		3	34.410354	0.6905	0.8582	0.354573	0.0188	0.0080373969
ss65249335	*rs43332963*	3	34.472949	0.6132	0.0409	0.839871	0.0039	0.0018481955
ss86338009		3	34.496539	0.8754	9.3711	0.002289	0.0844	0.0184189931
ss86340786		3	34.560922	0.3639	4.5528	0.033214	0.0421	0.0195102510
ss65089898	*rs43176196*	3	34.590186	0.2973	0.1963	0.657871	0.0092	0.0038339856
ss86335864		3	34.713459	0.7830	2.9031	0.088854	0.0375	0.0127320331
ss65249965	*rs43336490*	3	34.747329	0.5158	2.8119	0.094014	0.0311	0.0155471251
ss86305921		3	35.147724	0.3080	0.2944	0.587565	0.0108	0.0045846787
ss86315905		3	35.174031	0.9277	3.1779	0.075077	0.0616	0.0082697238
ss65250437	*rs43336764*	3	35.327605	0.6117	2.2085	0.137702	0.0277	0.0131659760
ss65250391	*rs43336218*	3	35.420886	0.7643	0.6812	0.409446	0.0176	0.0063445699
	rs29027497	3	35.442521	0.4269	4.8265	0.028355	0.0411	0.0201016172
ss86319955		3	35.474074	0.8152	1.6466	0.199849	0.0306	0.0092109354
ss61523540	*rs41621706*	3	35.495625	0.6848	8.0890	0.004584	0.0565	0.0243897169
ss86283033		3	35.516207	0.7572	0.0809	0.776110	0.0061	0.0022473495
ss86301721		3	35.540163	0.1691	5.4843	0.019469	0.0577	0.0162071405
ss86335654		3	35.583314	0.1297	6.5898	0.010464	0.0691	0.0155852939
ss86315419		3	35.635818	0.2085	2.1688	0.141285	0.0332	0.0109444061
ss117967126		3	35.686647	0.5430	4.7510	0.029616	0.0410	0.0203615732
ss86340459		3	35.74928	0.6769	1.1121	0.292002	0.0206	0.0090249513
ss61552501	*rs43710955*	3	35.816917	0.7908	0.0018	0.966373	0.0009	0.0003056525
	rs29023519	3	35.84379	0.4599	0.0825	0.774061	0.0053	0.0026207847
ss86333789		3	35.874468	0.2973	1.0326	0.309895	0.0205	0.0085820485

ss86339080		3	35.899933	0.5910	0.1047	0.746319	0.0060	0.0028898229
ss86303159		3	35.934472	0.4678	6.7634	0.009502	0.0487	0.0242400650
ss86326023		3	35.970193	0.1941	1.3198	0.251021	0.0273	0.0085559559
ss86308018		3	36.003486	0.5158	0.0100	0.920231	0.0019	0.0009278328
ss117962593		3	36.051206	0.4713	4.9702	0.026105	0.0424	0.0211300339
ss65248714	*rs43337440*	3	36.123593	0.5287	4.9702	0.026105	0.0424	0.0211300339
ss86335894		3	36.253694	0.6032	3.7967	0.051755	0.0370	0.0177046718
ss86316951		3	36.310222	0.5738	11.5241	0.000726	0.0636	0.0311112243
ss117962676		3	36.338393	0.3911	3.8318	0.050687	0.0375	0.0178601808
ss61552525	*rs43710977*	3	36.382244	0.7027	3.6027	0.058099	0.0385	0.0160803146
ss86323183		3	36.432761	0.8446	0.0926	0.760952	0.0078	0.0020421688
ss86340794		3	36.464334	0.4183	3.6976	0.054900	0.0364	0.0177134292
ss86318617		3	36.494621	0.7507	2.4795	0.115795	0.0332	0.0124215082
ss61552528	*rs43710980*	3	36.517119	0.0716	0.3921	0.531408	0.0227	0.0030248976
ss61552529	*rs43710981*	3	36.543962	0.1268	0.3615	0.547885	0.0169	0.0037418962
ss61552530	*rs43710982*	3	36.56614	0.7514	0.0033	0.954016	0.0012	0.0004617867
ss86330517		3	36.603946	0.6819	1.6499	0.199406	0.0255	0.0110541432
ss117962603		3	36.708595	0.1318	2.5803	0.108656	0.0432	0.0098821142
	rs29019267	3	36.754026	0.4234	0.1434	0.705036	0.0070	0.0034299176
ss86335854		3	36.797994	0.4234	0.1434	0.705036	0.0070	0.0034299176
ss86298586		3	36.833881	0.8338	7.6216	0.005919	0.0665	0.0184179838
ss46526998	*rs41255543*	3	36.855643	0.1769	2.2144	0.137183	0.0364	0.0106051870
ss61489058	*rs41589497*	3	36.914969	0.4592	0.3170	0.573613	0.0100	0.0049818555
ss61563775	*rs41664044*	3	37.010042	0.3961	1.6968	0.193134	0.0243	0.0116176214
ss61489063	*rs41589502*	3	37.027802	0.3460	1.0848	0.297981	0.0200	0.0090630092
ss61523542	*rs41621708*	3	37.070842	0.3460	0.7458	0.388100	0.0166	0.0075182348
ss61552631		3	37.136108	0.6934	4.6536	0.031329	0.0434	0.0184436054
ss61552632	*rs43711684*	3	37.156761	0.6927	5.0897	0.024379	0.0454	0.0193154616
ss86340297		3	37.201333	0.5888	7.6213	0.005920	0.0508	0.0246067570
ss86315374		3	37.24632	0.3847	0.8705	0.351141	0.0178	0.0084120481
ss86313794		3	37.396921	0.7679	0.0212	0.884191	0.0032	0.0011289284
ss86307195		3	37.427197	0.5107	0.4185	0.517895	0.0120	0.0060094237
ss86285628		3	37.448842	0.2801	0.0558	0.813353	0.0050	0.0020125597
ss61524274	*rs41619226*	3	37.626826	0.1526	2.0570	0.151960	0.0368	0.0095068225
ss61565968	*rs41664509*	3	37.69426	0.1583	1.1459	0.284778	0.0271	0.0072197523
ss86300268		3	37.719314	0.5752	0.8949	0.344490	0.0179	0.0087335036
ss117962608		3	37.819384	0.5731	1.8886	0.169808	0.0260	0.0127408245
ss86282871		3	37.862781	0.2944	0.0073	0.931801	0.0018	0.0007415553
	rs29012800	3	37.894289	0.8109	2.9789	0.084802	0.0415	0.0127219429
ss86330756		3	38.033865	0.7944	1.6740	0.196147	0.0298	0.0097419193
ss117962982		3	38.158441	0.9004	7.9446	0.004960	0.0869	0.0155782709
	rs29024428	3	38.503083	0.0938	9.4713	0.002169	0.0949	0.0161320515
ss117962679		3	38.578469	0.8223	6.4303	0.011437	0.0614	0.0179284376
ss61489087	*rs41589526*	3	38.712899	0.4212	0.6945	0.404937	0.0156	0.0075869001
ss86313555		3	38.75313	0.3352	0.9498	0.330102	0.0187	0.0083246376
ss61489086	*rs41589525*	3	39.056659	0.1454	0.0049	0.944407	0.0018	0.0004546432
ss86314115		3	39.173539	0.2758	0.6991	0.403380	0.0174	0.0069521198

ss86332281		3	39.220398	0.2342	1.4004	0.237052	0.0253	0.0090638543
ss86326747		3	39.29924	0.3274	3.9264	0.047928	0.0381	0.0167895828
ss86341098		3	39.339311	0.3274	3.9264	0.047928	0.0381	0.0167895828
ss86300136		3	39.366502	0.1010	1.0723	0.300787	0.0314	0.0057031124
ss61552660	*rs43711711*	3	39.603165	0.8983	3.2798	0.070569	0.0560	0.0102415729
	rs29026005	3	39.632431	0.3574	2.2471	0.134317	0.0295	0.0135575061
ss61489080	*rs41589519*	3	39.654836	0.3560	2.6636	0.103124	0.0321	0.0147128317
	rs29014873	3	39.710471	0.4341	2.5221	0.112718	0.0298	0.0146218148
ss86274973		3	39.81105	0.7385	1.3844	0.239758	0.0243	0.0093903941
ss117962577		3	39.864315	0.2915	9.9633	0.001666	0.0644	0.0265938647
ss61489073	*rs41589512*	3	39.904291	0.7944	5.0467	0.024986	0.0523	0.0170852269
ss86280804		3	40.023738	0.3625	9.1831	0.002533	0.0571	0.0263778622
ss86306214		3	40.097499	0.1110	0.0000	0.995676	0.0002	0.0000311064
	rs29012123	3	40.201986	0.6970	5.1271	0.023862	0.0454	0.0191671191
ss61523543	*rs41621709*	3	40.256657	0.7751	9.2424	0.002453	0.0685	0.0238775007
ss86291308		3	40.367336	0.1991	7.7860	0.005409	0.0655	0.0208913207
ss117967164		3	40.456982	0.1963	0.0286	0.865710	0.0040	0.0012668537
ss61494481	*rs41597673*	3	40.696964	0.8188	2.8121	0.094003	0.0399	0.0118554320
ss86304269		3	40.843612	0.0702	0.0210	0.884703	0.0054	0.0007019854
ss86286552		3	40.968961	0.5716	8.4778	0.003710	0.0581	0.0284492054
ss117967155		3	41.001626	0.1117	0.4760	0.490489	0.0207	0.0041119133
ss117962871		3	41.022468	0.8116	0.0188	0.891050	0.0033	0.0009963740
ss61565529	*rs41661577*	3	41.236272	0.7808	0.2314	0.630653	0.0107	0.0036597231
ss86323942		3	41.388051	0.2457	1.2888	0.256655	0.0239	0.0088699785
ss61564649	*rs41662216*	3	41.4862	0.5874	0.0094	0.922927	0.0018	0.0008877550
ss61523845	*rs41623602*	3	41.533517	0.4076	0.7660	0.381754	0.0164	0.0078973179
	rs29011628	3	41.82189	0.4262	0.3008	0.583557	0.0103	0.0050381603
	rs29011976	3	41.842788	0.3625	0.6458	0.421892	0.0155	0.0071476303
ss61566796	*rs41667416*	3	41.868771	0.3181	0.4444	0.505244	0.0131	0.0056981078
ss86298541		3	41.97506	0.4663	3.2904	0.070118	0.0349	0.0173932599
ss117962762		3	41.998519	0.1146	1.9795	0.159887	0.0410	0.0083184036
ss86331946		3	42.025294	0.0745	3.6118	0.057783	0.0665	0.0091762429
ss64755507	*rs42854273*	3	42.08445	0.7357	2.1953	0.138887	0.0318	0.0123602612
	rs29020073	3	42.117153	0.7486	0.0001	0.991695	0.0002	0.0000844586
ss63681643	*rs42309891*	3	42.180969	0.3718	1.2216	0.269436	0.0214	0.0100161576
ss61497421	*rs41596848*	3	42.244025	0.7923	0.8255	0.363889	0.0213	0.0070159356
ss63681075	*rs42318688*	3	42.265299	0.7758	0.0004	0.983254	0.0005	0.0001673186
ss61469981	*rs41565978*	3	42.29615	0.9420	1.8546	0.173694	0.0544	0.0059479190
ss86278139		3	42.320544	0.9126	1.6887	0.194202	0.0429	0.0068381467
ss86336505		3	42.354183	0.3582	0.0665	0.796573	0.0051	0.0023446055
ss61497414	*rs41596841*	3	42.385243	0.6368	2.7757	0.096156	0.0323	0.0149444770
ss86326982		3	42.444472	0.3703	0.3924	0.531226	0.0122	0.0056730621
	rs29014458	3	42.496624	0.2486	0.1389	0.709487	0.0079	0.0029408808
ss61489088	*rs41589527*	3	42.561047	0.4971	2.0766	0.150022	0.0262	0.0131232941
	rs29020672	3	42.717282	0.4957	2.0506	0.152592	0.0261	0.0130396627
ss86335974		3	42.91639	0.3388	0.0152	0.902026	0.0025	0.0011053951
ss61511568	*rs41613479*	3	42.953153	0.6633	0.0201	0.887368	0.0029	0.0012786927

ss64269858	*rs43773468*	3	42.985746	0.8725	3.6358	0.056960	0.0548	0.0121940305
ss64270052	*rs43773286*	3	43.018814	0.3352	0.0080	0.928714	0.0018	0.0008051274
ss64208574	*rs42526271*	3	43.089213	0.1447	2.1164	0.146177	0.0378	0.0093531685
ss64208368	*rs29004022*	3	43.156097	0.2758	0.2663	0.605964	0.0106	0.0042214396
ss117966992		3	43.225816	0.1426	0.2499	0.617300	0.0132	0.0032254062
ss61514875	*rs41616430*	3	43.284601	0.4370	1.5414	0.214834	0.0228	0.0112320281
ss86339227		3	43.337728	0.7027	0.0812	0.775785	0.0058	0.0024270814
ss64563200	*rs42699454*	3	43.374736	0.7221	0.1265	0.722215	0.0074	0.0029843983
ss64722700	*rs42820459*	3	43.40404	0.9026	1.0434	0.307384	0.0324	0.0057062103
ss117962667		3	43.428201	0.5595	0.4314	0.511498	0.0123	0.0060552000
	rs29013986	3	43.502278	0.4556	0.9569	0.328297	0.0183	0.0090886973
	rs29018679	3	43.656584	0.2199	0.3722	0.541997	0.0138	0.0047405945
ss61476900	*rs41580150*	3	43.739934	0.4420	2.1231	0.145542	0.0273	0.0134545671
	rs29013292	3	43.839121	0.4105	0.3009	0.583492	0.0106	0.0051391575
ss86340311		3	43.879876	0.1497	0.8220	0.364902	0.0239	0.0060876308
ss86314822		3	43.951431	0.3166	0.5699	0.450546	0.0152	0.0065771688
ss86341769		3	43.972343	0.5938	0.0069	0.934025	0.0016	0.0007739466
ss61522137	*rs41617640*	3	44.048765	0.2851	6.1083	0.013693	0.0517	0.0210802687
ss61512219		3	44.12966	0.6898	0.0917	0.762056	0.0063	0.0026974946
ss117967033		3	44.190402	0.7794	4.0117	0.045572	0.0452	0.0155298235
ss86315788		3	44.239452	0.0802	0.6894	0.406665	0.0291	0.0042931700
ss61522139	*rs41617642*	3	44.329923	0.1361	0.0399	0.841787	0.0056	0.0013113722
	rs29018229	3	44.394353	0.8567	0.3505	0.554024	0.0162	0.0039731552
	rs29012431	3	44.433531	0.7507	2.6705	0.102676	0.0355	0.0133002043
ss86340364		3	44.462888	0.3274	1.9364	0.164507	0.0274	0.0120493791
ss61569864		3	44.502769	0.7206	4.0173	0.045424	0.0427	0.0172118591
ss86320269		3	44.580799	0.4628	2.5938	0.107739	0.0302	0.0150042262
ss86333229		3	44.607828	0.2178	8.8213	0.003080	0.0648	0.0220864637
ss117962844		3	44.762646	0.2271	8.8460	0.003039	0.0632	0.0221867880
ss86340542		3	44.849843	0.9026	2.4377	0.118908	0.0513	0.0090138379
ss86336834		3	44.905166	0.4542	1.6014	0.206128	0.0233	0.0115750665
	rs29018907	3	44.937193	0.3524	3.1725	0.075324	0.0339	0.0154813786
ss61552681	*rs43712231*	3	44.983849	0.3567	3.5713	0.059202	0.0359	0.0164869771
ss61494966	*rs41592450*	3	45.032184	0.2500	5.3593	0.020902	0.0492	0.0184672748
ss61481236	*rs41576453*	3	45.091947	0.3847	5.1703	0.023280	0.0435	0.0205940063
ss86338782		3	45.142494	0.5330	1.3593	0.244064	0.0218	0.0108377417
ss86284915		3	45.181403	0.9083	1.3091	0.252951	0.0385	0.0064070317
ss61564855	*rs41663817*	3	45.206753	0.2980	7.0942	0.007912	0.0525	0.0219811198
ss86341096		3	45.261894	0.8252	0.3874	0.533863	0.0150	0.0043266727
ss86305780		3	45.595195	0.8718	4.1631	0.041690	0.0562	0.0125678811
	rs29011360	3	45.619248	0.1898	3.6970	0.054919	0.0466	0.0143433293
ss61552796	*rs43707151*	3	45.640498	0.6655	2.7231	0.099358	0.0323	0.0143743093
ss86325236		3	45.697251	0.6791	1.5785	0.209393	0.0249	0.0108443363
ss86285577		3	45.775674	0.0924	0.0751	0.784168	0.0090	0.0015118415
ss61495176	*rs41594758*	3	45.83753	0.5351	0.0351	0.851455	0.0034	0.0017037278
ss86337114		3	45.934868	0.5867	0.7196	0.396564	0.0159	0.0077117739
ss117962596		3	46.037471	0.6476	0.0209	0.885009	0.0028	0.0012957537

ss86332423		3	46.151917	0.8668	0.1026	0.748790	0.0086	0.0019941287
ss86335582		3	46.202712	0.1024	0.6881	0.407087	0.0250	0.0045964050
ss86313396		3	46.292567	0.1268	0.9077	0.341051	0.0260	0.0057617477
ss86325642		3	46.38949	0.7414	1.6361	0.201293	0.0276	0.0105852981
ss86286686		3	46.457017	0.5072	3.1333	0.077145	0.0323	0.0161262896
ss61552718	*rs43712268*	3	46.520677	0.5079	1.2107	0.271572	0.0205	0.0102577633
ss86288096		3	46.547426	0.8274	2.6212	0.105899	0.0407	0.0116157296
ss86331075		3	46.568043	0.7794	0.4145	0.519925	0.0144	0.0049594885
ss61552715	*rs43712265*	3	46.624506	0.4090	0.2958	0.586706	0.0105	0.0050940509
ss86292892		3	46.666806	0.0809	0.3457	0.556762	0.0201	0.0029969915
ss86338078		3	46.763793	0.5566	1.2898	0.256484	0.0209	0.0103219288
ss86288485		3	46.800081	0.4456	2.3715	0.124020	0.0284	0.0140479416
ss86337832		3	46.852798	0.5451	0.2265	0.634299	0.0089	0.0043970557
ss61489103	*rs41590442*	3	46.873033	0.3116	0.7451	0.388325	0.0173	0.0074325174
ss86310821		3	46.996597	0.4721	1.6596	0.198089	0.0235	0.0117071458
ss61510083	*rs41601230*	3	47.166786	0.6125	16.1606	0.000065	0.0754	0.0358053007
ss86278993		3	47.18722	0.5845	25.0105	0.000001	0.0926	0.0449894925
ss117966997		3	47.24771	0.7192	10.5958	0.001188	0.0656	0.0264962120
ss86321815		3	47.359312	0.7364	0.1311	0.717449	0.0080	0.0031077781
ss117962824		3	47.432521	0.5229	0.4693	0.493527	0.0128	0.0063880632
ss86285237		3	47.468075	0.5236	0.1943	0.659488	0.0082	0.0041049571
ss117962818		3	47.54144	0.5315	0.8274	0.363328	0.0171	0.0084964535
ss61552752	*rs43712302*	3	47.640052	0.3424	1.6915	0.193832	0.0258	0.0116066664
ss117967044		3	47.72475	0.7471	2.3715	0.124022	0.0332	0.0125573320
ss61489117	*rs41590454*	3	47.782541	0.3403	3.6918	0.055090	0.0366	0.0164425298
	rs29025951	3	47.803787	0.1067	1.2897	0.256489	0.0346	0.0065972345
ss86340332		3	47.871076	0.5652	2.5602	0.110040	0.0302	0.0148676289
ss86337349		3	48.415448	0.9484	3.0598	0.080695	0.0735	0.0071861494
ss117963688		3	48.668788	0.4355	0.4554	0.499998	0.0129	0.0063366479
ss61552734		3	48.715568	0.1067	0.1357	0.712660	0.0113	0.0021622839
ss117963664		3	48.920959	0.6941	0.0053	0.942206	0.0015	0.0006217506
ss61552720	*rs43712270*	3	49.095767	0.1970	0.0964	0.756280	0.0072	0.0022778428
ss86325714		3	49.259551	0.1160	0.2530	0.615164	0.0139	0.0028605266
ss117967067		3	49.462204	0.6010	0.6266	0.428859	0.0151	0.0072377557
ss64721777	*rs42820066*	3	49.55738	0.7880	1.2604	0.261968	0.0253	0.0084609788
	rs29011654	3	49.638177	0.6297	3.4739	0.062763	0.0351	0.0163542904
ss64851688	*rs42945878*	3	49.661697	0.8825	1.2747	0.259283	0.0319	0.0066243569
ss61526734	*rs41625620*	3	49.688244	0.6297	0.0555	0.813894	0.0046	0.0021245639
ss65023492	*rs43117607*	3	49.711587	0.9377	2.0840	0.149302	0.0567	0.0066275315
ss64323610	*rs42586116*	3	49.738985	0.3682	0.1698	0.680378	0.0080	0.0037250343
ss64323686	*rs42586854*	3	49.778568	0.6318	0.1698	0.680378	0.0080	0.0037250343
ss64615334	*rs42725189*	3	49.850075	0.3446	0.0002	0.989137	0.0003	0.0001206356
ss64915449	*rs43005354*	3	49.905825	0.7729	2.5864	0.108241	0.0354	0.0124282859
ss64817840	*rs42909241*	3	49.963439	0.8804	1.6355	0.201373	0.0355	0.0074736394
	rs29010802	3	49.982201	0.8345	1.1096	0.292545	0.0273	0.0075291228
ss117962853		3	50.021896	0.8030	0.0141	0.905379	0.0029	0.0009070869
ss61527501	*rs41624161*	3	50.065908	0.7013	0.7193	0.396659	0.0170	0.0071199003

ss64868240	*rs42967999*	3	50.093239	0.7744	6.6896	0.009900	0.0549	0.0191974908
ss64473148	*rs42653918*	3	50.138042	0.4570	2.8344	0.092712	0.0308	0.0152629146
ss86308746		3	50.278918	0.7436	0.8246	0.364148	0.0198	0.0075399739
	rs29003226	3	50.318222	0.5895	1.0506	0.305726	0.0194	0.0093977943
ss61562986	*rs41657786*	3	50.351709	0.5838	1.7741	0.183311	0.0248	0.0120471982
ss64166253	*rs42508850*	3	50.392565	0.0595	0.2964	0.586307	0.0213	0.0023786075
ss61526502	*rs41623692*	3	50.462811	0.4950	2.2993	0.129890	0.0275	0.0137684438
ss61507297	*rs41604693*	3	50.48555	0.8911	0.0350	0.851564	0.0055	0.0010698359
ss61562907	*rs41665994*	3	50.558159	0.0595	0.2964	0.586307	0.0213	0.0023786075
ss65586078	*rs43669621*	3	50.585133	0.3703	1.1990	0.273892	0.0210	0.0098116994
ss117967106		3	50.647465	0.5709	1.3312	0.248990	0.0215	0.0105466822
ss61568313	*rs41663111*	3	50.700761	0.4355	1.7694	0.183894	0.0250	0.0123140784
ss64940735	*rs43033012*	3	50.734391	0.1024	1.8373	0.175711	0.0422	0.0077627954
ss65585951	*rs43668594*	3	50.823643	0.6991	0.0454	0.831244	0.0045	0.0018880925
ss61562902	*rs41665989*	3	50.852628	0.9176	1.2898	0.256471	0.0372	0.0056242921
ss61562901	*rs41665988*	3	50.884062	0.2543	0.0002	0.989501	0.0003	0.0001056649
ss65585824	*rs43668168*	3	50.93722	0.7006	0.2440	0.621497	0.0099	0.0041625011
ss65585810	*rs43668154*	3	50.965844	0.5473	0.2335	0.629127	0.0091	0.0045168924
ss86336567		3	50.990747	0.9456	0.1052	0.745817	0.0131	0.0013527197
ss65251970	*rs43343393*	3	51.012902	0.8689	2.1417	0.143794	0.0407	0.0092663442
ss61552762	*rs43712312*	3	51.036932	0.8918	2.8718	0.090592	0.0526	0.0101562811
ss64668001	*rs42763924*	3	51.080481	0.2564	0.0857	0.769766	0.0062	0.0023806876
ss117962765		3	51.155494	0.4126	4.7227	0.030103	0.0418	0.0202784710
ss61475468	*rs41575042*	3	51.190573	0.7908	0.0380	0.845428	0.0044	0.0014608502
ss86319468		3	51.219672	0.1440	0.0940	0.759249	0.0081	0.0020013190
ss65251857	*rs43342280*	3	51.259098	0.7350	1.9007	0.168441	0.0291	0.0113451521
ss65251817	*rs43342240*	3	51.283983	0.1110	0.1969	0.657411	0.0127	0.0025073513
ss61489118	*rs41590455*	3	51.332017	0.1612	0.0087	0.925693	0.0023	0.0006329237
ss117962773		3	51.364124	0.8804	0.8606	0.353893	0.0271	0.0057017761
ss65251600	*rs43339424*	3	51.400769	0.5616	1.4831	0.223700	0.0234	0.0115438970
ss65251588	*rs43339412*	3	51.443092	0.5580	1.4384	0.230813	0.0230	0.0113508908
ss61552755	*rs43712305*	3	51.468871	0.3431	1.6677	0.196996	0.0256	0.0115546614
ss86289027		3	51.495123	0.8610	0.5451	0.460575	0.0196	0.0046912567
ss86290891		3	51.565568	0.7378	1.8652	0.172463	0.0287	0.0111062095
ss86311792		3	51.615652	0.8460	0.0037	0.951819	0.0015	0.0004035794
ss86338748		3	51.641777	0.2994	3.6006	0.058174	0.0389	0.0163016193
ss86311036		3	51.708131	0.5852	0.4952	0.481844	0.0135	0.0065459787
ss86284645		3	51.749745	0.7013	0.6630	0.415778	0.0165	0.0069207582
ss86331773		3	51.838452	0.2536	0.0937	0.759609	0.0065	0.0024524385
ss86302397		3	51.873849	0.4198	0.5734	0.449187	0.0143	0.0069871561
ss86336730		3	51.991861	0.6905	1.6359	0.201314	0.0257	0.0110030115
ss61564976	*rs41657642*	3	52.012236	0.1468	0.5185	0.471711	0.0191	0.0047755518
ss86324280		3	52.086774	0.2486	0.0001	0.992453	0.0002	0.0000793736
ss117967019		3	52.172366	0.9148	0.0078	0.929494	0.0029	0.0004593460
ss61523545	*rs41621711*	3	52.202655	0.1397	2.5532	0.110524	0.0425	0.0102178518
ss86341155		3	52.353722	0.8123	1.4063	0.236081	0.0280	0.0085400963
	rs29024776	3	52.489935	0.0480	0.0088	0.925164	0.0041	0.0003723230

ss61520323	*rs41613250*	3	52.585799	0.8288	0.9331	0.334397	0.0234	0.0066482772
ss86332725		3	52.677326	0.8302	0.6680	0.414035	0.0200	0.0056246502
ss86337214		3	52.680751	0.1698	0.6680	0.414035	0.0200	0.0056246502
ss117967030		3	52.709294	0.8295	0.6469	0.421484	0.0196	0.0055493997
ss86324373		3	52.726551	0.6297	0.0027	0.958878	0.0010	0.0004615684
ss86339072		3	52.759353	0.8345	6.2106	0.012931	0.0636	0.0175728299
ss86309214		3	52.794113	0.2285	2.5849	0.108339	0.0352	0.0123985249
ss86336331		3	52.836515	0.6368	3.4898	0.062168	0.0371	0.0171650171
ss86335145		3	52.859482	0.2629	0.0011	0.973623	0.0007	0.0002690597
	rs29015114	3	52.885251	0.8997	8.9520	0.002870	0.0912	0.0164632829
ss61511422	*rs41612739*	3	52.917632	0.5229	2.0846	0.149246	0.0282	0.0140504205
ss86337083		3	53.040877	0.7264	8.0811	0.004604	0.0609	0.0242289071
ss86338306		3	53.261579	0.8711	5.7689	0.016574	0.0687	0.0154367044
ss86329246		3	53.426654	0.5365	1.8337	0.176129	0.0261	0.0129663119
	rs29016370	3	53.513748	0.8202	0.0100	0.920364	0.0024	0.0007170852
	rs29015745	3	53.545997	0.8460	0.8861	0.346868	0.0240	0.0062477313
ss117963001		3	53.702311	0.8245	0.1739	0.676816	0.0102	0.0029615146
ss86274328		3	53.88275	0.3453	1.3642	0.243206	0.0233	0.0105313313
	rs29023272	3	54.013371	0.3961	0.2240	0.636152	0.0091	0.0043544049
ss61512266	*rs41610874*	3	54.061885	0.4470	0.1530	0.695765	0.0076	0.0037704385
ss117962847		3	54.347354	0.0817	1.2125	0.271219	0.0375	0.0056313198
ss61465403	*rs41565752*	3	54.39323	0.1311	0.1399	0.708508	0.0105	0.0023999338
ss61524017	*rs41624964*	3	54.524107	0.1175	1.4743	0.225084	0.0353	0.0073267143
ss117962816		3	54.677956	0.1160	1.4431	0.230042	0.0351	0.0071981723
ss86287940		3	54.769951	0.5236	1.0824	0.298513	0.0200	0.0099847212
	rs29019879	3	54.878968	0.8446	5.5021	0.019273	0.0601	0.0157701669
ss86283676		3	55.090963	0.4692	1.0024	0.317074	0.0193	0.0096073154
ss117962600		3	55.145705	0.2701	1.1830	0.277128	0.0229	0.0090179963
ss86316403		3	55.192481	0.6289	0.6030	0.437705	0.0148	0.0069170791
ss117962821		3	55.234369	0.3789	1.1842	0.276883	0.0208	0.0098003269
ss86320068		3	55.27847	0.2966	1.2680	0.260526	0.0227	0.0094625065
ss38329857	*rs29027468*	3	55.442801	0.4019	0.3558	0.551034	0.0112	0.0053898492
	rs29027404	3	55.54382	0.2163	0.0214	0.883666	0.0033	0.0011273931
ss86339409		3	55.579427	0.1905	0.1774	0.673777	0.0100	0.0030768572
ss86326363		3	55.606573	0.7951	0.0106	0.917849	0.0024	0.0007931142
ss61552889	*rs43707944*	3	55.679232	0.8446	3.6459	0.056617	0.0516	0.0135477284
ss86313283		3	55.852882	0.7249	1.0195	0.312979	0.0224	0.0089229103
ss61507567	*rs41607859*	3	56.083735	0.9284	4.5781	0.032730	0.0774	0.0102954547
	rs29013313	3	56.11155	0.4026	2.4992	0.114355	0.0312	0.0149969527
ss117962976		3	56.179319	0.4857	1.5148	0.218823	0.0240	0.0119711159
ss117962979		3	56.235823	0.6877	0.6571	0.417869	0.0165	0.0070685578
ss61511384	*rs41611902*	3	56.416977	0.0989	0.0010	0.974460	0.0010	0.0001776832
ss86314554		3	56.527357	0.8095	4.0506	0.044540	0.0479	0.0147612238
ss117962862		3	56.549448	0.1841	0.9809	0.322319	0.0240	0.0072070617
ss61552836	*rs43707191*	3	56.573832	0.1827	3.6003	0.058181	0.0462	0.0138085057
ss61552839	*rs43707194*	3	56.594316	0.8675	3.0847	0.079471	0.0477	0.0109707986
ss117962648		3	56.615223	0.7744	4.0863	0.043615	0.0453	0.0158167137

ss61510095	*rs41601642*	3	56.671796	0.0544	0.6942	0.405018	0.0342	0.0035226910
	rs29012393	3	56.699327	0.4470	3.7453	0.053360	0.0368	0.0181926847
ss86290738		3	56.72382	0.2321	0.1922	0.661244	0.0101	0.0036081802
ss86338225		3	56.802204	0.5924	2.4066	0.121276	0.0306	0.0147722058
ss86340402		3	56.826078	0.3646	0.7299	0.393226	0.0168	0.0077940031
ss86339097		3	56.865751	0.3202	1.4411	0.230370	0.0245	0.0106496461
ss86340875		3	56.970477	0.4026	0.4650	0.495508	0.0133	0.0063988524
ss86327797		3	57.01224	0.4133	0.6453	0.422089	0.0158	0.0076430051
	rs29021598	3	57.084079	0.5910	0.8868	0.346665	0.0184	0.0088738601
ss61552833	*rs43707188*	3	57.113999	0.1705	1.5762	0.209730	0.0303	0.0085797369
ss86336859		3	57.162903	0.5996	1.3842	0.239789	0.0228	0.0109599348
ss86335667		3	57.195196	0.2199	5.9466	0.014994	0.0553	0.0189865897
ss61532239	*rs41634249*	3	57.263996	0.2679	5.5339	0.018929	0.0506	0.0198604534
ss86336626		3	57.339352	0.1784	0.8856	0.347001	0.0224	0.0065549944
ss61570726	*rs41671277*	3	57.396246	0.8216	0.8856	0.347001	0.0224	0.0065549944
ss86339237		3	57.490065	0.8438	2.9016	0.088937	0.0433	0.0114118759
ss64163759	*rs42508010*	3	58.07466	0.2493	1.0579	0.304054	0.0249	0.0093349662
ss86288648		3	58.184581	0.1748	0.4672	0.494524	0.0172	0.0049584469
ss86313863		3	58.308602	0.9040	0.0027	0.958447	0.0016	0.0002836060
ss117966906		3	58.34283	0.9212	0.0177	0.894260	0.0046	0.0006620293
ss61489146	*rs41590476*	3	58.421338	0.1146	0.2735	0.601152	0.0153	0.0031134709
ss86293301		3	58.4754	0.8997	0.0640	0.800406	0.0078	0.0014093502
	rs29018851	3	58.505396	0.2715	1.6955	0.193308	0.0280	0.0110797568
	rs29015774	3	58.584905	0.1082	0.0416	0.838535	0.0061	0.0011845258
ss86292683		3	58.739288	0.1426	1.2574	0.262532	0.0305	0.0074493525
ss117967158		3	58.841486	0.1225	0.5436	0.461200	0.0208	0.0044660157
ss61521542	*rs41620445*	3	59.547392	0.3259	0.1961	0.658033	0.0088	0.0038736332
ss61474706	*rs41571698*	3	59.636445	0.3675	4.3932	0.036443	0.0408	0.0189484849
ss117966919		3	59.701487	0.5673	2.7131	0.099976	0.0310	0.0151993625
ss86338377		3	59.737045	0.1633	4.7250	0.030064	0.0542	0.0148043956
ss117962859		3	59.81914	0.4663	2.8222	0.093419	0.0310	0.0154316253
ss61537520	*rs41640613*	3	59.84056	0.5158	4.9623	0.026225	0.0416	0.0207564426
ss61474642	*rs41571637*	3	59.905073	0.1032	0.6051	0.436919	0.0236	0.0043671727
ss117974988		3	60.025569	0.4699	2.5268	0.112384	0.0298	0.0148227327
ss86330617		3	60.237444	0.1476	0.4315	0.511493	0.0168	0.0042230147
ss86336861		3	60.304883	0.0645	0.0002	0.988078	0.0006	0.0000684023
ss86302457		3	60.349874	0.6082	1.4268	0.232692	0.0230	0.0109815470
ss86337281		3	60.480983	0.3918	1.5060	0.220162	0.0231	0.0110074662
ss117962587		3	60.795059	0.4298	1.6948	0.193398	0.0243	0.0119078143
ss61503625	*rs41608809*	3	61.174841	0.3940	7.8304	0.005280	0.0539	0.0257164406
ss61489155	*rs41590484*	3	61.248092	0.5953	7.8834	0.005129	0.0541	0.0260794392
	rs29020629	3	61.340737	0.8345	1.0035	0.316802	0.0259	0.0071618097
ss61552892	*rs43707947*	3	61.367076	0.5258	3.7555	0.053039	0.0365	0.0181820039
	rs29010802	3	61.444603	0.8345	1.1096	0.292545	0.0273	0.0075291228
ss86334584		3	61.485083	0.2901	0.7892	0.374651	0.0178	0.0073263239
ss61466408		3	61.557633	0.6691	2.0765	0.150035	0.0281	0.0124610524
ss61471018	*rs41566590*	3	61.621627	0.1024	0.1001	0.751793	0.0098	0.0017995578

ss86289861		3	61.711857	0.6626	1.6331	0.201704	0.0252	0.0112798815
ss86338791		3	61.770268	0.0759	0.2826	0.595189	0.0190	0.0026719396
ss86305273		3	61.829475	0.5996	0.1284	0.720163	0.0069	0.0033135925
ss86341077		3	62.057758	0.7650	0.5901	0.442633	0.0170	0.0061138707
ss86335718		3	62.082468	0.2350	0.7893	0.374624	0.0197	0.0070690392
ss86309283		3	62.251618	0.3295	0.2213	0.638217	0.0094	0.0041431668
ss86340196		3	62.423283	0.2715	0.0002	0.988388	0.0003	0.0001206358
ss117962970		3	62.455054	0.4979	0.2332	0.629302	0.0087	0.0043738046
ss86273467		3	62.512719	0.0766	0.0018	0.966481	0.0015	0.0002084468
ss117962989		3	62.603725	0.6218	0.2790	0.597510	0.0102	0.0047933522
ss117962750		3	62.965411	0.7951	0.0116	0.914107	0.0025	0.0008035482
ss61552918	*rs43707973*	3	62.986355	0.5537	0.0760	0.782870	0.0052	0.0025801374
ss86283481		3	63.092824	0.0802	0.0628	0.802191	0.0084	0.0012359712
	rs29020444	3	63.120135	0.9133	0.0087	0.925553	0.0030	0.0004727917
ss61489170	*rs41590498*	3	63.219321	0.9198	0.0628	0.802191	0.0084	0.0012359712
ss117963700		3	63.58731	0.2744	3.5731	0.059139	0.0401	0.0159526665
ss65254151	*rs43338160*	3	63.613695	0.6569	2.5867	0.108219	0.0320	0.0144459763
ss86309717		3	63.637152	0.7436	0.0578	0.810123	0.0050	0.0019012667
ss86308484		3	63.667144	0.2486	0.1666	0.683235	0.0085	0.0031752753
ss86336103		3	63.700627	0.4234	2.2609	0.133128	0.0287	0.0140339844
ss61552974	*rs43708028*	3	63.847027	0.2178	4.0154	0.045473	0.0452	0.0153975305
ss65254909	*rs43345409*	3	63.939085	0.2077	3.1315	0.077231	0.0401	0.0132106190
	rs29026779	3	63.999689	0.2077	3.1315	0.077231	0.0401	0.0132106190
ss117975416		3	64.090602	0.5487	0.6108	0.434769	0.0144	0.0071269448
ss64846907	*rs42941841*	3	64.131842	0.4814	1.4294	0.232268	0.0225	0.0112238215
ss61526548	*rs41624536*	3	64.152773	0.2170	2.9819	0.084644	0.0394	0.0133976555
ss86298824		3	64.193823	0.5279	1.4142	0.234760	0.0218	0.0108513534
ss64043936	*rs42446597*	3	64.219875	0.3933	0.0080	0.928700	0.0017	0.0008088732
ss117967085		3	64.244294	0.1468	1.5206	0.217940	0.0323	0.0080935348
	rs29014148	3	64.276929	0.3617	0.0622	0.803140	0.0048	0.0022025697
ss64043464	*rs42445061*	3	64.333736	0.3424	7.0347	0.008176	0.0523	0.0235614929
ss61528356	*rs41624402*	3	64.37815	0.8252	3.1842	0.074789	0.0439	0.0126635110
ss86341518		3	64.401467	0.6769	6.7552	0.009546	0.0527	0.0230446856
ss61528362	*rs41624408*	3	64.44144	0.2514	3.2142	0.073435	0.0387	0.0145527115
ss61552962	*rs43708016*	3	64.564073	0.5007	0.4701	0.493185	0.0127	0.0063392810
	rs29011253	3	64.589452	0.4491	2.1116	0.146635	0.0272	0.0134546001
ss86340595		3	64.617624	0.6705	0.0187	0.891240	0.0027	0.0011915086
ss65254528	*rs43342535*	3	64.670065	0.1762	0.4713	0.492631	0.0163	0.0047210504
ss86302378		3	64.703944	0.5287	0.9395	0.332731	0.0178	0.0088495313
ss61510119	*rs41601666*	3	64.745171	0.1734	5.1216	0.023938	0.0546	0.0156470801
ss61552959	*rs43708013*	3	64.78197	0.3553	1.4002	0.237099	0.0231	0.0105913610
ss117962785		3	64.805904	0.2500	0.1560	0.693003	0.0085	0.0031745195
ss61520781	*rs41617503*	3	64.834128	0.7135	1.6346	0.201493	0.0266	0.0108606484
ss61498900	*rs43707401*	3	64.912925	0.3410	0.4049	0.524757	0.0125	0.0056007937
ss86338370		3	64.943211	0.5559	1.8453	0.174766	0.0260	0.0128375964
ss61526950	*rs41627219*	3	64.979729	0.4821	0.4550	0.500180	0.0127	0.0063443371
ss64855376	*rs42946940*	3	65.085635	0.5688	0.2181	0.640613	0.0090	0.0044084906

ss86288505		3	65.109108	0.3195	0.5898	0.442762	0.0151	0.0065839724
ss64678777	rs42773336	3	65.158706	0.9112	3.3715	0.066760	0.0600	0.0097198897
	rs29012951	3	65.181704	0.1074	1.3038	0.253907	0.0337	0.0064651013
ss64678882	rs42774341	3	65.201992	0.9083	3.7939	0.051842	0.0629	0.0104847692
ss65255025	rs43346319	3	65.228172	0.4721	0.0367	0.848068	0.0036	0.0017929053
ss61489191	rs41590518	3	65.367977	0.8532	4.6235	0.031880	0.0546	0.0136702244
ss61552986	rs43708640	3	65.411864	0.1153	1.8123	0.178666	0.0385	0.0078574390
ss61552987	rs43708641	3	65.463382	0.2736	1.5690	0.210777	0.0263	0.0104358703
ss86336509		3	65.607	0.4979	4.9880	0.025841	0.0414	0.0207186388
ss117962757		3	65.677457	0.4979	0.0775	0.780774	0.0051	0.0025740067
ss65255304	rs43338999	3	65.706442	0.7665	1.5763	0.209711	0.0276	0.0098773310
ss64572524	rs42708208	3	65.83287	0.6440	5.6434	0.017791	0.0462	0.0211658728
ss64572416	rs42708154	3	65.859623	0.3567	5.3940	0.020494	0.0452	0.0207285672
ss61567985	rs41666483	3	65.885416	0.8904	7.0037	0.008318	0.0784	0.0152962091
ss61567983	rs41666481	3	65.931689	0.8933	6.4360	0.011400	0.0769	0.0146689001
ss86338590		3	65.957568	0.4191	4.6919	0.030643	0.0392	0.0190937829
	rs29016955	3	66.019234	0.2342	0.4731	0.491795	0.0148	0.0052932639
ss117962838		3	66.044068	0.6956	0.0000	0.995123	0.0001	0.0000523594
ss65032937	rs43125678	3	66.074357	0.9198	0.0196	0.888619	0.0048	0.0007108873
ss64998381	rs43088411	3	66.223302	0.5616	0.0766	0.781987	0.0051	0.0025345989
ss86324089		3	66.245128	0.1791	2.1350	0.144420	0.0353	0.0103675638
ss61473001	rs41575220	3	66.290445	0.6347	0.8509	0.356629	0.0176	0.0081552395
ss63819597	rs42368890	3	66.347964	0.0874	4.3406	0.037579	0.0690	0.0110119253
ss61524177	rs41619134	3	66.478479	0.1827	1.6564	0.198520	0.0310	0.0092649909
ss61517740	rs41618104	3	66.506174	0.1891	2.8377	0.092523	0.0400	0.0122540692
ss61517743	rs41618107	3	66.531334	0.1870	0.6586	0.417348	0.0193	0.0058720136
ss64647583	rs42743981	3	66.580787	0.2006	1.3433	0.246857	0.0262	0.0083954073
ss64647560	rs42743958	3	66.608091	0.3546	0.0054	0.941478	0.0014	0.0006356793
ss64647598	rs42743996	3	66.642281	0.3539	0.0054	0.941314	0.0014	0.0006364553
ss86288572		3	66.663749	0.2966	0.1915	0.661795	0.0088	0.0036544487
ss117963708		3	66.716591	0.1139	0.0240	0.877009	0.0046	0.0009267791
ss117963680		3	66.737585	0.5179	2.0103	0.156685	0.0269	0.0134124874
ss64116601	rs42482493	3	66.819934	0.4850	2.0486	0.152800	0.0272	0.0135802651
ss64116557	rs42482471	3	66.846614	0.2085	0.6075	0.435983	0.0179	0.0059163796
ss61511298	rs41610817	3	67.032213	0.8438	2.0580	0.151856	0.0378	0.0099528823
ss117963682		3	67.170996	0.6633	0.3946	0.530073	0.0124	0.0055398395
ss61518179	rs41612148	3	67.34125	0.9112	3.8945	0.048839	0.0651	0.0105351497
ss61526858	rs41626429	3	67.496823	0.0931	3.0780	0.079797	0.0569	0.0096149198
ss61518184	rs41612153	3	67.569263	0.2550	4.2992	0.038496	0.0433	0.0164660713
ss64357803	rs42599294	3	67.64793	0.3596	3.2173	0.073299	0.0357	0.0164563573
	rs29009820	3	67.75911	0.3668	3.8233	0.050943	0.0388	0.0180275730
ss117967104		3	67.8148	0.9105	0.1673	0.682674	0.0136	0.0022204084
ss64677184	rs42775050	3	67.883364	0.1884	3.1151	0.078007	0.0433	0.0132489419
	rs29018018	3	67.904291	0.1383	3.5519	0.059894	0.0509	0.0121228920
ss117966965		3	68.012935	0.2951	0.9624	0.326925	0.0201	0.0083427500
ss117967109		3	68.05886	0.8338	1.5794	0.209271	0.0312	0.0086341215
ss64913596	rs43003409	3	68.081053	0.8453	2.0730	0.150378	0.0372	0.0097251498

ss86335699		3	68.105258	0.0917	0.9125	0.339775	0.0309	0.0051550920
ss65256052	*rs43336845*	3	68.129498	0.7987	0.3538	0.552138	0.0138	0.0044214636
ss65256043	*rs43336836*	3	68.155593	0.3646	0.5972	0.439929	0.0147	0.0067913170
ss65255963	*rs43346157*	3	68.187866	0.6461	0.9047	0.341852	0.0188	0.0085996417
ss61489204		3	68.215262	0.4133	2.0582	0.151840	0.0267	0.0129530184
ss65255785	*rs43344079*	3	68.241075	0.5802	2.1618	0.141928	0.0272	0.0132492619
ss61552993		3	68.276498	0.9047	8.5125	0.003641	0.0911	0.0157041570
ss65255680	*rs43343075*	3	68.348849	0.4835	7.6642	0.005783	0.0507	0.0253428255
ss65255605	*rs43342100*	3	68.369473	0.5043	6.1977	0.013024	0.0457	0.0228419959
ss65255580	*rs43342075*	3	68.413983	0.6605	0.9448	0.331385	0.0189	0.0084749996
ss86337950		3	68.451692	0.4133	2.2776	0.131706	0.0279	0.0135434165
ss64772764	*rs42867941*	3	68.569328	0.3875	1.3255	0.250006	0.0220	0.0104480281
ss61521910	*rs41622407*	3	68.593876	0.1898	0.3243	0.569234	0.0135	0.0041675812
ss64772794	*rs42867969*	3	68.619172	0.7299	0.0448	0.832352	0.0044	0.0017416994
ss117967091		3	68.644514	0.3546	0.1743	0.676460	0.0081	0.0036997298
ss117966917		3	68.710444	0.2063	0.2725	0.601800	0.0119	0.0038914796
ss64679960	*rs42775015*	3	68.750002	0.1741	8.0362	0.004718	0.0682	0.0196080926
ss64679981	*rs42775836*	3	68.770646	0.7944	0.1832	0.668764	0.0097	0.0031701353
ss86315284		3	68.861358	0.0802	1.0024	0.317069	0.0334	0.0049227040
	rs29027369	3	68.897722	0.8725	1.7984	0.180348	0.0376	0.0083698323
ss61474682		3	68.946016	0.7872	1.1132	0.291759	0.0241	0.0080803273
	rs29020116	3	69.041386	0.9126	1.9081	0.167622	0.0459	0.0073213384
ss86339545		3	69.086945	0.4785	0.7521	0.386122	0.0158	0.0078924434
ss61521482	*rs41619587*	3	69.118846	0.4807	0.7418	0.389379	0.0157	0.0078478667
ss61514737	*rs41615396*	3	69.263428	0.5029	0.1150	0.734573	0.0061	0.0030478115
ss61471534	*rs41570992*	3	69.335929	0.8202	5.7984	0.016300	0.0571	0.0168434465
ss61472200	*rs41568840*	3	69.422735	0.3431	2.5001	0.114295	0.0315	0.0142105630
ss86305267		3	69.476506	0.0903	16.2846	0.000061	0.1280	0.0210266313
ss63749459	*rs42335298*	3	69.512713	0.6060	1.5382	0.215304	0.0232	0.0110677831
ss117967061		3	69.538464	0.1884	0.2615	0.609236	0.0121	0.0036960721
ss63748952	*rs42333629*	3	69.586404	0.4105	3.5836	0.058767	0.0354	0.0171313353
ss61514733	*rs41615392*	3	69.622628	0.8080	0.1288	0.719802	0.0084	0.0026123452
ss64559502	*rs42696408*	3	69.834796	0.2443	2.0134	0.156358	0.0301	0.0111166435
ss64559556	*rs42697235*	3	69.86096	0.2249	0.0686	0.793490	0.0057	0.0019703947
ss61474034	*rs41566646*	3	69.920342	0.1411	0.5220	0.470217	0.0188	0.0045483647
ss61520123	*rs41611055*	3	69.947958	0.1633	0.7163	0.397642	0.0215	0.0058726493
ss61507272	*rs41604670*	3	70.008195	0.3410	0.9157	0.338937	0.0185	0.0082980906
ss64836693	*rs42936243*	3	70.035821	0.8847	13.3255	0.000281	0.1056	0.0215453021
	rs29019303	3	70.110222	0.7507	6.6438	0.010155	0.0557	0.0208307585
ss61552909	*rs43707964*	3	70.137985	0.2572	0.7807	0.377219	0.0185	0.0070794806
	rs29027552	3	70.170783	0.0587	0.2858	0.593081	0.0209	0.0023079676
ss61523548	*rs41621714*	3	70.21199	0.8087	0.5506	0.458313	0.0173	0.0053491988
ss61510107	*rs41601654*	3	70.253328	0.4979	1.5926	0.207372	0.0226	0.0113118943
ss86304264		3	70.290838	0.1927	0.7886	0.374829	0.0207	0.0064309487
ss65089040	*rs43178550*	3	70.342067	0.8073	0.7886	0.374829	0.0207	0.0064309487
ss61527296		3	70.378148	0.8460	0.3219	0.570652	0.0146	0.0038038402
ss61519802	*rs41618841*	3	70.523555	0.3517	0.0004	0.984706	0.0004	0.0001657800

ss86330838		3	70.568932	0.2049	2.8394	0.092424	0.0396	0.0128937594
ss61563027	*rs41657824*	3	70.622366	0.3431	5.1268	0.023865	0.0443	0.0199600746
ss117962741		3	70.642478	0.7149	1.2858	0.257216	0.0233	0.0095018201
ss61494166	*rs41594372*	3	70.722378	0.6160	11.0613	0.000928	0.0632	0.0298904556
ss86334313		3	70.747906	0.3768	12.7515	0.000380	0.0683	0.0320633295
ss61517428	*rs41614906*	3	70.781546	0.1447	24.8058	0.000001	0.1309	0.0323933202
ss63805480	*rs42360767*	3	70.851653	0.3510	1.0362	0.309068	0.0197	0.0089689539
ss63805500	*rs42360777*	3	70.879141	0.1812	10.1468	0.001510	0.0769	0.0228316828
	rs29011201	3	70.928101	0.1318	7.7700	0.005457	0.0771	0.0176380042
ss63805626	*rs42361540*	3	70.960408	0.1411	0.6900	0.406454	0.0223	0.0053994520
ss63805742	*rs42361598*	3	70.994401	0.8059	0.0879	0.767010	0.0070	0.0021893913
ss86294137		3	71.030639	0.5573	2.0286	0.154809	0.0263	0.0129776561
ss63805990	*rs42357120*	3	71.072565	0.6361	2.4078	0.121190	0.0304	0.0140956829
ss61472733	*rs41573956*	3	71.099159	0.8059	1.0990	0.294843	0.0244	0.0076293625
ss117967001		3	71.154513	0.3403	4.3143	0.038160	0.0406	0.0182427885
ss86293790		3	71.242168	0.2794	2.4287	0.119585	0.0326	0.0131076065
ss63806372	*rs42359211*	3	71.294714	0.3331	4.5934	0.032441	0.0419	0.0186369698
ss86315051		3	71.355624	0.8116	3.2917	0.070062	0.0435	0.0132916676
ss61556134	*rs41651446*	3	71.38673	0.8138	3.3974	0.065723	0.0444	0.0134723115
ss61563180	*rs41659269*	3	71.419333	0.3216	3.9969	0.045973	0.0394	0.0172027815
ss61563176	*rs41659265*	3	71.47848	0.3245	3.6363	0.056945	0.0379	0.0166296609
ss86315873		3	71.519802	0.5695	2.1095	0.146836	0.0266	0.0130216662
ss64172253	*rs42509344*	3	71.544906	0.8037	12.7233	0.000386	0.0805	0.0253823225
ss64822121	*rs42915167*	3	71.593404	0.2314	2.9184	0.088020	0.0377	0.0134022605
ss117966951		3	71.711225	0.2414	3.4431	0.063938	0.0388	0.0142108433
ss86323245		3	71.734055	0.7901	2.6885	0.101527	0.0361	0.0119892734
ss61553072	*rs43708725*	3	71.841367	0.5258	0.0061	0.937699	0.0015	0.0007376055
ss65257305	*rs43339294*	3	71.893119	0.3345	0.1508	0.697846	0.0078	0.0034904318
ss86325610		3	71.969672	0.3152	0.2262	0.634504	0.0096	0.0041363149
ss117962623		3	72.16444	0.4585	5.3243	0.021323	0.0419	0.0207822209
ss46526596	*rs41257053*	3	72.272225	0.6039	1.3832	0.239954	0.0228	0.0108937946
ss64470184	*rs42657137*	3	72.305545	0.0946	0.0008	0.977519	0.0009	0.0001533569
ss63507485	*rs42257870*	3	72.332307	0.8797	0.1991	0.655569	0.0129	0.0027328720
ss63507668	*rs42258931*	3	72.372752	0.3990	1.0180	0.313344	0.0196	0.0093822305
ss64956941	*rs43050707*	3	72.415577	0.4119	0.7307	0.392935	0.0165	0.0079940475
	rs29021260	3	72.437475	0.3381	1.4331	0.231664	0.0236	0.0105694359
ss61489235	*rs41591362*	3	72.457513	0.5265	0.0080	0.928576	0.0017	0.0008428236
ss65256973	*rs43346461*	3	72.482293	0.8954	0.3416	0.559070	0.0179	0.0033517467
ss65392289	*rs43475092*	3	72.526077	0.3173	2.1780	0.140451	0.0292	0.0126592683
ss86330140		3	72.593011	0.0967	0.2625	0.608590	0.0164	0.0028628960
ss86290018		3	72.666515	0.7751	2.4364	0.119003	0.0344	0.0120036048
ss63508922	*rs42253045*	3	72.789724	0.5788	2.3941	0.122246	0.0296	0.0144426974
ss63509030	*rs42253081*	3	72.825895	0.6691	2.7154	0.099838	0.0329	0.0145856786
ss63509060	*rs42253091*	3	72.855926	0.7536	1.0644	0.302566	0.0215	0.0079925362
ss86303183		3	72.889966	0.5516	1.7832	0.182193	0.0247	0.0122029726
ss63509942	*rs42254485*	3	72.921243	0.2385	1.0757	0.300028	0.0226	0.0082096597
ss86321299		3	72.95043	0.3424	3.2461	0.072028	0.0360	0.0161997140

ss86337582		3	72.983839	0.6383	8.1594	0.004412	0.0552	0.0254917520
ss63510587	*rs42255400*	3	73.00643	0.1905	0.5202	0.470986	0.0173	0.0053390731
ss64653499	*rs42750404*	3	73.072227	0.3804	0.2424	0.622616	0.0093	0.0044028643
ss64653462	*rs42750367*	3	73.094693	0.6289	0.3841	0.535612	0.0118	0.0055175782
ss61516796	*rs41618796*	3	73.115759	0.2650	6.8762	0.008926	0.0549	0.0213713353
ss86323144		3	73.200318	0.4463	5.0272	0.025267	0.0407	0.0200948316
ss63510710	*rs42255841*	3	73.221021	0.3517	5.0158	0.025432	0.0430	0.0196048061
ss61567138	*rs41660658*	3	73.242459	0.7830	0.5425	0.461640	0.0165	0.0055962964
ss61567133	*rs41660653*	3	73.274977	0.2221	0.5578	0.455417	0.0166	0.0057522586
ss86296533		3	73.303237	0.7636	0.2093	0.647449	0.0103	0.0037145206
ss64301122	*rs42576134*	3	73.32964	0.2421	0.5958	0.440449	0.0173	0.0063631948
ss64767215	*rs42860352*	3	73.357488	0.4549	2.6242	0.105696	0.0297	0.0147526117
ss86337961		3	73.38888	0.9004	0.1234	0.725499	0.0106	0.0019069555
ss86318395		3	73.446461	0.3424	2.1871	0.139624	0.0287	0.0129075294
ss117967152		3	73.476141	0.4742	8.5984	0.003475	0.0529	0.0263807046
ss61553081	*rs43709334*	3	73.508286	0.4140	5.6929	0.017300	0.0456	0.0221480709
ss65257963	*rs43343148*	3	73.543874	0.6970	2.3421	0.126373	0.0309	0.0130331987
ss61553078	*rs43709331*	3	73.624653	0.4764	0.3357	0.562525	0.0107	0.0053375787
ss64653050	*rs42758151*	3	73.723406	0.1590	0.0293	0.864069	0.0042	0.0011365000
ss65257670	*rs43341855*	3	73.83264	0.4033	4.4634	0.034984	0.0398	0.0191512514
ss65257637	*rs43341122*	3	73.855358	0.5358	5.5162	0.019120	0.0426	0.0211903175
ss65257607	*rs43341092*	3	73.882918	0.4033	4.4634	0.034984	0.0398	0.0191512514
ss117962444		3	73.903657	0.1347	0.0526	0.818745	0.0061	0.0014142959
ss64728984	*rs42827379*	3	73.931935	0.5752	8.0174	0.004767	0.0514	0.0251147022
ss86300170		3	73.974035	0.2242	1.0446	0.307099	0.0221	0.0076722477
ss65258725	*rs43344302*	3	74.007643	0.2450	0.2952	0.587081	0.0118	0.0043754665
ss86331112		3	74.042818	0.2715	0.2600	0.610316	0.0103	0.0040551418
ss65258759	*rs43345236*	3	74.063463	0.5809	0.9049	0.341814	0.0182	0.0088768908
ss65258812	*rs43345289*	3	74.106971	0.7693	0.0581	0.809520	0.0052	0.0018595242
ss65258829	*rs43345306*	3	74.131724	0.7264	0.9753	0.323695	0.0204	0.0080950620
ss61489254	*rs41591381*	3	74.162526	0.7693	0.0581	0.809520	0.0052	0.0018595242
ss65258692	*rs43344269*	3	74.192313	0.4183	0.0892	0.765238	0.0058	0.0028029303
ss65258649	*rs43343326*	3	74.215125	0.3847	2.5267	0.112386	0.0310	0.0146618877
ss65258597	*rs43343274*	3	74.26524	0.6605	10.5898	0.001192	0.0624	0.0280012151
ss65258431	*rs43341212*	3	74.301817	0.7357	14.4156	0.000159	0.0786	0.0305626793
ss61468731	*rs41572042*	3	74.334471	0.7357	14.4156	0.000159	0.0786	0.0305626793
ss117967073		3	74.378175	0.2593	14.8772	0.000125	0.0798	0.0306461702
ss61468730	*rs41572041*	3	74.415938	0.6268	4.7785	0.029150	0.0413	0.0193120733
ss61489251	*rs41591378*	3	74.450683	0.1017	0.0555	0.813778	0.0074	0.0013590979
	rs29013555	3	74.485925	0.7980	0.3453	0.556951	0.0133	0.0042830808
ss61553082	*rs43709335*	3	74.512971	0.3632	13.6674	0.000235	0.0697	0.0322254763
ss86300903		3	74.62047	0.1003	1.0075	0.315861	0.0308	0.0055629510
ss65258915	*rs43346590*	3	74.710935	0.1956	0.9855	0.321185	0.0230	0.0072433570
ss65258902	*rs43346577*	3	74.734151	0.7564	0.9545	0.328914	0.0212	0.0078279175
ss65259012	*rs43347387*	3	74.761256	0.5759	0.1983	0.656250	0.0084	0.0041155106
ss86332140		3	74.781904	0.5931	0.5754	0.448358	0.0145	0.0069969113
ss65259030	*rs43347405*	3	74.809054	0.1504	6.7115	0.009780	0.0664	0.0169748229

ss117963694		3	74.825632	0.5494	0.1510	0.697726	0.0072	0.0035831884
ss38332492	*rs29015993*	3	74.862523	0.8582	14.1128	0.000187	0.0985	0.0239775555
ss64341401	*rs42590727*	3	74.944183	0.3926	4.9119	0.026995	0.0419	0.0199691712
ss64903859	*rs42998274*	3	74.980952	0.2815	4.5968	0.032378	0.0440	0.0177987424
ss65220805	*rs43312703*	3	75.024418	0.6447	6.7898	0.009364	0.0515	0.0235795800
ss61541587	*rs41645118*	3	75.064564	0.6426	6.9273	0.008677	0.0519	0.0238369585
ss65220637	*rs43311641*	3	75.095922	0.3503	6.2929	0.012348	0.0495	0.0225281051
ss65220586	*rs43310690*	3	75.120716	0.6375	0.5342	0.465110	0.0139	0.0064202767
ss117963711		3	75.168358	0.4613	3.4090	0.065265	0.0359	0.0178483291
ss63497540	*rs42252935*	3	75.231044	0.7958	0.1869	0.665644	0.0103	0.0033378031
ss86301909		3	75.251161	0.1755	0.8642	0.352881	0.0222	0.0064374209
ss63497405	*rs42251791*	3	75.273117	0.1325	0.2406	0.623958	0.0138	0.0031713622
ss63984961	*rs42417908*	3	75.455836	0.6175	0.1984	0.656140	0.0085	0.0040334934
ss64834056	*rs42926915*	3	75.563512	0.3868	7.3981	0.006692	0.0511	0.0242594359
	rs29012776	3	75.607988	0.5279	6.9327	0.008651	0.0483	0.0240879477
ss63985025	*rs42418540*	3	75.692381	0.2593	5.6549	0.017675	0.0514	0.0197431822
ss86338647		3	75.715079	0.4706	5.3765	0.020699	0.0426	0.0212465950
ss63985283	*rs42419369*	3	75.763541	0.5344	8.5710	0.003527	0.0529	0.0263474462
ss63985545	*rs42420200*	3	75.805092	0.5974	1.4072	0.235933	0.0220	0.0105987765
ss63985689	*rs42420772*	3	75.82726	0.3832	5.6946	0.017284	0.0452	0.0213624842
ss64184113	*rs42516309*	3	75.867852	0.6340	0.7016	0.402531	0.0164	0.0076322469
ss61563674	*rs41663343*	3	75.896207	0.4176	0.0599	0.806757	0.0046	0.0022315159
ss61563673	*rs41663342*	3	75.948617	0.2564	0.2881	0.591619	0.0116	0.0044274948
ss61511272	*rs41610793*	3	76.043621	0.5251	0.0885	0.766154	0.0055	0.0027325770
ss61494296	*rs41595699*	3	76.079748	0.3231	0.0892	0.765301	0.0058	0.0025270139
ss63962079	*rs42407171*	3	76.126748	0.7586	13.0911	0.000318	0.0778	0.0284931191
ss64183415	*rs42513464*	3	76.159843	0.6225	4.3308	0.037794	0.0400	0.0187914973
ss64719959	*rs42821878*	3	76.235999	0.5451	2.0175	0.155944	0.0268	0.0132687946
ss86277882		3	76.335272	0.8259	0.7284	0.393689	0.0207	0.0059385868
ss86336186		3	76.445863	0.5437	2.1446	0.143524	0.0276	0.0136783989
ss117966959		3	76.469247	0.8281	5.2217	0.022607	0.0570	0.0162179878
ss64922303	*rs43016827*	3	76.492015	0.5630	3.0327	0.082043	0.0316	0.0155525995
ss63858652	*rs42385282*	3	76.514633	0.0709	0.0078	0.929754	0.0032	0.0004234693
ss65259169	*rs43342752*	3	76.54369	0.9248	3.4820	0.062460	0.0671	0.0093300830
ss63961907	*rs42406185*	3	76.636864	0.0301	0.0197	0.888330	0.0077	0.0004466815
ss63961709	*rs42404786*	3	76.693895	0.5946	10.9503	0.000984	0.0613	0.0295349891
ss63961511	*rs42403890*	3	76.759195	0.3574	2.0101	0.156704	0.0270	0.0124161034
ss61494778	*rs41590367*	3	76.820482	0.3990	2.3408	0.126482	0.0289	0.0138480983
ss63858458	*rs42384185*	3	76.852805	0.9112	0.2646	0.607130	0.0162	0.0026193036
ss61473879	*rs41574389*	3	76.884746	0.0895	0.2509	0.616588	0.0157	0.0025624632
ss86298123		3	77.219474	0.4334	4.9789	0.025976	0.0411	0.0201719058
ss86279666		3	77.31249	0.6232	7.2669	0.007193	0.0522	0.0245279321
ss86338046		3	77.488732	0.2722	0.0005	0.982201	0.0005	0.0001897307
ss86295019		3	77.550705	0.2686	0.0104	0.918951	0.0021	0.0008170593
ss117966912		3	77.638249	0.3302	1.0596	0.303665	0.0204	0.0090071805
ss61569157	*rs41665543*	3	77.71454	0.6354	0.0137	0.906723	0.0023	0.0010626580
ss86319112		3	77.786835	0.1318	1.8855	0.170152	0.0359	0.0082134934

	rs29026932	3	77.883375	0.5086	0.3306	0.565483	0.0106	0.0053201951
ss61477190	*rs41575437*	3	78.21892	0.4742	4.1865	0.041122	0.0373	0.0185761324
ss61549154	*rs41646851*	3	78.360898	0.4542	7.9060	0.005066	0.0512	0.0253807641
ss86278119		3	78.413115	0.5516	11.8600	0.000608	0.0634	0.0313755036
ss117966932		3	78.648495	0.8854	2.8420	0.092281	0.0490	0.0099498535
ss62471068	*rs41883544*	3	78.709111	0.7006	4.2810	0.038910	0.0417	0.0174943676
ss117967002		3	78.732586	0.7020	4.1139	0.042913	0.0410	0.0171531633
ss61472214	*rs41568853*	3	78.870021	0.1648	3.5719	0.059179	0.0454	0.0125082471
ss86326256		3	79.053464	0.8073	14.2320	0.000175	0.0883	0.0274592510
ss63431986	*rs42232131*	3	79.080389	0.1891	14.4935	0.000153	0.0889	0.0272614525
ss61515999	*rs41617819*	3	79.106892	0.8109	14.4935	0.000153	0.0889	0.0272614525
ss61570065	*rs41664831*	3	79.269651	0.8095	8.5750	0.003519	0.0659	0.0203227877
	rs29017982	3	79.391363	0.4155	1.2033	0.273048	0.0202	0.0097905896
ss61515997	*rs41617817*	3	79.416475	0.4155	1.2033	0.273048	0.0202	0.0097905896
ss86326070		3	79.449973	0.9076	0.0004	0.983653	0.0007	0.0001122718
ss61515970	*rs41617810*	3	79.515575	0.6039	7.4874	0.006372	0.0507	0.0242646092
ss61570064	*rs41672106*	3	79.585347	0.1490	1.0781	0.299478	0.0270	0.0068420118
	rs29010020	3	79.725603	0.2299	0.3579	0.549849	0.0131	0.0046540629
ss86298075		3	79.765906	0.8417	0.0592	0.807804	0.0060	0.0016096240
ss63431098	*rs42229148*	3	79.800924	0.4928	0.0879	0.766923	0.0053	0.0026628924
ss65259478	*rs43345760*	3	79.831715	0.8424	0.0069	0.933819	0.0021	0.0005468763
	rs29011390	3	79.938256	0.1132	0.3463	0.556391	0.0165	0.0033177626
ss61553093	*rs43709346*	3	80.058269	0.8431	15.8946	0.000074	0.0996	0.0263494418
ss61553094	*rs43709347*	3	80.167909	0.4011	1.1310	0.287930	0.0198	0.0095289912
ss61564475	*rs41661446*	3	80.240718	0.6096	5.7824	0.016448	0.0449	0.0213839605
ss86337838		3	80.567584	0.6984	0.9293	0.335390	0.0195	0.0082196903
ss86281562		3	80.591322	0.8918	0.0070	0.933315	0.0024	0.0004626297
ss61489267	*rs41591394*	3	80.614728	0.1168	0.2042	0.651487	0.0123	0.0025444569
ss61489268	*rs41591395*	3	80.650979	0.3173	0.0113	0.915453	0.0021	0.0009053838
ss61553091	*rs43709344*	3	80.750927	0.3560	0.0006	0.980574	0.0005	0.0002153916
ss86313608		3	80.775811	0.4169	10.5758	0.001201	0.0603	0.0293254525
ss86319999		3	80.803941	0.3166	12.5604	0.000420	0.0703	0.0304306315
ss61489271		3	80.866697	0.5451	5.6943	0.017286	0.0445	0.0220656781
ss65259356	*rs43344538*	3	80.89663	0.0881	0.2740	0.600853	0.0168	0.0026995447
ss86298637		3	80.942916	0.3130	6.5097	0.010942	0.0515	0.0221355876
ss86337695		3	81.031989	0.8947	0.2671	0.605464	0.0150	0.0028318052
ss64949571	*rs43041636*	3	81.060752	0.2049	11.8595	0.000608	0.0774	0.0252168835
ss61465469	*rs41565816*	3	81.099017	0.4520	8.8420	0.003046	0.0540	0.0267582423
ss64484242	*rs42663767*	3	81.122824	0.9054	0.2158	0.642436	0.0146	0.0024922569
ss64484172	*rs42663733*	3	81.14836	0.1110	0.0914	0.762434	0.0088	0.0017366543
ss86299093		3	81.303069	0.5158	4.8909	0.027322	0.0398	0.0198587938
ss86309999		3	81.385248	0.8954	0.2204	0.638893	0.0137	0.0025623996
ss65260429	*rs43345096*	3	81.419939	0.4542	1.4496	0.229009	0.0229	0.0113458633
ss86309132		3	81.44682	0.7636	0.5215	0.470453	0.0154	0.0055745405
ss65259813	*rs43348283*	3	81.466896	0.6418	9.7528	0.001865	0.0602	0.0276989930
ss65094846	*rs43181080*	3	81.487137	0.3145	4.9366	0.026614	0.0448	0.0193249352
ss65260316	*rs43344383*	3	81.51011	0.6146	0.4002	0.527219	0.0117	0.0055245761

ss65260302	*rs43344369*	3	81.539056	0.1039	3.5776	0.058977	0.0563	0.0104781092
ss65056336	*rs43144863*	3	81.579175	0.3660	16.1304	0.000066	0.0749	0.0347427818
ss61496022	*rs41595579*	3	81.618229	0.1117	4.7578	0.029499	0.0620	0.0123160373
ss65260247	*rs43344016*	3	81.676032	0.8968	3.3212	0.068819	0.0553	0.0102384204
ss65260018	*rs43350187*	3	81.864478	0.5702	1.1796	0.277805	0.0196	0.0096188313
ss86288489		3	81.893468	0.4678	3.2843	0.070378	0.0333	0.0165986873
ss61565485	*rs41661534*	3	81.931965	0.5681	0.9195	0.337940	0.0174	0.0085305786
ss64417020	*rs42635145*	3	81.976981	0.4312	0.9041	0.342011	0.0172	0.0084465642
ss64417042	*rs42635156*	3	82.002322	0.0903	0.6158	0.432884	0.0252	0.0041365601
ss117962583		3	82.133364	0.4986	1.7634	0.184642	0.0243	0.0121264517
ss117967099		3	82.39558	0.4936	4.9823	0.025925	0.0403	0.0201471442
ss65260672	*rs43346939*	3	82.497035	0.6053	12.1376	0.000525	0.0643	0.0307184639
ss61563611	*rs41662682*	3	82.517748	0.4728	4.3213	0.038005	0.0381	0.0190002379
ss64939903	*rs43031685*	3	82.576424	0.8854	1.7542	0.185782	0.0376	0.0076351440
ss86296504		3	82.668812	0.3059	10.7591	0.001089	0.0650	0.0275910187
ss86292520		3	82.752476	0.3883	7.0653	0.008040	0.0504	0.0239502805
ss117962627		3	82.776355	0.1411	9.0001	0.002796	0.0801	0.0194209649
ss86333492		3	82.868497	0.1547	0.2159	0.642304	0.0115	0.0030175191
ss61503642	*rs41608825*	3	82.931006	0.4893	5.5097	0.019190	0.0421	0.0210645508
ss65261042	*rs43348908*	3	82.977419	0.2436	9.7728	0.001845	0.0679	0.0250159473
ss61553101	*rs43709353*	3	82.999534	0.8281	4.5849	0.032601	0.0525	0.0149474575
ss64885780	*rs42976033*	3	83.14399	0.8761	0.0440	0.833930	0.0058	0.0012548098
ss65261197	*rs43345563*	3	83.225901	0.7063	4.4393	0.035478	0.0429	0.0178044350
ss65261167	*rs43345533*	3	83.261784	0.2006	2.7138	0.099932	0.0385	0.0123531685
ss64934239	*rs43024325*	3	83.319472	0.1046	0.2448	0.620901	0.0149	0.0027819460
ss65261223	*rs43345589*	3	83.34637	0.6024	7.1251	0.007779	0.0514	0.0246321735
ss61520215	*rs41612244*	3	83.438865	0.9370	1.5295	0.216599	0.0456	0.0053813781
ss86299929		3	83.503176	0.8832	0.4910	0.483733	0.0203	0.0041903126
ss86306041		3	83.539384	0.0781	0.0498	0.823483	0.0078	0.0011164918
ss61492976	*rs41590924*	3	83.57204	0.6139	10.3553	0.001351	0.0582	0.0275908039
ss65497028	*rs43577860*	3	83.612483	0.4262	10.0610	0.001581	0.0562	0.0274924869
ss61492975	*rs41590923*	3	83.635288	0.9620	0.0001	0.991967	0.0005	0.0000360202
ss86336503		3	83.693383	0.4907	9.6749	0.001944	0.0554	0.0277011421
ss65054853	*rs43141384*	3	83.729933	0.9592	0.0020	0.964200	0.0021	0.0001666564
ss64147923	*rs42498349*	3	83.78507	0.9413	0.0101	0.919784	0.0040	0.0004440315
ss86315636		3	83.8977	0.4542	3.1401	0.076828	0.0322	0.0159616654
ss61531611	*rs41628066*	3	83.932855	0.0817	1.1983	0.274040	0.0371	0.0055694868
ss86339089		3	83.986885	0.4807	5.1208	0.023949	0.0402	0.0200930708
ss86336630		3	84.013718	0.6440	1.8680	0.172145	0.0261	0.0119473209
ss61494640	*rs41588130*	3	84.059607	0.1347	0.4749	0.490988	0.0188	0.0043872000
ss63720329	*rs42330368*	3	84.096582	0.1468	0.1320	0.716525	0.0095	0.0023885825
ss61513384		3	84.144241	0.6146	2.1748	0.140736	0.0280	0.0132641707
ss61524045	*rs41624992*	3	84.27904	0.2514	0.4642	0.495872	0.0143	0.0053792276
ss61513383	*rs41612064*	3	84.337696	0.1182	0.2945	0.587536	0.0160	0.0033370778
ss117963652		3	84.435498	0.3854	4.8483	0.028000	0.0414	0.0196240842
ss63681975	*rs42312244*	3	84.619369	0.6203	5.8624	0.015722	0.0454	0.0213625433
ss63682051	*rs42312278*	3	84.65473	0.7994	0.3756	0.540165	0.0140	0.0045053691

ss61505871	*rs41600002*	3	84.684646	0.8001	0.6397	0.424091	0.0183	0.0058649044
ss61497468	*rs41596894*	3	84.70533	0.8302	0.0050	0.943763	0.0017	0.0004871415
ss63682393	*rs42313519*	3	84.750517	0.1676	0.0083	0.927405	0.0022	0.0006242969
ss86291480		3	84.774883	0.0559	0.0701	0.791322	0.0109	0.0011524193
ss86294398		3	84.85202	0.6977	0.1866	0.665873	0.0088	0.0036970109
ss61497459	*rs41596885*	3	84.880187	0.3023	0.1866	0.665873	0.0088	0.0036970109
ss63683350	*rs42317838*	3	84.953502	0.5544	7.4173	0.006622	0.0502	0.0248025863
ss63683548	*rs42317902*	3	85.027212	0.5337	9.6283	0.001994	0.0579	0.0288406707
ss62126668	*rs41761090*	3	85.411459	0.7643	0.0889	0.765635	0.0065	0.0023506934
ss117963669		3	85.497114	0.3331	1.4187	0.234031	0.0233	0.0103522439
ss86335574		3	85.794693	0.8990	2.2678	0.132540	0.0460	0.0083534026
ss86339921		3	85.953209	0.3524	0.0062	0.937361	0.0015	0.0006955404
ss61565546	*rs41661594*	3	86.033329	0.3102	0.1019	0.749630	0.0063	0.0026893272
ss61553112	*rs43709364*	3	86.128901	0.9348	0.0035	0.952979	0.0022	0.0002709108
ss86316224		3	86.181018	0.9054	0.0017	0.966868	0.0013	0.0002242284
ss86297836		3	86.23596	0.7715	0.0565	0.812124	0.0051	0.0018153616
ss61554798	*rs41647843*	3	86.273937	0.7557	1.1268	0.288831	0.0221	0.0081678930
ss86293135		3	86.305707	0.5193	2.6334	0.105093	0.0296	0.0147891855
ss61513250	*rs41611534*	3	86.358481	0.5444	3.0147	0.082954	0.0315	0.0156168695
ss61513245	*rs41610629*	3	86.38708	0.8123	1.0094	0.315400	0.0236	0.0071870961
ss64485864	*rs42661582*	3	86.417391	0.1067	0.6011	0.438440	0.0238	0.0045451496
	rs29019654	3	86.441966	0.9033	0.2465	0.619682	0.0158	0.0027605232
ss65262868	*rs43352135*	3	86.479894	0.6189	3.0177	0.082804	0.0337	0.0159024600
ss86339474		3	86.508407	0.3675	2.4182	0.120389	0.0305	0.0141646506
ss65263002	*rs43353669*	3	86.548134	0.5774	2.0988	0.147868	0.0277	0.0135142202
ss86338707		3	86.582346	0.2342	1.7548	0.185707	0.0286	0.0102708793
ss61553143	*rs43709395*	3	86.622155	0.1698	2.3699	0.124150	0.0379	0.0106856797
ss65263111	*rs43344871*	3	86.666233	0.9162	0.2705	0.603163	0.0174	0.0026768788
ss86334741		3	86.835079	0.5881	1.2367	0.266487	0.0209	0.0101466089
ss65263548	*rs43348806*	3	86.882323	0.4599	1.1797	0.277797	0.0194	0.0096483166
ss86338320		3	86.973361	0.3403	2.2374	0.135156	0.0287	0.0128966838
ss64849259	*rs42940865*	3	86.997973	0.8446	0.4937	0.482518	0.0185	0.0048594103
ss86340112		3	87.027619	0.6669	1.0822	0.298565	0.0202	0.0089694858
ss117967021		3	87.052958	0.6110	2.1745	0.140769	0.0276	0.0131189679
ss86330871		3	87.074102	0.7758	0.6230	0.430202	0.0183	0.0063608566
ss86287773		3	87.102164	0.7736	0.7142	0.398357	0.0194	0.0067905489
ss64893358	*rs42986163*	3	87.180145	0.8281	4.3815	0.036693	0.0527	0.0150082745
ss61507543	*rs41607835*	3	87.208303	0.0630	0.0968	0.755742	0.0120	0.0014218945
ss65262371	*rs43347438*	3	87.271614	0.2113	0.0233	0.878794	0.0036	0.0011897776
ss61553117	*rs43709369*	3	87.291653	0.5552	0.0215	0.883381	0.0028	0.0013892316
ss65261975	*rs43350440*	3	87.335135	0.0544	0.0694	0.792275	0.0108	0.0011152060
ss117967082		3	87.355269	0.2808	2.9506	0.086290	0.0357	0.0144388498
ss86291693		3	87.41628	0.3080	0.0105	0.918537	0.0021	0.0008783309
ss65261871	*rs43349836*	3	87.445558	0.8617	0.1244	0.724400	0.0096	0.0022796625
ss65261863	*rs43349328*	3	87.471248	0.2708	5.6836	0.017391	0.0497	0.0196402274
ss61553156	*rs43709408*	3	87.513313	0.3009	0.1095	0.740850	0.0067	0.0028304876
ss64789316	*rs42880121*	3	87.577317	0.2944	1.3060	0.253513	0.0236	0.0098190119

ss61553155	*rs43709407*	3	87.616495	0.6003	1.4794	0.224275	0.0234	0.0112524581
ss86335683		3	87.644021	0.6046	1.7658	0.184340	0.0259	0.0123707410
ss86313824		3	87.685318	0.2665	0.1644	0.685250	0.0086	0.0033439321
ss61553150	*rs43709402*	3	87.783883	0.7858	1.0715	0.300977	0.0236	0.0079354076
ss65263618	*rs43349476*	3	87.858211	0.3933	0.0026	0.959331	0.0010	0.0004767091
ss46526158	*rs41255301*	3	87.921868	0.8682	0.1946	0.659254	0.0122	0.0027897677
	rs29011278	3	88.184838	0.7428	0.2695	0.603843	0.0110	0.0041892933
ss61512633	*rs41614437*	3	88.325882	0.1862	2.8027	0.094555	0.0403	0.0122259143
ss61512632	*rs41614436*	3	88.349108	0.7500	1.1293	0.288293	0.0230	0.0086073731
ss65263954	*rs43352110*	3	88.566065	0.0509	0.0000	0.994585	0.0003	0.0000281511
ss61553172	*rs43709424*	3	88.821377	0.0652	0.0309	0.860498	0.0067	0.0008172106
ss61489316	*rs41592540*	3	89.047395	0.5702	0.0661	0.797150	0.0050	0.0024428303
ss65264396	*rs43350054*	3	89.150703	0.2199	0.2227	0.637149	0.0106	0.0036453865
ss61489275	*rs41591401*	3	89.241122	0.7242	6.7301	0.009679	0.0555	0.0221762296
ss65264872	*rs43354730*	3	89.275156	0.3847	4.5123	0.034005	0.0414	0.0195969794
ss61553178	*rs43709430*	3	89.315144	0.1168	1.4472	0.229388	0.0356	0.0073491899
ss117963691		3	89.340658	0.1168	1.4472	0.229388	0.0356	0.0073491899
ss61553194	*rs43710446*	3	89.385772	0.5473	1.7101	0.191404	0.0251	0.0124451285
ss61510144	*rs41601689*	3	89.433789	0.2643	1.9315	0.165040	0.0286	0.0111335804
ss86301129		3	89.55043	0.2944	0.3713	0.542477	0.0123	0.0051246685
ss65264676	*rs43353434*	3	89.577439	0.2328	0.0007	0.978988	0.0006	0.0002062302
ss61466598	*rs41571250*	3	89.759604	0.0645	0.3603	0.548528	0.0222	0.0026763004
ss86326219		3	89.949601	0.7135	2.0246	0.155216	0.0290	0.0118552855
ss86339407		3	89.994248	0.8181	0.8108	0.368192	0.0215	0.0064139843
ss64329392	*rs42587297*	3	90.132271	0.3109	1.1796	0.277823	0.0216	0.0092744175
ss64329380	*rs42587291*	3	90.17114	0.4986	0.0138	0.906419	0.0023	0.0011293592
ss86306855		3	90.193429	0.7493	0.8169	0.366409	0.0198	0.0074274859
ss64329120	*rs42586161*	3	90.244053	0.4771	0.0609	0.805169	0.0047	0.0023346295
	rs29013475	3	90.283494	0.6289	0.8348	0.361215	0.0176	0.0082027060
ss65264513	*rs43351271*	3	90.315654	0.8768	0.2801	0.596812	0.0152	0.0032851153
ss65270258	*rs43352693*	3	90.370706	0.1361	0.4075	0.523445	0.0173	0.0040644917
ss86329444		3	90.49672	0.7622	0.2026	0.652808	0.0099	0.0035970540
ss86306392		3	90.520815	0.3424	0.0158	0.899916	0.0026	0.0011585480
ss86274219		3	90.590105	0.9549	0.4841	0.486809	0.0315	0.0027160310
ss86301010		3	90.651243	0.3904	0.3644	0.546289	0.0120	0.0057160177
ss65265067	*rs43356825*	3	90.735288	0.9277	0.2575	0.611992	0.0187	0.0025160827
ss65265078	*rs43348336*	3	90.762985	0.9269	0.2413	0.623444	0.0181	0.0024478597
ss61553197	*rs43710449*	3	90.790767	0.1039	2.6035	0.107083	0.0501	0.0093209722
ss61468758	*rs41572068*	3	90.871517	0.2393	3.0301	0.082176	0.0382	0.0139057586
ss61553198	*rs43710450*	3	90.895159	0.1590	5.7849	0.016424	0.0621	0.0166178331
ss86294478		3	90.92619	0.6619	1.1380	0.286456	0.0216	0.0096546239
ss86303944		3	90.951609	0.4835	0.3824	0.536520	0.0122	0.0060787095
ss86337320		3	91.016994	0.5172	0.2808	0.596315	0.0104	0.0052047079
ss86306444		3	91.054776	0.2536	0.7765	0.378517	0.0190	0.0072080578
ss86332467		3	91.075882	0.6590	1.2603	0.261986	0.0227	0.0102032272
ss61567590	*rs41663596*	3	91.098339	0.7443	1.1936	0.274977	0.0236	0.0089898982
ss86323862		3	91.128035	0.7249	3.8279	0.050804	0.0420	0.0167545423

ss64552970	*rs42692576*	3	91.153077	0.9198	0.2534	0.614828	0.0173	0.0025531929
ss61514656	*rs41614117*	3	91.183723	0.4943	0.4225	0.515888	0.0128	0.0064202629
ss61506197	*rs41603415*	3	91.216203	0.5193	0.6914	0.405967	0.0152	0.0075841256
ss86318964		3	91.237383	0.3274	1.0360	0.309103	0.0204	0.0089914609
ss64324676	*rs42582745*	3	91.257727	0.9312	2.5736	0.109112	0.0591	0.0075708530
ss65182158	*rs43272732*	3	91.27074	0.4950	0.1048	0.746222	0.0059	0.0029553966
ss117967059		3	91.323694	0.4491	0.2954	0.586980	0.0103	0.0051042677
ss86339747		3	91.433937	0.4069	0.9602	0.327475	0.0181	0.0087285598
ss61531664	*rs41628116*	3	91.470068	0.4749	0.0322	0.857689	0.0033	0.0016395405
ss64177125	*rs42508255*	3	91.5957	0.8438	0.2622	0.608804	0.0128	0.0033665587
ss64176993	*rs42518488*	3	91.615873	0.2543	2.0631	0.151356	0.0301	0.0113974905
ss86308882		3	91.67974	0.6211	1.8235	0.177334	0.0255	0.0120154774
ss64176951	*rs42518468*	3	91.701503	0.0530	1.8510	0.174104	0.0565	0.0056738028
ss64176889	*rs42518437*	3	91.741757	0.2163	0.6310	0.427246	0.0177	0.0059968222
ss86296817		3	91.784495	0.9398	6.5585	0.010648	0.1005	0.0113637370
ss63879658	*rs41702751*	3	91.831643	0.4979	0.0802	0.777095	0.0052	0.0025775023
ss61520533	*rs41615557*	3	91.894236	0.1218	1.7037	0.192232	0.0384	0.0082187102
ss61520531	*rs41615555*	3	91.928235	0.3317	0.0294	0.864002	0.0032	0.0014403355
ss61474181	*rs41567393*	3	92.007241	0.9312	0.3299	0.565906	0.0205	0.0026250814
ss63879248	*rs41701446*	3	92.027497	0.9312	0.3299	0.565906	0.0205	0.0026250814
ss63879224	*rs41701434*	3	92.050073	0.8804	10.4828	0.001262	0.0889	0.0187334121
ss86298085		3	92.116681	0.2536	1.2032	0.273066	0.0232	0.0087968892
ss61553375	*rs43711424*	3	92.143728	0.8173	0.1621	0.687367	0.0099	0.0029428737
ss64684444	*rs42782925*	3	92.195871	0.3481	0.0131	0.909044	0.0022	0.0010153366
ss63879152	*rs41701198*	3	92.21744	0.5989	0.5828	0.445484	0.0143	0.0068645040
ss61489332		3	92.376913	0.0752	2.9651	0.085521	0.0605	0.0084152355
	rs29011460	3	92.52826	0.4814	0.7278	0.393885	0.0154	0.0076954636
ss65265411	*rs43351669*	3	92.549869	0.8166	0.0384	0.844754	0.0048	0.0014293712
ss86332320		3	92.572145	0.1862	0.0002	0.987738	0.0004	0.0001156154
ss86338304		3	92.607452	0.2457	0.6477	0.421213	0.0179	0.0066271425
ss86282254		3	92.631144	0.2428	0.7883	0.374910	0.0200	0.0073440396
ss86340209		3	92.672938	0.5595	0.0002	0.988584	0.0003	0.0001299500
ss64811458	*rs42909019*	3	92.706568	0.2586	0.0359	0.849836	0.0040	0.0015526944
ss86312885		3	92.766671	0.6282	0.1568	0.692272	0.0077	0.0036162796
ss61496300	*rs41598251*	3	92.79488	0.3811	0.3936	0.530594	0.0122	0.0057533303
ss86306281		3	92.831943	0.3782	0.4688	0.493748	0.0134	0.0062817040
ss61567621	*rs41663626*	3	92.862403	0.3775	0.9361	0.333629	0.0189	0.0088872115
ss86283312		3	92.885957	0.3617	0.4231	0.515617	0.0129	0.0059440450
ss61511786	*rs41615093*	3	92.92609	0.3990	0.0004	0.983620	0.0004	0.0001920393
ss86335813		3	93.058462	0.3446	1.9045	0.168022	0.0273	0.0123094956
ss86336018		3	93.173992	0.4936	4.9497	0.026415	0.0416	0.0208122386
	rs29011302	3	93.216104	0.1691	0.1784	0.672842	0.0106	0.0029785954
	rs29013441	3	93.241676	0.8309	0.1784	0.672842	0.0106	0.0029785954
ss86338742		3	93.341628	0.9319	0.3003	0.583890	0.0201	0.0025440258
ss86336977		3	93.414088	0.3181	0.4889	0.484655	0.0140	0.0060836698
ss86295015		3	93.451092	0.9670	4.5440	0.033383	0.1109	0.0070695471
ss86311685		3	93.485128	0.1927	0.7547	0.385296	0.0204	0.0063394205

ss86331651		3	93.536566	0.4076	0.0000	0.995369	0.0001	0.0000530879
ss86338394		3	93.587807	0.6648	0.0447	0.832533	0.0042	0.0018814465
ss61553384	*rs43712133*	3	93.612743	0.6648	0.0447	0.832533	0.0042	0.0018814465
	rs29026707	3	93.639483	0.6648	0.0447	0.832533	0.0042	0.0018814465
ss86306440		3	93.681825	0.7815	1.5013	0.220880	0.0273	0.0093352906
ss61553383	*rs43712132*	3	93.732192	0.4011	0.7423	0.389238	0.0162	0.0077878472
ss86322594		3	93.770581	0.2185	1.2507	0.263804	0.0250	0.0085228086
ss86309573		3	93.810949	0.7335	4.8367	0.028189	0.0458	0.0179233456
ss86280402		3	93.831753	0.3617	3.4658	0.063073	0.0359	0.0165756225
ss61510151	*rs41601696*	3	94.058166	0.2428	0.3814	0.537046	0.0135	0.0049797870
	rs29024002	3	94.144417	0.8417	0.1342	0.714253	0.0097	0.0025847561
ss61489339	*rs41592563*	3	94.282287	0.1447	0.0223	0.881458	0.0039	0.0009773541
ss86288517		3	94.355703	0.8711	1.5668	0.211097	0.0352	0.0079157861
ss61553215	*rs43710467*	3	94.427866	0.7858	2.2652	0.132763	0.0353	0.0118919267
ss65265781	*rs43356138*	3	94.542661	0.0652	0.0018	0.965918	0.0016	0.0001965214
ss86330938		3	94.640511	0.6110	2.3612	0.124841	0.0288	0.0136939829
ss61466814	*rs41573355*	3	94.699519	0.8739	0.1240	0.724895	0.0103	0.0022600272
ss65267284	*rs43350241*	3	94.727458	0.2665	0.0209	0.885108	0.0030	0.0011642105
ss86304187		3	94.753075	0.4183	0.0023	0.961518	0.0009	0.0004450136
ss86310217		3	94.81238	0.3331	0.2627	0.608406	0.0100	0.0044218539
ss38333751	*rs29017457*	3	94.928169	0.4585	0.1146	0.735110	0.0062	0.0030886427
ss86323532		3	94.969977	0.6297	0.4291	0.512625	0.0124	0.0057883646
ss65267209	*rs43349367*	3	95.058684	0.9219	0.7973	0.372196	0.0310	0.0044614045
ss65267195	*rs43349353*	3	95.08206	0.4606	2.4919	0.114891	0.0289	0.0143822689
ss86298216		3	95.106706	0.6139	2.8264	0.093172	0.0326	0.0154677912
ss86332833		3	95.15475	0.5688	2.9433	0.086681	0.0329	0.0161385899
ss65266974	*rs43357330*	3	95.181288	0.7744	2.1452	0.143466	0.0336	0.0117523981
ss86300944		3	95.20702	0.2350	2.8432	0.092209	0.0373	0.0134003952
ss65266842	*rs43356099*	3	95.232249	0.7973	2.4062	0.121311	0.0362	0.0117174473
ss65266827	*rs43356084*	3	95.256035	0.2385	3.0769	0.079850	0.0388	0.0141076579
ss86317814		3	95.322761	0.7371	2.6470	0.104195	0.0346	0.0134043411
ss65266641	*rs43353998*	3	95.350049	0.9212	1.0291	0.310723	0.0359	0.0052109700
ss86340052		3	95.37673	0.2693	1.1912	0.275456	0.0230	0.0090489605
ss86296097		3	95.412858	0.7758	10.6554	0.001151	0.0733	0.0254837587
ss86302422		3	95.454943	0.6096	1.7228	0.189769	0.0256	0.0121800236
ss65266505	*rs43352862*	3	95.482777	0.0731	2.0697	0.150696	0.0524	0.0070995110
ss65266473	*rs43351630*	3	95.552051	0.7278	13.8483	0.000214	0.0794	0.0314542737
ss65266420	*rs43351577*	3	95.572116	0.1734	5.7041	0.017191	0.0615	0.0176180034
ss86327007		3	95.593073	0.8066	4.8796	0.027501	0.0549	0.0171251270
ss117962682		3	95.631563	0.2708	15.7688	0.000079	0.0845	0.0333819322
ss61523551	*rs41621717*	3	95.667142	0.4126	5.7694	0.016569	0.0460	0.0223136570
ss61553227	*rs43710478*	3	95.699975	0.7163	11.5329	0.000723	0.0725	0.0294479925
ss86295982		3	95.731783	0.7629	0.2343	0.628506	0.0104	0.0037683248
ss65266083	*rs43348541*	3	95.776745	0.2027	2.5661	0.109633	0.0367	0.0118792802
ss86331728		3	95.822716	0.7629	2.7781	0.096011	0.0366	0.0132286810
ss65265629	*rs43354086*	3	95.878095	0.2099	4.4299	0.035672	0.0476	0.0158038677
ss64772992	*rs42869465*	3	95.973373	0.3123	0.1874	0.665211	0.0086	0.0036757324

ss61492069	*rs41593228*	3	96.068608	0.1583	0.0804	0.776842	0.0073	0.0019453594
ss46526142	*rs41255285*	3	96.096303	0.3768	0.1974	0.656959	0.0084	0.0039559180
ss86289741		3	96.12431	0.1232	0.4036	0.525430	0.0181	0.0039163311
ss117967064		3	96.195938	0.2779	0.0372	0.847019	0.0040	0.0016063777
ss86287219		3	96.219071	0.2808	0.0034	0.953296	0.0012	0.0004929499
ss63989029	*rs42421203*	3	96.230917	0.9054	2.1678	0.141379	0.0471	0.0080645614
ss86319764		3	96.258763	0.1325	2.7999	0.094718	0.0460	0.0105871279
ss117966975		3	96.300984	0.7264	0.1924	0.661073	0.0092	0.0036669773
ss86330505		3	96.331876	0.2736	0.1924	0.661073	0.0092	0.0036669773
ss61507225	*rs41603623*	3	96.360209	0.7199	0.0466	0.829170	0.0045	0.0018205298
ss63989205	*rs42422691*	3	96.387229	0.1798	1.7298	0.188869	0.0322	0.0094910249
ss86331167		3	96.412878	0.3546	1.2143	0.270861	0.0213	0.0097400326
ss65267378	*rs43350935*	3	96.480248	0.7772	0.1555	0.693494	0.0089	0.0030786181
ss65267391	*rs43350948*	3	96.503146	0.7758	0.1493	0.699360	0.0087	0.0030163508
ss86286434		3	96.532586	0.9427	2.7328	0.098758	0.0664	0.0071772114
ss61499915	*rs41596293*	3	96.555842	0.3739	0.0044	0.947061	0.0013	0.0006002856
ss61976012	*rs41712521*	3	96.578677	0.3274	0.0082	0.927788	0.0018	0.0008016420
ss61507292	*rs41604688*	3	96.633292	0.2729	0.0580	0.809711	0.0050	0.0020021452
ss117966957		3	96.77542	0.7056	0.4122	0.521083	0.0131	0.0054322198
ss63645574	*rs42299785*	3	96.805309	0.5208	0.7920	0.373812	0.0169	0.0084505112
ss63645319	*rs42298801*	3	96.969829	0.0924	0.0045	0.946793	0.0022	0.0003620508
ss61472442	*rs41570877*	3	97.065634	0.7693	2.5765	0.108915	0.0340	0.0120674750
	rs29026676	3	97.108858	0.7335	6.8526	0.009043	0.0554	0.0216391253
ss63790266	*rs42351691*	3	97.136392	0.0723	0.2392	0.624922	0.0175	0.0023451056
ss86314553		3	97.166421	0.8976	1.9328	0.164891	0.0426	0.0078312852
ss117966999		3	97.188259	0.6246	0.0712	0.789692	0.0053	0.0024668688
ss86317706		3	97.233213	0.4004	4.4509	0.035240	0.0391	0.0187574348
ss86338011		3	97.281718	0.2221	2.3700	0.124138	0.0343	0.0118432937
ss61516448		3	97.385777	0.9133	0.0575	0.810566	0.0082	0.0012904176
ss86339443		3	97.415177	0.5888	2.9198	0.087944	0.0321	0.0155430242
ss63789458	*rs42353684*	3	97.451623	0.7643	3.8978	0.048745	0.0446	0.0160544034
ss86303600		3	97.485271	0.2586	7.1988	0.007469	0.0591	0.0226797295
ss63788978	*rs42352144*	3	97.572112	0.5989	1.5336	0.215992	0.0234	0.0112525480
ss64915978	*rs43011369*	3	97.595189	0.3926	1.7594	0.185139	0.0252	0.0120154389
ss61553222	*rs43710473*	3	97.71122	0.2715	3.5416	0.060265	0.0394	0.0155926187
ss86293788		3	97.734438	0.3789	1.8942	0.169177	0.0268	0.0126258537
ss61468765	*rs41572075*	3	97.777601	0.1211	0.9327	0.334492	0.0275	0.0058498891
ss65265987	*rs43358044*	3	97.816403	0.2629	1.1217	0.289910	0.0224	0.0086635069
ss86288969		3	97.854125	0.9112	7.1516	0.007666	0.0905	0.0146472719
ss86337090		3	97.880203	0.0874	6.5580	0.010651	0.0872	0.0139159473
ss86333084		3	97.900931	0.3854	0.4508	0.502194	0.0130	0.0061732262
ss65267404	*rs43350961*	3	97.993198	0.4448	3.1944	0.074324	0.0329	0.0162347263
ss86274499		3	98.059928	0.8797	0.3543	0.551879	0.0164	0.0034780973
ss86336484		3	98.122606	0.4835	0.8579	0.354650	0.0167	0.0083338241
ss86325458		3	98.14527	0.6999	0.6759	0.411269	0.0171	0.0071664277
ss86300805		3	98.167287	0.5552	1.9381	0.164322	0.0269	0.0133066336
ss86320790		3	98.206563	0.7908	7.3808	0.006756	0.0606	0.0200600136

ss61489358	*rs41592581*	3	98.236333	0.6168	3.4950	0.061973	0.0363	0.0171674042
ss86323211		3	98.259481	0.8711	1.1565	0.282573	0.0291	0.0065438165
ss86288308		3	98.281044	0.4169	2.7141	0.099919	0.0305	0.0148510781
ss86308445		3	98.304233	0.3489	0.6166	0.432597	0.0151	0.0068445878
ss65267694	*rs43354149*	3	98.325824	0.2607	3.5553	0.059770	0.0396	0.0152571887
ss86339074		3	98.359306	0.6268	3.9069	0.048482	0.0367	0.0171869852
ss86341776		3	98.404381	0.3338	0.0329	0.856191	0.0036	0.0015831734
ss65267715	*rs43354170*	3	98.424595	0.9484	0.5273	0.467981	0.0305	0.0029871523
ss61489360	*rs41592583*	3	98.44862	0.6483	1.3464	0.246309	0.0216	0.0098418867
ss86291759		3	98.476735	0.3725	2.7505	0.097673	0.0305	0.0142619650
ss86316205		3	98.554537	0.1605	4.0615	0.044256	0.0529	0.0142435421
ss86318353		3	98.60103	0.5072	1.7761	0.183061	0.0247	0.0123300541
ss86324910		3	98.639043	0.6526	0.5554	0.456384	0.0145	0.0065872571
ss86286359		3	98.743279	0.0967	1.7415	0.187380	0.0416	0.0072623634
	rs29010425	3	98.789044	0.2915	1.5192	0.218155	0.0247	0.0101868649
ss86282769		3	98.84603	0.2787	0.9767	0.323359	0.0211	0.0084913810
ss65267972	*rs43355825*	3	98.897924	0.0595	1.6337	0.201614	0.0499	0.0055832610
ss65267957	*rs43355810*	3	98.948429	0.6454	0.0429	0.835888	0.0039	0.0017985599
ss61553280		3	98.972095	0.2679	2.1022	0.147542	0.0301	0.0118050958
ss86333392		3	98.99294	0.8381	0.0006	0.981012	0.0006	0.0001615125
ss65267882	*rs43355736*	3	99.016209	0.3668	0.1512	0.697513	0.0075	0.0034648356
ss86287927		3	99.038682	0.6268	0.2438	0.621639	0.0095	0.0044328194
	rs29026911	3	99.0785	0.3610	0.5638	0.453005	0.0144	0.0066294900
ss86299403		3	99.101196	0.3625	0.7098	0.399789	0.0162	0.0075056251
ss86336070		3	99.16591	0.3947	3.4863	0.062300	0.0354	0.0168963254
ss86308456		3	99.214605	0.2801	0.2083	0.648243	0.0095	0.0038350196
ss86296998		3	99.235603	0.5279	0.3008	0.583557	0.0099	0.0049233727
ss86275340		3	99.257136	0.5652	0.4483	0.503351	0.0124	0.0060704644
ss86341733		3	99.28016	0.2278	0.0004	0.983826	0.0004	0.0001524289
ss61553286	*rs43711336*	3	99.356803	0.9291	0.5861	0.444180	0.0283	0.0037228596
ss117963655		3	99.395954	0.8367	6.3156	0.012193	0.0641	0.0175061635
ss86336266		3	99.480131	0.7092	0.6736	0.412081	0.0170	0.0070155502
ss65268340	*rs43351188*	3	99.566585	0.2908	0.5840	0.445008	0.0158	0.0065355244
ss86289164		3	99.636629	0.2163	0.0444	0.833256	0.0048	0.0016311063
ss86321581		3	99.663066	0.1777	0.7715	0.380051	0.0219	0.0064112322
ss61533185	*rs41630751*	3	99.728784	0.6254	0.6922	0.405709	0.0157	0.0073427445
ss61479747	*rs41581806*	3	99.787373	0.5086	4.8747	0.027578	0.0410	0.0204757020
ss61500906	*rs41602049*	3	99.822518	0.5630	1.4663	0.226344	0.0227	0.0111886964
ss86332758		3	99.843061	0.2937	2.3286	0.127474	0.0305	0.0126664012
ss86310873		3	99.883978	0.6877	0.6337	0.426273	0.0161	0.0069363982
ss64598676	*rs42718732*	3	99.943369	0.5430	0.0215	0.883423	0.0027	0.0013391756
ss117962941		3	99.98331	0.1433	0.2439	0.621586	0.0133	0.0032725392
ss64606456	*rs42720314*	3	100.102898	0.4606	0.1000	0.751948	0.0059	0.0029280074
ss86281269		3	100.125557	0.4713	3.6019	0.058127	0.0372	0.0185556602
ss86315067		3	100.150361	0.1999	5.2464	0.022290	0.0522	0.0166985711
ss86283614		3	100.191284	0.3023	1.4112	0.235268	0.0244	0.0103055296
ss86306816		3	100.236951	0.8711	1.8770	0.171118	0.0380	0.0085423475

ss64434642	*rs42635403*	3	100.281682	0.1483	0.2923	0.588910	0.0143	0.0036119551
ss86288294		3	100.312943	0.0580	0.1687	0.681409	0.0162	0.0017745980
ss64434368	*rs42634366*	3	100.347774	0.4155	3.7339	0.053724	0.0358	0.0173681643
ss117962944		3	100.384188	0.2880	7.9314	0.004996	0.0549	0.0225292534
ss86339671		3	100.503117	0.2958	10.0772	0.001567	0.0611	0.0254461407
ss61489388	*rs41592610*	3	100.610696	0.3517	0.0445	0.833045	0.0040	0.0018442849
ss86313792		3	100.727789	0.7701	6.1159	0.013635	0.0537	0.0190162038
ss86338789		3	100.751982	0.5860	0.0647	0.799309	0.0048	0.0023211915
ss65269217	*rs43351756*	3	100.772128	0.4842	0.6241	0.429790	0.0147	0.0073442513
	rs29014008	3	100.811877	0.6547	2.3249	0.127774	0.0302	0.0136445114
ss65269291	*rs43351829*	3	100.927598	0.7278	0.4479	0.503565	0.0140	0.0055665391
ss65269320	*rs43352958*	3	100.951373	0.7256	0.3421	0.558787	0.0123	0.0048920928
ss65269782	*rs43357219*	3	100.977476	0.5824	5.8075	0.016216	0.0455	0.0221229618
ss61510148	*rs41601693*	3	101.025936	0.1024	0.4972	0.480991	0.0220	0.0040431051
ss86339531		3	101.147226	0.6218	0.4341	0.510224	0.0126	0.0059041981
ss86341720		3	101.294222	0.1841	18.5126	0.000019	0.0989	0.0297180148
ss65269845	*rs43358182*	3	101.424752	0.8009	1.5285	0.216755	0.0280	0.0089431306
ss117967128		3	101.449661	0.8223	0.9659	0.326055	0.0240	0.0070235984
ss86337512		3	101.500652	0.8968	0.6447	0.422296	0.0250	0.0046202288
ss65269905	*rs43359040*	3	101.563252	0.8968	0.6447	0.422296	0.0250	0.0046202288
ss65269933	*rs43359068*	3	101.597035	0.1060	0.6153	0.433056	0.0244	0.0046178836
ss86295007		3	101.632422	0.7428	0.0139	0.906128	0.0025	0.0009562356
ss65269968	*rs43359103*	3	101.666585	0.4183	2.4213	0.120148	0.0296	0.0144020332
ss86336676		3	101.766893	0.0530	21.8981	0.000003	0.1945	0.0195247498
ss65271932	*rs43360856*	3	101.818085	0.0838	1.3347	0.248365	0.0387	0.0059399992
ss86324091		3	101.916159	0.3023	3.2346	0.072530	0.0361	0.0152483267
ss61510150	*rs41601695*	3	101.978576	0.2479	1.2365	0.266534	0.0240	0.0089541643
ss65104430	*rs43191232*	3	102.006659	0.4814	3.6304	0.057144	0.0353	0.0176380469
ss61567540	*rs41663547*	3	102.083637	0.8059	0.8536	0.355849	0.0216	0.0067497698
ss46526266	*rs41255405*	3	102.131006	0.4914	4.7788	0.029145	0.0403	0.0201658957
ss117967010		3	102.159584	0.2221	8.8898	0.002968	0.0665	0.0229618038
ss65271093	*rs43356919*	3	102.279127	0.6576	4.0250	0.045218	0.0388	0.0174651926
ss86334022		3	102.307044	0.4126	1.9857	0.159243	0.0263	0.0127495792
ss65271036	*rs43356863*	3	102.444366	0.6325	0.3445	0.557423	0.0112	0.0052283935
ss65270952	*rs43356479*	3	102.498972	0.3560	0.3274	0.567386	0.0110	0.0050499125
ss117966934		3	102.529383	0.5415	0.0652	0.798561	0.0048	0.0023616810
ss61553397	*rs43712146*	3	102.597897	0.4756	0.1855	0.666796	0.0079	0.0039539291
ss65270859	*rs43355686*	3	102.623429	0.8897	3.0628	0.080546	0.0502	0.0098518469
ss61553394	*rs43712143*	3	102.645276	0.2607	0.0247	0.875262	0.0033	0.0012686666
ss86330819		3	102.697572	0.7371	0.0231	0.879157	0.0032	0.0012353742
ss61510152	*rs41601697*	3	102.850265	0.9155	0.1135	0.736331	0.0113	0.0017421899
ss117962733		3	102.87242	0.8926	1.6267	0.202579	0.0388	0.0074377731
ss86321328		3	102.914611	0.1325	5.5173	0.019108	0.0641	0.0147348135
ss86299997		3	102.940006	0.4599	7.5498	0.006157	0.0501	0.0248814790
	rs29018282	3	102.962478	0.1110	4.0926	0.043454	0.0600	0.0118399963
ss61553386	*rs43712135*	3	103.001186	0.6297	13.1850	0.000303	0.0685	0.0319508743
	rs29016142	3	103.040222	0.7070	7.3559	0.006849	0.0549	0.0227403919

ss64693527	*rs42792479*	3	103.199921	0.4706	4.5352	0.033555	0.0395	0.0197030730
ss61467018		3	103.236734	0.4835	4.8896	0.027344	0.0405	0.0202293497
ss86309342		3	103.299002	0.6827	1.1211	0.290059	0.0208	0.0090120750
	rs29023698	3	103.365893	0.8467	2.5445	0.111136	0.0419	0.0108662705
ss61535328	*rs41633663*	3	103.403872	0.7314	7.1729	0.007576	0.0551	0.0216535886
ss65158018	*rs43248153*	3	103.467073	0.1533	2.0246	0.155222	0.0378	0.0098010292
ss86314417		3	103.499469	0.4269	0.0647	0.799291	0.0047	0.0023000180
ss65271422	*rs43355452*	3	103.52879	0.1196	1.7181	0.190375	0.0376	0.0079290074
ss86340648		3	103.556154	0.6232	2.1038	0.147387	0.0276	0.0129743542
ss65271563	*rs43356693*	3	103.623458	0.2307	0.1010	0.750727	0.0071	0.0025373472
ss65271602	*rs43357732*	3	103.706279	0.2342	2.4209	0.120183	0.0341	0.0122472706
	rs29019997	3	103.727191	0.1082	17.5092	0.000032	0.1210	0.0233444854
ss65271675	*rs43357805*	3	103.755622	0.6125	5.3863	0.020584	0.0450	0.0213802821
ss86337774		3	103.797934	0.4026	3.6408	0.056792	0.0370	0.0177855564
ss86298052		3	103.869105	0.3116	0.5013	0.479181	0.0146	0.0062543868
ss65271765	*rs43358795*	3	103.896128	0.1347	3.3071	0.069412	0.0498	0.0115987013
ss86292878		3	103.918893	0.5000	0.0001	0.990534	0.0002	0.0001115917
ss65271823	*rs43359553*	3	103.94496	0.8539	16.8594	0.000045	0.1041	0.0259708745
ss64936221	*rs43024570*	3	103.991325	0.2350	1.7614	0.184885	0.0281	0.0101057836
ss61489428	*rs41585055*	3	104.014218	0.2314	1.3995	0.237217	0.0251	0.0089216649
ss61468777	*rs41572087*	3	104.061183	0.0831	5.0942	0.024315	0.0778	0.0118554241
ss61553433	*rs43712181*	3	104.122687	0.2708	0.0011	0.973833	0.0007	0.0002731209
	rs29018615	3	104.179957	0.1583	9.1724	0.002548	0.0762	0.0203148373
ss86295233		3	104.213691	0.8990	1.4570	0.227819	0.0372	0.0067498246
ss86338405		3	104.30574	0.4928	0.0102	0.919490	0.0019	0.0009410808
ss65274996	*rs43364305*	3	104.326083	0.9434	13.4638	0.000262	0.1450	0.0154839787
ss86340918		3	104.352203	0.6633	2.6425	0.104490	0.0314	0.0140174805
ss86331339		3	104.415649	0.4814	0.0004	0.983173	0.0004	0.0001957798
ss86296801		3	104.452415	0.4814	0.0004	0.983173	0.0004	0.0001957798
	rs29011444	3	104.508446	0.0910	0.1449	0.703539	0.0119	0.0019615221
ss86337895		3	104.528897	0.3052	13.5919	0.000245	0.0744	0.0315382115
ss117963658		3	104.551721	0.5244	0.1007	0.751052	0.0059	0.0029457200
ss86326019		3	104.612143	0.6297	0.1006	0.751203	0.0063	0.0029189142
ss65275682	*rs43361287*	3	104.634626	0.4298	0.7351	0.391518	0.0158	0.0077500107
ss65275762	*rs43361567*	3	104.657501	0.3596	1.3123	0.252371	0.0222	0.0102374310
ss61553449	*rs43712197*	3	104.69855	0.7135	0.2234	0.636573	0.0098	0.0040052501
ss65275944	*rs43362649*	3	104.739813	0.6211	0.2007	0.654285	0.0089	0.0041733558
ss61553453	*rs43712201*	3	104.781233	0.3037	0.1434	0.705028	0.0078	0.0033105400
ss86331531		3	104.813541	0.1039	0.9694	0.325182	0.0311	0.0057872570
ss61553460	*rs43712208*	3	104.850915	0.5996	0.0847	0.771073	0.0057	0.0027245386
ss86328731		3	104.916608	0.4384	0.3205	0.571499	0.0107	0.0052728694
ss64491720	*rs42663864*	3	104.961719	0.4427	1.3990	0.237299	0.0220	0.0108658701
ss86341423		3	105.007297	0.4764	0.1095	0.740837	0.0062	0.0030760567
ss61506046	*rs41601871*	3	105.073559	0.8976	0.3220	0.570578	0.0175	0.0032268174
ss86339696		3	105.089143	0.5107	0.1646	0.685065	0.0075	0.0037611323
ss86294370		3	105.112142	0.4721	0.7716	0.380035	0.0164	0.0081793056
ss86292957		3	105.13406	0.1153	0.3621	0.547524	0.0173	0.0035210353

ss61503682	*rs41609364*	3	105.172518	0.4928	0.9987	0.317981	0.0186	0.0093012381
ss65274130	*rs43356342*	3	105.207405	0.9019	0.3979	0.528377	0.0200	0.0035339570
ss86325035		3	105.247195	0.5029	3.5108	0.061388	0.0344	0.0172220869
ss86293695		3	105.295282	0.5308	5.8213	0.016091	0.0443	0.0220661884
ss86339834		3	105.320777	0.5043	0.1430	0.705418	0.0070	0.0034967995
ss86294356		3	105.344661	0.3123	0.0520	0.819690	0.0045	0.0019529615
ss86320459		3	105.528885	0.7679	6.2367	0.012743	0.0530	0.0189016400
ss61549999	*rs41646786*	3	105.634769	0.1060	0.0032	0.954662	0.0017	0.0003300252
	rs29011003	3	105.754138	0.4964	0.7565	0.384724	0.0160	0.0079876109
ss63120044	*rs42121147*	3	105.778709	0.6268	3.7625	0.052817	0.0370	0.0172913651
ss63119954	*rs42120317*	3	105.840888	0.5050	0.6741	0.411904	0.0153	0.0076458491
ss86305258		3	105.903381	0.7163	0.0790	0.778740	0.0058	0.0023646551
ss86339469		3	105.95588	0.1870	3.8254	0.050882	0.0479	0.0145577981
ss63119532	*rs42119377*	3	106.063245	0.4807	0.0032	0.954668	0.0011	0.0005285537
ss86336932		3	106.156165	0.7013	2.3158	0.128521	0.0312	0.0130560822
ss61532072	*rs41632506*	3	106.189578	0.8438	0.3239	0.569433	0.0142	0.0037482714
ss86312847		3	106.222883	0.3159	5.0702	0.024652	0.0440	0.0189983053
ss86336627		3	106.244387	0.0795	15.4102	0.000095	0.1327	0.0194178936
ss117966928		3	106.312362	0.2185	2.8107	0.094088	0.0381	0.0130278607
ss86333617		3	106.334383	0.1132	0.1822	0.669663	0.0125	0.0025092387
ss86319278		3	106.371941	0.7650	1.9437	0.163714	0.0313	0.0112667714
ss86336712		3	106.487246	0.2063	4.5350	0.033559	0.0497	0.0162610451
ss86294992		3	106.551663	0.1132	1.2506	0.263823	0.0318	0.0063930566
ss86295530		3	106.625153	0.7514	1.0450	0.307011	0.0221	0.0082443662
ss86335969		3	106.743432	0.2170	4.3084	0.038292	0.0469	0.0159293910
ss86321584		3	106.79442	0.7600	9.0100	0.002781	0.0662	0.0241651628
ss61503675	*rs41609357*	3	106.834234	0.8675	0.2815	0.595893	0.0150	0.0034526219
ss64869292	*rs42965276*	3	106.864174	0.3095	0.6165	0.432614	0.0163	0.0069466423
ss61527524	*rs41624184*	3	106.885103	0.1003	5.4602	0.019737	0.0720	0.0129848089
ss61553467	*rs43712215*	3	106.936757	0.1590	4.2855	0.038807	0.0522	0.0139644982
ss61553464	*rs43712212*	3	106.980663	0.3789	2.6530	0.103806	0.0317	0.0149014255
ss86336488		3	107.055855	0.7049	10.5442	0.001221	0.0649	0.0270157089
ss64320652	*rs42581452*	3	107.08665	0.0688	0.3772	0.539316	0.0232	0.0029676042
ss64501902	*rs42676399*	3	107.229634	0.8596	0.0114	0.914997	0.0029	0.0007044831
ss64320764	*rs42581502*	3	107.276458	0.2307	1.3386	0.247671	0.0254	0.0090296725
ss86335977		3	107.311646	0.6891	1.0910	0.296604	0.0206	0.0088369372
ss61513552	*rs41613020*	3	107.391277	0.5330	0.0004	0.983636	0.0004	0.0001916015
ss61510155	*rs41601700*	3	107.479882	0.7715	0.0033	0.954506	0.0013	0.0004639185
ss61553469	*rs43712217*	3	107.534337	0.8130	0.0027	0.958208	0.0013	0.0003833911
ss117967149		3	107.561368	0.4878	14.2875	0.000170	0.0690	0.0344651730
ss117963677		3	107.600746	0.5380	4.3945	0.036416	0.0386	0.0192123871
ss86293760		3	107.658215	0.4004	5.2930	0.021707	0.0430	0.0206357214
	rs29021851	3	107.915922	0.2808	2.4701	0.116484	0.0332	0.0134099575
ss61553418	*rs43712167*	3	107.991574	0.0731	10.1359	0.001519	0.1102	0.0149274723
ss65273053	*rs43360567*	3	108.014651	0.8352	19.6468	0.000011	0.1050	0.0288996212
ss86312925		3	108.063435	0.4857	4.7047	0.030417	0.0398	0.0198778144
ss86314483		3	108.109915	0.0487	2.5729	0.109160	0.0651	0.0060307607

ss65272887	*rs43359402*	3	108.13139	0.6232	1.4766	0.224715	0.0236	0.0111006166
ss61522576	*rs41620747*	3	108.155741	0.4054	1.1412	0.285761	0.0209	0.0100792890
ss86319205		3	108.191284	0.1426	3.2673	0.071105	0.0468	0.0114302603
ss65276508	*rs43361717*	3	108.219434	0.1340	0.0163	0.898316	0.0035	0.0008219014
ss86317216		3	108.302133	0.6999	2.7630	0.096918	0.0332	0.0139616452
ss86339585		3	108.367589	0.6282	0.5002	0.479632	0.0133	0.0062247946
ss86317562		3	108.391617	0.3331	0.0142	0.905108	0.0024	0.0010472725
ss86276090		3	108.57623	0.5645	1.8550	0.173646	0.0254	0.0124722158
ss86335338		3	108.640566	0.8632	1.6867	0.194472	0.0345	0.0081391934
ss86336334		3	108.688867	0.3245	0.1262	0.722509	0.0070	0.0030482067
ss61478890	*rs41578964*	3	108.70942	0.4370	2.5867	0.108216	0.0300	0.0147620725
ss61531428	*rs41626090*	3	108.730668	0.6583	0.3660	0.545395	0.0116	0.0052383875
ss86292962		3	108.800552	0.2156	0.0439	0.834084	0.0048	0.0016092864
ss86340751		3	108.91686	0.7042	0.4523	0.501462	0.0142	0.0059100501
ss86297350		3	108.938493	0.3489	0.3976	0.528562	0.0122	0.0055335513
ss65277183	*rs43369263*	3	109.008938	0.8539	9.0944	0.002657	0.0778	0.0194276740
ss86288030		3	109.031817	0.4936	2.0486	0.152799	0.0266	0.0133204911
ss65277280	*rs43359754*	3	109.109781	0.5172	2.0463	0.153025	0.0266	0.0132880843
ss86330021		3	109.147488	0.1970	0.0294	0.863810	0.0040	0.0012612424
ss61553509	*rs43712955*	3	109.186954	0.4778	0.2980	0.585315	0.0102	0.0051014763
ss61553514	*rs43712960*	3	109.282381	0.8080	0.4082	0.523091	0.0146	0.0045273437
ss86332373		3	109.359836	0.0537	5.1697	0.023287	0.0950	0.0096578211
ss86325151		3	109.381695	0.9219	5.3967	0.020462	0.0797	0.0114725478
ss86300076		3	109.411155	0.8188	0.0397	0.842059	0.0047	0.0014017860
ss86311044		3	109.44111	0.8109	0.1955	0.658515	0.0108	0.0033235695
ss86282755		3	109.552327	0.7221	7.1167	0.007814	0.0555	0.0222880858
ss86287301		3	109.574402	0.1254	3.0208	0.082646	0.0486	0.0106532510
ss86336478		3	109.604478	0.8102	1.3207	0.250869	0.0272	0.0083510183
ss86299430		3	109.627103	0.6726	3.2080	0.073714	0.0362	0.0159505643
ss86335632		3	109.672822	0.7056	2.9876	0.084348	0.0350	0.0145312525
ss61489475	*rs41585098*	3	109.711409	0.3496	0.0866	0.768592	0.0056	0.0025613040
ss61489480	*rs41585103*	3	109.756157	0.7192	0.7296	0.393299	0.0174	0.0070299632
ss61489489	*rs41585112*	3	109.793953	0.2479	1.8078	0.179210	0.0277	0.0103406405
ss61565129		3	109.815794	0.2643	0.0131	0.908766	0.0024	0.0009258684
ss86287865		3	109.85467	0.1067	3.2304	0.072718	0.0532	0.0101486268
ss86335110		3	109.957942	0.5193	8.0130	0.004778	0.0500	0.0249438788
ss86293649		3	109.990012	0.9112	7.6358	0.005873	0.0893	0.0144609814
ss86301178		3	110.035546	0.0917	10.0085	0.001626	0.1009	0.0168094651
ss86304947		3	110.063	0.8159	0.4916	0.483433	0.0168	0.0050521671
ss86298411		3	110.135214	0.4119	0.0629	0.802077	0.0048	0.0023012717
ss117967071		3	110.25483	0.8302	2.0460	0.153052	0.0355	0.0100196271
ss117966982		3	110.307083	0.0946	2.0915	0.148567	0.0463	0.0079319589
ss86341010		3	110.398047	0.2758	1.9994	0.157804	0.0305	0.0121807859
ss86311321		3	110.443317	0.4699	4.8909	0.027322	0.0421	0.0209775580
	rs29027626	3	110.512103	0.3890	0.1630	0.686525	0.0079	0.0037411303
ss86334915		3	110.564587	0.1153	0.0580	0.809786	0.0072	0.0014646656
ss117962688		3	110.65717	0.1325	1.5099	0.219570	0.0339	0.0077861149

ss61472203	*rs41568843*	3	110.888506	0.2944	0.5129	0.474110	0.0145	0.0060205610
ss64624002	*rs42725809*	3	110.913178	0.1748	0.1710	0.679327	0.0097	0.0028082206
ss86307162		3	110.957324	0.1168	0.9195	0.337948	0.0284	0.0058574098
ss86300613		3	111.095457	0.6748	0.9995	0.317772	0.0199	0.0087486280
ss86339728		3	111.158842	0.4799	2.5170	0.113080	0.0293	0.0146264116
ss86336141		3	111.20069	0.7980	1.5637	0.211538	0.0296	0.0095287661
ss86338642		3	111.260249	0.5265	13.7195	0.000229	0.0695	0.0346374792
ss86285380		3	111.280727	0.1526	0.0463	0.829738	0.0056	0.0014524623
ss86274458		3	111.32115	0.3911	0.4947	0.482063	0.0135	0.0064458024
ss65277974	*rs43367746*	3	111.370609	0.6017	0.6047	0.437050	0.0149	0.0071455134
ss86341331		3	111.393083	0.1433	0.0827	0.773819	0.0076	0.0018714809
ss86339363		3	111.441093	0.5415	0.4054	0.524519	0.0121	0.0060306746
ss86339683		3	111.490509	0.3983	1.8809	0.170675	0.0260	0.0124387644
ss86339556		3	111.523499	0.6855	0.8490	0.357151	0.0188	0.0081116196
ss86289781		3	111.556304	0.4842	0.2349	0.628060	0.0090	0.0044723168
ss86339534		3	111.586713	0.8338	1.1632	0.281185	0.0273	0.0075598994
ss86284409		3	111.623091	0.5136	0.0194	0.889194	0.0026	0.0013121447
ss86294127		3	111.649584	0.5093	0.0885	0.766229	0.0056	0.0027850411
ss86341066		3	111.68251	0.5544	1.5807	0.209082	0.0235	0.0116295352
ss86321724		3	111.821915	0.1905	0.6792	0.410154	0.0203	0.0062500261
ss61510165	*rs41601710*	3	111.846609	0.3875	0.3786	0.538579	0.0119	0.0056642046
ss86283078		3	111.885832	0.1619	1.8477	0.174490	0.0342	0.0092737069
ss86274075		3	111.914286	0.2056	0.0694	0.792350	0.0062	0.0020092058
ss86341511		3	111.953077	0.5487	1.8028	0.179816	0.0260	0.0128584579
	rs29012205	3	111.990685	0.5050	0.7174	0.397285	0.0161	0.0080739195
ss86325587		3	112.031838	0.8152	0.1784	0.672848	0.0105	0.0031696863
ss86328371		3	112.116463	0.1254	1.6273	0.202506	0.0369	0.0080891709
ss86336714		3	112.164375	0.2564	0.5570	0.455707	0.0163	0.0062096813
ss86311817		3	112.196196	0.1827	1.3965	0.237715	0.0294	0.0087811172
ss61497199	*rs41594628*	3	112.234442	0.8130	1.0371	0.308854	0.0252	0.0076758571
ss86319983		3	112.37498	0.4262	1.1803	0.277676	0.0207	0.0101105526
ss86338359		3	112.474978	0.3395	4.7474	0.029677	0.0433	0.0194141593
ss86313741		3	112.502156	0.1110	1.4928	0.222192	0.0359	0.0070941662
ss86339303		3	112.546077	0.3274	4.3161	0.038119	0.0419	0.0184317400
ss86273855		3	112.651625	0.1734	1.6442	0.200173	0.0319	0.0091541508
ss117974830		3	112.697733	0.6655	0.2702	0.603335	0.0108	0.0047962186
ss65272799	*rs43358923*	3	112.751916	0.3660	14.6638	0.000140	0.0727	0.0337359311
ss65272826	*rs43359341*	3	112.789958	0.4112	8.8843	0.002977	0.0561	0.0271605565
ss64949062	*rs43042350*	3	112.885093	0.5852	1.3648	0.243106	0.0227	0.0110118232
ss86338002		3	112.90779	0.1404	0.3141	0.575349	0.0149	0.0036042890
ss65272537	*rs43357861*	3	112.93666	0.2779	0.2531	0.615039	0.0107	0.0042975673
ss65272369	*rs43356993*	3	113.00541	0.3231	1.9736	0.160514	0.0289	0.0126396994
ss86323855		3	113.039037	0.3238	1.9532	0.162684	0.0289	0.0126402314
ss64694364	*rs42791325*	3	113.107425	0.5487	0.0156	0.900595	0.0024	0.0011756958
ss86301567		3	113.205348	0.3961	0.0184	0.892275	0.0027	0.0012923583
ss86305291		3	113.267772	0.6110	0.7681	0.381093	0.0173	0.0082345937
ss86307229		3	113.335452	0.7715	0.1386	0.709782	0.0084	0.0029698554

ss61474747	*rs41572539*	3	113.410906	0.8503	0.0485	0.825826		0.0058	0.0014693711
ss117964405		3	113.52688	0.7600	4.6893	0.030689		0.0474	0.0172734304
ss46526662	*rs41257117*	3	113.580157	0.3266	0.0823	0.774287		0.0058	0.0025486706
ss86333762		3	113.62169	0.6426	2.5979	0.107460		0.0314	0.0144045937
ss86311605		3	113.643752	0.7414	0.9204	0.337697		0.0213	0.0081765419
ss61489549	*rs41585872*	3	113.66383	0.7722	2.9011	0.088965		0.0371	0.0130573276
ss86334526		3	113.705959	0.2056	2.3034	0.129544		0.0359	0.0117298824
ss61553629	*rs43741236*	3	113.775015	0.5602	0.1398	0.708625		0.0070	0.0034603000
ss65278707	*rs43364576*	3	113.846496	0.6454	5.3456	0.021066		0.0441	0.0201653162
ss86296954		3	113.96176	0.9384	0.3043	0.581401		0.0213	0.0024580638
ss61489551	*rs41585874*	3	114.003014	0.4126	7.2324	0.007331		0.0499	0.0241685562
ss65278593	*rs43363362*	3	114.161872	0.7414	0.7834	0.376424		0.0197	0.0075413819
ss117962701		3	114.187127	0.2815	4.4611	0.035030		0.0449	0.0181433071
ss86320124		3	114.211713	0.0723	1.0592	0.303762		0.0367	0.0049323111
ss61553631	*rs43714175*	3	114.24295	0.3216	1.0210	0.312624		0.0209	0.0091062509
ss86338814		3	114.269627	0.4513	0.0755	0.783634		0.0052	0.0025865925
ss65278809	*rs43365877*	3	114.293021	0.3216	1.0210	0.312624		0.0209	0.0091062509
ss61553633	*rs43714177*	3	114.37318	0.3446	0.3335	0.563812		0.0116	0.0052407609
ss65278861	*rs43365929*	3	114.405181	0.9355	0.6430	0.422883		0.0297	0.0035768486
ss86341457		3	114.426292	0.8116	0.5461	0.460149		0.0177	0.0054226968
ss86310513		3	114.463139	0.4219	6.3845	0.011733		0.0483	0.0235528225
ss61570550	*rs41668907*	3	114.496065	0.8309	0.8938	0.344765		0.0235	0.0066058904
ss65278996	*rs43367964*	3	114.516239	0.4470	0.1843	0.667840		0.0083	0.0040808399
ss86336016		3	114.536451	0.1991	1.0344	0.309482		0.0232	0.0074124031
ss86303933		3	114.566921	0.3352	4.2214	0.040291		0.0409	0.0182338453
ss86326553		3	114.593246	0.1927	1.9063	0.167821		0.0315	0.0098040904
ss86317604		3	114.660143	0.5638	0.0999	0.752059		0.0060	0.0029347704
ss86300695		3	114.765445	0.3159	10.8046	0.001063		0.0628	0.0271410549
ss86322451		3	114.794128	0.6232	5.6606	0.017619		0.0436	0.0204934857
ss86329705		3	114.86235	0.8288	0.0017	0.967443		0.0010	0.0002768698
ss117967096		3	114.946415	0.5824	0.3102	0.577725		0.0104	0.0050791314
ss86296274		3	114.969218	0.4248	0.1321	0.716345		0.0068	0.0033350092
ss61489565	*rs41585883*	3	114.992851	0.3861	5.6750	0.017476		0.0443	0.0210090838
ss86321658		3	115.019014	0.5415	1.6183	0.203750		0.0235	0.0116550960
ss86338527		3	115.073925	0.5530	0.4619	0.496952		0.0129	0.0063591028
ss86340671		3	115.118764	0.1648	5.1384	0.023708		0.0574	0.0157878238
ss86335655		3	115.220938	0.6010	0.5061	0.477056		0.0133	0.0063619808
ss86293365		3	115.359338	0.8152	6.0708	0.013984		0.0599	0.0180461199
ss86300075		3	115.421855	0.1332	12.2650	0.000491		0.0963	0.0222362750
ss86291011		3	115.479209	0.1340	12.5585	0.000421		0.0972	0.0225634355
ss117962598		3	115.527863	0.5093	0.2445	0.621137		0.0094	0.0046874250
ss86321732		3	115.624875	0.3331	5.0149	0.025445		0.0442	0.0196182037
ss86318343		3	115.720544	0.4420	14.2483	0.000174		0.0678	0.0334383630
ss86273550		3	115.766879	0.4148	4.1574	0.041829		0.0378	0.0183379705
ss86339664		3	115.805214	0.8976	6.9179	0.008722		0.0816	0.0150122807
ss61553656	*rs43714200*	3	115.866022	0.1812	5.8718	0.015639		0.0592	0.0175688287
ss61553658		3	115.891784	0.8274	5.5230	0.019047		0.0585	0.0167224487

ss61553661	*rs43714205*	3	115.981929	0.2042	4.4102	0.036084		0.0489	0.0158795436
ss86315006		3	116.025925	0.9148	0.1755	0.675363		0.0140	0.0021771514
	rs29014115	3	116.067952	0.9277	0.9596	0.327616		0.0350	0.0047033792
ss86336726		3	116.135485	0.9126	0.0203	0.886779		0.0047	0.0007436083
ss86314280		3	116.184916	0.9119	0.0027	0.958754		0.0017	0.0002712882
ss86335733		3	116.207138	0.0738	0.1274	0.721206		0.0127	0.0017326849
ss86339451		3	116.261513	0.8718	0.4554	0.499988		0.0192	0.0042825376
ss86284770		3	116.400889	0.8460	1.0700	0.301298		0.0262	0.0068309741
ss117967079		3	116.468364	0.1877	5.9786	0.014727		0.0593	0.0180868829
	rs29024239	3	116.505274	0.7507	9.7650	0.001853		0.0669	0.0250379212
ss86284663		3	116.604287	0.0996	2.3190	0.128257		0.0460	0.0082537665
ss86320117		3	116.645828	0.5064	4.4247	0.035782		0.0403	0.0201664595
ss86334898		3	116.676449	0.1612	0.0120	0.912817		0.0027	0.0007275838
ss86332384		3	116.711803	0.4155	9.5402	0.002090		0.0564	0.0274085599
ss117967076		3	116.738084	0.0630	2.6216	0.105873		0.0583	0.0068844450
ss117962661		3	116.770309	0.8954	0.6988	0.403481		0.0253	0.0047410799
ss61489592	*rs41585910*	3	116.855061	0.5938	1.1286	0.288441		0.0201	0.0096849273
ss65280068	*rs43367040*	3	116.876049	0.1569	9.0657	0.002699		0.0747	0.0197512360
ss86294484		3	116.905852	0.5709	0.8291	0.362845		0.0176	0.0086097752
ss65280157	*rs43367129*	3	117.056784	0.4398	0.1571	0.692006		0.0076	0.0037272994
ss86283735		3	117.169569	0.3890	0.9556	0.328642		0.0185	0.0088056910
ss86295332		3	117.191431	0.3890	0.8674	0.352009		0.0177	0.0084170159
ss61489594	*rs41585912*	3	117.250428	0.4613	0.0791	0.778670		0.0053	0.0026108500
ss86326978		3	117.275649	0.0781	0.0847	0.771087		0.0101	0.0014553080
ss86341121		3	117.355252	0.5544	0.8187	0.365887		0.0170	0.0083907185
ss65280466	*rs43363539*	3	117.450737	0.1261	23.0790	0.000002		0.1357	0.0299102400
ss65280454	*rs43362627*	3	117.475428	0.4198	10.3142	0.001381		0.0599	0.0291717497
ss86319608		3	117.534629	0.3345	16.0360	0.000069		0.0812	0.0361497735
ss65280599	*rs43364472*	3	117.610619	0.6282	0.0152	0.901987		0.0024	0.0011171974
ss65280715	*rs43365588*	3	117.705066	0.6282	0.0152	0.901987		0.0024	0.0011171974
ss46527116	*rs41255659*	3	117.827118	0.6583	0.3289	0.566497		0.0115	0.0051890206
ss65280870	*rs43367641*	3	117.888155	0.2307	13.2989	0.000285		0.0829	0.0294235027
ss65280921	*rs43367692*	3	117.91419	0.6805	1.0599	0.303600		0.0205	0.0089210611
ss86318666		3	117.950332	0.5308	2.6988	0.100877		0.0311	0.0155049802
ss86321064		3	118.050789	0.3789	0.0922	0.761523		0.0059	0.0027963532
ss46526163	*rs41255305*	3	118.0992	0.5079	13.4593	0.000262		0.0660	0.0329730628
ss86336293		3	118.126544	0.5494	1.5235	0.217504		0.0238	0.0118016402
ss86311018		3	118.263833	0.5244	0.4958	0.481602		0.0135	0.0067157201
ss86328996		3	118.326811	0.3138	0.4942	0.482296		0.0141	0.0060783768
ss61553700	*rs43715244*	3	118.374345	0.3281	5.5181	0.019099		0.0458	0.0202060495
ss61489601	*rs41585919*	3	118.410717	0.2192	0.3652	0.545823		0.0135	0.0046075932
ss65281734	*rs43364106*	3	118.47076	0.8553	2.3504	0.125703		0.0400	0.0099003568
ss61553716	*rs43715258*	3	118.539992	0.0881	0.2651	0.606782		0.0168	0.0026993043
ss86312251		3	118.565759	0.5688	1.3074	0.253257		0.0224	0.0110120123
ss86309675		3	118.587971	0.1003	0.0211	0.884440		0.0044	0.0007986006
ss86340807		3	118.631474	0.1956	7.1854	0.007524		0.0622	0.0195801896
ss86314027		3	118.693377	0.5387	0.0060	0.938378		0.0015	0.0007424776

ss61510190	*rs41602634*	3	118.766097	0.6497	0.7899	0.374429	0.0175	0.0079488910
ss86305047		3	118.803985	0.6476	7.6585	0.005801	0.0541	0.0246754198
ss117966977		3	118.829194	0.1139	0.2483	0.618462	0.0150	0.0030206993
ss61553723	*rs43715265*	3	118.894012	0.3811	0.0234	0.878573	0.0029	0.0013646684
ss117967112		3	118.929394	0.7529	1.2212	0.269498	0.0243	0.0090325532
ss64964309	*rs43056622*	3	118.955174	0.1125	0.1020	0.749494	0.0094	0.0018830125
ss61517010	*rs41621207*	3	118.985873	0.1934	0.3447	0.557347	0.0139	0.0043468509
ss64659401	*rs42752353*	3	119.008234	0.2256	6.2088	0.012943	0.0552	0.0192837860
ss86324200		3	119.029352	0.2256	6.2088	0.012943	0.0552	0.0192837860
ss86323738		3	119.111469	0.3897	1.8456	0.174732	0.0263	0.0125127483
ss117967120		3	119.178718	0.1984	0.2659	0.606226	0.0111	0.0035253888
ss86315534		3	119.210685	0.0738	3.5612	0.059559	0.0655	0.0089544584
ss86329002		3	119.24292	0.5387	0.0193	0.889421	0.0026	0.0012831313
ss86302396		3	119.268832	0.3102	0.2452	0.620651	0.0099	0.0042227132
ss86339650		3	119.341669	0.5236	0.0029	0.956766	0.0010	0.0005009469
ss86274663		3	119.417315	0.5867	0.6305	0.427432	0.0149	0.0072196839
ss86303451		3	119.498721	0.4076	4.9128	0.026982	0.0415	0.0200645467
ss86336295		3	119.535085	0.6834	2.6012	0.107238	0.0326	0.0141109615
ss86327538		3	119.590651	0.5072	1.7922	0.181093	0.0260	0.0130027422
ss86285653		3	119.623416	0.5480	7.5146	0.006277	0.0528	0.0261383841
ss61474574	*rs41571073*	3	119.966549	0.2880	0.9983	0.318059	0.0204	0.0083600318
ss86335019		3	120.001314	0.2622	0.0063	0.936888	0.0017	0.0006658800
ss86289003		3	120.037622	0.1354	1.1008	0.294465	0.0282	0.0066041096
	rs29022776	3	120.066679	0.6476	0.0495	0.824033	0.0044	0.0019897315
ss117967131		3	120.190103	0.4269	0.7616	0.383145	0.0171	0.0083503211
ss86284769		3	120.327633	0.8496	3.5246	0.060883	0.0487	0.0124535244
ss86274774		3	120.356182	0.8847	1.0218	0.312446	0.0290	0.0059159725
ss65282761	*rs43365932*	3	120.3909	0.1905	0.8348	0.361215	0.0221	0.0068283760
ss86306809		3	120.451743	0.9148	0.0748	0.784520	0.0093	0.0014487954
ss46526304	*rs41255443*	3	120.474868	0.9112	0.0512	0.821040	0.0076	0.0012245445
ss86291362		3	120.534054	0.8109	0.1736	0.677059	0.0104	0.0031751151
ss64644746	*rs42737927*	3	120.598358	0.7930	0.4686	0.493866	0.0165	0.0054247702
ss64644748	*rs42737929*	3	120.621469	0.1060	1.0304	0.310417	0.0305	0.0057851265
ss61516461	*rs41615869*	3	120.686237	0.7228	7.4305	0.006574	0.0558	0.0223573630
ss64666152	*rs42765400*	3	120.741059	0.1060	1.0304	0.310417	0.0305	0.0057851265
ss86340230		3	120.806576	0.1834	1.3423	0.247018	0.0280	0.0083906550
ss86330748		3	120.878778	0.3503	1.6784	0.195568	0.0265	0.0120523300
ss86288619		3	120.919373	0.2213	3.0326	0.082051	0.0376	0.0129456874
ss86308235		3	120.941211	0.7572	0.1383	0.710057	0.0081	0.0029676673
ss86340874		3	121.002313	0.2794	2.6892	0.101484	0.0339	0.0136456974
ss64532720	*rs42680613*	3	121.062122	0.5043	0.0168	0.896820	0.0025	0.0012688457
ss61506159	*rs41603378*	3	121.090386	0.7500	3.1050	0.078489	0.0382	0.0143160679
ss86314675		3	121.137678	0.0616	0.1838	0.668233	0.0158	0.0018224501
ss86286888		3	121.234376	0.2436	0.9447	0.331403	0.0207	0.0076316566
ss86321071		3	121.254561	0.7865	0.0001	0.991900	0.0002	0.0000755252
ss86292945		3	121.340161	0.3023	0.8643	0.352873	0.0187	0.0078985948
ss61531514	*rs41626970*	3	121.409617	0.0480	7.6054	0.005972	0.1173	0.0107153318

ss86290571		3	121.465722	0.1325	1.1478	0.284386	0.0285	0.0065523000
	rs29012399	3	121.492211	0.1010	5.7921	0.016358	0.0728	0.0132212251
ss86323700		3	121.531267	0.7027	4.2642	0.039294	0.0408	0.0170575093
ss65284052	*rs43371017*	3	121.581917	0.1426	1.4634	0.226799	0.0323	0.0078981332
ss86317172		3	121.603358	0.7350	0.0043	0.947835	0.0014	0.0005430213
ss86290542		3	121.635187	0.4828	0.0420	0.837625	0.0037	0.0018661443
ss86321812		3	121.66497	0.3582	1.7346	0.188255	0.0245	0.0112836861
ss65283964	*rs43370129*	3	121.703298	0.7457	0.2134	0.644235	0.0095	0.0036209504
ss86328283		3	121.731293	0.1991	2.2062	0.137907	0.0331	0.0105707328
ss61553769	*rs43715311*	3	121.75342	0.3797	0.8305	0.362440	0.0173	0.0081438871
ss86289202		3	121.786598	0.3653	9.7465	0.001871	0.0601	0.0278811701
ss86341338		3	121.828132	0.1719	1.0703	0.301244	0.0257	0.0073272362
ss65283867	*rs43370032*	3	121.885231	0.7815	0.9322	0.334616	0.0217	0.0074274836
ss65283847	*rs43369212*	3	121.932575	0.2837	0.0001	0.993077	0.0002	0.0000711541
ss61553764	*rs43715306*	3	121.98031	0.7421	0.0398	0.841917	0.0043	0.0016510071
ss61489633	*rs41586351*	3	122.002228	0.2256	0.3031	0.582150	0.0121	0.0042252866
ss86285244		3	122.081407	0.5308	0.1704	0.679901	0.0075	0.0037356687
ss65102032	*rs43186182*	3	122.211019	0.4799	0.2555	0.613360	0.0094	0.0047023434
ss86288414		3	122.236999	0.1612	0.0005	0.982559	0.0005	0.0001486427
ss64051705	*rs42449369*	3	122.266608	0.5122	5.4215	0.020176	0.0427	0.0213590951
ss86337548		3	122.299372	0.3510	2.3335	0.127073	0.0300	0.0136589207
	rs29017745	3	122.331174	0.1605	4.7734	0.029235	0.0535	0.0144157217
ss86339108		3	122.365575	0.1526	7.7440	0.005535	0.0723	0.0186971966
ss61466373	*rs41568128*	3	122.400511	0.3138	2.2288	0.135911	0.0303	0.0130548622
ss86295178		3	122.428916	0.3546	1.1388	0.286285	0.0204	0.0093285533
ss86320045		3	122.45822	0.7385	15.1022	0.000112	0.0821	0.0317118032
ss61553775	*rs43715317*	3	122.491842	0.2636	14.3326	0.000166	0.0798	0.0309655284
ss86323642		3	122.532346	0.3166	6.6781	0.009963	0.0519	0.0224556826
ss86341318		3	122.56311	0.4878	0.4400	0.507330	0.0127	0.0063549351
ss61553779	*rs43715321*	3	122.623225	0.3603	0.3213	0.571019	0.0112	0.0051564783
ss86333103		3	122.873732	0.5917	0.0466	0.829117	0.0042	0.0020264527
ss65284541	*rs43368607*	3	122.900218	0.0781	0.0435	0.834821	0.0074	0.0010645403
ss86313536		3	122.927563	0.1769	4.2612	0.039361	0.0486	0.0141658238
ss65284573	*rs43369539*	3	122.95139	0.6132	0.0013	0.970845	0.0007	0.0003380200
ss86338068		3	122.976844	0.3739	0.5949	0.440810	0.0153	0.0071831515
ss86326711		3	122.997056	0.0938	0.8914	0.345434	0.0309	0.0052507196
ss86316017		3	123.025476	0.1447	6.8039	0.009291	0.0692	0.0171173647
ss86338711		3	123.047148	0.1325	5.2248	0.022567	0.0623	0.0143341903
ss86308374		3	123.108882	0.2393	6.1756	0.013186	0.0538	0.0195928296
ss86310884		3	123.148963	0.3474	5.1195	0.023966	0.0439	0.0199181736
ss65284893	*rs43372258*	3	123.21701	0.4341	3.8121	0.051283	0.0358	0.0176064826
ss86274715		3	123.24371	0.6676	11.1488	0.000886	0.0630	0.0279607170
ss86288012		3	123.29049	0.2443	0.4781	0.489509	0.0141	0.0052216121
ss86288538		3	123.338117	0.9255	0.8173	0.366281	0.0320	0.0044075584
ss38323789	*rs29012691*	3	123.35877	0.3360	0.0000	0.995386	0.0001	0.0000499422
ss86304427		3	123.413256	0.8152	0.4930	0.482846	0.0157	0.0047455491
ss86334691		3	123.436542	0.1769	6.6743	0.009984	0.0611	0.0177979092

ss86298793		3	123.465868	0.5738	16.8140	0.000046	0.0742	0.0362892340
ss86310937		3	123.513581	0.4728	10.3665	0.001343	0.0575	0.0286603855
ss86278905		3	123.54634	0.0595	3.3524	0.067535	0.0697	0.0077971963
ss86310256		3	123.582134	0.6175	8.0200	0.004760	0.0553	0.0261286704
ss86290046		3	123.62182	0.7837	0.2250	0.635415	0.0104	0.0035155415
ss86309386		3	123.676503	0.0953	0.9382	0.333074	0.0308	0.0053019014
ss62461147	*rs41886442*	3	123.708545	0.8037	6.0160	0.014421	0.0564	0.0178089604
ss86296634		3	123.748949	0.7693	2.6998	0.100812	0.0348	0.0123579810
ss86292171		3	123.912292	0.1318	1.4000	0.237122	0.0328	0.0075179117
ss86290343		3	123.933982	0.3403	1.9840	0.159411	0.0267	0.0120017678
ss86295003		3	123.955482	0.4628	0.2652	0.606730	0.0096	0.0047524808
ss86326692		3	123.988485	0.6920	3.6796	0.055492	0.0376	0.0160311218
	rs29024307	3	124.081193	0.5989	0.2941	0.587772	0.0097	0.0046825111
ss86321290		3	124.120588	0.4119	0.0000	0.994875	0.0001	0.0000560311
ss61489652		3	124.168778	0.7973	0.1566	0.692386	0.0088	0.0028574210
ss86305948		3	124.197624	0.7400	1.5631	0.211626	0.0262	0.0100828251
ss86291696		3	124.259023	0.2063	4.6881	0.030712	0.0500	0.0163795121
ss65286868	*rs43370727*	3	124.292202	0.2830	2.0039	0.157342	0.0299	0.0121339935
ss46526434	*rs41256898*	3	124.37964	0.2364	1.6399	0.200764	0.0278	0.0100234529
ss86340965		3	124.409438	0.3410	0.7188	0.396826	0.0162	0.0072644681
ss86300094		3	124.441054	0.5079	0.0001	0.993966	0.0001	0.0000688046
ss86336585		3	124.479926	0.1891	0.7976	0.372113	0.0210	0.0064389826
ss86321970		3	124.509943	0.1297	0.7441	0.388654	0.0237	0.0053379811
ss65287213	*rs43373567*	3	124.532095	0.1325	0.8673	0.352033	0.0255	0.0058718665
ss86333456		3	124.554975	0.2980	1.2871	0.256966	0.0223	0.0093394858
ss86323705		3	124.586031	0.3037	0.1780	0.673260	0.0085	0.0035841875
ss65287459	*rs43369920*	3	124.623722	0.0587	0.1342	0.714182	0.0140	0.0015487716
ss86336346		3	124.686895	0.8883	0.2126	0.644916	0.0137	0.0027142531
ss61553854	*rs41648102*	3	124.771548	0.8095	0.0020	0.964224	0.0010	0.0003195727
ss61510203	*rs41602647*	3	124.796281	0.7536	0.6757	0.411368	0.0175	0.0064986684
ss61553857	*rs41648105*	3	124.821625	0.5444	0.0714	0.789364	0.0048	0.0023774394
ss86339600		3	125.024838	0.8209	0.4712	0.492677	0.0166	0.0048926148
ss86332068		3	125.045825	0.7815	0.5730	0.449319	0.0167	0.0057081342
ss86305967		3	125.076515	0.8417	1.1965	0.274397	0.0274	0.0073088989
ss86320929		3	125.114116	0.7615	0.1325	0.715983	0.0078	0.0028256472
ss86323465		3	125.139134	0.1712	0.6086	0.435585	0.0191	0.0054119150
ss65287861	*rs43371819*	3	125.160405	0.1741	1.1967	0.274357	0.0264	0.0075825843
ss61489670	*rs41586388*	3	125.182251	0.8625	2.9277	0.087517	0.0471	0.0111845555
ss86306297		3	125.23158	0.1648	1.7081	0.191669	0.0321	0.0088476360
ss64304932	*rs42574137*	3	125.253337	0.1476	2.1985	0.138600	0.0385	0.0096934759
ss86341000		3	125.317891	0.6934	0.0003	0.986403	0.0003	0.0001451000
ss86296140		3	125.368229	0.3044	0.0673	0.795407	0.0052	0.0022048331
ss86302035		3	125.421304	0.6999	0.0097	0.921769	0.0020	0.0008321010
ss61519113	*rs41611166*	3	125.486288	0.9377	0.2942	0.587741	0.0211	0.0024634623
ss86327307		3	125.565151	0.5595	1.6335	0.201642	0.0241	0.0118992800
ss61553868	*rs41648116*	3	125.590407	0.7629	0.4267	0.513835	0.0145	0.0052370746
ss86275112		3	125.637877	0.6819	2.8930	0.089413	0.0360	0.0156060804

ss86339247		3	125.669302	0.5179	6.0731	0.013966	0.0438	0.0218811641
ss86322017		3	125.707822	0.5043	1.1513	0.283647	0.0200	0.0099852975
ss86335967		3	125.770792	0.6605	2.5992	0.107371	0.0315	0.0141145863
ss86340144		3	125.802628	0.4556	1.8863	0.170059	0.0245	0.0121656844
ss86325094		3	125.831817	0.7801	10.2504	0.001429	0.0711	0.0243940827
ss86295607		3	125.868208	0.3059	0.5692	0.450834	0.0157	0.0066817157
ss86284877		3	125.924301	0.9076	0.1663	0.683552	0.0130	0.0021737664
ss86306125		3	125.9769	0.7980	0.6124	0.434138	0.0176	0.0056842131
ss86273762		3	125.998161	0.6289	0.0543	0.815775	0.0045	0.0020932236
ss86330854		3	126.022868	0.4284	0.0460	0.830309	0.0040	0.0019716124
ss65285699	*rs43371663*	3	126.049084	0.2034	1.3408	0.247297	0.0266	0.0086238976
	rs29021928	3	126.097657	0.1096	0.0121	0.912536	0.0032	0.0006332727
ss86338072		3	126.164319	0.3668	3.2593	0.071452	0.0352	0.0163378052
ss86322674		3	126.213166	0.2557	0.3787	0.538520	0.0132	0.0050319174
ss86334523		3	126.248805	0.4105	1.1066	0.293183	0.0198	0.0095770591
ss86274454		3	126.273096	0.1562	2.0341	0.154252	0.0365	0.0096283863
ss86339168		3	126.335944	0.2586	0.6177	0.432164	0.0168	0.0064282666
ss65285645	*rs43370810*	3	126.357723	0.4054	4.7277	0.030017	0.0404	0.0194920463
ss64801186	*rs42898108*	3	126.499924	0.2944	1.9309	0.165109	0.0285	0.0118576753
ss86287897		3	126.607963	0.2486	0.0175	0.894780	0.0028	0.0010508383
ss86305683		3	126.729607	0.2872	1.5476	0.213903	0.0255	0.0104272487
ss86314444		3	126.751522	0.2851	1.0634	0.302802	0.0210	0.0085665380
ss86298814		3	126.774627	0.5208	0.6241	0.429780	0.0151	0.0075342406
ss86332598		3	126.79977	0.5795	0.1135	0.736255	0.0063	0.0030463165
ss86338978		3	126.822	0.1726	0.4840	0.486836	0.0171	0.0048960469
ss86305231		3	126.846089	0.2693	0.0046	0.945999	0.0014	0.0005523258
ss86340712		3	126.946316	0.4663	0.0008	0.977719	0.0005	0.0002638578
ss61469400	*rs41569507*	3	126.974934	0.2249	6.4517	0.011301	0.0562	0.0195950707
ss86322725		3	127.330958	0.4004	2.1915	0.139226	0.0285	0.0136799363
ss86297522		3	127.354774	0.2457	1.5845	0.208538	0.0282	0.0104442156
	rs29018845	3	127.404886	0.8546	0.5196	0.471261	0.0188	0.0046776467
ss65421324	*rs43505889*	3	127.435729	0.6418	4.6334	0.031699	0.0416	0.0191090534
ss86305177		3	127.465651	0.2966	0.7223	0.395696	0.0170	0.0070893956
ss86274091		3	127.49055	0.1999	0.2481	0.618590	0.0116	0.0037051364
ss61485804	*rs41586006*	3	127.520676	0.6676	1.5281	0.216808	0.0241	0.0107139579
ss86287507		3	127.574695	0.1160	0.2027	0.652676	0.0128	0.0026244083
ss86283446		3	127.61084	0.2615	3.7048	0.054665	0.0405	0.0156466333
ss38325947	*rs29011651*	3	127.631253	0.4491	1.3960	0.237794	0.0223	0.0110224136
ss86321332		3	127.658488	0.4506	1.2368	0.266475	0.0210	0.0103766574
ss86301354		3	127.719187	0.8503	0.0487	0.825452	0.0057	0.0014389975
ss86289217		3	127.769202	0.4735	0.0000	0.994811	0.0001	0.0000583963
ss86336875		3	127.817683	0.2350	2.8359	0.092626	0.0358	0.0128529566
ss86320262		3	127.849491	0.5795	1.4298	0.232212	0.0224	0.0109093193
ss86327764		3	127.874808	0.0974	3.2290	0.072780	0.0569	0.0099979549
ss86337572		3	127.908629	0.1182	0.8663	0.352294	0.0259	0.0054083120
ss86297879		4	0.0508	0.2980	0.0022	0.963028	0.0009	0.0003792670
ss86339480		4	0.114069	0.7271	0.0017	0.966917	0.0009	0.0003377387

	rs29002127	4	0.298878	0.8016	1.1510	0.283707		0.0245	0.0078026028
	rs29014886	4	0.499776	0.2887	0.1468	0.701744		0.0080	0.0032727480
	rs29024432	4	0.567587	0.3926	3.3068	0.069423		0.0343	0.0163560780
ss86301042		4	0.805718	0.4126	1.1685	0.280093		0.0204	0.0098693928
ss86319159		4	0.98435	0.6089	0.7708	0.380287		0.0170	0.0080903627
ss61525910	*rs41623211*	4	1.212237	0.3682	0.1587	0.690523		0.0079	0.0036578205
ss86313907		4	1.245618	0.3309	0.0458	0.830658		0.0042	0.0018732834
ss86332412		4	1.283427	0.6433	1.1591	0.282018		0.0204	0.0093418247
ss86293662		4	1.303984	0.5738	0.1349	0.713555		0.0068	0.0033150413
ss61496675	*rs41591327*	4	1.344228	0.0738	2.4807	0.115706		0.0558	0.0076314442
ss86323405		4	1.386888	0.6526	0.0312	0.859860		0.0035	0.0015802030
ss86286353		4	1.463657	0.2758	0.7857	0.375696		0.0182	0.0072563731
ss61469667	*rs41572164*	4	1.676023	0.5494	4.6897	0.030684		0.0387	0.0191738860
ss61564989	*rs41657654*	4	1.7599	0.8804	0.7748	0.379027		0.0253	0.0053331987
ss86341249		4	1.815715	0.2973	3.0147	0.082956		0.0344	0.0143890420
ss61471743	*rs41572492*	4	1.895804	0.4076	0.3575	0.550101		0.0115	0.0055324025
ss61471746	*rs41572495*	4	1.940369	0.5523	0.0031	0.955513		0.0011	0.0005205546
ss117967514		4	2.075446	0.3080	0.9894	0.320229		0.0206	0.0087910905
ss86286818		4	2.109103	0.4857	0.0794	0.778229		0.0054	0.0026990661
ss86274318		4	2.247073	0.4040	0.0906	0.763494		0.0059	0.0028197262
ss61474779	*rs41572571*	4	2.389224	0.2937	2.3503	0.125711		0.0321	0.0133354048
ss61521675	*rs41620977*	4	2.416363	0.2299	0.7193	0.396666		0.0183	0.0064805420
ss61506965	*rs41601373*	4	2.43643	0.8481	4.5056	0.034137		0.0530	0.0136591230
ss86326541		4	2.607776	0.3589	0.8835	0.347582		0.0184	0.0084744349
ss86300155		4	2.751606	0.1440	1.8855	0.170151		0.0356	0.0087827499
ss61498971	*rs43708070*	4	2.862401	0.2357	0.0024	0.960668		0.0011	0.0003857639
ss86280215		4	2.995921	0.2464	0.7402	0.389887		0.0195	0.0072446798
ss86288382		4	3.345194	0.1533	1.3734	0.241638		0.0302	0.0078452096
ss86302286		4	3.519031	0.8460	0.0204	0.886333		0.0037	0.0009528878
ss86284473		4	3.585213	0.0458	0.3759	0.540000		0.0267	0.0023390795
ss86337916		4	3.765511	0.5745	1.6249	0.202839		0.0241	0.0117911669
ss61495347	*rs41595923*	4	3.848355	0.4749	0.0173	0.895289		0.0025	0.0012403682
ss117967197		4	3.88505	0.6390	0.2252	0.635247		0.0091	0.0041810767
ss61500731		4	3.923958	0.2113	1.5029	0.220641		0.0282	0.0094052996
ss86287697		4	4.053169	0.5809	0.2251	0.635305		0.0088	0.0042705849
ss86339470		4	4.156718	0.2350	1.1445	0.285082		0.0241	0.0086695246
	rs29011086	4	4.247358	0.1662	0.0883	0.766415		0.0076	0.0020966539
ss61520604	*rs41615627*	4	4.294713	0.5423	1.5091	0.219692		0.0230	0.0114289617
ss86311214		4	4.387117	0.6146	0.2458	0.620180		0.0094	0.0044714219
ss117963746		4	4.407255	0.2042	0.4239	0.515222		0.0152	0.0049363695
ss86340317		4	4.485874	0.5917	0.4323	0.511097		0.0123	0.0059372101
ss86300158		4	4.608741	0.3116	0.0336	0.854683		0.0037	0.0016086109
ss61569335	*rs41666419*	4	4.664794	0.1117	5.9852	0.014673		0.0686	0.0136282426
ss86335060		4	4.694543	0.4491	0.0832	0.773148		0.0053	0.0026207204
ss61569338	*rs41666422*	4	4.746953	0.3911	0.3735	0.541325		0.0118	0.0056414097
ss86327720		4	4.800748	0.0781	0.3049	0.581018		0.0196	0.0028160448
ss86288580		4	4.827168	0.4520	0.5324	0.465832		0.0136	0.0067355846

ss86322857		4	4.866434	0.1762	0.8239	0.364364		0.0224	0.0065167347
ss86318934		4	4.889228	0.4943	2.6910	0.101366		0.0313	0.0156472117
ss86305818		4	4.939439	0.8732	2.3565	0.125212		0.0436	0.0096615601
ss86336719		4	5.029135	0.2622	0.0001	0.994036		0.0002	0.0000601821
ss86326755		4	5.051211	0.5487	1.2910	0.256264		0.0201	0.0099751239
ss86316059		4	5.085785	0.7865	0.1443	0.704145		0.0085	0.0028504918
ss86321231		4	5.128977	0.0766	0.3442	0.557614		0.0207	0.0029356383
ss86338237		4	5.158117	0.3037	0.0921	0.761602		0.0060	0.0025472365
ss86287834		4	5.262747	0.6024	0.9131	0.339617		0.0178	0.0085421143
ss86300767		4	5.285519	0.1146	0.7221	0.395749		0.0247	0.0050043511
ss86279317		4	5.31294	0.4176	0.6338	0.426253		0.0150	0.0072776672
ss86316516		4	5.354105	0.1655	2.6588	0.103431		0.0402	0.0110907413
ss117974056		4	5.44627	0.7980	0.0552	0.814295		0.0056	0.0017909010
ss86291020		4	5.484853	0.1268	2.3808	0.123292		0.0427	0.0094555929
ss86339299		4	5.532641	0.2872	2.8412	0.092323		0.0331	0.0135645983
ss86284308		4	5.5724	0.4656	0.7332	0.392131		0.0156	0.0077578730
ss61524034	*rs41624981*	4	5.630366	0.9398	0.0698	0.791736		0.0101	0.0011476105
ss117967484		4	5.657545	0.7615	3.4617	0.063228		0.0405	0.0147290618
ss86318206		4	5.748127	0.0408	0.0543	0.815839		0.0103	0.0008049314
	rs29016879	4	5.821477	0.1168	0.1533	0.695547		0.0110	0.0022626709
ss86292169		4	6.121101	0.6433	3.3960	0.065780		0.0335	0.0153895338
ss86335001		4	6.263127	0.1067	4.1871	0.041109		0.0622	0.0118642700
ss86297359		4	6.44101	0.7980	0.0470	0.828356		0.0049	0.0015931723
ss86313035		4	6.631915	0.5000	0.8768	0.349413		0.0168	0.0083973894
ss64918503	*rs43013883*	4	6.705772	0.4312	0.0576	0.810448		0.0045	0.0022173327
ss86324343		4	6.751872	0.6605	0.2255	0.635037		0.0093	0.0041691858
ss61471448	*rs41570110*	4	6.803249	0.6504	0.2693	0.603950		0.0101	0.0046153341
ss86276798		4	6.900817	0.6132	2.6965	0.101020		0.0315	0.0149355775
ss61555126	*rs41651662*	4	6.925524	0.1218	0.7979	0.372042		0.0252	0.0053890725
ss65289041	*rs43376796*	4	6.951943	0.6426	2.0110	0.156607		0.0271	0.0124627910
ss65288973	*rs43375928*	4	6.9771	0.4842	3.6221	0.057429		0.0378	0.0188636291
ss86320358		4	7.012541	0.2672	0.0027	0.958866		0.0011	0.0004190268
ss86312780		4	7.038463	0.5845	1.3137	0.252118		0.0218	0.0106051489
ss86331964		4	7.07915	0.8410	3.3495	0.067655		0.0457	0.0122228187
ss86339320		4	7.137987	0.5150	4.2886	0.038738		0.0376	0.0188007997
ss61512530	*rs41613734*	4	7.161977	0.3739	2.8387	0.092470		0.0315	0.0147553775
ss86300745		4	7.169247	0.9004	5.1399	0.023689		0.0668	0.0119762590
ss61520629	*rs41616552*	4	7.212692	0.3052	0.6291	0.427950		0.0159	0.0067636133
ss117967295		4	7.261104	0.2844	1.6844	0.194775		0.0268	0.0109081142
ss86339727		4	7.33071	0.6039	1.0381	0.308608		0.0196	0.0093635821
ss86292832		4	7.445382	0.3302	2.2948	0.130261		0.0299	0.0132441636
ss86323311		4	7.485403	0.8324	0.2060	0.650037		0.0113	0.0031409289
	rs29010398	4	7.535022	0.4448	3.5559	0.059749		0.0353	0.0174414077
ss61474430	*rs41570133*	4	7.581522	0.6662	0.6177	0.432173		0.0156	0.0069514351
ss63887985	*rs41810589*	4	7.604745	0.6784	0.0300	0.862463		0.0034	0.0015009450
ss86319182		4	7.696839	0.2758	2.2068	0.137857		0.0312	0.0124663365
ss61520959	*rs41618978*	4	7.717	0.3976	9.8055	0.001813		0.0599	0.0286765052

ss61520974	*rs41618993*	4	7.752917	0.1777	0.8696	0.351378		0.0225	0.0065649205
	rs29026788	4	7.788554	0.4040	5.5540	0.018715		0.0448	0.0215867379
ss86291905		4	8.054382	0.4477	11.6879	0.000666		0.0638	0.0315543860
ss86308135		4	8.259958	0.1562	4.1300	0.042508		0.0506	0.0133288783
ss61476725	*rs41578277*	4	8.400217	0.5064	1.0744	0.300316		0.0190	0.0095220455
ss61526958	*rs41627227*	4	8.455559	0.4792	0.1322	0.716322		0.0068	0.0033699854
ss86292770		4	8.493253	0.7865	0.5862	0.444153		0.0170	0.0056924397
ss86341356		4	8.5408	0.8252	0.1138	0.735931		0.0082	0.0023709117
ss64968158	*rs43062212*	4	8.628038	0.8696	0.6411	0.423578		0.0217	0.0049302513
ss64968043	*rs43061097*	4	8.655767	0.5365	3.8929	0.048887		0.0359	0.0178380455
ss86299065		4	8.682598	0.2142	1.2129	0.271135		0.0244	0.0082204599
ss86330857		4	8.707036	0.4592	0.1471	0.701400		0.0069	0.0034512384
ss61529376	*rs41624504*	4	8.744786	0.6211	3.9198	0.048115		0.0383	0.0180212164
ss86277940		4	8.776067	0.5473	2.0739	0.150287		0.0257	0.0127415583
ss86318680		4	8.803302	0.5487	1.7948	0.180785		0.0239	0.0118233809
ss86309900		4	8.840375	0.3202	3.5794	0.058916		0.0369	0.0160589306
ss117967328		4	8.897944	0.8610	0.8667	0.352206		0.0251	0.0060176289
ss86299176		4	8.951647	0.0487	0.2860	0.592966		0.0224	0.0020726131
ss86301484		4	9.006522	0.1261	0.9399	0.332635		0.0264	0.0058257862
ss62359025	*rs41848383*	4	9.034967	0.3052	0.3407	0.559615		0.0117	0.0049643852
ss86288324		4	9.059626	0.2944	0.0335	0.854923		0.0037	0.0015272148
ss86296038		4	9.236495	0.2500	0.5358	0.464445		0.0156	0.0058417643
ss64423028	*rs42632374*	4	9.2696	0.4957	7.9247	0.005014		0.0514	0.0257157298
ss65092627	*rs43183624*	4	9.294161	0.3718	2.2781	0.131667		0.0282	0.0131651140
ss64982456	*rs43075694*	4	9.321256	0.5423	6.3054	0.012263		0.0462	0.0229239881
ss64670369	*rs42772137*	4	9.36328	0.3474	2.8286	0.093049		0.0327	0.0148094797
ss86310931		4	9.430204	0.3675	0.0192	0.889901		0.0026	0.0012304783
	rs29012059	4	9.51286	0.3553	0.0515	0.820468		0.0043	0.0019812524
ss61568080	*rs41661386*	4	9.544381	0.8524	0.4840	0.486853		0.0181	0.0045562956
ss61554009	*rs41649756*	4	9.595151	0.4019	1.0468	0.306595		0.0192	0.0092394491
ss65289543	*rs43372200*	4	9.640761	0.6461	0.5940	0.441123		0.0153	0.0069757190
ss61490456	*rs41587962*	4	9.677923	0.8138	2.1908	0.139291		0.0354	0.0107210304
ss86309976		4	9.715963	0.3847	1.6801	0.195340		0.0246	0.0116358500
ss65289283	*rs43379437*	4	9.79943	0.2973	1.9790	0.159944		0.0283	0.0118353035
ss65289426	*rs43371283*	4	9.94847	0.7178	0.9101	0.340432		0.0196	0.0079379689
ss86338277		4	10.009287	0.4362	0.0274	0.868543		0.0031	0.0015332736
ss65289502	*rs43372159*	4	10.06206	0.2314	1.2516	0.263627		0.0249	0.0088512892
ss86309584		4	10.093466	0.5666	10.3446	0.001359		0.0607	0.0298198406
ss86339623		4	10.139426	0.4069	4.3281	0.037854		0.0380	0.0183358994
ss86336919		4	10.228074	0.2092	0.1024	0.749073		0.0070	0.0023141833
ss86305567		4	10.258408	0.4828	0.0033	0.954282		0.0010	0.0005218541
ss61564190	*rs41659364*	4	10.325003	0.7507	0.1214	0.727677		0.0075	0.0027887295
ss86309172		4	10.359086	0.8868	6.6891	0.009902		0.0760	0.0152551055
ss38322754	*rs29013854*	4	10.391308	0.6468	1.9017	0.168328		0.0266	0.0121435086
ss117967257		4	10.426563	0.5430	5.5345	0.018923		0.0430	0.0213413519
ss117967503		4	10.541199	0.1447	0.1849	0.667335		0.0113	0.0028033686
ss38324944	*rs29010437*	4	10.564903	0.1440	0.3653	0.545773		0.0159	0.0039286202

ss86294905		4	10.640831	0.6784	1.6366	0.201218	0.0252	0.0110034243
ss61472581	*rs41571604*	4	10.665698	0.2307	4.5192	0.033868	0.0457	0.0162241570
ss61472574	*rs41571597*	4	10.728416	0.9176	0.5135	0.473888	0.0246	0.0037197984
ss86279503		4	10.801271	0.6905	0.4794	0.488908	0.0137	0.0058446077
ss61516666	*rs41617967*	4	10.928648	0.0960	3.0219	0.082591	0.0554	0.0096103541
ss63798816	*rs42358349*	4	11.0107	0.4069	15.5587	0.000088	0.0731	0.0352616420
ss64848165	*rs42940192*	4	11.050078	0.4370	0.0835	0.772735	0.0052	0.0025372170
ss86298889		4	11.090689	0.1834	0.0031	0.955459	0.0013	0.0003974323
ss65289915	*rs43375268*	4	11.116698	0.5193	0.2423	0.622694	0.0089	0.0044493832
ss61489805	*rs41586923*	4	11.139266	0.2923	0.7120	0.399073	0.0169	0.0069715014
ss61519465	*rs41614715*	4	11.166002	0.2479	1.8509	0.174114	0.0290	0.0108212328
ss86290473		4	11.242405	0.6784	0.0599	0.806706	0.0049	0.0021373027
ss86332108		4	11.28587	0.7242	0.0588	0.808496	0.0050	0.0019901215
ss64907960	*rs42999837*	4	11.311538	0.8302	0.3492	0.554757	0.0142	0.0039924368
ss64858711	*rs42953746*	4	11.3694	0.4936	0.0085	0.926434	0.0017	0.0008410201
ss64889664	*rs42982373*	4	11.396664	0.9047	2.9869	0.084382	0.0538	0.0092725153
ss61514485	*rs41613051*	4	11.424495	0.3546	5.0778	0.024544	0.0424	0.0194287982
ss86280630		4	11.499841	0.3453	1.9515	0.162872	0.0267	0.0120873127
ss86304447		4	11.57829	0.2622	0.0944	0.758734	0.0063	0.0024473827
ss86337056		4	11.61895	0.5695	2.7257	0.099198	0.0303	0.0148394317
ss61531019	*rs41631082*	4	11.649677	0.9420	0.4543	0.500539	0.0266	0.0029092065
ss117967516		4	11.687105	0.7521	4.0977	0.043323	0.0420	0.0156604804
ss61485771	*rs41585974*	4	11.725154	0.0903	0.0176	0.894369	0.0042	0.0006861925
ss86276503		4	11.747179	0.5867	0.4839	0.486895	0.0130	0.0063115514
ss86323726		4	11.777619	0.0917	0.0832	0.773133	0.0090	0.0015030042
ss61545775	*rs41638431*	4	11.830564	0.3130	3.0972	0.078868	0.0342	0.0147155411
ss61545777	*rs41638433*	4	11.891499	0.3904	1.1014	0.294334	0.0195	0.0092794746
	rs29011076	4	11.913822	0.5996	0.6721	0.412595	0.0155	0.0074624526
ss65290059	*rs43375611*	4	11.954034	0.2679	0.3189	0.572480	0.0114	0.0044753619
	rs29024866	4	12.016245	0.8474	8.6455	0.003387	0.0762	0.0197158126
ss86324957		4	12.043676	0.3080	1.6425	0.200410	0.0249	0.0106014007
ss61503752	*rs41599744*	4	12.136819	0.8582	8.5780	0.003514	0.0748	0.0182203930
ss61494572	*rs41588062*	4	12.205208	0.8589	0.9560	0.328526	0.0250	0.0060576980
ss86329906		4	12.23421	0.1519	0.9155	0.338979	0.0242	0.0062267337
ss61554132	*rs41650679*	4	12.274536	0.1812	8.9523	0.002869	0.0719	0.0213326108
ss61477906	*rs41580726*	4	12.3423	0.2278	2.8265	0.093167	0.0362	0.0127434203
ss86308109		4	12.54132	0.0473	10.1698	0.001492	0.1361	0.0122634766
ss61554218		4	12.63443	0.0917	0.7651	0.382047	0.0279	0.0046480500
ss86286249		4	12.749443	0.9563	0.4733	0.491697	0.0311	0.0025972427
ss86278943		4	12.933828	0.7034	0.3241	0.569316	0.0113	0.0047110810
ss86324041		4	12.967887	0.7500	0.4324	0.511039	0.0134	0.0050394435
ss86292224		4	12.994343	0.2486	0.3833	0.536026	0.0127	0.0047438724
ss117967362		4	13.038424	0.3460	1.6581	0.198294	0.0248	0.0112278696
	rs29019276	4	13.063174	0.2915	1.1128	0.291845	0.0211	0.0087249235
ss61512482	*rs41612688*	4	13.115847	0.7070	0.6274	0.428595	0.0158	0.0065318291
ss86323303		4	13.142857	0.3524	0.4548	0.500292	0.0131	0.0059705545
ss117967481		4	13.174294	0.3474	1.7952	0.180735	0.0259	0.0117652658

ss86306822		4	13.326417	0.6748	1.4783	0.224454	0.0227	0.0099487738
ss61465414		4	13.357443	0.2679	0.0796	0.777985	0.0058	0.0022868379
ss86298630		4	13.433821	0.9019	0.2739	0.600924	0.0167	0.0029610084
ss61470344	*rs41569333*	4	13.457585	0.0989	0.7062	0.401000	0.0263	0.0046918741
ss86296901		4	13.520319	0.1920	0.3079	0.579125	0.0126	0.0039181011
ss86332275		4	13.689278	0.4162	2.0464	0.153011	0.0267	0.0129787934
ss86290678		4	13.757462	0.3682	0.5878	0.443519	0.0147	0.0068234598
ss86337077		4	13.791726	0.0888	0.0046	0.946132	0.0021	0.0003404525
ss86340923		4	13.83489	0.3102	0.4869	0.485545	0.0139	0.0059347904
ss86294910		4	13.894911	0.3474	1.2527	0.263429	0.0218	0.0098801389
ss61518116	*rs41611285*	4	13.926497	0.2930	1.7063	0.191897	0.0264	0.0109520049
ss86281879		4	13.952924	0.9212	0.2915	0.589402	0.0175	0.0025362967
ss86336999		4	13.981533	0.5236	0.5217	0.470377	0.0131	0.0065473992
ss61517653	*rs41617117*	4	14.006281	0.7063	1.8082	0.179157	0.0260	0.0107823724
	rs29015213	4	14.240717	0.7808	0.0786	0.779276	0.0061	0.0020822309
ss61531144	*rs41632104*	4	14.278865	0.8195	2.8456	0.092071	0.0385	0.0113965072
ss86319884		4	14.343312	0.1841	1.3121	0.252405	0.0269	0.0080884801
	rs29013953	4	14.396347	0.1762	2.2807	0.131446	0.0352	0.0102289857
ss61554961	*rs41649300*	4	14.479518	0.7020	7.8053	0.005353	0.0562	0.0235083238
ss86305087		4	14.537722	0.4534	6.5935	0.010443	0.0474	0.0234942682
ss61554349	*rs41651995*	4	14.620478	0.7507	2.1426	0.143715	0.0294	0.0110068695
ss86337938		4	14.644971	0.5559	4.2936	0.038625	0.0378	0.0186417627
ss86341166		4	14.721389	0.5222	2.1014	0.147614	0.0263	0.0131212798
ss61491617		4	14.936122	0.5107	1.2168	0.270378	0.0204	0.0102052581
ss61491618	*rs41589005*	4	14.965621	0.7020	2.8617	0.091159	0.0329	0.0137746170
ss86299434		4	15.021946	0.8718	0.0375	0.846458	0.0052	0.0011672587
ss61514885	*rs41617140*	4	15.048055	0.9054	0.2054	0.650549	0.0145	0.0024894977
ss86307289		4	15.139391	0.3804	1.3256	0.249987	0.0217	0.0102080197
ss61522215	*rs41617700*	4	15.178812	0.3861	0.5435	0.461232	0.0139	0.0065752352
ss61490112	*rs41589622*	4	15.383222	0.6146	0.2068	0.649454	0.0088	0.0041465687
ss61490065	*rs41589576*	4	15.404245	0.4076	0.9004	0.343017	0.0182	0.0087656589
ss38335258	*rs29018168*	4	15.464441	0.4928	0.4546	0.500388	0.0124	0.0062187999
ss86285411		4	15.514534	0.2049	1.5976	0.206672	0.0283	0.0092356388
	rs29026835	4	15.562084	0.5924	1.9246	0.165796	0.0248	0.0119636557
	rs29025631	4	15.633403	0.4140	9.4769	0.002163	0.0580	0.0281427745
ss86338476		4	15.683492	0.4148	0.0156	0.900511	0.0023	0.0011354919
ss117967350		4	15.745767	0.3653	0.0666	0.796487	0.0050	0.0022960190
ss86324722		4	15.781454	0.2636	0.3702	0.543078	0.0127	0.0049354812
ss86340678		4	15.80657	0.3352	3.4581	0.063364	0.0360	0.0160413552
ss86298159		4	15.872973	0.1748	1.0513	0.305567	0.0243	0.0070090670
ss61490019	*rs41588730*	4	15.919195	0.8954	0.1994	0.655377	0.0134	0.0025126653
ss61490011	*rs41588722*	4	15.979996	0.4413	0.0871	0.767933	0.0055	0.0026962148
ss86319319		4	16.04969	0.8474	0.4123	0.521041	0.0167	0.0043181472
	rs29022033	4	16.115605	0.4241	0.2607	0.609818	0.0093	0.0045301184
ss86292118		4	16.154062	0.7729	0.0257	0.872695	0.0035	0.0012349260
ss61562248	*rs41659149*	4	16.195428	0.8517	0.0024	0.960812	0.0012	0.0003115275
ss61562242	*rs41659143*	4	16.253485	0.1268	0.0447	0.832530	0.0059	0.0013013211

ss61562239	*rs41659140*	4	16.279543	0.8653	0.0681	0.794228	0.0071	0.0016507304
	rs29020531	4	16.324269	0.3532	0.5572	0.455636	0.0150	0.0068389911
	rs29026870	4	16.441065	0.7973	1.0706	0.301167	0.0240	0.0077694738
	rs29014143	4	16.533095	0.1461	2.0153	0.156168	0.0372	0.0092904526
ss86324591		4	16.563014	0.4491	3.5976	0.058278	0.0356	0.0176238549
ss86309927		4	16.616897	0.2729	0.7720	0.379894	0.0188	0.0074634198
ss86282488		4	16.688976	0.8610	1.9271	0.165521	0.0375	0.0089688006
ss86321886		4	16.789207	0.0924	0.0545	0.815445	0.0076	0.0012673739
ss86340701		4	16.812889	0.6168	0.6055	0.436767	0.0152	0.0071819117
ss61562269	*rs41659170*	4	16.886908	0.7851	0.0156	0.900653	0.0028	0.0009556461
ss86306379		4	16.929751	0.1340	0.2286	0.632712	0.0127	0.0029440857
ss86312539		4	16.98867	0.5544	2.4693	0.116548	0.0297	0.0146852835
ss86287083		4	17.133811	0.3037	0.9303	0.335116	0.0197	0.0083360430
ss117963753		4	17.307216	0.6182	0.3250	0.568816	0.0109	0.0051421342
ss61562260	*rs41659161*	4	17.358208	0.6691	0.0193	0.889635	0.0028	0.0012291204
ss61499196	*rs43709092*	4	17.387337	0.3252	0.2835	0.594590	0.0108	0.0047460472
ss61465700	*rs41568944*	4	17.44653	0.4405	0.1155	0.734078	0.0062	0.0030776645
ss86308725		4	17.530755	0.7235	1.1020	0.294199	0.0214	0.0085465514
ss61531442	*rs41626103*	4	17.552467	0.2736	0.9349	0.333918	0.0197	0.0078442480
ss86333980		4	17.67069	0.5838	2.1025	0.147505	0.0278	0.0135095036
ss86281945		4	17.856922	0.1354	0.7055	0.401238	0.0223	0.0052207158
ss117967438		4	17.920994	0.4713	1.9795	0.159890	0.0259	0.0128957744
ss61514512	*rs41613077*	4	17.966029	0.5745	0.3229	0.570060	0.0105	0.0051267143
ss61514507	*rs41613072*	4	18.025592	0.7128	1.5972	0.206719	0.0260	0.0106563157
ss61514502	*rs41613067*	4	18.082734	0.3861	3.9448	0.047409	0.0380	0.0180200616
ss86314647		4	18.157191	0.6648	0.0264	0.870898	0.0033	0.0014545072
	rs29016340	4	18.236753	0.6304	5.7608	0.016649	0.0462	0.0215187039
ss117963734		4	18.341079	0.4312	0.0214	0.883649	0.0028	0.0013634012
ss61553127		4	18.466019	0.4341	5.9857	0.014669	0.0468	0.0230030993
ss61553125	*rs43709377*	4	18.519898	0.3281	4.2778	0.038981	0.0421	0.0185574872
ss61472749	*rs41573972*	4	18.586133	0.2736	0.3272	0.567508	0.0120	0.0047757157
	rs29010248	4	18.62599	0.7586	1.4440	0.229903	0.0265	0.0096944489
ss86316602		4	18.659507	0.7135	0.2648	0.607036	0.0105	0.0042769229
ss61517835	*rs41619098*	4	18.711888	0.8095	0.0591	0.807978	0.0060	0.0018553447
ss64838440	*rs42933435*	4	18.740857	0.9011	0.0144	0.904616	0.0038	0.0006804265
ss61510312	*rs41603655*	4	18.860003	0.3123	2.3350	0.126953	0.0300	0.0128653226
ss65290509	*rs43373355*	4	18.889233	0.4577	0.5360	0.464356	0.0136	0.0067520547
ss61490165	*rs41590175*	4	18.922649	0.5287	0.0002	0.989175	0.0003	0.0001250234
ss86316784		4	18.971053	0.3825	0.0422	0.837242	0.0039	0.0018346092
ss61525582	*rs41621790*	4	19.023218	0.8331	1.2507	0.263806	0.0281	0.0078144553
ss86340896		4	19.053102	0.7034	0.4705	0.492976	0.0140	0.0058572267
ss61490182	*rs41590192*	4	19.073332	0.4327	0.8146	0.367080	0.0168	0.0082360988
	rs29021001	4	19.119603	0.1368	0.7702	0.380454	0.0232	0.0054869623
ss61554760	*rs41654200*	4	19.199835	0.7106	0.0694	0.792303	0.0054	0.0022337901
ss117974969		4	19.41115	0.3410	0.6329	0.426558	0.0156	0.0070097511
ss65291054	*rs43379196*	4	19.451629	0.1891	0.1655	0.684247	0.0098	0.0030111237
ss65291167	*rs43380309*	4	19.486421	0.8804	1.0667	0.302040	0.0287	0.0060530920

ss63875648	*rs41650055*	4	19.587258	0.1597	1.2295	0.267892		0.0273	0.0073358557
ss63875526	*rs41649095*	4	19.674049	0.1941	0.2446	0.621074		0.0118	0.0036812859
ss63875418	*rs41649041*	4	19.718653	0.6246	1.4963	0.221652		0.0230	0.0108056161
ss61479164	*rs41576135*	4	19.90711	0.5817	1.6089	0.205072		0.0234	0.0114029518
ss64905439	*rs42995849*	4	19.96431	0.0652	7.0700	0.008019		0.0984	0.0119978776
ss65291261	*rs43381701*	4	19.996988	0.9362	6.1288	0.013537		0.0925	0.0110450997
ss61479166	*rs41576137*	4	20.160329	0.7228	0.1169	0.732524		0.0074	0.0029483054
ss63540999	*rs42270895*	4	20.227219	0.6203	0.1987	0.655931		0.0087	0.0040821061
ss86339730		4	20.361118	0.2650	0.0338	0.854190		0.0040	0.0015422160
ss86281017		4	20.432461	0.6812	0.1839	0.668190		0.0085	0.0036773074
	rs29020746	4	20.531145	0.4649	0.4521	0.501584		0.0127	0.0063001221
ss63542040	*rs42264350*	4	20.607724	0.3116	0.9977	0.318216		0.0205	0.0088141717
ss63542244	*rs42264418*	4	20.643099	0.7185	0.7708	0.380260		0.0185	0.0074763370
ss86310971		4	20.676666	0.6755	0.4656	0.495224		0.0132	0.0057894494
ss63542412	*rs42265474*	4	20.698332	0.7629	0.7138	0.398483		0.0185	0.0066999323
ss117967168		4	20.892617	0.7593	0.0454	0.831416		0.0045	0.0016608494
ss61481520	*rs41579634*	4	20.919874	0.0974	0.0284	0.866180		0.0054	0.0009487401
ss64280550	*rs42560120*	4	20.96426	0.3259	0.1677	0.682261		0.0082	0.0036004813
ss61495854		4	21.022961	0.4585	0.0035	0.952693		0.0011	0.0005606397
ss61566608	*rs41666536*	4	21.04867	0.4713	0.0436	0.834745		0.0039	0.0019659166
ss86339215		4	21.091905	0.2328	0.1908	0.662401		0.0095	0.0033796672
ss61511607		4	21.149661	0.7479	1.0956	0.295591		0.0225	0.0084892361
ss86304521		4	21.19646	0.3574	0.7827	0.376618		0.0165	0.0075858335
ss65348508	*rs43432019*	4	21.219862	0.0580	0.8431	0.358832		0.0363	0.0039642153
ss61503878	*rs41600669*	4	21.250385	0.5229	0.0393	0.842823		0.0036	0.0017842561
ss61490337	*rs41591546*	4	21.27205	0.1511	1.2406	0.265739		0.0286	0.0073454425
ss65291460	*rs43372504*	4	21.328223	0.3840	0.1334	0.715022		0.0068	0.0032226794
ss117963732		4	21.357941	0.1397	0.2960	0.586579		0.0143	0.0034355846
ss61490311	*rs41590720*	4	21.506497	0.7213	0.1811	0.670586		0.0088	0.0035509106
ss86291291		4	21.538282	0.1691	0.0071	0.933025		0.0022	0.0006071411
ss65291701	*rs43375445*	4	21.56234	0.2013	1.8696	0.171958		0.0328	0.0105468258
ss117967264		4	21.611451	0.2113	0.1968	0.657469		0.0100	0.0033470198
ss61555055	*rs41650492*	4	21.635539	0.5946	1.0384	0.308549		0.0195	0.0093943414
ss65292116	*rs43379560*	4	21.67109	0.5867	0.1581	0.691003		0.0075	0.0036247250
ss61570568	*rs41668925*	4	21.722746	0.6562	0.0082	0.928030		0.0018	0.0008110379
ss65292191	*rs43380735*	4	21.756956	0.3804	0.0253	0.873680		0.0031	0.0014743258
ss61555099	*rs41651635*	4	21.807441	0.3947	0.5132	0.473998		0.0137	0.0065243129
ss65292285	*rs43380829*	4	21.82995	0.0745	1.1770	0.278338		0.0397	0.0054715467
	rs29022501	4	21.855977	0.1476	0.1065	0.744309		0.0086	0.0021573926
ss86338562		4	21.94765	0.8646	0.0340	0.853655		0.0050	0.0011677566
ss61555211		4	21.992116	0.2099	0.0384	0.844635		0.0046	0.0015403113
ss86341696		4	22.015576	0.2350	0.2515	0.616208		0.0111	0.0040066317
ss65292557	*rs43375405*	4	22.039295	0.7894	0.0163	0.898588		0.0030	0.0010038614
ss86296297		4	22.158743	0.5501	0.0181	0.892960		0.0026	0.0012987769
ss65292776	*rs43377222*	4	22.191402	0.3102	0.2538	0.614598		0.0103	0.0044228439
ss61570574	*rs41670131*	4	22.229768	0.6862	0.0911	0.762915		0.0062	0.0026543246
ss63383407	*rs42210739*	4	22.459683	0.2801	1.2101	0.271699		0.0226	0.0091009449

ss63383467	*rs42210769*	4	22.499222	0.0451	2.0349	0.154169	0.0646	0.0055632830
ss63383563	*rs42210816*	4	22.562648	0.6848	0.0742	0.785364	0.0056	0.0024167357
ss63383577	*rs42210823*	4	22.587804	0.6805	0.1637	0.685871	0.0083	0.0035875674
ss63383623	*rs42211846*	4	22.660108	0.3517	0.0767	0.781901	0.0055	0.0025251323
ss86313834		4	22.70066	0.7958	1.2397	0.265907	0.0256	0.0083104284
ss86294859		4	22.881621	0.7679	0.1395	0.708884	0.0084	0.0029932687
ss86293716		4	22.913341	0.3940	0.0560	0.813084	0.0045	0.0021710549
ss63383977	*rs42213121*	4	23.032572	0.6540	0.0002	0.987860	0.0003	0.0001375698
ss117967306		4	23.058026	0.3403	0.0196	0.888747	0.0027	0.0012194749
ss117967442		4	23.085517	0.3266	0.0023	0.961802	0.0009	0.0004077060
ss117967274		4	23.124765	0.7213	0.0859	0.769592	0.0061	0.0024609389
ss61570185	*rs41665850*	4	23.208148	0.7751	0.3089	0.578558	0.0122	0.0042673046
ss86317889		4	23.255761	0.6956	4.5224	0.033805	0.0439	0.0186114633
ss63384433	*rs42216046*	4	23.301546	0.1898	0.2713	0.602631	0.0125	0.0038319180
ss63384463	*rs42216061*	4	23.331433	0.5036	4.6405	0.031568	0.0394	0.0196804155
ss63384509	*rs42216084*	4	23.35997	0.8625	0.4555	0.499961	0.0188	0.0044579089
ss86317465		4	23.487903	0.1619	1.7221	0.189850	0.0327	0.0088856547
ss65292898	*rs43379643*	4	23.540614	0.1340	4.2463	0.039708	0.0555	0.0128690980
ss86290241		4	23.585869	0.2550	3.4912	0.062115	0.0391	0.0148488161
ss86292617		4	23.624638	0.1999	2.1997	0.138489	0.0342	0.0109529529
ss64561832	*rs42700367*	4	23.682083	0.6984	0.8755	0.349756	0.0186	0.0078547312
ss61490412	*rs41591617*	4	23.742638	0.1569	2.3108	0.128929	0.0401	0.0106025258
ss63384591	*rs42216125*	4	23.781991	0.0953	0.2573	0.612119	0.0159	0.0027461384
ss86337807		4	23.847509	0.6511	0.0291	0.864654	0.0033	0.0015189663
ss61555314	*rs41653938*	4	23.89244	0.3940	5.6212	0.018016	0.0436	0.0208178194
ss61490432	*rs41587943*	4	24.290694	0.5107	2.4259	0.119802	0.0284	0.0142170575
ss61490431	*rs41587942*	4	24.337367	0.8431	0.4507	0.502224	0.0173	0.0045758400
ss86323307		4	24.375957	0.1225	0.5989	0.439265	0.0213	0.0045762344
ss86337686		4	24.41112	0.4420	0.0243	0.876058	0.0030	0.0014636605
ss86330434		4	24.565328	0.1476	0.7331	0.392189	0.0229	0.0057708603
ss61523909	*rs41624266*	4	24.595521	0.8746	0.0936	0.759714	0.0087	0.0019034932
ss117967269		4	24.624406	0.5158	1.1917	0.275359	0.0212	0.0106065593
ss62343124	*rs41841065*	4	24.738194	0.7163	1.9931	0.158465	0.0286	0.0116427242
ss64297306	*rs42570765*	4	24.772942	0.4678	2.6845	0.101782	0.0306	0.0152267383
ss65293127	*rs43382171*	4	24.808218	0.4212	0.0098	0.921287	0.0019	0.0009124542
ss86341308		4	24.86728	0.8345	3.0624	0.080562	0.0438	0.0121007172
ss61538167	*rs41638755*	4	24.895184	0.6762	4.2319	0.040043	0.0405	0.0177433619
	rs29023731	4	24.924029	0.5752	0.1633	0.686296	0.0077	0.0037839197
	rs29024053	4	25.031892	0.6225	0.8970	0.343905	0.0185	0.0086978997
ss64362973	*rs42598643*	4	25.072742	0.2994	0.3313	0.565068	0.0118	0.0049308887
ss86316303		4	25.183065	0.4319	3.2038	0.073901	0.0337	0.0165257199
ss38328006	*rs29020105*	4	25.203804	0.3854	0.2452	0.620638	0.0096	0.0045417868
ss61468952	*rs41566065*	4	25.321539	0.4828	0.2673	0.605344	0.0098	0.0049096052
	rs29013301	4	25.355321	0.1583	0.7246	0.394924	0.0217	0.0057714071
ss65293453	*rs43385164*	4	25.381361	0.4463	0.4934	0.482643	0.0133	0.0065796128
ss65293472	*rs43385183*	4	25.403484	0.8589	1.0070	0.315962	0.0267	0.0064738687
ss61555334	*rs41653958*	4	25.428867	0.8102	0.0002	0.988179	0.0004	0.0001099925

ss64786285	*rs42879091*	4	25.509038	0.1791	0.9868	0.320878	0.0237	0.0069605690
ss64786304	*rs42879110*	4	25.546708	0.8209	0.9868	0.320878	0.0237	0.0069605690
ss65293691	*rs43377902*	4	25.602178	0.1791	0.9868	0.320878	0.0237	0.0069605690
ss61511267	*rs41610788*	4	25.686933	0.6777	0.1986	0.655990	0.0089	0.0038791605
ss64947523	*rs43037312*	4	25.717525	0.2400	3.4972	0.061894	0.0420	0.0153328241
ss64928448	*rs43020002*	4	25.743842	0.4019	0.2070	0.649261	0.0090	0.0043092432
ss86306964		4	25.945516	0.3496	1.0731	0.300616	0.0203	0.0092287547
ss61509972	*rs41600321*	4	25.976225	0.2958	1.3642	0.243213	0.0242	0.0100937020
ss61490455	*rs41587961*	4	26.102302	0.2729	0.9054	0.341658	0.0196	0.0077679815
ss65293778	*rs43379289*	4	26.193545	0.2364	0.0193	0.889664	0.0032	0.0011377840
ss65293796	*rs43379307*	4	26.216279	0.8954	1.7133	0.190988	0.0381	0.0071312047
ss61555343	*rs41653967*	4	26.238628	0.9362	2.0205	0.155639	0.0510	0.0060873650
ss61555345	*rs41653969*	4	26.32315	0.6225	0.1693	0.680857	0.0078	0.0036478783
ss61555348	*rs41653972*	4	26.380254	0.7787	0.0049	0.944428	0.0016	0.0005518983
ss86309946		4	26.403626	0.7801	0.0117	0.914006	0.0025	0.0008552642
ss86298313		4	26.43581	0.1963	3.8291	0.050770	0.0462	0.0145725870
ss65293996	*rs43381606*	4	26.540696	0.6440	2.6190	0.106046	0.0329	0.0150890954
ss86310004		4	26.601688	0.3546	2.8341	0.092730	0.0316	0.0144513922
ss65072842	*rs43163571*	4	26.647525	0.7421	3.7267	0.053954	0.0398	0.0152255281
ss63771697	*rs42345023*	4	26.78786	0.0960	4.7662	0.029358	0.0700	0.0121524542
ss61506269	*rs41604484*	4	26.89556	0.5774	4.9675	0.026147	0.0431	0.0210302382
ss86313434		4	26.971807	0.3288	1.5506	0.213473	0.0253	0.0111621027
ss63771397	*rs42344174*	4	27.002371	0.4964	2.0816	0.149537	0.0260	0.0129886393
ss63771317	*rs42344134*	4	27.084674	0.4749	0.2330	0.629433	0.0092	0.0045928386
ss61515585	*rs41615938*	4	27.108523	0.0774	0.0694	0.792262	0.0092	0.0013117909
ss63771165	*rs42343558*	4	27.135661	0.4083	0.0748	0.784555	0.0051	0.0024773672
ss61515582	*rs41615935*	4	27.162987	0.3080	10.4049	0.001316	0.0691	0.0294437321
ss61506270	*rs41604485*	4	27.240338	0.1791	0.4086	0.522910	0.0153	0.0044843588
ss64165261	*rs42503854*	4	27.316448	0.2837	0.3691	0.543686	0.0127	0.0051474762
ss64165311	*rs42503879*	4	27.339005	0.5093	0.2197	0.639378	0.0086	0.0043108708
ss86288038		4	27.393015	0.8431	6.1946	0.013047	0.0639	0.0169020772
ss64907462	*rs42997342*	4	27.436281	0.3840	2.5693	0.109409	0.0312	0.0147670559
ss64165385	*rs42503916*	4	27.465875	0.4699	0.8083	0.368931	0.0174	0.0086504995
ss61562939	*rs41657739*	4	27.487644	0.3804	0.2729	0.601568	0.0098	0.0046417187
ss64165437	*rs42504742*	4	27.513294	0.1325	2.0348	0.154180	0.0406	0.0093280944
ss64875287	*rs42973274*	4	27.554376	0.6375	1.5262	0.217102	0.0237	0.0109499343
ss64413790	*rs42627468*	4	27.580356	0.4885	0.3951	0.529826	0.0117	0.0058422768
ss64413854	*rs42627500*	4	27.627977	0.1468	1.4615	0.227103	0.0328	0.0082066768
ss64413936	*rs42628241*	4	27.686096	0.6547	2.2688	0.132454	0.0297	0.0134361016
	rs29027498	4	27.732337	0.0752	0.7799	0.377464	0.0308	0.0042876126
ss65295251	*rs43381856*	4	27.761909	0.3424	2.1566	0.142411	0.0288	0.0129874838
ss86310598		4	27.7945	0.5702	0.0392	0.843188	0.0037	0.0017898240
ss65295182	*rs43380987*	4	27.83256	0.6024	0.0383	0.844807	0.0038	0.0018258167
ss86276965		4	27.869426	0.7923	2.8414	0.092311	0.0389	0.0128069617
ss61555372	*rs41653995*	4	27.905508	0.2357	0.6803	0.409762	0.0181	0.0065383650
ss86284970		4	27.964029	0.3238	1.1257	0.289064	0.0209	0.0091459548
	rs29013539	4	28.014311	0.7328	0.4363	0.509138	0.0139	0.0054623759

ss61555369	rs41653992	4	28.072054	0.3295	0.7153	0.397981	0.0165	0.0072837274
	rs29020561	4	28.119051	0.3403	0.0135	0.907497	0.0022	0.0010016123
ss61490465	rs41587971	4	28.152251	0.3410	0.0237	0.877782	0.0029	0.0013246336
ss65294813	rs43378122	4	28.199311	0.3446	0.3869	0.534123	0.0119	0.0053963212
	rs29017061	4	28.239398	0.3309	0.2380	0.625819	0.0096	0.0042437717
ss65294733	rs43378045	4	28.263522	0.9019	0.5209	0.470718	0.0219	0.0038811400
ss65294479	rs43386788	4	28.345672	0.9112	2.0621	0.151454	0.0466	0.0075506281
ss61555365	rs41653989	4	28.448534	0.2407	3.5543	0.059806	0.0406	0.0148288248
ss86338195		4	28.500953	0.7042	5.8210	0.016093	0.0479	0.0199739560
ss86306078		4	28.583828	0.2665	1.8748	0.171368	0.0284	0.0111151016
ss86302887		4	28.659227	0.3302	6.8051	0.009285	0.0500	0.0221110667
ss61493788	rs41590313	4	28.754236	0.4928	9.8445	0.001775	0.0588	0.0293743226
ss63560293	rs42269361	4	28.805506	0.7385	5.2063	0.022806	0.0481	0.0185680082
ss117963729		4	28.848782	0.3524	0.1537	0.695136	0.0077	0.0034933017
ss61525205		4	28.916716	0.4334	0.9092	0.340653	0.0182	0.0089569080
ss86295118		4	28.946085	0.1841	2.4335	0.119223	0.0376	0.0113066425
	rs29017637	4	29.070462	0.2622	0.2811	0.596130	0.0113	0.0043718367
ss63559933	rs42268943	4	29.092761	0.6705	0.2235	0.636562	0.0096	0.0042578753
ss61563359	rs41660833	4	29.226201	0.2880	1.1094	0.292569	0.0217	0.0089187535
ss61494157	rs41594363	4	29.27902	0.3410	4.1744	0.041416	0.0399	0.0179351912
ss61477583	rs41577913	4	29.463118	0.5337	0.4930	0.482808	0.0136	0.0067650758
ss61563360	rs41660834	4	29.498592	0.4398	0.4223	0.515992	0.0127	0.0062466566
ss64860356	rs42951387	4	29.529357	0.1404	2.8658	0.090930	0.0437	0.0105507564
ss65295786	rs43377794	4	29.586774	0.7500	0.0311	0.860082	0.0038	0.0014403739
ss65295758	rs43377766	4	29.62126	0.3274	0.4219	0.516196	0.0131	0.0057831861
ss117963722		4	29.650678	0.7020	0.0245	0.875552	0.0032	0.0013471765
ss61553915	rs41648763	4	29.690929	0.7701	1.0093	0.315410	0.0224	0.0079465500
ss86304333		4	29.813581	0.2550	0.0020	0.964500	0.0009	0.0003600991
ss65295527	rs43376238	4	29.840417	0.4628	1.1520	0.283503	0.0201	0.0100048050
ss86277620		4	29.863126	0.3109	2.8505	0.091795	0.0334	0.0142913976
ss86336171		4	30.011687	0.7944	4.2039	0.040704	0.0475	0.0155176342
ss64550514	rs42689154	4	30.064405	0.7693	0.9730	0.324266	0.0209	0.0074284380
ss64104783	rs42475216	4	30.186009	0.2443	2.4031	0.121550	0.0328	0.0121017997
ss117975081		4	30.229108	0.3660	0.0031	0.955607	0.0011	0.0004924776
ss64104259	rs42473054	4	30.25985	0.8223	2.0714	0.150537	0.0340	0.0099350136
ss64105207	rs42476825	4	30.285845	0.1777	2.0714	0.150537	0.0340	0.0099350136
ss64237494	rs42544103	4	30.314123	0.8202	2.6922	0.101295	0.0387	0.0114046424
ss86299454		4	30.383802	0.6225	0.0008	0.977951	0.0005	0.0002437649
ss64542430	rs42687040	4	30.416403	0.0688	0.4588	0.498392	0.0247	0.0031611166
ss63804172	rs42356218	4	30.579559	0.1769	2.3648	0.124552	0.0364	0.0105872521
ss86288549		4	30.647622	0.1855	1.9911	0.158675	0.0328	0.0099089649
ss63804288	rs42356876	4	30.685081	0.7013	0.3527	0.552767	0.0120	0.0050341229
ss61472697	rs41572920	4	30.727733	0.4334	0.1902	0.662895	0.0082	0.0040208870
ss86329421		4	30.801551	0.5681	2.6351	0.104979	0.0305	0.0149844684
ss63804660	rs42357858	4	30.871795	0.6691	6.2106	0.012931	0.0478	0.0211822414
ss86323915		4	30.934597	0.2350	0.1110	0.739129	0.0074	0.0026462422
ss64856633	rs42949874	4	31.086332	0.2393	0.0311	0.860040	0.0038	0.0013966945

ss65295954	*rs43378861*	4	31.124198	0.2385	0.0736	0.786239	0.0059	0.0021512591
ss65295894	*rs43378301*	4	31.207342	0.7600	0.1343	0.714091	0.0080	0.0029178432
ss65295850	*rs43378257*	4	31.231342	0.2364	0.0183	0.892374	0.0030	0.0010753211
ss61489710	*rs41586428*	4	31.261759	0.7049	3.4031	0.065498	0.0367	0.0152885528
ss61553921	*rs41648769*	4	31.354284	0.5344	0.7173	0.397315	0.0155	0.0077279253
	rs29017613	4	31.394017	0.7235	0.4620	0.496895	0.0139	0.0055712394
ss86278877		4	31.501475	0.7521	0.6101	0.435020	0.0168	0.0062521422
ss61507889	*rs41599879*	4	31.542035	0.7235	0.5432	0.461356	0.0151	0.0060410183
ss64476632	*rs42655239*	4	31.615842	0.2479	0.7174	0.397301	0.0181	0.0067534998
ss61496714	*rs41592066*	4	31.712931	0.4771	0.8607	0.353873	0.0174	0.0086761646
ss86293773		4	31.738957	0.1032	7.6728	0.005755	0.0837	0.0154812236
ss86309400		4	31.793513	0.6533	5.6784	0.017442	0.0445	0.0201739473
ss64361941	*rs42600726*	4	31.867869	0.8231	0.1848	0.667410	0.0104	0.0030232696
ss86301024		4	31.954213	0.6053	0.1069	0.743774	0.0061	0.0029315247
ss86281575		4	31.983955	0.4900	0.0009	0.976113	0.0006	0.0002779482
ss65296721	*rs43378829*	4	32.017557	0.4513	0.1540	0.694839	0.0072	0.0035680984
ss86334156		4	32.162131	0.1691	0.0103	0.919211	0.0025	0.0006984531
ss86307898		4	32.313935	0.5666	0.0740	0.785713	0.0051	0.0025221415
ss65296082	*rs43379389*	4	32.345995	0.2837	0.1398	0.708604	0.0078	0.0031830744
ss86315732		4	32.435687	0.6884	1.6702	0.196655	0.0263	0.0112631178
ss86333941		4	32.467325	0.3453	4.9506	0.026402	0.0440	0.0198815077
ss86321308		4	32.499764	0.8890	2.5651	0.109699	0.0478	0.0094286019
ss65296851	*rs43380659*	4	32.751764	0.0652	3.2053	0.073834	0.0665	0.0081083442
ss86334901		4	32.864441	0.7314	0.1434	0.704994	0.0083	0.0032566612
ss117963763		4	32.921997	0.2049	2.5037	0.114037	0.0376	0.0122523020
ss65296972	*rs43381376*	4	32.966144	0.7822	1.3197	0.251036	0.0268	0.0091280860
ss86341524		4	33.001169	0.7958	1.5492	0.213673	0.0295	0.0095825971
ss86314805		4	33.044815	0.7271	4.3345	0.037712	0.0434	0.0172134622
ss86322917		4	33.204763	0.3567	0.0064	0.936444	0.0016	0.0007219466
ss65297547	*rs43379048*	4	33.30907	0.1218	0.1201	0.728986	0.0099	0.0021079706
ss86341049		4	33.336052	0.7529	1.7897	0.181399	0.0300	0.0111729143
ss61489724	*rs41586842*	4	33.396676	0.6232	0.5188	0.471609	0.0141	0.0066277160
ss64271864	*rs42562193*	4	33.961765	0.0659	0.5429	0.461494	0.0280	0.0034431916
ss65298496	*rs43386193*	4	34.286419	0.6347	0.0343	0.853059	0.0035	0.0016335469
ss86319253		4	34.317619	0.3417	0.5527	0.457471	0.0145	0.0065313811
ss65298449	*rs43386146*	4	34.362492	0.9148	3.6255	0.057312	0.0638	0.0099483975
ss65298430	*rs43385427*	4	34.401122	0.9062	0.4118	0.521266	0.0206	0.0035036499
ss65298214	*rs43383611*	4	34.451281	0.1848	0.1946	0.659247	0.0110	0.0033009205
ss64426758	*rs42635026*	4	34.549192	0.3216	5.7959	0.016323	0.0464	0.0202458321
ss61569843	*rs41670394*	4	34.574484	0.7371	3.9026	0.048605	0.0404	0.0156752146
	rs29025219	4	34.638645	0.5494	0.1097	0.740574	0.0061	0.0030042965
ss61527504	*rs41624164*	4	34.709498	0.8073	0.5349	0.464795	0.0175	0.0054393747
ss61527510	*rs41624170*	4	34.747886	0.5086	0.0306	0.861193	0.0033	0.0016417768
ss86290793		4	34.833439	0.3646	0.1395	0.708932	0.0071	0.0032905880
ss61540358	*rs41642704*	4	34.877755	0.4162	0.0000	0.995100	0.0001	0.0000562585
	rs29026882	4	34.905974	0.5824	0.8541	0.355725	0.0172	0.0083447289
ss61540363	*rs41642709*	4	34.9284	0.6132	0.0574	0.810779	0.0046	0.0021733105

ss64950350	*rs43038313*	4	35.000732	0.8632	0.5985	0.439424	0.0217	0.0051221203
ss63649003	*rs42302115*	4	35.043567	0.3532	1.9698	0.160920	0.0270	0.0123569415
ss61496863	*rs41592806*	4	35.071826	0.3195	2.6201	0.105973	0.0325	0.0141273103
ss61511932	*rs41609646*	4	35.103712	0.1519	3.0866	0.079379	0.0443	0.0114111540
ss64810267	*rs42910197*	4	35.249217	0.9198	1.0377	0.308708	0.0347	0.0051174854
ss86290605		4	35.387183	0.6705	0.0807	0.776424	0.0054	0.0023983197
ss63937195	*rs42390702*	4	35.417767	0.8367	0.7274	0.394022	0.0205	0.0056068907
ss63937293	*rs42391751*	4	35.452342	0.9298	1.6286	0.202325	0.0457	0.0059618423
ss63937473	*rs42392641*	4	35.503156	0.7221	0.1842	0.667935	0.0088	0.0035421243
ss63937799	*rs42393504*	4	35.621431	0.7772	0.0958	0.756982	0.0069	0.0023915821
ss86336898		4	35.770101	0.3574	2.0551	0.152144	0.0277	0.0127197342
ss86341247		4	35.919009	0.1798	2.8233	0.093355	0.0416	0.0122736510
ss65298716	*rs43381814*	4	36.086679	0.8532	2.3579	0.125108	0.0402	0.0100639600
ss65024780	*rs43115094*	4	36.434227	0.2092	3.3891	0.066052	0.0432	0.0143045807
ss86278751		4	36.496385	0.9047	0.5074	0.476516	0.0228	0.0039267319
ss63807880	*rs42358265*	4	36.548292	0.6218	0.0111	0.916176	0.0020	0.0009552217
ss65298918	*rs43384215*	4	36.629254	0.5337	1.1476	0.284419	0.0202	0.0100601172
ss86311616		4	36.663071	0.4105	3.3245	0.068682	0.0348	0.0168537870
ss61553961	*rs41648808*	4	36.688428	0.4756	5.2418	0.022349	0.0424	0.0211742203
ss61553968		4	36.713959	0.6468	1.2746	0.259303	0.0218	0.0099743502
ss86301499		4	36.769991	0.7693	5.1170	0.024000	0.0497	0.0176379722
ss61489726	*rs41586844*	4	36.814032	0.5673	0.8017	0.370889	0.0162	0.0079481819
ss65299282	*rs43387579*	4	36.850767	0.0852	1.3778	0.240885	0.0384	0.0059811035
ss86330835		4	36.872511	0.3997	0.0747	0.784756	0.0052	0.0024739117
ss64153347	*rs42500432*	4	36.979771	0.6010	0.9586	0.327872	0.0189	0.0090575368
ss86301786		4	37.011604	0.4821	2.0854	0.149166	0.0279	0.0139188442
	rs29014908	4	37.048319	0.5759	1.7664	0.184258	0.0251	0.0122581214
ss64153955	*rs42502736*	4	37.072537	0.2471	0.1420	0.706368	0.0082	0.0030341724
ss64829655	*rs42923947*	4	37.096062	0.7450	0.0303	0.861787	0.0036	0.0013668395
ss64829548	*rs42923240*	4	37.122658	0.6898	0.3778	0.538962	0.0124	0.0053025967
ss61562498	*rs41661797*	4	37.149015	0.2543	0.1009	0.750827	0.0065	0.0024757538
ss64154135	*rs42502826*	4	37.173302	0.5609	0.0024	0.960678	0.0009	0.0004472192
ss61513425	*rs41612105*	4	37.202697	0.3145	0.0279	0.867470	0.0034	0.0014457884
ss86316913		4	37.254423	0.7156	0.8939	0.344753	0.0198	0.0080730095
ss61566085	*rs41659730*	4	37.322394	0.2801	4.0020	0.045833	0.0404	0.0162901879
ss86337183		4	37.371783	0.5673	2.1138	0.146429	0.0267	0.0131114505
ss86276184		4	37.478536	0.8238	6.9576	0.008533	0.0628	0.0182222476
ss117967478		4	37.514065	0.5143	3.4298	0.064455	0.0345	0.0172162848
ss64058597	*rs42455643*	4	37.574828	0.4291	0.4871	0.485473	0.0130	0.0063913002
ss64058465	*rs42454677*	4	37.617189	0.2493	1.2523	0.263503	0.0243	0.0090936201
ss117967445		4	37.640846	0.3517	0.5610	0.454098	0.0143	0.0065039934
ss117967215		4	37.665546	0.7378	9.5671	0.002060	0.0646	0.0249873835
ss86286893		4	37.712896	0.2615	0.8753	0.349814	0.0196	0.0075698668
ss63855148	*rs42384861*	4	37.771661	0.3317	1.0785	0.299393	0.0205	0.0090756643
ss63855258	*rs42384916*	4	37.81182	0.1454	1.0193	0.313040	0.0264	0.0065721777
ss117967476		4	37.908642	0.0580	4.0009	0.045864	0.0798	0.0087283744
ss61473785	*rs41574101*	4	37.991515	0.4649	0.6751	0.411577	0.0155	0.0077074970

ss61474974	*rs41574160*	4	38.046214	0.8417	0.6086	0.435584	0.0205	0.0054733890
	rs29020436	4	38.111247	0.2450	0.2566	0.612603	0.0110	0.0040764143
ss61552152	*rs43708409*	4	38.167246	0.5838	0.1728	0.677766	0.0081	0.0039139009
ss63258790	*rs42172040*	4	38.225981	0.6805	0.0051	0.943339	0.0014	0.0006263585
ss63258202	*rs42169452*	4	38.291933	0.2414	3.5967	0.058308	0.0414	0.0151477068
ss86321549		4	38.315919	0.1927	4.9038	0.027121	0.0518	0.0161151838
ss63257386	*rs42165880*	4	38.354798	0.8789	0.7915	0.373951	0.0252	0.0053557817
ss61523577	*rs41622240*	4	38.407433	0.6261	0.6361	0.425409	0.0151	0.0070674103
ss86319386		4	38.457026	0.1160	0.0003	0.986751	0.0005	0.0001012613
	rs29010786	4	38.53075	0.8173	3.7242	0.054035	0.0476	0.0142164559
ss65299399	*rs43388196*	4	38.553348	0.3890	0.4259	0.514204	0.0126	0.0059917128
ss61553970	*rs41648817*	4	38.584854	0.8711	0.1384	0.709962	0.0104	0.0023372775
ss61510223	*rs41602667*	4	38.633156	0.4219	2.5437	0.111186	0.0309	0.0150611035
ss65301134	*rs43387230*	4	38.66537	0.7915	0.3064	0.580060	0.0121	0.0039790383
	rs29027495	4	38.699574	0.1318	0.0915	0.762396	0.0083	0.0019024618
ss65301261	*rs43388554*	4	38.740283	0.3023	0.0000	0.997041	0.0001	0.0000324113
	rs29015286	4	38.777811	0.6268	0.9867	0.320904	0.0192	0.0089682466
	rs29016986	4	38.894272	0.7665	1.2863	0.257125	0.0252	0.0090326928
ss61489742	*rs41586860*	4	39.037652	0.1254	0.0536	0.817016	0.0064	0.0013952175
ss61554026	*rs41649773*	4	39.075742	0.2342	1.2413	0.265611	0.0242	0.0086757134
ss61489744	*rs41586862*	4	39.121284	0.3302	0.0055	0.940672	0.0014	0.0006334472
ss61554028	*rs41649775*	4	39.174917	0.4713	3.4876	0.062250	0.0347	0.0172857294
ss61526437	*rs41623631*	4	39.229734	0.5602	2.8473	0.091973	0.0317	0.0156195742
ss61510229	*rs41602673*	4	39.29444	0.5201	0.5564	0.455970	0.0139	0.0069567123
ss86292967		4	39.322589	0.9105	0.9682	0.325483	0.0322	0.0052539479
ss65301855	*rs43385850*	4	39.344961	0.0960	3.9797	0.046443	0.0615	0.0106683170
ss61554071	*rs41649818*	4	39.369455	0.5981	1.6030	0.205900	0.0243	0.0116897692
ss65302102	*rs43387397*	4	39.391612	0.1569	6.3350	0.012062	0.0640	0.0169310496
ss65302114	*rs43387409*	4	39.413508	0.7170	3.6150	0.057673	0.0396	0.0160741373
	rs29011657	4	39.452723	0.1046	0.2105	0.646543	0.0138	0.0025786424
ss65302157	*rs43388052*	4	39.490596	0.6533	2.5970	0.107521	0.0312	0.0141561793
ss86309375		4	39.589163	0.4298	0.5705	0.450314	0.0147	0.0072191852
ss61554074	*rs41649821*	4	39.640113	0.2686	0.6336	0.426302	0.0170	0.0066680427
ss61489796	*rs41586914*	4	39.744935	0.5337	2.6553	0.103655	0.0302	0.0150086994
ss86341091		4	39.76926	0.5330	2.4042	0.121462	0.0287	0.0143009897
ss61510236	*rs41602679*	4	39.849632	0.5938	1.1691	0.279967	0.0202	0.0097344484
ss117967432		4	39.876876	0.6411	2.8836	0.089935	0.0321	0.0147743462
ss86335795		4	39.908793	0.3338	3.7127	0.054407	0.0383	0.0170177728
ss117967544		4	39.959463	0.4756	3.0810	0.079653	0.0321	0.0160277062
ss65302428	*rs43390122*	4	39.988301	0.3646	5.6025	0.018207	0.0463	0.0214504987
ss117963749		4	40.013652	0.8832	0.3932	0.530814	0.0182	0.0037524065
ss65302456	*rs43391250*	4	40.036346	0.8875	1.0032	0.316895	0.0296	0.0059076176
ss65607111	*rs43691962*	4	40.102805	0.1920	0.0207	0.885631	0.0031	0.0009754742
ss64901561	*rs42993006*	4	40.236015	0.2407	0.0591	0.808000	0.0053	0.0019416774
ss117967212		4	40.280466	0.5244	0.0047	0.945330	0.0013	0.0006236822
ss86340969		4	40.44736	0.2235	0.0150	0.902679	0.0028	0.0009687741
ss64015202	*rs42435059*	4	40.479754	0.2199	0.1320	0.716442	0.0083	0.0028473430

ss64015184	*rs42435050*	4	40.519906	0.7880	0.0815	0.775300	0.0066	0.0022168604
ss64797627	*rs42893124*	4	40.735158	0.3503	0.2462	0.619926	0.0098	0.0044503670
ss61489734	*rs41586852*	4	40.781673	0.8109	0.5661	0.452063	0.0183	0.0056274796
ss61554005	*rs41649752*	4	40.869775	0.5021	1.3555	0.244725	0.0218	0.0108997155
	rs29010856	4	40.957217	0.6426	0.0314	0.859316	0.0035	0.0016302189
ss65300759	*rs43384656*	4	41.001273	0.9520	1.1934	0.275024	0.0481	0.0043942455
ss86335823		4	41.126361	0.3861	3.8159	0.051168	0.0378	0.0178970598
ss86303143		4	41.15651	0.5136	1.9456	0.163503	0.0258	0.0129003317
ss61543095	*rs41638414*	4	41.191479	0.1082	4.4146	0.035992	0.0636	0.0122758467
ss64947186	*rs43043471*	4	41.23002	0.5301	1.2041	0.272875	0.0203	0.0100951909
ss86310679		4	41.309753	0.2120	1.7976	0.180440	0.0307	0.0102731571
ss86338995		4	41.381329	0.5272	1.6375	0.201099	0.0236	0.0117526309
ss65300455	*rs43388653*	4	41.550073	0.7600	0.6909	0.406142	0.0187	0.0068263928
ss61553983	*rs41648830*	4	41.603494	0.4319	0.0045	0.946270	0.0013	0.0006163665
ss61528843	*rs41629561*	4	41.683743	0.3976	1.2357	0.266687	0.0217	0.0104029739
ss86339661		4	41.822513	0.6454	0.1439	0.704534	0.0073	0.0033462515
ss65300122	*rs43385720*	4	41.929497	0.7242	0.5065	0.476891	0.0146	0.0058454914
ss86274698		4	41.989602	0.9448	1.1983	0.274048	0.0447	0.0046574688
ss61553977	*rs41648824*	4	42.106541	0.4348	1.9888	0.158913	0.0267	0.0131180023
ss86338706		4	42.235565	0.4799	1.2659	0.260922	0.0211	0.0105349720
ss86338961		4	42.266512	0.3231	0.5643	0.452794	0.0145	0.0063361691
ss86311492		4	42.310703	0.2421	0.3813	0.537099	0.0136	0.0049938949
ss62951409	*rs42053383*	4	42.333031	0.1275	0.4146	0.519837	0.0176	0.0039161942
ss62951499	*rs42053413*	4	42.361136	0.5537	2.1962	0.138803	0.0284	0.0140269884
ss63576315	*rs42277262*	4	42.483245	0.8266	1.3884	0.239074	0.0294	0.0084338257
ss63575751	*rs42275674*	4	42.55567	0.7858	0.5202	0.470979	0.0167	0.0056272663
ss61477422	*rs41576757*	4	42.591521	0.5036	0.3747	0.540672	0.0113	0.0056603052
ss86338324		4	42.620199	0.7056	0.8951	0.344426	0.0201	0.0083363893
ss86334117		4	42.800179	0.4964	0.0164	0.898113	0.0024	0.0011977990
ss86304192		4	42.822541	0.6397	0.4683	0.494011	0.0134	0.0061734163
ss64353011	*rs42595691*	4	42.875445	0.6404	4.4445	0.035371	0.0407	0.0187511734
ss64944195	*rs43039087*	4	42.90942	0.2099	2.4067	0.121271	0.0361	0.0119774135
ss64789301	*rs42880106*	4	43.033215	0.8481	1.1504	0.283829	0.0274	0.0070556707
ss61499865	*rs41596243*	4	43.144433	0.7951	1.5480	0.213846	0.0296	0.0096520273
ss65302565	*rs43384459*	4	43.207089	0.7586	2.6641	0.103087	0.0356	0.0130495827
ss65302663	*rs43385757*	4	43.295634	0.2264	1.7761	0.183061	0.0294	0.0103101608
ss65302669	*rs43385763*	4	43.321794	0.8087	4.2677	0.039213	0.0469	0.0145206309
ss64938419	*rs43027711*	4	43.351468	0.9133	1.0952	0.295686	0.0343	0.0054360085
ss117967317		4	43.381995	0.8911	1.5203	0.217998	0.0368	0.0071477106
ss61489803	*rs41586921*	4	43.431715	0.1841	5.6957	0.017272	0.0547	0.0164239800
ss65302819	*rs43386613*	4	43.458502	0.9019	1.4734	0.225220	0.0375	0.0066355400
ss61554103	*rs41650650*	4	43.485808	0.4004	0.8879	0.346368	0.0177	0.0084873514
ss65303033	*rs43388027*	4	43.53279	0.5580	0.0591	0.807970	0.0046	0.0022466014
ss61554104	*rs41650651*	4	43.583456	0.6640	0.0063	0.936889	0.0016	0.0006982639
ss65303162	*rs43389856*	4	43.731206	0.3059	0.0224	0.881012	0.0031	0.0013050535
ss61510237	*rs41602680*	4	43.767945	0.6390	0.1741	0.676591	0.0082	0.0038015454
ss65303314	*rs43391107*	4	43.820957	0.1956	3.2013	0.074016	0.0426	0.0134026816

ss65303367	*rs43392060*	4	43.962823	0.5315	0.3403	0.559868	0.0110	0.0054831881
ss61489806	*rs41586924*	4	44.001398	0.5129	0.1456	0.702928	0.0071	0.0035383038
ss86293458		4	44.086205	0.4506	0.0000	0.996122	0.0001	0.0000456653
ss86329973		4	44.193866	0.0931	1.1374	0.286580	0.0333	0.0056219919
ss65302495	*rs43391289*	4	44.351747	0.5451	0.0204	0.886495	0.0027	0.0013201935
	rs29010454	4	44.442125	0.0917	3.7360	0.053657	0.0625	0.0104097417
ss61554097	*rs41650644*	4	44.481811	0.2593	0.0186	0.891660	0.0029	0.0011128260
ss61472086	*rs41566326*	4	44.657517	0.5638	5.8836	0.015536	0.0455	0.0223654912
ss86341374		4	44.721099	0.4964	3.3749	0.066622	0.0343	0.0171483331
ss63774160	*rs42342755*	4	44.777465	0.3797	0.3236	0.569657	0.0111	0.0052299642
ss86335486		4	44.80747	0.3646	1.1120	0.292024	0.0206	0.0095331834
ss86337784		4	44.836921	0.6347	0.0001	0.992223	0.0002	0.0000883360
ss86341587		4	44.896412	0.3324	0.7363	0.391156	0.0168	0.0074552394
ss61515693	*rs41616643*	4	44.925214	0.9054	0.4923	0.483155	0.0221	0.0037857244
ss117967325		4	44.96986	0.3059	1.6526	0.199036	0.0256	0.0108863385
ss86320954		4	45.079735	0.5838	0.9189	0.338089	0.0186	0.0090147406
	rs29025758	4	45.13283	0.6934	1.6540	0.198845	0.0257	0.0109092366
	rs29027881	4	45.229418	0.7471	0.1343	0.714107	0.0080	0.0030381971
ss117967300		4	45.285053	0.4463	1.3238	0.250303	0.0214	0.0105612937
ss86294751		4	45.513144	0.5645	1.6538	0.198866	0.0246	0.0120737487
ss86307680		4	45.548221	0.1619	2.6476	0.104158	0.0427	0.0115988972
ss86338786		4	45.594744	0.6125	14.1419	0.000184	0.0709	0.0336546427
ss86319543		4	45.674441	0.3940	12.7335	0.000384	0.0679	0.0324318996
ss61554167	*rs41650714*	4	45.737962	0.3181	9.1554	0.002571	0.0605	0.0262367577
ss86323931		4	45.848456	0.8130	13.7337	0.000227	0.0869	0.0264227163
ss65305678	*rs43389354*	4	45.884388	0.4699	2.4170	0.120478	0.0289	0.0144181518
ss64670823	*rs42765191*	4	45.920772	0.5544	0.4746	0.491114	0.0132	0.0064978324
ss64670859	*rs42765227*	4	45.948968	0.3474	7.1086	0.007850	0.0517	0.0234432150
ss86329859		4	45.991115	0.4491	7.4308	0.006573	0.0512	0.0253465094
ss86288336		4	46.026181	0.5229	1.4387	0.230755	0.0224	0.0111884777
ss64739611	*rs42836039*	4	46.050088	0.4026	3.4281	0.064519	0.0356	0.0171403454
ss64739588	*rs42834916*	4	46.072693	0.6168	2.2711	0.132264	0.0295	0.0139472576
ss65306232	*rs43393207*	4	46.235011	0.4520	0.8984	0.343542	0.0179	0.0088821239
ss86304999		4	46.282765	0.7034	12.8173	0.000367	0.0720	0.0300273839
ss61554178	*rs41650725*	4	46.361233	0.3668	8.7766	0.003155	0.0557	0.0258679455
ss61554180	*rs41650727*	4	46.392809	0.3682	9.3364	0.002332	0.0575	0.0267455131
ss61489834	*rs41587652*	4	46.440724	0.6354	9.0609	0.002706	0.0573	0.0265474324
ss86292540		4	46.474987	0.2966	12.8173	0.000367	0.0720	0.0300273839
ss65305734	*rs43389410*	4	46.503608	0.6246	0.5074	0.476525	0.0135	0.0063280832
ss86286671		4	46.535844	0.2285	0.4065	0.523969	0.0144	0.0050734860
ss64058417	*rs42454653*	4	46.560259	0.8453	13.4297	0.000267	0.0919	0.0240296856
ss86305597		4	46.584751	0.1848	1.3678	0.242598	0.0280	0.0084400797
ss64058375	*rs42454632*	4	46.608973	0.2027	1.6951	0.193363	0.0298	0.0096324454
ss61477876	*rs41580696*	4	46.632488	0.2844	1.4688	0.225944	0.0253	0.0102864767
ss61529103	*rs41632013*	4	46.661233	0.0917	0.2450	0.620761	0.0159	0.0026509636
ss61536411	*rs41635035*	4	46.86884	0.7063	1.7189	0.190269	0.0269	0.0111688150
ss62178375	*rs41780457*	4	46.894344	0.2106	1.6707	0.196598	0.0293	0.0097507789

	rs29011243	4	46.920709	0.3438	0.6241	0.429793	0.0157	0.0070654064
ss86293217		4	46.986578	0.8338	0.1897	0.663294	0.0109	0.0030313614
ss61519145		4	47.033692	0.2235	3.4182	0.064904	0.0399	0.0138566931
ss86320946		4	47.217392	0.2765	0.8454	0.358189	0.0193	0.0077405375
ss64524030	*rs42685998*	4	47.316528	0.3668	2.3334	0.127078	0.0296	0.0137684331
	rs29019556	4	47.395263	0.2557	1.3989	0.237303	0.0257	0.0097735573
ss64100249	*rs42472777*	4	47.494072	0.4277	4.6428	0.031526	0.0398	0.0194894940
ss64100217	*rs42472761*	4	47.51596	0.4298	3.5835	0.058772	0.0350	0.0171611923
ss64100169	*rs42472737*	4	47.537655	0.6440	1.9745	0.160412	0.0271	0.0124116399
ss86308284		4	47.600874	0.3789	5.1545	0.023491	0.0427	0.0200981701
ss86279723		4	47.627222	0.4577	2.8419	0.092283	0.0310	0.0153992482
ss86310355		4	47.663284	0.5451	9.8416	0.001778	0.0585	0.0290017818
ss86300947		4	47.698509	0.5451	9.8416	0.001778	0.0585	0.0290017818
ss86274221		4	47.739621	0.3403	1.0407	0.308009	0.0206	0.0092396407
ss61515707	*rs41616657*	4	47.794576	0.2894	3.2440	0.072119	0.0374	0.0153803907
ss86333658		4	47.850185	0.6418	5.8405	0.015918	0.0468	0.0215202537
ss86306651		4	47.944011	0.9413	5.5658	0.018590	0.0937	0.0103559940
ss86336396		4	48.003524	0.0953	0.5592	0.454832	0.0237	0.0040894737
ss61554177	*rs41650724*	4	48.026806	0.5881	6.9587	0.008528	0.0504	0.0243987050
ss65306571	*rs43395610*	4	48.069816	0.9319	1.1128	0.291839	0.0383	0.0048608885
ss65306583	*rs43395622*	4	48.108047	0.1060	5.3492	0.021022	0.0665	0.0126107455
ss65306664	*rs43388503*	4	48.228823	0.9176	6.1977	0.013024	0.0802	0.0121179607
ss65307146	*rs43393384*	4	48.306083	0.0989	0.1379	0.710485	0.0115	0.0020564991
ss61489860	*rs41587674*	4	48.479945	0.6533	9.5620	0.002066	0.0602	0.0272552215
ss61489857	*rs41587671*	4	48.535409	0.7163	4.6318	0.031728	0.0426	0.0173218919
ss86334864		4	48.626059	0.3481	7.4536	0.006491	0.0514	0.0233410566
ss86286518		4	48.655362	0.8274	0.3109	0.577286	0.0140	0.0040001395
ss86306000		4	48.781574	0.5129	5.6348	0.017878	0.0445	0.0222223938
ss117967527		4	48.812128	0.1819	0.0188	0.890864	0.0033	0.0009921919
ss86321900		4	48.839442	0.5516	2.6702	0.102696	0.0310	0.0153124847
ss86282718		4	48.860502	0.5602	9.6296	0.001992	0.0584	0.0287863792
ss65307795	*rs43390531*	4	48.906886	0.6519	1.5958	0.206919	0.0252	0.0114431552
ss86328739		4	48.969928	0.7987	0.3221	0.570526	0.0127	0.0040933185
ss86333174		4	48.998894	0.0709	3.6196	0.057514	0.0656	0.0086407248
ss86290215		4	49.036517	0.3696	12.0045	0.000563	0.0643	0.0299702483
ss61489864	*rs41587678*	4	49.069016	0.7371	1.1909	0.275516	0.0227	0.0088083305
ss65307581	*rs43398415*	4	49.109713	0.6304	11.8564	0.000609	0.0641	0.0298738664
ss61510247	*rs41602690*	4	49.147498	0.3682	12.2900	0.000485	0.0651	0.0303106678
ss117967341		4	49.222871	0.1483	0.4397	0.507483	0.0171	0.0043235896
ss61554221	*rs41651168*	4	49.265709	0.8517	1.0638	0.302703	0.0272	0.0068826677
ss86305544		4	49.342925	0.4542	2.5830	0.108468	0.0294	0.0145899380
ss86326678		4	49.383226	0.5681	0.1368	0.711566	0.0068	0.0033549848
ss64669636	*rs42763207*	4	49.466353	0.1956	6.9955	0.008356	0.0627	0.0197368978
ss61472947	*rs41575166*	4	49.490028	0.1383	11.5882	0.000702	0.0892	0.0212492334
ss86303537		4	49.515299	0.8632	12.9143	0.000349	0.0943	0.0222765722
ss65307155	*rs43393393*	4	49.554897	0.5344	9.2110	0.002495	0.0554	0.0275621895
ss86316542		4	49.598121	0.2471	5.4680	0.019650	0.0499	0.0185750378

ss86322295		4	49.620614	0.1354	10.1403	0.001515	0.0834	0.0195302645
ss64655755	*rs42756348*	4	49.674072	0.2307	5.0694	0.024663	0.0487	0.0172818936
ss117975221		4	49.69992	0.5530	1.0098	0.315291	0.0186	0.0091821299
ss117962561		4	49.969868	0.9198	0.0001	0.991850	0.0003	0.0000504220
ss61554153	*rs41650700*	4	50.062727	0.4341	4.8003	0.028785	0.0403	0.0197985766
ss65304399	*rs43390276*	4	50.085486	0.7063	10.1198	0.001532	0.0630	0.0261469267
ss65304372	*rs43390259*	4	50.177284	0.2937	10.1198	0.001532	0.0630	0.0261469267
ss61522830		4	50.201207	0.9040	0.3829	0.536232	0.0196	0.0033937318
ss65304445	*rs43390322*	4	50.269153	0.4764	4.5202	0.033848	0.0393	0.0195977703
ss86336292		4	50.331898	0.4585	3.9029	0.048597	0.0367	0.0182446991
ss117967307		4	50.36066	0.7049	3.5633	0.059485	0.0384	0.0159919912
ss61554156	*rs41650703*	4	50.394779	0.2586	3.4272	0.064554	0.0401	0.0153622877
ss65304798	*rs43390376*	4	50.433561	0.2600	2.9988	0.083770	0.0375	0.0144270118
ss61554157	*rs41650704*	4	50.471395	0.7393	2.8642	0.091017	0.0366	0.0141285323
ss61554158	*rs41650705*	4	50.520683	0.1232	3.1822	0.074882	0.0511	0.0110416993
ss65304871	*rs43391149*	4	50.564772	0.7529	5.0456	0.025002	0.0470	0.0174855761
ss86311981		4	50.615977	0.6497	0.1965	0.657661	0.0086	0.0039227146
ss86285182		4	50.659241	0.2271	0.7540	0.385518	0.0194	0.0068213706
ss65305085	*rs43393062*	4	50.690605	0.1024	1.6593	0.198130	0.0386	0.0070925581
ss61554163	*rs41650710*	4	50.722265	0.1347	0.3468	0.556139	0.0163	0.0037914653
ss86308972		4	50.755926	0.8173	1.6556	0.198625	0.0306	0.0091287751
ss117967546		4	50.78136	0.4563	3.3863	0.066165	0.0338	0.0167858518
ss86308063		4	50.819539	0.9248	0.4660	0.495076	0.0240	0.0033431544
ss65305240	*rs43394317*	4	50.879948	0.2342	5.7916	0.016363	0.0501	0.0179720619
ss61554142	*rs41650689*	4	50.974271	0.1053	0.3539	0.552106	0.0175	0.0033024203
ss61554137	*rs41650684*	4	51.018248	0.8997	0.4940	0.482382	0.0212	0.0038208930
ss65304234	*rs43388824*	4	51.062861	0.8102	1.2874	0.256921	0.0268	0.0082437001
	rs29022883	4	51.115713	0.6060	0.0000	0.996857	0.0001	0.0000357229
ss86291105		4	51.237628	0.3990	6.9118	0.008752	0.0486	0.0232856626
ss117963771		4	51.278418	0.4069	6.8999	0.008810	0.0502	0.0242215857
ss86333536		4	51.360445	0.3037	0.4767	0.490130	0.0138	0.0058263848
ss61554115	*rs41650662*	4	51.436766	0.8768	4.3691	0.036959	0.0598	0.0129225226
ss61554123	*rs41650670*	4	51.482799	0.8883	5.2546	0.022187	0.0686	0.0136260417
ss86305883		4	51.503654	0.9341	6.5411	0.010752	0.0954	0.0117448456
ss86284048		4	51.54478	0.7436	14.2066	0.000178	0.0785	0.0299339412
ss64686891	*rs42783161*	4	51.568182	0.2163	15.3907	0.000096	0.0881	0.0298837617
ss117963756		4	51.650637	0.3431	17.1417	0.000039	0.0800	0.0360503399
ss61554127	*rs41650674*	4	51.699053	0.0437	5.0411	0.025066	0.1010	0.0084396333
ss65304061	*rs43387851*	4	51.743795	0.7457	3.2306	0.072710	0.0382	0.0144764112
ss38323343	*rs29012256*	4	51.77201	0.8940	10.0194	0.001617	0.0945	0.0179163462
ss86336790		4	51.830926	0.6927	6.5389	0.010765	0.0510	0.0217129951
ss86325958		4	51.859954	0.6913	6.5969	0.010423	0.0513	0.0218813308
ss86338537		4	51.906707	0.6956	5.6886	0.017343	0.0477	0.0201999153
ss86339561		4	52.038346	0.0738	3.6093	0.057869	0.0665	0.0090891132
ss61525304	*rs41620331*	4	52.060196	0.6074	14.6104	0.000144	0.0697	0.0332274191
ss61512698	*rs41614500*	4	52.240753	0.4828	10.6848	0.001133	0.0597	0.0298339187
ss86338251		4	52.271977	0.5745	0.5290	0.467279	0.0137	0.0067208414

ss86282886		4	52.339635	0.7822	1.2707	0.260033	0.0253	0.0086167471
ss86339366		4	52.374699	0.2034	2.9389	0.086914	0.0392	0.0127071016
ss63973010	*rs42413833*	4	52.406905	0.6619	6.8841	0.008887	0.0498	0.0222883145
	rs29013481	4	52.437364	0.7135	2.8770	0.090301	0.0334	0.0136385210
ss61529925	*rs41629426*	4	52.677782	0.1211	7.7794	0.005429	0.0800	0.0170323882
ss86311921		4	52.798744	0.6110	6.6279	0.010245	0.0483	0.0229592510
	rs29009770	4	52.897684	0.8682	10.4826	0.001262	0.0855	0.0195785854
	rs29025355	4	53.136032	0.3861	3.4200	0.064836	0.0356	0.0168898090
ss86339981		4	53.206872	0.4090	6.3873	0.011715	0.0478	0.0231326514
ss86336203		4	53.236897	0.3431	11.7407	0.000647	0.0665	0.0299561663
ss61503767	*rs41599758*	4	53.310385	0.1648	2.2244	0.136295	0.0374	0.0102916745
ss65309387	*rs43399120*	4	53.420826	0.4212	1.7810	0.182465	0.0254	0.0123759473
ss65308667	*rs43390704*	4	53.465567	0.9549	3.4010	0.065581	0.0821	0.0070729893
ss61554257	*rs41651204*	4	53.492252	0.1705	0.4285	0.512920	0.0161	0.0045399415
ss65308768	*rs43391805*	4	53.613165	0.8775	0.6851	0.408119	0.0233	0.0050115754
ss86287884		4	53.654311	0.5702	2.8284	0.093060	0.0317	0.0155376837
ss65309435	*rs43400068*	4	53.687143	0.0458	3.3139	0.069125	0.0805	0.0070395929
ss117963743		4	53.778229	0.8317	0.2010	0.654065	0.0112	0.0031408803
ss65309642	*rs43390779*	4	53.824363	0.9248	2.7324	0.098785	0.0582	0.0080996642
ss61554292	*rs41651938*	4	53.871898	0.6834	0.6403	0.423879	0.0164	0.0070769267
ss61492851	*rs41590599*	4	53.947199	0.9534	2.6378	0.104798	0.0713	0.0063349335
	rs29020726	4	53.972253	0.1812	2.8226	0.093393	0.0411	0.0122081938
ss86296845		4	54.054895	0.0616	0.0002	0.989275	0.0005	0.0000599380
ss65489611	*rs43568791*	4	54.098586	0.1375	2.0981	0.147931	0.0389	0.0092273067
ss61492850	*rs41590598*	4	54.139539	0.4971	10.0209	0.001615	0.0581	0.0290690114
ss61492849	*rs41590597*	4	54.168989	0.7650	0.0665	0.796621	0.0058	0.0020868209
ss61477449	*rs41576784*	4	54.249891	0.8639	0.2647	0.607100	0.0141	0.0033110029
ss86308501		4	54.419865	0.5509	9.5057	0.002129	0.0575	0.0284562181
ss64584076	*rs42712132*	4	54.477142	0.8961	0.1066	0.744135	0.0097	0.0018006810
ss64583956	*rs42711272*	4	54.512468	0.4520	6.1635	0.013276	0.0465	0.0230508614
ss61531443	*rs41626104*	4	54.560109	0.2586	20.7320	0.000006	0.0952	0.0364976969
ss64583596	*rs42709692*	4	54.592194	0.7479	17.7108	0.000029	0.0894	0.0337287338
ss64548296	*rs42697954*	4	54.63436	0.2622	20.0506	0.000009	0.0937	0.0362445055
ss117967536		4	54.72783	0.2285	1.1647	0.280860	0.0243	0.0085557909
ss86277977		4	54.749802	0.8001	0.0344	0.852828	0.0042	0.0013575266
ss117963717		4	54.775263	0.5938	4.6145	0.032047	0.0408	0.0196895593
ss63413698	*rs42228359*	4	54.900412	0.6447	11.0728	0.000922	0.0632	0.0289346714
ss63413653	*rs42228344*	4	54.932964	0.9076	4.6713	0.031011	0.0678	0.0113721204
ss86337126		4	55.070423	0.2994	5.1976	0.022919	0.0467	0.0195979744
ss63414187	*rs42229722*	4	55.167195	0.3775	7.1248	0.007780	0.0519	0.0243723762
ss63414646	*rs42219675*	4	55.243287	0.5415	11.4558	0.000753	0.0616	0.0305800617
ss86328704		4	55.285711	0.6547	21.8468	0.000004	0.0914	0.0413300220
ss63414790	*rs42219723*	4	55.327035	0.8990	18.3718	0.000021	0.1304	0.0236863054
ss86286174		4	55.36104	0.7292	0.0133	0.908290	0.0025	0.0009715338
ss63415087	*rs42220422*	4	55.450296	0.7192	0.2866	0.592577	0.0110	0.0044276464
ss86287748		4	55.486699	0.4011	0.4063	0.524088	0.0121	0.0058240160
ss61497442	*rs41596869*	4	55.532791	0.3797	1.5306	0.216443	0.0235	0.0110650133

ss61569859	*rs41670410*	4	55.606839	0.4255	23.7482	0.000001	0.0900	0.0439951080
ss63415432	*rs42221637*	4	55.703403	0.8954	0.3447	0.557341	0.0175	0.0032758325
ss86339622		4	55.756267	0.4384	4.9148	0.026949	0.0408	0.0200857233
ss63415648	*rs42221709*	4	55.783684	0.8768	2.5193	0.112916	0.0442	0.0095405995
ss65312352	*rs43399195*	4	55.854031	0.8947	0.6643	0.415334	0.0245	0.0046103826
ss65312465	*rs43400208*	4	55.899133	0.3116	9.0563	0.002712	0.0589	0.0252687251
ss65312508	*rs43401351*	4	55.926205	0.3087	8.1372	0.004465	0.0563	0.0240398159
ss117967424		4	55.953228	0.6977	9.6593	0.001961	0.0619	0.0260965017
ss61554329	*rs41651975*	4	55.987359	0.3954	0.0411	0.839309	0.0038	0.0018000565
ss117967498		4	56.008311	0.6110	0.0249	0.874647	0.0029	0.0013937973
ss65312840	*rs43394984*	4	56.039359	0.8832	0.0402	0.841109	0.0057	0.0011854817
ss86336595		4	56.070023	0.1125	0.0822	0.774452	0.0083	0.0016585925
ss65312929	*rs43396273*	4	56.133572	0.9241	0.1772	0.673894	0.0145	0.0020349258
ss61487401	*rs41583172*	4	56.442232	0.8574	0.0024	0.960874	0.0013	0.0003174077
ss86301290		4	56.574852	0.8589	1.4276	0.232569	0.0319	0.0077228814
ss63995730	*rs42421286*	4	56.74547	0.2887	13.2695	0.000290	0.0729	0.0299457170
ss63995886	*rs42421963*	4	56.819947	0.5673	3.8884	0.049017	0.0367	0.0180202429
ss63995782	*rs42421311*	4	56.870493	0.5466	4.2969	0.038550	0.0384	0.0190209762
ss86322783		4	56.911117	0.2407	13.6623	0.000236	0.0787	0.0287561531
ss63995958	*rs42421999*	4	56.969263	0.1361	0.2152	0.642871	0.0128	0.0030009429
ss61526758	*rs41626341*	4	57.000686	0.7486	0.5848	0.444691	0.0170	0.0063945881
ss61472896	*rs41574615*	4	57.428234	0.2586	0.0887	0.765926	0.0063	0.0024251256
ss63925702	*rs42285110*	4	57.466509	0.1318	4.3082	0.038295	0.0576	0.0131720080
ss62003270	*rs41724453*	4	57.499432	0.5014	1.2947	0.255569	0.0212	0.0106226777
ss86326874		4	57.556457	0.2364	1.0088	0.315529	0.0225	0.0081311226
ss64756290	*rs42851059*	4	57.643033	0.4857	1.5897	0.207788	0.0236	0.0117963208
ss63926056	*rs42286586*	4	57.670904	0.2185	1.3397	0.247480	0.0260	0.0088775581
ss63925938	*rs42286027*	4	57.69797	0.7772	1.8076	0.179237	0.0300	0.0103934174
ss63925996	*rs42286556*	4	57.732291	0.5229	1.2074	0.272232	0.0208	0.0104012307
ss64905741	*rs42998451*	4	57.763417	0.5201	1.5913	0.207563	0.0238	0.0118663526
ss65344795	*rs43431136*	4	57.802264	0.4771	1.2074	0.272232	0.0208	0.0104012307
ss61556627	*rs41656231*	4	57.829783	0.4721	1.3299	0.249223	0.0219	0.0108931159
ss61525165	*rs41626190*	4	57.871128	0.1533	3.9722	0.046648	0.0527	0.0136915373
ss65309888	*rs43391925*	4	57.979098	0.7249	6.0028	0.014528	0.0524	0.0208979814
ss65309939	*rs43392376*	4	58.047684	0.4026	19.9086	0.000009	0.0848	0.0407674350
ss86308572		4	58.137116	0.1146	1.5678	0.210953	0.0370	0.0075185950
ss65310263	*rs43394097*	4	58.422144	0.6626	0.4078	0.523303	0.0122	0.0054387909
ss65310282	*rs43394116*	4	58.459292	0.2765	1.7004	0.192665	0.0258	0.0103334542
	rs29011993	4	58.491656	0.3245	0.5420	0.461873	0.0140	0.0061336727
ss65310484	*rs43395215*	4	58.519198	0.4370	5.2675	0.022024	0.0422	0.0207770495
ss61554313	*rs41651959*	4	58.589812	0.6619	1.9994	0.157807	0.0267	0.0119469414
ss86339690		4	58.611855	0.6168	9.3259	0.002346	0.0584	0.0276300760
ss86340122		4	58.701573	0.2042	2.4715	0.116380	0.0356	0.0115604049
	rs29020566	4	58.779525	0.0817	6.3212	0.012155	0.0837	0.0125526097
ss65310705	*rs43392842*	4	58.814964	0.2586	0.8477	0.357534	0.0187	0.0071550668
ss65310715	*rs43392852*	4	58.84905	0.2550	0.6910	0.406108	0.0168	0.0064021481
ss61489928	*rs41588642*	4	58.961143	0.8231	11.6928	0.000664	0.0823	0.0239758829

	rs29010064	4	58.992523	0.4040	0.0042	0.948485	0.0012	0.0005787295
ss86289401		4	59.049048	0.7092	2.8750	0.090413	0.0352	0.0145158848
ss65050359	*rs43140316*	4	59.106277	0.6074	0.2532	0.614960	0.0093	0.0044269238
ss61564217	*rs41659390*	4	59.133598	0.1168	3.1113	0.078190	0.0514	0.0106112174
ss64204906	*rs42527853*	4	59.159711	0.4792	2.1021	0.147550	0.0268	0.0133908674
ss61564218	*rs41659391*	4	59.197977	0.3309	0.0230	0.879523	0.0031	0.0013611423
ss117967229		4	59.224564	0.2758	1.8708	0.171821	0.0292	0.0116716286
ss86327853		4	59.368969	0.5838	1.5083	0.219821	0.0240	0.0116545529
ss65311823	*rs43394422*	4	59.499436	0.3503	4.1730	0.041449	0.0399	0.0181646683
ss65311836	*rs43395435*	4	59.523021	0.6497	4.1730	0.041449	0.0399	0.0181646683
ss65312019	*rs43396375*	4	59.546601	0.5817	0.5176	0.472117	0.0132	0.0064408497
ss61489951	*rs41588662*	4	59.56854	0.7701	4.9381	0.026591	0.0491	0.0174036763
ss65311876	*rs43395462*	4	59.599409	0.9155	6.4338	0.011414	0.0843	0.0130522565
ss61554321	*rs41651967*	4	59.643372	0.3209	7.6547	0.005813	0.0563	0.0245389161
	rs29018949	4	59.793286	0.4628	8.3266	0.004028	0.0539	0.0268157505
ss86320066		4	59.838418	0.5229	9.4204	0.002229	0.0575	0.0286722556
ss61554322	*rs41651968*	4	59.884867	0.0702	0.9315	0.334817	0.0353	0.0046101094
	rs29014471	4	59.933197	0.1017	2.0500	0.152654	0.0444	0.0081119370
	rs29015972	4	59.971949	0.1139	7.7397	0.005548	0.0802	0.0161964407
ss86340555		4	60.084994	0.0559	0.1623	0.687152	0.0166	0.0017546753
ss64658595	*rs42754848*	4	60.120348	0.4792	0.1194	0.729780	0.0065	0.0032323167
ss61475203	*rs41572690*	4	60.249845	0.5193	17.9580	0.000026	0.0782	0.0390362758
ss63926608	*rs42288762*	4	60.292652	0.7643	0.0230	0.879379	0.0034	0.0012178502
	rs29013265	4	60.33925	0.5509	4.6316	0.031732	0.0398	0.0197119482
ss86341702		4	60.378727	0.6468	3.8564	0.049954	0.0393	0.0179775797
ss86319966		4	60.455507	0.0530	4.1404	0.042249	0.0833	0.0083593186
ss61522692	*rs41621463*	4	60.507102	0.6841	13.2042	0.000300	0.0707	0.0305606114
ss61475197	*rs41572684*	4	60.578223	0.0867	1.6305	0.202067	0.0411	0.0065088013
ss61466263	*rs41566922*	4	60.613548	0.5723	7.4490	0.006508	0.0503	0.0246400647
ss61474979	*rs41574165*	4	60.634717	0.5745	7.2741	0.007165	0.0499	0.0243860600
ss117963714		4	60.74083	0.4126	15.9040	0.000074	0.0749	0.0363048655
ss65313136	*rs43398580*	4	60.761809	0.9090	0.8585	0.354483	0.0299	0.0049401740
ss86338435		4	60.795074	0.8539	0.1589	0.690302	0.0103	0.0025652111
	rs29020557	4	60.81665	0.4011	9.9168	0.001708	0.0588	0.0282284254
ss61489967	*rs41588678*	4	60.854757	0.2894	5.8505	0.015828	0.0492	0.0202245512
ss65312273	*rs43398316*	4	60.891512	0.1168	5.0724	0.024621	0.0650	0.0134081741
ss65313204	*rs43399847*	4	60.946386	0.7600	0.6081	0.435767	0.0173	0.0063106338
ss61489975	*rs41588686*	4	61.01187	0.5867	14.2493	0.000174	0.0699	0.0338903444
ss61554334	*rs41651980*	4	61.042724	0.1791	1.0952	0.295689	0.0252	0.0074065293
ss61489977	*rs41588688*	4	61.087639	0.3087	1.2421	0.265452	0.0226	0.0096647428
ss61554343	*rs41651989*	4	61.164265	0.9456	1.5348	0.215805	0.0509	0.0052439695
ss86341651		4	61.200227	0.3166	7.3050	0.007044	0.0553	0.0239522615
ss65313736	*rs43394779*	4	61.239144	0.1017	0.2672	0.605380	0.0160	0.0029205110
ss65313756	*rs43394799*	4	61.278329	0.8976	0.3620	0.547583	0.0186	0.0034122678
ss65313930	*rs43396673*	4	61.349568	0.7113	9.9863	0.001645	0.0634	0.0260439107
ss65314263	*rs43399506*	4	61.424163	0.0681	0.1663	0.683589	0.0153	0.0019400428
ss86337374		4	61.497125	0.8424	3.1496	0.076382	0.0441	0.0117155056

ss65314331	*rs43400574*	4	61.538616	0.5014	0.0029	0.957382		0.0010	0.0005125853
ss86341745		4	61.594364	0.2235	4.5631	0.033016		0.0469	0.0162829479
ss61489987	*rs41588698*	4	61.624081	0.7915	1.8780	0.171004		0.0312	0.0103008525
ss64811496	*rs42909957*	4	61.717453	0.6132	0.7444	0.388562		0.0166	0.0078840681
ss61554355	*rs41652001*	4	61.823131	0.1139	0.0138	0.906644		0.0034	0.0006902826
ss86337016		4	61.90848	0.1999	0.9314	0.334832		0.0227	0.0072739848
ss86337423		4	61.963421	0.1139	10.7313	0.001106		0.0936	0.0188904401
ss86333423		4	62.003009	0.6626	4.6744	0.030956		0.0420	0.0187793899
ss61527540	*rs41624198*	4	62.027048	0.8173	10.6614	0.001148		0.0792	0.0236519374
ss86337263		4	62.057667	0.2135	7.3412	0.006905		0.0625	0.0209825387
ss86292404		4	62.077692	0.7858	6.9420	0.008607		0.0605	0.0203591295
ss86302586		4	62.171159	0.4112	0.2107	0.646343		0.0087	0.0041890222
ss86301869		4	62.192482	0.2536	1.2985	0.254886		0.0241	0.0091350039
ss86300526		4	62.273225	0.1683	3.2934	0.069988		0.0450	0.0125972378
ss86331253		4	62.298089	0.5595	27.4028	0.000000		0.0969	0.0477458015
ss86341421		4	62.327389	0.2120	7.8558	0.005207		0.0644	0.0215097613
ss61554366	*rs41652012*	4	62.406839	0.6719	3.8149	0.051198		0.0397	0.0175020890
ss86329309		4	62.447046	0.5996	3.4890	0.062197		0.0357	0.0171515371
ss61554375	*rs41652021*	4	62.504575	0.1812	0.0781	0.779929		0.0069	0.0020369232
ss117967286		4	62.536131	0.4355	2.2623	0.133007		0.0287	0.0141140631
ss61554362	*rs41652008*	4	62.623665	0.6175	11.5258	0.000725		0.0639	0.0302085163
ss86305542		4	62.665413	0.3897	17.9254	0.000026		0.0775	0.0368778394
ss86286595		4	62.748129	0.0860	2.0823	0.149463		0.0470	0.0073834172
ss86335907		4	62.76983	0.7285	1.3928	0.238336		0.0251	0.0099364032
ss86276676		4	62.798115	0.0938	2.0839	0.149306		0.0456	0.0077547529
ss86329403		4	62.99818	0.4248	10.8292	0.001050		0.0604	0.0295376807
ss65311326	*rs43396624*	4	63.048433	0.9097	0.6949	0.404796		0.0269	0.0044175638
ss86339466		4	63.097488	0.3603	8.9213	0.002918		0.0565	0.0260337609
ss65311050	*rs43395348*	4	63.157332	0.1705	11.9582	0.000577		0.0854	0.0241504911
ss86299792		4	63.2059	0.1511	13.9145	0.000207		0.0955	0.0245052955
	rs29012953	4	63.235448	0.1562	12.6836	0.000394		0.0905	0.0238601505
ss61510255	*rs41602698*	4	63.259925	0.7930	11.1834	0.000870		0.0750	0.0246193760
ss65310857	*rs43393755*	4	63.290916	0.2063	10.6562	0.001151		0.0736	0.0240889508
ss61490007	*rs41588718*	4	63.317686	0.4069	6.9178	0.008723		0.0494	0.0238568209
ss86340981		4	63.39483	0.2357	16.3443	0.000059		0.0868	0.0312542208
ss86332628		4	63.420604	0.5802	8.4240	0.003820		0.0548	0.0266799810
ss86301343		4	63.446868	0.4284	1.3505	0.245590		0.0222	0.0108901462
ss86330706		4	63.488873	0.7056	6.0906	0.013829		0.0505	0.0209838222
ss86340968		4	63.549818	0.4348	5.6210	0.018018		0.0439	0.0215625324
ss65315850	*rs43398188*	4	63.586217	0.7636	9.6010	0.002023		0.0651	0.0234966579
ss61510262	*rs41602705*	4	63.624837	0.7615	9.8531	0.001767		0.0661	0.0240153812
ss65315637	*rs43402375*	4	63.697916	0.1648	12.0099	0.000562		0.0844	0.0232419114
ss86318362		4	63.721147	0.7665	11.9209	0.000589		0.0727	0.0260243281
ss65018541	*rs43109323*	4	63.747481	0.6010	0.0368	0.847945		0.0035	0.0016995485
ss65315417	*rs43400956*	4	63.774417	0.2371	9.6853	0.001933		0.0654	0.0236442598
ss86323260		4	63.958726	0.4577	0.0310	0.860263		0.0033	0.0016252768
ss65315096	*rs43399236*	4	64.106535	0.1261	0.0091	0.924175		0.0027	0.0005948928

ss65314895	*rs43398038*	4	64.226067	0.1397	3.2554	0.071621	0.0480	0.0115437134
ss65314926	*rs43398069*	4	64.264033	0.6526	0.6867	0.407565	0.0162	0.0073321343
ss86337043		4	64.295179	0.4986	4.3147	0.038150	0.0393	0.0196453416
ss61489993	*rs41588704*	4	64.363335	0.7923	13.3575	0.000277	0.0835	0.0274843026
ss86328008		4	64.414285	0.8202	15.9188	0.000073	0.0946	0.0279102558
ss86307206		4	64.45407	0.7393	9.4559	0.002187	0.0636	0.0245162681
ss65316880	*rs43399802*	4	64.485536	0.8001	15.8465	0.000076	0.0929	0.0297022382
ss86322862		4	64.567621	0.5430	0.1859	0.666446	0.0081	0.0040415220
ss86307631		4	64.587839	0.1017	2.0699	0.150677	0.0438	0.0080127300
ss65317172	*rs43402794*	4	64.644425	0.4914	13.9891	0.000199	0.0672	0.0335876184
ss86316312		4	64.675961	0.8725	6.3998	0.011633	0.0697	0.0155114727
ss65317213	*rs43403435*	4	64.699032	0.3503	1.2058	0.272536	0.0211	0.0096267180
ss65317236	*rs43403458*	4	64.723604	0.5029	0.2032	0.652322	0.0084	0.0042069533
ss38333304	*rs29017015*	4	64.781061	0.4628	20.2786	0.000008	0.0805	0.0400178359
ss65317310	*rs43403530*	4	64.804437	0.6096	5.5306	0.018964	0.0448	0.0213223120
ss86336438		4	64.881395	0.1791	8.7459	0.003208	0.0719	0.0211471687
ss61554449	*rs41652394*	4	65.019171	0.4957	9.6040	0.002020	0.0582	0.0290754227
	rs29026148	4	65.039531	0.3202	8.6018	0.003469	0.0606	0.0263943855
ss61554442	*rs41652387*	4	65.080795	0.7686	11.4767	0.000744	0.0727	0.0258713004
ss61554434	*rs41652379*	4	65.11666	0.8195	6.9684	0.008482	0.0636	0.0188123183
ss61510270	*rs41602713*	4	65.149921	0.6368	1.4799	0.224210	0.0236	0.0109282578
ss86299172		4	65.216583	0.2400	1.2833	0.257681	0.0248	0.0090611597
	rs29019354	4	65.240494	0.1669	10.1000	0.001548	0.0791	0.0219977238
ss61554421	*rs41652366*	4	65.394505	0.3739	0.0121	0.912510	0.0021	0.0009896247
ss117967533		4	65.442852	0.8001	1.4456	0.229640	0.0269	0.0085896149
ss86282525		4	65.514417	0.2615	6.8169	0.009225	0.0560	0.0216131857
ss86312254		4	65.548614	0.1633	0.5963	0.440243	0.0192	0.0052572806
ss61486183	*rs41588581*	4	65.708922	0.0745	0.5254	0.468794	0.0254	0.0034966410
ss86323027		4	65.74566	0.8610	10.2119	0.001458	0.0845	0.0202179064
ss86335931		4	65.766008	0.3481	0.0072	0.932326	0.0017	0.0007575470
ss86335881		4	65.801711	0.5867	2.3040	0.129493	0.0283	0.0137419903
	rs29012129	4	65.830815	0.2529	10.1902	0.001475	0.0664	0.0250834089
ss86314263		4	65.930054	0.7213	0.4233	0.515495	0.0129	0.0052052392
ss61559140	*rs41655686*	4	65.953674	0.7564	0.7165	0.397584	0.0185	0.0068054869
ss61559141	*rs41655687*	4	65.998301	0.2722	1.5934	0.207265	0.0258	0.0102376583
ss61510269	*rs41602712*	4	66.160834	0.7471	0.2356	0.627555	0.0099	0.0037498540
ss61554394		4	66.331327	0.8789	6.4850	0.011093	0.0722	0.0153605749
ss61554392	*rs41652338*	4	66.395197	0.7013	4.1425	0.042197	0.0422	0.0176616530
ss86273827		4	66.460605	0.4441	4.7074	0.030371	0.0406	0.0200586735
ss61468852	*rs41573162*	4	66.495309	0.7830	0.5485	0.459193	0.0168	0.0057001793
ss86299262		4	66.610879	0.7170	0.0089	0.924658	0.0020	0.0007949913
ss61515177	*rs41612837*	4	66.651395	0.7758	0.1166	0.732825	0.0075	0.0025955189
ss61475268	*rs41573753*	4	66.671495	0.2328	0.3634	0.546795	0.0130	0.0046394416
ss86293318		4	66.698069	0.7701	0.2118	0.645530	0.0099	0.0035128521
	rs29015265	4	66.897418	0.2099	6.9453	0.008591	0.0606	0.0201128783
ss117967524		4	67.056697	0.2271	5.2693	0.022002	0.0517	0.0181473097
ss86300848		4	67.1258	0.8940	0.5680	0.451305	0.0225	0.0042721458

ss64052929	*rs42449182*	4	67.305953	0.6447	1.7616	0.184865	0.0256	0.0117197725
ss86335912		4	67.350198	0.3675	0.0002	0.989626	0.0002	0.0001144914
ss86322284		4	67.475296	0.7328	0.0071	0.932925	0.0018	0.0006993782
ss64181721	*rs42512924*	4	67.504021	0.3668	1.6820	0.195089	0.0255	0.0118599460
ss64181565	*rs42512849*	4	67.668287	0.3990	0.0009	0.976376	0.0006	0.0002779200
ss61511271	*rs41610792*	4	67.786346	0.6404	0.0063	0.936720	0.0015	0.0007116003
ss86291802		4	67.92164	0.1934	1.7294	0.188917	0.0313	0.0097519260
ss86337447		4	68.015733	0.9054	3.0700	0.080192	0.0551	0.0094430634
ss86319491		4	68.200163	0.2636	0.0015	0.969407	0.0008	0.0003164373
ss86276710		4	68.229055	0.7006	1.3601	0.243922	0.0232	0.0097190851
ss86301412		4	68.254433	0.3309	1.1266	0.288870	0.0204	0.0090317011
ss86286700		4	68.292929	0.6655	0.9797	0.322605	0.0189	0.0084134911
ss64219834	*rs42531861*	4	68.37972	0.6340	7.4660	0.006447	0.0516	0.0239402772
ss86337253		4	68.402618	0.4162	0.0960	0.756729	0.0060	0.0029010633
ss61466555	*rs41570308*	4	68.579641	0.4377	4.6969	0.030555	0.0408	0.0200796107
ss86282006		4	68.625841	0.0645	11.2486	0.000840	0.1229	0.0148300514
ss65317901	*rs43400520*	4	68.649908	0.4735	0.3704	0.542996	0.0116	0.0057849889
ss61490024	*rs41589535*	4	68.672195	0.9348	11.1743	0.000874	0.1220	0.0148711086
ss61490023	*rs41589534*	4	68.70153	0.3983	16.4170	0.000057	0.0776	0.0372040187
ss65317862	*rs43400481*	4	68.724828	0.6053	15.3862	0.000096	0.0749	0.0357869900
ss86308687		4	68.807474	0.2593	0.1510	0.697689	0.0084	0.0032170650
	rs29018051	4	68.833565	0.5602	12.0387	0.000553	0.0636	0.0313596789
ss65317951	*rs43401470*	4	68.857254	0.4198	17.5469	0.000032	0.0783	0.0381416638
ss65318066	*rs43402085*	4	68.924277	0.4685	0.9434	0.331753	0.0187	0.0093331302
ss61510274	*rs41602717*	4	68.971014	0.1110	10.0849	0.001561	0.0942	0.0185941131
ss86317199		4	69.006921	0.4620	6.6559	0.010086	0.0470	0.0233815504
ss61468860	*rs41573170*	4	69.05669	0.0895	5.5586	0.018666	0.0767	0.0125079997
ss86298817		4	69.106423	0.2607	18.2821	0.000022	0.0901	0.0347437661
ss46526668	*rs41257123*	4	69.130299	0.3453	1.1385	0.286345	0.0203	0.0091833474
ss61518465	*rs41614222*	4	69.210306	0.5408	6.9498	0.008570	0.0500	0.0248192288
ss86275517		4	69.254808	0.3560	0.1673	0.682613	0.0081	0.0037068603
ss86324882		4	69.283083	0.7987	0.4882	0.484969	0.0161	0.0051725330
ss61554491	*rs41652933*	4	69.354038	0.5358	8.5587	0.003551	0.0557	0.0277089184
ss61468870	*rs41573180*	4	69.390801	0.5688	2.1494	0.143078	0.0281	0.0138014518
ss65318457	*rs43404775*	4	69.459108	0.2830	3.6971	0.054915	0.0398	0.0161458645
ss61554483	*rs41652427*	4	69.4974	0.2808	2.6526	0.103831	0.0337	0.0136109210
ss86304800		4	69.590743	0.3983	1.3921	0.238448	0.0226	0.0108518223
ss86338112		4	69.656393	0.4097	2.5457	0.111049	0.0292	0.0141095487
ss86337864		4	69.788545	0.0387	9.4305	0.002217	0.1451	0.0107890330
ss65319193	*rs43403912*	4	69.828238	0.9434	1.2227	0.269209	0.0425	0.0045389064
ss61510277	*rs41602720*	4	70.054608	0.6691	7.1997	0.007465	0.0538	0.0238466874
ss61554497	*rs41652939*	4	70.080905	0.7493	1.9083	0.167597	0.0299	0.0112163778
ss86287925		4	70.103843	0.2658	9.4489	0.002195	0.0653	0.0254784722
ss86309272		4	70.161883	0.2371	2.3833	0.123089	0.0338	0.0122113207
ss117967173		4	70.284483	0.2658	3.1841	0.074795	0.0378	0.0147572701
ss61570559	*rs41668916*	4	70.317739	0.3044	1.7031	0.192313	0.0268	0.0113588939
ss65318941	*rs43401862*	4	70.344519	0.2421	1.7862	0.181828	0.0291	0.0106783461

ss65318847	*rs43400768*	4	70.423158	0.6554	0.5069	0.476714	0.0141	0.0063666851
ss61490036	*rs41589547*	4	70.474469	0.2536	5.7565	0.016689	0.0512	0.0193808688
ss86337180		4	70.55707	0.2486	1.3175	0.251440	0.0240	0.0089754013
ss61503790	*rs41599781*	4	70.595159	0.1870	14.8937	0.000124	0.0905	0.0275011460
ss117967541		4	70.809635	0.2915	0.0236	0.877929	0.0032	0.0013127444
ss61523595	*rs41622258*	4	70.859875	0.1433	1.5765	0.209686	0.0327	0.0080286160
ss86288452		4	70.880473	0.2507	12.2386	0.000498	0.0722	0.0271184716
ss61554518	*rs41652960*	4	71.052833	0.5996	1.6638	0.197515	0.0248	0.0118878670
ss117963769		4	71.133507	0.8961	5.6478	0.017747	0.0743	0.0138261248
ss61468872	*rs41573182*	4	71.16291	0.2163	3.8182	0.051099	0.0429	0.0145357275
ss61554519	*rs41652961*	4	71.222035	0.6891	0.3136	0.575632	0.0113	0.0048392375
ss61554523	*rs41652965*	4	71.289063	0.2751	0.8437	0.358673	0.0191	0.0076193622
ss61554525	*rs41652967*	4	71.31827	0.6891	0.2546	0.614001	0.0102	0.0043889529
ss86300269		4	71.401612	0.9069	1.1305	0.288037	0.0343	0.0057977963
ss86298557		4	71.437271	0.4499	6.8267	0.009174	0.0489	0.0241946776
ss86328349		4	71.540167	0.6461	0.8502	0.356811	0.0180	0.0082315169
ss86307951		4	71.668855	0.4491	7.0251	0.008220	0.0495	0.0245033602
ss86290929		4	71.709641	0.5229	9.2623	0.002427	0.0572	0.0285374483
ss86296136		4	71.778599	0.6999	0.4389	0.507851	0.0132	0.0055404967
ss86298593		4	71.817734	0.2006	0.1474	0.701174	0.0091	0.0029107367
ss61466361	*rs41568116*	4	72.057915	0.6698	14.6651	0.000140	0.0765	0.0338393291
ss86324880		4	72.105102	0.3718	0.0960	0.756722	0.0061	0.0028283028
ss61554541	*rs41652983*	4	72.339506	0.7493	11.3487	0.000797	0.0731	0.0274628114
ss86319055		4	72.359812	0.2070	11.7038	0.000660	0.0782	0.0256904211
ss86290144		4	72.533279	0.7923	12.2525	0.000494	0.0797	0.0262185853
ss61465800	*rs41569944*	4	72.561113	0.2085	12.4314	0.000450	0.0802	0.0264622286
ss86340079		4	72.605981	0.2085	12.4314	0.000450	0.0802	0.0264622286
ss86335829		4	72.654441	0.2092	12.2378	0.000498	0.0795	0.0263153208
ss86281977		4	72.674904	0.2393	3.6277	0.057238	0.0413	0.0150339836
ss61472645	*rs41572868*	4	72.707331	0.2457	2.9209	0.087886	0.0366	0.0135801092
ss86300369		4	72.737826	0.5638	1.7786	0.182752	0.0250	0.0122911720
ss61474121	*rs41567333*	4	72.802814	0.2901	1.1767	0.278414	0.0223	0.0091728233
ss86337772		4	72.831517	0.4355	2.0222	0.155461	0.0265	0.0130446293
ss61516853	*rs41620153*	4	72.939017	0.8782	5.4010	0.020412	0.0645	0.0137969931
ss61554552	*rs41652994*	4	72.967515	0.1805	11.9147	0.000591	0.0824	0.0243919505
ss86327760		4	72.988438	0.4341	2.4312	0.119400	0.0292	0.0143451592
ss86281666		4	73.017373	0.5673	2.8378	0.092518	0.0316	0.0154964727
ss86335702		4	73.177472	0.4327	2.8378	0.092518	0.0316	0.0154964727
ss86332919		4	73.204731	0.4298	3.0398	0.081686	0.0325	0.0159377191
ss86312371		4	73.230724	0.5666	3.0451	0.081420	0.0327	0.0160707536
ss86311899		4	73.33563	0.5666	2.3961	0.122091	0.0292	0.0143494498
ss61554553	*rs41652995*	4	73.37736	0.1676	14.4895	0.000153	0.0938	0.0261806249
ss61554558	*rs41653000*	4	73.406984	0.1676	14.4895	0.000153	0.0938	0.0261806249
ss86326232		4	73.435165	0.1884	16.9045	0.000044	0.0958	0.0292877765
ss86336441		4	73.504676	0.3001	0.5357	0.464482	0.0150	0.0062884893
ss86294065		4	73.537568	0.5666	2.3961	0.122091	0.0292	0.0143494498
ss61569723	*rs41669587*	4	73.717947	0.4549	9.1587	0.002567	0.0549	0.0272408916

ss86307865		4	73.899394	0.0659	2.7535	0.097494		0.0621	0.0076489216
ss117967530		4	74.071827	0.9062	0.7282	0.393776		0.0268	0.0045498485
ss86278785		4	74.277809	0.7658	0.5162	0.472700		0.0156	0.0055852094
ss86284614		4	74.330857	0.9090	0.3279	0.567068		0.0183	0.0030232131
ss86318119		4	74.579818	0.6082	4.0082	0.045668		0.0373	0.0177560454
ss61468894	*rs41573204*	4	74.616159	0.7271	0.0134	0.907917		0.0024	0.0009427534
ss61554621	*rs41653662*	4	74.706825	0.2085	2.4295	0.119526		0.0359	0.0118326006
ss61517774	*rs41619037*	4	74.926529	0.5566	3.8306	0.050723		0.0357	0.0176136315
ss61473375	*rs41569794*	4	74.993513	0.2600	0.0207	0.885752		0.0030	0.0011718267
ss64589450	*rs42709546*	4	75.030569	0.1691	2.3686	0.124256		0.0382	0.0107358866
ss86297652		4	75.235595	0.8883	1.6564	0.198516		0.0383	0.0076013049
ss65320195	*rs43405212*	4	75.267905	0.8152	0.6756	0.411382		0.0189	0.0057067225
ss86327193		4	75.296103	0.2514	0.2701	0.603448		0.0108	0.0040642588
ss86293277		4	75.528074	0.6590	2.9641	0.085575		0.0338	0.0151882693
ss61554630	*rs41653671*	4	75.559739	0.6619	3.4570	0.063407		0.0366	0.0164019341
ss61554636	*rs41653677*	4	75.592733	0.9506	0.1267	0.722000		0.0148	0.0013887230
ss65320665	*rs43410782*	4	75.662981	0.0666	0.1792	0.672155		0.0155	0.0019226120
ss65320770	*rs43403587*	4	75.693605	0.3130	0.0144	0.904402		0.0024	0.0010312571
ss86322335		4	75.723903	0.1683	0.5666	0.451869		0.0191	0.0053509349
ss61490125	*rs41590135*	4	75.751872	0.1855	1.2674	0.260647		0.0269	0.0081392809
ss65320965	*rs43404682*	4	75.855239	0.6655	0.2075	0.648853		0.0090	0.0040234829
ss61519966	*rs41620290*	4	75.888361	0.4205	13.2933	0.000286		0.0646	0.0314855675
ss64726870	*rs42824767*	4	75.924111	0.4312	13.1701	0.000305		0.0640	0.0314001344
ss86301260		4	75.990384	0.6404	10.8036	0.001064		0.0611	0.0281463801
ss64981018	*rs43072408*	4	76.02419	0.2271	0.3477	0.555620		0.0129	0.0045219131
ss86305527		4	76.143526	0.5466	8.3313	0.004017		0.0504	0.0249855643
ss65059270	*rs43147629*	4	76.191527	0.7414	20.5533	0.000007		0.0911	0.0349462796
ss64971951	*rs43061867*	4	76.215201	0.1318	6.0066	0.014498		0.0672	0.0153893987
ss64053667	*rs42450575*	4	76.239482	0.2092	15.1970	0.000106		0.0865	0.0286159878
ss64053815	*rs42450949*	4	76.289025	0.8037	1.4788	0.224380		0.0280	0.0088494334
ss61476396	*rs41575957*	4	76.325082	0.8582	5.0549	0.024868		0.0596	0.0145163003
ss64682013	*rs42777412*	4	76.361156	0.8617	4.4555	0.035145		0.0567	0.0135158649
ss86316983		4	76.406191	0.5573	12.9775	0.000338		0.0626	0.0309108811
ss86335526		4	76.456385	0.3926	5.4145	0.020256		0.0420	0.0200405900
ss117967371		4	76.555718	0.7027	1.0602	0.303525		0.0203	0.0084608971
ss65321430	*rs43407344*	4	76.668416	0.4105	8.6263	0.003423		0.0527	0.0254853919
ss64508820	*rs42669983*	4	76.748641	0.2794	0.0823	0.774328		0.0060	0.0024123110
ss86285771		4	76.781834	0.3331	3.5491	0.059994		0.0360	0.0159970982
ss61519626	*rs41616873*	4	76.867443	0.4484	2.4668	0.116732		0.0297	0.0146911615
ss86298594		4	76.907118	0.5358	1.8755	0.171286		0.0255	0.0126649561
ss61554677	*rs41653717*	4	77.139025	0.0702	0.1773	0.673821		0.0154	0.0020120957
ss61554676	*rs41653716*	4	77.186363	0.7206	2.6702	0.102696		0.0347	0.0139544321
ss86291738		4	77.236149	0.6468	1.4234	0.233256		0.0232	0.0105946265
ss86334590		4	77.257506	0.7586	0.5846	0.444783		0.0166	0.0060913227
ss86327771		4	77.278307	0.6003	2.1857	0.139753		0.0271	0.0129904730
ss86315514		4	77.304619	0.1719	0.2226	0.637250		0.0115	0.0032760552
ss65322249	*rs43408665*	4	77.364698	0.5193	0.1281	0.720512		0.0065	0.0032547572

ss86301939		4	77.391948	0.2679	2.1787	0.140383	0.0309	0.0121288563
ss86284349		4	77.472828	0.2120	1.4945	0.221939	0.0268	0.0089532005
ss65322396	*rs43409212*	4	77.499623	0.7307	0.0830	0.773388	0.0060	0.0023427038
ss86286159		4	77.52439	0.9549	1.3187	0.251219	0.0502	0.0043253968
ss61554700	*rs41654140*	4	77.544669	0.5867	5.6373	0.017852	0.0444	0.0215518611
ss86336486		4	77.565085	0.5265	6.3019	0.012287	0.0459	0.0229070222
ss65322594	*rs43411110*	4	77.626018	0.8532	8.1529	0.004427	0.0762	0.0191054286
ss86307562		4	77.750165	0.5774	6.8211	0.009203	0.0470	0.0229257474
ss86291547		4	77.858118	0.3367	6.4184	0.011513	0.0501	0.0223565552
ss61490154	*rs41590164*	4	77.882136	0.8539	7.6985	0.005675	0.0742	0.0185202689
ss65322848	*rs43406164*	4	77.965794	0.5630	9.3560	0.002308	0.0550	0.0270422428
ss61554709	*rs41654149*	4	78.000947	0.2450	0.5326	0.465782	0.0156	0.0057602371
ss65102686	*rs43190028*	4	78.03277	0.7615	0.2470	0.619374	0.0105	0.0038274164
ss65322792	*rs43404908*	4	78.161177	0.6383	12.2143	0.000504	0.0625	0.0288727482
ss61490167	*rs41590177*	4	78.222616	0.6289	12.7114	0.000388	0.0634	0.0296108095
ss86306344		4	78.246158	0.9090	1.0763	0.299898	0.0332	0.0054852653
ss86306584		4	78.273342	0.1397	2.1354	0.144388	0.0389	0.0093587750
ss86337182		4	78.297192	0.1597	4.6252	0.031849	0.0554	0.0148814707
ss86332387		4	78.358392	0.3926	9.7989	0.001819	0.0571	0.0272256285
ss65323327	*rs43410242*	4	78.398861	0.3360	14.4668	0.000155	0.0695	0.0310106896
ss65049929	*rs43138386*	4	78.458231	0.3152	13.2015	0.000300	0.0679	0.0293201624
ss86317881		4	78.515219	0.6268	8.3417	0.003995	0.0524	0.0244996604
ss61490170	*rs41590180*	4	78.567049	0.6841	13.2692	0.000290	0.0681	0.0294429798
ss61490177	*rs41590187*	4	78.634248	0.6246	9.0209	0.002765	0.0547	0.0256411362
ss86283171		4	78.655427	0.3202	13.4330	0.000266	0.0685	0.0298129748
ss86338457		4	78.716287	0.6999	0.0094	0.922656	0.0020	0.0008209884
ss86340417		4	78.754854	0.8102	0.8504	0.356762	0.0214	0.0065702251
ss65323859	*rs43406674*	4	78.791314	0.3195	13.2377	0.000295	0.0680	0.0295574599
ss117967189		4	78.832662	0.5931	10.8468	0.001040	0.0607	0.0292961561
ss86308830		4	78.918962	0.6289	4.5501	0.033266	0.0399	0.0186159534
ss86337583		4	79.015764	0.8818	1.2527	0.263431	0.0328	0.0068299383
ss86292026		4	79.080649	0.6540	5.1287	0.023840	0.0415	0.0187661439
ss86304448		4	79.145416	0.1576	9.5759	0.002051	0.0770	0.0204493695
ss86315148		4	79.176999	0.3503	2.0969	0.148052	0.0282	0.0128273029
ss86309238		4	79.21761	0.5552	4.9467	0.026460	0.0409	0.0201851482
ss117967500		4	79.31665	0.3976	0.5184	0.471787	0.0134	0.0064109680
ss86318290		4	79.349125	0.0723	0.4082	0.523104	0.0226	0.0030360476
ss86329250		4	79.527531	0.2450	4.8800	0.027494	0.0454	0.0168106181
ss46525999	*rs41255147*	4	79.623055	0.4427	4.3758	0.036814	0.0387	0.0190776560
ss86283798		4	79.648511	0.4599	5.8125	0.016170	0.0444	0.0220549759
ss86327850		4	79.680118	0.3338	4.6523	0.031354	0.0417	0.0185515867
ss86338073		4	79.701138	0.5566	4.7212	0.030129	0.0401	0.0197859323
ss86284461		4	79.763952	0.3223	0.0649	0.798966	0.0051	0.0022376623
ss86298954		4	79.828828	0.2335	0.0054	0.941245	0.0017	0.0005996658
ss61510281	*rs41602724*	4	79.862089	0.7049	0.4197	0.517289	0.0134	0.0055945078
ss86340153		4	79.907063	0.6898	1.7697	0.183851	0.0274	0.0117287785
ss86338750		4	79.961322	0.5630	4.7273	0.030023	0.0408	0.0200537377

ss65319471	*rs43406888*	4	80.021672	0.0537	9.5195	0.002114	0.1286	0.0130717460
ss65319442	*rs43406859*	4	80.06333	0.0430	3.4086	0.065283	0.0824	0.0067755201
ss86336617		4	80.31631	0.2844	0.7268	0.394225	0.0175	0.0071286332
ss86339537		4	80.336324	0.7407	1.4748	0.224999	0.0258	0.0099195165
ss86337903		4	80.429747	0.2378	3.5542	0.059812	0.0408	0.0147998902
ss63506279	*rs42253570*	4	80.530607	0.7715	8.1771	0.004369	0.0623	0.0219579814
	rs29026587	4	80.56853	0.8961	3.1495	0.076389	0.0542	0.0100807665
ss61477453		4	80.675668	0.9119	2.1729	0.140910	0.0480	0.0077183349
ss86288950		4	80.698121	0.3481	1.2557	0.262850	0.0218	0.0099149107
ss86285338		4	80.771833	0.8001	6.1996	0.013010	0.0576	0.0184203849
ss61520857	*rs41618376*	4	80.807275	0.4255	6.1631	0.013279	0.0460	0.0225059560
ss61520860	*rs41618379*	4	80.835902	0.3345	3.3191	0.068909	0.0361	0.0160849913
ss61528225	*rs41631261*	4	80.892532	0.3266	2.5531	0.110534	0.0319	0.0140435304
ss86338440		4	80.920621	0.0938	1.4911	0.222460	0.0393	0.0066787154
ss86335717		4	80.954539	0.6576	3.4032	0.065495	0.0360	0.0162023339
ss86329224		4	81.053882	0.2787	0.7608	0.383364	0.0179	0.0072032542
ss61477452	*rs41576787*	4	81.291686	0.6426	1.2762	0.259002	0.0219	0.0100432475
ss63505946	*rs42252160*	4	81.331204	0.5960	0.3838	0.535776	0.0118	0.0056914623
ss86336449		4	81.576883	0.5716	4.1129	0.042938	0.0403	0.0197444092
ss117967456		4	81.872381	0.8367	9.5843	0.002041	0.0783	0.0213901886
ss86298988		4	81.897716	0.7085	0.4037	0.525407	0.0132	0.0054589831
ss86323329		4	81.928381	0.4191	17.4201	0.000034	0.0799	0.0389031338
ss86337167		4	81.976223	0.6311	5.4081	0.020330	0.0438	0.0204178342
ss65323923	*rs43407438*	4	82.027126	0.9592	2.9486	0.086397	0.0798	0.0062497625
ss65323977	*rs43407489*	4	82.054705	0.7213	0.4465	0.504243	0.0138	0.0055314915
ss86337834		4	82.146284	0.4291	0.1544	0.694502	0.0073	0.0035884520
ss64247686	*rs42549080*	4	82.216389	0.6705	2.5158	0.113164	0.0309	0.0136659267
ss86319069		4	82.259607	0.6297	0.3666	0.545069	0.0112	0.0052237385
ss86334537		4	82.322977	0.3725	0.1408	0.707625	0.0071	0.0033181932
ss64944995	*rs43037387*	4	82.486998	0.4191	0.7605	0.383472	0.0173	0.0084016303
ss65324033	*rs43407845*	4	82.5331	0.6855	0.9487	0.330399	0.0199	0.0085770987
ss86311419		4	82.556898	0.5809	0.6547	0.418711	0.0159	0.0077462696
ss61468900	*rs41573210*	4	82.601296	0.1168	11.7326	0.000650	0.0979	0.0202008926
ss65324084	*rs43407896*	4	82.622601	0.1755	8.7151	0.003262	0.0740	0.0214106745
ss61470534	*rs41570519*	4	82.824272	0.5688	20.9100	0.000006	0.0847	0.0415251444
ss61512791	*rs41615292*	4	82.846243	0.5716	20.7211	0.000006	0.0848	0.0415517942
ss63702216	*rs42315691*	4	82.961318	0.9033	4.4167	0.035947	0.0645	0.0112769602
ss61470531	*rs41570516*	4	83.055377	0.6913	1.0288	0.310791	0.0201	0.0085731880
ss63702708	*rs42316855*	4	83.07726	0.0824	11.3363	0.000802	0.1137	0.0171865453
ss61470533	*rs41570518*	4	83.099656	0.6332	2.8166	0.093742	0.0327	0.0151673178
ss86311720		4	83.151173	0.7486	18.8611	0.000016	0.0934	0.0351762008
ss61512790	*rs41615291*	4	83.205716	0.7285	0.1421	0.706307	0.0081	0.0032175932
ss63703200	*rs42317419*	4	83.237982	0.1705	20.4601	0.000007	0.1073	0.0303538165
ss61470546	*rs43745280*	4	83.272843	0.4799	7.9359	0.004984	0.0514	0.0256422675
ss86340046		4	83.325371	0.7135	5.1249	0.023892	0.0462	0.0188958883
ss63703845	*rs42318933*	4	83.347928	0.4234	2.7000	0.100799	0.0306	0.0149414726
ss61554740	*rs41654180*	4	83.384901	0.9083	2.1309	0.144805	0.0456	0.0075959350

	rs29023393	4	83.420024	0.6547	4.4756	0.034736	0.0406	0.0183493386
ss61503814	*rs41599805*	4	83.44517	0.2185	7.6568	0.005806	0.0618	0.0211211004
ss61554745	*rs41654185*	4	83.469087	0.6003	8.6993	0.003290	0.0564	0.0270694924
ss61490191	*rs41590201*	4	83.611096	0.5158	22.7993	0.000002	0.0855	0.0427104666
ss61554748	*rs41654188*	4	83.707179	0.9133	5.1923	0.022990	0.0767	0.0121492507
ss86278166		4	83.768797	0.6418	5.3829	0.020623	0.0456	0.0209460500
ss61526404	*rs41623098*	4	83.836676	0.4943	0.0540	0.816262	0.0044	0.0021995392
ss61554751	*rs41654191*	4	83.890517	0.2135	0.0522	0.819363	0.0052	0.0017557616
	rs29021024	4	83.928825	0.8510	0.3361	0.562282	0.0149	0.0037898808
ss86320652		4	83.983603	0.5021	2.0971	0.148025	0.0276	0.0137966623
ss61505005	*rs41601973*	4	84.024719	0.4155	2.6672	0.102888	0.0314	0.0152641478
ss62036943	*rs41734418*	4	84.250725	0.5014	8.2514	0.004196	0.0551	0.0275687018
ss62037099	*rs41734870*	4	84.274885	0.5050	8.5602	0.003548	0.0559	0.0279536240
ss64390856	*rs42615283*	4	84.532343	0.1096	0.8073	0.369225	0.0276	0.0053799094
ss86334467		4	84.56137	0.5723	2.3696	0.124177	0.0290	0.0141960575
ss64391050	*rs42616380*	4	84.628509	0.4448	4.4463	0.035334	0.0399	0.0197180596
ss62036763	*rs41734358*	4	84.706768	0.1877	22.9475	0.000002	0.1117	0.0340577925
ss62036337	*rs41733215*	4	84.729443	0.8059	21.6548	0.000004	0.1062	0.0332131085
ss62036247	*rs41733185*	4	84.764068	0.1017	18.3675	0.000021	0.1281	0.0234104764
ss62036145	*rs41733152*	4	84.787899	0.9456	0.2229	0.636984	0.0191	0.0019713500
ss86330155		4	84.810854	0.2307	1.7743	0.183285	0.0286	0.0101381521
ss117963737		4	84.89721	0.2213	1.9398	0.164130	0.0304	0.0104905362
ss63038774	*rs42080428*	4	84.949496	0.8424	3.9498	0.047269	0.0475	0.0126005536
ss86338233		4	85.006402	0.2249	0.5666	0.451882	0.0166	0.0057815927
ss65324443	*rs43410555*	4	85.033952	0.7830	0.7661	0.381721	0.0193	0.0065446481
ss86291000		4	85.064487	0.3195	0.0261	0.871657	0.0033	0.0014139025
ss61523598	*rs41622261*	4	85.116647	0.1168	1.3576	0.244354	0.0343	0.0070657872
ss86316325		4	85.207109	0.3345	1.3496	0.245753	0.0227	0.0101182177
ss63919250	*rs42229487*	4	85.270694	0.6153	12.9134	0.000349	0.0666	0.0315062934
ss63919332	*rs42229528*	4	85.293378	0.1089	2.1542	0.142633	0.0442	0.0085692096
ss61522497	*rs41619869*	4	85.332103	0.1003	1.2147	0.270792	0.0341	0.0061530450
ss61524300	*rs41619752*	4	85.37705	0.1920	4.0718	0.043988	0.0460	0.0142764891
ss86292447		4	85.401762	0.1927	3.7079	0.054564	0.0439	0.0136589864
ss86336689		4	85.449034	0.4828	0.0062	0.937017	0.0015	0.0007387006
ss63920366	*rs42233645*	4	85.532016	0.4635	0.0519	0.819871	0.0042	0.0020831149
ss117967344		4	85.644103	0.2077	1.5624	0.211729	0.0279	0.0091854047
	rs29021284	4	85.65953	0.5014	6.7707	0.009464	0.0470	0.0235075265
ss63920486	*rs42233703*	4	85.681606	0.4850	0.0339	0.853889	0.0034	0.0016772061
ss64049475	*rs42448658*	4	85.784182	0.3037	0.6942	0.405038	0.0170	0.0071733896
ss86298371		4	85.823298	0.7787	5.2766	0.021909	0.0502	0.0173095482
ss61511159	*rs41610284*	4	85.865487	0.2328	0.0051	0.942972	0.0016	0.0005594465
ss86326452		4	85.888762	0.7486	11.5018	0.000735	0.0709	0.0266875069
ss86310434		4	85.923634	0.0809	3.2262	0.072902	0.0609	0.0090645059
ss86330496		4	85.960216	0.4133	4.8306	0.028288	0.0410	0.0198888190
ss61528660	*rs41627293*	4	85.998609	0.7436	3.3244	0.068687	0.0378	0.0144119515
ss64155925	*rs42502017*	4	86.031519	0.2407	8.8777	0.002987	0.0641	0.0234459828
ss86314903		4	86.17568	0.2500	6.0281	0.014323	0.0534	0.0200289462

ss64874042	*rs42968957*	4	86.213244	0.3603	2.8759	0.090363	0.0334	0.0154168185
ss61568293	*rs41663091*	4	86.246427	0.6347	2.7116	0.100073	0.0323	0.0149906621
	rs29024319	4	86.283586	0.2958	0.5039	0.478019	0.0146	0.0060864849
ss61568310	*rs41663108*	4	86.307557	0.8854	1.1276	0.288665	0.0305	0.0061896949
ss63644110	*rs42305691*	4	86.341657	0.1132	0.9966	0.318484	0.0288	0.0057778461
ss61496687	*rs41592039*	4	86.372608	0.8782	0.6515	0.419868	0.0223	0.0047785953
ss61568299	*rs41663097*	4	86.401913	0.2178	0.0032	0.954792	0.0013	0.0004308605
	rs29013346	4	86.439992	0.8968	0.3334	0.563840	0.0179	0.0033037193
ss61496689	*rs41592041*	4	86.46129	0.7872	0.0009	0.976370	0.0007	0.0002228242
ss86336038		4	86.550973	0.2378	2.8338	0.092750	0.0363	0.0131634103
ss63644617	*rs42297068*	4	86.588685	0.7156	0.3004	0.583793	0.0110	0.0044942687
ss64761827	*rs42860860*	4	86.744973	0.0638	4.4727	0.034796	0.0793	0.0094656582
ss64959980	*rs43050927*	4	86.777168	0.2958	2.7227	0.099383	0.0345	0.0143832853
ss86336680		4	86.827076	0.9405	1.6998	0.192750	0.0503	0.0056300813
ss86340822		4	86.850397	0.7063	0.0268	0.870092	0.0034	0.0013969841
ss86291413		4	87.02881	0.3223	8.2093	0.004293	0.0561	0.0245282383
ss61525389	*rs41620414*	4	87.082107	0.7027	2.9426	0.086715	0.0343	0.0143321966
ss63954970	*rs42408732*	4	87.163542	0.8933	0.2473	0.619147	0.0152	0.0028950584
ss63955010	*rs42408752*	4	87.189665	0.7715	0.7865	0.375457	0.0189	0.0066707566
ss86334815		4	87.230261	0.7385	6.8287	0.009164	0.0545	0.0210480677
ss86289911		4	87.345389	0.4398	7.2585	0.007227	0.0499	0.0245651482
ss63955862	*rs42403778*	4	87.370786	0.5344	7.1838	0.007531	0.0491	0.0244446502
ss86335742		4	87.394227	0.2500	7.4379	0.006547	0.0578	0.0216827332
	rs29009595	4	87.511031	0.8632	0.0622	0.803085	0.0067	0.0015822503
ss64284164	*rs42567625*	4	87.53515	0.3904	0.1551	0.693852	0.0075	0.0035509431
ss86322254		4	87.56588	0.5795	0.1129	0.737014	0.0063	0.0030754027
ss61566634	*rs41666560*	4	87.593638	0.5681	0.0212	0.884157	0.0027	0.0013307882
ss64757079	*rs42850841*	4	87.680418	0.0573	0.0588	0.808470	0.0099	0.0010692860
ss64833147	*rs42925710*	4	87.790298	0.3059	2.6023	0.107165	0.0320	0.0135945744
ss61554737	*rs41654177*	4	87.812971	0.4026	0.3968	0.528965	0.0116	0.0055868336
ss65324701	*rs43412212*	4	87.849857	0.4470	0.6124	0.434157	0.0149	0.0073500190
ss117967496		4	87.877702	0.7085	0.2169	0.641558	0.0095	0.0039206511
ss117963719		4	87.907646	0.3489	0.6266	0.428886	0.0153	0.0069579886
ss86285180		4	87.942904	0.7221	3.4153	0.065020	0.0382	0.0153218869
ss61554739	*rs41654179*	4	88.00953	0.5953	1.8015	0.179968	0.0248	0.0119431953
ss61514291	*rs41610664*	4	88.046022	0.3195	5.1267	0.023868	0.0462	0.0200864928
ss64530218	*rs42688382*	4	88.071518	0.8238	0.0009	0.976202	0.0007	0.0002057303
ss61514292	*rs41610665*	4	88.104196	0.2264	3.7463	0.053330	0.0429	0.0150364610
ss63913470	*rs42177800*	4	88.219564	0.3703	9.0006	0.002795	0.0567	0.0264619609
ss63913486	*rs42177807*	4	88.269814	0.3653	0.0709	0.790135	0.0051	0.0023848001
ss86319927		4	88.295214	0.3259	4.1480	0.042062	0.0394	0.0173064362
ss61517629	*rs41617093*	4	88.505619	0.3166	0.3485	0.555155	0.0120	0.0051771147
ss64670666	*rs42764136*	4	88.536201	0.3188	0.4692	0.493578	0.0137	0.0059704439
ss86299833		4	88.562391	0.3195	0.4889	0.484673	0.0140	0.0061038871
ss86293686		4	88.599954	0.5287	0.4586	0.498507	0.0123	0.0061278393
ss86340469		4	88.630298	0.5609	0.0086	0.926038	0.0017	0.0008543840
ss64791848	*rs42886825*	4	88.714139	0.3660	3.6533	0.056369	0.0377	0.0175155441

ss61551785	*rs43706156*	4	88.748827	0.3668	3.6060	0.057986	0.0375	0.0174292757
ss63332887	*rs42187999*	4	88.80851	0.3216	8.1638	0.004401	0.0539	0.0235130804
ss86337905		4	88.939598	0.5903	0.7189	0.396812	0.0153	0.0073950091
	rs29020619	4	88.986303	0.7049	4.4245	0.035785	0.0426	0.0177213571
ss61551791	*rs43706162*	4	89.020565	0.8617	2.3780	0.123506	0.0404	0.0096189179
ss86341333		4	89.052623	0.4334	0.0455	0.831217	0.0041	0.0020257832
ss63333469	*rs42190193*	4	89.087907	0.6297	0.0556	0.813672	0.0047	0.0021839066
ss61488595	*rs41586543*	4	89.129909	0.1433	0.0505	0.822213	0.0059	0.0014468113
ss61551797	*rs43706168*	4	89.159543	0.5544	0.4164	0.518930	0.0126	0.0062086465
ss86325248		4	89.201904	0.6096	5.2664	0.022038	0.0440	0.0209334713
ss61482952	*rs41583631*	4	89.223952	0.1275	0.0479	0.826781	0.0060	0.0013402962
ss86284796		4	89.303857	0.6877	0.8371	0.360557	0.0185	0.0079366313
ss65490364	*rs43577241*	4	89.335844	0.2479	0.3025	0.582491	0.0117	0.0043480263
ss86317393		4	89.36244	0.1891	0.0888	0.765754	0.0071	0.0021816978
	rs29016492	4	89.457377	0.3553	0.2312	0.630823	0.0094	0.0042939752
ss61529685	*rs41627097*	4	89.484088	0.5093	0.4263	0.514038	0.0124	0.0062212478
ss65325184	*rs43410892*	4	89.52833	0.2249	0.0003	0.985813	0.0004	0.0001349233
ss86308008		4	89.572685	0.7486	0.6027	0.437818	0.0169	0.0063643370
ss63517700	*rs42257351*	4	89.606682	0.7543	0.0074	0.931668	0.0018	0.0006760591
ss63517760	*rs42257371*	4	89.658795	0.6683	0.9102	0.340398	0.0188	0.0083174062
ss86284874		4	89.704062	0.7457	1.1775	0.278240	0.0224	0.0085065064
ss86320273		4	89.766808	0.7693	0.7979	0.372029	0.0195	0.0069218655
ss61478102		4	89.829524	0.1232	6.2527	0.012629	0.0694	0.0150007952
ss86336296		4	89.907223	0.2615	8.1958	0.004325	0.0588	0.0227267068
	rs29018180	4	89.980127	0.3037	8.7754	0.003157	0.0567	0.0239862342
ss63978717	*rs42417654*	4	90.00874	0.2765	4.4789	0.034670	0.0451	0.0180618399
ss61476339	*rs41575600*	4	90.209357	0.9420	0.3024	0.582529	0.0223	0.0024387302
ss117967549		4	90.249365	0.5193	0.0956	0.757265	0.0059	0.0029260406
ss61475435	*rs41574512*	4	90.38026	0.1146	1.7327	0.188506	0.0386	0.0078400259
	rs29020693	4	90.418033	0.4570	4.2726	0.039100	0.0381	0.0188885360
ss86332202		4	90.512732	0.4993	0.0097	0.921624	0.0019	0.0009427084
ss86341416		4	90.652398	0.2679	0.0048	0.944824	0.0015	0.0005753368
ss86325866		4	90.699542	0.4047	0.2689	0.604260	0.0094	0.0045506492
ss61567042	*rs41659862*	4	90.72069	0.4090	0.1186	0.730636	0.0063	0.0030233808
ss61547968	*rs41644987*	4	90.985709	0.9119	0.5359	0.464399	0.0241	0.0038676727
ss86311826		4	91.006407	0.6870	0.0128	0.909950	0.0023	0.0009932316
	rs29017652	4	91.026712	0.3116	0.0058	0.939549	0.0016	0.0006659558
ss61468368	*rs41567487*	4	91.078598	0.3109	0.0051	0.943071	0.0015	0.0006260339
ss86336945		4	91.1	0.6891	0.0051	0.943071	0.0015	0.0006260339
ss117963726		4	91.217498	0.4878	0.1580	0.691155	0.0075	0.0037253564
ss86293678		4	91.261835	0.7607	0.1777	0.673481	0.0089	0.0032466836
ss86340353		4	91.340565	0.8173	1.9552	0.162469	0.0328	0.0097911206
ss62919171	*rs42038819*	4	91.492313	0.2958	4.9570	0.026305	0.0435	0.0181214641
ss61547977		4	91.514964	0.5380	0.0721	0.788430	0.0051	0.0025346981
ss86292784		4	91.592734	0.5201	0.1908	0.662360	0.0079	0.0039437464
ss61507589	*rs41607880*	4	91.627026	0.4706	0.0785	0.779486	0.0052	0.0025896375
ss86332777		4	91.649261	0.8438	0.6648	0.415147	0.0205	0.0054090719

ss61528527	*rs41626572*	4	91.682788	0.3567	2.0123	0.156474	0.0266	0.0122174295
ss61512403	*rs41611709*	4	91.813061	0.7550	0.0038	0.951110	0.0013	0.0004675669
ss61512402	*rs41611708*	4	91.955102	0.5996	0.0032	0.955138	0.0010	0.0005039746
	rs29017544	4	91.976234	0.3467	2.0692	0.150754	0.0272	0.0123334923
ss86285884		4	92.087106	0.5766	1.1322	0.287665	0.0195	0.0095449338
ss64045677	*rs42447466*	4	92.136302	0.3968	0.7115	0.399233	0.0157	0.0075378052
ss64045683	*rs42447469*	4	92.158599	0.2507	0.0793	0.778283	0.0060	0.0022444129
ss64776543	*rs42871995*	4	92.193078	0.8109	0.3943	0.530265	0.0150	0.0046029885
ss117967166		4	92.221551	0.4835	0.3609	0.548182	0.0112	0.0055955169
ss61499125	*rs43708323*	4	92.263952	0.4606	0.1619	0.687502	0.0076	0.0037820390
ss64580842	*rs42708915*	4	92.310809	0.4491	3.6977	0.054897	0.0362	0.0179311626
ss86327047		4	92.400328	0.6726	1.4435	0.229978	0.0235	0.0103707362
ss61476341	*rs41575602*	4	92.617122	0.7479	8.4620	0.003742	0.0593	0.0223625313
ss86326040		4	92.658825	0.5394	6.5771	0.010538	0.0472	0.0234375895
ss61567756		4	92.680991	0.1340	3.9233	0.048014	0.0543	0.0126052761
ss64852841	*rs42946916*	4	92.711412	0.8660	3.9233	0.048014	0.0543	0.0126052761
ss64023565	*rs42436989*	4	92.854945	0.2865	3.6222	0.057426	0.0385	0.0157216617
ss86334899		4	92.917825	0.6855	4.8657	0.027722	0.0422	0.0181843990
ss64023425	*rs42436619*	4	92.945549	0.5666	0.5645	0.452701	0.0138	0.0067666552
ss61527744	*rs41625988*	4	93.020619	0.0759	3.2796	0.070578	0.0622	0.0087258069
ss64023291	*rs42436552*	4	93.050422	0.5007	1.8463	0.174658	0.0245	0.0122711995
ss117967384		4	93.076506	0.1440	0.1763	0.674677	0.0107	0.0026487454
ss65099689	*rs43191790*	4	93.186666	0.2221	0.0009	0.975643	0.0007	0.0002289873
ss61477136	*rs41574883*	4	93.212579	0.2221	0.0009	0.975643	0.0007	0.0002289873
ss61475860	*rs41577429*	4	93.254704	0.3059	0.1141	0.735616	0.0066	0.0027865576
ss61472537	*rs41571562*	4	93.400261	0.9133	1.7571	0.185418	0.0442	0.0069935313
ss64298664	*rs42572206*	4	93.475757	0.8797	0.1068	0.743955	0.0093	0.0019772087
	rs29015630	4	93.502428	0.3102	0.0000	0.995553	0.0001	0.0000482960
ss64862652	*rs42956663*	4	93.539307	0.3166	0.3752	0.540367	0.0121	0.0052466233
	rs29022155	4	93.578601	0.6834	0.3752	0.540367	0.0121	0.0052466233
ss61570750	*rs41671301*	4	93.603761	0.3166	0.3752	0.540367	0.0121	0.0052466233
ss64724753	*rs42823455*	4	93.703298	0.1254	0.4584	0.498609	0.0185	0.0040643117
ss64724720	*rs42822822*	4	93.759862	0.8746	0.4584	0.498609	0.0185	0.0040643117
ss38333748	*rs29017454*	4	93.795298	0.6648	4.0073	0.045690	0.0378	0.0168510894
ss86300276		4	93.837806	0.8202	0.3100	0.577862	0.0134	0.0039549572
ss86298568		4	93.879303	0.1626	2.2033	0.138164	0.0369	0.0100375407
ss65326181	*rs43410990*	4	93.938877	0.3567	2.4733	0.116249	0.0309	0.0141671463
ss65326209	*rs43411018*	4	93.97831	0.4835	0.3753	0.540335	0.0111	0.0055682119
ss86291350		4	94.153275	0.6074	2.0241	0.155273	0.0262	0.0124864680
ss86295580		4	94.253899	0.3302	1.1350	0.287080	0.0214	0.0094636061
ss61520235	*rs41612264*	4	94.33079	0.2128	0.0028	0.957943	0.0012	0.0004109812
ss65015954	*rs43105279*	4	94.406346	0.3660	0.0320	0.857972	0.0036	0.0016762327
ss86337606		4	94.432828	0.5122	0.2189	0.640051	0.0090	0.0045166023
ss86297686		4	94.477476	0.6032	0.2165	0.641830	0.0089	0.0042771819
ss86337747		4	94.520469	0.3309	3.1753	0.075196	0.0348	0.0154094428
ss65325553	*rs43415359*	4	94.554481	0.5774	0.0295	0.863591	0.0032	0.0015747510
ss65325556	*rs43415362*	4	94.579189	0.3510	0.0042	0.948549	0.0013	0.0005811862

	rs29013711	4	94.612979	0.3940	0.8595	0.354199	0.0176	0.0083861904
ss65325613	*rs43415419*	4	94.639476	0.1390	0.2871	0.592267	0.0142	0.0034028069
ss86341216		4	94.666845	0.6060	0.8595	0.354199	0.0176	0.0083861904
ss61475388	*rs41574468*	4	94.709605	0.8481	0.9200	0.337808	0.0252	0.0064869586
ss65325982	*rs43408491*	4	94.742277	0.7371	4.1892	0.041057	0.0428	0.0166069143
ss65326002	*rs43408511*	4	94.772013	0.6125	3.6574	0.056229	0.0371	0.0175963845
ss65326028	*rs43409537*	4	94.826706	0.8489	1.5310	0.216381	0.0319	0.0081947241
ss65326013	*rs43408522*	4	94.870406	0.6074	3.3476	0.067731	0.0355	0.0169125730
ss63988343	*rs42417266*	4	94.975831	0.3324	5.9359	0.015085	0.0481	0.0213350739
ss86325278		4	95.008422	0.5143	1.9842	0.159392	0.0261	0.0130356090
ss86338522		4	95.049418	0.6490	0.0578	0.810079	0.0047	0.0021527088
ss63988461	*rs42417324*	4	95.09795	0.1189	3.7945	0.051821	0.0554	0.0116140834
ss117967381		4	95.125025	0.2579	0.3760	0.539940	0.0131	0.0050243909
ss61476515	*rs41576873*	4	95.162745	0.3918	0.1217	0.727340	0.0067	0.0031841343
ss86305583		4	95.185082	0.3897	0.0764	0.782319	0.0053	0.0025194566
ss63988605	*rs42418694*	4	95.214077	0.1468	0.4753	0.490795	0.0164	0.0040986821
ss63988615	*rs42418699*	4	95.240634	0.5287	1.0188	0.313160	0.0188	0.0093759293
ss61499909	*rs41596287*	4	95.274122	0.6160	0.0297	0.863167	0.0032	0.0015306121
ss117967461		4	95.382435	0.6182	2.1810	0.140178	0.0288	0.0135959415
ss61528237	*rs41631273*	4	95.44667	0.0752	2.1977	0.138670	0.0527	0.0073330880
ss86326960		4	95.486227	0.2550	0.0281	0.866935	0.0035	0.0013443329
ss86332036		4	95.639598	0.4463	0.5671	0.451671	0.0140	0.0069219127
ss86289533		4	95.685552	0.5100	0.1245	0.724318	0.0067	0.0033401059
ss86286726		4	95.7155	0.4377	0.0288	0.865229	0.0033	0.0016014439
ss86282424		4	95.741416	0.7493	0.0378	0.845996	0.0041	0.0015524059
ss86318833		4	95.825044	0.1361	0.0008	0.976775	0.0008	0.0001815782
ss61468903	*rs41573213*	4	95.943153	0.0888	0.1209	0.728208	0.0116	0.0018804237
ss61490219	*rs41590228*	4	96.049524	0.5158	0.6059	0.436618	0.0146	0.0072773723
ss86336586		4	96.070649	0.1168	0.0269	0.869750	0.0048	0.0009812708
ss86337285		4	96.110844	0.6433	0.9982	0.318099	0.0196	0.0089925823
ss86285703		4	96.134776	0.6096	0.4534	0.500931	0.0132	0.0062974050
ss61503824	*rs41599815*	4	96.164823	0.3940	0.4879	0.485121	0.0137	0.0065618172
ss86321667		4	96.194788	0.6053	0.6155	0.432988	0.0154	0.0073579871
ss65326768	*rs43408576*	4	96.254538	0.7120	2.8614	0.091177	0.0342	0.0140210404
ss86326548		4	96.298171	0.7944	0.2630	0.608236	0.0117	0.0038174210
ss65326756	*rs43408564*	4	96.323701	0.1640	1.5323	0.216184	0.0309	0.0084739498
ss86331636		4	96.489307	0.1640	1.8050	0.179545	0.0339	0.0092975402
ss61554780	*rs41654220*	4	96.509524	0.8044	0.5160	0.472798	0.0165	0.0052009399
ss61490218	*rs41590227*	4	96.53917	0.8360	1.8050	0.179545	0.0339	0.0092975402
ss61554778	*rs41654218*	4	96.57736	0.3789	0.0131	0.908917	0.0023	0.0010604281
ss117967448		4	96.600292	0.6404	0.0144	0.904410	0.0024	0.0010987192
ss117967220		4	96.631818	0.0960	0.6428	0.422969	0.0254	0.0044109706
ss86337551		4	96.652122	0.0960	0.6428	0.422969	0.0254	0.0044109706
ss65326455	*rs43414162*	4	96.731025	0.5473	0.0180	0.893186	0.0025	0.0012313731
ss65326349	*rs43412957*	4	96.757469	0.6196	1.2166	0.270407	0.0206	0.0097059075
ss65326382	*rs43412990*	4	96.780602	0.0989	0.5374	0.463776	0.0230	0.0040985770
ss86275201		4	96.804516	0.5423	0.5003	0.479621	0.0131	0.0064991337

ss86296759		4	96.828049	0.1039	0.0479	0.826825	0.0066	0.0012336010
ss61490200	rs41590209	4	96.852385	0.2822	0.5293	0.467164	0.0149	0.0060341985
ss86337142		4	96.891566	0.2493	0.6163	0.432675	0.0166	0.0062050755
ss86291735		4	96.992456	0.1956	0.0247	0.875236	0.0037	0.0011693471
ss64703498	rs42803633	4	97.184123	0.1246	3.9777	0.046497	0.0569	0.0124162607
ss64718730	rs42815661	4	97.267735	0.4520	0.1767	0.674337	0.0081	0.0039891565
ss61554788	rs41647833	4	97.326516	0.1039	4.5681	0.032920	0.0631	0.0117412330
ss61490223	rs41590632	4	97.348859	0.4205	0.0001	0.992633	0.0002	0.0000862840
ss65327230	rs43412238	4	97.373434	0.7443	1.9134	0.167030	0.0296	0.0112625119
ss65327215	rs43411523	4	97.397151	0.6426	0.0001	0.992639	0.0002	0.0000803479
ss86305412		4	97.443438	0.2149	0.5185	0.471744	0.0162	0.0054801269
ss65327325	rs43413033	4	97.466649	0.8696	2.4842	0.115449	0.0424	0.0096249553
ss117963759		4	97.492385	0.7514	0.8403	0.359633	0.0194	0.0072597045
ss86277975		4	97.558079	0.8044	0.0043	0.948033	0.0015	0.0004750916
ss61523599	rs41622262	4	97.581015	0.1024	0.9671	0.325738	0.0301	0.0055397012
ss64400764	rs42618422	4	97.733712	0.8059	0.1412	0.707171	0.0087	0.0027366440
ss86288363		4	97.760957	0.4792	1.3438	0.246755	0.0216	0.0107589853
ss65327398	rs43413106	4	97.813378	0.1547	3.0808	0.079660	0.0460	0.0120279142
ss65327415	rs43413123	4	97.840055	0.1956	0.1567	0.692357	0.0092	0.0028938589
ss61490225	rs41590634	4	97.890174	0.5924	3.3804	0.066402	0.0339	0.0163877753
ss86338220		4	98.025904	0.7622	1.7107	0.191321	0.0276	0.0100021359
ss86333092		4	98.09786	0.5358	0.2663	0.606017	0.0096	0.0047737428
ss86325477		4	98.125012	0.1003	0.4050	0.524740	0.0200	0.0036086207
ss65104673	rs43192772	4	98.332302	0.3367	0.1764	0.674573	0.0083	0.0037288354
ss117963766		4	98.442159	0.5437	0.7857	0.375704	0.0163	0.0080853539
ss86303785		4	98.463138	0.8424	12.4008	0.000457	0.0890	0.0236408872
ss86323365		4	98.50755	0.5587	0.9665	0.325907	0.0180	0.0088885345
ss86331174		4	98.545037	0.3424	1.7362	0.188050	0.0255	0.0114948921
ss86339639		4	98.670569	0.7958	3.6288	0.057200	0.0447	0.0145199216
ss86310412		4	98.695328	0.1984	6.5111	0.010933	0.0606	0.0192780798
ss61490238	rs41590647	4	98.721098	0.6361	1.5786	0.209385	0.0232	0.0107310823
ss61490237	rs41590646	4	98.823259	0.5057	0.5131	0.474046	0.0135	0.0067546788
	rs29023486	4	98.848662	0.1282	0.0989	0.753290	0.0087	0.0019468349
ss65328360	rs43415667	4	98.898746	0.3711	0.0795	0.778113	0.0056	0.0025942808
ss61490235	rs41590644	4	99.033102	0.3288	1.0001	0.317636	0.0202	0.0089242491
	rs29022385	4	99.054353	0.2049	0.7056	0.401186	0.0192	0.0062516304
ss61510326	rs41603668	4	99.108441	0.5000	0.0709	0.790155	0.0050	0.0025211418
ss86337915		4	99.134963	0.1089	0.0208	0.885325	0.0044	0.0008603085
ss117963774		4	99.164426	0.1956	0.0044	0.947251	0.0015	0.0004759613
ss86333818		4	99.198019	0.2765	0.9459	0.331108	0.0194	0.0077608136
ss86293538		4	99.232084	0.7120	5.8921	0.015462	0.0496	0.0203544139
ss86326030		4	99.260855	0.2894	4.0985	0.043304	0.0414	0.0170251391
ss117967314		4	99.303591	0.4212	1.8485	0.174401	0.0252	0.0122707616
ss86332859		4	99.331652	0.3403	2.0794	0.149751	0.0276	0.0123745937
ss86314473		4	99.37632	0.1390	1.7108	0.191319	0.0333	0.0079721610
ss86295080		4	99.412225	0.2407	4.4971	0.034305	0.0465	0.0169842885
ss86333475		4	99.531639	0.6841	0.6308	0.427323	0.0160	0.0069137403

ss86288361		4	99.58719	0.9628	1.7699	0.183831		0.0643	0.0046142357
ss86301298		4	99.608627	0.1039	0.0383	0.844989		0.0060	0.0011211968
ss86316026		4	99.65028	0.0960	0.0622	0.803177		0.0076	0.0013276154
ss86324981		4	99.675002	0.1268	0.4225	0.515911		0.0181	0.0040006302
ss86293672		4	99.738285	0.5802	2.9243	0.087702		0.0324	0.0158014819
ss61490259	*rs41590668*	4	99.88186	0.7099	1.3322	0.248807		0.0228	0.0093768120
ss61517548	*rs41616120*	4	99.997655	0.2099	0.5956	0.440521		0.0174	0.0057723246
ss86296164		4	100.169378	0.4126	18.5629	0.000019		0.0821	0.0397919373
ss86328779		4	100.428045	0.7665	0.1540	0.694822		0.0086	0.0030633464
ss61510332		4	100.634713	0.5688	0.0535	0.817203		0.0045	0.0022179801
ss86298460		4	100.680605	0.4448	1.2499	0.263958		0.0210	0.0103535799
ss61554853	*rs41647894*	4	100.906347	0.1812	2.6843	0.101795		0.0388	0.0115051125
ss86341582		4	100.962281	0.7486	9.1601	0.002565		0.0623	0.0234327022
ss86283454		4	100.994229	0.1769	3.0472	0.081320		0.0427	0.0124382389
ss86333674		4	101.028764	0.1540	10.1888	0.001477		0.0810	0.0211104308
ss86340566		4	101.207399	0.7958	19.1557	0.000014		0.0964	0.0313143292
ss61514099	*rs41610176*	4	101.329673	0.7915	4.0256	0.045201		0.0463	0.0152664255
ss86338762		4	101.597918	0.5315	4.8149	0.028545		0.0403	0.0200790148
ss86341433		4	101.672116	0.7822	11.4141	0.000769		0.0748	0.0254998676
ss86306854		4	101.912398	0.6297	0.0089	0.924937		0.0019	0.0008875440
ss86338469		4	101.940139	0.6318	5.3627	0.020862		0.0429	0.0199496370
ss86321725		4	102.186665	0.3231	13.2558	0.000292		0.0698	0.0305236268
	rs29020598	4	102.242252	0.2335	0.1926	0.660881		0.0096	0.0034253714
	rs29020595	4	102.271366	0.2758	1.3899	0.238826		0.0244	0.0097462486
ss61466748	*rs41572290*	4	102.346051	0.9162	2.7441	0.098066		0.0555	0.0085163793
ss86275000		4	102.378315	0.3696	7.1309	0.007754		0.0517	0.0240944651
ss61490278	*rs41590687*	4	102.433636	0.5265	0.0237	0.877801		0.0028	0.0013871837
ss61490279	*rs41590688*	4	102.460352	0.4685	0.0109	0.916764		0.0019	0.0009464886
ss86308747		4	102.489302	0.5258	0.0802	0.777097		0.0051	0.0025504277
ss86331228		4	102.527277	0.2564	0.0878	0.767081		0.0064	0.0024498616
ss61490283	*rs41590692*	4	102.554811	0.1691	0.1910	0.662192		0.0108	0.0030205970
ss61569946	*rs41671195*	4	102.748783	0.8811	0.0103	0.919139		0.0029	0.0006093873
ss86295036		4	102.770444	0.4527	13.1834	0.000303		0.0686	0.0339932750
ss61554966	*rs41649305*	4	102.964465	0.8567	6.1175	0.013622		0.0650	0.0159567047
ss86325615		4	103.070403	0.0437	2.7298	0.098942		0.0759	0.0063395362
ss86317131		4	103.106041	0.1597	0.4577	0.498912		0.0167	0.0044699377
ss86328189		4	103.159292	0.1605	1.2134	0.271031		0.0272	0.0073296602
ss117967519		4	103.278069	0.2400	4.0997	0.043273		0.0433	0.0157867293
ss61465807		4	103.334333	0.1576	0.1640	0.685657		0.0103	0.0027246625
ss86341368		4	103.370909	0.8424	0.1640	0.685657		0.0103	0.0027246625
ss117967176		4	103.395989	0.5867	3.8664	0.049658		0.0382	0.0185249785
ss61468904	*rs41573214*	4	103.444965	0.7070	4.7212	0.030130		0.0432	0.0178940237
ss86336915		4	103.493279	0.8202	3.9844	0.046313		0.0465	0.0137023000
ss61554868	*rs41647909*	4	103.534136	0.1762	5.2997	0.021623		0.0539	0.0156632459
ss86336791		4	103.598601	0.6583	1.4024	0.236721		0.0236	0.0106011614
ss86340536		4	103.636164	0.6168	0.4978	0.480701		0.0133	0.0062655664
ss61554962	*rs41649301*	4	103.671058	0.1246	4.9063	0.027082		0.0629	0.0137171039

	rs29027076	4	103.691147	0.5688	0.0865	0.768751	0.0055	0.0026813036
ss61554958	*rs41649297*	4	103.715724	0.3317	0.1597	0.689579	0.0079	0.0034827759
ss86282135		4	103.739341	0.5251	3.4786	0.062590	0.0359	0.0178834956
	rs29018244	4	103.861402	0.3410	4.4945	0.034358	0.0422	0.0189773916
	rs29011228	4	103.982433	0.2378	3.5901	0.058539	0.0419	0.0151792616
ss86324815		4	104.079987	0.1390	0.1683	0.681768	0.0110	0.0026428787
ss86319950		4	104.26084	0.5938	0.0624	0.802863	0.0047	0.0022590868
ss86314840		4	104.373988	0.4613	0.0571	0.811167	0.0044	0.0021930899
ss61554940	*rs41649280*	4	104.396726	0.6340	0.0894	0.765090	0.0057	0.0026461641
ss61554932	*rs41649272*	4	104.489864	0.2672	2.6604	0.103326	0.0341	0.0133702585
ss86325732		4	104.522968	0.9112	2.3618	0.124792	0.0495	0.0080070309
ss86314958		4	104.575404	0.5473	2.6526	0.103832	0.0309	0.0153128697
ss86311584		4	104.607495	0.5623	0.5642	0.452807	0.0139	0.0068425344
ss61554928	*rs41649268*	4	104.67198	0.0903	0.8258	0.363790	0.0286	0.0047029057
ss61554921	*rs41649262*	4	104.721041	0.6203	0.2945	0.587537	0.0107	0.0050288354
ss86308633		4	104.806864	0.7586	1.8680	0.172140	0.0303	0.0110832105
ss61554908	*rs41649249*	4	104.848333	0.1447	1.8366	0.175790	0.0343	0.0084874181
ss61554904	*rs41649245*	4	104.899816	0.2543	0.2041	0.651572	0.0097	0.0036933688
	rs29010271	4	104.945288	0.5509	5.9230	0.015195	0.0469	0.0231902127
ss86317200		4	104.976016	0.5566	6.2293	0.012796	0.0480	0.0237069865
ss86275296		4	105.001874	0.1583	0.0201	0.887164	0.0036	0.0009484789
ss61554878	*rs41647919*	4	105.182566	0.8825	2.8332	0.092782	0.0493	0.0102304939
ss86289811		4	105.252775	0.5064	0.0355	0.850538	0.0036	0.0018134435
ss86316681		4	105.308829	0.4370	0.5515	0.457955	0.0143	0.0070332726
ss86314920		4	105.339491	0.9441	1.2427	0.265336	0.0447	0.0047145416
ss61514354	*rs41610727*	4	105.440186	0.8517	1.1732	0.279117	0.0283	0.0071429380
ss61529934	*rs41630435*	4	105.766006	0.8653	0.2326	0.629720	0.0132	0.0030847169
ss61529931	*rs41630432*	4	105.814687	0.2221	0.7149	0.398102	0.0190	0.0065675530
ss61529930	*rs41630431*	4	105.873184	0.2342	0.8890	0.346080	0.0206	0.0073970934
ss86323105		4	105.894232	0.8668	0.0771	0.781353	0.0077	0.0017688717
ss86339220		4	105.942246	0.6397	9.0437	0.002731	0.0571	0.0263132417
ss86339725		4	106.034361	0.0795	0.0838	0.772271	0.0101	0.0014749140
ss86341055		4	106.071945	0.4742	1.8094	0.179019	0.0249	0.0124053740
ss86280503		4	106.092621	0.5996	2.3091	0.129070	0.0285	0.0136953776
ss86302532		4	106.177066	0.2020	2.5065	0.113834	0.0363	0.0117150022
ss86301052		4	106.212719	0.2170	2.6363	0.104897	0.0357	0.0121413912
ss86337829		4	106.237931	0.1347	1.0538	0.304990	0.0273	0.0063669654
ss86311152		4	106.258727	0.1898	3.2147	0.073414	0.0422	0.0129691819
ss61562577	*rs41662774*	4	106.32555	0.8854	1.4252	0.232964	0.0341	0.0069143764
ss61468924	*rs41566037*	4	106.423778	0.2994	5.0939	0.024320	0.0457	0.0191575304
ss61555051	*rs41650488*	4	106.501045	0.8904	1.1396	0.286106	0.0313	0.0061185808
ss61468921	*rs41566034*	4	106.626334	0.8116	0.0470	0.828411	0.0051	0.0015642198
	rs29010048	4	106.698261	0.8023	9.3004	0.002378	0.0720	0.0228345318
ss86300143		4	106.730145	0.7930	0.0903	0.763937	0.0071	0.0023375368
ss86304367		4	106.764604	0.5630	0.0479	0.826796	0.0041	0.0020017906
ss86341327		4	106.801743	0.6626	1.9726	0.160618	0.0280	0.0124995851
ss61554970	*rs41649309*	4	106.826148	0.4205	5.2654	0.022050	0.0440	0.0214228747

ss86284330		4	106.883634	0.3288	1.9192	0.166391		0.0279	0.0123207434
ss117967374		4	106.961852	0.3560	0.0425	0.836753		0.0039	0.0018100133
ss61554973	*rs41649312*	4	107.042059	0.4405	1.3572	0.244430		0.0221	0.0108944983
ss86337579		4	107.07136	0.4842	2.5552	0.110386		0.0311	0.0155558181
ss86336867		4	107.092603	0.8152	7.2770	0.007154		0.0650	0.0195920158
ss86288831		4	107.126535	0.5924	2.2697	0.132377		0.0282	0.0136419792
	rs29013402	4	107.155361	0.3309	1.5817	0.208943		0.0248	0.0109871780
ss86340049		4	107.272181	0.2085	0.0663	0.796862		0.0059	0.0019606592
ss86338311		4	107.379671	0.4549	3.7752	0.052419		0.0373	0.0185193502
ss61468914	*rs41573224*	4	107.402256	0.2851	12.8310	0.000365		0.0746	0.0304240200
ss86320768		4	107.435328	0.5158	1.7142	0.190869		0.0242	0.0121030610
ss46526399	*rs41256864*	4	107.519988	0.5451	6.0678	0.014007		0.0455	0.0225497081
ss86301026		4	107.641867	0.9348	0.2665	0.605838		0.0199	0.0024254182
ss86336091		4	107.675833	0.7235	0.5567	0.455851		0.0154	0.0061465195
ss86335519		4	107.700847	0.0695	0.0688	0.793125		0.0095	0.0012337784
ss86307363		4	107.768243	0.8245	0.0508	0.821788		0.0054	0.0015553975
ss86299108		4	108.012523	0.6648	1.1266	0.288862		0.0204	0.0091089596
ss86318070		4	108.042533	0.8560	0.0075	0.931052		0.0023	0.0005748133
ss86339266		4	108.082811	0.1175	0.2771	0.598743		0.0152	0.0031568110
ss86335607		4	108.121013	0.1404	0.4416	0.506584		0.0179	0.0043210340
ss86338882		4	108.167119	0.4513	1.6207	0.203425		0.0247	0.0122182498
ss86315725		4	108.366036	0.4491	4.3172	0.038094		0.0388	0.0192049430
ss61555017	*rs41650455*	4	108.401013	0.1132	0.8619	0.353521		0.0264	0.0053087157
ss117967195		4	108.421151	0.4248	0.2567	0.612575		0.0095	0.0046233313
		4	108.443272	0.4334	0.1590	0.690187		0.0075	0.0037045604
ss86300611		4	108.468144	0.2400	10.3342	0.001366		0.0716	0.0261182747
ss86304836		4	108.605181	0.8281	4.4042	0.036209		0.0533	0.0151630233
ss117974978		4	108.660872	0.2708	2.1513	0.142905		0.0303	0.0119647356
ss86280147		4	108.694252	0.4470	1.8691	0.172023		0.0256	0.0126569301
ss86332001		4	108.844692	0.2006	0.0881	0.766711		0.0068	0.0021764713
ss86307426		4	108.884659	0.7980	0.0454	0.831250		0.0049	0.0015702314
ss86331969		4	109.102807	0.7235	0.6207	0.431067		0.0167	0.0066655303
ss86302318		4	109.164086	0.5466	15.0192	0.000116		0.0728	0.0360792134
ss86309259		4	109.186461	0.2557	0.4054	0.524514		0.0132	0.0050070335
	rs29022854	4	109.231938	0.1970	1.1693	0.279928		0.0249	0.0078929079
ss61554997	*rs41650436*	4	109.293494	0.1948	0.3402	0.559874		0.0135	0.0042492662
ss86335171		4	109.314081	0.4234	1.5799	0.209190		0.0242	0.0117919150
ss86284920		4	109.607343	0.3532	2.6388	0.104732		0.0319	0.0145597118
ss86299631		4	109.839273	0.5981	3.2358	0.072477		0.0336	0.0161494858
ss86324609		4	110.022607	0.4255	9.2287	0.002472		0.0555	0.0271319800
ss117967507		4	110.06308	0.5903	5.9896	0.014637		0.0449	0.0217001566
ss61490346	*rs41591555*	4	110.097549	0.2779	0.6753	0.411485		0.0169	0.0067632980
ss86293194		4	110.146154	0.8582	1.1168	0.290975		0.0285	0.0069290478
ss61522475	*rs41619849*	4	110.172404	0.3510	5.8781	0.015584		0.0464	0.0211531003
ss61555042		4	110.277457	0.1160	1.7512	0.186159		0.0371	0.0076063893
ss86333337		4	110.40431	0.2135	0.0001	0.990303		0.0003	0.0000902540
ss86318042		4	110.540812	0.1046	0.0528	0.818389		0.0071	0.0013331834

ss86335544		4	110.603388	0.6404	3.4795	0.062552	0.0370	0.0170308431
ss61555010	*rs41650449*	4	110.715865	0.8847	1.1935	0.274998	0.0320	0.0065228823
ss86335796		4	110.748287	0.7242	0.0039	0.950392	0.0013	0.0005032402
ss86338989		4	110.778413	0.6318	7.9703	0.004891	0.0533	0.0247877455
ss86338091		4	110.856143	0.4950	9.0068	0.002786	0.0544	0.0272105877
ss61554995		4	110.900952	0.3653	7.4343	0.006560	0.0515	0.0238972795
ss86290480		4	110.933741	0.6117	10.1962	0.001471	0.0582	0.0276327797
ss86318276		4	110.965357	0.7235	5.5139	0.019145	0.0480	0.0191879509
ss86292096		4	111.135847	0.0967	1.0092	0.315452	0.0322	0.0056252400
ss86317493		4	111.165392	0.0881	2.0824	0.149460	0.0466	0.0074827780
ss61510342	*rs41603684*	4	111.186895	0.2966	5.9963	0.014581	0.0495	0.0206495500
ss61511421	*rs41612738*	4	111.327098	0.8703	0.2791	0.597448	0.0149	0.0033682406
ss86315076		4	111.350422	0.3252	10.9054	0.001008	0.0648	0.0284583292
ss117967470		4	111.399075	0.4341	2.8515	0.091735	0.0302	0.0148181654
ss86322374		4	111.422402	0.6683	1.3232	0.250418	0.0226	0.0100136693
ss61555013	*rs41650452*	4	111.515378	0.9298	2.3969	0.122033	0.0565	0.0073782450
ss61475389	*rs41574469*	4	111.545101	0.3130	7.7669	0.005466	0.0552	0.0237615215
ss86316311		4	111.584899	0.8152	2.1884	0.139505	0.0354	0.0106675930
ss61510363	*rs41603704*	4	111.659295	0.7070	0.8381	0.360271	0.0184	0.0076257995
ss86282717		4	111.793575	0.8438	4.5346	0.033567	0.0543	0.0143159136
ss86287715		4	111.916517	0.3832	2.5618	0.109926	0.0305	0.0144222952
ss86308902		4	111.954676	0.6418	4.9508	0.026399	0.0431	0.0198313764
ss86339528		4	112.055669	0.9169	3.7177	0.054245	0.0655	0.0099832323
ss61490355	*rs41591564*	4	112.100667	0.1383	1.1050	0.293546	0.0283	0.0067408248
ss86303132		4	112.133333	0.0322	0.1368	0.711623	0.0191	0.0011901680
ss86303056		4	112.181264	0.1160	0.7218	0.395856	0.0249	0.0051007362
ss86337586		4	112.214063	0.8553	0.1350	0.713449	0.0099	0.0024607431
ss86300376		4	112.250879	0.7693	0.0998	0.752112	0.0068	0.0024129226
ss61555087		4	112.275052	0.8159	3.9790	0.046461	0.0487	0.0146375007
ss61555098	*rs41651634*	4	112.321308	0.5029	1.9537	0.162639	0.0259	0.0129384835
ss61514310	*rs41610683*	4	112.675286	0.5895	0.2816	0.595839	0.0098	0.0047484238
ss61514307	*rs41610680*	4	112.700198	0.9226	1.7953	0.180714	0.0461	0.0065812349
ss86338829		4	112.725287	0.8188	0.0376	0.846251	0.0046	0.0013746931
ss86313192		4	112.841938	0.8288	0.1433	0.705092	0.0092	0.0026086835
ss61555110	*rs41651646*	4	113.042167	0.3309	1.3936	0.238204	0.0227	0.0100346164
ss86279423		4	113.28174	0.7307	5.2181	0.022654	0.0463	0.0182229393
ss86302915		4	113.625038	0.7615	0.1317	0.716789	0.0078	0.0028485886
ss65331388	*rs43419390*	4	113.786532	0.3567	3.4484	0.063733	0.0357	0.0163746359
ss61468938	*rs41566051*	4	113.822987	0.2593	0.0122	0.911939	0.0024	0.0009048056
ss86313018		4	113.888781	0.4914	0.2252	0.635281	0.0092	0.0045769983
	rs29025506	4	114.094438	0.3789	7.8933	0.005101	0.0515	0.0242411180
ss61510367	*rs41603708*	4	114.135856	0.3231	3.5296	0.060698	0.0375	0.0163855186
ss38327126	*rs29019238*	4	114.174316	0.9169	0.0064	0.936423	0.0027	0.0004125600
ss65331816	*rs43413618*	4	114.207583	0.8302	6.6413	0.010169	0.0630	0.0177682006
	rs29012198	4	114.244351	0.6511	2.0071	0.157011	0.0266	0.0120931390
ss65331981	*rs43415283*	4	114.27465	0.7937	2.9027	0.088879	0.0392	0.0128519400
ss117963724		4	114.307093	0.4799	0.1324	0.716054	0.0068	0.0033750131

ss65332108	*rs43415809*	4	114.396569	0.9312	7.7786	0.005431	0.0979	0.0125327198
ss86294393		4	114.48273	0.7464	1.3288	0.249407	0.0248	0.0093982043
ss117967467		4	114.530114	0.7923	7.7063	0.005651	0.0621	0.0204469117
ss61490404	*rs41591610*	4	114.575984	0.4140	0.0805	0.776647	0.0053	0.0025735118
ss86308799		4	114.618561	0.8610	0.9480	0.330570	0.0251	0.0060022852
ss86341142		4	114.640314	0.0315	2.8667	0.090878	0.0770	0.0046986183
ss65334917	*rs43416014*	4	114.678979	0.7235	3.4418	0.063990	0.0391	0.0156338912
	rs29027425	4	114.711306	0.7794	5.6577	0.017648	0.0529	0.0181810352
ss61555287	*rs41652816*	4	114.803443	0.6196	0.2919	0.589199	0.0103	0.0048567641
ss117967406		4	114.847964	0.5265	0.3794	0.538135	0.0115	0.0057513786
ss61567966	*rs41666464*	4	114.943619	0.5566	0.6171	0.432389	0.0145	0.0071465869
ss61567974	*rs41666472*	4	114.972512	0.4713	0.0816	0.775218	0.0052	0.0025879252
	rs29009830	4	115.013167	0.7450	0.1975	0.656895	0.0091	0.0034759992
ss117967409		4	115.086524	0.0731	0.1041	0.747064	0.0113	0.0015277917
ss64342253	*rs42593352*	4	115.126384	0.2443	0.0612	0.804608	0.0054	0.0019905703
ss117967464		4	115.178713	0.7364	0.3960	0.529377	0.0135	0.0052574373
ss64662767	*rs42761145*	4	115.223881	0.4943	1.2744	0.259325	0.0210	0.0104861379
ss86296842		4	115.262729	0.0831	5.1697	0.023287	0.0755	0.0115046794
ss64131665	*rs42493217*	4	115.29832	0.1862	1.0956	0.295601	0.0242	0.0073434283
ss64131631	*rs42493200*	4	115.321493	0.4033	2.7490	0.097769	0.0323	0.0155500571
ss64131521	*rs42493145*	4	115.36228	0.1956	1.2657	0.260971	0.0270	0.0084984289
ss117967453		4	115.385992	0.1347	0.1123	0.737665	0.0088	0.0020560756
	rs29026602	4	115.438256	0.4162	0.0366	0.848379	0.0036	0.0017401131
ss64131279	*rs42491625*	4	115.462026	0.4405	0.0022	0.963005	0.0009	0.0004203456
ss63705951	*rs42321535*	4	115.547518	0.4026	0.4763	0.490338	0.0132	0.0063712021
ss117967364		4	115.629647	0.5186	2.9202	0.087922	0.0314	0.0156868421
ss86306570		4	115.650551	0.6913	7.0684	0.008026	0.0508	0.0216680486
ss86327199		4	115.674382	0.6397	5.4636	0.019699	0.0447	0.0206223346
ss63704472	*rs42319641*	4	115.699819	0.2973	3.4034	0.065486	0.0366	0.0152867244
ss86308896		4	115.725375	0.2815	0.3884	0.533350	0.0127	0.0051214193
ss64773329	*rs42869203*	4	115.786536	0.1282	2.7603	0.097078	0.0456	0.0101903114
ss86318487		4	115.86414	0.8625	1.3839	0.239836	0.0306	0.0072622539
ss38330406	*rs29021912*	4	115.889323	0.6332	0.7296	0.393316	0.0168	0.0078068624
ss86339172		4	115.917735	0.3109	0.2816	0.595853	0.0105	0.0045167436
ss86291790		4	115.958626	0.7156	0.1380	0.710396	0.0074	0.0030306437
ss61499501	*rs43710689*	4	116.014501	0.0867	3.1781	0.075068	0.0595	0.0094268419
ss86324094		4	116.078251	0.6003	0.2734	0.601253	0.0097	0.0046471949
ss86305431		4	116.110117	0.2543	3.6331	0.057051	0.0398	0.0151119112
	rs29026543	4	116.13046	0.1805	0.1834	0.668580	0.0102	0.0030213886
ss86315536		4	116.187404	0.2944	0.2228	0.637055	0.0098	0.0040513256
ss117967226		4	116.241275	0.8087	0.2506	0.616801	0.0119	0.0036911571
ss86293085		4	116.299861	0.4599	0.0231	0.879283	0.0029	0.0014461278
ss61490409	*rs41591614*	4	116.419884	0.7185	5.0999	0.024237	0.0476	0.0192683366
ss86276381		4	116.459938	0.8603	9.1127	0.002631	0.0812	0.0195078900
ss86339202		4	116.500498	0.3231	0.4106	0.521872	0.0130	0.0056787252
ss46526771	*rs41257222*	4	116.537075	0.6956	0.3281	0.566963	0.0117	0.0049381448
ss61468945	*rs41566058*	4	116.568848	0.7658	4.3093	0.038272	0.0467	0.0167566098

ss61555282	*rs41652811*	4	116.594586	0.4620	0.0089	0.924665	0.0017	0.0008667316
ss86327371		4	116.629491	0.7213	0.1786	0.672732	0.0088	0.0035567438
ss86320156		4	116.68597	0.9026	0.0413	0.838943	0.0066	0.0011544973
ss61555285	*rs41652814*	4	116.80399	0.4785	0.0327	0.856629	0.0034	0.0016984398
ss86302003		4	116.870882	0.5451	3.6588	0.056183	0.0353	0.0175074049
ss86318003		4	116.911127	0.7686	1.7540	0.185811	0.0288	0.0102419443
ss86291346		4	116.951736	0.6476	2.7242	0.099289	0.0323	0.0147375721
ss86338200		4	116.973757	0.6669	2.3073	0.129219	0.0299	0.0132914987
ss86303659		4	116.998606	0.1511	0.5663	0.451977	0.0192	0.0049290609
ss86341143		4	117.025237	0.1519	0.5581	0.455265	0.0190	0.0049063680
ss86310783		4	117.048104	0.6984	0.9297	0.335278	0.0195	0.0082268921
ss61555256	*rs41652790*	4	117.07493	0.8546	0.4667	0.494743	0.0184	0.0045616670
ss86339119		4	117.101284	0.6010	2.3938	0.122269	0.0300	0.0143900684
ss117967450		4	117.126598	0.3961	2.4918	0.114893	0.0306	0.0146427824
ss117967184		4	117.205613	0.3789	0.1221	0.726926	0.0066	0.0030964588
ss86288392		4	117.357863	0.2242	4.5926	0.032456	0.0477	0.0165999664
ss61525863	*rs41623164*	4	117.54536	0.4284	0.1853	0.666988	0.0080	0.0039147919
	rs29011115	4	117.572536	0.2170	0.4681	0.494107	0.0154	0.0052179430
ss86340612		4	117.783906	0.4176	0.0318	0.858509	0.0033	0.0016186295
ss86301969		4	117.80802	0.5258	0.1018	0.749817	0.0061	0.0030480934
ss86297248		4	117.831339	0.7178	0.9727	0.324344	0.0203	0.0082241594
ss86316063		4	117.854165	0.4183	2.3962	0.122088	0.0283	0.0137624106
ss86340539		4	117.876014	0.2006	0.0066	0.935496	0.0018	0.0005882139
ss86338818		4	117.964927	0.9083	1.6161	0.204056	0.0408	0.0067913508
ss86340709		4	117.996499	0.6913	2.5036	0.114038	0.0312	0.0133084241
ss86326513		4	118.019013	0.3087	14.9258	0.000122	0.0774	0.0330357829
ss86320619		4	118.044403	0.4398	0.1998	0.655057	0.0084	0.0041333908
ss86322571		4	118.159261	0.5552	1.1613	0.281565	0.0201	0.0099353838
ss65337170	*rs43418684*	4	118.208859	0.9119	0.9167	0.338666	0.0312	0.0050103561
ss86331483		4	118.243168	0.6089	0.5815	0.445985	0.0144	0.0068468066
ss86295885		4	118.269976	0.5029	2.7490	0.097764	0.0307	0.0153353700
ss86341402		4	118.348424	0.0910	1.1284	0.288478	0.0345	0.0057075440
	rs29020849	4	118.400171	0.2844	2.4409	0.118663	0.0329	0.0133980193
ss86283864		4	118.472696	0.8539	0.0998	0.752121	0.0082	0.0020544478
ss61555230	*rs41652764*	4	118.508145	0.2307	0.0876	0.767290	0.0063	0.0022308982
ss86328470		4	118.545584	0.5007	1.1025	0.294089	0.0192	0.0095754494
ss86307406		4	118.636146	0.2421	0.3394	0.560365	0.0127	0.0046578659
ss86332775		4	118.668226	0.4155	0.5018	0.478947	0.0135	0.0065506310
ss86326990		4	118.701921	0.3446	2.7784	0.095993	0.0327	0.0147649371
ss65333925	*rs43414927*	4	118.846747	0.5186	0.0614	0.804424	0.0044	0.0022147820
ss61555291	*rs41652820*	4	118.955437	0.3997	0.8865	0.346749	0.0172	0.0082498155
ss61555289	*rs41652818*	4	118.98417	0.5731	9.3284	0.002342	0.0577	0.0282181720
ss65335087	*rs43416981*	4	119.007667	0.8739	1.8180	0.177985	0.0377	0.0083080179
ss61517735	*rs41618099*	4	119.032553	0.7450	0.7065	0.400894	0.0180	0.0068540127
ss86336535		4	119.056947	0.3159	1.8859	0.170105	0.0279	0.0120689227
ss61482608	*rs41579994*	4	119.161914	0.8918	0.0397	0.842031	0.0060	0.0011504924
ss86314619		4	119.189609	0.2765	0.2418	0.623063	0.0103	0.0041336224

ss62379179	*rs41860069*	4	119.244437	0.3317	0.4180	0.518153	0.0126	0.0055829594
ss86325428		4	119.272013	0.2772	0.2871	0.592275	0.0112	0.0044951428
ss86296462		4	119.32493	0.3052	1.3268	0.249773	0.0239	0.0101193679
ss86321412		4	119.37235	0.2364	0.3129	0.576067	0.0125	0.0045271984
ss64634424	*rs42737300*	4	119.399008	0.8625	6.5210	0.010873	0.0703	0.0166675955
ss86304600		4	119.423366	0.5731	1.3061	0.253486	0.0223	0.0109328698
ss86308301		4	119.489507	0.1619	4.2378	0.039905	0.0529	0.0143562005
ss61478877	*rs41578951*	4	119.506143	0.0802	1.9978	0.157978	0.0490	0.0072315177
ss61568884	*rs41667265*	4	119.681674	0.3481	0.0158	0.900069	0.0025	0.0011501639
ss86282619		4	119.703517	0.0824	1.2897	0.256494	0.0380	0.0057391255
ss64383573	*rs42611147*	4	119.764534	0.4792	0.0490	0.824851	0.0042	0.0020943338
ss64383613	*rs42611167*	4	119.79503	0.3266	1.7741	0.183310	0.0260	0.0114285985
ss86322656		4	119.827236	0.6712	1.4455	0.229668	0.0235	0.0103677149
ss117967320		4	119.861099	0.7536	1.9583	0.162140	0.0286	0.0106275276
	rs29022837	4	119.887118	0.3918	0.0309	0.860565	0.0033	0.0015717215
ss61517000	*rs41621197*	4	119.90772	0.1110	1.2185	0.270039	0.0317	0.0062496889
	rs29015028	4	119.955478	0.3345	1.4393	0.230655	0.0234	0.0104115817
ss117967206		4	120.098333	0.4291	0.5998	0.438899	0.0146	0.0071725504
ss61569307		4	120.140722	0.5702	0.6631	0.415754	0.0154	0.0075292506
ss117975433		4	120.171024	0.4807	0.0008	0.977528	0.0005	0.0002638965
ss86302070		4	120.316724	0.8009	0.5090	0.475812	0.0163	0.0052016448
ss86326605		4	120.425263	0.2378	0.2366	0.626854	0.0105	0.0038003630
ss117967510		4	120.452314	0.2937	0.0001	0.993046	0.0002	0.0000719649
ss61513293	*rs41611576*	4	120.479462	0.2128	0.0244	0.875845	0.0035	0.0011850825
ss64487020	*rs42659260*	4	120.510193	0.6791	6.1799	0.013155	0.0501	0.0218322826
ss64487186	*rs42659843*	4	120.569436	0.8940	0.9215	0.337424	0.0283	0.0053591780
ss86310978		4	120.624969	0.4334	2.2258	0.136172	0.0281	0.0138003957
ss86314063		4	120.652172	0.3775	0.0046	0.946146	0.0012	0.0005833959
ss86289531		4	120.699997	0.5638	0.0002	0.988205	0.0003	0.0001347937
ss86274013		4	120.746156	0.5244	0.0051	0.942978	0.0013	0.0006553056
ss86314906		4	120.793493	0.5766	2.8198	0.093558	0.0303	0.0148114565
ss86303722		4	120.843656	0.4778	0.0035	0.952669	0.0011	0.0005516830
ss65336620	*rs43420430*	4	120.866044	0.6160	7.6964	0.005681	0.0533	0.0252123704
ss86331570		4	120.889471	0.2242	4.2483	0.039660	0.0458	0.0159428719
ss86338869		4	120.930703	0.3173	10.2315	0.001443	0.0629	0.0272365720
	rs29019697	4	120.976228	0.3811	0.8846	0.347271	0.0184	0.0086761567
ss86274256		4	120.997548	0.1318	0.6188	0.431772	0.0219	0.0050057020
ss86337227		4	121.023742	0.5351	0.6063	0.436438	0.0143	0.0071269298
	rs29025578	4	121.082429	0.0623	0.0014	0.970364	0.0014	0.0001607582
ss86333685		4	121.118369	0.3854	0.0213	0.884068	0.0028	0.0013492312
ss86313798		4	121.155952	0.5946	0.9102	0.340400	0.0181	0.0087271878
ss86304300		4	121.240468	0.4957	2.7395	0.098348	0.0316	0.0157855649
ss61569106	*rs41664794*	4	121.365407	0.4857	0.6259	0.429141	0.0149	0.0074413233
ss86336020		4	121.386641	0.5208	0.3100	0.577839	0.0103	0.0051230655
ss86306107		4	121.406973	0.3754	3.1501	0.076357	0.0343	0.0160865167
ss86340894		4	121.44349	0.1454	3.3112	0.069237	0.0474	0.0117741767
ss86331005		4	121.4668	0.9062	0.0243	0.876179	0.0050	0.0008461852

ss63659193	*rs42307234*	4	121.496305	0.8080	0.4651	0.495476	0.0160	0.0049506917
ss61497014	*rs41593945*	4	121.525513	0.3610	0.1431	0.705300	0.0072	0.0033177754
ss86313289		4	121.547246	0.4628	0.1059	0.744925	0.0060	0.0029894792
ss86290165		4	121.620897	0.5021	0.7317	0.392629	0.0158	0.0079091685
ss63660242	*rs42299884*	4	121.700062	0.1784	1.3413	0.247204	0.0275	0.0080530607
ss86297792		4	121.73256	0.0595	0.5481	0.459357	0.0293	0.0032790730
ss61470010	*rs41566007*	4	121.772583	0.3438	1.2651	0.261078	0.0220	0.0099420099
ss117963751		4	121.802945	0.7514	0.0100	0.920463	0.0021	0.0007784093
ss86323785		4	121.851048	0.1540	1.3744	0.241455	0.0296	0.0077011611
ss86299272		4	121.91691	0.5408	3.1263	0.077475	0.0322	0.0159896788
ss86305812		4	121.990265	0.7400	6.2491	0.012655	0.0510	0.0196402260
ss117967505		4	122.050101	0.8625	0.4087	0.522839	0.0173	0.0041134630
ss65337515	*rs43422223*	4	122.073236	0.4019	0.0874	0.767576	0.0054	0.0026164325
ss86301007		4	122.097244	0.4620	0.3580	0.549794	0.0112	0.0055848568
	rs29023352	4	122.117992	0.4370	2.2488	0.134174	0.0282	0.0138627658
ss86340042		4	122.159188	0.2937	0.6506	0.420159	0.0159	0.0065896576
ss86314278		4	122.357511	0.4799	0.7513	0.386353	0.0163	0.0081153933
ss86338974		4	122.418701	0.6970	0.9832	0.321759	0.0196	0.0082947733
ss64234478	*rs42540403*	4	122.456664	0.8181	0.0019	0.965317	0.0011	0.0003174343
ss64234204	*rs42539167*	4	122.525712	0.3095	0.9614	0.327175	0.0194	0.0082717268
	rs29012231	4	122.588184	0.1927	0.2650	0.606861	0.0126	0.0039278280
ss86327601		4	122.639047	0.7135	3.4181	0.064908	0.0375	0.0153275749
ss86302542		4	122.666075	0.5057	5.7638	0.016621	0.0445	0.0222271818
ss86337416		4	122.70328	0.1347	1.2073	0.272240	0.0297	0.0069287207
ss86338341		4	122.756006	0.6003	0.9446	0.331440	0.0181	0.0086677036
ss86331595		4	122.782035	0.5487	0.2950	0.587193	0.0100	0.0049589412
ss86337047		4	122.853218	0.0738	1.1889	0.275939	0.0374	0.0051139120
ss86320005		4	122.894207	0.3080	1.8553	0.173614	0.0276	0.0117514980
ss65337199	*rs43418713*	4	122.922453	0.0552	1.0644	0.302568	0.0417	0.0043445355
ss86313568		4	122.950505	0.1468	2.3990	0.121871	0.0405	0.0101387720
ss86329412		4	123.008402	0.9133	0.3060	0.580345	0.0178	0.0028139460
ss65288149	*rs43373704*	4	123.03594	0.3388	7.1588	0.007635	0.0506	0.0226801960
ss86337330		4	123.058633	0.5251	0.5730	0.449344	0.0140	0.0069814979
ss86303754		4	123.116777	0.7593	0.0056	0.940632	0.0016	0.0005838301
ss86273532		4	123.148334	0.0910	0.3681	0.544243	0.0189	0.0031268997
ss86284248		4	123.171033	0.2385	0.1799	0.671570	0.0097	0.0035325514
ss86303478		4	123.191838	0.7120	3.5677	0.059330	0.0405	0.0166272639
ss61860111	*rs41667692*	4	123.226036	0.3976	0.0552	0.814356	0.0045	0.0021567297
ss86305158		4	123.252562	0.0766	2.0408	0.153579	0.0484	0.0068488244
ss86340124		4	123.302252	0.5165	0.9889	0.320353	0.0179	0.0089458387
ss86311857		4	123.334327	0.1461	1.1580	0.282255	0.0277	0.0069058888
ss86336010		4	123.369638	0.2421	2.9353	0.087106	0.0362	0.0132682027
ss86307688		4	123.425145	0.8761	1.5985	0.206539	0.0350	0.0075952221
ss86338597		4	123.458956	0.1669	0.0228	0.879996	0.0038	0.0010684674
ss86290218		4	123.482857	0.1819	0.0089	0.924726	0.0022	0.0006574325
ss86299383		4	123.58208	0.3166	0.2369	0.626622	0.0095	0.0041318691
ss86339578		4	123.632989	0.3918	0.1261	0.722665	0.0068	0.0032526844

ss86315837		4	123.728306	0.1984	1.8114	0.178774	0.0314	0.0099845150
ss86324046		4	123.807542	0.0745	0.0792	0.778447	0.0097	0.0013430679
ss86307052		4	123.831435	0.5996	1.7327	0.188498	0.0245	0.0117827005
ss86285632		4	123.906489	0.3532	0.2969	0.585981	0.0104	0.0047638071
ss86339701		4	123.941925	0.3145	0.2611	0.609558	0.0097	0.0041975997
ss86327985		4	123.973622	0.1153	0.0554	0.814046	0.0068	0.0013880671
ss86341205		4	124.015803	0.3223	7.5766	0.006067	0.0552	0.0241240678
ss117967331		4	124.072768	0.7514	1.5484	0.213790	0.0266	0.0099418094
ss86311471		4	124.098717	0.1504	3.5326	0.060592	0.0491	0.0125502964
ss64394240	*rs42620770*	5	0.00853	0.6168	0.0537	0.816855	0.0045	0.0021176611
ss61497074	*rs41594004*	5	0.0434	0.5595	0.7332	0.392148	0.0162	0.0079785401
ss86318760		5	0.0742	0.3947	0.1692	0.680963	0.0078	0.0037487262
ss64393992	*rs42620146*	5	0.0965	0.3947	0.1692	0.680963	0.0078	0.0037487262
ss64729038	*rs42828033*	5	0.288685	0.4692	0.1275	0.721123	0.0066	0.0032788687
ss64729080	*rs42828075*	5	0.479278	0.1332	1.7153	0.190738	0.0356	0.0082161101
ss61563944	*rs41665108*	5	0.653701	0.8653	0.5550	0.456515	0.0199	0.0046424754
ss61525121	*rs41626147*	5	0.700392	0.7271	0.0082	0.927941	0.0018	0.0007333387
ss61525120	*rs41626146*	5	0.720681	0.1440	0.0217	0.882824	0.0038	0.0009464368
ss61516975	*rs41621172*	5	0.757437	0.6032	0.0284	0.866210	0.0032	0.0015397784
ss64958263	*rs43050319*	5	0.858422	0.8625	7.6493	0.005830	0.0751	0.0178103011
ss86321156		5	0.88064	0.3138	0.1110	0.739108	0.0067	0.0028724739
ss64677335	*rs42776099*	5	0.902217	0.1999	0.5216	0.470388	0.0161	0.0051622897
ss61516973	*rs41621170*	5	0.962456	0.8159	0.0035	0.952707	0.0014	0.0004267821
ss86309687		5	0.985518	0.1848	3.4597	0.063304	0.0430	0.0129629856
ss64677359	*rs42776123*	5	1.043745	0.1254	0.1616	0.687809	0.0114	0.0025040015
ss64753387	*rs42849506*	5	1.066709	0.5688	5.5055	0.019236	0.0430	0.0210837190
ss64753356	*rs42849475*	5	1.104259	0.4506	3.2046	0.073865	0.0327	0.0162040966
ss86317091		5	1.166618	0.8911	0.4936	0.482547	0.0204	0.0039575914
ss63484073	*rs42244571*	5	1.237388	0.4448	0.0780	0.780156	0.0053	0.0026314249
ss63484034	*rs42244558*	5	1.293419	0.1232	3.4811	0.062493	0.0520	0.0112383602
ss61525379	*rs41620404*	5	1.316258	0.1612	1.1708	0.279606	0.0262	0.0070763953
ss65104286	*rs43189388*	5	1.437818	0.1841	1.5846	0.208517	0.0300	0.0089997357
ss63483629	*rs42252721*	5	1.481094	0.7092	0.5544	0.456789	0.0150	0.0062020738
ss63483245	*rs42251493*	5	1.541265	0.7285	0.4477	0.503642	0.0139	0.0055157076
ss61521561	*rs41620464*	5	1.684228	0.5458	2.4797	0.115779	0.0294	0.0145535563
ss86335150		5	1.792059	0.2178	0.0003	0.985317	0.0004	0.0001433424
ss63903610	*rs42020386*	5	1.851325	0.1605	5.8057	0.016233	0.0619	0.0166826095
ss86283736		5	1.879281	0.2937	2.1323	0.144679	0.0301	0.0124897904
ss117967858		5	1.968822	0.6476	1.8980	0.168740	0.0272	0.0123978063
ss64944189	*rs43039081*	5	2.027096	0.5931	2.0516	0.152493	0.0270	0.0130454711
ss61519434	*rs41614684*	5	2.055695	0.8130	4.2342	0.039990	0.0493	0.0149891477
ss61498699	*rs43706104*	5	2.078935	0.7170	0.5991	0.439201	0.0160	0.0064902409
ss64477804	*rs42659994*	5	2.161915	0.3453	1.5829	0.208765	0.0238	0.0107546172
ss64477708	*rs42659946*	5	2.190509	0.3395	0.2825	0.595227	0.0105	0.0047179461
ss65089258	*rs43180367*	5	2.258052	0.6590	1.5386	0.215238	0.0235	0.0105709408
ss65337968	*rs43428062*	5	2.300468	0.7486	0.0001	0.993713	0.0002	0.0000644314
ss86339668		5	2.344369	0.7428	0.1822	0.669579	0.0094	0.0035931868

ss64239772	*rs42539832*	5	2.421286	0.8646	4.4965	0.034318	0.0569	0.0133282034
ss61565604	*rs41662351*	5	2.451715	0.6168	0.7898	0.374472	0.0164	0.0077513860
ss61495340	*rs41595916*	5	2.497546	0.0566	2.0154	0.156156	0.0552	0.0058922684
ss61565603	*rs41662350*	5	2.535881	0.7701	0.3845	0.535433	0.0137	0.0048402702
ss65338005	*rs43428099*	5	2.572531	0.0974	1.7852	0.181946	0.0409	0.0071976801
ss65338037	*rs43429131*	5	2.595078	0.3639	0.1936	0.660088	0.0084	0.0038784216
ss61556142	*rs41651454*	5	2.690256	0.4413	1.7484	0.186517	0.0244	0.0120484162
ss65067779	*rs43157783*	5	2.731742	0.3890	0.0045	0.946452	0.0013	0.0006082160
ss61513038	*rs41609836*	5	2.783729	0.7543	0.3430	0.558272	0.0124	0.0045886211
ss61513026	*rs41609624*	5	2.808798	0.4262	0.0068	0.934170	0.0016	0.0007690640
ss64834697	*rs42932350*	5	2.874511	0.1819	4.0526	0.044488	0.0499	0.0148646434
ss65338280	*rs43420065*	5	2.943183	0.9262	5.2375	0.022404	0.0810	0.0110664037
ss86322588		5	3.320426	0.4950	3.5562	0.059739	0.0350	0.0175060585
ss86319695		5	3.498352	0.3252	3.1352	0.077055	0.0347	0.0152455742
ss61467011	*rs41565247*	5	3.563151	0.6046	5.4357	0.020014	0.0425	0.0203016645
ss64112387	*rs42479101*	5	3.58419	0.2693	3.3594	0.067251	0.0387	0.0152309000
ss64112401	*rs42479108*	5	3.60706	0.2486	3.1092	0.078291	0.0392	0.0146542797
ss61531361	*rs41634015*	5	3.639813	0.8832	0.5739	0.448977	0.0219	0.0045241052
ss61530599	*rs41626670*	5	3.71	0.4019	1.0109	0.315032	0.0188	0.0090149417
ss61524487	*rs41620726*	5	3.738566	0.2385	0.1561	0.692859	0.0084	0.0030666858
ss64112763	*rs42480979*	5	3.784893	0.4670	0.6777	0.410661	0.0148	0.0073757459
	rs29026845	5	3.837868	0.5559	0.0584	0.809119	0.0045	0.0022217228
ss63008649	*rs42070565*	5	3.858443	0.4348	1.0327	0.309885	0.0186	0.0091502102
ss63008811	*rs42070619*	5	3.897847	0.6999	0.5684	0.451141	0.0151	0.0063643477
	rs29023737	5	3.951318	0.7328	0.0903	0.763857	0.0063	0.0024542879
ss117967563		5	4.008642	0.4979	0.4342	0.510149	0.0120	0.0060160088
ss63009324	*rs42072090*	5	4.090048	0.5788	0.0981	0.754263	0.0060	0.0029421068
ss61548568	*rs41650871*	5	4.129013	0.7758	0.5889	0.443090	0.0177	0.0061565083
ss117967894		5	4.206051	0.7199	0.3176	0.573220	0.0116	0.0046787381
ss65338688	*rs43424770*	5	4.235716	0.2199	0.0272	0.869022	0.0036	0.0012486928
	rs29019196	5	4.343366	0.3883	0.1361	0.712294	0.0069	0.0032623337
ss86309591		5	4.384602	0.6168	5.1498	0.023555	0.0424	0.0200637643
ss63010398	*rs42066717*	5	4.407672	0.4040	0.0035	0.952838	0.0011	0.0005245193
ss86341299		5	4.475257	0.9040	2.0093	0.156784	0.0448	0.0077735938
ss86313062		5	4.528723	0.6361	2.0249	0.155188	0.0269	0.0124619316
ss61475112	*rs41571503*	5	4.627084	0.2213	2.4723	0.116320	0.0365	0.0125716219
ss65339328	*rs43421982*	5	4.649042	0.2643	2.5008	0.114240	0.0334	0.0129776665
ss117967864		5	4.67108	0.0831	0.3190	0.572384	0.0190	0.0028909888
ss86322878		5	4.694788	0.2192	2.3109	0.128922	0.0342	0.0116928583
ss61490514	*rs41588020*	5	4.755368	0.5358	0.7942	0.373126	0.0167	0.0083269655
ss65338849	*rs43426731*	5	4.786395	0.8775	0.0698	0.791684	0.0076	0.0016356119
ss61555438	*rs41654759*	5	4.815612	0.3739	1.7883	0.181574	0.0260	0.0121746456
ss86322236		5	4.85649	0.4979	0.0418	0.838048	0.0038	0.0019022269
ss86285495		5	4.909774	0.1977	0.2546	0.614027	0.0119	0.0037825920
ss61532090	*rs41632524*	5	4.963141	0.7550	0.1939	0.659796	0.0096	0.0035463870
ss64370141	*rs42605002*	5	4.987905	0.9448	0.1379	0.710462	0.0154	0.0016063976
ss64370277	*rs42605669*	5	5.054654	0.2242	0.0385	0.844402	0.0043	0.0014816457

ss61562468		5	5.155187	0.7128	3.4662	0.063056	0.0379	0.0155315180
ss86306901		5	5.294127	0.3181	0.5433	0.461317	0.0146	0.0063127970
ss61555536	*rs41655855*	5	5.331063	0.9520	0.0586	0.808817	0.0107	0.0009756277
ss86335370		5	5.402408	0.5079	1.8656	0.172425	0.0266	0.0133043717
ss65340130	*rs43428177*	5	5.490457	0.0989	0.3895	0.532770	0.0194	0.0034481480
ss65340034	*rs43427285*	5	5.572163	0.3983	4.9048	0.027105	0.0413	0.0198024694
ss61510415	*rs41604543*	5	5.59539	0.8696	0.0301	0.862211	0.0048	0.0010896758
ss65339859	*rs43425810*	5	5.695186	0.5860	0.0182	0.892803	0.0025	0.0012160876
ss61490577	*rs41589082*	5	5.720724	0.7643	7.3957	0.006701	0.0607	0.0218855833
ss61490566	*rs41589071*	5	5.857305	0.2615	0.9826	0.321896	0.0204	0.0078836861
ss86321277		5	5.883056	0.8274	3.4233	0.064707	0.0434	0.0123855413
ss65339560	*rs43423711*	5	5.933753	0.1834	1.4450	0.229747	0.0289	0.0086704015
ss65339618	*rs43424269*	5	5.960399	0.1777	2.0795	0.149735	0.0349	0.0101967964
ss61490557	*rs41589062*	5	5.988015	0.8216	1.7928	0.181020	0.0324	0.0094917629
ss63799936	*rs42354308*	5	6.014387	0.5351	2.5231	0.112643	0.0298	0.0148311282
ss61516692	*rs41617993*	5	6.038134	0.8997	0.0021	0.963704	0.0014	0.0002583174
ss86307461		5	6.063438	0.7908	0.0093	0.923048	0.0022	0.0007404407
ss86337639		5	6.098049	0.5509	0.1598	0.689496	0.0076	0.0037757539
ss64810458	*rs42912687*	5	6.123543	0.1798	0.1168	0.732580	0.0083	0.0024391466
ss61465777	*rs41569021*	5	6.270311	0.0645	0.0004	0.983742	0.0008	0.0000930653
ss63799690	*rs42361784*	5	6.297071	0.5322	3.9404	0.047532	0.0378	0.0188452828
ss61465775	*rs41569019*	5	6.337073	0.0638	0.0010	0.975314	0.0012	0.0001404913
ss64619390	*rs42733108*	5	6.418359	0.8840	3.5482	0.060025	0.0551	0.0113128700
ss117967919		5	6.528588	0.3123	1.4351	0.231339	0.0234	0.0100596360
ss117963800		5	6.579675	0.0652	0.7216	0.395904	0.0320	0.0039037480
ss117967697		5	6.666949	0.1390	0.0665	0.796557	0.0068	0.0016313668
ss61490786	*rs41590791*	5	6.688967	0.7264	1.4416	0.230283	0.0241	0.0095954593
ss46526976	*rs41257422*	5	6.713153	0.3181	0.0669	0.796046	0.0051	0.0022181238
ss117967574		5	6.74448	0.2966	0.3514	0.553532	0.0117	0.0048849403
ss86319613		5	6.803754	0.4957	0.7936	0.373335	0.0164	0.0082063282
ss86339532		5	6.828002	0.6648	1.5346	0.215839	0.0243	0.0108324661
ss61555925	*rs41648938*	5	6.908133	0.6905	0.7667	0.381538	0.0174	0.0074345814
ss65341804	*rs43425658*	5	6.940606	0.8890	3.8495	0.050160	0.0572	0.0112898802
ss65341771	*rs43425225*	5	6.974444	0.2987	0.3267	0.567784	0.0115	0.0048280201
ss65341720	*rs43425174*	5	6.996301	0.7120	0.1834	0.668572	0.0087	0.0035736115
ss86305094		5	7.030849	0.1984	0.4298	0.512313	0.0151	0.0048004693
ss61555901	*rs41647814*	5	7.062982	0.4011	0.4772	0.489944	0.0131	0.0062928940
ss65341635	*rs43424389*	5	7.101805	0.9262	1.1247	0.289284	0.0385	0.0052557407
ss65341483	*rs43424137*	5	7.180111	0.1232	0.0161	0.899195	0.0036	0.0007876793
ss86303693		5	7.231689	0.8152	1.2668	0.260752	0.0271	0.0081630624
ss61555856	*rs41647771*	5	7.280834	0.8009	0.2790	0.597530	0.0119	0.0037804730
ss61490714		5	7.356008	0.2615	0.4344	0.510078	0.0144	0.0055438206
ss86328085		5	7.38462	0.9635	0.5342	0.465094	0.0364	0.0025619188
ss86279902		5	7.419295	0.3030	0.2763	0.599290	0.0103	0.0043712416
ss86337325		5	7.473431	0.3696	1.3350	0.248318	0.0224	0.0104355970
ss61555796		5	7.528994	0.7736	0.4371	0.508738	0.0151	0.0052765272
ss61556057	*rs41650269*	5	7.569039	0.8352	3.3609	0.067188	0.0456	0.0125552914

ss86330499		5	7.625517	0.7908	7.3721	0.006789	0.0617	0.0203979064
ss64875558	*rs42967439*	5	7.751535	0.7693	6.3364	0.012052	0.0556	0.0197234974
ss65340657	*rs43424711*	5	7.79338	0.4212	9.5794	0.002047	0.0583	0.0284213626
ss65340556	*rs43424011*	5	7.840016	0.0702	7.3910	0.006718	0.0980	0.0127881487
ss61555760	*rs41657458*	5	7.864147	0.2457	3.9524	0.047195	0.0419	0.0155192003
ss86275097		5	7.897917	0.1089	0.8506	0.356696	0.0275	0.0053402486
ss86307172		5	8.051431	0.5179	2.0239	0.155288	0.0255	0.0127127321
	rs29010908	5	8.150823	0.4427	2.0975	0.147990	0.0262	0.0129390452
ss64675535	*rs42774123*	5	8.203733	0.3173	1.9923	0.158548	0.0274	0.0118766110
ss61506495	*rs41606105*	5	8.237367	0.7593	0.1222	0.726754	0.0074	0.0027013313
	rs29023586	5	8.26526	0.7120	0.2566	0.612600	0.0106	0.0043308249
ss61531516	*rs41626972*	5	8.296806	0.1540	1.5005	0.221014	0.0305	0.0079351682
ss64117221	*rs42485601*	5	8.33959	0.7421	7.7919	0.005392	0.0594	0.0227202122
ss64117237	*rs42485609*	5	8.455044	0.1082	0.9281	0.335684	0.0286	0.0055183001
ss64414790	*rs42632865*	5	8.565017	0.1318	0.3901	0.532478	0.0171	0.0039045848
ss61497293	*rs41595322*	5	8.589431	0.3073	4.2131	0.040485	0.0414	0.0176174301
ss65344335	*rs43426079*	5	8.730302	0.6991	2.1611	0.141995	0.0305	0.0128262579
ss86319254		5	8.793355	0.5659	0.3206	0.571415	0.0108	0.0053230342
ss61556610	*rs41655514*	5	8.849759	0.7572	1.0786	0.299377	0.0225	0.0082837142
ss86337689		5	8.894773	0.7980	2.3700	0.124143	0.0354	0.0114122786
ss64769890	*rs42866127*	5	8.923235	0.0888	4.3095	0.038267	0.0685	0.0110954501
ss65344676	*rs43429617*	5	8.959436	0.7736	1.5834	0.208699	0.0276	0.0096807549
ss117963803		5	9.087185	0.7077	0.5745	0.448752	0.0153	0.0063270991
ss61510471	*rs41604598*	5	9.107822	0.6941	1.8128	0.178617	0.0274	0.0116192300
ss86300090		5	9.1457	0.8911	3.6388	0.056858	0.0555	0.0107751294
ss86308522		5	9.170731	0.5860	1.1942	0.274856	0.0204	0.0099021380
ss86291917		5	9.208312	0.5487	16.3228	0.000059	0.0810	0.0401362317
ss61556149	*rs41651461*	5	9.301476	0.9119	0.1637	0.685854	0.0131	0.0021022844
ss65342558	*rs43426012*	5	9.335896	0.0759	1.2657	0.260970	0.0399	0.0055951984
ss117963829		5	9.36849	0.5430	5.2883	0.021764	0.0432	0.0214566785
ss65342724	*rs43427878*	5	9.411764	0.6519	2.8949	0.089306	0.0327	0.0148462929
ss86312469		5	9.445481	0.8968	2.4370	0.118956	0.0473	0.0087517671
ss61510502		5	10.097638	0.6705	1.6115	0.204705	0.0242	0.0107067993
ss86314488		5	10.134451	0.6218	0.0771	0.781283	0.0053	0.0024826991
ss86274927		5	10.164298	0.1691	0.1111	0.738975	0.0081	0.0022701620
ss86333642		5	10.200637	0.6533	3.2312	0.072683	0.0336	0.0152113157
ss86317447		5	10.277314	0.3782	1.6096	0.204970	0.0239	0.0112355775
ss117967914		5	10.445153	0.6963	0.0232	0.878962	0.0030	0.0012704946
ss86274047		5	10.484582	0.3381	4.3609	0.037137	0.0403	0.0180383402
ss86305473		5	10.533868	0.9205	1.5590	0.212231	0.0418	0.0061180230
ss117967596		5	10.579603	0.3481	0.3372	0.561656	0.0116	0.0052748101
ss86337712		5	10.657977	0.3274	4.6582	0.031246	0.0436	0.0191937061
ss86282539		5	10.733468	0.0788	0.8335	0.361571	0.0304	0.0044104024
ss61556480	*rs41654485*	5	10.757745	0.2128	0.2103	0.646710	0.0105	0.0035154617
ss86309542		5	10.803982	0.0809	0.5645	0.452715	0.0255	0.0037893925
ss117962453		5	10.846184	0.0809	0.5645	0.452715	0.0255	0.0037893925
ss65342968	*rs43429822*	5	10.905567	0.2880	0.2563	0.612824	0.0105	0.0043251899

ss65343109	*rs43431259*	5	11.055635	0.6877	2.0420	0.153460	0.0283	0.0121366079
ss86304694		5	11.079902	0.5322	11.2410	0.000843	0.0647	0.0322343184
ss61556305	*rs41652716*	5	11.110523	0.4928	0.9127	0.339741	0.0184	0.0091803277
ss117967827		5	11.139888	0.2020	0.4460	0.504470	0.0151	0.0048802746
ss65343306	*rs43425847*	5	11.217227	0.4341	6.6126	0.010332	0.0500	0.0245797353
ss65343337	*rs43425877*	5	11.276201	0.2020	0.6179	0.432095	0.0185	0.0059597146
ss65343399	*rs43426639*	5	11.304816	0.2020	0.6179	0.432095	0.0185	0.0059597146
ss65343401	*rs43426641*	5	11.327591	0.6640	5.7197	0.017040	0.0474	0.0211651070
ss61490928	*rs41592931*	5	11.350756	0.7915	0.2723	0.601991	0.0122	0.0040150524
ss61556406	*rs41653612*	5	11.372141	0.4054	2.7635	0.096886	0.0314	0.0151493115
ss61556410	*rs41653616*	5	11.407909	0.7980	0.6179	0.432095	0.0185	0.0059597146
ss61469051	*rs41566962*	5	11.436643	0.7564	1.0272	0.311163	0.0229	0.0084279323
ss86274899		5	11.48331	0.4477	10.0976	0.001550	0.0637	0.0314970597
	rs29024363	5	11.588989	0.5079	0.0421	0.837551	0.0038	0.0019123317
ss61521813	*rs41621912*	5	11.742598	0.5294	0.0239	0.877071	0.0029	0.0014394017
ss61521831	*rs41621929*	5	11.809991	0.3761	2.5492	0.110807	0.0314	0.0147388561
ss86327904		5	11.834975	0.4355	0.1867	0.665814	0.0080	0.0039381958
ss63906162	*rs42072957*	5	11.861264	0.5537	0.1048	0.746287	0.0062	0.0030628223
ss63905890	*rs42071421*	5	11.889745	0.7650	0.6212	0.430872	0.0169	0.0060932507
ss86322835		5	11.98247	0.1468	3.9229	0.048026	0.0521	0.0130533376
ss65345354	*rs43427698*	5	12.020696	0.5544	5.4807	0.019509	0.0438	0.0216302261
ss61556699	*rs41656301*	5	12.059219	0.4262	2.8216	0.093452	0.0324	0.0158244688
ss86320709		5	12.116514	0.0989	0.3672	0.544723	0.0185	0.0033020292
ss86299336		5	12.188814	0.5387	5.7752	0.016515	0.0440	0.0218819237
ss61510559	*rs41605183*	5	12.280079	0.8453	0.7409	0.389676	0.0218	0.0056907439
ss86316849		5	12.325355	0.6232	0.8049	0.369952	0.0167	0.0078571511
ss61491157	*rs41595053*	5	12.36512	0.0788	0.4090	0.522688	0.0210	0.0030445616
	rs29009927	5	12.423628	0.5981	2.6235	0.105747	0.0318	0.0152812336
ss117963797		5	12.451777	0.3302	0.7496	0.386913	0.0174	0.0076892549
ss64757023	*rs42849885*	5	12.500451	0.8202	0.3037	0.581775	0.0133	0.0039336479
	rs29018681	5	12.67005	0.5272	0.0154	0.901155	0.0023	0.0011688652
ss61507641	*rs41607929*	5	12.746398	0.7450	0.2746	0.600423	0.0112	0.0042542869
ss86336753		5	12.814009	0.0895	1.7947	0.180788	0.0423	0.0069023072
ss86338060		5	12.887932	0.7579	0.7807	0.377239	0.0190	0.0069850289
ss86338682		5	12.916993	0.4219	0.0186	0.891641	0.0026	0.0012675420
ss61477454	*rs41576789*	5	12.986154	0.9506	0.0011	0.973254	0.0014	0.0001351471
ss117967706		5	13.011	0.2077	1.0061	0.316194	0.0228	0.0075162138
ss64038312	*rs42450205*	5	13.099762	0.7665	0.0543	0.815857	0.0051	0.0018297657
	rs29017245	5	13.197167	0.7285	0.0217	0.882990	0.0031	0.0012279729
ss65346274	*rs43426517*	5	13.220938	0.7966	2.3781	0.123502	0.0369	0.0119530245
ss61491165	*rs41595061*	5	13.241405	0.4212	0.2331	0.629422	0.0093	0.0045176058
ss65346464	*rs43428905*	5	13.285398	0.4355	0.8758	0.349688	0.0170	0.0083820831
ss61491180	*rs41595076*	5	13.328732	0.8818	4.3983	0.036336	0.0612	0.0127583044
ss65346757	*rs43431798*	5	13.357025	0.1125	5.5751	0.018493	0.0700	0.0139644248
ss64314024	*rs42580540*	5	13.440319	0.7944	1.0377	0.308711	0.0237	0.0077407908
ss61567379	*rs41662594*	5	13.480778	0.1060	0.0165	0.897907	0.0037	0.0007021149
ss65346843	*rs43432784*	5	13.505734	0.2966	1.9441	0.163674	0.0279	0.0116463301

	rs29019788	5	13.694971	0.6074	1.4453	0.229692	0.0238	0.0113692680
ss86336066		5	13.749477	0.4900	1.1822	0.277283	0.0203	0.0101273173
ss86313353		5	13.846763	0.5093	1.2025	0.273206	0.0204	0.0102034285
ss86292359		5	14.162079	0.7034	0.0000	0.996589	0.0001	0.0000351904
ss86303832		5	14.212691	0.8424	6.4129	0.011548	0.0638	0.0169526510
ss86299638		5	14.252639	0.3474	0.7632	0.382616	0.0164	0.0074339271
ss86288874		5	14.376171	0.9033	0.9034	0.342196	0.0302	0.0052817168
ss61491182	*rs41595078*	5	14.416893	0.5315	1.1577	0.282317	0.0204	0.0101499646
ss86303476		5	14.445709	0.1547	0.1042	0.746909	0.0084	0.0021983721
ss86274666		5	14.500784	0.5372	1.0659	0.302228	0.0197	0.0097980015
ss86316054		5	14.793743	0.6547	0.0047	0.945186	0.0013	0.0006080228
ss117967848		5	14.827653	0.4477	0.7953	0.372821	0.0165	0.0081607849
ss86290471		5	14.896723	0.7128	0.0044	0.947329	0.0014	0.0005530902
ss61556862	*rs41649864*	5	14.916199	0.3567	0.8424	0.359015	0.0173	0.0079190581
ss117967657		5	15.002499	0.2106	0.0064	0.936271	0.0018	0.0005939543
ss86312970		5	15.052165	0.2149	9.7159	0.001902	0.0719	0.0242753300
ss86340723		5	15.146624	0.0659	3.1067	0.078411	0.0661	0.0081338643
ss61524756	*rs41623285*	5	15.189869	0.4026	0.2852	0.593477	0.0101	0.0048678431
ss86338602		5	15.22181	0.1991	0.6245	0.429656	0.0178	0.0056820644
ss86298750		5	15.309838	0.2128	2.2067	0.137867	0.0330	0.0110666322
ss86311662		5	15.353413	0.6526	0.9645	0.326392	0.0189	0.0085886230
ss61475665	*rs41576637*	5	15.392995	0.6447	0.7531	0.385800	0.0166	0.0076261477
ss86324429		5	15.440647	0.6275	0.0974	0.755025	0.0060	0.0028022796
ss61555381	*rs41654003*	5	15.738452	0.7185	2.1883	0.139518	0.0306	0.0123757226
ss117967901		5	16.101188	0.5537	3.6247	0.057339	0.0345	0.0170711802
ss117967810		5	16.310948	0.4577	7.2138	0.007407	0.0493	0.0244630424
ss86314499		5	16.671105	0.3804	4.1477	0.042069	0.0395	0.0186151789
ss86296958		5	16.745341	0.9312	5.2806	0.021860	0.0835	0.0106887152
	rs29018522	5	16.862261	0.6719	0.0571	0.811263	0.0047	0.0020646895
ss61563163	*rs41659255*	5	16.928871	0.5516	2.7274	0.099094	0.0298	0.0147224339
ss61469518	*rs41570420*	5	16.957758	0.8539	6.2808	0.012432	0.0663	0.0165363074
ss63555411	*rs42272533*	5	16.989714	0.5100	0.9843	0.321479	0.0184	0.0091968731
ss63555465	*rs42272551*	5	17.02581	0.8481	7.5700	0.006089	0.0712	0.0183402894
	rs29023979	5	17.082135	0.9391	0.1731	0.677498	0.0166	0.0018937335
ss61494065	*rs41593275*	5	17.141183	0.7579	0.0015	0.968726	0.0008	0.0003100215
ss61494068	*rs41593278*	5	17.163849	0.5652	0.5439	0.461080	0.0135	0.0066569301
ss86332404		5	17.212508	0.5630	0.9155	0.339002	0.0176	0.0086378743
	rs29010308	5	17.272102	0.6282	1.2294	0.267916	0.0212	0.0098986880
ss64416802	*rs42634036*	5	17.336522	0.1132	0.8481	0.357396	0.0267	0.0053690061
ss62865762	*rs42024216*	5	17.364022	0.0458	0.4901	0.484105	0.0305	0.0026655707
ss86338732		5	17.390411	0.1232	0.0409	0.839839	0.0056	0.0012202002
ss117967832		5	17.411891	0.7357	10.4418	0.001290	0.0688	0.0267704217
ss62866179	*rs42025555*	5	17.445798	0.3159	6.5224	0.010864	0.0516	0.0223059633
ss62995638	*rs42066176*	5	17.509648	0.3818	0.2811	0.596170	0.0099	0.0046632944
ss61548474	*rs41649678*	5	17.55662	0.5122	0.3106	0.577498	0.0103	0.0051584818
ss61555398	*rs41654020*	5	17.69979	0.0681	10.2655	0.001417	0.1177	0.0149254581
ss86307143		5	17.862609	0.7693	8.3725	0.003928	0.0630	0.0223740922

ss61490476	*rs41587982*	5	17.886085	0.5795	12.9476	0.000343		0.0688	0.0335206856
ss61495956	*rs41594514*	5	17.970665	0.6655	12.0319	0.000555		0.0679	0.0302362334
ss61490487	*rs41587993*	5	18.161333	0.2170	1.1397	0.286087		0.0244	0.0082765547
ss86341265		5	18.244334	0.7894	1.2177	0.270186		0.0259	0.0086024210
ss86298359		5	18.395406	0.8367	0.1622	0.687290		0.0101	0.0027639435
	rs29024540	5	18.453056	0.1117	0.1815	0.670189		0.0129	0.0025608617
ss86336597		5	18.499884	0.2901	0.1211	0.727994		0.0069	0.0028432315
ss61476868	*rs41579319*	5	18.533365	0.8066	0.0712	0.789683		0.0063	0.0019757528
ss61507375	*rs41605669*	5	18.563475	0.3195	0.0000	0.998649		0.0000	0.0000140513
ss86281895		5	18.753377	0.4248	5.7940	0.016340		0.0455	0.0222547819
ss117967821		5	18.775861	0.1017	0.4543	0.500523		0.0207	0.0037911186
ss86296709		5	18.83606	0.4928	5.8464	0.015864		0.0449	0.0224279874
ss61564393		5	18.899096	0.3123	3.8888	0.049004		0.0419	0.0179780667
ss61516677	*rs41617978*	5	18.94947	0.1110	0.0066	0.935197		0.0024	0.0004804234
ss61513808		5	19.030252	0.3188	0.2083	0.648236		0.0092	0.0039999978
ss86299197		5	19.098436	0.6877	0.0169	0.896557		0.0027	0.0011459804
ss86337070		5	19.230363	0.1755	0.0051	0.943313		0.0018	0.0005211979
ss86337913		5	19.445367	0.2156	2.6349	0.104992		0.0364	0.0123257385
ss86273892		5	19.603246	0.7787	10.0118	0.001623		0.0684	0.0235840327
ss86298388		5	19.749878	0.6067	1.8539	0.173776		0.0254	0.0120991016
ss61490861	*rs41591866*	5	20.300856	0.4756	3.8758	0.049382		0.0372	0.0185636319
ss61490864	*rs41591869*	5	20.327892	0.5072	3.4406	0.064034		0.0352	0.0175979351
ss61556195	*rs41651507*	5	20.352509	0.4964	3.6778	0.055549		0.0362	0.0181035793
ss61490866	*rs41591871*	5	20.380401	0.7170	1.4323	0.231797		0.0241	0.0097761923
ss61499510	*rs43710698*	5	20.631886	0.8933	1.5774	0.209551		0.0374	0.0071227247
ss61499511	*rs43710699*	5	20.635797	0.1554	0.8539	0.355786		0.0239	0.0062787151
ss61555406	*rs41654028*	5	20.889598	0.2006	0.0236	0.878027		0.0036	0.0011602081
ss61468955	*rs41566068*	5	20.911219	0.8367	0.8809	0.348270		0.0234	0.0063960388
ss86294811		5	21.019724	0.3388	11.1682	0.000877		0.0649	0.0290957852
ss117963804		5	21.082215	0.5057	5.5298	0.018973		0.0443	0.0221300461
	rs29010787	5	21.55708	0.5330	0.1754	0.675505		0.0077	0.0038132577
ss86338066		5	21.665723	0.1905	0.5216	0.470393		0.0168	0.0051897552
ss86336208		5	21.695352	0.1562	1.6149	0.204234		0.0316	0.0083209422
ss86288891		5	21.817803	0.3317	15.6727	0.000083		0.0749	0.0331941519
ss61510393	*rs41603721*	5	21.894275	0.2607	0.7896	0.374516		0.0188	0.0072463467
ss61503946	*rs41601737*	5	22.029123	0.8997	2.1901	0.139356		0.0457	0.0082519575
ss61510391	*rs41603719*	5	22.049266	0.8940	1.6947	0.193416		0.0395	0.0074870433
ss86338863		5	22.084737	0.7077	0.6538	0.419046		0.0160	0.0066231881
ss86322176		5	22.121817	0.5709	4.5568	0.033136		0.0403	0.0197591056
ss86339279		5	22.184576	0.1877	1.3219	0.250649		0.0271	0.0082698307
ss61478610	*rs41576996*	5	22.523125	0.2235	1.7781	0.182823		0.0285	0.0098787358
ss61530936	*rs41630099*	5	22.575325	0.7206	1.3096	0.252866		0.0242	0.0097582224
ss61527135	*rs41629693*	5	22.708445	0.4370	1.4291	0.232313		0.0216	0.0106215108
ss86338014		5	22.760647	0.2135	2.2261	0.136150		0.0323	0.0108464923
ss86339864		5	22.8502	0.8338	0.9625	0.326896		0.0242	0.0067138655
ss86338250		5	22.951902	0.1246	1.8041	0.179658		0.0363	0.0079282402
	rs29021228	5	23.090937	0.7034	0.3481	0.555405		0.0116	0.0048196500

ss117975210		5	23.245067	0.7085	3.2773	0.070676	0.0353	0.0145748437
ss86295578		5	23.546206	0.6067	0.0231	0.879160	0.0028	0.0013553294
ss61511150	*rs41610275*	5	23.619361	0.4699	6.6288	0.010240	0.0458	0.0228228143
ss86318839		5	23.669889	0.1812	3.8208	0.051020	0.0466	0.0138292545
ss61555403	*rs41654025*	5	23.757933	0.5888	5.3931	0.020504	0.0427	0.0206992397
ss86285225		5	24.033439	0.1196	3.6539	0.056349	0.0557	0.0117253056
	rs29024423	5	24.102497	0.5021	0.0026	0.958986	0.0010	0.0004831868
ss61465495	*rs41566742*	5	24.201836	0.1089	7.8864	0.005120	0.0826	0.0160219946
ss86338900		5	24.345662	0.0437	7.6618	0.005790	0.1243	0.0103867489
ss86326848		5	24.407627	0.0509	7.0780	0.007983	0.1088	0.0105078779
ss61566276	*rs41662318*	5	24.468018	0.1540	1.0150	0.314049	0.0262	0.0068312201
	rs29014779	5	24.576366	0.3023	7.2783	0.007149	0.0546	0.0230141015
ss86303752		5	24.69411	0.1669	6.5654	0.010608	0.0638	0.0177425523
ss61490517	*rs41588023*	5	24.718974	0.3668	0.1899	0.663098	0.0086	0.0039895509
ss86308217		5	24.75232	0.6719	17.4255	0.000034	0.0793	0.0349783675
ss61555451	*rs41654770*	5	24.775452	0.7586	12.4346	0.000449	0.0727	0.0266252839
ss61555455	*rs41654774*	5	24.796503	0.2213	1.1174	0.290837	0.0235	0.0080852523
ss61555456	*rs41654775*	5	24.900385	0.4112	3.3811	0.066376	0.0346	0.0167514844
ss38323769	*rs29012672*	5	24.929508	0.3109	0.0828	0.773646	0.0057	0.0024595474
ss86299459		5	25.063813	0.4563	0.8605	0.353922	0.0175	0.0086690397
ss86337928		5	25.22541	0.5781	0.0104	0.918963	0.0019	0.0009180184
ss86325433		5	25.311563	0.3496	0.2034	0.652142	0.0086	0.0039016054
ss86323984		5	25.369822	0.6769	0.4097	0.522330	0.0127	0.0055521861
ss86321691		5	25.409795	0.3266	0.3016	0.583072	0.0109	0.0047847955
ss61555468	*rs41654787*	5	25.576097	0.4656	1.6470	0.199790	0.0241	0.0119883564
ss61510401	*rs41603729*	5	25.620558	0.2901	0.2606	0.609838	0.0105	0.0043287355
ss86339980		5	25.667587	0.3159	0.2333	0.629275	0.0096	0.0041647013
ss86327393		5	25.69117	0.9119	2.6177	0.106129	0.0527	0.0084605505
ss86323825		5	25.739783	0.5559	0.4765	0.490256	0.0133	0.0065758384
ss117967726		5	25.783408	0.2077	0.2015	0.653666	0.0102	0.0033733279
ss117967907		5	25.807554	0.7923	0.2015	0.653666	0.0102	0.0033733279
ss61510400	*rs41603728*	5	26.014546	0.3245	1.2580	0.262416	0.0220	0.0096285604
ss61555464	*rs41654783*	5	26.044534	0.2643	3.4246	0.064654	0.0376	0.0146085155
ss61490531	*rs41589037*	5	26.096864	0.1848	1.1232	0.289595	0.0252	0.0075861068
ss61555462	*rs41654781*	5	26.118924	0.3811	0.5420	0.461872	0.0144	0.0067917585
ss61555461	*rs41654780*	5	26.152526	0.5960	0.0108	0.917199	0.0020	0.0009736119
ss61555460	*rs41654779*	5	26.204616	0.4828	1.3434	0.246827	0.0222	0.0110659390
ss86303405		5	26.456477	0.1110	2.4230	0.120024	0.0447	0.0088247555
ss86307735		5	26.493188	0.0501	0.0035	0.953054	0.0025	0.0002392499
ss117967790		5	26.558621	0.6554	5.1322	0.023793	0.0430	0.0194089275
ss117967777		5	26.752705	0.1963	4.1584	0.041806	0.0491	0.0154841127
ss86335850		5	26.80906	0.7450	0.1598	0.689438	0.0086	0.0032804515
ss117967756		5	26.855329	0.5351	5.1872	0.023056	0.0416	0.0206751483
ss86332806		5	27.11451	0.8804	0.2898	0.590506	0.0156	0.0032828333
ss61471612	*rs41571762*	5	27.138813	0.2400	0.1372	0.711240	0.0081	0.0029428123
ss86339824		5	27.16314	0.2120	0.0444	0.833143	0.0048	0.0015972666
ss86289057		5	27.223979	0.2407	1.3579	0.244297	0.0257	0.0093880870

ss86308294		5	27.244976	0.8854	1.8481	0.174445		0.0401	0.0081483053
ss86302756		5	27.317732	0.4771	6.7674	0.009481		0.0472	0.0235635326
ss86331553		5	27.340132	0.5201	6.3441	0.012000		0.0460	0.0229438389
ss61555509	*rs41654828*	5	27.433606	0.2507	3.6969	0.054922		0.0427	0.0160297181
ss61555506	*rs41654825*	5	27.491391	0.8603	0.0009	0.975510		0.0008	0.0001985370
ss61570157	*rs41664922*	5	27.5384	0.1368	0.0129	0.909751		0.0031	0.0007273514
	rs29027200	5	27.675106	0.7192	3.9454	0.047393		0.0397	0.0160179621
ss61555491	*rs41654810*	5	27.807102	0.3560	0.0398	0.841941		0.0039	0.0017797219
ss46526312	*rs41255451*	5	27.842227	0.3317	0.0000	0.999919		0.0000	0.0000008824
ss61555543	*rs41655862*	5	27.934465	0.7615	3.2449	0.072079		0.0384	0.0139401439
ss117967749		5	27.989564	0.4004	1.8254	0.177105		0.0253	0.0121353242
ss86290021		5	28.211689	0.7385	0.0870	0.768066		0.0063	0.0024210538
ss86326892		5	28.289182	0.3797	0.9081	0.340962		0.0187	0.0088135941
ss46526967	*rs41257413*	5	28.324317	0.6232	0.3686	0.543970		0.0120	0.0056498305
ss117962514		5	28.37099	0.7178	0.7500	0.386785		0.0191	0.0077515720
ss117962485		5	28.398161	0.4491	0.7811	0.377107		0.0173	0.0085535150
ss61555476	*rs41654795*	5	28.427626	0.7385	2.3886	0.122678		0.0324	0.0125262941
ss117962490		5	28.466749	0.2987	1.2918	0.256100		0.0230	0.0096492807
ss61555478	*rs41654797*	5	28.503922	0.2636	1.9004	0.168473		0.0287	0.0111569374
ss117962506		5	28.528518	0.0573	0.1472	0.701382		0.0154	0.0016665428
ss117962473		5	28.578361	0.7665	0.1391	0.709276		0.0083	0.0029845023
ss86311819		5	28.68701	0.6554	4.5760	0.032770		0.0427	0.0192654616
ss86301523		5	28.748319	0.1597	0.7237	0.395241		0.0215	0.0057654319
ss61490541	*rs41589047*	5	28.774243	0.7507	0.2695	0.603844		0.0111	0.0041549712
ss86312714		5	28.845313	0.2643	0.2172	0.641313		0.0102	0.0039863396
ss86315996		5	29.198579	0.7915	0.2188	0.640104		0.0108	0.0035602254
ss61490610	*rs41589115*	5	29.246453	0.2099	0.2263	0.634393		0.0111	0.0036975080
ss61555663	*rs41656661*	5	29.26647	0.7085	0.0326	0.856686		0.0038	0.0015807953
ss117962482		5	29.3233	0.9119	0.7862	0.375552		0.0297	0.0047739074
ss61555556	*rs41655875*	5	29.40821	0.8718	0.6593	0.417082		0.0230	0.0051503448
ss86290746		5	29.448074	0.8675	0.8644	0.352828		0.0256	0.0058911271
ss86316285		5	29.69632	0.5609	1.9971	0.158045		0.0261	0.0128579262
ss86289348		5	29.768434	0.6791	5.0068	0.025563		0.0437	0.0190601590
ss86290313		5	29.878026	0.5050	1.1142	0.291535		0.0198	0.0099141178
ss86335766		5	29.92145	0.9319	2.4329	0.119267		0.0583	0.0074009394
ss86317507		5	30.020214	0.7958	0.2634	0.607953		0.0121	0.0039233446
ss86319114		5	30.072106	0.1476	4.4892	0.034463		0.0556	0.0139809641
ss86302956		5	30.222828	0.7593	0.0000	0.994503		0.0001	0.0000544536
ss61490556	*rs41589061*	5	30.325976	0.3159	0.1359	0.712533		0.0075	0.0032500611
ss86340513		5	30.357916	0.7822	1.5224	0.217670		0.0283	0.0096390445
ss86323411		5	30.569474	0.6060	2.4362	0.119017		0.0296	0.0141339059
ss86329530		5	30.599173	0.3503	2.3738	0.123839		0.0299	0.0136249431
ss117967911		5	30.628058	0.1648	0.0584	0.809092		0.0060	0.0016438028
ss86302913		5	30.72055	0.3159	1.9789	0.159950		0.0269	0.0116252818
ss86295366		5	30.752692	0.4169	1.6695	0.196751		0.0236	0.0114502296
ss86307159		5	30.782703	0.1669	0.0330	0.855816		0.0044	0.0012293848
ss86323794		5	30.84745	0.5287	2.3171	0.128409		0.0278	0.0138523952

ss86330985		5	30.877797	0.3288	1.3611	0.243746	0.0221	0.0097423394
ss86340835		5	31.028075	0.1676	0.3011	0.583338	0.0136	0.0037916753
ss86306763		5	31.054333	0.4663	0.9534	0.329189	0.0180	0.0089521571
ss61555606	*rs41655905*	5	31.126581	0.4556	0.2736	0.601092	0.0096	0.0047401034
ss86301156		5	31.160371	0.1898	0.0321	0.857877	0.0043	0.0013304028
ss86321901		5	31.260663	0.6218	0.4506	0.502284	0.0130	0.0061049673
ss86340447		5	31.292206	0.2930	0.0006	0.980254	0.0005	0.0002149463
ss86341120		5	31.329776	0.1261	1.2411	0.265646	0.0311	0.0068606758
ss86289994		5	31.350345	0.3195	0.1364	0.711990	0.0076	0.0032847141
ss86336205		5	31.482741	0.1905	0.8049	0.369946	0.0216	0.0066601405
ss61490558	*rs41589063*	5	31.502927	0.4642	0.0216	0.883270	0.0029	0.0014364461
ss86320464		5	31.544775	0.2228	0.0268	0.869979	0.0037	0.0012900101
ss86341772		5	31.680196	0.7049	0.5168	0.472443	0.0143	0.0059417469
ss61555611	*rs41655910*	5	31.827087	0.1275	0.5497	0.458672	0.0214	0.0047658206
ss86338929		5	31.926025	0.1963	6.1969	0.013030	0.0586	0.0184836229
ss61555544	*rs41655863*	5	32.001789	0.1132	1.5818	0.208927	0.0376	0.0075415019
ss61568481	*rs41664575*	5	32.085144	0.1741	1.1895	0.275808	0.0269	0.0077204321
ss86301978		5	32.109076	0.3166	0.7166	0.397540	0.0164	0.0071020277
ss61568485	*rs41664579*	5	32.19941	0.1741	1.1895	0.275808	0.0269	0.0077204321
ss117963786		5	32.230795	0.1232	0.0148	0.903119	0.0035	0.0007459338
ss61490572	*rs41589077*	5	32.338738	0.4413	0.1990	0.655635	0.0083	0.0041049779
ss117967565		5	32.38664	0.3130	0.0010	0.974651	0.0007	0.0002796436
ss86341237		5	32.681478	0.9155	2.9773	0.084885	0.0564	0.0087310870
ss61555631	*rs41655930*	5	32.770185	0.2822	0.1450	0.703514	0.0079	0.0031816752
ss86290734		5	32.80135	0.6046	2.1467	0.143325	0.0271	0.0129750983
ss117963789		5	32.829968	0.5573	5.7491	0.016759	0.0439	0.0216796170
ss117967876		5	32.89968	0.6734	0.7395	0.390115	0.0170	0.0074999398
ss38328770	*rs29021075*	5	32.923078	0.2844	0.3591	0.549203	0.0123	0.0050116327
ss61555660	*rs41656658*	5	32.952817	0.4635	0.5772	0.447681	0.0142	0.0070700599
ss86340294		5	32.997753	0.1261	1.7647	0.184475	0.0375	0.0082688439
ss86335501		5	33.048112	0.3474	3.6128	0.057748	0.0368	0.0166937192
ss61490563	*rs41589068*	5	33.136024	0.2593	0.7032	0.401997	0.0181	0.0069683853
ss117962496		5	33.21437	0.6282	0.1814	0.670277	0.0082	0.0038083326
ss117962493		5	33.234563	0.3711	0.1564	0.692615	0.0076	0.0035301697
	rs29024515	5	33.277669	0.6017	1.0067	0.316047	0.0192	0.0092089604
ss86285888		5	33.33703	0.3238	0.2399	0.624399	0.0098	0.0042944203
ss86274790		5	33.515141	0.1762	3.1487	0.076424	0.0437	0.0126902441
ss86283356		5	33.535612	0.5910	0.2889	0.591088	0.0101	0.0048631197
ss86335510		5	33.560805	0.2199	1.5387	0.215233	0.0280	0.0096163278
	rs29015703	5	33.711793	0.3489	1.3337	0.248550	0.0217	0.0098638651
ss86292364		5	33.751972	0.5351	0.6551	0.418556	0.0148	0.0073850054
ss86320097		5	33.784282	0.3252	0.4701	0.493176	0.0135	0.0059251218
ss86319211		5	33.831446	0.6748	0.4701	0.493176	0.0135	0.0059251218
ss117962519		5	34.034784	0.8152	0.1267	0.722014	0.0087	0.0026304281
ss117963807		5	34.116567	0.1110	0.1342	0.714198	0.0107	0.0021216543
ss117974950		5	34.648815	0.2335	0.5801	0.446539	0.0168	0.0060063840
ss86337528		5	34.772404	0.1913	0.2555	0.613411	0.0118	0.0036444272

ss61555711	*rs41656709*	5	35.120268	0.5423	0.0522	0.819297	0.0043	0.0021300461
ss65349522	*rs43433932*	5	35.145075	0.2149	2.3396	0.126580	0.0353	0.0119237753
ss86286413		5	35.237698	0.8610	4.1884	0.041076	0.0569	0.0136206405
ss86331455		5	35.327855	0.7235	1.1928	0.275152	0.0226	0.0090563885
ss86291706		5	35.444336	0.5029	0.2751	0.600083	0.0097	0.0048388325
ss86296067		5	35.519769	0.3918	0.9651	0.326253	0.0185	0.0088297423
ss61478118	*rs41574634*	5	35.857485	0.7901	3.1818	0.074897	0.0394	0.0130556010
ss86274086		5	35.900142	0.2113	3.9812	0.046399	0.0442	0.0147171383
ss64750616	*rs42849305*	5	35.938527	0.2178	4.6181	0.031981	0.0479	0.0163027579
ss117963792		5	35.966308	0.7958	3.5933	0.058427	0.0437	0.0142137129
ss86340021		5	36.051713	0.6941	1.0128	0.314592	0.0215	0.0091147039
ss86337184		5	36.092499	0.6125	0.8850	0.347167	0.0178	0.0084695652
ss86322536		5	36.115981	0.2249	0.1235	0.725349	0.0077	0.0026758138
ss61506194	*rs41603412*	5	36.284516	0.6705	0.6660	0.414720	0.0162	0.0071371971
ss63851807	*rs42384792*	5	36.346655	0.8410	0.0828	0.773618	0.0074	0.0019794119
ss117967690		5	36.37195	0.7930	0.2899	0.590461	0.0127	0.0041590369
ss63851725	*rs42384751*	5	36.412688	0.5989	1.0404	0.308084	0.0191	0.0091839579
ss63851681	*rs42384029*	5	36.473999	0.6791	1.7530	0.185932	0.0258	0.0112607257
ss63851609	*rs42383993*	5	36.495909	0.3173	2.1004	0.147710	0.0283	0.0122640105
ss86283300		5	36.51918	0.4857	1.8222	0.177493	0.0242	0.0121005062
ss61517163		5	36.650281	0.4119	0.1057	0.745184	0.0060	0.0028980974
ss61465817	*rs41569961*	5	36.725906	0.1705	0.7408	0.389714	0.0206	0.0058321681
ss64969479	*rs43063209*	5	36.74864	0.7701	1.0483	0.306262	0.0227	0.0080252756
ss61494298	*rs41595701*	5	36.771734	0.1655	0.3463	0.556431	0.0144	0.0039705312
ss63809721	*rs42364886*	5	36.795402	0.8202	0.4833	0.487142	0.0168	0.0049423008
ss63809623	*rs42364837*	5	36.841345	0.6547	0.9798	0.322586	0.0192	0.0086637477
ss61472766	*rs41573985*	5	36.89438	0.1096	0.5776	0.447496	0.0230	0.0044917638
ss86320862		5	36.92679	0.8797	10.5161	0.001240	0.0899	0.0190288533
ss64388494	*rs42613707*	5	36.959168	0.7822	0.7378	0.390647	0.0189	0.0064493519
ss61569056	*rs41664746*	5	36.987738	0.7693	1.0764	0.299865	0.0230	0.0081656658
ss64388202	*rs42616860*	5	37.015064	0.7457	1.4243	0.233104	0.0248	0.0094204506
ss86273632		5	37.036477	0.4993	0.2518	0.615952	0.0090	0.0045120187
ss64388100	*rs42616309*	5	37.072641	0.1676	0.7919	0.373845	0.0219	0.0061068808
ss64388054	*rs42616286*	5	37.108971	0.7751	0.0014	0.970652	0.0008	0.0002828676
ss86335926		5	37.14912	0.7751	0.0000	0.994502	0.0002	0.0000529737
ss64758300	*rs42852055*	5	37.18638	0.3302	0.5976	0.439751	0.0154	0.0068137868
ss64106655	*rs42477538*	5	37.484343	0.2937	0.0229	0.879638	0.0030	0.0012370216
ss61478756	*rs41577937*	5	37.56067	0.8295	0.1700	0.680253	0.0102	0.0028954507
ss64811586	*rs42910947*	5	37.893606	0.7887	0.1994	0.655321	0.0100	0.0033485880
ss86338101		5	37.930355	0.2221	0.9196	0.337922	0.0213	0.0073601066
ss86336773		5	38.023653	0.7357	4.2929	0.038641	0.0428	0.0166543387
ss86338416		5	38.109781	0.3682	2.2686	0.132473	0.0294	0.0136931865
ss86275154		5	38.220213	0.2772	0.0821	0.774490	0.0058	0.0023208263
ss61527760	*rs41626004*	5	38.696588	0.4448	0.1137	0.736116	0.0064	0.0031595339
ss117967560		5	38.728958	0.2149	0.1666	0.683305	0.0089	0.0029939305
ss61548873	*rs41645387*	5	38.997928	0.7994	0.7301	0.393159	0.0190	0.0060937491
ss86293628		5	39.080769	0.5344	2.4182	0.120388	0.0297	0.0147819434

ss86296223		5	39.161931	0.6117	1.1950	0.274696		0.0204	0.0096990162
ss86336068		5	39.414107	0.5924	0.0230	0.879395		0.0029	0.0013791314
ss86292438		5	39.553826	0.5287	0.0000	0.998930		0.0000	0.0000126597
ss65030381	*rs43119961*	5	39.578475	0.2758	0.7385	0.390439		0.0178	0.0071026272
ss65349097	*rs43435407*	5	39.603517	0.7572	1.2481	0.264310		0.0240	0.0088335511
ss65348994	*rs43434704*	5	39.745981	0.7930	0.7392	0.390224		0.0191	0.0062820716
ss65348970	*rs43434680*	5	39.79798	0.3266	3.2060	0.073803		0.0350	0.0153972532
ss65348938	*rs43434648*	5	39.866126	0.7027	0.0449	0.832288		0.0041	0.0017321393
ss117962522		5	39.943548	0.9441	2.5051	0.113930		0.0642	0.0067784365
ss61490587	*rs41589092*	5	40.012999	0.4606	0.4985	0.480403		0.0135	0.0066884083
ss86320334		5	40.097746	0.7579	0.4051	0.524655		0.0135	0.0049574254
ss86298489		5	40.134803	0.1297	1.2541	0.263166		0.0314	0.0070924019
ss64653113	*rs42758214*	5	40.261056	0.1196	2.4094	0.121062		0.0440	0.0092688974
ss63895272	*rs41912825*	5	40.457394	0.4355	0.9482	0.330517		0.0182	0.0089466093
ss63895172	*rs41912776*	5	40.492993	0.2722	0.1896	0.663408		0.0092	0.0036264407
ss86322523		5	40.619088	0.1325	0.0062	0.937364		0.0022	0.0005078523
	rs29021717	5	40.715657	0.9054	1.1152	0.291318		0.0341	0.0058366975
	rs29013530	5	40.852254	0.3223	2.1869	0.139643		0.0288	0.0126008152
ss61490646	*rs41590051*	5	40.939193	0.8266	0.0172	0.895809		0.0031	0.0008875848
ss61507895	*rs41599885*	5	40.972821	0.5523	0.1630	0.686532		0.0073	0.0036202600
ss61531731	*rs41629283*	5	41.011603	0.3489	0.1499	0.698711		0.0073	0.0033102411
ss86307553		5	41.085428	0.8044	0.0026	0.959118		0.0012	0.0003697732
ss86283485		5	41.154328	0.0659	13.5459	0.000251		0.1337	0.0164549721
	rs29017167	5	41.296054	0.1741	3.0871	0.079353		0.0411	0.0118250573
ss86300320		5	41.377022	0.6511	1.6504	0.199333		0.0239	0.0108507816
ss61555721	*rs41656719*	5	41.397618	0.7385	0.9881	0.320541		0.0201	0.0077731149
ss61555723	*rs41656721*	5	41.436755	0.0766	1.2974	0.255078		0.0387	0.0054727885
ss61555725	*rs41656723*	5	41.512974	0.5279	1.8313	0.176419		0.0249	0.0124292599
ss61468993	*rs41566105*	5	41.566044	0.5265	1.6415	0.200551		0.0236	0.0117718640
ss61490648	*rs41590053*	5	41.752964	0.3324	4.3617	0.037118		0.0400	0.0177334108
ss61555732	*rs41656730*	5	41.800846	0.6755	4.8063	0.028687		0.0426	0.0186910706
ss86337277		5	41.873951	0.7973	1.9505	0.162983		0.0315	0.0101886866
ss86284611		5	41.913753	0.4549	1.3796	0.240566		0.0221	0.0109622072
	rs29016364	5	41.970029	0.2357	1.9218	0.166100		0.0297	0.0106936403
ss86299924		5	42.33463	0.2206	1.7583	0.185272		0.0292	0.0100410278
ss86334020		5	42.54682	0.2042	2.5786	0.108773		0.0364	0.0118180361
ss86283421		5	42.655632	0.5208	0.9661	0.325992		0.0179	0.0089314983
ss86285627		5	42.727064	0.7851	0.2007	0.654313		0.0101	0.0034234831
ss86283780		5	42.813872	0.7328	4.6985	0.030528		0.0437	0.0171123595
ss86279979		5	42.945172	0.7679	0.3324	0.564423		0.0128	0.0045676484
	rs29016423	5	43.286974	0.3037	1.9169	0.166645		0.0280	0.0118358282
ss86277105		5	43.340934	0.4384	1.6808	0.195247		0.0236	0.0115997434
	rs29026394	5	43.372802	0.4312	5.2860	0.021792		0.0420	0.0206158873
ss64447596	*rs42643123*	5	43.421223	0.0602	1.8701	0.171908		0.0532	0.0060120406
ss86314951		5	43.492244	0.3087	0.8727	0.350519		0.0191	0.0081423437
ss61518681	*rs41617233*	5	43.522731	0.4391	0.0935	0.759823		0.0056	0.0027485746
ss61518679	*rs41617231*	5	43.557911	0.5380	0.2728	0.601610		0.0094	0.0046598361

ss86338062		5	43.643123	0.7908	0.0005	0.981336	0.0005	0.0001806530
ss86295268		5	43.66955	0.3474	0.0249	0.874682	0.0030	0.0013552771
ss61490643	*rs41590048*	5	43.711543	0.6375	0.1643	0.685315	0.0076	0.0035242689
ss117963834		5	43.735761	0.3617	0.1450	0.703458	0.0072	0.0033247502
ss65349756	*rs43435863*	5	43.772107	0.3625	0.1643	0.685315	0.0076	0.0035242689
	rs29026986	5	43.820844	0.6390	0.1550	0.693963	0.0074	0.0034316580
ss64717859	*rs42816289*	5	43.8513	0.7070	0.2923	0.588928	0.0106	0.0043979368
ss64717843	*rs42816273*	5	43.876907	0.6605	0.1839	0.668157	0.0082	0.0036680043
ss64717819	*rs42816250*	5	43.905559	0.2779	0.5053	0.477430	0.0142	0.0057079836
ss86334546		5	43.974412	0.6619	1.8185	0.177927	0.0261	0.0116598320
ss63650047	*rs42306358*	5	44.016078	0.2837	0.5233	0.469662	0.0144	0.0058331131
ss63650152	*rs42306393*	5	44.049852	0.7199	0.2786	0.597792	0.0106	0.0042618985
ss61568783	*rs41666667*	5	44.075126	0.1726	1.0379	0.308660	0.0253	0.0072149325
ss63650263	*rs42306430*	5	44.101768	0.7529	4.3858	0.036600	0.0440	0.0163768507
ss64385059	*rs42611084*	5	44.146732	0.7529	4.3858	0.036600	0.0440	0.0163768507
ss64385019	*rs42611064*	5	44.176107	0.4807	0.0075	0.930908	0.0016	0.0007907161
ss61568780	*rs41666664*	5	44.35064	0.3016	4.2401	0.039851	0.0419	0.0176632601
ss117967555		5	44.471549	0.0723	7.9714	0.004888	0.1004	0.0134723856
ss61474730		5	44.684057	0.1970	0.2046	0.651201	0.0107	0.0033798178
	rs29010249	5	44.751133	0.8130	0.2763	0.599282	0.0122	0.0036957155
ss64083193	*rs42468401*	5	44.794498	0.1691	0.5238	0.469460	0.0176	0.0049532182
ss61478344	*rs41575744*	5	44.836132	0.7966	0.1935	0.660142	0.0100	0.0032443080
ss117967663		5	44.873523	0.8603	0.2028	0.652629	0.0122	0.0029274229
ss64829383	*rs42922175*	5	44.904072	0.7966	0.1935	0.660142	0.0100	0.0032443080
ss86336106		5	45.154889	0.6712	0.0944	0.758766	0.0061	0.0027045424
ss61514809	*rs41616365*	5	45.346212	0.6440	7.0089	0.008294	0.0501	0.0229609557
ss86318226		5	45.427315	0.2357	0.7534	0.385708	0.0192	0.0069041223
ss86309955		5	45.448667	0.3181	0.2713	0.602610	0.0103	0.0044892295
	rs29025831	5	45.473335	0.0981	0.1303	0.718265	0.0113	0.0020050946
ss61495184	*rs41594766*	5	45.500693	0.2529	0.0127	0.910140	0.0024	0.0009147382
ss86299648		5	45.547143	0.0981	0.1773	0.673830	0.0132	0.0023392697
ss86328534		5	45.67967	0.4936	14.3727	0.000163	0.0690	0.0344879211
ss86327428		5	45.775857	0.5580	13.3748	0.000274	0.0667	0.0329128276
ss61522028	*rs41623021*	5	45.854016	0.2307	0.7368	0.390997	0.0191	0.0067634775
ss61506995	*rs41601399*	5	45.904554	0.6934	4.6435	0.031514	0.0424	0.0180387769
ss63910216	*rs42129284*	5	45.926535	0.4148	1.5619	0.211812	0.0229	0.0110943289
ss63910270	*rs42129311*	5	45.9667	0.4269	0.5356	0.464506	0.0136	0.0066526945
ss63910294	*rs42129323*	5	45.992976	0.8918	1.4031	0.236603	0.0349	0.0067361501
ss86326117		5	46.025315	0.3524	0.6571	0.417850	0.0151	0.0068980734
ss86304241		5	46.086409	0.6526	3.0171	0.082835	0.0331	0.0150310170
	rs29012326	5	46.117941	0.3489	3.2739	0.070822	0.0344	0.0156093615
ss117967568		5	46.145022	0.0989	8.8989	0.002953	0.0885	0.0157744161
	rs29014396	5	46.227236	0.7923	1.3072	0.253290	0.0256	0.0084376318
ss117962479		5	46.294772	0.5279	7.2461	0.007276	0.0501	0.0249587257
ss65350180	*rs43440583*	5	46.325628	0.3890	5.3387	0.021149	0.0435	0.0206537183
ss65350162	*rs43440565*	5	46.348871	0.1855	2.0829	0.149403	0.0336	0.0101406163
ss86295713		5	46.432699	0.3037	6.7530	0.009557	0.0512	0.0216429345

ss86291852		5	46.476277	0.6139	0.1964	0.657773	0.0083	0.0039270390
ss65350425	*rs43434133*	5	46.499792	0.5487	1.9576	0.162215	0.0260	0.0128933320
ss64983933	*rs43072965*	5	46.539973	0.8159	1.0093	0.315411	0.0245	0.0073537797
ss64351807	*rs42600489*	5	46.591356	0.5652	1.3594	0.244036	0.0214	0.0105013183
ss64351853	*rs42600512*	5	46.64281	0.5681	1.2203	0.269691	0.0203	0.0099779181
ss64351923	*rs42601646*	5	46.70715	0.6168	3.6375	0.056904	0.0356	0.0168060853
ss61496610	*rs43745946*	5	46.743524	0.4828	1.5836	0.208660	0.0232	0.0115649075
ss61568120	*rs41661425*	5	46.770848	0.4047	1.2653	0.261035	0.0211	0.0101806395
ss86294546		5	46.795546	0.2658	2.7980	0.094833	0.0355	0.0138696165
ss64693396	*rs42791351*	5	46.856408	0.1655	0.3245	0.569113	0.0145	0.0040139973
ss65350768	*rs43436975*	5	46.963919	0.9606	0.0461	0.830076	0.0103	0.0007822589
ss65350759	*rs43436966*	5	46.996517	0.4140	2.8944	0.089334	0.0319	0.0154608358
ss61555746	*rs41657444*	5	47.059557	0.9262	0.2655	0.606523	0.0185	0.0025282772
ss61490661	*rs41590066*	5	47.111583	0.5215	0.0662	0.797041	0.0047	0.0023217804
ss86337778		5	47.198426	0.2056	0.0443	0.833450	0.0051	0.0016539921
ss65350326	*rs43432834*	5	47.235568	0.9033	0.0038	0.950633	0.0019	0.0003389632
ss117967676		5	47.318407	0.9241	0.1148	0.734807	0.0117	0.0016373513
ss117962467		5	47.366085	0.4112	2.8241	0.093307	0.0316	0.0153138852
ss117962470		5	47.388935	0.6433	9.2558	0.002436	0.0583	0.0267628942
ss117962516		5	47.445471	0.2851	7.2464	0.007275	0.0542	0.0220910621
ss65350109	*rs43439412*	5	47.526224	0.8775	7.0164	0.008260	0.0733	0.0157472223
ss61530831	*rs41628796*	5	47.652925	0.1053	0.1026	0.748786	0.0095	0.0017845424
ss86277669		5	47.885309	0.3610	8.2128	0.004285	0.0529	0.0244043763
ss64769789	*rs42865526*	5	47.911774	0.4706	0.5280	0.467675	0.0130	0.0064952511
ss65350357	*rs43432865*	5	48.040438	0.4592	2.0120	0.156507	0.0261	0.0129446113
ss86274989		5	48.195504	0.8181	3.7728	0.052495	0.0470	0.0139958244
ss86274207		5	48.229978	0.4735	0.9401	0.332587	0.0174	0.0086676092
ss86321985		5	48.434551	0.5494	1.9467	0.163384	0.0263	0.0130258900
ss61515439	*rs41614295*	5	48.474879	0.1225	2.3967	0.122048	0.0436	0.0093820003
ss61465645	*rs41567990*	5	48.495106	0.5852	2.1335	0.144563	0.0265	0.0128842174
ss86317399		5	48.529055	0.8804	2.7617	0.096999	0.0472	0.0099347635
ss61490662	*rs41590067*	5	48.554814	0.6691	6.6071	0.010364	0.0494	0.0218689336
ss61495990	*rs41595547*	5	48.759231	0.1032	7.1663	0.007603	0.0829	0.0153430899
ss117967654		5	48.875778	0.4656	1.8199	0.177766	0.0258	0.0128148757
ss86287186		5	49.078491	0.6347	0.0156	0.900549	0.0024	0.0010947902
ss86278051		5	49.133516	0.5372	0.7736	0.379423	0.0164	0.0081550874
ss86335452		5	49.186342	0.4613	1.0354	0.309258	0.0191	0.0094685183
ss117962500		5	49.295543	0.8976	0.5934	0.441385	0.0232	0.0042576449
ss117962512		5	49.355996	0.8216	2.8482	0.091925	0.0399	0.0117023476
ss61490664	*rs41590069*	5	49.446398	0.5602	0.6070	0.436189	0.0139	0.0068477544
ss61555754	*rs41657452*	5	49.661894	0.1934	5.3504	0.021008	0.0515	0.0160667798
ss86315413		5	49.850261	0.2307	2.3849	0.122968	0.0325	0.0115361373
ss86327441		5	50.022924	0.6547	0.0013	0.970955	0.0007	0.0003218971
ss86338886		5	50.069068	0.3245	0.0029	0.957148	0.0011	0.0004623928
ss86307440		5	50.098067	0.3968	0.0429	0.835881	0.0038	0.0018056895
ss61470023	*rs41566020*	5	50.323094	0.1368	1.1550	0.282876	0.0295	0.0069609115
ss86280507		5	50.40137	0.8797	0.5141	0.473601	0.0200	0.0042295697

ss86341260		5	50.537959	0.1626	0.6184	0.431900		0.0199	0.0054137384
ss61569153	*rs41665539*	5	50.562257	0.4692	1.4168	0.234329		0.0216	0.0107610609
ss61497060	*rs41593990*	5	50.589135	0.7865	2.7363	0.098540		0.0366	0.0122937715
ss86340660		5	50.654895	0.5860	4.7792	0.029138		0.0412	0.0199911375
	rs29022391	5	50.69822	0.8360	0.2602	0.610139		0.0122	0.0033432754
ss86305510		5	50.816331	0.6963	0.9907	0.319916		0.0198	0.0083622686
ss86324491		5	51.050725	0.1375	0.0086	0.926234		0.0025	0.0005863197
ss86289207		5	51.145166	0.4277	3.7801	0.052270		0.0370	0.0181292917
ss86312098		5	51.595012	0.5122	0.2517	0.616012		0.0092	0.0046083728
	rs29016809	5	51.770121	0.0723	6.4599	0.011249		0.0896	0.0120263089
ss61490671	*rs41590076*	5	51.821819	0.8560	3.5113	0.061369		0.0483	0.0119150335
ss86335800		5	52.00652	0.6039	1.3734	0.241626		0.0215	0.0102944056
ss86316266		5	52.160767	0.1791	1.3548	0.244842		0.0280	0.0082465632
ss86325004		5	52.336245	0.4785	0.2797	0.597052		0.0096	0.0047853303
ss61555763	*rs41657461*	5	52.454561	0.8395	0.0129	0.909629		0.0029	0.0007731087
ss61555761	*rs41657459*	5	52.537293	0.4527	0.5608	0.454185		0.0134	0.0066417863
ss86281045		5	52.667682	0.1225	6.3853	0.011728		0.0704	0.0151367449
ss61466975	*rs41565111*	5	52.747836	0.8352	0.0803	0.776985		0.0072	0.0019815994
ss61478669	*rs41577251*	5	52.807103	0.4556	0.1388	0.709583		0.0068	0.0033489204
ss86334747		5	52.858156	0.0838	10.2593	0.001422		0.1066	0.0163719823
ss117967615		5	53.19013	0.5136	0.0687	0.793372		0.0046	0.0023159295
ss61555766	*rs41657464*	5	53.209941	0.3961	1.3734	0.241626		0.0215	0.0102944056
	rs29021741	5	53.391515	0.4807	0.0149	0.902935		0.0022	0.0010755461
ss86300644		5	53.415544	0.0559	4.9357	0.026628		0.0874	0.0092222733
ss86295748		5	53.490126	0.9097	3.4851	0.062344		0.0591	0.0097091757
ss61500582	*rs41599332*	5	53.527535	0.7944	3.9668	0.046797		0.0475	0.0155145537
ss86289276		5	53.566385	0.5215	2.2523	0.133874		0.0269	0.0134312071
ss86315304		5	53.681011	0.7443	4.1778	0.041333		0.0422	0.0160675765
ss86340726		5	53.815448	0.5895	0.0091	0.923887		0.0017	0.0008450857
ss86338221		5	53.920783	0.5723	1.0978	0.295104		0.0192	0.0094050816
ss86320488		5	54.077583	0.0573	5.8939	0.015446		0.0936	0.0101120760
ss61466977	*rs41565113*	5	54.13139	0.4155	0.0001	0.992038		0.0002	0.0000884578
ss61466979	*rs41565115*	5	54.157733	0.1146	0.0006	0.981075		0.0007	0.0001435674
ss86331829		5	54.366286	0.4943	0.0207	0.885692		0.0025	0.0012723041
ss61505171	*rs41602930*	5	54.390702	0.4993	1.0141	0.314275		0.0183	0.0091462876
ss61525795	*rs41622498*	5	54.6047	0.0638	0.0247	0.875187		0.0057	0.0006788443
ss61525791	*rs41622494*	5	54.650797	0.8066	0.3691	0.543711		0.0142	0.0044264867
ss86322526		5	54.764504	0.7342	0.0173	0.895410		0.0028	0.0010803784
ss117967741		5	54.79093	0.6669	0.5590	0.454914		0.0143	0.0063408536
ss86340055		5	54.95258	0.5122	1.4555	0.228057		0.0217	0.0108618619
ss86297916		5	54.987541	0.4900	1.5371	0.215469		0.0222	0.0111103795
ss86310574		5	55.037007	0.0666	0.5212	0.470560		0.0254	0.0031600990
ss86326368		5	55.064958	0.5244	0.3116	0.576890		0.0101	0.0050566615
ss86341602		5	55.106542	0.3016	0.4725	0.492088		0.0135	0.0057057684
ss61469000	*rs41566112*	5	55.137439	0.4148	0.2545	0.614070		0.0095	0.0046279036
ss61490688	*rs41590093*	5	55.176317	0.5888	0.4016	0.526488		0.0120	0.0057874258
ss86340106		5	55.200885	0.4398	0.6744	0.411787		0.0154	0.0076087231

ss117967851		5	55.226468	0.1082	0.0336	0.854609	0.0054	0.0010336644
ss117967867		5	55.428817	0.3782	0.2271	0.633860	0.0092	0.0043121840
ss61530546	*rs41625417*	5	55.549405	0.3711	0.1196	0.729618	0.0066	0.0030649299
ss61530548	*rs41625419*	5	55.584503	0.8926	0.0000	0.997035	0.0001	0.0000208602
ss86336563		5	55.642116	0.0752	1.3944	0.238061	0.0416	0.0057819047
ss86340310		5	55.712818	0.5795	0.0339	0.853887	0.0034	0.0016339660
ss86287495		5	55.743332	0.4176	0.4852	0.486300	0.0127	0.0061945181
ss117967882		5	55.881324	0.6791	0.6122	0.434223	0.0153	0.0066884487
ss86340869		5	55.947825	0.3109	0.3089	0.578535	0.0109	0.0046776580
ss86283025		5	56.194785	0.4857	3.5843	0.058744	0.0348	0.0173740924
ss61490685	*rs41590090*	5	56.26737	0.7049	1.3886	0.239049	0.0231	0.0096286846
ss61555771	*rs41657469*	5	56.458335	0.8918	2.5408	0.111389	0.0472	0.0091003290
ss61555768	*rs41657466*	5	56.568072	0.5609	0.0469	0.828700	0.0040	0.0019920024
ss86308855		5	56.748063	0.4205	0.8487	0.357243	0.0176	0.0085836461
ss61497443	*rs41596870*	5	57.566227	0.8102	1.9496	0.163074	0.0316	0.0097211953
ss86341613		5	57.59969	0.4592	0.7474	0.387613	0.0166	0.0082661188
ss61555812	*rs41657510*	5	57.660253	0.8875	2.3549	0.125342	0.0439	0.0087639546
ss61511120	*rs41610245*	5	57.697065	0.6132	1.5712	0.210452	0.0247	0.0117285221
ss117967687		5	57.72705	0.4570	0.6564	0.418125	0.0155	0.0077065623
ss61547670	*rs41647288*	5	58.000199	0.9140	2.6784	0.102171	0.0547	0.0086023274
ss61562877	*rs41665964*	5	58.236172	0.6411	0.2981	0.585253	0.0108	0.0049658035
	rs29020121	5	58.294794	0.8904	0.0112	0.915649	0.0032	0.0006299114
ss86296582		5	58.402883	0.7722	0.1883	0.664480	0.0097	0.0033990497
ss86339488		5	58.541252	0.5946	2.1198	0.145860	0.0282	0.0135921139
ss61499306	*rs43709701*	5	58.579367	0.2128	0.1329	0.715532	0.0084	0.0028184887
ss86297591		5	58.638814	0.5745	0.3778	0.538976	0.0119	0.0058352897
ss86319998		5	58.672811	0.5530	1.3154	0.251818	0.0217	0.0107116935
	rs29018609	5	59.034171	0.5179	2.5505	0.110714	0.0300	0.0149657770
ss86341763		5	59.065276	0.1039	0.0045	0.946333	0.0021	0.0003860366
ss61563793	*rs41664061*	5	59.29478	0.5788	0.0272	0.869042	0.0032	0.0015381068
ss61545767	*rs41637723*	5	59.41935	0.5809	0.1552	0.693770	0.0077	0.0037371444
ss61545754	*rs41637710*	5	59.482668	0.1841	2.4345	0.119148	0.0369	0.0110743492
ss86321921		5	59.511545	0.4943	0.5906	0.442453	0.0148	0.0073985665
ss61509385	*rs41604139*	5	59.614768	0.1777	2.0982	0.147925	0.0352	0.0102912925
ss86341169		5	59.694619	0.2486	0.3993	0.527683	0.0131	0.0049090374
ss86312226		5	59.720694	0.4499	3.2978	0.069802	0.0352	0.0174112439
	rs29026607	5	59.756375	0.2880	0.7418	0.389382	0.0177	0.0072402278
ss86291264		5	59.972528	0.6189	0.6517	0.419770	0.0157	0.0074230004
ss117967571		5	60.053202	0.0960	0.6189	0.431740	0.0251	0.0043495933
ss86339191		5	60.097023	0.6103	1.5774	0.209557	0.0246	0.0117209773
ss86307567		5	60.31844	0.4255	2.8613	0.091183	0.0330	0.0161316265
ss61555814		5	60.366995	0.3890	2.1344	0.144479	0.0284	0.0135058818
ss61510435	*rs41604562*	5	60.570822	0.7550	1.8624	0.172787	0.0297	0.0109689934
ss86299194		5	60.694919	0.9277	0.3811	0.537206	0.0221	0.0029629549
ss86312879		5	60.763987	0.5014	0.6471	0.421422	0.0152	0.0076064943
ss86334364		5	60.79776	0.1282	0.0786	0.779226	0.0078	0.0017333279
ss117967752		5	60.840049	0.8145	1.2118	0.271357	0.0262	0.0079134927

ss86297204		5	61.244601	0.2027	0.7744	0.379165	0.0204	0.0065986172
ss61555781	*rs41657479*	5	61.32314	0.0587	1.3506	0.245573	0.0450	0.0049800712
ss86289784		5	61.546443	0.1984	0.0459	0.830471	0.0050	0.0015980285
ss117963814		5	61.624223	0.7650	0.1805	0.671056	0.0094	0.0033944453
ss117967842		5	61.64472	0.1404	6.3579	0.011908	0.0664	0.0160298460
ss117967879		5	61.798502	0.2536	0.4261	0.514104	0.0141	0.0053417003
ss86309257		5	61.84763	0.7042	0.9833	0.321724	0.0205	0.0085228103
	rs29018280	5	61.921458	0.3797	1.4733	0.225242	0.0232	0.0109408348
ss86312818		5	62.192672	0.6719	0.0022	0.962441	0.0009	0.0004117605
	rs29017368	5	62.242551	0.3317	0.0004	0.983711	0.0004	0.0001793692
ss65041425	*rs43129156*	5	62.264656	0.1640	3.1699	0.075445	0.0430	0.0117965684
ss64937370	*rs43027185*	5	62.377657	0.8403	2.5984	0.107425	0.0394	0.0105660585
ss117975023		5	62.500108	0.8116	3.3459	0.067802	0.0421	0.0128864605
ss86286550		5	62.801616	0.1913	0.7439	0.388716	0.0202	0.0062529446
ss86323925		5	62.856947	0.5272	5.0031	0.025617	0.0424	0.0211251777
ss64525610	*rs42683471*	5	63.506239	0.8195	0.7852	0.375848	0.0222	0.0065651651
ss86278729		5	64.141257	0.9069	0.4222	0.516065	0.0206	0.0034775867
ss86284707		5	64.385282	0.8181	1.5837	0.208655	0.0300	0.0089405075
ss117967712		5	64.455407	0.7579	2.6253	0.105625	0.0351	0.0128798884
ss61515076	*rs41611937*	5	64.486269	0.9219	0.2588	0.611130	0.0179	0.0025698941
	rs29024740	5	64.565965	0.4334	3.1205	0.077754	0.0336	0.0164930530
	rs17871338	5	64.666893	0.4592	1.8166	0.178162	0.0247	0.0122695847
ss86285540		5	64.70574	0.1855	2.6580	0.103486	0.0384	0.0115962894
ss86287833		5	64.748878	0.7722	4.6282	0.031794	0.0470	0.0165282193
ss86326942		5	64.775116	0.1433	0.0055	0.940655	0.0020	0.0004849858
ss86300819		5	64.795413	0.2421	1.7743	0.183285	0.0293	0.0107435681
ss86340206		5	64.833595	0.2722	0.5083	0.476119	0.0150	0.0059596557
	rs29023088	5	64.856925	0.2693	0.6476	0.421232	0.0171	0.0067288162
ss86338655		5	64.903236	0.0867	2.3366	0.126822	0.0491	0.0077708177
ss86334713		5	65.126504	0.9033	4.4104	0.036081	0.0633	0.0110585406
ss61490726	*rs41590731*	5	65.203225	0.6683	5.1606	0.023409	0.0453	0.0200817244
	rs29018854	5	65.375215	0.1676	0.3648	0.546029	0.0152	0.0042341438
ss61555886	*rs41647799*	5	65.413314	0.0774	0.3579	0.549865	0.0211	0.0030078099
ss86306575		5	65.485285	0.9319	0.5479	0.459441	0.0274	0.0034771888
ss61490742	*rs41590747*	5	65.508513	0.5487	2.0154	0.156157	0.0263	0.0130189932
ss117963783		5	65.557655	0.5308	3.4544	0.063504	0.0331	0.0165050552
ss86335384		5	65.611738	0.5308	3.4544	0.063504	0.0331	0.0165050552
ss61490732	*rs41590737*	5	65.726669	0.7851	3.3389	0.068086	0.0411	0.0138715341
ss86318325		5	65.845475	0.7794	0.0802	0.777057	0.0064	0.0022017468
ss61555883	*rs41647796*	5	66.07573	0.1612	0.4269	0.513720	0.0169	0.0045589576
ss61555884	*rs41647797*	5	66.177655	0.1261	1.7401	0.187556	0.0363	0.0080063769
ss86307683		5	66.26943	0.7335	0.2109	0.646172	0.0098	0.0038250072
ss61513013	*rs41609613*	5	66.398152	0.9312	0.3140	0.575430	0.0207	0.0026453324
ss61555851	*rs41647766*	5	66.457435	0.1024	5.7241	0.016998	0.0726	0.0133425177
ss86338129		5	66.656794	0.5731	0.0243	0.876191	0.0030	0.0014449578
ss61555853	*rs41647768*	5	66.684286	0.8317	9.7083	0.001910	0.0746	0.0208955262
ss61490721	*rs41590126*	5	66.76927	0.4742	0.2216	0.637944	0.0087	0.0043166334

ss61496818	*rs41592763*	5	66.802942	0.0802	0.0004	0.983168	0.0007	0.0001082333
	rs29009717	5	66.843803	0.0709	0.0679	0.794495	0.0094	0.0012378601
ss86330968		5	66.878452	0.7206	3.8170	0.051136	0.0397	0.0159932158
ss117967642		5	66.901704	0.6511	3.5322	0.060604	0.0356	0.0161960363
ss86302692		5	67.308526	0.1246	0.0069	0.933645	0.0024	0.0005137328
ss86328200		5	67.346752	0.0974	7.3504	0.006870	0.0842	0.0148034881
ss86337867		5	67.594681	0.7135	0.9566	0.328388	0.0197	0.0080666261
ss86328140		5	67.653431	0.1153	2.4495	0.118018	0.0440	0.0089829587
	rs29012227	5	67.700905	0.8703	0.0482	0.826301	0.0061	0.0013847003
ss86285179		5	67.793652	0.2342	0.9817	0.322124	0.0224	0.0080437190
ss117967709		5	67.879236	0.0809	5.1973	0.022924	0.0765	0.0113781029
ss86338379		5	67.956066	0.5494	0.3246	0.569067	0.0106	0.0052356338
ss86275606		5	67.982625	0.3087	0.9939	0.319137	0.0198	0.0084697190
ss86337396		5	68.010221	0.3009	1.5292	0.216654	0.0247	0.0104068443
ss86310376		5	68.059572	0.6798	0.1827	0.669213	0.0083	0.0036043348
ss61476880	*rs41580131*	5	68.304742	0.2521	3.1542	0.076171	0.0367	0.0138439112
ss86302087		5	68.361574	0.3295	1.2201	0.269718	0.0214	0.0094729309
ss86276271		5	68.389821	0.5301	1.9000	0.168523	0.0254	0.0126588868
ss86333631		5	68.521314	0.3080	0.0746	0.784788	0.0055	0.0023493814
ss86290349		5	68.697074	0.6311	4.5415	0.033433	0.0393	0.0182838269
ss61490756	*rs41590761*	5	68.762564	0.1074	1.9136	0.167012	0.0402	0.0077174888
ss61488849	*rs41587390*	5	68.912199	0.0910	3.5405	0.060305	0.0586	0.0096915363
	rs29016129	5	68.957931	0.0924	3.4549	0.063486	0.0576	0.0096600070
	rs29003804	5	69.087625	0.9105	1.2625	0.261559	0.0364	0.0059408957
ss61471039	*rs41566611*	5	69.152442	0.5838	0.4759	0.490500	0.0129	0.0062787093
	rs29019791	5	69.384406	0.7937	2.9184	0.088023	0.0390	0.0127647596
ss86291082		5	69.519099	0.9341	2.0023	0.157506	0.0536	0.0066048604
ss61504011	*rs41601801*	5	69.658437	0.3524	0.0067	0.934640	0.0016	0.0007091140
ss86331272		5	69.768248	0.5451	6.7880	0.009374	0.0468	0.0232235896
ss86304768		5	69.812225	0.3789	0.4864	0.485772	0.0134	0.0062987690
	rs29011749	5	69.845096	0.5394	0.0329	0.856191	0.0033	0.0016363870
ss86318909		5	69.948865	0.3374	3.9145	0.048265	0.0374	0.0167173913
ss86297136		5	70.136778	0.1612	7.1732	0.007575	0.0648	0.0175171854
	rs29027362	5	70.277351	0.6282	5.6686	0.017539	0.0449	0.0209654214
ss86293765		5	70.505309	0.1053	0.3638	0.546574	0.0184	0.0034617532
ss86329267		5	70.569839	0.6053	4.3173	0.038092	0.0387	0.0184868048
ss61493712	*rs41590238*	5	70.594855	0.4105	11.1740	0.000874	0.0610	0.0295020291
ss61562356	*rs43743037*	5	70.636536	0.7600	12.3278	0.000475	0.0732	0.0266831312
ss61562354	*rs41660455*	5	70.679596	0.7708	9.4860	0.002152	0.0654	0.0231174355
ss117962879		5	70.71986	0.0895	0.3513	0.553592	0.0183	0.0029918294
ss86283624		5	70.750898	0.5788	1.0407	0.308018	0.0191	0.0093309276
ss86341050		5	70.980624	0.5158	0.2811	0.596173	0.0098	0.0049098937
ss86335125		5	71.073539	0.6297	2.6383	0.104767	0.0315	0.0146841448
ss86293228		5	71.120681	0.1146	0.4345	0.510009	0.0195	0.0039612159
ss86335889		5	71.169823	0.6375	1.1230	0.289633	0.0208	0.0096128746
ss86332822		5	71.381565	0.5845	0.6548	0.418690	0.0153	0.0074386880
	rs29027014	5	71.427363	0.4040	0.0574	0.810656	0.0046	0.0021960906

ss61512962	*rs41609564*	5	71.536766	0.3746	0.6499	0.420424	0.0155	0.0072533239
ss86288510		5	71.572795	0.4305	0.6542	0.418900	0.0150	0.0073686014
ss61555892	*rs41647805*	5	71.627713	0.6340	0.5267	0.468251	0.0139	0.0064526404
ss61555894		5	71.750159	0.6196	0.3166	0.573837	0.0108	0.0051004493
ss86274372		5	72.003902	0.1841	0.4336	0.510462	0.0154	0.0046209357
ss86291162		5	72.205151	0.3918	3.9949	0.046028	0.0378	0.0179945246
ss86283830		5	72.449007	0.3653	3.5724	0.059162	0.0361	0.0167236569
ss86319418		5	72.515408	0.5573	3.6972	0.054912	0.0359	0.0176947825
ss61465929	*rs41565193*	5	72.724501	0.2242	2.6045	0.107015	0.0363	0.0126276006
ss61541378	*rs41643212*	5	72.906053	0.3102	0.3494	0.554655	0.0118	0.0050654969
ss86301733		5	72.950723	0.3381	0.0076	0.930379	0.0017	0.0007666436
ss61541376	*rs41643210*	5	72.980152	0.5387	0.0237	0.877585	0.0029	0.0014266435
ss86340588		5	73.020557	0.7085	0.0036	0.952438	0.0013	0.0005196261
ss86304863		5	73.048762	0.7249	3.9720	0.046652	0.0406	0.0161752019
ss86292643		5	73.119237	0.6784	0.1497	0.698977	0.0078	0.0033959165
ss86340458		5	73.211725	0.4878	0.0452	0.831650	0.0039	0.0019640536
ss61541372	*rs41643207*	5	73.235051	0.5372	0.4067	0.523867	0.0117	0.0057995780
ss86339457		5	73.273822	0.8660	1.5120	0.219246	0.0332	0.0076945257
ss61541368	*rs41643203*	5	73.297801	0.8883	0.9366	0.333491	0.0282	0.0055977485
ss86319548		5	73.319544	0.5014	0.0050	0.943575	0.0013	0.0006658052
	rs29022240	5	73.568308	0.7027	0.0016	0.967926	0.0008	0.0003400089
ss61502325	*rs41606235*	5	73.634207	0.4112	0.8999	0.343136	0.0179	0.0086898534
ss117967685		5	73.892592	0.1404	1.2552	0.262957	0.0296	0.0071566799
ss61542868	*rs41636991*	5	73.929236	0.3682	2.1582	0.142266	0.0278	0.0129328283
ss86303397		5	73.964924	0.1275	1.4379	0.230890	0.0330	0.0073392361
	rs29013289	5	74.052333	0.2550	0.0534	0.817293	0.0049	0.0018533988
	rs29016208	5	74.226161	0.7206	0.3691	0.543685	0.0124	0.0049828009
ss86301749		5	74.256167	0.7830	0.3938	0.530504	0.0140	0.0047539302
ss117967873		5	74.285886	0.7650	0.5322	0.465925	0.0164	0.0059124599
	rs29027086	5	74.321916	0.6576	0.0512	0.821000	0.0046	0.0020680406
ss86311432		5	74.344114	0.7328	1.1142	0.291528	0.0227	0.0088877865
ss61542874	*rs41636997*	5	74.490654	0.3517	2.4146	0.120667	0.0295	0.0134559911
ss86326353		5	74.523359	0.8904	0.8140	0.367256	0.0267	0.0052101492
ss86284957		5	74.780989	0.2170	0.0042	0.948095	0.0015	0.0005176528
ss117967793		5	74.823222	0.7407	0.4845	0.486614	0.0148	0.0056999662
ss86314209		5	74.852133	0.8947	5.2288	0.022515	0.0709	0.0133600239
ss61555929	*rs41648942*	5	74.87824	0.2013	0.0733	0.786719	0.0063	0.0020285848
ss61555924	*rs41648937*	5	74.978375	0.1734	0.3429	0.558366	0.0143	0.0040987850
ss86295448		5	75.00499	0.5215	0.1436	0.704829	0.0071	0.0035241374
	rs29018750	5	75.063295	0.5322	0.1159	0.733658	0.0064	0.0031627673
ss61555923	*rs41648936*	5	75.084465	0.2894	0.2068	0.649454	0.0093	0.0038267956
ss86296775		5	75.10946	0.2880	0.3306	0.565464	0.0118	0.0048388951
ss61504010	*rs41601800*	5	75.141726	0.7679	0.0000	0.995021	0.0001	0.0000508396
ss86338355		5	75.170631	0.8754	0.7471	0.387693	0.0240	0.0052371517
ss61510446	*rs41604573*	5	75.27339	0.2128	0.1306	0.717953	0.0081	0.0027086310
	rs29016859	5	75.59374	0.3646	0.5615	0.453921	0.0140	0.0064666498
ss86309018		5	75.705848	0.3761	0.2999	0.584120	0.0101	0.0047360008

ss86311691		5	75.805132	0.6239	0.4140	0.520181	0.0119	0.0055627295
ss86309610		5	75.853519	0.1096	2.6436	0.104418	0.0464	0.0090519574
ss86313028		5	75.903427	0.1504	1.4581	0.227636	0.0306	0.0078266194
ss86341140		5	75.950948	0.7880	0.2948	0.587325	0.0121	0.0040299403
ss86292950		5	75.982263	0.1669	0.3458	0.556665	0.0145	0.0040196981
ss86310614		5	76.077475	0.1024	2.2755	0.131886	0.0445	0.0081758112
ss86307641		5	76.282701	0.3560	0.3619	0.547644	0.0116	0.0053229935
ss86338354		5	76.30696	0.7199	0.1754	0.675512	0.0086	0.0034666546
ss86337860		5	76.354312	0.3976	1.2227	0.269220	0.0206	0.0098678737
ss86299798		5	76.395002	0.7951	3.9811	0.046404	0.0470	0.0153110136
ss86316433		5	76.428731	0.4513	1.4615	0.227107	0.0231	0.0114237519
ss86316308		5	76.574446	0.2529	0.5114	0.474759	0.0150	0.0056748759
ss86287728		5	76.630478	0.6934	0.0000	0.998265	0.0000	0.0000184922
ss86291554		5	76.653386	0.7615	1.6144	0.204298	0.0277	0.0100495036
ss86316391		5	76.682086	0.1576	0.0594	0.807540	0.0060	0.0015970693
ss46526764	*rs41257215*	5	76.714911	0.8345	0.2412	0.623520	0.0120	0.0033145999
ss86340205		5	76.788488	0.7493	1.9976	0.157998	0.0300	0.0112792246
ss61555969	*rs41648982*	5	76.882813	0.3066	0.0390	0.843427	0.0039	0.0016734799
ss61490776	*rs41590781*	5	76.927344	0.7951	0.7450	0.388369	0.0192	0.0062611675
ss61555965	*rs41648978*	5	76.947975	0.7464	0.1326	0.715898	0.0076	0.0028841185
ss86325399		5	76.985632	0.5000	0.9380	0.333141	0.0174	0.0087207003
ss86298866		5	77.045941	0.8410	0.0925	0.761069	0.0074	0.0019775010
ss86332778		5	77.088602	0.7593	0.8302	0.362529	0.0204	0.0074597667
ss86300943		5	77.146408	0.9169	4.6363	0.031645	0.0728	0.0110951692
ss86339707		5	77.16763	0.5115	1.0439	0.307280	0.0183	0.0091538482
ss86290558		5	77.202262	0.7378	0.5203	0.470963	0.0153	0.0059143540
ss61469827	*rs41572821*	5	77.24755	0.6225	0.5331	0.465533	0.0137	0.0064522547
ss86303449		5	77.292932	0.3360	0.9170	0.338597	0.0186	0.0082848465
ss86312122		5	77.316926	0.2099	2.1157	0.146250	0.0332	0.0110084237
ss64295570	*rs42570401*	5	77.345546	0.7629	0.7613	0.383231	0.0191	0.0069199889
ss86338278		5	77.366076	0.5057	0.0402	0.841095	0.0037	0.0018729400
ss86337970		5	77.492406	0.2744	0.2146	0.643297	0.0095	0.0037894836
ss86309934		5	77.517622	0.6948	0.9137	0.339461	0.0191	0.0081039664
ss86318187		5	77.546765	0.4090	2.0010	0.157644	0.0264	0.0127714805
ss86319352		5	77.591403	0.0745	0.2491	0.617899	0.0175	0.0024138591
ss86318576		5	77.758525	0.2801	1.0348	0.309379	0.0210	0.0084555317
ss86329229		5	77.805842	0.2801	1.0348	0.309379	0.0210	0.0084555317
ss86317303		5	77.969543	0.7163	0.3756	0.540188	0.0125	0.0050881511
ss86319677		5	78.209179	0.1024	0.0129	0.909736	0.0034	0.0006187239
	rs29023691	5	78.222736	0.7106	0.3030	0.582173	0.0116	0.0047661643
ss86333127		5	78.338146	0.8997	0.1351	0.713305	0.0110	0.0019939169
ss61522119	*rs41623805*	5	78.425256	0.6082	0.4480	0.503493	0.0126	0.0060050708
ss86295475		5	78.5607	0.8553	1.0768	0.299770	0.0277	0.0068658449
ss86336745		5	78.672224	0.6726	0.5633	0.453189	0.0148	0.0065261108
ss86274546		5	78.829932	0.2493	0.1350	0.713456	0.0080	0.0029781307
ss86317837		5	78.914355	0.1332	0.5450	0.460599	0.0200	0.0046143269
ss86325696		5	78.95404	0.7049	1.6397	0.200797	0.0258	0.0107508912

ss61555994	*rs41649007*	5	79.01156	0.7099	0.6217	0.430692		0.0163	0.0067190935
ss86331488		5	79.05221	0.5723	2.6563	0.103594		0.0299	0.0146432462
ss86315426		5	79.085074	0.5745	2.9011	0.088966		0.0313	0.0152796982
ss86330623		5	79.171292	0.0903	0.0795	0.778084		0.0090	0.0014858627
ss86337935		5	79.195619	0.4377	3.5849	0.058722		0.0341	0.0168003088
ss86290211		5	79.220556	0.4635	3.5601	0.059601		0.0342	0.0170303175
ss86279452		5	79.358893	0.2966	0.0042	0.948643		0.0013	0.0005531093
ss86330487		5	79.380322	0.6956	0.2642	0.607397		0.0107	0.0045254275
ss86322490		5	79.410796	0.6798	0.6858	0.407878		0.0164	0.0071409133
ss86303111		5	79.46505	0.5222	0.3241	0.569357		0.0108	0.0053915215
ss86340614		5	79.537934	0.3739	0.3771	0.539337		0.0120	0.0056292506
ss61490796	*rs41590801*	5	79.625349	0.4334	0.8062	0.369562		0.0170	0.0083591037
ss86330028		5	79.699554	0.2822	0.9270	0.335970		0.0198	0.0080081208
ss86299048		5	79.773373	0.6340	0.0151	0.902164		0.0024	0.0010939125
ss86317921		5	79.797889	0.3639	0.0014	0.970588		0.0007	0.0003276186
ss86309308		5	80.140048	0.7235	0.1036	0.747664		0.0068	0.0027182342
ss61510466	*rs41604593*	5	80.175282	0.9004	0.0288	0.865274		0.0053	0.0009588385
ss86341323		5	80.256053	0.9284	0.4262	0.514061		0.0228	0.0030286768
ss86302865		5	80.345044	0.5000	0.2412	0.623529		0.0094	0.0047013253
ss86340617		5	80.368329	0.6769	0.5250	0.468940		0.0145	0.0063602456
ss86323308		5	80.391651	0.2192	0.3064	0.580056		0.0125	0.0042698900
ss86299696		5	80.418079	0.2894	0.8823	0.347911		0.0193	0.0079501179
ss61469020	*rs41566931*	5	80.459083	0.2242	0.6610	0.416468		0.0182	0.0063320933
ss86286083		5	80.4812	0.3897	0.4805	0.488445		0.0136	0.0064559511
ss86330025		5	80.520085	0.4663	0.0098	0.921318		0.0019	0.0009625721
ss86329988		5	80.617143	0.1734	0.0060	0.938233		0.0019	0.0005365538
ss61556030	*rs41650242*	5	80.699306	0.4850	0.6883	0.407038		0.0159	0.0079481395
ss61523629	*rs41622292*	5	80.742886	0.5759	0.5833	0.445276		0.0150	0.0073257991
ss86311241		5	80.799913	0.5817	0.3296	0.566059		0.0112	0.0054727331
ss86303871		5	80.830137	0.5394	0.0127	0.910242		0.0021	0.0010443980
ss86336032		5	80.88027	0.5143	0.5702	0.450440		0.0141	0.0070312497
ss86338696		5	80.907729	0.3331	0.4487	0.503174		0.0132	0.0058678033
ss61490810		5	80.941261	0.5136	0.0881	0.766699		0.0053	0.0026620403
ss61565823	*rs41663866*	5	80.965297	0.3073	0.5914	0.442151		0.0157	0.0066960204
ss86297623		5	81.003369	0.5029	0.2236	0.636436		0.0088	0.0043815949
ss86308210		5	81.038347	0.4971	0.1685	0.681576		0.0076	0.0037911423
ss86332640		5	81.251722	0.7557	0.8709	0.351026		0.0206	0.0076186613
ss86303470		5	81.312782	0.2385	1.0003	0.317592		0.0211	0.0076662070
ss86337303		5	81.431996	0.7564	0.8796	0.348624		0.0207	0.0076416058
ss86339869		5	81.535732	0.5523	0.1937	0.659991		0.0083	0.0041286636
ss86292444		5	81.579061	0.4735	0.0000	0.995937		0.0001	0.0000473554
ss86337262		5	81.603591	0.4735	0.0000	0.995937		0.0001	0.0000473554
ss86310331		5	81.691901	0.7099	0.6993	0.403318		0.0173	0.0071450629
ss61516921	*rs41620220*	5	81.722415	0.4900	0.3432	0.558160		0.0109	0.0054303752
ss86306911		5	81.760666	0.2357	1.3247	0.250137		0.0260	0.0093673772
ss64853889	*rs42949956*	5	81.807214	0.7056	1.1147	0.291426		0.0219	0.0090781633
ss86324892		5	81.830122	0.3352	0.3785	0.538621		0.0125	0.0055812734

ss117967625		5	81.874749	0.6662	0.4295	0.512435	0.0133	0.0059268612
ss64718454	*rs42812985*	5	81.913048	0.5559	0.3133	0.575860	0.0107	0.0052920564
ss64964039	*rs43054854*	5	81.979214	0.1583	0.1869	0.665673	0.0110	0.0029203612
ss61493910	*rs41591125*	5	82.012037	0.4670	0.0097	0.921674	0.0018	0.0008940905
ss64310362	*rs42578610*	5	82.050819	0.1605	0.1021	0.749402	0.0081	0.0021764726
ss64310318	*rs42578588*	5	82.080903	0.4649	0.0032	0.954891	0.0010	0.0005165447
ss61478162	*rs41574677*	5	82.25549	0.1755	0.0030	0.956061	0.0013	0.0003864612
	rs29015767	5	82.308997	0.1755	0.0030	0.956061	0.0013	0.0003864612
ss64288680	*rs42562777*	5	82.348249	0.1777	0.0193	0.889586	0.0034	0.0009812090
ss64288746	*rs42562810*	5	82.374418	0.8216	0.0129	0.909735	0.0027	0.0008030392
ss64946936	*rs43040321*	5	82.425057	0.8245	0.0030	0.956061	0.0013	0.0003864612
ss64288324	*rs42560399*	5	82.461955	0.8245	0.0030	0.956061	0.0013	0.0003864612
ss86291183		5	82.556838	0.4957	0.3975	0.528579	0.0118	0.0058910092
ss65355394	*rs43436389*	5	82.614683	0.1576	0.1376	0.710798	0.0094	0.0024995206
ss61556073	*rs41650285*	5	82.635193	0.6533	0.1939	0.659842	0.0089	0.0040296871
ss117967651		5	82.792168	0.3317	0.5061	0.477087	0.0145	0.0064227390
ss65356197	*rs43441981*	5	82.829033	0.5057	0.3776	0.539069	0.0115	0.0057411397
ss61480090	*rs41585344*	5	82.888535	0.5458	0.2206	0.638704	0.0089	0.0043975347
ss61533822	*rs41629983*	5	82.928824	0.3582	0.0032	0.955035	0.0011	0.0005210231
ss86311052		5	82.966712	0.3188	1.6396	0.200802	0.0263	0.0114078655
ss86330447		5	83.080463	0.6862	1.2728	0.259631	0.0231	0.0099415079
ss61556104	*rs41650316*	5	83.24648	0.6798	1.8463	0.174656	0.0278	0.0121167211
ss86329480		5	83.294153	0.6777	2.0878	0.148930	0.0295	0.0128671285
ss86336591		5	83.501139	0.6390	3.8500	0.050144	0.0383	0.0176710688
ss61556107	*rs41650319*	5	83.624593	0.1160	3.5167	0.061173	0.0522	0.0107127489
ss86328027		5	83.668124	0.7178	0.0009	0.975504	0.0006	0.0002452429
ss61490835	*rs41591840*	5	83.715993	0.2701	0.1242	0.724605	0.0074	0.0029243921
	rs29018073	5	83.776222	0.2715	0.1014	0.750279	0.0067	0.0026604758
ss61471361		5	83.897768	0.4319	0.0595	0.807433	0.0045	0.0021924626
ss61556121	*rs41651433*	5	83.989225	0.2106	1.1594	0.281965	0.0250	0.0083227845
ss86331997		5	84.068558	0.1067	1.4122	0.235089	0.0357	0.0068040355
ss86326250		5	84.213229	0.3231	1.6297	0.202169	0.0257	0.0112366218
ss86285496		5	84.217004	0.6633	0.3695	0.543498	0.0122	0.0054476126
ss117967839		5	84.279557	0.3073	0.2538	0.614539	0.0102	0.0043238921
	rs29016994	5	84.391581	0.2779	0.0242	0.876433	0.0032	0.0012726307
ss86322091		5	84.414929	0.1347	0.0978	0.754547	0.0085	0.0019860356
ss86314839		5	84.441704	0.8660	0.1219	0.727072	0.0095	0.0022103635
ss61556054	*rs41650266*	5	84.462454	0.1182	0.0036	0.952481	0.0017	0.0003560992
ss86337200		5	84.49794	0.2722	1.6341	0.201569	0.0269	0.0106500191
ss61570168	*rs41665833*	5	84.676527	0.1160	0.4044	0.525056	0.0185	0.0037902613
ss86293973		5	84.81234	0.3940	0.1584	0.690784	0.0076	0.0036497323
ss86291351		5	84.854836	0.3617	3.6437	0.056692	0.0372	0.0171677685
ss86295318		5	84.932965	0.3639	1.7347	0.188249	0.0256	0.0118299484
ss61473396	*rs41569814*	5	84.990071	0.3202	0.0004	0.983837	0.0004	0.0001755869
ss86303380		5	85.209328	0.7020	3.3460	0.067795	0.0370	0.0154844918
ss86334671		5	85.325856	0.7708	0.8025	0.370663	0.0200	0.0070844560
ss86340818		5	85.391596	0.8159	0.1650	0.684704	0.0094	0.0028244749

ss86321090		5	85.420299	0.8954	0.0223	0.881346	0.0046	0.0008528770
	rs29012617	5	85.482143	0.8216	0.2075	0.648890	0.0107	0.0031341071
ss61507042	*rs41602344*	5	85.502405	0.2228	0.1971	0.657239	0.0100	0.0034640546
ss61507043	*rs41602345*	5	85.534665	0.3173	0.0073	0.931862	0.0017	0.0007411257
ss61566488	*rs41664318*	5	85.589896	0.4642	0.5643	0.452799	0.0141	0.0070297282
ss61529134	*rs41632944*	5	85.621882	0.4183	0.7375	0.390759	0.0167	0.0081147938
ss61490885	*rs41591889*	5	85.928491	0.8524	0.0099	0.920928	0.0026	0.0006440768
ss86296399		5	86.050668	0.2751	0.2952	0.587064	0.0114	0.0045455337
ss117967812		5	86.097969	0.2342	0.9551	0.328755	0.0215	0.0077259924
ss86273559		5	86.178491	0.2006	0.3282	0.566895	0.0135	0.0043395238
ss86300440		5	86.246835	0.2958	0.1749	0.675917	0.0087	0.0036378076
ss61556220	*rs41652632*	5	86.293274	0.6160	0.6539	0.418988	0.0155	0.0073269939
ss86277315		5	86.338527	0.3181	0.0818	0.774951	0.0058	0.0025299651
ss117967856		5	86.546096	0.7063	0.0494	0.824160	0.0046	0.0019085075
ss86296894		5	86.594626	0.1583	0.1718	0.678633	0.0105	0.0027980008
ss61556140	*rs41651452*	5	86.619373	0.3066	7.4443	0.006524	0.0547	0.0232546548
ss86327794		5	86.679675	0.8403	0.3219	0.570629	0.0147	0.0039373848
ss61556128	*rs41651440*	5	86.720672	0.4327	7.3123	0.007016	0.0528	0.0259146074
ss61490847	*rs41591852*	5	86.756396	0.1447	0.9829	0.321822	0.0256	0.0063398383
ss61475774	*rs41577345*	5	87.173206	0.2844	1.6057	0.205515	0.0262	0.0106518214
ss61500294	*rs41598548*	5	87.21818	0.0845	2.6908	0.101384	0.0532	0.0082360126
ss86332120		5	87.296619	0.4556	5.9567	0.014910	0.0460	0.0228175971
	rs29014852	5	87.354092	0.7063	0.2092	0.647529	0.0094	0.0038839800
ss86289949		5	87.41231	0.0372	0.2330	0.629449	0.0234	0.0016768802
ss61525682		5	87.826082	0.5029	3.4683	0.062976	0.0346	0.0172838222
ss86313440		5	87.896982	0.5838	10.2273	0.001446	0.0607	0.0295016185
ss86315478		5	87.959844	0.2665	0.0393	0.842921	0.0040	0.0015771443
ss61525679	*rs41621984*	5	88.007172	0.2736	0.9766	0.323374	0.0212	0.0084200470
ss86311380		5	88.126959	0.1146	2.1588	0.142210	0.0407	0.0082600836
	rs29018202	5	88.2105	0.8868	1.4285	0.232419	0.0353	0.0070818919
ss86303481		5	88.252891	0.1855	5.8259	0.016049	0.0585	0.0176888312
ss86302393		5	88.299415	0.1032	1.2701	0.260139	0.0343	0.0063398401
ss86337963		5	88.422415	0.3911	2.9181	0.088035	0.0329	0.0156927636
ss61472646	*rs41572869*	5	88.529662	0.6769	3.9821	0.046375	0.0386	0.0168829164
ss61498341	*rs43711242*	5	88.57752	0.4348	0.9051	0.341745	0.0181	0.0088986409
ss86340713		5	88.646348	0.3603	3.4537	0.063531	0.0354	0.0163364216
ss61523948	*rs41624303*	5	88.671798	0.3675	1.8750	0.171340	0.0263	0.0122373157
ss86339758		5	88.698929	0.1948	1.8451	0.174798	0.0316	0.0099091546
ss86322530		5	88.803877	0.8503	11.3107	0.000813	0.0866	0.0220545695
ss61506991	*rs41601395*	5	88.892982	0.4778	0.0190	0.890284	0.0025	0.0012608780
ss61507845	*rs41599836*	5	89.207112	0.4807	1.3023	0.254185	0.0211	0.0105451528
ss117963832		5	89.297079	0.7550	0.5433	0.461304	0.0157	0.0058035118
ss61490891	*rs41591894*	5	89.352038	0.8868	1.9490	0.163143	0.0408	0.0081913732
ss117963823		5	89.471574	0.6211	0.8512	0.356538	0.0171	0.0080539391
ss61556237	*rs41652649*	5	89.694748	0.2034	0.0184	0.892090	0.0032	0.0010296852
ss86293309		5	89.832592	0.6218	1.3488	0.245888	0.0219	0.0103212164
ss86339726		5	89.885309	0.1640	0.0123	0.911677	0.0028	0.0007613940

ss86296194		5	89.916368	0.1261	0.5516	0.457922	0.0208	0.0045802483
ss86274257		5	89.963701	0.6640	0.4571	0.499193	0.0134	0.0059786562
ss61556255	*rs41652667*	5	89.992022	0.3016	0.1579	0.691207	0.0080	0.0033800582
ss86304546		5	90.096455	0.8510	0.1374	0.710957	0.0100	0.0025330057
ss86300153		5	90.134541	0.1554	0.0492	0.824496	0.0057	0.0014900193
ss61556252	*rs41652664*	5	90.16879	0.2020	0.7679	0.381163	0.0201	0.0064768367
ss61564389	*rs41660560*	5	90.278464	0.4026	0.7445	0.388523	0.0161	0.0077233332
ss61490910	*rs41591913*	5	90.422342	0.7400	11.4087	0.000772	0.0721	0.0277629661
ss86332959		5	90.472733	0.7149	0.0079	0.929145	0.0018	0.0007302849
ss61556289	*rs41652700*	5	90.582089	0.7865	0.1002	0.751697	0.0072	0.0024124258
	rs29013879	5	90.670436	0.1562	0.5791	0.446932	0.0192	0.0050600592
ss61537860	*rs41635650*	5	90.775506	0.4226	1.4549	0.228147	0.0218	0.0106601299
ss61537861	*rs41635651*	5	90.777948	0.9054	1.1511	0.283692	0.0338	0.0057909770
ss61540695	*rs43743836*	5	90.849344	0.8711	0.7486	0.387223	0.0247	0.0055379624
ss61511833	*rs41616137*	5	90.96965	0.2414	0.0267	0.870338	0.0035	0.0012990011
ss86304189		5	91.062496	0.5301	1.7537	0.185848	0.0242	0.0120662781
ss86301835		5	91.165726	0.7801	2.8159	0.093783	0.0366	0.0125725638
ss86313142		5	91.294088	0.2736	1.9749	0.160378	0.0297	0.0118100461
ss86337468		5	91.354493	0.7901	4.3696	0.036947	0.0476	0.0157894973
ss117967634		5	91.418743	0.3725	0.2241	0.636053	0.0089	0.0041837622
ss61490904	*rs41591907*	5	91.479479	0.4499	3.4418	0.063989	0.0338	0.0167078966
ss86320410		5	91.536955	0.6275	0.1110	0.739157	0.0062	0.0029167220
ss61556278	*rs41652689*	5	91.575754	0.5795	1.8640	0.172602	0.0251	0.0122236076
ss61556282	*rs41652693*	5	91.616162	0.8360	2.6849	0.101755	0.0413	0.0113275953
ss61556288	*rs41652699*	5	91.672259	0.1991	1.2498	0.263984	0.0254	0.0081060094
ss117967628		5	91.817478	0.2092	0.6279	0.428393	0.0184	0.0060757389
ss65357165	*rs43445847*	5	91.886763	0.7264	0.0003	0.986338	0.0004	0.0001428724
ss61556302	*rs41652713*	5	91.933439	0.3832	0.4435	0.505656	0.0127	0.0060208485
ss61556303	*rs41652714*	5	91.98004	0.0695	0.0112	0.915794	0.0039	0.0005025030
ss61490914	*rs41591917*	5	92.14262	0.4133	0.1311	0.717361	0.0067	0.0032526132
ss86336109		5	92.172835	0.5301	0.0034	0.953213	0.0011	0.0005268042
ss61556309	*rs41652720*	5	92.232472	0.4814	0.0809	0.776187	0.0051	0.0025619454
ss61499191	*rs43709087*	5	92.258527	0.5638	0.6190	0.431694	0.0143	0.0070220751
ss117967738		5	92.297979	0.9298	0.7295	0.393343	0.0302	0.0039414643
ss86339983		5	92.502081	0.2400	0.7240	0.395132	0.0184	0.0067128317
ss86284955		5	92.623904	0.2285	1.9974	0.158021	0.0305	0.0107427161
ss86320683		5	92.671126	0.1060	4.6026	0.032269	0.0644	0.0122024992
	rs29024062	5	92.754106	0.0695	0.0232	0.878885	0.0055	0.0007081680
ss86333289		5	92.802901	0.7264	1.0081	0.315707	0.0205	0.0081493703
ss64392424	*rs42614867*	5	92.931085	0.5817	1.3097	0.252834	0.0216	0.0104880407
ss86335885		5	92.99413	0.0681	1.0113	0.314945	0.0381	0.0048306940
ss61569176	*rs41665561*	5	93.046854	0.1032	1.1777	0.278211	0.0324	0.0059957718
ss63359957	*rs42206857*	5	93.072548	0.8574	0.1849	0.667336	0.0115	0.0028227835
ss61479340	*rs41577209*	5	93.100618	0.5501	13.3198	0.000282	0.0710	0.0351397500
ss86304203		5	93.15375	0.6898	0.6985	0.403588	0.0169	0.0072416403
ss61479348	*rs41577217*	5	93.217252	0.3933	1.8034	0.179745	0.0247	0.0118099940
ss61499525	*rs43710712*	5	93.275618	0.2686	0.0224	0.881182	0.0032	0.0012393461

	rs29012671	5	93.343335	0.5079	13.2151	0.000298	0.0671	0.0335392356
ss86316102		5	93.457264	0.6196	2.6268	0.105529	0.0305	0.0143720092
ss86295484		5	93.640403	0.1268	4.5751	0.032786	0.0591	0.0130932532
ss65267345	*rs43350302*	5	93.699286	0.2536	0.4180	0.518143	0.0137	0.0051683633
	rs29011704	5	93.79822	0.5960	0.0224	0.880973	0.0028	0.0013297878
ss86284316		5	93.821567	0.7414	0.3755	0.540207	0.0130	0.0049851840
ss65356571	*rs43440753*	5	93.858263	0.2106	3.6563	0.056268	0.0421	0.0139968575
ss61556224	*rs41652636*	5	93.880242	0.6784	3.3982	0.065692	0.0364	0.0158959998
ss65356594	*rs43440776*	5	93.919721	0.2751	11.9671	0.000574	0.0700	0.0279111446
ss61556228	*rs41652640*	5	93.968342	0.4198	0.1309	0.717582	0.0066	0.0032266672
ss61556232	*rs41652644*	5	94.14088	0.4663	0.5693	0.450800	0.0139	0.0069000680
ss86341060		5	94.226441	0.7951	1.0813	0.298769	0.0238	0.0077466578
ss65358431	*rs43439308*	5	94.261896	0.0752	2.2168	0.136972	0.0524	0.0072877476
ss86285779		5	94.284624	0.3023	0.3247	0.568974	0.0114	0.0048270801
ss65358406	*rs43439283*	5	94.309095	0.1848	1.0128	0.314592	0.0236	0.0071214749
ss61497073	*rs41594003*	5	94.386403	0.3961	0.0001	0.992543	0.0002	0.0000843304
		5	94.444027	0.5616	6.2926	0.012351	0.0481	0.0237016038
ss65358343	*rs43438620*	5	94.506734	0.5509	3.5378	0.060403	0.0335	0.0166004660
ss65357936	*rs43443417*	5	94.556315	0.5251	3.8267	0.050842	0.0347	0.0172948471
ss61556315	*rs41652726*	5	94.578773	0.4420	1.3814	0.240257	0.0213	0.0104884370
ss64604046	*rs42717013*	5	94.6072	0.1683	9.4462	0.002199	0.0726	0.0203171185
ss61515618	*rs41615970*	5	94.639968	0.1691	9.3417	0.002326	0.0721	0.0202578168
ss63954700	*rs42406600*	5	94.732589	0.7027	0.0096	0.922156	0.0019	0.0008060353
ss63954416	*rs42405458*	5	94.781853	0.2428	2.5357	0.111755	0.0346	0.0127262471
ss61475944	*rs41578409*	5	94.809922	0.1497	0.0775	0.780756	0.0072	0.0018272934
ss86292412		5	94.882027	0.2629	0.0567	0.811796	0.0052	0.0019988044
ss63954198	*rs42404349*	5	94.912299	0.4176	7.3004	0.007062	0.0482	0.0234472048
	rs29024365	5	94.94849	0.5745	0.3919	0.531530	0.0117	0.0057138492
	rs29023212	5	94.981763	0.5509	2.9487	0.086393	0.0315	0.0155705863
ss86331972		5	95.068491	0.0967	0.0000	0.999127	0.0000	0.0000060384
ss86290589		5	95.100936	0.8044	0.2132	0.644452	0.0108	0.0034085219
ss64331868	*rs42589608*	5	95.412167	0.3087	4.3736	0.036862	0.0408	0.0174195632
ss64808026	*rs42901988*	5	95.43971	0.3059	3.7776	0.052345	0.0384	0.0162939682
ss63932909	*rs42396867*	5	95.451429	0.6884	4.0197	0.045359	0.0390	0.0167101933
ss61522846	*rs41622115*	5	95.498051	0.8138	0.0046	0.945774	0.0016	0.0004877356
ss63932458	*rs42394542*	5	95.538814	0.4105	4.0352	0.044946	0.0378	0.0182945795
ss63932494	*rs42394560*	5	95.571058	0.4685	0.5697	0.450648	0.0143	0.0071369891
ss63932382	*rs42393904*	5	95.595197	0.9284	0.0023	0.961516	0.0017	0.0002306921
ss86341523		5	95.708745	0.6812	0.2299	0.631727	0.0095	0.0041457878
ss86321611		5	95.818721	0.2464	4.2457	0.039720	0.0444	0.0164853156
ss86322341		5	95.878364	0.8202	0.0173	0.895280	0.0033	0.0009608052
ss86337685		5	95.978871	0.0938	0.3611	0.548065	0.0190	0.0032318923
ss86283541		5	96.029205	0.6074	0.1436	0.704816	0.0073	0.0034747024
ss86291750		5	96.126213	0.1669	0.0537	0.816868	0.0059	0.0016522946
ss86340691		5	96.166631	0.7815	0.0319	0.858340	0.0040	0.0013608336
ss61529025	*rs41631936*	5	96.263715	0.3489	1.4513	0.228726	0.0223	0.0101366635
ss61566468	*rs41664298*	5	96.301213	0.5451	1.5627	0.211694	0.0232	0.0114829853

ss61556326	*rs41653536*	5	96.336627	0.5451	1.5229	0.217602	0.0229	0.0113393265
ss61556336	*rs41653543*	5	96.358013	0.3489	2.2056	0.137964	0.0290	0.0131762316
ss61549584	*rs41649578*	5	96.394668	0.6225	1.8755	0.171287	0.0263	0.0123444858
	rs29010421	5	96.419166	0.3467	2.2141	0.137203	0.0290	0.0131410139
ss61549586	*rs41649580*	5	96.448995	0.5444	1.6443	0.200158	0.0239	0.0118607891
ss61487501	*rs41584068*	5	96.481082	0.5831	1.0365	0.308994	0.0192	0.0093251216
ss86288064		5	96.517829	0.4993	0.2139	0.643839	0.0084	0.0042159242
ss86338672		5	96.936269	0.1619	0.0075	0.930815	0.0022	0.0005974138
ss86340908		5	97.00361	0.7808	0.0559	0.813103	0.0054	0.0018471168
	rs29019289	5	97.117452	0.8847	0.0992	0.752881	0.0094	0.0019224562
ss86273870		5	97.22432	0.7686	0.2767	0.599071	0.0116	0.0041233151
ss61518121	*rs41611290*	5	97.296412	0.6289	2.0181	0.155879	0.0262	0.0122450843
ss117967593		5	97.370233	0.1798	0.0818	0.774999	0.0070	0.0020551224
ss86298048		5	97.487313	0.6877	0.1257	0.723079	0.0072	0.0030735632
ss61518127	*rs41611296*	5	97.515555	0.2987	3.4473	0.063778	0.0363	0.0151901490
	rs29018508	5	97.572285	0.8567	0.0067	0.934639	0.0022	0.0005335468
ss61518129	*rs41611298*	5	97.597051	0.6705	2.4015	0.121676	0.0292	0.0129138498
ss86332828		5	97.658635	0.7242	1.4661	0.226367	0.0251	0.0100152197
ss86295772		5	97.68635	0.5974	0.5369	0.463967	0.0138	0.0066306658
ss61518136	*rs41611305*	5	97.722066	0.6870	1.5763	0.209716	0.0244	0.0104939446
ss61518120	*rs41611289*	5	97.744509	0.4269	0.2428	0.622370	0.0090	0.0043901016
ss86330862		5	97.802852	0.2170	2.1369	0.144240	0.0317	0.0107816001
ss61516799	*rs41618799*	5	97.880361	0.4427	1.1032	0.293919	0.0200	0.0098824065
ss61472626		5	97.927246	0.8095	0.5414	0.462105	0.0171	0.0052882228
ss61518892	*rs41618642*	5	98.048094	0.1848	1.3429	0.246912	0.0272	0.0082011953
ss86285292		5	98.082173	0.6769	0.3174	0.573345	0.0111	0.0048691063
ss86341747		5	98.192386	0.3818	0.0960	0.756735	0.0059	0.0027671210
ss61524715	*rs41623244*	5	98.260277	0.1734	0.5562	0.456050	0.0180	0.0051541961
ss61475289	*rs41573769*	5	98.351611	0.2314	0.3207	0.571350	0.0121	0.0043010997
ss117967558		5	98.414334	0.6769	2.1168	0.146138	0.0276	0.0120588382
ss61475602	*rs41575674*	5	98.456164	0.4477	0.7604	0.383518	0.0166	0.0082133005
	rs29019412	5	98.508234	0.1375	0.0113	0.915217	0.0028	0.0006736044
ss117963794		5	98.531974	0.6232	1.7920	0.181117	0.0255	0.0119698295
ss117967834		5	98.725146	0.2908	1.8810	0.170662	0.0279	0.0115261257
ss61525837	*rs41623138*	5	98.950819	0.2328	0.0092	0.923739	0.0021	0.0007496491
ss61476180	*rs41575243*	5	99.041668	0.3911	0.1624	0.687046	0.0078	0.0037219477
ss38322961	*rs29014060*	5	99.145392	0.5330	0.3257	0.568381	0.0104	0.0051807773
ss86309248		5	99.192441	0.6039	1.1825	0.277225	0.0207	0.0098871261
ss61477737	*rs41579566*	5	99.266934	0.5523	0.6497	0.420513	0.0150	0.0074027714
ss117967861		5	99.378352	0.7579	0.6796	0.409994	0.0178	0.0065193243
ss86292092		5	99.564582	0.7299	0.2145	0.643372	0.0096	0.0037797413
ss61481328		5	99.617785	0.6354	0.2272	0.633791	0.0096	0.0044448975
ss86335855		5	99.656228	0.6963	0.6960	0.404419	0.0167	0.0070455871
ss61490918	*rs41591921*	5	100.149531	0.6246	0.0436	0.834687	0.0040	0.0018775376
ss61504061	*rs41602750*	5	100.301746	0.4606	0.7985	0.371850	0.0167	0.0082737263
ss86339297		5	100.45876	0.3138	1.6851	0.194681	0.0257	0.0110527651
ss61526128	*rs41624919*	5	100.659318	0.8861	0.1456	0.702879	0.0110	0.0022230932

ss86340767		5	100.71407	0.1898	0.0931	0.760350	0.0073	0.0022370311
ss86315998		5	100.826449	0.4549	1.4495	0.229010	0.0216	0.0107028372
ss61490921	*rs41591924*	5	100.942703	0.4964	0.2365	0.626931	0.0091	0.0045542567
ss61469050		5	100.985379	0.2314	2.0728	0.150394	0.0315	0.0111982397
ss61556353	*rs41653559*	5	101.015512	0.2135	0.1107	0.739430	0.0075	0.0025288956
ss61556352	*rs41653558*	5	101.042396	0.7837	0.0087	0.925634	0.0021	0.0007156962
	rs29016908	5	101.090418	0.8510	0.0751	0.784166	0.0070	0.0017860418
ss86297225		5	101.219012	0.1017	5.1565	0.023464	0.0681	0.0124379306
ss61514468	*rs41613034*	5	101.300392	0.2779	0.7427	0.389101	0.0173	0.0069411045
	rs29018542	5	101.351329	0.7013	0.1624	0.687115	0.0081	0.0033994198
ss86332177		5	101.615264	0.4255	2.3659	0.124467	0.0282	0.0138061080
ss46526426	*rs41256890*	5	101.979581	0.2865	0.9517	0.329632	0.0198	0.0080778806
ss86316560		5	102.090888	0.2185	0.4461	0.504419	0.0149	0.0051019211
ss117963810		5	102.164053	0.1146	2.5378	0.111606	0.0454	0.0092113484
ss117963817		5	102.184667	0.1146	2.5378	0.111606	0.0454	0.0092113484
ss86339519		5	102.261488	0.3825	0.7591	0.383900	0.0165	0.0077933613
ss117967888		5	102.836546	0.8474	0.9827	0.321888	0.0257	0.0066541859
ss86310527		5	102.893359	0.0924	0.1236	0.725321	0.0108	0.0018041990
ss86283983		5	102.991053	0.7149	2.6521	0.103864	0.0332	0.0135427649
ss117967917		5	103.047993	0.2156	4.8854	0.027409	0.0494	0.0166935979
ss86334511		5	103.177649	0.3395	0.1164	0.733046	0.0068	0.0030484832
ss86287923		5	103.218934	0.3123	0.2044	0.651370	0.0092	0.0039392406
ss86337865		5	103.263501	0.7328	3.2999	0.069713	0.0370	0.0145033261
ss86337356		5	103.296591	0.5616	0.7232	0.395387	0.0165	0.0081085243
ss86290821		5	103.31924	0.3360	2.4308	0.119425	0.0299	0.0133408234
ss86322686		5	103.348205	0.2593	0.4493	0.502870	0.0142	0.0054727132
ss86291173		5	103.492378	0.6741	0.1695	0.680727	0.0082	0.0035906396
ss86306692		5	103.550723	0.6948	1.4724	0.225378	0.0238	0.0101016222
ss61504062	*rs41602751*	5	103.598356	0.1734	0.3208	0.571291	0.0137	0.0039137088
ss86327040		5	103.709354	0.1705	0.7439	0.388720	0.0209	0.0059003336
ss86322982		5	103.793175	0.7307	1.9457	0.163501	0.0295	0.0115956545
ss86340696		5	103.967264	0.6913	0.0040	0.949834	0.0012	0.0005290584
ss86307990		5	104.022454	0.5795	0.2945	0.587521	0.0101	0.0049253279
ss86305408		5	104.059941	0.4341	0.6558	0.418333	0.0156	0.0076701489
ss86326412		5	104.081649	0.5136	0.2074	0.648983	0.0086	0.0042852500
ss86335964		5	104.121523	0.1834	3.9710	0.046681	0.0479	0.0143505455
ss86282403		5	104.184797	0.6232	2.4172	0.120465	0.0301	0.0141151092
	rs29009713	5	104.421186	0.7307	0.7229	0.395497	0.0174	0.0068679501
ss86336071		5	104.455532	0.7092	1.0918	0.296434	0.0219	0.0090250038
ss86331310		5	104.477431	0.1125	2.7006	0.100762	0.0477	0.0095198798
ss86303062		5	104.498287	0.6719	0.2398	0.624497	0.0097	0.0042965024
ss86290204		5	104.60143	0.1074	4.1598	0.041772	0.0605	0.0115959411
ss61490953	*rs41592954*	5	104.810755	0.6017	0.9970	0.318381	0.0188	0.0090040485
ss86336982		5	105.049078	0.4413	1.7322	0.188568	0.0257	0.0126709748
ss61465988	*rs41565451*	5	105.238435	0.4054	3.7702	0.052576	0.0368	0.0177339791
ss61519358		5	105.350649	0.4649	3.9418	0.047494	0.0372	0.0184870363
ss86318918		5	105.389166	0.2894	2.3860	0.122880	0.0318	0.0130721511

ss38326913	*rs29015215*	5	105.469665	0.6232	2.8332	0.092781	0.0314	0.0147592888
ss86333258		5	105.561501	0.0989	6.0519	0.014133	0.0754	0.0134273380
ss86336649		5	105.632311	0.7593	0.1244	0.724373	0.0079	0.0028876234
ss61519349	*rs41613602*	5	105.666729	0.4907	0.2870	0.592304	0.0098	0.0048938915
ss86273543		5	105.802563	0.1676	3.6644	0.055997	0.0473	0.0131933301
ss61473714	*rs41573130*	5	105.874102	0.2658	7.2079	0.007431	0.0550	0.0214798231
ss86333064		5	105.907487	0.3460	8.6345	0.003408	0.0575	0.0260402813
ss61519365	*rs41613618*	5	105.928882	0.4792	0.0671	0.795674	0.0047	0.0023598621
ss61519351	*rs41613604*	5	105.958155	0.6934	0.2242	0.636019	0.0095	0.0040277684
ss86337082		5	105.981907	0.4140	2.1284	0.145042	0.0273	0.0132287344
ss61469544	*rs43771070*	5	106.180833	0.2142	0.9379	0.333158	0.0225	0.0075570926
ss117967829		5	106.262879	0.5501	1.7509	0.186196	0.0240	0.0118781937
ss61472858	*rs41574577*	5	106.299196	0.7235	0.3245	0.569097	0.0122	0.0048746193
ss86276984		5	106.338732	0.6927	5.4010	0.020413	0.0471	0.0200565679
ss86312052		5	106.451095	0.6454	1.0990	0.294856	0.0199	0.0091068017
ss61495078	*rs41593661*	5	106.492787	0.3653	0.0013	0.971220	0.0007	0.0003304157
ss117963820		5	106.919475	0.1970	9.5126	0.002121	0.0716	0.0226493758
ss61490946	*rs41592947*	5	107.020028	0.2428	6.0667	0.014017	0.0541	0.0198932025
ss117963826		5	107.086995	0.8288	0.0026	0.959306	0.0013	0.0003592503
ss86310575		5	107.2557	0.8754	3.5463	0.060096	0.0527	0.0115086992
ss117962450		5	107.286368	0.1676	0.7330	0.392223	0.0215	0.0060112313
ss61514536	*rs41613097*	5	107.331704	0.7636	3.8658	0.049676	0.0419	0.0151329981
ss61556441	*rs41654447*	5	107.367507	0.1583	0.0046	0.945969	0.0017	0.0004554170
ss86339815		5	107.428034	0.4542	3.4007	0.065594	0.0345	0.0171046550
ss86337618		5	107.4753	0.6404	0.7505	0.386625	0.0170	0.0078086710
ss86328931		5	108.087763	0.2077	0.3204	0.571540	0.0133	0.0043754086
ss61529216	*rs41633024*	5	108.117321	0.3431	0.5490	0.458973	0.0144	0.0065083533
ss61556468	*rs41654473*	5	108.214401	0.2758	0.5126	0.474252	0.0147	0.0058868153
ss117962464		5	108.253675	0.4728	2.8753	0.090396	0.0319	0.0159167832
ss117962461		5	108.288992	0.9413	0.9682	0.325472	0.0392	0.0043333792
ss86297731		5	108.339147	0.4850	6.5720	0.010569	0.0485	0.0242111822
ss86338028		5	108.461316	0.5143	7.1043	0.007868	0.0498	0.0248835865
ss86311599		5	108.586541	0.7170	0.0144	0.904621	0.0025	0.0010183309
ss86306490		5	108.678413	0.2729	0.1008	0.750949	0.0066	0.0026319510
ss117967897		5	108.768987	0.3403	0.1110	0.739122	0.0066	0.0029600238
ss86287761		5	108.835956	0.1963	0.9110	0.340184	0.0218	0.0068654874
ss61524649	*rs41622179*	5	108.904534	0.5143	0.3984	0.528136	0.0112	0.0056123238
ss86324894		5	108.980835	0.4986	0.2204	0.638883	0.0083	0.0041747455
ss61524638	*rs41622168*	5	109.01501	0.4019	5.2692	0.022002	0.0423	0.0203577507
ss86321335		5	109.104304	0.8438	0.5386	0.463252	0.0189	0.0049734118
ss61475578		5	109.182393	0.6168	4.2137	0.040473	0.0388	0.0183488214
ss61524633	*rs41622163*	5	109.235853	0.7428	0.1714	0.678975	0.0088	0.0033647062
ss61523631	*rs41622294*	5	109.470973	0.4771	2.3285	0.127477	0.0289	0.0144343373
ss86304876		5	110.110357	0.1010	10.8270	0.001051	0.0985	0.0178842535
ss61494712	*rs41589401*	5	110.191418	0.8453	0.0104	0.918661	0.0025	0.0006471637
ss86341520		5	110.286334	0.4284	1.3986	0.237362	0.0222	0.0108857133
ss86281847		5	110.342531	0.5888	3.5958	0.058338	0.0354	0.0171174765

ss86327597		5	110.417771	0.5523	0.6637	0.415543	0.0150	0.0073995403
ss86334372		5	110.828928	0.4928	3.7609	0.052868	0.0354	0.0177094849
ss86340200		5	110.954315	0.3309	3.2608	0.071387	0.0349	0.0154598309
ss65359848	*rs43448718*	5	110.984107	0.1805	2.1989	0.138558	0.0353	0.0104347059
ss117963845		5	111.016741	0.1870	3.6634	0.056029	0.0448	0.0136325859
ss86298524		5	111.070118	0.8947	0.8467	0.357813	0.0269	0.0050719078
ss117963840		5	111.141609	0.4219	0.0027	0.958860	0.0010	0.0004649463
ss86329780		5	111.196613	0.1913	15.3093	0.000100	0.0901	0.0278645440
ss117967774		5	111.274583	0.4133	2.3024	0.129629	0.0279	0.0135506380
ss86299228		5	111.31518	0.7292	1.7042	0.192170	0.0271	0.0106878920
ss86274970		5	111.370383	0.4527	2.6213	0.105892	0.0296	0.0146672196
ss86301771		5	111.443282	0.9019	8.7228	0.003248	0.0905	0.0160172083
ss61497662	*rs43703389*	5	111.514959	0.3811	7.7254	0.005592	0.0508	0.0239750482
ss86297838		5	111.552297	0.6368	6.1215	0.013592	0.0456	0.0210991156
ss61470671	*rs41572347*	5	111.574914	0.5580	10.6174	0.001175	0.0578	0.0285028931
ss86284566		5	111.597559	0.4219	10.6051	0.001182	0.0575	0.0280648216
ss61490994	*rs41592994*	5	111.659042	0.5702	10.7537	0.001093	0.0579	0.0283702746
ss61556514	*rs41654519*	5	111.744502	0.2378	3.2851	0.070342	0.0402	0.0145852176
ss86295342		5	111.764611	0.6096	14.1445	0.000184	0.0688	0.0327448594
ss86330796		5	111.809977	0.5817	18.5773	0.000019	0.0768	0.0373596557
ss61523635	*rs41622298*	5	111.840713	0.5788	16.2695	0.000061	0.0722	0.0351959831
ss86296272		5	111.861216	0.4828	15.9278	0.000073	0.0704	0.0351662556
ss86337464		5	111.897898	0.3159	9.0137	0.002776	0.0580	0.0250640746
ss86324015		5	111.923342	0.6354	8.1648	0.004399	0.0548	0.0253969799
ss61556550	*rs41655454*	5	111.966641	0.3510	7.6445	0.005845	0.0532	0.0242417127
ss86297035		5	112.040374	0.6920	4.4260	0.035754	0.0423	0.0180251351
ss61556549	*rs41655453*	5	112.115684	0.6991	5.1136	0.024047	0.0457	0.0192405412
ss86327989		5	112.138481	0.4205	3.0330	0.082031	0.0331	0.0161390943
ss86313671		5	112.169783	0.6010	3.7447	0.053380	0.0368	0.0176689161
ss61556548	*rs41655452*	5	112.218625	0.0587	5.7423	0.016824	0.0938	0.0103750594
ss61556546	*rs41655450*	5	112.243198	0.9413	7.9811	0.004862	0.1104	0.0122110405
ss46526970	*rs41257416*	5	112.340739	0.1648	0.2006	0.654358	0.0114	0.0031265598
ss86275942		5	112.442505	0.3911	10.5653	0.001208	0.0610	0.0290643364
ss86339913		5	112.532633	0.4427	11.6652	0.000674	0.0631	0.0311514492
ss86285343		5	112.55433	0.8825	1.6551	0.198693	0.0364	0.0075570728
ss61556523	*rs41654528*	5	112.606498	0.1039	0.7833	0.376428	0.0262	0.0048841669
ss86301307		5	112.645234	0.1182	3.2307	0.072702	0.0514	0.0107170362
ss86311266		5	112.671019	0.8840	1.4026	0.236689	0.0344	0.0070576264
ss86329556		5	112.749461	0.6612	2.9254	0.087642	0.0323	0.0144694004
ss61556531	*rs41655435*	5	112.869616	0.0544	4.3925	0.036458	0.0846	0.0087072242
ss86340005		5	112.912611	0.4277	9.6392	0.001982	0.0563	0.0275846776
ss86340936		5	112.942478	0.4054	1.0902	0.296793	0.0194	0.0093688507
ss86286536		5	113.025544	0.0430	3.0760	0.079894	0.0769	0.0063229338
ss86303550		5	113.064552	0.5272	4.5095	0.034060	0.0395	0.0197091497
ss86275653		5	113.097085	0.8238	1.4827	0.223762	0.0289	0.0084043988
ss86320736		5	113.117946	0.6812	9.6333	0.001988	0.0603	0.0261821960
ss86338997		5	113.197776	0.4169	5.4170	0.020228	0.0436	0.0212046783

ss86340632		5	113.310281	0.4778	7.8883	0.005115	0.0516	0.0257511866
ss86289800		5	113.331587	0.5458	4.3284	0.037848	0.0382	0.0189560117
ss86304708		5	113.357543	0.5007	5.5045	0.019247	0.0432	0.0216162709
ss86289395		5	113.445839	0.3933	5.1178	0.023990	0.0428	0.0204045719
ss86337833		5	113.485095	0.4334	1.5318	0.216259	0.0227	0.0111722721
ss86309245		5	113.570138	0.5731	11.4216	0.000766	0.0623	0.0304789757
ss86287303		5	113.599961	0.4993	0.7856	0.375739	0.0166	0.0082993984
ss86336132		5	113.622404	0.4348	1.1073	0.293031	0.0197	0.0097065493
ss86291432		5	113.649925	0.4348	1.1073	0.293031	0.0197	0.0097065493
ss86306612		5	113.68201	0.2400	0.9779	0.323069	0.0217	0.0079111332
ss86336232		5	113.709318	0.6726	0.5508	0.458246	0.0143	0.0063078104
	rs29018274	5	113.734416	0.0881	1.0932	0.296116	0.0343	0.0055193844
ss86329116		5	113.775569	0.3983	9.8280	0.001791	0.0601	0.0287926913
ss86297919		5	113.797751	0.4986	1.4945	0.221939	0.0226	0.0112765390
ss61510520	*rs41605147*	5	113.822516	0.5458	2.1551	0.142548	0.0269	0.0133161217
ss86310087		5	113.860011	0.6311	7.2677	0.007190	0.0524	0.0243812343
ss86281480		5	113.922226	0.6332	7.6737	0.005753	0.0533	0.0247723128
ss86303626		5	113.955877	0.6583	12.6574	0.000399	0.0697	0.0313730279
ss86315359		5	114.058059	0.1798	10.9004	0.001011	0.0784	0.0231235532
ss86287696		5	114.080455	0.4506	0.4078	0.523300	0.0118	0.0058408882
ss86289133		5	114.110806	0.2887	11.5031	0.000734	0.0694	0.0284843206
	rs29010247	5	114.177327	0.3854	0.3283	0.566873	0.0108	0.0051383979
ss86330222		5	114.197493	0.6153	0.3960	0.529347	0.0119	0.0056352702
ss86337824		5	114.288699	0.6884	4.9271	0.026761	0.0435	0.0186734960
ss86337155		5	114.329444	0.6762	8.9086	0.002938	0.0586	0.0256641656
ss86316845		5	114.35181	0.2722	4.7210	0.030133	0.0450	0.0178233424
ss86304808		5	114.390497	0.5287	0.6085	0.435624	0.0142	0.0070659103
ss86338695		5	114.582058	0.4964	12.8342	0.000364	0.0672	0.0335916586
ss46526768	*rs41257219*	5	114.618072	0.5616	13.6358	0.000239	0.0703	0.0346167957
ss86288443		5	114.710763	0.6340	3.2511	0.071808	0.0352	0.0163471461
	rs29022872	5	114.748595	0.8883	5.1269	0.023865	0.0669	0.0132748692
ss117967854		5	114.771508	0.1103	4.5694	0.032896	0.0634	0.0124486926
ss61556574	*rs41655478*	5	114.799036	0.1010	3.6056	0.057999	0.0580	0.0105336362
	rs29018564	5	114.827202	0.1404	0.0409	0.839874	0.0054	0.0012998170
ss86281305		5	114.860779	0.1461	0.0145	0.904147	0.0031	0.0007831435
ss61556572	*rs41655476*	5	114.883742	0.8947	3.7225	0.054091	0.0580	0.0109253828
ss38327301	*rs29019411*	5	114.960868	0.5301	0.0720	0.788554	0.0050	0.0024875036
ss86332091		5	114.989397	0.3861	2.1929	0.139100	0.0289	0.0136858766
ss61556569	*rs41655473*	5	115.009425	0.9477	0.6439	0.422583	0.0336	0.0033294988
ss86294575		5	115.047898	0.5165	2.9617	0.085703	0.0328	0.0164064054
ss86302181		5	115.107086	0.5917	0.0874	0.767584	0.0056	0.0027134715
ss86321708		5	115.131443	0.3560	3.8825	0.049187	0.0387	0.0177371341
ss86338663		5	115.156757	0.8532	0.0274	0.868694	0.0044	0.0010999155
ss86336905		5	115.221654	0.1082	6.4207	0.011498	0.0738	0.0142291154
ss86309450		5	115.288129	0.7256	0.0032	0.954655	0.0012	0.0004629922
ss86299593		5	115.310537	0.7056	0.6615	0.416326	0.0162	0.0067152597
ss86284088		5	115.473165	0.5917	0.6826	0.408983	0.0163	0.0078631931

ss86337326		5	115.509871	0.2586	0.1753	0.675583	0.0091	0.0034757997
ss86285083		5	115.570039	0.1791	6.1967	0.013031	0.0577	0.0169632854
ss86292353		5	115.595172	0.8181	5.3430	0.021097	0.0535	0.0159187188
ss86339132		5	115.620132	0.4413	0.9625	0.326897	0.0189	0.0093190517
ss86333568		5	115.659659	0.7650	4.7830	0.029075	0.0464	0.0166729563
ss86300341		5	115.70106	0.1648	0.9487	0.330382	0.0247	0.0068001522
ss86313785		5	115.721641	0.2056	0.0145	0.904322	0.0029	0.0009349039
ss86303038		5	115.74523	0.4026	7.9111	0.005052	0.0540	0.0259803462
ss86299439		5	115.76669	0.2951	0.0011	0.973042	0.0007	0.0002827596
ss86316332		5	115.808996	0.2564	1.8374	0.175699	0.0285	0.0108875494
ss86315410		5	115.919741	0.6934	2.6216	0.105872	0.0321	0.0136295945
ss117963811		5	116.017255	0.4176	0.0820	0.774731	0.0054	0.0026340773
ss86293796		5	116.079099	0.2643	1.9999	0.157754	0.0302	0.0117402472
ss46526219	*rs41255358*	5	116.152846	0.1870	1.9756	0.160302	0.0334	0.0101585299
ss86314626		5	116.173851	0.7658	1.9898	0.158810	0.0303	0.0108557382
ss86334941		5	116.19806	0.7092	1.2272	0.268328	0.0221	0.0091199690
ss86274830		5	116.338745	0.2629	2.3876	0.122755	0.0322	0.0124937039
ss86299524		5	116.362061	0.2507	1.7873	0.181692	0.0282	0.0105892991
ss86327034		5	116.431072	0.3797	6.0629	0.014047	0.0461	0.0216985641
ss61469059	*rs41566970*	5	116.453083	0.2149	0.7184	0.396947	0.0187	0.0062945811
ss86304344		5	116.561404	0.6039	2.1575	0.142327	0.0285	0.0136546056
ss86305611		5	116.682019	0.4728	0.1601	0.689222	0.0076	0.0037704895
ss86335995		5	116.713739	0.4076	0.9574	0.328175	0.0185	0.0089513412
ss86340938		5	116.80328	0.3009	0.5558	0.456197	0.0151	0.0063421007
ss86290914		5	116.857055	0.6913	0.4673	0.494462	0.0139	0.0059246925
ss86305325		5	116.877481	0.3016	0.6823	0.409091	0.0167	0.0070384720
ss86293471		5	116.911193	0.1855	0.9447	0.331406	0.0231	0.0069958265
ss86331548		5	116.956592	0.3897	1.8151	0.178334	0.0258	0.0122950916
ss86322731		5	116.992453	0.2414	0.2300	0.631677	0.0106	0.0038932468
ss86330340		5	117.041807	0.6010	3.2461	0.072028	0.0346	0.0165720872
ss86298205		5	117.065472	0.2958	0.2781	0.598107	0.0108	0.0044795713
ss61491021	*rs41593021*	5	117.126364	0.3553	0.4462	0.504356	0.0128	0.0058428258
ss86288488		5	117.167197	0.5996	1.1613	0.281562	0.0207	0.0099210971
ss86286806		5	117.235579	0.1017	7.5531	0.006146	0.0842	0.0153866079
ss86293923		5	117.268231	0.1032	8.0188	0.004763	0.0857	0.0158514723
ss86341486		5	117.376967	0.1074	0.1440	0.704459	0.0120	0.0023104807
ss86301610		5	117.428896	0.2149	1.0631	0.302874	0.0228	0.0076791467
ss86316410		5	117.450407	0.4628	0.1410	0.707388	0.0070	0.0035027947
ss61556622	*rs41655526*	5	117.473896	0.5358	0.0620	0.803446	0.0047	0.0023226425
ss86308857		5	117.544276	0.4885	0.0011	0.973005	0.0006	0.0003140363
ss86297501		5	117.6495	0.6139	4.8867	0.027389	0.0405	0.0191959228
ss86290920		5	117.865856	0.2178	0.5472	0.459728	0.0160	0.0054513810
ss86308772		5	117.905973	0.1877	0.0785	0.779395	0.0067	0.0020429334
ss86286524		5	117.952546	0.3617	1.3141	0.252056	0.0221	0.0102142328
ss86298834		5	118.037169	0.5115	2.7160	0.099800	0.0302	0.0150828002
ss86317208		5	118.077443	0.3675	1.5986	0.206519	0.0234	0.0108919766
ss117967904		5	118.137031	0.4993	2.6883	0.101540	0.0301	0.0150389692

ss86311810		5	118.185122	0.3223	0.0710	0.790013	0.0052	0.0022500315
ss86321951		5	118.219331	0.6784	5.5709	0.018537	0.0477	0.0208272274
ss86334574		5	118.266541	0.7034	1.0360	0.309106	0.0205	0.0085722749
ss86281157		5	118.291279	0.6010	0.0451	0.831807	0.0039	0.0018574928
ss86341536		5	118.321023	0.0989	4.0384	0.044862	0.0623	0.0111042710
ss86310282		5	118.348266	0.1655	1.2813	0.258042	0.0279	0.0077138202
ss86291464		5	118.424541	0.2493	2.9721	0.085157	0.0358	0.0133920348
ss86319957		5	118.493391	0.3804	0.7194	0.396626	0.0160	0.0075576308
ss86338632		5	118.518528	0.6683	2.2070	0.137835	0.0284	0.0125964704
ss86332836		5	118.545419	0.5852	0.5961	0.440341	0.0142	0.0069013628
ss86292544		5	118.566442	0.8417	1.1042	0.293700	0.0257	0.0068592336
	rs29020811	5	118.645508	0.5981	0.4283	0.513062	0.0125	0.0060001853
	rs29010136	5	118.682288	0.6268	0.6143	0.433447	0.0147	0.0068993909
ss86335570		5	118.720236	0.7586	1.1207	0.290127	0.0245	0.0089779470
ss86310081		5	118.756039	0.0874	4.4421	0.035420	0.0687	0.0109510439
ss86289235		5	118.886721	0.0831	0.0015	0.969285	0.0013	0.0001990039
ss61491054	*rs41593854*	5	118.958298	0.4670	2.0980	0.147938	0.0278	0.0138268897
ss117967607		5	119.004574	0.5330	1.8761	0.171216	0.0262	0.0130372660
ss86282893		5	119.105634	0.2923	0.8842	0.347367	0.0195	0.0080524945
ss86340700		5	119.128022	0.9069	1.7435	0.187124	0.0425	0.0071789274
ss86276041		5	119.154972	0.5745	0.6692	0.413601	0.0155	0.0075750220
ss86330774		5	119.223666	0.5838	0.3999	0.527368	0.0119	0.0058045935
ss61556640		5	119.280548	0.5609	2.0807	0.149623	0.0270	0.0132869977
ss38335819	*rs29018725*	5	119.31985	0.4928	4.5030	0.034188	0.0391	0.0195270257
ss61556646	*rs41656249*	5	119.362944	0.1956	1.7295	0.188903	0.0299	0.0093943193
ss86338502		5	119.41084	0.8001	2.0186	0.155826	0.0319	0.0102028542
ss86304990		5	119.514667	0.8360	1.1851	0.276688	0.0259	0.0070921079
ss86309374		5	119.547549	0.5530	0.3195	0.572080	0.0100	0.0049585374
ss86310306		5	119.580284	0.5229	0.9991	0.317878	0.0179	0.0089185714
ss86288140		5	119.674608	0.8617	1.0173	0.313521	0.0275	0.0065424832
ss61491068	*rs41593868*	5	119.708891	0.6884	0.0171	0.895974	0.0027	0.0011416659
ss61499194	*rs43709090*	5	119.734178	0.2937	0.1673	0.682664	0.0083	0.0034461323
ss86315201		5	119.953013	0.2579	1.3558	0.244671	0.0254	0.0097204237
ss86309820		5	120.038095	0.3324	2.3618	0.124789	0.0309	0.0137354890
ss117967579		5	120.067834	0.6719	1.8505	0.174164	0.0277	0.0122044925
ss86301569		5	120.098782	0.3317	2.7690	0.096558	0.0336	0.0148927605
ss86326933		5	120.128437	0.3403	1.2560	0.262791	0.0225	0.0101001393
ss46526971	*rs41257417*	5	120.200556	0.2686	12.6580	0.000399	0.0769	0.0302261714
ss86336810		5	120.238291	0.7328	11.9255	0.000587	0.0752	0.0294538147
ss86304727		5	120.262095	0.2994	2.1247	0.145395	0.0299	0.0125352782
ss86323662		5	120.362864	0.7636	0.8546	0.355587	0.0208	0.0074919430
ss86341226		5	120.385101	0.3696	3.1922	0.074424	0.0340	0.0158568830
ss86318440		5	120.440619	0.7665	0.6922	0.405687	0.0187	0.0066929356
ss86294499		5	120.466874	0.3696	3.1922	0.074424	0.0340	0.0158568830
ss86291317		5	120.498492	0.3438	4.6387	0.031602	0.0423	0.0190838053
ss86334338		5	120.538804	0.4040	1.2354	0.266740	0.0221	0.0106431446
ss86339065		5	120.595372	0.8345	8.9986	0.002798	0.0747	0.0206432985

ss86335698		5	120.63386	0.1848	4.5845	0.032610	0.0516	0.0155390386
	rs29026125	5	120.786285	0.6905	0.1421	0.706341	0.0074	0.0031438002
ss86318540		5	120.861966	0.5666	1.1295	0.288260	0.0190	0.0093271445
ss61556664	*rs41656267*	5	120.911372	0.5681	1.4247	0.233031	0.0213	0.0104716924
ss86317632		5	120.946294	0.4319	1.4247	0.233031	0.0213	0.0104716924
ss61528683	*rs41627316*	5	120.980457	0.5738	1.1051	0.293521	0.0189	0.0092419514
ss86323047		5	121.007469	0.4413	1.0477	0.306398	0.0182	0.0089503487
ss86301980		5	121.050512	0.3768	1.0405	0.308053	0.0202	0.0094737897
ss86302720		5	121.102317	0.2034	0.0008	0.977412	0.0007	0.0002169762
ss86295932		5	121.129351	0.4312	0.0073	0.931885	0.0017	0.0008115093
ss86283412		5	121.184153	0.5809	2.7734	0.096292	0.0322	0.0156823947
ss86291377		5	121.206637	0.0903	0.0016	0.968182	0.0013	0.0002177483
ss86274909		5	121.235537	0.2199	3.7349	0.053693	0.0434	0.0148898069
ss86289115		5	121.384505	0.8689	0.1640	0.685663	0.0113	0.0025825141
ss86341300		5	121.42469	0.1268	0.0833	0.773017	0.0079	0.0017436785
ss86286661		5	121.467972	0.5466	4.1076	0.043071	0.0368	0.0182285923
ss86327029		5	121.50258	0.3295	1.4031	0.236616	0.0232	0.0102488897
ss86274506		5	121.531019	0.2400	2.5185	0.112971	0.0330	0.0120248040
ss86331739		5	121.557022	0.5129	0.0512	0.821124	0.0043	0.0021290025
ss86329598		5	121.596678	0.2593	0.0513	0.820874	0.0047	0.0018136824
ss61490030	*rs41589541*	5	121.661106	0.5115	0.2877	0.591849	0.0097	0.0048316849
ss86322573		5	121.788861	0.3188	0.3191	0.572307	0.0109	0.0047478662
ss86332851		5	122.038743	0.2672	0.0275	0.868399	0.0035	0.0013827907
ss86302568		5	122.080981	0.1232	0.0011	0.973288	0.0010	0.0002063574
ss86309211		5	122.14899	0.1433	2.9827	0.084602	0.0460	0.0113022517
ss86313980		5	122.241829	0.1855	0.3141	0.575373	0.0134	0.0040624660
ss86283603		5	122.281891	0.0580	0.7151	0.398063	0.0330	0.0036023017
ss61491075	*rs41593875*	5	122.325271	0.8789	0.0484	0.825890	0.0060	0.0012834377
ss86319828		5	122.371904	0.5874	0.0005	0.981807	0.0004	0.0002097296
ss61556703	*rs41656305*	5	122.410166	0.3918	0.0193	0.889650	0.0027	0.0012771918
ss86283344		5	122.453525	0.1024	9.3076	0.002369	0.0939	0.0172718533
ss86298122		5	122.493052	0.2142	1.0402	0.308139	0.0229	0.0077138717
ss86296291		5	122.528414	0.2521	0.2929	0.588540	0.0119	0.0044776852
ss86329357		5	122.644078	0.6060	2.3304	0.127327	0.0287	0.0136854940
ss86313514		5	122.66756	0.2837	2.4138	0.120724	0.0317	0.0128997536
ss86299671		5	122.708405	0.3080	0.5838	0.445092	0.0156	0.0066516229
ss86336111		5	122.735171	0.8159	0.1004	0.751476	0.0076	0.0022912060
ss86296546		5	122.778435	0.1433	2.5092	0.113640	0.0422	0.0103664200
	rs29018295	5	122.873689	0.4219	0.1677	0.682285	0.0075	0.0036807760
ss86282726		5	122.89661	0.4219	0.1677	0.682285	0.0075	0.0036807760
ss86276347		5	122.932455	0.7751	2.7998	0.094725	0.0381	0.0132818679
ss86328341		5	122.952523	0.5645	2.0499	0.152667	0.0262	0.0129001043
ss86299992		5	122.985897	0.7385	0.2292	0.632245	0.0100	0.0038677958
ss61556755	*rs41657057*	5	123.12711	0.1390	0.3183	0.572827	0.0148	0.0035443271
ss86340118		5	123.156376	0.1325	0.1636	0.685980	0.0113	0.0025988373
ss86323360		5	123.214644	0.2471	9.5479	0.002082	0.0663	0.0246777928
ss86313314		5	123.290119	0.5272	2.4984	0.114417	0.0279	0.0139298902

ss86326767		5	123.321098	0.6948	1.1606	0.281724	0.0212	0.0089942856
ss86288513		5	123.3472	0.6726	3.3675	0.066922	0.0351	0.0154444820
ss86319448		5	123.38118	0.2185	1.5515	0.213337	0.0288	0.0098362814
ss86310669		5	123.422821	0.1461	7.1896	0.007506	0.0693	0.0172855736
ss86327983		5	123.541152	0.1648	10.0281	0.001609	0.0793	0.0218323352
ss86303562		5	123.572177	0.1913	2.2181	0.136857	0.0342	0.0105790879
ss86335641		5	123.598295	0.2500	2.3125	0.128792	0.0326	0.0122170467
ss86297651		5	123.626393	0.4534	0.0331	0.855652	0.0035	0.0017221370
ss86287166		5	123.654038	0.6383	9.7904	0.001827	0.0600	0.0277223834
ss86305039		5	123.701613	0.5158	9.8962	0.001727	0.0571	0.0284972795
ss86274835		5	123.722293	0.7185	7.1932	0.007492	0.0552	0.0223194182
ss86339318		5	123.754208	0.3582	2.6550	0.103676	0.0308	0.0141474180
ss86336743		5	123.778611	0.2056	0.0155	0.901002	0.0028	0.0009270539
ss86274475		5	123.840785	0.4083	0.9939	0.319136	0.0187	0.0090484184
	rs29024200	5	123.879286	0.7428	6.1074	0.013700	0.0508	0.0194194311
ss86310809		5	123.934422	0.6397	0.0047	0.945643	0.0013	0.0005946147
ss86305301		5	123.961828	0.7371	1.0305	0.310399	0.0210	0.0081323485
ss86323989		5	123.99844	0.6375	17.6845	0.000029	0.0781	0.0360754566
ss86327450		5	124.040706	0.1884	0.0167	0.897086	0.0031	0.0009505470
ss86335968		5	124.084532	0.1841	0.0013	0.971342	0.0009	0.0002604438
ss86323511		5	124.123033	0.2056	0.0219	0.882334	0.0035	0.0011408132
ss86317298		5	124.145197	0.3897	12.2384	0.000498	0.0631	0.0299955000
ss86337943		5	124.169695	0.8689	0.0888	0.765798	0.0081	0.0018520219
ss86321375		5	124.191544	0.4448	9.1034	0.002644	0.0543	0.0268385127
ss86302919		5	124.298738	0.6125	6.1747	0.013193	0.0460	0.0218526626
ss86303477		5	124.342962	0.4735	1.5992	0.206444	0.0237	0.0118153280
ss86329869		5	124.36877	0.5623	1.4425	0.230140	0.0218	0.0107352638
ss86295112		5	124.419445	0.9241	0.1547	0.694220	0.0139	0.0019571030
ss86334597		5	124.644788	0.2450	1.7761	0.183071	0.0279	0.0103325143
ss117967845		5	124.682278	0.5580	5.8799	0.015568	0.0452	0.0222863303
ss61471007	*rs41566580*	5	124.715381	0.1383	1.5155	0.218723	0.0333	0.0079412297
ss86337688		5	124.736445	0.1905	0.0219	0.882331	0.0036	0.0010954135
ss86319187		5	124.78349	0.3489	1.3159	0.251723	0.0221	0.0100411634
ss61513646	*rs41614014*	5	124.807961	0.9054	4.8292	0.028310	0.0701	0.0120062201
ss86340811		5	124.831696	0.6662	0.3473	0.555810	0.0116	0.0051681630
ss86291445		5	124.871788	0.4706	0.0598	0.806884	0.0046	0.0022780956
ss86336547		5	124.894917	0.6039	2.3095	0.129039	0.0293	0.0140154355
ss86289083		5	124.92246	0.3918	0.1007	0.751144	0.0061	0.0028950920
ss86330959		5	124.955596	0.4377	1.7934	0.180953	0.0259	0.0127467764
ss61479940	*rs41583795*	5	124.980157	0.1182	0.0342	0.853285	0.0054	0.0011299864
ss61497454	*rs41596880*	5	125.036256	0.0989	6.0944	0.013800	0.0759	0.0135199270
ss86335663		5	125.058665	0.3825	0.0598	0.806949	0.0047	0.0022411648
	rs29015305	5	125.087083	0.0867	5.3022	0.021593	0.0774	0.0122612930
ss61512053	*rs41610063*	5	125.117272	0.0638	0.0016	0.968350	0.0015	0.0001826697
ss86296744		5	125.147296	0.8567	0.6501	0.420351	0.0214	0.0052470002
ss86313649		5	125.222959	0.4943	1.3516	0.245393	0.0214	0.0107048169
ss86295678		5	125.24434	0.0681	7.3617	0.006828	0.0990	0.0125570964

ss86323818		5	125.275404	0.6017	0.7050	0.401385	0.0162	0.0077442745
ss86339174		5	125.338219	0.3080	15.3564	0.000098	0.0769	0.0327799927
ss61570010	*rs41672057*	5	125.40589	0.2536	0.0362	0.849176	0.0040	0.0015244773
ss86301017		5	125.445916	0.4527	2.0676	0.150905	0.0268	0.0132955225
ss86319576		5	125.488364	0.6784	8.0770	0.004614	0.0555	0.0242060404
ss86335183		5	125.588038	0.7070	11.7829	0.000633	0.0683	0.0283006673
ss86316882		5	125.618716	0.7808	6.1250	0.013566	0.0557	0.0190523508
ss86336173		5	125.642871	0.0781	0.6015	0.438272	0.0266	0.0038346819
ss86330909		5	125.702331	0.7156	1.3425	0.246988	0.0241	0.0098194229
ss86304726		5	125.729597	0.8832	0.1530	0.695794	0.0113	0.0023220220
ss86326950		5	125.756132	0.6562	0.1649	0.684779	0.0077	0.0034839849
ss61567144	*rs41660664*	6	0.514959	0.4713	6.7790	0.009420	0.0476	0.0237230915
ss86317899		6	0.545274	0.5279	7.0954	0.007907	0.0487	0.0242985749
ss117967949		6	0.574156	0.5279	7.0954	0.007907	0.0487	0.0242985749
ss61566889	*rs41668402*	6	0.714622	0.5193	0.0090	0.924276	0.0018	0.0008765748
ss64813830	*rs42904886*	6	0.88389	0.0917	3.2149	0.073404	0.0585	0.0097433079
ss61522171	*rs41617665*	6	0.91816	0.7034	5.8175	0.016125	0.0479	0.0199860574
ss61512278	*rs41610886*	6	1.010997	0.6361	0.3382	0.561058	0.0111	0.0051390371
ss61475461	*rs41575036*	6	1.037224	0.6862	0.0225	0.880692	0.0029	0.0012559658
ss61515012	*rs41611475*	6	1.096576	0.3574	1.1642	0.280971	0.0212	0.0097387660
ss86305631		6	1.133117	0.6411	0.8917	0.345351	0.0185	0.0085241200
ss86325747		6	1.202245	0.6411	1.0067	0.316036	0.0196	0.0090284638
ss64319960	*rs42584307*	6	1.374013	0.8073	2.5191	0.112928	0.0377	0.0117348019
ss64762358	*rs42855375*	6	1.452846	0.7163	1.0922	0.296351	0.0215	0.0087313399
ss64762273	*rs42854690*	6	1.496085	0.2256	2.2301	0.135794	0.0331	0.0115651600
ss65363951	*rs43445730*	6	1.53242	0.5580	1.4881	0.222921	0.0222	0.0109358799
ss65363973	*rs43446552*	6	1.556121	0.5580	1.4881	0.222921	0.0222	0.0109358799
ss61473919	*rs41574428*	6	1.710775	0.4742	0.0003	0.986890	0.0003	0.0001514696
ss117963855		6	1.790979	0.4327	1.9268	0.165551	0.0259	0.0126926320
ss38327106	*rs29019218*	6	1.843722	0.4692	0.0407	0.840118	0.0037	0.0018568676
ss86294232		6	1.871737	0.2092	7.5783	0.006061	0.0624	0.0206392710
	rs29015797	6	1.923177	0.8574	1.1248	0.289257	0.0277	0.0067717404
ss64710001	*rs42802002*	6	1.953557	0.4026	0.0134	0.907803	0.0021	0.0010340079
ss61567238	*rs41661655*	6	1.987527	0.8274	0.0836	0.772628	0.0071	0.0020334444
ss64709981	*rs42801982*	6	2.017216	0.7070	1.0137	0.314370	0.0207	0.0085717972
ss64306348	*rs42579218*	6	2.242801	0.7328	0.9458	0.331141	0.0210	0.0082177028
ss61567235	*rs41661652*	6	2.469455	0.3166	5.4816	0.019499	0.0454	0.0196410732
	rs29025265	6	2.526657	0.3166	5.6111	0.018119	0.0456	0.0197540936
ss61491607	*rs41588994*	6	2.757585	0.6898	2.2485	0.134194	0.0309	0.0132137215
	rs29026861	6	2.839922	0.4069	1.1322	0.287684	0.0198	0.0095464625
ss61557519	*rs41653804*	6	2.888816	0.3725	0.0000	0.994894	0.0001	0.0000582706
ss65364103	*rs43447181*	6	2.931929	0.7443	5.3690	0.020787	0.0469	0.0178571485
ss86312887		6	2.956054	0.2958	2.9499	0.086327	0.0346	0.0143971631
ss65364032	*rs41256438*	6	2.994631	0.2808	6.2683	0.012520	0.0499	0.0201486192
	rs29014980	6	3.050801	0.4076	0.0035	0.952943	0.0011	0.0005299802
	rs29014449	6	3.215714	0.3367	3.4893	0.062188	0.0365	0.0162949242
ss61510705	*rs41606028*	6	3.350928	0.5372	4.7789	0.029143	0.0406	0.0201868215

ss65364695	*rs43445974*	6	3.372536	0.6089	2.9011	0.088967		0.0320	0.0152600487
ss61491868	*rs41591932*	6	3.398026	0.5158	4.4400	0.035463		0.0381	0.0190214574
ss86336248		6	3.51783	0.3825	6.5584	0.010649		0.0467	0.0220560005
ss86301481		6	3.53861	0.6103	1.4271	0.232639		0.0225	0.0107136704
ss86302599		6	3.560843	0.0544	0.4113	0.521547		0.0257	0.0026408316
ss46526887	*rs41257336*	6	3.5873	0.3983	1.0793	0.299226		0.0204	0.0097943380
ss117968002		6	3.626655	0.2113	3.4244	0.064664		0.0412	0.0137412338
ss61525130	*rs41626156*	6	3.655625	0.0881	0.0085	0.926443		0.0030	0.0004885404
ss64807764	*rs42900126*	6	3.684492	0.0817	0.0007	0.978800		0.0009	0.0001363197
ss61527395	*rs41623357*	6	3.722574	0.1182	0.1058	0.745035		0.0090	0.0018834438
ss64866694	*rs42961863*	6	3.7472	0.4735	1.5245	0.217363		0.0230	0.0114788144
ss86339143		6	3.851046	0.4971	2.3290	0.127439		0.0283	0.0141275406
ss86311515		6	4.017889	0.5852	1.5666	0.211120		0.0236	0.0114742274
ss86292801		6	4.091879	0.3059	0.2448	0.620898		0.0097	0.0040984170
ss63960032	*rs42404150*	6	4.128489	0.6096	2.6460	0.104265		0.0305	0.0145220821
ss63960184	*rs42404226*	6	4.153412	0.8904	0.0134	0.907784		0.0033	0.0006505705
ss63960340	*rs42405104*	6	4.190285	0.0967	4.1627	0.041700		0.0643	0.0112295073
ss86341276		6	4.217984	0.4076	0.4915	0.483499		0.0136	0.0065478624
ss86308024		6	4.243788	0.4470	2.0458	0.153078		0.0270	0.0133472864
ss86283431		6	4.433247	0.9355	0.0007	0.979475		0.0010	0.0001147944
ss65365293	*rs43452468*	6	4.456293	0.4878	8.7068	0.003277		0.0534	0.0266593297
ss65365311	*rs43452486*	6	4.494602	0.4140	1.8578	0.173318		0.0259	0.0125888446
ss65365351	*rs43452526*	6	4.530263	0.4241	1.1494	0.284044		0.0204	0.0099666595
ss65365485	*rs43454260*	6	4.594144	0.5602	1.0093	0.315408		0.0190	0.0093396000
ss65056249	*rs43144276*	6	4.683078	0.7335	0.0303	0.861815		0.0037	0.0014375995
ss64785208	*rs42879003*	6	4.716583	0.2235	0.1650	0.684762		0.0092	0.0031836108
ss86326470		6	4.74403	0.3345	3.4900	0.062161		0.0363	0.0161746670
ss64700798	*rs42798182*	6	4.792809	0.3023	1.2219	0.269365		0.0222	0.0093564444
ss64332963	*rs42584657*	6	4.923325	0.2471	0.4295	0.512448		0.0139	0.0051838525
ss86299324		6	5.065188	0.8517	3.2495	0.071879		0.0473	0.0119365958
ss86274628		6	5.186791	0.9126	0.2576	0.611952		0.0169	0.0026965863
ss65366583	*rs43446153*	6	6.093399	0.1103	0.0000	0.998614		0.0001	0.0000099132
ss64865010	*rs42962195*	6	6.133489	0.7170	2.2723	0.132160		0.0307	0.0124704925
ss86337335		6	6.181513	0.4355	3.6007	0.058170		0.0342	0.0168351512
ss64590874	*rs42715455*	6	6.694747	0.9090	0.1293	0.719217		0.0113	0.0018694631
ss64590500	*rs42713568*	6	6.725663	0.1060	0.1196	0.729620		0.0103	0.0019583597
ss86289877		6	6.785505	0.7650	0.0046	0.946117		0.0015	0.0005277070
ss117968507		6	6.84558	0.7543	0.0047	0.945637		0.0014	0.0005367175
ss65366903	*rs43448170*	6	6.874908	0.2471	0.0298	0.863038		0.0037	0.0013628091
ss86293314		6	6.904028	0.0681	0.3095	0.578193		0.0197	0.0024982691
ss86335118		6	6.995394	0.3453	6.9185	0.008719		0.0509	0.0230016580
ss86282822		6	7.032821	0.0903	0.3901	0.532441		0.0195	0.0032029711
ss61510597	*rs41605220*	6	7.091617	0.0566	0.1505	0.698199		0.0151	0.0016081600
ss61507082	*rs41602384*	6	7.112168	0.8489	1.4695	0.225837		0.0310	0.0079554155
ss86326807		6	7.178336	0.3202	0.0807	0.776380		0.0056	0.0024245524
	rs29011329	6	7.228428	0.8911	0.0526	0.818662		0.0068	0.0013197521
ss86316554		6	7.270852	0.7464	0.9390	0.332873		0.0210	0.0079318551

ss64963259	*rs43058181*	6	7.303014	0.5996	0.1290	0.719536		0.0068	0.0032655989
ss86287649		6	7.329803	0.1168	0.8694	0.351443		0.0276	0.0056945105
ss64836036	*rs42928688*	6	7.354027	0.8797	1.1214	0.289981		0.0310	0.0065719460
ss86284196		6	7.480916	0.5709	0.0007	0.979406		0.0005	0.0002441963
ss65366518	*rs43454188*	6	7.501457	0.2027	0.3651	0.545892		0.0138	0.0044753479
ss65366402	*rs43453275*	6	7.525602	0.2020	0.3578	0.549918		0.0137	0.0044208891
ss64885474	*rs42981531*	6	7.675177	0.2665	0.5180	0.471950		0.0152	0.0059458025
ss65366329	*rs43452402*	6	7.717403	0.7357	0.0117	0.914051		0.0023	0.0008805030
ss65366239	*rs43451112*	6	7.741467	0.3467	0.5716	0.449868		0.0147	0.0066758318
ss61557107	*rs41651306*	6	7.812534	0.1590	0.4946	0.482102		0.0176	0.0047001515
ss117968179		6	7.856063	0.7636	0.3439	0.557758		0.0128	0.0046138380
ss86318751		6	7.882522	0.8711	0.1527	0.696050		0.0111	0.0024901055
ss86338826		6	7.920291	0.2500	1.5261	0.217118		0.0260	0.0097483403
ss65366108	*rs43449982*	6	7.962426	0.1726	1.3231	0.250439		0.0282	0.0080580136
ss61557066	*rs41651267*	6	8.008552	0.1891	0.3824	0.536530		0.0141	0.0043264424
ss61557038	*rs41651239*	6	8.069248	0.0802	4.2597	0.039396		0.0681	0.0100546269
ss65365975	*rs43448750*	6	8.101295	0.0809	0.0552	0.814371		0.0079	0.0011753864
ss65365940	*rs43447915*	6	8.133705	0.9191	0.0552	0.814371		0.0079	0.0011753864
ss61499299	*rs43709694*	6	8.179014	0.0852	0.0059	0.938608		0.0025	0.0003965608
ss61491244	*rs41595940*	6	8.235476	0.3037	1.1764	0.278467		0.0216	0.0091348602
ss65365617	*rs43444992*	6	8.374342	0.6633	0.7372	0.390842		0.0166	0.0074326793
ss61556948	*rs41650750*	6	8.410986	0.8954	3.8355	0.050577		0.0595	0.0111475684
ss61517994	*rs41621357*	6	8.447718	0.1375	1.3185	0.251263		0.0297	0.0070435375
ss61557421	*rs41653411*	6	8.564704	0.7543	0.0639	0.800493		0.0055	0.0020238371
ss61473137	*rs41567558*	6	8.628612	0.2450	1.0707	0.301155		0.0222	0.0082175127
ss64531918	*rs42686628*	6	8.722376	0.9011	0.4608	0.497481		0.0215	0.0038299363
ss117967926		6	8.807367	0.2206	0.0078	0.929799		0.0020	0.0006952996
ss65038007	*rs43132272*	6	8.850886	0.7529	0.2276	0.633461		0.0105	0.0039165295
ss65071239	*rs43156932*	6	8.89371	0.6225	1.6104	0.204857		0.0237	0.0111185906
ss64879312	*rs42978088*	6	8.923843	0.3782	1.6258	0.202707		0.0238	0.0111876389
ss63422623	*rs42225323*	6	9.230391	0.5738	0.4822	0.487673		0.0126	0.0061527762
	rs29016228	6	9.256392	0.4807	0.0000	0.996616		0.0001	0.0000384006
ss63422698	*rs42226048*	6	9.317054	0.2436	1.2874	0.256922		0.0239	0.0088237268
ss61471431	*rs41570100*	6	9.337873	0.5738	0.4822	0.487673		0.0126	0.0061527762
ss86290531		6	9.417347	0.5229	0.8667	0.352198		0.0170	0.0084713723
ss61514537	*rs41613098*	6	9.440713	0.4477	3.0685	0.080264		0.0325	0.0160789752
ss63422974	*rs42226640*	6	9.511319	0.6254	1.3313	0.248969		0.0217	0.0101488225
ss117968753		6	9.612713	0.6361	0.0358	0.849954		0.0036	0.0016559131
ss86284507		6	9.766976	0.2364	0.7277	0.393910		0.0184	0.0066587479
ss65086667	*rs43172792*	6	9.804028	0.0938	0.0036	0.951947		0.0019	0.0003183512
ss64663489	*rs42760767*	6	9.899254	0.7600	1.5908	0.207641		0.0276	0.0100593217
ss63423910	*rs42225053*	6	10.046379	0.8467	3.8067	0.051450		0.0500	0.0129699456
ss64736572	*rs42831710*	6	10.273924	0.1533	0.0030	0.956675		0.0014	0.0003658333
ss64736546	*rs42831684*	6	10.31384	0.1383	0.0650	0.798781		0.0070	0.0016576419
ss64955328	*rs43051651*	6	10.43617	0.5122	0.0186	0.891484		0.0025	0.0012453698
ss61507551	*rs41607843*	6	10.495988	0.7450	0.4660	0.495062		0.0142	0.0053776681
ss64041788	*rs42445623*	6	10.526584	0.4656	0.0363	0.849037		0.0035	0.0017564424

ss38332992	*rs29016488*	6	10.571433	0.5036	0.0018	0.966300	0.0008	0.0003914628
ss64041970	*rs42446712*	6	10.600743	0.5673	0.4454	0.504735	0.0125	0.0061331841
ss61499895	*rs41596273*	6	10.649428	0.5766	0.8771	0.349322	0.0176	0.0085765058
ss64844080	*rs42934534*	6	10.674871	0.2278	0.1873	0.665296	0.0096	0.0033680227
ss61528452	*rs41625497*	6	10.704013	0.4370	0.1224	0.726505	0.0066	0.0032517496
ss64895907	*rs42991877*	6	10.726964	0.2299	0.0946	0.758521	0.0068	0.0024080350
	rs29023384	6	10.762144	0.8682	0.0107	0.917704	0.0029	0.0006539107
	rs29013320	6	10.805966	0.5523	0.0004	0.984456	0.0004	0.0001787637
ss86329950		6	10.843018	0.3489	0.0860	0.769428	0.0056	0.0025491921
ss61563921	*rs41665085*	6	10.929393	0.1454	0.7954	0.372775	0.0237	0.0058912554
ss61530870	*rs41630035*	6	10.958688	0.1361	1.7177	0.190417	0.0354	0.0083312665
	rs29011609	6	11.000273	0.4062	0.0235	0.878310	0.0029	0.0014065635
ss65367605	*rs43448267*	6	11.025692	0.3360	0.1859	0.666520	0.0086	0.0038166242
ss86295566		6	11.053368	0.5566	0.0839	0.772182	0.0054	0.0026744861
ss61507934	*rs41599924*	6	11.08294	0.9334	0.7580	0.384241	0.0329	0.0040974154
ss65367541	*rs43452189*	6	11.113331	0.1554	0.2417	0.623154	0.0127	0.0033329876
ss61491467	*rs41587860*	6	11.134421	0.0695	1.6846	0.194746	0.0471	0.0060896940
ss38327132	*rs29019244*	6	11.210497	0.3675	0.0428	0.836156	0.0041	0.0018837284
ss64948370	*rs43036259*	6	11.271796	0.0802	0.5807	0.446287	0.0260	0.0038317007
ss117968481		6	11.332157	0.4821	0.8755	0.349775	0.0174	0.0086944758
ss86322069		6	11.405908	0.5874	0.2464	0.619793	0.0093	0.0045300474
ss64292192	*rs42568408*	6	11.439346	0.2020	0.6851	0.408135	0.0191	0.0061675796
ss61495979	*rs41595536*	6	11.472659	0.5136	0.8426	0.358981	0.0171	0.0085580348
ss64292728	*rs42566480*	6	11.49752	0.8897	0.1008	0.750992	0.0096	0.0018808645
ss86341044		6	11.573836	0.5129	0.0333	0.855188	0.0033	0.0016464887
ss64997444	*rs43087872*	6	11.610877	0.8961	0.3081	0.579017	0.0172	0.0032078000
ss61495978	*rs41595535*	6	11.648469	0.7808	0.0001	0.994227	0.0002	0.0000553481
ss86340015		6	11.717259	0.8861	0.1553	0.693628	0.0116	0.0023413427
ss117968605		6	11.793953	0.1554	0.1687	0.681419	0.0104	0.0027290844
ss61557455	*rs41653743*	6	11.842042	0.2371	0.3252	0.568686	0.0125	0.0045146404
ss61469146	*rs41567757*	6	11.898721	0.6010	0.1632	0.686321	0.0075	0.0035799308
ss86330769		6	11.944693	0.1160	0.0039	0.950124	0.0018	0.0003787839
	rs29022442	6	12.02806	0.6010	0.0068	0.934199	0.0016	0.0007586834
ss86295997		6	12.080158	0.3446	0.3369	0.561817	0.0115	0.0051979894
ss64489644	*rs42664659*	6	12.114504	0.9047	0.2752	0.600058	0.0166	0.0028674872
ss117968484		6	12.170493	0.0917	2.0755	0.150129	0.0459	0.0076396218
ss64703056	*rs42798392*	6	12.191817	0.7027	0.2548	0.613907	0.0103	0.0043017050
ss61518562	*rs41615514*	6	12.323808	0.6819	0.0582	0.809419	0.0049	0.0021140406
ss61473339		6	12.358731	0.6046	0.0375	0.846577	0.0037	0.0017653378
ss64729383	*rs42826051*	6	12.43494	0.6146	0.6342	0.426075	0.0148	0.0070084908
ss61563714	*rs41663383*	6	12.472476	0.4463	0.0594	0.807543	0.0044	0.0021887629
ss61522664	*rs41621435*	6	12.541392	0.1576	1.3320	0.248849	0.0289	0.0076681399
ss64984404	*rs43078436*	6	12.562241	0.5559	0.0000	0.997907	0.0000	0.0000240058
ss61525339	*rs41620366*	6	12.58284	0.4026	0.0034	0.953668	0.0011	0.0005105788
	rs29016355	6	12.6174	0.6913	1.2502	0.263891	0.0219	0.0093619145
ss86313626		6	12.655296	0.5544	1.4089	0.235636	0.0219	0.0108195923
ss86279496		6	12.696915	0.5580	3.1186	0.077843	0.0331	0.0163398262

ss65227851	*rs43317085*	6	12.717445	0.2092	2.0976	0.147983	0.0333	0.0110029771
ss61513968	*rs41568139*	6	12.811968	0.6612	0.0421	0.837409	0.0041	0.0018359110
ss86339793		6	12.874271	0.7278	6.7900	0.009363	0.0545	0.0215860525
ss86313780		6	13.016128	0.7923	2.2526	0.133848	0.0342	0.0112643264
ss61557676	*rs41654960*	6	13.076723	0.2536	0.3982	0.528233	0.0136	0.0051427920
ss86303022		6	13.116893	0.4026	0.9727	0.324339	0.0186	0.0089640843
	rs29003287	6	13.138269	0.3123	0.1560	0.692986	0.0083	0.0035451066
ss65367897	*rs43450155*	6	13.16276	0.7643	0.5603	0.454402	0.0166	0.0059740722
ss61491536	*rs41587923*	6	13.225584	0.7665	0.5735	0.449118	0.0168	0.0060083872
ss63807934	*rs42358292*	6	13.246352	0.1211	0.1405	0.707851	0.0106	0.0022547664
ss86327806		6	13.269348	0.3768	0.1025	0.748917	0.0061	0.0028502984
ss61517134	*rs41612539*	6	13.308406	0.9628	0.6959	0.404464	0.0396	0.0028378511
ss86284183		6	13.429879	0.4026	0.0117	0.913798	0.0021	0.0009927581
ss65368154	*rs43451309*	6	13.459802	0.3968	0.0096	0.922014	0.0019	0.0008896170
ss63808669	*rs42361260*	6	13.62435	0.1777	0.0334	0.855125	0.0045	0.0013005444
ss86338305		6	13.649338	0.2915	0.7888	0.374771	0.0179	0.0074061839
ss86328346		6	13.676185	0.7572	1.2951	0.255499	0.0250	0.0091761011
ss64808725	*rs42903653*	6	13.706809	0.1948	0.4213	0.516511	0.0159	0.0049847351
ss61516368	*rs41614784*	6	13.733521	0.0451	0.1841	0.668017	0.0182	0.0015683155
ss86285010		6	13.780271	0.1297	0.4408	0.506959	0.0181	0.0040849221
ss86322531		6	13.85335	0.5021	0.4006	0.526992	0.0118	0.0059204538
	rs29013632	6	13.856009	0.3804	0.0026	0.959242	0.0010	0.0004563152
ss117968053		6	13.877386	0.5494	0.0747	0.784646	0.0051	0.0025388048
ss86341147		6	13.96073	0.1232	0.0006	0.979827	0.0007	0.0001528036
	rs29021212	6	13.990004	0.8202	0.7578	0.384312	0.0212	0.0062669421
ss61557858	*rs41653154*	6	14.011941	0.1024	0.5698	0.450589	0.0230	0.0042207922
ss86340151		6	14.076806	0.7822	1.0125	0.314660	0.0222	0.0075684206
ss86340592		6	14.146105	0.5136	0.7342	0.391832	0.0161	0.0080522591
ss65368576	*rs43448332*	6	14.176553	0.4169	2.6607	0.103306	0.0310	0.0150720171
ss86337411		6	14.222133	0.2615	0.6413	0.423508	0.0168	0.0064820419
ss86284596		6	14.262635	0.3159	0.0861	0.769306	0.0058	0.0025254924
ss117968143		6	14.286191	0.3481	0.4357	0.509439	0.0126	0.0057193161
ss86286784		6	14.349394	0.8825	1.9417	0.163930	0.0408	0.0084665619
ss86330103		6	14.391192	0.7658	0.0617	0.803960	0.0055	0.0019638942
ss65368204	*rs43451559*	6	14.421551	0.5595	0.2894	0.590772	0.0099	0.0048916203
ss86307748		6	14.443073	0.2572	0.7885	0.374846	0.0180	0.0068773819
ss61517967	*rs41620630*	6	14.479379	0.7393	0.8846	0.347258	0.0191	0.0073503232
ss86339698		6	14.619692	0.6669	4.2317	0.040049	0.0394	0.0175091991
	rs29021218	6	14.683309	0.6497	0.0066	0.935083	0.0016	0.0007255852
	rs29024201	6	14.776572	0.6719	2.7643	0.096839	0.0328	0.0144491683
ss64595726	*rs42711763*	6	14.921721	0.2006	2.9736	0.085077	0.0400	0.0128237022
ss61565097	*rs41658357*	6	15.12926	0.7708	0.0206	0.885835	0.0030	0.0010771655
ss61495118	*rs41593701*	6	15.189471	0.3388	1.7848	0.181992	0.0258	0.0115737347
ss61565101	*rs41658361*	6	15.221529	0.3345	5.2133	0.022715	0.0452	0.0201099662
ss86339331		6	15.246757	0.4169	0.4927	0.482948	0.0137	0.0066418558
ss86319824		6	15.311684	0.7457	0.1248	0.724013	0.0073	0.0027746626
ss63585504	*rs42279610*	6	15.382028	0.2758	3.0339	0.081983	0.0360	0.0143628168

ss61505262	*rs41604020*	6	15.457972	0.1003	3.2028	0.073948	0.0528	0.0095215402
ss86338926		6	15.496042	0.3603	4.1704	0.041513	0.0376	0.0173417245
ss61511449	*rs41612765*	6	15.538869	0.5330	7.9050	0.005069	0.0521	0.0259509299
ss61495120	*rs41593703*	6	15.573698	0.3897	4.1326	0.042444	0.0383	0.0182107413
ss86339784		6	15.595024	0.7665	0.1120	0.738007	0.0071	0.0025321440
ss63586314	*rs42283280*	6	15.638313	0.0351	1.9117	0.167220	0.0685	0.0046405764
ss86301199		6	15.684054	0.2378	1.0888	0.297094	0.0224	0.0081049074
ss86274744		6	15.731862	0.5229	0.8459	0.358031	0.0167	0.0083346926
ss63587637	*rs42279220*	6	15.827598	0.4420	0.6309	0.427302	0.0148	0.0072776395
ss86337036		6	15.852885	0.3138	0.7243	0.395043	0.0169	0.0072655690
ss86291926		6	15.874388	0.0845	6.8502	0.009056	0.0879	0.0135992297
ss65369259	*rs43454815*	6	15.909224	0.8596	0.0256	0.872939	0.0041	0.0010003694
	rs29021238	6	15.954146	0.2808	2.3621	0.124768	0.0310	0.0125029742
ss61520607	*rs41615630*	6	16.001116	0.5523	1.2127	0.271186	0.0200	0.0098992283
ss61558339	*rs41656194*	6	16.145596	0.1103	6.3949	0.011665	0.0760	0.0149221100
ss61491847	*rs41591011*	6	16.231243	0.2156	4.6282	0.031795	0.0473	0.0159857972
ss61558344	*rs41656198*	6	16.338863	0.6576	0.3013	0.583226	0.0110	0.0049511921
ss61558346	*rs41656200*	6	16.373919	0.3474	0.3737	0.541194	0.0122	0.0055223580
ss86336772		6	16.572208	0.3474	12.0044	0.000563	0.0649	0.0294071160
ss61520752	*rs41617474*	6	16.766841	0.3073	0.9335	0.334299	0.0192	0.0081827281
ss64935413	*rs43026165*	6	16.79361	0.0917	5.9338	0.015103	0.0794	0.0132249455
ss86341017		6	16.827787	0.5838	0.0260	0.871845	0.0032	0.0015321309
ss86316469		6	16.859247	0.6626	0.1215	0.727568	0.0067	0.0029843226
	rs29025372	6	16.888424	0.4004	2.5732	0.109142	0.0309	0.0148213124
ss86321295		6	16.912955	0.9119	0.0110	0.916346	0.0035	0.0005606343
ss65229429	*rs43317449*	6	16.976928	0.6676	1.3072	0.253293	0.0225	0.0099829594
ss86329062		6	17.000884	0.5150	1.4481	0.229248	0.0221	0.0110297551
ss86327912		6	17.067507	0.6605	4.0786	0.043813	0.0395	0.0177348400
ss65367420	*rs43451368*	6	17.102865	0.8589	0.2282	0.633038	0.0122	0.0029633985
ss61487873	*rs41586736*	6	17.173804	0.6941	14.4457	0.000157	0.0760	0.0322909092
ss86296449		6	17.199977	0.4413	12.3241	0.000476	0.0642	0.0316596948
ss86320438		6	17.224645	0.2636	0.8137	0.367333	0.0186	0.0072064715
ss86281767		6	17.345919	0.8940	1.1975	0.274198	0.0325	0.0061532640
ss61506465	*rs41606075*	6	17.402053	0.2880	1.5619	0.211813	0.0249	0.0102095170
ss61517445	*rs41614917*	6	17.425127	0.5301	1.8764	0.171191	0.0251	0.0124960341
ss86339188		6	17.449033	0.1289	12.9646	0.000340	0.0979	0.0220022634
ss65370900	*rs43453005*	6	17.471208	0.9176	4.0171	0.045429	0.0674	0.0101945486
ss117968682		6	17.529594	0.5974	18.4530	0.000020	0.0810	0.0389657736
ss65370959	*rs43453564*	6	17.575308	0.3044	8.2986	0.004090	0.0573	0.0242736402
ss65371023	*rs43453628*	6	17.624569	0.4219	2.3945	0.122220	0.0289	0.0140827789
ss65371068	*rs43453873*	6	17.662992	0.5201	4.3877	0.036561	0.0382	0.0190611935
ss61558350	*rs41656204*	6	17.703326	0.5215	4.1700	0.041522	0.0373	0.0186369020
ss86334804		6	17.737164	0.5258	2.5894	0.108037	0.0292	0.0145748464
ss65371196	*rs43454501*	6	17.769611	0.2908	4.0025	0.045819	0.0386	0.0159404701
ss65369268	*rs43454824*	6	17.790255	0.1884	2.3178	0.128352	0.0355	0.0108601142
ss61491667	*rs41589954*	6	17.810294	0.2729	5.1442	0.023630	0.0451	0.0179016553
ss65369377	*rs43456733*	6	17.883043	0.3574	0.1102	0.740022	0.0062	0.0028676999

ss117963886		6	17.96941	0.5960	0.0685	0.793618	0.0050	0.0023842663
ss86320017		6	18.027817	0.1383	2.2587	0.133323	0.0404	0.0096229581
ss61558016	*rs41654110*	6	18.094559	0.7744	3.7477	0.053284	0.0433	0.0151165022
ss61491714	*rs41590000*	6	18.119104	0.8617	0.2719	0.602200	0.0140	0.0033270802
	rs29022514	6	18.153464	0.2801	0.4033	0.525617	0.0127	0.0051334060
ss86298187		6	18.19616	0.2908	0.2091	0.647617	0.0091	0.0037431957
ss86289557		6	18.266804	0.8703	1.4696	0.225824	0.0338	0.0076391682
ss86280679		6	18.293251	0.7450	0.4745	0.491137	0.0146	0.0055495869
ss86319528		6	18.331172	0.6368	0.0593	0.807644	0.0046	0.0021074245
ss61469484	*rs41570391*	6	18.368732	0.0559	1.7980	0.180390	0.0538	0.0056738279
ss64129199	*rs42488988*	6	18.388864	0.7063	1.3338	0.248532	0.0235	0.0097379945
ss64129323	*rs42489948*	6	18.436575	0.5681	1.8320	0.176334	0.0256	0.0125544675
ss86310344		6	18.456745	0.2364	0.8034	0.370403	0.0192	0.0069391287
ss64129429	*rs42490001*	6	18.481438	0.3302	0.5124	0.474329	0.0141	0.0062546339
ss86322547		6	18.508514	0.7142	0.4012	0.526680	0.0129	0.0052610716
ss61524502	*rs41621541*	6	18.549387	0.1719	0.6014	0.438294	0.0191	0.0054381364
ss86324476		6	18.638264	0.4398	5.4288	0.020093	0.0447	0.0220120011
ss61531942	*rs41631481*	6	18.662522	0.1368	4.8977	0.027216	0.0571	0.0134869857
ss86297087		6	18.774775	0.3904	0.1484	0.700161	0.0073	0.0034928270
	rs29011400	6	18.874633	0.7443	0.1300	0.718543	0.0078	0.0029598617
ss86286951		6	18.901101	0.8030	0.0755	0.783522	0.0066	0.0020876484
ss86304208		6	19.016154	0.3109	0.1222	0.726743	0.0069	0.0029521800
ss86325906		6	19.12572	0.5308	0.6355	0.425633	0.0148	0.0073899012
ss65369974	*rs43453727*	6	19.149629	0.0652	2.1729	0.140913	0.0554	0.0067506901
ss63351868	*rs42196536*	6	19.231818	0.0666	0.6090	0.435438	0.0295	0.0036653129
ss61533010	*rs41628986*	6	19.369917	0.9205	0.0035	0.953086	0.0020	0.0002999380
ss61533000	*rs41628977*	6	19.411669	0.4234	0.5039	0.478035	0.0132	0.0064493316
ss86338145		6	19.439733	0.6218	1.2526	0.263452	0.0212	0.0099947374
	rs29014889	6	19.484685	0.3231	1.1984	0.274014	0.0214	0.0093581473
	rs29013947	6	19.512537	0.3610	0.0653	0.798386	0.0049	0.0022391408
ss63352312	*rs42196980*	6	19.600417	0.3818	0.1184	0.730826	0.0066	0.0031318340
ss86329076		6	19.63512	0.8281	0.3604	0.548491	0.0146	0.0041452388
ss86322304		6	19.660702	0.5838	0.0721	0.788450	0.0050	0.0024267614
ss61518104	*rs41611273*	6	19.695406	0.0903	3.1877	0.074628	0.0571	0.0093699725
ss86341296		6	19.728466	0.3116	6.9314	0.008658	0.0518	0.0222239202
ss65046681	*rs43134918*	6	19.789728	0.2586	0.3919	0.531521	0.0131	0.0050394126
ss61519692	*rs41617836*	6	19.841029	0.9076	0.3978	0.528455	0.0199	0.0033322356
ss117968863		6	19.88312	0.3840	2.0651	0.151150	0.0268	0.0127006791
ss86322942		6	19.98308	0.3646	0.0245	0.875673	0.0029	0.0013538791
ss61520247	*rs41612274*	6	20.088218	0.3954	0.0454	0.831311	0.0039	0.0018859902
ss63869838	*rs41595780*	6	20.117237	0.5981	1.5867	0.208216	0.0237	0.0113716387
ss61520252	*rs41612279*	6	20.1381	0.6984	0.1595	0.689724	0.0077	0.0032629932
ss46527007	*rs41255552*	6	20.330489	0.6017	1.3948	0.238008	0.0218	0.0104644944
ss61520248	*rs41612275*	6	20.42575	0.2643	0.8392	0.359940	0.0190	0.0074054462
ss61520250	*rs41612277*	6	20.463275	0.2650	0.7562	0.384829	0.0181	0.0070429928
ss64802915	*rs42894728*	6	20.510425	0.6619	0.9344	0.334049	0.0190	0.0085256532
ss64802817	*rs42894330*	6	20.532295	0.8503	4.9172	0.026913	0.0568	0.0144556161

ss61551874	rs43706944	6	20.565525	0.0444	0.0404	0.840790	0.0088	0.0007432811
ss63339871	rs42189699	6	20.586033	0.9563	0.0097	0.921586	0.0043	0.0003609762
ss86296895		6	20.609815	0.1698	0.5725	0.449535	0.0184	0.0051885531
ss63339781	rs42189669	6	20.710881	0.2944	0.0572	0.810971	0.0050	0.0020695317
ss86289053		6	20.742396	0.2335	3.6090	0.057882	0.0414	0.0148226423
ss63339706	rs42189644	6	20.804337	0.7077	1.7050	0.192070	0.0270	0.0111523957
ss61551871	rs43706941	6	21.016434	0.9713	0.2000	0.654886	0.0242	0.0013490944
ss61558162	rs41655747	6	21.108876	0.1576	2.2043	0.138082	0.0378	0.0100334205
ss65369830	rs43452284	6	21.129451	0.5315	0.7291	0.393481	0.0156	0.0077593980
ss86297642		6	21.151409	0.0630	0.5923	0.441789	0.0298	0.0035156822
ss65369725	rs43450879	6	21.197924	0.1203	6.0269	0.014333	0.0688	0.0145598063
ss86300054		6	21.238723	0.7278	1.7720	0.183567	0.0281	0.0111152103
ss65369672	rs43449526	6	21.272756	0.5867	6.1464	0.013404	0.0454	0.0220245336
ss86338976		6	21.307415	0.5186	4.9129	0.026979	0.0407	0.0203142030
ss65369565	rs43457821	6	21.331143	0.0451	3.8516	0.050098	0.0872	0.0075179084
ss61491746	rs41590030	6	21.40849	0.8087	0.1878	0.664851	0.0104	0.0032311136
ss64967975	rs43060029	6	21.538643	0.2973	0.9385	0.332989	0.0192	0.0080220642
ss117968279		6	21.679441	0.2715	1.9863	0.159172	0.0296	0.0117200304
ss86341527		6	21.699951	0.0623	0.1256	0.723196	0.0136	0.0015883945
ss61558359	rs41656213	6	21.712341	0.8617	0.1751	0.675747	0.0113	0.0026811311
ss61558363	rs41656217	6	21.736845	0.0251	0.0891	0.765453	0.0177	0.0008642368
	rs29011449	6	21.796335	0.7973	0.2098	0.647063	0.0104	0.0033585485
ss86337669		6	21.888948	0.3073	0.0250	0.874357	0.0032	0.0013705556
ss65371316	rs43455115	6	21.937107	0.1032	0.2996	0.584297	0.0166	0.0030745374
ss61524245	rs41619201	6	21.991393	0.2872	1.4104	0.235403	0.0249	0.0102120059
ss86340903		6	22.013011	0.9191	1.4916	0.222382	0.0414	0.0061552570
ss86340575		6	22.057626	0.3238	2.2317	0.135658	0.0299	0.0131103786
ss64658663	rs42754916	6	22.087546	0.1125	1.0006	0.317524	0.0297	0.0059319551
ss64658705	rs42756258	6	22.11207	0.4420	0.0689	0.792975	0.0049	0.0023964803
ss86336191		6	22.163543	0.3832	1.3183	0.251289	0.0212	0.0100318878
ss64257474	rs42553026	6	22.210179	0.1490	2.7034	0.100589	0.0428	0.0108500845
ss117968690		6	22.31705	0.6920	1.0092	0.315436	0.0205	0.0087279693
ss61470853	rs41573624	6	22.359287	0.6884	1.8347	0.176016	0.0267	0.0114756559
ss86318214		6	22.383466	0.0831	1.1230	0.289645	0.0346	0.0052742748
ss64489194	rs42662336	6	22.406491	0.0609	0.6626	0.415915	0.0320	0.0036539695
ss86340496		6	22.528096	0.1383	4.6553	0.031299	0.0583	0.0138937373
ss64459486	rs42647010	6	22.55669	0.5895	0.2813	0.595991	0.0103	0.0049715557
ss61470468	rs41570453	6	22.616876	0.5530	2.3615	0.124816	0.0297	0.0147023869
ss65378251	rs43464493	6	22.710062	0.2543	0.0593	0.807751	0.0051	0.0019523516
ss65378209	rs43464456	6	22.737174	0.0845	1.5128	0.219124	0.0392	0.0060701705
ss86337716		6	22.801627	0.2063	0.8616	0.353606	0.0210	0.0068644008
ss117975070		6	22.840396	0.4756	0.1409	0.707484	0.0069	0.0034251987
ss64025629	rs42435921	6	22.877833	0.7285	0.2557	0.613231	0.0106	0.0041897915
ss64025667	rs42436739	6	22.914656	0.9040	2.7705	0.096468	0.0540	0.0093682789
ss64534856	rs42682580	6	22.9593	0.2407	0.1621	0.687365	0.0089	0.0032464878
ss64681408	rs42782754	6	23.211286	0.3044	0.0016	0.968042	0.0008	0.0003345548
ss61517988	rs41621351	6	23.283249	0.6748	0.0000	0.997220	0.0001	0.0000307834

ss61491858	*rs41591022*	6	23.312426	0.6748	0.0000	0.997220	0.0001	0.0000307834
ss117963870		6	23.332869	0.3252	0.0000	0.997220	0.0001	0.0000307834
ss86333565		6	23.422853	0.1454	1.5765	0.209682	0.0336	0.0083483130
ss117968769		6	23.457827	0.6927	0.0633	0.801413	0.0051	0.0021647313
ss65370216	*rs43457467*	6	23.553952	0.1640	0.0253	0.873742	0.0040	0.0010842835
ss65370341	*rs43458592*	6	23.637197	0.8360	0.0253	0.873742	0.0040	0.0010842835
ss86328063		6	23.692578	0.6096	2.3024	0.129631	0.0290	0.0138184798
ss65370434	*rs43459884*	6	23.720886	0.0766	0.2015	0.653630	0.0161	0.0022783371
ss61469212	*rs41567822*	6	23.744743	0.6375	0.2350	0.628023	0.0092	0.0042458226
ss86330590		6	23.782075	0.4277	0.2961	0.586500	0.0105	0.0051410864
ss61491842	*rs41591006*	6	23.826442	0.6626	0.2683	0.604667	0.0106	0.0047597400
ss86337851		6	23.878187	0.4033	2.2991	0.129904	0.0285	0.0137145761
ss65370624	*rs43451680*	6	23.999006	0.8983	0.5891	0.443022	0.0245	0.0044704281
ss61531243	*rs41633202*	6	24.143205	0.3288	0.8955	0.344333	0.0189	0.0083425965
ss86285897		6	24.182984	0.8689	0.0000	0.999290	0.0000	0.0000056214
ss86294642		6	24.220362	0.4062	0.1346	0.713778	0.0067	0.0032530036
ss86287065		6	24.25127	0.8245	0.1875	0.665105	0.0101	0.0029357466
ss61529259	*rs41633767*	6	24.356726	0.7958	0.6086	0.435565	0.0176	0.0057204753
ss86299857		6	24.420716	0.8668	0.0441	0.833702	0.0058	0.0013377288
ss86284793		6	24.45146	0.1798	0.8139	0.367290	0.0208	0.0061463264
ss61505492	*rs41606230*	6	24.493632	0.4255	2.5805	0.108638	0.0308	0.0150779654
ss64300368	*rs42571657*	6	24.528301	0.7722	0.2442	0.621326	0.0108	0.0038025028
ss64300070	*rs42579003*	6	24.61854	0.9499	0.1140	0.735772	0.0144	0.0013692112
ss64690938	*rs42785271*	6	24.72997	0.3926	3.9120	0.048335	0.0384	0.0183009854
ss64690955	*rs42785288*	6	24.758573	0.4921	1.6019	0.206064	0.0240	0.0119962143
ss64733527	*rs42827584*	6	24.836889	0.9620	0.4197	0.517297	0.0317	0.0023159541
ss61526611	*rs41624597*	6	24.901932	0.5709	0.7159	0.397788	0.0156	0.0076508798
ss61476568	*rs41576923*	6	24.924734	0.4269	1.1155	0.291258	0.0194	0.0094801861
ss86276571		6	24.955549	0.5143	0.0029	0.957129	0.0010	0.0004795960
	rs29012386	6	24.997937	0.1762	6.6814	0.009945	0.0619	0.0179797291
ss61495556	*rs41598026*	6	25.055121	0.5473	0.3153	0.574638	0.0103	0.0050910570
ss64252132	*rs42547291*	6	25.121859	0.0709	5.1723	0.023253	0.0712	0.0093883001
ss64252142	*rs42547296*	6	25.165208	0.1576	0.4054	0.524544	0.0161	0.0042831038
ss61565956	*rs41664497*	6	25.195079	0.5208	0.2897	0.590564	0.0099	0.0049509124
ss61565964	*rs41664505*	6	25.226037	0.5430	2.3162	0.128489	0.0291	0.0144429890
ss61481202	*rs41583316*	6	25.269091	0.6117	0.2609	0.609670	0.0098	0.0046606176
ss86312804		6	25.327693	0.4807	0.9752	0.323718	0.0181	0.0090418587
ss117963899		6	25.360042	0.5330	0.1727	0.677811	0.0077	0.0038356865
ss61481201	*rs41583315*	6	25.469242	0.7980	0.8149	0.366981	0.0209	0.0067246515
ss86288551		6	25.497929	0.7092	0.0910	0.763056	0.0062	0.0025536430
ss86312651		6	25.55154	0.9040	5.0414	0.025061	0.0720	0.0124892430
ss61513374	*rs41612056*	6	25.590915	0.5824	0.8015	0.370947	0.0168	0.0081735833
ss86285550		6	25.658566	0.6110	0.1551	0.693869	0.0074	0.0035408975
ss61515462	*rs41614318*	6	25.837739	0.5401	0.0719	0.788662	0.0049	0.0024334455
ss61562983	*rs41657783*	6	25.869514	0.5380	0.1282	0.720413	0.0066	0.0032629050
ss61562982	*rs41657782*	6	25.933489	0.7328	0.4319	0.511251	0.0139	0.0054432375
ss86296956		6	25.954072	0.2672	0.4319	0.511251	0.0139	0.0054432375

ss61477972		6	25.98133	0.7077	0.0000	0.996607	0.0001	0.0000363153
ss61491871	*rs41591935*	6	26.027105	0.8811	9.1159	0.002627	0.0859	0.0179942047
ss86338844		6	26.237471	0.2042	0.0450	0.831982	0.0049	0.0015998977
ss61521309	*rs41618228*	6	26.378567	0.4047	0.0045	0.946565	0.0013	0.0006049917
ss117968394		6	26.537284	0.9298	0.0458	0.830646	0.0079	0.0010343911
ss86290235		6	26.706544	0.2550	0.7204	0.396303	0.0186	0.0070809513
ss117968493		6	26.871284	0.6590	0.2932	0.588363	0.0110	0.0049489186
ss86290080		6	26.923915	0.2837	0.2079	0.648552	0.0097	0.0039433442
ss86313625		6	26.945718	0.7163	1.9903	0.158759	0.0294	0.0119360229
	rs29013799	6	26.979605	0.9062	0.0218	0.882724	0.0047	0.0008000331
ss61544482	*rs41644577*	6	27.132607	0.5251	0.3554	0.551288	0.0104	0.0051976650
ss61513484	*rs41612964*	6	27.23391	0.7156	0.2711	0.602758	0.0106	0.0043204117
ss61513479	*rs41612959*	6	27.256501	0.6268	0.2333	0.629227	0.0094	0.0044170887
ss86328602		6	27.335927	0.3138	5.6889	0.017340	0.0476	0.0205122114
	rs29017678	6	27.36078	0.2822	0.0007	0.978888	0.0005	0.0002195526
ss86292923		6	27.476705	0.6390	5.7031	0.017201	0.0446	0.0205816793
ss61524048		6	27.504768	0.9162	1.8278	0.176832	0.0461	0.0070782265
ss86335528		6	27.59299	0.6669	7.3418	0.006903	0.0515	0.0229012694
ss86327765		6	27.653522	0.5415	1.8281	0.176786	0.0245	0.0121493474
ss86338456		6	27.675521	0.5437	1.5417	0.214790	0.0225	0.0111415993
ss86340493		6	27.76108	0.4900	0.1043	0.746852	0.0061	0.0030260201
ss86305923		6	27.786722	0.1640	0.0859	0.769604	0.0076	0.0020964344
ss61556874	*rs41649876*	6	27.831793	0.6605	0.4197	0.517316	0.0130	0.0058175731
ss86340399		6	27.887362	0.8582	8.7302	0.003235	0.0790	0.0192409837
ss61469091	*rs41567002*	6	27.967005	0.1218	0.9175	0.338459	0.0268	0.0057406144
ss86340531		6	28.129913	0.4821	10.0430	0.001596	0.0605	0.0302241643
ss86283819		6	28.169845	0.3245	0.0203	0.886778	0.0029	0.0012720789
ss117968137		6	28.281604	0.5809	6.9217	0.008704	0.0499	0.0242791533
ss86299397		6	28.314059	0.3510	10.6804	0.001136	0.0655	0.0298605898
ss61496196	*rs41597449*	6	28.485878	0.6175	2.0861	0.149099	0.0284	0.0134151760
ss61511173	*rs41610298*	6	28.535175	0.7636	3.7249	0.054014	0.0434	0.0156601245
ss61565459	*rs41660811*	6	28.567609	0.2235	2.2457	0.134441	0.0344	0.0119297277
ss61521103	*rs41620119*	6	28.599716	0.7751	2.2555	0.133597	0.0344	0.0120028770
ss86316632		6	28.622869	0.9219	1.6143	0.204310	0.0454	0.0065422877
ss86293630		6	28.849193	0.5795	0.0001	0.992358	0.0002	0.0000894191
ss117968822		6	28.912009	0.6920	10.1203	0.001532	0.0648	0.0276127286
ss117968165		6	29.083108	0.0337	0.0247	0.875226	0.0078	0.0005066860
ss117963864		6	29.139242	0.1282	0.0805	0.776689	0.0081	0.0018040535
ss86318987		6	29.162223	0.6354	1.9038	0.168093	0.0270	0.0124989637
ss86330005		6	29.212237	0.6934	0.1699	0.680309	0.0082	0.0034958755
	rs29017713	6	29.239362	0.3095	8.8363	0.003055	0.0601	0.0256687458
ss86329287		6	29.252296	0.6332	1.2368	0.266469	0.0217	0.0100856399
ss86331652		6	29.529308	0.6948	1.1483	0.284270	0.0217	0.0092156756
ss61556897	*rs41649899*	6	29.803112	0.7156	0.0824	0.774221	0.0060	0.0024319860
ss86308392		6	29.836485	0.1304	2.5541	0.110461	0.0445	0.0100959528
ss86310321		6	29.920728	0.4563	0.5429	0.461474	0.0141	0.0069971657
ss61472156	*rs41567696*	6	30.004186	0.5702	7.6568	0.005806	0.0527	0.0258128426

ss86283952		6	30.157491	0.1777	4.3517	0.037335	0.0523	0.0152751862
ss61472167	*rs41567707*	6	30.242396	0.0387	1.0596	0.303669	0.0489	0.0036401975
ss86310063		6	30.503077	0.2521	0.4538	0.500754	0.0145	0.0054508216
ss61491195	*rs41595091*	6	30.691546	0.3797	6.1992	0.013013	0.0491	0.0231363370
	rs29013454	6	30.750198	0.7436	3.7723	0.052510	0.0407	0.0155245182
	rs29023061	6	30.817481	0.6282	4.7150	0.030237	0.0408	0.0190373473
ss61515341	*rs41613697*	6	30.85546	0.8052	0.0277	0.867966	0.0039	0.0012230457
ss61491196	*rs41595092*	6	30.899614	0.3646	1.2779	0.258687	0.0219	0.0101593432
ss61472785	*rs41574004*	6	30.94061	0.8789	0.3527	0.552763	0.0168	0.0035711229
	rs29016266	6	31.006416	0.5394	0.2469	0.619411	0.0093	0.0046161345
ss86297301		6	31.038646	0.3489	1.2472	0.264471	0.0210	0.0095292402
ss117968875		6	31.065581	0.0337	15.1094	0.000111	0.1947	0.0126666160
ss61491200	*rs41595096*	6	31.147081	0.0401	15.2791	0.000102	0.1814	0.0139669126
ss86300703		6	31.210288	0.0587	1.0116	0.314857	0.0400	0.0044245187
ss86340757		6	31.235325	0.9076	3.7965	0.051762	0.0617	0.0103561148
ss86330859		6	31.264649	0.7156	4.0905	0.043507	0.0421	0.0171179355
ss86286040		6	31.285198	0.3804	0.4735	0.491621	0.0133	0.0062644491
ss61556915	*rs41649917*	6	31.357382	0.7034	1.0103	0.315183	0.0201	0.0083868738
ss61529369	*rs41624497*	6	31.444334	0.7436	2.2201	0.136675	0.0327	0.0124572221
ss61556917	*rs41649919*	6	31.470687	0.4613	0.0003	0.986210	0.0003	0.0001647615
ss117963858		6	31.521514	0.2328	0.0117	0.913791	0.0025	0.0008897576
	rs29025673	6	31.579806	0.5244	0.2518	0.615950	0.0094	0.0046701525
ss61466344	*rs41568099*	6	31.664149	0.3653	2.5969	0.107529	0.0293	0.0135792581
ss61523653	*rs41622316*	6	31.729357	0.0738	3.9585	0.047026	0.0696	0.0095074233
ss86329848		6	31.783986	0.3252	0.4254	0.514492	0.0124	0.0054527871
ss61556925	*rs41649927*	6	31.807491	0.7264	0.1171	0.732251	0.0071	0.0028043167
ss38327163	*rs29019275*	6	31.895196	0.1884	0.0133	0.908386	0.0027	0.0008256769
ss61523659	*rs41622322*	6	31.915392	0.1905	0.3476	0.555670	0.0137	0.0042406693
	rs29009843	6	31.937564	0.3926	0.2946	0.587451	0.0101	0.0048023715
ss117963896		6	31.963669	0.4663	1.5893	0.207855	0.0240	0.0119656766
ss61556926	*rs41649928*	6	32.023121	0.0874	0.0120	0.912666	0.0037	0.0005828100
ss61491207	*rs41595103*	6	32.064221	0.9019	0.1116	0.738398	0.0107	0.0018882594
ss61522780	*rs41622049*	6	32.130121	0.7414	1.4088	0.235656	0.0246	0.0094236867
	rs29024115	6	32.154208	0.8990	0.0732	0.786833	0.0085	0.0015526680
ss117968817		6	32.240785	0.6870	0.7215	0.395930	0.0175	0.0075407420
ss86319866		6	32.336516	0.1812	0.8621	0.353483	0.0221	0.0065533074
ss61498782	*rs43706686*	6	32.685287	0.2715	4.9326	0.026676	0.0451	0.0178506191
ss86314658		6	32.960408	0.4728	8.7481	0.003204	0.0555	0.0276713515
ss61514559	*rs41613120*	6	33.01193	0.0394	1.5131	0.219087	0.0582	0.0044051932
ss61514566	*rs41613127*	6	33.070179	0.7987	3.5874	0.058632	0.0441	0.0141723593
ss86314390		6	33.278019	0.7020	1.0082	0.315693	0.0203	0.0084889489
ss61556931	*rs41650733*	6	33.328168	0.7393	6.8769	0.008923	0.0544	0.0209743647
ss86329252		6	33.400069	0.3087	0.0562	0.812733	0.0045	0.0019196952
ss117968366		6	33.444496	0.5537	0.1073	0.743393	0.0062	0.0030729383
ss61491209	*rs41595105*	6	33.49858	0.4814	3.2492	0.071890	0.0315	0.0157250282
ss117968353		6	33.521995	0.6640	1.7337	0.188377	0.0261	0.0116530757
ss117968732		6	33.542442	0.3546	0.0037	0.951828	0.0011	0.0005238327

ss117968740		6	33.596514	0.3073	0.3051	0.580863	0.0111	0.0047169983
ss117968742		6	33.620176	0.9477	5.3906	0.020533	0.0982	0.0097293163
ss117968819		6	33.689669	0.5695	11.7061	0.000659	0.0621	0.0304693663
ss117968455		6	33.720459	0.5516	8.3258	0.004029	0.0535	0.0264631462
ss117968721		6	33.761326	0.2579	1.1891	0.275879	0.0222	0.0084964344
ss117968553		6	33.785059	0.2622	1.3860	0.239480	0.0239	0.0092417800
ss117968321		6	33.825465	0.3976	2.9405	0.086830	0.0314	0.0150496219
ss117968183		6	33.847045	0.0681	7.3377	0.006918	0.0985	0.0124978006
ss117968693		6	33.880373	0.2321	1.5719	0.210358	0.0270	0.0096225227
ss117968556		6	33.914492	0.3145	0.1266	0.722113	0.0069	0.0029673486
	rs29018333	6	33.989254	0.3410	2.2179	0.136868	0.0299	0.0134426717
ss117968632		6	34.061075	0.8424	9.0715	0.002690	0.0750	0.0199196817
ss61556960	*rs41650762*	6	34.10266	0.7421	0.9167	0.338664	0.0204	0.0078213020
ss61556961	*rs41650763*	6	34.135835	0.2579	0.9167	0.338664	0.0204	0.0078213020
ss61556962	*rs41650764*	6	34.257928	0.5967	1.2529	0.263379	0.0211	0.0101675610
ss117975029		6	34.30926	0.2292	1.7857	0.181892	0.0292	0.0103036619
ss117975026		6	34.337627	0.8259	8.6449	0.003388	0.0707	0.0203398888
ss117975030		6	34.391776	0.8266	8.3169	0.004049	0.0694	0.0199037172
ss61477204	*rs41575451*	6	34.740607	0.4807	0.2598	0.610391	0.0097	0.0048190917
ss61500094	*rs41597872*	6	34.765833	0.4950	0.3918	0.531560	0.0112	0.0056029167
ss61507445	*rs41606639*	6	34.790008	0.5716	2.6699	0.102717	0.0303	0.0148164078
ss86332943		6	34.991977	0.2693	4.7691	0.029309	0.0453	0.0178441805
ss61491239	*rs41595935*	6	35.070616	0.0602	0.9366	0.333502	0.0376	0.0042576703
ss61491238	*rs41595934*	6	35.095139	0.7299	5.5732	0.018512	0.0473	0.0186448261
ss61491232	*rs41595128*	6	35.250054	0.3388	0.2760	0.599481	0.0103	0.0046365249
ss61469099	*rs41567010*	6	35.346416	0.7099	2.8919	0.089475	0.0336	0.0138558028
	rs29024248	6	35.46292	0.7070	1.9687	0.161028	0.0277	0.0114807989
ss117968452		6	35.622831	0.3961	0.1643	0.685356	0.0077	0.0036607334
ss61471476	*rs41570937*	6	35.690337	0.2135	0.1365	0.711923	0.0082	0.0027651633
ss61471475	*rs41570936*	6	35.743452	0.2099	0.0977	0.754707	0.0070	0.0023339201
ss61570206	*rs41665871*	6	35.778162	0.6583	0.0027	0.958300	0.0010	0.0004516167
	rs29012836	6	35.815787	0.0903	10.6976	0.001126	0.1048	0.0172145639
ss86274147		6	35.84384	0.9140	4.8659	0.027718	0.0730	0.0114681056
ss86305280		6	35.895396	0.5788	0.0072	0.932538	0.0016	0.0007684216
	rs29013268	6	35.955586	0.8875	14.3836	0.000162	0.1075	0.0214634682
ss86340820		6	36.088414	0.2887	0.0810	0.776058	0.0057	0.0023443413
ss86279767		6	36.280418	0.3474	1.5237	0.217471	0.0240	0.0108683700
ss61496872	*rs41592814*	6	36.496735	0.1361	6.5148	0.010910	0.0683	0.0160572583
ss61568729	*rs41665813*	6	36.535377	0.8940	0.6064	0.436409	0.0238	0.0045173906
		6	36.720301	0.5444	0.5800	0.446556	0.0137	0.0067794187
ss117963880		6	36.784645	0.6612	0.2217	0.637885	0.0094	0.0042060899
ss86317675		6	36.825784	0.8811	0.0603	0.806105	0.0067	0.0014133491
ss61491272	*rs41595968*	6	36.848689	0.9305	1.4197	0.233859	0.0424	0.0054824338
ss117968212		6	36.886557	0.6554	6.0945	0.013799	0.0465	0.0210251261
ss117968515		6	36.911818	0.2457	2.5686	0.109456	0.0329	0.0122114926
ss117968771		6	36.937295	0.3395	3.4533	0.063548	0.0350	0.0157135943
ss117968215		6	36.969463	0.6927	1.2784	0.258585	0.0223	0.0095127464

ss117968110		6	37.024133	0.0716	3.1061	0.078438	0.0601	0.0079886754
ss117968790		6	37.045882	0.7736	0.1821	0.669684	0.0093	0.0032642100
ss117968078		6	37.096526	0.3897	1.9725	0.160634	0.0260	0.0123564272
ss117968140		6	37.135014	0.7085	2.8786	0.090212	0.0327	0.0135210133
ss117968157		6	37.193156	0.2572	0.6216	0.430723	0.0162	0.0061797024
ss61556992	*rs41650794*	6	37.231102	0.4678	0.8959	0.344200	0.0172	0.0085495425
ss117968209		6	37.260383	0.7371	1.2657	0.260959	0.0232	0.0089966611
ss86312899		6	37.317021	0.2736	11.5834	0.000704	0.0699	0.0277807083
ss61477537	*rs41577868*	6	37.374911	0.8009	0.8586	0.354463	0.0210	0.0066990815
	rs29010895	6	37.433108	0.2951	5.6190	0.018039	0.0479	0.0199118148
ss61523660	*rs41622323*	6	37.45441	0.1497	3.6099	0.057849	0.0494	0.0125682835
ss117968323		6	37.524839	0.7736	3.1017	0.078651	0.0377	0.0132180408
ss117968399		6	37.56438	0.4047	1.5123	0.219211	0.0237	0.0114332599
ss117968745		6	37.594439	0.3532	1.1824	0.277248	0.0210	0.0095785853
ss117968260		6	37.624773	0.5308	0.0034	0.953751	0.0010	0.0005110732
ss117968203		6	37.647259	0.5573	0.0905	0.763677	0.0054	0.0026876680
ss117968206		6	37.669926	0.1032	0.0873	0.767748	0.0088	0.0016360457
ss117968512		6	37.704774	0.3446	1.3453	0.246495	0.0230	0.0103832138
		6	37.746632	0.8216	2.4332	0.119247	0.0379	0.0111123722
ss117968523		6	37.852401	0.5365	0.5211	0.470610	0.0130	0.0064551436
ss86311757		6	37.874925	0.5394	0.5522	0.457672	0.0134	0.0066804342
ss117968245		6	37.963148	0.1533	0.5003	0.479592	0.0184	0.0047706244
ss86338310		6	38.014255	0.2056	3.3054	0.069484	0.0421	0.0137431053
ss86276045		6	38.102328	0.8080	4.5475	0.033316	0.0512	0.0158728040
ss61519674	*rs41616921*	6	38.133291	0.7178	1.1357	0.286930	0.0213	0.0086096846
ss117968338		6	38.212942	0.3288	12.2937	0.000484	0.0678	0.0299389768
ss117968027		6	38.233089	0.6691	11.6490	0.000680	0.0658	0.0291218189
ss117968573		6	38.25689	0.7170	10.9188	0.001001	0.0656	0.0266382791
ss117968779		6	38.301285	0.1726	5.0061	0.025574	0.0560	0.0159998418
ss117968776		6	38.326148	0.8087	4.6252	0.031850	0.0516	0.0159711481
ss117968414		6	38.420477	0.6934	1.0666	0.302073	0.0205	0.0087049357
ss86319765		6	38.455997	0.1368	6.2445	0.012687	0.0680	0.0160575026
ss117968155		6	38.479644	0.2156	4.7630	0.029411	0.0483	0.0163371975
ss117968638		6	38.50021	0.7844	4.7630	0.029411	0.0483	0.0163371975
ss117967969		6	38.533539	0.0716	4.1801	0.041276	0.0740	0.0098447974
ss117968496		6	38.558527	0.3395	12.0889	0.000539	0.0671	0.0300915941
	rs29026121	6	38.600495	0.8252	4.6194	0.031956	0.0529	0.0152527536
ss117968417		6	38.638963	0.7299	0.0214	0.883646	0.0029	0.0011626886
ss64727336	*rs42820837*	6	38.658981	0.1540	0.4031	0.525723	0.0160	0.0041715248
ss86285734		6	38.697374	0.8345	4.7723	0.029254	0.0555	0.0153170413
ss117968562		6	38.729867	0.6691	2.9213	0.087863	0.0327	0.0145016611
ss117968730		6	38.756336	0.4477	2.0308	0.154585	0.0264	0.0130769651
ss64051193	*rs42447219*	6	38.824039	0.2744	1.5256	0.217188	0.0254	0.0101003739
ss64051173	*rs42447209*	6	38.846448	0.4162	0.0216	0.883125	0.0027	0.0013332521
ss117968446		6	38.888187	0.5817	0.0695	0.792113	0.0049	0.0023888313
ss117968748		6	38.914557	0.5537	1.6613	0.197858	0.0237	0.0117017072
ss117968277		6	38.982339	0.4105	3.2833	0.070420	0.0335	0.0162368913

ss117968388		6	39.052609	0.4327	3.3623	0.067132	0.0339	0.0166566568
ss86341290		6	39.072638	0.5036	2.6799	0.102072	0.0306	0.0152908639
ss117968064		6	39.108079	0.3503	5.0723	0.024622	0.0422	0.0191893951
ss117968677		6	39.139503	0.2787	3.4933	0.062038	0.0390	0.0156670089
ss117968369		6	39.159588	0.6884	0.8351	0.361132	0.0184	0.0078742198
ss117968293		6	39.202497	0.4513	1.6794	0.195438	0.0239	0.0118589181
ss117968640		6	39.223438	0.9262	3.1184	0.077852	0.0620	0.0084749431
ss61557018	*rs41650820*	6	39.486005	0.5423	6.2116	0.012923	0.0470	0.0233162893
ss65383999	*rs43467099*	6	39.509021	0.3145	0.4631	0.496388	0.0137	0.0059116857
ss117968799		6	39.531542	0.4599	0.0322	0.857637	0.0034	0.0016647520
ss117968440		6	39.604432	0.4520	0.7051	0.401369	0.0161	0.0079760163
ss117968069		6	39.632777	0.4685	5.9694	0.014804	0.0452	0.0225077892
ss117968056		6	39.770116	0.6583	1.3942	0.238107	0.0224	0.0100803422
ss117968371		6	39.794335	0.5301	0.3526	0.552846	0.0108	0.0053639301
ss117968478		6	39.844524	0.2056	0.0052	0.942653	0.0016	0.0005357647
ss117968067		6	39.945339	0.4405	0.5652	0.452445	0.0140	0.0068849758
ss117968489		6	39.97721	0.8496	0.7738	0.379358	0.0224	0.0057218773
ss117968048		6	39.997429	0.4355	2.5218	0.112737	0.0292	0.0143600579
ss64881896	*rs42973724*	6	40.024243	0.4241	2.5081	0.113716	0.0293	0.0143207200
ss117968717		6	40.096369	0.5802	2.8318	0.092866	0.0311	0.0151425815
ss117968559		6	40.151937	0.4857	5.5194	0.019086	0.0442	0.0220746235
ss117968435		6	40.183166	0.6755	5.2997	0.021623	0.0443	0.0194151306
ss117975041		6	40.421547	0.6755	5.2997	0.021623	0.0443	0.0194151306
ss86297866		6	40.470573	0.2113	0.9847	0.321392	0.0228	0.0075866999
ss117968802		6	40.616626	0.3216	3.5807	0.058870	0.0383	0.0167300641
ss117967985		6	40.655229	0.3030	0.0858	0.769626	0.0058	0.0024371792
	rs29017603	6	40.699508	0.3496	3.7104	0.054482	0.0385	0.0175046426
ss65376454	*rs43463315*	6	40.720194	0.4219	0.4367	0.508955	0.0123	0.0060200569
ss65376475	*rs43464536*	6	40.741316	0.2643	0.1150	0.734588	0.0072	0.0027916114
ss86287550		6	40.778484	0.5802	0.6140	0.433569	0.0146	0.0071284295
ss65529970	*rs43610172*	6	40.838116	0.2772	0.9432	0.331786	0.0202	0.0081063239
ss117968329		6	40.891659	0.5573	0.8157	0.366749	0.0167	0.0082383656
	rs29027071	6	40.966663	0.5860	2.0026	0.157473	0.0270	0.0130914246
ss117968299		6	40.99504	0.5150	2.3707	0.124089	0.0280	0.0139705019
ss117968698		6	41.028974	0.1153	8.3327	0.004014	0.0854	0.0174228311
ss117968296		6	41.092138	0.7199	0.1115	0.738513	0.0072	0.0029139087
ss117968599		6	41.155258	0.2636	1.5308	0.216408	0.0266	0.0103451789
ss117967982		6	41.208357	0.2385	0.0066	0.935280	0.0018	0.0006456555
ss65376428	*rs43463290*	6	41.242588	0.6024	2.9214	0.087859	0.0319	0.0152971715
ss117968118		6	41.276776	0.7751	10.1016	0.001547	0.0712	0.0248327868
ss117968758		6	41.300912	0.5236	3.5360	0.060467	0.0353	0.0175944092
ss117968102		6	41.323841	0.7779	3.6112	0.057803	0.0446	0.0154075554
ss117968124		6	41.373556	0.5158	0.0642	0.799981	0.0047	0.0023712283
ss117968419		6	41.399983	0.3223	0.0211	0.884464	0.0029	0.0012468412
ss117967954		6	41.429808	0.6519	3.4379	0.064139	0.0356	0.0161803868
ss117968696		6	41.462783	0.3840	2.2907	0.130606	0.0290	0.0137139066
ss65374229	*rs43459713*	6	41.50694	0.3718	7.8730	0.005158	0.0518	0.0241768000

ss86301437		6	41.541415	0.2321	1.5111	0.219382		0.0274	0.0097827261
ss65373967	*rs43457658*	6	41.568482	0.3324	0.1567	0.692341		0.0080	0.0035476871
ss86300871		6	41.625127	0.2880	0.0071	0.932900		0.0017	0.0007115327
ss61557056	*rs41651257*	6	41.653749	0.8030	0.4036	0.525432		0.0149	0.0047296586
ss61557057	*rs41651258*	6	41.73503	0.3181	1.1532	0.283262		0.0215	0.0093148766
ss65375030	*rs43459808*	6	41.768622	0.6619	3.0797	0.079714		0.0342	0.0152878343
ss86319785		6	41.806866	0.3181	2.2559	0.133560		0.0300	0.0130196652
ss61491262	*rs41595958*	6	41.832768	0.6805	3.4975	0.061882		0.0377	0.0163959286
ss61557043		6	41.867043	0.4341	7.4764	0.006410		0.0512	0.0251755068
ss65374622	*rs43464000*	6	41.902577	0.9183	0.4684	0.493965		0.0229	0.0034399954
ss61491257	*rs41595953*	6	41.951591	0.8274	3.4990	0.061826		0.0470	0.0134162536
ss86321551		6	41.978851	0.7034	1.0983	0.294995		0.0211	0.0088031096
ss61491271	*rs41595967*	6	42.006515	0.5652	0.0687	0.793359		0.0049	0.0024045682
ss65375414	*rs43464892*	6	42.099468	0.2643	2.4266	0.119748		0.0329	0.0127849108
ss61557082	*rs41651283*	6	42.122347	0.5817	0.0068	0.934465		0.0016	0.0007597144
ss65375397	*rs43464875*	6	42.157837	0.3933	0.0724	0.787953		0.0051	0.0024466136
ss117968397		6	42.204901	0.4205	0.0233	0.878642		0.0029	0.0013979091
ss65375217	*rs43462195*	6	42.321332	0.4405	0.5766	0.447920		0.0143	0.0070608477
ss65375148	*rs43460926*	6	42.348465	0.3596	0.0221	0.881914		0.0029	0.0013460517
ss61557072	*rs41651273*	6	42.376126	0.2285	0.3711	0.542606		0.0134	0.0047196508
ss61510571	*rs41605195*	6	42.434621	0.1404	0.8755	0.349756		0.0253	0.0060992092
ss86297744		6	42.489221	0.7536	0.3779	0.538937		0.0127	0.0047129657
ss61557071	*rs41651272*	6	42.512115	0.2973	0.2450	0.620767		0.0097	0.0040699093
ss61523662	*rs41622325*	6	42.555613	0.3617	4.6913	0.030654		0.0407	0.0188044193
ss61557063	*rs41651264*	6	42.597176	0.3617	4.6913	0.030654		0.0407	0.0188044193
ss61469914	*rs41573706*	6	42.786668	0.3718	1.3839	0.239840		0.0225	0.0104966552
	rs29014966	6	42.810749	0.3080	0.0161	0.899041		0.0025	0.0010765462
ss64333867	*rs42590100*	6	42.838772	0.8825	2.7576	0.097244		0.0476	0.0098743867
ss64333941	*rs42591335*	6	42.89248	0.0516	0.0310	0.860247		0.0075	0.0007357460
ss64333957	*rs42591343*	6	42.923659	0.1870	1.3012	0.254394		0.0265	0.0080542488
	rs29027897	6	42.944536	0.4312	5.2542	0.022192		0.0418	0.0205220014
ss61564085	*rs43746588*	6	42.980062	0.7830	3.3047	0.069513		0.0407	0.0138463363
ss61529414	*rs41625135*	6	43.071077	0.8073	0.8193	0.365697		0.0213	0.0066372708
ss65376528	*rs43464589*	6	43.09726	0.1519	0.3559	0.551006		0.0152	0.0039283873
ss86308379		6	43.163442	0.6132	4.3915	0.036478		0.0406	0.0192635057
ss117968869		6	43.184224	0.5387	1.2627	0.261527		0.0213	0.0105926953
ss86329942		6	43.220732	0.8632	0.0540	0.816252		0.0064	0.0015116862
ss86274997		6	43.25361	0.4355	2.8884	0.089666		0.0329	0.0161974630
ss62756028	*rs41983306*	6	43.296256	0.6125	1.2214	0.269473		0.0209	0.0099408042
ss86311376		6	43.378455	0.1554	0.0534	0.817362		0.0059	0.0015601324
ss117968811		6	43.422442	0.6870	4.4339	0.035590		0.0418	0.0179811411
ss86289558		6	43.46417	0.2034	0.8403	0.359633		0.0214	0.0069327463
ss61491285	*rs41595981*	6	43.522483	0.2228	4.0504	0.044545		0.0458	0.0158444228
ss65376632	*rs43465493*	6	43.561116	0.7844	3.0781	0.079795		0.0400	0.0135377701
ss65376628	*rs43465489*	6	43.566157	0.5530	0.1755	0.675393		0.0081	0.0040029865
ss64909694	*rs43002212*	6	43.660167	0.8073	6.4736	0.011164		0.0595	0.0185012603
ss65106873	*rs43197793*	6	43.78515	0.3403	3.0229	0.082537		0.0341	0.0152944607

ss86327593		6	43.805236	0.8109	7.2885	0.007108	0.0636	0.0195092810
ss65376574	*rs43465435*	6	43.854917	0.0530	0.0323	0.857469	0.0075	0.0007503543
ss64990453	*rs43081151*	6	44.000279	0.0709	0.2852	0.593507	0.0197	0.0025943044
ss117968470		6	44.190262	0.1605	1.1117	0.292089	0.0270	0.0072832480
ss117968307		6	44.222974	0.4842	1.9622	0.161726	0.0257	0.0128398462
ss117968271		6	44.248876	0.4685	5.3172	0.021409	0.0423	0.0210622540
ss117968788		6	44.303954	0.6318	0.2924	0.588838	0.0104	0.0048190788
ss117968310		6	44.3272	0.6203	0.0120	0.912814	0.0021	0.0009943608
ss61568611	*rs41665299*	6	44.347309	0.3739	0.7186	0.396887	0.0165	0.0077447641
	rs29021261	6	44.43364	0.5344	0.5140	0.473636	0.0132	0.0065864899
ss117968464		6	44.503123	0.3868	0.5304	0.466676	0.0137	0.0065006324
ss86338064		6	44.530078	0.6662	0.0248	0.874871	0.0031	0.0013656517
ss61557106	*rs41651305*	6	44.553263	0.8410	4.5324	0.033609	0.0523	0.0139897654
ss117968423		6	44.579097	0.3818	21.0442	0.000005	0.0859	0.0405491792
ss117968426		6	44.611252	0.5287	19.0649	0.000015	0.0786	0.0391592046
ss117968537		6	44.643941	0.5272	18.6579	0.000018	0.0780	0.0388604961
ss117968613		6	44.67059	0.7851	1.3803	0.240446	0.0265	0.0089390466
ss117968302		6	44.699342	0.5781	2.2351	0.135365	0.0283	0.0138026920
ss117968505		6	44.729117	0.5752	2.6906	0.101396	0.0309	0.0151043448
ss117968703		6	44.772186	0.5530	0.0293	0.864088	0.0033	0.0016096698
ss117968186		6	44.80441	0.4642	1.9311	0.165082	0.0264	0.0131190312
ss117968089		6	44.84632	0.8030	2.1005	0.147702	0.0336	0.0106361917
ss117967988		6	44.870028	0.1383	0.8136	0.367382	0.0243	0.0057963442
ss65377757	*rs43458005*	6	44.89589	0.2099	1.4545	0.228225	0.0273	0.0090458624
ss117968568		6	44.930625	0.4198	0.6947	0.404839	0.0157	0.0076678121
ss117968571		6	44.952607	0.3603	17.7919	0.000028	0.0776	0.0357832582
ss61469116	*rs41567027*	6	45.009371	0.5630	3.9420	0.047486	0.0373	0.0183579907
ss117968531		6	45.031341	0.5573	4.2975	0.038535	0.0388	0.0191634359
ss117968671		6	45.072465	0.4893	21.1136	0.000005	0.0841	0.0420463746
ss117968218		6	45.109597	0.7128	0.0013	0.971502	0.0007	0.0002947820
ss117968646		6	45.157066	0.5874	0.6723	0.412524	0.0156	0.0075629386
ss117968591		6	45.238022	0.5330	0.6035	0.437513	0.0143	0.0071437214
ss86297961		6	45.267007	0.9420	0.5057	0.477248	0.0288	0.0031533193
ss86341086		6	45.334395	0.3668	0.1487	0.699858	0.0074	0.0034488299
ss61469117	*rs41567028*	6	45.354708	0.7242	8.6907	0.003306	0.0611	0.0244059780
ss117968332		6	45.377549	0.8166	4.9284	0.026741	0.0526	0.0157451120
ss86302342		6	45.423391	0.7557	3.6628	0.056049	0.0407	0.0150081342
ss61557145	*rs41652044*	6	45.45713	0.2221	3.1641	0.075711	0.0395	0.0136307801
ss86296135		6	45.606123	0.0415	7.6409	0.005857	0.1248	0.0099405334
ss86302250		6	45.627477	0.7106	0.2575	0.612021	0.0111	0.0045751433
ss86278563		6	45.64979	0.0709	0.8247	0.364125	0.0338	0.0044564333
ss86329802		6	45.722686	0.8381	4.1935	0.040954	0.0539	0.0146328676
ss61475106	*rs41571497*	6	45.758668	0.2256	1.5065	0.220092	0.0290	0.0101237968
	rs29023972	6	45.896642	0.2923	5.1190	0.023972	0.0481	0.0199011059
ss61491317	*rs41596013*	6	45.960115	0.5903	3.4174	0.064936	0.0344	0.0166620800
ss61557144	*rs41652043*	6	45.986604	0.8546	0.0779	0.780223	0.0076	0.0018781703
	rs29025709	6	46.068198	0.8868	0.0215	0.883465	0.0043	0.0008543577

ss86300829		6	46.232007	0.8775	0.0704	0.790891	0.0074	0.0015891519
ss117968832		6	46.289362	0.3410	0.5361	0.464302	0.0143	0.0064114905
ss86338660		6	46.340972	0.3052	3.0395	0.081705	0.0350	0.0148402014
ss86323939		6	46.364204	0.1977	0.2116	0.645640	0.0103	0.0032525694
ss86277999		6	46.429904	0.5122	0.0225	0.880773	0.0027	0.0013483395
ss86307779		6	46.452282	0.3861	2.1129	0.146510	0.0276	0.0130798072
ss61557143	*rs41652042*	6	46.473849	0.1268	0.0002	0.987762	0.0004	0.0000925614
ss46526804	*rs41257255*	6	46.522537	0.2149	2.3239	0.127857	0.0330	0.0111321333
ss86325372		6	46.560134	0.3066	0.6153	0.433055	0.0157	0.0066872432
ss86297594		6	46.59165	0.4685	0.4703	0.493062	0.0126	0.0062870415
ss117968827		6	46.63954	0.8911	2.0985	0.147891	0.0435	0.0084345494
ss61557152	*rs41652051*	6	46.920843	0.5021	0.0065	0.935592	0.0015	0.0007643438
ss86290125		6	46.968138	0.7586	3.1060	0.078443	0.0378	0.0138272479
ss61523664		6	47.086301	0.8911	0.3030	0.582160	0.0163	0.0031654601
ss117968808		6	47.21071	0.2579	0.3188	0.572502	0.0116	0.0044233892
ss61557155	*rs41652054*	6	47.261192	0.1862	1.4704	0.225700	0.0285	0.0086320076
ss86287215		6	47.292122	0.2428	0.0481	0.826549	0.0047	0.0017457885
ss86289686		6	47.613909	0.8381	4.4115	0.036056	0.0530	0.0143899508
ss86327315		6	47.659956	0.4105	0.6896	0.406594	0.0157	0.0075751479
ss86294338		6	47.682628	0.9441	0.3446	0.557400	0.0239	0.0025170709
ss86282978		6	47.736612	0.5695	6.5492	0.010704	0.0474	0.0232561949
	rs29016265	6	47.812158	0.7529	0.0120	0.912848	0.0024	0.0008843880
ss65378394	*rs43466935*	6	47.853056	0.1211	11.5938	0.000700	0.0925	0.0196887794
ss86331827		6	48.044634	0.4577	3.4372	0.064167	0.0348	0.0172943442
ss117968825		6	48.08501	0.2708	12.4131	0.000454	0.0734	0.0289988536
ss86338040		6	48.130754	0.3360	23.6963	0.000001	0.0957	0.0427142166
ss61491324	*rs41596020*	6	48.249828	0.0960	0.5822	0.445699	0.0233	0.0040482308
ss86288726		6	48.302379	0.1440	1.7933	0.180964	0.0344	0.0084778007
ss64231486	*rs42543116*	6	48.334787	0.2020	0.3813	0.537111	0.0144	0.0046320827
	rs29018489	6	48.404933	0.8446	0.0096	0.921976	0.0025	0.0006494275
ss86300060		6	48.491443	0.6203	2.0842	0.149279	0.0272	0.0127966692
ss65379092	*rs43462529*	6	48.528614	0.3754	3.4440	0.063906	0.0346	0.0162246780
ss86306693		6	48.562604	0.2887	10.1493	0.001508	0.0646	0.0265492417
ss65378762	*rs43459400*	6	48.615303	0.0344	0.0542	0.815912	0.0119	0.0007916396
ss86304580		6	48.639805	0.4348	3.0918	0.079129	0.0322	0.0158491061
	rs29010402	6	48.676373	0.3030	10.0659	0.001577	0.0643	0.0271797139
ss86310957		6	48.715278	0.7471	14.6301	0.000143	0.0811	0.0306282987
ss86308904		6	48.747117	0.4957	6.6980	0.009854	0.0483	0.0241471186
ss64201500	*rs42520768*	6	48.782733	0.3424	1.1095	0.292545	0.0205	0.0092122639
ss64201584	*rs42520810*	6	48.80495	0.8797	1.4485	0.229170	0.0343	0.0072665402
		6	48.862294	0.8732	3.3380	0.068127	0.0500	0.0110628854
ss61519141	*rs41611194*	6	48.863981	0.5580	0.0939	0.759401	0.0056	0.0027539499
ss64705887	*rs42806106*	6	48.928335	0.9398	0.3546	0.551724	0.0235	0.0026553272
ss65018090	*rs43106284*	6	49.009713	0.6096	5.3410	0.021121	0.0432	0.0205546503
ss64453996	*rs42649586*	6	49.080812	0.0766	0.0868	0.768320	0.0105	0.0014899404
ss64453764	*rs42648470*	6	49.108244	0.5265	0.9373	0.333299	0.0182	0.0090672459
ss64453728	*rs42648452*	6	49.14039	0.0638	1.2585	0.262321	0.0437	0.0052224343

ss64863410	*rs42955509*	6	49.17671	0.6648	13.4785	0.000260		0.0717	0.0319599490
ss64887234	*rs42978749*	6	49.217287	0.3374	11.7072	0.000659		0.0667	0.0298410261
ss61466718	*rs41572270*	6	49.244601	0.3345	13.5677	0.000248		0.0719	0.0320132302
ss65379274	*rs43464811*	6	49.290357	0.3567	19.9521	0.000009		0.0856	0.0392722162
ss65379326	*rs43465963*	6	49.313358	0.6418	15.9232	0.000073		0.0766	0.0352062121
ss38330383	*rs29021889*	6	49.334858	0.3725	15.1510	0.000109		0.0732	0.0342211147
ss65379383	*rs43466020*	6	49.361008	0.6268	17.9588	0.000026		0.0794	0.0371370740
ss61557208	*rs41652103*	6	49.435978	0.3066	0.7341	0.391857		0.0172	0.0073201111
ss65379628	*rs43468063*	6	49.456373	0.2364	8.7002	0.003289		0.0636	0.0229719486
ss65037955	*rs43131320*	6	49.476443	0.8847	5.3947	0.020486		0.0679	0.0138469161
ss64660946	*rs42756089*	6	49.51862	0.1239	2.4002	0.121777		0.0435	0.0094353889
ss65379921	*rs43461550*	6	49.552577	0.6734	3.0644	0.080466		0.0344	0.0151357445
ss86276092		6	49.581933	0.0430	14.1329	0.000185		0.1724	0.0141830772
ss65379852	*rs43460781*	6	49.611109	0.9169	1.4199	0.233827		0.0404	0.0061574154
ss86288663		6	49.814265	0.2056	0.3380	0.561153		0.0133	0.0043392540
ss61506758	*rs41608253*	6	49.972459	0.6447	2.0547	0.152183		0.0286	0.0131104152
ss61526408	*rs41623102*	6	50.117544	0.3066	1.7238	0.189641		0.0269	0.0114380468
ss61476479	*rs41576837*	6	50.149884	0.7500	1.2333	0.267151		0.0241	0.0090428277
	rs29023010	6	50.201212	0.9556	0.0123	0.911788		0.0051	0.0004292791
ss61557213		6	50.259955	0.8367	0.6495	0.420579		0.0203	0.0055448323
ss65380149	*rs43462767*	6	50.291769	0.3503	1.6818	0.195111		0.0254	0.0115405366
ss61557280	*rs41652872*	6	50.331503	0.1633	0.3470	0.556011		0.0151	0.0041164685
ss65380397	*rs43464313*	6	50.375516	0.2636	0.6610	0.416484		0.0164	0.0063494414
ss61469497	*rs41570399*	6	50.416045	0.8639	4.6721	0.030996		0.0572	0.0134563345
ss64836600	*rs42934850*	6	50.549254	0.3804	3.1129	0.078113		0.0320	0.0150855519
ss86320446		6	50.645903	0.6067	8.6478	0.003383		0.0551	0.0262732750
ss64162597	*rs42507629*	6	50.691754	0.2880	5.1426	0.023651		0.0465	0.0190695868
ss63801452	*rs42360066*	6	50.737861	0.1289	2.5961	0.107582		0.0444	0.0099632809
ss63801610	*rs42360543*	6	50.774091	0.6605	1.1223	0.289786		0.0202	0.0090722142
ss63801648	*rs42360562*	6	50.824702	0.3130	1.1219	0.289887		0.0208	0.0089250695
ss65017280	*rs43107549*	6	50.860417	0.4019	0.6135	0.433740		0.0148	0.0071304464
ss65380627	*rs43466343*	6	50.886857	0.1655	2.7345	0.098651		0.0414	0.0114270567
ss61500596	*rs41599346*	6	50.980972	0.3288	0.7451	0.388322		0.0165	0.0072649403
ss64105745	*rs42479882*	6	51.007155	0.4040	0.0375	0.846500		0.0036	0.0017296008
ss65091101	*rs43180057*	6	51.063101	0.0372	0.4714	0.492560		0.0339	0.0024304746
ss64435666	*rs42639714*	6	51.089277	0.4083	0.0056	0.940590		0.0014	0.0006762362
ss64435578	*rs42639670*	6	51.147563	0.6182	0.0950	0.758021		0.0058	0.0027232277
ss86337163		6	51.260255	0.5809	3.5168	0.061168		0.0360	0.0175211336
ss86325408		6	51.341956	0.7049	3.3365	0.068186		0.0368	0.0152950225
ss61522788	*rs41622057*	6	51.4067	0.1726	0.6308	0.427316		0.0185	0.0052944079
ss61469821	*rs41572815*	6	51.436623	0.8295	0.7615	0.383173		0.0203	0.0057448938
ss64289964	*rs42569499*	6	51.458541	0.2987	0.0208	0.885337		0.0030	0.0012397155
ss61465496	*rs41566743*	6	51.53214	0.7887	0.0102	0.919528		0.0023	0.0007771466
ss64505680	*rs42675214*	6	51.567323	0.1877	4.4210	0.035859		0.0500	0.0152502721
ss61513719	*rs41614983*	6	51.603449	0.5415	2.2099	0.137576		0.0279	0.0138779699
ss64299838	*rs42577887*	6	51.648197	0.3768	1.7447	0.186978		0.0260	0.0122294872
ss38333670	*rs29017377*	6	51.685675	0.3660	0.3393	0.560404		0.0112	0.0052104238

ss64812117	*rs42905978*	6	51.709444	0.2321	10.6135	0.001177		0.0713	0.0254282234
ss86326791		6	51.749548	0.8331	7.1389	0.007719		0.0672	0.0186974841
ss61530518	*rs41625390*	6	51.772055	0.8109	6.9359	0.008636		0.0632	0.0193707832
ss65600391	*rs43679404*	6	51.811956	0.3990	0.8515	0.356460		0.0174	0.0083286696
ss61512613	*rs41613817*	6	51.851114	0.8503	7.4830	0.006387		0.0717	0.0182662312
ss65600245	*rs43678158*	6	51.889391	0.7615	3.1698	0.075448		0.0383	0.0139048924
ss64089433	*rs42473408*	6	51.958782	0.4234	3.7083	0.054549		0.0367	0.0179374005
ss61530517	*rs41625389*	6	52.027976	0.3861	1.8799	0.170783		0.0262	0.0124305054
ss61472527	*rs41571552*	6	52.059851	0.6898	3.4638	0.063147		0.0373	0.0159444982
ss61518152	*rs41611321*	6	52.094032	0.3095	3.3163	0.069027		0.0364	0.0155777897
ss86323714		6	52.116479	0.2543	0.2025	0.652883		0.0098	0.0037180840
ss65022557	*rs43115884*	6	52.169453	0.6913	3.2174	0.073293		0.0360	0.0153738712
ss61526209	*rs41625791*	6	52.247529	0.7092	0.0899	0.764380		0.0063	0.0026061048
ss61466543	*rs41570296*	6	52.27878	0.6913	3.2174	0.073293		0.0360	0.0153738712
ss64616820	*rs42725330*	6	52.30301	0.1870	8.0127	0.004779		0.0660	0.0200591307
ss117963901		6	52.326213	0.7622	3.2646	0.071221		0.0397	0.0143784235
ss117967979		6	52.438316	0.3603	0.0225	0.880913		0.0029	0.0013383468
ss61557285	*rs41652877*	6	52.461052	0.6404	0.0413	0.838928		0.0039	0.0018130815
ss117968335		6	52.482067	0.1999	0.4777	0.489712		0.0162	0.0051917684
ss61491355	*rs41597149*	6	52.526151	0.4756	7.3349	0.006929		0.0493	0.0245949615
ss65381048	*rs43461963*	6	52.546396	0.4756	7.3349	0.006929		0.0493	0.0245949615
ss117968401		6	52.57313	0.8438	6.2128	0.012914		0.0649	0.0170992510
ss65381027	*rs43461942*	6	52.602521	0.3890	1.4658	0.226427		0.0231	0.0110003940
ss86324488		6	52.640931	0.2357	0.7498	0.386846		0.0189	0.0068170111
ss65380987	*rs43461502*	6	52.671075	0.4857	3.7150	0.054333		0.0352	0.0176022612
ss117968033		6	52.708577	0.3990	1.1035	0.293861		0.0191	0.0091725519
ss117968467		6	52.743693	0.5766	6.5621	0.010627		0.0501	0.0244842189
	rs29022511	6	52.77627	0.4606	1.6372	0.201138		0.0248	0.0123266141
ss117968221		6	52.811751	0.6046	0.5498	0.458636		0.0140	0.0066972097
ss117968168		6	52.848991	0.3847	0.5204	0.470921		0.0138	0.0065271992
ss65380680	*rs43466396*	6	52.876816	0.8259	0.5973	0.439868		0.0194	0.0055726749
ss117968847		6	52.912652	0.3517	0.6936	0.405217		0.0160	0.0073038989
ss117968582		6	52.975496	0.5831	1.3059	0.253535		0.0211	0.0102487402
ss117968391		6	53.000926	0.6605	0.0043	0.947634		0.0013	0.0005732771
ss117968099		6	53.026137	0.4341	0.6003	0.438722		0.0141	0.0069517772
ss117968449		6	53.104687	0.5831	1.3059	0.253535		0.0211	0.0102487402
ss86337063		6	53.162427	0.6096	0.6331	0.426491		0.0147	0.0070013991
ss64861966	*rs42957282*	6	53.224549	0.5831	1.3059	0.253535		0.0211	0.0102487402
ss117968786		6	53.279489	0.6705	2.0443	0.153225		0.0282	0.0124804845
ss117968622		6	53.329684	0.9327	14.8541	0.000127		0.1453	0.0182518091
ss117968443		6	53.385027	0.9205	0.7742	0.379235		0.0303	0.0044413932
ss117968045		6	53.445429	0.7464	2.6438	0.104404		0.0354	0.0134124236
ss117967961		6	53.60597	0.4506	0.5005	0.479515		0.0129	0.0063761846
		6	53.64678	0.4534	0.6875	0.407316		0.0151	0.0074925209
ss61491363	*rs41597156*	6	53.671572	0.4620	0.0176	0.894431		0.0024	0.0012178750
ss61557291	*rs41652883*	6	53.719949	0.4764	0.8734	0.350347		0.0169	0.0084271634
ss65381296	*rs43463508*	6	53.747773	0.1461	7.9579	0.004924		0.0732	0.0182555489

ss86291414		6	53.852963	0.9298	1.1064	0.293222	0.0380	0.0049630018
ss117968403		6	53.873257	0.4692	0.3731	0.541509	0.0115	0.0057492675
ss117968189		6	53.898894	0.4692	0.1203	0.728763	0.0064	0.0031882409
ss117968649		6	53.921384	0.4642	0.3181	0.572954	0.0105	0.0052020761
ss117967999		6	53.943356	0.4140	0.5791	0.446931	0.0143	0.0069374143
ss61557299	*rs41652891*	6	54.085643	0.4821	2.1508	0.142947	0.0276	0.0137630371
ss117968486		6	54.118748	0.3087	0.0098	0.920972	0.0020	0.0008388507
ss117968287		6	54.156739	0.5544	0.0263	0.871202	0.0030	0.0014874275
ss86315144		6	54.227934	0.2901	2.1517	0.142865	0.0308	0.0126730126
ss61563086	*rs41658480*	6	54.32847	0.3123	0.7031	0.402017	0.0172	0.0073708329
ss61563081	*rs41658475*	6	54.354528	0.3489	0.3335	0.563766	0.0110	0.0049986194
ss117967967		6	54.415176	0.5530	0.2837	0.594434	0.0098	0.0048411672
ss117968350		6	54.459621	0.6375	2.5462	0.111016	0.0304	0.0140576819
ss86307850		6	54.503529	0.5738	7.3469	0.006883	0.0510	0.0249536350
ss117968268		6	54.526955	0.7156	4.6194	0.031956	0.0426	0.0173210739
ss117968411		6	54.550438	0.6884	8.1320	0.004478	0.0555	0.0238077418
ss86335627		6	54.576778	0.5874	5.7630	0.016629	0.0450	0.0218354373
ss63811949	*rs42361198*	6	54.612482	0.2958	6.1701	0.013227	0.0489	0.0203555416
ss61517235	*rs41612629*	6	54.643464	0.2973	6.1526	0.013357	0.0488	0.0203952452
ss61517232	*rs41612626*	6	54.69901	0.0731	4.5079	0.034090	0.0746	0.0101096489
ss63811379	*rs42365512*	6	54.741249	0.2858	4.5524	0.033222	0.0420	0.0171435924
ss63811335	*rs42365490*	6	54.78804	0.5143	1.7000	0.192722	0.0272	0.0135842522
ss65049963	*rs43138420*	6	54.918599	0.0867	6.6456	0.010145	0.0833	0.0131922005
ss86333613		6	54.944965	0.7049	1.7398	0.187602	0.0271	0.0112673015
ss86340690		6	55.009766	0.3818	0.0029	0.956814	0.0010	0.0004776842
ss86338115		6	55.038931	0.6182	0.0029	0.956814	0.0010	0.0004776842
ss86297484		6	55.121391	0.3617	1.7872	0.181700	0.0270	0.0124476830
ss62812593	*rs42007479*	6	55.167283	0.3582	0.0445	0.832988	0.0040	0.0018600028
ss62812482	*rs42007442*	6	55.196036	0.3911	0.2935	0.588146	0.0101	0.0047929907
ss61545179	*rs41641533*	6	55.222361	0.6125	0.2890	0.591030	0.0099	0.0046927079
ss62811828	*rs42005124*	6	55.24305	0.3875	0.2890	0.591030	0.0099	0.0046927079
ss62811663	*rs42005069*	6	55.266546	0.5924	0.3123	0.576426	0.0105	0.0050704307
ss62811720	*rs42005088*	6	55.293701	0.1053	2.7644	0.096833	0.0497	0.0093676117
ss62810964	*rs42003736*	6	55.353385	0.3574	1.2101	0.271690	0.0210	0.0096516738
ss61485542		6	55.385887	0.6426	1.2101	0.271690	0.0210	0.0096516738
ss62810610	*rs42002518*	6	55.410621	0.6426	1.2101	0.271690	0.0210	0.0096516738
ss62810337	*rs42001829*	6	55.476904	0.7822	8.6183	0.003438	0.0643	0.0219036566
ss86314157		6	55.506268	0.3574	0.2289	0.632499	0.0094	0.0043093684
ss61568904	*rs41667285*	6	55.581785	0.6698	2.9387	0.086928	0.0344	0.0152332187
ss64384633	*rs42608971*	6	55.61567	0.3188	2.5277	0.112321	0.0320	0.0138952300
ss64384617	*rs42608963*	6	55.642282	0.0466	0.9620	0.327025	0.0431	0.0038237584
ss86309304		6	55.664412	0.5716	0.0081	0.928503	0.0016	0.0007948842
ss64240580	*rs42542335*	6	55.716232	0.7443	3.9824	0.046367	0.0420	0.0159990003
ss64240570	*rs42541530*	6	55.738132	0.7822	2.8955	0.089272	0.0379	0.0128997238
ss117968131		6	55.765188	0.7844	1.7374	0.187900	0.0291	0.0098287661
ss61495379	*rs41596755*	6	55.857696	0.2579	0.1446	0.703852	0.0083	0.0031678873
ss86324290		6	55.916486	0.6569	1.0020	0.317169	0.0199	0.0089836976

ss61567211	*rs41660728*	6	56.002136	0.6203	0.0099	0.920783		0.0019	0.0008927498
ss61469844		6	56.038249	0.2708	2.0367	0.153994		0.0304	0.0120075717
ss38322443	*rs29013548*	6	56.074791	0.1096	1.9069	0.167747		0.0404	0.0078905741
ss86328867		6	56.434873	0.0845	2.2479	0.134245		0.0500	0.0077424769
ss63616365	*rs42294009*	6	56.471463	0.5287	4.0102	0.045612		0.0371	0.0184925283
ss117968860		6	56.495043	0.2779	0.0077	0.930030		0.0018	0.0007395688
ss86300164		6	56.53701	0.5265	2.2599	0.133215		0.0279	0.0139232261
ss63615702	*rs42291290*	6	56.595391	0.9155	0.2860	0.592997		0.0172	0.0026576681
ss63615666	*rs42291278*	6	56.634157	0.7221	0.0077	0.930030		0.0018	0.0007395688
ss61567212		6	56.662125	0.6934	0.3140	0.575432		0.0113	0.0047983853
ss86320950		6	56.711278	0.5946	2.3418	0.126395		0.0288	0.0138744833
ss63614775	*rs42287981*	6	56.736868	0.6289	0.8458	0.358050		0.0182	0.0084855887
ss65381971	*rs43462578*	6	56.781359	0.7156	0.0090	0.924617		0.0019	0.0007858572
ss65381935	*rs43462543*	6	57.062313	0.5795	0.2976	0.585582		0.0106	0.0051776629
ss64674062	*rs42771950*	6	57.116272	0.2371	3.4541	0.063517		0.0401	0.0144913265
ss61465841	*rs41569985*	6	57.139918	0.8360	0.4807	0.488338		0.0176	0.0048255433
ss65381809	*rs43461417*	6	57.16125	0.8395	1.7951	0.180746		0.0333	0.0089663238
ss64304840	*rs42573191*	6	57.187298	0.5050	0.3309	0.565337		0.0108	0.0054047825
ss61935809	*rs41700040*	6	57.308678	0.2056	1.3803	0.240456		0.0270	0.0088118909
ss117968829		6	57.337062	0.7923	0.3359	0.562395		0.0137	0.0045014809
ss61508128	*rs41601515*	6	57.392471	0.7830	0.3911	0.531933		0.0139	0.0047366250
ss61500470	*rs41598922*	6	57.416735	0.8417	1.9499	0.163045		0.0359	0.0095656037
ss86340139		6	57.495853	0.0616	7.8244	0.005297		0.1086	0.0125554843
ss61499857	*rs41596235*	6	57.548761	0.8553	0.5670	0.451691		0.0203	0.0050183761
ss86337900		6	57.612503	0.6698	0.0052	0.942549		0.0014	0.0006274940
ss86337613		6	57.680752	0.2020	0.7781	0.378036		0.0202	0.0065170551
	rs29021880	6	57.763159	0.3195	0.1913	0.661942		0.0087	0.0038003546
ss61526168	*rs41625756*	6	57.806017	0.6110	0.0799	0.777484		0.0054	0.0025862992
ss86291446		6	58.528972	0.4405	0.1470	0.701503		0.0071	0.0035112347
ss86324544		6	58.573303	0.0645	0.0205	0.886089		0.0055	0.0006622959
ss86277926		6	58.85595	0.2808	1.9649	0.161434		0.0290	0.0116961408
ss61557327	*rs41652918*	6	58.902629	0.1046	4.6383	0.031608		0.0655	0.0122712356
	rs29017197	6	58.924722	0.1046	4.6383	0.031608		0.0655	0.0122712356
ss61527769	*rs41626012*	6	58.966652	0.9463	2.6747	0.102405		0.0666	0.0067739822
ss86323889		6	58.998182	0.0774	3.2016	0.074002		0.0622	0.0088864059
ss86310794		6	59.055715	0.1662	0.2833	0.594694		0.0131	0.0036333242
ss61469141	*rs41567752*	6	59.11841	0.3503	0.5081	0.476185		0.0138	0.0062854621
ss86306339		6	59.221344	0.7837	0.0212	0.884406		0.0034	0.0011446405
ss86289991		6	59.335774	0.2443	2.4214	0.120142		0.0331	0.0122105526
ss86323574		6	59.39777	0.4534	0.0349	0.851830		0.0036	0.0017811456
ss117963875		6	59.524518	0.5595	0.0317	0.858717		0.0033	0.0016467805
ss117963894		6	59.547953	0.8832	1.6393	0.200845		0.0368	0.0075836914
ss86315755		6	59.730341	0.0616	0.2041	0.651560		0.0174	0.0020150526
	rs29026792	6	59.753787	0.3424	0.0914	0.762452		0.0059	0.0026617501
	rs29012688	6	59.787613	0.3417	0.0632	0.801572		0.0049	0.0022164200
ss61557376	*rs41653366*	6	59.860619	0.4506	2.1509	0.142938		0.0270	0.0133489496
	rs29014106	6	59.971641	0.0487	4.0951	0.043390		0.0856	0.0079325639

ss86330417		6	60.148298	0.1089	0.0055	0.940906	0.0022	0.0004234917
ss86316701		6	60.235599	0.4054	0.0414	0.838833	0.0038	0.0018208739
ss86302048		6	60.288902	0.7357	5.2018	0.022865	0.0479	0.0186325586
ss86299014		6	60.343228	0.4069	0.0721	0.788345	0.0050	0.0023954590
ss86307272		6	60.382591	0.5279	1.4183	0.234090	0.0221	0.0110380844
ss117968201		6	60.452406	0.6934	0.1872	0.665427	0.0091	0.0038640858
ss61568843	*rs41666725*	6	60.548448	0.4355	4.4315	0.035638	0.0394	0.0193913382
	rs29025767	6	60.652062	0.6769	0.7356	0.391383	0.0179	0.0078496066
ss61496943	*rs41593582*	6	60.704108	0.7450	4.4806	0.034636	0.0456	0.0173077729
	rs29017690	6	60.904765	0.1103	8.2089	0.004294	0.0829	0.0162686368
ss86330465		6	60.926607	0.1461	2.6199	0.105983	0.0432	0.0107690865
ss61491392	*rs41597184*	6	60.950073	0.8503	5.9260	0.015170	0.0640	0.0162912958
ss117963873		6	60.982162	0.2486	7.9935	0.004829	0.0614	0.0229342654
ss86287043		6	61.019162	0.8603	11.8720	0.000604	0.0935	0.0224745741
ss86332364		6	61.049599	0.7744	0.7670	0.381457	0.0201	0.0070221649
ss117963877		6	61.127332	0.6547	4.7328	0.029929	0.0421	0.0190183635
ss61539393	*rs41638945*	6	61.163886	0.7815	17.0030	0.000042	0.0915	0.0312412406
ss86293495		6	61.252857	0.8696	3.9235	0.048010	0.0553	0.0125494650
ss61532738	*rs41626829*	6	61.38245	0.7557	14.7957	0.000131	0.0810	0.0299041798
ss61479508	*rs41579471*	6	61.422622	0.4506	3.8276	0.050814	0.0371	0.0183545440
ss86312262		6	61.506135	0.2543	1.3601	0.243920	0.0248	0.0094094523
ss61557433	*rs41653422*	6	61.532963	0.0981	2.4687	0.116588	0.0483	0.0085550036
ss61491409	*rs41597201*	6	61.569919	0.8926	1.8951	0.169069	0.0411	0.0078864127
ss86295907		6	61.65002	0.1605	0.4835	0.487085	0.0174	0.0046801614
ss117968134		6	61.673987	0.1655	1.9367	0.164474	0.0343	0.0094798106
ss61510612	*rs41605935*	6	61.715697	0.7077	0.6675	0.414194	0.0167	0.0069165434
ss86328839		6	61.8022	0.3173	20.2208	0.000008	0.0872	0.0377635187
ss86333561		6	61.917425	0.1977	8.0254	0.004746	0.0675	0.0214141243
ss86287153		6	61.938806	0.7944	4.5479	0.033309	0.0498	0.0162653224
ss86293581		6	61.967076	0.6805	2.3924	0.122383	0.0310	0.0134829639
ss86336677		6	61.999923	0.6769	8.9942	0.002805	0.0587	0.0256932404
ss61510613	*rs41605936*	6	62.02971	0.9384	0.0337	0.854487	0.0071	0.0008180958
ss61557423	*rs41653413*	6	62.107296	0.7679	3.5818	0.058832	0.0410	0.0145982209
ss61557427	*rs41653417*	6	62.149881	0.7221	0.3023	0.582605	0.0110	0.0044339678
ss86323742		6	62.196909	0.8632	0.3497	0.554463	0.0163	0.0038418815
ss61489290		6	62.234812	0.4656	4.8338	0.028235	0.0412	0.0204958188
ss61553099	*rs43709351*	6	62.265819	0.2550	3.1229	0.077638	0.0388	0.0147554985
ss86319473		6	62.298184	0.3223	0.7140	0.398405	0.0165	0.0072007388
ss61491406	*rs41597198*	6	62.334031	0.1977	0.0111	0.916213	0.0025	0.0007798314
ss65382862	*rs43465268*	6	62.357058	0.1827	0.5618	0.453785	0.0181	0.0054130489
ss65382851	*rs43465257*	6	62.383394	0.7414	0.0026	0.959036	0.0011	0.0004301962
ss86292489		6	62.405609	0.0544	0.0141	0.905551	0.0050	0.0005101768
ss64548092	*rs42697154*	6	62.444984	0.8825	0.0475	0.827551	0.0064	0.0013171312
ss86338152		6	62.475579	0.9226	2.3186	0.128285	0.0530	0.0075673654
ss61510564	*rs41605188*	6	62.552757	0.7887	0.5379	0.463561	0.0170	0.0056647285
ss86321723		6	62.582443	0.8696	0.7490	0.387094	0.0239	0.0054277631
ss65372409	*rs43458406*	6	62.662352	0.4377	0.2805	0.596561	0.0098	0.0048311534

ss61556986	*rs41650788*	6	62.70663	0.0795	4.3754	0.036824	0.0699	0.0102265142
ss61556990	*rs41650792*	6	62.74126	0.0974	3.6868	0.055253	0.0583	0.0102535778
ss61491394	*rs41597186*	6	62.774664	0.8904	3.7933	0.051859	0.0566	0.0110392000
ss61557392	*rs41653382*	6	62.805339	0.9183	4.0994	0.043281	0.0670	0.0100480098
ss86312831		6	62.876299	0.1225	1.9759	0.160273	0.0393	0.0084452167
ss86340687		6	62.962734	0.4097	2.7460	0.097947	0.0319	0.0154339163
ss86340697		6	62.985095	0.3324	3.7143	0.054355	0.0383	0.0169977700
ss86304490		6	63.053085	0.8961	1.2799	0.258312	0.0343	0.0063762866
ss65383131	*rs43468632*	6	63.164212	0.3173	0.0859	0.769598	0.0058	0.0025095640
ss65383223	*rs43468724*	6	63.186624	0.5093	10.1103	0.001540	0.0594	0.0296991061
ss65383240	*rs43470141*	6	63.208708	0.3288	0.2523	0.615599	0.0098	0.0043176897
ss65383390	*rs43471791*	6	63.305334	0.6662	0.1028	0.748579	0.0062	0.0027790224
ss65383325	*rs43470226*	6	63.474386	0.6612	0.0390	0.843475	0.0038	0.0017093659
ss86299880		6	63.70646	0.3854	0.0510	0.821361	0.0043	0.0020265511
ss86275273		6	63.76901	0.8087	5.4935	0.019368	0.0568	0.0175579126
ss86296387		6	63.850642	0.5179	1.0652	0.302384	0.0186	0.0092706072
ss86339960		6	63.8973	0.8438	0.2463	0.619850	0.0128	0.0033753055
ss86336240		6	63.928489	0.0738	2.6037	0.107071	0.0560	0.0076475731
ss63821688	*rs42373334*	6	63.995648	0.5115	1.1806	0.277615	0.0202	0.0100914647
ss86300351		6	64.02371	0.5838	0.0252	0.873842	0.0030	0.0014594367
ss63821542	*rs42372161*	6	64.048163	0.7980	1.1228	0.289686	0.0241	0.0077714398
ss64874289	*rs42971076*	6	64.090481	0.2213	0.0226	0.880617	0.0033	0.0011337800
ss63821466	*rs42371123*	6	64.178161	0.4864	1.7246	0.189540	0.0243	0.0121237773
ss117968199		6	64.205385	0.7880	1.1937	0.274970	0.0246	0.0082229853
ss86332947		6	64.260179	0.5172	1.4145	0.234709	0.0220	0.0109958134
ss86309430		6	64.385034	0.1261	0.1963	0.657839	0.0125	0.0027462886
ss86297476		6	64.451074	0.6053	1.2087	0.271965	0.0206	0.0098394047
ss117968565		6	64.741833	0.7235	5.9582	0.014897	0.0519	0.0207648763
ss61517727	*rs41618091*	6	64.84608	0.8044	1.6588	0.198190	0.0301	0.0094610365
ss61517731	*rs41618095*	6	64.888083	0.5573	1.2440	0.265089	0.0212	0.0104842983
ss61517730	*rs41618094*	6	64.910529	0.7471	0.3174	0.573368	0.0122	0.0046006722
ss86338631		6	64.948197	0.5136	4.6154	0.032031	0.0400	0.0199899514
ss61557444	*rs41653733*	6	65.061092	0.6246	2.7763	0.096118	0.0319	0.0149390680
ss61557443	*rs41653732*	6	65.128713	0.2665	9.1625	0.002561	0.0640	0.0250218073
ss61557435	*rs41653424*	6	65.162298	0.3138	7.2523	0.007251	0.0537	0.0231104859
ss61477128	*rs41574875*	6	65.401359	0.3231	4.7177	0.030191	0.0435	0.0190183230
ss64022907	*rs42434064*	6	65.596037	0.5731	1.1705	0.279675	0.0204	0.0100034030
ss61477123	*rs41574870*	6	65.644571	0.4262	1.0178	0.313396	0.0191	0.0093428832
ss61477115	*rs41574862*	6	65.672359	0.3854	0.0006	0.980995	0.0005	0.0002136362
ss64022197	*rs42437809*	6	65.708016	0.5974	0.2197	0.639407	0.0089	0.0042616513
ss61527727	*rs41625974*	6	65.739411	0.6203	0.0240	0.876834	0.0029	0.0013776497
ss64021769	*rs42436495*	6	65.770567	0.7779	0.0076	0.930772	0.0020	0.0006803301
ss64021743	*rs42436482*	6	65.792843	0.7572	0.1414	0.707041	0.0084	0.0030808792
ss61491418	*rs41597210*	6	65.86281	0.2858	2.4150	0.120634	0.0319	0.0130430558
ss65383490	*rs43472791*	6	65.905415	0.4305	10.1848	0.001480	0.0612	0.0300006847
ss61557451	*rs41653740*	6	65.939427	0.6297	0.0237	0.877821	0.0030	0.0013793105
ss65383745	*rs43465747*	6	65.978447	0.5107	0.0049	0.944043	0.0013	0.0006445070

ss65383791	*rs43465793*	6	66.013886	0.5093	0.0168	0.897018	0.0024	0.0011917238
ss86287174		6	66.116522	0.2521	0.0002	0.987605	0.0003	0.0001285874
ss64467204	*rs42651155*	6	66.150018	0.4470	3.8961	0.048794	0.0367	0.0181379372
ss86340008		6	66.199733	0.1597	0.0000	0.999095	0.0000	0.0000076135
ss86310521		6	66.239507	0.1196	0.7814	0.377023	0.0251	0.0052840132
ss117968162		6	66.300665	0.3059	0.9668	0.325823	0.0202	0.0085873865
ss64704665	*rs42803295*	6	66.383308	0.6504	5.2674	0.022025	0.0446	0.0202775328
ss86337401		6	66.459823	0.3152	5.3213	0.021359	0.0452	0.0195000247
ss65384253	*rs43469091*	6	66.481495	0.3682	1.0513	0.305570	0.0195	0.0090734445
ss65384260	*rs43469098*	6	66.525176	0.6132	0.2790	0.597557	0.0100	0.0047461373
ss86311041		6	66.567677	0.3847	0.3162	0.574054	0.0106	0.0050332602
ss65384370	*rs43470108*	6	66.599142	0.2564	7.7117	0.005634	0.0577	0.0220126882
ss86286151		6	66.660327	0.0473	2.4054	0.121374	0.0676	0.0060897575
ss65384466	*rs43471103*	6	66.713317	0.3668	1.2418	0.265514	0.0211	0.0098033659
ss61494012	*rs41592222*	6	66.760426	0.7486	6.9166	0.008729	0.0547	0.0205877275
ss64936351	*rs43025599*	6	66.809119	0.1841	0.2954	0.586931	0.0131	0.0039382121
ss64601184	*rs42717584*	6	66.934703	0.6246	3.0018	0.083616	0.0331	0.0155293815
ss64600976	*rs42716880*	6	66.961617	0.3403	0.9676	0.325615	0.0191	0.0085554042
ss64600954	*rs42716869*	6	66.988764	0.4477	3.4357	0.064225	0.0349	0.0172437834
	rs29015787	6	67.033353	0.8274	3.5012	0.061743	0.0452	0.0129075627
ss64122887	*rs42485661*	6	67.096535	0.2242	0.3301	0.565760	0.0131	0.0045579162
ss64122809	*rs42484822*	6	67.131293	0.1633	2.1377	0.144170	0.0375	0.0102540456
ss117968113		6	67.163032	0.6139	0.2209	0.638525	0.0089	0.0042384512
ss61524497	*rs41621536*	6	67.190574	0.5867	0.8531	0.356008	0.0173	0.0083990045
ss64122691	*rs42484763*	6	67.215576	0.1146	0.2555	0.613355	0.0149	0.0030160437
ss61496555	*rs41599002*	6	67.244199	0.7658	0.0025	0.960186	0.0011	0.0004079337
ss64346179	*rs42595906*	6	67.284943	0.6182	0.0070	0.933402	0.0016	0.0007587296
ss64122399	*rs42482917*	6	67.307791	0.1411	0.4002	0.527188	0.0172	0.0041784612
ss61531715	*rs41629267*	6	67.330002	0.5050	5.7151	0.017084	0.0445	0.0222566974
ss64122467	*rs42483651*	6	67.367771	0.8044	4.8709	0.027639	0.0511	0.0160840251
ss64122611	*rs42483723*	6	67.395438	0.4964	5.3766	0.020697	0.0434	0.0217155309
ss64346233	*rs42596633*	6	67.44573	0.4957	4.9831	0.025913	0.0419	0.0209421436
ss61496554	*rs41599001*	6	67.482658	0.5043	4.9831	0.025913	0.0419	0.0209421436
ss64730255	*rs42824023*	6	67.507939	0.7937	0.5061	0.477072	0.0159	0.0052133684
ss86292055		6	67.53534	0.1024	4.9935	0.025759	0.0662	0.0121698321
ss64957662	*rs43046920*	6	67.764816	0.7178	1.1087	0.292720	0.0222	0.0090125673
ss117967925		6	67.79032	0.2636	0.3693	0.543577	0.0130	0.0050411366
ss61570082	*rs41664848*	6	67.810819	0.2636	0.3693	0.543577	0.0130	0.0050411366
ss61472916	*rs41575135*	6	67.908094	0.7235	0.5015	0.479080	0.0151	0.0060457320
ss63817181	*rs42368174*	6	67.931489	0.4334	2.2605	0.133166	0.0281	0.0137993758
	rs29017634	6	67.951709	0.5609	2.2961	0.130152	0.0281	0.0138628436
ss117968115		6	67.977546	0.5602	2.5977	0.107470	0.0300	0.0147587067
ss64994212	*rs43082958*	6	68.031626	0.4477	2.9475	0.086454	0.0320	0.0158346835
ss86299679		6	68.090243	0.3761	0.0031	0.955961	0.0011	0.0005248386
ss63816439	*rs42364613*	6	68.139931	0.1519	3.0492	0.081217	0.0448	0.0115407897
ss63422266	*rs42225005*	6	68.465932	0.4964	1.4202	0.233774	0.0216	0.0107989274
ss64939909	*rs43031691*	6	68.535675	0.0702	0.6732	0.412223	0.0294	0.0038342867

ss63422386	*rs42225245*	6	68.565963	0.7951	0.1278	0.720861	0.0081	0.0026268868
ss65385386	*rs43471621*	6	68.597523	0.2493	0.1669	0.682999	0.0086	0.0032351389
ss86324855		6	68.624366	0.5258	2.3175	0.128380	0.0287	0.0142922395
ss61472292	*rs41568930*	6	68.676533	0.4112	0.1828	0.669115	0.0081	0.0039096174
ss86287087		6	68.748125	0.4341	0.0104	0.918677	0.0019	0.0009247256
ss61514149	*rs41610224*	6	68.79644	0.6812	1.9978	0.157972	0.0281	0.0121887589
ss86303099		6	68.821615	0.2987	2.1607	0.142035	0.0298	0.0125034820
ss86337111		6	68.937847	0.5458	0.0093	0.923341	0.0018	0.0008859531
	rs29023994	6	68.995756	0.9148	0.1189	0.730346	0.0114	0.0017721674
ss61497893	*rs43705708*	6	69.032987	0.7471	4.5427	0.033410	0.0452	0.0170713293
ss86296974		6	69.246378	0.0709	1.2735	0.259504	0.0416	0.0054755282
ss86337306		6	69.423043	0.4756	0.2636	0.607816	0.0093	0.0046189515
ss86339862		6	69.487142	0.6146	1.8198	0.177773	0.0256	0.0121218507
ss86292594		6	69.739268	0.6719	5.7030	0.017201	0.0456	0.0201202763
	rs29010988	6	69.834893	0.7113	4.4500	0.035258	0.0420	0.0172422213
ss61466000		6	69.93095	0.8668	0.3536	0.552256	0.0161	0.0037076384
ss86328209		6	69.958338	0.7607	1.8002	0.180126	0.0307	0.0111616008
ss86339645		6	70.1304	0.7980	0.0787	0.779138	0.0065	0.0020904425
ss86338092		6	70.169299	0.2149	0.0193	0.889592	0.0032	0.0010662829
ss86300727		6	70.336282	0.5043	0.2780	0.598195	0.0099	0.0049398329
ss86316752		6	70.403629	0.3761	0.6058	0.436621	0.0151	0.0070726833
ss86299168		6	70.43239	0.5630	0.0393	0.842985	0.0037	0.0018360298
	rs29025576	6	70.521278	0.6404	3.3017	0.069638	0.0342	0.0157418209
ss61557496	*rs41653781*	6	70.559448	0.3797	3.7032	0.054714	0.0372	0.0175244203
ss86327870		6	70.727602	0.5917	0.1391	0.709306	0.0070	0.0033916896
	rs29013964	6	70.795269	0.2543	0.7099	0.399752	0.0178	0.0067441313
ss61516076	*rs41611000*	6	70.960913	0.0781	0.0590	0.808087	0.0083	0.0011910744
ss86341640		6	70.98184	0.4979	4.3338	0.037727	0.0389	0.0194564236
ss86326586		6	71.006567	0.2865	5.0147	0.025448	0.0451	0.0184290001
ss86337358		6	71.045656	0.0781	0.0059	0.938705	0.0026	0.0003735802
	rs29027147	6	71.189705	0.1203	0.5669	0.451765	0.0219	0.0046385309
	rs29017018	6	71.217154	0.8668	0.6221	0.430531	0.0218	0.0050434037
ss86337742		6	71.301279	0.6905	4.0725	0.043971	0.0399	0.0170719097
ss61516074	*rs41610998*	6	71.356946	0.6297	0.1668	0.683080	0.0077	0.0036095223
ss86278477		6	71.392965	0.7966	0.1048	0.746299	0.0077	0.0024808484
ss86337626		6	71.459503	0.1418	0.0104	0.918850	0.0028	0.0006709510
ss61491447	*rs41587840*	6	71.490803	0.8596	0.0006	0.979798	0.0007	0.0001658903
ss61491450	*rs41587843*	6	71.526956	0.1748	0.0055	0.941132	0.0018	0.0005206819
ss86322041		6	71.562445	0.6117	0.0001	0.991417	0.0002	0.0000946850
ss86278111		6	71.66066	0.7264	1.6277	0.202452	0.0274	0.0108778607
ss86336698		6	71.708424	0.3252	2.1447	0.143516	0.0283	0.0124408454
ss86335521		6	71.949142	0.3940	0.0266	0.870504	0.0030	0.0014498999
ss86337720		6	72.023822	0.1619	0.6859	0.407840	0.0203	0.0055122020
ss117968126		6	72.061135	0.9219	4.0997	0.043273	0.0709	0.0102001400
ss117967941		6	72.104529	0.4040	0.1239	0.724961	0.0066	0.0031935358
ss86338620		6	72.192332	0.0752	0.3017	0.582988	0.0198	0.0027537666
ss86321860		6	72.214685	0.6454	1.6651	0.197345	0.0248	0.0113702918

ss117975251		6	72.27545	0.0845	3.9110	0.048366	0.0671	0.0103895820
ss86338433		6	72.299969	0.7486	0.3289	0.566494	0.0124	0.0046537439
ss117975254		6	72.346412	0.4140	0.0623	0.802926	0.0046	0.0022424081
ss117975248		6	72.377595	0.5817	0.0657	0.797753	0.0048	0.0023160071
ss86297216		6	72.401391	0.4635	0.0715	0.789285	0.0049	0.0024195523
ss117968429		6	72.445235	0.7436	0.0404	0.840696	0.0040	0.0015403013
ss117968655		6	72.478577	0.2958	0.2359	0.627356	0.0095	0.0039732680
	rs29016956	6	72.504923	0.2951	1.2053	0.272637	0.0218	0.0090730164
ss117968594		6	72.784128	0.5752	6.1032	0.013732	0.0457	0.0223492012
ss117968409		6	72.821175	0.9298	0.0058	0.939270	0.0028	0.0003636896
ss86326621		6	72.940304	0.7536	5.2303	0.022496	0.0495	0.0183703378
ss117968652		6	72.962935	0.8302	0.2937	0.588053	0.0131	0.0037034586
ss117968075		6	73.006018	0.5845	2.4976	0.114474	0.0297	0.0144183090
ss117968502		6	73.035436	0.2163	7.2664	0.007196	0.0616	0.0208852548
ss117968190		6	73.084308	0.8309	0.0017	0.967288	0.0010	0.0002795128
ss117968406		6	73.11225	0.8252	0.1446	0.703915	0.0092	0.0026438222
ss117968223		6	73.144754	0.8517	0.7208	0.396169	0.0219	0.0055382431
ss86305868		6	73.217013	0.0795	0.2720	0.602166	0.0178	0.0026106741
ss86337508		6	73.2543	0.9112	2.9589	0.085850	0.0554	0.0089634847
ss65386756	*rs43477886*	6	73.298164	0.7170	0.0007	0.979362	0.0005	0.0002124291
ss86329661		6	73.342812	0.4434	3.8729	0.049470	0.0364	0.0179538709
ss86308679		6	73.391535	0.1862	2.9511	0.086265	0.0421	0.0127719645
ss86339284		6	73.412899	0.8166	4.0166	0.045441	0.0461	0.0138075494
	rs29017695	6	73.591857	0.3524	0.2619	0.608969	0.0104	0.0047407475
ss61557545	*rs41653830*	6	73.736271	0.4778	3.1249	0.077544	0.0337	0.0167977245
ss86298884		6	73.770668	0.6719	0.3016	0.583038	0.0107	0.0047155221
ss86327492		6	73.794466	0.6719	0.3016	0.583038	0.0107	0.0047155221
ss64915804	*rs43008807*	6	73.86881	0.8474	3.2798	0.070571	0.0459	0.0118675260
ss65386356	*rs43473386*	6	73.931597	0.8474	2.8283	0.093063	0.0424	0.0109663318
	rs29024839	6	73.955792	0.8474	2.8283	0.093063	0.0424	0.0109663318
ss86314674		6	73.991481	0.8510	1.7543	0.185777	0.0336	0.0085193400
ss86335719		6	74.045215	0.4649	7.2638	0.007206	0.0500	0.0248732137
ss86303133		6	74.080182	0.5208	5.9562	0.014914	0.0453	0.0226012429
	rs29014080	6	74.110718	0.6877	1.2901	0.256424	0.0235	0.0100748036
ss61557475	*rs41653762*	6	74.152819	0.7149	1.8863	0.170064	0.0281	0.0114538932
	rs29023146	6	74.17891	0.2557	0.1816	0.670133	0.0088	0.0033595056
ss86319938		6	74.20561	0.6103	5.4924	0.019379	0.0455	0.0216426246
ss86316012		6	74.268925	0.6404	4.3490	0.037394	0.0406	0.0186939698
ss61557465	*rs41653752*	6	74.360999	0.7980	0.7714	0.380089	0.0210	0.0067703573
ss65384632	*rs43473669*	6	74.475135	0.1590	2.2071	0.137832	0.0382	0.0102307930
ss86339252		6	74.516703	0.8718	3.0055	0.083424	0.0480	0.0107325332
ss46526125	*rs41255269*	6	74.539152	0.5193	4.1952	0.040914	0.0387	0.0193439260
ss61491496	*rs41587889*	6	74.588016	0.3087	1.2698	0.260186	0.0228	0.0097462230
ss86308385		6	74.633646	0.1103	5.0001	0.025662	0.0597	0.0117283512
ss117968083		6	74.690551	0.8052	1.5700	0.210629	0.0297	0.0093274215
ss65386943	*rs43468476*	6	74.79618	0.3295	0.6802	0.409786	0.0165	0.0073053108
ss65386902	*rs43468435*	6	74.833571	0.3295	0.6802	0.409786	0.0165	0.0073053108

ss86299600		6	74.871316	0.1418	2.0381	0.153855	0.0391	0.0095139460
ss61557520	*rs41653805*	6	74.911169	0.2364	3.0342	0.081970	0.0391	0.0141259905
ss86285449		6	74.99407	0.9019	3.0501	0.081171	0.0562	0.0099458095
ss65386868	*rs43467901*	6	75.030033	0.1311	0.9331	0.334384	0.0271	0.0061697962
ss86324560		6	75.063475	0.2292	0.0546	0.815358	0.0053	0.0018580639
ss61557562	*rs41654347*	6	75.092837	0.1117	3.2772	0.070678	0.0529	0.0105026546
ss65387161	*rs43469693*	6	75.120885	0.4269	0.5053	0.477418	0.0134	0.0065481199
ss61557561	*rs41654346*	6	75.226227	0.7586	10.1978	0.001469	0.0679	0.0248726499
ss61491502	*rs41587895*	6	75.259157	0.6640	7.6212	0.005921	0.0537	0.0239688504
ss61491795	*rs43741126*	6	75.285715	0.5408	0.1318	0.716636	0.0068	0.0033641795
ss86313833		6	75.359135	0.2206	3.5248	0.060876	0.0430	0.0147709417
ss65386967	*rs43468499*	6	75.415533	0.7772	1.5262	0.217102	0.0280	0.0097072600
ss86307422		6	75.484083	0.7787	2.6717	0.102601	0.0376	0.0129458457
ss61491468	*rs41587861*	6	75.551943	0.2292	1.0066	0.316073	0.0222	0.0078623017
ss65385819	*rs43467654*	6	75.584865	0.8360	7.6175	0.005933	0.0676	0.0185340161
ss86336055		6	75.618288	0.5831	0.0275	0.868451	0.0031	0.0015285420
	rs29025531	6	75.645613	0.2407	5.3085	0.021515	0.0506	0.0184819778
ss61523670	*rs41622833*	6	75.682699	0.8832	6.0944	0.013800	0.0719	0.0148395418
ss65387639	*rs43473866*	6	75.743787	0.3825	0.0504	0.822371	0.0043	0.0020424517
ss86339620		6	75.828151	0.0559	0.1339	0.714528	0.0145	0.0015300717
ss86327777		6	75.960191	0.0509	0.0618	0.803717	0.0102	0.0009879833
	rs29022489	6	76.200452	0.3066	0.2900	0.590409	0.0110	0.0046694492
ss86332636		6	76.320348	0.1483	4.2118	0.040516	0.0536	0.0135268440
	rs29024480	6	76.5808	0.5050	1.3752	0.241320	0.0215	0.0107351844
ss61491506	*rs41587899*	6	76.626037	0.4936	1.2165	0.270434	0.0203	0.0101258941
	rs29022820	6	76.946177	0.1483	0.7611	0.383271	0.0233	0.0058883356
ss117968421		6	77.031025	0.0595	0.4295	0.512434	0.0263	0.0029403267
ss117963892		6	77.293136	0.8467	1.0669	0.302013	0.0263	0.0068353806
	rs29021769	6	77.34491	0.8274	0.1589	0.690339	0.0095	0.0027069854
ss86326216		6	77.644005	0.1705	0.0847	0.771130	0.0070	0.0019706615
ss61569022	*rs41668300*	6	77.941426	0.3381	0.1988	0.655831	0.0088	0.0039329749
ss61470005	*rs41566002*	6	77.977191	0.2858	6.0032	0.014525	0.0488	0.0199200417
ss61531833	*rs41630374*	6	78.55484	0.5050	2.4233	0.119998	0.0303	0.0151288889
ss86329753		6	78.603002	0.3266	3.7928	0.051874	0.0391	0.0171854287
ss117968284		6	78.652911	0.8288	1.4413	0.230342	0.0295	0.0083666819
ss61563645	*rs41662716*	6	78.754038	0.4828	0.2573	0.612149	0.0095	0.0047276863
ss86340377		6	78.884806	0.5143	1.2341	0.266996	0.0201	0.0100312478
ss86294908		6	79.189	0.4183	0.0205	0.886264	0.0028	0.0013419064
ss61557588	*rs41654373*	6	79.611413	0.2299	5.3881	0.020562	0.0525	0.0186091586
ss61557603	*rs41654388*	6	79.640756	0.7951	4.1893	0.041054	0.0486	0.0158420047
ss61469118	*rs41567029*	6	79.779971	0.1769	0.5016	0.479042	0.0176	0.0051251825
ss61469120	*rs41567731*	6	79.885829	0.2063	0.0818	0.775002	0.0068	0.0022117102
ss86317429		6	79.908104	0.1963	0.1952	0.658778	0.0107	0.0033605672
ss61557125	*rs41651324*	6	79.944604	0.5437	0.0051	0.943215	0.0014	0.0006767956
ss86316180		6	80.157014	0.1562	0.1767	0.674368	0.0107	0.0028174463
	rs29012305	6	80.37377	0.6275	1.0660	0.302208	0.0195	0.0090977322
ss86332658		6	80.493302	0.7013	1.6123	0.204593	0.0256	0.0107146744

ss61557140	*rs41652039*	6	80.517633	0.6991	1.6514	0.199198	0.0258	0.0108637910
	rs29018853	6	80.56873	0.4628	6.6260	0.010256	0.0489	0.0243316387
ss61510584	*rs41605207*	6	80.630673	0.0974	0.0971	0.755410	0.0097	0.0017117702
ss61466347	*rs41568102*	6	80.688834	0.1504	1.8489	0.174347	0.0348	0.0088883840
ss61557142	*rs41652041*	6	80.715298	0.2736	0.8480	0.357432	0.0186	0.0074129252
ss65377941	*rs43460488*	6	80.813606	0.5365	0.7005	0.402908	0.0158	0.0078595842
	rs29026113	6	80.885418	0.2880	0.5092	0.475733	0.0148	0.0060720856
ss86320422		6	80.957319	0.5595	0.0243	0.876171	0.0029	0.0014278420
ss65388143	*rs43469868*	6	81.052161	0.2966	0.0268	0.869953	0.0033	0.0013644560
ss65340802	*rs43426254*	6	81.092861	0.8696	3.2935	0.069985	0.0504	0.0114368286
ss86340725		6	81.134003	0.6640	0.1380	0.710404	0.0069	0.0030917622
ss65388102	*rs43469330*	6	81.159095	0.1132	2.1258	0.145284	0.0433	0.0086887362
ss65388043	*rs43469271*	6	81.185309	0.2149	1.0652	0.302383	0.0232	0.0078215266
ss64917069	*rs43014254*	6	81.244922	0.3324	3.5810	0.058857	0.0376	0.0167054560
ss64917060	*rs43014245*	6	81.270445	0.4549	1.0518	0.305453	0.0194	0.0096046199
ss65387951	*rs43468580*	6	81.296476	0.1325	2.8598	0.091268	0.0460	0.0105858513
ss117967943		6	81.322876	0.5444	2.1626	0.141854	0.0279	0.0138279158
ss86302398		6	81.354371	0.7672	0.0184	0.892172	0.0029	0.0010483118
ss61563696	*rs41663365*	6	81.396637	0.6970	8.8682	0.003003	0.0590	0.0249151579
ss61523822	*rs43743074*	6	81.48985	0.3560	1.7824	0.182293	0.0258	0.0118223834
ss64184711	*rs42518899*	6	81.524194	0.1254	2.5499	0.110757	0.0439	0.0096160948
ss64184643	*rs42518865*	6	81.550584	0.1196	2.7751	0.096189	0.0464	0.0097649282
ss61523820	*rs41623578*	6	81.605854	0.3209	2.9398	0.086868	0.0336	0.0146333240
ss61563701	*rs41663370*	6	81.649289	0.2170	0.3060	0.580330	0.0123	0.0041876445
ss61494300	*rs41595703*	6	81.707428	0.1361	0.8962	0.344122	0.0250	0.0058836041
ss64920791	*rs43009638*	6	81.773117	0.1361	0.8962	0.344122	0.0250	0.0058836041
ss64555772	*rs42695871*	6	81.808192	0.1347	0.9908	0.319884	0.0264	0.0061473653
ss64556252	*rs42697111*	6	81.8728	0.1239	2.1610	0.142003	0.0405	0.0087990261
ss61527415	*rs41623376*	6	81.912662	0.1254	2.4148	0.120645	0.0427	0.0093613915
	rs29015635	6	81.95867	0.0974	3.7221	0.054102	0.0611	0.0107498298
ss65092507	*rs43182706*	6	81.996358	0.2163	10.2533	0.001426	0.0705	0.0239168255
ss64805722	*rs42896819*	6	82.200484	0.7679	7.1535	0.007657	0.0578	0.0206180829
ss64805553	*rs42895750*	6	82.226494	0.1304	1.6584	0.198251	0.0346	0.0078444587
ss64905608	*rs42996618*	6	82.251809	0.2393	7.5844	0.006041	0.0588	0.0213948546
		6	82.278023	0.8754	1.6307	0.202038	0.0349	0.0076242522
ss64254428	*rs42551311*	6	82.356852	0.7235	0.0024	0.960814	0.0010	0.0004188490
ss65388352	*rs43471376*	6	82.579209	0.9341	0.6192	0.431602	0.0295	0.0036347519
ss65388414	*rs43472038*	6	82.601979	0.3374	0.1788	0.672560	0.0081	0.0036399911
ss61527804	*rs41626846*	6	82.641003	0.3295	0.3628	0.547172	0.0116	0.0051294792
ss61510638	*rs41605961*	6	82.70259	0.9355	0.5179	0.471997	0.0272	0.0032855954
ss65388759	*rs43474282*	6	82.726335	0.2629	6.8729	0.008942	0.0549	0.0212843071
ss65388590	*rs43473013*	6	82.749974	0.0630	0.6901	0.406426	0.0317	0.0037476301
ss38327294	*rs29019404*	6	82.782065	0.2880	3.0730	0.080044	0.0355	0.0145712532
ss65389058	*rs43468982*	6	82.813973	0.0602	0.6283	0.428245	0.0309	0.0034894922
ss65388825	*rs43467549*	6	82.842471	0.3689	3.8941	0.048852	0.0383	0.0178133130
ss86309154		6	82.899723	0.2887	3.5787	0.058941	0.0383	0.0157457329
ss86287725		6	82.924932	0.2887	3.5787	0.058941	0.0383	0.0157457329

ss61476972	*rs41580222*	6	83.023197	0.5179	0.9355	0.333771	0.0181	0.0090295867
	rs29022785	6	83.05472	0.5072	0.1064	0.744420	0.0061	0.0030333951
ss117968363		6	83.106835	0.5874	0.7062	0.401000	0.0160	0.0077333433
ss61517242	*rs41613836*	6	83.250025	0.3983	0.4192	0.517568	0.0123	0.0059095287
	rs29025335	6	83.365546	0.3388	0.0008	0.977046	0.0006	0.0002526920
ss117968152		6	83.608822	0.2077	2.7759	0.096145	0.0391	0.0128549877
ss86339162		6	83.730034	0.2285	0.0878	0.767048	0.0064	0.0022654562
ss61557629	*rs41654414*	6	83.768852	0.1361	4.1613	0.041735	0.0551	0.0129637376
ss86275221		6	83.815542	0.6784	5.6452	0.017773	0.0471	0.0205427068
ss117968251		6	83.860962	0.2385	6.0450	0.014188	0.0551	0.0200229404
ss117968013		6	83.920998	0.2350	7.5140	0.006279	0.0614	0.0220742374
ss117968616		6	83.957417	0.1769	5.5327	0.018942	0.0589	0.0171519066
ss117968226		6	84.016058	0.5079	3.7133	0.054388	0.0357	0.0178470736
ss117968597		6	84.064886	0.1884	8.8024	0.003111	0.0714	0.0218359267
ss117968378		6	84.087732	0.5122	3.4024	0.065527	0.0341	0.0170377284
ss61557698	*rs41654981*	6	84.153368	0.1390	6.0774	0.013933	0.0656	0.0157032648
ss61557685	*rs41654969*	6	84.174078	0.3510	0.0000	0.997810	0.0001	0.0000239542
ss117968104		6	84.194536	0.2199	0.1165	0.732971	0.0077	0.0026270796
ss117968375		6	84.219063	0.4413	4.9939	0.025753	0.0412	0.0203376836
ss117968304		6	84.272973	0.7092	0.0876	0.767286	0.0061	0.0025145177
ss61557680	*rs41654964*	6	84.321562	0.9427	1.1882	0.276067	0.0433	0.0046739457
ss61510643	*rs41605966*	6	84.352937	0.9463	0.0422	0.837391	0.0084	0.0008497201
	rs29012675	6	84.387496	0.1304	0.4604	0.497643	0.0188	0.0042635133
ss117968159		6	84.414774	0.3775	0.4233	0.515491	0.0126	0.0059218803
ss86293802		6	84.630135	0.1103	0.1585	0.690647	0.0120	0.0023548068
ss61557647	*rs41654932*	6	84.782128	0.4205	0.1707	0.679577	0.0077	0.0037562825
ss61557644	*rs41654429*	6	84.894568	0.2801	9.7955	0.001823	0.0650	0.0261975104
ss61475400	*rs41574480*	6	84.921612	0.9413	0.7069	0.400766	0.0330	0.0036459757
ss61557643	*rs41654428*	6	84.955938	0.3431	2.0441	0.153244	0.0282	0.0126980937
ss86341514		6	84.981828	0.3431	2.0441	0.153244	0.0282	0.0126980937
ss117968624		6	85.012545	0.3431	2.0441	0.153244	0.0282	0.0126980937
ss61515605	*rs41615958*	6	85.179167	0.1476	11.3810	0.000783	0.0902	0.0227040450
ss117963861		6	85.289127	0.9219	3.0972	0.078865	0.0604	0.0086907192
ss86300738		6	85.39776	0.3360	0.6896	0.406592	0.0162	0.0072392530
ss61570086	*rs41664852*	6	85.5263	0.6468	0.0615	0.804234	0.0050	0.0022950110
ss86290654		6	85.616293	0.4212	0.2933	0.588294	0.0102	0.0049567267
ss86326966		6	85.640057	0.3009	0.0224	0.880953	0.0031	0.0013029092
ss86336379		6	85.663648	0.7686	2.1391	0.144042	0.0332	0.0118126151
ss61518051	*rs41621414*	6	85.738923	0.6562	0.3712	0.542563	0.0117	0.0052646542
ss61518059	*rs41621422*	6	85.769868	0.6340	0.9871	0.320794	0.0186	0.0086207326
ss61523671	*rs41622834*	6	85.918972	0.2378	5.7681	0.016581	0.0509	0.0184534490
	rs29001782	6	86.128028	0.5781	5.8938	0.015447	0.0475	0.0231775220
ss61506487	*rs41606097*	6	86.365127	0.8911	0.0632	0.801575	0.0077	0.0014897940
ss61557722	*rs41655005*	6	86.434939	0.6662	6.5733	0.010561	0.0518	0.0230370369
ss61518891	*rs41618641*	6	86.613446	0.4148	1.8807	0.170695	0.0262	0.0127208779
ss86291015		6	86.671686	0.8840	0.4683	0.493984	0.0197	0.0040492085
ss86297176		6	86.712349	0.4613	0.0007	0.978616	0.0005	0.0002540019

	rs29010229	6	86.810567	0.1067	0.0000	0.994412	0.0002	0.0000401501
ss61473489	*rs41570706*	6	86.908338	0.7120	0.0037	0.951286	0.0013	0.0005203898
ss117967957		6	87.103073	0.4850	2.1686	0.141307	0.0271	0.0135296499
ss86307579		6	87.255541	0.6125	5.9736	0.014769	0.0461	0.0218899138
ss86291546		6	87.370507	0.4277	3.0180	0.082790	0.0328	0.0160454076
ss61466227	*rs41566887*	6	87.480011	0.8725	0.2430	0.622175	0.0139	0.0030989465
ss86324844		6	87.663733	0.8768	0.2151	0.642909	0.0128	0.0027700927
ss86317213		6	87.879378	0.9305	0.5735	0.449120	0.0281	0.0036350921
ss61516066	*rs41610991*	6	87.904281	0.9456	0.5310	0.466445	0.0304	0.0031285388
	rs29010267	6	87.989289	0.0552	0.5780	0.447350	0.0315	0.0032861398
	rs29015041	6	88.160022	0.0788	0.2520	0.615801	0.0172	0.0024969571
ss117968170		6	88.263655	0.5831	4.5375	0.033509	0.0401	0.0194952753
ss117968347		6	88.302638	0.2464	0.0922	0.761474	0.0065	0.0024293995
ss117968764		6	88.326005	0.7414	0.0570	0.811302	0.0050	0.0019074103
ss117968472		6	88.350095	0.1862	0.1247	0.724050	0.0082	0.0024750733
ss117968030		6	88.370145	0.0458	0.4293	0.512529	0.0290	0.0025328106
ss117968525		6	88.42776	0.1332	0.6127	0.434032	0.0209	0.0048172549
ss117968176		6	88.452829	0.6576	2.8138	0.093904	0.0324	0.0146079660
ss117968780		6	88.473587	0.4749	0.9814	0.322209	0.0181	0.0090191800
ss61491563	*rs41588950*	6	88.508848	0.5150	2.8151	0.093830	0.0310	0.0154942316
ss61465597	*rs41567942*	6	88.724565	0.1447	1.2828	0.257761	0.0305	0.0075376770
ss86312906		6	88.806896	0.3861	1.2439	0.265113	0.0211	0.0099866264
ss117968738		6	88.983536	0.5179	0.4581	0.498733	0.0123	0.0061235316
ss86326721		6	89.03023	0.4248	3.7334	0.053741	0.0363	0.0177197003
ss65391865	*rs43470976*	6	89.05906	0.0709	0.0038	0.950644	0.0022	0.0002942070
ss117968849		6	89.081282	0.6175	0.6356	0.425582	0.0153	0.0072336649
ss64840851	*rs42936833*	6	89.106703	0.0716	0.0065	0.935839	0.0029	0.0003846995
ss86341106		6	89.212073	0.4212	0.2277	0.633396	0.0088	0.0043059727
ss117968685		6	89.25383	0.1139	1.8062	0.179402	0.0390	0.0078772654
	rs29010419	6	89.274693	0.1812	2.5590	0.110122	0.0375	0.0111301380
ss86285294		6	89.355143	0.8847	5.9267	0.015164	0.0691	0.0140914719
ss61491594	*rs41588981*	6	89.387112	0.0652	3.1817	0.074904	0.0640	0.0078043093
	rs29022799	6	89.603521	0.9520	1.0342	0.309521	0.0441	0.0040278005
ss61524338	*rs41619788*	6	89.623897	0.5523	3.7792	0.052295	0.0357	0.0176640183
ss38332444	*rs29015947*	6	89.730161	0.8875	4.6282	0.031794	0.0607	0.0121168930
ss86337596		6	89.774923	0.2865	13.3176	0.000283	0.0723	0.0295444854
ss65257149	*rs43338539*	6	89.838828	0.5330	8.3529	0.003971	0.0533	0.0265103516
ss65257178	*rs43338568*	6	89.926346	0.8231	0.0251	0.874243	0.0039	0.0011245856
ss86296213		6	90.0081	0.6812	3.5756	0.059048	0.0368	0.0160024580
ss64672790	*rs42766480*	6	90.075264	0.4771	7.6428	0.005851	0.0504	0.0251319124
ss86302265		6	90.259359	0.9191	3.3544	0.067453	0.0613	0.0091244056
ss61528083	*rs41629224*	6	90.288992	0.1325	5.0421	0.025052	0.0599	0.0137826410
ss61562683	*rs41663978*	6	90.31754	0.5494	2.1967	0.138762	0.0277	0.0136930029
ss61524397	*rs41620643*	6	90.356014	0.6777	10.0042	0.001630	0.0626	0.0273447944
ss86297489		6	90.415521	0.5215	24.8285	0.000001	0.0896	0.0447323057
ss86317874		6	90.485681	0.9284	1.9767	0.160182	0.0509	0.0067751340
ss61528080	*rs41629221*	6	90.508813	0.7457	2.9882	0.084316	0.0378	0.0143305645

ss61507506	*rs41606699*	6	90.564545	0.1948	0.8806	0.348370	0.0211	0.0066280965
	rs29024026	6	90.644773	0.8639	0.3865	0.534374	0.0169	0.0039727109
ss61522200	*rs41617692*	6	90.670191	0.7894	1.5106	0.219464	0.0279	0.0092730782
ss63313756	*rs42187586*	6	90.693272	0.8130	0.7304	0.393034	0.0202	0.0061487926
ss64962805	*rs43052931*	6	90.715457	0.8789	3.5771	0.058998	0.0552	0.0117493596
ss64962814	*rs43052940*	6	90.737718	0.2486	0.0128	0.909822	0.0024	0.0008802570
ss64837631	*rs42932743*	6	90.990507	0.1762	0.0558	0.813338	0.0057	0.0016555994
ss61557794	*rs41655374*	6	91.047326	0.2822	0.6832	0.408774	0.0174	0.0070447401
ss61523674	*rs41622837*	6	91.140234	0.1648	0.5061	0.477060	0.0177	0.0048592861
ss62425951	*rs41869408*	6	91.190938	0.4169	2.2279	0.135988	0.0277	0.0134835115
ss62426140	*rs41870471*	6	91.303461	0.9033	1.6953	0.193330	0.0404	0.0070533545
ss63192437	*rs42149287*	6	91.388559	0.8746	3.0313	0.082113	0.0503	0.0110309344
ss63192380	*rs42149268*	6	91.417375	0.0659	5.0078	0.025549	0.0817	0.0100629552
ss61550747	*rs41646333*	6	91.445585	0.4735	6.2035	0.012982	0.0447	0.0222677995
ss61550746	*rs41646332*	6	91.553826	0.1991	2.3660	0.124460	0.0352	0.0112349948
ss61557678	*rs41654962*	6	91.692661	0.7636	0.1935	0.660190	0.0093	0.0033512601
ss65389460	*rs43471476*	6	91.8978	0.4004	0.0673	0.795327	0.0049	0.0023348149
ss86334878		6	91.918868	0.2342	0.0472	0.828118	0.0046	0.0016654851
ss61557674	*rs41654958*	6	91.961906	0.2085	2.7627	0.096935	0.0373	0.0123247072
ss86311372		6	92.030875	0.5981	12.1768	0.000514	0.0656	0.0315229546
ss61557664	*rs41654948*	6	92.150445	0.5244	5.3915	0.020522	0.0422	0.0210708093
ss86339292		6	92.17932	0.5272	5.9824	0.014696	0.0443	0.0220713570
ss86339125		6	92.216839	0.3768	5.7505	0.016746	0.0435	0.0204163859
	rs29016177	6	92.240041	0.7894	3.6542	0.056339	0.0420	0.0139772262
ss64865885	*rs42959067*	6	92.274349	0.1669	7.1495	0.007674	0.0663	0.0184334747
ss61557797	*rs41655377*	6	92.307061	0.1590	7.6785	0.005737	0.0734	0.0196222504
ss38334622	*rs29024522*	6	92.395113	0.4499	4.6125	0.032084	0.0390	0.0193009986
ss64314234	*rs42581544*	6	92.434964	0.2901	3.4406	0.064036	0.0369	0.0151863458
ss61496193	*rs41597446*	6	92.473531	0.9011	6.2937	0.012343	0.0796	0.0141734690
ss86285114		6	92.517199	0.3668	12.1865	0.000512	0.0645	0.0299746085
ss86300582		6	92.646174	0.6153	6.0249	0.014349	0.0474	0.0224292180
ss86335839		6	92.767318	0.5136	8.4566	0.003753	0.0534	0.0266960045
ss61568524	*rs41664614*	6	92.788189	0.4756	8.6321	0.003412	0.0532	0.0265405279
ss86310987		6	92.835502	0.7192	0.0592	0.807852	0.0053	0.0021486707
ss64675844	*rs42773532*	6	92.854145	0.8173	0.4280	0.513185	0.0162	0.0048499617
ss86333471		6	92.886878	0.7615	0.1397	0.708683	0.0085	0.0030821271
ss64676046	*rs42775634*	6	92.914805	0.7629	0.1838	0.668278	0.0097	0.0035210176
ss61496362	*rs41598313*	6	92.949327	0.1289	0.4388	0.507913	0.0188	0.0042118192
ss64330136	*rs42592169*	6	93.002335	0.5344	12.3240	0.000476	0.0624	0.0310733609
ss64330198	*rs42592200*	6	93.03131	0.1691	7.5745	0.006074	0.0672	0.0188719039
ss86313553		6	93.055655	0.3302	6.0161	0.014420	0.0481	0.0212663089
ss64330632	*rs42582518*	6	93.155306	0.7006	2.5001	0.114290	0.0319	0.0134025862
ss64161295	*rs42503682*	6	93.267214	0.9527	0.8586	0.354457	0.0411	0.0037027780
ss61493860	*rs41591081*	6	93.305749	0.3761	9.5018	0.002134	0.0568	0.0266606259
ss61562734	*rs41664028*	6	93.33751	0.9520	1.4137	0.234856	0.0524	0.0047858822
ss61569057	*rs41664747*	6	93.371537	0.5000	8.1432	0.004451	0.0512	0.0256000663
ss64388804	*rs42615162*	6	93.463203	0.1784	1.0973	0.295229	0.0259	0.0075842606

ss65393667	*rs43479253*	6	93.545536	0.6024	4.5092	0.034064	0.0406	0.0194490191
ss86316121		6	93.640924	0.1583	3.6874	0.055233	0.0482	0.0128406385
ss64254760	*rs42553777*	6	93.682965	0.3023	2.4514	0.117871	0.0320	0.0134874457
ss64254786	*rs42553790*	6	93.70472	0.3983	2.6343	0.105031	0.0311	0.0148921548
ss61495618	*rs41591189*	6	93.72987	0.0272	1.8719	0.171697	0.0758	0.0040164508
ss64902335	*rs42992679*	6	93.767253	0.7421	0.0014	0.970241	0.0008	0.0003112422
ss64255070	*rs42555832*	6	93.796991	0.4305	5.5306	0.018965	0.0443	0.0217254870
ss64255152	*rs42555873*	6	93.850918	0.5659	6.6408	0.010171	0.0482	0.0236859327
ss64254846	*rs42553820*	6	93.91827	0.0652	0.3172	0.573468	0.0212	0.0025860272
ss86291415		6	93.962054	0.7908	3.2283	0.072811	0.0406	0.0134247988
ss86283450		6	93.993831	0.2027	10.9579	0.000980	0.0773	0.0249798465
ss117967932		6	94.050758	0.0931	1.7596	0.185113	0.0431	0.0072776808
ss117968835		6	94.082192	0.9097	1.1648	0.280846	0.0355	0.0058249599
ss46526536	*rs41256838*	6	94.129425	0.1755	2.1769	0.140549	0.0346	0.0100156300
ss86289048		6	94.204422	0.7887	1.5501	0.213538	0.0279	0.0092947412
ss86294120		6	94.293112	0.8080	0.3870	0.534109	0.0147	0.0045551093
ss86336873		6	94.318083	0.1003	4.9147	0.026951	0.0689	0.0124336043
ss65390703	*rs43477315*	6	94.384508	0.8926	0.0976	0.754762	0.0094	0.0017968822
ss86313930		6	94.433955	0.9047	5.5259	0.019015	0.0725	0.0124960410
ss86336041		6	94.601619	0.4943	10.4041	0.001316	0.0595	0.0297410153
ss86322902		6	94.640251	0.5480	8.9495	0.002874	0.0560	0.0277442088
ss86341394		6	94.736056	0.5107	13.4897	0.000258	0.0689	0.0344235610
ss86339206		6	94.788664	0.5723	7.3174	0.006996	0.0506	0.0247818322
ss86293643		6	94.827333	0.4470	0.0831	0.773213	0.0052	0.0025871427
ss86332222		6	94.977129	0.6576	0.0527	0.818513	0.0045	0.0020144872
ss86289530		6	94.997341	0.2772	0.4183	0.517987	0.0133	0.0053404674
ss61557762	*rs41655345*	6	95.042614	0.4864	2.1080	0.146984	0.0268	0.0134007264
ss65391801	*rs43480110*	6	95.080503	0.9570	1.8428	0.175063	0.0617	0.0050784663
ss86330106		6	95.103435	0.7299	9.8537	0.001767	0.0641	0.0252910870
ss61472560	*rs41571584*	6	95.229148	0.9649	8.0921	0.004576	0.1378	0.0093345078
ss86286430		6	95.269006	0.6182	2.1508	0.142948	0.0282	0.0133166929
ss86332567		6	95.528038	0.4040	6.0161	0.014421	0.0462	0.0222278245
ss117968857		6	95.643455	0.3797	5.2041	0.022835	0.0437	0.0205704365
ss86306932		6	95.704461	0.6189	5.3352	0.021191	0.0440	0.0207629547
ss61528051	*rs41629192*	6	95.719797	0.0996	3.7594	0.052916	0.0604	0.0108341984
	rs29020798	6	95.739959	0.4241	0.6951	0.404711	0.0154	0.0075147796
ss65393812	*rs43479594*	6	95.770023	0.4219	0.6836	0.408634	0.0151	0.0073794699
ss61491603	*rs41588990*	6	95.877326	0.3073	9.4902	0.002147	0.0596	0.0253734494
ss61557804	*rs41655384*	6	95.925106	0.9649	2.3945	0.122217	0.0768	0.0052051101
ss64705294	*rs42801113*	6	96.0233	0.9570	0.2885	0.591359	0.0249	0.0020459498
ss86341675		6	96.063578	0.7457	1.1532	0.283262	0.0230	0.0087073191
ss86289414		6	96.193688	0.9556	0.2185	0.640362	0.0213	0.0018102224
ss86310942		6	96.220858	0.5788	0.2853	0.593400	0.0099	0.0048280652
ss61557846	*rs41653142*	6	96.259023	0.3066	1.5046	0.220381	0.0243	0.0103238876
ss86324329		6	96.29955	0.8381	0.0573	0.810815	0.0058	0.0015799952
ss65394896	*rs43476086*	6	96.322849	0.1383	1.0052	0.316405	0.0275	0.0065522513
ss65394854	*rs43476044*	6	96.344644	0.1877	6.0858	0.013867	0.0582	0.0177369445

ss86296705		6	96.426277	0.2693	4.0977	0.043323	0.0423	0.0166398441
ss86287334		6	96.466808	0.3990	5.9779	0.014733	0.0456	0.0218477182
ss65394577	*rs43482362*	6	96.513911	0.8854	7.8135	0.005328	0.0805	0.0163332948
ss86340510		6	96.601545	0.3911	10.1207	0.001531	0.0593	0.0282540208
ss65394227	*rs43479020*	6	96.704583	0.5852	1.6799	0.195373	0.0239	0.0116062106
	rs29020620	6	96.724594	0.8295	7.4521	0.006496	0.0652	0.0184468117
ss86279901		6	96.748063	0.8560	11.9118	0.000591	0.0885	0.0218180507
ss64695147	*rs42790107*	6	96.844637	0.1375	8.0469	0.004691	0.0761	0.0180455839
ss61518585	*rs41616437*	6	96.884951	0.4936	3.5977	0.058273	0.0342	0.0170773627
ss61540596	*rs41636046*	6	96.979905	0.3976	0.4821	0.487697	0.0131	0.0062939053
ss62562624	*rs41912428*	6	97.005043	0.6003	0.3912	0.531882	0.0119	0.0056964412
ss62562183	*rs41911481*	6	97.031122	0.3632	0.0105	0.918570	0.0020	0.0009187757
ss62562090	*rs41911450*	6	97.058544	0.1132	2.7251	0.099231	0.0489	0.0098213560
ss86328432		6	97.087469	0.4011	0.4890	0.484625	0.0132	0.0063220112
ss61557866	*rs41653162*	6	97.116102	0.9456	0.3048	0.581091	0.0224	0.0023057800
ss65395280	*rs43479067*	6	97.217327	0.4198	4.7910	0.028940	0.0403	0.0196313537
ss61507301	*rs41604697*	6	97.243068	0.9398	0.1052	0.745827	0.0125	0.0014107329
ss61557865	*rs41653161*	6	97.277586	0.5301	2.4397	0.118750	0.0278	0.0138586250
ss65395249	*rs43479036*	6	97.313642	0.5953	5.9160	0.015255	0.0465	0.0224256974
ss86340606		6	97.334936	0.5888	2.5077	0.113749	0.0298	0.0144256809
ss86317936		6	97.36483	0.4527	2.9555	0.086029	0.0314	0.0155368803
ss86284818		6	97.388879	0.1139	0.3844	0.535456	0.0181	0.0036562903
ss61557862	*rs41653158*	6	97.410705	0.1139	0.3844	0.535456	0.0181	0.0036562903
ss86318741		6	97.508322	0.2994	29.6085	0.000000	0.1031	0.0432479824
ss65395497	*rs43480584*	6	97.580459	0.7650	28.8025	0.000000	0.1116	0.0401328475
ss61557869	*rs41653165*	6	97.606827	0.7213	38.8013	0.000000	0.1198	0.0481785829
ss61557876	*rs41653172*	6	97.666165	0.4728	11.0084	0.000954	0.0605	0.0301576203
ss65395852	*rs43475842*	6	97.726008	0.4499	1.7623	0.184775	0.0245	0.0121453621
ss65395946	*rs43476636*	6	98.144739	0.0494	2.2654	0.132742	0.0644	0.0060498568
ss86337198		6	98.372241	0.1375	0.0289	0.865143	0.0046	0.0010927120
ss64140301	*rs42500966*	6	98.467431	0.9327	3.7386	0.053575	0.0709	0.0089079434
ss64954276	*rs43046934*	6	98.534076	0.8940	6.8696	0.008959	0.0775	0.0146820599
ss61510674	*rs41605997*	6	98.684783	0.8653	2.2953	0.130219	0.0408	0.0095175112
ss86338273		6	98.73528	0.9577	0.0780	0.780086	0.0130	0.0010548169
	rs29013689	6	98.767643	0.9284	0.6507	0.420123	0.0275	0.0036567280
ss65396590	*rs43481380*	6	98.801684	0.9191	0.8812	0.348192	0.0301	0.0044824032
ss64798400	*rs42901363*	6	98.915954	0.4692	8.7895	0.003133	0.0564	0.0281053248
ss61477262	*rs41575507*	6	98.966524	0.3582	0.1679	0.682143	0.0080	0.0036872771
ss64717798	*rs42815429*	6	99.086447	0.8961	1.7278	0.189131	0.0411	0.0076536587
ss64981718	*rs43077190*	6	99.135154	0.7371	0.2897	0.590593	0.0115	0.0044407676
ss61491660	*rs41589947*	6	99.171706	0.9527	3.9957	0.046006	0.0870	0.0078417799
ss61504243	*rs41605032*	6	99.240715	0.9628	3.4535	0.063542	0.0898	0.0064409670
ss65396321	*rs43479211*	6	99.268177	0.7536	0.0085	0.926693	0.0020	0.0007257707
ss65396340	*rs43479230*	6	99.298384	0.7407	0.4023	0.526088	0.0132	0.0050763658
ss61557887	*rs41653183*	6	99.412163	0.1927	5.9214	0.015209	0.0564	0.0175592692
ss61510673	*rs41605996*	6	99.503347	0.2092	2.8321	0.092848	0.0380	0.0125735470
	rs29021982	6	99.581269	0.0437	5.2884	0.021762	0.1036	0.0086543217

ss86314682		6	99.625068	0.4620	0.6113	0.434559	0.0144	0.0071433365
ss65397605	*rs43484093*	6	99.688092	0.2507	5.0600	0.024795	0.0484	0.0181755569
ss61557940	*rs41654035*	6	99.709444	0.1655	19.2832	0.000013	0.1066	0.0294455790
ss86283281		6	99.754237	0.2378	0.0065	0.935770	0.0017	0.0006188236
ss117968855		6	99.80464	0.8460	0.0765	0.782187	0.0072	0.0018859712
ss61557947	*rs41654042*	6	99.827767	0.1289	3.5916	0.058486	0.0529	0.0118744594
ss65397814	*rs43486402*	6	99.991094	0.6397	7.0345	0.008177	0.0532	0.0245233218
ss65397855	*rs43478543*	6	100.021521	0.3832	0.0842	0.771761	0.0055	0.0026011536
ss86292307		6	100.085847	0.0788	1.7954	0.180701	0.0468	0.0067944394
ss86336580		6	100.133706	0.0845	4.7908	0.028943	0.0743	0.0114965484
ss65398133	*rs43480419*	6	100.165426	0.0358	4.8447	0.028058	0.1081	0.0074680596
ss86306075		6	100.206953	0.0989	7.7806	0.005425	0.0855	0.0152301014
ss61510681	*rs41606004*	6	100.440173	0.8288	0.1366	0.711757	0.0090	0.0025479484
ss86305784		6	100.473518	0.2672	6.9859	0.008400	0.0558	0.0218644414
ss61557927	*rs41653222*	6	100.707084	0.1590	8.4577	0.003751	0.0732	0.0195682208
ss61557921	*rs41653216*	6	100.740254	0.7822	0.2193	0.639757	0.0104	0.0035390950
ss65396788	*rs43482772*	6	100.773335	0.3288	5.8210	0.016093	0.0479	0.0211206144
ss65396748	*rs43482733*	6	100.823985	0.3582	1.0394	0.308318	0.0194	0.0088997442
ss86312504		6	100.846827	0.2787	19.7287	0.000010	0.0889	0.0357586468
ss46526011	*rs41255159*	6	100.922971	0.4799	4.9874	0.025849	0.0399	0.0199389676
ss65397380	*rs43482268*	6	101.022926	0.4470	2.9054	0.088727	0.0315	0.0155552721
ss61527982	*rs41627923*	6	101.058356	0.6182	0.1054	0.745580	0.0063	0.0029540762
ss63505511	*rs42250315*	6	101.096253	0.1024	1.1663	0.280533	0.0321	0.0058990941
	rs29022306	6	101.204543	0.5466	20.0080	0.000009	0.0809	0.0401173191
ss86298448		6	101.240916	0.2070	17.1316	0.000039	0.0907	0.0297791837
ss63504851	*rs42257604*	6	101.292548	0.6884	14.8560	0.000127	0.0744	0.0319294340
ss63504722	*rs42257561*	6	101.321685	0.6662	0.5204	0.470928	0.0148	0.0065768749
ss86326136		6	101.343014	0.3646	1.4882	0.222906	0.0235	0.0108819878
ss63504488	*rs42256786*	6	101.411124	0.5953	3.2800	0.070561	0.0341	0.0164116858
ss86306712		6	101.486933	0.7464	0.3066	0.579941	0.0121	0.0045646376
ss117968520		6	101.68415	0.7958	0.1838	0.668265	0.0100	0.0032445798
	rs29009653	6	101.738361	0.2192	8.9601	0.002857	0.0656	0.0224594679
ss86338249		6	101.790798	0.6483	10.9898	0.000964	0.0647	0.0295089187
ss61500128	*rs41597906*	6	101.82201	0.6533	8.8767	0.002989	0.0584	0.0264354456
ss64946099	*rs43042688*	6	101.933613	0.2113	0.6063	0.436457	0.0182	0.0060743524
ss86327652		6	101.96304	0.7872	0.4052	0.524615	0.0149	0.0049882380
ss86325486		6	101.998968	0.4670	10.4036	0.001317	0.0598	0.0297857661
ss64290328	*rs42562582*	6	102.024779	0.9527	4.4969	0.034309	0.0922	0.0083084022
ss64290404	*rs42562620*	6	102.045048	0.3668	3.2016	0.074000	0.0328	0.0152411363
	rs29018486	6	102.16327	0.6003	11.4812	0.000743	0.0622	0.0298526217
ss61495948	*rs41594506*	6	102.229156	0.2722	1.5737	0.210091	0.0253	0.0100307205
ss86301311		6	102.270383	0.2751	20.4035	0.000007	0.0891	0.0355326295
ss61566801	*rs41667421*	6	102.295201	0.4943	1.3170	0.251518	0.0214	0.0107024413
ss86321079		6	102.320475	0.5093	1.0249	0.311706	0.0189	0.0094587768
ss86312538		6	102.342064	0.4907	1.0249	0.311706	0.0189	0.0094587768
ss86294392		6	102.40096	0.2887	5.8765	0.015598	0.0480	0.0197266901
ss61523435	*rs41620902*	6	102.641891	0.7650	0.4200	0.517163	0.0142	0.0051094609

ss86335966		6	102.668375	0.3732	0.0008	0.977951	0.0005	0.0002430412
ss86333121		6	102.693144	0.3711	0.0027	0.958245	0.0010	0.0004612830
ss64808610	*rs42902741*	6	102.756312	0.2049	4.2923	0.038653	0.0488	0.0158988923
ss65398832	*rs43488014*	6	102.911972	0.3689	5.1177	0.023991	0.0424	0.0197566440
ss86338183		6	102.962587	0.2443	21.8377	0.000004	0.0962	0.0355145595
ss86340787		6	103.005874	0.5129	1.1478	0.284387	0.0196	0.0097881797
ss86341689		6	103.030961	0.7006	0.0949	0.758106	0.0061	0.0025700009
ss65398528	*rs43484810*	6	103.079732	0.4090	5.2938	0.021697	0.0418	0.0201992034
ss86338508		6	103.105926	0.2278	1.4445	0.229815	0.0251	0.0088366170
ss65398501	*rs43484783*	6	103.15233	0.2693	20.1230	0.000008	0.0931	0.0366576890
ss61557964	*rs41654059*	6	103.176852	0.4033	1.1941	0.274882	0.0208	0.0099954760
ss86339069		6	103.225703	0.4663	0.4509	0.502119	0.0125	0.0062133721
ss86304772		6	103.302474	0.8181	1.7937	0.180918	0.0317	0.0094218745
ss65398271	*rs43482454*	6	103.340146	0.4319	2.0632	0.151343	0.0259	0.0127017822
ss61491711	*rs41589997*	6	103.38199	0.3911	6.6427	0.010161	0.0465	0.0221466944
ss86336733		6	103.43118	0.7658	4.2129	0.040491	0.0444	0.0159340697
ss117968344		6	103.508689	0.5179	9.2225	0.002480	0.0543	0.0270923567
ss117968783		6	103.531866	0.6211	8.4353	0.003797	0.0539	0.0253760008
ss117968341		6	103.570611	0.3130	0.0111	0.916229	0.0021	0.0008954383
ss61558014		6	103.612625	0.4133	5.3073	0.021530	0.0427	0.0207164273
ss117975430		6	103.627411	0.4398	7.3552	0.006852	0.0491	0.0241844643
ss117968432		6	103.640466	0.5530	6.3049	0.012266	0.0455	0.0224743674
	rs29015928	6	103.676859	0.4928	19.7241	0.000010	0.0804	0.0401868545
ss117968796		6	103.722889	0.1827	1.4718	0.225466	0.0284	0.0084842879
ss117968239		6	103.75273	0.1834	1.6836	0.194877	0.0304	0.0090948261
ss86337136		6	103.77829	0.5888	1.9622	0.161726	0.0261	0.0126386559
ss61558007	*rs41654101*	6	103.816257	0.1827	4.5762	0.032767	0.0505	0.0150662732
ss86305580		6	103.835959	0.6970	4.4088	0.036113	0.0422	0.0178215723
	rs29015919	6	103.878116	0.6805	32.8729	0.000000	0.1127	0.0489952899
ss117968039		6	103.955461	0.1712	10.6075	0.001181	0.0777	0.0220616026
ss117968237		6	103.978459	0.0623	13.3662	0.000276	0.1357	0.0158549535
ss117968058		6	104.006008	0.5967	13.2091	0.000299	0.0679	0.0326757884
ss117967996		6	104.117721	0.0745	4.9465	0.026464	0.0800	0.0110345260
ss117968679		6	104.134072	0.0745	4.9465	0.026464	0.0800	0.0110345260
ss61494273	*rs41595677*	6	104.161908	0.5129	4.6240	0.031871	0.0398	0.0198773157
ss117968540		6	104.266362	0.7622	8.5928	0.003486	0.0647	0.0234624875
ss117968173		6	104.316904	0.1762	3.7195	0.054187	0.0465	0.0135086432
ss117968662		6	104.340019	0.3632	27.8996	0.000000	0.1001	0.0462962988
ss86305350		6	104.363348	0.3625	27.8703	0.000000	0.1003	0.0463429293
ss117968659		6	104.406133	0.8460	6.7341	0.009658	0.0673	0.0175500019
ss86323811		6	104.4367	0.1375	0.3366	0.561989	0.0161	0.0038293489
ss117968658		6	104.462242	0.8474	2.4002	0.121778	0.0402	0.0104004767
ss117968235		6	104.532715	0.1576	3.1487	0.076426	0.0444	0.0118002496
ss86336874		6	104.567927	0.6053	0.0780	0.780072	0.0054	0.0025609622
ss117967951		6	104.612743	0.3489	2.9984	0.083791	0.0340	0.0154479123
ss61491699	*rs41589985*	6	104.669379	0.1440	9.7917	0.001826	0.0837	0.0206419005
ss61557982	*rs41654077*	6	104.771265	0.4420	11.9508	0.000579	0.0637	0.0314425310

ss117968774		6	104.882599	0.9599	0.0571	0.811163	0.0110	0.0008473369
ss86301206		6	104.932315	0.9205	18.1200	0.000024	0.1464	0.0214235460
ss86277309		6	104.956061	0.9191	16.4681	0.000055	0.1387	0.0206412006
ss86300399		6	105.078642	0.8159	3.5777	0.058976	0.0453	0.0135951687
ss117968072		6	105.100588	0.1798	5.9945	0.014596	0.0586	0.0172971776
ss86310753		6	105.177567	0.3868	9.9189	0.001706	0.0584	0.0276966186
ss61557978	*rs41654073*	6	105.211364	0.1146	0.6421	0.423228	0.0236	0.0047796382
ss117968061		6	105.235485	0.0516	3.6098	0.057854	0.0775	0.0075782612
	rs29022867	6	105.325397	0.9563	4.6828	0.030805	0.0991	0.0082855454
ss65399190	*rs43483670*	6	105.348076	0.7414	18.4095	0.000020	0.0882	0.0338116793
ss86290408		6	105.402482	0.1812	27.3032	0.000000	0.1240	0.0368113347
	rs29019638	6	105.500024	0.7314	1.3295	0.249293	0.0231	0.0090835301
ss86297867		6	105.525596	0.4470	0.0175	0.894649	0.0024	0.0011927260
ss117968229		6	105.547129	0.6676	0.0129	0.909682	0.0023	0.0010070214
ss117968022		6	105.572119	0.3804	14.3656	0.000164	0.0690	0.0325470002
ss117968665		6	105.62252	0.5953	2.7913	0.095225	0.0312	0.0150229619
ss117968761		6	105.669098	0.5609	6.7902	0.009362	0.0473	0.0233219102
ss117968262		6	105.689824	0.3345	6.7518	0.009564	0.0503	0.0223997939
ss65401348	*rs43485280*	6	105.769809	0.4434	0.0065	0.935712	0.0015	0.0007515759
ss117968866		6	105.801932	0.9413	0.3598	0.548802	0.0236	0.0026063215
ss65402394	*rs43485109*	6	105.84371	0.5809	4.2083	0.040600	0.0382	0.0185783955
ss117963867		6	105.866911	0.0544	0.6748	0.411661	0.0342	0.0035245086
	rs29022956	6	106.000636	0.9534	1.8949	0.169091	0.0614	0.0054524052
ss117967964		6	106.044403	0.4384	2.0341	0.154249	0.0268	0.0131927295
ss65400945	*rs43480825*	6	106.0665	0.3145	4.3599	0.037158	0.0417	0.0179761870
ss61558018	*rs41654112*	6	106.088918	0.4577	2.4334	0.119227	0.0294	0.0145863612
ss61516611	*rs41617012*	6	106.187786	0.1798	7.3395	0.006911	0.0673	0.0198451215
ss86316584		6	106.219046	0.6597	3.9499	0.047266	0.0384	0.0172284276
ss86336887		6	106.352941	0.2085	41.6450	0.000000	0.1440	0.0475252717
ss61558023	*rs41654115*	6	106.399637	0.0659	11.7203	0.000654	0.1274	0.0156834126
ss65401357	*rs43485289*	6	106.447445	0.5215	17.1639	0.000039	0.0767	0.0382849691
ss86321126		6	106.496879	0.4069	7.3524	0.006863	0.0508	0.0245333352
ss61558026	*rs41654118*	6	106.537207	0.7314	2.7936	0.095091	0.0345	0.0135581915
ss86324073		6	106.579005	0.6325	10.0200	0.001616	0.0613	0.0284739656
ss86301083		6	106.674608	0.4398	2.3894	0.122613	0.0297	0.0146404888
ss65401502	*rs43486122*	6	106.698098	0.1755	7.5503	0.006155	0.0652	0.0188574359
ss86314349		6	106.710494	0.2314	18.1843	0.000023	0.0922	0.0328092974
ss86311681		6	106.733889	0.5365	16.4189	0.000056	0.0747	0.0371432588
ss65401802	*rs43489322*	6	106.756953	0.0888	4.7624	0.029421	0.0695	0.0112499153
ss86307535		6	106.778582	0.7428	2.9491	0.086370	0.0373	0.0142412619
ss86280373		6	106.818601	0.9269	0.1567	0.692368	0.0138	0.0018732231
ss86296492		6	106.906972	0.5967	0.3791	0.538311	0.0117	0.0056159255
ss86286135		6	106.943666	0.5093	2.6797	0.102086	0.0301	0.0150592874
ss86308603		6	106.987214	0.6891	1.3881	0.239131	0.0249	0.0106640436
ss86284980		6	107.013335	0.3546	7.1913	0.007500	0.0534	0.0244350509
ss61478614	*rs41577000*	6	107.103152	0.5458	1.3363	0.248088	0.0223	0.0110358850
ss86283357		6	107.135329	0.5301	23.9245	0.000001	0.0875	0.0435937641

ss86284562		6	107.181569	0.6461	4.1748	0.041405	0.0394	0.0180175374
ss86335799		6	107.203483	0.5086	13.1908	0.000302	0.0665	0.0332628587
ss86300887		6	107.243011	0.3539	2.1267	0.145200	0.0272	0.0124232239
ss86329546		6	107.269201	0.3360	16.1622	0.000064	0.0777	0.0346497791
	rs29022499	6	107.336375	0.7622	13.8484	0.000214	0.0791	0.0286832152
ss61497867	*rs43705689*	6	107.358156	0.5265	0.3749	0.540561	0.0112	0.0056004479
ss86340251		6	107.444429	0.5516	9.1593	0.002566	0.0575	0.0284231779
ss86297281		6	107.469545	0.7063	8.4043	0.003861	0.0604	0.0250617249
ss86330165		6	107.489856	0.7607	9.8808	0.001741	0.0663	0.0241433041
ss86290123		6	107.565592	0.1583	11.7761	0.000635	0.0876	0.0233320041
ss86312877		6	107.622094	0.1590	11.4483	0.000756	0.0863	0.0230707974
ss86324720		6	107.653226	0.5516	0.5408	0.462366	0.0134	0.0066083173
ss86338224		6	107.677125	0.7142	0.6758	0.411331	0.0166	0.0067925927
ss61491736	*rs41590022*	6	107.810804	0.1275	2.3180	0.128336	0.0418	0.0092969770
ss86288432		6	107.853754	0.7235	30.0017	0.000000	0.1124	0.0449525655
ss86319504		6	107.896576	0.5731	10.9189	0.001001	0.0618	0.0302444737
ss61523678	*rs41622841*	6	108.154713	0.8961	8.3860	0.003900	0.0880	0.0163821622
ss86341209		6	108.202551	0.6046	14.9739	0.000119	0.0723	0.0345740107
ss86284128		6	108.228419	0.3940	15.3886	0.000096	0.0732	0.0349371469
ss86274615		6	108.26669	0.7285	1.9335	0.164820	0.0286	0.0113287011
ss61558072	*rs41655064*	6	108.330908	0.9004	6.5819	0.010510	0.0799	0.0143314838
ss86318116		6	108.370732	0.0967	6.5833	0.010502	0.0807	0.0141055027
ss61558075	*rs41655067*	6	108.50673	0.6662	34.2515	0.000000	0.1128	0.0501494586
	rs29024349	6	108.55427	0.2980	8.3836	0.003905	0.0594	0.0248563815
ss86341779		6	108.591754	0.0530	0.1588	0.690351	0.0166	0.0016634635
ss61558073	*rs41655065*	6	108.621298	0.8897	0.2639	0.607630	0.0154	0.0030186820
ss86303759		6	108.659926	0.1855	13.1101	0.000315	0.0852	0.0257426259
ss61491747	*rs41590931*	6	108.682637	0.9370	3.3725	0.066720	0.0691	0.0081605610
ss86309723		6	108.743094	0.7980	6.3229	0.012143	0.0573	0.0184788570
ss86274526		6	108.79252	0.8188	23.1061	0.000002	0.1154	0.0342429500
ss61558088	*rs41655080*	6	108.928946	0.4126	11.5823	0.000704	0.0641	0.0310770967
ss65402905	*rs43490419*	6	109.041127	0.4678	3.5629	0.059501	0.0364	0.0181406543
ss86315365		6	109.07304	0.2479	1.0664	0.302109	0.0217	0.0080939266
ss65402965	*rs43482079*	6	109.107646	0.2751	5.0563	0.024848	0.0463	0.0184574818
ss86337598		6	109.14235	0.4470	0.2935	0.588151	0.0103	0.0050932411
ss64716796	*rs42814038*	6	109.308467	0.5645	3.1103	0.078239	0.0326	0.0160298450
ss86293015		6	109.328786	0.2607	0.0824	0.774208	0.0059	0.0022772772
ss61558060	*rs41655052*	6	109.362266	0.1877	5.3527	0.020981	0.0541	0.0164846668
ss86295170		6	109.396634	0.9348	2.1428	0.143689	0.0538	0.0065570018
ss86327352		6	109.51217	0.5394	1.4152	0.234599	0.0221	0.0109684175
ss86332283		6	109.544352	0.9284	3.0790	0.079751	0.0643	0.0085468821
ss65410324	*rs43501532*	6	109.575919	0.4520	1.3973	0.237578	0.0219	0.0108394164
	rs29012008	6	109.596224	0.3732	2.0186	0.155835	0.0269	0.0125912380
	rs29012203	6	109.619481	0.5695	3.6485	0.056530	0.0353	0.0173032759
ss86312936		6	109.651675	0.5501	2.0565	0.152003	0.0267	0.0132059254
ss86327085		6	109.680595	0.3367	2.8514	0.091745	0.0329	0.0146749813
ss86273508		6	109.807844	0.6905	0.0484	0.825892	0.0044	0.0018921407

ss86303970		6	109.847952	0.7457	0.0113	0.915333	0.0023	0.0008735124
ss61558109		6	109.999231	0.7357	8.1692	0.004388	0.0620	0.0241080668
ss86335818		6	110.024215	0.0831	0.0256	0.873020	0.0055	0.0008347863
ss65403583	*rs43487894*	6	110.053357	0.7436	9.1526	0.002575	0.0660	0.0251603927
ss86311577		6	110.144034	0.4413	2.3401	0.126535	0.0282	0.0139029528
ss86341297		6	110.182875	0.5344	0.3550	0.551490	0.0109	0.0054476474
ss86297938		6	110.285671	0.2994	0.3203	0.571616	0.0112	0.0047061130
ss65404469	*rs43487459*	6	110.30853	0.4355	2.0457	0.153086	0.0262	0.0128601592
ss65404405	*rs43486695*	6	110.333199	0.7199	1.5768	0.209640	0.0260	0.0104888427
ss86336615		6	110.418684	0.7729	0.7118	0.399139	0.0191	0.0067176639
ss65404250	*rs43486149*	6	110.452258	0.2529	1.3783	0.240801	0.0249	0.0093955182
ss86299883		6	110.49219	0.6476	5.2770	0.021905	0.0443	0.0202151204
ss65404388	*rs43486678*	6	110.525067	0.5473	0.1439	0.704579	0.0069	0.0034225802
ss86341683		6	110.545568	0.9076	6.6335	0.010213	0.0828	0.0138919297
ss117968716		6	110.570778	0.3997	3.2849	0.070349	0.0342	0.0164117430
ss86312279		6	110.59186	0.3016	0.5983	0.439476	0.0155	0.0065374411
ss86302140		6	110.633095	0.4076	0.7386	0.390421	0.0164	0.0079029765
ss61513560		6	110.718634	0.1189	3.3127	0.069176	0.0538	0.0112793198
ss61470983	*rs41566556*	6	110.785732	0.2794	2.6309	0.105254	0.0339	0.0136487230
ss61513567	*rs41613935*	6	110.929682	0.6125	0.9486	0.330413	0.0187	0.0088979413
ss61513583	*rs41613951*	6	110.998873	0.5931	4.9180	0.026900	0.0403	0.0194327095
ss61513584	*rs41613952*	6	111.019298	0.8890	1.5387	0.215230	0.0377	0.0074427746
ss86300146		6	111.043978	0.7357	1.1024	0.294098	0.0214	0.0083199009
ss63725891	*rs42326694*	6	111.07879	0.2385	1.1862	0.276465	0.0234	0.0085089429
ss86312587		6	111.143796	0.2264	2.6590	0.103417	0.0356	0.0124646075
ss61517496	*rs41616068*	6	111.174814	0.2292	1.4552	0.228101	0.0263	0.0092992690
ss61517488	*rs41616060*	6	111.212681	0.5372	0.0040	0.949675	0.0012	0.0005780629
ss61517487	*rs41616059*	6	111.383112	0.6712	1.8042	0.179638	0.0265	0.0116966659
ss61517485	*rs41616057*	6	111.414805	0.1913	3.1422	0.076730	0.0403	0.0124805030
ss61472890	*rs41574609*	6	111.508077	0.4441	0.0277	0.867957	0.0031	0.0015137711
	rs29009956	6	111.541597	0.8789	1.7853	0.181940	0.0386	0.0082181628
ss63815147	*rs42365968*	6	111.573895	0.4413	0.0017	0.966788	0.0008	0.0003790124
ss65404785	*rs43488925*	6	111.612203	0.3295	0.0775	0.780767	0.0053	0.0023296913
ss63814839	*rs42364715*	6	111.657009	0.8983	1.1528	0.283341	0.0322	0.0058919763
	rs29022149	6	111.703786	0.8438	0.7693	0.380741	0.0224	0.0059003777
ss64214078	*rs42529422*	6	111.736127	0.1282	0.8470	0.357725	0.0260	0.0058098865
ss65404727	*rs43488867*	6	111.867774	0.1827	5.5085	0.019203	0.0554	0.0165408997
ss65404533	*rs43487523*	6	111.903071	0.7235	3.9134	0.048297	0.0408	0.0163191343
ss86312325		6	111.967167	0.8166	0.0453	0.831519	0.0051	0.0015390986
ss64916433	*rs43015922*	6	112.027324	0.1074	1.6121	0.204624	0.0383	0.0073516158
ss64895547	*rs42988832*	6	112.052548	0.4549	14.1054	0.000187	0.0693	0.0343592465
ss86304852		6	112.086305	0.7686	1.9091	0.167501	0.0296	0.0105158605
ss64799168	*rs42897411*	6	112.130182	0.1304	0.4589	0.498382	0.0190	0.0043187993
ss64816575	*rs42912489*	6	112.162814	0.6791	2.4340	0.119183	0.0313	0.0136351678
ss86316735		6	112.242225	0.9298	12.7332	0.000384	0.1295	0.0169003311
ss86324235		6	112.320267	0.1232	0.4099	0.522209	0.0184	0.0039799419
ss61527562	*rs41624219*	6	112.484937	0.1440	9.4997	0.002136	0.0829	0.0204322683

ss61567741	*rs41665145*	6	112.506348	0.8890	0.0115	0.914552		0.0031	0.0006208502
ss86297932		6	112.566375	0.3360	0.1640	0.685629		0.0081	0.0036161851
ss64668340	*rs42766762*	6	112.63939	0.0838	9.3031	0.002375		0.1025	0.0157450661
ss86317984		6	112.694128	0.1511	12.2523	0.000494		0.0898	0.0230497296
ss61558139	*rs41655124*	6	112.714177	0.7557	0.0536	0.817012		0.0049	0.0018047162
ss64710733	*rs42808728*	6	112.794512	0.5688	1.3621	0.243570		0.0218	0.0106705027
ss63454570	*rs42235384*	6	112.849904	0.1719	0.5039	0.478049		0.0180	0.0051127802
ss86323008		6	112.970463	0.6497	0.5508	0.458252		0.0146	0.0066365884
	rs29025321	6	113.02266	0.1182	0.1325	0.715923		0.0107	0.0022370894
ss61474056	*rs41566668*	6	113.137422	0.3918	0.0046	0.945993		0.0013	0.0005993995
ss86341268		6	113.166607	0.1941	0.1482	0.700339		0.0093	0.0028976507
ss86332198		6	113.225037	0.6691	0.1675	0.682485		0.0082	0.0036373091
ss63455185	*rs42236483*	6	113.255903	0.9477	0.2484	0.618352		0.0205	0.0020368142
ss61520195	*rs41611124*	6	113.296732	0.4441	10.9543	0.000982		0.0609	0.0300685458
ss86331797		6	113.326851	0.7292	4.7092	0.030339		0.0454	0.0179428177
ss86338453		6	113.364026	0.7020	0.2182	0.640568		0.0098	0.0041208637
ss63455500	*rs42236787*	6	113.399931	0.9692	0.0269	0.869664		0.0088	0.0005275987
ss86341119		6	113.430134	0.5215	4.1723	0.041466		0.0384	0.0191835678
ss63455515	*rs42236792*	6	113.456838	0.0308	0.0269	0.869664		0.0088	0.0005275987
ss61520204	*rs41612233*	6	113.492276	0.3474	7.9327	0.004992		0.0541	0.0245465162
ss86303893		6	113.537074	0.4620	5.7560	0.016695		0.0444	0.0220952827
ss86326805		6	113.582943	0.2242	0.7494	0.386949		0.0195	0.0067731757
ss86331696		6	113.618851	0.7085	0.5399	0.462712		0.0152	0.0062775070
ss86338425		6	113.657945	0.9176	0.0054	0.941606		0.0025	0.0003734884
ss61474057	*rs41566669*	6	113.70137	0.5573	6.0768	0.013937		0.0463	0.0228667800
ss86339548		6	113.747005	0.3961	15.7973	0.000078		0.0729	0.0348972321
	rs29025538	6	113.779025	0.3689	0.0859	0.769585		0.0056	0.0026145825
ss63456922	*rs42236164*	6	113.799698	0.5122	0.0108	0.917377		0.0019	0.0009614372
	rs29019592	6	113.830596	0.3861	0.2503	0.617041		0.0096	0.0045354175
ss117968700		6	113.915652	0.4735	0.2831	0.594822		0.0101	0.0050258141
ss64771928	*rs42868916*	6	113.959899	0.7529	0.0677	0.794768		0.0054	0.0020043621
ss61466848	*rs41573388*	6	113.982482	0.1891	0.0001	0.991222		0.0003	0.0000777616
ss64919224	*rs43014084*	6	114.009606	0.7049	0.5625	0.453505		0.0154	0.0064194806
ss65106598	*rs43195323*	6	114.021675	0.2013	0.3614	0.547915		0.0135	0.0043449021
ss117968844		6	114.089012	0.6160	0.8043	0.370127		0.0173	0.0081914359
ss61469703	*rs41572200*	6	114.120926	0.4040	0.2576	0.611901		0.0096	0.0046190413
ss86302179		6	114.197915	0.4405	0.1132	0.736658		0.0062	0.0030792416
ss61469705	*rs41572202*	6	114.240707	0.2149	0.0020	0.964556		0.0010	0.0003355748
ss61477736	*rs41579565*	6	114.311884	0.7292	1.0579	0.304042		0.0219	0.0086303802
ss86330605		6	114.349274	0.2736	3.6411	0.056782		0.0394	0.0156782677
ss65405026	*rs43484165*	6	114.382854	0.2085	0.1253	0.723498		0.0081	0.0026726355
ss61517087	*rs41611392*	6	114.476759	0.2099	2.0917	0.148552		0.0325	0.0107690305
ss64923058	*rs43015979*	6	114.50684	0.5279	0.9527	0.329381		0.0176	0.0087932567
ss86300358		6	114.544618	0.2292	0.0001	0.991724		0.0002	0.0000806346
ss61522756	*rs41621527*	6	114.57794	0.6533	1.1426	0.285480		0.0209	0.0094872641
ss86336901		6	114.606833	0.4097	0.1802	0.671354		0.0083	0.0040359522
ss86319091		6	114.633447	0.5337	0.4647	0.495654		0.0127	0.0063200702

ss86340027		6	114.656553	0.0752	0.8795	0.348653	0.0311	0.0043306834
ss61491766		6	114.717018	0.3417	0.8001	0.371382	0.0175	0.0078802873
ss65405807	*rs43489344*	6	114.786768	0.2722	0.3026	0.582428	0.0114	0.0045096127
	rs29022851	6	114.817705	0.3381	2.1299	0.144902	0.0290	0.0130019666
ss117968509		6	114.86842	0.5466	1.8201	0.177742	0.0252	0.0124679135
ss65405758	*rs43488496*	6	114.893889	0.3381	4.2656	0.039261	0.0402	0.0179733091
ss65405649	*rs43488087*	6	114.961173	0.1418	0.2205	0.638802	0.0126	0.0030632126
ss61558147	*rs41655732*	6	114.981203	0.8489	1.1454	0.284895	0.0289	0.0074073586
ss86273669		6	115.010286	0.7729	1.2604	0.261955	0.0248	0.0087106929
ss61491774	*rs41590958*	6	115.030645	0.1877	12.4660	0.000442	0.0844	0.0257317923
ss65405530	*rs43487368*	6	115.06277	0.2665	8.8924	0.002964	0.0613	0.0239461243
ss86308042		6	115.101936	0.5573	14.1164	0.000186	0.0683	0.0336929615
ss86285222		6	115.161753	0.5380	2.8711	0.090628	0.0311	0.0154711819
ss61558143	*rs41655128*	6	115.220007	0.5279	0.6904	0.406323	0.0155	0.0077458273
	rs29023406	6	115.2537	0.2034	0.0225	0.880858	0.0034	0.0011074259
ss86279612		6	115.332736	0.8259	1.5574	0.212472	0.0304	0.0087446769
ss61474943	*rs41573529*	6	115.375178	0.6676	5.5175	0.019105	0.0456	0.0202279050
ss64776943	*rs42875794*	6	115.408712	0.3489	4.1709	0.041499	0.0397	0.0180270178
ss86334147		6	115.430946	0.8940	6.7328	0.009665	0.0778	0.0147400277
ss64637570	*rs42732852*	6	115.469948	0.4083	0.6021	0.438038	0.0143	0.0069179349
ss86330408		6	115.504832	0.7693	0.6787	0.410308	0.0179	0.0063622033
ss86336313		6	115.546293	0.9198	0.0304	0.861651	0.0058	0.0008505659
ss86336321		6	115.61247	0.5802	1.1871	0.276297	0.0200	0.0097586333
ss86274660		6	115.635485	0.1769	1.7687	0.183980	0.0323	0.0094030582
ss86317555		6	115.707876	0.6390	0.3428	0.558385	0.0113	0.0052190480
ss61488781	*rs41587025*	6	115.754208	0.5745	1.5473	0.213957	0.0231	0.0112913223
ss86340331		6	115.813747	0.9090	2.2599	0.133219	0.0488	0.0080772680
ss86339051		6	115.862936	0.8596	6.1348	0.013491	0.0662	0.0159778263
ss65407061	*rs43488774*	6	115.885782	0.1074	0.6302	0.427553	0.0240	0.0046005192
ss86321495		6	115.970494	0.1304	15.8847	0.000074	0.1076	0.0243926276
ss64010212	*rs42435779*	6	116.044882	0.3940	22.7970	0.000002	0.0870	0.0415467924
	rs29014035	6	116.092429	0.6361	3.0360	0.081880	0.0336	0.0155341085
ss61491817	*rs41590982*	6	116.228739	0.3639	0.1110	0.739147	0.0064	0.0029439917
ss61504271	*rs41605048*	6	116.268659	0.8961	15.2300	0.000104	0.1137	0.0211634738
ss86315979		6	116.316931	0.2686	0.7745	0.379127	0.0177	0.0069548277
ss61507587	*rs41607879*	6	116.3564	0.9011	24.8546	0.000001	0.1496	0.0266477525
ss86336470		6	116.376452	0.5881	13.4271	0.000267	0.0675	0.0326934052
ss86336374		6	116.415691	0.9054	0.5065	0.476895	0.0219	0.0037463327
ss117968629		6	116.526743	0.0681	4.6942	0.030603	0.0810	0.0102777199
ss63928511	*rs42340214*	6	116.560383	0.2822	1.9086	0.167567	0.0284	0.0114911294
ss86299790		6	116.595148	0.0681	11.5207	0.000727	0.1243	0.0157697836
ss63928206	*rs42339561*	6	116.615923	0.9255	4.0805	0.043763	0.0691	0.0095313357
ss117968588		6	116.76859	0.8087	0.0291	0.864557	0.0041	0.0012788848
ss86319558		6	116.910839	0.5795	0.0840	0.772093	0.0054	0.0026213517
ss86299169		6	116.973665	0.2851	1.2463	0.264643	0.0227	0.0092418654
	rs29017692	6	116.998217	0.4262	0.8423	0.359051	0.0170	0.0083025208
ss86315897		6	117.061741	0.3725	0.0021	0.963100	0.0009	0.0004093378

ss86307340		6	117.103361	0.5623	0.0275	0.868344	0.0031	0.0015209116
ss61558266	*rs41655825*	6	117.128356	0.2665	0.0073	0.931933	0.0017	0.0006662312
ss65409813	*rs43494122*	6	117.169152	0.9126	0.0109	0.916996	0.0034	0.0005345301
ss65040853	*rs43132988*	6	117.241565	0.3861	0.0003	0.985585	0.0003	0.0001587401
ss117968838		6	117.300222	0.4176	0.3374	0.561503	0.0107	0.0051987561
ss86324975		6	117.325619	0.7779	0.0047	0.945522	0.0015	0.0005160844
ss61469202	*rs41567812*	6	117.517597	0.0766	1.6426	0.200397	0.0443	0.0062767821
ss65033017	*rs43126758*	6	117.584429	0.5616	0.0359	0.849878	0.0034	0.0016854799
ss86282378		6	117.809567	0.1605	5.7654	0.016606	0.0598	0.0161201241
ss86317913		6	117.830308	0.2321	0.0354	0.850825	0.0040	0.0014293024
ss86339829		6	117.886285	0.2636	0.0723	0.788108	0.0055	0.0021465545
ss61529780	*rs41628387*	6	117.928864	0.7364	0.0723	0.788108	0.0055	0.0021465545
ss86321795		6	118.029315	0.1884	9.6109	0.002012	0.0725	0.0221645872
ss86291170		6	118.096014	0.2865	0.8536	0.355853	0.0189	0.0077429923
ss86307817		6	118.120364	0.6719	9.1801	0.002537	0.0590	0.0260268170
ss46526085	*rs41255232*	6	118.154736	0.4226	0.0001	0.990748	0.0002	0.0001049642
ss86341026		6	118.175859	0.1834	7.8805	0.005137	0.0663	0.0198589328
ss86340016		6	118.197106	0.1870	12.0745	0.000543	0.0812	0.0246820479
ss86296397		6	118.232279	0.1275	0.7645	0.382212	0.0239	0.0053259354
ss86318675		6	118.267987	0.1361	0.9012	0.342778	0.0252	0.0059258942
ss65407555	*rs43494465*	6	118.295066	0.1540	5.0451	0.025009	0.0563	0.0146682840
ss86307469		6	118.387391	0.6511	0.4881	0.485029	0.0137	0.0062328780
ss86338106		6	118.460274	0.8274	0.0506	0.822110	0.0056	0.0015907281
ss86339502		6	118.673772	0.1046	1.6998	0.192745	0.0407	0.0076138122
ss86305572		6	118.748365	0.3095	3.2024	0.073966	0.0354	0.0151134661
ss86303039		6	118.790442	0.4355	0.3151	0.574755	0.0103	0.0050570663
ss86291390		6	118.819518	0.6662	1.4119	0.235144	0.0225	0.0099977233
ss65407084	*rs43488797*	6	118.855816	0.1297	0.1140	0.735742	0.0093	0.0020990301
ss61525955	*rs41623953*	6	118.978623	0.8940	0.8142	0.367182	0.0272	0.0051468405
ss86283974		6	119.00319	0.8489	0.8447	0.358371	0.0236	0.0060652794
ss86301337		6	119.038392	0.2493	0.0491	0.824722	0.0048	0.0017992606
ss86337359		6	119.068909	0.3883	9.1413	0.002591	0.0575	0.0273175907
ss86310627		6	119.205071	0.5265	9.8659	0.001755	0.0567	0.0282559167
ss86307458		6	119.241601	0.4742	9.9887	0.001643	0.0569	0.0283884743
ss86286875		6	119.339223	0.2106	5.0330	0.025182	0.0510	0.0169726370
ss86309840		6	119.366212	0.3775	1.5980	0.206605	0.0241	0.0113258623
ss86280731		6	119.397215	0.9542	6.3178	0.012178	0.1126	0.0098470723
ss86302665		6	119.418044	0.4448	3.4452	0.063857	0.0351	0.0173219529
ss86293555		6	119.498445	0.4198	3.3614	0.067170	0.0328	0.0159697529
ss86325219		6	119.531088	0.3646	0.8823	0.347906	0.0181	0.0083746986
ss86304497		6	119.562018	0.4943	0.9385	0.333011	0.0175	0.0087438791
ss86295927		6	119.618063	0.8016	1.0090	0.315501	0.0232	0.0073757539
ss86339838		6	119.645887	0.3102	0.2673	0.605292	0.0104	0.0044561052
ss86324841		6	119.675062	0.3274	0.9053	0.341695	0.0186	0.0081909456
ss86285533		6	119.738213	0.3223	0.0782	0.779876	0.0054	0.0023665195
ss38331637	*rs29025744*	6	119.771391	0.2092	0.0088	0.925168	0.0021	0.0006967339
ss86335129		6	119.861471	0.9527	1.4615	0.227103	0.0504	0.0045365199

ss61558189	*rs41655774*	6	119.883824	0.6476	0.3664	0.545157	0.0119	0.0054155034
ss86297273		6	119.934419	0.2299	3.3677	0.066914	0.0392	0.0138867124
ss86328174		6	119.96392	0.8009	9.1051	0.002642	0.0694	0.0221314546
ss86329733		6	120.000305	0.9277	0.0597	0.807089	0.0085	0.0011374874
ss86285690		6	120.03899	0.6268	1.0464	0.306689	0.0188	0.0087824423
ss86302817		6	120.085691	0.6812	0.4933	0.482672	0.0138	0.0059831025
ss61497718	*rs43704741*	6	120.113165	0.7872	0.3631	0.546983	0.0135	0.0045198348
ss64486934	*rs42658517*	6	120.134591	0.3130	0.2155	0.642621	0.0095	0.0040871930
ss61513288	*rs41611571*	6	120.156646	0.2128	0.3631	0.546983	0.0135	0.0045198348
ss86298383		6	120.199153	0.0996	1.4031	0.236602	0.0353	0.0063214152
ss86319522		6	120.245524	0.1375	2.8658	0.090926	0.0438	0.0103886772
ss86314753		6	120.303386	0.5981	0.5167	0.472495	0.0136	0.0065311981
ss86303889		6	120.450993	0.1662	0.3009	0.583472	0.0137	0.0038086333
ss86289536		6	120.473416	0.6254	6.1738	0.013199	0.0481	0.0225446670
ss86292861		6	120.493765	0.2077	7.8678	0.005173	0.0616	0.0202664701
ss86285227		6	120.570413	0.5215	0.1914	0.661865	0.0081	0.0040604260
ss86284429		6	120.611396	0.2937	0.3738	0.541164	0.0123	0.0051118515
ss86333062		6	120.640219	0.1297	0.6423	0.423168	0.0221	0.0049850687
ss86319700		6	120.763629	0.2715	4.4754	0.034742	0.0446	0.0176608664
ss86328338		6	120.813917	0.2564	5.8336	0.015980	0.0517	0.0197204013
ss86309524		6	120.840427	0.5007	0.1960	0.658088	0.0081	0.0040521176
ss86290714		6	120.889754	0.7292	6.6098	0.010349	0.0521	0.0205943658
ss86341289		6	120.915574	0.5372	1.8315	0.176386	0.0257	0.0127568815
ss86333310		6	120.956557	0.1397	0.4128	0.520785	0.0170	0.0040872615
ss86340530		6	121.029687	0.6168	0.1438	0.704684	0.0072	0.0033895726
ss61473881	*rs41574391*	6	121.057132	0.1605	0.1007	0.751136	0.0079	0.0021406806
ss86310209		6	121.097896	0.4155	1.9109	0.167311	0.0260	0.0126138835
ss86274432		6	121.130115	0.1218	2.2079	0.137760	0.0425	0.0090917068
ss86336892		6	121.19725	0.8868	0.0312	0.859812	0.0052	0.0010533279
ss86300325		6	121.267774	0.5659	0.0395	0.842459	0.0037	0.0018305849
ss117963889		6	121.287816	0.7830	1.3760	0.241182	0.0261	0.0088679431
ss86295450		6	121.357124	0.2808	0.0313	0.859534	0.0037	0.0015105755
ss86329449		6	121.448284	0.2428	0.8880	0.346350	0.0203	0.0074512608
ss86289248		6	121.47158	0.3990	0.2021	0.653151	0.0086	0.0041234008
ss86284699		6	121.536835	0.3352	0.9053	0.341682	0.0187	0.0083201192
ss86340083		6	121.559686	0.1211	0.3527	0.552800	0.0175	0.0037259276
ss86339243		6	121.656386	0.7077	0.0005	0.981615	0.0005	0.0001988468
ss86283417		6	121.696216	0.2092	0.0065	0.935965	0.0018	0.0006082709
ss86331396		6	121.805255	0.5509	5.1594	0.023426	0.0431	0.0213088066
ss86293256		6	121.838565	0.3811	0.7561	0.384855	0.0168	0.0079109406
ss86297644		6	121.89539	0.3804	0.8996	0.343227	0.0183	0.0086431665
ss86310236		6	121.920646	0.4549	0.3378	0.561286	0.0108	0.0053801153
ss86292346		6	122.093343	0.6146	0.7822	0.376776	0.0173	0.0081814527
ss86324830		6	122.128942	0.4327	1.2869	0.257013	0.0211	0.0103829410
ss86327846		6	122.1561	0.5681	1.5109	0.219415	0.0229	0.0112321248
ss86303290		6	122.178912	0.3496	0.3780	0.538861	0.0121	0.0055021514
ss86336363		6	122.21213	0.8395	5.5891	0.018346	0.0597	0.0160846854

ss86292615		6	122.255359	0.8725	3.3347	0.068260		0.0507	0.0112841720
ss65411524	*rs43497903*	6	122.286509	0.3976	1.1245	0.289327		0.0200	0.0095874891
ss86326531		6	122.316075	0.4205	0.0004	0.984224		0.0004	0.0001790498
ss117968461		6	122.338005	0.5351	0.5101	0.475353		0.0131	0.0065208331
ss86320448		6	122.386739	0.4878	0.4371	0.508752		0.0120	0.0060031207
ss86302666		6	122.474493	0.5681	0.0191	0.890226		0.0026	0.0012777225
ss86326321		6	122.50974	0.2622	3.2544	0.071664		0.0383	0.0148051780
ss86318444		6	122.54336	0.6132	0.4129	0.520694		0.0123	0.0058407589
ss63836360	*rs42371917*	7	0.367862	0.2034	4.9671	0.026152		0.0511	0.0165658165
ss63836302	*rs42371888*	7	0.394766	0.2034	4.9671	0.026152		0.0511	0.0165658165
ss63836030	*rs42371152*	7	0.430234	0.7887	3.3677	0.066914		0.0416	0.0138625924
ss86316571		7	0.502837	0.6132	0.3278	0.567170		0.0112	0.0053251639
ss61518650	*rs41616502*	7	0.527291	0.8395	8.6548	0.003370		0.0751	0.0202313089
ss63835806	*rs42379740*	7	0.592664	0.4642	0.2144	0.643481		0.0087	0.0043452851
ss86292431		7	0.668774	0.0910	0.2002	0.654722		0.0138	0.0022827032
ss64682654	*rs42774439*	7	0.698096	0.6819	1.1166	0.291016		0.0209	0.0090449126
ss86327004		7	0.727998	0.7865	0.2495	0.617553		0.0113	0.0037894885
ss86337383		7	0.762393	0.5709	6.5201	0.010878		0.0457	0.0223811896
ss86292002		7	0.786856	0.3947	0.7694	0.380712		0.0170	0.0081110246
ss65415818	*rs43494255*	7	0.81203	0.4004	0.6293	0.427883		0.0151	0.0072595934
ss117969152		7	0.83903	0.3883	0.5244	0.469199		0.0141	0.0066877358
ss64650918	*rs42749054*	7	0.910018	0.2042	0.3429	0.558368		0.0136	0.0044220223
ss86337567		7	0.94451	0.8539	5.5151	0.019132		0.0608	0.0151833286
ss64898362	*rs42986586*	7	0.967501	0.8961	7.7255	0.005592		0.0879	0.0163661361
ss86337267		7	1.064696	0.3166	1.0464	0.306700		0.0207	0.0089778204
ss86308900		7	1.099146	0.1948	0.0850	0.770728		0.0066	0.0020724155
ss63191575	*rs42146001*	7	1.126788	0.2063	5.3586	0.020910		0.0542	0.0177569874
ss63192086	*rs42148072*	7	1.189304	0.8317	0.0060	0.938187		0.0020	0.0005464356
ss61516221	*rs41613340*	7	1.304083	0.2077	2.3601	0.124927		0.0352	0.0115891425
ss86301830		7	1.343221	0.2643	2.4182	0.120388		0.0318	0.0123515484
ss86282164		7	1.371738	0.7500	2.2328	0.135562		0.0304	0.0113953023
ss65413034	*rs43494687*	7	1.431994	0.5100	2.7593	0.097143		0.0309	0.0154550136
ss117963953		7	1.467373	0.1089	1.0995	0.294738		0.0301	0.0058504282
ss86291992		7	1.493916	0.3195	10.1516	0.001506		0.0627	0.0272440835
ss86336724		7	1.555932	0.4835	0.7378	0.390654		0.0159	0.0079607119
ss65412866	*rs43493530*	7	1.608265	0.4663	0.7251	0.394756		0.0161	0.0079972024
ss86321706		7	1.654299	0.4112	1.9239	0.165866		0.0262	0.0126747327
ss65204679	*rs43288658*	7	1.707072	0.2493	4.9598	0.026262		0.0473	0.0177084204
ss86313630		7	1.751275	0.6834	1.2268	0.268412		0.0230	0.0099376440
ss65204668	*rs43288647*	7	1.782963	0.5444	10.3967	0.001321		0.0598	0.0296472642
ss86336340		7	1.803678	0.4699	0.0091	0.923865		0.0018	0.0008927092
ss86314004		7	1.853587	0.2729	0.2263	0.634431		0.0100	0.0039789622
ss86327261		7	1.911167	0.1397	0.0047	0.945189		0.0018	0.0004366110
ss86325772		7	1.93648	0.7693	0.9377	0.333198		0.0211	0.0074814406
ss86312518		7	1.981464	0.3818	1.3483	0.245966		0.0223	0.0105141354
ss86289421		7	2.004773	0.2579	0.2725	0.601797		0.0110	0.0042279421
ss86337673		7	2.030864	0.5408	0.0037	0.951826		0.0011	0.0005679419

ss86338462		7	2.081522	0.6741	0.3870	0.534067		0.0124	0.0054314639
ss64396582	*rs42616635*	7	2.137412	0.2321	1.4722	0.225409		0.0267	0.0095125416
	rs29009679	7	2.190302	0.6390	0.5668	0.451773		0.0149	0.0068540131
ss86306914		7	2.287323	0.4391	16.5870	0.000052		0.0750	0.0369266183
ss64650498	*rs42746836*	7	2.310382	0.6433	2.3803	0.123330		0.0295	0.0135309126
ss65020203	*rs43110173*	7	2.35576	0.3761	2.7107	0.100130		0.0316	0.0148437614
ss86320711		7	2.419766	0.2371	0.4111	0.521615		0.0139	0.0050371173
ss65415298	*rs43499630*	7	2.447659	0.3274	0.4836	0.487011		0.0139	0.0061260798
ss65413546	*rs43497398*	7	2.501139	0.3345	1.1255	0.289104		0.0212	0.0094330452
	rs29020997	7	2.523562	0.6375	0.9334	0.334324		0.0193	0.0089042794
ss61559721	*rs41658739*	7	2.562749	0.8009	8.1161	0.004517		0.0668	0.0212988423
ss86311894		7	2.594917	0.4277	0.6969	0.404105		0.0160	0.0078087854
ss86326158		7	2.640092	0.7830	0.4923	0.483132		0.0159	0.0054064261
ss86338801		7	2.661218	0.6282	0.0017	0.967361		0.0008	0.0003697193
ss86332390		7	2.683387	0.0860	6.5687	0.010588		0.0845	0.0132842157
ss61568115	*rs41661420*	7	2.718022	0.3496	2.2334	0.135507		0.0287	0.0130611390
ss86276727		7	2.774563	0.7106	0.0021	0.963732		0.0010	0.0003935425
ss86326748		7	2.795395	0.1562	1.0159	0.313838		0.0263	0.0069243096
ss86311831		7	2.821397	0.6547	0.0049	0.944349		0.0014	0.0006166692
ss65414151	*rs43496892*	7	2.873892	0.3854	1.9659	0.161335		0.0266	0.0126007993
ss86310587		7	2.921636	0.6905	1.0369	0.308887		0.0207	0.0088318523
ss86325250		7	2.953542	0.4420	0.0379	0.845741		0.0036	0.0017561968
ss86339005		7	2.996415	0.5838	2.2087	0.137688		0.0278	0.0134851837
ss61491933	*rs41591996*	7	3.025284	0.1640	2.8314	0.092888		0.0424	0.0116162260
ss86314492		7	3.077085	0.7930	6.5000	0.011001		0.0575	0.0188735764
ss86316564		7	3.110604	0.1948	2.8976	0.089160		0.0391	0.0122737300
ss65414040	*rs43496181*	7	3.140731	0.0845	0.0312	0.859879		0.0058	0.0009036912
ss86318114		7	3.168296	0.7529	10.6420	0.001159		0.0681	0.0253334599
ss65414000	*rs43496141*	7	3.20162	0.4499	0.9505	0.329928		0.0180	0.0089130772
ss86338441		7	3.27493	0.4799	1.0383	0.308578		0.0182	0.0090806932
ss86341229		7	3.296249	0.5143	1.1337	0.287361		0.0191	0.0095287610
ss65413964	*rs43495318*	7	3.318915	0.2450	1.2198	0.269788		0.0240	0.0088807724
ss86296141		7	3.365021	0.5974	4.3330	0.037746		0.0391	0.0188210038
ss86335680		7	3.415388	0.7980	2.1693	0.141242		0.0340	0.0109636701
ss65413663	*rs43492217*	7	3.448582	0.8059	2.3380	0.126705		0.0356	0.0111307495
ss86298130		7	3.487776	0.4119	0.0991	0.752964		0.0058	0.0028283408
ss61558436	*rs41656888*	7	3.533552	0.8102	0.0426	0.836505		0.0050	0.0015231584
ss86319902		7	3.573708	0.5645	1.2690	0.260343		0.0205	0.0100947901
ss86334402		7	3.599612	0.4448	0.4496	0.502761		0.0123	0.0060837509
ss86305159		7	3.699745	0.2034	0.9936	0.319215		0.0226	0.0073214298
ss86297914		7	3.744509	0.1698	0.6119	0.434336		0.0192	0.0053986656
ss117969002		7	3.778611	0.6383	0.3686	0.543946		0.0119	0.0054930624
ss86284933		7	3.810035	0.8474	0.4747	0.491078		0.0178	0.0045919152
ss86299271		7	3.831973	0.8961	0.7028	0.402145		0.0256	0.0047627791
ss86313086		7	3.8708	0.6218	0.9968	0.318438		0.0193	0.0090699961
ss86327346		7	3.996029	0.4857	7.8086	0.005343		0.0538	0.0268904965
ss86301265		7	4.053055	0.7142	0.7651	0.382029		0.0185	0.0075519648

ss86335846		7	4.072281	0.2951	3.5646	0.059441	0.0387	0.0161213369
ss86326345		7	4.093461	0.8725	3.4540	0.063522	0.0527	0.0117193223
ss86292440		7	4.1303	0.5766	1.1039	0.293772	0.0200	0.0097633371
ss86306715		7	4.181161	0.8309	2.4306	0.119442	0.0369	0.0103726408
ss86341081		7	4.242917	0.0738	7.4157	0.006628	0.0972	0.0132807575
ss86341439		7	4.281656	0.6726	5.5429	0.018832	0.0461	0.0203011011
ss86337862		7	4.384417	0.4183	2.6501	0.103998	0.0290	0.0141363369
ss65414568	*rs43500409*	7	4.497388	0.4885	0.7526	0.385952	0.0163	0.0081560113
ss86282883		7	4.519777	0.1067	0.0274	0.868585	0.0050	0.0009561343
ss86326576		7	4.586601	0.2436	0.0382	0.845125	0.0042	0.0015525220
ss86317316		7	4.627816	0.4900	0.0417	0.838321	0.0038	0.0018978953
ss65414517	*rs43500358*	7	4.650135	0.1440	0.0009	0.975831	0.0008	0.0001963214
ss86329905		7	4.670578	0.8202	0.1864	0.666096	0.0104	0.0030746601
ss65414449	*rs43499490*	7	4.721795	0.7393	0.5492	0.458885	0.0154	0.0059561492
ss86317171		7	4.761108	0.1905	1.2695	0.260249	0.0257	0.0079323499
ss86273620		7	4.824508	0.2858	3.0366	0.081847	0.0346	0.0141382789
		7	4.884365	0.0731	0.5578	0.455405	0.0256	0.0034644626
ss86289626		7	4.922644	0.3718	1.4993	0.221198	0.0227	0.0106204389
ss65416145	*rs43497182*	7	4.959838	0.0781	8.7558	0.003191	0.1023	0.0147279827
ss46527024	*rs41255569*	7	5.006979	0.0688	0.0066	0.935491	0.0030	0.0003827225
ss86340761		7	5.033417	0.6160	1.2593	0.262174	0.0212	0.0100318930
ss86297254		7	5.057297	0.5222	2.8070	0.094303	0.0303	0.0151400786
ss86285245		7	5.135721	0.6540	0.1989	0.655741	0.0087	0.0039225747
ss86339370		7	5.160524	0.2471	8.6916	0.003304	0.0618	0.0230012575
ss61559130	*rs41655676*	7	5.201553	0.1834	0.0007	0.979230	0.0006	0.0001836733
ss86318516		7	5.322348	0.1662	0.4493	0.502887	0.0164	0.0045538628
ss86299525		7	5.363554	0.8431	0.1584	0.690794	0.0100	0.0026431505
ss86320276		7	5.410559	0.3338	2.2888	0.130767	0.0295	0.0131145746
ss86304945		7	5.472246	0.4319	1.6418	0.200510	0.0245	0.0120239662
ss86335595		7	5.502195	0.4470	0.0356	0.850295	0.0035	0.0017469759
ss86298272		7	5.524011	0.0960	1.3583	0.244233	0.0378	0.0065529000
ss86315348		7	5.577342	0.4470	0.5290	0.467275	0.0136	0.0067279067
ss86331856		7	5.614153	0.6032	6.1785	0.013165	0.0463	0.0221812604
ss86340655		7	5.694113	0.5093	0.6171	0.432405	0.0150	0.0074899908
ss86328906		7	5.71674	0.4305	0.7137	0.398497	0.0157	0.0076974912
ss86314596		7	5.771721	0.4033	1.4028	0.236664	0.0224	0.0107873539
ss65415171	*rs43498404*	7	5.794634	0.4054	2.1394	0.144012	0.0277	0.0133695807
ss86334350		7	5.817123	0.9348	0.0054	0.941389	0.0028	0.0003384349
ss65415206	*rs43499539*	7	5.854637	0.3811	1.2178	0.270180	0.0203	0.0095989908
ss86293811		7	5.886681	0.5652	1.8248	0.177179	0.0254	0.0124848713
ss64763783	*rs42863771*	7	5.924991	0.5659	1.8243	0.177236	0.0254	0.0124984884
ss86273872		7	5.977525	0.1497	0.2221	0.637578	0.0120	0.0030645142
ss61559577	*rs41657401*	7	6.541583	0.4362	1.3925	0.238379	0.0223	0.0109495825
ss86286321		7	6.567327	0.4334	2.0572	0.151940	0.0262	0.0128866817
ss86318242		7	6.599675	0.6139	0.7193	0.396677	0.0157	0.0074199156
ss86297993		7	6.628953	0.3102	0.0029	0.956775	0.0011	0.0004679760
ss86332097		7	6.665465	0.9148	0.3633	0.546881	0.0203	0.0031605253

ss86304039		7	6.694538	0.2128	0.4082	0.523092		0.0140	0.0046799243
ss86335561		7	6.727847	0.5738	0.2957	0.586744		0.0101	0.0049315601
ss86311478		7	6.771763	0.5215	0.4751	0.490861		0.0125	0.0062250733
ss61567205		7	6.79969	0.5602	0.0177	0.894181		0.0025	0.0012075396
ss86295707		7	6.8268	0.3761	0.0129	0.909452		0.0022	0.0010139258
	rs29021041	7	6.848459	0.2923	0.1294	0.719205		0.0074	0.0030471954
ss86280914		7	6.877926	0.8102	2.5059	0.113872		0.0379	0.0116577055
ss86322386		7	6.915156	0.3295	1.0202	0.312819		0.0196	0.0086392571
ss86330322		7	6.936994	0.3252	4.0777	0.043836		0.0393	0.0172342979
ss86310273		7	6.958509	0.2371	2.9366	0.087039		0.0385	0.0139361576
ss65417365	*rs43504877*	7	6.998895	0.1920	0.6663	0.414607		0.0196	0.0060888307
ss86320848		7	7.024584	0.6920	0.3567	0.550548		0.0125	0.0053251028
ss86295594		7	7.092639	0.5817	0.1032	0.748159		0.0062	0.0030403846
ss86336262		7	7.127292	0.8567	0.3330	0.564072		0.0156	0.0038223835
ss86336056		7	7.153568	0.3517	0.2167	0.641694		0.0090	0.0040901591
ss86325514		7	7.192457	0.2299	0.4693	0.493520		0.0150	0.0053157650
	rs29012637	7	7.215132	0.4054	0.4977	0.480763		0.0132	0.0063479533
ss86313604		7	7.245141	0.8403	0.0286	0.865696		0.0042	0.0011396310
ss86289425		7	7.274494	0.8596	0.1135	0.736351		0.0087	0.0020929944
ss86331227		7	7.328633	0.8947	7.6245	0.005910		0.0836	0.0157450260
ss61564645	*rs41662212*	7	7.523313	0.1368	1.0013	0.317333		0.0253	0.0059798534
ss64795633	*rs42889433*	7	7.926601	0.4448	1.5986	0.206530		0.0240	0.0118707829
ss86284505		7	8.110307	0.5473	1.1131	0.291777		0.0207	0.0102369048
ss63732299	*rs42334102*	7	8.231682	0.4348	1.9902	0.158767		0.0262	0.0129015566
ss65009201	*rs43103070*	7	8.328074	0.4391	0.0012	0.972135		0.0007	0.0003614734
ss86290882		7	8.359303	0.3324	7.2255	0.007359		0.0527	0.0233978687
ss86338392		7	8.419584	0.3446	5.5120	0.019165		0.0452	0.0204301879
ss86336314		7	8.447311	0.4713	2.5441	0.111161		0.0293	0.0145868666
ss86335350		7	8.486384	0.4456	4.1394	0.042275		0.0377	0.0186416840
ss86307625		7	8.549082	0.3446	5.5672	0.018575		0.0457	0.0206535411
ss86309969		7	8.573944	0.1540	0.8020	0.370800		0.0233	0.0060773965
ss86291657		7	8.663724	0.7292	1.5018	0.220805		0.0262	0.0103409101
ss86304247		7	8.709787	0.1103	0.0503	0.822638		0.0067	0.0013176265
ss86338335		7	8.939223	0.6426	0.0110	0.916346		0.0020	0.0009359050
ss61559693	*rs41658112*	7	8.962193	0.6777	1.2928	0.255921		0.0220	0.0096306303
ss38328364	*rs29020675*	7	9.100473	0.0236	0.2715	0.602480		0.0317	0.0014635601
ss61492473	*rs41597420*	7	9.188594	0.6526	1.3682	0.242529		0.0225	0.0102051520
ss86290412		7	9.338125	0.3001	3.3736	0.066674		0.0369	0.0154895285
ss86309315		7	9.369544	0.6769	5.8764	0.015599		0.0475	0.0207953554
ss117969020		7	9.380975	0.6354	3.2982	0.069785		0.0348	0.0161078646
ss86293087		7	9.468766	0.1082	0.4457	0.504617		0.0202	0.0038895705
ss86333384		7	9.541881	0.4269	0.1471	0.701451		0.0073	0.0035628074
ss86286623		7	9.853377	0.3266	1.4293	0.232288		0.0233	0.0102343941
ss86336976		7	9.992684	0.1468	0.6719	0.412659		0.0215	0.0053838309
ss86324187		7	10.159954	0.0881	0.0019	0.964996		0.0014	0.0002328805
ss86329209		7	10.305959	0.1390	0.5732	0.449253		0.0201	0.0048130025
ss61492499	*rs41588251*	7	10.438422	0.7156	0.7582	0.384188		0.0179	0.0072744199

ss117969035		7	10.465459	0.4448	0.3235	0.569723	0.0106	0.0052124068
ss86336042		7	10.494753	0.3897	1.9606	0.161900	0.0264	0.0125622235
ss86338280		7	10.584444	0.2285	1.0716	0.300951	0.0226	0.0079738473
ss117963904		7	10.644281	0.4570	0.0595	0.807396	0.0046	0.0022643150
ss61469306	*rs41568613*	7	10.679751	0.6203	0.0173	0.895462	0.0025	0.0011797172
ss86305220		7	10.712163	0.4319	1.9308	0.165118	0.0254	0.0124561085
ss86340415		7	10.738455	0.1211	1.0082	0.315694	0.0292	0.0062071323
ss86284791		7	10.762672	0.6196	0.5372	0.463830	0.0143	0.0067447641
ss86335837		7	10.786577	0.6225	1.2941	0.255689	0.0223	0.0104632330
ss86305828		7	10.939993	0.1762	2.3158	0.128519	0.0358	0.0104061867
ss86338204		7	10.974918	0.8703	0.1420	0.706415	0.0103	0.0023274216
ss46526160	*rs41255303*	7	11.08864	0.5473	1.8928	0.169328	0.0256	0.0127005876
ss61559709	*rs41658128*	7	11.212021	0.4921	3.5162	0.061192	0.0344	0.0172129932
ss86284813		7	11.295639	0.8617	0.0402	0.841195	0.0053	0.0012722251
ss117963966		7	11.463951	0.8209	1.8416	0.175207	0.0318	0.0093607603
ss86324038		7	11.49008	0.0946	1.5066	0.220068	0.0400	0.0068538415
	rs29021864	7	11.531893	0.2256	0.0001	0.990911	0.0003	0.0000917337
ss61558781	*rs41660028*	7	11.561843	0.7120	1.8319	0.176344	0.0280	0.0115001933
ss86337150		7	11.594483	0.1669	2.9811	0.084687	0.0422	0.0117419070
ss86337819		7	11.68756	0.9355	2.1069	0.147089	0.0549	0.0066201855
ss86307168		7	11.76669	0.3030	0.1786	0.672685	0.0086	0.0036299256
ss65434348	*rs43516006*	7	11.810965	0.1454	1.2214	0.269469	0.0292	0.0072500951
ss86275275		7	12.010836	0.7514	1.3733	0.241647	0.0257	0.0095933434
ss86330315		7	12.058445	0.1039	1.6267	0.202584	0.0398	0.0074171287
ss86317224		7	12.133277	0.7557	1.8494	0.174294	0.0305	0.0112700739
ss86303490		7	12.241735	0.2170	3.1673	0.075565	0.0413	0.0140263997
ss86294798		7	12.329979	0.3266	0.2010	0.654069	0.0091	0.0039998707
ss86341239		7	12.748011	0.2930	0.9640	0.326516	0.0204	0.0084545748
ss86316604		7	12.825567	0.6734	1.1041	0.293727	0.0211	0.0092639996
ss86323490		7	12.900911	0.8926	1.9035	0.168126	0.0427	0.0081809752
ss61558430	*rs41656882*	7	12.924369	0.2342	0.0018	0.966018	0.0009	0.0003380666
ss86325739		7	12.947882	0.3095	0.1272	0.721489	0.0073	0.0031023200
ss86338173		7	13.020287	0.7006	0.7090	0.400050	0.0176	0.0073652706
ss86335507		7	13.04204	0.8030	0.4480	0.503503	0.0154	0.0048837523
ss61559732	*rs41658750*	7	13.106373	0.2579	0.4579	0.498855	0.0145	0.0055310898
ss86328214		7	13.130621	0.4950	0.2287	0.632625	0.0088	0.0044197459
ss86338177		7	13.158672	0.4928	0.1704	0.679903	0.0075	0.0037324128
ss61559731	*rs41658749*	7	13.283948	0.2987	2.8881	0.089682	0.0348	0.0145661326
ss86311069		7	13.347718	0.2600	3.9460	0.047375	0.0434	0.0167189063
ss86291830		7	13.43433	0.1017	9.5985	0.002026	0.0969	0.0177116135
ss86315805		7	13.482622	0.0953	3.2289	0.072785	0.0578	0.0099613942
ss117963944		7	13.531426	0.2199	4.9669	0.026155	0.0493	0.0169100032
ss86337902		7	13.572768	0.5695	4.9767	0.026009	0.0405	0.0198727675
ss86295109		7	13.604103	0.4842	4.3878	0.036558	0.0385	0.0192351408
ss61498508		7	13.630378	0.7794	13.0068	0.000333	0.0815	0.0280405781
ss86323078		7	13.676664	0.7063	2.3238	0.127866	0.0323	0.0134183229
ss86291902		7	13.716273	0.3381	3.8387	0.050483	0.0380	0.0169970064

ss86328832		7	13.745591	0.3911	5.7162	0.017074		0.0448	0.0213251878
ss46526644	*rs41257099*	7	13.813909	0.6024	5.7593	0.016663		0.0449	0.0215168303
ss86286457		7	13.837316	0.1984	10.5289	0.001231		0.0744	0.0236654972
ss61491877	*rs41591941*	7	13.860485	0.3453	3.0488	0.081240		0.0342	0.0154413806
ss86330229		7	13.902404	0.2679	1.3536	0.245049		0.0246	0.0096435507
ss86305961		7	13.928419	0.1848	12.9169	0.000349		0.0842	0.0253736084
ss86341461		7	13.953342	0.6390	4.2175	0.040382		0.0395	0.0182468501
ss86338683		7	14.035064	0.8976	0.0636	0.800934		0.0077	0.0014231080
ss86302114		7	14.081324	0.1383	1.6381	0.201017		0.0346	0.0082500281
ss86328781		7	14.112021	0.4513	8.5388	0.003589		0.0541	0.0267852946
ss86314261		7	14.206319	0.7378	1.4013	0.236903		0.0257	0.0099297297
ss86293830		7	14.275547	0.3080	15.9703	0.000071		0.0780	0.0332495980
ss86322445		7	14.635618	0.3403	6.6914	0.009890		0.0492	0.0220689440
ss86338153		7	14.721613	0.6533	7.4979	0.006335		0.0527	0.0238731628
ss86293915		7	14.758121	0.7106	10.3011	0.001391		0.0646	0.0265770330
ss86339495		7	14.856981	0.3123	0.0568	0.811760		0.0048	0.0020407777
ss86340972		7	14.91308	0.3746	8.1508	0.004432		0.0547	0.0256147053
ss86304548		7	14.939912	0.3338	8.0150	0.004773		0.0548	0.0243537942
ss86296599		7	14.981355	0.2880	0.7421	0.389294		0.0175	0.0071571124
ss86328356		7	15.004559	0.2443	1.3886	0.239046		0.0246	0.0090880024
ss86300928		7	15.13957	0.3123	0.8316	0.362135		0.0181	0.0077573646
ss86308816		7	15.171628	0.6583	4.0354	0.044941		0.0392	0.0176236004
	rs29024430	7	15.211137	0.7765	3.8218	0.050991		0.0435	0.0151071486
ss86341530		7	15.257993	0.6741	6.7660	0.009489		0.0519	0.0227844000
ss86283399		7	15.283519	0.7636	0.5287	0.467409		0.0161	0.0058136610
ss86327333		7	15.388001	0.4427	6.2418	0.012706		0.0460	0.0227030406
	rs29010998	7	15.429053	0.8152	2.3631	0.124689		0.0356	0.0107272328
ss46526689	*rs41257143*	7	15.512601	0.5666	2.8112	0.094060		0.0313	0.0153917118
ss86314448		7	15.585109	0.3904	1.0922	0.296351		0.0199	0.0094945917
ss117969125		7	15.613497	0.8030	0.5970	0.439985		0.0178	0.0056184043
ss86338565		7	15.65346	0.5201	4.2947	0.038599		0.0383	0.0190975530
ss117969135		7	15.692314	0.7765	3.8218	0.050991		0.0435	0.0151071486
ss61491924	*rs41591988*	7	15.713798	0.7701	1.9800	0.159832		0.0312	0.0110424320
ss86284518		7	15.945409	0.5458	0.0671	0.795647		0.0049	0.0024166892
ss86292760		7	16.032355	0.1368	1.9738	0.160487		0.0366	0.0086442110
ss61558492	*rs41657943*	7	16.055661	0.9162	2.3507	0.125681		0.0513	0.0078822813
ss86292500		7	16.083245	0.7987	0.5390	0.463091		0.0167	0.0053833337
ss117963981		7	16.290225	0.4362	0.1949	0.658977		0.0084	0.0041488655
ss61531722	*rs41629274*	7	16.391433	0.0946	0.5025	0.478628		0.0233	0.0039914363
ss86337973		7	16.671812	0.7299	0.5817	0.445902		0.0157	0.0061741546
ss86293089		7	16.744244	0.7242	0.6146	0.433322		0.0161	0.0064187288
ss86337245		7	16.819121	0.3610	1.0586	0.303882		0.0207	0.0095442935
ss86330081		7	17.016067	0.5623	0.5834	0.445258		0.0145	0.0071377204
ss61558469	*rs41656921*	7	17.055254	0.1168	0.1144	0.735241		0.0100	0.0020659063
ss86297117		7	17.101824	0.2378	2.3748	0.123761		0.0329	0.0119225949
ss117963963		7	17.167955	0.7350	6.8952	0.008833		0.0531	0.0206893239
ss65530335	*rs43611937*	7	17.329509	0.7042	8.7852	0.003141		0.0573	0.0238531967

ss86313916		7	17.389539	0.2765	2.1980	0.138641	0.0294	0.0117611574
ss86303068		7	17.429746	0.2443	3.6418	0.056756	0.0397	0.0146661423
ss86337556		7	17.512975	0.7335	1.4502	0.228904	0.0241	0.0094202700
ss86301572		7	17.547281	0.3861	5.1460	0.023605	0.0447	0.0211825021
ss86316106		7	17.620512	0.3804	5.1502	0.023549	0.0444	0.0209249590
ss86300202		7	17.665909	0.4857	1.2293	0.267931	0.0208	0.0103897599
ss86289360		7	17.692784	0.4448	0.3706	0.542861	0.0116	0.0057114378
ss86295826		7	17.718674	0.2163	0.2435	0.621854	0.0109	0.0036933944
ss86325045		7	17.790274	0.4226	2.3826	0.123149	0.0294	0.0143569492
ss86291517		7	17.818733	0.4699	0.7240	0.395142	0.0160	0.0079648599
ss86293610		7	17.853365	0.8868	1.2985	0.254884	0.0336	0.0067442983
ss61558503	*rs41657954*	7	17.957377	0.9133	0.9661	0.325988	0.0322	0.0050954604
ss65420212	*rs43505295*	7	17.983798	0.9112	0.4539	0.500721	0.0219	0.0035411630
ss86338361		7	18.152989	0.9148	1.0326	0.309911	0.0338	0.0052704031
ss86337452		7	18.201191	0.3130	6.1840	0.013125	0.0504	0.0216605063
ss86280468		7	18.276029	0.6956	0.2654	0.606611	0.0102	0.0043336704
ss86335959		7	18.315328	0.6877	0.4472	0.503897	0.0133	0.0057300972
ss65420067	*rs43503652*	7	18.372688	0.7149	0.7991	0.371658	0.0183	0.0074628876
ss65420454	*rs43508635*	7	18.399705	0.7514	0.7786	0.377870	0.0188	0.0070396865
ss86283711		7	18.425199	0.7242	1.3406	0.247325	0.0239	0.0095465869
ss86331295		7	18.521503	0.7314	2.7811	0.095834	0.0346	0.0135885126
ss86307431		7	18.671993	0.5566	1.2163	0.270469	0.0209	0.0103217697
ss86317964		7	18.693417	0.8052	0.7543	0.385425	0.0204	0.0064012056
ss61558504	*rs41657955*	7	18.719158	0.4857	3.9852	0.046291	0.0362	0.0181065960
ss86295337		7	18.833894	0.8360	3.0861	0.079403	0.0435	0.0119370948
ss86276220		7	18.861656	0.4742	1.3262	0.249885	0.0219	0.0109092865
ss86336894		7	18.889358	0.1225	0.1272	0.721412	0.0100	0.0021452207
ss86326929		7	18.921779	0.4857	2.9729	0.085115	0.0326	0.0162663655
ss117969084		7	18.953163	0.4878	2.4025	0.121594	0.0293	0.0146488062
ss86319066		7	18.975319	0.5265	3.9204	0.048096	0.0373	0.0185873924
ss86338327		7	19.016099	0.5946	3.9000	0.048682	0.0377	0.0181665592
ss86317177		7	19.051896	0.4706	5.0204	0.025366	0.0421	0.0209598621
ss86310399		7	19.077901	0.4126	6.9543	0.008548	0.0492	0.0238607835
ss117963969		7	19.105662	0.1519	2.0832	0.149375	0.0370	0.0095370455
ss86316787		7	19.13413	0.0681	0.0671	0.795735	0.0094	0.0011883914
	rs29027155	7	19.224932	0.1218	0.9994	0.317798	0.0284	0.0060774594
ss86340683		7	19.305817	0.0745	0.7900	0.374404	0.0296	0.0040783493
ss86315598		7	19.359346	0.4405	4.7470	0.029684	0.0401	0.0197468204
ss86318450		7	19.495494	0.1483	0.2473	0.619143	0.0129	0.0032646406
ss86320016		7	19.583296	0.7701	0.1627	0.686831	0.0089	0.0031653495
ss117963958		7	19.658646	0.2199	0.7840	0.376240	0.0198	0.0068056802
ss61491948	*rs41592011*	7	19.712991	0.7772	1.1781	0.278114	0.0243	0.0084097085
ss86310793		7	19.746464	0.2564	0.6076	0.435940	0.0164	0.0062471416
ss117963906		7	19.781598	0.8632	0.0000	0.995949	0.0001	0.0000325626
ss61504302	*rs41605079*	7	19.825491	0.3546	1.1615	0.281526	0.0210	0.0095990278
ss86339660		7	19.914482	0.5716	4.8527	0.027930	0.0415	0.0203151172
ss86304934		7	19.948893	0.1189	0.0054	0.941398	0.0021	0.0004312338

ss86322215		7	20.00206	0.8639	1.1983	0.274033	0.0290	0.0068132209
ss86339711		7	20.060705	0.0981	0.0009	0.976065	0.0009	0.0001599164
ss61558529	*rs41657980*	7	20.085247	0.3188	1.5797	0.209221	0.0253	0.0109680456
ss86338876		7	20.11295	0.3653	3.6631	0.056041	0.0375	0.0173700579
ss86336941		7	20.340119	0.3080	0.9319	0.334702	0.0202	0.0085913073
ss64981819	*rs43071386*	7	20.385996	0.3990	3.5357	0.060478	0.0367	0.0176096200
ss64194865	*rs42522740*	7	20.42114	0.5874	3.5154	0.061219	0.0364	0.0176377562
ss64194791	*rs42522205*	7	20.453402	0.6039	2.3609	0.124861	0.0300	0.0143590782
ss86283892		7	20.473703	0.6769	1.0109	0.315038	0.0207	0.0090427352
ss61563864	*rs41664129*	7	20.531009	0.6626	0.6143	0.433447	0.0149	0.0066757869
ss86341057		7	20.663725	0.2951	0.8239	0.364365	0.0185	0.0076772491
ss86338719		7	20.703764	0.7178	0.0069	0.933682	0.0017	0.0006894875
ss86340533		7	20.724916	0.4305	11.9104	0.000592	0.0663	0.0325031799
	rs29013765	7	20.792311	0.4893	4.2680	0.039207	0.0386	0.0192746633
ss65424465	*rs43509325*	7	20.81467	0.7199	0.0077	0.929889	0.0018	0.0007399246
	rs29023390	7	20.886837	0.1153	0.4852	0.486301	0.0204	0.0041686195
ss86298094		7	20.907891	0.1053	0.9162	0.338806	0.0293	0.0055292784
ss86307782		7	20.957627	0.3596	5.3342	0.021203	0.0444	0.0204583453
ss86338893		7	21.085807	0.5265	0.2692	0.604003	0.0097	0.0048186394
ss86324740		7	21.144866	0.4413	0.9753	0.323709	0.0185	0.0091299921
ss86326365		7	21.17868	0.0817	0.0380	0.845555	0.0064	0.0009584148
ss65424265	*rs43507728*	7	21.251167	0.5229	0.1686	0.681461	0.0075	0.0037545856
ss86275182		7	21.280096	0.4850	0.3135	0.575699	0.0103	0.0051241171
ss64833978	*rs42926840*	7	21.331087	0.1841	0.1228	0.726121	0.0082	0.0024519724
ss65422390	*rs43509952*	7	21.586851	0.5616	3.1003	0.078719	0.0317	0.0156021300
ss64795174	*rs42889979*	7	21.648902	0.0609	0.0755	0.783524	0.0104	0.0011870602
ss65422316	*rs43509078*	7	21.73394	0.1289	1.7593	0.185143	0.0350	0.0078677886
ss86333726		7	21.814993	0.6712	1.7150	0.190776	0.0251	0.0110788879
ss61491952	*rs41592015*	7	21.842127	0.8324	0.4194	0.517444	0.0159	0.0044267472
ss64701144	*rs42795131*	7	21.923678	0.6777	3.4886	0.062212	0.0345	0.0150778227
ss65046460	*rs43132502*	7	21.946039	0.2758	5.0601	0.024794	0.0435	0.0173958431
ss86336537		7	21.968749	0.2772	6.0008	0.014545	0.0472	0.0189346530
ss86312336		7	22.015191	0.3553	0.8122	0.367772	0.0162	0.0074329755
ss61558561	*rs41658012*	7	22.050398	0.7679	1.1352	0.287040	0.0229	0.0081692963
ss61491962	*rs41592025*	7	22.071747	0.2321	1.1352	0.287040	0.0229	0.0081692963
ss65423664	*rs43512226*	7	22.100974	0.2321	1.1352	0.287040	0.0229	0.0081692963
ss86285603		7	22.147152	0.3725	1.7344	0.188279	0.0248	0.0116119595
ss65423310	*rs43509173*	7	22.167815	0.4105	3.4196	0.064850	0.0348	0.0168649145
ss86302372		7	22.197069	0.7722	1.6976	0.193032	0.0285	0.0100169444
	rs29021148	7	22.24005	0.7564	1.3628	0.243446	0.0252	0.0092982209
ss61558557	*rs41658008*	7	22.339658	0.9398	0.0042	0.948052	0.0024	0.0002765353
ss117969131		7	22.364832	0.4054	1.7677	0.184107	0.0242	0.0116448387
ss61510730	*rs41606953*	7	22.400852	0.7693	0.9560	0.328545	0.0221	0.0078583324
ss65422921	*rs43504285*	7	22.422241	0.9019	1.3536	0.245057	0.0351	0.0062169748
ss65422903	*rs43504267*	7	22.447013	0.0931	0.9564	0.328436	0.0300	0.0050736219
ss61491960	*rs41592023*	7	22.467527	0.8990	1.2268	0.268418	0.0320	0.0058038808
ss86335172		7	22.541353	0.5623	0.0470	0.828433	0.0039	0.0018982651

ss86292992		7	22.575646	0.4771	0.3938	0.530532		0.0112	0.0055878081
ss86316060		7	22.596143	0.0781	0.5765	0.447960		0.0258	0.0037207719
ss61491958	*rs41592021*	7	22.631209	0.3395	0.3666	0.545087		0.0119	0.0053150554
ss86335344		7	22.653362	0.6583	0.2793	0.597361		0.0103	0.0046472881
ss61558538	*rs41657989*	7	22.84173	0.2622	0.4642	0.495872		0.0138	0.0053367882
ss65422519	*rs43510880*	7	22.932775	0.0838	0.0382	0.845155		0.0066	0.0010068794
ss61491956	*rs41592019*	7	22.998779	0.1533	3.6356	0.056967		0.0494	0.0128131793
ss61491955	*rs41592018*	7	23.021011	0.9183	3.1847	0.074768		0.0620	0.0093052341
ss61491954	*rs41592017*	7	23.084537	0.2880	0.3633	0.546856		0.0121	0.0049497613
ss86339509		7	23.12855	0.4226	9.9418	0.001685		0.0573	0.0279846285
ss86273670		7	23.370304	0.4090	9.7289	0.001889		0.0583	0.0281953210
	rs29012267	7	23.447387	0.4011	16.6093	0.000051		0.0753	0.0361831580
ss86284992		7	23.525592	0.5609	2.0088	0.156839		0.0266	0.0131113012
ss117968901		7	23.606613	0.6819	2.0268	0.154992		0.0279	0.0121110236
ss86308514		7	23.680298	0.8582	13.0314	0.000328		0.0951	0.0231491541
ss86339526		7	23.704086	0.2736	0.1078	0.742756		0.0070	0.0027709040
ss61558582	*rs41658833*	7	23.725387	0.3073	0.9339	0.334195		0.0199	0.0084750436
ss86337361		7	23.896397	0.7378	0.0275	0.868250		0.0034	0.0013178321
ss86274175		7	24.009009	0.4033	0.0019	0.964805		0.0009	0.0004106309
ss61491985	*rs41593146*	7	24.045556	0.8181	0.3874	0.533891		0.0143	0.0042648646
ss86306322		7	24.076455	0.5924	0.0117	0.913831		0.0020	0.0009590238
ss61558600		7	24.135376	0.4692	0.1070	0.743740		0.0061	0.0030581833
ss61491991	*rs41593152*	7	24.176656	0.3037	1.0199	0.312883		0.0207	0.0087514087
ss61558599	*rs41658850*	7	24.211188	0.6927	1.3177	0.251405		0.0235	0.0100007334
ss61558598	*rs41658849*	7	24.237633	0.8288	0.4613	0.497258		0.0164	0.0046561192
ss61558596	*rs41658847*	7	24.299301	0.6891	1.5214	0.217832		0.0247	0.0105989949
ss86335009		7	24.540171	0.7858	0.2460	0.620072		0.0111	0.0037319146
ss86275910		7	24.567264	0.6812	0.8445	0.358425		0.0185	0.0080243270
ss65387739	*rs43474365*	7	24.696123	0.6719	0.9440	0.331577		0.0189	0.0083495588
ss65387804	*rs43467733*	7	24.809179	0.7335	2.3603	0.124913		0.0319	0.0124754228
ss65387816	*rs43467745*	7	24.837442	0.2930	5.2305	0.022494		0.0448	0.0185794620
ss64972516	*rs43064627*	7	24.861453	0.0731	1.7614	0.184887		0.0453	0.0061385104
ss86295390		7	24.889946	0.7278	1.6185	0.203721		0.0272	0.0107676724
ss63832040	*rs42369864*	7	24.934881	0.1175	1.6090	0.205055		0.0375	0.0077785107
ss86335506		7	24.978108	0.5165	3.5762	0.059028		0.0360	0.0179623027
ss86306122		7	25.019405	0.7135	0.3607	0.548319		0.0125	0.0051109355
ss86293711		7	25.240263	0.2034	9.6771	0.001942		0.0708	0.0229531985
ss86292285		7	25.330773	0.4835	0.0348	0.852128		0.0034	0.0017149222
ss61517149	*rs41612549*	7	25.399514	0.8087	0.0245	0.875612		0.0036	0.0011011969
ss61517152	*rs41612552*	7	25.512482	0.3037	1.3015	0.254333		0.0228	0.0096538998
ss61517153	*rs41612553*	7	25.557309	0.4090	0.2135	0.644177		0.0086	0.0041376126
ss63438505	*rs42232105*	7	25.639233	0.4749	0.3703	0.543037		0.0112	0.0055816115
ss86287995		7	25.676802	0.0845	9.1868	0.002528		0.1006	0.0155684532
ss61472761	*rs41573980*	7	25.701931	0.4434	0.0159	0.899763		0.0023	0.0011388241
ss63439021	*rs43729378*	7	25.731202	0.4119	1.0764	0.299867		0.0189	0.0091734185
ss86321391		7	25.758156	0.8331	2.3249	0.127770		0.0377	0.0104813362
ss86330233		7	25.796961	0.3997	1.1182	0.290669		0.0192	0.0092148225

ss86310294		7	25.85254	0.5716	1.9816	0.159663	0.0262	0.0128455588
ss63439600	*rs43731571*	7	25.8729	0.6003	0.4574	0.499070	0.0128	0.0061246212
ss63439855	*rs43732856*	7	25.930941	0.3768	5.3365	0.021175	0.0452	0.0212149152
ss61506430	*rs41606042*	7	25.952407	0.8840	1.4002	0.237100	0.0344	0.0070479481
ss63440020	*rs43732911*	7	25.981915	0.7457	0.6100	0.435051	0.0162	0.0061542248
ss86299717		7	26.03846	0.2822	0.0421	0.837564	0.0041	0.0016721693
ss86327473		7	26.096018	0.1891	0.0007	0.978242	0.0006	0.0001925511
ss65425468	*rs43507130*	7	26.160235	0.8754	5.5978	0.018256	0.0670	0.0146122797
ss61558611	*rs41658862*	7	26.190629	0.1648	5.0791	0.024526	0.0540	0.0148491189
ss117963936		7	26.241939	0.4305	0.1797	0.671784	0.0077	0.0037607025
ss86303296		7	26.267373	0.2765	0.5319	0.466065	0.0149	0.0059724068
ss86277215		7	26.323706	0.8746	0.2011	0.653994	0.0124	0.0027192415
ss61497350	*rs41596179*	7	26.347332	0.1404	0.1908	0.662417	0.0116	0.0027971389
ss61569762	*rs41669622*	7	26.383841	0.3245	0.0085	0.926742	0.0018	0.0007992773
ss63676902	*rs42312500*	7	26.407888	0.5021	1.6306	0.202046	0.0232	0.0115754813
ss61497365	*rs41596192*	7	26.429731	0.4771	5.9701	0.014797	0.0451	0.0224943914
ss117968894		7	26.449892	0.7457	3.2162	0.073345	0.0383	0.0145143479
ss61497348	*rs41596177*	7	26.478901	0.3632	0.3663	0.545252	0.0119	0.0055035814
	rs29016353	7	26.567786	0.1812	0.6990	0.403404	0.0195	0.0057742685
ss117969087		7	26.769327	0.5967	0.2533	0.614949	0.0093	0.0044932103
ss86318532		7	26.820261	0.7443	0.0432	0.835348	0.0044	0.0016645317
ss86320732		7	26.868802	0.6067	0.1183	0.730966	0.0064	0.0030513061
ss64458356	*rs42650543*	7	27.087087	0.3804	4.0910	0.043494	0.0374	0.0176356235
ss64458246	*rs42649488*	7	27.138107	0.7987	0.0005	0.981349	0.0005	0.0001732036
ss86317134		7	27.250872	0.4105	5.5942	0.018293	0.0461	0.0222901197
ss61530696	*rs41627667*	7	27.298401	0.7013	1.4470	0.229423	0.0236	0.0098972839
ss86297778		7	27.338673	0.5158	11.2636	0.000833	0.0616	0.0307752066
ss86275282		7	27.423796	0.7027	11.1354	0.000892	0.0663	0.0276883545
ss86309152		7	27.485026	0.1268	6.6050	0.010376	0.0709	0.0156932887
	rs29019771	7	27.601127	0.6218	0.4342	0.510136	0.0132	0.0061854923
ss86328825		7	27.711753	0.8059	0.8478	0.357496	0.0215	0.0067260341
ss117969155		7	27.732278	0.1819	0.3174	0.573372	0.0133	0.0039657181
ss86320597		7	27.766972	0.5752	10.4117	0.001311	0.0590	0.0288341884
ss61527617	*rs41625073*	7	27.799057	0.6418	0.3903	0.532367	0.0117	0.0053929905
ss64017540	*rs42432882*	7	27.838557	0.6067	0.4922	0.483178	0.0129	0.0061456294
ss86277182		7	27.874122	0.1905	2.0713	0.150540	0.0332	0.0102325531
ss86337410		7	28.087886	0.5494	5.2707	0.021984	0.0423	0.0209339850
ss86281714		7	28.12145	0.1734	2.9550	0.086057	0.0418	0.0119878798
ss86339967		7	28.198778	0.3059	11.4375	0.000760	0.0691	0.0293306545
ss86292842		7	28.304384	0.7199	1.3874	0.239244	0.0241	0.0097198636
ss65426052	*rs43507815*	7	28.327691	0.7163	1.0468	0.306591	0.0203	0.0082482665
ss65426015	*rs43507778*	7	28.355569	0.1948	0.0182	0.892703	0.0031	0.0009611986
ss65425969	*rs43507732*	7	28.410027	0.7722	0.1903	0.662817	0.0096	0.0033893080
ss86337315		7	28.450991	0.3395	1.0564	0.304396	0.0197	0.0088477083
ss65156562	*rs43242434*	7	28.485211	0.3324	2.0814	0.149551	0.0279	0.0123701196
ss61533018	*rs41628990*	7	28.528438	0.7593	10.3695	0.001341	0.0692	0.0253107955
ss86341362		7	28.556469	0.3610	1.5061	0.220150	0.0230	0.0105912623

ss61467169	*rs41565600*	7	28.581753	0.4434	0.9054	0.341668	0.0177	0.0087431528
ss86329244		7	28.610221	0.6053	2.7836	0.095681	0.0303	0.0144617657
ss61479660	*rs41580620*	7	28.670524	0.5989	6.2271	0.012811	0.0470	0.0225805107
ss64799948	*rs42896275*	7	28.760642	0.1017	1.6841	0.194817	0.0401	0.0073307338
ss86338295		7	28.825102	0.6261	7.9702	0.004891	0.0533	0.0249709197
ss86317015		7	28.860678	0.1024	1.6043	0.205719	0.0391	0.0071828428
ss63358245	*rs42197612*	7	28.905697	0.8095	0.0422	0.837342	0.0049	0.0015269309
ss65424728	*rs43503391*	7	29.072906	0.9112	2.1064	0.147137	0.0476	0.0077046816
ss86339550		7	29.106474	0.7471	0.0239	0.877184	0.0034	0.0012668920
ss86303824		7	29.147308	0.4169	0.7757	0.378769	0.0170	0.0082609859
ss65424861	*rs43503824*	7	29.194444	0.7027	3.2937	0.069977	0.0374	0.0156227037
ss63358029	*rs42205405*	7	29.272219	0.9155	2.3758	0.123681	0.0510	0.0078933676
ss86318535		7	29.3043	0.7701	0.0136	0.907281	0.0026	0.0009310963
ss86339319		7	29.349922	0.4778	1.8103	0.178907	0.0247	0.0123224289
ss61492001	*rs41593162*	7	29.519165	0.1619	0.4918	0.483378	0.0174	0.0047118564
ss86335417		7	29.65793	0.5630	0.3158	0.574326	0.0102	0.0050239922
ss65427326	*rs43509535*	7	29.779548	0.1117	5.1996	0.022893	0.0683	0.0135676155
ss61471390	*rs41570059*	7	29.822744	0.5544	0.5894	0.442912	0.0140	0.0069323277
ss86307322		7	29.884157	0.4191	1.5825	0.208825	0.0237	0.0115324970
ss64541368	*rs42688520*	7	29.910307	0.7887	1.5917	0.207508	0.0276	0.0091955875
ss64436764	*rs42639063*	7	29.946434	0.2285	2.8031	0.094534	0.0357	0.0125833330
ss86295009		7	29.997167	0.5523	5.6420	0.017805	0.0436	0.0215842239
	rs29016545	7	30.070267	0.3875	0.2293	0.632228	0.0090	0.0042680912
ss86332854		7	30.184788	0.7915	4.1285	0.042547	0.0466	0.0153788995
ss86292270		7	30.208006	0.9191	3.1820	0.074887	0.0603	0.0089740171
ss61983989	*rs41714378*	7	30.248327	0.0860	11.5217	0.000727	0.1117	0.0175527028
ss86280514		7	30.31594	0.1920	2.4558	0.117549	0.0371	0.0115045620
ss86326236		7	30.410585	0.8030	0.2995	0.584353	0.0124	0.0039386150
ss65427358	*rs43509567*	7	30.470183	0.2163	2.5264	0.112410	0.0349	0.0118415637
ss65427379	*rs43509588*	7	30.492275	0.5817	2.4905	0.114987	0.0301	0.0146277656
ss61983314	*rs41712653*	7	30.629387	0.7328	1.8095	0.179006	0.0287	0.0112473073
ss64879515	*rs42969791*	7	30.776834	0.3610	0.1792	0.672165	0.0083	0.0038284343
ss64879563	*rs42970939*	7	30.806151	0.9255	0.3992	0.527688	0.0221	0.0030437508
	rs29027355	7	30.837806	0.4377	1.8532	0.173855	0.0254	0.0124857795
ss86283291		7	30.891114	0.3567	11.1353	0.000892	0.0639	0.0293486878
ss86314937		7	31.028273	0.4649	2.0565	0.152011	0.0271	0.0134847674
ss64689705	*rs42782643*	7	31.05131	0.1834	0.0674	0.795207	0.0061	0.0018231893
ss61518301	*rs41613165*	7	31.086156	0.0996	1.5655	0.211278	0.0381	0.0068260816
ss86294367		7	31.114919	0.5487	0.4163	0.518998	0.0122	0.0060180084
ss61520645	*rs41616568*	7	31.178692	0.1383	0.4598	0.497962	0.0178	0.0042501243
ss61566092	*rs41660937*	7	31.275326	0.1383	4.8539	0.027911	0.0595	0.0141678528
	rs29027266	7	31.305955	0.1404	5.3104	0.021492	0.0616	0.0148591185
ss63599067	*rs42287574*	7	31.326394	0.6748	8.3196	0.004043	0.0562	0.0246590351
ss86282007		7	31.365838	0.4534	0.7925	0.373652	0.0165	0.0081589342
ss61496354	*rs41598305*	7	31.424929	0.4549	1.2316	0.267473	0.0208	0.0103255520
ss61505582	*rs41607218*	7	31.465597	0.9212	8.1595	0.004411	0.0974	0.0141334537
ss86325223		7	31.557237	0.6784	10.2244	0.001449	0.0619	0.0270142065

ss86341627		7	31.6094	0.1067	7.6091	0.005960		0.0824	0.0157166846
ss64719114	*rs42813037*	7	31.655498	0.7421	0.3319	0.564730		0.0124	0.0047421110
ss63560785	*rs42269818*	7	31.873339	0.1497	1.8337	0.176125		0.0359	0.0091523471
ss63560872	*rs42270547*	7	32.007402	0.3438	9.1368	0.002597		0.0595	0.0268323185
ss86339759		7	32.075728	0.4706	3.4395	0.064079		0.0342	0.0170223091
ss117968891		7	32.096931	0.0903	5.6926	0.017303		0.0775	0.0127272000
ss63561370	*rs42271012*	7	32.121704	0.8854	0.9175	0.338475		0.0281	0.0057053826
ss63561502	*rs42271656*	7	32.174183	0.4749	1.2365	0.266539		0.0218	0.0108575514
ss64964097	*rs43054912*	7	32.195122	0.6569	3.1961	0.074251		0.0339	0.0152824359
ss61505074	*rs41602841*	7	32.240599	0.1017	5.1423	0.023656		0.0681	0.0124394432
ss117968960		7	32.271914	0.6110	0.6008	0.438550		0.0146	0.0069528603
ss64249818	*rs42546442*	7	32.450298	0.3789	23.9473	0.000001		0.0908	0.0427597555
ss63561865	*rs42271977*	7	32.543149	0.3553	17.7176	0.000029		0.0787	0.0360396246
ss63562069	*rs42272644*	7	32.643966	0.7371	0.2933	0.588260		0.0113	0.0043749298
ss61469756	*rs41572752*	7	32.773092	0.3080	4.0083	0.045664		0.0417	0.0177606798
ss63562267	*rs42272708*	7	32.793432	0.9248	1.4761	0.224796		0.0432	0.0060123619
ss64249726	*rs42555196*	7	32.837731	0.9162	0.1369	0.711454		0.0122	0.0018742028
ss61473356	*rs41569775*	7	32.867777	0.1913	6.2658	0.012537		0.0582	0.0179900920
ss61518571	*rs41615523*	7	32.89999	0.5122	13.6073	0.000243		0.0681	0.0340228644
ss61506603	*rs41607109*	7	32.921033	0.8481	3.5518	0.059899		0.0489	0.0125964921
ss117963926		7	32.953331	0.5946	0.0023	0.962053		0.0009	0.0004295044
ss61525892	*rs41623193*	7	32.993263	0.4176	0.2041	0.651560		0.0083	0.0040308326
ss117966063		7	33.067228	0.4520	2.1102	0.146771		0.0267	0.0132305442
ss86307371		7	33.330686	0.2658	1.5804	0.209129		0.0261	0.0101679865
ss117969033		7	33.379635	0.6841	0.3285	0.566734		0.0114	0.0049455969
ss61529484	*rs41625202*	7	33.45978	0.1669	1.5930	0.207316		0.0321	0.0089269236
	rs29022006	7	33.602792	0.6332	2.7613	0.097017		0.0320	0.0148756448
ss61478041	*rs41582758*	7	33.626309	0.2013	3.7168	0.054274		0.0444	0.0142914676
ss86340254		7	33.675285	0.9040	2.7334	0.098724		0.0526	0.0091332945
ss61465518	*rs41566764*	7	33.923146	0.0881	3.4141	0.065067		0.0606	0.0097386927
ss63732749	*rs42326854*	7	33.944666	0.4936	0.9281	0.335701		0.0180	0.0089810530
ss86321059		7	33.965698	0.8847	0.1168	0.732678		0.0099	0.0020175188
ss86280070		7	33.995262	0.6139	0.3733	0.541424		0.0117	0.0055390596
ss86337403		7	34.102144	0.4291	3.7360	0.053658		0.0361	0.0176885280
ss86289786		7	34.126018	0.5100	6.3662	0.011853		0.0467	0.0233628331
ss61478044	*rs41582761*	7	34.186669	0.6590	4.7179	0.030187		0.0423	0.0190208680
ss117969011		7	34.229773	0.2350	0.1006	0.751244		0.0069	0.0024692591
ss61521169	*rs41617589*	7	34.338805	0.1576	0.3711	0.542598		0.0158	0.0041919409
ss61520501	*rs41614625*	7	34.370351	0.7185	0.2331	0.629382		0.0101	0.0040682952
ss86335789		7	34.41494	0.6669	1.5965	0.206822		0.0251	0.0111535258
ss86337041		7	34.614168	0.4212	0.4146	0.519863		0.0119	0.0058148758
ss86287445		7	34.694266	0.8181	1.4878	0.222969		0.0295	0.0087725339
ss86320120		7	34.837473	0.5186	5.8778	0.015587		0.0439	0.0219104757
ss86339349		7	34.879984	0.7765	2.5525	0.110577		0.0354	0.0122988910
ss64891681	*rs42988115*	7	34.900718	0.4097	0.0001	0.991362		0.0002	0.0000983942
ss86303930		7	34.923101	0.5258	1.6583	0.198267		0.0240	0.0119926781
ss64891728	*rs42989162*	7	34.957516	0.5723	0.7387	0.390379		0.0159	0.0077603059

ss86340455		7	34.993321	0.3861	0.4018	0.526345		0.0120	0.0056988416
ss61492003	*rs41593163*	7	35.09187	0.2830	1.5390	0.215184		0.0250	0.0101641135
ss65427726	*rs43512635*	7	35.12008	0.3746	0.9827	0.321889		0.0188	0.0088143236
ss65427421	*rs43509630*	7	35.199221	0.3166	0.9864	0.320959		0.0191	0.0082764263
ss61558652	*rs41658903*	7	35.229475	0.9327	0.4074	0.523510		0.0232	0.0029102149
ss86337884		7	35.373211	0.7407	2.2730	0.132098		0.0311	0.0119393590
ss86287096		7	35.498847	0.2507	0.0607	0.805443		0.0052	0.0019660901
ss86273714		7	35.525852	0.1103	0.0036	0.952283		0.0017	0.0003366992
ss64961684	*rs43049722*	7	35.673975	0.1476	0.0514	0.820717		0.0060	0.0015079583
ss65428035	*rs43508329*	7	35.82421	0.7135	5.8145	0.016153		0.0475	0.0194015555
	rs29018585	7	35.864296	0.6132	9.3741	0.002286		0.0560	0.0265689984
ss65428129	*rs43508823*	7	35.886767	0.3804	1.8095	0.179003		0.0251	0.0118528172
	rs29011315	7	35.916985	0.5759	2.4423	0.118556		0.0287	0.0140235830
ss117963912		7	35.965305	0.4585	0.4645	0.495762		0.0124	0.0061503813
ss65428539	*rs43512248*	7	36.047086	0.1898	1.1311	0.287914		0.0254	0.0078072006
ss117968920		7	36.314161	0.7213	0.5780	0.447369		0.0157	0.0063016223
ss61523700	*rs41622859*	7	36.422388	0.1426	3.2110	0.073578		0.0472	0.0115269480
ss86279776		7	36.518792	0.2658	0.0206	0.885825		0.0031	0.0011999887
ss65429102	*rs43510416*	7	36.548226	0.6261	0.0080	0.928580		0.0017	0.0008078882
ss86313325		7	36.577264	0.6497	4.9530	0.026366		0.0418	0.0190190607
ss86289967		7	36.645611	0.8138	0.1452	0.703274		0.0090	0.0027262256
ss86319015		7	36.673736	0.3417	4.4742	0.034766		0.0400	0.0180096875
ss86337467		7	36.695444	0.5007	6.8827	0.008894		0.0482	0.0240941883
ss61510749	*rs41606970*	7	36.731958	0.7163	0.9146	0.339238		0.0192	0.0078195056
ss61558676	*rs41658927*	7	36.752108	0.6977	2.1945	0.138958		0.0290	0.0122133397
ss61558672	*rs41658923*	7	36.784991	0.3030	2.2767	0.131785		0.0295	0.0124633256
ss86329638		7	36.808428	0.8832	3.0248	0.082443		0.0506	0.0104448216
ss64127397	*rs42489589*	7	36.831105	0.3223	2.7821	0.095770		0.0324	0.0141374868
ss86284167		7	36.851535	0.5351	1.1615	0.281527		0.0198	0.0098444780
ss86327006		7	36.876166	0.7128	1.8455	0.174753		0.0267	0.0109516855
ss64127053	*rs42487828*	7	36.935755	0.6218	2.6285	0.105417		0.0302	0.0141856908
ss61500698	*rs41599453*	7	36.966599	0.3338	0.6082	0.435735		0.0151	0.0066944400
ss86309917		7	36.996678	0.4348	4.0115	0.045577		0.0370	0.0182061220
ss61531848	*rs41630389*	7	37.029643	0.3711	1.7056	0.191989		0.0245	0.0114570838
ss86335550		7	37.102776	0.3818	6.5177	0.010893		0.0480	0.0226616501
ss86327249		7	37.162832	0.3897	8.3576	0.003960		0.0531	0.0252365559
ss86338693		7	37.195149	0.4212	3.7808	0.052248		0.0354	0.0172510166
ss86329542		7	37.235877	0.5616	3.7950	0.051806		0.0359	0.0176829137
ss86307514		7	37.274511	0.3703	4.6925	0.030633		0.0407	0.0189679243
ss86336640		7	37.318754	0.2507	1.0649	0.302463		0.0220	0.0082575978
ss86305591		7	37.339263	0.7894	4.3546	0.037273		0.0472	0.0156907617
ss117969144		7	37.440257	0.2436	1.7881	0.181598		0.0286	0.0105451486
ss61558687	*rs41659938*	7	37.473155	0.1182	1.5936	0.207236		0.0365	0.0076161166
ss61492020	*rs41593179*	7	37.534232	0.4054	2.7525	0.097550		0.0313	0.0150887388
ss86339173		7	37.558971	0.3388	6.2612	0.012570		0.0483	0.0216206545
ss86311967		7	37.598166	0.8983	2.8797	0.090150		0.0531	0.0096949400
ss86319549		7	37.619024	0.2679	4.5576	0.033121		0.0431	0.0169025366

ss86340441		7	37.701463	0.3617	6.8108	0.009255	0.0484	0.0223688734
ss61514106	*rs41610183*	7	37.741113	0.3625	6.9966	0.008351	0.0490	0.0226389868
ss86310481		7	37.808445	0.0702	0.4380	0.508291	0.0234	0.0030562000
ss117963976		7	37.884123	0.2944	4.6656	0.031114	0.0427	0.0177485003
ss86293727		7	38.0058	0.0494	0.0921	0.761588	0.0123	0.0011515468
ss46526050	*rs41255197*	7	38.089446	0.3309	8.4148	0.003839	0.0546	0.0241827014
ss86336130		7	38.119299	0.3732	0.3478	0.555545	0.0112	0.0052573494
ss86339543		7	38.155562	0.5365	2.9734	0.085089	0.0311	0.0154896774
ss86313082		7	38.289331	0.4756	2.4107	0.120963	0.0280	0.0139797270
ss86307502		7	38.466196	0.1003	0.6444	0.422411	0.0244	0.0044112082
ss61469218	*rs41567828*	7	38.504902	0.1605	0.2190	0.639969	0.0117	0.0031631126
ss86274592		7	38.529808	0.1218	1.3268	0.249775	0.0308	0.0065917795
ss61491978	*rs41593140*	7	38.55145	0.1175	1.6084	0.205147	0.0357	0.0073951929
ss86306126		7	38.573452	0.9341	0.4619	0.496941	0.0249	0.0030675935
ss86341381		7	38.601863	0.9004	0.0705	0.790743	0.0079	0.0014186800
ss117969118		7	38.623882	0.9090	0.6689	0.413698	0.0259	0.0042891236
ss86335704		7	38.660122	0.8202	7.5818	0.006050	0.0645	0.0190378082
ss86331819		7	38.681695	0.1340	4.2738	0.039074	0.0564	0.0130806715
ss64865989	*rs42959669*	7	38.709766	0.0953	0.4596	0.498042	0.0214	0.0036868734
ss64515322	*rs42676815*	7	38.784605	0.3983	2.0430	0.153361	0.0268	0.0128454584
ss86274194		7	38.815462	0.6017	2.0430	0.153361	0.0268	0.0128454584
ss61513960	*rs41609741*	7	38.848627	0.3983	2.0430	0.153361	0.0268	0.0128454584
ss64669780	*rs42765250*	7	38.939709	0.1311	2.5827	0.108490	0.0422	0.0096241653
ss65016215	*rs43107706*	7	38.964468	0.6755	6.9320	0.008654	0.0514	0.0225301946
ss61518866	*rs41618316*	7	39.033557	0.2278	0.9595	0.327662	0.0221	0.0077578685
ss117963979		7	39.069059	0.6311	2.2557	0.133581	0.0287	0.0133573691
ss62228723	*rs41801185*	7	39.126294	0.4656	2.9555	0.086030	0.0310	0.0154252406
ss65087759	*rs43173276*	7	39.169443	0.0989	0.1718	0.678671	0.0130	0.0023152819
ss86298117		7	39.283934	0.4857	1.2660	0.260899	0.0205	0.0102277217
ss65425200	*rs43505962*	7	39.355387	0.4842	1.2897	0.256490	0.0206	0.0102929490
ss86335525		7	39.401546	0.4054	3.4043	0.065450	0.0349	0.0168248610
ss86325122		7	39.465112	0.5086	10.3788	0.001334	0.0573	0.0286489392
ss65430271	*rs43512867*	7	39.485509	0.1999	12.7124	0.000388	0.0811	0.0259455347
ss86292325		7	39.507518	0.4921	10.3074	0.001386	0.0570	0.0285154490
ss65430359	*rs43513249*	7	39.531064	0.8266	11.2042	0.000860	0.0825	0.0236566854
ss86324422		7	39.651671	0.4957	5.8656	0.015695	0.0433	0.0216679197
ss86317533		7	39.673758	0.5129	6.3327	0.012077	0.0447	0.0223557346
ss86300217		7	39.697742	0.4835	6.6760	0.009974	0.0458	0.0228550938
ss86327902		7	39.761343	0.4735	4.1546	0.041899	0.0375	0.0186931383
ss61492027	*rs41593186*	7	39.844794	0.1956	0.4593	0.498180	0.0161	0.0050642044
ss86299556		7	39.932581	0.2758	0.2365	0.626918	0.0102	0.0040574901
ss86333891		7	40.03004	0.3338	2.3549	0.125346	0.0295	0.0131184110
ss86316981		7	40.117112	0.3266	2.6378	0.104798	0.0314	0.0138306834
ss65492011	*rs43571986*	7	40.195727	0.5344	1.3844	0.239763	0.0222	0.0110581679
ss86291256		7	40.248557	0.2815	0.0106	0.917963	0.0022	0.0008819288
ss64593716	*rs42713772*	7	40.279435	0.7521	0.2368	0.626705	0.0109	0.0040551639
ss117963939		7	40.308075	0.2550	0.0993	0.752786	0.0070	0.0026412916

ss64973214	*rs43066122*	7	40.384178	0.0852	0.1453	0.703145	0.0122	0.0019058179
ss86333341		7	40.404422	0.4792	0.6799	0.409888	0.0147	0.0073456280
ss65053239	*rs43147363*	7	40.455809	0.9198	0.1151	0.734488	0.0117	0.0017223094
ss64888476	*rs42982780*	7	40.49039	0.7307	0.8762	0.349580	0.0198	0.0078104887
ss65431024	*rs43511212*	7	40.566539	0.1490	6.3435	0.012004	0.0634	0.0160763350
ss65431164	*rs43513552*	7	40.641857	0.3066	2.1169	0.146127	0.0304	0.0129427402
ss65431334	*rs43514522*	7	40.664184	0.3059	2.2454	0.134469	0.0313	0.0133055308
ss63812847	*rs42364419*	7	40.797251	0.8532	0.0005	0.982982	0.0006	0.0001390786
ss63813145	*rs42365868*	7	40.830916	0.1504	0.0001	0.993769	0.0002	0.0000516519
ss63813315	*rs42366653*	7	40.858771	0.1483	0.0063	0.936632	0.0021	0.0005213593
ss86323666		7	41.04162	0.3016	0.0433	0.835151	0.0040	0.0016922076
ss63814297	*rs42362844*	7	41.152538	0.6533	0.2475	0.619001	0.0093	0.0042143286
ss86334383		7	41.176729	0.1791	2.8123	0.093991	0.0408	0.0119815425
ss63814417	*rs42362904*	7	41.204642	0.6569	0.0625	0.802682	0.0047	0.0021322728
ss86306257		7	41.244917	0.5437	0.9998	0.317708	0.0179	0.0088615599
ss86298597		7	41.277834	0.4499	1.0684	0.301668	0.0185	0.0091700935
ss86320413		7	41.312905	0.5860	0.7430	0.389000	0.0155	0.0075313496
ss65106653	*rs43196177*	7	41.353074	0.4083	0.7869	0.375342	0.0160	0.0077266067
ss86297622		7	41.40163	0.1798	2.5779	0.108820	0.0390	0.0115010604
ss64522280	*rs42683328*	7	41.428236	0.7063	0.2543	0.614240	0.0106	0.0043875312
ss64522232	*rs42683304*	7	41.466717	0.1619	4.7338	0.029912	0.0544	0.0147491749
ss64979909	*rs43068203*	7	41.51673	0.8768	2.6230	0.105778	0.0454	0.0098153882
ss65093488	*rs43181177*	7	41.573546	0.2980	0.0827	0.773774	0.0060	0.0025087707
ss65431616	*rs43518404*	7	41.602455	0.6999	0.0632	0.801532	0.0052	0.0022054307
ss86322499		7	41.627522	0.3080	0.0658	0.797621	0.0053	0.0022678353
ss65007340	*rs43095974*	7	41.81655	0.7557	9.7756	0.001842	0.0651	0.0240461219
ss65431837	*rs43521023*	7	41.839918	0.4592	0.1148	0.734858	0.0060	0.0030039731
ss86305859		7	41.91393	0.1211	1.3477	0.246084	0.0315	0.0067139649
ss86338752		7	41.937788	0.1433	0.2099	0.646972	0.0117	0.0028813747
ss86325140		7	41.971469	0.1984	1.8484	0.174413	0.0325	0.0103510189
ss86281478		7	41.995202	0.7915	2.2073	0.137809	0.0350	0.0115584349
ss65432082	*rs43512367*	7	42.027753	0.8890	0.6511	0.420009	0.0235	0.0046298901
ss86334442		7	42.141847	0.2923	0.0961	0.756598	0.0065	0.0026881605
ss86338968		7	42.171212	0.2923	0.0644	0.799766	0.0053	0.0021989661
ss61478409	*rs41575808*	7	42.209468	0.7213	9.5467	0.002083	0.0606	0.0243739627
ss86303579		7	42.229745	0.5509	3.3777	0.066510	0.0332	0.0164172257
ss117963933		7	42.28765	0.1784	2.8626	0.091111	0.0420	0.0123040995
ss86305657		7	42.315131	0.1812	3.1269	0.077449	0.0437	0.0129768750
ss86294560		7	42.342404	0.8238	2.4734	0.116243	0.0397	0.0115391827
ss86323735		7	42.409567	0.1734	0.0022	0.962255	0.0011	0.0003245544
ss86284729		7	42.565968	0.1325	0.3790	0.538363	0.0168	0.0038708453
ss61523702	*rs41622861*	7	42.604464	0.1375	3.6821	0.055407	0.0523	0.0123995598
ss86322503		7	42.739572	0.8589	0.4751	0.490876	0.0184	0.0044660009
ss86317775		7	42.765273	0.1325	0.2867	0.592486	0.0146	0.0033662234
ss86337811		7	42.787608	0.2600	1.5919	0.207478	0.0257	0.0099048915
ss86336681		7	42.81614	0.7314	2.6830	0.101874	0.0331	0.0129958110
ss86320535		7	42.843579	0.0659	0.0504	0.822402	0.0082	0.0010153647

ss86337789		7	42.949666	0.9026	5.7219	0.017019		0.0750	0.0131831556
ss86294604		7	43.041915	0.7872	5.3975	0.020452		0.0523	0.0175176515
ss86299849		7	43.10997	0.3266	10.8103	0.001060		0.0665	0.0292629432
ss86300165		7	43.137169	0.2923	0.2332	0.629344		0.0097	0.0039966396
ss86327677		7	43.16293	0.2915	0.5324	0.465861		0.0146	0.0060470968
ss65432465	*rs43515727*	7	43.183118	0.2930	0.6531	0.419265		0.0162	0.0066988241
ss86291870		7	43.204245	0.1712	7.8935	0.005101		0.0690	0.0195699602
ss86299309		7	43.256289	0.4642	0.3031	0.582123		0.0102	0.0050841506
ss86298482		7	43.362707	0.7858	3.1991	0.074113		0.0398	0.0134090350
ss86293894		7	43.384787	0.4434	4.2371	0.039921		0.0401	0.0197992085
ss86340434		7	43.439119	0.7844	4.1697	0.041531		0.0452	0.0152879303
ss86315959		7	43.487934	0.5566	4.2371	0.039921		0.0401	0.0197992085
ss86295038		7	43.514786	0.1991	0.8582	0.354560		0.0217	0.0069080454
ss64199460	*rs42520848*	7	43.5353	0.1956	5.9257	0.015172		0.0549	0.0172593443
ss86329602		7	43.894147	0.1511	2.2660	0.132695		0.0370	0.0094897947
ss61510763	*rs41606984*	7	43.975176	0.2249	3.5686	0.059297		0.0397	0.0138496613
ss86314138		7	44.027624	0.7428	4.5778	0.032736		0.0443	0.0169328351
ss86299810		7	44.051725	0.6583	0.2021	0.653185		0.0087	0.0038942527
ss61510767	*rs41606988*	7	44.285673	0.1619	0.2809	0.596271		0.0133	0.0036066092
ss86304145		7	44.389591	0.6583	2.9902	0.084213		0.0338	0.0152064955
ss86292143		7	44.456554	0.2980	0.8508	0.356639		0.0193	0.0080681427
ss61564054	*rs41658533*	7	44.483619	0.3095	0.4704	0.493018		0.0143	0.0061038965
ss86288930		7	44.508344	0.4577	1.9766	0.160196		0.0270	0.0134035657
ss64199326	*rs42525981*	7	44.535245	0.4642	0.0457	0.830708		0.0041	0.0020401061
ss86324955		7	44.652305	0.3854	0.4378	0.508387		0.0129	0.0061137007
ss117969098		7	44.68036	0.6275	0.0047	0.945340		0.0013	0.0006279089
ss86296183		7	44.702455	0.1039	1.5202	0.218005		0.0376	0.0070013339
ss86301883		7	44.731006	0.3574	1.1782	0.278093		0.0211	0.0097050231
ss86306523		7	44.752891	0.4742	0.9018	0.342636		0.0184	0.0091965797
ss86336439		7	44.783303	0.5236	0.9116	0.340034		0.0187	0.0093502381
ss65433068	*rs43514731*	7	44.804507	0.2908	3.6184	0.057556		0.0384	0.0158358770
ss86332241		7	44.851117	0.8539	2.1829	0.140008		0.0384	0.0095839252
ss86283287		7	44.873922	0.3847	0.2933	0.588295		0.0107	0.0050565150
ss86304220		7	44.912827	0.1819	4.9763	0.026015		0.0538	0.0160006526
ss86299455		7	44.949725	0.1948	4.8858	0.027402		0.0513	0.0160816978
ss61510771	*rs41606992*	7	44.983242	0.8030	4.9320	0.026685		0.0512	0.0161977925
ss61506959	*rs41601367*	7	45.216666	0.8188	6.0429	0.014205		0.0592	0.0175781389
ss64863151	*rs42961451*	7	45.321188	0.8016	4.1640	0.041668		0.0481	0.0152894912
ss65434176	*rs43515236*	7	45.4133	0.7987	3.1741	0.075251		0.0414	0.0133032408
ss86337681		7	45.443715	0.8009	3.4532	0.063551		0.0436	0.0138986863
ss65434077	*rs43514838*	7	45.472603	0.7693	4.8723	0.027617		0.0483	0.0171247722
ss61492068	*rs41593227*	7	45.497521	0.2292	4.6534	0.031334		0.0474	0.0167391642
ss86315060		7	45.559922	0.2428	0.0529	0.818109		0.0050	0.0018341189
ss65433978	*rs43513841*	7	45.590073	0.1777	0.3526	0.552815		0.0143	0.0041849708
ss61523707	*rs41622866*	7	45.618923	0.7328	1.0560	0.304495		0.0212	0.0082971758
ss86331500		7	45.673529	0.8238	1.3396	0.247507		0.0287	0.0083184084
ss86297474		7	45.693952	0.1855	0.0943	0.758854		0.0072	0.0021827614

ss86292246		7	45.735186	0.6103	0.0108	0.917095	0.0020	0.0009610571
ss86334034		7	45.779191	0.6060	0.0004	0.984819	0.0004	0.0001731749
ss86314163		7	45.822825	0.6060	0.0004	0.984819	0.0004	0.0001731749
ss86326409		7	45.885471	0.3904	0.0018	0.965906	0.0008	0.0003895928
ss61558768	*rs41660015*	7	45.95682	0.4577	0.0516	0.820420	0.0042	0.0020861065
ss86311982		7	45.980935	0.5580	0.1680	0.681993	0.0076	0.0037566790
ss86309196		7	46.046225	0.5666	0.4673	0.494478	0.0127	0.0062222945
ss65434399	*rs43516757*	7	46.075718	0.4735	1.8923	0.169388	0.0253	0.0126175479
ss86311114		7	46.139544	0.5552	1.1333	0.287434	0.0194	0.0095996164
ss86338798		7	46.172592	0.5322	0.1417	0.706705	0.0069	0.0034572129
ss86283107		7	46.317556	0.6361	0.1776	0.673556	0.0081	0.0037413329
ss86293319		7	46.459358	0.1239	0.6284	0.428229	0.0223	0.0048351616
ss86320682		7	46.489573	0.1139	1.4883	0.222888	0.0356	0.0071861766
ss64344495	*rs42599672*	7	46.516412	0.6175	1.7576	0.185356	0.0255	0.0120437863
ss86293900		7	46.555028	0.3911	0.3811	0.537206	0.0118	0.0056151119
ss65433696	*rs43520059*	7	46.607004	0.6239	0.5384	0.463347	0.0142	0.0066466962
ss86341452		7	46.850512	0.6920	3.0825	0.079579	0.0349	0.0148912888
ss61496418	*rs41598668*	7	46.942665	0.1304	1.6473	0.199752	0.0357	0.0080964007
ss86307730		7	47.075418	0.0480	0.5331	0.465559	0.0322	0.0029414472
ss86329938		7	47.325272	0.4291	5.5206	0.019073	0.0434	0.0212402320
ss86274826		7	47.394154	0.2178	1.8551	0.173633	0.0304	0.0103625289
ss86339866		7	47.525083	0.7400	0.0930	0.760435	0.0065	0.0024951973
ss86274276		7	47.657919	0.5301	12.7922	0.000372	0.0664	0.0330964817
ss86329857		7	47.688319	0.3990	7.5754	0.006071	0.0518	0.0248654903
ss86314729		7	47.769216	0.4456	1.6448	0.200094	0.0237	0.0117121562
ss86324621		7	47.794423	0.8804	2.3542	0.125399	0.0438	0.0092287857
ss86303158		7	47.836957	0.0974	1.8324	0.176290	0.0432	0.0076022888
ss86287876		7	47.858364	0.1712	1.1314	0.287840	0.0262	0.0074409931
ss86286998		7	47.907164	0.6977	3.9447	0.047411	0.0390	0.0164633903
ss86289116		7	47.982186	0.3868	0.3575	0.550066	0.0113	0.0053373608
ss86339687		7	48.015297	0.7128	0.0238	0.877434	0.0032	0.0013033968
ss86319663		7	48.07929	0.5895	0.1280	0.720613	0.0069	0.0033311970
ss61510781		7	48.119384	0.8582	4.9234	0.026817	0.0599	0.0145749671
ss86301956		7	48.140019	0.3166	0.8118	0.367893	0.0188	0.0081559910
ss86318110		7	48.172335	0.6146	0.0277	0.867829	0.0033	0.0015678287
ss86297353		7	48.208882	0.3417	0.2669	0.605607	0.0099	0.0044699966
ss86340654		7	48.326706	0.0444	0.3665	0.545126	0.0276	0.0023447450
ss61469240	*rs41568550*	7	48.389062	0.0530	0.1960	0.658142	0.0187	0.0018758848
ss61504972	*rs41601946*	7	48.475188	0.2070	1.1509	0.283730	0.0239	0.0078339941
ss86304509		7	49.278164	0.7830	0.7794	0.377623	0.0202	0.0068671152
ss86294732		7	49.975005	0.9176	4.2961	0.038567	0.0717	0.0108465017
ss61558741	*rs41659988*	7	50.10689	0.8481	2.8536	0.091616	0.0441	0.0113582607
ss86279424		7	50.175096	0.0587	0.1848	0.667394	0.0171	0.0018942323
ss86326491		7	50.234538	0.0745	5.8683	0.015670	0.0871	0.0120166190
ss86340219		7	50.258304	0.2486	1.1297	0.288218	0.0229	0.0085493041
ss86336356		7	50.287502	0.4799	0.5108	0.475023	0.0135	0.0067364664
ss86339786		7	50.312228	0.5193	0.5247	0.469090	0.0137	0.0068168843

ss86324099		7	50.349477	0.5093	0.1569	0.692195	0.0075	0.0037512178
ss61492047	*rs41593206*	7	50.430381	0.2264	5.0745	0.024591	0.0497	0.0174227065
ss61558738	*rs41659985*	7	50.502484	0.7779	4.4042	0.036211	0.0468	0.0161780279
ss61492040	*rs41593199*	7	50.544209	0.6719	2.8922	0.089458	0.0332	0.0146341680
ss86326957		7	50.742688	0.0774	0.1986	0.655989	0.0150	0.0021468231
ss61523706	*rs41622865*	7	50.953035	0.2077	0.1988	0.655816	0.0101	0.0033393641
ss86304235		7	50.974548	0.7915	0.2964	0.586344	0.0124	0.0040854589
ss86282217		7	51.075946	0.7507	1.3703	0.242160	0.0254	0.0094924193
ss61558745	*rs41659992*	7	51.106447	0.7521	1.4617	0.227077	0.0262	0.0097652653
ss86340061		7	51.61144	0.6332	1.3971	0.237614	0.0227	0.0105614878
ss61497145	*rs41594575*	7	51.659785	0.3603	1.7141	0.190883	0.0253	0.0116446037
ss86313732		7	51.684858	0.6332	1.3971	0.237614	0.0227	0.0105614878
ss46526752	*rs41257204*	7	51.830535	0.4835	0.3856	0.534843	0.0117	0.0058588785
ss86323613		7	51.896765	0.5172	0.3053	0.580744	0.0104	0.0052064486
	rs29009979	7	51.918028	0.2622	0.0001	0.993669	0.0002	0.0000656524
ss86301564		7	51.959228	0.8145	0.9374	0.333278	0.0234	0.0070651587
ss61558796	*rs41661243*	7	52.082243	0.4749	0.2992	0.584547	0.0100	0.0050019311
ss86314973		7	52.163786	0.4986	0.8827	0.347780	0.0177	0.0088250825
ss86328475		7	52.205427	0.4928	0.5543	0.456814	0.0140	0.0070209581
ss86339166		7	52.278288	0.5150	0.3270	0.567624	0.0107	0.0053355196
ss86303042		7	52.320917	0.3281	0.0007	0.979174	0.0005	0.0002316701
ss61558819	*rs41661266*	7	52.462171	0.4799	0.0087	0.925907	0.0017	0.0008551807
ss86340242		7	52.499242	0.4785	0.0134	0.907919	0.0021	0.0010635554
ss117963984		7	52.532837	0.3854	0.5068	0.476764	0.0135	0.0063823122
ss86294999		7	52.769873	0.1705	4.6673	0.031083	0.0540	0.0152694167
ss86328969		7	52.803709	0.0716	4.8730	0.027605	0.0799	0.0106252205
	rs29013232	7	52.832107	0.2328	0.2274	0.633627	0.0104	0.0037093201
ss86340943		7	53.028986	0.7837	1.0385	0.308529	0.0238	0.0080676751
ss86308416		7	53.054606	0.6490	0.0244	0.875909	0.0031	0.0014026771
ss61552298	*rs43709854*	7	53.080179	0.1762	1.4399	0.230557	0.0296	0.0085849996
ss86284924		7	53.109081	0.4878	0.0751	0.784109	0.0052	0.0026080210
ss86296256		7	53.187903	0.1748	1.6630	0.197626	0.0319	0.0092098329
ss86339379		7	53.258158	0.4570	1.6686	0.196869	0.0232	0.0115309046
ss86304972		7	53.394607	0.8410	1.8756	0.171276	0.0339	0.0090622749
	rs29024901	7	53.416095	0.2521	0.5376	0.463668	0.0157	0.0059226240
ss61516412	*rs41614823*	7	53.751091	0.3388	1.2196	0.269818	0.0219	0.0098047253
ss86339101		7	53.809613	0.5623	0.7291	0.393479	0.0153	0.0075454332
	rs29025316	7	53.858216	0.8209	1.0674	0.301887	0.0242	0.0071247306
ss61472423	*rs41570858*	7	53.976768	0.2507	1.8664	0.172330	0.0286	0.0107368881
ss86329226		7	54.104406	0.5279	0.4292	0.512582	0.0122	0.0060747858
ss63788840	*rs42351475*	7	54.129001	0.3496	0.0073	0.931794	0.0017	0.0007790709
ss63788858	*rs42351484*	7	54.149945	0.4069	0.6178	0.432126	0.0148	0.0071521649
ss86292212		7	54.202181	0.8460	0.1792	0.672186	0.0110	0.0028777213
ss86336643		7	54.443545	0.4011	0.2160	0.642284	0.0088	0.0042297824
ss86289831		7	54.46827	0.4011	0.2160	0.642284	0.0088	0.0042297824
ss61514414	*rs41611787*	7	54.588886	0.4212	0.0948	0.758193	0.0058	0.0028077873
ss63743720	*rs42334389*	7	54.627418	0.6010	4.3445	0.037493	0.0388	0.0186143494

ss63743684	*rs42334377*	7	54.673758	0.4463	3.7078	0.054565	0.0356	0.0176149778
ss61514411	*rs41611784*	7	54.695095	0.4513	3.7240	0.054041	0.0355	0.0175683865
ss63743078	*rs42332875*	7	54.758124	0.5874	0.5472	0.459730	0.0139	0.0067341382
ss63743012	*rs42332853*	7	54.816893	0.5315	0.5957	0.440498	0.0140	0.0069871099
ss65434784	*rs43519222*	7	54.864464	0.5967	0.6197	0.431434	0.0150	0.0072420895
ss86286086		7	54.957742	0.0494	0.4137	0.520305	0.0275	0.0025884445
	rs29019286	7	55.023686	0.3223	0.1781	0.673157	0.0086	0.0037434551
ss86305946		7	55.046625	0.5723	0.0004	0.983470	0.0004	0.0001915984
ss86337485		7	55.183605	0.6160	0.6356	0.425581	0.0151	0.0071491083
ss61563936	*rs41665100*	7	55.201362	0.3739	0.9603	0.327455	0.0190	0.0088746113
ss61563935	*rs41665099*	7	55.233884	0.0645	0.0504	0.822420	0.0084	0.0010134939
ss86329370		7	55.305991	0.5953	0.0016	0.968289	0.0008	0.0003738945
ss64659672	*rs42753623*	7	55.326638	0.4219	0.3100	0.577846	0.0103	0.0050185380
ss64977395	*rs43070876*	7	55.357107	0.4234	0.2682	0.604728	0.0096	0.0047060185
	rs29019273	7	55.468018	0.9305	1.6922	0.193746	0.0458	0.0059203928
	rs29025710	7	55.533133	0.7894	0.6212	0.430855	0.0178	0.0059210137
ss61523901	*rs41624258*	7	55.577265	0.6426	1.1829	0.277138	0.0207	0.0095170370
ss61566500	*rs41664330*	7	55.673124	0.2371	0.2033	0.652207	0.0096	0.0034868912
ss63605292	*rs42282844*	7	55.715461	0.6554	0.0002	0.989499	0.0003	0.0001162591
ss86336860		7	55.761848	0.4391	0.0354	0.850876	0.0035	0.0017105449
ss63605841	*rs42283427*	7	55.973879	0.3489	0.2395	0.624739	0.0094	0.0042852287
	rs29022947	7	56.058967	0.4857	0.0206	0.885993	0.0027	0.0013501641
ss63606009	*rs42284383*	7	56.231981	0.5623	0.1191	0.730095	0.0066	0.0032266150
ss63606117	*rs42284419*	7	56.273715	0.8567	0.6082	0.435748	0.0208	0.0051026220
ss117974919		7	56.312956	0.7529	0.2020	0.653223	0.0096	0.0035571934
ss63606351	*rs42285197*	7	56.351443	0.2744	11.7248	0.000653	0.0691	0.0275064433
ss65050502	*rs43136259*	7	56.455454	0.4148	4.2757	0.039030	0.0386	0.0187265881
ss86288195		7	56.514957	0.4778	4.1518	0.041969	0.0380	0.0189607564
ss86317067		7	56.642217	0.9097	0.3785	0.538604	0.0201	0.0032985288
ss86333264		7	56.663449	0.3059	1.8136	0.178516	0.0270	0.0114477392
ss86336456		7	56.72268	0.7822	0.1514	0.697314	0.0089	0.0030401144
ss86274250		7	56.743226	0.7256	1.1424	0.285525	0.0218	0.0086789150
ss65435889	*rs43523724*	7	56.769253	0.1390	1.7430	0.187189	0.0351	0.0083984887
	rs29015334	7	56.817698	0.1554	2.0337	0.154291	0.0364	0.0095520097
ss65435842	*rs43523679*	7	56.845817	0.9076	1.9219	0.166089	0.0441	0.0074035441
ss61558997	*rs41655244*	7	56.881151	0.1390	0.6984	0.403623	0.0222	0.0053027753
ss65434995	*rs43513736*	7	57.023065	0.1877	4.7689	0.029311	0.0511	0.0155716539
ss38332052	*rs29026156*	7	57.128567	0.1755	0.2413	0.623437	0.0123	0.0035555754
ss86287268		7	57.2357	0.9499	0.3167	0.573753	0.0239	0.0022801479
ss86285233		7	57.315877	0.2901	5.5998	0.018235	0.0495	0.0203974917
ss65435106	*rs43515046*	7	57.364752	0.2880	5.9108	0.015300	0.0510	0.0209159908
ss61504352	*rs41605127*	7	57.423737	0.7042	5.8081	0.016211	0.0506	0.0210649318
ss86279836		7	57.508232	0.0953	0.1960	0.658102	0.0142	0.0024422364
ss86340802		7	57.618367	0.6289	0.0002	0.987920	0.0003	0.0001356406
ss86315241		7	57.651091	0.2156	0.3946	0.530087	0.0147	0.0049873730
ss61558980	*rs41654629*	7	57.68504	0.2128	2.3090	0.129085	0.0344	0.0115214092
ss65435347	*rs43517685*	7	57.712078	0.6189	7.4389	0.006544	0.0512	0.0241437595

ss65435378	*rs43517716*	7	57.746997	0.6633	6.1167	0.013629	0.0481	0.0214972420
ss61563508	*rs41661880*	7	57.80292	0.2256	0.0549	0.814772	0.0051	0.0017884985
ss65436497	*rs43520433*	7	57.834628	0.3360	5.9908	0.014627	0.0478	0.0213105099
ss65436561	*rs43520497*	7	57.869612	0.0802	0.4310	0.511699	0.0223	0.0032933005
ss86297953		7	57.932582	0.3431	5.6719	0.017507	0.0463	0.0208860286
ss86297463		7	57.965096	0.3446	4.9100	0.027025	0.0428	0.0193170578
ss117968904		7	58.077198	0.5186	0.2272	0.633747	0.0087	0.0043456711
ss65436627	*rs43521663*	7	58.158097	0.5573	0.1540	0.694900	0.0075	0.0036814050
ss65436650	*rs43521686*	7	58.189351	0.2650	0.2449	0.620860	0.0106	0.0041165001
ss65437777	*rs43519410*	7	58.354585	0.2987	1.0534	0.305092	0.0209	0.0087513459
ss65437707	*rs43519340*	7	58.392733	0.5709	5.2059	0.022811	0.0422	0.0206513621
ss62430868	*rs41875144*	7	58.446221	0.9169	0.8889	0.346114	0.0323	0.0049203621
ss64755343	*rs42852759*	7	58.515018	0.3460	0.3411	0.559369	0.0111	0.0050305703
ss64789393	*rs42880998*	7	58.536271	0.6748	0.6154	0.433014	0.0151	0.0066117755
ss64789345	*rs42880950*	7	58.56111	0.8231	0.2953	0.586994	0.0128	0.0037352241
ss86282432		7	58.666786	0.1067	0.0473	0.827854	0.0066	0.0012675587
ss86337494		7	58.690613	0.6074	0.0307	0.860918	0.0033	0.0015521016
ss61541119	*rs41641457*	7	59.312778	0.5057	0.3038	0.581689	0.0102	0.0050986751
ss61523244	*rs41619517*	7	59.364887	0.2564	0.5058	0.477215	0.0148	0.0056607842
ss62516578	*rs41905068*	7	59.425068	0.5330	0.8158	0.366736	0.0169	0.0084083908
ss64718027	*rs42809559*	7	59.453637	0.2042	0.3865	0.534340	0.0139	0.0045090804
ss86340649		7	59.483061	0.7464	0.6820	0.409188	0.0180	0.0068118692
ss86293160		7	59.553448	0.1676	6.0582	0.014083	0.0628	0.0175126577
ss86340830		7	59.579803	0.9362	2.5327	0.111962	0.0591	0.0070525510
ss64596756	*rs42717372*	7	59.631492	0.8209	1.6444	0.200147	0.0306	0.0089891530
ss117963909		7	59.683707	0.8797	1.6809	0.195236	0.0368	0.0077921978
ss65438909	*rs43521628*	7	59.723565	0.2335	1.8275	0.176858	0.0299	0.0106988263
ss65438876	*rs43521597*	7	59.859835	0.3403	10.2372	0.001439	0.0623	0.0279770797
	rs29013244	7	59.967967	0.3610	4.7329	0.029926	0.0422	0.0194809942
ss86273570		7	59.991982	0.3044	1.9942	0.158347	0.0287	0.0121634259
ss86338163		7	60.024248	0.3675	1.4085	0.235714	0.0226	0.0104935778
ss86299015		7	60.12408	0.6977	5.3463	0.021058	0.0488	0.0205958947
ss86296068		7	60.167466	0.2658	0.7681	0.381123	0.0191	0.0074491139
ss86335862		7	60.238951	0.2149	2.7627	0.096938	0.0373	0.0126013708
ss86312548		7	60.26328	0.9148	0.1464	0.702111	0.0129	0.0020087681
ss86334931		7	60.299573	0.3245	3.3174	0.068978	0.0359	0.0157587934
ss117963955		7	60.320809	0.2930	2.4374	0.118928	0.0312	0.0129413921
ss61492189	*rs41595443*	7	60.350546	0.3302	3.8458	0.050270	0.0385	0.0170170482
ss65438597	*rs43519519*	7	60.394578	0.6418	5.8925	0.015458	0.0482	0.0221819976
ss65438530	*rs43519452*	7	60.417648	0.4713	0.5481	0.459334	0.0139	0.0069033049
ss86307992		7	60.439108	0.4842	0.3349	0.562950	0.0110	0.0054782898
ss64788447	*rs42880752*	7	60.468097	0.8245	0.9420	0.332097	0.0244	0.0070724754
ss86309901		7	60.498397	0.8266	0.8323	0.361913	0.0230	0.0066003336
ss61559043	*rs41655289*	7	60.521809	0.1719	0.7061	0.401043	0.0212	0.0060452993
ss86308073		7	60.68019	0.5609	5.8522	0.015813	0.0447	0.0220267895
ss86340829		7	60.703866	0.5616	5.7366	0.016878	0.0442	0.0217797699
ss65438198	*rs43516322*	7	60.727958	0.2421	6.4003	0.011630	0.0540	0.0198352006

ss65438239	*rs43517163*	7	60.751584	0.2414	3.1015	0.078658	0.0385	0.0141177046
ss65438262	*rs43517186*	7	60.772153	0.2536	4.1899	0.041040	0.0442	0.0167285947
ss61492186	*rs41595440*	7	60.796319	0.2536	4.1899	0.041040	0.0442	0.0167285947
ss65438372	*rs43517994*	7	60.826082	0.1340	0.2668	0.605628	0.0137	0.0031671968
ss86338010		7	60.855463	0.6117	4.4092	0.036105	0.0402	0.0191058875
ss86319207		7	60.94216	0.4663	0.0082	0.927763	0.0017	0.0008368914
ss61492154	*rs41594309*	7	60.966446	0.7106	0.0060	0.938441	0.0016	0.0006571173
ss86335102		7	60.987029	0.6426	5.6856	0.017371	0.0460	0.0211175687
ss65435512	*rs43520350*	7	61.015174	0.4835	2.7366	0.098527	0.0313	0.0156151371
ss86286016		7	61.310798	0.9169	0.0251	0.874114	0.0053	0.0008133921
ss86330707		7	61.402452	0.5659	1.4493	0.229045	0.0229	0.0112575901
	rs29014078	7	61.434242	0.5623	1.6542	0.198813	0.0245	0.0120431674
ss65435695	*rs43522633*	7	61.461472	0.6368	0.0991	0.752995	0.0063	0.0028982184
ss86338913		7	61.489726	0.5372	1.9140	0.166968	0.0262	0.0130504459
ss117963931		7	61.523524	0.5759	0.0924	0.761282	0.0058	0.0028296272
ss65435732	*rs43522669*	7	61.72151	0.8381	1.9357	0.164586	0.0354	0.0095989220
ss86285091		7	61.756234	0.5265	0.2075	0.648871	0.0086	0.0043054440
ss86315188		7	61.785844	0.3030	0.0888	0.765858	0.0061	0.0025563381
ss86333508		7	61.814356	0.2837	0.3283	0.566822	0.0120	0.0048758794
ss86340478		7	61.90475	0.6999	1.4054	0.236232	0.0248	0.0104157197
ss86335332		7	61.934761	0.6297	4.2871	0.038771	0.0410	0.0191318755
ss86337923		7	61.99475	0.3782	0.5903	0.442548	0.0151	0.0070972289
ss65439321	*rs43519853*	7	62.034171	0.0967	1.0529	0.305202	0.0329	0.0057524642
ss65439566	*rs43521798*	7	62.064598	0.8625	13.1146	0.000314	0.0962	0.0228218856
ss86315074		7	62.115345	0.0967	1.7307	0.188749	0.0426	0.0074350639
ss117963950		7	62.174884	0.5372	2.1531	0.142736	0.0277	0.0137657969
ss86312015		7	62.273994	0.8539	0.2378	0.625926	0.0132	0.0032891799
ss61506130	*rs41603350*	7	62.418273	0.3660	0.1400	0.708443	0.0072	0.0033630315
ss61471264	*rs41569234*	7	62.486891	0.4663	0.5944	0.441002	0.0145	0.0072037750
ss86333014		7	62.525452	0.4828	0.3169	0.573680	0.0107	0.0053275596
ss86290391		7	62.62739	0.7557	2.7565	0.097312	0.0351	0.0129767682
ss65439710	*rs43523142*	7	62.649307	0.4362	0.5674	0.451547	0.0140	0.0069101403
ss65439767	*rs43523199*	7	62.700205	0.4592	0.3331	0.564053	0.0107	0.0053220782
ss65439937	*rs43524769*	7	62.771151	0.3868	0.1400	0.708378	0.0071	0.0033518909
ss86337102		7	62.800839	0.6232	0.8665	0.352247	0.0176	0.0082645160
ss61492203	*rs41595457*	7	62.825608	0.2672	3.6909	0.055118	0.0407	0.0159376227
ss65440069	*rs43519602*	7	62.851744	0.4635	0.8453	0.358218	0.0170	0.0084751895
ss86339013		7	62.876442	0.4255	0.2622	0.608750	0.0095	0.0046519759
ss86298622		7	62.955705	0.3574	0.0598	0.806923	0.0046	0.0021290712
ss64448032	*rs42644939*	7	62.980032	0.1039	0.0185	0.891781	0.0041	0.0007679164
ss86333474		7	63.019783	0.4083	0.4620	0.496920	0.0126	0.0060686254
ss64516106	*rs42680303*	7	63.061066	0.1447	0.0931	0.760380	0.0083	0.0020494438
ss61471165	*rs41568136*	7	63.107849	0.1562	0.0844	0.771546	0.0077	0.0020373259
ss61513964	*rs41609745*	7	63.135225	0.2543	3.9192	0.048130	0.0422	0.0160119163
ss61506106	*rs41601930*	7	63.156537	0.3202	0.2871	0.592272	0.0106	0.0046073167
ss65050429	*rs43140786*	7	63.196194	0.7364	5.3414	0.021116	0.0487	0.0189088167
ss63837300	*rs42375881*	7	63.221359	0.2636	5.3414	0.021116	0.0487	0.0189088167

ss63837224	*rs42375843*	7	63.250995	0.8510	0.0015	0.969559	0.0010	0.0002560998
ss63837206	*rs42375834*	7	63.277876	0.1440	0.0388	0.844001	0.0053	0.0013021522
ss86300292		7	63.30059	0.4463	0.2439	0.621580	0.0091	0.0045028071
ss117969133		7	63.357689	0.5158	1.0425	0.307600	0.0194	0.0096835496
ss86318898		7	63.41657	0.1519	1.5322	0.216194	0.0324	0.0083439943
ss63836920	*rs42374197*	7	63.443142	0.5007	1.3841	0.239810	0.0223	0.0111271178
ss86297038		7	63.469597	0.4613	0.6210	0.430940	0.0150	0.0074434347
ss86337523		7	63.541379	0.3911	0.0490	0.824875	0.0041	0.0019728285
ss86338853		7	63.609102	0.6454	0.0001	0.991687	0.0002	0.0000912695
ss86338784		7	63.664393	0.3617	0.0003	0.985486	0.0003	0.0001598351
ss86296015		7	63.709982	0.4857	1.5496	0.213617	0.0227	0.0113205896
ss86335309		7	63.732561	0.4076	0.7344	0.391770	0.0159	0.0076839798
ss61531687	*rs41629239*	7	63.850503	0.2966	3.4487	0.063724	0.0378	0.0157906819
ss86330144		7	63.876252	0.2930	3.4981	0.061857	0.0383	0.0158755990
ss61531674	*rs41628126*	7	64.045932	0.6841	0.0487	0.825440	0.0046	0.0019729956
ss61478997	*rs41579868*	7	64.073839	0.7572	0.0054	0.941541	0.0016	0.0005949700
ss64897345	*rs42986508*	7	64.095706	0.1089	2.7191	0.099605	0.0492	0.0095458025
ss64897305	*rs42986468*	7	64.120587	0.6633	1.5451	0.214284	0.0240	0.0107142252
ss63533853	*rs42261624*	7	64.18665	0.2622	2.3931	0.122326	0.0318	0.0123010748
ss86295414		7	64.207963	0.8883	0.8957	0.344271	0.0277	0.0055012372
ss65023003	*rs43111530*	7	64.266736	0.2178	0.0063	0.936724	0.0018	0.0006000998
ss61531669	*rs41628121*	7	64.300099	0.8381	0.0291	0.864542	0.0042	0.0011525071
ss63533334	*rs42260951*	7	64.368085	0.6977	2.2912	0.130561	0.0297	0.0125462930
ss63533280	*rs42260933*	7	64.392688	0.2185	10.4268	0.001300	0.0710	0.0242603859
ss63533142	*rs42260289*	7	64.436121	0.2156	10.7255	0.001109	0.0712	0.0240795179
ss64715237	*rs42810286*	7	64.467558	0.6132	6.8260	0.009178	0.0497	0.0235705037
ss64715190	*rs42810239*	7	64.495621	0.5036	0.0169	0.896691	0.0024	0.0011919537
ss64715150	*rs42809299*	7	64.516175	0.2629	1.9528	0.162726	0.0291	0.0112631360
ss65440905	*rs43527435*	7	64.536895	0.5774	2.1039	0.147376	0.0267	0.0130387978
ss86325859		7	64.572804	0.6920	0.3759	0.539981	0.0121	0.0051784144
ss65441073	*rs43522403*	7	64.677828	0.8610	0.3612	0.548040	0.0160	0.0038208026
ss38334668	*rs29024568*	7	64.712171	0.6741	0.4639	0.496027	0.0136	0.0059714044
ss61559129	*rs41655675*	7	64.752308	0.5086	0.0020	0.964327	0.0008	0.0004176014
ss86311307		7	64.782086	0.3424	2.9842	0.084526	0.0335	0.0150737992
ss61568886	*rs41667267*	7	64.811863	0.6669	2.3586	0.125049	0.0300	0.0133172992
ss86327634		7	64.841966	0.2701	1.1116	0.292106	0.0221	0.0087297354
ss65441437	*rs43524366*	7	64.863945	0.6139	3.7214	0.054125	0.0359	0.0170052388
ss86341487		7	64.911379	0.5501	0.1946	0.659261	0.0081	0.0040319304
ss61559091	*rs41655637*	7	64.934488	0.5201	0.3065	0.580002	0.0103	0.0051495899
ss61510811		7	64.955332	0.4355	0.5619	0.453740	0.0139	0.0068334769
ss86291710		7	65.029334	0.1805	0.8577	0.354701	0.0224	0.0066196218
ss86319850		7	65.09295	0.6984	0.5065	0.476880	0.0138	0.0058061067
ss86341221		7	65.191007	0.5408	0.0653	0.798431	0.0046	0.0022994368
ss86319763		7	65.225649	0.3524	0.0488	0.825150	0.0043	0.0019629588
ss86319067		7	65.268622	0.7228	0.0735	0.786393	0.0058	0.0023147922
ss86331087		7	65.321996	0.3675	0.0578	0.810084	0.0045	0.0020889576
ss65440514	*rs43523944*	7	65.652719	0.1956	0.0328	0.856430	0.0042	0.0013224355

ss64871043	*rs42964769*	7	65.734707	0.8804	0.7021	0.402354	0.0241	0.0050754539
ss65440865	*rs43526795*	7	65.80752	0.1626	0.0846	0.771215	0.0073	0.0019948940
ss64545468	*rs42691441*	7	65.892769	0.1576	0.1058	0.745092	0.0082	0.0021892562
	rs29012520	7	65.978303	0.9011	0.6677	0.414122	0.0256	0.0045610911
ss86295807		7	66.102696	0.3582	4.8071	0.028673	0.0422	0.0193818847
ss86334232		7	66.199921	0.1468	3.4555	0.063464	0.0480	0.0120379059
ss61476536	*rs41576892*	7	66.225486	0.1383	2.5239	0.112587	0.0425	0.0101184879
ss61494564	*rs41588055*	7	66.254043	0.4219	0.1648	0.684861	0.0076	0.0037146158
ss61516865	*rs41620165*	7	66.27903	0.0931	0.1655	0.684231	0.0136	0.0022946442
ss86332347		7	66.330924	0.1447	0.1459	0.702598	0.0103	0.0025594740
ss64655681	*rs42754983*	7	66.352796	0.3338	0.3734	0.541381	0.0120	0.0053377470
ss64686192	*rs42785571*	7	66.432516	0.3546	0.2624	0.608628	0.0101	0.0046076887
ss63580298	*rs42278390*	7	66.471562	0.9097	0.5026	0.478616	0.0220	0.0036078266
ss65010848	*rs43098727*	7	66.589302	0.7980	0.0224	0.880943	0.0035	0.0011383281
ss117969067		7	66.639014	0.3883	0.3252	0.568709	0.0108	0.0051448263
ss61564834	*rs41663796*	7	66.812721	0.3539	0.0592	0.807819	0.0047	0.0021360577
ss63579008	*rs42276260*	7	66.837493	0.1984	2.5870	0.108199	0.0388	0.0123504748
ss61494956	*rs41592440*	7	66.870287	0.4570	0.6503	0.420298	0.0153	0.0075828082
ss61511420	*rs41612737*	7	66.964424	0.5645	0.7404	0.389833	0.0161	0.0079186606
ss64947134	*rs43042619*	7	67.036478	0.2027	0.8872	0.346577	0.0218	0.0070357692
	rs29023505	7	67.098616	0.1010	0.0002	0.988046	0.0005	0.0000854014
ss61522340	*rs41618518*	7	67.148623	0.3840	1.1712	0.279540	0.0209	0.0098858793
ss86309086		7	67.199009	0.4119	1.3667	0.242776	0.0221	0.0106916777
ss86321408		7	67.225926	0.7471	0.0000	0.997584	0.0001	0.0000243904
ss63737894	*rs42332347*	7	67.402484	0.5444	1.3930	0.238300	0.0219	0.0108609227
ss63737267	*rs42330247*	7	67.463706	0.5938	0.6977	0.403831	0.0160	0.0077266547
ss86300706		7	67.487998	0.5845	1.2481	0.264298	0.0214	0.0104105394
ss86304795		7	67.585164	0.4212	2.7000	0.100803	0.0311	0.0151734450
ss86304246		7	67.615864	0.6948	0.3785	0.538619	0.0126	0.0053244991
ss86290741		7	67.727426	0.5244	1.3614	0.243688	0.0214	0.0106509488
ss63845544	*rs42382366*	7	67.849538	0.2536	4.2478	0.039671	0.0411	0.0155471087
ss86336367		7	67.917191	0.0888	0.9879	0.320609	0.0323	0.0052232805
ss86305725		7	68.169924	0.5659	0.1885	0.664314	0.0081	0.0040005581
ss86313807		7	68.241263	0.7242	0.7484	0.387296	0.0179	0.0071594016
ss86283929		7	68.269553	0.1948	1.1102	0.292406	0.0251	0.0078820295
ss61465953	*rs41565217*	7	68.301351	0.2744	0.9060	0.341510	0.0197	0.0078488370
ss86335244		7	68.330972	0.1927	0.0192	0.889761	0.0033	0.0010125986
ss61473533	*rs41571949*	7	68.36405	0.8847	0.0011	0.974137	0.0010	0.0001984145
ss86291165		7	68.608336	0.4585	1.5496	0.213607	0.0235	0.0116549145
ss86320888		7	68.711144	0.1519	0.0314	0.859361	0.0047	0.0012144255
ss86323778		7	68.76034	0.3875	1.0274	0.311116	0.0193	0.0091526456
ss86313043		7	68.862104	0.1691	0.9273	0.335894	0.0229	0.0064298052
ss86341275		7	68.921809	0.5860	0.4742	0.491287	0.0131	0.0063337447
ss61492223	*rs41595477*	7	69.0871	0.4362	0.0139	0.906293	0.0022	0.0010769131
	rs29018719	7	69.133027	0.2708	2.5156	0.113182	0.0341	0.0134732340
	rs29024581	7	69.16168	0.2701	2.5977	0.107471	0.0347	0.0136660565
ss86306495		7	69.266102	0.3911	0.0220	0.882214	0.0030	0.0014178433

ss65441987	*rs43527115*	7	69.438968	0.7443	3.6488	0.056521		0.0420	0.0159899099
ss65441978	*rs43527106*	7	69.480804	0.2421	3.9580	0.047039		0.0443	0.0162551019
ss64756109	*rs42849418*	7	69.525525	0.2808	2.3674	0.124349		0.0329	0.0132711276
ss65441947	*rs43527075*	7	69.587209	0.1963	0.9869	0.320852		0.0234	0.0073746913
ss86341365		7	69.609513	0.7808	0.9976	0.318227		0.0227	0.0077684280
ss86311204		7	69.771992	0.8309	1.4257	0.232869		0.0292	0.0082088800
ss86299011		7	69.815581	0.8309	1.7232	0.189717		0.0323	0.0090654439
ss86325973		7	69.935212	0.3732	0.0031	0.955902		0.0010	0.0004840506
ss86309747		7	70.052876	0.7994	1.5232	0.217559		0.0282	0.0090575269
ss86317606		7	70.115735	0.1884	1.3826	0.240054		0.0272	0.0083305040
ss65442164	*rs43522992*	7	70.137835	0.4148	1.0444	0.307157		0.0191	0.0092957773
ss65442175	*rs43523003*	7	70.164929	0.4936	1.1475	0.284450		0.0199	0.0099266425
ss86284870		7	70.194703	0.5845	0.1081	0.742379		0.0063	0.0030454898
ss61559187	*rs41656033*	7	70.274148	0.5337	0.8918	0.345330		0.0176	0.0087493625
ss65442367	*rs43524193*	7	70.424166	0.4004	1.2139	0.270934		0.0205	0.0098437014
ss117968999		7	70.460686	0.5136	0.6230	0.430221		0.0144	0.0071763694
ss86292354		7	70.491678	0.0681	0.3684	0.544074		0.0220	0.0027865382
ss61504382	*rs41606457*	7	70.517489	0.3625	0.5151	0.473169		0.0141	0.0065265887
ss61559193	*rs41656039*	7	70.543072	0.9284	2.1396	0.143994		0.0503	0.0066912544
ss61559196	*rs41656042*	7	70.588063	0.8761	0.0716	0.789047		0.0075	0.0016325760
ss65442411	*rs43524937*	7	70.613702	0.0781	0.0010	0.974727		0.0011	0.0001553371
ss65442476	*rs43525002*	7	70.677273	0.0831	3.5066	0.061545		0.0640	0.0097564644
ss86293967		7	70.807097	0.0688	0.2982	0.585206		0.0197	0.0025213734
ss86285464		7	70.865762	0.6103	1.3800	0.240509		0.0218	0.0103755561
ss86274514		7	70.912526	0.2708	5.6796	0.017431		0.0479	0.0189265417
ss61498638	*rs43706044*	7	71.000777	0.2844	0.0060	0.938308		0.0016	0.0006468680
ss61518783	*rs41618235*	7	71.027631	0.9413	0.3123	0.576478		0.0222	0.0024594254
ss65441669	*rs43525497*	7	71.078407	0.1139	0.1847	0.667526		0.0126	0.0025505991
ss61559139	*rs41655685*	7	71.106573	0.4936	3.1435	0.076668		0.0314	0.0156945552
ss86341301		7	71.165935	0.2156	6.5820	0.010510		0.0555	0.0187890915
ss86338598		7	71.217402	0.5201	4.7132	0.030269		0.0399	0.0198958226
ss86334094		7	71.278487	0.3574	1.5730	0.210195		0.0240	0.0110466341
ss65442910	*rs43528436*	7	71.302085	0.5401	7.4371	0.006550		0.0497	0.0247130556
ss86293378		7	71.325885	0.2958	8.2928	0.004103		0.0566	0.0235724044
ss61492241	*rs41595495*	7	71.361555	0.8138	0.0847	0.771124		0.0068	0.0020544199
ss86341162		7	71.382252	0.6605	2.6519	0.103877		0.0322	0.0144627848
ss65442815	*rs43527941*	7	71.417758	0.1490	2.7089	0.100240		0.0431	0.0109235261
ss86340449		7	71.459808	0.4484	6.8994	0.008812		0.0484	0.0239249870
ss65442702	*rs43526428*	7	71.483796	0.6010	5.4161	0.020238		0.0439	0.0210532874
ss86341674		7	71.515139	0.3009	2.5635	0.109810		0.0312	0.0131172857
ss86329726		7	71.559639	0.8818	0.6313	0.427143		0.0236	0.0049213230
ss86286400		7	71.591632	0.0688	0.8536	0.355857		0.0329	0.0042196710
ss86293586		7	71.629521	0.6390	15.3002	0.000101		0.0723	0.0333718778
ss86308031		7	71.777442	0.6203	16.0356	0.000069		0.0732	0.0344792530
ss61559220	*rs41656065*	7	71.845957	0.1189	1.2897	0.256493		0.0325	0.0068176673
ss86337073		7	71.886017	0.5888	0.0002	0.988057		0.0003	0.0001339858
ss65571728	*rs43653036*	7	71.917276	0.6956	0.3522	0.553037		0.0122	0.0051751614

ss63834268	*rs42371574*	7	72.094724	0.1791	0.0387	0.844049	0.0048	0.0014076498
ss86318554		7	72.116701	0.2980	8.5801	0.003510	0.0597	0.0249883416
ss86311133		7	72.164726	0.0659	0.5682	0.451234	0.0276	0.0034011233
ss63834070	*rs42370275*	7	72.201955	0.2872	2.4866	0.115273	0.0313	0.0128073392
ss86332218		7	72.224904	0.6089	0.1215	0.727506	0.0064	0.0030611056
ss63833978	*rs42376828*	7	72.286037	0.6726	0.7262	0.394409	0.0171	0.0075356760
ss63833928	*rs42376803*	7	72.317258	0.5122	0.1158	0.733683	0.0063	0.0031633914
ss63833848	*rs42376763*	7	72.344926	0.3102	3.0448	0.081439	0.0347	0.0148319681
ss61473315	*rs41569734*	7	72.381118	0.2378	5.9599	0.014883	0.0525	0.0190410502
ss63833528	*rs42375204*	7	72.425963	0.2364	6.4773	0.011141	0.0547	0.0197651288
ss63833458	*rs42375170*	7	72.455595	0.4499	2.4422	0.118568	0.0286	0.0141682219
ss61473318	*rs41569737*	7	72.483557	0.7650	0.2997	0.584217	0.0118	0.0042448075
ss86323712		7	72.519164	0.2937	5.5113	0.019173	0.0469	0.0194447970
ss86304975		7	72.719277	0.3403	0.2626	0.608501	0.0102	0.0045803408
ss61471149	*rs41567220*	7	72.745526	0.2801	0.0136	0.907162	0.0024	0.0009723900
ss64514464	*rs42673098*	7	72.791704	0.2801	0.0136	0.907162	0.0024	0.0009723900
ss86319372		7	72.870917	0.3532	0.7312	0.392784	0.0166	0.0075621811
ss61496101	*rs41596555*	7	72.962595	0.6769	1.4699	0.225771	0.0233	0.0101847034
ss86307981		7	73.07701	0.3524	0.1659	0.683945	0.0078	0.0035461887
	rs29022304	7	73.108132	0.4678	0.6712	0.412918	0.0150	0.0074443497
ss86333861		7	73.142079	0.4112	0.2954	0.586974	0.0101	0.0048963372
ss63613149	*rs42293841*	7	73.294794	0.6683	11.6508	0.000679	0.0663	0.0293873311
	rs29016821	7	73.417139	0.3317	9.8105	0.001808	0.0611	0.0270862004
ss65443146	*rs43524272*	7	73.450611	0.1655	4.4509	0.035239	0.0526	0.0145135880
ss65443156	*rs43524282*	7	73.483184	0.8338	4.6750	0.030945	0.0536	0.0148440776
ss65443168	*rs43524294*	7	73.513983	0.1676	4.5743	0.032802	0.0529	0.0147596838
ss64749451	*rs42844354*	7	73.593533	0.2650	1.1526	0.283383	0.0227	0.0088399479
ss64749529	*rs42844632*	7	73.634585	0.8668	5.2696	0.021998	0.0625	0.0144378877
ss117969101		7	73.687204	0.2665	1.2084	0.272026	0.0232	0.0090503896
ss86291557		7	73.748931	0.6017	0.0020	0.964480	0.0008	0.0004067579
ss65451489	*rs43530556*	7	73.815531	0.2128	1.0271	0.311184	0.0225	0.0075531583
ss86290523		7	73.876321	0.1383	1.0646	0.302521	0.0279	0.0066569580
ss86303976		7	74.054703	0.6777	0.1445	0.703951	0.0077	0.0033638717
ss86328549		7	74.122181	0.2858	4.3940	0.036427	0.0420	0.0171604931
ss65451343	*rs43529710*	7	74.202119	0.1898	1.6647	0.197396	0.0306	0.0094142701
ss65451297	*rs43529664*	7	74.284605	0.1884	3.1600	0.075901	0.0415	0.0126822371
ss65451163	*rs43528937*	7	74.350477	0.2880	3.0427	0.081543	0.0342	0.0140307274
ss63844214	*rs42375807*	7	74.587278	0.8897	0.9886	0.320434	0.0302	0.0059364713
ss63843774	*rs42383787*	7	74.615596	0.1203	0.1762	0.674793	0.0122	0.0025762789
ss61498652	*rs43706057*	7	74.666389	0.7765	4.1202	0.042754	0.0447	0.0155063727
ss117968898		7	74.958174	0.7880	0.0053	0.942073	0.0016	0.0005358342
ss64304432	*rs42571188*	7	75.011554	0.2701	1.4843	0.223512	0.0251	0.0099138564
ss64304128	*rs42581136*	7	75.036433	0.7278	1.8247	0.177196	0.0278	0.0110077132
ss64303872	*rs42579108*	7	75.063373	0.3281	0.2448	0.620934	0.0099	0.0043458620
ss86337148		7	75.188968	0.3309	3.6923	0.055072	0.0382	0.0169091066
ss64303814	*rs42579079*	7	75.217745	0.7299	0.6420	0.423246	0.0166	0.0065478123
ss61495433	*rs41596806*	7	75.310271	0.3754	0.9494	0.330207	0.0185	0.0086976002

ss117968972		7	75.355176	0.8897	0.6238	0.429922		0.0233	0.0045675193
ss61511391	*rs41611908*	7	75.389301	0.0480	0.2382	0.625647		0.0212	0.0019330444
ss61494933	*rs41591520*	7	75.41424	0.0781	5.2599	0.022120		0.0764	0.0109937476
ss61520355	*rs41613282*	7	75.458245	0.3159	5.1435	0.023639		0.0448	0.0193738663
ss61520357	*rs41613284*	7	75.513102	0.2586	1.5323	0.216190		0.0264	0.0101393266
ss86285381		7	75.599005	0.6741	5.4233	0.020155		0.0460	0.0202051255
ss61875299	*rs41680333*	7	75.658763	0.6003	4.3769	0.036791		0.0397	0.0190553587
ss61499649	*rs41594638*	7	75.69332	0.3138	8.2470	0.004206		0.0570	0.0245533460
ss61524827	*rs41623856*	7	75.720878	0.6347	0.0598	0.806925		0.0047	0.0021711418
ss63936207	*rs42394307*	7	75.787074	0.6397	0.0580	0.809756		0.0047	0.0021883523
ss63936339	*rs42395173*	7	75.830003	0.2536	0.9442	0.331546		0.0206	0.0078006972
ss61475682	*rs41576653*	7	75.861642	0.7493	0.5475	0.459590		0.0157	0.0058951512
ss86327392		7	75.898166	0.6383	0.0164	0.898012		0.0025	0.0011470317
ss86318899		7	75.941924	0.5666	0.2904	0.590151		0.0096	0.0047085779
ss63936827	*rs42397917*	7	76.012194	0.2951	3.2616	0.071350		0.0352	0.0146544077
ss64942168	*rs43039178*	7	76.052507	0.6977	2.5361	0.111727		0.0316	0.0133352770
ss61516046	*rs41610972*	7	76.126824	0.4900	3.4422	0.063972		0.0348	0.0174158197
	rs29012591	7	76.167617	0.6977	1.1093	0.292606		0.0210	0.0088581265
ss86296588		7	76.233551	0.9105	0.2326	0.629761		0.0157	0.0025666829
ss86298644		7	76.39397	0.8403	0.3751	0.540436		0.0154	0.0041396687
ss117969120		7	76.415983	0.1096	0.6858	0.407867		0.0238	0.0046495926
ss61504414	*rs41606489*	7	76.437168	0.2393	0.6869	0.407489		0.0180	0.0065459875
ss86341648		7	76.603352	0.1168	0.2077	0.648702		0.0134	0.0027614522
ss63846304	*rs42377946*	7	76.64619	0.3539	0.0115	0.914760		0.0021	0.0009520956
ss117963914		7	76.674264	0.0860	0.0013	0.971310		0.0012	0.0001876207
ss61477608	*rs41578538*	7	76.690557	0.3725	1.2078	0.272140		0.0207	0.0096626798
ss86316573		7	76.794488	0.4219	0.0351	0.851343		0.0035	0.0017008872
ss86285491		7	77.443327	0.3682	0.1209	0.728128		0.0067	0.0031132366
ss64076095	*rs42460254*	7	77.479968	0.6526	1.4188	0.234008		0.0227	0.0103066026
ss64076163	*rs42460288*	7	77.503888	0.1017	1.7700	0.183815		0.0423	0.0077217684
ss64076397	*rs42460805*	7	77.533592	0.1726	0.6789	0.410261		0.0200	0.0057029690
ss64076463	*rs42461138*	7	77.58273	0.4735	2.7748	0.096209		0.0303	0.0151064535
ss61473612	*rs41572028*	7	77.619497	0.7994	0.0640	0.800283		0.0059	0.0018769631
ss86328035		7	77.647755	0.0487	1.1640	0.281017		0.0465	0.0043096507
ss86341217		7	77.669573	0.8811	0.0002	0.988146		0.0004	0.0000873709
ss63845906	*rs42384547*	7	77.694858	0.5287	2.2206	0.136636		0.0276	0.0137606813
ss65445474	*rs43528172*	7	77.733707	0.4448	1.0618	0.303156		0.0191	0.0094317016
ss65445372	*rs43526976*	7	77.79224	0.6461	0.2090	0.647676		0.0090	0.0041330630
ss61559302	*rs41656543*	7	77.855574	0.5480	0.8481	0.357417		0.0167	0.0082620036
ss64498186	*rs42667719*	7	77.940643	0.2213	2.9435	0.086667		0.0370	0.0127439004
ss64498206	*rs42667729*	7	77.97404	0.7514	1.2780	0.258652		0.0237	0.0088363453
ss86273545		7	78.00238	0.3811	1.1131	0.291777		0.0203	0.0095893246
ss64498356	*rs42668304*	7	78.033194	0.8245	3.1297	0.077316		0.0420	0.0121687566
ss65445145	*rs43525349*	7	78.103316	0.3087	0.3187	0.572550		0.0112	0.0047934323
ss86328715		7	78.140378	0.5451	3.6891	0.055177		0.0365	0.0181114251
ss117963973		7	78.292822	0.1698	0.4303	0.512051		0.0164	0.0046362944
ss117963971		7	78.324642	0.9083	1.5929	0.207333		0.0410	0.0068247987

ss63295852	*rs42184827*	7	78.349176	0.9054	0.0742	0.785457		0.0088	0.0015080046
ss61551542	*rs41651015*	7	78.405027	0.8037	11.5172	0.000729		0.0768	0.0242267361
ss61488462	*rs41585015*	7	78.442055	0.6576	1.9972	0.158039		0.0277	0.0124713425
ss65444626	*rs43528715*	7	78.499112	0.4928	0.0030	0.956031		0.0010	0.0005050536
ss63945087	*rs42400920*	7	78.55926	0.9291	0.2481	0.618576		0.0181	0.0023902698
ss63945227	*rs42395990*	7	78.653345	0.1633	4.9784	0.025983		0.0551	0.0150478889
ss63945327	*rs42396940*	7	78.68421	0.2285	0.0678	0.794684		0.0058	0.0020586755
ss61525044	*rs41625271*	7	78.748241	0.0838	0.0456	0.830893		0.0070	0.0010757248
ss63945565	*rs42397759*	7	78.789233	0.1676	6.1127	0.013659		0.0605	0.0168711049
ss61475772	*rs41577343*	7	78.925262	0.6705	0.9716	0.324613		0.0204	0.0090114660
ss86335886		7	79.005644	0.8317	0.4657	0.495220		0.0174	0.0048585755
ss86317457		7	79.026129	0.8481	2.7030	0.100611		0.0424	0.0109210854
ss61559254	*rs41656096*	7	79.054825	0.8145	0.0001	0.993506		0.0002	0.0000610217
ss65443454	*rs43526579*	7	79.095992	0.4019	0.0975	0.754993		0.0057	0.0027558314
ss86341477		7	79.165008	0.4398	0.4132	0.520544		0.0121	0.0059512631
ss65443535	*rs43527360*	7	79.187759	0.0989	0.0386	0.844209		0.0063	0.0011242215
ss65443561	*rs43527386*	7	79.258652	0.7335	0.0114	0.914979		0.0022	0.0008695613
ss61510821	*rs41607741*	7	79.51787	0.8918	0.0343	0.853062		0.0057	0.0010933852
ss65443596	*rs43527421*	7	79.747427	0.4592	0.6694	0.413530		0.0153	0.0075951244
	rs29014058	7	79.796626	0.4692	0.7000	0.403056		0.0154	0.0076905701
ss65446146	*rs43535725*	7	80.033758	0.2421	0.0477	0.827164		0.0047	0.0017258452
ss86341214		7	80.110479	0.5924	0.0000	0.995049		0.0001	0.0000558924
ss117963917		7	80.131725	0.2607	0.0025	0.959936		0.0011	0.0004154107
	rs29023471	7	80.208881	0.3768	0.0506	0.822097		0.0044	0.0020627477
ss86340181		7	80.236543	0.1662	0.1607	0.688661		0.0099	0.0027305013
ss65445695	*rs43530793*	7	80.274799	0.7894	0.4414	0.506685		0.0153	0.0050847339
ss65445797	*rs43532095*	7	80.402824	0.6748	2.9145	0.088230		0.0326	0.0143015122
ss65445852	*rs43533350*	7	80.428144	0.6913	0.9814	0.322203		0.0193	0.0082283818
ss65445863	*rs43533361*	7	80.450525	0.9241	0.3822	0.536620		0.0212	0.0029802137
ss61559313	*rs41656547*	7	80.504516	0.7321	4.3424	0.037539		0.0428	0.0168069152
ss61492276	*rs41595528*	7	80.563681	0.2500	4.2589	0.039415		0.0438	0.0164140485
ss61559353	*rs41656579*	7	80.713754	0.5695	11.7147	0.000656		0.0637	0.0312297129
ss61510824	*rs41607744*	7	80.750153	0.3295	7.6909	0.005699		0.0536	0.0237039144
ss86290811		7	80.7816	0.3295	7.6909	0.005699		0.0536	0.0237039144
ss86335634		7	80.814334	0.2077	9.2138	0.002492		0.0707	0.0232804469
ss86335628		7	80.919655	0.2020	0.0820	0.774667		0.0065	0.0021079686
	rs29021930	7	80.959068	0.4993	0.1089	0.741533		0.0060	0.0030035817
ss86294852		7	81.01287	0.1053	1.9943	0.158340		0.0433	0.0081521172
ss117969095		7	81.049742	0.4907	0.0009	0.975817		0.0006	0.0002793586
ss65446446	*rs43526926*	7	81.079165	0.4491	1.3965	0.237715		0.0220	0.0108856074
ss65446394	*rs43526874*	7	81.207547	0.1203	0.0425	0.836679		0.0060	0.0012631045
ss86327340		7	81.241988	0.1304	3.3720	0.066740		0.0486	0.0110245228
ss86296117		7	81.353858	0.3539	2.1674	0.141415		0.0288	0.0131609287
ss86317010		7	81.375357	0.3116	0.2695	0.603804		0.0106	0.0045587321
ss86330195		7	81.416243	0.4119	1.4584	0.227598		0.0232	0.0112568026
ss61504427	*rs41606502*	7	81.449648	0.3431	5.8423	0.015902		0.0483	0.0217916457
ss86295899		7	81.521952	0.4742	0.2580	0.611665		0.0096	0.0047902447

ss64363521	*rs42600078*	7	81.575749	0.7822	0.0223	0.881431	0.0034	0.0011581711
ss86299625		7	81.597509	0.1254	0.7708	0.380263	0.0246	0.0053904299
ss64941131	*rs43035908*	7	81.679041	0.0709	0.0317	0.858803	0.0066	0.0008741494
ss64427628	*rs42629054*	7	81.753211	0.2543	6.2746	0.012476	0.0561	0.0212949717
ss86274397		7	81.937271	0.1318	0.2833	0.594745	0.0143	0.0032761237
ss65444314	*rs43526440*	7	82.056402	0.3080	0.4479	0.503556	0.0134	0.0057207526
ss86300029		7	82.077065	0.3166	1.1184	0.290622	0.0216	0.0093491228
ss65444179	*rs43524505*	7	82.123473	0.9205	1.1842	0.276881	0.0369	0.0053964586
ss65443932	*rs43529757*	7	82.283978	0.3739	0.5937	0.441247	0.0155	0.0072344500
ss86332766		7	82.311287	0.5781	0.2575	0.611982	0.0100	0.0048947381
ss61559265	*rs41656107*	7	82.336699	0.8317	0.0091	0.923997	0.0023	0.0006359944
ss61549920	*rs41645715*	7	82.529919	0.3861	0.0245	0.875612	0.0030	0.0014213480
ss63113935	*rs42123132*	7	82.578292	0.4692	0.4443	0.505252	0.0123	0.0061417810
ss61489303	*rs41591428*	7	82.607019	0.1970	2.1291	0.144980	0.0341	0.0107784614
	rs29020071	7	82.669513	0.4527	0.0800	0.777326	0.0053	0.0026286014
ss86333574		7	82.816338	0.1640	3.1013	0.078669	0.0435	0.0119283288
ss64882216	*rs42975843*	7	82.845772	0.1669	0.8767	0.349430	0.0231	0.0064192072
ss63899718	*rs41968142*	7	82.951459	0.6261	2.8247	0.093274	0.0319	0.0149182889
ss63899514	*rs41967340*	7	82.987222	0.1390	5.1885	0.023039	0.0618	0.0147881182
ss86295954		7	83.026983	0.2937	0.3833	0.536019	0.0126	0.0052461140
ss117974870		7	83.21391	0.7665	1.6982	0.192958	0.0284	0.0101584526
ss64481188	*rs42658564*	7	83.258834	0.8832	3.2948	0.069928	0.0509	0.0105002988
ss86328746		7	83.316157	0.7206	10.9769	0.000970	0.0667	0.0268747739
ss64039570	*rs42445244*	7	83.397047	0.8374	5.7882	0.016394	0.0591	0.0160910435
ss86294909		7	83.597028	0.3223	3.6273	0.057251	0.0367	0.0160443816
ss64040296	*rs42449005*	7	83.625073	0.6640	0.6373	0.424955	0.0163	0.0072703994
ss86339589		7	83.649776	0.4907	0.4354	0.509563	0.0122	0.0060921301
ss86310934		7	83.669934	0.9298	2.3677	0.124326	0.0556	0.0072581540
ss86335567		7	83.693721	0.2500	0.6894	0.406644	0.0179	0.0067011606
ss64121667	*rs42489251*	7	83.749558	0.3052	0.4452	0.504860	0.0133	0.0056535809
ss117969082		7	83.855166	0.4162	7.0018	0.008327	0.0506	0.0246069156
ss86291851		7	83.895345	0.5996	0.0000	0.997214	0.0001	0.0000324220
ss64403530	*rs42623004*	7	84.085464	0.7521	2.6090	0.106714	0.0359	0.0133682157
ss64403492	*rs42622985*	7	84.114385	0.4090	2.1044	0.147331	0.0285	0.0138005939
ss64403436	*rs42622958*	7	84.158708	0.7450	4.3882	0.036550	0.0441	0.0167612701
ss61475025	*rs41574211*	7	84.209047	0.6583	0.1391	0.709305	0.0073	0.0032819327
ss86334220		7	84.240897	0.1318	2.8771	0.090293	0.0467	0.0106957530
ss86297578		7	84.27605	0.6347	6.9571	0.008535	0.0518	0.0240311649
ss86338577		7	84.317094	0.4026	0.2148	0.643153	0.0090	0.0043286959
ss64643405	*rs42745513*	7	84.400306	0.7235	0.6490	0.420751	0.0169	0.0067694728
ss86324079		7	84.420333	0.4857	2.9006	0.088991	0.0323	0.0161345492
ss61524126	*rs41625672*	7	84.440932	0.5444	7.0487	0.008114	0.0505	0.0250442373
ss61559402	*rs41656628*	7	84.57074	0.6347	0.6543	0.418873	0.0156	0.0072423920
ss61531789	*rs41630338*	7	84.613473	0.6289	0.6902	0.406369	0.0160	0.0074465458
ss86331119		7	84.683535	0.4377	1.0628	0.302935	0.0196	0.0096331059
ss64124189	*rs42481806*	7	84.709476	0.1476	5.9703	0.014796	0.0640	0.0161126598
ss65449485	*rs43530462*	7	84.749747	0.4434	5.7545	0.016708	0.0464	0.0229043472

ss61570634	*rs41670188*	7	84.820294	0.1504	7.3530	0.006860	0.0706	0.0180576504
ss64124021	*rs42490821*	7	84.854094	0.7851	0.2087	0.647946	0.0102	0.0034474364
ss64123971	*rs42490796*	7	84.889942	0.7299	1.9070	0.167737	0.0287	0.0113201421
ss61474568	*rs41571067*	7	84.928163	0.8539	4.3672	0.036999	0.0560	0.0139713546
ss64759601	*rs42856855*	7	84.978387	0.7600	4.7572	0.029510	0.0457	0.0166705538
ss61496605	*rs41591260*	7	85.048709	0.3990	0.4091	0.522633	0.0124	0.0059653516
ss63638878	*rs42296555*	7	85.12091	0.1289	0.0066	0.935106	0.0022	0.0004984219
ss86331131		7	85.142222	0.6626	0.4141	0.520127	0.0122	0.0054474682
ss61568111		7	85.177925	0.1547	1.3516	0.245403	0.0299	0.0078203953
ss63638245	*rs42304145*	7	85.217195	0.7500	0.0026	0.959286	0.0011	0.0004157274
ss86321883		7	85.242291	0.2543	0.1399	0.708486	0.0079	0.0029840211
ss63638053	*rs42302981*	7	85.279899	0.3489	2.1993	0.138530	0.0290	0.0131739951
ss86336190		7	85.311447	0.9413	0.9767	0.323349	0.0388	0.0042936027
ss63637600	*rs42300630*	7	85.393039	0.8274	0.6237	0.429933	0.0191	0.0054430593
ss86293886		7	85.445307	0.3245	1.4510	0.228773	0.0240	0.0105183430
ss86337168		7	85.482121	0.6246	0.3893	0.532864	0.0121	0.0056576780
ss64957475	*rs43046541*	7	85.520618	0.0881	0.0240	0.876875	0.0051	0.0008135685
ss86289974		7	85.559041	0.4613	0.0297	0.863169	0.0032	0.0016083302
ss65129554	*rs43216041*	7	85.58212	0.8947	2.6628	0.103172	0.0488	0.0091955101
ss86336620		7	85.607286	0.2994	0.7756	0.378799	0.0177	0.0074235147
ss86339276		7	85.694923	0.4534	0.2382	0.625651	0.0088	0.0043658689
ss64937240	*rs43026255*	7	85.751504	0.3646	0.1018	0.749761	0.0061	0.0028292703
ss64865534	*rs42957016*	7	85.825569	0.2564	0.1113	0.738718	0.0071	0.0027174900
ss86300696		7	85.904613	0.2242	6.6665	0.010027	0.0556	0.0193480696
ss64174043	*rs42512279*	7	86.060867	0.7887	0.1637	0.685861	0.0093	0.0030845741
ss61522000	*rs41622993*	7	86.376789	0.5129	0.5354	0.464576	0.0135	0.0067630750
ss86340861		7	86.444459	0.6848	0.3428	0.558434	0.0118	0.0051087935
ss64308520	*rs42571294*	7	86.471982	0.6805	0.3053	0.580759	0.0111	0.0048069856
ss86335777		7	86.515458	0.3209	0.2453	0.620545	0.0099	0.0043235950
ss64845884	*rs42942827*	7	86.554884	0.8517	0.3989	0.527848	0.0154	0.0038947818
ss86320593		7	86.670949	0.2256	0.1897	0.663281	0.0095	0.0033105756
ss61530373	*rs41624649*	7	86.692184	0.2249	0.1173	0.732099	0.0075	0.0026084533
ss65447997	*rs43535575*	7	86.744958	0.9219	0.0058	0.939262	0.0026	0.0003775539
ss61559377	*rs41656603*	7	86.79969	0.8123	0.4901	0.484099	0.0172	0.0052304558
ss86332624		7	86.846001	0.5294	0.0037	0.951808	0.0012	0.0005766869
ss65448182	*rs43527761*	7	86.905201	0.1476	11.3419	0.000800	0.0885	0.0222688238
	rs29022931	7	87.003238	0.3861	8.6874	0.003311	0.0561	0.0265958899
	rs29016960	7	87.033497	0.5458	9.6191	0.002003	0.0560	0.0277829806
ss65448196	*rs43527775*	7	87.079015	0.3231	2.3487	0.125843	0.0298	0.0130362712
ss65448394	*rs43529973*	7	87.101791	0.8539	10.2808	0.001406	0.0851	0.0212349100
	rs29021609	7	87.171544	0.6461	1.7612	0.184916	0.0254	0.0116161612
ss65448695	*rs43533773*	7	87.203186	0.5587	8.0644	0.004646	0.0520	0.0256595715
ss65448817	*rs43534895*	7	87.230875	0.6777	8.1295	0.004484	0.0561	0.0245190990
ss86283679		7	87.289169	0.6748	8.1063	0.004541	0.0562	0.0246443977
ss86287208		7	87.40121	0.1304	0.0449	0.832183	0.0057	0.0012996314
ss65448994	*rs43536772*	7	87.489089	0.7600	2.8359	0.092628	0.0375	0.0136661286
ss64788622	*rs42881627*	7	87.519638	0.2966	0.9430	0.331842	0.0206	0.0086080875

ss64995525	*rs43087567*	7	87.553381	0.7923	0.0297	0.863188	0.0041	0.0013374866
ss64158571	*rs42506632*	7	87.579322	0.7930	0.0386	0.844331	0.0047	0.0015281137
ss64158367	*rs42504930*	7	87.609579	0.8223	0.4479	0.503572	0.0167	0.0048660932
	rs29017987	7	87.6916	0.6203	2.0513	0.152529	0.0283	0.0133473326
ss61562663	*rs41663959*	7	87.723956	0.1325	8.9505	0.002872	0.0831	0.0190959336
ss64158065	*rs42504080*	7	87.750581	0.9183	0.0014	0.969980	0.0013	0.0001908774
ss117969128		7	87.785271	0.2235	4.0098	0.045624	0.0464	0.0160900776
ss61521998	*rs41622992*	7	87.864322	0.8675	8.9505	0.002872	0.0831	0.0190959336
ss86336377		7	87.919362	0.4613	2.9866	0.084402	0.0328	0.0163181857
ss65449307	*rs43528584*	7	87.975145	0.6490	14.4645	0.000155	0.0719	0.0327585251
ss61529984	*rs41630482*	7	88.031714	0.6590	4.8951	0.027257	0.0423	0.0189902882
ss65449360	*rs43529337*	7	88.089051	0.6426	8.3368	0.004005	0.0539	0.0247397328
ss86341659		7	88.123862	0.5616	4.7816	0.029098	0.0396	0.0195186115
ss61559370	*rs41656596*	7	88.265053	0.3926	4.4308	0.035654	0.0391	0.0186237440
ss86328815		7	88.377481	0.4749	0.0981	0.754274	0.0058	0.0028714817
ss86306618		7	88.425867	0.8095	4.3025	0.038423	0.0494	0.0152245473
ss86338702		7	88.679093	0.2830	3.2004	0.074055	0.0372	0.0150926857
ss63917692	*rs42178907*	7	88.733463	0.4477	10.7689	0.001084	0.0610	0.0301509693
ss86338581		7	88.802221	0.9262	0.0703	0.790967	0.0094	0.0012849227
ss61520127	*rs41611059*	7	88.84685	0.5021	8.3979	0.003875	0.0533	0.0266535955
ss61469281	*rs41568591*	7	88.936675	0.1834	4.6651	0.031123	0.0498	0.0149122200
	rs29027443	7	88.959203	0.7385	0.3155	0.574527	0.0117	0.0045135720
ss65449778	*rs43533455*	7	89.045479	0.7364	0.0018	0.966556	0.0009	0.0003306147
ss65449689	*rs43532566*	7	89.083103	0.2708	0.0078	0.929568	0.0018	0.0007071399
	rs29023123	7	89.120332	0.3087	0.4698	0.493286	0.0137	0.0058598524
ss86309796		7	89.445043	0.6562	0.1479	0.700696	0.0077	0.0034686926
ss61559412	*rs41656938*	7	89.538308	0.5458	0.0027	0.958307	0.0010	0.0004930099
ss61559411	*rs41656937*	7	89.636288	0.7027	3.0094	0.083227	0.0337	0.0140718644
ss86340305		7	89.781738	0.7378	0.3349	0.562960	0.0120	0.0046340182
	rs29026571	7	89.805334	0.5387	5.1807	0.023143	0.0423	0.0210144436
ss61510826	*rs41607746*	7	89.864925	0.4262	4.8909	0.027323	0.0421	0.0205838046
	rs29018330	7	90.030969	0.6913	1.9044	0.168025	0.0277	0.0118418076
ss61550663	*rs41653434*	7	90.107229	0.5602	0.0031	0.955736	0.0011	0.0005292925
	rs29027252	7	90.138602	0.7464	1.1256	0.289081	0.0226	0.0085735848
ss117968986		7	90.305805	0.3324	2.0297	0.154699	0.0278	0.0123545233
ss86336069		7	90.329329	0.3904	0.5438	0.461091	0.0138	0.0065606130
ss61527644	*rs41625100*	7	90.425062	0.7529	1.9292	0.165295	0.0292	0.0108583191
ss61527643	*rs41625099*	7	90.492332	0.5229	3.2041	0.073888	0.0327	0.0163312676
ss86293017		7	90.516166	0.4234	0.9436	0.331701	0.0179	0.0087326388
ss61527655	*rs41625108*	7	90.549127	0.7264	0.0054	0.941499	0.0015	0.0006002526
ss61527654	*rs41625107*	7	90.58518	0.3252	0.2165	0.641874	0.0090	0.0039660716
ss86296735		7	90.661453	0.4678	5.8246	0.016061	0.0441	0.0219513333
ss61507422	*rs41605716*	7	90.704154	0.3367	0.4209	0.516698	0.0130	0.0057856913
ss61477056	*rs41580804*	7	90.726624	0.3381	0.4289	0.512736	0.0131	0.0058582627
ss86282915		7	90.768814	0.3424	0.2811	0.596163	0.0106	0.0047870396
ss86336046		7	90.797395	0.3460	1.0733	0.300561	0.0198	0.0089491536
ss61527534	*rs41624194*	7	91.027448	0.4248	0.1153	0.734296	0.0063	0.0030761046

ss61527653	*rs41625106*	7	91.119703	0.4986	0.7141	0.398361	0.0159	0.0079595993
ss61527652	*rs41625105*	7	91.17395	0.6160	0.0259	0.872260	0.0031	0.0014641389
ss86285499		7	91.300574	0.4850	0.1999	0.654922	0.0084	0.0042195814
ss117969093		7	91.481019	0.7428	0.9596	0.327616	0.0207	0.0079063367
ss61484182	*rs41585631*	7	91.545854	0.8789	3.3777	0.066510	0.0533	0.0113358692
	rs29011503	7	91.570409	0.8890	4.2700	0.039160	0.0625	0.0123301925
ss86311371		7	91.676212	0.9269	0.0946	0.758524	0.0111	0.0014993503
ss86290254		7	91.836261	0.6440	0.5831	0.445366	0.0141	0.0064437107
ss86332631		7	91.903227	0.6884	0.0000	0.998403	0.0000	0.0000163598
ss86280972		7	92.033644	0.6519	0.3887	0.533199	0.0116	0.0052529882
ss86294154		7	92.215011	0.2779	0.0586	0.808785	0.0048	0.0019414348
	rs29009626	7	92.504781	0.3539	0.0711	0.789849	0.0051	0.0023363308
	rs29024390	7	92.69065	0.3317	0.0001	0.993706	0.0002	0.0000699054
ss61526690	*rs41625576*	7	92.868667	0.7787	0.0003	0.987328	0.0003	0.0001182192
ss61478202	*rs41574715*	7	92.888136	0.7923	1.0136	0.314383	0.0226	0.0074410256
ss61563813	*rs41664078*	7	93.097844	0.3338	4.3380	0.037635	0.0386	0.0171560688
	rs29013278	7	93.142767	0.6318	2.7963	0.094934	0.0311	0.0144803860
ss117969005		7	93.145559	0.9040	0.7610	0.383329	0.0276	0.0047850974
ss86286315		7	93.268631	0.7536	0.2385	0.625465	0.0109	0.0040479378
ss61494395	*rs41596695*	7	93.311642	0.3216	4.7531	0.029580	0.0435	0.0189947292
ss117969030		7	93.401744	0.4348	2.7300	0.098930	0.0300	0.0147352356
ss61563814	*rs41664079*	7	93.438236	0.5759	3.3573	0.067336	0.0350	0.0171076550
ss61563816	*rs41664081*	7	93.504361	0.0630	4.8975	0.027220	0.0841	0.0099360787
ss86326193		7	93.736297	0.2049	15.7302	0.000081	0.0907	0.0295392215
ss61559452	*rs41656976*	7	93.780024	0.8209	12.8999	0.000352	0.0863	0.0253815942
ss61559451	*rs41656975*	7	93.869683	0.6576	2.9569	0.085957	0.0328	0.0147580690
ss61559446	*rs43746616*	7	93.922166	0.9112	3.2189	0.073227	0.0577	0.0093404161
	rs29026678	7	93.990409	0.7765	1.3729	0.241718	0.0262	0.0090831725
ss86338661		7	94.060139	0.2543	0.4370	0.508784	0.0140	0.0053220664
ss86285630		7	94.356254	0.7822	17.3513	0.000035	0.0906	0.0308716122
ss61517350	*rs41614839*	7	94.489759	0.6777	1.3840	0.239830	0.0228	0.0099569032
ss86334134		7	94.513589	0.1798	12.6943	0.000392	0.0863	0.0254580057
ss86296398		7	94.535753	0.2779	1.0079	0.315758	0.0210	0.0084321961
ss61566368	*rs41663308*	7	94.770689	0.7328	0.0816	0.775291	0.0060	0.0023512455
ss86285580		7	94.836553	0.1354	3.9820	0.046379	0.0559	0.0130837120
ss117969070		7	94.986066	0.8646	1.1528	0.283332	0.0300	0.0070128417
	rs29025271	7	95.12464	0.9198	6.5532	0.010680	0.0867	0.0128005704
ss61504442	*rs41606517*	7	95.25307	0.5616	0.0168	0.896844	0.0024	0.0011765075
ss86311472		7	95.485131	0.2428	0.4760	0.490486	0.0146	0.0053534292
ss86339263		7	95.640021	0.2715	0.4404	0.507143	0.0141	0.0055748239
ss61492330		7	95.708058	0.0458	5.3842	0.020608	0.1007	0.0088096318
ss86285235		7	95.762286	0.1597	0.6279	0.428413	0.0200	0.0053764499
ss86312079		7	95.791895	0.3209	4.6231	0.031887	0.0425	0.0185339433
ss86301690		7	95.883104	0.4176	0.8259	0.363788	0.0168	0.0081904917
	rs29021192	7	95.977847	0.7106	0.5419	0.461891	0.0145	0.0059724111
ss86274515		7	96.019023	0.1769	0.3128	0.576140	0.0132	0.0038549217
ss61559461	*rs41656985*	7	96.098484	0.3804	1.9252	0.165723	0.0261	0.0123240177

ss61559463	*rs41656987*	7	96.206183	0.8639	2.4671	0.116704	0.0432	0.0101632361
ss86329974		7	96.301561	0.5423	5.3980	0.020447	0.0428	0.0212358259
ss61516512	*rs41615915*	7	96.386515	0.5602	3.3154	0.069063	0.0335	0.0165214283
ss61472319		7	96.468756	0.6540	0.7677	0.381233	0.0172	0.0077706972
ss86312419		7	96.734416	0.1411	8.4503	0.003766	0.0779	0.0188908613
ss61494074	*rs41593284*	7	96.855312	0.6583	0.1495	0.699160	0.0074	0.0033340641
ss61494071	*rs41593281*	7	96.89315	0.0831	0.3514	0.553489	0.0199	0.0030320878
ss86298376		7	96.985983	0.3754	0.0018	0.966191	0.0008	0.0003769168
ss117968909		7	97.011276	0.6254	0.0464	0.829433	0.0042	0.0019484616
ss86290989		7	97.169062	0.2973	0.0743	0.785304	0.0054	0.0022713746
ss61564070	*rs41658549*	7	97.190597	0.5917	0.0003	0.985674	0.0003	0.0001608564
ss61469583	*rs41571181*	7	97.218356	0.8474	2.1429	0.143679	0.0371	0.0096014346
ss61564063	*rs41658542*	7	97.249604	0.6554	0.1547	0.694193	0.0076	0.0034372161
ss86329349		7	97.371047	0.9255	0.1530	0.695804	0.0137	0.0018888820
ss86321563		7	97.49291	0.2471	2.4861	0.115309	0.0331	0.0123136293
ss117962530		7	97.524769	0.7543	2.5843	0.108385	0.0339	0.0125515949
ss117962527		7	97.561406	0.8603	2.3182	0.128324	0.0401	0.0096412872
ss86300200		7	97.632214	0.6268	1.1203	0.290225	0.0200	0.0093724753
ss86315737		7	97.677302	0.5415	1.0642	0.302614	0.0191	0.0094869772
	rs29018251	7	97.73061	0.7536	3.3380	0.068127	0.0396	0.0146969142
ss86303738		7	97.761821	0.3847	0.8967	0.343984	0.0177	0.0083924697
ss86324113		7	97.828088	0.6397	2.5473	0.110938	0.0305	0.0140720562
ss61469288	*rs41568595*	7	97.894515	0.9463	0.2097	0.647184	0.0189	0.0019265037
ss86291843		7	97.914944	0.2808	0.0297	0.863196	0.0036	0.0014404089
ss86339659		7	97.981951	0.5659	0.3617	0.547739	0.0117	0.0057408634
ss117968912		7	98.032494	0.5638	0.0000	0.996812	0.0001	0.0000362910
ss86333606		7	98.176318	0.8625	2.0863	0.149078	0.0391	0.0092803224
ss61473255	*rs41568774*	7	98.223702	0.5917	0.4976	0.480788	0.0131	0.0063534351
ss86324013		7	98.260898	0.8639	0.1730	0.677612	0.0115	0.0027115039
ss61523713	*rs41622872*	7	98.399838	0.4176	0.2942	0.587729	0.0104	0.0050475349
ss117968895		7	98.456214	0.9370	3.4110	0.065188	0.0722	0.0085268540
ss86296856		7	98.548322	0.0938	2.3991	0.121862	0.0506	0.0086033674
ss86339853		7	98.691267	0.7543	1.4672	0.226195	0.0266	0.0098626218
ss61559501	*rs41657025*	7	98.743036	0.6812	0.3970	0.528851	0.0119	0.0051810989
ss61528290	*rs41624339*	7	98.900125	0.3173	1.8630	0.172723	0.0275	0.0119167324
ss86339607		7	98.978449	0.2579	0.2677	0.605076	0.0113	0.0043221013
ss86310226		7	99.073213	0.3166	0.6469	0.421488	0.0163	0.0070398838
ss86336756		7	99.16808	0.8811	3.3839	0.066263	0.0534	0.0111827889
ss65450471	*rs43531745*	7	99.304962	0.8875	2.2117	0.137422	0.0450	0.0089772301
ss86326217		7	99.482585	0.7414	2.5548	0.110414	0.0338	0.0129782765
ss61492363	*rs41596511*	7	99.649983	0.4484	0.0300	0.862462	0.0032	0.0015913355
ss86300776		7	99.797026	0.6511	4.1588	0.041796	0.0391	0.0177538149
ss86339956		7	99.846551	0.5523	8.4385	0.003790	0.0544	0.0268926773
ss86300655		7	99.898036	0.4126	6.4603	0.011247	0.0481	0.0233130772
ss61521596	*rs41620499*	7	100.06466	0.2457	1.5769	0.209623	0.0269	0.0099864796
	rs29013403	7	100.388083	0.2937	1.3932	0.238269	0.0239	0.0099056909
ss61469556	*rs41571155*	7	100.457006	0.5623	0.0001	0.990839	0.0002	0.0001057319

ss61494277	*rs41595681*	7	100.579805	0.9405	3.1783	0.075056	0.0705	0.0078843088
	rs29015952	7	100.6029	0.4298	10.5683	0.001206	0.0613	0.0300298613
ss86336975		7	100.629673	0.5308	0.0685	0.793679	0.0048	0.0023660547
ss86290443		7	100.770417	0.9097	5.1720	0.023257	0.0735	0.0120750395
ss86308219		7	100.800038	0.3589	11.5995	0.000698	0.0639	0.0293905872
ss86307361		7	100.894204	0.3410	6.3135	0.012207	0.0488	0.0219382226
	rs29019536	7	101.006571	0.7092	13.3210	0.000282	0.0709	0.0292327573
ss61510835	*rs41607755*	7	101.083258	0.1089	5.9151	0.015263	0.0718	0.0139323527
ss86327509		7	101.281411	0.1426	4.0736	0.043941	0.0528	0.0129066232
ss86314844		7	101.320532	0.7285	0.9188	0.338127	0.0200	0.0078970857
ss86340624		7	101.380503	0.2765	4.3244	0.037936	0.0435	0.0173856722
ss61492395	*rs41597343*	7	101.480446	0.5501	1.6787	0.195522	0.0238	0.0117976136
ss61496620	*rs41591273*	7	101.53684	0.4434	1.5776	0.209522	0.0234	0.0115531301
ss117963941		7	101.607989	0.6347	2.1354	0.144383	0.0279	0.0129466877
ss61523714	*rs41622873*	7	101.648111	0.3890	2.2562	0.133530	0.0292	0.0138664655
ss117963928		7	101.671	0.5938	0.0488	0.825316	0.0042	0.0020272983
ss61559581	*rs41657405*	7	101.699847	0.4076	0.0172	0.895663	0.0025	0.0011944231
ss117969040		7	101.794324	0.2314	1.1338	0.287326	0.0237	0.0084235853
ss61498557	*rs43712657*	7	101.951515	0.6490	1.1775	0.278242	0.0217	0.0098698449
	rs29027660	7	102.131471	0.6605	1.7037	0.192238	0.0259	0.0116133838
ss86273767		7	102.351903	0.7672	0.3088	0.578624	0.0126	0.0044868898
ss61540558	*rs41644897*	7	102.434087	0.6082	0.2528	0.615245	0.0095	0.0045280550
	rs29015255	7	102.499012	0.0688	0.9181	0.338312	0.0353	0.0045271882
ss86318787		7	102.762373	0.2586	1.4511	0.228765	0.0262	0.0100652842
ss86337319		7	102.876595	0.2178	0.0062	0.937201	0.0018	0.0006105420
ss61492397	*rs41597345*	7	103.07112	0.4742	0.0835	0.772721	0.0054	0.0027089763
ss61559583	*rs43746618*	7	103.091829	0.4341	0.3342	0.563389	0.0107	0.0052797768
ss117968885		7	103.302145	0.6726	0.0286	0.865722	0.0033	0.0014349752
ss86314668		7	103.339926	0.1089	1.8838	0.170350	0.0390	0.0075744794
ss117969043		7	103.379367	0.3825	0.3980	0.528331	0.0120	0.0056627204
ss64505828	*rs42676285*	7	103.411819	0.4277	0.4164	0.518974	0.0120	0.0058885075
ss64495602	*rs42665334*	7	103.438374	0.4592	0.1131	0.736716	0.0063	0.0031067462
ss64495710	*rs42665388*	7	103.482104	0.2665	3.0041	0.083498	0.0372	0.0145392687
ss64495742	*rs42665404*	7	103.511536	0.6547	4.9004	0.027174	0.0441	0.0199309040
ss61513441	*rs41612121*	7	103.556746	0.2679	0.8085	0.368874	0.0191	0.0074925535
ss65602596	*rs43681794*	7	103.601009	0.2400	2.3920	0.122415	0.0348	0.0126893346
ss61498038	*rs43707450*	7	103.644612	0.6927	0.2607	0.609822	0.0104	0.0044460261
ss61515231	*rs41612889*	7	103.717241	0.5086	2.9980	0.083812	0.0320	0.0160042855
ss65602696	*rs43682391*	7	103.743577	0.2106	0.0859	0.769504	0.0068	0.0022558885
ss65602772	*rs43683267*	7	103.791944	0.4792	1.3680	0.242548	0.0217	0.0108156277
ss65602783	*rs43683278*	7	103.820399	0.2729	0.0849	0.770796	0.0062	0.0024511996
ss64823016	*rs42915745*	7	103.904288	0.5523	0.3071	0.579612	0.0103	0.0050910783
ss61476129	*rs41574792*	7	103.939526	0.2128	0.3548	0.551617	0.0133	0.0044483822
ss65452688	*rs43533656*	7	104.003205	0.4878	0.5299	0.466875	0.0136	0.0067946113
ss65452058	*rs43534024*	7	104.065538	0.7163	0.0778	0.780338	0.0058	0.0023599650
ss86336568		7	104.101316	0.7980	1.0901	0.296813	0.0238	0.0076859430
ss61523716	*rs41622875*	7	104.179359	0.8947	0.6824	0.409057	0.0254	0.0047797429

ss61559610	*rs41657430*	7	104.203277	0.6913	0.0173	0.895293	0.0027	0.0011408127
ss65452375	*rs43531443*	7	104.241937	0.2844	0.2964	0.586317	0.0112	0.0045751246
ss61492413	*rs41597361*	7	104.27168	0.7937	0.7458	0.388117	0.0200	0.0065489366
ss65452442	*rs43531510*	7	104.329278	0.3474	2.8625	0.091115	0.0331	0.0150142992
ss65452453	*rs43531521*	7	104.350905	0.2514	3.0058	0.083408	0.0377	0.0142078256
ss65452552	*rs43532220*	7	104.384053	0.2529	2.4095	0.121056	0.0337	0.0127249117
ss65452559	*rs43532227*	7	104.418983	0.7923	0.6362	0.425351	0.0187	0.0061616280
ss65451747	*rs43532413*	7	104.525769	0.5774	0.4808	0.488300	0.0130	0.0063605020
ss86321881		7	104.572419	0.8754	0.3027	0.582384	0.0155	0.0033867897
ss65451860	*rs43532926*	7	104.604981	0.0602	1.1311	0.287915	0.0409	0.0046228522
ss65451906	*rs43533572*	7	104.641305	0.6755	1.6464	0.199883	0.0256	0.0112305646
ss86290514		7	104.719139	0.9384	0.1939	0.659829	0.0170	0.0019632600
ss61568012	*rs41666510*	7	104.748666	0.8138	2.0361	0.154053	0.0337	0.0102293217
ss117969074		7	104.780199	0.5910	0.8527	0.356119	0.0175	0.0084594913
ss65095673	*rs43188982*	7	104.97781	0.3102	0.2639	0.607585	0.0103	0.0043944669
ss64341327	*rs42590690*	7	105.014382	0.4477	1.7219	0.189876	0.0249	0.0123003093
ss61511871	*rs41616175*	7	105.085833	0.1232	0.0009	0.975767	0.0008	0.0001819656
	rs29024551	7	105.125482	0.8639	5.5283	0.018989	0.0626	0.0147153622
ss63104298	*rs42118405*	7	105.245062	0.4821	0.0589	0.808259	0.0045	0.0022683104
ss61550450	*rs41650229*	7	105.27702	0.1805	2.4651	0.116853	0.0378	0.0111936712
ss86304478		7	105.350788	0.0910	0.5429	0.461480	0.0239	0.0039602334
ss61570526	*rs41668883*	7	105.382975	0.7708	4.8160	0.028527	0.0479	0.0169311326
ss61509848	*rs41607486*	7	105.48859	0.7715	0.3625	0.547292	0.0137	0.0048165790
		7	105.52115	0.1741	0.0096	0.921896	0.0024	0.0006939075
ss63104907	*rs42112216*	7	105.557331	0.2844	1.5339	0.215952	0.0258	0.0104992912
	rs29010461	7	105.595592	0.5967	0.0062	0.937025	0.0015	0.0007314815
ss86341751		7	105.617666	0.3990	0.4619	0.496964	0.0131	0.0063005426
ss61550461	*rs41651340*	7	105.643697	0.0989	0.0092	0.923695	0.0030	0.0005306261
ss86335820		7	105.683886	0.6948	1.6568	0.198467	0.0265	0.0112307645
ss86303318		7	105.716728	0.4670	1.5556	0.212730	0.0240	0.0119444026
ss63105800	*rs42116462*	7	105.743176	0.4850	4.4160	0.035963	0.0389	0.0194281874
ss63105882	*rs42116502*	7	105.769863	0.4864	4.9288	0.026734	0.0410	0.0204615153
ss86296035		7	105.796845	0.9069	2.3471	0.125973	0.0468	0.0079042639
ss86300697		7	105.825089	0.5788	3.4612	0.063248	0.0343	0.0167351324
ss86310910		7	105.879513	0.5845	2.9078	0.088596	0.0319	0.0155114816
ss63106729	*rs42121425*	7	105.905908	0.4620	0.7133	0.398625	0.0157	0.0077829691
ss86319321		7	105.930935	0.6411	0.7815	0.376982	0.0165	0.0075734151
ss86322915		7	105.972688	0.8825	1.2006	0.273589	0.0317	0.0065671791
ss63106996	*rs42112860*	7	105.996749	0.5107	0.6596	0.416972	0.0149	0.0074346086
ss86314228		7	106.068177	0.1626	1.4751	0.224961	0.0313	0.0085110985
ss61487920	*rs41586783*	7	106.098897	0.2063	2.4691	0.116559	0.0374	0.0122572940
ss86340188		7	106.311684	0.6984	1.3246	0.250172	0.0229	0.0096624054
ss86307699		7	106.47141	0.6497	1.0267	0.311285	0.0195	0.0088904344
ss61559605	*rs41657425*	7	106.599921	0.0580	0.6910	0.406105	0.0333	0.0036394627
ss61492403	*rs41597351*	7	106.68858	0.6268	0.0004	0.983879	0.0004	0.0001803478
ss86300838		7	106.837701	0.7987	2.2838	0.131184	0.0352	0.0113214684
ss61559588	*rs41657410*	7	106.918055	0.4964	0.5981	0.439561	0.0140	0.0070097095

ss61570676	*rs41670228*	7	106.943604	0.9155	0.0054	0.941568	0.0024	0.0003684716
ss61559593	*rs41657415*	7	107.01593	0.3832	0.1517	0.697003	0.0076	0.0035805555
ss86331941		7	107.069215	0.4821	0.0514	0.820720	0.0042	0.0021030496
ss86300229		7	107.109199	0.2228	0.7524	0.386005	0.0190	0.0065726672
ss86335644		7	107.129421	0.3646	1.6862	0.194536	0.0251	0.0116294796
ss64506396	*rs42668574*	7	107.184273	0.7428	0.4958	0.481580	0.0153	0.0058412549
ss61484367	*rs41578220*	7	107.255548	0.8288	0.1180	0.731364	0.0085	0.0024098317
ss65238430	*rs43323619*	7	107.311186	0.4463	3.8922	0.048907	0.0356	0.0175950977
ss117969123		7	107.345909	0.4026	0.1682	0.681865	0.0077	0.0037032414
ss86339396		7	107.392719	0.4642	0.1791	0.672278	0.0079	0.0039134320
ss61484372	*rs41578225*	7	107.455853	0.7479	3.8299	0.050744	0.0424	0.0159868191
ss64894848	*rs42989439*	7	107.500146	0.6461	0.5218	0.470336	0.0141	0.0064279627
ss65239226	*rs43324416*	7	107.642456	0.4771	0.0030	0.956539	0.0010	0.0005123074
ss86313055		7	107.665091	0.7822	2.0401	0.153652	0.0322	0.0109620789
ss65454410	*rs43536970*	7	107.689831	0.9176	0.1341	0.714324	0.0125	0.0018857123
ss61510842	*rs41607762*	7	107.712051	0.2658	1.7349	0.188224	0.0281	0.0109757124
ss61521526	*rs41619629*	7	107.808267	0.3861	8.2621	0.004172	0.0557	0.0263955189
ss64789080	*rs42884082*	7	107.83189	0.3861	8.2621	0.004172	0.0557	0.0263955189
	rs29019494	7	107.881813	0.2557	6.3583	0.011906	0.0550	0.0209402250
ss61559667	*rs41658086*	7	107.912492	0.2736	5.9768	0.014742	0.0499	0.0198527260
ss61492426	*rs41597374*	7	107.996289	0.1827	0.1230	0.725869	0.0084	0.0025057969
ss86325355		7	108.058107	0.2915	1.9866	0.159148	0.0294	0.0121283452
	rs29019418	7	108.101557	0.1540	0.1726	0.677952	0.0109	0.0028364151
ss65454061	*rs43537121*	7	108.13731	0.3052	0.0041	0.948813	0.0013	0.0005454667
ss86339193		7	108.181826	0.3947	5.0463	0.024991	0.0434	0.0207156235
ss61494471	*rs41597663*	7	108.232787	0.4785	4.8581	0.027843	0.0404	0.0201810667
ss86301518		7	108.316033	0.7450	2.1730	0.140907	0.0308	0.0116850134
ss65453783	*rs43536043*	7	108.346051	0.1096	0.9417	0.332174	0.0293	0.0057190200
ss61559655	*rs41658074*	7	108.412625	0.5229	8.5585	0.003551	0.0542	0.0270339309
ss61492421	*rs41597369*	7	108.504458	0.2772	0.2531	0.615059	0.0104	0.0041731817
ss65453553	*rs43533922*	7	108.530203	0.4692	0.4000	0.527289	0.0113	0.0056356030
ss86302782		7	108.605547	0.3295	0.0841	0.771855	0.0057	0.0025301627
ss86323652		7	108.62946	0.2314	0.2285	0.632764	0.0110	0.0038975570
ss86337455		7	108.729353	0.0745	0.7483	0.387304	0.0306	0.0042180217
ss61559638	*rs41658058*	7	108.755813	0.8675	0.6354	0.425655	0.0223	0.0051256791
ss64859714	*rs42952749*	7	108.8288	0.5272	0.7560	0.384874	0.0164	0.0081627611
ss86337725		7	108.855521	0.0874	0.8828	0.347757	0.0305	0.0048599746
ss64938378	*rs43027670*	7	108.914323	0.4183	2.4265	0.119756	0.0298	0.0144966082
ss86303483		7	108.973157	0.6426	0.2834	0.594682	0.0106	0.0048561163
ss86281965		7	109.046166	0.6476	0.1408	0.707624	0.0075	0.0034279005
ss86311623		7	109.125189	0.8517	1.4215	0.233563	0.0308	0.0077833883
ss61492420	*rs41597368*	7	109.280373	0.5774	0.9852	0.321260	0.0183	0.0089285948
ss65452907	*rs43535175*	7	109.301932	0.4191	1.7195	0.190190	0.0241	0.0117383973
ss61559634	*rs41658054*	7	109.339825	0.1648	3.0130	0.083043	0.0439	0.0120782155
ss61559631	*rs41658051*	7	109.437925	0.8403	2.2985	0.129950	0.0369	0.0099045511
ss61469295	*rs41568602*	7	109.463597	0.1640	2.4889	0.115104	0.0378	0.0103791412
ss86331666		7	109.536245	0.8589	0.3680	0.544296	0.0160	0.0038762014

ss117968878		7	109.711675	0.8496	7.0303	0.008196	0.0670	0.0171299046
	rs29017056	7	109.777701	0.6010	1.6738	0.196180	0.0235	0.0112905414
ss61475855	*rs41577424*	7	109.802048	0.5938	1.9541	0.162592	0.0254	0.0122352181
ss86283438		7	109.989872	0.4900	0.0074	0.931322	0.0016	0.0007859815
ss64934081	*rs43023667*	7	110.010993	0.4885	0.0012	0.972660	0.0006	0.0003134183
ss86285429		7	110.03212	0.4907	0.0021	0.963099	0.0008	0.0004213475
ss63878650	*rs41711247*	7	110.052509	0.5093	0.0021	0.963099	0.0008	0.0004213475
ss86332971		7	110.084098	0.5115	0.0012	0.972660	0.0006	0.0003134183
ss86338837		7	110.169243	0.4907	0.0021	0.963099	0.0008	0.0004213475
ss61524252	*rs41619208*	7	110.203989	0.1411	0.0000	0.999011	0.0000	0.0000080571
ss86292811		7	110.25756	0.1426	0.0294	0.863960	0.0046	0.0011202224
ss86328187		7	110.291407	0.1956	4.4875	0.034498	0.0486	0.0153010396
ss117968948		7	110.355843	0.3868	3.1409	0.076789	0.0338	0.0160399623
ss61559676	*rs41658095*	7	110.514981	0.3596	0.1924	0.661022	0.0084	0.0038639302
ss117969138		7	110.569116	0.2256	0.0007	0.978277	0.0006	0.0002074397
ss61492439	*rs41597387*	7	110.593287	0.1024	0.1140	0.735785	0.0104	0.0019198698
	rs29013903	7	110.624388	0.4140	2.1764	0.140592	0.0272	0.0131963065
ss86275930		7	110.64505	0.4097	2.0502	0.152635	0.0266	0.0128830066
ss61510849	*rs41607769*	7	110.703019	0.3761	2.7809	0.095844	0.0324	0.0151821268
ss86309982		7	110.746796	0.1963	0.8329	0.361742	0.0220	0.0069311573
ss65204481	*rs43294959*	7	110.794993	0.2135	1.1780	0.278147	0.0252	0.0084758015
ss117963920		7	110.838847	0.7049	0.0153	0.901517	0.0026	0.0010888782
ss65204590	*rs43296066*	7	110.882994	0.8926	1.9509	0.162935	0.0415	0.0079665437
ss65455659	*rs43540216*	7	110.923314	0.7063	0.6535	0.419132	0.0164	0.0068120496
ss86336907		7	110.953657	0.2228	2.2990	0.129913	0.0327	0.0113219788
ss61492454	*rs41597402*	7	110.977902	0.5136	1.5964	0.206842	0.0237	0.0118402324
ss61559690	*rs41658109*	7	111.019866	0.2414	2.4615	0.117121	0.0345	0.0126346190
ss86314316		7	111.069469	0.5301	0.7405	0.389784	0.0161	0.0079977820
ss86327443		7	111.098878	0.5881	1.0088	0.315544	0.0189	0.0091531957
ss61473351	*rs41569770*	7	111.124242	0.4957	0.0152	0.901841	0.0023	0.0011489427
ss61542548	*rs41646063*	7	111.146516	0.1297	0.4279	0.513217	0.0180	0.0040692391
ss86285665		7	111.175223	0.3632	0.4465	0.504218	0.0126	0.0058419973
ss65204814	*rs43289593*	7	111.201049	0.4914	0.0608	0.805255	0.0045	0.0022402214
ss65456259	*rs43536617*	7	111.264376	0.2221	2.8870	0.089743	0.0378	0.0130712205
ss86298174		7	111.312257	0.2120	5.4953	0.019348	0.0519	0.0173306795
	rs29017214	7	111.35204	0.7113	1.9833	0.159493	0.0285	0.0117020584
ss86309625		7	111.393021	0.6848	0.1084	0.742037	0.0065	0.0028118720
ss61492463	*rs41597411*	7	111.427541	0.2543	0.1057	0.745160	0.0070	0.0026592596
ss86338082		7	111.465529	0.2256	4.0321	0.045028	0.0434	0.0151760153
ss65455789	*rs43541946*	7	111.525209	0.5745	3.2499	0.071859	0.0341	0.0166657864
ss117963947		7	111.547923	0.3703	1.0642	0.302620	0.0198	0.0092232046
ss117969110		7	111.575808	0.4656	0.2577	0.611840	0.0094	0.0046651876
ss65455686	*rs43541043*	7	111.614427	0.1447	1.2604	0.261969	0.0296	0.0073331878
ss64749843	*rs42843241*	7	111.649814	0.5559	0.0503	0.822626	0.0042	0.0020505870
ss86316411		7	111.675447	0.4026	1.1467	0.284605	0.0199	0.0095880044
	rs29025231	7	111.707944	0.4126	0.2350	0.628014	0.0090	0.0043463220
ss64500684	*rs42669594*	7	111.7613	0.6024	0.0408	0.840000	0.0038	0.0018336282

	rs29016860	7	111.801945	0.3625	0.5837	0.445121	0.0146	0.0067613546
ss63662774	rs42309214	7	112.064213	0.6461	0.2983	0.585117	0.0105	0.0048193595
ss61563379	rs41660853	8	0.0117	0.6039	0.0225	0.880797	0.0028	0.0013424206
ss86304486		8	0.28017	0.2242	1.2882	0.256775	0.0253	0.0087894179
ss86341204		8	0.305873	0.7837	1.6505	0.199314	0.0289	0.0098035256
ss86280264		8	0.355813	0.6827	0.1496	0.699037	0.0077	0.0033445251
ss61495408	rs41596781	8	0.394503	0.5494	1.8782	0.170977	0.0252	0.0124930945
ss86301328		8	0.426519	0.4470	1.6995	0.192790	0.0245	0.0121143641
ss86320131		8	0.504129	0.4620	0.3593	0.549103	0.0114	0.0056831422
ss86308959		8	0.534384	0.5143	1.8060	0.179431	0.0251	0.0125255770
ss86289867		8	0.567528	0.3660	0.1267	0.721992	0.0070	0.0032667845
ss86318676		8	0.616591	0.7070	2.2075	0.137793	0.0303	0.0125406470
ss61560899	rs41662006	8	0.656679	0.1855	0.2112	0.645941	0.0111	0.0033512733
ss86298639		8	0.680175	0.7156	2.1840	0.139902	0.0303	0.0123125678
ss86273934		8	0.700593	0.1777	0.6897	0.406557	0.0204	0.0059637891
ss86341473		8	0.738529	0.0673	15.4382	0.000094	0.1446	0.0181629033
ss46526300	rs41255439	8	0.79182	0.5960	1.3378	0.247821	0.0218	0.0105031503
ss86288629		8	0.874007	0.0845	0.0114	0.914962	0.0036	0.0005517699
ss86304221		8	0.908	0.2393	0.1568	0.692266	0.0086	0.0031155733
	rs29017545	8	0.942551	0.7378	0.0011	0.973404	0.0007	0.0002713570
	rs29016504	8	1.016023	0.4527	0.4721	0.492244	0.0127	0.0063172616
ss117969338		8	1.116319	0.7822	4.7034	0.030441	0.0506	0.0172240168
ss86341615		8	1.176327	0.2228	3.5874	0.058634	0.0439	0.0152090942
ss86321389		8	1.296715	0.5953	0.0392	0.843089	0.0038	0.0018333347
ss86329977		8	1.41067	0.3202	0.0190	0.890409	0.0028	0.0012019739
ss61473049	rs41566470	8	1.452491	0.0874	0.0891	0.765417	0.0098	0.0015708229
ss61473057	rs41566478	8	1.486623	0.5931	1.1423	0.285543	0.0204	0.0098322286
ss86341668		8	1.581185	0.7643	0.2275	0.633562	0.0107	0.0038409871
ss86306745		8	1.603297	0.6125	0.6481	0.421059	0.0154	0.0073247752
ss86292787		8	1.629615	0.9398	3.8645	0.049715	0.0783	0.0088533896
ss86291472		8	1.652288	0.4577	0.5248	0.469055	0.0136	0.0067572552
ss61517830	rs41619093	8	1.678816	0.5244	13.1521	0.000308	0.0687	0.0342798708
ss117969424		8	1.869793	0.5917	9.8242	0.001795	0.0588	0.0283886421
ss86323610		8	1.95775	0.1526	0.5258	0.468609	0.0191	0.0049509906
ss86304991		8	2.197308	0.9277	0.8757	0.349694	0.0342	0.0045932585
ss86340104		8	2.293641	0.1977	5.6958	0.017272	0.0550	0.0174628028
ss86336436		8	2.488721	0.2858	0.0030	0.956630	0.0012	0.0004705755
ss61500326		8	2.508974	0.7693	0.5704	0.450345	0.0169	0.0060148686
ss86333228		8	2.538248	0.2758	1.3441	0.246705	0.0253	0.0101236208
ss86298910		8	2.827532	0.8868	0.4530	0.501158	0.0202	0.0040503485
ss61559878	rs41659595	8	2.943624	0.3718	2.7175	0.099705	0.0319	0.0148857729
ss86340263		8	2.984067	0.6977	0.0089	0.924877	0.0019	0.0007941095
ss117969472		8	3.154189	0.8617	0.7941	0.373181	0.0242	0.0057620387
ss86336665		8	3.216922	0.3832	0.0765	0.782184	0.0054	0.0025643279
ss86339714		8	3.29082	0.5645	0.2955	0.586863	0.0104	0.0051284260
ss86339420		8	3.39617	0.0974	4.2390	0.039877	0.0664	0.0116856585
ss61496462	rs41598711	8	3.428274	0.5867	0.1455	0.703028	0.0073	0.0035416878

ss86338077		8	3.520746	0.5179	4.0562	0.044393		0.0387	0.0193369806
ss64774965	*rs42871524*	8	3.602911	0.5544	3.6933	0.055040		0.0365	0.0180420132
ss61563239	*rs41659319*	8	3.671308	0.4678	2.1838	0.139924		0.0284	0.0141255740
ss63951620	*rs42400277*	8	3.696293	0.4986	4.7138	0.030259		0.0410	0.0204986812
ss61525210	*rs41619243*	8	3.728164	0.1683	4.2799	0.038935		0.0522	0.0146030222
ss117964015		8	3.820329	0.1612	2.0664	0.151022		0.0365	0.0098669658
ss63951114	*rs42403123*	8	3.969083	0.0953	5.7465	0.016784		0.0763	0.0131601000
ss63951102	*rs42403117*	8	3.996552	0.0845	1.6067	0.205375		0.0432	0.0066796249
ss61562625	*rs41662822*	8	4.139375	0.6705	1.7739	0.183340		0.0258	0.0113944091
	rs29015318	8	4.16884	0.8216	0.3001	0.583981		0.0129	0.0037955297
ss64563872	*rs42701990*	8	4.202572	0.6705	1.7739	0.183340		0.0258	0.0113944091
ss61562626	*rs41662823*	8	4.251416	0.1791	0.5966	0.440146		0.0183	0.0053698652
ss64972277	*rs43063489*	8	4.286789	0.1798	0.4483	0.503362		0.0158	0.0046662199
ss64652485	*rs42751090*	8	4.393189	0.3087	0.2178	0.640881		0.0093	0.0039509728
ss65005830	*rs43097793*	8	4.511934	0.6053	0.5578	0.455386		0.0144	0.0069018568
ss61566108	*rs41660952*	8	4.555303	0.8789	5.1154	0.024022		0.0633	0.0134622381
ss61566111	*rs41660955*	8	4.694715	0.0638	7.1856	0.007523		0.0992	0.0118464776
	rs29025491	8	4.723366	0.3395	1.5425	0.214666		0.0250	0.0111977647
ss61507162		8	4.760919	0.1125	0.0049	0.944379		0.0021	0.0004118843
ss64829200	*rs42920892*	8	4.83313	0.2593	1.9150	0.166849		0.0286	0.0109812555
ss61526820	*rs41626391*	8	4.87753	0.1848	2.3598	0.124953		0.0371	0.0111693941
ss64653067	*rs42758168*	8	4.900994	0.0681	7.5946	0.006008		0.1041	0.0131985848
ss64653075	*rs42758176*	8	4.926727	0.2672	0.0018	0.965840		0.0009	0.0003585667
ss61516718	*rs41618019*	8	4.966617	0.7414	0.0054	0.941566		0.0016	0.0006174209
ss86298498		8	4.998893	0.5552	0.0217	0.882827		0.0028	0.0013935357
ss64597696	*rs42712847*	8	5.025696	0.1504	7.3895	0.006724		0.0695	0.0177555510
ss86339137		8	5.084378	0.6683	0.4450	0.504954		0.0131	0.0058272051
ss86337700		8	5.108359	0.5251	0.9010	0.342841		0.0176	0.0087998082
ss86274549		8	5.133332	0.5229	1.2060	0.272506		0.0204	0.0101953850
ss86291421		8	5.163179	0.6246	0.0016	0.968063		0.0008	0.0003527643
ss86339574		8	5.196132	0.4807	0.8693	0.351476		0.0173	0.0086182654
ss61485007	*rs41583336*	8	5.277544	0.2199	1.5199	0.218048		0.0282	0.0096633026
ss61560112	*rs41656436*	8	5.360216	0.2944	6.8734	0.008940		0.0530	0.0220301368
ss61560121	*rs41656445*	8	5.406137	0.1039	3.6449	0.056653		0.0567	0.0105590165
ss61492661		8	5.452118	0.7729	9.6121	0.002011		0.0679	0.0238293158
ss61560138	*rs41656462*	8	5.477232	0.0738	5.1305	0.023816		0.0811	0.0110786767
ss86291042		8	5.505897	0.3739	14.8335	0.000128		0.0727	0.0340218468
ss61469327	*rs41569434*	8	5.541397	0.3703	4.6888	0.030698		0.0427	0.0199360340
ss65457430	*rs43540473*	8	5.567016	0.1891	7.2986	0.007069		0.0645	0.0197695687
ss65457472	*rs43540515*	8	5.627475	0.2894	6.2406	0.012715		0.0499	0.0205286883
ss86293383		8	5.703635	0.5358	9.9482	0.001679		0.0567	0.0281799185
ss61492706	*rs41589856*	8	5.776925	0.2794	3.8747	0.049415		0.0416	0.0167554205
ss65457611	*rs43542854*	8	5.802879	0.8646	2.5284	0.112265		0.0420	0.0098405918
ss86336637		8	5.843055	0.6762	8.5096	0.003647		0.0557	0.0243733877
ss64832219	*rs42925203*	8	5.869534	0.8711	3.2472	0.071977		0.0482	0.0108303769
ss65457809	*rs43545252*	8	5.991802	0.5487	4.1257	0.042615		0.0378	0.0187236734
ss65457912	*rs43546255*	8	6.05749	0.8152	0.6582	0.417480		0.0191	0.0057465961

ss86335203		8	6.088789	0.5924	0.1111	0.739045	0.0064	0.0030713282
ss61531703	*rs41629255*	8	6.369477	0.3266	0.0546	0.815277	0.0046	0.0020184649
ss61560344	*rs41658264*	8	6.398541	0.8696	0.9670	0.325775	0.0281	0.0063752267
ss65458162	*rs43548504*	8	6.430122	0.7034	7.3077	0.007033	0.0556	0.0232011179
ss61522693	*rs41621464*	8	6.458794	0.6719	9.8713	0.001750	0.0609	0.0268456554
ss61475209	*rs41572696*	8	6.544486	0.6726	9.9229	0.001702	0.0618	0.0272001382
ss65458641	*rs43540985*	8	6.714834	0.1626	0.0914	0.762507	0.0076	0.0020761537
ss117969297		8	6.746055	0.4542	4.2224	0.040266	0.0387	0.0192079795
ss86337575		8	6.769906	0.8009	24.3851	0.000001	0.1137	0.0362586464
ss64721703	*rs42819192*	8	6.798635	0.5638	5.5880	0.018357	0.0446	0.0219165872
ss64721709	*rs42819198*	8	6.82294	0.5616	5.7583	0.016673	0.0452	0.0222717273
ss61492572	*rs41588323*	8	7.253694	0.5372	3.2332	0.072594	0.0330	0.0164312277
ss86341164		8	7.384682	0.5029	0.0189	0.890703	0.0026	0.0013072272
ss86318464		8	7.42614	0.9327	1.3810	0.240338	0.0432	0.0054305720
ss86303940		8	7.456284	0.5086	4.1050	0.043137	0.0381	0.0190644139
ss86309523		8	7.497689	0.5208	0.6699	0.413380	0.0152	0.0075914947
ss86311225		8	7.537876	0.5989	1.9357	0.164584	0.0261	0.0125326063
ss86302983		8	7.727459	0.3703	7.1729	0.007576	0.0511	0.0238234760
ss61560014	*rs41655541*	8	7.749008	0.9033	1.2912	0.256224	0.0362	0.0063168739
ss61528193	*rs41631232*	8	7.803955	0.7865	0.0672	0.795550	0.0058	0.0019558607
ss86335865		8	7.956687	0.8467	0.1807	0.670908	0.0112	0.0029063586
ss86313909		8	8.021322	0.1504	0.7942	0.373155	0.0232	0.0059320974
ss86288401		8	8.054729	0.6597	1.6956	0.193293	0.0255	0.0114279624
	rs29011988	8	8.127601	0.4269	0.3036	0.581841	0.0108	0.0052911027
ss86290901		8	8.154064	0.8582	3.2572	0.071543	0.0500	0.0121671342
ss86298339		8	8.190408	0.5981	4.6188	0.031967	0.0400	0.0192470796
ss86273939		8	8.259374	0.4527	1.4509	0.228797	0.0224	0.0111178853
ss61493996	*rs41592207*	8	8.350609	0.7973	0.6469	0.421510	0.0193	0.0062230972
ss86289890		8	8.381039	0.1239	0.0006	0.980014	0.0007	0.0001561670
ss86329057		8	8.405673	0.4592	1.3971	0.237616	0.0214	0.0106341579
ss86322896		8	8.507052	0.8030	4.5261	0.033732	0.0524	0.0165742138
ss61521868	*rs41622366*	8	8.54674	0.8474	1.2746	0.259301	0.0290	0.0074893863
ss86278368		8	8.797188	0.5050	0.1097	0.740606	0.0063	0.0031504910
ss86338005		8	8.903526	0.8166	0.0838	0.772296	0.0070	0.0021022184
ss86280332		8	8.958329	0.3675	7.0764	0.007991	0.0507	0.0235542137
ss86295563		8	9.098594	0.4499	1.6387	0.200934	0.0237	0.0117412007
ss86299653		8	9.13915	0.0946	1.3035	0.253967	0.0362	0.0062042675
	rs29020666	8	9.195348	0.3338	7.3061	0.007040	0.0522	0.0231945303
ss65361734	*rs43447696*	8	9.233285	0.6203	1.1833	0.277071	0.0211	0.0099600806
ss86284595		8	9.300077	0.8646	2.7554	0.097376	0.0460	0.0107600020
ss86332089		8	9.338787	0.4291	0.3361	0.562291	0.0108	0.0053121899
ss86302750		8	9.361847	0.1655	2.3403	0.126521	0.0378	0.0104368128
ss86319968		8	9.399789	0.3001	6.9301	0.008664	0.0526	0.0221065203
ss62393813	*rs41864434*	8	9.469143	0.6074	1.5686	0.210827	0.0232	0.0110451548
ss86334270		8	9.497028	0.4656	1.0873	0.297432	0.0201	0.0100272398
ss61539948	*rs41638903*	8	9.540174	0.8274	0.2581	0.611588	0.0126	0.0035890331
ss61539871	*rs41637926*	8	9.56806	0.1712	0.3454	0.556908	0.0146	0.0041318835

ss86332191		8	9.605961	0.7464	0.0287	0.865579	0.0036	0.0013739639
ss86290918		8	9.639135	0.1404	0.5857	0.444353	0.0206	0.0049783079
ss86325105		8	9.711848	0.1196	2.4385	0.118845	0.0452	0.0095221929
ss62392715	*rs41860668*	8	9.747088	0.1870	2.5598	0.110067	0.0375	0.0114059669
ss62392535	*rs41859808*	8	9.778897	0.8023	1.4956	0.221757	0.0281	0.0089124936
ss62392058	*rs41858749*	8	9.812005	0.1368	1.0936	0.296031	0.0291	0.0068718081
ss61482994	*rs41583668*	8	9.848206	0.1347	1.2011	0.273486	0.0306	0.0071376707
ss86340014		8	10.015674	0.7557	1.7267	0.189262	0.0284	0.0104818693
	rs29020862	8	10.171258	0.6519	5.0832	0.024469	0.0441	0.0200084213
ss65458767	*rs43541811*	8	10.223401	0.2135	0.7919	0.373845	0.0201	0.0067459301
ss65458869	*rs43542313*	8	10.262317	0.2006	0.6610	0.416483	0.0189	0.0060555549
ss86291312		8	10.349402	0.3854	1.3196	0.251058	0.0215	0.0101959044
ss61504637	*rs41608510*	8	10.43721	0.8417	1.7903	0.181327	0.0342	0.0091143660
ss86341043		8	10.516588	0.1103	5.1274	0.023858	0.0651	0.0127793505
ss86337635		8	10.647555	0.7070	3.0322	0.082068	0.0354	0.0146721518
ss86282958		8	10.668468	0.6125	0.4244	0.514969	0.0125	0.0059328336
ss61492984	*rs41591732*	8	10.713029	0.8360	6.8252	0.009182	0.0601	0.0164814484
ss61492988	*rs41591735*	8	10.76925	0.3446	1.8178	0.178012	0.0259	0.0117046288
ss86315863		8	10.789758	0.3266	1.6228	0.203133	0.0251	0.0110589696
ss86299189		8	10.823846	0.8302	1.8403	0.175360	0.0334	0.0094208434
ss86327674		8	10.84685	0.7622	0.2768	0.598957	0.0115	0.0041772025
ss86334320		8	10.90208	0.1669	2.7200	0.099549	0.0406	0.0112812217
ss86338775		8	10.947034	0.4477	1.4441	0.229886	0.0221	0.0109200714
ss46526301	*rs41255440*	8	11.023242	0.9026	1.6282	0.202373	0.0400	0.0070411300
ss86338381		8	11.071699	0.9183	0.1932	0.660378	0.0147	0.0022055645
	rs29023295	8	11.137155	0.0989	0.1698	0.680433	0.0133	0.0023621019
ss61493034	*rs41591781*	8	11.196224	0.4491	1.6553	0.198664	0.0242	0.0119821896
ss86334095		8	11.227774	0.5759	6.5801	0.010521	0.0480	0.0234270281
ss86333815		8	11.285156	0.4126	3.1054	0.078474	0.0332	0.0160718446
ss86290076		8	11.332423	0.7765	0.2528	0.615264	0.0114	0.0039416434
ss86338618		8	11.371807	0.3510	4.2759	0.039025	0.0405	0.0184481818
ss86290057		8	11.558499	0.7744	0.9049	0.341799	0.0214	0.0074663408
ss61492882	*rs41590630*	8	11.578561	0.1275	4.9698	0.026112	0.0615	0.0136905499
ss61504616	*rs41608489*	8	11.6191	0.2156	0.2337	0.628925	0.0106	0.0035895213
ss61560895	*rs41662002*	8	11.649044	0.1275	4.9698	0.026112	0.0615	0.0136905499
ss86339446		8	11.698893	0.7049	0.4265	0.513913	0.0132	0.0055101680
ss86335575		8	11.721121	0.6447	5.4175	0.020221	0.0444	0.0203514554
ss61493035	*rs41591782*	8	11.858677	0.8159	0.7635	0.382532	0.0208	0.0062522518
	rs29024272	8	11.938975	0.4054	0.2908	0.589867	0.0102	0.0049224634
ss61560901	*rs41662008*	8	11.963819	0.2113	2.1579	0.142287	0.0331	0.0110360155
ss86295579		8	12.135315	0.3797	16.5501	0.000053	0.0772	0.0363848763
ss86341343		8	12.228846	0.4979	8.1591	0.004412	0.0527	0.0263443402
ss86290709		8	12.298863	0.6913	2.3247	0.127791	0.0302	0.0129089960
ss61531699	*rs41629251*	8	12.600853	0.6067	1.7792	0.182685	0.0251	0.0119817253
ss61531700	*rs41629252*	8	12.699661	0.3188	0.7488	0.387144	0.0170	0.0073842478
ss61526481	*rs41623672*	8	12.720833	0.6526	1.0381	0.308624	0.0199	0.0090084578
ss61522644	*rs41620815*	8	12.939344	0.4319	2.3411	0.126455	0.0275	0.0134721825

ss61560902	rs41662009	8	12.997184	0.4835	0.0761	0.782694	0.0052	0.0025895049
ss86300020		8	13.181581	0.8388	11.2983	0.000818	0.0826	0.0223425860
ss61469369	rs41569476	8	13.222866	0.5451	0.3414	0.559234	0.0112	0.0055571249
ss86307105		8	13.269093	0.1311	5.8173	0.016127	0.0600	0.0136579667
ss86307437		8	13.431912	0.2170	4.6658	0.031109	0.0506	0.0171956282
ss117969458		8	13.562781	0.7242	2.6955	0.101081	0.0348	0.0139141914
ss86335852		8	13.621831	0.7385	0.1599	0.689345	0.0085	0.0032897778
ss86317816		8	13.680888	0.6913	1.7712	0.183671	0.0258	0.0110164227
ss86295358		8	13.789617	0.6719	0.9880	0.320587	0.0195	0.0086026509
ss117969485		8	13.862405	0.7221	0.7914	0.373972	0.0181	0.0072808565
ss61560934	rs41655952	8	13.976912	0.6683	0.7155	0.397925	0.0167	0.0073841304
ss86341716		8	14.129793	0.0602	11.7181	0.000655	0.1322	0.0149550241
ss61560944	rs41655962	8	14.284239	0.3302	0.1454	0.703104	0.0070	0.0030854984
ss61560954	rs41655972	8	14.310664	0.8732	4.0851	0.043645	0.0570	0.0126167911
	rs29014860	8	14.333391	0.6096	0.2813	0.596039	0.0101	0.0047933421
ss86336023		8	14.590831	0.8818	0.9402	0.332557	0.0290	0.0060515898
ss61478805	rs41577985	8	14.650089	0.1189	0.0476	0.827349	0.0063	0.0013187415
ss61495486	rs41597956	8	14.684959	0.8646	3.1095	0.078276	0.0475	0.0111276638
ss86283851		8	14.731874	0.3968	1.6213	0.203330	0.0240	0.0114697643
ss61562283	rs41659184	8	14.754084	0.8166	0.5145	0.473451	0.0169	0.0050569621
ss61507168		8	14.891603	0.1791	2.7070	0.100358	0.0395	0.0116151668
ss65459487	rs43540319	8	15.041047	0.3746	4.2399	0.039856	0.0397	0.0186210139
ss63371277	rs42204367	8	15.140103	0.8675	0.3353	0.562762	0.0160	0.0036750394
ss86289746		8	15.196205	0.4198	0.5782	0.447272	0.0136	0.0066288708
ss65219359	rs43306765	8	15.608418	0.2822	7.8484	0.005228	0.0563	0.0228119720
ss86303322		8	15.653104	0.5064	1.4512	0.228738	0.0222	0.0111105604
ss86324616		8	15.726186	0.3259	3.7063	0.054614	0.0386	0.0169477011
ss65219774	rs43311280	8	15.748252	0.8223	0.1405	0.707924	0.0094	0.0027356453
ss86281318		8	15.772082	0.1648	7.8416	0.005247	0.0723	0.0199015262
ss64750020	rs42844216	8	15.808005	0.2199	0.4351	0.509737	0.0149	0.0050993373
ss65219838	rs43303344	8	15.865122	0.4255	1.6935	0.193574	0.0246	0.0120381793
ss86325155		8	15.913958	0.2908	0.0574	0.810664	0.0047	0.0019363449
ss64941943	rs43037454	8	15.940104	0.6991	0.1763	0.674742	0.0083	0.0034730152
ss86335423		8	15.996793	0.4907	3.7032	0.054716	0.0368	0.0183826992
ss64465486	rs42653396	8	16.031831	0.5208	2.5037	0.114034	0.0303	0.0151401256
ss86339116		8	16.07219	0.3625	4.2694	0.039175	0.0397	0.0183608393
ss61475923	rs41578388	8	16.15107	0.0702	0.2105	0.646506	0.0162	0.0021192872
ss64922356	rs43017780	8	16.194448	0.7514	0.4364	0.509064	0.0144	0.0053934110
ss61528016	rs41629157	8	16.233551	0.1404	0.2951	0.587154	0.0148	0.0035803158
ss61560964	rs41655982	8	16.36965	0.3668	12.2564	0.000493	0.0660	0.0306541095
ss86337176		8	16.457914	0.4126	8.2795	0.004132	0.0538	0.0260743721
ss65459714	rs43543740	8	16.524496	0.7765	0.3796	0.538004	0.0138	0.0047888614
ss61560968	rs41655986	8	16.555803	0.1232	3.6841	0.055341	0.0558	0.0120626442
ss65459855	rs43545081	8	16.616814	0.8059	2.8517	0.091724	0.0407	0.0127363039
ss65459780	rs43543806	8	16.652419	0.6254	2.3297	0.127382	0.0292	0.0136674267
ss65459890	rs43545116	8	16.835603	0.8037	2.6672	0.102885	0.0393	0.0124035398
ss65459981	rs43546207	8	16.87696	0.1590	0.9483	0.330500	0.0249	0.0066538662

	rs29027941	8	16.98776	0.3417	2.8796	0.090156	0.0335	0.0150585278
	rs29010780	8	17.008969	0.1017	0.3782	0.538745	0.0189	0.0034566929
ss86322615		8	17.094003	0.8431	1.2125	0.271225	0.0285	0.0075508810
ss61559738	*rs41658756*	8	17.134669	0.7199	0.0004	0.983737	0.0004	0.0001707010
ss86293105		8	17.158174	0.7708	8.5604	0.003547	0.0652	0.0230439596
ss65460529	*rs43541254*	8	17.246223	0.1691	2.0889	0.148822	0.0358	0.0100672033
ss61559746	*rs41658764*	8	17.272741	0.1569	2.0747	0.150213	0.0368	0.0097447007
ss61559744	*rs41658762*	8	17.339634	0.3209	3.1118	0.078166	0.0355	0.0154847722
ss86312630		8	17.386435	0.6719	1.4703	0.225705	0.0239	0.0105308477
ss86334335		8	17.492385	0.8152	0.0085	0.926521	0.0022	0.0006715052
ss86336451		8	17.513183	0.2851	0.8959	0.344207	0.0199	0.0081282605
ss86340492		8	17.59571	0.3360	1.2779	0.258677	0.0221	0.0098661370
ss86279584		8	17.834458	0.6447	0.4948	0.482028	0.0136	0.0062384267
ss86337125		8	17.894667	0.9054	0.5623	0.453606	0.0240	0.0041142969
ss65460933	*rs43546058*	8	17.933117	0.5494	1.6845	0.194757	0.0241	0.0119105777
ss65461067	*rs43547192*	8	18.009672	0.6053	0.7525	0.385971	0.0164	0.0078428172
ss117969230		8	18.048902	0.2457	0.0071	0.932951	0.0018	0.0006608746
ss86319875		8	18.19028	0.4971	6.0700	0.013991	0.0455	0.0227280963
ss86329472		8	18.292019	0.5466	2.3774	0.123561	0.0287	0.0142161724
ss86318504		8	18.370608	0.6497	4.5480	0.033307	0.0419	0.0190609703
ss86273644		8	18.499827	0.9198	14.0021	0.000198	0.1300	0.0191891522
ss61568101	*rs41661406*	8	18.526495	0.3646	0.3837	0.535828	0.0119	0.0055217502
	rs29011096	8	18.563139	0.7908	0.0595	0.807389	0.0056	0.0018434015
ss117969474		8	18.644876	0.2622	0.3193	0.572214	0.0119	0.0045849197
ss64752344	*rs42848878*	8	18.896696	0.3367	2.4430	0.118502	0.0307	0.0136969971
ss64752296	*rs42847930*	8	18.927383	0.7228	0.1962	0.657908	0.0091	0.0036307840
ss63511124	*rs42256578*	8	18.990741	0.8904	0.1738	0.676910	0.0123	0.0023917321
ss63511196	*rs42256602*	8	19.017814	0.9291	0.7381	0.390561	0.0303	0.0039944815
	rs29014938	8	19.059864	0.1340	2.2357	0.135306	0.0396	0.0091828510
ss63511589	*rs42252233*	8	19.108638	0.4033	4.9642	0.026196	0.0415	0.0199792356
ss86302210		8	19.147865	0.3302	0.0020	0.964023	0.0009	0.0003951039
ss63512069	*rs42253191*	8	19.211204	0.2758	0.4602	0.497738	0.0138	0.0055299060
ss86310493		8	19.235646	0.2049	0.0537	0.816759	0.0052	0.0016816723
ss61528744	*rs41628670*	8	19.273191	0.4943	3.3751	0.066615	0.0329	0.0164660558
ss63512225	*rs42253443*	8	19.301041	0.7521	0.4514	0.501896	0.0143	0.0053291490
ss61477690	*rs41578619*	8	19.327953	0.2077	0.6481	0.421063	0.0182	0.0059880158
ss63512381	*rs42253495*	8	19.366437	0.9585	1.5462	0.214122	0.0574	0.0045684581
ss117964006		8	19.404855	0.6841	1.3045	0.253782	0.0229	0.0099079098
ss86328827		8	19.459909	0.2106	3.2455	0.072053	0.0410	0.0136344670
	rs29012369	8	19.486653	0.2987	0.0023	0.962124	0.0010	0.0004254771
ss61528747	*rs41628673*	8	19.589107	0.1920	0.8273	0.363361	0.0211	0.0065428686
ss61528664	*rs41627297*	8	19.65739	0.8395	2.8786	0.090212	0.0421	0.0113376319
ss65188814	*rs43273003*	8	19.805984	0.3245	1.7473	0.186646	0.0257	0.0112827329
ss63513980	*rs42256127*	8	19.858978	0.2572	0.1982	0.656351	0.0095	0.0036368145
ss64921709	*rs43011042*	8	20.026265	0.1633	0.2001	0.654798	0.0111	0.0030381487
ss61511955	*rs41609668*	8	20.161751	0.6196	0.1332	0.715271	0.0070	0.0033203028
ss64386849	*rs42612776*	8	20.215966	0.3532	0.8994	0.343260	0.0186	0.0084887689

ss61502688	*rs41608993*	8	20.288845	0.6734	0.7898	0.374478	0.0175	0.0076918851
ss86274610		8	20.350058	0.7070	0.3007	0.583594	0.0111	0.0046039626
	rs29023279	8	20.452102	0.1941	0.1797	0.671758	0.0103	0.0032079189
ss61485613		8	20.476821	0.4563	0.4008	0.526864	0.0116	0.0057797123
ss61479181	*rs41576152*	8	20.504189	0.8252	2.2858	0.131019	0.0372	0.0107380760
ss86341305		8	20.536779	0.8481	0.2048	0.650975	0.0118	0.0030492650
ss61500742	*rs41599497*	8	20.584598	0.8997	0.0603	0.806167	0.0079	0.0014171334
ss64136219	*rs42488893*	8	20.609108	0.1447	0.0214	0.883664	0.0040	0.0009959732
ss61479182	*rs41576153*	8	20.63658	0.4448	4.5914	0.032478	0.0400	0.0197491710
ss61519493	*rs41615642*	8	20.681563	0.2400	1.0145	0.314182	0.0222	0.0081110837
ss64891251	*rs42984188*	8	20.735091	0.5566	4.5179	0.033894	0.0398	0.0196461713
ss86281023		8	20.893495	0.1941	0.6717	0.412743	0.0192	0.0060035305
ss86337944		8	21.016274	0.4377	0.3581	0.549735	0.0111	0.0054802109
ss86304552		8	21.06427	0.4814	0.0215	0.883418	0.0028	0.0013811887
ss61532080	*rs41632514*	8	21.085812	0.8009	2.3525	0.125533	0.0368	0.0117244087
ss86288394		8	21.131013	0.6734	5.8211	0.016093	0.0465	0.0204425567
ss64891909	*rs42991142*	8	21.159543	0.8768	1.8253	0.177124	0.0378	0.0081743260
	rs29017104	8	21.187193	0.5387	1.1511	0.283689	0.0205	0.0101961064
	rs29021936	8	21.215936	0.5602	0.6297	0.427730	0.0151	0.0074291620
ss86340374		8	21.25969	0.5072	1.3621	0.243577	0.0220	0.0109919934
ss86284600		8	21.290586	0.1089	0.9437	0.331657	0.0292	0.0056594890
ss86288744		8	21.337907	0.1189	2.7700	0.096493	0.0481	0.0100788333
ss61510865	*rs41607785*	8	21.382811	0.4427	0.0276	0.868004	0.0031	0.0015290415
	rs29022036	8	21.461838	0.0752	0.1017	0.749872	0.0115	0.0015983391
ss64744909	*rs42839645*	8	21.532296	0.1913	0.8655	0.352517	0.0220	0.0067962677
ss64744925	*rs42839661*	8	21.553517	0.9391	0.0553	0.814208	0.0086	0.0009843815
ss117975327		8	21.668944	0.8746	2.0769	0.149994	0.0409	0.0089728197
ss64705363	*rs42801482*	8	21.711	0.4140	0.7178	0.397149	0.0162	0.0078826491
ss61494179	*rs41594385*	8	21.748309	0.7285	0.6774	0.410754	0.0172	0.0068106484
ss61525878	*rs41623179*	8	21.806531	0.5064	0.4502	0.502467	0.0124	0.0061815433
ss61521577	*rs41620480*	8	21.859672	0.2858	0.7743	0.379203	0.0184	0.0075129730
ss64768773	*rs42866310*	8	21.896858	0.3188	0.8117	0.367936	0.0178	0.0077335230
ss86324702		8	21.927541	0.5781	1.3803	0.240444	0.0229	0.0111696214
ss63971361	*rs42407808*	8	21.950482	0.5781	0.7941	0.373177	0.0173	0.0084585938
ss63971243	*rs42407749*	8	21.977293	0.5795	0.6272	0.428672	0.0154	0.0074971264
ss63971147	*rs42414701*	8	22.033383	0.5795	0.6272	0.428672	0.0154	0.0074971264
ss86279548		8	22.060286	0.4585	0.0465	0.829306	0.0039	0.0019290981
ss86302732		8	22.104442	0.7479	1.9670	0.161213	0.0299	0.0112931605
ss61495670	*rs41592339*	8	22.229282	0.3854	0.4566	0.499421	0.0127	0.0060389526
ss86316495		8	22.249546	0.1633	0.8742	0.350110	0.0243	0.0066401484
ss64259534	*rs42557250*	8	22.368887	0.2256	1.0932	0.296135	0.0226	0.0079103982
ss61506795	*rs41608289*	8	22.605375	0.3281	0.0346	0.852487	0.0036	0.0015868726
ss86329124		8	22.688346	0.6662	0.2900	0.590361	0.0108	0.0048090771
ss64811408	*rs42908969*	8	22.780541	0.5150	0.0909	0.763103	0.0057	0.0028592142
ss86315034		8	22.80681	0.1884	0.0020	0.963966	0.0011	0.0003214142
ss63722222	*rs42323794*	8	22.833262	0.0860	0.1134	0.736357	0.0109	0.0017124828
ss86326542		8	22.856145	0.0723	0.4295	0.512453	0.0229	0.0030796788

ss61513545	*rs41613013*	8	22.890176	0.1798	0.0348	0.852110	0.0045	0.0013316256
ss86323242		8	22.940837	0.2894	0.1055	0.745437	0.0066	0.0027091379
ss61513541	*rs41613012*	8	22.996779	0.5451	0.0632	0.801643	0.0047	0.0023117988
ss61559804	*rs41658821*	8	23.044469	0.6311	1.5215	0.217802	0.0247	0.0115054991
ss61570643	*rs41670197*	8	23.073711	0.1390	2.2459	0.134422	0.0411	0.0098255130
ss86286554		8	23.097126	0.6554	0.4219	0.516218	0.0125	0.0056538550
ss61492516	*rs41588268*	8	23.174424	0.1397	1.9979	0.157966	0.0387	0.0092955861
ss65461719	*rs43543044*	8	23.245647	0.8352	0.1915	0.661845	0.0110	0.0030222405
ss86337734		8	23.272597	0.3496	0.1098	0.740456	0.0067	0.0030676289
ss86325133		8	23.299533	0.5337	5.5194	0.019085	0.0433	0.0215488864
ss61488205	*rs41588359*	8	23.341938	0.4971	4.2376	0.039909	0.0378	0.0189146970
ss63216100	*rs42154453*	8	23.391058	0.4936	1.4853	0.223359	0.0224	0.0111743263
ss61517055	*rs41611360*	8	23.464376	0.5036	1.6504	0.199330	0.0249	0.0124574752
ss61472476	*rs41570910*	8	23.551032	0.4728	3.2176	0.073284	0.0345	0.0171916535
ss64912957	*rs43004871*	8	23.91459	0.6819	2.6621	0.103217	0.0336	0.0145884241
ss64807680	*rs42900042*	8	24.238258	0.4835	0.2710	0.602829	0.0099	0.0049577526
ss86288806		8	24.397208	0.4319	1.6541	0.198833	0.0242	0.0118809217
ss86317401		8	24.428897	0.4563	2.0244	0.155240	0.0256	0.0126850015
ss61570081	*rs41664847*	8	24.451701	0.3173	1.2378	0.266279	0.0217	0.0093983664
ss86339662		8	24.493891	0.3181	1.1726	0.279245	0.0211	0.0091343508
ss61506403	*rs41605415*	8	24.743333	0.8918	2.0747	0.150207	0.0424	0.0081721019
ss61498367	*rs43711268*	8	24.77928	0.3202	1.1504	0.283837	0.0208	0.0090613447
ss86338917		8	24.926244	0.4484	4.8835	0.027440	0.0409	0.0202153137
ss62870388	*rs42029743*	8	24.965863	0.4413	2.2232	0.136402	0.0274	0.0135022399
ss86295822		8	25.007323	0.8904	0.5380	0.463518	0.0214	0.0041722324
ss61546115	*rs41640762*	8	25.067642	0.4420	1.3679	0.242569	0.0216	0.0106788081
ss62869842	*rs42027461*	8	25.102359	0.1383	5.5467	0.018792	0.0625	0.0149029864
ss62869713	*rs42026418*	8	25.147659	0.5681	2.7693	0.096541	0.0311	0.0152747216
ss61485981	*rs41587179*	8	25.179998	0.5681	2.7693	0.096541	0.0311	0.0152747216
	rs29019642	8	25.203175	0.7822	0.1197	0.729451	0.0079	0.0026784505
ss86337539		8	25.233351	0.1476	4.3198	0.038038	0.0542	0.0136392237
ss61473452	*rs41570670*	8	25.303233	0.1203	0.3999	0.527350	0.0179	0.0037906322
ss64539526	*rs42687502*	8	25.37133	0.1633	1.3514	0.245434	0.0292	0.0079897997
ss64539548	*rs42687513*	8	25.395611	0.2063	0.0002	0.988480	0.0003	0.0001060804
ss65098220	*rs43185342*	8	25.431784	0.1905	0.9362	0.333589	0.0227	0.0070072236
ss62502691	*rs41897801*	8	25.489497	0.4183	0.0001	0.991722	0.0002	0.0000983947
ss62502613	*rs41897775*	8	25.516628	0.6218	4.8378	0.028170	0.0419	0.0196919506
ss61482951	*rs41582530*	8	25.542841	0.4463	4.3840	0.036640	0.0396	0.0195777595
	rs29012710	8	25.568606	0.6784	4.9052	0.027099	0.0444	0.0193733009
ss86328934		8	25.598414	0.6934	1.6670	0.197085	0.0254	0.0107944400
ss61513615	*rs41613983*	8	25.628681	0.1490	1.6521	0.199106	0.0343	0.0086869929
	rs29015117	8	25.663711	0.8510	1.6521	0.199106	0.0343	0.0086869929
ss61513617	*rs41613985*	8	25.717623	0.1941	4.6348	0.031673	0.0513	0.0160484331
ss86275492		8	25.830958	0.9169	0.2262	0.634512	0.0163	0.0024840320
ss65462974	*rs43546484*	8	25.868403	0.5158	0.9692	0.325229	0.0185	0.0092294506
ss86274287		8	25.907885	0.8259	6.1564	0.013329	0.0602	0.0172954882
ss86327292		8	25.949059	0.3797	0.0745	0.784973	0.0052	0.0024567135

	rs29017715	8	26.01582	0.3374	1.6838	0.194847	0.0254	0.0113734670
ss61560914	*rs41655932*	8	26.046237	0.9219	0.0041	0.949117	0.0023	0.0003250323
ss86303162		8	26.092022	0.1275	2.0453	0.153127	0.0395	0.0087950953
ss86312316		8	26.121535	0.8324	0.4475	0.503750	0.0166	0.0046203854
ss61513591	*rs41613959*	8	26.157816	0.1691	0.2647	0.607050	0.0126	0.0035446469
ss86301528		8	26.256392	0.3725	4.9512	0.026393	0.0424	0.0197999382
ss63380495	*rs42215728*	8	26.283951	0.7385	0.6926	0.405575	0.0170	0.0065595177
ss61475311	*rs41573791*	8	26.321222	0.4183	3.0846	0.079477	0.0334	0.0162764411
ss86307106		8	26.365757	0.7120	2.9713	0.085196	0.0365	0.0149703154
ss61475309	*rs41573789*	8	26.395198	0.8660	1.3316	0.248913	0.0321	0.0074590526
ss63379927	*rs42213444*	8	26.421038	0.1340	1.1113	0.292167	0.0294	0.0068144372
ss61522883	*rs41622752*	8	26.463347	0.8675	0.8134	0.367429	0.0250	0.0057574865
ss63379839	*rs42212700*	8	26.518176	0.8696	1.5167	0.218528	0.0346	0.0078419229
ss86323061		8	26.54106	0.1304	1.5167	0.218528	0.0346	0.0078419229
ss86341372		8	26.599562	0.2880	0.2465	0.619728	0.0101	0.0041558162
ss86291681		8	26.636536	0.8231	1.8461	0.174672	0.0335	0.0097656392
ss86303377		8	26.727546	0.2328	4.7683	0.029322	0.0484	0.0172816437
ss86309093		8	26.841513	0.8116	1.0537	0.305007	0.0242	0.0073968058
ss86338076		8	26.887277	0.8897	6.2708	0.012502	0.0720	0.0141254579
ss86302507		8	26.90894	0.1261	1.0522	0.305347	0.0293	0.0064564708
ss86296058		8	26.936152	0.4713	6.7316	0.009672	0.0491	0.0244773494
ss86290716		8	26.978997	0.2006	0.6630	0.415781	0.0186	0.0059786113
ss86337197		8	27.00109	0.4642	5.1278	0.023852	0.0441	0.0219145715
ss63379051	*rs42208507*	8	27.069305	0.5587	3.2623	0.071321	0.0329	0.0162215573
ss86336497		8	27.205336	0.1669	0.0725	0.787816	0.0067	0.0018653525
ss63378531	*rs42212953*	8	27.286116	0.0845	0.0536	0.817041	0.0074	0.0011505427
	rs29010933	8	27.314754	0.7027	5.7791	0.016478	0.0485	0.0202466206
ss63378353	*rs42211964*	8	27.363523	0.2471	2.5114	0.113479	0.0335	0.0124707168
ss61570154	*rs41664919*	8	27.39162	0.3116	3.6294	0.057179	0.0391	0.0167714708
ss61531054	*rs41631117*	8	27.419426	0.3840	11.4602	0.000751	0.0645	0.0305267665
ss86282214		8	27.49524	0.6920	8.0413	0.004705	0.0572	0.0243687604
ss86280208		8	27.534559	0.4047	1.2507	0.263796	0.0218	0.0105256954
ss86335674		8	27.615848	0.4534	0.4818	0.487852	0.0130	0.0064211582
ss61473669	*rs41573085*	8	27.651742	0.2564	3.6607	0.056121	0.0417	0.0158903193
ss86303967		8	27.676725	0.1619	0.2670	0.605532	0.0134	0.0036379269
ss86304303		8	27.772307	0.4499	0.6022	0.437999	0.0151	0.0074854315
ss63543030	*rs42267378*	8	27.816984	0.4921	0.4393	0.507678	0.0128	0.0064199635
ss61532288	*rs41634296*	8	27.851465	0.4205	0.0170	0.896421	0.0024	0.0011822624
ss61479285	*rs41577155*	8	27.935351	0.7908	5.5506	0.018750	0.0541	0.0178865287
ss63543375	*rs42268293*	8	27.962282	0.2923	0.0647	0.799305	0.0053	0.0021733114
ss86298929		8	27.986395	0.2937	0.0482	0.826212	0.0045	0.0018838504
ss86305374		8	28.027736	0.9019	1.6614	0.197839	0.0398	0.0070415065
ss86274668		8	28.090392	0.2915	0.1136	0.736143	0.0070	0.0028750648
ss61532292		8	28.154994	0.1139	6.9227	0.008699	0.0777	0.0156904248
ss86299052		8	28.184892	0.7321	0.1812	0.670466	0.0091	0.0035691986
ss63544401	*rs42263436*	8	28.222661	0.7507	0.7020	0.402398	0.0185	0.0069076666
ss63544440	*rs42263449*	8	28.24903	0.5616	1.4041	0.236444	0.0220	0.0108206616

	rs29020992	8	28.279427	0.2493	0.7020	0.402398		0.0185	0.0069076666
ss63544518	*rs42263474*	8	28.315959	0.2486	0.7603	0.383536		0.0193	0.0072036060
ss61532294	*rs41634302*	8	28.49287	0.2994	11.2556	0.000837		0.0704	0.0295426731
ss86338234		8	28.515183	0.6927	10.5445	0.001221		0.0672	0.0286173993
ss63545166	*rs42264490*	8	28.551907	0.4047	0.1359	0.712465		0.0073	0.0035234987
ss64978519	*rs43070389*	8	28.594157	0.5809	0.0053	0.941756		0.0014	0.0006665925
ss61498644	*rs43706049*	8	28.632446	0.2744	2.6531	0.103798		0.0326	0.0129831287
ss64702057	*rs42794193*	8	28.67085	0.3080	0.2900	0.590387		0.0106	0.0045308936
ss61559815	*rs41659532*	8	28.721492	0.7650	2.7950	0.095009		0.0368	0.0132468760
ss65093195	*rs43179389*	8	28.796838	0.7887	0.0035	0.952680		0.0014	0.0004528896
ss117964022		8	28.847073	0.7772	4.7961	0.028855		0.0486	0.0168398382
ss86298227		8	28.867114	0.2228	4.7961	0.028855		0.0486	0.0168398382
ss61566649	*rs41666575*	8	28.983594	0.2400	0.4041	0.525163		0.0141	0.0051502929
ss86274185		8	29.013204	0.3181	0.0020	0.964174		0.0009	0.0003822272
ss63940245	*rs42394902*	8	29.041969	0.7987	1.5108	0.219431		0.0285	0.0091670388
ss117969480		8	29.111473	0.1999	0.4462	0.504383		0.0157	0.0050082889
ss63940823	*rs42397090*	8	29.235117	0.7400	1.8218	0.177534		0.0291	0.0111987961
ss61475718	*rs41576689*	8	29.274043	0.2321	5.8346	0.015970		0.0540	0.0192503224
ss63940883	*rs42397120*	8	29.308131	0.6662	0.1985	0.656095		0.0088	0.0039084397
ss86324132		8	29.388741	0.4799	4.8305	0.028289		0.0413	0.0206383118
ss63941185	*rs42394472*	8	29.427259	0.7436	0.9589	0.327792		0.0214	0.0081639002
ss86330654		8	29.466105	0.3266	5.9521	0.014948		0.0492	0.0216539811
ss64471128	*rs42653609*	8	29.507939	0.1948	0.1966	0.657626		0.0104	0.0032613680
ss86335318		8	29.550271	0.6067	4.9530	0.026365		0.0428	0.0204377823
ss63717426	*rs42325801*	8	29.579448	0.4670	11.2208	0.000852		0.0616	0.0306777958
ss61513366	*rs41612048*	8	29.647279	0.5365	4.0624	0.044231		0.0372	0.0185116791
ss86280243		8	29.728918	0.6533	2.8825	0.089993		0.0333	0.0150642204
ss86293407		8	29.804956	0.0394	3.3782	0.066489		0.0851	0.0064411133
ss63718356	*rs42323514*	8	29.835847	0.0895	2.0387	0.153793		0.0475	0.0077429887
ss86327154		8	29.863476	0.4269	0.0213	0.883982		0.0028	0.0013779061
ss64818923	*rs42911180*	8	29.94953	0.2006	4.6198	0.031949		0.0506	0.0162230150
ss86286545		8	29.983955	0.1168	0.9100	0.340447		0.0275	0.0056681624
ss86311473		8	30.008315	0.1168	0.9100	0.340447		0.0275	0.0056681624
ss86313461		8	30.071775	0.1160	0.3892	0.532930		0.0179	0.0036698942
ss86315317		8	30.096742	0.6461	6.1659	0.013258		0.0474	0.0216793587
ss63719148	*rs42326576*	8	30.123501	0.1139	2.9110	0.088422		0.0488	0.0098418702
ss86341774		8	30.151764	0.5523	4.5962	0.032388		0.0396	0.0195996937
ss86338987		8	30.247816	0.9004	0.9534	0.329185		0.0313	0.0056092014
ss65463324	*rs43540834*	8	30.2762	0.8990	0.9929	0.319387		0.0318	0.0057674015
ss65463306	*rs43539716*	8	30.306351	0.0960	1.9047	0.168002		0.0443	0.0076928354
	rs29025915	8	30.415638	0.8159	0.4538	0.500743		0.0163	0.0049004215
	rs29024524	8	30.486703	0.6089	0.1815	0.670229		0.0082	0.0039186755
ss61559829	*rs41659546*	8	30.561589	0.2880	3.9982	0.045937		0.0414	0.0169739697
ss64996655	*rs43088891*	8	30.633316	0.9355	3.3184	0.068937		0.0688	0.0082951355
ss61479332	*rs41577201*	8	30.71181	0.2665	5.6633	0.017591		0.0508	0.0198570081
ss65463151	*rs43547661*	8	30.74897	0.1483	0.0129	0.909726		0.0029	0.0007412040
ss61492528	*rs41588280*	8	30.79503	0.5143	2.3934	0.122300		0.0289	0.0144289169

ss86311540		8	30.847968	0.8983	1.0691	0.301499	0.0313	0.0057187977
ss61559823	*rs41659540*	8	31.07301	0.0845	2.9588	0.085854	0.0590	0.0091345987
ss61559820	*rs41659537*	8	31.155828	0.5766	4.6880	0.030712	0.0402	0.0196417386
ss86308438		8	31.194737	0.8660	2.2254	0.136210	0.0412	0.0095660037
ss117969449		8	31.322024	0.8782	1.8679	0.172155	0.0373	0.0079820627
ss61491803	*rs41590974*	8	31.359291	0.3517	0.1872	0.665418	0.0082	0.0037511976
ss86303057		8	31.439256	0.4212	4.2027	0.040735	0.0397	0.0193786075
ss117964018		8	31.46516	0.3238	3.2034	0.073921	0.0355	0.0155538328
ss86302084		8	31.495259	0.4255	0.0135	0.907463	0.0022	0.0010846446
ss65409218	*rs43500197*	8	31.576976	0.1189	0.4431	0.505859	0.0187	0.0039095437
ss65409130	*rs43499009*	8	31.663726	0.3324	2.1095	0.146839	0.0287	0.0127383083
ss65409082	*rs43498962*	8	31.743352	0.4792	4.5247	0.033760	0.0393	0.0196262500
ss65409044	*rs43498124*	8	31.772089	0.5201	4.6371	0.031631	0.0397	0.0198397442
ss86336279		8	31.832255	0.5738	3.7764	0.052384	0.0363	0.0177714140
ss86315538		8	31.877139	0.4470	1.6916	0.193816	0.0243	0.0120083918
ss65408932	*rs43496812*	8	31.936109	0.5910	1.4269	0.232680	0.0227	0.0109908393
ss65408896	*rs43496776*	8	31.980304	0.5007	0.0100	0.920180	0.0019	0.0009337677
ss61491802	*rs41590973*	8	32.008645	0.2350	2.3373	0.126762	0.0327	0.0117473287
	rs29012932	8	32.030164	0.3696	2.4573	0.117434	0.0302	0.0140922906
ss65055323	*rs43143572*	8	32.063864	0.6103	2.8740	0.090468	0.0322	0.0153211177
ss65408805	*rs43495585*	8	32.112292	0.8639	0.2648	0.606996	0.0134	0.0031521176
ss65408757	*rs43495537*	8	32.150578	0.1046	3.8406	0.050425	0.0605	0.0113307251
ss86298900		8	32.188298	0.4398	0.0137	0.906999	0.0022	0.0010837303
ss65408619	*rs43493199*	8	32.226076	0.8789	0.2717	0.602361	0.0152	0.0032246320
ss86341635		8	32.30504	0.2658	0.1463	0.702235	0.0082	0.0031942887
ss86340147		8	32.338998	0.7128	0.0374	0.846700	0.0040	0.0016483371
ss64163687	*rs42507974*	8	32.414303	0.9635	0.4971	0.481033	0.0351	0.0024721582
	rs29016072	8	32.446265	0.1777	0.1598	0.689449	0.0095	0.0027817750
ss64163359	*rs42505210*	8	32.492403	0.4377	0.1693	0.680823	0.0080	0.0039536142
ss86302787		8	32.524791	0.3345	0.2230	0.636896	0.0096	0.0042931376
ss86300886		8	32.562536	0.2314	4.9172	0.026914	0.0484	0.0172060835
ss117969490		8	32.577723	0.4484	1.0974	0.295191	0.0188	0.0093045495
ss86284241		8	32.669995	0.0860	0.4525	0.501360	0.0217	0.0034135490
ss64695684	*rs42793723*	8	32.697509	0.2213	11.7763	0.000635	0.0754	0.0259916099
ss117969396		8	32.875215	0.2686	5.0267	0.025274	0.0458	0.0179826319
ss86329630		8	32.896233	0.1626	5.5598	0.018653	0.0600	0.0163463363
	rs29013453	8	33.016309	0.6476	0.4357	0.509410	0.0132	0.0060122689
ss65463389	*rs43540899*	8	33.092376	0.7106	0.0600	0.806543	0.0050	0.0020561869
ss86335996		8	33.160486	0.7486	6.3713	0.011819	0.0515	0.0193898156
ss117969272		8	33.249985	0.7500	0.0196	0.888753	0.0029	0.0010868689
ss64930957	*rs43019452*	8	33.273564	0.2242	0.3671	0.544764	0.0129	0.0044761417
ss64335710	*rs42589512*	8	33.338661	0.6354	1.3839	0.239833	0.0217	0.0100577383
ss117969385		8	33.374483	0.3911	1.7346	0.188256	0.0242	0.0115445135
ss86331370		8	33.48537	0.2593	0.0010	0.974779	0.0006	0.0002476868
ss86332074		8	33.53448	0.7808	0.0007	0.979010	0.0006	0.0001959567
ss64994653	*rs43085699*	8	33.579164	0.7951	0.5711	0.450087	0.0167	0.0054281246
ss61499505	*rs43710693*	8	33.623839	0.1404	0.2322	0.630054	0.0124	0.0029873436

ss61559892	*rs41659609*	8	33.690309	0.5351	0.1046	0.746498	0.0059	0.0029469716
ss61559893	*rs41659610*	8	33.712248	0.2049	2.4374	0.118929	0.0358	0.0116694312
ss61475157	*rs41572645*	8	33.764152	0.2407	5.7005	0.017226	0.0499	0.0182231090
ss61466294	*rs41568049*	8	33.807898	0.5401	0.0167	0.897199	0.0024	0.0011840226
	rs29027535	8	33.851945	0.4133	0.0741	0.785607	0.0050	0.0024262245
ss65465336	*rs43546900*	8	33.881267	0.0996	4.8243	0.028390	0.0678	0.0121582956
ss86326776		8	33.92329	0.6698	0.0319	0.858216	0.0034	0.0015104319
ss86276391		8	33.954401	0.5695	3.3485	0.067696	0.0339	0.0166022738
ss61472009	*rs41566251*	8	33.977106	0.9004	1.8721	0.171674	0.0410	0.0073489618
ss61535650	*rs41628583*	8	34.049606	0.2350	0.0343	0.853055	0.0041	0.0014682985
	rs29018412	8	34.086109	0.2378	0.2578	0.611777	0.0110	0.0039775957
ss62100490	*rs41754995*	8	34.132138	0.5509	2.9991	0.083752	0.0321	0.0158660661
ss65381573	*rs43465685*	8	34.216239	0.5702	1.4266	0.232734	0.0225	0.0110220035
	rs29025610	8	34.280295	0.7579	0.1277	0.720979	0.0076	0.0027791513
ss65381714	*rs43466516*	8	34.38381	0.5967	0.0754	0.783686	0.0051	0.0024593054
ss86322626		8	34.519839	0.6819	0.0012	0.971894	0.0007	0.0002999346
ss61469138	*rs41567749*	8	34.6648	0.6153	0.0043	0.947834	0.0012	0.0005822045
ss86321894		8	34.694187	0.8159	1.5775	0.209541	0.0290	0.0087133111
ss64727291	*rs42819792*	8	34.786444	0.8223	0.5308	0.466509	0.0176	0.0051341684
ss64727322	*rs42819823*	8	34.830186	0.7708	1.0128	0.314572	0.0221	0.0078156125
ss61566620	*rs41666548*	8	34.883193	0.5437	0.0973	0.755219	0.0058	0.0028988532
ss61569986	*rs41672035*	8	34.946122	0.3503	2.2742	0.132000	0.0290	0.0132211220
ss61469829	*rs41572823*	8	35.060954	0.0989	0.3274	0.567404	0.0181	0.0032190062
ss86327725		8	35.129967	0.2264	0.0565	0.812112	0.0054	0.0018760964
ss86340512		8	35.264697	0.7966	2.4639	0.116947	0.0364	0.0117911889
ss86321711		8	35.464028	0.4764	1.0532	0.305131	0.0189	0.0094079047
ss63684910	*rs42311470*	8	35.493324	0.6168	0.2596	0.610565	0.0098	0.0046194462
ss61499512	*rs43710700*	8	35.520244	0.7249	3.7665	0.052693	0.0388	0.0154858599
ss61516197	*rs41612416*	8	35.542959	0.7636	0.1138	0.735979	0.0073	0.0026509087
ss64636588	*rs42730076*	8	35.566523	0.4792	0.0835	0.772631	0.0054	0.0027071216
ss61531806	*rs41630354*	8	35.60022	0.4577	0.8703	0.351195	0.0171	0.0084643029
ss64062439	*rs42454966*	8	35.756715	0.8617	0.0270	0.869626	0.0044	0.0010529579
ss64062423	*rs42454958*	8	35.784603	0.5043	1.5860	0.208316	0.0231	0.0115300886
ss64062327	*rs42454311*	8	35.876082	0.6483	3.5479	0.060039	0.0358	0.0163293673
ss64989539	*rs43080595*	8	35.95861	0.9291	1.7839	0.182103	0.0482	0.0063549936
ss65378603	*rs43469342*	8	36.051155	0.6812	2.1079	0.146995	0.0291	0.0126581546
ss86328658		8	36.089341	0.5681	0.0510	0.821457	0.0043	0.0021153700
ss63768753	*rs42342256*	8	36.171902	0.6268	0.0515	0.820613	0.0043	0.0020273753
ss117969368		8	36.232703	0.4556	0.9361	0.333608	0.0179	0.0088848492
	rs29014632	8	36.274939	0.5544	0.9462	0.331017	0.0181	0.0089212994
ss61515412	*rs41614268*	8	36.369229	0.3940	0.0669	0.795972	0.0049	0.0023371628
ss86327156		8	36.394248	0.3367	1.0781	0.299484	0.0206	0.0092173351
ss64945710	*rs43038099*	8	36.41892	0.4542	2.6902	0.101421	0.0298	0.0147601178
ss64383133	*rs42609327*	8	36.468395	0.4928	6.1898	0.013082	0.0459	0.0229287899
ss61515414	*rs41614270*	8	36.494112	0.0301	2.8108	0.094079	0.0911	0.0053167253
ss63768985	*rs42342871*	8	36.534997	0.2887	5.6699	0.017526	0.0487	0.0199965483
ss64383025	*rs42609273*	8	36.570294	0.4993	5.4240	0.020147	0.0429	0.0214631327

ss64110279	*rs42478148*	8	36.683361	0.1877	1.5896	0.207805	0.0294	0.0089524237
ss64110327	*rs42478172*	8	36.708699	0.5609	0.4850	0.486399	0.0128	0.0063024242
ss64382617	*rs42607469*	8	36.733374	0.4169	2.5484	0.110858	0.0296	0.0144001119
ss64950731	*rs43041882*	8	36.75624	0.4176	2.6716	0.102607	0.0304	0.0147641593
	rs29021644	8	36.779293	0.3968	2.2975	0.130033	0.0276	0.0132033944
ss64110517	*rs42478767*	8	36.805886	0.2393	0.0209	0.885000	0.0030	0.0010805679
ss61531187	*rs41633147*	8	36.919474	0.6211	0.2181	0.640610	0.0088	0.0041383582
ss117969158		8	36.988726	0.1963	0.2951	0.587119	0.0124	0.0039213074
ss86283001		8	37.070048	0.6691	0.2728	0.601638	0.0099	0.0043678803
ss61520417	*rs41614542*	8	37.12669	0.2163	0.0003	0.985306	0.0004	0.0001312232
ss86337730		8	37.163738	0.2142	0.0005	0.981858	0.0005	0.0001615381
ss61520421	*rs41614546*	8	37.197678	0.0974	0.1352	0.713242	0.0116	0.0020345810
ss86284174		8	37.229388	0.4112	0.6578	0.417630	0.0152	0.0073374717
ss65070757	*rs43161947*	8	37.257076	0.6511	0.0628	0.802203	0.0047	0.0021301654
ss61474125	*rs41567337*	8	37.359317	0.7744	0.2944	0.587619	0.0112	0.0039271181
ss65071335	*rs43157028*	8	37.439096	0.4341	5.4215	0.020176	0.0429	0.0210546803
ss62359973	*rs41850898*	8	37.461263	0.1239	9.7980	0.001820	0.0865	0.0187908156
ss62359217	*rs41849346*	8	37.563462	0.2185	0.9620	0.327017	0.0217	0.0074197267
ss61524429	*rs41620671*	8	37.702631	0.6289	2.1833	0.139969	0.0283	0.0132023553
ss65463525	*rs43542405*	8	37.898415	0.7521	1.4061	0.236104	0.0254	0.0094620730
ss65463679	*rs43545658*	8	38.000741	0.3990	0.0380	0.845418	0.0036	0.0017378930
ss61559838	*rs41659555*	8	38.039162	0.2034	0.2944	0.587578	0.0123	0.0039896342
ss86334565		8	38.137795	0.4327	0.4354	0.509577	0.0124	0.0060813382
ss86321887		8	38.264652	0.2572	0.0995	0.752559	0.0064	0.0024628516
ss86282280		8	38.287758	0.0731	2.7923	0.095170	0.0601	0.0081370175
ss61492531	*rs41588283*	8	38.334145	0.4033	0.0101	0.919793	0.0020	0.0009400200
ss61559843	*rs41659560*	8	38.485958	0.8696	0.5568	0.455792	0.0201	0.0045631561
ss61559852	*rs41659569*	8	38.510344	0.5050	0.3472	0.555868	0.0108	0.0053985359
ss61559855	*rs41659572*	8	38.53118	0.3625	1.8277	0.176840	0.0258	0.0119041054
ss86274574		8	38.559429	0.3560	1.4217	0.233536	0.0230	0.0105311076
ss61559869	*rs41659586*	8	38.739501	0.2958	0.6475	0.421291	0.0163	0.0067975377
ss65463999	*rs43548976*	8	38.82821	0.2837	2.2150	0.137127	0.0302	0.0122620146
ss61519703	*rs41617847*	8	38.894958	0.4993	4.0701	0.044032	0.0356	0.0178240147
ss63856630	*rs42384674*	8	38.926209	0.3302	0.4542	0.500569	0.0132	0.0058251888
ss61519704	*rs41617848*	8	38.954373	0.2572	1.2542	0.263138	0.0233	0.0089037253
ss86335072		8	38.981372	0.8997	4.0257	0.045197	0.0595	0.0107374691
ss117969265		8	39.003786	0.4269	0.4881	0.484991	0.0131	0.0063927538
ss64780049	*rs42880677*	8	39.038086	0.6662	3.8175	0.051120	0.0380	0.0168997875
ss65464219	*rs43550196*	8	39.075904	0.3732	1.2780	0.258666	0.0207	0.0096918543
ss65464252	*rs43541832*	8	39.122862	0.1304	5.9650	0.014840	0.0652	0.0147770700
ss61508552	*rs41604817*	8	39.248746	0.3023	1.1915	0.275410	0.0218	0.0091860692
	rs29014429	8	39.297545	0.3718	0.4022	0.526138	0.0126	0.0058679920
ss61476040	*rs41579403*	8	39.373847	0.5466	2.1395	0.144003	0.0266	0.0131909342
ss65465753	*rs43549314*	8	39.450555	0.6519	1.5526	0.213166	0.0245	0.0111206591
ss61559906	*rs41654633*	8	39.478707	0.7564	9.1953	0.002517	0.0642	0.0236474072
ss61492563	*rs41588314*	8	39.505359	0.2421	0.0319	0.858259	0.0039	0.0014196516
ss65465568	*rs43548029*	8	39.530036	0.3001	1.3718	0.241899	0.0236	0.0099319076

ss86339110		8	39.552955	0.5616	3.0364	0.081860	0.0337	0.0166136877
	rs29022255	8	39.578151	0.2328	2.1958	0.138837	0.0333	0.0118789311
ss86288859		8	39.661514	0.0766	5.9385	0.015063	0.0867	0.0122701358
ss86288860		8	39.697877	0.3116	0.0001	0.990641	0.0002	0.0001033209
ss86317079		8	39.736516	0.5014	3.1214	0.077708	0.0336	0.0168034329
ss86298503		8	39.820921	0.5165	7.6429	0.005850	0.0504	0.0251862857
ss65465915	*rs43551076*	8	40.04296	0.6304	2.7762	0.096125	0.0317	0.0147877367
ss61492571	*rs41588322*	8	40.083297	0.5444	0.2570	0.612357	0.0096	0.0047549169
ss86293453		8	40.298699	0.0946	0.8602	0.353995	0.0300	0.0051346409
ss61559919	*rs41654646*	8	40.380204	0.4448	2.6796	0.102095	0.0310	0.0153232985
ss117969452		8	40.453706	0.6297	0.0012	0.972223	0.0007	0.0003214948
ss117969312		8	40.584774	0.6619	0.2210	0.638414	0.0094	0.0042247736
ss61523966	*rs41624318*	8	40.845203	0.2643	0.2642	0.607444	0.0111	0.0043074539
ss61568381	*rs41663679*	8	40.896552	0.8725	1.1841	0.276902	0.0290	0.0064577144
	rs29011524	8	40.949566	0.2672	0.2079	0.648592	0.0097	0.0038053088
ss61496723	*rs41592075*	8	40.991156	0.2658	0.1952	0.658743	0.0095	0.0037022233
	rs29026051	8	41.027508	0.5716	1.6815	0.195149	0.0244	0.0119724966
ss61567377	*rs41662592*	8	41.059185	0.7163	0.0771	0.781299	0.0058	0.0023619832
ss61567375	*rs41662590*	8	41.082667	0.6948	0.1147	0.734981	0.0069	0.0029379266
ss86316770		8	41.18656	0.4126	2.8038	0.094493	0.0315	0.0152563358
ss61567373	*rs41662588*	8	41.226728	0.4155	3.6732	0.055705	0.0360	0.0175010117
ss86303601		8	41.3927	0.2342	0.5484	0.459222	0.0164	0.0058829155
ss86303799		8	41.457555	0.0824	0.7275	0.393993	0.0285	0.0043052855
ss61496187	*rs41597440*	8	41.583935	0.8990	0.8441	0.358543	0.0279	0.0050695245
ss61496190	*rs41597443*	8	41.664452	0.3417	0.1366	0.711749	0.0072	0.0032530077
	rs29016846	8	41.794679	0.6698	0.0164	0.898087	0.0025	0.0011212793
ss61497916	*rs43705730*	8	41.831509	0.2385	3.1862	0.074696	0.0395	0.0143567911
ss61559910	*rs41654637*	8	41.868874	0.4642	0.6249	0.429499	0.0150	0.0074664193
ss61559914	*rs41654641*	8	41.890014	0.8632	3.1875	0.074640	0.0467	0.0110238000
ss86316254		8	41.925465	0.7901	3.8898	0.048975	0.0438	0.0145356722
ss86284668		8	41.954333	0.1791	1.0140	0.314306	0.0244	0.0071683222
ss86276101		8	41.978519	0.6218	1.6997	0.192756	0.0249	0.0117219208
ss61492570	*rs41588321*	8	42.043084	0.3274	0.1087	0.741678	0.0065	0.0028455843
ss86306269		8	42.113456	0.3460	0.0430	0.835870	0.0041	0.0018396677
ss86323302		8	42.18263	0.3976	0.3295	0.566133	0.0111	0.0053326733
	rs29019858	8	42.236487	0.6032	0.1099	0.740313	0.0062	0.0029604236
ss86294509		8	42.261061	0.6447	0.3097	0.578038	0.0110	0.0050551252
ss117969292		8	42.343641	0.1483	0.6610	0.416476	0.0215	0.0054253899
ss86337130		8	42.37644	0.3073	0.0511	0.821154	0.0045	0.0019042329
ss117969461		8	42.424473	0.7593	0.2061	0.650013	0.0100	0.0036545292
	rs29026130	8	42.492164	0.8109	2.3461	0.126047	0.0370	0.0113373276
ss117969399		8	42.539116	0.8044	2.2428	0.134688	0.0356	0.0112011046
ss117969402		8	42.561621	0.2915	1.9707	0.160816	0.0286	0.0118337771
ss86321500		8	42.694558	0.0888	0.5501	0.458523	0.0237	0.0038323710
ss86334227		8	42.756802	0.5788	0.1197	0.729475	0.0064	0.0031441638
ss86307560		8	42.784577	0.3367	0.0325	0.856974	0.0035	0.0015412006
ss61559951	*rs41654678*	8	42.809561	0.0659	0.8589	0.354375	0.0348	0.0042831327

ss64687266	*rs42786735*	8	42.929959	0.0817	0.3898	0.532589	0.0207	0.0031069890
ss61559950	*rs41654677*	8	42.973043	0.6232	0.9644	0.326426	0.0187	0.0088023024
ss65468612	*rs43549667*	8	42.997646	0.8510	0.7423	0.389235	0.0225	0.0057038608
ss65466122	*rs43552383*	8	43.262834	0.7235	3.2548	0.071648	0.0369	0.0147594449
ss65466255	*rs43553416*	8	43.320534	0.9592	10.5085	0.001245	0.1497	0.0117250683
ss64131987	*rs42495378*	8	43.380159	0.0881	0.1799	0.671632	0.0137	0.0022020203
ss64131967	*rs42495368*	8	43.477293	0.0852	0.0218	0.882570	0.0048	0.0007533499
ss64131899	*rs42495334*	8	43.513036	0.3510	1.3319	0.248862	0.0217	0.0098741991
ss61526393	*rs41623088*	8	43.608098	0.3059	0.1314	0.717098	0.0071	0.0030116080
ss86278754		8	43.646372	0.8524	0.5644	0.452727	0.0195	0.0049156190
ss61492543	*rs41588294*	8	43.709405	0.1275	0.4585	0.498542	0.0185	0.0041169106
ss61476812	*rs41579263*	8	43.752639	0.5530	2.2468	0.134346	0.0281	0.0138850069
ss86288333		8	43.787242	0.4477	2.1026	0.147495	0.0270	0.0133740312
ss61559881	*rs41659598*	8	43.885136	0.2507	2.5933	0.107773	0.0347	0.0130511673
ss86303257		8	43.932302	0.8059	0.0100	0.920318	0.0023	0.0007173712
ss86339572		8	43.960706	0.8589	1.0850	0.297940	0.0278	0.0067503672
ss86304955		8	44.020087	0.4850	0.8517	0.356385	0.0170	0.0085159040
	rs29021338	8	44.047256	0.1490	1.0108	0.315061	0.0252	0.0064029320
ss65464538	*rs43543016*	8	44.074919	0.6920	1.3382	0.247755	0.0230	0.0097935521
ss86290862		8	44.124019	0.1748	2.2876	0.130866	0.0381	0.0109833958
ss86337289		8	44.243868	0.2221	5.6231	0.017997	0.0519	0.0179228212
ss117969335		8	44.315465	0.9105	0.0990	0.753096	0.0099	0.0016079290
ss64561518	*rs42698410*	8	44.346149	0.0766	6.5741	0.010556	0.0894	0.0126582051
ss61514847	*rs41616402*	8	44.376597	0.0688	3.8057	0.051478	0.0711	0.0091084660
ss61514855	*rs41616410*	8	44.401608	0.5723	2.6920	0.101306	0.0303	0.0148541058
ss64561746	*rs42699424*	8	44.426621	0.7192	0.0595	0.807349	0.0049	0.0019867484
ss63891422	*rs41859505*	8	44.459315	0.0580	1.8056	0.179480	0.0538	0.0058778351
ss86323127		8	44.502308	0.3016	3.8608	0.049823	0.0397	0.0167384843
ss86310767		8	44.658986	0.2285	4.7524	0.029592	0.0480	0.0169152247
ss86321542		8	44.693064	0.6862	2.2251	0.136236	0.0290	0.0125010999
ss63891586	*rs41860286*	8	44.737849	0.2450	0.2881	0.591604	0.0118	0.0043685404
ss63891778	*rs41860482*	8	44.798005	0.0709	0.0281	0.866830	0.0059	0.0007799927
ss64855589	*rs42949046*	8	44.951017	0.2479	0.5486	0.459127	0.0158	0.0058751217
ss64738809	*rs42835641*	8	44.990441	0.0738	3.9910	0.046132	0.0707	0.0096608524
ss86315787		8	45.029704	0.2428	0.5342	0.465097	0.0165	0.0060823444
ss86285544		8	45.210364	0.1182	2.3159	0.128508	0.0436	0.0090919157
ss63901824	*rs42020892*	8	45.244158	0.9749	0.7672	0.381391	0.0518	0.0025343289
ss86316707		8	45.275579	0.5129	1.8717	0.171726	0.0247	0.0123209856
ss117969455		8	45.656882	0.5695	0.0000	0.998103	0.0000	0.0000219208
ss61512297	*rs41610905*	8	45.693223	0.7443	1.1593	0.281987	0.0230	0.0087687462
ss63687810	*rs42312419*	8	45.767135	0.3188	0.5722	0.449623	0.0150	0.0065218595
ss86290960		8	45.889663	0.9635	3.5251	0.060866	0.0933	0.0065691346
ss65469241	*rs43553193*	8	46.14694	0.5781	3.6102	0.057838	0.0354	0.0172828618
ss86340499		8	46.26685	0.4864	5.1240	0.023905	0.0429	0.0214384068
ss86328295		8	46.304264	0.5129	4.9393	0.026573	0.0421	0.0210192684
ss117969405		8	46.360788	0.2701	2.3426	0.126332	0.0321	0.0126455767
	rs29024181	8	46.412911	0.2658	2.3690	0.124218	0.0330	0.0128656365

ss65468862	*rs43550716*	8	46.574051	0.7708	0.8281	0.363127	0.0204	0.0071986758
ss86310155		8	46.609062	0.4463	3.7739	0.052461	0.0362	0.0178886813
ss86321035		8	46.645136	0.8840	1.5966	0.206806	0.0360	0.0073907747
ss65468831	*rs43550686*	8	46.668308	0.8911	1.4448	0.229774	0.0354	0.0068694184
ss65468802	*rs43550657*	8	46.698939	0.9506	1.5959	0.206914	0.0533	0.0050090581
ss86326492		8	46.744994	0.2944	0.4273	0.513542	0.0140	0.0058120684
ss46526078	*rs41255225*	8	46.785407	0.6483	1.8185	0.177930	0.0262	0.0119253028
	rs29019622	8	46.807444	0.4090	0.5366	0.464082	0.0137	0.0066221214
ss65468026	*rs43553681*	8	47.049494	0.5179	5.7452	0.016796	0.0457	0.0227984478
ss86311531		8	47.075477	0.2693	2.4486	0.118088	0.0325	0.0128084474
ss61492584	*rs41588835*	8	47.097548	0.2815	0.0046	0.946007	0.0014	0.0005746499
ss86322423		8	47.132216	0.0681	2.9376	0.086984	0.0613	0.0077761862
ss86307995		8	47.162095	0.7149	0.4448	0.505045	0.0143	0.0058126327
ss65468204	*rs43555559*	8	47.193589	0.3675	2.7570	0.097283	0.0334	0.0155336009
ss86294526		8	47.230313	0.0946	0.7782	0.377983	0.0278	0.0047591134
	rs29026689	8	47.260881	0.4585	0.3331	0.564004	0.0111	0.0055331726
ss65468291	*rs43548046*	8	47.284711	0.2507	3.5316	0.060626	0.0403	0.0151416511
ss65468302	*rs43548057*	8	47.339034	0.0931	0.0001	0.994221	0.0002	0.0000385076
ss65468370	*rs43548125*	8	47.364552	0.4835	1.2409	0.265687	0.0210	0.0104756941
ss86278759		8	47.389671	0.0681	4.7386	0.029828	0.0814	0.0103261004
ss86336477		8	47.540425	0.1956	1.0057	0.316280	0.0239	0.0075271341
ss65466568	*rs43548927*	8	47.582318	0.1196	1.1595	0.281944	0.0310	0.0065295632
ss86340195		8	47.614756	0.6039	3.1684	0.075513	0.0341	0.0162960661
ss86310932		8	47.660962	0.2264	0.2011	0.653988	0.0097	0.0033908647
ss61559932	*rs41654659*	8	47.691608	0.2636	4.5714	0.032857	0.0451	0.0175072058
ss86331637		8	47.728533	0.1160	0.6639	0.415448	0.0230	0.0047250264
ss61559930	*rs41654657*	8	47.75237	0.5201	2.2867	0.130941	0.0280	0.0139957371
ss86335601		8	47.774025	0.3625	0.4620	0.496922	0.0126	0.0058390964
ss117963993		8	47.837608	0.5401	2.8966	0.089214	0.0315	0.0156573135
ss64697503	*rs42797230*	8	47.86207	0.6010	3.6599	0.056148	0.0359	0.0172307291
ss86297639		8	47.895369	0.3252	2.8184	0.093636	0.0335	0.0147021257
ss61531391	*rs41626053*	8	47.958164	0.8152	4.9172	0.026913	0.0549	0.0165383994
ss86341491		8	47.990263	0.6655	0.1780	0.673249	0.0084	0.0037398372
ss86305130		8	48.053636	0.5014	0.0802	0.777139	0.0053	0.0026636414
ss61531383	*rs41626045*	8	48.073842	0.3804	0.0541	0.816138	0.0044	0.0020793840
ss86312614		8	48.109951	0.8295	2.3958	0.122116	0.0384	0.0108539926
ss64470366	*rs42657226*	8	48.305565	0.1010	0.0213	0.883982	0.0044	0.0007985733
ss64470222	*rs42657154*	8	48.401809	0.4062	0.0130	0.909333	0.0021	0.0010354614
ss86332207		8	48.680421	0.7915	5.4937	0.019365	0.0546	0.0180052500
ss117974997		8	48.778664	0.4842	2.2555	0.133593	0.0282	0.0140633591
ss117966029		8	48.816008	0.8954	0.9110	0.340191	0.0292	0.0054601155
ss63596781	*rs42280921*	8	48.844403	0.9140	0.3270	0.567586	0.0187	0.0029308291
ss86339813		8	48.882283	0.3489	0.2963	0.586393	0.0109	0.0049298690
ss65503200	*rs43584893*	8	49.00401	0.8395	0.1944	0.659378	0.0110	0.0029721661
ss86338827		8	49.066666	0.4126	1.7404	0.187528	0.0254	0.0123196655
ss61504813	*rs41599688*	8	49.117484	0.8173	0.2876	0.591962	0.0129	0.0038416000
ss86336774		8	49.203189	0.5838	4.5949	0.032414	0.0420	0.0204113768

ss86339499		8	49.286873	0.8646	0.6214	0.430781	0.0212	0.0049638292
ss63597885	*rs42284584*	8	49.356525	0.4706	0.7549	0.385226	0.0163	0.0081387299
ss64229160	*rs42538990*	8	49.386282	0.7994	0.2238	0.636325	0.0115	0.0036749958
ss61469725	*rs41572222*	8	49.432712	0.1168	0.2401	0.624309	0.0139	0.0028698319
ss86328966		8	49.457424	0.8832	0.2401	0.624309	0.0139	0.0028698319
ss61565188	*rs41659045*	8	49.52659	0.7787	4.4574	0.035107	0.0476	0.0164215925
ss86340221		8	49.560091	0.1948	1.1885	0.276016	0.0257	0.0080698851
ss86284334		8	49.580322	0.1203	3.9198	0.048115	0.0560	0.0118545425
ss86306291		8	49.618713	0.6146	0.4784	0.489389	0.0137	0.0065126886
ss86294171		8	49.653651	0.4563	2.1983	0.138614	0.0287	0.0142561407
ss65021906	*rs43109452*	8	49.722023	0.9298	2.4367	0.118982	0.0570	0.0074378351
ss61492595	*rs41588846*	8	49.750487	0.1318	0.4196	0.517332	0.0177	0.0040447378
ss65036948	*rs43127117*	8	49.775559	0.5501	0.7746	0.379114	0.0163	0.0080909724
ss61490607	*rs41589112*	8	49.801065	0.4491	0.8855	0.347018	0.0175	0.0086652401
ss86277015		8	49.821791	0.3933	3.9858	0.046275	0.0378	0.0180200346
ss86324701		8	49.904285	0.5609	1.5488	0.213726	0.0234	0.0115314854
ss86337853		8	49.928007	0.3223	0.2517	0.616059	0.0099	0.0043327170
ss86316998		8	50.029389	0.6476	1.6326	0.201763	0.0258	0.0117750415
ss86294018		8	50.159953	0.7271	3.9480	0.047317	0.0408	0.0161810303
ss86300939		8	50.280049	0.1576	3.5074	0.061514	0.0471	0.0125025114
ss86283867		8	50.32814	0.7966	0.8717	0.350804	0.0206	0.0066821362
ss86326324		8	50.35647	0.8374	1.3275	0.249654	0.0297	0.0080887233
ss117969476		8	50.511232	0.9090	1.7622	0.184788	0.0435	0.0072021724
ss61559972	*rs41654699*	8	50.61275	0.1590	0.0629	0.801970	0.0064	0.0017087423
ss61492592	*rs41588843*	8	50.66497	0.1927	1.8960	0.168967	0.0323	0.0100630995
ss61492593	*rs41588844*	8	50.710409	0.0917	0.0098	0.921079	0.0032	0.0005258821
ss64293408	*rs42569020*	8	50.793509	0.2170	7.4578	0.006476	0.0611	0.0207570748
ss61506906	*rs41600114*	8	50.840964	0.4885	9.7651	0.001852	0.0575	0.0287529731
ss86311732		8	50.861584	0.1540	0.7328	0.392265	0.0206	0.0053640390
ss61504751	*rs41609223*	8	50.884918	0.1540	0.5631	0.453277	0.0180	0.0047030392
ss65515534	*rs43598310*	8	50.914708	0.4370	16.0133	0.000070	0.0744	0.0366256827
ss65515513	*rs43598289*	8	50.963605	0.5895	22.8981	0.000002	0.0918	0.0444395605
ss86317861		8	51.089228	0.5007	17.5344	0.000032	0.0772	0.0386213679
ss86338022		8	51.143287	0.1862	6.4235	0.011480	0.0606	0.0183781120
ss86318208		8	51.212486	0.2629	7.3789	0.006763	0.0576	0.0223057966
ss61561328	*rs41659738*	8	51.240376	0.3617	0.0920	0.761752	0.0057	0.0026288030
ss86333358		8	51.292315	0.4384	5.5242	0.019033	0.0435	0.0214045608
ss65515404	*rs43597380*	8	51.334362	0.3009	1.5780	0.209467	0.0255	0.0107239799
ss65470206	*rs43554940*	8	51.406456	0.8610	0.2733	0.601288	0.0142	0.0034008844
ss86341539		8	51.438864	0.5029	0.3454	0.556922	0.0108	0.0053918100
ss61527833	*rs41626875*	8	51.571368	0.5494	1.3300	0.249194	0.0218	0.0108076418
ss86308709		8	51.599344	0.2221	0.9265	0.336103	0.0218	0.0075358308
ss86303837		8	51.629724	0.3639	0.5539	0.456978	0.0151	0.0070042253
ss86299424		8	51.675837	0.1719	0.5259	0.468566	0.0180	0.0051116560
	rs29023531	8	51.718478	0.8940	0.7298	0.393236	0.0252	0.0047782954
ss61559964	*rs41654691*	8	51.778384	0.6898	0.7864	0.375506	0.0178	0.0076250966
ss65469358	*rs43548314*	8	51.858422	0.9398	1.9652	0.161407	0.0552	0.0062435711

ss65469326	*rs43548282*	8	51.889977	0.3797	0.0438	0.834375	0.0041	0.0019321963
ss61492587	*rs41588838*	8	52.070787	0.6139	1.8509	0.174118	0.0255	0.0121026358
ss61525984	*rs41623982*	8	52.162972	0.2350	0.1251	0.723713	0.0080	0.0028787881
ss64700341	*rs42794425*	8	52.24013	0.9563	0.5583	0.455193	0.0343	0.0028691188
ss61517758	*rs41618121*	8	52.26671	0.1999	0.6002	0.438765	0.0179	0.0057400734
	rs29014626	8	52.301027	0.8417	3.4518	0.063605	0.0462	0.0123062554
ss64674972	*rs42770762*	8	52.330803	0.8424	3.3132	0.069155	0.0455	0.0120886324
ss61563866	*rs41665031*	8	52.35985	0.1562	3.5782	0.058956	0.0474	0.0124912478
ss64195601	*rs42520205*	8	52.429155	0.3746	0.9604	0.327417	0.0190	0.0088850580
	rs29027171	8	52.519334	0.5809	1.1938	0.274935	0.0207	0.0100974811
ss65058363	*rs43151767*	8	52.59892	0.4384	0.0027	0.958675	0.0010	0.0004859570
ss65470803	*rs43554340*	8	52.622729	0.5029	2.8194	0.093578	0.0312	0.0155871057
ss65470731	*rs43553468*	8	52.673382	0.5150	0.4297	0.512366	0.0125	0.0062672298
ss65470696	*rs43553433*	8	52.698617	0.5129	0.4920	0.483252	0.0134	0.0067172079
ss86340669		8	52.742241	0.7479	0.0003	0.987128	0.0004	0.0001353661
ss65470506	*rs43551343*	8	52.772691	0.1927	0.4232	0.515542	0.0157	0.0048871838
ss86288301		8	52.808074	0.3123	0.7876	0.375137	0.0185	0.0079311800
ss86325667		8	52.839299	0.5222	0.0147	0.903529	0.0023	0.0011495314
ss61518641	*rs41616493*	8	52.879515	0.2371	0.1551	0.693836	0.0087	0.0031404406
ss86340854		8	52.984594	0.4405	0.7526	0.385946	0.0167	0.0082240977
ss86307158		8	53.070747	0.4090	0.1444	0.704033	0.0071	0.0034107526
	rs29027060	8	53.091379	0.5996	0.5111	0.474920	0.0136	0.0065179525
ss117969239		8	53.113747	0.5967	2.2218	0.136527	0.0289	0.0139128319
ss61559977	*rs41654704*	8	53.147401	0.2264	0.0062	0.937054	0.0018	0.0006201858
ss65471253	*rs43557189*	8	53.208328	0.6440	4.1537	0.041922	0.0395	0.0181118582
ss117969315		8	53.232127	0.2615	1.4497	0.228982	0.0252	0.0097413516
ss61473657	*rs41573073*	8	53.274397	0.3274	0.1033	0.747954	0.0063	0.0027697086
ss86340105		8	53.331045	0.1139	1.5362	0.215608	0.0356	0.0071910088
ss64925409	*rs43020150*	8	53.370149	0.4728	0.9108	0.340224	0.0177	0.0088245851
	rs29024656	8	53.409257	0.3746	1.0143	0.314236	0.0192	0.0090136624
ss65472680	*rs43554015*	8	53.499374	0.2264	0.4487	0.503192	0.0147	0.0051441989
ss64731040	*rs42829702*	8	53.599974	0.5093	4.2588	0.039417	0.0392	0.0195736868
ss64731135	*rs42830396*	8	53.630814	0.3173	0.4899	0.484195	0.0139	0.0060184958
ss61477653	*rs41578582*	8	53.670621	0.5766	3.1829	0.074849	0.0333	0.0162623858
ss61560026	*rs41655552*	8	53.741018	0.1146	6.6970	0.009859	0.0752	0.0152651845
ss86312018		8	53.765347	0.4198	8.8295	0.003066	0.0546	0.0265890574
ss65472511	*rs43552948*	8	53.793115	0.8775	0.6018	0.438154	0.0213	0.0045826667
ss65472445	*rs43551782*	8	53.829683	0.2658	0.2167	0.641746	0.0097	0.0038027634
ss86325546		8	53.877404	0.2221	2.0344	0.154220	0.0324	0.0112111340
ss61523735	*rs41622894*	8	53.906227	0.6633	1.4819	0.223888	0.0237	0.0105699432
ss65472218	*rs43561652*	8	53.942222	0.4527	0.1541	0.694761	0.0072	0.0035698888
ss65472168	*rs43560602*	8	53.965339	0.5996	1.1283	0.288516	0.0200	0.0096075667
	rs29022887	8	53.991683	0.7328	0.1085	0.741904	0.0069	0.0027015630
ss61504503	*rs41607576*	8	54.058043	0.7865	0.4374	0.508584	0.0150	0.0050385864
ss65471958	*rs43557995*	8	54.168949	0.4957	0.7446	0.388503	0.0159	0.0079349521
ss86302693		8	54.24976	0.2751	0.0935	0.759871	0.0063	0.0025049540
ss65471890	*rs43556627*	8	54.274063	0.1605	17.5432	0.000032	0.1030	0.0277591333

ss65471827	*rs43556564*	8	54.325204	0.5881	2.8820	0.090024	0.0320	0.0155175045
ss65471785	*rs43555722*	8	54.348397	0.3574	0.0167	0.897366	0.0025	0.0011660410
ss86295249		8	54.401521	0.2292	1.1894	0.275838	0.0237	0.0083877445
ss86304141		8	54.487061	0.7693	1.3073	0.253278	0.0248	0.0087925808
ss65471599	*rs43554236*	8	54.529421	0.5759	2.2772	0.131745	0.0283	0.0138376119
ss62669477	*rs41955698*	8	54.613312	0.5960	3.1560	0.076085	0.0339	0.0163289397
ss65471515	*rs43553252*	8	54.700872	0.1476	2.3562	0.125237	0.0416	0.0104634817
ss61523734	*rs41622893*	8	54.749195	0.2958	5.4730	0.019595	0.0477	0.0198608028
ss61559987	*rs41654714*	8	54.780848	0.7658	5.1186	0.023978	0.0501	0.0179866428
ss61559986	*rs41654713*	8	54.836332	0.7314	1.6150	0.204209	0.0266	0.0104585110
ss61559984	*rs41654711*	8	54.86488	0.2679	1.8331	0.176203	0.0283	0.0111191449
ss86300619		8	54.914245	0.1798	3.2103	0.073611	0.0425	0.0125206816
ss86307163		8	54.996604	0.7199	3.9538	0.047157	0.0411	0.0165738477
ss86340272		8	55.062808	0.6540	0.1575	0.691635	0.0078	0.0035247505
ss86290594		8	55.128197	0.8102	4.9848	0.025888	0.0537	0.0165141667
	rs29016114	8	55.229629	0.7242	10.8633	0.001031	0.0676	0.0269954885
ss86322102		8	55.273004	0.3338	16.1442	0.000065	0.0771	0.0342735046
ss61536791	*rs41634715*	8	55.326763	0.6597	16.9340	0.000043	0.0797	0.0357744657
ss86315692		8	55.399562	0.6125	3.3670	0.066942	0.0344	0.0163072386
ss61501472	*rs41607697*	8	55.439161	0.8668	3.8580	0.049907	0.0523	0.0120802213
ss86321112		8	55.461356	0.5695	8.8559	0.003023	0.0556	0.0272458921
ss62231609	*rs41797681*	8	55.489018	0.1332	3.8580	0.049907	0.0523	0.0120802213
ss117964029		8	55.556938	0.5287	14.8902	0.000125	0.0715	0.0356381074
ss62230800	*rs41796434*	8	55.673718	0.6211	11.2921	0.000821	0.0636	0.0299328877
ss62230734	*rs41796112*	8	55.768968	0.3789	11.2921	0.000821	0.0636	0.0299328877
ss62230677	*rs41796094*	8	55.811014	0.2607	3.6517	0.056423	0.0400	0.0154094370
ss86336385		8	55.865369	0.2242	7.7152	0.005623	0.0606	0.0210943693
ss61536783	*rs41634707*	8	55.958244	0.6103	5.2685	0.022011	0.0421	0.0200148576
ss61481456	*rs41578771*	8	56.012491	0.6289	5.1688	0.023301	0.0442	0.0206362393
ss86330204		8	56.061135	0.3460	9.4979	0.002138	0.0592	0.0268054895
ss86291401		8	56.093202	0.3797	1.9170	0.166633	0.0262	0.0123322836
ss61481454	*rs41578769*	8	56.1193	0.4362	0.8077	0.369117	0.0170	0.0083531738
ss61467510		8	56.157161	0.0960	1.1541	0.283074	0.0335	0.0058086049
ss86336200		8	56.18377	0.1153	1.3535	0.245062	0.0348	0.0071023331
ss86314057		8	56.217968	0.9377	0.2608	0.609728	0.0201	0.0023497716
ss61536768	*rs41634692*	8	56.243526	0.2679	8.7725	0.003162	0.0611	0.0239859007
ss86313931		8	56.266689	0.1898	9.5815	0.002044	0.0731	0.0224718894
ss86316611		8	56.353214	0.7085	4.1975	0.040857	0.0425	0.0175499180
ss61536765	*rs41634689*	8	56.465727	0.2557	3.6546	0.056324	0.0400	0.0152189880
ss86334863		8	56.516674	0.7958	3.4752	0.062715	0.0437	0.0141845817
ss86338044		8	56.596925	0.4613	17.6628	0.000030	0.0759	0.0377427797
ss86339705		8	56.621848	0.5014	13.2259	0.000296	0.0656	0.0327885553
ss86312613		8	56.643409	0.4907	12.4862	0.000437	0.0633	0.0316528431
ss86329710		8	56.742648	0.1440	0.7693	0.380726	0.0231	0.0056883468
ss86325434		8	56.850009	0.4040	13.3011	0.000285	0.0677	0.0325912648
ss64439748	*rs42636837*	8	56.895067	0.6963	9.0268	0.002756	0.0595	0.0251707024
ss86324847		8	56.954829	0.5193	0.8161	0.366641	0.0169	0.0084554868

ss61477349	*rs41575894*	8	57.049938	0.2650	0.9922	0.319558	0.0211	0.0082025787
ss64652971	*rs42757072*	8	57.090563	0.9262	0.7887	0.374810	0.0319	0.0043588225
	rs29014649	8	57.133138	0.3653	10.1568	0.001502	0.0589	0.0273082186
	rs29025230	8	57.157503	0.3639	9.8944	0.001728	0.0581	0.0268782887
ss86300035		8	57.182941	0.7199	0.3338	0.563625	0.0118	0.0047540578
ss64418784	*rs42628227*	8	57.213002	0.6046	1.2244	0.268883	0.0205	0.0098055117
ss61569719	*rs41669583*	8	57.237849	0.6304	1.0004	0.317565	0.0193	0.0089976660
ss86316138		8	57.343901	0.8460	0.2825	0.595250	0.0132	0.0034421515
ss64882019	*rs29015202*	8	57.372212	0.5236	5.1711	0.023269	0.0426	0.0212437232
ss86285123		8	57.408606	0.5766	4.2112	0.040532	0.0383	0.0187049291
ss64837677	*rs42932787*	8	57.431747	0.4678	4.1772	0.041347	0.0383	0.0190550809
	rs29015846	8	57.634144	0.3388	1.9479	0.163254	0.0272	0.0122025619
	rs29014049	8	57.66275	0.0946	1.1536	0.283169	0.0338	0.0057944537
ss61472268	*rs41568906*	8	57.74301	0.8997	0.0311	0.859991	0.0056	0.0010016149
ss64631504	*rs42731426*	8	57.768228	0.7034	4.2935	0.038627	0.0418	0.0174530271
ss86306831		8	57.820861	0.2586	3.0593	0.080719	0.0360	0.0138070441
ss65033106	*rs43127846*	8	57.852126	0.2607	3.1142	0.078049	0.0362	0.0139570104
ss61475067	*rs41571460*	8	57.876267	0.3030	6.1389	0.013461	0.0499	0.0210884051
ss63916100	*rs43771864*	8	57.912242	0.2450	4.5764	0.032762	0.0455	0.0168224086
ss86315576		8	57.936158	0.7120	5.9752	0.014755	0.0500	0.0204938459
ss86304441		8	57.97082	0.2529	4.7608	0.029449	0.0463	0.0175102415
ss86279159		8	58.021686	0.2450	4.5755	0.032779	0.0455	0.0168220195
ss61475068	*rs41571461*	8	58.050292	0.7228	6.0684	0.014003	0.0513	0.0205590930
ss61522404	*rs41619378*	8	58.083517	0.7486	3.3442	0.067868	0.0388	0.0145892443
ss86323784		8	58.152019	0.2600	5.5766	0.018476	0.0492	0.0189485953
	rs29024221	8	58.194225	0.2436	4.0153	0.045477	0.0426	0.0157022514
ss86332307		8	58.254074	0.7564	4.0153	0.045477	0.0426	0.0157022514
ss38326906	*rs29015208*	8	58.282118	0.2572	5.8770	0.015594	0.0507	0.0193779293
ss86338559		8	58.313859	0.2529	5.6728	0.017498	0.0498	0.0188320281
ss63484817	*rs42245819*	8	58.408507	0.2428	4.0002	0.045882	0.0425	0.0156447464
ss61525535	*rs41621747*	8	58.447039	0.8811	5.4852	0.019459	0.0683	0.0143085917
ss63485288	*rs42247876*	8	58.47623	0.8417	1.9358	0.164574	0.0360	0.0095939166
ss86286182		8	58.507937	0.7006	6.5095	0.010943	0.0504	0.0211305415
	rs29026472	8	58.554852	0.1046	10.7362	0.001103	0.0940	0.0176022700
ss86308981		8	58.6071	0.1927	3.6882	0.055208	0.0451	0.0140395509
ss117969161		8	58.683837	0.3746	0.1265	0.722194	0.0065	0.0030660200
ss86317009		8	58.72358	0.1927	3.6882	0.055208	0.0451	0.0140395509
ss61525536	*rs41621748*	8	58.77963	0.3825	2.0072	0.157006	0.0281	0.0132532920
ss117969328		8	58.883278	0.9119	0.0316	0.858920	0.0058	0.0009270664
ss61476041	*rs41579404*	8	59.113845	0.3754	1.0922	0.296350	0.0205	0.0096082399
ss65472730	*rs43555065*	8	59.169696	0.9047	0.1086	0.741827	0.0105	0.0018050577
ss61560027	*rs41655553*	8	59.223332	0.7686	0.0345	0.852636	0.0040	0.0014261021
ss86287992		8	59.25249	0.1282	3.2894	0.070157	0.0494	0.0110346618
	rs29025504	8	59.275887	0.2328	0.0519	0.819912	0.0049	0.0017546031
ss64620906	*rs42730267*	8	59.321042	0.4456	2.3541	0.125411	0.0280	0.0138434902
ss64795046	*rs42889651*	8	59.364382	0.7722	3.4139	0.065074	0.0396	0.0139312315
ss64645889	*rs42739820*	8	59.497452	0.6117	0.7309	0.392899	0.0159	0.0075370526

ss65472823	*rs43556358*	8	59.564172	0.2371	2.1884	0.139509	0.0323	0.0116957007
	rs29019488	8	59.593651	0.5136	0.8928	0.345053	0.0171	0.0085648268
ss64750267	*rs42846759*	8	59.643335	0.7292	0.7323	0.392422	0.0181	0.0071480694
	rs29018794	8	59.69169	0.7514	2.1466	0.143337	0.0305	0.0113962964
ss65472922	*rs43557756*	8	59.720374	0.0702	1.4269	0.232676	0.0447	0.0058333745
ss86336011		8	59.752975	0.8016	0.0008	0.977476	0.0006	0.0002061725
ss86275871		8	59.780227	0.4054	0.0277	0.867972	0.0030	0.0014690736
ss65472938	*rs43557772*	8	59.822719	0.1741	9.1799	0.002538	0.0728	0.0209214346
ss86305903		8	59.874172	0.7092	0.2798	0.597012	0.0108	0.0044354527
ss61560032	*rs41655558*	8	59.915904	0.3352	0.5739	0.448987	0.0147	0.0065366641
ss86313510		8	59.981296	0.2822	0.1042	0.746956	0.0066	0.0026686949
ss61492621	*rs41588871*	8	60.007262	0.5580	0.3549	0.551552	0.0109	0.0053609992
ss61560033	*rs41655559*	8	60.043454	0.5931	3.3550	0.067429	0.0338	0.0163272668
ss61480124		8	60.077464	0.8503	2.7714	0.096411	0.0441	0.0112351087
ss86315678		8	60.127887	0.1433	2.0280	0.154871	0.0382	0.0093733113
ss117964002		8	60.221695	0.7579	0.8469	0.357757	0.0200	0.0073225909
ss61923719	*rs41694419*	8	60.265856	0.8345	4.9432	0.026514	0.0541	0.0149280160
ss61465725	*rs41568969*	8	60.300453	0.6418	1.1335	0.287394	0.0206	0.0094587129
ss61511915	*rs41616219*	8	60.573313	0.5337	1.1875	0.276217	0.0205	0.0101859614
ss64366901	*rs42607294*	8	60.611943	0.0544	1.2216	0.269429	0.0454	0.0046757724
ss61568511	*rs41664602*	8	60.666425	0.2679	1.0278	0.311035	0.0210	0.0082185061
ss65004708	*rs43097079*	8	60.812736	0.4420	1.2402	0.265825	0.0206	0.0101781913
ss61531719	*rs41629271*	8	60.83871	0.4420	1.2402	0.265825	0.0206	0.0101781913
ss65473060	*rs43558894*	8	60.886376	0.1318	3.7956	0.051789	0.0516	0.0118203407
ss117969427		8	60.945964	0.8832	1.8323	0.176300	0.0389	0.0080253469
ss86320869		8	61.078765	0.4090	0.9111	0.340160	0.0186	0.0089741572
ss86287078		8	61.16506	0.1103	1.4547	0.228193	0.0353	0.0069279903
ss61525346	*rs41620373*	8	61.207443	0.4090	4.3244	0.037935	0.0387	0.0186971187
ss64812274	*rs42907435*	8	61.23761	0.6082	0.1618	0.687590	0.0077	0.0036855029
ss61560038	*rs41655564*	8	61.290604	0.8789	0.0003	0.985557	0.0005	0.0001128092
ss86315151		8	61.353281	0.1196	0.1144	0.735248	0.0099	0.0020931527
ss64337076	*rs42588799*	8	61.392974	0.4040	1.1455	0.284869	0.0197	0.0095029665
ss61567856	*rs41665654*	8	61.562582	0.4649	0.0337	0.854497	0.0035	0.0017466881
ss86301177		8	61.605459	0.4678	0.4116	0.521355	0.0112	0.0055968254
ss64626230	*rs42727217*	8	61.630618	0.4062	0.2119	0.645412	0.0084	0.0040498532
ss86335499		8	61.710727	0.6268	0.4551	0.500168	0.0130	0.0060674729
ss65473298	*rs43550935*	8	61.756587	0.7607	0.8761	0.349599	0.0200	0.0072644678
ss65473318	*rs43550954*	8	61.787747	0.1511	3.5457	0.060116	0.0478	0.0122579585
ss61570646	*rs41670200*	8	61.976168	0.2880	0.9504	0.329947	0.0200	0.0082086203
ss86327799		8	61.998307	0.0473	0.7648	0.382122	0.0375	0.0033815623
ss61504516	*rs41607589*	8	62.04975	0.9334	0.2738	0.600944	0.0195	0.0024301396
ss65473520	*rs43553056*	8	62.150285	0.9234	0.0444	0.833101	0.0074	0.0010423118
	rs29026049	8	62.198462	0.7837	0.5460	0.460220	0.0167	0.0056509975
ss86337065		8	62.281844	0.4105	3.9061	0.048507	0.0360	0.0174249627
	rs29018512	8	62.353462	0.3102	6.9905	0.008379	0.0520	0.0222452950
ss86314494		8	62.402745	0.4499	2.9672	0.085414	0.0309	0.0152753236
ss86325541		8	62.437153	0.1361	6.3876	0.011712	0.0667	0.0156796994

ss86294603		8	62.470656	0.8173	0.0189	0.890810	0.0032	0.0009703244
ss61560051	*rs41655577*	8	62.513762	0.2428	0.2117	0.645577	0.0099	0.0036261226
ss86333860		8	62.534945	0.0946	3.0112	0.083132	0.0541	0.0092699871
ss86289550		8	62.564652	0.3331	0.7354	0.391427	0.0167	0.0074382512
ss86274460		8	62.597297	0.1153	2.0862	0.149084	0.0429	0.0087516972
	rs29020823	8	62.689454	0.6805	2.3010	0.129748	0.0296	0.0128590087
ss61521493	*rs41619597*	8	62.972343	0.8130	2.8429	0.092224	0.0397	0.0120552100
ss86340631		8	63.04359	0.3861	0.0755	0.783540	0.0054	0.0025582081
ss64503442	*rs42673808*	8	63.091237	0.8288	0.3922	0.531352	0.0148	0.0041859885
ss86323515		8	63.141007	0.4334	0.0272	0.869138	0.0031	0.0015301502
ss86286379		8	63.178504	0.7292	0.0142	0.905322	0.0025	0.0009834843
ss86341447		8	63.200985	0.6325	0.0030	0.956221	0.0010	0.0004850918
ss86308009		8	63.25764	0.5122	0.1528	0.696016	0.0069	0.0034524788
ss65476144	*rs43558581*	8	63.376582	0.1698	0.3227	0.570163	0.0140	0.0039509445
ss61560168	*rs41656490*	8	63.400419	0.4864	0.3858	0.534713	0.0114	0.0056937232
ss61560169	*rs41656491*	8	63.427913	0.4785	0.0722	0.788206	0.0049	0.0024669007
ss86285894		8	63.475442	0.7450	0.1609	0.688439	0.0084	0.0031789701
ss117969463		8	63.49563	0.1741	0.2626	0.608515	0.0125	0.0035992345
ss65474313	*rs43552248*	8	63.545234	0.6032	0.9353	0.333820	0.0177	0.0084906515
ss61492656	*rs41588906*	8	63.572524	0.1841	1.3262	0.249877	0.0270	0.0081019339
ss61560111	*rs41656435*	8	63.596203	0.8481	1.7274	0.189173	0.0340	0.0087624709
ss86308752		8	63.646896	0.9004	0.0056	0.940208	0.0023	0.0004199894
ss86325006		8	63.692303	0.7665	1.0763	0.299886	0.0223	0.0079661194
ss86275152		8	63.736105	0.0516	0.0018	0.966232	0.0018	0.0001769683
ss86301513		8	63.778877	0.4957	0.3922	0.531364	0.0115	0.0057251654
ss86336679		8	63.829036	0.2865	1.9617	0.161780	0.0285	0.0116360354
ss86312767		8	63.859949	0.7149	1.8954	0.169035	0.0280	0.0113990954
ss86322805		8	63.885569	0.1024	1.3660	0.242907	0.0353	0.0064961671
ss86320103		8	63.915439	0.2436	0.0346	0.852405	0.0042	0.0015360262
ss65229084	*rs43313506*	8	64.005593	0.9198	0.5324	0.465834	0.0251	0.0037052671
ss65229094	*rs43313516*	8	64.029861	0.9198	0.5324	0.465834	0.0251	0.0037052671
ss65229127	*rs43314549*	8	64.072482	0.0838	0.6289	0.428030	0.0268	0.0041228704
ss86338170		8	64.104054	0.8001	2.2071	0.137825	0.0348	0.0111419713
ss65474856	*rs43558286*	8	64.139578	0.0824	0.5772	0.447660	0.0259	0.0039139321
ss65474797	*rs43557027*	8	64.199052	0.0809	0.5034	0.478233	0.0243	0.0036203623
ss61560137	*rs41656461*	8	64.225125	0.2851	2.5372	0.111649	0.0327	0.0133312346
ss61560130	*rs41656454*	8	64.296858	0.2020	4.6693	0.031047	0.0495	0.0159698438
ss46526288	*rs41255427*	8	64.338782	0.8073	1.9743	0.160434	0.0333	0.0103560348
ss61560124	*rs41656448*	8	64.36502	0.0630	3.2081	0.073707	0.0692	0.0081753805
ss86299244		8	64.405696	0.1812	1.5917	0.207512	0.0306	0.0090898368
ss86307657		8	64.430931	0.8145	1.4647	0.226595	0.0290	0.0087600167
ss86317494		8	64.498603	0.6440	0.4805	0.488427	0.0137	0.0062880906
ss86284598		8	64.537453	0.3524	0.3621	0.547535	0.0120	0.0054684134
ss86295837		8	64.601988	0.1526	1.8447	0.174843	0.0349	0.0090373113
ss61510892	*rs41607812*	8	64.685646	0.2085	0.4110	0.521653	0.0145	0.0047792692
ss86335941		8	64.795928	0.2586	0.2617	0.609083	0.0108	0.0041370209
ss86335261		8	64.817059	0.1476	1.1303	0.288086	0.0287	0.0072142833

ss86309240		8	64.841354	0.6956	0.0784	0.779546	0.0058	0.0024742223
ss86328615		8	64.871498	0.8352	1.2298	0.267832	0.0266	0.0073148788
ss61492633	*rs41588883*	8	64.903026	0.3789	1.0336	0.309664	0.0191	0.0089897942
ss86298899		8	64.962959	0.2428	0.4271	0.513649	0.0142	0.0052159432
ss65474095	*rs43559023*	8	64.992133	0.8324	1.3794	0.240612	0.0286	0.0079780217
ss86305956		8	65.136891	0.6705	5.6825	0.017402	0.0466	0.0206042432
ss86327640		8	65.161365	0.3940	0.1161	0.733364	0.0065	0.0030877015
ss86337327		8	65.18225	0.4033	0.0782	0.779777	0.0053	0.0025535981
ss117969330		8	65.224134	0.1511	0.7938	0.373262	0.0239	0.0061297015
ss86334978		8	65.288271	0.1289	10.3322	0.001368	0.0891	0.0200146260
ss38325262	*rs29010976*	8	65.569796	0.1640	9.1893	0.002525	0.0745	0.0204420903
	rs29018125	8	65.595479	0.1841	9.3285	0.002342	0.0717	0.0215450300
ss117969483		8	65.707979	0.2192	2.5550	0.110398	0.0358	0.0122511732
ss86318375		8	65.733362	0.3431	1.2586	0.262312	0.0222	0.0099899296
ss86285282		8	65.785347	0.5745	2.3478	0.125917	0.0290	0.0141694556
ss61560182	*rs41656504*	8	65.835036	0.5802	0.2039	0.651719	0.0086	0.0042115325
ss86302168		8	65.933357	0.1325	0.5224	0.470079	0.0199	0.0045822693
ss65476693	*rs43556128*	8	65.975257	0.8675	0.3463	0.556384	0.0162	0.0037317443
ss86339241		8	66.00593	0.8524	0.0584	0.809189	0.0062	0.0015622618
	rs29025228	8	66.105222	0.1454	2.8919	0.089472	0.0454	0.0112856287
ss86330729		8	66.205535	0.8481	3.0161	0.082885	0.0459	0.0118133604
ss86304179		8	66.24904	0.1691	0.8587	0.354428	0.0225	0.0063323768
ss65476913	*rs43557846*	8	66.308739	0.1748	0.3543	0.551868	0.0143	0.0041339526
ss86297564		8	66.334531	0.1741	0.4376	0.508488	0.0160	0.0046041061
ss65476723	*rs43556658*	8	66.363635	0.5630	0.0301	0.862208	0.0033	0.0016476700
ss61560185	*rs41656507*	8	66.388546	0.2407	3.5211	0.061010	0.0406	0.0148359630
ss86283535		8	66.419039	0.5566	0.1414	0.706983	0.0074	0.0036438699
ss86297096		8	66.443148	0.5709	0.0687	0.793291	0.0049	0.0024035638
ss86304086		8	66.465601	0.2858	1.5368	0.215508	0.0253	0.0103183070
	rs29025935	8	66.500033	0.2292	6.8666	0.008974	0.0578	0.0204121492
ss86335363		8	66.591477	0.2034	4.2069	0.040635	0.0475	0.0154007321
	rs29021656	8	66.635618	0.3159	3.3105	0.069267	0.0361	0.0156192349
ss61492719	*rs41589869*	8	66.726693	0.6354	6.5839	0.010499	0.0507	0.0235078297
ss64496946	*rs42670201*	8	66.775377	0.5208	3.6462	0.056610	0.0357	0.0178028418
ss86323536		8	66.813593	0.5351	1.1934	0.275024	0.0202	0.0100333524
ss86313305		8	66.835378	0.7364	4.0019	0.045838	0.0416	0.0161656024
ss86308707		8	66.872684	0.3195	1.8276	0.176849	0.0264	0.0114990062
ss86317882		8	66.893701	0.6805	0.2263	0.634397	0.0094	0.0040779534
ss63496580	*rs42248819*	8	66.929361	0.6361	0.0153	0.901496	0.0024	0.0010971324
ss63496793	*rs42249787*	8	67.02641	0.2636	0.3513	0.553584	0.0123	0.0047710197
ss86323363		8	67.103063	0.1605	0.0004	0.984987	0.0005	0.0001309394
ss63497252	*rs42251740*	8	67.152471	0.8223	2.1241	0.145447	0.0354	0.0103570680
ss86319246		8	67.189619	0.8309	0.0000	0.996011	0.0001	0.0000356814
ss65478123	*rs43563355*	8	67.222701	0.1984	2.3468	0.125992	0.0359	0.0114257270
ss61492717	*rs41589867*	8	67.24715	0.5086	7.5693	0.006092	0.0505	0.0252252228
ss61560252	*rs41657273*	8	67.282321	0.4914	8.8916	0.002965	0.0545	0.0272632619
ss86296222		8	67.320236	0.8926	0.9858	0.321116	0.0289	0.0055445054

ss117969351		8	67.349748	0.1640	0.0437	0.834453		0.0051	0.0014045990
ss61498964	*rs43708063*	8	67.376484	0.9011	0.0495	0.824008		0.0067	0.0011939344
ss63497984	*rs42254183*	8	67.446307	0.1010	8.5411	0.003585		0.0902	0.0163858163
ss61476597	*rs41577752*	8	67.695502	0.4628	0.0183	0.892533		0.0025	0.0012449102
ss86336492		8	67.988154	0.9105	1.5759	0.209769		0.0410	0.0066791489
ss63094705	*rs42112716*	8	68.027546	0.7113	0.1660	0.683829		0.0083	0.0033959806
ss86340815		8	68.294393	0.4577	0.0800	0.777335		0.0056	0.0027596269
ss61505413	*rs41606159*	8	68.358361	0.6218	0.8629	0.353249		0.0178	0.0083672322
ss86300762		8	68.392366	0.2436	7.1522	0.007663		0.0589	0.0217061096
ss86340910		8	68.438335	0.5938	0.2248	0.635559		0.0092	0.0044407695
ss64968240	*rs43063081*	8	68.525502	0.5938	0.2248	0.635559		0.0092	0.0044407695
ss65000575	*rs43088996*	8	68.577512	0.9620	6.5449	0.010729		0.1246	0.0091009466
ss86331577		8	68.645164	0.5415	0.1035	0.747738		0.0063	0.0031435962
ss86338729		8	68.680839	0.4871	1.6424	0.200426		0.0242	0.0120780060
ss64864553	*rs42958947*	8	68.757477	0.3109	0.0916	0.762218		0.0061	0.0026167957
ss64245130	*rs42545505*	8	68.846278	0.1454	1.8892	0.169737		0.0359	0.0089247090
ss86308976		8	69.286959	0.6476	0.2067	0.649483		0.0087	0.0039552867
ss63663278	*rs42302384*	8	69.412907	0.6648	0.0243	0.876208		0.0030	0.0013386543
ss86305424		8	69.441041	0.1655	3.1697	0.075453		0.0444	0.0122691212
ss63663812	*rs42304560*	8	69.476591	0.8589	6.5199	0.010880		0.0676	0.0163895017
ss86337089		8	69.510506	0.4971	1.0317	0.310117		0.0190	0.0094950210
ss64524776	*rs42679356*	8	69.618406	0.5466	0.0002	0.989150		0.0003	0.0001251362
ss86337846		8	69.668226	0.1003	4.6035	0.032252		0.0662	0.0119390638
ss86327142		8	69.820393	0.2765	1.8374	0.175700		0.0278	0.0111421104
ss86323597		8	69.840959	0.2500	1.1709	0.279591		0.0228	0.0085574276
ss117966013		8	69.997378	0.4907	0.7421	0.389275		0.0161	0.0080458717
ss86337294		8	70.027532	0.5279	3.1070	0.078394		0.0327	0.0163000274
ss86288142		8	70.16672	0.1877	1.8822	0.170523		0.0334	0.0101819638
ss64203896	*rs42523953*	8	70.27076	0.1189	0.2101	0.646813		0.0133	0.0027874858
ss117963987		8	70.303316	0.5215	2.3051	0.129405		0.0283	0.0141217049
ss117969209		8	70.345144	0.4828	2.1175	0.146078		0.0273	0.0136114908
ss86340634		8	70.513319	0.1784	0.3977	0.528477		0.0152	0.0044642532
ss86337607		8	70.574616	0.0738	1.7436	0.187118		0.0468	0.0064021179
ss86307011		8	70.599185	0.0716	1.9003	0.168482		0.0494	0.0065768483
ss86293536		8	70.661245	0.8274	0.1549	0.693996		0.0097	0.0027842867
	rs29027144	8	70.710766	0.1533	0.4773	0.489884		0.0180	0.0046780431
ss86321975		8	70.740506	0.5989	0.1510	0.697721		0.0077	0.0036928701
ss86319344		8	70.761615	0.5437	0.1607	0.688591		0.0079	0.0039131651
ss64460926	*rs42647421*	8	70.820444	0.5817	1.7347	0.188249		0.0253	0.0122911789
ss86290393		8	70.884249	0.5344	0.0283	0.866373		0.0032	0.0015806835
ss86309694		8	70.91093	0.4054	0.0053	0.941901		0.0014	0.0006744690
ss86304171		8	70.981832	0.5695	0.0644	0.799707		0.0050	0.0024310777
ss86325550		8	71.013397	0.3675	0.8594	0.354232		0.0178	0.0082898284
ss61515780	*rs41616723*	8	71.035719	0.3309	0.7089	0.400102		0.0174	0.0076891172
ss86341639		8	71.089122	0.6755	7.3979	0.006693		0.0532	0.0233164952
ss61507603	*rs41607892*	8	71.152774	0.6956	0.0817	0.775056		0.0058	0.0024438991
ss86297374		8	71.191368	0.1941	0.0156	0.900734		0.0030	0.0009382941

ss63706770	*rs42323908*	8	71.250905	0.7221	0.0133	0.908367	0.0025	0.0009977728
ss63707391	*rs42326114*	8	71.341783	0.8782	0.3558	0.551033	0.0169	0.0036236083
	rs29012097	8	71.382488	0.1755	0.9639	0.326553	0.0237	0.0068656618
	rs29013326	8	71.40863	0.3095	0.0147	0.903561	0.0025	0.0010486804
	rs29010321	8	71.438675	0.9083	0.1548	0.694106	0.0128	0.0021251446
	rs29023459	8	71.463369	0.1010	3.3763	0.066565	0.0569	0.0103363679
ss86336229		8	71.505453	0.2013	1.6237	0.202998	0.0296	0.0095084815
ss86311279		8	71.52268	0.4413	0.6198	0.431402	0.0151	0.0074270368
	rs29021271	8	71.547144	0.1039	0.5433	0.461328	0.0221	0.0041226203
ss117969253		8	71.590809	0.4785	1.7592	0.185162	0.0245	0.0122038277
ss86291197		8	71.643119	0.8854	0.0620	0.803470	0.0072	0.0014511489
ss86302898		8	71.799052	0.7736	2.0686	0.150808	0.0316	0.0110549253
ss86324349		8	71.831267	0.3374	9.9082	0.001716	0.0614	0.0274423789
ss61560289	*rs41657309*	8	71.875776	0.7607	2.4669	0.116719	0.0333	0.0121044598
ss86318054		8	71.955583	0.3102	1.4614	0.227114	0.0238	0.0101918686
ss86280367		8	71.997119	0.2708	1.2250	0.268773	0.0228	0.0089890470
ss86339546		8	72.054744	0.3123	0.4487	0.503175	0.0136	0.0058407141
ss86336742		8	72.104543	0.4255	0.0728	0.787442	0.0053	0.0025706939
ss86278996		8	72.136389	0.5000	0.4215	0.516416	0.0126	0.0062978070
ss61560283	*rs41657303*	8	72.16048	0.5143	1.0599	0.303594	0.0196	0.0097803845
ss86335878		8	72.190513	0.6418	4.9554	0.026329	0.0431	0.0198189731
ss61492739	*rs41589887*	8	72.230104	0.2966	0.3588	0.549391	0.0120	0.0050165405
ss86283546		8	72.254902	0.2801	0.2563	0.612821	0.0107	0.0043064426
ss86292452		8	72.326124	0.3668	0.7726	0.379708	0.0171	0.0079490736
ss86293642		8	72.361839	0.3095	0.6727	0.412397	0.0166	0.0070755866
ss65475589	*rs43555328*	8	72.386887	0.7156	2.0702	0.150650	0.0299	0.0121786177
ss86314192		8	72.427755	0.1383	0.9548	0.328849	0.0270	0.0064238008
ss86282117		8	72.487664	0.5115	1.1455	0.284866	0.0204	0.0101731332
ss86318104		8	72.585461	0.4377	15.7007	0.000082	0.0731	0.0359835065
ss65475392	*rs43554821*	8	72.661667	0.1984	0.2289	0.632456	0.0110	0.0034926678
ss86296182		8	72.704598	0.2865	3.7225	0.054090	0.0388	0.0158526688
ss86320331		8	72.76182	0.2765	3.9450	0.047402	0.0398	0.0159235916
ss117964012		8	72.78895	0.5931	3.9318	0.047773	0.0365	0.0175984564
ss61523738	*rs41622897*	8	72.826006	0.6110	2.0544	0.152215	0.0264	0.0125562682
ss64610024	*rs42717895*	8	72.867992	0.4413	9.2245	0.002477	0.0548	0.0270289608
ss64610008	*rs42717887*	8	72.917818	0.8524	2.8842	0.089899	0.0445	0.0111990271
ss46526014	*rs41255162*	8	72.995807	0.6748	0.4083	0.523033	0.0126	0.0055419284
ss86340420		8	73.029995	0.4635	0.6304	0.427483	0.0146	0.0072648416
ss61510905	*rs41609434*	8	73.06727	0.2335	4.9148	0.026951	0.0476	0.0170492969
ss86300830		8	73.129951	0.1855	0.6854	0.408009	0.0195	0.0058795714
ss86340409		8	73.156161	0.2371	5.3784	0.020677	0.0517	0.0187082670
ss61560139	*rs41656463*	8	73.179532	0.5931	0.0848	0.770966	0.0055	0.0026564270
ss65121867	*rs43207718*	8	73.850806	0.1533	0.3635	0.546776	0.0155	0.0040342131
ss117969167		8	73.945616	0.1490	0.7497	0.386880	0.0225	0.0056960184
ss86337031		8	73.976064	0.6175	0.9920	0.319591	0.0191	0.0090441046
ss86334525		8	74.012187	0.2170	0.0699	0.791501	0.0059	0.0020085203
ss86304558		8	74.043769	0.6168	0.6816	0.409310	0.0164	0.0077619928

ss86292001		8	74.105201	0.8768	2.9359	0.087077	0.0468	0.0101154032
ss86337131		8	74.138951	0.5272	1.7574	0.185383	0.0243	0.0121325503
ss86273563		8	74.167325	0.3782	8.6605	0.003360	0.0559	0.0263014648
	rs29024484	8	74.208708	0.2421	7.6056	0.005971	0.0579	0.0212360979
ss86299292		8	74.274147	0.6862	1.3539	0.244994	0.0232	0.0099921738
ss86340507		8	74.412638	0.2285	5.5272	0.019001	0.0540	0.0190364800
ss61560298	*rs41657318*	8	74.438659	0.7350	0.2489	0.618041	0.0106	0.0041289000
ss65480035	*rs43562058*	8	74.468366	0.3983	0.3749	0.540571	0.0118	0.0056748435
ss117969418		8	74.54221	0.5064	0.0500	0.823196	0.0042	0.0021156029
ss61494892	*rs41591479*	8	74.59916	0.3181	0.2605	0.609946	0.0101	0.0044016219
ss86309208		8	74.85036	0.3653	0.0500	0.823137	0.0043	0.0019938041
ss86287824		8	74.93774	0.6734	0.5751	0.448510	0.0152	0.0066733634
ss64423934	*rs42630027*	8	75.171544	0.5516	7.1993	0.007466	0.0500	0.0247306908
ss65092058	*rs43179179*	8	75.215235	0.1096	0.6777	0.410674	0.0245	0.0047889487
ss63853418	*rs42383597*	8	75.237598	0.5774	1.8676	0.172195	0.0258	0.0126043309
ss63853564	*rs42384370*	8	75.260556	0.0688	1.0960	0.295508	0.0395	0.0050545829
ss63853424	*rs42383600*	8	75.28508	0.6762	0.0768	0.781762	0.0055	0.0024159170
ss86300902		8	75.462841	0.2722	1.0665	0.302087	0.0219	0.0086832945
ss64991107	*rs43078791*	8	75.510312	0.7901	5.8290	0.016021	0.0544	0.0180341225
ss86287406		8	75.555515	0.1547	1.7229	0.189756	0.0331	0.0086710000
ss117975014		8	75.619457	0.2529	1.3340	0.248483	0.0245	0.0092645371
ss86340296		8	75.656958	0.2529	1.3340	0.248483	0.0245	0.0092645371
ss65476328	*rs43559664*	8	75.719992	0.7106	7.6693	0.005766	0.0568	0.0233411498
ss65476262	*rs43559099*	8	75.770552	0.1777	1.5571	0.212515	0.0305	0.0089122696
ss65481690	*rs43562510*	8	75.850763	0.6519	22.0618	0.000003	0.0916	0.0415794914
ss86335847		8	76.17495	0.6519	1.8569	0.173425	0.0269	0.0122044751
ss65481583	*rs43561903*	8	76.218601	0.3610	0.5841	0.444970	0.0151	0.0069772416
ss86298801		8	76.258746	0.3403	0.0512	0.820992	0.0045	0.0020398076
ss61560325	*rs41658245*	8	76.280028	0.9140	2.9644	0.085561	0.0570	0.0089634747
ss65481297	*rs43565716*	8	76.326384	0.9097	1.2121	0.271290	0.0362	0.0059470664
ss86309326		8	76.414522	0.9176	1.0209	0.312653	0.0341	0.0051502514
ss61560318	*rs41658238*	8	76.449943	0.0681	1.4332	0.231644	0.0438	0.0055593392
ss86318842		8	76.496014	0.0516	3.8860	0.049087	0.0827	0.0080905972
ss65481166	*rs43565085*	8	76.527562	0.8625	3.8180	0.051105	0.0532	0.0126303834
ss65481080	*rs43564499*	8	76.646783	0.5616	5.3539	0.020966	0.0439	0.0216346677
ss65480971	*rs43563590*	8	76.760017	0.4126	0.7327	0.392298	0.0161	0.0077835658
ss61560315	*rs41658235*	8	76.786317	0.1626	5.3056	0.021550	0.0579	0.0157588822
ss61492758	*rs41589906*	8	76.825888	0.4219	1.8737	0.171494	0.0259	0.0126116579
ss61560312	*rs41658232*	8	76.8517	0.8345	0.0342	0.853248	0.0046	0.0012614775
ss86314132		8	76.892937	0.0509	0.0173	0.895447	0.0056	0.0005375423
ss61523740	*rs41622899*	8	76.92685	0.9083	0.0037	0.951783	0.0020	0.0003325713
ss65480792	*rs43562311*	8	76.958677	0.4549	4.0611	0.044266	0.0381	0.0188936408
ss86273596		8	77.112508	0.1440	0.0170	0.896417	0.0036	0.0008760720
ss63249347	*rs42163449*	8	77.381654	0.5996	7.9408	0.004970	0.0537	0.0258022735
ss86322837		8	77.426334	0.3066	5.2200	0.022628	0.0450	0.0191183616
ss46526215	*rs41255355*	8	77.582293	0.5759	6.7578	0.009532	0.0477	0.0232821817
ss61569145	*rs41665531*	8	77.604145	0.0681	5.2670	0.022030	0.0837	0.0106200935

ss86283803		8	77.630641	0.2221	5.2637	0.022072	0.0499	0.0172494938
ss86318512		8	77.681871	0.1862	8.2100	0.004292	0.0678	0.0205619802
ss86323196		8	77.712795	0.4993	3.7864	0.052072	0.0363	0.0181585297
ss65481956	*rs43564576*	8	77.742333	0.6755	2.9301	0.087388	0.0330	0.0144818118
ss86325215		8	77.765331	0.8138	9.0119	0.002778	0.0710	0.0215286340
ss61492766		8	77.807388	0.6633	2.2236	0.136370	0.0281	0.0125465240
ss86290739		8	77.83666	0.3317	4.6871	0.030730	0.0413	0.0183026249
ss61492763	*rs41589911*	8	77.918502	0.6046	3.7980	0.051714	0.0368	0.0175976190
ss86337084		8	78.011418	0.6404	2.0165	0.156046	0.0276	0.0127023406
ss65481721	*rs43563141*	8	78.108675	0.1948	9.0095	0.002782	0.0687	0.0215540550
ss64768746	*rs42866283*	8	78.344101	0.2328	11.3680	0.000788	0.0764	0.0272800602
	rs29024476	8	78.477543	0.7665	0.2243	0.635946	0.0106	0.0038066255
ss46526915	*rs41257363*	8	78.507764	0.5852	15.3937	0.000096	0.0737	0.0357648060
ss86330001		8	78.586801	0.1504	3.8101	0.051344	0.0515	0.0131632714
ss61555809	*rs41657507*	8	78.625598	0.1468	4.1650	0.041645	0.0530	0.0132853809
ss86299149		8	78.682135	0.2092	2.2279	0.135990	0.0345	0.0114284034
ss86293337		8	78.809585	0.2894	0.1919	0.661486	0.0093	0.0038394085
ss86302217		8	78.832861	0.8367	2.0023	0.157509	0.0358	0.0097743113
ss65351382	*rs43432590*	8	78.863377	0.6648	0.2354	0.627693	0.0094	0.0041839321
ss86322817		8	78.893035	0.2600	1.5754	0.209840	0.0261	0.0100251546
ss65351302	*rs43431410*	8	78.922676	0.7400	1.5754	0.209840	0.0261	0.0100251546
ss86335815		8	78.955853	0.4255	0.0014	0.969897	0.0007	0.0003492996
ss86331857		8	79.006748	0.1196	2.3469	0.125991	0.0428	0.0090213477
ss86318876		8	79.035195	0.4749	0.0008	0.977709	0.0005	0.0002584414
ss86309544		8	79.144683	0.0695	8.8243	0.003075	0.1060	0.0137110823
ss65482463	*rs43562582*	8	79.194415	0.0910	0.1874	0.665236	0.0142	0.0023443371
ss86338548		8	79.245047	0.7436	5.0891	0.024387	0.0466	0.0177666775
ss61526100	*rs41624893*	8	79.313791	0.1139	6.4587	0.011257	0.0729	0.0147177781
ss86319500		8	79.342941	0.1590	4.6431	0.031521	0.0555	0.0148576523
ss61492756	*rs41589904*	8	79.396919	0.8453	5.6843	0.017385	0.0608	0.0159143674
ss61560305	*rs41657325*	8	79.435106	0.5731	12.9949	0.000335	0.0661	0.0323383763
ss86320938		8	79.483118	0.1934	2.6265	0.105548	0.0370	0.0115527755
ss65480745	*rs43562265*	8	79.591195	0.1110	6.0457	0.014182	0.0711	0.0140371098
ss65480775	*rs43562294*	8	79.625442	0.1110	6.0457	0.014182	0.0711	0.0140371098
ss61560343	*rs41658263*	8	79.658483	0.9219	3.5237	0.060917	0.0637	0.0091703908
ss86335692		8	79.714486	0.1569	5.0015	0.025641	0.0575	0.0152052805
ss117969414		8	79.752196	0.4728	3.3374	0.068150	0.0338	0.0168497309
	rs29013400	8	79.784555	0.5974	1.5170	0.218497	0.0231	0.0110879491
ss86325891		8	79.838872	0.8840	7.6314	0.005887	0.0781	0.0160300887
ss86337199		8	79.876201	0.1125	7.2835	0.007128	0.0771	0.0153824621
ss65483110	*rs43566825*	8	79.910969	0.1125	7.2835	0.007128	0.0771	0.0153824621
ss65483132	*rs43567147*	8	79.935266	0.4011	1.6421	0.200464	0.0239	0.0114991826
ss86315210		8	79.961572	0.4921	3.6498	0.056487	0.0355	0.0177464216
ss86277786		8	79.986529	0.1519	4.8712	0.027635	0.0565	0.0145549490
	rs29020898	8	80.008264	0.8481	4.8712	0.027635	0.0565	0.0145549490
ss86338769		8	80.08719	0.5867	0.0042	0.948273	0.0012	0.0005893952
ss61560382	*rs41658302*	8	80.135795	0.3173	0.8883	0.346260	0.0188	0.0081496806

ss86290384		8	80.165257	0.3030	2.3264	0.127648	0.0310	0.0130847804
ss65483347	*rs43568361*	8	80.212252	0.5580	0.2406	0.623925	0.0092	0.0045391938
ss86310622		8	80.278918	0.5301	2.2063	0.137897	0.0275	0.0137086876
ss86337336		8	80.31402	0.8804	0.2714	0.602557	0.0143	0.0030134786
ss86302950		8	80.395975	0.4140	0.7139	0.398456	0.0156	0.0075935298
ss61560385	*rs41658305*	8	80.46003	0.4112	0.8911	0.345518	0.0177	0.0085697504
ss86284397		8	80.489903	0.0509	0.2509	0.616595	0.0212	0.0020506988
ss61504585	*rs41608458*	8	80.51342	0.4491	0.9530	0.329288	0.0182	0.0089824540
ss86290604		8	80.55096	0.9398	1.6930	0.193641	0.0494	0.0055868765
ss86313508		8	80.712734	0.3245	0.3304	0.565623	0.0111	0.0048553882
ss65483787	*rs43566905*	8	80.747739	0.7385	4.8306	0.028287	0.0464	0.0179287125
ss86335869		8	80.774643	0.1282	6.7711	0.009462	0.0694	0.0155069186
ss86341616		8	80.878832	0.0917	3.1422	0.076727	0.0563	0.0093806537
ss62401016	*rs41859720*	8	80.952422	0.8095	9.5339	0.002097	0.0706	0.0217854154
ss64916764	*rs43010550*	8	81.074522	0.1461	0.3610	0.548135	0.0155	0.0038734057
ss117969195		8	81.18859	0.1862	0.0902	0.764066	0.0070	0.0021217663
ss63633777	*rs42294864*	8	81.231127	0.0509	0.0003	0.986815	0.0007	0.0000674967
ss86302369		8	81.371307	0.1354	0.4672	0.494517	0.0178	0.0041695308
ss61568043	*rs41661349*	8	81.492678	0.6791	3.5366	0.060446	0.0369	0.0160846443
ss63634395	*rs42296470*	8	81.53826	0.1884	0.0226	0.880503	0.0035	0.0010615147
ss117963990		8	81.579869	0.2822	1.5005	0.221009	0.0245	0.0099365854
ss86290560		8	81.645926	0.9155	7.5738	0.006077	0.0914	0.0141506867
ss63635475	*rs42294331*	8	81.775956	0.7421	0.1727	0.677821	0.0086	0.0033005248
ss61496291	*rs41598242*	8	81.971244	0.9205	1.6508	0.199274	0.0447	0.0065451875
ss61471896	*rs41573836*	8	82.241177	0.8761	0.7064	0.400915	0.0236	0.0051293157
ss64751517	*rs42852250*	8	82.286732	0.7851	0.0117	0.913837	0.0024	0.0008146042
	rs29020952	8	82.37308	0.7693	1.2783	0.258608	0.0255	0.0090670451
ss117969430		8	82.397505	0.8360	1.8205	0.177692	0.0342	0.0093735613
ss65484216	*rs43572534*	8	82.428048	0.0616	0.5357	0.464475	0.0286	0.0033015236
ss65484254	*rs43572572*	8	82.452309	0.8703	1.1853	0.276666	0.0310	0.0069936199
	rs29025478	8	82.498313	0.8381	0.8938	0.344783	0.0243	0.0065977777
ss61492806	*rs41590554*	8	82.573072	0.0666	0.0123	0.911749	0.0042	0.0005214541
ss86336341		8	82.613365	0.7063	1.6694	0.196767	0.0267	0.0110909109
ss61564794	*rs41663757*	8	82.656147	0.0888	0.0633	0.801451	0.0083	0.0013484861
ss86316648		8	82.714418	0.7142	0.2545	0.614057	0.0103	0.0042161851
ss64585996	*rs42710122*	8	82.749667	0.0996	10.0551	0.001586	0.0992	0.0177926961
ss64586096	*rs42711572*	8	82.776494	0.1203	0.2104	0.646615	0.0135	0.0028530613
ss64586140	*rs42711594*	8	82.809415	0.1827	0.3365	0.562038	0.0143	0.0042622184
ss61510933	*rs41609462*	8	82.873606	0.1282	0.0637	0.800750	0.0070	0.0015696974
ss65484515	*rs43564933*	8	82.899412	0.8739	0.0749	0.784408	0.0077	0.0016963987
ss86297528		8	82.931654	0.5215	0.4317	0.511355	0.0123	0.0061480183
ss86338580		8	82.96106	0.1898	2.5030	0.114083	0.0373	0.0114734252
ss86322364		8	83.028617	0.9062	0.0636	0.800979	0.0081	0.0013782588
ss86303864		8	83.399497	0.8560	1.2285	0.268084	0.0290	0.0071455526
ss62942832	*rs42052322*	8	83.437238	0.9563	1.0855	0.297824	0.0470	0.0039305141
ss61492797	*rs41590545*	8	83.521311	0.0809	1.4268	0.232695	0.0405	0.0060320444
ss61547479	*rs41646004*	8	83.652457	0.4542	0.2549	0.613787	0.0096	0.0047477919

ss61564126	*rs41658602*	8	83.712406	0.0580	1.3152	0.251849	0.0453	0.0049465308
ss65484921	*rs43569337*	8	83.761444	0.6368	0.4597	0.498003	0.0133	0.0061308342
ss64902321	*rs42992665*	8	83.882361	0.7185	9.3450	0.002322	0.0623	0.0252081721
ss86291303		8	83.907554	0.0387	10.9540	0.000982	0.1536	0.0114232368
ss63810191	*rs42359721*	8	83.952465	0.5308	0.0039	0.950394	0.0011	0.0005669073
ss86338891		8	83.980798	0.9205	0.6018	0.438173	0.0267	0.0039132447
ss63810127	*rs42359689*	8	84.13916	0.6311	2.1475	0.143251	0.0274	0.0127497628
ss61517194	*rs41612589*	8	84.225077	0.3553	0.2162	0.642111	0.0090	0.0041248688
ss86300850		8	84.301872	0.0917	4.1644	0.041660	0.0622	0.0103619818
ss65024433	*rs43113452*	8	84.39362	0.6612	5.7700	0.016563	0.0473	0.0211845858
ss64412750	*rs42626251*	8	84.421508	0.2443	0.1050	0.746035	0.0069	0.0025610250
ss61569643	*rs41668712*	8	84.45666	0.1039	12.6469	0.000402	0.1083	0.0201697303
ss86321599		8	84.623201	0.8517	0.6031	0.437643	0.0193	0.0048799425
ss64412968	*rs42627158*	8	84.655235	0.1440	0.0246	0.875507	0.0042	0.0010242049
ss117964004		8	84.68582	0.4713	0.3168	0.573745	0.0107	0.0053151284
ss64786759	*rs42884165*	8	84.713506	0.8997	9.2492	0.002445	0.0912	0.0164533105
ss65485358	*rs43574073*	8	84.889571	0.8181	3.8161	0.051162	0.0463	0.0137717880
ss63496298	*rs42247625*	8	84.943315	0.0552	0.0634	0.801201	0.0104	0.0010884653
ss86336421		8	84.971958	0.4241	0.1325	0.715982	0.0070	0.0034213398
ss63496118	*rs42247568*	8	85.053144	0.5451	0.0052	0.942585	0.0014	0.0006842879
ss86307353		8	85.081036	0.3202	4.7908	0.028944	0.0444	0.0193448131
ss63495302	*rs42249397*	8	85.179419	0.2242	4.7675	0.029335	0.0483	0.0168051909
ss86299291		8	85.205148	0.6211	4.6145	0.032047	0.0406	0.0191051746
ss86306174		8	85.263491	0.2622	3.9884	0.046203	0.0427	0.0165074490
ss61466570	*rs41570322*	8	85.387955	0.7758	2.1907	0.139296	0.0321	0.0111733423
ss63493466	*rs42246486*	8	85.420152	0.8009	0.1070	0.743731	0.0075	0.0024054212
ss117969164		8	85.451267	0.2715	0.0303	0.861947	0.0036	0.0014330518
ss63492398	*rs42254030*	8	85.796306	0.2185	1.5798	0.209204	0.0281	0.0095893470
ss86289258		8	85.869379	0.4542	0.1265	0.722165	0.0066	0.0032933105
ss61570657	*rs41670211*	8	85.898128	0.1160	0.0799	0.777563	0.0082	0.0016860727
ss117964031		8	86.000953	0.4979	0.6682	0.413975	0.0147	0.0073551197
ss86301737		8	86.028312	0.1397	6.8325	0.009145	0.0699	0.0168015200
	rs29019864	8	86.054772	0.1390	6.8578	0.009018	0.0705	0.0168730214
ss86316802		8	86.297121	0.8331	6.5025	0.010986	0.0642	0.0178500269
ss65485717	*rs43567234*	8	86.324157	0.2249	2.7233	0.099346	0.0372	0.0129578915
ss65485736	*rs43567253*	8	86.469416	0.4391	1.7858	0.181880	0.0242	0.0119205594
ss86288224		8	86.512829	0.3811	0.5554	0.456367	0.0138	0.0064926101
ss86341158		8	86.544298	0.1182	4.3378	0.037639	0.0609	0.0126903623
ss63765011	*rs43734727*	8	86.57682	0.8395	7.9530	0.004937	0.0720	0.0194092845
ss63764933	*rs43734701*	8	86.639075	0.9284	3.1017	0.078651	0.0625	0.0083087120
	rs29012379	8	86.673876	0.9040	0.7844	0.376095	0.0278	0.0048246453
ss61470513	*rs41570498*	8	86.731099	0.7930	1.4406	0.230457	0.0279	0.0091626031
ss86334471		8	86.931892	0.6218	0.9144	0.339275	0.0178	0.0083853402
ss64499540	*rs42669028*	8	87.04393	0.7629	0.3078	0.579242	0.0121	0.0043757359
ss61494018	*rs41592228*	8	87.201769	0.5158	0.1060	0.744875	0.0060	0.0030039836
ss86288435		8	87.229587	0.6211	4.2451	0.039736	0.0392	0.0184619215
ss86320445		8	87.263985	0.1848	6.2333	0.012767	0.0579	0.0174444223

ss117969487		8	87.311751	0.6418	2.7910	0.095243	0.0315	0.0144958124
ss86321151		8	87.368037	0.9269	0.4095	0.522427	0.0220	0.0029850588
ss65486539	*rs43566450*	8	87.39634	0.3266	6.4096	0.011570	0.0496	0.0218129007
ss86316663		8	87.422313	0.9248	0.2954	0.586970	0.0185	0.0025767416
ss65486507	*rs43565418*	8	87.455205	0.5265	13.7308	0.000228	0.0683	0.0340612757
ss65486448	*rs43565360*	8	87.494137	0.1712	1.7699	0.183836	0.0319	0.0090503103
ss61560426	*rs41658946*	8	87.535962	0.7149	9.0768	0.002683	0.0615	0.0250515586
ss65486332	*rs43574244*	8	87.609748	0.5387	11.4694	0.000747	0.0617	0.0306870866
ss65486000	*rs43569312*	8	87.695264	0.3453	1.0076	0.315827	0.0198	0.0089707692
ss65487745	*rs43568453*	8	87.864665	0.7436	10.3372	0.001364	0.0681	0.0259757560
ss65049999	*rs43138756*	8	87.887594	0.0544	3.7419	0.053470	0.0793	0.0081649082
ss61560471	*rs41658990*	8	87.920993	0.2156	3.8459	0.050265	0.0438	0.0147999336
ss65487469	*rs43565278*	8	87.963873	0.2701	0.6914	0.405988	0.0178	0.0070052178
	rs29019444	8	88.011069	0.1311	4.5488	0.033290	0.0581	0.0132247068
ss86289218		8	88.066884	0.1096	0.1901	0.662963	0.0130	0.0025356356
ss65487354	*rs43574961*	8	88.097431	0.2665	7.5180	0.006265	0.0584	0.0228497674
ss61492834	*rs41590582*	8	88.129259	0.9405	1.8361	0.175849	0.0536	0.0059945319
ss61492266	*rs41595520*	8	88.29211	0.5752	4.0036	0.045791	0.0373	0.0182480014
ss117969203		8	88.314772	0.0723	1.3718	0.241905	0.0418	0.0056172781
ss86278195		8	88.52808	0.3968	0.1481	0.700445	0.0073	0.0034931694
ss63869768	*rs41595745*	8	88.551552	0.0895	1.0478	0.306361	0.0320	0.0052104545
ss86341585		8	88.621405	0.3689	0.7510	0.386467	0.0170	0.0079264051
ss63869460	*rs41593792*	8	88.644806	0.2335	0.4049	0.524776	0.0137	0.0049172257
ss86294173		8	88.677969	0.1784	4.3111	0.038230	0.0505	0.0147985213
ss86275207		8	88.775043	0.5115	2.5344	0.111846	0.0291	0.0145342866
ss86304895		8	88.870085	0.2872	0.0454	0.831402	0.0042	0.0017374226
ss61520225	*rs41612254*	8	88.960234	0.3417	0.0194	0.889313	0.0027	0.0012087127
ss86317351		8	88.997318	0.4019	0.6117	0.434412	0.0148	0.0071173121
ss86319610		8	89.035762	0.4979	0.2194	0.639682	0.0087	0.0043614708
ss86289826		8	89.202041	0.2729	2.7898	0.095318	0.0345	0.0137114068
ss86301613		8	89.259276	0.4499	0.4989	0.480217	0.0128	0.0063362358
ss61560489	*rs41659007*	8	89.289325	0.7772	1.1082	0.292846	0.0224	0.0077670730
ss86319872		8	89.317772	0.3281	0.2407	0.623886	0.0096	0.0042484219
ss61544523	*rs41644614*	8	89.397241	0.2593	0.5163	0.472677	0.0152	0.0058453172
ss86292637		8	89.419469	0.6325	0.2797	0.597064	0.0102	0.0047360757
ss86338555		8	89.471684	0.4993	1.8978	0.168765	0.0262	0.0131042591
ss61544520	*rs41644611*	8	89.527371	0.6110	9.9548	0.001673	0.0586	0.0278466578
ss86325384		8	89.548462	0.5996	10.1007	0.001548	0.0583	0.0279772562
ss65425124	*rs43505386*	8	89.57599	0.6003	10.4804	0.001264	0.0598	0.0286935169
ss61537241	*rs41638645*	8	89.637697	0.6153	2.3813	0.123246	0.0295	0.0139644435
ss86291257		8	89.66756	0.3840	2.4947	0.114685	0.0302	0.0142698751
ss65203906	*rs43289386*	8	89.813287	0.4642	0.0655	0.798032	0.0047	0.0023429591
ss65203854	*rs43289334*	8	89.845142	0.3317	1.7366	0.188001	0.0257	0.0113996673
ss86333734		8	89.876678	0.8775	0.8177	0.366154	0.0257	0.0055328984
ss65015343	*rs43107279*	8	90.019529	0.6533	0.0588	0.808499	0.0046	0.0020970584
ss86331786		8	90.088376	0.4470	0.0011	0.973912	0.0006	0.0002995673
ss86337658		8	90.159585	0.1941	14.6854	0.000139	0.0878	0.0274604687

	rs29012807	8	90.183274	0.5616	3.0199	0.082693	0.0316	0.0155676484
ss86340789		8	90.218871	0.5415	3.6008	0.058166	0.0342	0.0170020120
ss86310313		8	90.25372	0.7872	0.0310	0.860397	0.0040	0.0013245919
ss86329138		8	90.282204	0.2113	0.0878	0.767140	0.0067	0.0022206724
ss86339709		8	90.328408	0.2937	0.3380	0.561193	0.0117	0.0048580220
ss61492823	*rs41590571*	8	90.389403	0.4979	6.8958	0.008830	0.0490	0.0245059592
ss86326073		8	90.476779	0.6074	7.7162	0.005620	0.0523	0.0249250661
ss117969325		8	90.609371	0.7851	2.4139	0.120720	0.0358	0.0120968268
ss65487237	*rs43574545*	8	90.906374	0.0408	2.7089	0.100239	0.0764	0.0059854593
ss86341071		8	90.972337	0.8510	3.0757	0.079912	0.0460	0.0116634111
ss86296196		8	90.994683	0.3073	4.1070	0.043086	0.0412	0.0175549490
ss86331790		8	91.017104	0.7027	3.0804	0.079682	0.0358	0.0149558639
ss86321499		8	91.102803	0.0709	18.8137	0.000017	0.1542	0.0203258062
ss61560474	*rs41658993*	8	91.192901	0.1862	9.4080	0.002244	0.0740	0.0224349670
ss65487990	*rs43570798*	8	91.242777	0.9577	2.9384	0.086944	0.0770	0.0062334186
ss86288083		8	91.271994	0.3195	5.6095	0.018136	0.0472	0.0205368696
ss61560485	*rs41659004*	8	91.326289	0.3066	4.9764	0.026014	0.0452	0.0192370226
ss86331039		8	91.373924	0.9570	3.0676	0.080310	0.0781	0.0064275728
ss65488083	*rs43572091*	8	91.407193	0.8424	15.8372	0.000076	0.0996	0.0264447638
ss86335346		8	91.547075	0.6934	31.3034	0.000000	0.1099	0.0467241158
ss65488476	*rs43566673*	8	91.64331	0.3775	31.7420	0.000000	0.1059	0.0497555984
	rs29022959	8	91.719223	0.7213	35.0788	0.000000	0.1190	0.0478369057
ss86299466		8	91.843007	0.9420	0.0113	0.915309	0.0041	0.0004480087
ss117963996		8	91.886013	0.6984	2.1123	0.146567	0.0291	0.0122793344
ss117969197		8	91.91872	0.0595	1.5740	0.210055	0.0490	0.0054789833
ss86302282		8	91.938833	0.0537	1.0075	0.315847	0.0409	0.0041565012
ss61570034	*rs41672080*	8	91.961227	0.5817	16.4119	0.000057	0.0775	0.0376944522
ss64673330	*rs42769314*	8	92.231833	0.2364	10.7645	0.001086	0.0721	0.0260169108
ss86285205		8	92.278364	0.7872	0.0043	0.947587	0.0015	0.0004926499
ss86334238		8	92.306989	0.6648	1.5143	0.218904	0.0242	0.0107728001
ss64511366	*rs42673549*	8	92.496587	0.0888	0.0208	0.885284	0.0048	0.0007796458
ss64511284	*rs42672708*	8	92.538	0.0917	16.8324	0.000046	0.1303	0.0217029724
ss86311905		8	92.71445	0.8589	15.1353	0.000110	0.1030	0.0249703778
ss117969176		8	92.756299	0.0322	0.4853	0.486286	0.0367	0.0022915884
ss86329367		8	92.785737	0.2586	1.8351	0.175969	0.0287	0.0110231343
ss86304229		8	92.806965	0.7679	8.3275	0.004026	0.0632	0.0225259579
ss86299975		8	92.866681	0.7471	10.2749	0.001410	0.0683	0.0258228186
ss117975319		8	92.890008	0.3274	6.7414	0.009619	0.0499	0.0219833701
ss86303680		8	92.922408	0.2292	2.3775	0.123546	0.0329	0.0116106440
ss86296740		8	93.043064	0.7264	10.4841	0.001261	0.0674	0.0267734771
ss86276062		8	93.083408	0.8381	2.5475	0.110922	0.0400	0.0108466429
ss86324743		8	93.104407	0.7106	4.7594	0.029473	0.0438	0.0179953465
	rs29020453	8	93.13934	0.1232	0.0798	0.777613	0.0081	0.0017414740
ss86329653		8	93.193612	0.6225	16.1332	0.000065	0.0759	0.0356517058
ss86329268		8	93.299391	0.5566	11.2643	0.000833	0.0637	0.0314504124
ss86308593		8	93.388859	0.6719	10.3976	0.001321	0.0637	0.0280898412
ss86330963		8	93.433816	0.6332	11.4667	0.000748	0.0671	0.0311461739

ss86292487		8	93.455383	0.2822	10.8419	0.001042	0.0681	0.0275851473
ss86296233		8	93.506859	0.6153	10.7856	0.001074	0.0640	0.0303079435
ss86289399		8	93.539709	0.4764	7.7387	0.005551	0.0526	0.0262432951
ss86328646		8	93.596428	0.8145	0.3770	0.539418	0.0150	0.0045335392
ss61492874	*rs41590622*	8	93.666799	0.1096	2.7644	0.096836	0.0505	0.0098607908
ss117969478		8	93.743415	0.0809	1.2012	0.273466	0.0382	0.0056906148
ss86315954		8	93.766862	0.1999	0.0123	0.911851	0.0027	0.0008733986
ss86329976		8	93.802094	0.8746	0.0804	0.776815	0.0082	0.0018021091
ss86332392		8	93.84192	0.1454	0.0032	0.955111	0.0015	0.0003813809
	rs29021328	8	93.873872	0.7686	2.7040	0.100548	0.0396	0.0141011962
ss86337360		8	93.920979	0.1834	0.9531	0.329273	0.0248	0.0074154788
ss117969408		8	93.945194	0.9090	7.7238	0.005597	0.0915	0.0151281019
	rs29015158	8	93.966442	0.1862	1.8899	0.169653	0.0347	0.0105205339
ss61485142	*rs41584168*	8	94.083838	0.7658	0.0010	0.974295	0.0007	0.0002675222
ss86317601		8	94.119745	0.6576	14.0141	0.000196	0.0750	0.0337947478
ss61562386	*rs41660485*	8	94.231319	0.1039	0.0857	0.769759	0.0091	0.0016923439
ss86309839		8	94.276063	0.9362	0.2894	0.590782	0.0210	0.0025024185
ss117969192		8	94.30851	0.1726	0.8799	0.348549	0.0245	0.0069954785
ss86337053		8	94.332829	0.1655	0.7676	0.381265	0.0231	0.0063790250
ss86284403		8	94.37105	0.1039	0.0266	0.870505	0.0051	0.0009425059
ss86305348		8	94.67003	0.1060	0.2524	0.615579	0.0149	0.0028230558
ss61507648	*rs41608636*	8	94.690856	0.7393	7.6212	0.005921	0.0602	0.0232017945
ss61477748	*rs41579577*	8	94.712098	0.8811	13.1828	0.000303	0.1045	0.0218966963
ss61507649	*rs41608637*	8	94.732858	0.8761	11.6085	0.000694	0.0963	0.0209156355
ss86311668		8	94.972035	0.1053	6.6656	0.010032	0.0750	0.0141296559
ss86339722		8	95.079922	0.2901	0.3688	0.543843	0.0130	0.0053598839
ss86330799		8	95.161539	0.8317	0.1221	0.726869	0.0090	0.0025145111
	rs29019309	8	95.244862	0.8424	1.2889	0.256646	0.0288	0.0076564055
ss61495768	*rs41593435*	8	95.349996	0.1103	0.2085	0.648082	0.0138	0.0027052902
ss61471199	*rs41568170*	8	95.574886	0.7715	1.7914	0.181194	0.0299	0.0105340596
ss86295375		8	95.64291	0.6633	0.7131	0.398706	0.0171	0.0076404030
ss86310175		8	95.681399	0.2328	0.8411	0.359391	0.0210	0.0075077306
ss64229212	*rs42539016*	8	95.773222	0.1576	0.0035	0.952600	0.0016	0.0004159071
ss61560531	*rs41659449*	8	95.825137	0.2070	0.0503	0.822523	0.0052	0.0017204161
ss65489384	*rs43576665*	8	95.855873	0.9384	4.4601	0.035051	0.0822	0.0095045153
ss61560520	*rs41659438*	8	95.898889	0.6855	1.5024	0.220719	0.0258	0.0111149841
ss86293462		8	95.991334	0.8001	2.7722	0.096363	0.0398	0.0127232573
ss86295493		8	96.16335	0.8761	1.2237	0.269018	0.0313	0.0067959146
ss61525274	*rs41619303*	8	96.209636	0.8345	10.4747	0.001268	0.0814	0.0224823995
ss65611916	*rs43694530*	8	96.246616	0.7693	9.7447	0.001873	0.0699	0.0248140967
ss65611881	*rs43694495*	8	96.281583	0.8159	7.1989	0.007468	0.0646	0.0193970689
ss86314482		8	96.335956	0.3682	2.9859	0.084437	0.0345	0.0160615118
ss86317835		8	96.362722	0.8331	10.2947	0.001395	0.0797	0.0221758649
ss61471638	*rs41571787*	8	96.445395	0.1999	4.0165	0.045443	0.0473	0.0151341866
ss63424246	*rs42226565*	8	96.475948	0.2142	11.1275	0.000896	0.0770	0.0259191416
ss86274937		8	96.550119	0.1798	1.1443	0.285106	0.0255	0.0075194836
ss64881152	*rs42976848*	8	96.575603	0.7830	1.1455	0.284868	0.0243	0.0082670834

ss117969283		8	96.822369	0.8660	3.2667	0.071132	0.0486	0.0112789836
ss64802634	*rs42893750*	8	96.988952	0.3059	1.1927	0.275158	0.0228	0.0096706647
ss61512904		8	97.070496	0.2557	12.6208	0.000407	0.0782	0.0297866322
ss61512891	*rs41616289*	8	97.099323	0.7443	12.6208	0.000407	0.0782	0.0297866322
ss86288159		8	97.173064	0.8438	13.9979	0.000198	0.0928	0.0244674832
ss65491024	*rs43573900*	8	97.224196	0.8288	10.1033	0.001546	0.0771	0.0218769567
ss65491183	*rs43575758*	8	97.303757	0.7557	7.2026	0.007453	0.0573	0.0211481008
ss117969435		8	97.335529	0.8818	26.2912	0.000000	0.1417	0.0295363189
ss61560595	*rs41659512*	8	97.419093	0.9563	0.7490	0.387087	0.0397	0.0033202551
ss65491447	*rs43577721*	8	97.47563	0.9556	0.6565	0.418072	0.0369	0.0031343126
ss61504614	*rs41608487*	8	97.542131	0.1640	8.2576	0.004182	0.0707	0.0193915437
ss61560596	*rs41659513*	8	97.573747	0.8324	17.4016	0.000034	0.1022	0.0285138707
ss65491501	*rs43569177*	8	97.601766	0.1426	9.3277	0.002343	0.0783	0.0191336870
ss86284343		8	97.628233	0.2679	4.9433	0.026512	0.0467	0.0183090130
ss86280375		8	97.723569	0.9205	5.8771	0.015593	0.0849	0.0124328135
	rs29025529	8	97.914408	0.1784	2.1966	0.138766	0.0371	0.0108748388
ss64751737	*rs42843871*	8	97.972512	0.7650	8.7439	0.003212	0.0657	0.0236076562
ss61560559	*rs41659477*	8	98.089569	0.1562	16.1411	0.000065	0.1008	0.0265711690
ss65489906	*rs43572784*	8	98.187113	0.8503	2.9009	0.088975	0.0462	0.0117624014
ss86339203		8	98.220777	0.1003	1.0110	0.315008	0.0321	0.0057938672
ss65490312	*rs43576289*	8	98.337255	0.6426	0.3616	0.547838	0.0119	0.0054649830
ss65490322	*rs43576299*	8	98.364672	0.3446	0.9127	0.339742	0.0192	0.0086505782
ss64105989	*rs42473905*	8	98.398932	0.8302	12.3799	0.000462	0.0871	0.0245586182
ss64106039	*rs42473930*	8	98.436793	0.8360	11.1974	0.000863	0.0835	0.0229041172
ss86307193		8	98.509012	0.8209	5.8340	0.015976	0.0574	0.0168704883
ss63930410	*rs42341918*	8	98.656166	0.1125	3.2357	0.072482	0.0508	0.0101503027
ss86282430		8	98.725978	0.3517	0.6370	0.425070	0.0163	0.0074337861
ss86332333		8	98.821682	0.7930	10.3363	0.001365	0.0727	0.0238645563
ss63930900	*rs42345861*	8	98.861573	0.0931	7.3396	0.006911	0.0861	0.0145360459
ss86292045		8	99.059241	0.7163	6.3167	0.012186	0.0527	0.0214042584
ss65490908	*rs43572486*	8	99.362848	0.6397	0.1073	0.743391	0.0066	0.0030274632
ss86336345		8	99.390364	0.7701	4.3262	0.037895	0.0467	0.0165550327
ss61560583		8	99.47233	0.9191	0.3156	0.574462	0.0196	0.0029223355
ss61492868	*rs41590616*	8	99.493647	0.3961	4.8611	0.027795	0.0430	0.0205716667
ss61560573	*rs41659490*	8	99.574157	0.9269	5.1549	0.023486	0.0797	0.0108023836
ss61492867	*rs41590615*	8	99.69923	0.9212	4.3924	0.036460	0.0716	0.0103897974
ss86335502		8	99.794571	0.8625	17.2899	0.000036	0.1096	0.0259954722
ss61520778	*rs41617500*	8	99.89259	0.6812	1.1694	0.279896	0.0225	0.0097751945
ss61511553	*rs41613464*	8	99.961134	0.7471	0.0814	0.775460	0.0060	0.0022765352
ss64789216	*rs42884618*	8	100.005894	0.8460	0.3020	0.582838	0.0142	0.0036996057
ss64267654	*rs42557168*	8	100.125328	0.6669	2.6638	0.103105	0.0328	0.0145542466
ss86338705		8	100.15712	0.3181	3.7855	0.052099	0.0388	0.0168104435
ss63418639	*rs42224398*	8	100.324743	0.8037	1.5607	0.211989	0.0295	0.0092982629
ss86328682		8	100.356204	0.1970	1.6696	0.196734	0.0305	0.0096386473
ss86325310		8	100.395004	0.3510	0.8351	0.361119	0.0184	0.0084027656
ss86340812		8	100.441578	0.3166	0.0214	0.883854	0.0029	0.0012659644
ss63418501	*rs42224352*	8	100.538648	0.2557	2.1769	0.140546	0.0319	0.0121492678

ss63418375	*rs42223810*	8	100.567685	0.2085	0.1934	0.660203	0.0103	0.0033999377
ss61513365	*rs41612047*	8	100.602821	0.9642	0.1304	0.718088	0.0182	0.0012541100
	rs29012580	8	100.628706	0.3367	1.1169	0.290950	0.0216	0.0096302539
ss61513364	*rs41612046*	8	100.799975	0.7815	3.1620	0.075805	0.0408	0.0139272543
ss61513352	*rs41612034*	8	100.850428	0.2722	0.0120	0.912955	0.0022	0.0008913248
ss61470865	*rs41574234*	8	100.878878	0.2572	0.0250	0.874381	0.0033	0.0012631900
ss117969438		8	101.080409	0.3560	0.0768	0.781775	0.0054	0.0024901354
ss86326516		8	101.113861	0.1762	2.8392	0.092436	0.0403	0.0117002429
ss86335776		8	101.223038	0.7264	0.3029	0.582242	0.0112	0.0044593024
	rs29013523	8	101.299086	0.6605	0.5527	0.457483	0.0143	0.0064257036
ss63416905	*rs42224726*	8	101.376846	0.7006	6.1046	0.013721	0.0502	0.0210688611
ss61470862	*rs43745251*	8	101.402046	0.6275	0.7505	0.386631	0.0165	0.0077299967
ss86333090		8	101.424048	0.3804	0.1467	0.701805	0.0073	0.0034465557
ss63416641	*rs42224640*	8	101.452956	0.3689	0.4145	0.519921	0.0123	0.0057058452
ss63416383	*rs42223954*	8	101.493446	0.2650	1.8216	0.177559	0.0277	0.0107842738
ss86328822		8	101.608252	0.6039	1.7421	0.187303	0.0249	0.0119233219
ss61513016	*rs41609616*	8	101.731288	0.6110	2.6858	0.101700	0.0302	0.0143623079
ss86288383		8	101.773529	0.1862	3.0012	0.083644	0.0410	0.0124234132
ss86333400		8	101.90193	0.1511	5.1477	0.023582	0.0587	0.0150632724
ss86319219		8	102.179273	0.3195	0.7077	0.400494	0.0167	0.0072416532
ss61465406	*rs41565755*	8	102.294154	0.7880	4.2783	0.038971	0.0458	0.0152986502
ss86339096		8	102.400086	0.6404	15.5450	0.000089	0.0744	0.0342546409
ss86324961		8	102.44335	0.5681	2.6781	0.102190	0.0301	0.0147644583
ss61525037	*rs41625264*	8	102.483789	0.4585	5.5705	0.018541	0.0427	0.0211829188
ss117969218		8	102.524088	0.9606	2.7034	0.100588	0.0761	0.0057596453
ss61516250	*rs41613367*	8	102.558344	0.4298	4.4286	0.035700	0.0385	0.0188869535
ss86324396		8	102.596583	0.5523	4.8364	0.028194	0.0404	0.0199788020
ss86340300		8	102.634965	0.8510	5.4485	0.019868	0.0583	0.0147766411
ss86339104		8	102.676159	0.3539	0.0656	0.797989	0.0049	0.0022469698
ss86317999		8	102.759095	0.1275	3.9136	0.048292	0.0561	0.0124920673
ss65494982	*rs43580047*	8	102.953014	0.3582	4.6687	0.031059	0.0426	0.0196069624
ss61512469	*rs41612675*	8	103.230022	0.4083	0.0496	0.823793	0.0042	0.0020416868
ss64449184	*rs42644615*	8	103.255798	0.5201	1.9749	0.160374	0.0263	0.0131317551
ss86340628		8	103.280571	0.4864	1.1474	0.284471	0.0201	0.0100260103
ss63819205	*rs42367095*	8	103.319234	0.8668	0.2127	0.644787	0.0125	0.0028775687
	rs29013940	8	103.345432	0.2593	2.4255	0.119827	0.0321	0.0123483727
ss61517675	*rs41618039*	8	103.411294	0.8560	2.2195	0.136731	0.0389	0.0095956388
ss63819303	*rs42368043*	8	103.441523	0.5638	6.4196	0.011505	0.0489	0.0240418713
ss86325641		8	103.489394	0.8130	0.0074	0.931296	0.0020	0.0006132040
ss61472974	*rs41575193*	8	103.552066	0.4721	6.4931	0.011043	0.0480	0.0239295226
ss86312786		8	103.591314	0.3961	9.2014	0.002508	0.0570	0.0272933160
ss86324309		8	103.687465	0.3968	1.6260	0.202689	0.0239	0.0114217920
ss86294332		8	103.758998	0.0981	3.1241	0.077581	0.0539	0.0095497216
ss61472306	*rs41569844*	8	103.797179	0.3030	1.7957	0.180675	0.0273	0.0115369693
ss63784304	*rs42348715*	8	103.890007	0.8739	6.8800	0.008908	0.0722	0.0159062851
ss86309814		8	103.916909	0.3152	0.5621	0.453650	0.0148	0.0063869177
ss86337005		8	103.988832	0.8983	0.0552	0.814361	0.0072	0.0013236908

ss86340282		8	104.010981	0.1010	0.0500	0.823103	0.0069	0.0012553570
ss63784760	*rs42350743*	8	104.05264	0.5315	0.2508	0.616670	0.0091	0.0045138963
ss86327642		8	104.230025	0.5394	0.0026	0.959030	0.0010	0.0004749183
ss61562450	*rs41661749*	8	104.260479	0.8797	1.3446	0.246621	0.0326	0.0069123272
ss64152823	*rs42496573*	8	104.292166	0.2156	2.9806	0.084713	0.0385	0.0130109923
ss86302073		8	104.371406	0.1261	0.0713	0.789543	0.0074	0.0016233816
ss86336829		8	104.403099	0.6784	4.8109	0.028610	0.0437	0.0190844250
ss61562448	*rs41661747*	8	104.4478	0.4076	0.6546	0.418748	0.0153	0.0073760214
ss86294795		8	104.493113	0.6562	1.8072	0.179283	0.0255	0.0115150862
ss86339192		8	104.523917	0.7113	0.0450	0.831979	0.0045	0.0018406534
ss86340085		8	104.587519	0.5831	0.1348	0.713635	0.0069	0.0033586056
ss86327497		8	104.608021	0.6218	6.2773	0.012457	0.0461	0.0216622699
ss86326095		8	104.635073	0.4341	2.0967	0.148069	0.0273	0.0133992792
ss65493890	*rs43578762*	8	104.668054	0.6519	0.2735	0.601156	0.0103	0.0046764185
ss86337936		8	104.697944	0.2199	0.7980	0.371988	0.0203	0.0069655961
ss86287677		8	104.730904	0.7858	2.2535	0.133763	0.0329	0.0110625964
ss86335482		8	104.762992	0.3073	3.6887	0.055189	0.0383	0.0163075644
ss65493667	*rs43577039*	8	104.804277	0.2486	0.0338	0.854283	0.0040	0.0014862957
ss65494382	*rs43583654*	8	104.929794	0.9377	0.0002	0.989291	0.0005	0.0000587271
ss61492934	*rs41590882*	8	104.971777	0.0981	5.3115	0.021478	0.0727	0.0128729762
ss65494456	*rs43583728*	8	105.124101	0.7149	0.6456	0.421973	0.0165	0.0067141203
ss117969391		8	105.305234	0.1311	0.8829	0.347722	0.0259	0.0059113669
ss65494659	*rs43577131*	8	105.337809	0.4341	0.2607	0.609789	0.0097	0.0047556917
ss86281996		8	105.375322	0.3911	0.2711	0.602760	0.0099	0.0047179150
ss117969348		8	105.482166	0.6999	1.8724	0.171644	0.0279	0.0117334141
ss65495239	*rs43581982*	8	105.500395	0.3560	3.2632	0.071281	0.0343	0.0157214573
ss65495275	*rs43582018*	8	105.528014	0.1640	3.3356	0.068225	0.0453	0.0124236173
ss86287548		8	105.556801	0.4434	4.2698	0.039166	0.0391	0.0192777374
ss65495353	*rs43582896*	8	105.592833	0.1218	0.6668	0.414460	0.0236	0.0050408462
ss65495382	*rs43582925*	8	105.64384	0.4857	7.8146	0.005325	0.0502	0.0250564768
ss61492951	*rs41590899*	8	105.855351	0.3883	8.2026	0.004309	0.0575	0.0273376144
ss65495576	*rs43575220*	8	105.879483	0.8854	2.1428	0.143693	0.0429	0.0087148740
ss86295239		8	105.928163	0.4663	9.7783	0.001839	0.0563	0.0280244809
ss65016009	*rs43105327*	8	106.001747	0.5415	1.0397	0.308236	0.0196	0.0097113547
ss86284158		8	106.047649	0.2249	1.2104	0.271625	0.0248	0.0086363320
ss86293942		8	106.093282	0.3567	0.0341	0.853592	0.0035	0.0016177601
ss61492955	*rs41590903*	8	106.122453	0.7572	1.2410	0.265667	0.0243	0.0089406093
ss86323867		8	106.178471	0.1232	1.4440	0.229897	0.0349	0.0075347233
ss86335665		8	106.207583	0.5093	0.2516	0.616081	0.0092	0.0045899091
ss86293494		8	106.236862	0.5430	3.0244	0.082462	0.0315	0.0156222971
ss117969365		8	106.259905	0.8840	1.0257	0.311514	0.0296	0.0060667203
ss65495983	*rs43577427*	8	106.285042	0.4241	1.8611	0.172940	0.0266	0.0129966180
ss61472420	*rs41570855*	8	106.309441	0.3875	1.0007	0.317477	0.0190	0.0090340902
ss64642362	*rs42746477*	8	106.33634	0.4592	0.3934	0.530706	0.0115	0.0057351235
ss64642405	*rs42746518*	8	106.370665	0.8983	0.8234	0.364496	0.0277	0.0050549897
ss65494297	*rs43582569*	8	106.398881	0.1010	0.8491	0.357132	0.0282	0.0051129445
ss61492932	*rs41590880*	8	106.429907	0.1891	0.1305	0.717990	0.0085	0.0026096440

ss86287930		8	106.474144	0.3653	4.8803	0.027489	0.0424	0.0196636122
ss86308070		8	106.507123	0.6297	0.5396	0.462845	0.0138	0.0064301169
ss117969466		8	106.562097	0.1633	15.3554	0.000098	0.0941	0.0257303018
ss61510967	*rs41609496*	8	106.58864	0.5809	6.0462	0.014178	0.0459	0.0223394070
	rs29010990	8	106.616133	0.4635	0.7664	0.381640	0.0159	0.0079149356
ss61562846	*rs41665936*	8	106.6486	0.4613	18.0217	0.000025	0.0744	0.0369593789
ss86332174		8	106.675517	0.7314	6.3470	0.011981	0.0522	0.0205178402
ss61562850	*rs41665939*	8	106.73224	0.2779	1.8918	0.169442	0.0281	0.0112847486
ss86287146		8	106.765654	0.2908	12.2189	0.000503	0.0711	0.0293412903
ss61562842	*rs41665932*	8	106.821827	0.5924	25.4650	0.000001	0.0899	0.0434305240
ss61562852	*rs41665941*	8	106.875288	0.5881	22.9959	0.000002	0.0856	0.0414739343
	rs29016547	8	106.955033	0.3625	1.8498	0.174250	0.0265	0.0122396492
ss117969394		8	106.996524	0.1024	8.3136	0.004056	0.0887	0.0163063261
ss86292492		8	107.149026	0.8080	4.1551	0.041886	0.0478	0.0148324727
ss61492966	*rs41590914*	8	107.194011	0.5673	0.0127	0.910398	0.0021	0.0010355467
ss86311729		8	107.52903	0.0824	11.3265	0.000806	0.1078	0.0163042177
ss61560639	*rs41660356*	8	107.599216	0.9062	1.7096	0.191474	0.0413	0.0070250866
ss117964020		8	107.781298	0.7056	3.3227	0.068760	0.0360	0.0149693845
ss38328464	*rs29020774*	8	107.84054	0.3825	1.6565	0.198508	0.0249	0.0117669537
ss38329339	*rs29026953*	8	107.893512	0.7987	12.4787	0.000439	0.0799	0.0257023952
ss86333757		8	107.959582	0.2471	6.3151	0.012197	0.0543	0.0202022258
	rs29009571	8	108.003058	0.3116	0.8847	0.347241	0.0188	0.0080617608
ss86338610		8	108.035533	0.5337	1.6678	0.196978	0.0245	0.0121917999
ss86292417		8	108.127201	0.7092	10.6555	0.001151	0.0647	0.0266755434
ss65492355	*rs43573630*	8	108.181738	0.2192	0.3657	0.545547	0.0133	0.0045604690
ss86310163		8	108.209372	0.2092	0.0006	0.980075	0.0006	0.0001847601
ss86280411		8	108.230989	0.2744	1.0113	0.314945	0.0210	0.0083761287
ss86323386		8	108.304424	0.7228	15.2049	0.000106	0.0796	0.0318869753
ss61469361	*rs41569468*	8	108.34991	0.0652	0.2985	0.584974	0.0208	0.0025391621
ss86337094		8	108.397581	0.7758	5.9066	0.015336	0.0533	0.0185455956
ss61492973	*rs41590921*	8	108.44317	0.6576	0.0883	0.766399	0.0057	0.0025466124
ss61560740	*rs41661149*	8	108.476756	0.3481	0.0430	0.835782	0.0039	0.0017658302
ss86303445		8	108.498328	0.4234	15.4930	0.000091	0.0693	0.0338410355
ss65496502	*rs43575246*	8	108.561158	0.5953	1.5318	0.216263	0.0220	0.0105838614
ss86336543		8	108.585264	0.5158	1.6947	0.193416	0.0233	0.0116214588
ss86327283		8	108.647941	0.1862	7.5950	0.006006	0.0654	0.0198099418
ss86295489		8	108.742131	0.5494	9.9249	0.001700	0.0561	0.0277990532
ss117969442		8	108.801252	0.6418	0.3540	0.552064	0.0115	0.0053022508
ss86306081		8	108.856282	0.9191	1.0441	0.307231	0.0334	0.0049693885
ss86311763		8	108.926359	0.8754	1.0499	0.305880	0.0285	0.0062263877
ss86335572		8	108.961259	0.5709	1.2598	0.262075	0.0209	0.0102540880
ss86290287		8	109.075882	0.7715	3.4503	0.063663	0.0409	0.0144243798
ss61560409	*rs41658329*	8	109.124106	0.3689	1.1240	0.289426	0.0198	0.0092322535
ss86339777		8	109.152363	0.0745	5.0192	0.025382	0.0773	0.0106581544
ss61560408	*rs41658328*	8	109.184354	0.5759	0.9296	0.335299	0.0187	0.0091316449
ss86341294		8	109.235771	0.1705	10.0561	0.001585	0.0769	0.0217406271
ss86290804		8	109.332921	0.5609	1.6677	0.196990	0.0242	0.0119178696

ss65484851	*rs43568167*	8	109.388915	0.8489	0.0366	0.848412		0.0050	0.0012765519
ss86295839		8	109.418038	0.3324	2.5485	0.110853		0.0323	0.0143270224
ss65484638	*rs43565954*	8	109.445776	0.1741	8.9408	0.002887		0.0719	0.0206738569
ss64969429	*rs43063159*	8	109.509384	0.2135	0.6052	0.436859		0.0175	0.0058677330
ss64988289	*rs43078546*	8	109.571442	0.4971	0.5004	0.479545		0.0134	0.0067063055
ss86296913		8	109.595286	0.7285	0.1884	0.664394		0.0090	0.0035657968
ss65496849	*rs43576983*	8	109.624284	0.4191	0.3332	0.563990		0.0109	0.0052910667
ss86330409		8	109.64968	0.8782	0.0845	0.771401		0.0080	0.0017070986
ss61522153	*rs41617656*	8	109.669849	0.5509	0.0258	0.872402		0.0030	0.0014736887
ss117969469		8	109.81433	0.4577	1.2480	0.264327		0.0206	0.0102325852
ss86282233		8	109.835729	0.7987	10.4872	0.001259		0.0737	0.0236831408
ss86312342		8	109.884887	0.2586	9.6376	0.001984		0.0648	0.0248469901
ss86282603		8	109.914268	0.4993	0.4726	0.492010		0.0125	0.0062430632
ss63913254	*rs42176692*	8	109.941933	0.8825	0.4245	0.514892		0.0185	0.0038301779
ss61526946	*rs41627216*	8	109.986045	0.6519	5.9334	0.015107		0.0473	0.0214686643
	rs29022784	8	110.029561	0.3266	4.5588	0.033099		0.0418	0.0184049184
ss63563344	*rs42269970*	8	110.073135	0.5394	0.0015	0.969491		0.0007	0.0003509043
ss86323073		8	110.114254	0.3259	5.9957	0.014587		0.0457	0.0200782534
ss86300074		8	110.205022	0.0981	1.5977	0.206646		0.0378	0.0066844124
ss63562858	*rs42273605*	8	110.23958	0.5910	1.5747	0.209944		0.0231	0.0111762144
ss63562627	*rs42273128*	8	110.346952	0.4979	0.1688	0.681324		0.0076	0.0038043480
ss63562522	*rs42273093*	8	110.374473	0.7486	5.1509	0.023539		0.0492	0.0185389831
ss61563760	*rs41663429*	8	110.411506	0.4979	12.8364	0.000364		0.0643	0.0321423552
ss61563752	*rs41663421*	8	110.481515	0.3446	1.8122	0.178678		0.0262	0.0118416326
ss61469563	*rs41571162*	8	110.541819	0.3718	4.4864	0.034519		0.0401	0.0187105574
ss38329754	*rs29027367*	8	110.754362	0.1798	0.0877	0.767242		0.0073	0.0021466289
ss61492993	*rs41591740*	8	110.822636	0.6984	5.9932	0.014607		0.0507	0.0213442665
ss86295192		8	110.850662	0.5451	0.0330	0.855985		0.0033	0.0016394597
ss86308369		8	110.879683	0.7385	10.4991	0.001251		0.0682	0.0263344109
ss86300472		8	110.931437	0.1913	10.6172	0.001175		0.0790	0.0244274810
ss86340482		8	110.975153	0.3553	3.2604	0.071403		0.0343	0.0157148758
ss86326886		8	111.049092	0.3016	0.0000	0.998718		0.0000	0.0000131586
ss86281672		8	111.091223	0.1934	2.7642	0.096848		0.0388	0.0121206564
ss86339930		8	111.150638	0.6734	0.0655	0.798007		0.0050	0.0021866522
	rs29025196	8	111.188461	0.5079	1.2638	0.261313		0.0209	0.0104432821
ss61560750	*rs41661159*	8	111.22493	0.4642	1.6524	0.199064		0.0239	0.0118861986
ss86307761		8	111.259654	0.6461	0.7656	0.381887		0.0165	0.0075338485
ss86308454		8	111.309165	0.7113	7.3483	0.006878		0.0534	0.0219297090
ss86300890		8	111.357535	0.2801	6.9956	0.008355		0.0528	0.0213079174
ss61523746	*rs41622905*	8	111.423183	0.7135	6.8303	0.009156		0.0516	0.0211086888
	rs29018510	8	111.454943	0.2572	7.1263	0.007773		0.0552	0.0210885419
ss86320441		8	111.510008	0.2672	8.1561	0.004420		0.0580	0.0227307025
ss86326105		8	111.584405	0.1676	0.2200	0.639186		0.0117	0.0032693007
ss86301995		8	111.649385	0.4348	0.8796	0.348623		0.0175	0.0086256606
ss86336117		8	111.689122	0.3116	3.3481	0.067712		0.0369	0.0158171112
ss61560775	*rs41661184*	8	111.819523	0.7966	0.4932	0.482746		0.0163	0.0052761754
ss86294760		8	111.857107	0.1927	0.2498	0.617360		0.0118	0.0036820415

ss61560795	*rs41661204*	8	111.887854	0.8295	0.3571	0.550295		0.0146	0.0041268856
ss86329452		8	111.943405	0.8797	0.0054	0.941637		0.0020	0.0004334106
ss61560805	*rs41661214*	8	112.037401	0.8883	0.0476	0.827440		0.0063	0.0012544608
ss86322587		8	112.12674	0.1977	3.3046	0.069517		0.0432	0.0136930751
ss86300119		8	112.158654	0.5258	0.0755	0.783621		0.0050	0.0024914406
ss61523747	*rs41622906*	8	112.213316	0.3732	12.2405	0.000497		0.0667	0.0312283945
ss61493006	*rs41591753*	8	112.255431	0.6948	6.3493	0.011966		0.0491	0.0208356729
ss64875833	*rs42968613*	8	112.304729	0.6160	0.4689	0.493726		0.0127	0.0059849496
ss61510979	*rs41609508*	8	112.330368	0.3840	0.4689	0.493726		0.0127	0.0059849496
ss64960232	*rs43053569*	8	112.370482	0.4226	6.7721	0.009457		0.0465	0.0226698222
ss86285961		8	112.417235	0.5931	1.0215	0.312512		0.0193	0.0093175023
ss65498575	*rs43578102*	8	112.443479	0.8639	0.1244	0.724444		0.0093	0.0021880239
ss86292434		8	112.466825	0.1483	0.8009	0.371137		0.0234	0.0059060318
ss86327351		8	112.533635	0.2701	4.9865	0.025863		0.0464	0.0182900390
ss61560816	*rs41661224*	8	112.573868	0.7958	0.6215	0.430741		0.0179	0.0058285993
	rs29023511	8	112.655069	0.8739	8.1789	0.004365		0.0776	0.0171085182
	rs29021038	8	112.707691	0.8639	13.4504	0.000264		0.0961	0.0226084882
ss65498267	*rs43583896*	8	112.756816	0.3073	7.5045	0.006312		0.0547	0.0232820118
ss61560812	*rs41661220*	8	112.816509	0.7593	0.0025	0.960030		0.0011	0.0003886073
ss86274389		8	112.853658	0.8524	0.1443	0.704206		0.0099	0.0024850587
ss61510981	*rs41609510*	8	112.917792	0.7772	5.3271	0.021289		0.0518	0.0179417961
ss64430798	*rs42633795*	8	113.075384	0.5860	0.7393	0.390192		0.0159	0.0076964373
ss64430678	*rs42633736*	8	113.161807	0.6447	0.0618	0.803774		0.0049	0.0022466912
ss117969416		8	113.240716	0.7973	1.8791	0.170875		0.0331	0.0107097366
ss86320260		8	113.269307	0.1096	1.1391	0.286204		0.0311	0.0060649228
ss61470355	*rs41569342*	8	113.327413	0.1082	1.4342	0.231483		0.0350	0.0067530045
ss86293799		8	113.404155	0.7113	3.6355	0.056971		0.0390	0.0160122735
ss61560818	*rs41661226*	8	113.425734	0.9205	3.1798	0.074991		0.0602	0.0088135780
ss61555149	*rs41651685*	8	113.602114	0.6117	4.7133	0.030267		0.0411	0.0195241087
ss65554855	*rs43639693*	8	113.626198	0.0910	0.0092	0.923426		0.0032	0.0005216622
ss65554940	*rs43640778*	8	113.653185	0.5623	5.1357	0.023745		0.0418	0.0205679788
ss86275496		8	113.94358	0.2049	1.4850	0.223399		0.0288	0.0093768329
	rs29018745	8	114.018977	0.2744	1.1692	0.279945		0.0221	0.0088095350
ss61493012	*rs41591759*	8	114.063991	0.4062	1.5560	0.212676		0.0231	0.0111365534
ss86284947		8	114.098893	0.2335	2.5881	0.108125		0.0354	0.0126871382
ss86338337		8	114.134677	0.0824	0.7829	0.376562		0.0301	0.0045553881
ss65498862	*rs43581187*	8	114.187268	0.3625	0.0118	0.913651		0.0021	0.0009553048
ss86331453		8	114.215199	0.3460	0.2635	0.607915		0.0099	0.0044956201
ss86288610		8	114.24569	0.6741	0.5764	0.447975		0.0149	0.0065344322
ss86323270		8	114.396189	0.2787	0.7157	0.397859		0.0171	0.0068791387
ss86339704		8	114.439914	0.5107	0.0107	0.917632		0.0019	0.0009423537
ss61470056	*rs41567053*	8	114.479725	0.6182	2.0719	0.150483		0.0283	0.0133692748
ss117969309		8	114.502352	0.8381	0.0277	0.867917		0.0043	0.0011758594
ss61560840	*rs41661948*	8	114.536206	0.2342	0.9825	0.321925		0.0219	0.0078512376
ss65499158	*rs43584582*	8	114.575716	0.4162	0.1036	0.747673		0.0059	0.0028469738
ss61493017	*rs41591764*	8	114.621587	0.6984	0.2307	0.631172		0.0096	0.0040294514
ss65499189	*rs43584613*	8	114.655302	0.1741	0.8226	0.364736		0.0218	0.0062803068

ss61493021	*rs41591768*	8	114.686541	0.3875	3.3047	0.069512		0.0339	0.0160723146
ss86325041		8	114.76053	0.5172	3.3163	0.069027		0.0340	0.0169834220
ss86335989		8	114.908338	0.9155	3.4142	0.065062		0.0590	0.0091342403
ss117969411		8	115.053579	0.5165	0.1218	0.727150		0.0064	0.0032198164
ss61569395	*rs41667176*	8	115.118244	0.5666	0.5636	0.453079		0.0139	0.0068442198
ss86308191		8	115.141165	0.3023	4.5386	0.033489		0.0420	0.0176962605
ss86323461		8	115.163584	0.5910	0.6043	0.437204		0.0148	0.0071696551
ss86311804		8	115.193809	0.5967	2.0746	0.150216		0.0282	0.0135691589
ss61497173	*rs41594603*	8	115.218286	0.8123	1.0951	0.295711		0.0250	0.0076314013
ss86338589		8	115.238775	0.6798	0.0145	0.904030		0.0024	0.0010639317
ss86313951		8	115.280017	0.7256	0.1103	0.739959		0.0069	0.0027477701
ss86287117		8	115.326933	0.4713	0.0744	0.785151		0.0050	0.0025001112
ss86306974		8	115.358974	0.1354	2.2182	0.136843		0.0395	0.0092436660
ss65500186	*rs43584514*	8	115.394575	0.6354	0.1996	0.655170		0.0086	0.0039664154
ss117969447		8	115.426853	0.3976	0.2176	0.641025		0.0085	0.0040864358
ss86333704		8	115.460044	0.6239	1.1922	0.275262		0.0204	0.0095652057
ss86307454		8	115.480193	0.0817	0.1996	0.655207		0.0156	0.0023346371
ss65499872	*rs43581101*	8	115.568454	0.2536	0.1217	0.727326		0.0074	0.0027972006
ss86275977		8	115.607566	0.5451	6.3603	0.011892		0.0470	0.0232888361
ss86337282		8	115.629566	0.4864	6.2214	0.012853		0.0462	0.0230973273
ss86273575		8	115.656012	0.1132	4.2919	0.038662		0.0618	0.0124085098
ss86340759		8	115.680785	0.3331	0.8353	0.361051		0.0178	0.0079176596
ss86336722		8	115.705957	0.5064	0.9195	0.337933		0.0174	0.0087232406
ss86340817		8	115.739788	0.6734	0.7051	0.401377		0.0171	0.0075257598
ss61560865	*rs41661973*	8	115.77359	0.3775	0.0023	0.961396		0.0009	0.0004305810
ss86295926		8	115.809532	0.6354	0.1776	0.673577		0.0080	0.0036918573
ss86328954		8	115.831428	0.5745	0.8623	0.353428		0.0172	0.0084023898
ss86329079		8	115.898072	0.8309	3.6385	0.056870		0.0472	0.0132638319
	rs29023047	8	115.941563	0.6218	1.5469	0.214013		0.0234	0.0110194771
ss64066577	*rs42459035*	8	115.96763	0.0451	6.3866	0.011719		0.1121	0.0096606499
ss61529557	*rs41626273*	8	115.992777	0.7507	4.2917	0.038667		0.0425	0.0159055281
ss117963999		8	116.028293	0.4484	0.0015	0.968820		0.0007	0.0003562742
ss86285664		8	116.106105	0.6669	3.9785	0.046474		0.0391	0.0173714853
ss61529552	*rs41626268*	8	116.180625	0.2092	2.1948	0.138931		0.0340	0.0112527464
ss64151821	*rs42498573*	8	116.218714	0.1562	2.1442	0.143563		0.0383	0.0101050108
ss86305169		8	116.25807	0.4936	0.6922	0.405716		0.0150	0.0075119653
ss86328642		8	116.293952	0.2693	2.0003	0.157717		0.0302	0.0118871888
ss86338099		8	116.329311	0.4427	1.4453	0.229695		0.0219	0.0107884570
ss86319291		8	116.363892	0.2328	1.1014	0.294328		0.0228	0.0081358402
ss86339540		8	116.391888	0.1755	1.0229	0.312184		0.0238	0.0068815682
ss86286836		8	116.415153	0.4534	8.8442	0.003042		0.0539	0.0267332105
ss86322354		8	116.485213	0.4277	0.0342	0.853344		0.0035	0.0017022655
ss86316698		8	116.558079	0.2887	0.9251	0.336480		0.0193	0.0079339574
ss86339990		8	116.588716	0.7034	0.2749	0.600198		0.0105	0.0043761212
ss86303486		8	116.614482	0.6461	7.0202	0.008243		0.0509	0.0232758133
ss61507123	*rs41602423*	8	116.635526	0.2808	1.5177	0.218391		0.0251	0.0101563348
ss86301367		8	116.664295	0.5645	3.1090	0.078299		0.0324	0.0159372523

ss86307964		8	116.705008	0.8840	4.9294	0.026725	0.0644	0.0132062104
ss86293010		8	116.736799	0.2715	1.8867	0.170013	0.0279	0.0110278880
ss86320686		8	116.770992	0.0673	0.0348	0.852062	0.0069	0.0008707066
ss86293870		8	116.795449	0.3625	6.3255	0.012126	0.0477	0.0220335132
ss86335059		8	116.888811	0.7607	0.0254	0.873305	0.0034	0.0012510688
ss86318477		8	116.913131	0.6576	2.3798	0.123367	0.0294	0.0132320109
ss65500769	*rs43581378*	8	116.938581	0.0652	6.6980	0.009853	0.0971	0.0118286010
ss86310026		9	0.025	0.5193	0.1730	0.677629	0.0078	0.0038722809
ss117969763		9	0.108513	0.3424	1.3672	0.242691	0.0234	0.0105312939
ss63596568	*rs42280884*	9	0.152679	0.0831	0.0483	0.826170	0.0075	0.0011468258
	rs29010931	9	0.247512	0.7787	0.5424	0.461680	0.0164	0.0056363032
ss86308512		9	0.28005	0.4792	0.0138	0.906514	0.0022	0.0010864350
ss63596523	*rs42280869*	9	0.306393	0.4850	0.0260	0.871954	0.0030	0.0014987753
ss63596451	*rs42280845*	9	0.330468	0.4728	0.8219	0.364936	0.0170	0.0084556190
ss63596418	*rs42280834*	9	0.374225	0.4642	0.2179	0.640787	0.0087	0.0043429155
ss86336358		9	0.408418	0.7479	0.6178	0.432144	0.0173	0.0065179723
ss61471001	*rs41566574*	9	0.530111	0.5838	3.6105	0.057830	0.0361	0.0175187153
ss61513628	*rs41613996*	9	0.576263	0.8431	1.5538	0.212993	0.0322	0.0085120163
ss61471004	*rs41566577*	9	0.608084	0.6189	4.5883	0.032537	0.0410	0.0193452591
ss63596193	*rs42279959*	9	0.733698	0.5666	4.3770	0.036790	0.0397	0.0194962824
ss64973460	*rs43070064*	9	0.833818	0.3181	1.8910	0.169530	0.0282	0.0122162938
ss65501607	*rs43579416*	9	0.867971	0.8625	3.5317	0.060625	0.0511	0.0121299966
ss65501485	*rs43588693*	9	0.900895	0.8954	6.0082	0.014484	0.0733	0.0137308227
ss61531707	*rs41629259*	9	1.053431	0.4305	2.1374	0.144199	0.0283	0.0138958360
ss61469453	*rs41570360*	9	1.092162	0.9441	0.4963	0.481363	0.0286	0.0030200516
ss65501253	*rs43586861*	9	1.116052	0.0974	2.0273	0.154941	0.0451	0.0079307641
ss86334406		9	1.137332	0.2772	0.6067	0.436310	0.0163	0.0065151524
ss61521642	*rs41620944*	9	1.16568	0.3603	0.3697	0.543362	0.0119	0.0054978137
ss64770371	*rs42863905*	9	1.203864	0.2629	3.8897	0.048979	0.0421	0.0163104678
ss65086742	*rs43173666*	9	1.399219	0.7894	1.7238	0.189634	0.0294	0.0097867931
ss64854096	*rs42952059*	9	1.463199	0.5788	0.2987	0.584863	0.0106	0.0051608948
ss64146661	*rs42500815*	9	1.5985	0.2794	0.1027	0.748712	0.0068	0.0027190503
ss61566545	*rs41665373*	9	1.687717	0.0910	1.0488	0.306131	0.0333	0.0055014985
ss61528872	*rs41629590*	9	1.74445	0.4649	1.7755	0.183142	0.0244	0.0121546707
ss86335062		9	1.779515	0.2715	2.3978	0.121961	0.0335	0.0132497293
ss64544344	*rs42695682*	9	1.843118	0.2751	0.1602	0.689120	0.0084	0.0033456065
ss64544610	*rs42687919*	9	1.874733	0.3338	1.1201	0.290269	0.0207	0.0092268215
ss64544284	*rs42695652*	9	1.901798	0.7264	0.2473	0.619109	0.0105	0.0041557464
ss65501924	*rs43582995*	9	1.977918	0.1805	0.1352	0.713176	0.0088	0.0026024455
ss86278157		9	2.021169	0.1454	7.1367	0.007729	0.0699	0.0173836580
ss86289903		9	2.044971	0.1827	4.0040	0.045780	0.0482	0.0143870043
ss65501901	*rs43582972*	9	2.088959	0.5129	2.5307	0.112102	0.0303	0.0151335945
ss65501864	*rs43582937*	9	2.137653	0.2779	1.5731	0.210176	0.0256	0.0102878050
ss65501733	*rs43581241*	9	2.194846	0.7529	0.0210	0.884719	0.0030	0.0011022265
ss86324783		9	2.238435	0.8968	1.0409	0.307973	0.0317	0.0058744778
ss117975297		9	2.289237	0.2507	0.4445	0.505198	0.0135	0.0050778927
ss64715715	*rs42815263*	9	2.365513	0.2027	0.0597	0.807075	0.0054	0.0017555614

ss64933408	*rs43024134*	9	2.433494	0.3317	1.9440	0.163678	0.0274	0.0121278846
ss86312448		9	2.590067	0.3216	0.2646	0.607118	0.0105	0.0045726772
ss86314801		9	2.619224	0.3739	0.0820	0.774688	0.0056	0.0026175479
ss86285011		9	2.733562	0.4663	1.2575	0.262508	0.0211	0.0104987933
ss86339613		9	2.757961	0.9148	2.1271	0.145169	0.0481	0.0074966145
ss86298686		9	2.798929	0.0716	0.1207	0.728345	0.0125	0.0016579840
ss117974861		9	2.918894	0.9054	3.7148	0.054339	0.0602	0.0103031311
ss61511190	*rs41610313*	9	3.139316	0.5688	1.0358	0.309144	0.0193	0.0094879208
ss61526246	*rs41622548*	9	3.215142	0.7529	0.0578	0.810022	0.0054	0.0019983543
ss61561144	*rs41657557*	9	3.339189	0.6132	0.7677	0.381233	0.0172	0.0081455411
ss61561130	*rs41657543*	9	3.407305	0.5036	0.0027	0.958397	0.0010	0.0004833414
ss61504730	*rs41609202*	9	3.446353	0.3539	0.1143	0.735380	0.0067	0.0030567877
ss61566978	*rs41669889*	9	3.476846	0.7192	1.0131	0.314522	0.0213	0.0085939913
ss61563932	*rs41665096*	9	3.52379	0.5752	0.0493	0.824438	0.0042	0.0020681298
ss86286662		9	3.546077	0.2808	1.0131	0.314522	0.0213	0.0085939913
ss61569698	*rs41669562*	9	3.628463	0.3130	0.0596	0.807233	0.0050	0.0021434226
ss64371063	*rs42607660*	9	3.717781	0.4362	1.0846	0.298041	0.0198	0.0097275179
ss61496807	*rs41592752*	9	3.758052	0.1705	3.5269	0.060799	0.0462	0.0130745508
ss64371315	*rs42608386*	9	3.789411	0.1368	7.6047	0.005974	0.0727	0.0171678230
ss61568568	*rs41665257*	9	3.823908	0.6633	0.9417	0.332172	0.0190	0.0084806172
ss86305088		9	3.899172	0.2851	6.8641	0.008986	0.0544	0.0221646779
ss86319531		9	3.93265	0.7436	1.3174	0.251448	0.0246	0.0093808188
ss86304314		9	3.969849	0.8510	9.6640	0.001956	0.0796	0.0201961181
ss65006830	*rs43099374*	9	4.091763	0.5946	4.6900	0.030678	0.0409	0.0197208706
ss64713962	*rs42808258*	9	4.183032	0.4047	4.6516	0.031366	0.0408	0.0196690930
ss65502480	*rs43588949*	9	4.214968	0.9470	1.3953	0.237913	0.0491	0.0049277287
ss65502446	*rs43588215*	9	4.238169	0.5193	0.0004	0.983131	0.0004	0.0001968726
ss65502393	*rs43588162*	9	4.288026	0.7822	0.0836	0.772554	0.0066	0.0022421790
ss61561247	*rs41658659*	9	4.366424	0.7543	4.5867	0.032567	0.0460	0.0170492433
ss61561237	*rs41658649*	9	4.40812	0.8617	0.8784	0.348973	0.0249	0.0059378566
	rs29026928	9	4.448395	0.1067	1.1475	0.284454	0.0321	0.0061282879
ss65502067	*rs43584938*	9	4.470488	0.8381	0.9253	0.336414	0.0237	0.0064383228
ss64404512	*rs42618695*	9	4.507582	0.1762	0.8583	0.354543	0.0222	0.0064326934
ss64404426	*rs42618652*	9	4.529731	0.0989	5.0490	0.024953	0.0670	0.0119344177
ss117969603		9	4.613989	0.4398	3.1834	0.074826	0.0327	0.0161209354
ss38335041	*rs29024933*	9	4.685719	0.7937	0.3483	0.555273	0.0132	0.0043183888
ss86332686		9	4.862737	0.3152	2.9895	0.084251	0.0338	0.0146051290
ss64817542	*rs42913444*	9	4.905205	0.9155	5.1395	0.023693	0.0728	0.0112737646
ss64817486	*rs42912588*	9	4.927069	0.0731	3.6472	0.056575	0.0659	0.0089284111
ss64433662	*rs42635314*	9	5.105861	0.1447	0.0207	0.885748	0.0037	0.0009052844
ss86328285		9	5.160585	0.6160	3.8455	0.050278	0.0358	0.0169464942
ss64253440	*rs42553043*	9	5.214444	0.4606	0.8025	0.370665	0.0167	0.0082927567
ss61566027	*rs41659674*	9	5.251549	0.5415	0.6463	0.421705	0.0149	0.0074083575
ss64253000	*rs42550223*	9	5.301216	0.0659	0.0216	0.883157	0.0056	0.0006873749
ss86298166		9	5.531752	0.2113	0.3714	0.542438	0.0143	0.0047554429
ss86339859		9	5.560086	0.1612	0.0002	0.990177	0.0003	0.0000858560
ss63759422	*rs42343404*	9	5.58155	0.8660	1.2570	0.262600	0.0309	0.0071787903

ss86332216		9	5.936329	0.8474	1.4458	0.229605	0.0308	0.0079775908
ss61495328	*rs41595904*	9	5.992606	0.6010	0.2110	0.646146	0.0085	0.0040979574
	rs29018260	9	6.017778	0.7908	0.0531	0.817851	0.0053	0.0017538436
ss61565563	*rs41661610*	9	6.049114	0.7894	0.0187	0.891289	0.0031	0.0010451523
ss86336391		9	6.077234	0.7980	0.0508	0.821781	0.0053	0.0017096879
ss86287885		9	6.105143	0.1648	0.0488	0.825143	0.0055	0.0015104261
ss86335198		9	6.195064	0.8553	6.8523	0.009045	0.0690	0.0170800411
ss86283704		9	6.415255	0.8968	0.0089	0.924793	0.0029	0.0005437900
ss86285016		9	6.776175	0.1948	0.0227	0.880330	0.0034	0.0010743135
ss86284443		9	6.819418	0.6777	0.2760	0.599485	0.0103	0.0045075961
ss86309279		9	7.101034	0.9062	0.0612	0.804658	0.0079	0.0013417864
ss86317659		9	7.234951	0.2765	1.4774	0.224593	0.0246	0.0098330472
ss86311511		9	7.26312	0.3539	4.8189	0.028479	0.0433	0.0198200593
ss61466873	*rs41573413*	9	7.324516	0.3059	3.8774	0.049335	0.0396	0.0168235806
ss61529788	*rs41628395*	9	7.382705	0.3202	0.0400	0.841468	0.0041	0.0017940749
ss61529785	*rs41628392*	9	7.408657	0.5129	0.0033	0.953959	0.0011	0.0005297613
ss61561349	*rs41659759*	9	7.434969	0.8281	1.3625	0.243510	0.0278	0.0079216536
ss61475416	*rs41574495*	9	7.504603	0.4742	1.9547	0.162529	0.0266	0.0132480017
ss86274572		9	7.524637	0.5573	0.4287	0.512863	0.0123	0.0060794270
ss86284631		9	7.546237	0.3324	0.6062	0.436483	0.0154	0.0068523116
ss86338207		9	7.60014	0.2213	0.0033	0.954396	0.0013	0.0004540499
ss86340529		9	7.626581	0.8897	0.1236	0.725310	0.0102	0.0019945985
ss86300375		9	7.67469	0.7271	2.2885	0.130789	0.0314	0.0124603239
ss61561397	*rs41659807*	9	7.704447	0.9126	10.3682	0.001342	0.1028	0.0163982190
ss61561422	*rs41661032*	9	7.727244	0.2328	3.0764	0.079874	0.0360	0.0128573617
ss86288374		9	7.795892	0.4721	0.1419	0.706560	0.0068	0.0033886334
ss117969502		9	7.818601	0.5208	0.3052	0.580814	0.0100	0.0049686691
ss86315670		9	7.88878	0.3424	0.2582	0.611504	0.0098	0.0044338858
ss86292513		9	7.932084	0.3374	0.1036	0.747625	0.0063	0.0027963024
ss61561498	*rs41661108*	9	7.978591	0.3173	0.3564	0.550724	0.0118	0.0050927830
ss86303137		9	8.00626	0.4878	0.8598	0.354125	0.0170	0.0085035579
ss86336619		9	8.027657	0.4871	0.7265	0.394305	0.0156	0.0078064765
ss86335604		9	8.111423	0.4327	0.7376	0.390717	0.0159	0.0077829595
ss86339067		9	8.1322	0.4506	0.0455	0.831130	0.0038	0.0019008307
ss86309015		9	8.372746	0.6383	0.2229	0.636991	0.0090	0.0041489248
ss61469402	*rs41569509*	9	8.476054	0.8023	3.0923	0.079105	0.0400	0.0126895709
ss117964045		9	8.499321	0.4319	2.5876	0.108159	0.0291	0.0142942821
ss86307398		9	8.5316	0.6905	9.7524	0.001865	0.0615	0.0262731380
	rs29013550	9	8.619027	0.6454	1.3237	0.250319	0.0221	0.0100981900
ss86315562		9	8.897451	0.7199	2.4617	0.117109	0.0324	0.0130847223
ss86302582		9	8.945116	0.6447	1.6452	0.200041	0.0245	0.0112415451
ss61517396	*rs41614874*	9	9.190373	0.5394	0.0989	0.753295	0.0059	0.0029092706
ss86285697		9	9.24803	0.6791	2.1434	0.143639	0.0291	0.0126889317
ss86278552		9	9.439531	0.4420	1.5115	0.219329	0.0229	0.0112863791
ss86324164		9	9.55506	0.4348	0.0302	0.862164	0.0031	0.0015226025
ss61561057	*rs41656773*	9	9.671463	0.7880	3.0079	0.083302	0.0381	0.0127467179
ss86340065		9	9.846923	0.2858	1.6396	0.200805	0.0263	0.0107377073

ss117969756		9	9.88621	0.5910	1.2853	0.257310	0.0213	0.0102976430
ss86303189		9	9.957289	0.6877	8.8392	0.003050	0.0582	0.0249920388
ss61493105	*rs41592651*	9	9.977956	0.7285	5.2299	0.022502	0.0482	0.0190855651
ss86286049		9	10.079999	0.8159	15.7862	0.000078	0.0966	0.0290256904
ss61531619	*rs41628074*	9	10.295706	0.7142	9.0155	0.002773	0.0619	0.0252591588
ss86329439		9	10.436345	0.1683	12.6026	0.000411	0.0884	0.0247539815
ss86293528		9	10.599762	0.5480	9.0204	0.002766	0.0544	0.0269403855
ss86287603		9	10.86779	0.7342	7.7570	0.005496	0.0583	0.0227708944
ss61566478	*rs41664308*	9	10.997778	0.4570	8.8519	0.003029	0.0551	0.0273604704
ss86308446		9	11.028753	0.2357	5.7661	0.016600	0.0520	0.0187468740
ss61566472	*rs41664302*	9	11.130827	0.5344	0.9107	0.340269	0.0172	0.0085620429
ss86289599		9	11.188981	0.6633	5.3373	0.021165	0.0457	0.0204006263
ss61566473	*rs41664303*	9	11.236334	0.7973	0.2920	0.589106	0.0124	0.0040182188
ss63760649	*rs43732417*	9	11.271076	0.2235	0.0951	0.757836	0.0070	0.0024180078
	rs29015574	9	11.341601	0.2149	9.5755	0.002051	0.0682	0.0230046689
ss61471731	*rs41572480*	9	11.363455	0.5473	2.4013	0.121686	0.0291	0.0144249750
ss86287742		9	11.533511	0.6698	1.4907	0.222526	0.0231	0.0101969319
ss86337220		9	11.568298	0.0888	9.2456	0.002449	0.0992	0.0160535157
ss117969588		9	11.61531	0.0996	11.7422	0.000647	0.1062	0.0190426801
ss61561893	*rs41665495*	9	11.648284	0.2443	0.8150	0.366966	0.0191	0.0070470014
ss86337694		9	11.884953	0.6504	5.7633	0.016626	0.0465	0.0211564484
ss61493485	*rs41595013*	9	11.905525	0.2787	1.6194	0.203595	0.0269	0.0108109553
	rs29026961	9	11.932335	0.9334	3.1544	0.076160	0.0649	0.0080682089
ss86285132		9	11.979284	0.6411	6.7818	0.009406	0.0503	0.0231595384
ss61469435	*rs41570342*	9	12.013313	0.4241	4.1035	0.043176	0.0382	0.0186836047
ss86288989		9	12.088349	0.3517	0.6809	0.409556	0.0159	0.0072400443
ss61493505	*rs41588138*	9	12.144952	0.8703	24.1434	0.000001	0.1337	0.0301686276
ss86316316		9	12.167171	0.0716	8.5881	0.003495	0.1011	0.0134456241
ss86326396		9	12.218486	0.8037	11.3024	0.000816	0.0755	0.0238087476
ss86277300		9	12.327874	0.1805	0.3635	0.546754	0.0144	0.0042696228
ss86328287		9	12.454648	0.0745	8.4339	0.003800	0.0988	0.0136298628
ss86332087		9	12.673638	0.1146	0.0225	0.880769	0.0045	0.0009221203
ss86317257		9	12.90243	0.7615	0.6507	0.420126	0.0177	0.0064281592
ss86339090		9	12.980171	0.3668	2.6152	0.106296	0.0300	0.0139520944
ss86310861		9	13.034443	0.8331	2.0003	0.157717	0.0352	0.0097971614
ss64127907	*rs42492243*	9	13.167602	0.9334	10.5457	0.001220	0.1164	0.0144722795
ss86304615		9	13.208373	0.7715	7.6309	0.005889	0.0585	0.0206358824
ss86282355		9	13.237156	0.3001	2.5897	0.108014	0.0328	0.0137589932
ss86297099		9	13.287945	0.6956	2.0611	0.151547	0.0285	0.0120644817
ss64662239	*rs42758714*	9	13.384362	0.4799	7.5838	0.006043	0.0528	0.0263586437
ss61519978	*rs41620302*	9	13.437845	0.6569	0.6988	0.403473	0.0161	0.0072749969
ss86324931		9	13.464325	0.2013	0.8748	0.349945	0.0212	0.0068242385
ss63865114	*rs42387923*	9	13.642168	0.1755	0.3595	0.548955	0.0146	0.0042151251
	rs29021716	9	13.671799	0.2314	2.0473	0.152927	0.0312	0.0111087098
ss63864746	*rs42386739*	9	13.704175	0.3009	0.4548	0.500266	0.0136	0.0057294400
ss61565359	*rs41660114*	9	13.729565	0.4019	0.8497	0.356953	0.0173	0.0082957884
ss61506796	*rs41608290*	9	13.787279	0.1347	3.6542	0.056340	0.0493	0.0114963135

ss64733321	*rs42825878*	9	13.821087	0.4620	0.3816	0.536969	0.0114	0.0056916369
ss117969710		9	13.861926	0.5351	0.8977	0.343733	0.0173	0.0086059283
ss64845337	*rs42937080*	9	13.929349	0.5029	1.2712	0.259937	0.0211	0.0105555538
ss86311604		9	13.958461	0.4298	1.0796	0.299138	0.0194	0.0095139312
ss61563201	*rs41659288*	9	14.0351	0.9076	6.5847	0.010494	0.0772	0.0129489721
ss117969506		9	14.061745	0.0931	6.3094	0.012235	0.0754	0.0127361333
ss65505597	*rs43587874*	9	14.092685	0.7987	0.5884	0.443303	0.0176	0.0056617337
ss64773902	*rs42875976*	9	14.1706	0.8023	1.3785	0.240757	0.0276	0.0087582481
ss64773939	*rs42876012*	9	14.211551	0.7923	1.9532	0.162685	0.0322	0.0106005912
ss86338950		9	14.323246	0.5021	1.7194	0.190202	0.0246	0.0122824442
ss65505225	*rs43585209*	9	14.345512	0.4979	1.7194	0.190202	0.0246	0.0122824442
ss65505156	*rs43585140*	9	14.393906	0.8818	2.5470	0.110955	0.0467	0.0097260370
ss117964071		9	14.501478	0.5616	0.0063	0.936920	0.0015	0.0007346034
ss65505004	*rs43583789*	9	14.520363	0.3431	3.9611	0.046953	0.0372	0.0167809476
ss117969504		9	14.545788	0.6103	2.8334	0.092773	0.0329	0.0156361477
ss86305633		9	14.572032	0.2865	6.6126	0.010333	0.0530	0.0216841552
ss86322890		9	14.612315	0.7228	5.3597	0.020898	0.0489	0.0195908195
ss86337629		9	14.693807	0.9405	9.0994	0.002650	0.1173	0.0131150625
ss86340069		9	14.805473	0.4062	1.4568	0.227855	0.0227	0.0109613882
ss61465506	*rs41566752*	9	14.829441	0.4405	12.1751	0.000515	0.0629	0.0310004271
ss86318088		9	14.849514	0.7901	4.3198	0.038036	0.0477	0.0158191901
ss117969601		9	14.893068	0.3388	0.8954	0.344351	0.0190	0.0085264198
ss86274743		9	14.920948	0.2650	2.2157	0.137070	0.0316	0.0123204678
ss86339691		9	14.941433	0.7958	0.4966	0.481221	0.0159	0.0051540257
ss86316196		9	14.997862	0.2156	0.1967	0.657510	0.0096	0.0032358027
ss64551050	*rs42690619*	9	15.021036	0.7822	0.1406	0.707844	0.0081	0.0027518010
	rs29009707	9	15.046354	0.2149	0.2188	0.640076	0.0101	0.0034056010
ss61562009	*rs41656415*	9	15.096593	0.7464	0.2815	0.595877	0.0107	0.0040595919
ss86294619		9	15.170291	0.2256	4.0579	0.044350	0.0442	0.0154548349
ss117969614		9	15.208643	0.0573	0.7264	0.394346	0.0322	0.0034759492
ss61517104	*rs41611409*	9	15.232993	0.9047	0.1266	0.722064	0.0111	0.0019137134
ss86287053		9	15.267826	0.7951	0.2179	0.640816	0.0112	0.0036383704
ss64661303	*rs42754745*	9	15.299048	0.7636	3.7614	0.052852	0.0413	0.0149272926
ss61562050	*rs41657156*	9	15.32955	0.2256	3.8543	0.050016	0.0431	0.0150715633
	rs29010513	9	15.359626	0.1762	0.0011	0.973643	0.0008	0.0002375538
ss65503640	*rs43581733*	9	15.392921	0.5294	2.7198	0.099560	0.0318	0.0158562480
ss65503652	*rs43581745*	9	15.414089	0.3510	17.4230	0.000034	0.0799	0.0363864769
ss117964068		9	15.543753	0.0638	0.0128	0.909935	0.0044	0.0005202848
ss65503921	*rs43583113*	9	15.617704	0.0688	2.0648	0.151182	0.0523	0.0067016242
ss65503948	*rs43583540*	9	15.641092	0.9255	1.8567	0.173443	0.0476	0.0065639214
ss65504066	*rs43584258*	9	15.698076	0.9491	0.7296	0.393298	0.0361	0.0034873984
ss65504214	*rs43585106*	9	15.721586	0.5638	3.6050	0.058021	0.0357	0.0175573159
ss86287541		9	15.745458	0.7837	9.3172	0.002357	0.0665	0.0225477596
ss64213146	*rs42534251*	9	15.797738	0.4585	6.2434	0.012695	0.0454	0.0225573921
ss64213220	*rs42534288*	9	15.820168	0.4126	3.0702	0.080181	0.0328	0.0159081111
ss61511503	*rs41612816*	9	15.854577	0.6304	1.9093	0.167485	0.0265	0.0123526650
ss63060053	*rs42093661*	9	15.878711	0.1289	1.5190	0.218184	0.0340	0.0076325172

ss61562171	*rs41658175*	9	15.908191	0.5638	1.4112	0.235266	0.0228	0.0111915650
ss64213384	*rs42535370*	9	15.958289	0.6239	4.7583	0.029492	0.0434	0.0203765024
ss86292891		9	16.017243	0.6153	1.7833	0.182187	0.0259	0.0122498455
ss86280351		9	16.14885	0.3954	2.5768	0.108895	0.0300	0.0143533898
ss86300877		9	16.215898	0.5716	2.7052	0.100475	0.0308	0.0151006564
ss65066985	*rs43159199*	9	16.419335	0.8446	0.0149	0.902938	0.0032	0.0008473099
ss64890698	*rs42989034*	9	16.453004	0.9097	0.6280	0.428377	0.0258	0.0042450926
ss117974913		9	16.486324	0.4871	0.5478	0.459458	0.0142	0.0070734064
ss64511140	*rs42672636*	9	16.543551	0.6956	0.0321	0.857919	0.0037	0.0015527527
ss64622398	*rs42727013*	9	16.598606	0.7486	0.1779	0.673284	0.0087	0.0032830508
ss65504589	*rs43587677*	9	16.672669	0.8052	0.0025	0.960528	0.0012	0.0003652076
ss61493634	*rs41589266*	9	16.70126	0.5888	4.3035	0.038401	0.0389	0.0188527418
ss86325533		9	16.733047	0.7736	3.4292	0.064477	0.0432	0.0151416339
ss86276690		9	16.902237	0.8087	2.9336	0.087198	0.0407	0.0126033816
ss86309864		9	16.957637	0.3553	1.9420	0.163894	0.0269	0.0123341032
ss86280071		9	17.2637	0.1297	1.2881	0.256794	0.0307	0.0069292282
	rs29018912	9	17.377902	0.3016	6.6938	0.009876	0.0519	0.0218552874
ss61526195	*rs41625777*	9	17.463109	0.4126	2.9380	0.086962	0.0313	0.0151596897
ss61526192	*rs41625774*	9	17.512695	0.9105	6.5559	0.010663	0.0805	0.0131312984
ss61567063	*rs41659883*	9	17.610514	0.7314	7.8154	0.005323	0.0591	0.0232029270
ss117969496		9	17.655859	0.2679	6.4162	0.011527	0.0537	0.0210767304
ss61519857	*rs41618893*	9	17.693536	0.8510	0.0000	0.997101	0.0001	0.0000243293
ss86333950		9	17.806627	0.2006	2.4164	0.120524	0.0357	0.0114401101
ss86280793		9	17.955339	0.9083	0.0081	0.928144	0.0028	0.0004736116
ss86315017		9	17.975396	0.6117	2.2793	0.131564	0.0296	0.0140524039
ss86309646		9	18.010962	0.9370	0.6492	0.420676	0.0300	0.0035430082
ss61563174	*rs41659263*	9	18.056232	0.3746	4.1850	0.041158	0.0393	0.0184192240
ss86334311		9	18.644125	0.5330	4.8797	0.027499	0.0406	0.0202174845
ss61475443	*rs41574520*	9	18.693964	0.8632	5.6613	0.017612	0.0624	0.0147378823
ss86341262		9	18.729012	0.6841	0.0044	0.947271	0.0014	0.0005862951
ss86298592		9	18.803717	0.2901	0.0033	0.954078	0.0012	0.0004804258
ss61525101	*rs41625327*	9	18.858333	0.1117	0.7228	0.395518	0.0245	0.0048609449
ss86275119		9	18.956179	0.8016	2.8584	0.091347	0.0397	0.0126237550
ss86274165		9	19.223825	0.3073	3.3012	0.069657	0.0365	0.0155237707
ss86283719		9	19.286193	0.0530	0.0088	0.925416	0.0038	0.0003802723
ss86315255		9	19.364989	0.0681	9.4507	0.002193	0.1118	0.0141778617
ss117964034		9	19.431988	0.2944	0.0049	0.944376	0.0014	0.0005918777
ss61530023	*rs41630520*	9	19.559728	0.8009	0.6923	0.405662	0.0199	0.0063537937
ss61569742	*rs41669605*	9	19.634221	0.1612	10.1642	0.001496	0.0790	0.0213664903
ss61569741	*rs41669604*	9	19.654672	0.8388	10.1956	0.001471	0.0787	0.0212902130
ss86327337		9	19.695949	0.7128	2.6250	0.105648	0.0331	0.0135493397
ss86328179		9	19.79126	0.2256	0.5336	0.465358	0.0162	0.0056457103
ss61562202	*rs41658206*	9	19.876523	0.3331	1.9160	0.166746	0.0270	0.0119754151
	rs29012613	9	19.913659	0.3202	1.4778	0.224524	0.0242	0.0105356056
ss86289869		9	20.050569	0.8904	0.0107	0.917723	0.0030	0.0005922528
ss86311302		9	20.108422	0.1289	1.4203	0.233765	0.0327	0.0073432448
ss61505512	*rs41607150*	9	20.150962	0.7314	0.0474	0.827733	0.0044	0.0017404803

ss61562209		9	20.270919	0.7070	0.2125	0.644997	0.0091	0.0037712293
ss86337473		9	20.359435	0.5946	7.2415	0.007295	0.0506	0.0244075225
ss61493654	*rs41589283*	9	20.426833	0.1819	6.5831	0.010503	0.0606	0.0180489873
ss61476628	*rs41577782*	9	20.501671	0.1984	13.2375	0.000295	0.0831	0.0264414098
ss65505711	*rs43585289*	9	20.55901	0.5057	3.6787	0.055520	0.0354	0.0177145886
ss64696603	*rs42797639*	9	20.704626	0.4405	17.3528	0.000035	0.0792	0.0390165458
ss65006132	*rs43100186*	9	21.025181	0.5473	1.2504	0.263856	0.0208	0.0103124818
ss64937758	*rs43030751*	9	21.062121	0.1089	1.0735	0.300527	0.0304	0.0058900016
ss63500117	*rs42251694*	9	21.103785	0.3926	7.3827	0.006749	0.0520	0.0247865704
ss64980533	*rs43073626*	9	21.171139	0.6053	7.6305	0.005891	0.0525	0.0250940991
ss61469517	*rs41570419*	9	21.19869	0.6383	4.8865	0.027391	0.0432	0.0199262792
ss105263759		9	21.298747	0.9011	0.1969	0.657363	0.0139	0.0024764437
ss63501131	*rs42254873*	9	21.423098	0.0766	3.1549	0.076138	0.0626	0.0088632574
ss61507411	*rs41605705*	9	21.473706	0.3152	0.0189	0.890814	0.0027	0.0011572439
ss61476957	*rs41580207*	9	21.554831	0.7378	1.0414	0.307857	0.0216	0.0083503749
ss63501569	*rs42256018*	9	21.624088	0.0494	0.0078	0.929602	0.0038	0.0003560028
ss64731770	*rs42829233*	9	21.870659	0.5917	0.0037	0.951527	0.0011	0.0005509712
ss61474061	*rs41566673*	9	21.917247	0.6046	8.6674	0.003347	0.0554	0.0264671390
ss86330918		9	21.998197	0.4792	0.0995	0.752519	0.0057	0.0028544633
ss65507886	*rs43589255*	9	22.086493	0.4585	1.2469	0.264522	0.0206	0.0102201754
ss65507860	*rs43588529*	9	22.13288	0.8961	1.8051	0.179530	0.0401	0.0074680616
ss61493081	*rs41591827*	9	22.370327	0.5480	0.0014	0.970147	0.0007	0.0003404523
ss65507736	*rs43587505*	9	22.407445	0.2672	0.4455	0.504683	0.0143	0.0055939906
ss65507605	*rs43594973*	9	22.469683	0.2529	5.5635	0.018614	0.0507	0.0191736682
ss65507581	*rs43594949*	9	22.49131	0.7830	4.8352	0.028213	0.0490	0.0166543466
ss65506241	*rs43590019*	9	22.513187	0.2185	4.3747	0.036837	0.0462	0.0157764861
ss65506189	*rs43589967*	9	22.533481	0.3711	4.1509	0.041990	0.0396	0.0184738528
ss61562217	*rs41658220*	9	22.591498	0.3954	2.3867	0.122828	0.0297	0.0141837030
ss61493658	*rs41589286*	9	22.616748	0.1597	4.4572	0.035110	0.0529	0.0142035579
ss86285843		9	22.678277	0.1748	0.4471	0.503957	0.0161	0.0046513596
ss86329362		9	22.706983	0.6719	5.2628	0.022083	0.0463	0.0203921673
ss65506378	*rs43591456*	9	22.744932	0.1397	3.0975	0.078853	0.0461	0.0110744494
ss86322026		9	22.90558	0.8718	9.7152	0.001903	0.0855	0.0191157449
ss86296758		9	22.949111	0.1289	8.9470	0.002877	0.0820	0.0184102371
ss65022010	*rs43110256*	9	22.984955	0.1383	3.6475	0.056565	0.0498	0.0118716443
ss64094197	*rs42470375*	9	23.136071	0.8682	0.1937	0.659972	0.0117	0.0026809827
ss86339201		9	23.161749	0.8725	0.0013	0.971486	0.0010	0.0002245894
ss61530673	*rs41627644*	9	23.197138	0.3417	0.8709	0.351036	0.0186	0.0083480535
ss86335413		9	23.224758	0.2772	0.1936	0.660074	0.0091	0.0036308605
ss65506564	*rs43593442*	9	23.270145	0.8209	0.2617	0.609111	0.0123	0.0036063121
	rs29022325	9	23.298098	0.5208	0.0197	0.888472	0.0026	0.0013019439
ss65506496	*rs43592374*	9	23.334734	0.7235	1.5384	0.215280	0.0261	0.0104489548
ss61562222	*rs41658225*	9	23.378695	0.2908	0.4773	0.489869	0.0141	0.0058218360
ss86292792		9	23.418979	0.7120	2.7412	0.098241	0.0332	0.0136256000
ss61562225	*rs41658228*	9	23.600605	0.7500	15.7652	0.000079	0.0859	0.0322307635
ss86286021		9	23.639928	0.6913	5.7011	0.017220	0.0483	0.0206368384
ss86338408		9	23.699368	0.2443	0.0001	0.993356	0.0002	0.0000661899

ss86305715		9	23.736145	0.3725	6.4593	0.011253	0.0470	0.0219556525
ss117962884		9	23.842547	0.6089	1.7476	0.186610	0.0251	0.0119328557
ss64706833	*rs42805852*	9	23.883941	0.7178	0.0808	0.776277	0.0058	0.0023650533
ss65507067	*rs43590141*	9	23.952021	0.5559	0.0001	0.990694	0.0002	0.0001077438
ss65507076	*rs43590150*	9	23.977598	0.8023	0.1182	0.731117	0.0080	0.0025331965
ss61493064	*rs41591810*	9	24.021404	0.2765	0.0095	0.922284	0.0020	0.0007981344
ss86292872		9	24.042376	0.8080	0.3457	0.556747	0.0138	0.0042904347
ss86306726		9	24.079997	0.8188	0.1077	0.742923	0.0077	0.0022983319
ss86288636		9	24.148125	0.4950	0.8470	0.357735	0.0168	0.0083881792
ss86339835		9	24.174837	0.4592	0.3333	0.563903	0.0107	0.0053361339
ss86335513		9	24.302376	0.7228	4.4412	0.035439	0.0423	0.0169675922
ss64039352	*rs42444241*	9	24.328277	0.2715	4.5810	0.032676	0.0434	0.0171685362
ss86339542		9	24.362922	0.3524	2.2528	0.133825	0.0290	0.0132368193
ss117969713		9	24.389744	0.6203	1.5478	0.213878	0.0239	0.0112765881
ss86336423		9	24.431908	0.4384	0.1528	0.695964	0.0075	0.0036952460
	rs29014647	9	24.452332	0.2815	2.3511	0.125652	0.0315	0.0127229495
ss61531690	*rs41629242*	9	24.488898	0.1669	1.7830	0.182216	0.0324	0.0090105356
ss64121895	*rs42490265*	9	24.591291	0.8725	0.0106	0.917911	0.0030	0.0006564888
ss64121847	*rs42490241*	9	24.624216	0.4842	4.1949	0.040920	0.0365	0.0182460619
ss63435256	*rs42234120*	9	24.756412	0.1089	5.6093	0.018137	0.0683	0.0132498562
ss86311126		9	24.832488	0.2665	0.6849	0.408177	0.0172	0.0067322841
ss86299943		9	24.865475	0.3997	0.2928	0.588611	0.0102	0.0048954869
ss63434416	*rs42232241*	9	25.092629	0.1153	5.5088	0.019200	0.0656	0.0133930355
ss86284363		9	25.141936	0.1454	7.1307	0.007755	0.0685	0.0170340091
ss63434611	*rs42232306*	9	25.20373	0.8890	0.2227	0.637137	0.0142	0.0028004419
ss63434980	*rs42233228*	9	25.244579	0.1927	0.2071	0.649201	0.0108	0.0033567355
ss65508501	*rs43594819*	9	25.431	0.4628	1.2184	0.270054	0.0208	0.0103177268
ss64733726	*rs42828583*	9	25.567348	0.7543	2.5092	0.113639	0.0345	0.0128057028
ss65508664	*rs43596682*	9	25.679657	0.1597	2.3778	0.123521	0.0388	0.0104081635
	rs29013870	9	25.735289	0.4011	1.1368	0.286702	0.0203	0.0097355870
ss86316755		9	25.761293	0.3990	1.3206	0.250876	0.0218	0.0104773662
ss61560984	*rs41656002*	9	25.805685	0.8403	2.8520	0.091711	0.0431	0.0115796702
ss65073244	*rs43167970*	9	25.837776	0.8424	2.6007	0.107269	0.0413	0.0109748104
ss65035931	*rs43128600*	9	25.863098	0.6017	0.0089	0.924953	0.0018	0.0008606282
ss65508009	*rs43590078*	9	25.961316	0.1468	3.4519	0.063599	0.0478	0.0119873969
ss65507913	*rs43589282*	9	25.986872	0.8553	3.5997	0.058205	0.0496	0.0122672972
ss61560991	*rs41656009*	9	26.04363	0.1870	2.9617	0.085705	0.0407	0.0123741901
ss65508967	*rs43589584*	9	26.090528	0.2042	0.5177	0.472075	0.0168	0.0054470854
ss86301358		9	26.113913	0.5630	6.7180	0.009745	0.0486	0.0239261264
ss61560994	*rs41656012*	9	26.146425	0.3725	2.1886	0.139485	0.0289	0.0135171744
ss65509105	*rs43590402*	9	26.16915	0.1232	0.4361	0.509240	0.0197	0.0042466956
ss61560995	*rs41656013*	9	26.189626	0.3102	0.0830	0.773299	0.0060	0.0025696247
ss61493092	*rs41592638*	9	26.276976	0.6325	3.0043	0.083488	0.0338	0.0156982479
	rs29017323	9	26.400149	0.4040	2.6105	0.106609	0.0302	0.0145357404
ss61561002	*rs41656020*	9	26.420262	0.1662	0.0782	0.779869	0.0069	0.0019113732
ss86313412		9	26.50404	0.8266	2.2122	0.137377	0.0366	0.0104759142
ss65509323	*rs43591914*	9	26.563582	0.6203	1.2937	0.255764	0.0215	0.0101217983

ss61512440	*rs41612646*	9	26.720516	0.3489	7.0871	0.007944	0.0517	0.0234909747
	rs29012689	9	26.819326	0.6189	1.7917	0.181157	0.0259	0.0122097832
ss86310166		9	26.888319	0.4011	0.1821	0.669703	0.0080	0.0038465691
ss86321039		9	26.971344	0.7149	4.7175	0.030194	0.0440	0.0179552141
ss86295051		9	27.004484	0.8625	0.5962	0.440309	0.0208	0.0049344088
ss86295525		9	27.041204	0.5215	2.3100	0.128996	0.0277	0.0138161228
ss86338213		9	27.062878	0.4477	3.6301	0.057156	0.0347	0.0171709514
ss86337146		9	27.095987	0.4807	1.3422	0.247045	0.0213	0.0106510219
ss117969753		9	27.368575	0.3696	0.3263	0.568052	0.0110	0.0051084704
ss86312043		9	27.404409	0.7178	0.4555	0.499956	0.0140	0.0056642681
ss86290171		9	27.510202	0.1819	0.1152	0.734411	0.0080	0.0023913035
ss86293445		9	27.682831	0.7156	12.8112	0.000368	0.0738	0.0300320063
ss61498480	*rs43712080*	9	27.739862	0.5996	1.4060	0.236123	0.0226	0.0108691698
ss64333573	*rs42588658*	9	27.856638	0.1103	0.6999	0.403094	0.0251	0.0049205152
ss64333469	*rs42587506*	9	27.889053	0.1640	0.0981	0.754181	0.0079	0.0021620418
ss61496422	*rs41598671*	9	27.950737	0.6791	5.5456	0.018804	0.0468	0.0204115380
ss64333265	*rs42586005*	9	27.986666	0.5852	7.2740	0.007165	0.0495	0.0240369154
ss61518467	*rs41614223*	9	28.078389	0.8847	0.2042	0.651529	0.0134	0.0027247963
ss86282101		9	28.172899	0.8875	1.1544	0.282994	0.0317	0.0063376772
ss64037336	*rs42443623*	9	28.239724	0.5480	4.6792	0.030870	0.0397	0.0196738983
ss117969682		9	28.327369	0.4054	2.4585	0.117344	0.0303	0.0145875759
ss61550712	*rs41653483*	9	28.395055	0.5831	3.1121	0.078152	0.0334	0.0162377018
ss64467452	*rs42651779*	9	28.463456	0.7292	3.6211	0.057464	0.0401	0.0158194022
ss86310762		9	28.519316	0.6010	0.3244	0.569174	0.0109	0.0052058560
ss64948677	*rs43038766*	9	28.670905	0.5100	0.2725	0.601798	0.0095	0.0047342142
ss64836113	*rs42930065*	9	28.699072	0.5165	0.4540	0.500686	0.0123	0.0061356955
ss86297665		9	28.724343	0.4835	0.4540	0.500686	0.0123	0.0061356955
ss64877837	*rs42970704*	9	28.759225	0.4842	0.4723	0.492152	0.0125	0.0062669664
ss64877852	*rs42970719*	9	28.784894	0.4162	0.4930	0.482835	0.0133	0.0064478789
ss64549854	*rs42695424*	9	28.8119	0.3582	2.1753	0.140696	0.0288	0.0132618655
ss64549754	*rs42695374*	9	28.849995	0.8947	0.0384	0.844762	0.0059	0.0011145353
ss86323810		9	28.891412	0.6734	0.0761	0.782701	0.0054	0.0023916697
ss61466157	*rs41565918*	9	28.93435	0.7593	0.0011	0.973010	0.0007	0.0002707160
ss63465373	*rs42245066*	9	28.954808	0.6762	0.2810	0.596219	0.0105	0.0046076738
ss86320461		9	28.976117	0.2113	0.6563	0.418148	0.0189	0.0062957201
ss86327900		9	29.00047	0.4427	0.3502	0.554175	0.0115	0.0056787902
ss86298391		9	29.057963	0.4384	0.2839	0.594331	0.0100	0.0049173499
ss117969556		9	29.188811	0.1619	2.4368	0.118969	0.0391	0.0106009247
ss86330571		9	29.391945	0.4885	0.0000	0.997400	0.0001	0.0000312014
ss86341122		9	29.415106	0.4914	0.0118	0.913423	0.0021	0.0010469237
ss65509689	*rs43594577*	9	29.436521	0.4477	0.5721	0.449696	0.0144	0.0071089461
ss61511010	*rs41609936*	9	29.601393	0.2514	0.5115	0.474715	0.0149	0.0055963646
ss117963041		9	29.658952	0.1082	1.7053	0.192028	0.0390	0.0075321442
ss86289988		9	29.71985	0.1597	3.9987	0.045924	0.0513	0.0137603379
ss61504700	*rs41609173*	9	29.758237	0.9019	0.0036	0.952171	0.0019	0.0003307530
ss65510237	*rs43593733*	9	29.880844	0.6812	6.1334	0.013502	0.0498	0.0216269949
ss64786982	*rs42886388*	9	29.903063	0.3797	1.3311	0.249000	0.0213	0.0100554554

ss65510259	*rs43593755*	9	30.100532	0.9054	0.0238	0.877501	0.0049	0.0008328294
ss65510314	*rs43593810*	9	30.151648	0.3202	0.9129	0.339691	0.0192	0.0083676861
ss65510341	*rs43594837*	9	30.184607	0.5817	3.3488	0.067680	0.0349	0.0169723417
ss65510349	*rs43594845*	9	30.223865	0.4140	1.9228	0.165996	0.0259	0.0125632172
ss63834596	*rs42373638*	9	30.387865	0.8503	3.5202	0.061043	0.0491	0.0124912711
ss61495022	*rs41592505*	9	30.630752	0.1067	0.2036	0.651969	0.0136	0.0025853173
ss65510832	*rs43589828*	9	30.686893	0.1590	0.3480	0.555465	0.0146	0.0039098817
ss86300934		9	30.713943	0.4656	0.0371	0.847308	0.0036	0.0017797109
ss86308100		9	30.743138	0.2414	0.0359	0.849687	0.0042	0.0015262065
ss61562696	*rs41663991*	9	30.78435	0.5029	3.0470	0.081329	0.0320	0.0160122180
ss86333956		9	30.817465	0.7830	0.3568	0.550504	0.0133	0.0045082699
ss117964074		9	30.842199	0.6225	0.1700	0.680225	0.0077	0.0036326508
ss65510587	*rs43596483*	9	30.879305	0.0781	0.3982	0.528248	0.0215	0.0030960800
ss64904362	*rs42994077*	9	30.908192	0.9219	0.2368	0.626679	0.0164	0.0023652645
	rs29017499	9	31.066009	0.2285	4.9263	0.026772	0.0474	0.0167247922
ss61504704	*rs41609177*	9	31.107151	0.8524	4.9829	0.025916	0.0578	0.0145433437
ss117969537		9	31.167837	0.3574	1.4402	0.230515	0.0233	0.0106984103
	rs29011985	9	31.302036	0.4620	0.5733	0.449219	0.0150	0.0074719293
ss63817963	*rs42362775*	9	31.568105	0.7328	0.8824	0.347872	0.0194	0.0075776516
ss64725765	*rs42822165*	9	31.601684	0.6397	5.3614	0.020878	0.0434	0.0200284711
ss64725717	*rs42821318*	9	31.628293	0.5287	7.0407	0.008149	0.0481	0.0239706217
ss64764067	*rs42857230*	9	31.706068	0.2615	1.9430	0.163786	0.0293	0.0113099312
ss64764053	*rs42857216*	9	31.732791	0.2643	2.3065	0.129292	0.0317	0.0123208224
ss64285656	*rs42568071*	9	31.757698	0.2658	2.1910	0.139269	0.0308	0.0120107720
ss64285716	*rs42568101*	9	31.783413	0.7020	9.6682	0.001951	0.0612	0.0256111601
ss117975291		9	32.141154	0.6755	1.2352	0.266780	0.0220	0.0096657896
ss64286298	*rs42560293*	9	32.217742	0.7228	1.0917	0.296451	0.0217	0.0086800212
ss64786131	*rs42885665*	9	32.367996	0.4069	2.0732	0.150358	0.0264	0.0127504825
ss64583200	*rs42707394*	9	32.421296	0.5688	0.6653	0.414972	0.0150	0.0073452225
ss86280251		9	32.460806	0.2142	0.9912	0.319786	0.0227	0.0076257157
ss64703082	*rs42798418*	9	32.543147	0.2837	2.0000	0.157744	0.0289	0.0117561402
ss65062404	*rs43154305*	9	32.690344	0.3524	3.5014	0.061736	0.0345	0.0157392463
ss61518603	*rs41616455*	9	32.721104	0.1640	0.8036	0.370324	0.0223	0.0061043320
ss63835062	*rs42375369*	9	32.753252	0.8703	1.0760	0.299957	0.0286	0.0064552712
ss64278564	*rs42559133*	9	32.778459	0.9384	0.4020	0.526244	0.0244	0.0028238163
ss61495839	*rs41593504*	9	32.806505	0.2636	1.2981	0.254955	0.0239	0.0092705555
ss61506627	*rs41607933*	9	32.902437	0.6003	0.1295	0.719015	0.0067	0.0032101548
ss61566585	*rs41665413*	9	32.927281	0.5838	0.2853	0.593442	0.0100	0.0048411695
ss86341540		9	33.007626	0.0673	0.0001	0.994246	0.0003	0.0000333010
ss63835414	*rs42377544*	9	33.040831	0.5781	8.9994	0.002797	0.0542	0.0264598775
ss61473370	*rs41569789*	9	33.069313	0.1418	0.1348	0.713660	0.0096	0.0023319274
ss63835546	*rs42377610*	9	33.092145	0.5301	0.0098	0.921141	0.0019	0.0009224968
ss63835588	*rs42378531*	9	33.114663	0.3660	0.0705	0.790618	0.0051	0.0023591166
ss86340202		9	33.147181	0.5745	1.8766	0.171162	0.0254	0.0124083801
ss86336285		9	33.232826	0.5537	2.8731	0.090518	0.0303	0.0149594671
ss86303987		9	33.261215	0.5480	0.5523	0.457620	0.0136	0.0067422492
ss64754441	*rs42853357*	9	33.283957	0.1024	0.1435	0.704962	0.0118	0.0021745078

ss64754627	*rs42855643*	9	33.304227	0.4470	2.8862	0.089788	0.0302	0.0149352525
ss64692878	*rs42786248*	9	33.434127	0.2027	1.1170	0.290925	0.0252	0.0081435570
ss64650134	*rs42744672*	9	33.455004	0.7679	0.3344	0.563271	0.0131	0.0046807882
ss61516644	*rs41617945*	9	33.476109	0.2299	0.2964	0.586308	0.0124	0.0043808454
ss64692849	*rs42785019*	9	33.618287	0.7844	0.4862	0.485874	0.0163	0.0055057036
ss117969534		9	33.748427	0.8009	0.2413	0.623433	0.0111	0.0035490446
ss86307194		9	33.791205	0.1920	3.7056	0.054636	0.0453	0.0140542414
ss86275754		9	33.83064	0.5552	1.5977	0.206656	0.0235	0.0116109112
ss61561012	*rs41656030*	9	33.885032	0.5931	0.0184	0.892269	0.0027	0.0012835956
ss65509569	*rs43593958*	9	33.950007	0.8911	0.0075	0.931129	0.0026	0.0005123675
ss64899104	*rs41256102*	9	34.007712	0.7851	4.4729	0.034791	0.0480	0.0161848677
ss86304729		9	34.030837	0.6511	0.0001	0.993383	0.0002	0.0000732222
ss61493109	*rs41592654*	9	34.059598	0.7256	1.5321	0.216210	0.0255	0.0101614671
ss86310961		9	34.096628	0.2285	2.1055	0.147225	0.0315	0.0111024764
ss86335849		9	34.124581	0.6103	0.2783	0.598015	0.0103	0.0048833885
ss61477625	*rs41578555*	9	34.196853	0.8052	0.0449	0.832262	0.0050	0.0015553330
	rs29021315	9	34.236547	0.4362	1.8971	0.168847	0.0259	0.0127337995
ss65511174	*rs43594170*	9	34.29782	0.1741	3.2070	0.073760	0.0430	0.0123764667
ss86320562		9	34.411485	0.0831	1.3595	0.244030	0.0391	0.0059567784
ss86298407		9	34.51402	0.8668	0.9830	0.321806	0.0271	0.0062531596
ss61518635	*rs41616487*	9	34.602197	0.7020	0.0949	0.758168	0.0063	0.0026463181
ss61561064	*rs41656780*	9	34.631838	0.7887	1.6207	0.203419	0.0287	0.0095528780
ss61511011	*rs41609937*	9	34.655353	0.2865	0.0007	0.979062	0.0006	0.0002259605
ss117964084		9	34.705797	0.8961	0.0163	0.898365	0.0039	0.0007329716
ss61473390	*rs41569809*	9	34.729139	0.3159	1.5057	0.220218	0.0245	0.0105860364
ss61518638	*rs41616490*	9	34.753883	0.7915	0.1575	0.691547	0.0094	0.0031183387
ss86328884		9	34.814012	0.0645	7.0595	0.008065	0.0977	0.0117821595
ss65511880	*rs43590472*	9	34.870418	0.2880	0.2312	0.630815	0.0099	0.0040404178
ss65511894	*rs43590486*	9	34.931916	0.6948	0.0172	0.895668	0.0026	0.0010850912
ss61561075	*rs41656790*	9	34.956517	0.4649	0.3123	0.576470	0.0101	0.0050292294
ss61561076	*rs41656791*	9	34.981931	0.8539	1.9480	0.163247	0.0364	0.0090776211
ss61561078	*rs41656793*	9	35.015741	0.8603	0.6220	0.430583	0.0209	0.0050299900
ss61561081	*rs41656796*	9	35.092935	0.1819	5.9954	0.014589	0.0571	0.0169895364
ss65508096	*rs43590115*	9	35.216258	0.8138	1.2297	0.267841	0.0262	0.0079488869
ss65508130	*rs43591349*	9	35.27155	0.7794	0.3127	0.576236	0.0122	0.0041899005
ss65508140	*rs43591359*	9	35.401735	0.5774	0.3396	0.560257	0.0110	0.0053577028
ss86322213		9	35.431519	0.6970	1.7579	0.185325	0.0267	0.0112982296
ss117975105		9	35.45476	0.2034	0.9258	0.336292	0.0218	0.0070767722
ss65511627	*rs43598415*	9	35.528244	0.6805	0.3181	0.572940	0.0114	0.0049528154
ss65511553	*rs43598341*	9	35.552974	0.4635	0.2200	0.639221	0.0088	0.0043942797
ss65511413	*rs43596401*	9	35.583355	0.4334	0.0385	0.844479	0.0038	0.0018520879
ss65511434	*rs43596422*	9	35.670291	0.5086	0.1063	0.744510	0.0060	0.0030064372
ss65511521	*rs43597509*	9	35.691589	0.1268	0.4270	0.513700	0.0181	0.0040111294
ss65511514	*rs43597502*	9	35.718533	0.8725	0.3830	0.536192	0.0171	0.0038113783
ss61504705	*rs41609178*	9	35.93861	0.1203	0.4630	0.496438	0.0193	0.0040952479
ss86327070		9	35.960712	0.6311	0.0719	0.788711	0.0052	0.0024150344
ss86300298		9	36.000103	0.6841	0.1435	0.704919	0.0076	0.0032778890

ss65511222	*rs43594217*	9	36.022791	0.1074	0.7125	0.398897	0.0251	0.0048170177
ss61476393	*rs41575954*	9	36.045359	0.1082	0.6150	0.433181	0.0233	0.0044919362
	rs29014022	9	36.082226	0.0487	0.7315	0.392677	0.0368	0.0034103855
ss65512337	*rs43593025*	9	36.222498	0.3059	4.7197	0.030156	0.0436	0.0185256574
ss65512370	*rs43593858*	9	36.275838	0.7393	6.2704	0.012505	0.0531	0.0204844366
ss65512402	*rs43593890*	9	36.310815	0.0358	0.1237	0.725175	0.0177	0.0012210413
ss65512430	*rs43593918*	9	36.333992	0.0358	0.1349	0.713517	0.0185	0.0012751544
ss61561088	*rs41656803*	9	36.357708	0.0430	2.4056	0.121358	0.0717	0.0058976816
ss65512466	*rs43594354*	9	36.383825	0.8016	0.3647	0.546079	0.0144	0.0045963785
	rs29009651	9	36.408576	0.7185	0.2197	0.639421	0.0099	0.0040028653
ss61561089	*rs41656804*	9	36.461948	0.7701	0.4218	0.516254	0.0143	0.0050565014
ss61493122	*rs41592667*	9	36.500708	0.6819	0.4974	0.480891	0.0141	0.0061056824
ss65512593	*rs43595079*	9	36.526858	0.1110	0.5731	0.449289	0.0226	0.0044622931
ss64860530	*rs42953660*	9	36.5785	0.3976	0.2991	0.584605	0.0101	0.0048217637
ss64860538	*rs42953668*	9	36.608206	0.5738	0.8614	0.353659	0.0172	0.0084168775
ss61552536	*rs43710988*	9	36.623903	0.7213	0.9437	0.331658	0.0205	0.0082295348
ss65248911	*rs43339636*	9	36.652174	0.3295	3.7911	0.051927	0.0380	0.0167808326
ss65248951	*rs43339676*	9	36.705578	0.8947	0.0150	0.902409	0.0037	0.0006964911
	rs29013916	9	36.732358	0.5072	1.3193	0.251114	0.0214	0.0106806079
ss65512758	*rs43589849*	9	36.771012	0.7557	0.0241	0.876675	0.0034	0.0012394948
ss64981926	*rs43071975*	9	36.816834	0.4678	1.8780	0.171004	0.0253	0.0125842266
	rs29017529	9	36.901494	0.5501	0.2664	0.605902	0.0100	0.0049436985
ss65238028	*rs43321323*	9	36.926868	0.8539	1.0800	0.299054	0.0269	0.0067138120
ss86341369		9	36.989725	0.8811	1.3163	0.251659	0.0334	0.0070031221
ss65073515	*rs43160312*	9	37.134721	0.4943	5.2513	0.022228	0.0434	0.0216882876
ss65073516	*rs43160313*	9	37.173449	0.4620	0.3605	0.548400	0.0115	0.0057347295
ss117969637		9	37.215456	0.3940	0.8958	0.344245	0.0183	0.0087178130
ss63979839	*rs42412988*	9	37.366965	0.3231	0.9763	0.323468	0.0195	0.0085485332
ss61890450	*rs41685105*	9	37.401721	0.6017	0.6067	0.436304	0.0150	0.0071775751
ss61533491	*rs41627557*	9	37.422684	0.3517	0.0133	0.908117	0.0023	0.0010331226
ss61890393	*rs41685086*	9	37.442895	0.6977	2.4959	0.114596	0.0320	0.0135045657
ss86313760		9	37.480678	0.5279	0.9627	0.326852	0.0184	0.0091603746
ss86340769		9	37.532777	0.6046	2.8250	0.093253	0.0310	0.0148444985
ss86305548		9	37.781932	0.5781	1.5897	0.207797	0.0239	0.0116489260
ss86336605		9	37.896394	0.2428	8.4000	0.003870	0.0641	0.0235728358
ss86337395		9	37.918503	0.8066	3.4017	0.065555	0.0422	0.0131641830
ss86279134		9	37.950037	0.7321	0.2952	0.587105	0.0119	0.0046832741
ss61562915	*rs41666002*	9	37.986536	0.4226	2.1351	0.144415	0.0272	0.0132509330
ss86322484		9	38.045164	0.5809	2.7186	0.099637	0.0308	0.0150157533
ss61527923		9	38.132527	0.6669	0.0927	0.760871	0.0060	0.0026773779
ss86283818		9	38.196921	0.0953	2.7652	0.096784	0.0535	0.0092262772
ss86307254		9	38.303752	0.3904	1.1731	0.279134	0.0206	0.0097816626
ss86338029		9	38.348612	0.7500	0.6793	0.410108	0.0179	0.0067150758
ss86313515		9	38.425092	0.8431	1.2318	0.267440	0.0286	0.0075566176
ss63897410	*rs41966290*	9	38.570505	0.0817	1.1966	0.274387	0.0359	0.0053847987
ss86285965		9	38.602109	0.7557	1.0977	0.295135	0.0230	0.0084897906
ss63897422	*rs41966296*	9	38.625839	0.6196	0.7855	0.375756	0.0175	0.0082604362

ss61521294	*rs41618213*	9	38.648877	0.3804	0.7855	0.375756		0.0175	0.0082604362
ss86291262		9	38.711556	0.7027	0.1707	0.679603		0.0083	0.0034637094
ss86309741		9	38.738119	0.3044	0.2647	0.607072		0.0102	0.0043010578
ss63898316	*rs41971041*	9	38.798481	0.2350	0.6858	0.407881		0.0186	0.0066819246
	rs29014434	9	38.823931	0.6963	1.0262	0.311399		0.0202	0.0085387372
ss61496084	*rs41596540*	9	38.905678	0.7844	1.9769	0.160165		0.0318	0.0107692521
ss64300906	*rs42574126*	9	38.947304	0.1311	1.4722	0.225409		0.0337	0.0076804137
ss64300866	*rs42574106*	9	39.015501	0.9291	1.0547	0.304795		0.0373	0.0049198787
ss65512284	*rs43592972*	9	39.062436	0.7285	2.6285	0.105416		0.0339	0.0134106549
ss61493121	*rs41592666*	9	39.095851	0.0759	4.1889	0.041065		0.0717	0.0100549429
ss63109112	*rs42116548*	9	39.137079	0.0616	0.0025	0.960187		0.0019	0.0002225123
ss61487563	*rs41584130*	9	39.212024	0.7600	0.4877	0.485195		0.0150	0.0054884770
ss65512007	*rs43591299*	9	39.268064	0.8274	4.6443	0.031499		0.0529	0.0151011003
	rs29022845	9	39.369495	0.3345	0.3305	0.565546		0.0113	0.0050250649
ss61493118		9	39.465568	0.2163	3.1004	0.078711		0.0393	0.0133327666
ss86301501		9	39.520093	0.1784	2.7981	0.094828		0.0393	0.0115267149
ss61525915	*rs41623216*	9	39.59766	0.4799	0.0039	0.950305		0.0011	0.0005680396
ss86300400		9	39.724516	0.5480	0.0537	0.816833		0.0043	0.0021408273
ss63857122	*rs42385819*	9	39.75148	0.2636	0.0000	0.999785		0.0000	0.0000022062
ss61519736	*rs41617880*	9	39.772317	0.7622	0.1535	0.695297		0.0081	0.0029406304
ss86283794		9	39.795848	0.2564	2.3782	0.123496		0.0326	0.0124406453
ss86293313		9	39.907135	0.6590	1.6122	0.204603		0.0255	0.0114520003
ss86314464		9	39.964298	0.3668	0.2248	0.635532		0.0089	0.0041516956
ss86285393		9	40.064188	0.6024	0.4162	0.519044		0.0121	0.0057843461
ss86288134		9	40.099385	0.1024	1.1732	0.279124		0.0332	0.0061109835
ss117969622		9	40.130044	0.4477	0.5582	0.455248		0.0141	0.0069672207
	rs29021351	9	40.153426	0.1232	0.4295	0.512472		0.0179	0.0038700176
ss86292384		9	40.264091	0.5480	0.0782	0.779856		0.0052	0.0025762313
ss61561097	*rs41656812*	9	40.318699	0.7794	0.7685	0.380981		0.0196	0.0067276252
ss86306288		9	40.366449	0.2113	0.5305	0.466645		0.0167	0.0055564318
ss61561103	*rs41656818*	9	40.393986	0.5086	0.0195	0.888923		0.0026	0.0013210927
ss61523758	*rs41622917*	9	40.425232	0.5143	1.9296	0.165251		0.0272	0.0135726590
ss86303646		9	40.49279	0.8625	0.4336	0.510466		0.0180	0.0042812782
ss86338862		9	40.512872	0.3804	0.0299	0.862729		0.0035	0.0016401194
ss86338437		9	40.635016	0.3567	0.5271	0.468084		0.0146	0.0067096939
ss86308061		9	40.663792	0.0824	2.1932	0.139077		0.0512	0.0077469851
ss86278481		9	40.948365	0.8503	0.0540	0.816266		0.0060	0.0015231076
ss61561115	*rs41656828*	9	41.010476	0.8890	0.0464	0.829443		0.0065	0.0012783820
ss86305877		9	41.10142	0.6705	0.8795	0.348672		0.0188	0.0082946964
ss61561118	*rs41657531*	9	41.138118	0.3460	1.4400	0.230543		0.0236	0.0106597169
	rs29017977	9	41.409832	0.2493	4.5826	0.032646		0.0454	0.0169873461
ss61493132	*rs41592677*	9	41.437448	0.1934	6.2035	0.012982		0.0580	0.0181031371
ss61561119	*rs41657532*	9	41.457793	0.8066	6.2035	0.012982		0.0580	0.0181031371
ss86326057		9	41.487011	0.7951	4.7000	0.030500		0.0493	0.0160601988
ss86331343		9	41.527926	0.4234	0.0091	0.923854		0.0018	0.0008717099
ss61493159	*rs41592704*	9	41.55644	0.5623	0.0121	0.912429		0.0020	0.0009940087
ss86292506		9	41.579852	0.5953	0.0008	0.978112		0.0005	0.0002447124

ss86331423		9	41.609335	0.1060	4.8119	0.028594	0.0676	0.0128136886
ss61525594	*rs41621801*	9	41.75401	0.4448	2.8175	0.093690	0.0304	0.0150184991
ss61476061	*rs41579424*	9	41.814129	0.5566	1.6453	0.200026	0.0242	0.0119438654
ss117969582		9	41.92103	0.9470	0.2781	0.598126	0.0219	0.0022008166
ss61476059	*rs41579422*	9	41.941788	0.3173	4.0575	0.044360	0.0392	0.0169810001
ss61520528	*rs41615552*	9	42.053079	0.0802	0.1522	0.696540	0.0134	0.0019796546
ss61525598	*rs41621805*	9	42.125337	0.0809	0.1180	0.731270	0.0118	0.0017516682
ss86290720		9	42.237213	0.0852	0.1177	0.731693	0.0114	0.0017853593
ss117969666		9	42.266193	0.1468	0.0466	0.829208	0.0057	0.0014260338
ss61521575	*rs41620478*	9	42.387103	0.9155	1.0304	0.310416	0.0334	0.0051617798
ss86298858		9	42.422904	0.7529	0.1262	0.722536	0.0076	0.0028243753
ss61521039	*rs41620058*	9	42.477656	0.4255	0.0126	0.910788	0.0021	0.0010196528
ss86336268		9	42.561961	0.4728	3.5509	0.059930	0.0345	0.0171995593
ss86326708		9	43.126818	0.3281	12.5038	0.000433	0.0689	0.0303768489
ss61521741	*rs41621841*	9	43.345586	0.1712	0.2764	0.599237	0.0131	0.0037171043
ss117964086		9	43.497787	0.2500	0.2218	0.637847	0.0102	0.0038421286
ss86287516		9	43.713693	0.7622	0.2377	0.626004	0.0105	0.0037918131
ss61524655	*rs41622185*	9	43.873504	0.7328	1.9757	0.160293	0.0293	0.0114775700
ss61524653	*rs41622183*	9	43.918321	0.7543	1.6841	0.194811	0.0280	0.0103653591
ss61563875	*rs41665040*	9	43.994654	0.4556	0.5138	0.473731	0.0132	0.0065573016
ss86307771		9	44.051017	0.1612	1.0640	0.302674	0.0268	0.0072573434
ss61548505	*rs41649709*	9	44.095822	0.7521	0.6117	0.434426	0.0171	0.0063606525
ss86289169		9	44.200288	0.5466	1.0703	0.301240	0.0192	0.0095061887
ss86282816		9	44.230587	0.8510	0.4283	0.513038	0.0176	0.0044547777
ss86330361		9	44.255942	0.7980	2.5332	0.111930	0.0373	0.0120303017
ss61561249	*rs41658661*	9	44.332571	0.7944	0.1686	0.681444	0.0097	0.0031732993
ss86296867		9	44.691417	0.7314	5.2773	0.021901	0.0487	0.0191388709
ss86340388		9	44.743824	0.2314	7.4344	0.006560	0.0596	0.0212079863
ss86330497		9	45.006567	0.7937	0.0330	0.856001	0.0041	0.0013436151
ss61511026	*rs41609952*	9	45.049572	0.7063	2.5733	0.109134	0.0314	0.0130402468
ss61512642	*rs41614446*	9	45.070471	0.7063	2.5733	0.109134	0.0314	0.0130402468
ss86336855		9	45.108957	0.7063	2.5733	0.109134	0.0314	0.0130402468
ss86336002		9	45.165435	0.8854	1.9184	0.166472	0.0411	0.0083314760
ss86273539		9	45.345901	0.6956	1.5906	0.207666	0.0246	0.0104307466
ss86292779		9	45.37325	0.6017	5.4627	0.019710	0.0430	0.0205873507
ss61494404	*rs41596704*	9	45.435722	0.6390	5.8125	0.016170	0.0466	0.0214781618
	rs29009884	9	45.457232	0.5809	4.8606	0.027803	0.0392	0.0190927343
ss86306487		9	45.497534	0.5895	3.1852	0.074743	0.0320	0.0155054460
ss61496842	*rs41592785*	9	45.553408	0.4097	3.1355	0.077043	0.0318	0.0153637060
ss86288121		9	45.590254	0.5895	3.1852	0.074743	0.0320	0.0155054460
ss86316334		9	45.887171	0.7034	3.3683	0.066890	0.0362	0.0151138359
ss86312880		9	46.023799	0.3539	2.8513	0.091749	0.0323	0.0147778234
ss86322943		9	46.362363	0.2615	1.8348	0.176007	0.0282	0.0109009596
ss61472628	*rs41572851*	9	46.423923	0.8410	1.4896	0.222689	0.0323	0.0086319617
ss61516807	*rs41618807*	9	46.464059	0.4635	0.5560	0.456131	0.0138	0.0068433689
ss86308098		9	46.521058	0.4699	2.0220	0.155482	0.0259	0.0129065967
ss61511032	*rs41609958*	9	46.58059	0.2486	2.7378	0.098451	0.0354	0.0132169750

ss38332880	*rs29016380*	9	46.600974	0.6282	0.1155	0.734105	0.0065	0.0030496926
ss86291660		9	46.695809	0.3811	5.4683	0.019647	0.0448	0.0211525289
ss61504739	*rs41609211*	9	46.773784	0.5086	0.8904	0.345687	0.0184	0.0091759461
ss117964095		9	46.80796	0.1182	1.3863	0.239425	0.0350	0.0072997110
ss61493200	*rs41593345*	9	46.879988	0.5666	0.9674	0.325675	0.0188	0.0092317617
ss61566463	*rs41664293*	9	46.984915	0.9341	2.8652	0.090964	0.0641	0.0078979318
ss86305342		9	47.107876	0.5265	6.9141	0.008741	0.0490	0.0244338491
	rs29026560	9	47.205532	0.0673	2.3694	0.124190	0.0578	0.0072648705
ss117964036		9	47.307856	0.4721	0.6971	0.404035	0.0162	0.0080785512
ss61469386	*rs41569493*	9	47.344924	0.1082	2.3299	0.127368	0.0454	0.0087576915
ss86334564		9	47.465436	0.0623	0.0971	0.755480	0.0119	0.0013955619
ss86302682		9	47.588816	0.1332	3.2204	0.073162	0.0494	0.0114053363
ss61470207	*rs41567899*	9	47.675618	0.5702	0.2225	0.637309	0.0088	0.0043037728
ss117969745		9	47.704649	0.4298	0.2225	0.637309	0.0088	0.0043037728
ss86315907		9	47.873757	0.1347	0.0004	0.984753	0.0005	0.0001229843
ss61516045	*rs41610971*	9	47.895434	0.0946	3.3776	0.066515	0.0598	0.0102392884
ss61498155	*rs43708865*	9	47.934344	0.7020	1.4307	0.232065	0.0240	0.0100391206
ss61561264	*rs41658676*	9	48.020408	0.1053	0.0474	0.827642	0.0067	0.0012582724
ss117969708		9	48.117824	0.8732	0.6680	0.414027	0.0231	0.0051163648
ss86336886		9	48.28345	0.1626	0.8612	0.353733	0.0241	0.0065711068
ss61561278		9	48.362593	0.2772	0.4375	0.508566	0.0134	0.0053574652
ss61477393	*rs41575929*	9	48.721582	0.8130	0.0391	0.843352	0.0046	0.0014000316
ss61493204	*rs41593348*	9	48.756253	0.1870	0.0391	0.843352	0.0046	0.0014000316
ss61561297	*rs41658708*	9	48.842987	0.3789	0.3538	0.552152	0.0112	0.0052897825
ss61561301	*rs41658712*	9	48.903381	0.6304	0.1406	0.707783	0.0073	0.0033839293
ss61523764	*rs41622923*	9	48.934407	0.4670	1.0707	0.301154	0.0192	0.0095633059
ss61550714	*rs41653485*	9	49.17514	0.2185	0.0451	0.831899	0.0048	0.0016546118
ss86274230		9	49.218698	0.5380	0.0111	0.916310	0.0019	0.0009628655
ss86307500		9	49.463461	0.6533	0.8709	0.351044	0.0184	0.0083302108
ss61552628	*rs43711680*	9	49.652895	0.2615	0.0476	0.827357	0.0046	0.0017890493
ss61489051	*rs41589490*	9	49.682842	0.2213	0.3810	0.537249	0.0136	0.0047038956
ss61510065	*rs41601212*	9	49.704768	0.1862	0.5461	0.460181	0.0171	0.0051802047
ss61468702	*rs41570813*	9	49.803632	0.7622	0.1882	0.664524	0.0095	0.0034393264
ss61552624	*rs43711676*	9	49.902303	0.2378	0.1882	0.664524	0.0095	0.0034393264
ss86318327		9	50.183018	0.3317	0.0055	0.941126	0.0014	0.0006225128
ss86292134		9	50.229934	0.5394	0.6707	0.413088	0.0151	0.0074933636
ss61561306	*rs41658717*	9	50.326472	0.6777	1.8967	0.168890	0.0268	0.0117193587
ss86273588		9	50.389542	0.6769	0.4865	0.485718	0.0135	0.0058982791
ss65514694	*rs43600077*	9	50.410126	0.3868	0.4486	0.503213	0.0126	0.0059639383
ss65514729	*rs43600112*	9	50.434276	0.6153	0.5925	0.441710	0.0144	0.0068243180
ss65514769	*rs43591055*	9	50.483385	0.3130	1.0272	0.311180	0.0201	0.0086606237
ss65514792	*rs43591078*	9	50.506104	0.8954	0.0229	0.879769	0.0045	0.0008452628
ss65514820	*rs43591106*	9	50.539661	0.1433	0.4993	0.480049	0.0185	0.0045427679
	rs29021754	9	50.597261	0.1590	0.0396	0.842392	0.0051	0.0013524026
ss64756585	*rs42853453*	9	50.832907	0.5387	0.0064	0.936115	0.0015	0.0007311526
ss86340233		9	50.89258	0.2894	0.1028	0.748567	0.0065	0.0026859202
ss64102867	*rs42476458*	9	50.94033	0.1483	0.3353	0.562748	0.0151	0.0038217837

ss38323912	*rs29012814*	9	51.02874	0.8130	0.1044	0.746672	0.0076	0.0023066833
ss64103473	*rs42478960*	9	51.105284	0.8782	1.4740	0.225128	0.0342	0.0073194771
ss64103613	*rs42479030*	9	51.127931	0.2178	1.4079	0.235803	0.0258	0.0087812790
ss64181859	*rs42513591*	9	51.150753	0.6898	0.3276	0.567240	0.0114	0.0048757453
ss117969639		9	51.178941	0.7872	1.3613	0.243703	0.0255	0.0085430748
ss61561311	*rs41658722*	9	51.288089	0.1074	1.1963	0.274443	0.0329	0.0063174473
ss65515214	*rs43595191*	9	51.309234	0.7679	0.0654	0.798275	0.0057	0.0020194018
ss61494289	*rs41595692*	9	51.342317	0.4534	0.2943	0.587655	0.0100	0.0049662808
ss65021860	*rs43117405*	9	51.375991	0.2808	1.3362	0.248098	0.0242	0.0097807073
ss61494286	*rs41595689*	9	51.409122	0.8596	0.0076	0.930546	0.0023	0.0005639006
ss61563625	*rs41662696*	9	51.438155	0.1397	0.0101	0.920105	0.0027	0.0006435055
ss61563622	*rs41662693*	9	51.460142	0.1404	0.0587	0.808620	0.0065	0.0015584843
ss86281193		9	51.493578	0.2170	4.3154	0.038135	0.0454	0.0154389420
ss46526560	*rs41257018*	9	51.556482	0.2958	1.0835	0.298278	0.0212	0.0088281673
ss86323004		9	51.639399	0.2056	2.4697	0.116514	0.0358	0.0117021845
ss64091875	*rs42466129*	9	51.661514	0.6397	1.3254	0.250013	0.0216	0.0099789819
ss64091819	*rs42466101*	9	51.688446	0.5437	0.0828	0.773642	0.0052	0.0025910476
ss64091665	*rs42472925*	9	51.829789	0.5738	0.7145	0.398258	0.0161	0.0078548195
ss61561307	*rs41658718*	9	51.89141	0.4864	0.5110	0.474947	0.0132	0.0065982336
ss61506983	*rs41601387*	9	51.990056	0.1239	0.0032	0.955035	0.0016	0.0003420574
	rs29023608	9	52.100143	0.5272	0.8825	0.347855	0.0170	0.0084697560
ss65515097	*rs43594476*	9	52.140769	0.7056	1.4052	0.236255	0.0237	0.0098422866
ss65515105	*rs43594484*	9	52.160813	0.6991	1.7359	0.188090	0.0260	0.0109563760
ss64905329	*rs42995339*	9	52.181591	0.6182	0.1319	0.716613	0.0070	0.0032941917
ss86328476		9	52.205823	0.6791	1.3595	0.244028	0.0225	0.0098064114
ss86331136		9	52.240034	0.8030	6.0678	0.014008	0.0574	0.0181700323
ss61561345		9	52.271111	0.6648	10.4999	0.001251	0.0610	0.0272021436
ss117964065		9	52.31434	0.2507	0.3698	0.543324	0.0134	0.0050306080
ss65515732	*rs43600707*	9	52.342854	0.2457	0.0161	0.899219	0.0028	0.0010345432
ss61561348	*rs41659758*	9	52.379947	0.2292	8.5756	0.003518	0.0637	0.0225249260
ss65515766	*rs43595845*	9	52.410202	0.2192	0.0386	0.844272	0.0045	0.0015477399
ss61561491	*rs41661101*	9	52.432832	0.5143	3.5176	0.061138	0.0364	0.0181666559
ss65520352	*rs43604391*	9	52.475303	0.5888	4.9138	0.026965	0.0423	0.0204703461
ss65520326	*rs43604365*	9	52.502822	0.5888	4.9138	0.026965	0.0423	0.0204703461
ss61561483	*rs41661093*	9	52.588472	0.2299	8.4740	0.003718	0.0634	0.0224363444
ss65520234	*rs43603273*	9	52.62622	0.2278	11.0000	0.000958	0.0721	0.0253595853
ss86332875		9	52.658395	0.7901	8.9702	0.002842	0.0668	0.0221533239
ss86292616		9	52.684866	0.4090	7.0332	0.008184	0.0485	0.0234419881
ss61523768	*rs41622927*	9	52.865467	0.1662	3.8807	0.049239	0.0493	0.0136717585
ss64948239	*rs43045326*	9	52.890628	0.6905	4.9551	0.026333	0.0434	0.0185610981
ss86333843		9	52.912817	0.8489	1.1166	0.291015	0.0273	0.0070024862
ss86274121		9	52.973575	0.6261	9.0595	0.002708	0.0552	0.0258619801
ss61525625	*rs41621932*	9	53.120451	0.1676	0.0000	0.997923	0.0001	0.0000180642
ss61525621	*rs41621828*	9	53.145976	0.9262	0.7882	0.374945	0.0315	0.0043042296
ss61525623	*rs41621830*	9	53.168292	0.4549	1.8018	0.179934	0.0247	0.0122566095
ss117969547		9	53.198488	0.5093	15.2467	0.000104	0.0707	0.0353581275
ss63964351	*rs42408909*	9	53.425067	0.5573	6.1116	0.013668	0.0458	0.0226208600

ss86287472		9	53.486208	0.1196	0.4600	0.497871	0.0195	0.0041161502
ss61515590	*rs41615943*	9	53.521096	0.5802	3.9174	0.048183	0.0365	0.0177904604
ss86335564		9	53.550146	0.5208	4.0165	0.045443	0.0363	0.0181142228
ss86327380		9	53.580189	0.1848	12.1708	0.000516	0.0823	0.0248005501
ss117964089		9	53.619232	0.1476	0.1189	0.730290	0.0091	0.0022999683
ss86299565		9	53.659431	0.1569	4.1542	0.041908	0.0508	0.0134499438
ss61470936	*rs41574304*	9	53.682387	0.1562	4.0401	0.044816	0.0502	0.0132287034
ss61513435	*rs41612115*	9	53.711858	0.3288	1.1576	0.282329	0.0216	0.0095208723
ss61505387	*rs41606134*	9	53.859717	0.3918	6.4639	0.011224	0.0486	0.0231691063
ss63720818	*rs42321632*	9	53.888113	0.8009	0.1859	0.666508	0.0098	0.0031181541
ss63720626	*rs42321070*	9	53.947222	0.2063	0.1941	0.659647	0.0098	0.0031947954
	rs29020404	9	53.993832	0.1913	0.0151	0.902278	0.0030	0.0009145448
ss86289247		9	54.107052	0.4234	11.2050	0.000860	0.0627	0.0305904924
ss61497765	*rs43704787*	9	54.176473	0.3095	0.3242	0.569269	0.0114	0.0048663122
ss86277885		9	54.247544	0.1361	1.9211	0.166175	0.0375	0.0088074361
ss63745772	*rs42332072*	9	54.320602	0.1017	8.0183	0.004765	0.0874	0.0159659548
ss86319435		9	54.361071	0.1956	6.1609	0.013295	0.0579	0.0182083617
ss63744950	*rs42329999*	9	54.41318	0.3130	0.3402	0.559884	0.0115	0.0049564858
ss63893102	*rs41912442*	9	54.497531	0.2966	13.9298	0.000205	0.0742	0.0309735761
ss63893140	*rs41912461*	9	54.526638	0.2872	12.5412	0.000425	0.0722	0.0295760930
ss117975276		9	54.652754	0.3854	0.7082	0.400332	0.0158	0.0074647110
ss63744590	*rs42336479*	9	54.725193	0.7464	2.0451	0.153145	0.0313	0.0118490194
ss63828946	*rs42371824*	9	54.898937	0.3754	8.0404	0.004707	0.0540	0.0253423444
	rs29017433	9	54.939267	0.3818	8.0212	0.004757	0.0535	0.0252762877
ss61473201	*rs41567620*	9	54.979053	0.7958	1.9647	0.161457	0.0317	0.0103143001
ss61518158	*rs41611327*	9	55.001487	0.2013	2.5998	0.107328	0.0369	0.0118523752
ss63829166	*rs42373432*	9	55.054595	0.1103	0.9149	0.339137	0.0288	0.0056624667
ss61518160	*rs41611329*	9	55.07533	0.6848	6.8345	0.009135	0.0535	0.0230794970
ss63829244	*rs42373471*	9	55.096609	0.7278	5.4893	0.019414	0.0493	0.0195176047
ss86313014		9	55.121685	0.6977	5.6431	0.017794	0.0490	0.0206682471
ss61506558	*rs41607066*	9	55.147064	0.3388	0.6772	0.410829	0.0164	0.0073266439
ss61518164	*rs41612133*	9	55.176579	0.5523	7.5826	0.006047	0.0525	0.0259486486
ss86337046		9	55.211567	0.5158	3.9135	0.048293	0.0375	0.0187240286
ss63830086	*rs42369791*	9	55.267684	0.2701	0.2962	0.586446	0.0112	0.0044131475
ss86323969		9	55.422621	0.3868	0.3476	0.555644	0.0115	0.0054494166
ss61493215	*rs41593359*	9	55.45113	0.6383	6.5367	0.010778	0.0505	0.0233252805
ss61493219	*rs41593363*	9	55.486403	0.2192	1.9242	0.165844	0.0311	0.0106514122
ss65516099	*rs43597270*	9	55.516173	0.8052	2.0608	0.151583	0.0339	0.0106338408
ss65516117	*rs43597288*	9	55.577834	0.1934	2.4011	0.121703	0.0364	0.0113720872
ss65516143	*rs43597314*	9	55.607087	0.1232	3.0209	0.082638	0.0482	0.0104099979
	rs29021602	9	55.659014	0.6870	0.0302	0.862096	0.0035	0.0014969035
	rs29020429	9	55.73827	0.2779	3.1619	0.075811	0.0366	0.0146999405
ss117969759		9	55.856081	0.4026	8.8282	0.003068	0.0571	0.0274899344
ss61493221	*rs41593365*	9	55.904551	0.4427	8.0109	0.004784	0.0532	0.0262339831
ss61525808	*rs41622511*	9	56.005621	0.8417	2.9486	0.086399	0.0437	0.0116543416
ss61522513	*rs41619884*	9	56.055016	0.8403	3.5573	0.059700	0.0474	0.0127149195
ss61478425	*rs41575824*	9	56.108838	0.1590	0.1232	0.725728	0.0088	0.0023594564

ss63970433	*rs42411844*	9	56.131285	0.6819	4.6578	0.031254	0.0444	0.0192604250
ss117964039		9	56.206601	0.5330	1.7147	0.190803	0.0246	0.0122402954
ss86296693		9	56.244449	0.1791	1.4522	0.228591	0.0288	0.0084548628
ss61525809		9	56.284119	0.0953	0.0004	0.984055	0.0006	0.0001083940
ss63970231	*rs42411044*	9	56.305152	0.2937	6.6335	0.010213	0.0538	0.0223145128
ss63970205	*rs42411031*	9	56.344928	0.7006	7.5098	0.006294	0.0573	0.0240314187
ss65517206	*rs43599782*	9	56.369308	0.3016	5.8736	0.015623	0.0502	0.0211315310
ss65517186	*rs43599762*	9	56.44838	0.5552	3.0325	0.082052	0.0322	0.0159105925
ss65517149	*rs43598825*	9	56.492483	0.3496	9.5977	0.002027	0.0615	0.0279778981
ss86285251		9	56.523385	0.7858	0.2369	0.626609	0.0109	0.0036597085
ss64854007	*rs42950972*	9	56.550397	0.2779	2.2528	0.133830	0.0312	0.0125135686
ss86340637		9	56.580849	0.2142	0.2369	0.626609	0.0109	0.0036597085
ss64894921	*rs42989505*	9	56.601043	0.4842	4.9291	0.026729	0.0416	0.0207587444
ss117969686		9	56.734583	0.7163	3.5917	0.058481	0.0390	0.0158382755
ss65517058	*rs43598734*	9	56.78344	0.3001	0.1837	0.668373	0.0086	0.0036040870
	rs29026815	9	56.830046	0.6791	0.8983	0.343569	0.0188	0.0081908271
ss64938901	*rs43032893*	9	56.851107	0.0938	1.3147	0.251934	0.0362	0.0061602540
ss61561357	*rs41659767*	9	56.922714	0.0910	0.9355	0.333771	0.0309	0.0051099112
ss61561356	*rs41659766*	9	56.960718	0.3345	0.0037	0.951668	0.0012	0.0005247023
ss65516908	*rs43596784*	9	56.984971	0.6576	0.0053	0.941969	0.0014	0.0006368013
ss65516773	*rs43595950*	9	57.03066	0.1483	0.2053	0.650618	0.0118	0.0029731188
ss61561355	*rs41659765*	9	57.06656	0.7314	0.1946	0.659216	0.0093	0.0036560148
ss65516665	*rs43601136*	9	57.110578	0.8646	0.0000	0.995233	0.0002	0.0000367992
ss117969560		9	57.185609	0.4957	0.7466	0.387856	0.0159	0.0079574426
ss65516381	*rs43599152*	9	57.318411	0.9033	0.3293	0.566264	0.0181	0.0031626123
ss65516366	*rs43599137*	9	57.351979	0.3539	5.1834	0.023106	0.0438	0.0200521177
ss65516239	*rs43598010*	9	57.413566	0.8052	1.1431	0.285363	0.0245	0.0076904807
ss64188295	*rs42516892*	9	57.480772	0.7629	0.8854	0.347057	0.0209	0.0075561313
ss64535582	*rs42686238*	9	57.515851	0.2371	0.8854	0.347057	0.0209	0.0075561313
ss61514379	*rs41611752*	9	57.550085	0.0981	0.0241	0.876620	0.0048	0.0008491211
ss61514383	*rs41611756*	9	57.595191	0.7701	0.4015	0.526510	0.0141	0.0049911435
ss65025391	*rs43113305*	9	57.618813	0.7708	0.4978	0.480692	0.0158	0.0055694531
ss61563774	*rs41664043*	9	57.643837	0.2285	0.4765	0.490264	0.0154	0.0054378670
ss86278315		9	57.675776	0.7693	0.2891	0.590954	0.0118	0.0041984362
ss64188933	*rs42519306*	9	57.697893	0.2844	0.6810	0.409518	0.0169	0.0068918774
ss64845374	*rs42937117*	9	57.732397	0.2049	5.2739	0.021943	0.0518	0.0168739780
ss64734435	*rs42827092*	9	57.796887	0.3503	0.1899	0.663117	0.0087	0.0039442056
ss64734477	*rs42827834*	9	57.828665	0.2013	0.0136	0.907249	0.0027	0.0008750014
ss61478475	*rs41576073*	9	57.911531	0.5845	0.4608	0.497468	0.0130	0.0063039583
ss61530574	*rs41626645*	9	58.04276	0.2314	10.3728	0.001338	0.0691	0.0245750154
ss64091095	*rs42470942*	9	58.073653	0.1819	7.7613	0.005483	0.0671	0.0199808676
ss64090993	*rs42470591*	9	58.105835	0.0931	2.1806	0.140209	0.0463	0.0078283360
ss86327679		9	58.200423	0.6254	1.9178	0.166538	0.0262	0.0122901539
ss61493226	*rs41593369*	9	58.255092	0.5573	0.7775	0.378201	0.0162	0.0080164998
ss64550956	*rs42690572*	9	58.380745	0.3990	2.4947	0.114682	0.0303	0.0145500972
ss63670121	*rs42307752*	9	58.626197	0.8281	2.8388	0.092458	0.0406	0.0115542701
ss65039960	*rs43131902*	9	58.651974	0.8288	2.1728	0.140923	0.0356	0.0100942802

ss61569436	*rs41667217*	9	58.686543	0.7192	10.1687	0.001492	0.0655	0.0264710086
ss61497187	*rs41594616*	9	58.722783	0.5408	2.4346	0.119137	0.0288	0.0143012385
ss63671370	*rs42309569*	9	58.873624	0.1110	2.1978	0.138662	0.0433	0.0085551900
ss63671586	*rs42310141*	9	58.936254	0.8897	3.5535	0.059835	0.0547	0.0107438804
ss63671835	*rs42310224*	9	58.974749	0.1862	2.0195	0.155743	0.0341	0.0103453850
ss63671760	*rs42310199*	9	59.005978	0.9513	1.8863	0.170062	0.0582	0.0053958554
ss63672015	*rs42310584*	9	59.031537	0.5509	6.0211	0.014380	0.0453	0.0223938046
ss63672153	*rs42306231*	9	59.061472	0.4355	3.8622	0.049783	0.0362	0.0178081721
ss64869475	*rs42961160*	9	59.200869	0.1461	1.0303	0.310434	0.0269	0.0067156655
ss86314616		9	59.261542	0.8016	0.0050	0.943575	0.0017	0.0005385717
ss64908264	*rs43001239*	9	59.282563	0.0881	0.9474	0.330729	0.0320	0.0051465268
ss61493229	*rs41593372*	9	59.362078	0.6533	0.0045	0.946530	0.0013	0.0005985229
ss86337229		9	59.404535	0.1211	0.0000	0.998750	0.0000	0.0000094528
ss64252802	*rs42549324*	9	59.4254	0.9162	6.3080	0.012245	0.0808	0.0124066234
ss61566018		9	59.455174	0.4319	7.8075	0.005346	0.0524	0.0257327091
ss64882585	*rs42977412*	9	59.513271	0.7758	0.0273	0.868819	0.0037	0.0012814005
ss86278658		9	59.572683	0.2364	4.8402	0.028131	0.0482	0.0174163600
ss64252886	*rs42550166*	9	59.614386	0.2443	5.7522	0.016730	0.0515	0.0190318889
ss65006296	*rs43095652*	9	59.675251	0.5573	0.8081	0.368999	0.0162	0.0080175365
ss61563280		9	59.696021	0.4248	1.4472	0.229379	0.0228	0.0111364440
ss64544872	*rs42689048*	9	59.759509	0.5143	0.4787	0.489262	0.0128	0.0063903275
ss64544830	*rs42688727*	9	59.823512	0.9477	1.0416	0.307795	0.0420	0.0041667095
ss61514514	*rs41613079*	9	59.86123	0.3410	9.0721	0.002689	0.0560	0.0251555094
ss64607068	*rs42723319*	9	59.893742	0.5523	0.0068	0.934276	0.0015	0.0007618783
ss61515650	*rs41616002*	9	59.924681	0.0480	1.3729	0.241716	0.0500	0.0045684351
ss64332227	*rs42591988*	9	60.00531	0.7729	1.0080	0.315736	0.0218	0.0076387807
ss61472631	*rs41572854*	9	60.045257	0.5523	1.6995	0.192789	0.0245	0.0121309796
ss86323512		9	60.1964	0.7851	7.8421	0.005246	0.0641	0.0216411416
ss61561392	*rs41659802*	9	60.287711	0.4054	2.4688	0.116581	0.0289	0.0139406431
ss65517433	*rs43601909*	9	60.33115	0.7643	1.4924	0.222255	0.0266	0.0095871047
	rs29015048	9	60.37666	0.2120	0.4499	0.502614	0.0152	0.0050793101
ss61561387	*rs41659797*	9	60.420897	0.4484	3.9362	0.047649	0.0355	0.0175546516
ss86339684		9	60.466519	0.8023	0.0029	0.956755	0.0012	0.0003786993
	rs29017608	9	60.508687	0.7564	0.0273	0.868700	0.0034	0.0012369826
ss61548419	*rs41648624*	9	60.616816	0.8195	0.1596	0.689627	0.0091	0.0027049025
ss62983263	*rs42059669*	9	60.721222	0.6017	0.0166	0.897459	0.0024	0.0011568470
ss61554263	*rs41651210*	9	60.819486	0.1497	0.3441	0.557685	0.0148	0.0037673418
ss86323449		9	60.843944	0.8546	0.5380	0.463513	0.0185	0.0046049576
ss61489900	*rs41587714*	9	60.898135	0.0817	0.7970	0.372287	0.0300	0.0044975625
	rs29014427	9	60.969755	0.4491	0.1788	0.672520	0.0078	0.0038485647
ss65517748	*rs43604219*	9	61.016788	0.3223	0.1037	0.747521	0.0062	0.0027213649
ss65518069	*rs43599414*	9	61.169079	0.7034	0.8423	0.359052	0.0181	0.0075539527
ss86290360		9	61.25267	0.8725	1.3965	0.237707	0.0328	0.0073066597
ss86322903		9	61.299966	0.6884	1.7745	0.183256	0.0260	0.0111627606
ss65518376	*rs43602021*	9	61.326951	0.2357	6.7749	0.009442	0.0570	0.0205310845
ss65518452	*rs43602597*	9	61.35197	0.9269	4.6820	0.030819	0.0768	0.0103990299
ss65518538	*rs43603583*	9	61.376418	0.0774	5.9777	0.014734	0.0848	0.0121035769

ss86300649		9	61.400014	0.8080	0.8471	0.357707	0.0213	0.0066166120
ss65518684	*rs43604729*	9	61.426424	0.3811	1.3630	0.243413	0.0220	0.0103817612
ss61561396	*rs41659806*	9	61.447029	0.6189	1.3630	0.243413	0.0220	0.0103817612
ss65518747	*rs43605692*	9	61.473313	0.5523	1.0990	0.294858	0.0193	0.0095486217
ss61474514	*rs41570215*	9	61.529985	0.6461	0.1501	0.698550	0.0076	0.0034595121
ss86323213		9	61.566331	0.8238	2.2314	0.135683	0.0369	0.0107178784
ss86339516		9	61.701831	0.1053	0.6316	0.427024	0.0233	0.0043947434
ss65518872	*rs43597117*	9	61.746009	0.2428	3.8012	0.051618	0.0427	0.0156938581
ss86327302		9	61.807478	0.8266	3.3459	0.067802	0.0433	0.0124146281
ss61561416	*rs41659826*	9	61.827562	0.4685	0.0017	0.966932	0.0008	0.0003775429
ss65518919	*rs43597863*	9	61.874926	0.3524	0.0001	0.992760	0.0002	0.0000818950
ss86286680		9	61.93199	0.5186	0.0755	0.783539	0.0051	0.0025662299
	rs29021263	9	61.952168	0.1132	0.3680	0.544298	0.0173	0.0034710991
	rs29024932	9	62.00338	0.7973	1.7126	0.191083	0.0294	0.0095081579
ss86296434		9	62.034433	0.9542	2.4053	0.121381	0.0673	0.0058911230
ss61561453	*rs41661063*	9	62.054644	0.5501	1.6932	0.193609	0.0239	0.0118183991
ss61493248	*rs41593391*	9	62.096134	0.1941	4.1090	0.043035	0.0472	0.0147654494
ss65519218	*rs43599859*	9	62.123127	0.5186	0.2194	0.639609	0.0087	0.0043236368
ss86334618		9	62.194887	0.4979	0.4857	0.486064	0.0127	0.0063316884
ss65519322	*rs43600462*	9	62.228278	0.6053	0.1743	0.676417	0.0077	0.0036704812
ss61561467	*rs41661077*	9	62.250901	0.1024	8.7782	0.003153	0.0862	0.0158523491
ss86332520		9	62.281443	0.6741	1.1427	0.285443	0.0213	0.0093778645
ss65520185	*rs43602225*	9	62.313226	0.6318	5.8198	0.016104	0.0463	0.0215578763
ss86314908		9	62.427474	0.1547	0.4463	0.504326	0.0176	0.0046056964
ss86286504		9	62.45949	0.1003	1.6965	0.193174	0.0410	0.0073908762
ss65519596	*rs43602035*	9	62.541982	0.9305	0.8641	0.352924	0.0346	0.0044741039
ss61493257	*rs41593400*	9	62.57206	0.2385	11.7874	0.000632	0.0734	0.0266576545
ss65519638	*rs43602077*	9	62.601103	0.0938	0.0065	0.935902	0.0026	0.0004364695
ss65519657	*rs43602096*	9	62.623191	0.7622	10.4412	0.001290	0.0691	0.0250596756
ss65519679	*rs43602118*	9	62.646204	0.0723	1.0155	0.313935	0.0369	0.0049523387
ss61561475	*rs41661085*	9	62.672003	0.5559	0.6593	0.417088	0.0152	0.0075163384
ss86332889		9	62.69734	0.8517	4.4486	0.035287	0.0538	0.0135832413
ss61561476	*rs41661086*	9	62.84833	0.3926	3.7759	0.052398	0.0369	0.0175843922
ss86308587		9	62.910337	0.7994	0.5986	0.439392	0.0178	0.0057109952
ss65519950	*rs43600193*	9	62.991283	0.3223	0.0061	0.937775	0.0015	0.0006695725
	rs29027423	9	63.019666	0.2708	2.0917	0.148550	0.0300	0.0118499216
ss65520010	*rs43601252*	9	63.074296	0.2951	1.4095	0.235547	0.0241	0.0100194740
ss61561478	*rs41661088*	9	63.150202	0.1633	0.0159	0.899842	0.0031	0.0008389964
	rs29017340	9	63.181337	0.6196	0.0380	0.845541	0.0038	0.0017853438
ss86288945		9	63.235582	0.8682	2.6236	0.105741	0.0452	0.0103467402
ss65520412	*rs43605351*	9	63.302111	0.6547	1.1279	0.288601	0.0208	0.0094008469
ss86308755		9	63.352121	0.1433	14.4990	0.000153	0.0981	0.0240799148
ss65520459	*rs43605398*	9	63.380539	0.4728	1.1331	0.287481	0.0200	0.0099520385
ss61504789	*rs41599664*	9	63.403595	0.5895	0.4431	0.505870	0.0126	0.0061155082
ss65520725	*rs43608864*	9	63.436436	0.3711	0.1986	0.655990	0.0085	0.0039878147
ss86320674		9	63.467364	0.8875	15.8021	0.000078	0.1152	0.0229987587
ss86299258		9	63.559034	0.3711	0.9078	0.341033	0.0181	0.0084298041

ss86337128		9	63.674372	0.4943	2.2097	0.137596	0.0270	0.0135200561
ss86291780		9	63.830138	0.0838	4.0852	0.043643	0.0669	0.0102755565
ss117969569		9	64.42151	0.4613	0.1314	0.717143	0.0068	0.0033720135
ss86340407		9	64.446096	0.5430	0.8092	0.368669	0.0168	0.0083406694
	rs29022960	9	64.535566	0.5781	1.2144	0.270840	0.0206	0.0100355713
ss61561500	*rs41661110*	9	64.585194	0.5394	0.0768	0.781813	0.0052	0.0025669049
	rs29025947	9	64.68953	0.3883	0.0689	0.793091	0.0049	0.0023408321
ss65520949	*rs43600388*	9	64.722212	0.4771	4.1750	0.041402	0.0376	0.0187467814
ss117969729		9	64.770217	0.7414	4.7489	0.029652	0.0454	0.0174179369
ss86298670		9	64.792016	0.3023	3.0808	0.079663	0.0344	0.0144927274
ss61493273	*rs41593416*	9	64.816075	0.6117	2.6444	0.104370	0.0297	0.0141140399
	rs29019504	9	64.867433	0.9277	3.9024	0.048612	0.0713	0.0095665015
ss86300167		9	64.904381	0.7364	1.6216	0.203294	0.0267	0.0103540773
ss64660019	*rs42756668*	9	65.017751	0.7393	0.1620	0.687413	0.0084	0.0032356084
ss86337489		9	65.094401	0.5387	2.5796	0.108703	0.0298	0.0148326572
ss61569045	*rs41664735*	9	65.116499	0.5122	4.4150	0.035982	0.0389	0.0194350659
ss86328804		9	65.148991	0.8539	8.2293	0.004247	0.0732	0.0182784477
ss64691370	*rs42788303*	9	65.180643	0.8582	7.4592	0.006471	0.0703	0.0171035011
ss64770535	*rs42865669*	9	65.209003	0.2471	0.7532	0.385752	0.0188	0.0070077021
ss86283636		9	65.260825	0.7693	0.0009	0.976047	0.0007	0.0002367037
ss86296818		9	65.382846	0.0831	0.5862	0.444154	0.0250	0.0038116324
ss86292575		9	65.436237	0.1067	5.9751	0.014757	0.0725	0.0138257840
	rs29026475	9	65.582785	0.2529	3.5040	0.061641	0.0400	0.0151250895
ss64748068	*rs42844501*	9	65.609208	0.6332	0.4029	0.525803	0.0125	0.0058081399
ss86339139		9	65.790393	0.3610	0.6485	0.420936	0.0157	0.0072563553
ss64170033	*rs42507734*	9	65.813375	0.3617	0.5064	0.476921	0.0140	0.0064627191
ss61563135	*rs41658528*	9	65.841043	0.4728	0.1375	0.710902	0.0068	0.0033778546
ss64587080	*rs42715661*	9	66.045863	0.0587	8.2549	0.004188	0.1122	0.0124120861
	rs29024610	9	66.065874	0.6082	0.0002	0.989492	0.0003	0.0001235956
ss86302344		9	66.107037	0.2837	2.5253	0.112490	0.0324	0.0131516509
ss64764086	*rs42858549*	9	66.128743	0.8918	0.3287	0.566585	0.0167	0.0032127073
ss117966061		9	66.156867	0.7529	0.2689	0.604219	0.0108	0.0040103052
ss65521070	*rs43601408*	9	66.238326	0.9277	1.5480	0.213850	0.0449	0.0060325436
ss86341465		9	66.26284	0.6404	0.8560	0.355193	0.0177	0.0081426371
ss117969545		9	66.303348	0.2693	0.3211	0.571111	0.0115	0.0045364228
ss61561503	*rs41661113*	9	66.340188	0.4413	0.3367	0.561903	0.0105	0.0051949625
ss86328537		9	66.360745	0.4441	0.1344	0.714022	0.0067	0.0033007281
ss61511043	*rs41609969*	9	66.40163	0.4191	0.6793	0.410126	0.0152	0.0074073012
ss86307082		9	66.49743	0.3209	0.5946	0.440922	0.0147	0.0064055358
ss86341287		9	66.517819	0.7278	0.0002	0.989177	0.0003	0.0001088354
ss86302348		9	66.561163	0.3789	0.2928	0.588612	0.0101	0.0047757403
ss65521715	*rs43608953*	9	66.624656	0.4463	0.0142	0.905256	0.0022	0.0010668799
ss61469392	*rs41569499*	9	66.647571	0.4463	0.0142	0.905256	0.0022	0.0010668799
ss117969723		9	66.690354	0.9312	3.0383	0.081762	0.0620	0.0079450537
ss61474777	*rs41572569*	9	66.821259	0.0881	0.1258	0.722941	0.0117	0.0018790539
	rs29009579	9	66.989154	0.8574	1.9255	0.165699	0.0346	0.0084632729
ss86302198		9	67.129908	0.4950	0.1987	0.655892	0.0081	0.0040704317

ss61561516	*rs41661126*	9	67.183717	0.4986	0.3380	0.561169	0.0105	0.0052720530
	rs29016449	9	67.217947	0.4871	4.9764	0.026013	0.0411	0.0205488869
	rs29014990	9	67.292607	0.7063	0.0212	0.884151	0.0029	0.0011941793
ss86315120		9	67.397196	0.7579	0.0710	0.789954	0.0057	0.0020745972
ss86280803		9	67.470723	0.6862	5.7020	0.017211	0.0474	0.0204189315
ss86289371		9	67.514672	0.8832	5.3394	0.021140	0.0649	0.0133946707
ss61534034	*rs41631595*	9	67.711413	0.7221	1.2429	0.265305	0.0229	0.0091976953
ss61534032	*rs41631593*	9	67.798021	0.3653	3.0975	0.078850	0.0336	0.0155876028
ss61534029	*rs41631590*	9	67.979832	0.2507	1.3512	0.245460	0.0241	0.0090714242
ss61527917	*rs41627858*	9	68.07249	0.3016	0.6193	0.431590	0.0159	0.0066927810
ss117969598		9	68.120471	0.9348	0.1908	0.662384	0.0167	0.0020309965
ss117969515		9	68.242583	0.4778	0.0009	0.976118	0.0006	0.0002874961
ss86336474		9	68.270966	0.5917	0.0320	0.858127	0.0035	0.0016815731
ss86310002		9	68.299084	0.5315	0.0028	0.957621	0.0010	0.0005049919
ss86301859		9	68.411525	0.1834	2.0972	0.148019	0.0344	0.0102905342
ss61527691	*rs41625944*	9	68.66732	0.6282	6.6713	0.010001	0.0488	0.0228182387
ss86327752		9	68.959084	0.4269	0.7712	0.380162	0.0158	0.0077110683
ss61523771	*rs41622930*	9	69.097619	0.3152	0.4262	0.514085	0.0127	0.0054846483
	rs29011074	9	69.688196	0.8997	3.5848	0.058723	0.0566	0.0102103531
ss61493292	*rs41593429*	9	69.718441	0.8209	3.0662	0.080378	0.0419	0.0123161288
ss86308611		9	69.742781	0.1132	4.1649	0.041647	0.0575	0.0115352798
ss117969608		9	70.039544	0.1920	5.3108	0.021487	0.0540	0.0167502659
	rs29009858	9	70.056929	0.4585	9.7526	0.001865	0.0588	0.0291874178
	rs29022461	9	70.13819	0.8030	6.2534	0.012624	0.0586	0.0185394426
ss61493296	*rs41594133*	9	70.171346	0.9133	2.0324	0.154425	0.0462	0.0073149300
ss86314335		9	70.209736	0.5766	0.1779	0.673279	0.0081	0.0039662831
ss61493298	*rs41594135*	9	70.270213	0.0895	2.0189	0.155797	0.0452	0.0073641611
ss86302226		9	70.301901	0.6691	0.0138	0.906478	0.0023	0.0010119685
ss61561530	*rs41662440*	9	70.354793	0.9169	3.7655	0.052723	0.0638	0.0097148403
ss61561531	*rs41662441*	9	70.378981	0.7163	2.4523	0.117810	0.0310	0.0125936107
	rs29022215	9	70.552066	0.6920	0.0351	0.851385	0.0037	0.0015837263
ss86294050		9	70.57828	0.2615	0.1272	0.721415	0.0073	0.0028210801
	rs29016144	9	70.733934	0.4821	2.0063	0.157097	0.0257	0.0128554932
	rs29011422	9	70.76041	0.1676	0.0088	0.925316	0.0024	0.0006578093
ss86307420		9	70.782445	0.1877	5.6906	0.017322	0.0548	0.0166983821
ss86326761		9	70.881572	0.6039	7.0633	0.008049	0.0492	0.0235332726
ss117964059		9	70.976752	0.2421	0.1180	0.731297	0.0076	0.0027826166
ss61561540	*rs41662450*	9	71.016975	0.4491	12.5115	0.000431	0.0639	0.0316412025
ss117969585		9	71.105688	0.9477	1.5118	0.219287	0.0506	0.0050193200
ss61523773	*rs41623532*	9	71.328646	0.4362	1.4527	0.228505	0.0223	0.0109591047
ss61561549	*rs41662459*	9	71.362852	0.5021	4.8304	0.028290	0.0401	0.0200379967
ss117969677		9	71.435002	0.1497	6.8921	0.008848	0.0646	0.0164392394
ss61561552	*rs41662462*	9	71.554874	0.3209	5.4544	0.019802	0.0451	0.0196627641
ss86295435		9	71.595319	0.2436	6.8417	0.009098	0.0553	0.0203869396
ss86336039		9	71.62219	0.6540	6.1785	0.013165	0.0470	0.0212765203
ss86337737		9	71.680317	0.0888	0.1631	0.686405	0.0133	0.0021480420
ss61493300	*rs41594137*	9	71.707806	0.9083	0.3699	0.543234	0.0198	0.0032901114

ss61470880	*rs41574248*	9	71.734132	0.3983	0.2819	0.595609	0.0101	0.0048336949
ss61465484	*rs41566731*	9	71.767225	0.6898	1.1341	0.287268	0.0212	0.0090602082
ss117969740		9	71.811035	0.1426	0.6719	0.412664	0.0210	0.0051346244
ss86340941		9	72.056987	0.7128	5.6568	0.017657	0.0477	0.0195290844
ss61496915	*rs41593554*	9	72.111835	0.0759	0.6232	0.430126	0.0269	0.0037707804
ss61568785	*rs41666668*	9	72.184411	0.8223	5.6522	0.017703	0.0548	0.0160015357
ss86304166		9	72.269966	0.8646	0.0075	0.930844	0.0023	0.0005344776
ss61561554	*rs41662464*	9	72.408821	0.4642	0.0559	0.813227	0.0042	0.0020991555
ss61493302	*rs41594139*	9	72.584123	0.4950	0.0012	0.972710	0.0006	0.0003160415
ss86296785		9	72.628414	0.7256	0.1794	0.672046	0.0088	0.0034877222
ss64973180	*rs43066088*	9	72.733806	0.8560	0.7708	0.380278	0.0227	0.0056057507
		9	72.770112	0.2027	1.7040	0.192200	0.0323	0.0104337567
ss61561584	*rs41662492*	9	72.883245	0.1275	3.9980	0.045944	0.0541	0.0120434859
ss86332952		9	72.981353	0.6562	0.9362	0.333594	0.0182	0.0082063308
ss86318730		9	73.036514	0.1597	7.7785	0.005432	0.0702	0.0188568148
ss86308305		9	73.067859	0.6426	0.1374	0.710974	0.0071	0.0032585026
ss117969721		9	73.15482	0.6590	9.8499	0.001770	0.0596	0.0268059089
ss61561594	*rs41662502*	9	73.208406	0.0910	6.6949	0.009871	0.0853	0.0141068004
ss61561604	*rs41662512*	9	73.425019	0.6182	1.8734	0.171528	0.0261	0.0123220167
ss61561612	*rs41662520*	9	73.651821	0.4312	4.1918	0.040996	0.0376	0.0184669628
ss61561608	*rs41662516*	9	73.678946	0.1483	11.3798	0.000784	0.0851	0.0215031850
ss61561607	*rs41662515*	9	73.700047	0.1827	12.8235	0.000366	0.0825	0.0246268556
ss117969743		9	73.723383	0.1597	6.7670	0.009483	0.0658	0.0176622280
ss86301426		9	73.817698	0.7722	8.4115	0.003846	0.0631	0.0221846304
ss117969542		9	73.863695	0.2249	9.1216	0.002619	0.0659	0.0229905225
ss61493311	*rs41594147*	9	73.894894	0.4105	4.0464	0.044650	0.0365	0.0176822391
ss61561614	*rs41662522*	9	73.959682	0.9090	5.0193	0.025381	0.0720	0.0119148288
ss86335619		9	74.372203	0.6956	0.1893	0.663658	0.0086	0.0036255527
ss86309059		9	74.406044	0.7235	0.0532	0.817686	0.0046	0.0018552027
ss86340154		9	74.446035	0.6017	0.1524	0.696415	0.0076	0.0036593790
ss86306735		9	74.559036	0.1361	2.2677	0.132548	0.0404	0.0094982890
ss61493304	*rs41594141*	9	74.586395	0.6390	0.4294	0.512498	0.0130	0.0060194181
ss61493306	*rs41594143*	9	74.635719	0.8395	4.3513	0.037344	0.0537	0.0144764163
ss86334603		9	74.693661	0.3087	2.2226	0.136459	0.0290	0.0123625994
ss61511058	*rs41609984*	9	74.717739	0.4742	0.2154	0.642708	0.0085	0.0042516933
ss86299118		9	74.795365	0.3324	5.2355	0.022429	0.0438	0.0194393068
ss86319485		9	74.937315	0.2450	8.5864	0.003498	0.0626	0.0231497622
ss61561625	*rs41663433*	9	74.968033	0.5415	14.1895	0.000179	0.0703	0.0348930029
ss61561671	*rs41663479*	9	75.092787	0.5000	1.7063	0.191892	0.0235	0.0117508725
ss86320182		9	75.192004	0.4169	0.0666	0.796384	0.0048	0.0023304670
		9	75.3578	0.7880	1.4448	0.229767	0.0271	0.0090543973
ss61561657	*rs41663465*	9	75.394275	0.7708	1.6278	0.202432	0.0280	0.0098859740
ss86317940		9	75.442811	0.8360	0.0581	0.809523	0.0059	0.0016168348
ss61561655	*rs41663463*	9	75.484137	0.3539	2.1131	0.146491	0.0285	0.0130527688
ss86323768		9	75.512134	0.3052	1.3501	0.245658	0.0240	0.0101839837
ss61561652	*rs41663460*	9	75.600297	0.6519	6.2450	0.012684	0.0493	0.0223644221
ss86314375		9	75.673612	0.8861	5.4345	0.020028	0.0684	0.0138042957

ss86340094		9	75.731647	0.4434	7.3637	0.006820		0.0501	0.0247190831
ss61561649	*rs41663457*	9	75.802146	0.6318	4.4456	0.035348		0.0404	0.0187798450
ss61561643	*rs41663451*	9	75.843362	0.5272	0.0005	0.982721		0.0004	0.0002027035
ss86292503		9	75.920644	0.5000	0.0000	0.999451		0.0000	0.0000062801
ss86338088		9	75.940572	0.2264	1.3611	0.243748		0.0254	0.0088894264
ss86336857		9	75.967127	0.8001	7.6687	0.005768		0.0644	0.0206016971
ss86288987		9	76.038687	0.1132	0.1861	0.666328		0.0125	0.0025010045
	rs29010366	9	76.064306	0.4857	0.0758	0.783154		0.0051	0.0025601854
ss61476748	*rs41578299*	9	76.293489	0.8625	2.9017	0.088934		0.0455	0.0107905403
ss38329056	*rs29026671*	9	76.320697	0.1397	2.6205	0.105941		0.0431	0.0103525024
ss117969642		9	76.370249	0.7436	19.0065	0.000015		0.0915	0.0349073176
ss61493342	*rs41594178*	9	76.407545	0.8352	4.4986	0.034276		0.0539	0.0148434473
ss61493339	*rs41594175*	9	76.754517	0.2013	0.1567	0.692346		0.0092	0.0029440496
ss61504806	*rs41599681*	9	76.867142	0.5752	5.8220	0.016085		0.0472	0.0230742260
ss86335734		9	76.959628	0.4599	6.3084	0.012242		0.0497	0.0246806890
	rs29017484	9	77.028664	0.5781	0.4272	0.513598		0.0119	0.0058168977
ss86317939		9	77.053824	0.7887	0.8959	0.344213		0.0210	0.0070057418
ss61565083	*rs41658343*	9	77.1005	0.3818	11.3517	0.000795		0.0640	0.0302062965
ss61512584	*rs41613788*	9	77.191321	0.8037	16.0336	0.000069		0.0940	0.0296597561
ss117969726		9	77.225084	0.7070	0.7470	0.387735		0.0175	0.0072593202
ss86294329		9	77.364314	0.2450	3.6608	0.056117		0.0428	0.0158206820
	rs29012162	9	77.393362	0.2378	7.2166	0.007395		0.0605	0.0219180384
ss61524026	*rs41624973*	9	77.439074	0.5279	0.4618	0.497030		0.0127	0.0063301546
ss86312902		9	77.598567	0.3080	9.4602	0.002182		0.0628	0.0267733745
ss61512575	*rs41613779*	9	77.629139	0.2564	2.2823	0.131315		0.0317	0.0120968079
ss86304687		9	77.695767	0.8739	13.7973	0.000220		0.1036	0.0228382692
ss61561706	*rs41663514*	9	77.785143	0.7915	0.5606	0.454288		0.0170	0.0056213638
ss61561699	*rs41663507*	9	77.808521	0.5179	1.9421	0.163884		0.0256	0.0127668993
ss61561688	*rs41663496*	9	77.830519	0.2013	0.3272	0.567472		0.0131	0.0042200587
ss86340316		9	77.870594	0.8388	0.1636	0.685978		0.0103	0.0027725238
ss86313487		9	77.903086	0.3883	3.9022	0.048618		0.0380	0.0180689722
ss61566627	*rs41666553*	9	77.933282	0.1927	2.2225	0.136462		0.0340	0.0105875049
ss86295623		9	78.060827	0.2693	0.8344	0.361330		0.0197	0.0077358713
ss86296494		9	78.148319	0.3768	0.0003	0.986140		0.0003	0.0001504176
ss86335944		9	78.185375	0.3804	0.0856	0.769983		0.0053	0.0025081114
ss86317636		9	78.207894	0.2328	0.2226	0.637238		0.0102	0.0036386511
ss61493344	*rs41594180*	9	78.266905	0.6368	24.8099	0.000001		0.0937	0.0433569921
ss61561707	*rs41663515*	9	78.313892	0.8245	19.1074	0.000014		0.1049	0.0303523122
ss86306346		9	78.350282	0.6490	0.3779	0.538916		0.0118	0.0053608140
ss61561708	*rs41663516*	9	78.37533	0.8309	1.4635	0.226787		0.0304	0.0085443317
ss61561711	*rs41663519*	9	78.442559	0.6340	0.9113	0.340098		0.0185	0.0086089495
ss86294340		9	78.514742	0.1490	2.4224	0.120066		0.0388	0.0098515084
ss61476613	*rs41577768*	9	78.564041	0.8682	1.7568	0.185456		0.0357	0.0081697778
ss61493345	*rs41594181*	9	78.625418	0.4069	0.8976	0.343754		0.0181	0.0087336424
ss61561715	*rs41663523*	9	78.722251	0.1082	3.8898	0.048977		0.0593	0.0114419722
ss117969661		9	78.854532	0.5802	0.0556	0.813610		0.0044	0.0021314030
ss86336167		9	79.0133	0.4592	4.0023	0.045825		0.0358	0.0178030930

ss61507584	*rs41607876*	9	79.133468	0.1927	0.4407	0.507022	0.0153	0.0047636686
ss61525052	*rs41625279*	9	79.202385	0.8424	17.5876	0.000031	0.1050	0.0278913538
ss61528523	*rs41626568*	9	79.375994	0.5938	4.7888	0.028976	0.0405	0.0195265788
	rs29021143	9	79.476449	0.7794	1.4845	0.223485	0.0268	0.0092165336
ss61561745	*rs41664353*	9	79.666231	0.2808	5.7894	0.016382	0.0502	0.0202801929
ss61523782	*rs41623541*	9	79.690071	0.6748	0.5200	0.471105	0.0144	0.0063114139
ss61561747	*rs41664355*	9	79.724697	0.6769	0.9546	0.328891	0.0198	0.0086680155
ss117969688		9	79.814364	0.8603	0.9782	0.322987	0.0255	0.0061340529
ss86339900		9	79.83513	0.4434	0.9304	0.335106	0.0180	0.0089023546
ss86338906		9	79.901517	0.8510	4.9174	0.026910	0.0599	0.0152027957
ss61567996	*rs41666494*	9	80.146263	0.2128	2.5837	0.108421	0.0372	0.0124708319
ss61567995	*rs41666493*	9	80.192954	0.5638	1.0123	0.314690	0.0187	0.0092052324
ss61516048	*rs41610974*	9	80.31402	0.0609	6.3514	0.011951	0.0985	0.0112637835
ss61472237	*rs41568875*	9	80.362954	0.0645	1.6365	0.201228	0.0489	0.0059045124
ss61479049	*rs41579916*	9	80.520855	0.4835	2.0742	0.150254	0.0274	0.0136954201
ss86313046		9	80.74046	0.1132	0.2531	0.615067	0.0149	0.0029980709
ss61505154	*rs41602913*	9	81.017236	0.1698	2.6908	0.101381	0.0406	0.0114531556
ss61561754	*rs41664361*	9	81.171189	0.2815	0.3391	0.560561	0.0125	0.0050765327
	rs29014693	9	81.304249	0.7120	2.2987	0.129938	0.0310	0.0127212702
ss64823921	*rs42916147*	9	81.376122	0.4699	5.3197	0.021379	0.0432	0.0215434465
ss86340857		9	82.007562	0.2085	0.6210	0.430928	0.0184	0.0060567222
ss117966023		9	82.175488	0.8782	3.6029	0.058093	0.0550	0.0117538361
ss61561743	*rs41664351*	9	82.321187	0.2564	4.7000	0.030500	0.0475	0.0181327364
ss61570670	*rs41670223*	9	82.384856	0.8617	0.7530	0.385825	0.0236	0.0056308352
ss61561742	*rs41664350*	9	82.419659	0.7213	0.6792	0.410150	0.0177	0.0071035099
ss61561733	*rs41664341*	9	82.609869	0.3338	0.6037	0.437421	0.0155	0.0069154596
ss86286366		9	82.638893	0.1175	4.0875	0.043583	0.0579	0.0120143322
	rs29026781	9	82.712069	0.4764	0.4051	0.524684	0.0118	0.0058769443
ss86336072		9	82.757569	0.5652	0.0038	0.951041	0.0012	0.0005787780
ss86306604		9	82.84339	0.5100	0.4595	0.498103	0.0130	0.0065187084
	rs29026831	9	82.909986	0.9226	0.0096	0.921952	0.0034	0.0004923785
	rs29026461	9	82.938615	0.2722	2.3952	0.122162	0.0325	0.0128915012
ss61523981		9	82.959866	0.1784	1.5554	0.212754	0.0305	0.0089448268
ss61515106	*rs41611967*	9	82.991887	0.2342	14.4284	0.000158	0.0844	0.0302900920
ss86284926		9	83.021671	0.2787	7.2503	0.007260	0.0555	0.0223280709
ss117969657		9	83.163086	0.8668	0.3452	0.557033	0.0164	0.0037778585
ss63763799	*rs43732766*	9	83.201258	0.9420	1.3479	0.246038	0.0471	0.0051450409
ss64727949	*rs42826435*	9	83.280597	0.3295	0.0695	0.792147	0.0051	0.0022723565
ss61473986		9	83.302784	0.2536	2.9201	0.087928	0.0365	0.0138120389
ss61498012	*rs43706625*	9	83.383675	0.4506	9.6225	0.002000	0.0577	0.0285555788
ss86301103		9	83.426544	0.0530	0.1584	0.690744	0.0157	0.0015737125
ss86305428		9	83.457426	0.5645	6.0832	0.013887	0.0453	0.0222626078
ss61498019	*rs43707432*	9	83.499267	0.4148	3.6238	0.057372	0.0353	0.0171192537
ss64663053	*rs42762731*	9	83.536096	0.9319	0.2901	0.590324	0.0189	0.0023961612
	rs29024426	9	83.567937	0.9327	0.0002	0.988782	0.0005	0.0000615615
ss64663036	*rs42762514*	9	83.591134	0.0372	2.4620	0.117083	0.0774	0.0055488232
ss65524684	*rs43610712*	9	83.666857	0.1905	2.7268	0.099126	0.0388	0.0119659393

ss86320282		9	83.724286	0.2421	3.2906	0.070107	0.0387	0.0142074397
ss65524503	*rs43609731*	9	83.745887	0.2880	1.4554	0.228068	0.0246	0.0100824914
ss61511065	*rs41609990*	9	83.767162	0.5380	0.1884	0.664406	0.0080	0.0039603887
ss86339695		9	83.800277	0.9477	0.4844	0.486656	0.0291	0.0028865637
	rs29025414	9	83.826695	0.6311	0.0145	0.904064	0.0023	0.0010525416
ss86284306		9	83.875984	0.6798	0.0177	0.894205	0.0026	0.0011125737
ss65524289	*rs43606817*	9	83.921379	0.4470	0.3311	0.565180	0.0108	0.0053447403
ss86339023		9	83.970853	0.6447	2.1860	0.139727	0.0285	0.0130394309
ss86331641		9	84.032883	0.6769	6.9317	0.008656	0.0503	0.0219848312
ss65524205	*rs43606733*	9	84.11227	0.3983	0.0001	0.990732	0.0002	0.0001052681
ss86327532		9	84.176606	0.6426	0.8875	0.346470	0.0184	0.0084452516
ss86333590		9	84.201346	0.5587	0.0017	0.967322	0.0008	0.0003848127
ss86275197		9	84.317888	0.6211	0.5550	0.456551	0.0144	0.0067598031
ss65526210	*rs43605234*	9	84.338662	0.3496	2.3633	0.124672	0.0293	0.0133104992
ss65524161	*rs43605889*	9	84.360555	0.3560	2.6756	0.102349	0.0309	0.0141590451
ss65524117	*rs43605845*	9	84.387084	0.6268	1.6554	0.198651	0.0240	0.0112259558
ss61493368	*rs41594200*	9	84.413829	0.6963	2.9891	0.084272	0.0354	0.0149781156
ss86322267		9	84.43612	0.8259	0.0470	0.828449	0.0054	0.0015573933
ss61561763	*rs41664370*	9	84.489996	0.3295	0.7527	0.385908	0.0170	0.0075299884
ss61511063	*rs41609988*	9	84.516161	0.0702	2.1198	0.145858	0.0521	0.0067951210
ss61561759	*rs41664366*	9	84.551449	0.7772	3.1066	0.078415	0.0401	0.0138863866
ss65523623	*rs43609252*	9	84.580933	0.1683	1.1222	0.289821	0.0262	0.0073390212
ss65523596	*rs43608225*	9	84.609287	0.8202	5.4943	0.019359	0.0568	0.0167400769
ss61493365	*rs41594197*	9	84.634816	0.1855	0.3366	0.562010	0.0139	0.0041977638
ss65523501	*rs43607230*	9	84.658186	0.1819	0.2231	0.636818	0.0114	0.0033911704
ss61493363	*rs41594195*	9	84.67979	0.8682	1.0185	0.313220	0.0275	0.0062937729
ss86318036		9	84.720254	0.0688	2.2555	0.133595	0.0541	0.0069348393
ss61561756	*rs41664363*	9	84.783845	0.6769	0.3922	0.531356	0.0127	0.0055341868
ss64309916	*rs42576888*	9	84.832223	0.1805	0.9750	0.323773	0.0243	0.0071935685
ss64309774	*rs42576017*	9	84.852484	0.3489	0.2909	0.589798	0.0103	0.0046958890
ss64309964	*rs42576912*	9	84.87326	0.1812	0.9839	0.321585	0.0244	0.0072434404
ss65523395	*rs43606125*	9	84.91302	0.8574	0.1574	0.691720	0.0109	0.0026702910
ss86286579		9	84.949725	0.5021	5.4393	0.019973	0.0427	0.0213697605
ss61561755	*rs41664362*	9	84.989472	0.4577	2.5436	0.111194	0.0297	0.0147359215
ss64309702	*rs42575981*	9	85.026625	0.6590	3.8478	0.050211	0.0379	0.0170245207
	rs29012632	9	85.062846	0.5136	5.8391	0.015930	0.0445	0.0222522089
ss64425940	*rs42629820*	9	85.101503	0.4499	0.1433	0.705141	0.0072	0.0035598422
ss64425710	*rs42629005*	9	85.200612	0.4893	0.7821	0.376809	0.0166	0.0082936446
ss86291035		9	85.246326	0.4814	0.1512	0.697507	0.0074	0.0037114730
ss86284092		9	85.274882	0.5143	1.0021	0.317143	0.0188	0.0093961322
ss86318890		9	85.31175	0.8589	2.3719	0.123988	0.0422	0.0102379018
ss86313716		9	85.34677	0.2837	8.5307	0.003605	0.0598	0.0243167765
ss86339294		9	85.379574	0.3589	0.0437	0.834397	0.0042	0.0019122480
ss86338646		9	85.402443	0.6340	3.2789	0.070609	0.0344	0.0159590174
ss86307672		9	85.450752	0.8181	0.5026	0.478611	0.0172	0.0051340403
ss61475066	*rs41571459*	9	85.474843	0.3009	5.2537	0.022198	0.0464	0.0195057821
ss86337873		9	85.539969	0.5179	5.2036	0.022841	0.0430	0.0214714862

ss86340082		9	85.574435	0.6956	4.2658	0.039257	0.0420	0.0177944419
ss86318458		9	85.64499	0.1669	1.7786	0.182757	0.0332	0.0092313520
ss86303157		9	85.896321	0.3718	0.8200	0.365495	0.0167	0.0077897349
ss65107374	*rs43193272*	9	85.934553	0.7772	2.1536	0.142685	0.0334	0.0115650865
	rs29015116	9	86.008056	0.8711	2.5066	0.113823	0.0430	0.0096675136
ss86297089		9	86.348462	0.1089	0.9905	0.319960	0.0299	0.0058029346
ss86339179		9	86.457251	0.3489	6.7436	0.009607	0.0493	0.0224197274
ss86328568		9	86.477819	0.6397	5.6405	0.017820	0.0450	0.0207250684
ss61499540	*rs43710727*	9	86.500532	0.4620	18.5583	0.000019	0.0774	0.0384556042
ss61518321	*rs41613185*	9	86.614998	0.2106	5.4043	0.020374	0.0515	0.0171272901
	rs29010238	9	86.636674	0.3646	1.5637	0.211541	0.0234	0.0108371606
ss61498555	*rs43712655*	9	86.683884	0.3016	0.7694	0.380713	0.0176	0.0073966648
ss86321205		9	86.906523	0.3883	0.5812	0.446118	0.0141	0.0067075938
ss117967143		9	87.074542	0.5996	2.1209	0.145755	0.0280	0.0134309370
ss61561838	*rs41665445*	9	87.253444	0.2199	0.7855	0.375763	0.0199	0.0068242667
ss61561829	*rs41665436*	9	87.314747	0.7521	0.0079	0.929000	0.0019	0.0007081384
ss61493440	*rs41594971*	9	87.461171	0.5050	2.9319	0.087289	0.0317	0.0158717456
ss86325037		9	87.493754	0.1433	5.7428	0.016819	0.0627	0.0153816124
ss61561822		9	87.514382	0.6032	0.9927	0.319419	0.0187	0.0089519703
ss61561821	*rs41664428*	9	87.604199	0.1913	16.3057	0.000060	0.0939	0.0290449697
ss86302160		9	87.852894	0.6168	3.2097	0.073636	0.0343	0.0162373693
ss117969528		9	88.157049	0.4871	0.6723	0.412533	0.0152	0.0075856336
ss61493449	*rs41594980*	9	88.178141	0.0974	0.0008	0.977727	0.0009	0.0001556584
ss61493447	*rs41594978*	9	88.27602	0.6540	2.0344	0.154222	0.0271	0.0122811473
ss65526061	*rs43603886*	9	88.297771	0.5946	0.0983	0.753953	0.0059	0.0028452736
ss65526170	*rs43604594*	9	88.317811	0.1963	1.9592	0.162045	0.0324	0.0102278654
	rs29009799	9	88.355172	0.5766	1.0164	0.313716	0.0189	0.0092060388
ss61561862	*rs41665468*	9	88.416412	0.5559	0.6061	0.436510	0.0145	0.0071803972
ss65525839	*rs43602867*	9	88.454941	0.2077	3.7514	0.053169	0.0438	0.0144037023
ss65525790	*rs43612517*	9	88.529232	0.5681	6.4053	0.011597	0.0467	0.0229239420
ss117969595		9	88.560515	0.7385	0.1393	0.709134	0.0078	0.0029957624
ss86336830		9	88.580635	0.7708	2.7440	0.098069	0.0365	0.0128930357
ss65525629	*rs43611158*	9	88.642748	0.2479	1.4514	0.228712	0.0260	0.0097043508
ss65525567	*rs43610096*	9	88.671964	0.3352	13.5288	0.000253	0.0704	0.0313621969
ss61561859	*rs41665465*	9	88.70121	0.6490	9.5894	0.002036	0.0626	0.0285097788
ss86327015		9	88.742826	0.2292	2.4410	0.118657	0.0360	0.0127221824
ss117969769		9	88.782099	0.5208	1.1479	0.284363	0.0201	0.0100248118
ss86312303		9	88.810719	0.8861	3.1445	0.076619	0.0518	0.0104583736
ss86314798		9	88.849121	0.3689	9.1837	0.002532	0.0582	0.0271122568
ss65525165	*rs43605995*	9	88.899515	0.6476	0.0004	0.984195	0.0004	0.0001788756
ss86337591		9	88.937664	0.2020	0.7091	0.400041	0.0195	0.0062780213
ss65525332	*rs43608061*	9	89.02665	0.6576	5.3170	0.021412	0.0441	0.0198543575
	rs29022968	9	89.057232	0.6927	9.4505	0.002193	0.0596	0.0253873724
ss86296358		9	89.138011	0.4040	4.5675	0.032932	0.0409	0.0196924840
ss61561851	*rs41665457*	9	89.179018	0.6676	2.3620	0.124775	0.0303	0.0134548854
ss61561854	*rs41665460*	9	89.204613	0.6390	0.0902	0.763987	0.0058	0.0026555531
ss86284269		9	89.227002	0.5609	0.3520	0.553201	0.0113	0.0055590098

ss65525551	*rs43610080*	9	89.269245	0.5201	2.0887	0.148843	0.0265	0.0132144167
ss65527015	*rs43605537*	9	89.296619	0.3983	1.6935	0.193576	0.0248	0.0118710812
	rs29012585	9	89.329674	0.8668	9.5761	0.002050	0.0811	0.0187310312
ss86338421		9	89.405023	0.7242	9.1024	0.002646	0.0630	0.0251594396
ss46525997	*rs41255145*	9	89.501312	0.5709	8.2534	0.004191	0.0557	0.0273100837
ss86289565		9	89.523252	0.2249	2.1678	0.141381	0.0343	0.0119442442
ss61561882	*rs41665486*	9	89.558592	0.8231	3.6260	0.057296	0.0464	0.0135006525
ss61512694	*rs41614496*	9	89.595205	0.4277	0.4976	0.480779	0.0132	0.0064694614
ss86314662		9	89.633799	0.5408	4.8258	0.028366	0.0403	0.0200126051
ss61546441	*rs41642776*	9	89.75216	0.2235	20.6645	0.000006	0.1000	0.0347069829
ss86326899		9	89.871362	0.2973	0.0147	0.903417	0.0024	0.0010027313
	rs29018921	9	89.916177	0.4448	3.9080	0.048451	0.0366	0.0180842569
ss65454613	*rs43537771*	9	90.044447	0.8152	0.5009	0.479328	0.0166	0.0050129968
ss65454580	*rs43537739*	9	90.096917	0.1862	0.9687	0.325338	0.0230	0.0069693996
ss64710952	*rs42802551*	9	90.138029	0.4585	2.7029	0.100619	0.0299	0.0148597104
ss61475473	*rs41575047*	9	90.171281	0.2958	2.7952	0.094998	0.0333	0.0138686868
ss86338836		9	90.422987	0.3632	0.5418	0.461941	0.0170	0.0078775936
ss65058609	*rs43153913*	9	90.498763	0.5946	10.1448	0.001512	0.0602	0.0290334053
ss86305187		9	90.579031	0.5595	6.4149	0.011535	0.0465	0.0229391287
ss64666477	*rs42760519*	9	90.600676	0.1554	0.1533	0.695478	0.0101	0.0026432777
ss65093721	*rs43182474*	9	90.687358	0.3381	2.8127	0.093971	0.0334	0.0149398837
ss64381249	*rs42611486*	9	90.712032	0.3367	2.3635	0.124657	0.0308	0.0137476359
ss86327920		9	90.743221	0.7042	0.8005	0.371266	0.0187	0.0077873318
ss64880801	*rs42972397*	9	90.796432	0.6089	0.1405	0.707871	0.0071	0.0033679207
ss61546440	*rs41642775*	9	90.858069	0.8825	5.9800	0.014716	0.0731	0.0151583749
ss62878242	*rs42027938*	9	90.879255	0.1189	0.3338	0.563642	0.0167	0.0034943093
ss61509462	*rs41604212*	9	90.92502	0.6103	0.0618	0.803670	0.0047	0.0022327327
	rs29012728	9	91.011839	0.4076	0.1075	0.743150	0.0062	0.0029746797
ss86340390		9	91.102602	0.2944	9.7413	0.001876	0.0618	0.0256874174
ss62877693	*rs42025957*	9	91.12657	0.7292	10.7461	0.001097	0.0673	0.0265856371
ss61546436	*rs41642771*	9	91.149298	0.6275	3.9843	0.046315	0.0377	0.0176336953
ss86326725		9	91.178779	0.0867	4.6346	0.031676	0.0710	0.0112373624
ss61493479	*rs41595007*	9	91.239057	0.1891	7.7120	0.005633	0.0639	0.0195878546
ss86313036		9	91.267686	0.0802	0.8649	0.352681	0.0313	0.0046221378
ss86274650		9	91.361559	0.8431	2.4847	0.115415	0.0399	0.0105489350
ss61561898	*rs41665500*	9	91.459687	0.0903	0.0414	0.838733	0.0068	0.0011095009
ss86323522		9	91.49251	0.7228	0.3657	0.545562	0.0123	0.0049163697
ss46526986	*rs41255531*	9	91.539465	0.4341	0.0333	0.855162	0.0034	0.0016658376
ss65528794	*rs43615598*	9	91.589794	0.6984	1.5561	0.212656	0.0248	0.0104665639
ss86301732		9	91.655594	0.1691	0.4665	0.494830	0.0170	0.0047724650
ss86327756		9	91.798548	0.1698	0.7217	0.395880	0.0212	0.0059758094
ss65528483	*rs43612994*	9	91.853777	0.3023	2.0751	0.150172	0.0305	0.0128763868
ss65528165	*rs43609676*	9	92.020865	0.5021	13.6450	0.000238	0.0678	0.0338986865
ss65528056	*rs43608567*	9	92.068337	0.8703	0.0609	0.805102	0.0068	0.0015370476
ss61561894	*rs41665496*	9	92.122144	0.3481	2.0994	0.147810	0.0282	0.0127851663
ss65527989	*rs43607404*	9	92.154967	0.3438	2.3185	0.128300	0.0295	0.0133092715
ss61561891	*rs41665493*	9	92.215829	0.7042	2.3739	0.123835	0.0307	0.0128056064

ss65527893	*rs43606608*	9	92.25084	0.3875	6.7179	0.009745	0.0505	0.0239499522
ss65527879	*rs43606594*	9	92.305197	0.6318	0.3358	0.562443	0.0116	0.0054156794
ss61561885	*rs41665489*	9	92.328442	0.1841	0.0095	0.922450	0.0024	0.0007097998
ss86276432		9	92.490073	0.1476	0.9562	0.328481	0.0254	0.0064006760
ss61493482	*rs41595010*	9	92.579347	0.5989	5.4672	0.019659	0.0450	0.0216352510
ss65529056	*rs43609859*	9	92.622686	0.4026	1.3400	0.247439	0.0215	0.0103561880
ss86313774		9	92.732157	0.3768	2.0624	0.151423	0.0270	0.0127025795
ss65527489	*rs43608403*	9	92.775509	0.3087	0.6941	0.405068	0.0168	0.0071591147
ss65527409	*rs43607823*	9	92.833882	0.7815	1.9154	0.166810	0.0320	0.0109157154
	rs29025303	9	92.945614	0.6340	0.1008	0.750990	0.0060	0.0028018108
ss117969706		9	92.972445	0.1433	2.9608	0.085750	0.0453	0.0111149426
ss86341760		9	93.110745	0.5107	0.2849	0.593677	0.0100	0.0049944667
ss64456236	*rs42651479*	9	93.165878	0.1211	0.1494	0.699244	0.0109	0.0023279956
ss86313380		9	93.221053	0.8804	11.9395	0.000583	0.0973	0.0204994729
ss64856138	*rs42953594*	9	93.2529	0.5050	0.1139	0.735869	0.0063	0.0031561125
ss117969734		9	93.44331	0.9262	3.1417	0.076750	0.0641	0.0087616032
ss65526439	*rs43606363*	9	93.56224	0.7994	0.8756	0.349721	0.0223	0.0071425340
ss61469418	*rs41569525*	9	93.608905	0.7672	3.9395	0.047556	0.0430	0.0153780039
ss65526551	*rs43607069*	9	93.64485	0.7615	3.2280	0.072823	0.0393	0.0142601224
ss65526588	*rs43607106*	9	93.679736	0.1554	0.5462	0.460110	0.0188	0.0049409426
ss65526717	*rs43608435*	9	93.762277	0.4599	0.3451	0.557076	0.0108	0.0053834833
ss65526728	*rs43608446*	9	93.782334	0.4656	0.1599	0.689399	0.0074	0.0037029504
ss65526807	*rs43608525*	9	93.809495	0.1862	0.4624	0.496717	0.0155	0.0046924332
ss65067093	*rs43159906*	9	93.844874	0.1898	0.0198	0.888018	0.0033	0.0010134248
ss86337522		9	93.877954	0.6168	1.9678	0.161132	0.0270	0.0127604858
ss64911929	*rs43003144*	9	93.90348	0.7658	4.5378	0.033505	0.0472	0.0169387508
	rs29022272	9	93.98368	0.2507	3.3045	0.069520	0.0395	0.0148281179
ss61569599	*rs41668674*	9	94.137281	0.9534	0.5331	0.465572	0.0326	0.0028958482
ss61569601	*rs41668676*	9	94.161445	0.3961	1.7944	0.180831	0.0255	0.0121883560
ss61497222	*rs41595251*	9	94.188186	0.7128	0.3826	0.536419	0.0122	0.0049799157
ss38329350	*rs29026964*	9	94.222827	0.3403	0.0337	0.854329	0.0035	0.0015822671
ss61520407	*rs41614532*	9	94.260793	0.5136	0.0108	0.917402	0.0019	0.0009704895
	rs29013969	9	94.364803	0.4957	7.4166	0.006624	0.0511	0.0255441767
ss86303894		9	94.421974	0.2808	2.0886	0.148853	0.0304	0.0122945261
ss86287055		9	94.497195	0.7020	1.4990	0.221246	0.0247	0.0103365147
ss65529648	*rs43615650*	9	94.579943	0.2077	11.0843	0.000917	0.0754	0.0248086898
ss86334193		9	94.602978	0.2385	7.4458	0.006519	0.0597	0.0216768677
ss61493495	*rs41595023*	9	94.653024	0.0774	0.0940	0.759249	0.0106	0.0015099934
ss61561938	*rs41656346*	9	94.680196	0.0809	1.2756	0.259112	0.0372	0.0055327841
ss61561935	*rs41656343*	9	94.703203	0.2944	0.2377	0.626043	0.0100	0.0041494313
	rs29023391	9	94.734868	0.1139	0.2337	0.628937	0.0139	0.0028084122
ss86305349		9	94.766825	0.5165	6.4899	0.011063	0.0472	0.0235657841
ss65529417	*rs43613520*	9	94.795395	0.2650	0.3414	0.559192	0.0122	0.0047529189
ss86318908		9	94.825398	0.0824	1.3198	0.251019	0.0395	0.0059667601
ss86328279		9	94.874077	0.4405	1.1241	0.289415	0.0196	0.0096768151
ss61561923	*rs41656331*	9	94.962747	0.8087	0.2320	0.630176	0.0113	0.0034846128
ss61561918	*rs41665520*	9	95.049199	0.7751	0.0204	0.886373	0.0032	0.0011206851

ss65529336	*rs43613439*	9	95.082917	0.7278	1.8278	0.176828	0.0284	0.0112371495
ss86337439		9	95.19461	0.4441	4.9970	0.025708	0.0423	0.0208705148
ss86311882		9	95.224822	0.1024	0.5434	0.461267	0.0222	0.0040906951
ss65529129	*rs43610932*	9	95.255366	0.8295	0.9475	0.330693	0.0230	0.0065157653
ss86338753		9	95.361555	0.1562	0.3209	0.571245	0.0141	0.0037231633
ss86280379		9	95.459953	0.2242	1.1220	0.289854	0.0231	0.0080479720
ss86336305		9	95.499624	0.2543	0.3114	0.577022	0.0119	0.0045145882
ss86288118		9	95.56289	0.5802	2.4671	0.116704	0.0290	0.0141195807
ss86323052		9	95.630756	0.2586	0.0731	0.787017	0.0058	0.0022283027
ss61561913	*rs41665515*	9	95.741863	0.6046	0.0100	0.920362	0.0019	0.0008990454
ss86283387		9	95.783554	0.1784	2.9559	0.086007	0.0405	0.0118673526
ss86340286		9	95.832142	0.7378	1.0602	0.303537	0.0213	0.0082457970
ss86339448		9	95.863973	0.3711	0.0375	0.846547	0.0037	0.0017154291
ss61561939	*rs41656347*	9	95.993546	0.4255	15.4078	0.000095	0.0739	0.0361181077
	rs29026423	9	96.039657	0.6504	7.8444	0.005239	0.0547	0.0248910896
ss61493518	*rs41588151*	9	96.22919	0.2872	2.8393	0.092434	0.0338	0.0138228884
ss86318845		9	96.255786	0.5931	0.5328	0.465693	0.0137	0.0066073073
ss86327158		9	96.34319	0.3746	0.4441	0.505352	0.0127	0.0059360533
ss61493510	*rs41588143*	9	96.402492	0.4663	10.2555	0.001425	0.0584	0.0290596506
ss86337278		9	96.448044	0.3202	2.0342	0.154242	0.0282	0.0122970944
ss117969761		9	96.468569	0.6304	1.4108	0.235335	0.0223	0.0104111069
ss86337852		9	96.499853	0.4083	11.3093	0.000813	0.0615	0.0297321826
ss117964078		9	96.580539	0.4878	0.1733	0.677298	0.0078	0.0039056411
ss86336154		9	96.620314	0.7242	0.0824	0.774163	0.0060	0.0024020545
ss61561967		9	96.732328	0.8245	3.1815	0.074914	0.0428	0.0123862067
ss61511089	*rs41610014*	9	96.755004	0.8252	0.2634	0.607982	0.0123	0.0035444858
ss61504829	*rs41599704*	9	96.785306	0.6633	0.7740	0.379282	0.0170	0.0075957402
ss86311385		9	96.836455	0.1476	5.3987	0.020439	0.0609	0.0153113928
ss86339426		9	96.927246	0.8625	3.3685	0.066881	0.0499	0.0118460513
ss86324286		9	96.968435	0.6576	4.7442	0.029731	0.0426	0.0191873156
ss86297492		9	97.058732	0.4456	0.0510	0.821372	0.0042	0.0020504454
ss86317704		9	97.150276	0.4277	0.8749	0.349925	0.0171	0.0083790430
	rs29018437	9	97.307453	0.6347	0.0642	0.800030	0.0050	0.0023214553
ss61529087	*rs41631997*	9	97.33329	0.6132	0.0242	0.876485	0.0030	0.0014022991
ss61466815	*rs41573356*	9	97.35756	0.6347	0.0642	0.800030	0.0050	0.0023214553
ss61504837	*rs41599712*	9	97.381354	0.4685	1.4754	0.224902	0.0228	0.0113564518
ss86339638		9	97.443797	0.6354	0.0835	0.772639	0.0057	0.0026518640
ss117969517		9	97.46526	0.5086	1.0824	0.298523	0.0192	0.0096039026
ss86289287		9	97.485505	0.5072	1.0909	0.296644	0.0193	0.0096694871
ss86340216		9	97.510218	0.4928	1.2960	0.255341	0.0211	0.0105354493
ss86338790		9	97.579509	0.4477	4.4526	0.035205	0.0396	0.0195609838
ss86290160		9	97.658361	0.6168	0.0178	0.893950	0.0025	0.0011935541
ss86333973		9	97.754255	0.8854	5.3281	0.021277	0.0666	0.0135136645
ss86286009		9	97.778825	0.5308	1.6312	0.201966	0.0235	0.0117294818
ss86287869		9	97.799323	0.4019	15.8908	0.000074	0.0731	0.0351609982
ss86332594		9	97.919714	0.4556	4.9722	0.026077	0.0411	0.0203868424
ss86312915		9	97.951104	0.4635	6.6215	0.010281	0.0471	0.0234120159

ss86316337		9	97.995299	0.6554	0.0546	0.815312	0.0046	0.0020613035
ss86341636		9	98.017393	0.5236	18.9209	0.000016	0.0781	0.0389834134
ss86294729		9	98.048063	0.8231	1.8075	0.179247	0.0326	0.0094810245
ss61493542	*rs41588175*	9	98.078552	0.5265	3.2861	0.070299	0.0335	0.0167156853
ss86293037		9	98.113316	0.3933	0.6712	0.412931	0.0159	0.0075797886
ss46526966	*rs41257412*	9	98.275783	0.5981	0.4883	0.484910	0.0131	0.0062909024
ss61496042	*rs41595599*	9	98.310529	0.6812	0.0019	0.965018	0.0009	0.0003697512
ss86330886		9	98.349338	0.7156	0.1459	0.702629	0.0077	0.0031400677
ss117969700		9	98.419372	0.2178	0.3983	0.528190	0.0136	0.0046453183
ss86334265		9	98.450642	0.9484	10.0796	0.001565	0.1325	0.0129669724
ss86337228		9	98.497972	0.4599	3.9265	0.047924	0.0362	0.0179905958
	rs29024438	9	98.534072	0.6146	3.8047	0.051510	0.0371	0.0175611608
ss61493547	*rs41588180*	9	98.619639	0.5179	0.0011	0.973212	0.0006	0.0003109626
	rs29012081	9	98.673543	0.4821	0.0018	0.966104	0.0008	0.0003935225
ss86327423		9	98.770032	0.4198	0.8977	0.343724	0.0176	0.0085966100
ss61511084		9	98.8326	0.8001	0.5519	0.457813	0.0171	0.0054559605
ss86293864		9	98.878095	0.2163	2.7732	0.096305	0.0373	0.0126547683
ss86289024		9	98.91251	0.1999	0.3809	0.537336	0.0144	0.0046174352
ss86333071		9	99.011503	0.5702	2.7512	0.097632	0.0307	0.0150531706
ss61493500	*rs41588133*	9	99.032772	0.0616	2.7744	0.096233	0.0657	0.0075973388
ss61561966	*rs41656373*	9	99.084698	0.1554	8.7690	0.003168	0.0765	0.0200839548
ss86310842		9	99.321699	0.6927	13.7132	0.000230	0.0722	0.0307488872
ss61469862	*rs41573656*	9	99.364024	0.1203	0.4503	0.502430	0.0192	0.0040555950
ss86295106		9	99.394933	0.2701	0.0035	0.952517	0.0012	0.0004871433
ss86304324		9	99.423029	0.6533	1.6317	0.201888	0.0243	0.0109934600
ss86313030		9	99.472394	0.1884	0.0190	0.890278	0.0033	0.0010092060
ss86326094		9	99.589545	0.3725	2.2289	0.135907	0.0279	0.0130491652
ss86336236		9	99.617342	0.3496	4.6277	0.031804	0.0422	0.0191776395
ss86339636		9	99.746295	0.7213	8.7071	0.003276	0.0596	0.0239414552
ss61566583	*rs41665411*	9	99.803157	0.5351	0.6507	0.420143	0.0146	0.0072639119
ss86339478		9	99.82995	0.3653	1.8312	0.176427	0.0259	0.0120130104
ss86299907		9	99.911919	0.7192	1.4728	0.225319	0.0254	0.0102529034
ss61469443	*rs41570350*	9	99.970673	0.1877	0.0198	0.888033	0.0034	0.0010311090
ss86325738		9	100.008903	0.2013	3.5770	0.059001	0.0431	0.0138676864
ss86340899		9	100.141875	0.2092	5.4853	0.019458	0.0538	0.0177831601
ss86334107		9	100.169524	0.6583	12.4097	0.000455	0.0687	0.0309015337
ss86337026		9	100.1999	0.6640	13.5429	0.000251	0.0718	0.0320566080
ss61562069	*rs41657173*	9	100.234256	0.1318	2.2279	0.135991	0.0416	0.0095294309
ss61562068	*rs41657172*	9	100.284335	0.2199	10.9275	0.000996	0.0752	0.0258083074
ss86309056		9	100.312899	0.3567	9.8180	0.001801	0.0595	0.0272952869
ss86330556		9	100.340695	0.5860	6.3706	0.011824	0.0472	0.0228808809
ss61469444	*rs41570351*	9	100.404231	0.7794	8.8800	0.002984	0.0673	0.0231392485
ss61493570	*rs41588203*	9	100.456903	0.6074	7.8560	0.005206	0.0539	0.0256900858
ss86331703		9	100.504006	0.3489	2.8074	0.094278	0.0326	0.0148016518
ss61562024	*rs41656430*	9	100.524834	0.2185	3.9839	0.046328	0.0455	0.0155264268
ss61493552		9	100.553557	0.1232	0.0511	0.821148	0.0062	0.0013378432
ss61493574	*rs41588207*	9	100.608493	0.0845	0.1806	0.671017	0.0147	0.0022810323

ss86325640		9	100.643615	0.4513	13.8842	0.000210	0.0694	0.0343494085
ss86296888		9	100.704132	0.2987	6.3456	0.011991	0.0523	0.0219077084
ss86340075		9	100.746587	0.4713	1.0546	0.304817	0.0190	0.0094893857
ss61466355	*rs41568110*	9	100.775838	0.3223	4.7334	0.029917	0.0437	0.0190714722
ss61562038	*rs41657144*	9	100.919607	0.7930	2.4284	0.119607	0.0350	0.0114982385
ss117964042		9	100.948909	0.4585	9.7526	0.001865	0.0588	0.0291874178
	rs29018256	9	100.972166	0.1182	0.2859	0.593049	0.0154	0.0032166877
ss86317281		9	100.988504	0.0659	4.2935	0.038626	0.0775	0.0095429479
ss61491086	*rs41593884*	9	101.02831	0.2292	0.0190	0.890512	0.0031	0.0010871755
ss86326479		9	101.057554	0.1862	1.7409	0.187464	0.0328	0.0099469123
ss61556725	*rs41656327*	9	101.106304	0.0924	6.4574	0.011265	0.0810	0.0135922557
ss117969645		9	101.241026	0.1239	0.5799	0.446623	0.0217	0.0047095423
ss86331967		9	101.275495	0.5000	0.3725	0.541844	0.0111	0.0055353161
ss86313699		9	101.300976	0.6390	3.2769	0.070694	0.0354	0.0163368692
ss86298218		9	101.337502	0.3001	6.4939	0.011038	0.0527	0.0221299691
ss61493583	*rs41588216*	9	101.364396	0.7636	3.4650	0.063104	0.0418	0.0150989833
ss86300835		9	101.393315	0.8281	3.9948	0.046029	0.0501	0.0142696531
ss86341278		9	101.417179	0.2357	2.4011	0.121708	0.0344	0.0123795985
ss86329450		9	101.441449	0.5852	5.2594	0.022125	0.0438	0.0212877037
ss86335373		9	101.590455	0.4678	0.3428	0.558402	0.0108	0.0053527332
ss61562044	*rs41657150*	9	101.652862	0.2650	1.9555	0.162440	0.0300	0.0116894986
ss61562045	*rs41657151*	9	101.723747	0.3181	0.5553	0.456431	0.0151	0.0065525733
ss86337226		9	101.746779	0.7493	0.2087	0.647950	0.0096	0.0036196809
ss61562051	*rs41657157*	9	101.789323	0.7027	0.9085	0.340853	0.0194	0.0081077753
ss86322234		9	101.851619	0.3245	10.1868	0.001478	0.0650	0.0284767520
ss117969632		9	101.91461	0.6254	9.2184	0.002485	0.0602	0.0281911981
ss61562065	*rs41657169*	9	102.204506	0.7880	0.0990	0.753145	0.0070	0.0023254330
ss86341420		9	102.271587	0.1741	0.2735	0.601142	0.0130	0.0037308896
ss86341342		9	102.360314	0.3725	2.1003	0.147717	0.0279	0.0130437332
ss86295388		9	102.436349	0.8854	10.2839	0.001403	0.0935	0.0189767381
ss86340376		9	102.476577	0.4155	0.2880	0.591671	0.0102	0.0049736635
ss86308141		9	102.519092	0.5029	0.0665	0.796557	0.0049	0.0024333033
ss61562094	*rs41657198*	9	102.561223	0.4062	0.0874	0.767591	0.0056	0.0027012068
ss86337029		9	102.651231	0.3632	12.5944	0.000413	0.0683	0.0316055277
ss86341451		9	102.675734	0.2149	3.7970	0.051746	0.0430	0.0145226529
ss86285774		9	102.701531	0.6891	0.8771	0.349334	0.0185	0.0079359061
ss61511099	*rs41610024*	9	102.815772	0.9405	6.7120	0.009777	0.0996	0.0111449040
ss86336494		9	103.035008	0.4850	3.2429	0.072165	0.0337	0.0168245376
ss86315105		9	103.080856	0.1533	2.9641	0.085574	0.0450	0.0116721146
ss86288626		9	103.103998	0.1590	2.9013	0.088953	0.0433	0.0115702232
ss86340553		9	103.138944	0.8854	0.0015	0.969366	0.0011	0.0002275884
ss86299360		9	103.225024	0.1905	0.1716	0.678835	0.0098	0.0030175711
ss61493599	*rs41589232*	9	103.282244	0.3539	0.7822	0.376767	0.0170	0.0077523754
ss86309506		9	103.346537	0.4011	0.0117	0.913732	0.0021	0.0009955791
ss61472414	*rs41570849*	9	103.417346	0.2170	0.0238	0.877525	0.0035	0.0011819860
ss61493623	*rs41589256*	9	103.488684	0.3052	1.0660	0.302215	0.0206	0.0087276808
ss61562130	*rs41658134*	9	103.533283	0.8954	0.8883	0.346253	0.0287	0.0053815102

ss61493622	*rs41589255*	9	103.601637	0.2285	4.4795	0.034658	0.0452	0.0159288205
ss86283243		9	103.659514	0.4105	2.1245	0.145412	0.0275	0.0133267071
ss61493616	*rs41589249*	9	103.775975	0.6110	6.7489	0.009579	0.0485	0.0230658198
ss86279859		9	103.899331	0.1404	0.1210	0.728007	0.0096	0.0023066543
ss86300714		9	103.925238	0.8567	0.4485	0.503271	0.0183	0.0044898643
	rs29022857	9	103.975784	0.3947	5.3656	0.020828	0.0440	0.0210437432
ss86281782		9	104.024547	0.5172	8.1082	0.004537	0.0534	0.0266704611
ss61504866	*rs41600741*	9	104.073154	0.6304	6.1541	0.013346	0.0480	0.0223578614
ss117964097		9	104.195617	0.6870	0.8626	0.353344	0.0183	0.0078680063
ss61562115	*rs41657219*	9	104.222717	0.3087	0.4275	0.513421	0.0128	0.0054533196
ss61516025	*rs41610951*	9	104.333966	0.2414	9.1752	0.002544	0.0643	0.0235344415
ss86326053		9	104.389554	0.7471	8.0054	0.004798	0.0598	0.0225968781
ss86337569		9	104.441903	0.2070	10.4143	0.001309	0.0711	0.0233310653
ss61568685	*rs41665771*	9	104.462688	0.6791	1.0757	0.300024	0.0203	0.0088542927
ss61505736	*rs41608871*	9	104.499415	0.3209	8.7491	0.003203	0.0583	0.0253994063
ss86312644		9	104.519718	0.6361	1.6106	0.204832	0.0243	0.0112318932
ss86290757		9	104.560883	0.5451	14.0100	0.000197	0.0690	0.0342043206
ss86274291		9	104.581465	0.8345	0.1868	0.665739	0.0106	0.0029344577
ss86317203		9	104.643271	0.3302	0.0001	0.992930	0.0002	0.0000756515
ss61553251	*rs43710501*	9	104.723209	0.4305	2.9616	0.085709	0.0331	0.0162093843
ss86302221		9	104.797482	0.5136	6.3752	0.011794	0.0466	0.0232776395
ss86327858		9	104.865593	0.1232	0.0935	0.759846	0.0086	0.0018630064
	rs29023535	9	104.932464	0.7822	1.4782	0.224465	0.0278	0.0094641291
ss61469455	*rs41570362*	9	104.993111	0.6383	5.1560	0.023471	0.0434	0.0200589222
ss86287862		9	105.015862	0.0609	1.4277	0.232541	0.0446	0.0050988294
ss86307637		9	105.067571	0.3367	0.3167	0.573751	0.0108	0.0048314921
ss86317330		9	105.092377	0.1640	2.2789	0.131596	0.0377	0.0103320395
ss86335767		9	105.172904	0.5852	2.6201	0.105974	0.0307	0.0149032383
	rs29018722	9	105.238552	0.7650	0.6819	0.409204	0.0184	0.0065996147
ss86337324		9	105.28572	0.5559	0.4985	0.480392	0.0130	0.0064172212
ss86341438		9	105.408677	0.8123	0.3335	0.563813	0.0134	0.0040726365
ss86298353		9	105.436232	0.6741	2.1767	0.140564	0.0295	0.0129653804
ss86316019		9	105.471833	0.5344	1.4415	0.230313	0.0231	0.0114723399
ss86304974		9	105.540439	0.5645	4.2307	0.040071	0.0399	0.0196337460
ss86314706		9	105.676731	0.3582	0.9286	0.335554	0.0190	0.0087422324
ss86302840		9	105.704978	0.1640	2.1628	0.141839	0.0375	0.0102833316
ss86307035		9	105.745279	0.1719	0.7034	0.401924	0.0208	0.0059120674
ss86308920		9	105.816623	0.3195	0.7379	0.390634	0.0175	0.0076218548
ss86290732		9	105.856088	0.3438	0.4032	0.525639	0.0120	0.0054222149
ss86317558		9	105.894472	0.6160	0.3387	0.560772	0.0118	0.0056034304
ss86337965		9	105.920758	0.3231	1.1249	0.289236	0.0220	0.0096220510
ss86339476		9	105.954836	0.6096	4.2080	0.040607	0.0396	0.0188688718
ss86339058		9	105.993179	0.7436	0.0527	0.818567	0.0047	0.0018050162
ss86330783		9	106.02694	0.2872	1.0694	0.301437	0.0203	0.0082922810
ss86292262		9	106.05221	0.2292	1.5755	0.209828	0.0268	0.0094709091
ss86291837		9	106.081358	0.5107	3.2889	0.070181	0.0350	0.0175024202
ss117969693		9	106.108336	0.9362	0.4553	0.500054	0.0248	0.0029587973

ss86327901		9	106.136492	0.2579	1.2434	0.265197	0.0230	0.0087969841
ss86313766		9	106.157975	0.3811	0.0595	0.807417	0.0047	0.0022043946
ss86324516		9	106.201788	0.6440	0.6547	0.418713	0.0159	0.0072966804
ss86278210		9	106.226558	0.4542	0.0556	0.813637	0.0044	0.0021957897
ss86319501		9	106.325553	0.4620	0.0120	0.912899	0.0021	0.0010547247
ss86296825		9	106.414098	0.3811	2.3135	0.128706	0.0292	0.0137720108
ss86284040		9	106.437678	0.5193	0.8964	0.344089	0.0180	0.0089941944
ss86341282		9	106.530192	0.4176	0.6333	0.426407	0.0146	0.0070823756
ss86337481		9	106.621284	0.1397	2.2861	0.130989	0.0390	0.0093667362
ss61466210	*rs41566870*	9	106.668145	0.8109	0.5518	0.457838	0.0177	0.0054375355
ss86323412		9	106.717245	0.3940	0.2778	0.598346	0.0099	0.0047109826
ss86283750		9	106.749812	0.1053	0.2353	0.627773	0.0145	0.0027412762
ss86275156		9	106.794854	0.0544	0.4759	0.490512	0.0272	0.0028021989
ss86308434		9	106.825007	0.0874	0.4933	0.482715	0.0234	0.0037402016
ss86317807		9	106.851426	0.7185	0.6655	0.414916	0.0169	0.0068259419
ss86275347		9	106.877985	0.8302	4.7172	0.030200	0.0529	0.0149243698
ss61565044	*rs41657708*	9	106.912369	0.6540	1.9815	0.159676	0.0264	0.0119641912
ss86318507		9	107.005751	0.1956	9.2964	0.002383	0.0703	0.0221280468
ss86274772		9	107.061286	0.7135	1.7205	0.190055	0.0273	0.0111697920
ss86290564		9	107.19642	0.2514	3.3655	0.067003	0.0399	0.0150038299
ss86340231		9	107.227876	0.3746	2.6893	0.101478	0.0313	0.0146684782
ss86313662		9	107.286656	0.3625	2.1691	0.141265	0.0283	0.0130998977
ss86338238		9	107.310637	0.3238	1.1409	0.285828	0.0207	0.0090760033
ss61506305	*rs41604520*	9	107.344738	0.3403	0.0010	0.974568	0.0006	0.0002751014
ss86301512		9	107.392503	0.6060	4.4520	0.035216	0.0399	0.0190737026
ss86336517		9	107.419845	0.1110	0.6063	0.436454	0.0228	0.0044964084
ss61524207	*rs41619164*	9	107.458921	0.5100	8.5994	0.003473	0.0543	0.0271248217
ss86339417		9	107.552593	0.2357	0.2858	0.593111	0.0115	0.0041490871
ss86309510		9	107.597162	0.8023	1.5983	0.206570	0.0298	0.0094691040
ss86310257		9	107.622501	0.1848	0.1279	0.720770	0.0086	0.0025985814
ss86298642		9	107.678537	0.1898	0.0538	0.816717	0.0054	0.0016614166
ss86274260		9	107.962209	0.1862	2.8872	0.089731	0.0404	0.0122495214
ss86337749		9	108.072259	0.4936	1.9526	0.162751	0.0260	0.0129828038
ss86332117		10	0.557027	0.2887	0.0178	0.893885	0.0027	0.0010972791
ss117964147		10	0.648932	0.3102	0.3814	0.537062	0.0125	0.0053299646
ss86335743		10	0.729149	0.5938	0.5106	0.475103	0.0133	0.0064292070
ss61478346	*rs41575746*	10	0.772105	0.8746	0.0057	0.939818	0.0021	0.0004583749
ss86334410		10	0.863064	0.0752	0.0020	0.964256	0.0016	0.0002224435
ss86314696		10	0.887654	0.3109	0.6749	0.411634	0.0161	0.0068885409
ss61515125	*rs41611986*	10	0.915095	0.3030	3.9741	0.046594	0.0415	0.0175484285
ss61515814	*rs41617356*	10	0.980527	0.1905	2.2013	0.138348	0.0339	0.0104535666
ss86327353		10	1.019176	0.4384	0.1591	0.690121	0.0073	0.0035785740
ss86303663		10	1.058416	0.5473	0.0391	0.843249	0.0039	0.0019086661
	rs29027600	10	1.288074	0.8532	1.2581	0.262391	0.0286	0.0071589519
ss86273585		10	1.447764	0.2106	0.5473	0.459678	0.0167	0.0055452300
	rs29014484	10	1.483572	0.4405	1.2854	0.257288	0.0210	0.0103679561
ss86339109		10	1.517453	0.8288	0.4359	0.509335	0.0157	0.0044433710

ss86288654		10	1.607109	0.9312	1.3132	0.252203		0.0413	0.0052851268
ss86298169		10	1.649037	0.7278	0.3928	0.531011		0.0134	0.0053195868
ss61476899	*rs41580149*	10	1.770631	0.7980	3.6391	0.056849		0.0446	0.0143931815
ss86330045		10	1.839417	0.4470	0.0950	0.758034		0.0059	0.0029288917
ss61527404	*rs41623366*	10	1.862681	0.8589	1.0176	0.313447		0.0274	0.0066364160
ss86338933		10	1.888338	0.2937	0.0015	0.968836		0.0008	0.0003217064
ss61519832	*rs41618869*	10	1.972167	0.4993	0.9418	0.332150		0.0176	0.0088043645
ss86284171		10	2.073295	0.3388	1.6735	0.196215		0.0253	0.0113231599
ss86313655		10	2.149357	0.4549	0.0053	0.941792		0.0014	0.0006743727
ss61519834	*rs41618871*	10	2.172947	0.4140	1.5022	0.220755		0.0227	0.0110272087
ss61487143	*rs41587818*	10	2.258969	0.5781	0.5898	0.442759		0.0143	0.0069514697
ss86284664		10	2.306533	0.5609	1.8249	0.177169		0.0245	0.0120556660
ss86310901		10	2.377497	0.4513	0.9296	0.335297		0.0173	0.0085769999
ss86336164		10	2.403282	0.4771	1.1952	0.274671		0.0194	0.0096766785
ss86308352		10	2.427644	0.4878	1.3227	0.250495		0.0217	0.0108226837
	rs29027204	10	2.473345	0.0960	0.2408	0.623808		0.0156	0.0027147176
ss86290311		10	2.660596	0.1032	0.4343	0.510121		0.0206	0.0038034074
ss86338662		10	2.690048	0.3152	2.5332	0.111931		0.0307	0.0132671188
ss86308967		10	2.797232	0.3668	0.3853	0.534994		0.0117	0.0054269958
ss64872489	*rs42962818*	10	2.818174	0.7350	5.0733	0.024607		0.0478	0.0186349250
ss86300364		10	2.841924	0.3976	0.5547	0.456667		0.0140	0.0067259233
ss63857278	*rs42386397*	10	2.876404	0.7507	3.4587	0.063342		0.0406	0.0151858131
ss86286547		10	2.910712	0.5867	5.7322	0.016920		0.0446	0.0216230176
ss86321111		10	2.997286	0.6648	0.6991	0.403371		0.0161	0.0071858178
ss117969801		10	3.034699	0.5931	1.5590	0.212225		0.0247	0.0119014506
ss86336198		10	3.057414	0.3961	5.0174	0.025409		0.0436	0.0208765893
	rs29012068	10	3.10016	0.2880	6.1412	0.013443		0.0503	0.0206147315
ss64550114	*rs42697353*	10	3.129044	0.4792	5.2301	0.022499		0.0428	0.0213670273
ss64549954	*rs42696473*	10	3.15262	0.0444	1.1883	0.276051		0.0472	0.0040097246
ss86311128		10	3.260458	0.4083	0.3647	0.546128		0.0117	0.0056502199
ss61496423	*rs41598672*	10	3.316687	0.3617	0.1105	0.739623		0.0066	0.0030404631
ss86320912		10	3.371027	0.7872	0.4685	0.493918		0.0153	0.0051374966
ss86315635		10	3.459883	0.8145	0.0783	0.779690		0.0066	0.0019900701
ss86326992		10	3.514955	0.2958	11.3299	0.000805		0.0685	0.0285470925
ss86318748		10	3.646693	0.2815	0.2870	0.592343		0.0109	0.0044219200
ss86326342		10	3.722265	0.3016	2.0137	0.156327		0.0284	0.0119438407
ss117969966		10	3.791276	0.9076	0.8934	0.344877		0.0298	0.0049961667
	rs29022216	10	3.820157	0.5860	2.0860	0.149101		0.0268	0.0129970678
ss86329774		10	3.840464	0.6153	6.2571	0.012598		0.0468	0.0221421643
ss86323734		10	3.90578	0.8166	0.2213	0.638235		0.0114	0.0034211231
ss61498362	*rs43711263*	10	3.949015	0.3854	0.6776	0.410687		0.0158	0.0074627323
ss117969786		10	3.980899	0.2607	0.2090	0.647659		0.0095	0.0036475756
ss61480455	*rs41578099*	10	4.067117	0.7787	3.2558	0.071603		0.0404	0.0139324325
ss61475342	*rs41573822*	10	4.104288	0.6676	1.6146	0.204278		0.0247	0.0109809257
ss86310751		10	4.178537	0.2457	4.1499	0.042014		0.0444	0.0164658212
ss86288422		10	4.334646	0.7013	0.5803	0.446449		0.0163	0.0068174216
	rs29024571	10	4.359554	0.7715	0.0736	0.786197		0.0061	0.0021495131

ss86288789		10	4.497181	0.8238	0.0000	0.995641		0.0001	0.0000395155
ss61552468	*rs43710822*	10	4.732552	0.4470	5.7032	0.017200		0.0446	0.0220662557
ss61526470	*rs41623661*	10	4.756591	0.5523	5.8698	0.015657		0.0452	0.0223645722
ss86285189		10	4.797903	0.2650	0.3739	0.541067		0.0133	0.0051781855
ss86295798		10	4.848688	0.3961	0.2121	0.645307		0.0084	0.0040095323
ss86337955		10	4.879422	0.5981	0.5156	0.472976		0.0130	0.0062689160
ss86328362		10	4.945003	0.3567	0.0168	0.896822		0.0025	0.0011449334
ss61556729	*rs41657031*	10	4.979261	0.4921	0.4846	0.486588		0.0132	0.0066113633
ss86290456		10	5.025113	0.1741	7.1392	0.007718		0.0638	0.0183326781
ss86284692		10	5.055794	0.4678	1.1290	0.288356		0.0202	0.0100580641
ss86323678		10	5.193671	0.4090	0.0465	0.829348		0.0042	0.0020088380
ss86314855		10	5.231228	0.7163	1.6307	0.202038		0.0268	0.0108830886
ss86336493		10	5.25214	0.1891	2.6815	0.101973		0.0376	0.0115415872
ss61507518	*rs41606710*	10	5.289511	0.6605	0.0122	0.912224		0.0022	0.0009928343
ss105238084		10	5.459855	0.3181	0.0109	0.916783		0.0021	0.0009273252
ss86336370		10	5.60479	0.1275	8.6714	0.003340		0.0801	0.0178230385
ss117970021		10	5.630804	0.6060	0.2007	0.654265		0.0086	0.0040870496
ss86341622		10	5.664729	0.7042	0.7480	0.387397		0.0176	0.0073235970
	rs29016875	10	5.69951	0.1139	5.0654	0.024719		0.0646	0.0130295398
ss86322330		10	5.741927	0.1848	2.6852	0.101740		0.0392	0.0118190106
ss86337067		10	5.797442	0.4047	0.7378	0.390650		0.0167	0.0080342097
ss86341029		10	5.925159	0.4857	4.8138	0.028563		0.0417	0.0208423099
ss105238125		10	5.946571	0.3489	0.5793	0.446837		0.0143	0.0064840887
ss86337337		10	5.977669	0.3761	0.4224	0.515941		0.0120	0.0056446746
ss86292297		10	6.017455	0.3453	4.5433	0.033398		0.0418	0.0188876203
ss86306033		10	6.048028	0.3933	2.1080	0.146977		0.0268	0.0128021886
ss86338528		10	6.08049	0.0867	0.0120	0.912722		0.0037	0.0005853361
ss86305057		10	6.127404	0.3352	0.7927	0.373604		0.0174	0.0077697185
ss61477648	*rs41578577*	10	6.14889	0.4026	0.7907	0.374197		0.0167	0.0080317419
ss61477642		10	6.226444	0.6490	0.2152	0.642853		0.0091	0.0041553345
ss86287920		10	6.248185	0.4377	3.0767	0.079860		0.0322	0.0158635854
ss86283260		10	6.273374	0.2880	0.1784	0.672920		0.0086	0.0035318268
ss86339217		10	6.305368	0.1175	0.0021	0.963412		0.0013	0.0002790634
ss61549842	*rs41645638*	10	6.397102	0.2493	3.9399	0.047546		0.0420	0.0157143930
ss61549812	*rs41644709*	10	6.493882	0.7256	2.1746	0.140757		0.0307	0.0122309230
ss86289471		10	6.647599	0.5050	0.2437	0.621685		0.0089	0.0044661285
ss61549266	*rs41647563*	10	6.704916	0.0559	0.0620	0.803379		0.0100	0.0010566703
ss86325258		10	6.758359	0.2020	3.9733	0.046616		0.0462	0.0149012924
ss61548744	*rs41644760*	10	6.942261	0.2221	0.9366	0.333495		0.0213	0.0073675677
ss86338493		10	6.995824	0.5337	2.2406	0.134880		0.0275	0.0137106895
ss61548265	*rs41647472*	10	7.037829	0.3181	4.7152	0.030234		0.0430	0.0186599604
ss86323923		10	7.083154	0.8868	3.2292	0.072768		0.0532	0.0106820929
ss86323174		10	7.121081	0.8668	0.1021	0.749372		0.0087	0.0020081885
ss117969930		10	7.179469	0.6447	0.1025	0.748987		0.0061	0.0027944817
ss86329584		10	7.210626	0.6225	0.0088	0.925361		0.0018	0.0008441598
ss86286222		10	7.231931	0.2679	0.5348	0.464851		0.0150	0.0058960326
ss86309755		10	7.282566	0.9484	1.1231	0.289621		0.0446	0.0043588511

ss86310178		10	7.313262	0.3832	3.7023	0.054745	0.0369	0.0174338539
ss61474668	*rs41571663*	10	7.359045	0.8460	0.4630	0.496461	0.0175	0.0045648616
ss86337314		10	7.429386	0.4785	5.6451	0.017774	0.0430	0.0214750897
ss86317616		10	7.467464	0.4506	0.7812	0.377077	0.0163	0.0080692291
ss86298943		10	7.531428	0.3553	0.9602	0.327466	0.0191	0.0087342605
ss86328134		10	7.58856	0.3030	0.0555	0.813773	0.0047	0.0019949526
ss86335688		10	7.778383	0.2350	0.1885	0.664273	0.0092	0.0033093280
ss86332995		10	7.814328	0.3116	3.8211	0.051011	0.0389	0.0167044930
ss117970024		10	7.858887	0.7543	4.5230	0.033794	0.0457	0.0169221468
ss117969795		10	7.88644	0.6096	0.0323	0.857504	0.0034	0.0016042738
ss86329465		10	7.909649	0.5688	1.8194	0.177818	0.0245	0.0120029596
ss86331276		10	8.026422	0.6511	0.2103	0.646678	0.0090	0.0041070460
ss117962532		10	8.049873	0.1504	0.2737	0.601005	0.0136	0.0034694894
ss86288221		10	8.078778	0.2299	0.1943	0.659465	0.0096	0.0033881100
ss86335578		10	8.135519	0.8001	0.3163	0.574034	0.0129	0.0041117946
ss86339464		10	8.170262	0.6246	0.0236	0.877871	0.0030	0.0013868539
ss86282887		10	8.208481	0.8617	4.5843	0.032613	0.0575	0.0136938073
ss61551950	*rs43707020*	10	8.295325	0.1060	0.4270	0.513702	0.0197	0.0037321137
ss61488692	*rs41586937*	10	8.330344	0.9140	3.6823	0.055402	0.0625	0.0098233699
ss86341285		10	8.36572	0.5953	0.8696	0.351387	0.0174	0.0083942979
	rs29016947	10	8.432881	0.5967	0.2850	0.593629	0.0100	0.0048052237
ss86324350		10	8.493047	0.5573	1.4634	0.226803	0.0222	0.0109655033
	rs29015344	10	8.522422	0.7830	0.9853	0.321240	0.0221	0.0075026864
ss86307766		10	8.887867	0.6547	3.1954	0.074282	0.0349	0.0157565956
ss86284452		10	8.988431	0.3625	2.9744	0.085037	0.0333	0.0153884176
ss86314007		10	9.018362	0.3245	0.0134	0.907761	0.0024	0.0010432149
ss86299542		10	9.237561	0.2915	5.8008	0.016278	0.0488	0.0201452746
ss86338118		10	9.292548	0.6232	0.3375	0.561444	0.0109	0.0051150823
ss86294243		10	9.350712	0.1218	4.2280	0.040134	0.0578	0.0123705054
ss105296777		10	9.392662	0.1082	0.0465	0.829318	0.0065	0.0012458474
ss86309045		10	9.439543	0.3840	0.0505	0.822315	0.0042	0.0019836629
ss86337393		10	9.467862	0.0924	0.0245	0.875576	0.0050	0.0008364039
ss86285451		10	9.499521	0.2579	4.6591	0.031231	0.0449	0.0171795452
ss86333924		10	9.576936	0.6676	0.0011	0.973202	0.0007	0.0002992386
ss86282947		10	9.688344	0.2636	0.0674	0.795307	0.0054	0.0020952897
ss86339648		10	9.760323	0.1927	2.2893	0.130725	0.0369	0.0114676881
ss86335612		10	9.801559	0.1963	0.0240	0.876996	0.0036	0.0011456866
ss86307843		10	9.842777	0.3159	5.9423	0.015031	0.0479	0.0207065764
ss61518361	*rs41613225*	10	9.874408	0.1877	0.0701	0.791322	0.0063	0.0019211065
ss105241675		10	9.985952	0.4756	3.0951	0.078968	0.0323	0.0160975325
ss86339770		10	10.076641	0.2364	1.0961	0.295493	0.0229	0.0082560856
ss86274278		10	10.119573	0.7794	0.3649	0.545999	0.0136	0.0046763038
	rs29019953	10	10.149754	0.8589	0.1539	0.694973	0.0105	0.0025467360
ss61555697	*rs41656695*	10	10.350926	0.3374	0.3980	0.528314	0.0124	0.0055657139
ss86339917		10	10.37163	0.6777	0.4646	0.495728	0.0136	0.0059213362
ss86307719		10	10.502242	0.1526	3.0447	0.081444	0.0452	0.0116963984
ss86298634		10	10.595915	0.6175	0.0489	0.825109	0.0042	0.0019913144

ss65531762	*rs43616744*	10	10.724077	0.7944	4.8106	0.028615	0.0486	0.0158781113
ss86321648		10	10.752752	0.2378	2.9035	0.088835	0.0386	0.0139999388
ss65531704	*rs43616186*	10	10.777376	0.3381	0.0468	0.828850	0.0043	0.0019367905
ss86326811		10	10.815124	0.6920	16.1984	0.000063	0.0794	0.0338477728
ss86334205		10	10.850592	0.1748	0.8001	0.371381	0.0214	0.0061770995
ss86331723		10	10.871879	0.1003	0.2705	0.603152	0.0154	0.0027874773
ss65531472	*rs43614654*	10	10.898725	0.7851	1.3097	0.252843	0.0252	0.0084878618
ss86296393		10	10.925926	0.7572	0.4444	0.505237	0.0151	0.0055362073
ss86325460		10	10.948238	0.8675	0.9424	0.332003	0.0269	0.0061759768
ss86336778		10	10.976145	0.1905	2.0936	0.148371	0.0332	0.0102389266
ss86281080		10	10.998462	0.0330	0.0628	0.802246	0.0125	0.0007981116
ss65531180	*rs43612564*	10	11.037491	0.2120	0.0005	0.982417	0.0005	0.0001673318
ss65531228	*rs43612612*	10	11.074733	0.8438	0.4287	0.512866	0.0168	0.0044344097
ss65531301	*rs43613184*	10	11.16456	0.0731	0.1685	0.681561	0.0139	0.0018812100
ss65534483	*rs43618959*	10	11.24132	0.8890	6.1949	0.013045	0.0720	0.0142186320
ss86337076		10	11.271994	0.2163	0.4552	0.500090	0.0152	0.0051600452
ss86316818		10	11.292422	0.4176	2.0564	0.152021	0.0264	0.0128296166
ss65534497	*rs43618973*	10	11.344781	0.9033	2.0199	0.155698	0.0437	0.0076345254
ss65065669	*rs43158016*	10	11.394109	0.2249	8.9163	0.002926	0.0643	0.0224321481
ss65534559	*rs43620235*	10	11.453471	0.7421	1.1760	0.278554	0.0240	0.0091847028
ss64829885	*rs42921077*	10	11.522041	0.7307	0.6316	0.427032	0.0174	0.0068500200
	rs29024630	10	11.548328	0.1669	6.7008	0.009838	0.0626	0.0174191994
ss64792865	*rs42889844*	10	11.575735	0.7701	2.1233	0.145527	0.0326	0.0115379619
ss61561788	*rs41664395*	10	11.633721	0.1805	1.8181	0.177973	0.0329	0.0097264239
ss86299661		10	11.682625	0.0616	0.2118	0.645485	0.0171	0.0019798385
ss65535065	*rs43613632*	10	11.755419	0.1805	1.0534	0.305072	0.0241	0.0071320835
ss86297028		10	11.852953	0.8238	2.4190	0.120323	0.0377	0.0109380939
ss65535400	*rs43615966*	10	11.873889	0.4520	1.3228	0.250494	0.0210	0.0103889983
ss65535424	*rs43615990*	10	11.911525	0.6089	1.1928	0.275146	0.0204	0.0097119663
ss61561790	*rs41664397*	10	11.93839	0.0702	0.5622	0.453622	0.0271	0.0035383873
ss86290579		10	11.991393	0.7178	0.1590	0.690188	0.0084	0.0034153418
ss86306278		10	12.075569	0.0795	0.0257	0.872697	0.0056	0.0008183613
ss86323090		10	12.099791	0.7815	0.0679	0.794503	0.0059	0.0020194159
ss61561794	*rs41664401*	10	12.125696	0.6748	0.1699	0.680303	0.0081	0.0035459459
ss86310543		10	12.149324	0.1017	0.0022	0.962794	0.0015	0.0002737350
ss117969969		10	12.22622	0.7679	4.1440	0.042160	0.0437	0.0155933642
ss86310362		10	12.261551	0.0946	1.0120	0.314781	0.0314	0.0053808842
ss86322004		10	12.288079	0.2937	0.1984	0.656126	0.0089	0.0037008698
ss86324763		10	12.361828	0.1834	0.1598	0.689479	0.0095	0.0028562535
ss61493409	*rs41594941*	10	12.394224	0.6841	0.4667	0.494748	0.0138	0.0059435393
	rs29013264	10	12.484782	0.4277	1.5232	0.217552	0.0234	0.0114782024
ss64609068	*rs42724716*	10	12.534813	0.8904	2.0417	0.153486	0.0399	0.0077856901
ss86334476		10	12.585173	0.1827	0.0001	0.991042	0.0003	0.0000801830
ss86288819		10	12.606114	0.0702	4.7876	0.028997	0.0780	0.0101870101
ss86327099		10	12.626969	0.0709	5.7988	0.016296	0.0855	0.0112643680
	rs29021091	10	12.645044	0.0702	4.7876	0.028997	0.0780	0.0101870101
ss86328401		10	12.695255	0.3460	11.5821	0.000704	0.0641	0.0290104847

ss86297641		10	12.717367	0.6827	0.2963	0.586366	0.0108	0.0046794249
ss86330569		10	12.749194	0.2049	0.6606	0.416617	0.0188	0.0061309681
ss86333695		10	12.776959	0.6433	0.3247	0.568973	0.0108	0.0049514635
ss65532605	*rs43616983*	10	12.799367	0.5530	6.3985	0.011641	0.0459	0.0226762913
ss61491624	*rs41589011*	10	12.838546	0.8596	7.5642	0.006108	0.0738	0.0178082722
ss86298948		10	12.87822	0.1540	3.7066	0.054604	0.0482	0.0125505502
ss86336124		10	12.899363	0.6017	0.0622	0.803115	0.0048	0.0023073090
ss86329256		10	12.953683	0.7414	0.0485	0.825762	0.0047	0.0017988250
ss86326599		10	13.034549	0.4799	3.6442	0.056675	0.0356	0.0177819922
ss86301598		10	13.072923	0.6755	0.4014	0.526588	0.0126	0.0055180258
ss46526290	*rs41255429*	10	13.104345	0.5480	3.3986	0.065676	0.0342	0.0169195643
ss86324415		10	13.176125	0.3467	0.1175	0.731849	0.0068	0.0030754208
ss65534005	*rs43613384*	10	13.197923	0.7507	0.0061	0.937729	0.0017	0.0006235388
ss62337924	*rs41839792*	10	13.225603	0.7199	0.0729	0.787190	0.0057	0.0022791470
ss65533992	*rs43613371*	10	13.247673	0.0595	0.7034	0.401942	0.0336	0.0037612671
ss86308340		10	13.274059	0.6741	2.2301	0.135802	0.0282	0.0123958772
ss65533958	*rs43613337*	10	13.29433	0.6798	2.6662	0.102956	0.0311	0.0135377044
ss86336534		10	13.315665	0.2235	0.5053	0.477399	0.0149	0.0051887800
ss105239515		10	13.343961	0.7364	0.2804	0.596615	0.0111	0.0043111106
ss86327559		10	13.369709	0.2378	0.5203	0.470972	0.0153	0.0055314543
ss65533865	*rs43612145*	10	13.391642	0.3274	0.2418	0.623075	0.0098	0.0043056638
ss65533827	*rs43622407*	10	13.474906	0.2228	0.6636	0.415563	0.0180	0.0062473626
ss117964127		10	13.514227	0.6132	0.1302	0.718302	0.0068	0.0032371679
ss86301191		10	13.59171	0.4119	2.1041	0.147354	0.0267	0.0129491794
ss86338343		10	13.612165	0.9391	0.5512	0.458067	0.0288	0.0032891607
ss86299114		10	13.644834	0.8739	0.2980	0.585293	0.0150	0.0033163518
ss86325631		10	13.666564	0.4542	3.2515	0.071793	0.0335	0.0165965107
ss61560096	*rs41655621*	10	13.703506	0.3467	4.3844	0.036629	0.0412	0.0186649696
ss86285143		10	13.730463	0.6691	4.3809	0.036705	0.0411	0.0181806255
ss86322260		10	13.817212	0.5867	0.6453	0.422076	0.0147	0.0071461178
ss86332789		10	13.839922	0.1748	0.0008	0.977472	0.0007	0.0002014051
ss65533559	*rs43620039*	10	13.936705	0.4885	2.9263	0.087594	0.0312	0.0155984443
ss86304627		10	13.961943	0.3073	3.4386	0.064111	0.0380	0.0161658931
ss65533448	*rs43617528*	10	14.028462	0.3854	0.2057	0.650314	0.0086	0.0040959825
ss105239516		10	14.071412	0.3825	0.3798	0.537915	0.0117	0.0055449807
ss86336696		10	14.099231	0.3825	0.5190	0.471516	0.0138	0.0065023742
ss86318566		10	14.154478	0.6905	0.0258	0.872490	0.0032	0.0013643574
ss61492119	*rs41594274*	10	14.186894	0.3095	0.0258	0.872490	0.0032	0.0013643574
ss86313812		10	14.285134	0.3718	11.6043	0.000696	0.0634	0.0296220741
ss86320843		10	14.320715	0.4893	0.0775	0.780835	0.0051	0.0025441924
ss86335682		10	14.412072	0.7765	0.7017	0.402501	0.0188	0.0065098997
ss117969826		10	14.451559	0.7056	1.3212	0.250768	0.0230	0.0095646533
ss86300858		10	14.471604	0.7765	6.0308	0.014301	0.0552	0.0191464273
ss86338087		10	14.522573	0.5960	17.8976	0.000026	0.0761	0.0366398232
ss61514489	*rs41613055*	10	14.563522	0.3990	3.0629	0.080539	0.0337	0.0161795411
ss38334949	*rs29024841*	10	14.675597	0.1562	0.0148	0.903247	0.0031	0.0008100612
ss86322994		10	14.893832	0.3632	3.3236	0.068722	0.0336	0.0155348701

	rs29020937	10	14.915334	0.8087	12.2635	0.000491	0.0812	0.0251174229
ss86305935		10	14.944621	0.4792	0.0179	0.893533	0.0025	0.0012724981
ss86307243		10	14.969075	0.3309	0.5013	0.479161	0.0135	0.0059689243
ss86339718		10	15.006349	0.7536	2.0735	0.150331	0.0300	0.0111549719
ss86337379		10	15.045341	0.6096	1.5527	0.213155	0.0233	0.0111007231
ss86290351		10	15.067097	0.3696	7.2217	0.007375	0.0494	0.0230386780
ss86299289		10	15.101458	0.3933	1.1668	0.280426	0.0202	0.0096505380
ss86341617		10	15.137264	0.6032	5.5396	0.018868	0.0417	0.0199572750
ss61548242	*rs41647455*	10	15.172466	0.7213	2.0655	0.151113	0.0282	0.0113337883
ss86277049		10	15.196084	0.8059	3.6974	0.054906	0.0470	0.0147152308
ss86324618		10	15.252638	0.4979	1.1912	0.275468	0.0198	0.0098774878
ss86322117		10	15.298901	0.4577	5.9332	0.015108	0.0425	0.0211196651
ss86327825		10	15.331905	0.1117	1.5172	0.218458	0.0362	0.0071793235
ss61479208	*rs41576179*	10	15.35474	0.9785	5.8202	0.016101	0.1529	0.0064296247
ss86303265		10	15.513723	0.1067	0.0105	0.918401	0.0030	0.0005702717
ss61568665	*rs41665751*	10	15.578192	0.1662	0.0070	0.933535	0.0021	0.0005684956
ss64377081	*rs42612671*	10	15.659184	0.3209	0.7640	0.382381	0.0168	0.0073109153
ss65533572	*rs43620052*	10	15.775383	0.9298	6.7868	0.009379	0.0948	0.0123793518
ss117970026		10	15.794482	0.3173	6.8930	0.008843	0.0506	0.0219023668
ss86307953		10	15.832596	0.2794	4.7141	0.030254	0.0448	0.0180509507
ss86288339		10	15.866884	0.6941	3.5188	0.061095	0.0381	0.0161579214
ss86292363		10	15.919969	0.2966	5.4011	0.020411	0.0477	0.0198969904
ss86338535		10	16.082335	0.0831	6.2405	0.012716	0.0861	0.0131157772
ss86340410		10	16.125413	0.3610	4.1010	0.043240	0.0376	0.0173337712
ss86321276		10	16.154402	0.1282	0.0010	0.975167	0.0008	0.0001878444
ss86337384		10	16.211357	0.7342	1.8333	0.176176	0.0274	0.0107115644
ss61516096	*rs41611020*	10	16.250194	0.5351	0.0829	0.773485	0.0054	0.0027031307
ss105240214		10	16.392627	0.2966	4.5512	0.033245	0.0422	0.0175885310
ss86310001		10	16.415272	0.4499	4.0921	0.043467	0.0381	0.0188401399
ss86338016		10	16.437277	0.4692	2.2899	0.130671	0.0274	0.0136702879
ss86319980		10	16.466632	0.1390	0.3078	0.579187	0.0149	0.0035710359
ss86323420		10	16.512388	0.3202	3.9789	0.046462	0.0383	0.0166824159
ss86317957		10	16.6013	0.6275	2.7823	0.095759	0.0306	0.0143168970
ss117969782		10	16.686046	0.4212	0.0038	0.951186	0.0011	0.0005563485
ss86295364		10	16.706426	0.5208	3.2995	0.069729	0.0330	0.0164791977
ss86299461		10	16.734592	0.6433	1.5316	0.216293	0.0239	0.0109701519
ss86339670		10	16.761173	0.8102	0.0082	0.928090	0.0021	0.0006504408
ss86340995		10	16.797956	0.6612	10.2150	0.001456	0.0605	0.0271175445
ss86338832		10	16.822082	0.6211	1.7252	0.189453	0.0247	0.0116133889
ss61493150		10	16.880474	0.8274	0.3497	0.554476	0.0146	0.0041752674
ss86306566		10	16.903576	0.5616	5.3548	0.020956	0.0423	0.0208274169
ss86274866		10	16.960329	0.1769	3.6156	0.057653	0.0440	0.0128266503
ss86313827		10	17.012414	0.8216	4.2533	0.039544	0.0477	0.0139757666
ss86341611		10	17.03985	0.3968	0.7844	0.376119	0.0170	0.0081615948
ss86315233		10	17.091907	0.6117	0.1074	0.743168	0.0063	0.0029745707
ss61493140	*rs41592685*	10	17.12706	0.7722	6.8223	0.009197	0.0562	0.0197792343
ss61561137	*rs41657550*	10	17.147706	0.7500	0.4514	0.501900	0.0144	0.0053822516

ss86291466		10	17.177811	0.2830	2.3274	0.127565	0.0313	0.0126887821
ss86294035		10	17.208952	0.8617	2.0370	0.153956	0.0379	0.0090250921
ss86297572		10	17.259502	0.3095	0.0979	0.754517	0.0062	0.0026421103
ss86313644		10	17.374725	0.9405	0.0796	0.777876	0.0110	0.0012342731
ss86315896		10	17.402154	0.4678	0.0388	0.843849	0.0037	0.0018398475
ss86312257		10	17.48152	0.2636	0.0420	0.837590	0.0043	0.0016696599
ss86291794		10	17.583452	0.7400	0.2543	0.614251	0.0106	0.0040683357
ss86283423		10	17.663428	0.3990	7.1813	0.007541	0.0508	0.0243760531
ss86335622		10	17.693573	0.4004	0.2742	0.600684	0.0099	0.0047307620
ss61548747	*rs41644763*	10	17.857142	0.8410	3.7448	0.053378	0.0478	0.0127856388
ss86291036		10	17.885328	0.3639	0.3262	0.568060	0.0109	0.0050513271
ss86322619		10	17.953747	0.3023	0.0235	0.878275	0.0030	0.0012855504
ss61522982	*rs41623450*	10	18.013485	0.4255	2.1783	0.140423	0.0269	0.0131405516
ss86318723		10	18.061309	0.0444	0.1039	0.747289	0.0144	0.0012264347
ss61486914	*rs41586492*	10	18.107336	0.4749	3.0079	0.083302	0.0320	0.0159420158
ss86326369		10	18.160492	0.7314	1.9827	0.159558	0.0299	0.0117317126
ss86284884		10	18.216233	0.4914	4.0844	0.043663	0.0371	0.0185317760
ss86332231		10	18.268712	0.7779	0.8863	0.346808	0.0206	0.0071190134
ss61548754	*rs41644770*	10	18.357195	0.4642	0.0445	0.833030	0.0037	0.0018626498
ss86324465		10	18.444804	0.5688	0.6181	0.432036	0.0143	0.0070021602
	rs29025357	10	18.489784	0.5809	1.0756	0.300039	0.0190	0.0092287383
ss86332039		10	18.518758	0.4663	1.7334	0.188408	0.0248	0.0123228082
ss86290576		10	18.553572	0.0716	3.7038	0.054696	0.0689	0.0091606983
ss86307236		10	18.578031	0.7951	0.0137	0.906801	0.0027	0.0008894620
ss86314436		10	18.633559	0.6526	0.8192	0.365728	0.0176	0.0079870935
ss117970018		10	18.65462	0.8768	1.7930	0.181001	0.0387	0.0083671955
ss61525732	*rs41622436*	10	18.690306	0.0974	0.0053	0.941998	0.0023	0.0003996869
ss86333545		10	18.820914	0.0559	1.3592	0.244068	0.0454	0.0047942395
ss86341184		10	18.842693	0.7042	1.8616	0.172882	0.0280	0.0116766021
ss61502994	*rs41602305*	10	18.893343	0.9499	0.3299	0.565908	0.0241	0.0022928238
ss86305650		10	18.920847	0.6891	0.0551	0.814547	0.0048	0.0020446104
ss86287529		10	18.982584	0.4291	0.2581	0.611569	0.0096	0.0047095398
ss86302538		10	19.037765	0.1963	0.7956	0.372706	0.0206	0.0064952916
ss86282876		10	19.177511	0.8259	8.5960	0.003480	0.0693	0.0199371310
ss86280525		10	19.205404	0.0981	6.1939	0.013052	0.0754	0.0133499907
ss61531446	*rs41626107*	10	19.2444	0.5795	1.4988	0.221275	0.0228	0.0110889755
ss61531449	*rs41626110*	10	19.277284	0.1526	0.8310	0.362307	0.0239	0.0061903696
ss105236643		10	19.315327	0.5702	1.0466	0.306637	0.0193	0.0094769040
ss86324698		10	19.39684	0.1741	1.2522	0.263521	0.0276	0.0079403031
ss86340788		10	19.424044	0.5623	2.0658	0.151083	0.0278	0.0136814882
ss61526771	*rs41626343*	10	19.457961	0.3173	0.9796	0.322629	0.0198	0.0085924261
ss61548250	*rs41647457*	10	19.461	0.5788	4.8022	0.028754	0.0412	0.0200863501
ss86337129		10	19.497689	0.5272	1.9662	0.161295	0.0263	0.0131146984
ss86318336		10	19.539712	0.2170	0.2471	0.619278	0.0111	0.0037617518
ss86283582		10	19.563253	0.5244	1.7851	0.181963	0.0251	0.0124994615
ss86336805		10	19.741015	0.9083	0.6927	0.405549	0.0263	0.0043787586
ss86297047		10	19.84501	0.3589	1.3132	0.252215	0.0222	0.0102063880

ss86312335		10	19.876317	0.6748	1.2431	0.265251	0.0218	0.0095876352
ss86329764		10	19.941578	0.5688	0.0082	0.927826	0.0017	0.0008449064
ss86273613		10	20.010708	0.1963	6.4985	0.011010	0.0600	0.0189311334
ss117970031		10	20.036047	0.5401	0.7438	0.388756	0.0159	0.0078927984
ss61475989	*rs41579354*	10	20.112404	0.0738	1.6277	0.202446	0.0457	0.0062422591
ss86300803		10	20.141623	0.2299	0.6666	0.414519	0.0179	0.0063322392
ss86335602		10	20.166462	0.2851	0.6453	0.422088	0.0176	0.0071586186
ss61469993	*rs41565990*	10	20.200257	0.8009	2.0385	0.153814	0.0332	0.0105832160
	rs29009655	10	20.238386	0.3016	0.1169	0.732566	0.0070	0.0029610928
ss61521347	*rs41618564*	10	20.371003	0.1082	1.0565	0.304378	0.0297	0.0057207702
ss86330842		10	20.396978	0.2342	2.3978	0.121964	0.0336	0.0120513984
ss86341159		10	20.434438	0.0509	0.5753	0.448415	0.0303	0.0029268184
ss86309555		10	20.455714	0.3431	0.3417	0.559034	0.0113	0.0051028393
ss86309716		10	20.47784	0.9133	2.4096	0.121046	0.0517	0.0081871667
ss61521346	*rs41618563*	10	20.498489	0.2951	0.0005	0.981365	0.0005	0.0001962741
ss86320415		10	20.56499	0.9277	0.5732	0.449246	0.0271	0.0036314148
ss117969878		10	20.593697	0.2887	7.2631	0.007208	0.0550	0.0225866809
ss61521352	*rs43744410*	10	20.677218	0.5179	2.2056	0.137959	0.0267	0.0133277126
ss86309445		10	20.770558	0.6569	3.3029	0.069586	0.0360	0.0162074293
ss86340208		10	20.807369	0.5817	0.2777	0.598381	0.0101	0.0048958800
ss86303611		10	20.836263	0.2500	2.3716	0.124017	0.0331	0.0124037409
ss86328453		10	20.909334	0.6877	0.4751	0.490885	0.0138	0.0059280305
	rs29013509	10	20.944649	0.2607	0.1638	0.685821	0.0087	0.0033550981
ss86321943		10	20.997142	0.4341	1.6547	0.198746	0.0243	0.0119255335
ss86286444		10	21.026548	0.0444	0.0007	0.979538	0.0012	0.0000976504
ss105236652		10	21.056606	0.2708	0.5227	0.469918	0.0156	0.0061767621
ss86292991		10	21.083528	0.2958	0.4215	0.516400	0.0135	0.0056222700
ss86307068		10	21.114073	0.1476	0.0291	0.864556	0.0043	0.0010940200
ss86336668		10	21.139315	0.1519	0.0099	0.920847	0.0025	0.0006483333
ss86336968		10	21.167023	0.5745	5.7939	0.016341	0.0450	0.0220182570
ss117964112		10	21.208729	0.2930	1.0642	0.302609	0.0217	0.0089875363
ss86297966		10	21.266129	0.0938	1.7277	0.189144	0.0415	0.0070560575
ss86287681		10	21.398344	0.8138	3.4824	0.062446	0.0446	0.0135155814
ss86282226		10	21.43156	0.8173	3.2385	0.072361	0.0433	0.0129419891
ss61548761	*rs41644777*	10	21.464225	0.1927	0.2085	0.648066	0.0107	0.0033441962
ss86296420		10	21.509012	0.3338	5.9607	0.014876	0.0488	0.0217165894
ss86295569		10	21.560952	0.3689	0.8455	0.358134	0.0182	0.0084685223
ss86291655		10	21.587515	0.4291	0.8371	0.360537	0.0179	0.0087640732
ss86296554		10	21.626577	0.4993	0.2303	0.631429	0.0087	0.0043721579
ss86317906		10	21.655458	0.4183	0.0305	0.861351	0.0032	0.0015658894
ss105236668		10	21.682679	0.7894	2.6376	0.104811	0.0363	0.0120633587
ss86332869		10	21.73187	0.4140	1.1258	0.289051	0.0202	0.0097817967
ss86336139		10	21.786092	0.9205	9.3647	0.002297	0.1028	0.0150520489
ss86340454		10	21.808419	0.0638	9.5425	0.002088	0.1139	0.0135935144
ss61487353	*rs41589718*	10	21.969646	0.7092	1.1886	0.275986	0.0232	0.0095895089
ss86299255		10	22.024691	0.6433	1.2923	0.256017	0.0224	0.0102609697
ss61487355	*rs41589720*	10	22.152445	0.7249	0.5063	0.476978	0.0153	0.0060891413

ss61487356	rs41589721	10	22.223864	0.3639	4.0638	0.044195	0.0404	0.0187014615
ss61549263	rs41647560	10	22.248293	0.3639	4.0638	0.044195	0.0404	0.0187014615
ss86315402		10	22.270654	0.7758	0.9091	0.340674	0.0218	0.0075988879
ss86321497		10	22.326415	0.5172	8.6731	0.003337	0.0560	0.0279629357
ss86294769		10	22.452207	0.6024	1.3627	0.243465	0.0230	0.0109953122
ss86332997		10	22.487111	0.1182	0.2208	0.638595	0.0136	0.0028336226
ss86330513		10	22.515209	0.6877	1.4422	0.230187	0.0240	0.0103206962
ss86289568		10	22.611753	0.8295	1.8021	0.179893	0.0332	0.0093993078
ss105237380		10	22.642189	0.1827	2.0025	0.157484	0.0336	0.0100284789
ss86334142		10	22.672607	0.4441	0.1754	0.675502	0.0081	0.0039888234
ss86302387		10	22.716645	0.7113	13.3903	0.000272	0.0744	0.0305465772
ss61563426	rs41660899	10	22.767135	0.2529	2.7450	0.098012	0.0366	0.0138135831
ss86336108		10	22.831437	0.6153	0.0379	0.845701	0.0037	0.0017338999
ss86285043		10	23.177003	0.7851	2.4547	0.117631	0.0356	0.0120176118
		10	23.813257	0.8746	0.2916	0.589345	0.0146	0.0031909933
	rs29027640	10	24.912901	0.3431	1.0485	0.306213	0.0204	0.0091926029
ss65073075	rs43165704	10	24.937217	0.8596	0.1202	0.728947	0.0089	0.0021497729
ss46526902	rs41257350	10	25.070336	0.4842	0.6662	0.414647	0.0153	0.0076579852
	rs29026524	10	25.201004	0.4585	0.0696	0.792003	0.0049	0.0024346053
ss86286578		10	25.221614	0.6275	1.1903	0.275638	0.0213	0.0099749748
ss61509718	rs41606764	10	25.26776	0.1211	0.0084	0.927060	0.0026	0.0005544233
ss86284971		10	25.310025	0.5688	0.3034	0.581917	0.0103	0.0050610748
ss86326698		10	25.43212	0.7400	0.3316	0.564913	0.0124	0.0047566046
ss86338189		10	25.457293	0.7400	0.3316	0.564913	0.0124	0.0047566046
ss86330751		10	25.501824	0.2701	0.3705	0.542919	0.0128	0.0050300839
ss86304640		10	25.53369	0.7966	0.2534	0.614831	0.0115	0.0037361171
ss46526975	rs41257421	10	25.659591	0.0766	1.7789	0.182725	0.0443	0.0062732204
ss86341425		10	25.696039	0.6232	1.5048	0.220352	0.0238	0.0111779308
ss86307081		10	25.776447	0.5888	2.2183	0.136837	0.0277	0.0134282587
ss61487576	rs41584743	10	25.801391	0.8968	3.5941	0.058398	0.0566	0.0104728402
ss86340344		10	25.8495	0.5774	0.8599	0.354087	0.0176	0.0086131378
ss105236680		10	25.89702	0.1261	0.3478	0.555533	0.0162	0.0035719111
ss86334577		10	25.923637	0.6719	4.8534	0.027918	0.0430	0.0189740101
ss64661852	rs42757293	10	25.954757	0.6698	4.4888	0.034472	0.0408	0.0180662832
	rs29023280	10	25.981428	0.2837	0.2572	0.612218	0.0101	0.0040986897
ss86325417		10	26.082814	0.2120	0.7582	0.384187	0.0201	0.0067186427
ss117969986		10	26.121822	0.3381	1.8159	0.178239	0.0259	0.0115750290
ss61549806	rs41644703	10	26.182493	0.1039	0.0266	0.870472	0.0050	0.0009347896
ss65537478	rs43622040	10	26.208772	0.5824	13.4030	0.000270	0.0692	0.0336594105
ss61549808	rs41644705	10	26.243651	0.7486	3.8195	0.051058	0.0414	0.0155904127
ss105237359		10	26.33931	0.6003	0.4774	0.489830	0.0133	0.0063646162
ss61507240	rs41604638	10	26.371397	0.5616	0.1135	0.736306	0.0062	0.0030431126
	rs29022126	10	26.404116	0.4885	0.0560	0.812926	0.0043	0.0021490070
ss86336767		10	26.463688	0.4563	0.1997	0.655082	0.0084	0.0041675169
ss86309636		10	26.48911	0.3016	0.4838	0.486929	0.0139	0.0058453335
ss117969973		10	26.509327	0.5287	0.0279	0.867358	0.0031	0.0015460775
ss105237376		10	26.589861	0.5057	0.0167	0.897063	0.0024	0.0011794790

ss64202708	*rs42527460*	10	26.624133	0.5559	0.1077	0.742866	0.0060	0.0029500057
ss117964106		10	26.661443	0.5609	0.1249	0.723886	0.0064	0.0031626304
ss86321914		10	26.727801	0.7629	1.5534	0.213056	0.0258	0.0093298546
ss61466761	*rs41572303*	10	26.852878	0.9062	0.4086	0.522884	0.0205	0.0034915538
ss86325117		10	26.890931	0.9420	5.7131	0.017104	0.0894	0.0097772896
ss61473767	*rs41574083*	10	26.951989	0.5351	0.0090	0.924528	0.0018	0.0008901709
ss65055831	*rs43142162*	10	26.981431	0.5423	0.7045	0.401556	0.0157	0.0077890001
ss65541170	*rs43619962*	10	27.028538	0.7643	0.9547	0.328869	0.0206	0.0074186777
ss65541053	*rs43619145*	10	27.057544	0.8123	0.0746	0.784772	0.0063	0.0019210371
ss65541020	*rs43618612*	10	27.215403	0.2249	0.0134	0.907877	0.0025	0.0008863219
ss65043442	*rs43130859*	10	27.249812	0.2063	0.0000	0.998503	0.0000	0.0000138810
ss65540778	*rs43626569*	10	27.34497	0.0673	2.8907	0.089539	0.0619	0.0077767924
ss63028387	*rs42079558*	10	27.38567	0.3252	0.1851	0.667143	0.0086	0.0037835072
ss63028279	*rs42078604*	10	27.431248	0.0824	0.3014	0.583173	0.0179	0.0026996348
ss86311123		10	27.459807	0.2063	1.1318	0.287755	0.0232	0.0075969694
ss63028365	*rs42079547*	10	27.506558	0.0681	2.9377	0.086976	0.0622	0.0078849231
ss65540764	*rs43626555*	10	27.602363	0.3345	0.1654	0.684369	0.0080	0.0035802110
ss65540756	*rs43626547*	10	27.627008	0.3266	0.0807	0.776494	0.0057	0.0024976550
ss65540719	*rs43625710*	10	27.657735	0.0681	2.6845	0.101779	0.0594	0.0075382268
ss64983299	*rs43075230*	10	27.686039	0.9319	2.6845	0.101779	0.0594	0.0075382268
ss65540671	*rs43625662*	10	27.740531	0.0681	3.1319	0.077211	0.0648	0.0082163459
ss86304882		10	27.790082	0.9427	2.0344	0.154223	0.0574	0.0062031012
ss65537759	*rs43624117*	10	27.874182	0.1934	0.1969	0.657347	0.0101	0.0031652749
ss65537783	*rs43624741*	10	27.912275	0.7206	0.4675	0.494346	0.0137	0.0055239567
ss65537825	*rs43624783*	10	27.946899	0.8725	4.3245	0.037932	0.0602	0.0133858324
ss65537899	*rs43616259*	10	27.979476	0.5394	2.4644	0.116905	0.0292	0.0145199250
ss86286651		10	28.020376	0.3546	0.1466	0.701890	0.0073	0.0033211385
	rs29009928	10	28.09605	0.7600	0.9667	0.325835	0.0215	0.0078604593
	rs29018189	10	28.118487	0.8968	0.0268	0.869965	0.0051	0.0009503910
ss65538483	*rs43621939*	10	28.152262	0.5566	0.1368	0.711595	0.0069	0.0034283041
ss65538560	*rs43622016*	10	28.174655	0.5372	0.1496	0.699066	0.0071	0.0035395730
ss61528238	*rs41631274*	10	28.221489	0.8023	0.9547	0.328864	0.0225	0.0071237670
ss86319021		10	28.251644	0.4613	0.6568	0.417980	0.0154	0.0076352767
ss61549840	*rs41645636*	10	28.307863	0.5967	11.4116	0.000771	0.0634	0.0305196962
ss86338409		10	28.359601	0.8940	0.0002	0.988082	0.0004	0.0000818303
ss86291638		10	28.400483	0.1769	0.1371	0.711328	0.0089	0.0025821334
ss86323847		10	28.44001	0.9334	4.9101	0.027023	0.0815	0.0101379149
ss61568808	*rs41666691*	10	28.466244	0.5294	0.6897	0.406544	0.0153	0.0076422332
ss65538860	*rs43624714*	10	28.498095	0.6418	1.5210	0.217877	0.0229	0.0105250510
ss105237392		10	28.518913	0.2744	0.7156	0.397897	0.0175	0.0069623027
ss86305679		10	28.559824	0.5544	0.1593	0.689969	0.0074	0.0036596553
ss65538938	*rs43617264*	10	28.601211	0.0437	1.4363	0.231141	0.0523	0.0043749080
ss61549844	*rs41645640*	10	28.629903	0.9026	2.9445	0.086615	0.0526	0.0092543619
ss61487582	*rs41584748*	10	28.694001	0.7156	0.6772	0.410849	0.0166	0.0067756771
ss64740840	*rs42833917*	10	28.833332	0.7006	0.2545	0.614091	0.0101	0.0042474382
ss86315633		10	28.870432	0.9491	0.8958	0.344247	0.0400	0.0038659786
ss64283282	*rs42563984*	10	28.892597	0.3195	0.6086	0.435570	0.0156	0.0067650346

ss86336593		10	28.995793	0.4248	0.0269	0.869768		0.0031	0.0014954369
ss86333345		10	29.035026	0.9312	0.9786	0.322897		0.0360	0.0046169775
ss61550373	*rs41650152*	10	29.140912	0.2643	1.4418	0.230254		0.0253	0.0098277440
ss61521806	*rs41621905*	10	29.177263	0.1375	0.0380	0.845499		0.0052	0.0012335917
ss63921126	*rs42230224*	10	29.247747	0.3632	0.9634	0.326678		0.0187	0.0086464866
ss117969838		10	29.27554	0.8123	0.1216	0.727359		0.0083	0.0025410563
ss86318366		10	29.325017	0.1397	0.3049	0.580999		0.0146	0.0035208899
ss86296114		10	29.353259	0.4241	0.0614	0.804359		0.0046	0.0022354045
ss61487905	*rs41586768*	10	29.454917	0.2249	0.0958	0.757046		0.0068	0.0023648648
ss86321935		10	29.64976	0.2865	0.4921	0.483248		0.0142	0.0058112544
ss86322344		10	29.755802	0.6519	3.5398	0.060328		0.0360	0.0163618606
ss86285223		10	29.858011	0.4764	1.0770	0.299731		0.0191	0.0095506546
ss61477364		10	30.188333	0.8009	0.0741	0.785566		0.0063	0.0019989600
ss61528308	*rs41624356*	10	30.271093	0.5738	1.0283	0.310902		0.0189	0.0092338134
ss61529943	*rs41630444*	10	30.298678	0.5566	1.9622	0.161721		0.0255	0.0126030934
ss61550350	*rs41649230*	10	30.339487	0.1662	7.8672	0.005174		0.0699	0.0193654798
ss65540056	*rs43619578*	10	30.480052	0.1612	0.0408	0.840029		0.0051	0.0013697769
ss65540090	*rs43619612*	10	30.527426	0.2364	7.3967	0.006697		0.0592	0.0213705278
ss65540092	*rs43619614*	10	30.562309	0.8489	2.3297	0.127380		0.0405	0.0104027991
ss65540175	*rs43620672*	10	30.720068	0.1984	4.1614	0.041733		0.0501	0.0159245517
ss64853118	*rs42948789*	10	30.745396	0.7185	0.9114	0.340079		0.0197	0.0079866296
ss65540188	*rs43620685*	10	30.834674	0.3066	0.3194	0.572128		0.0115	0.0048893058
ss65540252	*rs43621849*	10	30.873951	0.1991	3.9922	0.046101		0.0462	0.0147367198
ss65540276	*rs43621873*	10	30.902786	0.5014	1.0296	0.310608		0.0187	0.0093272737
ss64739183	*rs42838413*	10	30.990261	0.2915	11.2400	0.000844		0.0708	0.0292283623
ss61524250	*rs41619206*	10	31.01223	0.0917	2.1936	0.139037		0.0468	0.0078010495
ss61564274	*rs41660145*	10	31.051133	0.3718	3.3016	0.069641		0.0337	0.0157312168
ss61550349	*rs41649229*	10	31.203575	0.2450	4.1494	0.042028		0.0431	0.0159584424
ss64205912	*rs42523663*	10	31.240589	0.4527	3.3714	0.066765		0.0342	0.0169526891
ss61564272	*rs41660143*	10	31.273187	0.7880	0.1986	0.655968		0.0099	0.0033047875
ss62546245	*rs41912995*	10	31.316503	0.4771	0.1922	0.661212		0.0081	0.0040526484
ss65540012	*rs43619534*	10	31.372924	0.8037	1.8728	0.171595		0.0326	0.0102926610
ss86301473		10	31.398052	0.4821	0.3164	0.573961		0.0103	0.0051231013
ss65540030	*rs43619552*	10	31.420947	0.0817	0.0023	0.962076		0.0016	0.0002386777
	rs29019500	10	31.457483	0.8976	8.4855	0.003695		0.0881	0.0162042842
ss61473660	*rs41573076*	10	31.494283	0.3797	0.5958	0.440440		0.0151	0.0070970666
ss117969920		10	31.589547	0.7135	0.3316	0.564933		0.0117	0.0047723253
ss65539046	*rs43617772*	10	31.807351	0.2679	2.6658	0.102976		0.0336	0.0131756244
ss61549846	*rs41645642*	10	31.845931	0.2049	1.7303	0.188801		0.0299	0.0097569408
ss86322387		10	31.917589	0.3711	0.2968	0.586074		0.0110	0.0051257548
ss65539258	*rs43619084*	10	31.948428	0.8446	0.6516	0.419807		0.0206	0.0054177945
ss65539280	*rs43619106*	10	31.973422	0.3918	0.1664	0.683486		0.0082	0.0038978748
ss65539296	*rs43619122*	10	31.994369	0.8818	2.7864	0.095519		0.0467	0.0097430178
ss117964151		10	32.078349	0.8532	0.9700	0.325033		0.0261	0.0065282619
ss61487586		10	32.29404	0.6060	3.2996	0.069726		0.0339	0.0161810193
ss65539713	*rs43621738*	10	32.359101	0.6218	10.1509	0.001507		0.0622	0.0292716304
ss86291263		10	32.387737	0.1562	0.3751	0.540421		0.0155	0.0040942585

ss117964113		10	32.413055	0.0874	3.5706	0.059226		0.0592	0.0094484618
ss61550345	*rs41649225*	10	32.433589	0.7801	3.4386	0.064111		0.0396	0.0135840853
ss61487898	*rs41586761*	10	32.453851	0.7564	3.3259	0.068627		0.0386	0.0142216574
ss61550348	*rs41649228*	10	32.544761	0.3596	0.0010	0.974187		0.0006	0.0002844463
ss64696252	*rs42793494*	10	32.612944	0.0630	0.0029	0.956993		0.0021	0.0002496575
ss86321040		10	32.633723	0.8818	1.4042	0.236434		0.0337	0.0070187334
ss61516707	*rs41618008*	10	32.788634	0.9205	0.5434	0.461273		0.0257	0.0037588514
ss61472118	*rs41567658*	10	32.999078	0.4183	4.1689	0.041550		0.0389	0.0189240954
ss86291770		10	33.04051	0.9542	2.0333	0.154337		0.0640	0.0056005765
ss61532146	*rs41633266*	10	33.075958	0.6132	3.4871	0.062271		0.0353	0.0167318589
	rs29013224	10	33.312367	0.3632	0.0179	0.893684		0.0026	0.0012005568
ss86341618		10	33.353076	0.7830	1.1136	0.291678		0.0234	0.0079536663
ss86340032		10	33.391447	0.3123	5.4090	0.020320		0.0455	0.0195642530
	rs29011308	10	33.429682	0.2020	0.8797	0.348605		0.0218	0.0070409755
ss86273438		10	33.492966	0.4556	3.9806	0.046416		0.0379	0.0187929028
ss61551496	*rs41650970*	10	33.531528	0.0946	0.5013	0.479167		0.0229	0.0039216452
ss86337670		10	33.563694	0.5186	0.2479	0.618727		0.0093	0.0046486776
ss61522985	*rs41623453*	10	33.649825	0.7564	3.5423	0.060238		0.0421	0.0154992836
ss61550950	*rs41647633*	10	33.694607	0.2887	2.4255	0.119831		0.0325	0.0133332243
ss65541335	*rs43620826*	10	33.735732	0.1067	7.7781	0.005433		0.0827	0.0157779892
ss61503407	*rs41606909*	10	33.786899	0.0645	4.4011	0.036276		0.0782	0.0094341854
ss61522983	*rs41623451*	10	33.842892	0.5824	1.8803	0.170742		0.0256	0.0124576985
ss86336757		10	33.966279	0.1655	1.3077	0.253211		0.0283	0.0078118796
ss86285925		10	34.033741	0.3381	1.4479	0.229267		0.0232	0.0103724246
ss86320128		10	34.088197	0.6232	2.2590	0.133292		0.0285	0.0133684398
ss86333144		10	34.109563	0.9685	0.8471	0.357679		0.0450	0.0027454023
ss86338740		10	34.214397	0.5437	10.0630	0.001579		0.0578	0.0286827587
	rs29015924	10	34.285267	0.0967	0.1027	0.748760		0.0103	0.0017959517
ss86281070		10	34.319595	0.6741	1.9473	0.163327		0.0271	0.0119015002
ss86273923		10	34.387178	0.3266	1.7694	0.183897		0.0259	0.0113989345
ss117969829		10	34.700167	0.1834	0.0439	0.834136		0.0048	0.0014485207
ss61510045	*rs41601192*	10	34.852032	0.7464	5.4495	0.019858		0.0487	0.0184388776
ss105294632		10	34.889814	0.1855	0.0171	0.895986		0.0031	0.0009222834
ss61488956	*rs41588497*	10	34.913744	0.5129	0.1463	0.702177		0.0071	0.0035369510
ss86290437		10	34.965552	0.2758	5.8567	0.015773		0.0494	0.0197151799
	rs29017491	10	35.014359	0.0638	0.6160	0.432814		0.0298	0.0035606669
ss61551980		10	35.049077	0.7543	0.0175	0.894887		0.0028	0.0010391395
	rs29016462	10	35.069086	0.0451	1.1410	0.285811		0.0475	0.0040948480
ss61488442	*rs41584995*	10	35.27474	0.2292	0.2146	0.643297		0.0100	0.0035408421
ss61509925	*rs41600274*	10	35.547266	0.8460	0.3906	0.532177		0.0155	0.0040318102
ss86313193		10	35.61501	0.3095	0.0033	0.953991		0.0011	0.0004741252
ss86308762		10	35.690158	0.2765	1.6133	0.204451		0.0256	0.0102506075
ss86327942		10	35.821814	0.3037	4.3584	0.037191		0.0415	0.0175415932
ss117969996		10	35.862279	0.6383	0.0375	0.846459		0.0037	0.0017118332
ss61522986	*rs41623454*	10	35.904354	0.3338	7.6713	0.005760		0.0540	0.0240318332
	rs29013243	10	35.928855	0.8811	2.9976	0.083830		0.0502	0.0105233408
ss86341453		10	35.950875	0.8238	0.4637	0.496139		0.0166	0.0048061344

ss117969830		10	36.002649	0.2593	5.4180	0.020216	0.0488	0.0187520301
ss86329592		10	36.051859	0.8295	0.0275	0.868352	0.0040	0.0011283384
ss61551968	*rs43707538*	10	36.172833	0.5000	3.4201	0.064830	0.0341	0.0170596449
ss86290079		10	36.218304	0.4312	4.2017	0.040757	0.0376	0.0184498203
ss86322104		10	36.264772	0.9212	1.6416	0.200527	0.0439	0.0063692635
ss86289527		10	36.285827	0.8883	0.0060	0.938150	0.0023	0.0004467291
ss86330850		10	36.371676	0.3682	1.5048	0.220352	0.0234	0.0108938115
ss86277599		10	36.425618	0.6963	0.0884	0.766335	0.0059	0.0025142957
ss86324334		10	36.464718	0.3854	1.0552	0.304674	0.0197	0.0093460684
	rs29017586	10	36.557398	0.4950	0.0145	0.904224	0.0022	0.0011013748
ss61551947	*rs43707017*	10	36.601977	0.8897	3.7233	0.054064	0.0591	0.0115922486
ss86285705		10	36.630015	0.1683	1.1849	0.276746	0.0258	0.0072293181
ss86273985		10	36.740462	0.3911	1.0312	0.310241	0.0196	0.0093240993
ss61488444	*rs41584997*	10	36.772362	0.0874	0.4233	0.515525	0.0213	0.0033926102
ss86318575		10	36.816524	0.3918	1.3219	0.250647	0.0221	0.0105390597
ss86300755		10	36.924888	0.5738	0.4529	0.501205	0.0123	0.0059955343
ss86324201		10	37.010008	0.5408	0.1113	0.738721	0.0061	0.0030154354
ss86314372		10	37.080763	0.8811	0.0024	0.960715	0.0014	0.0002976095
ss61488445	*rs41584998*	10	37.352406	0.5358	3.0191	0.082731	0.0324	0.0161016792
ss86325506		10	37.390013	0.6526	0.9915	0.319724	0.0193	0.0087700941
ss86337874		10	37.452335	0.3460	0.0014	0.970583	0.0007	0.0003230064
ss117964135		10	37.50574	0.4427	1.8922	0.169400	0.0252	0.0124494405
ss61509791	*rs41607431*	10	37.589441	0.5072	5.6307	0.017920	0.0430	0.0215144400
ss86303590		10	37.625931	0.3818	3.5345	0.060521	0.0351	0.0165811538
ss61549834	*rs41644730*	10	37.647412	0.5380	0.0275	0.868288	0.0030	0.0015015148
ss105238098		10	37.6754	0.9692	1.6120	0.204636	0.0684	0.0040833310
ss86303435		10	37.782884	0.8811	3.8607	0.049826	0.0573	0.0120134257
ss38323372	*rs29012285*	10	37.80458	0.8825	3.7821	0.052207	0.0570	0.0118121261
ss86319575		10	37.835626	0.6404	7.7460	0.005529	0.0541	0.0249396229
ss61477637	*rs41578566*	10	37.884711	0.3288	5.5941	0.018294	0.0466	0.0205814379
ss86321377		10	37.932901	0.6404	7.7460	0.005529	0.0541	0.0249396229
ss86317226		10	37.977006	0.3553	7.4042	0.006670	0.0531	0.0243256777
ss61503522	*rs41608115*	10	38.157709	0.8675	2.2611	0.133116	0.0399	0.0091794110
	rs29010222	10	38.216576	0.0494	3.6931	0.055047	0.0821	0.0077164747
ss117970008		10	38.26464	0.0544	2.2633	0.132923	0.0609	0.0062705839
ss86330500		10	38.292369	0.9663	8.2617	0.004172	0.1446	0.0094082218
ss86340397		10	38.363541	0.4499	0.1172	0.732237	0.0063	0.0031356097
	rs29022040	10	38.463822	0.4470	0.5307	0.466564	0.0135	0.0066571076
ss61499817	*rs41595401*	10	38.625718	0.6862	0.0063	0.936832	0.0016	0.0006755147
ss61499827	*rs41595410*	10	38.698787	0.5122	1.4871	0.223084	0.0227	0.0113422447
	rs29013631	10	38.725565	0.4391	0.0856	0.769931	0.0055	0.0027157303
	rs29018232	10	38.75509	0.1182	0.7452	0.388292	0.0248	0.0051618691
ss61525991	*rs41623989*	10	38.78329	0.7779	0.2983	0.585134	0.0122	0.0042265985
ss63974658	*rs42413754*	10	38.806658	0.6404	1.3815	0.240255	0.0233	0.0107490530
ss105238783		10	38.836921	0.6189	1.4965	0.221617	0.0231	0.0108784290
ss86287376		10	38.857217	0.7070	3.2020	0.073981	0.0365	0.0151236181
ss63975232	*rs42415241*	10	38.88862	0.3696	0.0027	0.958872	0.0010	0.0004721764

ss105238769		10	38.937048	0.5372	0.1063	0.744492	0.0059	0.0029097811
ss63975791	*rs42411422*	10	39.248823	0.2837	2.7982	0.094818	0.0344	0.0139784242
ss61476307	*rs41575568*	10	39.373018	0.6383	0.1261	0.722666	0.0068	0.0031623843
ss61541511	*rs41645043*	10	39.461269	0.7428	9.4896	0.002148	0.0647	0.0247102074
ss65217673	*rs43306682*	10	39.50585	0.5021	1.1669	0.280404	0.0204	0.0102075031
ss64828080	*rs42920582*	10	39.540553	0.9477	7.0548	0.008087	0.1089	0.0107973688
ss65542290	*rs43622181*	10	39.639943	0.5874	3.0717	0.080105	0.0332	0.0160766378
ss61488699	*rs41586944*	10	39.780856	0.9011	0.4467	0.504141	0.0206	0.0036694139
ss61488698	*rs41586943*	10	39.819292	0.7364	2.2858	0.131021	0.0310	0.0120372616
ss86338779		10	39.881051	0.1032	3.8033	0.051553	0.0562	0.0104055750
ss65542157	*rs43621348*	10	39.953528	0.5659	3.3429	0.067923	0.0342	0.0168161595
	rs29023056	10	39.996113	0.7830	0.9950	0.318878	0.0220	0.0074940853
ss65542136	*rs43620627*	10	40.016226	0.2163	1.2169	0.270348	0.0246	0.0083537775
ss65542131	*rs43620622*	10	40.042453	0.4341	3.0225	0.082558	0.0324	0.0159002591
ss86340556		10	40.125897	0.2206	1.2483	0.264259	0.0240	0.0082702768
ss86331699		10	40.200595	0.8388	0.9805	0.322415	0.0244	0.0065916107
ss105238789		10	40.240611	0.1762	4.0767	0.043861	0.0492	0.0142980506
ss105238790		10	40.262341	0.0516	0.6133	0.433829	0.0335	0.0032743523
ss61514420	*rs41611793*	10	40.284242	0.1132	5.6343	0.017882	0.0669	0.0134386766
ss86298742		10	40.316725	0.2020	0.0001	0.993623	0.0002	0.0000586523
ss117969804		10	40.338477	0.1991	0.0090	0.924514	0.0022	0.0006949705
ss61520382	*rs41613309*	10	40.392218	0.6211	12.3382	0.000472	0.0675	0.0317636972
ss105238792		10	40.426574	0.4076	10.1511	0.001507	0.0610	0.0294507238
ss65542074	*rs43620565*	10	40.463162	0.5408	17.3884	0.000034	0.0770	0.0382529836
	rs29011689	10	40.499266	0.5666	12.5897	0.000414	0.0661	0.0324764428
ss65541888	*rs43625379*	10	40.519327	0.8001	1.2075	0.272200	0.0253	0.0080900044
ss117969894		10	40.540809	0.7550	0.1529	0.695914	0.0084	0.0031019877
ss86301760		10	40.626545	0.3868	9.3813	0.002277	0.0581	0.0275412704
ss86341558		10	40.701091	0.3181	0.6555	0.418413	0.0154	0.0066642389
ss61524214		10	40.769608	0.5423	3.6732	0.055702	0.0356	0.0176706766
ss86332187		10	40.851625	0.3883	9.2977	0.002381	0.0577	0.0274206467
ss61511942	*rs41609656*	10	41.029995	0.2815	1.5869	0.208196	0.0254	0.0102772284
ss61568800	*rs41666683*	10	41.051429	0.6039	4.0812	0.043746	0.0384	0.0183824446
ss64985903	*rs43073314*	10	41.217233	0.8381	2.3681	0.124295	0.0384	0.0104250729
ss64717948	*rs42817178*	10	41.245304	0.8653	2.9119	0.088372	0.0463	0.0107802734
ss86284132		10	41.267334	0.6354	2.7205	0.099521	0.0309	0.0143109897
ss64199050	*rs42525044*	10	41.302784	0.1354	3.8657	0.049680	0.0532	0.0124525728
ss86302213		10	41.340062	0.4298	0.4039	0.525311	0.0120	0.0058584149
ss64199134	*rs42525085*	10	41.373532	0.8868	2.0534	0.152313	0.0414	0.0083019428
	rs29018736	10	41.456993	0.3532	1.2967	0.255210	0.0213	0.0097237470
ss86318227		10	41.502842	0.1433	3.4078	0.065312	0.0470	0.0115280491
ss86309033		10	41.553558	0.8489	2.3703	0.124118	0.0381	0.0097775196
ss61568370	*rs41663668*	10	41.595091	0.3410	0.0049	0.944202	0.0013	0.0006055194
ss61488962	*rs41588503*	10	41.672043	0.5294	1.1009	0.294435	0.0196	0.0097543037
ss61552457	*rs43710811*	10	41.694452	0.6905	1.6731	0.196276	0.0254	0.0108622653
ss117969807		10	41.731531	0.5308	0.3037	0.581756	0.0099	0.0049287952
ss61488960	*rs41588501*	10	41.789863	0.8173	0.1574	0.691654	0.0095	0.0028297427

ss86332196		10	41.94767	0.1991	0.4569	0.499312	0.0150	0.0047833487
ss117970009		10	41.99495	0.5394	3.4111	0.065185	0.0331	0.0164459637
ss86333453		10	42.030319	0.5931	5.1511	0.023537	0.0409	0.0197522977
ss86315156		10	42.061982	0.6125	4.1088	0.043042	0.0373	0.0177014825
ss105289409		10	42.083541	0.8295	0.1963	0.657836	0.0112	0.0031546884
ss61515632	*rs41615984*	10	42.111842	0.6511	2.5865	0.108230	0.0301	0.0136770366
ss61521371		10	42.175126	0.4721	3.3255	0.068641	0.0328	0.0163508977
ss63901104	*rs42018935*	10	42.22148	0.0688	1.0358	0.309153	0.0371	0.0047555670
ss63900916	*rs42018441*	10	42.242862	0.9312	1.0358	0.309153	0.0371	0.0047555670
ss86313162		10	42.288346	0.4943	4.3509	0.037354	0.0366	0.0182791036
ss86298019		10	42.315342	0.5057	4.3509	0.037354	0.0366	0.0182791036
ss63900068	*rs41969117*	10	42.455281	0.1110	2.1964	0.138786	0.0433	0.0085497294
ss61521370	*rs41618585*	10	42.530889	0.4914	3.8521	0.050081	0.0357	0.0178660657
ss86296748		10	42.562596	0.5172	3.3460	0.067795	0.0332	0.0165806503
ss64002870	*rs42425539*	10	42.701387	0.3947	6.9674	0.008487	0.0476	0.0227511751
ss61488975	*rs41588516*	10	42.779052	0.6067	7.0638	0.008046	0.0479	0.0228419741
ss61527124	*rs41629682*	10	42.860319	0.2729	1.4505	0.228859	0.0242	0.0096094936
ss86340475		10	42.892585	0.4885	0.1245	0.724321	0.0065	0.0032278841
ss61506780	*rs41608275*	10	42.979583	0.2650	0.0001	0.992182	0.0002	0.0000804109
ss86327410		10	43.043376	0.2951	0.7605	0.383473	0.0173	0.0072117609
	rs29012019	10	43.279114	0.2586	7.3229	0.006975	0.0561	0.0215184423
ss61552459	*rs43710813*	10	43.335574	0.2779	0.8435	0.358707	0.0185	0.0074214477
	rs29010446	10	43.390123	0.8825	2.6083	0.106760	0.0464	0.0096244269
ss117970002		10	43.736139	0.6734	2.2239	0.136343	0.0291	0.0127965969
ss86301124		10	44.047785	0.4441	0.1743	0.676482	0.0077	0.0037919765
ss64223628	*rs42532333*	10	44.071503	0.8689	0.6438	0.422614	0.0225	0.0051220176
	rs29012840	10	44.093124	0.3818	0.2010	0.654040	0.0085	0.0040080152
ss64902235	*rs42997179*	10	44.185609	0.5523	0.1892	0.663730	0.0080	0.0039564310
ss86339686		10	44.219427	0.5115	0.0135	0.907449	0.0021	0.0010728450
ss64041284	*rs42443894*	10	44.244662	0.8689	0.6438	0.422614	0.0225	0.0051220176
ss117969999		10	44.361986	0.7751	2.6517	0.103889	0.0348	0.0121473146
ss64040952	*rs42442128*	10	44.396498	0.9112	2.2515	0.133942	0.0478	0.0077441855
ss61970167	*rs41713070*	10	44.436577	0.7307	2.4383	0.118859	0.0325	0.0127822651
ss61523107	*rs41624073*	10	44.497953	0.6769	1.2118	0.271348	0.0219	0.0095870129
ss61528302	*rs41624350*	10	44.529546	0.8259	0.0076	0.930671	0.0021	0.0006001945
ss86288082		10	44.592369	0.3259	0.0170	0.896451	0.0026	0.0011391575
ss63168580	*rs42138402*	10	44.631287	0.6848	0.0156	0.900573	0.0025	0.0010776921
ss61552480	*rs43710934*	10	44.666034	0.4964	2.6042	0.107037	0.0301	0.0150652732
	rs29026819	10	44.71102	0.5989	0.2239	0.636210	0.0089	0.0042661027
ss86341568		10	44.946212	0.6203	0.2840	0.594288	0.0102	0.0047821656
ss61472289	*rs41568927*	10	45.022993	0.5372	0.4193	0.517523	0.0120	0.0059669512
ss86328627		10	45.062592	0.3804	0.2140	0.643775	0.0086	0.0040591747
ss86305973		10	45.087919	0.6375	0.3739	0.541102	0.0111	0.0051356665
ss86320679		10	45.110714	0.0580	2.1337	0.144548	0.0569	0.0062230784
ss86307479		10	45.137348	0.3338	9.3906	0.002265	0.0574	0.0255150103
ss61552470	*rs43710824*	10	45.158535	0.3546	0.0127	0.910341	0.0022	0.0009892735
ss105289853		10	45.189043	0.1347	0.0217	0.882926	0.0040	0.0009380672

ss65543436	*rs43625528*	10	45.210845	0.4327	0.5209	0.470690		0.0135	0.0066089610
ss61552469	*rs43710823*	10	45.2315	0.8496	0.0022	0.962184		0.0012	0.0003145288
ss61488967	*rs41588508*	10	45.270368	0.6418	0.0720	0.788538		0.0052	0.0023757921
ss61488966	*rs41588507*	10	45.310517	0.4327	0.7349	0.391583		0.0158	0.0077569037
ss105239475		10	45.359878	0.5673	0.7349	0.391583		0.0158	0.0077569037
ss61552492	*rs43710946*	10	45.395006	0.4341	0.8623	0.353410		0.0170	0.0083760648
ss65543207	*rs43623799*	10	45.524238	0.2479	0.2089	0.647745		0.0096	0.0035949370
ss65543189	*rs43623781*	10	45.553973	0.1060	0.5113	0.474812		0.0209	0.0039556906
ss86309954		10	45.585411	0.1103	0.5587	0.455055		0.0215	0.0042293613
ss86340439		10	45.605587	0.1067	0.5859	0.444281		0.0220	0.0041883625
	rs17871435	10	45.640747	0.6354	0.0002	0.987784		0.0003	0.0001345337
	rs29027593	10	45.670483	0.1898	0.3991	0.527753		0.0149	0.0045724545
ss86341207		10	45.698672	0.2464	2.5427	0.111262		0.0344	0.0127835099
ss117969823		10	45.747642	0.7536	2.5427	0.111262		0.0344	0.0127835099
ss65544362	*rs43625852*	10	45.776469	0.4527	0.4204	0.516952		0.0121	0.0059714511
ss86306758		10	45.799476	0.5473	0.4204	0.516952		0.0121	0.0059714511
ss65544287	*rs43625577*	10	45.821547	0.5387	0.3189	0.572454		0.0105	0.0052189427
ss86337466		10	45.845882	0.5380	0.2960	0.586599		0.0101	0.0050348192
ss61552484	*rs43710938*	10	45.87825	0.5387	0.3189	0.572454		0.0105	0.0052189427
ss64907671	*rs42998148*	10	46.001271	0.5938	6.6890	0.009903		0.0480	0.0231663978
ss46526487	*rs41256950*	10	46.013388	0.5981	5.7463	0.016786		0.0447	0.0214729736
ss65543894	*rs43630286*	10	46.040797	0.6927	14.4649	0.000155		0.0743	0.0316349993
ss65543824	*rs43629416*	10	46.069471	0.6941	15.8692	0.000075		0.0779	0.0330987972
ss61488978	*rs41588519*	10	46.137556	0.3675	11.7079	0.000659		0.0643	0.0298804132
ss86292370		10	46.244873	0.8152	4.6830	0.030802		0.0511	0.0154099580
ss86339765		10	46.307933	0.8446	0.6634	0.415656		0.0212	0.0055640238
ss86341363		10	46.339718	0.2335	6.7999	0.009312		0.0542	0.0194085538
ss86279620		10	46.370704	0.5258	0.0281	0.866975		0.0030	0.0014847643
ss86300772		10	46.394175	0.9656	2.2563	0.133526		0.0752	0.0049948344
ss117964100		10	46.415088	0.3574	4.3217	0.037995		0.0387	0.0177623946
ss61567499	*rs41663208*	10	46.436973	0.2815	3.4656	0.063079		0.0381	0.0154143177
ss86312266		10	46.628034	0.2013	1.6604	0.197978		0.0294	0.0094449501
ss86278380		10	46.671243	0.0652	0.9161	0.338820		0.0361	0.0043962461
ss86333974		10	46.733169	0.8947	1.3991	0.237275		0.0362	0.0068205348
ss86332489		10	46.769448	0.1232	1.5843	0.208564		0.0347	0.0074981685
ss86278068		10	46.791557	0.1218	1.6542	0.198816		0.0358	0.0076613963
ss86290459		10	46.854374	0.8087	0.1629	0.686643		0.0093	0.0028698595
ss86283965		10	46.881315	0.8517	0.5171	0.472337		0.0186	0.0046937867
ss86291544		10	46.921672	0.5458	0.2736	0.601124		0.0091	0.0045213200
ss86306662		10	46.950342	0.8324	0.8160	0.366667		0.0221	0.0061641397
ss64743871	*rs42838037*	10	46.978825	0.1662	8.0091	0.004789		0.0681	0.0188822904
ss86319611		10	47.014001	0.2113	0.8737	0.350259		0.0211	0.0070352911
ss86330762		10	47.054697	0.3968	3.5055	0.061583		0.0350	0.0167383419
	rs29025964	10	47.086408	0.1103	0.0037	0.951508		0.0018	0.0003501507
ss86330619		10	47.150297	0.3095	7.7992	0.005370		0.0557	0.0237900941
ss86300592		10	47.283344	0.5723	2.7524	0.097559		0.0309	0.0151238916
ss117964116		10	47.349638	0.4835	10.7825	0.001076		0.0617	0.0308037615

ss65545022	*rs43625309*	10	47.404438	0.5451	6.1767	0.013178	0.0433	0.0214597543
ss86337560		10	47.426958	0.5458	1.1664	0.280521	0.0193	0.0095740912
ss86336211		10	47.500126	0.2457	3.5809	0.058862	0.0397	0.0147187039
ss86335725		10	47.523092	0.5537	3.5541	0.059816	0.0343	0.0169350576
ss86296203		10	47.637385	0.5788	5.1693	0.023293	0.0422	0.0205842201
ss86315892		10	47.669994	0.5501	1.6415	0.200551	0.0229	0.0113585224
ss86337756		10	47.702322	0.5795	4.6425	0.031531	0.0389	0.0189377364
ss86307513		10	47.73448	0.6196	3.0194	0.082717	0.0322	0.0151696122
ss86336540		10	47.837203	0.5208	5.4486	0.019868	0.0413	0.0206244220
ss86331960		10	47.879183	0.2486	4.6747	0.030951	0.0438	0.0163644960
ss86314594		10	47.932267	0.2679	6.6283	0.010242	0.0536	0.0210433562
ss86341136		10	47.965966	0.5924	3.0782	0.079790	0.0320	0.0154657303
ss86335791		10	47.995194	0.0996	1.2250	0.268756	0.0343	0.0061475305
ss65546086	*rs43625971*	10	48.020417	0.4857	0.0660	0.797367	0.0048	0.0023999677
ss86334665		10	48.052955	0.4477	0.0000	0.999892	0.0000	0.0000012620
ss65545964	*rs43625049*	10	48.079825	0.8603	3.9534	0.047168	0.0523	0.0125685624
ss86336937		10	48.111506	0.3617	0.8996	0.343217	0.0181	0.0083465276
ss86302892		10	48.135081	0.0745	4.3351	0.037700	0.0741	0.0102242708
ss86318586		10	48.161338	0.7908	4.1685	0.041559	0.0468	0.0154934163
ss86330792		10	48.18331	0.0466	0.2867	0.592495	0.0235	0.0020879957
ss117970039		10	48.243965	0.8603	2.0937	0.148360	0.0378	0.0090859207
ss61553029	*rs43708682*	10	48.430288	0.3997	2.9469	0.086489	0.0320	0.0153366286
ss61553027	*rs43708680*	10	48.54696	0.6970	0.0651	0.798690	0.0051	0.0021435177
ss86293469		10	48.712186	0.4219	0.6374	0.424908	0.0154	0.0075176943
	rs29015700	10	48.742687	0.2034	7.9939	0.004828	0.0656	0.0212528323
ss105237423		10	48.791887	0.1526	1.8532	0.173858	0.0357	0.0092342466
ss86340716		10	48.819304	0.3589	1.1074	0.293023	0.0208	0.0095526859
ss86340464		10	48.858275	0.1633	4.1082	0.043056	0.0500	0.0136759162
ss61553022	*rs43708675*	10	48.920614	0.7414	3.3080	0.069375	0.0375	0.0143978162
ss61553016	*rs43708669*	10	48.942782	0.1511	6.0842	0.013880	0.0621	0.0159330519
ss86338317		10	48.964251	0.7586	0.7563	0.384787	0.0190	0.0069619441
ss61553007	*rs43708660*	10	49.03185	0.7213	3.9585	0.047026	0.0404	0.0162296421
ss86316383		10	49.141301	0.8918	0.2400	0.624375	0.0149	0.0028696560
ss105237425		10	49.231331	0.4448	9.0425	0.002733	0.0527	0.0260423363
ss105237426		10	49.317357	0.1089	0.5290	0.467265	0.0217	0.0042116601
ss61553613	*rs43714159*	10	49.362294	0.2958	0.5173	0.472223	0.0147	0.0061050406
ss86337573		10	49.40112	0.4391	2.5127	0.113386	0.0297	0.0146317727
ss61554039	*rs41649786*	10	49.436407	0.5781	1.5221	0.217715	0.0225	0.0109902491
ss61468825	*rs41573135*	10	49.469368	0.5652	0.0569	0.811600	0.0042	0.0020793957
ss65549247	*rs43627914*	10	49.493817	0.7314	2.7518	0.097596	0.0327	0.0128675180
ss65549196	*rs43627863*	10	49.543894	0.1325	0.1782	0.673021	0.0118	0.0027113512
ss117970052		10	49.567212	0.3317	0.9073	0.341166	0.0183	0.0081291627
ss61489779	*rs41586897*	10	49.641164	0.2113	10.6343	0.001164	0.0728	0.0242638556
ss61554055	*rs41649802*	10	49.715652	0.7486	5.5327	0.018942	0.0486	0.0182790331
ss61510233	*rs41602676*	10	49.757097	0.3295	3.1374	0.076955	0.0345	0.0152577447
ss86337608		10	49.802294	0.4592	1.6030	0.205904	0.0231	0.0114878890
ss86326909		10	49.824458	0.8653	0.0020	0.964432	0.0012	0.0002799182

	rs29026478	10	49.890209	0.3703	12.4125	0.000454	0.0687	0.0320398118
ss61510232	*rs41586899*	10	49.91533	0.6655	1.0794	0.299189	0.0207	0.0092160635
ss65503466	*rs43587755*	10	49.946229	0.4219	0.0777	0.780484	0.0052	0.0025400505
ss105237435		10	49.977938	0.3352	0.2065	0.649645	0.0088	0.0039427378
ss61554049	*rs41649796*	10	50.070696	0.0910	3.7314	0.053804	0.0639	0.0105658286
ss65549913	*rs43633259*	10	50.112937	0.1318	0.0503	0.822679	0.0059	0.0013568760
ss86316637		10	50.21088	0.2830	0.2854	0.593327	0.0109	0.0044081472
ss65550027	*rs43627777*	10	50.244575	0.2178	1.7313	0.188680	0.0293	0.0099760695
ss86317782		10	50.273405	0.4857	0.8271	0.363438	0.0167	0.0083312273
ss86300677		10	50.297274	0.3703	0.6450	0.422196	0.0156	0.0072753959
ss86311095		10	50.333808	0.6454	0.3662	0.545289	0.0116	0.0053268642
ss65550327	*rs43630077*	10	50.435867	0.1526	0.2436	0.621803	0.0128	0.0032992187
ss65550426	*rs43630776*	10	50.534788	0.1741	1.3891	0.238954	0.0291	0.0083544104
ss65550361	*rs43630111*	10	50.562941	0.5229	0.2067	0.649480	0.0084	0.0042050247
	rs29027193	10	50.600524	0.4112	3.4465	0.063807	0.0345	0.0167129956
ss86314251		10	50.629309	0.3854	0.0358	0.849917	0.0037	0.0017328309
ss65550584	*rs43632233*	10	50.697852	0.4921	0.6816	0.409302	0.0155	0.0077423688
ss61489776	*rs41586894*	10	50.731718	0.3539	1.2090	0.271904	0.0217	0.0099266935
ss61517831	*rs41619094*	10	50.789974	0.4893	0.0457	0.830846	0.0040	0.0019984083
ss61527474	*rs41624135*	10	50.804749	0.4520	1.2258	0.268609	0.0208	0.0103227849
ss61566266	*rs41662308*	10	50.896763	0.3352	0.3031	0.582103	0.0107	0.0047874798
ss86282001		10	50.929847	0.3388	0.2640	0.607564	0.0099	0.0044287042
ss86291232		10	50.950117	0.1999	3.5853	0.058708	0.0445	0.0142328946
ss64265750	*rs42557533*	10	51.020252	0.3431	0.4790	0.489094	0.0135	0.0060683130
ss61566267	*rs41662309*	10	51.073231	0.2364	2.3731	0.123899	0.0330	0.0119008741
ss64265584	*rs42556851*	10	51.0971	0.0895	0.4578	0.498888	0.0215	0.0035096654
	rs29012389	10	51.138335	0.2966	0.2245	0.635807	0.0096	0.0039963522
ss86308078		10	51.226055	0.8266	4.7971	0.028839	0.0543	0.0155596526
ss61471897	*rs41573837*	10	51.334752	0.8539	0.2143	0.643561	0.0122	0.0030326193
ss86337972		10	51.411472	0.7178	0.5376	0.463663	0.0155	0.0062757880
ss86275240		10	51.494336	0.0623	6.5607	0.010635	0.0953	0.0111413597
ss65551292	*rs43632440*	10	51.530234	0.1784	1.1061	0.293292	0.0262	0.0076656373
ss65551224	*rs43631172*	10	51.596604	0.6777	0.8565	0.355040	0.0180	0.0078678936
ss86323014		10	51.62757	0.4327	0.2179	0.640821	0.0088	0.0042976169
ss86333388		10	51.64986	0.3073	0.0939	0.759317	0.0061	0.0025809057
ss86327592		10	51.693427	0.1282	0.3798	0.537887	0.0164	0.0036679525
ss65550923	*rs43634467*	10	51.718534	0.8682	0.1816	0.670131	0.0113	0.0025786072
ss86330401		10	51.739671	0.7830	3.0022	0.083595	0.0386	0.0131344760
ss86284178		10	51.76288	0.5308	0.1312	0.717327	0.0067	0.0033399002
ss86298176		10	51.803868	0.3274	0.2443	0.621289	0.0094	0.0041206358
ss86330668		10	51.843123	0.7658	0.0090	0.924620	0.0021	0.0007481467
ss63207562	*rs42148819*	10	51.898573	0.7736	0.0610	0.804976	0.0056	0.0019601315
ss61494909	*rs41591496*	10	51.925799	0.3832	0.0074	0.931368	0.0019	0.0008925831
ss65549744	*rs43631592*	10	51.963015	0.8324	4.3169	0.038101	0.0515	0.0143602154
ss105238105		10	52.019521	0.4635	0.0759	0.783073	0.0050	0.0024953928
ss65546959	*rs43625144*	10	52.088335	0.5530	1.0540	0.304939	0.0188	0.0093005814
ss61553572	*rs43713018*	10	52.11907	0.8639	2.3520	0.125579	0.0409	0.0096167934

ss65547002	*rs43625187*	10	52.141276	0.3381	0.0342	0.853290	0.0036	0.0016020859
ss61525227	*rs41619260*	10	52.176936	0.2242	0.0201	0.887326	0.0031	0.0010782325
	rs29019899	10	52.197726	0.3532	0.0021	0.963554	0.0009	0.0004061348
ss65547239	*rs43627019*	10	52.237474	0.2199	7.0602	0.008062	0.0591	0.0202633365
ss65547224	*rs43627004*	10	52.298327	0.2751	3.1940	0.074344	0.0372	0.0148545527
ss86313448		10	52.322403	0.5466	0.2873	0.592151	0.0098	0.0048486912
ss86297509		10	52.345301	0.3854	0.0056	0.940556	0.0014	0.0006665561
ss86321431		10	52.387321	0.5509	0.3876	0.533782	0.0118	0.0058196264
ss86285663		10	52.473527	0.2636	6.8309	0.009153	0.0558	0.0216619314
ss61468782	*rs41572092*	10	52.505035	0.0645	0.0098	0.921226	0.0038	0.0004627380
ss86338945		10	52.526873	0.8080	3.1579	0.075998	0.0420	0.0130420552
ss61489531	*rs41585854*	10	52.57959	0.7514	3.7148	0.054337	0.0415	0.0154874943
ss65000265	*rs43087086*	10	52.660587	0.7658	1.6300	0.202129	0.0277	0.0099289087
ss61553576	*rs43713022*	10	52.684233	0.1440	0.0014	0.970133	0.0010	0.0002429431
ss65547514	*rs43629990*	10	52.714675	0.2156	4.9865	0.025863	0.0500	0.0169271956
ss65547535	*rs43630011*	10	52.737514	0.7450	11.1513	0.000885	0.0705	0.0267983366
ss61553578	*rs43713024*	10	52.94449	0.6734	0.4628	0.496546	0.0138	0.0060549514
ss86341021		10	52.966014	0.6734	0.4310	0.511729	0.0133	0.0058430481
ss117969833		10	53.007998	0.4097	7.3378	0.006918	0.0494	0.0239186534
ss65547987	*rs43626465*	10	53.031692	0.5050	0.4063	0.524041	0.0123	0.0061396896
ss65548003	*rs43626481*	10	53.07923	0.8037	4.5199	0.033854	0.0494	0.0155985084
ss86312176		10	53.2538	0.2579	0.0813	0.775589	0.0061	0.0023258277
ss86307808		10	53.286203	0.3904	1.2502	0.263893	0.0210	0.0099973716
ss86299120		10	53.307504	0.4076	2.7512	0.097632	0.0309	0.0149253872
ss65548403	*rs43630376*	10	53.33127	0.5638	0.1509	0.697766	0.0073	0.0035961033
	rs29019736	10	53.355085	0.3968	2.8200	0.093546	0.0319	0.0152621349
ss86294941		10	53.382466	0.6526	0.1787	0.672648	0.0083	0.0037651115
ss61489543	*rs41585866*	10	53.404872	0.0895	4.5890	0.032524	0.0675	0.0109982977
ss61553590	*rs43714136*	10	53.427058	0.7887	1.4630	0.226868	0.0276	0.0091871169
ss86340119		10	53.469469	0.2378	1.1750	0.278741	0.0238	0.0086293947
ss86340078		10	53.512385	0.4821	3.0842	0.079495	0.0318	0.0158878616
ss61553599	*rs43714145*	10	53.551455	0.4026	0.2364	0.626970	0.0092	0.0044445921
ss65548880	*rs43634750*	10	53.640116	0.9026	0.0058	0.939299	0.0024	0.0004248227
ss61553600	*rs43714146*	10	53.697156	0.2486	2.3678	0.124318	0.0332	0.0124053168
	rs29017141	10	53.747496	0.4628	0.7950	0.372898	0.0165	0.0082112483
ss61465880	*rs41565146*	10	53.918005	0.9169	2.4912	0.114942	0.0535	0.0081485277
ss86315046		10	54.012682	0.3746	1.4602	0.227314	0.0228	0.0106613908
ss65549000	*rs43626374*	10	54.041553	0.9062	0.0277	0.867799	0.0053	0.0009097887
ss63370191	*rs42201733*	10	54.074583	0.0974	0.0058	0.939299	0.0024	0.0004248227
ss117969891		10	54.131185	0.2128	0.4083	0.523059	0.0141	0.0047253710
ss61532727	*rs41626818*	10	54.157166	0.2077	1.1518	0.283553	0.0245	0.0080656842
ss61532725	*rs41626816*	10	54.178436	0.0974	0.0058	0.939299	0.0024	0.0004248227
ss86291767		10	54.208387	0.9126	8.3969	0.003877	0.0925	0.0147538157
	rs29026853	10	54.285303	0.2765	2.1917	0.139211	0.0301	0.0120503770
ss63629202	*rs42300153*	10	54.385812	0.3983	3.1020	0.078633	0.0328	0.0157067102
ss63629244	*rs42300167*	10	54.442449	0.8789	0.8932	0.344934	0.0271	0.0057660736
ss117969857		10	54.517057	0.2500	1.2361	0.266601	0.0245	0.0091936579

ss61511319	*rs41611838*	10	54.632431	0.3553	2.9003	0.089011		0.0328	0.0150371262
ss61475189	*rs41572676*	10	54.676852	0.8539	0.0139	0.906275		0.0030	0.0007544170
ss61563347	*rs41660322*	10	54.714669	0.6404	2.6758	0.102333		0.0309	0.0142473902
ss63629595	*rs42291384*	10	54.740373	0.2607	1.7351	0.188200		0.0271	0.0104318303
ss63629514	*rs42291357*	10	54.760455	0.6519	2.8507	0.091783		0.0321	0.0145656771
ss61496468	*rs41598717*	10	54.810138	0.6626	2.2090	0.137662		0.0281	0.0125651024
ss63290746	*rs42177028*	10	54.860472	0.3116	0.0089	0.924965		0.0019	0.0008044458
ss65552898	*rs43635143*	10	54.907155	0.3116	0.0089	0.924965		0.0019	0.0008044458
ss65552869	*rs43634714*	10	54.955034	0.7314	0.2632	0.608074		0.0107	0.0042003816
ss86293829		10	55.038545	0.5079	0.4999	0.479795		0.0131	0.0065367862
ss105238132		10	55.090697	0.2958	2.1804	0.140226		0.0304	0.0126454360
ss86324814		10	55.188735	0.4019	0.0271	0.869395		0.0031	0.0015058284
ss86310763		10	55.23483	0.6805	1.1411	0.285789		0.0207	0.0090012415
ss86293470		10	55.263005	0.1304	1.0517	0.305476		0.0278	0.0063112131
	rs29026593	10	55.286328	0.5358	0.3939	0.530448		0.0120	0.0059508785
ss65552428	*rs43632773*	10	55.306768	0.7579	0.4754	0.490727		0.0148	0.0054254022
ss61490079	*rs41589590*	10	55.331778	0.2092	7.7253	0.005592		0.0628	0.0207720263
ss86331422		10	55.384413	0.8840	5.2248	0.022567		0.0667	0.0136812993
ss105238134		10	55.436073	0.6562	1.2398	0.265895		0.0217	0.0097742228
ss86323690		10	55.46776	0.3438	1.2398	0.265895		0.0217	0.0097742228
ss105238136		10	55.521784	0.2615	0.8226	0.364746		0.0187	0.0072052239
	rs29015976	10	55.55309	0.2636	0.9044	0.341937		0.0196	0.0075958529
ss61503807	*rs41599798*	10	55.573829	0.7851	0.2014	0.653766		0.0102	0.0034380176
ss61468878	*rs41573188*	10	55.618424	0.2278	1.3498	0.245707		0.0249	0.0087466518
ss61503806	*rs41599797*	10	55.700823	0.0924	0.1266	0.722042		0.0111	0.0018655504
ss61554574	*rs41653016*	10	55.743359	0.6791	0.4045	0.524973		0.0124	0.0053889524
ss65552199	*rs43631446*	10	55.833997	0.8080	3.0101	0.083189		0.0400	0.0124188490
ss63481340	*rs42245263*	10	55.909279	0.8345	4.1200	0.042758		0.0507	0.0140099330
ss86338887		10	55.935088	0.9183	0.3710	0.542642		0.0199	0.0029796226
ss65554259	*rs43634899*	10	56.169008	0.8123	0.6388	0.424433		0.0195	0.0059338354
ss65554350	*rs43635289*	10	56.224025	0.6605	0.2469	0.619391		0.0097	0.0043468103
ss65551388	*rs43633836*	10	56.318826	0.7593	5.5086	0.019203		0.0506	0.0185045301
ss86282140		10	56.402882	0.2464	6.0440	0.014196		0.0526	0.0195255021
ss65553936	*rs43640275*	10	56.428419	0.7564	6.0830	0.013889		0.0531	0.0195824846
	rs29012019	10	56.497455	0.2586	7.3229	0.006975		0.0561	0.0215184423
	rs29014555	10	56.525119	0.8446	1.7004	0.192664		0.0349	0.0091765520
ss65551831	*rs43637574*	10	56.557412	0.1547	2.3141	0.128664		0.0410	0.0107341172
ss61494417	*rs41596717*	10	56.586774	0.1390	15.4526	0.000093		0.1026	0.0245585316
ss61494418	*rs41596718*	10	56.624332	0.8539	13.1818	0.000303		0.0933	0.0232795922
ss65553616	*rs43637158*	10	56.655967	0.3037	4.2538	0.039533		0.0419	0.0177068322
ss61490086	*rs41589596*	10	56.680721	0.6812	5.2817	0.021846		0.0458	0.0198758016
ss38325785	*rs29011490*	10	56.807631	0.2085	1.8969	0.168872		0.0312	0.0102976150
ss38337307	*rs29026620*	10	56.831532	0.5337	3.0515	0.081104		0.0328	0.0163103902
ss65553353	*rs43634395*	10	56.894493	0.3861	0.1028	0.748575		0.0062	0.0029313577
ss86338653		10	56.917088	0.7958	2.4646	0.116888		0.0353	0.0114690432
ss65553206	*rs43633149*	10	56.973047	0.2421	0.6959	0.404446		0.0177	0.0065056018
ss65553167	*rs43631810*	10	56.994966	0.2228	1.4376	0.230928		0.0262	0.0090752733

ss86317463		10	57.018384	0.2199	1.6492	0.199500	0.0283	0.0097129461
ss64927582	rs43021288	10	57.042296	0.7830	2.1296	0.144928	0.0322	0.0109426362
ss86289356		10	57.244623	0.5623	0.0884	0.766373	0.0054	0.0026770979
ss86320846		10	57.303644	0.4484	0.1627	0.686772	0.0073	0.0036089798
ss64908062	rs43000038	10	57.377256	0.7830	0.0078	0.929754	0.0020	0.0006668837
ss63751325	rs42332515	10	57.52722	0.5458	0.5936	0.441299	0.0145	0.0071834960
ss86339827		10	57.596318	0.8037	3.1870	0.074660	0.0417	0.0131554687
ss61471562	rs41571019	10	57.638141	0.1648	2.0932	0.148407	0.0361	0.0099274914
ss61514777	rs41616336	10	57.688532	0.8739	2.2863	0.130973	0.0414	0.0091256255
ss63750074	rs42337103	10	57.724527	0.8481	2.0315	0.154519	0.0368	0.0094676068
ss61506208	rs41603426	10	57.744636	0.1984	3.7682	0.052641	0.0445	0.0141409126
ss86296891		10	58.021767	0.4040	0.0067	0.934550	0.0016	0.0007560282
ss65458195	rs43537839	10	58.087291	0.8883	1.6009	0.206192	0.0372	0.0073819287
ss61551861	rs43706931	10	58.149676	0.8632	1.2907	0.256318	0.0308	0.0072780518
ss86327536		10	58.563915	0.1232	2.6733	0.102495	0.0462	0.0099768699
ss105240924		10	58.655494	0.3968	8.1941	0.004329	0.0541	0.0259043801
ss86332802		10	58.677957	0.1468	3.1891	0.074568	0.0478	0.0119861227
ss63593106	rs42286918	10	58.708937	0.7880	0.2349	0.628042	0.0108	0.0036198923
ss63593079	rs42286909	10	58.745598	0.7672	1.0758	0.300008	0.0227	0.0081155354
ss61565437	rs41660791	10	58.83048	0.4090	1.9809	0.159739	0.0269	0.0129864442
ss63592974	rs42286874	10	58.876561	0.6476	0.0002	0.989612	0.0003	0.0001192327
ss61511345	rs41611863	10	58.909914	0.4484	0.2160	0.642270	0.0086	0.0042362591
ss61562343	rs41660444	10	58.935577	0.6526	1.7160	0.190638	0.0248	0.0112243091
ss86275512		10	58.992412	0.3675	1.8462	0.174671	0.0258	0.0119947527
ss61565439	rs41660793	10	59.107639	0.7371	8.7018	0.003286	0.0606	0.0234734005
ss63592257	rs42285242	10	59.129308	0.0845	3.0955	0.078949	0.0570	0.0088233194
	rs29013533	10	59.150259	0.0759	2.8961	0.089240	0.0572	0.0080336926
ss86300214		10	59.20305	0.2221	0.0601	0.806474	0.0054	0.0018827724
ss61511481	rs41612795	10	59.257162	0.0953	4.8309	0.028283	0.0685	0.0118025566
ss63592644	rs42286164	10	59.302139	0.9226	2.7134	0.099959	0.0551	0.0078599224
ss61555135	rs41651671	10	59.501552	0.9542	1.1727	0.279219	0.0486	0.0042550617
ss61555129	rs41651665	10	59.55146	0.0466	1.0910	0.296613	0.0466	0.0041366772
ss86310247		10	59.58085	0.2715	4.0081	0.045671	0.0429	0.0169612529
ss86284520		10	59.606891	0.7443	7.1164	0.007816	0.0566	0.0215645622
	rs29019795	10	59.669115	0.1891	0.0480	0.826645	0.0050	0.0015294269
ss65554493	rs43636232	10	59.69701	0.3109	0.3574	0.550154	0.0122	0.0052168564
ss61554615	rs41653656	10	59.769258	0.9613	0.3827	0.536383	0.0289	0.0021523895
ss61519681	rs41616925	10	59.934509	0.6519	0.6165	0.432618	0.0153	0.0069353561
ss61519679	rs41616923	10	59.959777	0.8711	1.5202	0.218007	0.0347	0.0077916862
ss117970033		10	60.001421	0.6777	0.1323	0.716141	0.0073	0.0031692644
ss86286301		10	60.223488	0.0946	0.2397	0.624550	0.0156	0.0026643740
ss86322312		10	60.375485	0.1963	6.2288	0.012799	0.0574	0.0180994490
ss61497582	rs41597808	10	60.412249	0.1404	0.0117	0.913899	0.0029	0.0006943221
ss64448980	rs42643313	10	60.438299	0.1583	0.0741	0.785584	0.0068	0.0018008107
ss86288300		10	60.463661	0.1913	6.3613	0.011886	0.0584	0.0180660345
ss64667093	rs42766718	10	60.493252	0.9148	2.6799	0.102077	0.0529	0.0082517043
ss64667063	rs42766688	10	60.520414	0.6526	1.6046	0.205676	0.0252	0.0114112934

ss86338717		10	60.582941	0.0824	1.6691	0.196807	0.0408	0.0061747225
ss86315269		10	60.612526	0.0716	3.3786	0.066476	0.0681	0.0090548264
ss86320281		10	60.777884	0.2987	0.0684	0.793807	0.0052	0.0021836402
ss64121551	*rs42488393*	10	60.832738	0.6289	0.4034	0.525538	0.0120	0.0056098607
ss64121531	*rs42488383*	10	60.860434	0.5552	0.2309	0.630988	0.0091	0.0045120932
ss64121465	*rs42488350*	10	60.885444	0.4083	1.0037	0.316758	0.0190	0.0091652537
ss86317641		10	60.949189	0.7758	0.0029	0.956830	0.0012	0.0004051644
ss61507887	*rs41599877*	10	60.978566	0.4269	2.3619	0.124785	0.0285	0.0139329202
ss64121277	*rs42487356*	10	61.029596	0.0308	0.9184	0.338229	0.0515	0.0030773067
ss64121227	*rs42487331*	10	61.066582	0.9692	0.9184	0.338229	0.0515	0.0030773067
ss61552267	*rs43709224*	10	61.165187	0.9692	0.9184	0.338229	0.0515	0.0030773067
	rs29027143	10	61.188043	0.5380	0.9930	0.319347	0.0185	0.0091907669
ss61494213	*rs41594419*	10	61.288405	0.1812	1.5400	0.215037	0.0301	0.0089320336
ss61563517	*rs41661889*	10	61.308735	0.6433	0.9604	0.327428	0.0197	0.0090299606
ss61555171	*rs41651707*	10	61.379612	0.8603	0.2006	0.654361	0.0118	0.0028377298
ss61490367	*rs41591576*	10	61.401896	0.1497	3.0199	0.082690	0.0449	0.0114434171
ss61517357	*rs41614846*	10	61.464191	0.4277	1.3470	0.246209	0.0209	0.0102368206
ss64854711	*rs42947777*	10	61.595218	0.5967	1.3239	0.250297	0.0210	0.0101139378
	rs29024208	10	61.648016	0.1762	0.1187	0.730574	0.0085	0.0024637521
ss86331546		10	61.67046	0.8001	5.2546	0.022187	0.0529	0.0169146266
ss61512299	*rs41610907*	10	61.82742	0.6913	0.0447	0.832545	0.0043	0.0018399227
	rs29025856	10	62.171363	0.8976	0.2854	0.593345	0.0164	0.0030138208
ss86334210		10	62.263317	0.1719	1.2039	0.272923	0.0264	0.0075060203
ss61563882	*rs41665047*	10	62.410139	0.3632	0.9925	0.319470	0.0190	0.0087890061
ss61494432	*rs41597632*	10	62.466742	0.7457	0.3303	0.565694	0.0124	0.0046861128
ss117969904		10	62.487184	0.5709	3.2242	0.072990	0.0337	0.0165293584
ss86274915		10	62.511021	0.2837	0.0304	0.861733	0.0035	0.0014302631
ss86306734		10	62.538581	0.4792	11.5003	0.000735	0.0616	0.0307382224
ss86302647		10	62.637827	0.5201	11.1889	0.000867	0.0607	0.0302829353
ss86330877		10	62.662238	0.3696	2.5495	0.110782	0.0310	0.0144347974
ss86283266		10	62.71252	0.1877	0.4667	0.494745	0.0161	0.0049085184
ss61465778	*rs41569022*	10	62.734148	0.9384	10.6990	0.001125	0.1221	0.0141202064
ss86280194		10	62.898446	0.8381	2.6509	0.103941	0.0408	0.0110597712
ss86341460		10	63.023942	0.5838	3.1742	0.075244	0.0319	0.0155008611
ss86311676		10	63.139836	0.5595	0.9821	0.322015	0.0187	0.0092023458
ss61555181	*rs41651716*	10	63.170779	0.3539	6.3303	0.012093	0.0500	0.0228650933
ss61555180	*rs41651715*	10	63.201599	0.8209	3.3298	0.068463	0.0434	0.0127668114
ss65555397	*rs43638123*	10	63.224428	0.3209	0.0596	0.807125	0.0048	0.0020703849
ss86325023		10	63.340486	0.2128	0.2234	0.636645	0.0107	0.0035784032
ss65555499	*rs43639317*	10	63.448237	0.1655	3.3890	0.066056	0.0457	0.0126091679
ss65555557	*rs43640575*	10	63.492538	0.4642	1.9517	0.162848	0.0254	0.0126130057
ss61490371	*rs41591578*	10	63.536821	0.3281	0.1481	0.700448	0.0076	0.0033399697
	rs29024832	10	63.707097	0.0852	1.7554	0.185640	0.0425	0.0066317155
ss86337344		10	63.762591	0.6096	0.2416	0.623206	0.0094	0.0044972363
ss86282924		10	63.792319	0.1755	0.3209	0.571242	0.0132	0.0038130608
ss86297843		10	63.815048	0.6583	1.5103	0.219507	0.0241	0.0108557419
ss86278607		10	63.85648	0.1433	1.2870	0.256985	0.0296	0.0072555709

ss61490611	*rs41589116*	10	63.917114	0.2192	1.9446	0.163612	0.0309	0.0105601784
ss86310921		10	64.041482	0.1791	0.4609	0.497412	0.0165	0.0048653618
ss65556092	*rs43635411*	10	64.107325	0.1798	0.1386	0.709839	0.0088	0.0026067615
ss61555666	*rs41656664*	10	64.128838	0.7428	0.5359	0.464373	0.0153	0.0058581377
ss86331121		10	64.248735	0.3854	0.3470	0.556013	0.0109	0.0051487592
ss86305439		10	64.278716	0.2493	0.4936	0.482569	0.0146	0.0054827570
ss86329174		10	64.458211	0.1605	1.6357	0.201346	0.0321	0.0086528903
ss86325608		10	64.483387	0.2951	1.4050	0.236293	0.0243	0.0100998373
	rs29021167	10	64.551775	0.3668	6.1201	0.013603	0.0488	0.0226657543
ss86318193		10	64.62306	0.5759	0.5273	0.467997	0.0136	0.0066253585
ss61490622	*rs41589127*	10	64.7983	0.7521	1.8619	0.172849	0.0287	0.0107129775
ss61510416	*rs41604544*	10	64.861568	0.4842	0.2636	0.607794	0.0099	0.0049612076
ss61490624		10	65.047747	0.7414	0.9722	0.324472	0.0213	0.0081759682
ss105240907		10	65.090977	0.8216	0.1334	0.715017	0.0088	0.0025785865
ss61555696	*rs41656694*	10	65.159485	0.4420	0.8793	0.348707	0.0177	0.0087179990
ss61518588	*rs41616440*	10	65.295695	0.3546	0.0263	0.871336	0.0031	0.0014270461
ss86316712		10	65.360878	0.8632	0.0040	0.949315	0.0017	0.0004007354
ss61510417	*rs41604545*	10	65.575428	0.5516	0.0372	0.847115	0.0035	0.0017544057
	rs29016349	10	65.664709	0.1168	0.0009	0.975802	0.0009	0.0001811229
ss61568342	*rs41663640*	10	65.73584	0.8532	0.0375	0.846531	0.0050	0.0012640766
ss105240223		10	65.757089	0.8610	2.7786	0.095982	0.0432	0.0103396271
ss105292076		10	65.821338	0.8360	0.1849	0.667367	0.0106	0.0029137571
ss86324885		10	65.849013	0.5079	0.6432	0.422841	0.0148	0.0073962566
ss117969902		10	65.916304	0.1440	0.3368	0.561898	0.0151	0.0037255806
ss86300630		10	65.952065	0.8868	0.6438	0.422618	0.0235	0.0047204574
ss61473413	*rs41570631*	10	66.006068	0.8968	0.1938	0.659928	0.0133	0.0024586449
ss86339022		10	66.129485	0.8324	0.1271	0.721536	0.0088	0.0024644348
ss86302728		10	66.240212	0.5788	0.6962	0.404351	0.0156	0.0076227611
ss86299869		10	66.284779	0.7264	4.7820	0.029091	0.0443	0.0176281786
ss86290239		10	66.371497	0.1060	0.5092	0.475712	0.0206	0.0039026810
ss86293242		10	66.436003	0.1175	3.0153	0.082927	0.0491	0.0101724673
ss105240927		10	66.498375	0.1426	0.0312	0.859837	0.0047	0.0011472933
ss86299608		10	66.531017	0.6934	0.8673	0.352030	0.0182	0.0077549414
ss86334695		10	66.603261	0.2436	1.5316	0.216293	0.0264	0.0097269692
ss61506637	*rs41607942*	10	66.638386	0.7672	1.0537	0.305002	0.0221	0.0078992811
ss86283275		10	66.801007	0.1354	0.2861	0.592906	0.0140	0.0032751036
ss61510419	*rs41604547*	10	66.861714	0.8539	0.3577	0.549984	0.0152	0.0037872532
ss86309151		10	67.275736	0.1870	0.1301	0.718398	0.0085	0.0025700124
ss86300609		10	67.368839	0.7944	0.9932	0.319318	0.0230	0.0075240580
ss105293410		10	67.419427	0.4026	0.1031	0.748252	0.0061	0.0029538006
	rs29009945	10	67.485299	0.5838	0.1719	0.678516	0.0079	0.0038555415
ss86296197		10	67.569886	0.4606	1.5457	0.214184	0.0236	0.0117030583
ss86294863		10	67.625517	0.2436	4.0523	0.044495	0.0444	0.0163591805
ss105240939		10	67.793097	0.5602	0.2359	0.627304	0.0091	0.0044882127
	rs29010337	10	68.029462	0.5903	4.7273	0.030023	0.0400	0.0193382099
ss61512408	*rs41611714*	10	68.108177	0.2034	0.0007	0.979472	0.0006	0.0001861232
ss61470287	*rs41568676*	10	68.128346	0.4907	0.2833	0.594685	0.0102	0.0051090012

ss117969932		10	68.156898	0.2808	0.0240	0.876861	0.0033	0.0013206346
ss86275180		10	68.18565	0.1862	2.1339	0.144528	0.0359	0.0108674569
ss105241614		10	68.206921	0.6834	9.0605	0.002706	0.0590	0.0255263543
ss86338164		10	68.322985	0.8438	1.0438	0.307302	0.0254	0.0066814081
ss86336311		10	68.355898	0.6060	4.2742	0.039064	0.0395	0.0188581235
ss61512405	*rs41611711*	10	68.376573	0.6060	1.5939	0.207196	0.0242	0.0115464037
	rs29021678	10	68.46713	0.5473	1.6067	0.205373	0.0240	0.0118742717
	rs29020033	10	68.492506	0.5587	1.7029	0.192341	0.0249	0.0122950634
ss61470284	*rs41568673*	10	68.586182	0.5179	0.8690	0.351553	0.0171	0.0085149065
ss86289642		10	68.606585	0.0408	0.0740	0.785639	0.0124	0.0009733879
ss105241615		10	68.826019	0.7421	12.5497	0.000423	0.0735	0.0281222800
ss117975218		10	68.941586	0.8023	2.6607	0.103306	0.0377	0.0119720741
ss86294876		10	68.96443	0.0695	6.5378	0.010772	0.0913	0.0118122141
ss86337947		10	69.05025	0.9563	0.0316	0.858986	0.0079	0.0006595184
ss86284266		10	69.136326	0.8947	8.5696	0.003530	0.0860	0.0162015623
ss61556155	*rs41651467*	10	69.177539	0.2715	2.7740	0.096256	0.0351	0.0138860149
ss86306178		10	69.199384	0.5258	13.3383	0.000280	0.0666	0.0331888714
ss105241610		10	69.247532	0.2271	2.8229	0.093378	0.0375	0.0131501361
ss86341754		10	69.359687	0.5745	9.6618	0.001958	0.0583	0.0284996119
ss105241611		10	69.497384	0.8123	4.4400	0.035464	0.0491	0.0149636943
ss86286563		10	69.589399	0.2693	0.2126	0.644857	0.0096	0.0037815719
ss61569332	*rs41666416*	10	69.649242	0.2693	0.1815	0.670181	0.0088	0.0034825394
ss86340468		10	69.696863	0.0688	6.2694	0.012512	0.0898	0.0115043607
ss86338444		10	69.723802	0.1755	0.0286	0.865814	0.0040	0.0011692638
ss61569331	*rs41666415*	10	69.760269	0.1963	5.5874	0.018363	0.0535	0.0168653833
ss65072504	*rs43160537*	10	69.796083	0.1017	0.1408	0.707635	0.0112	0.0020547927
	rs29012644	10	69.880972	0.8532	0.0421	0.837577	0.0055	0.0013699744
ss63411979	*rs42221086*	10	69.947439	0.4169	0.7025	0.402225	0.0154	0.0074631018
ss105241630		10	69.975517	0.8668	0.0103	0.919006	0.0028	0.0006516508
ss61497152	*rs41594582*	10	69.999526	0.4971	3.7454	0.053356	0.0353	0.0176585302
ss86338667		10	70.036339	0.2779	6.4029	0.011613	0.0516	0.0206913234
ss86337271		10	70.064822	0.4011	3.8129	0.051259	0.0382	0.0183347740
ss117969846		10	70.096967	0.6117	5.7318	0.016924	0.0450	0.0213928895
ss86334409		10	70.121373	0.8417	3.9846	0.046307	0.0513	0.0136666098
ss86329348		10	70.147745	0.4921	6.1848	0.013119	0.0459	0.0229667541
ss61569328	*rs41666412*	10	70.193477	0.1934	1.2882	0.256773	0.0257	0.0080151300
ss61497148	*rs41594578*	10	70.226964	0.5007	0.3179	0.573083	0.0103	0.0051537279
ss117969865		10	70.2621	0.4441	0.2363	0.627017	0.0088	0.0043507889
ss63410602	*rs42227324*	10	70.287191	0.1827	0.0063	0.936918	0.0019	0.0005697141
ss117970012		10	70.341718	0.3417	13.8303	0.000216	0.0728	0.0327575876
ss63410236	*rs42226002*	10	70.373013	0.6032	1.9213	0.166155	0.0259	0.0124141532
ss86327686		10	70.407328	0.6454	5.5222	0.019055	0.0467	0.0213775286
ss86287108		10	70.430549	0.6182	3.8795	0.049277	0.0379	0.0178835021
ss117970059		10	70.455224	0.2163	0.1681	0.681976	0.0094	0.0031941253
ss86285068		10	70.531394	0.9112	0.1441	0.704323	0.0127	0.0020515318
ss86310484		10	70.552188	0.0967	0.1904	0.662691	0.0141	0.0024665327
ss61569322	*rs41666406*	10	70.581259	0.8675	1.1600	0.281843	0.0306	0.0070394514

ss86334133		10	70.719018	0.8510	0.0630	0.801894	0.0065	0.0016610138
	rs29010868	10	70.863042	0.3231	3.3662	0.066975	0.0362	0.0158382443
ss64238278	*rs42540090*	10	70.951968	0.1554	1.6496	0.199440	0.0329	0.0086326474
ss86323371		10	71.11956	0.4979	3.2856	0.070323	0.0325	0.0162301646
ss86299178		10	71.237689	0.9155	0.8154	0.366836	0.0314	0.0048543094
ss86334722		10	71.261025	0.1082	0.0322	0.857616	0.0055	0.0010517520
ss61530388	*rs41624664*	10	71.288958	0.6540	1.1482	0.284304	0.0213	0.0096282576
ss86335790		10	71.340536	0.7085	2.1894	0.139417	0.0299	0.0123659447
	rs29025208	10	71.397865	0.0774	1.6958	0.193264	0.0432	0.0061644669
ss65557030	*rs43636746*	10	71.442939	0.1905	0.0071	0.932951	0.0019	0.0005967235
ss117969909		10	71.50653	0.8324	1.3576	0.244350	0.0290	0.0080834297
ss65556636	*rs43639551*	10	71.75121	0.7543	0.3396	0.560276	0.0126	0.0046880312
ss65556621	*rs43639536*	10	71.776091	0.1139	5.8091	0.016202	0.0705	0.0142298658
ss65556598	*rs43638613*	10	71.806995	0.2421	0.9902	0.320051	0.0213	0.0078049170
ss61504043	*rs41602732*	10	71.842089	0.3080	1.5001	0.221073	0.0242	0.0103374391
ss61556175	*rs41651487*	10	71.863233	0.7106	1.7024	0.192407	0.0260	0.0107125529
ss86315893		10	71.892726	0.4542	0.0197	0.888355	0.0025	0.0012588220
ss65556368	*rs43637084*	10	71.951996	0.0931	5.1493	0.023561	0.0705	0.0119117354
ss64883121	*rs42977845*	10	72.008707	0.2958	3.9180	0.048165	0.0396	0.0165000542
ss64883089	*rs42977113*	10	72.036074	0.6970	3.7530	0.053116	0.0384	0.0162127038
	rs29009594	10	72.073024	0.9205	2.7869	0.095490	0.0581	0.0085000482
	rs29024594	10	72.094679	0.6798	1.3912	0.238600	0.0232	0.0100847225
ss61490881	*rs41591885*	10	72.132822	0.4097	1.0550	0.304715	0.0192	0.0093108331
	rs29012457	10	72.176473	0.6289	2.1247	0.145396	0.0277	0.0129241235
ss65558746	*rs43642357*	10	72.18089	0.9219	0.9064	0.341403	0.0321	0.0046144535
ss86341575		10	72.253646	0.6189	9.1768	0.002542	0.0562	0.0265306693
ss86340421		10	72.367586	0.6404	8.4577	0.003751	0.0563	0.0259194101
ss61490870	*rs41591875*	10	72.409841	0.2672	0.4608	0.497468	0.0140	0.0054805526
ss61556203	*rs41651515*	10	72.483724	0.6354	2.5876	0.108155	0.0310	0.0143420968
ss61490868	*rs41591873*	10	72.560546	0.1862	0.3469	0.556066	0.0142	0.0042986975
ss65557296	*rs43639008*	10	72.830873	0.7564	0.0630	0.801903	0.0055	0.0020333915
ss86325013		10	72.854481	0.0523	7.1133	0.007829	0.1128	0.0111765372
ss65557273	*rs43638985*	10	72.886487	0.3130	1.2035	0.272999	0.0221	0.0094972343
ss64296136	*rs42571083*	10	72.975248	0.4398	0.7555	0.385053	0.0160	0.0078927239
ss86339281		10	73.015646	0.6397	0.0326	0.856863	0.0035	0.0016076153
ss64295804	*rs42570718*	10	73.0668	0.6426	0.0007	0.978574	0.0005	0.0002393618
ss61566962		10	73.13476	0.6390	0.0018	0.966494	0.0008	0.0003747873
ss64814895	*rs42905207*	10	73.206854	0.7994	0.8997	0.343193	0.0218	0.0069943200
ss86314792		10	73.405615	0.2077	0.1975	0.656917	0.0098	0.0032295045
ss86282380		10	73.4272	0.2092	0.0876	0.767285	0.0066	0.0021702784
ss63892290	*rs41862338*	10	73.505882	0.3381	1.0387	0.308487	0.0194	0.0086776846
ss63892308	*rs41862346*	10	73.53333	0.6497	3.8336	0.050633	0.0363	0.0165286276
ss117964125		10	73.565351	0.3703	0.0319	0.858284	0.0034	0.0015767776
ss86307427		10	73.650991	0.3245	3.1242	0.077574	0.0337	0.0147643919
ss86331036		10	73.724114	0.1755	0.2200	0.639209	0.0109	0.0031609061
ss117970056		10	73.749216	0.6562	3.1204	0.077758	0.0331	0.0149330174
ss61521075	*rs41620093*	10	73.801364	0.4878	4.2075	0.040619	0.0363	0.0181555524

ss64711007	*rs42802606*	10	73.87675	0.7686	7.1073	0.007855	0.0577	0.0205205154
ss61504125	*rs41602814*	10	73.935992	0.1497	0.9751	0.323760	0.0260	0.0066181032
	rs29025644	10	73.972074	0.3804	3.8611	0.049815	0.0364	0.0171410935
ss117969774		10	73.998289	0.3668	3.6790	0.055510	0.0360	0.0167428258
ss61504124	*rs41602813*	10	74.020674	0.8675	3.1436	0.076663	0.0478	0.0109852762
ss65558989	*rs43643999*	10	74.050643	0.7264	0.0255	0.873239	0.0034	0.0013326161
ss64671142	*rs42768806*	10	74.079101	0.2572	0.2751	0.600097	0.0112	0.0042747026
ss64671151	*rs42768815*	10	74.101857	0.6383	1.1009	0.294433	0.0204	0.0094133242
ss86298437		10	74.1424	0.3596	0.8556	0.355299	0.0180	0.0082814694
ss65559021	*rs43636532*	10	74.380448	0.9592	0.4918	0.483346	0.0315	0.0024650740
ss61472710	*rs41573933*	10	74.414413	0.3782	3.6971	0.054915	0.0362	0.0170442780
ss63805042	*rs42359348*	10	74.4501	0.3718	2.0972	0.148021	0.0272	0.0127112024
ss63805224	*rs42360239*	10	74.523159	0.3560	1.1970	0.274304	0.0204	0.0093722079
ss64063637	*rs42453465*	10	75.246968	0.8138	0.5651	0.452475	0.0178	0.0054007608
ss86283754		10	75.317705	0.5344	1.5650	0.211350	0.0230	0.0114564997
ss86335500		10	75.338903	0.1361	0.0046	0.945726	0.0018	0.0004267561
ss86329575		10	75.381733	0.4420	5.1866	0.023064	0.0421	0.0207558994
ss61569312	*rs41666396*	10	75.422714	0.2715	0.6516	0.419831	0.0166	0.0065472068
ss86303246		10	75.459155	0.8610	0.3273	0.567435	0.0152	0.0036283528
ss64399898	*rs42624287*	10	75.492508	0.2550	0.4724	0.492101	0.0143	0.0054173510
ss86337465		10	75.601775	0.7894	11.3710	0.000787	0.0738	0.0245354670
ss117969843		10	75.660031	0.3266	6.0606	0.014064	0.0475	0.0209102658
ss86278752		10	75.716786	0.3080	2.9781	0.084842	0.0342	0.0145901596
ss61556723	*rs41656325*	10	75.745784	0.6791	2.1145	0.146362	0.0288	0.0125469297
ss86299068		10	75.781974	0.6762	9.2768	0.002408	0.0612	0.0268013842
ss86294938		10	75.813927	0.6948	10.8725	0.001026	0.0668	0.0283341899
ss117970055		10	75.84164	0.6948	10.8725	0.001026	0.0668	0.0283341899
ss86299564		10	75.872732	0.9277	9.7983	0.001820	0.1113	0.0149423865
ss117964149		10	75.989025	0.8460	0.0036	0.952199	0.0015	0.0003938271
ss86313413		10	76.01092	0.1526	0.0285	0.865897	0.0043	0.0011032746
ss86296429		10	76.045717	0.6841	3.6886	0.055194	0.0381	0.0164793651
ss61529064	*rs41631975*	10	76.116048	0.6282	2.9096	0.088502	0.0327	0.0152945038
ss105304199		10	76.152577	0.4069	8.1936	0.004330	0.0529	0.0255175663
ss86303104		10	76.179313	0.7658	4.0746	0.043916	0.0431	0.0154671251
ss86332935		10	76.489794	0.5652	2.0574	0.151916	0.0272	0.0133446001
	rs29011199	10	76.602726	0.5910	0.2182	0.640581	0.0089	0.0042849545
ss86328892		10	76.694741	0.4327	0.0199	0.887884	0.0026	0.0012669392
ss86291779		10	76.981291	0.8567	1.3217	0.250684	0.0307	0.0075269178
ss117969792		10	77.095218	0.5566	0.0003	0.986080	0.0003	0.0001630369
ss61512824	*rs41615325*	10	77.253854	0.2063	1.3896	0.238884	0.0276	0.0090487498
ss86290668		10	77.299312	0.0731	0.7069	0.400774	0.0309	0.0041897221
ss61505970	*rs41600499*	10	77.353438	0.8632	1.0924	0.296306	0.0282	0.0066504999
ss86288865		10	77.451636	0.3675	2.3759	0.123674	0.0293	0.0136148873
ss86337479		10	77.564227	0.1261	5.9987	0.014562	0.0682	0.0150268890
ss117964103		10	77.594016	0.9384	1.9560	0.162388	0.0532	0.0061528551
ss86299444		10	77.672167	0.4878	0.4535	0.500886	0.0122	0.0060753835
ss61491079	*rs41593879*	10	77.73708	0.9097	1.3376	0.247852	0.0370	0.0060803602

ss61469067	*rs41566978*	10	77.832357	0.2486	2.2302	0.135788	0.0321	0.0119897583
ss61556709	*rs41656311*	10	77.864156	0.2292	3.3626	0.067119	0.0402	0.0142177746
ss86336966		10	77.927864	0.6067	7.3746	0.006779	0.0510	0.0243551025
ss61556710	*rs41656312*	10	78.070718	0.6046	5.6598	0.017626	0.0441	0.0210888626
ss86288306		10	78.143999	0.0609	4.3033	0.038406	0.0801	0.0091581301
ss86285750		10	78.283667	0.5609	0.0058	0.939298	0.0014	0.0006799409
ss117964144		10	78.31429	0.3274	0.7326	0.392344	0.0176	0.0077531674
ss86295557		10	78.394823	0.2436	2.3192	0.128238	0.0327	0.0120620024
ss86323563		10	78.415407	0.2042	0.5707	0.450223	0.0178	0.0057991309
ss86300560		10	78.490365	0.4592	3.6131	0.057739	0.0371	0.0184132166
ss61491337	*rs41597133*	10	78.524351	0.8317	3.8969	0.048770	0.0486	0.0136070300
ss61491098	*rs41593896*	10	78.553975	0.2278	0.5688	0.450972	0.0163	0.0057437110
ss86325283		10	78.597509	0.3990	0.6997	0.403180	0.0154	0.0073853139
ss86323843		10	78.665533	0.8524	0.6938	0.405171	0.0222	0.0055744059
	rs29015590	10	78.753759	0.1540	1.0997	0.294692	0.0274	0.0071499824
ss61491095	*rs41593893*	10	78.817442	0.1297	1.8765	0.171172	0.0375	0.0084592190
ss117969897		10	78.870385	0.8718	2.0303	0.154641	0.0391	0.0087425293
ss86304559		10	78.918396	0.1948	0.5817	0.445919	0.0174	0.0054634114
ss86313501		10	78.992457	0.8259	0.1001	0.751775	0.0075	0.0021429539
ss86339112		10	79.035951	0.6254	0.3095	0.578146	0.0107	0.0050188740
ss86285426		10	79.100241	0.7815	0.0399	0.841781	0.0045	0.0015376715
ss86327788		10	79.132369	0.5415	0.0071	0.932947	0.0016	0.0007919573
ss86283273		10	79.160472	0.6132	1.4761	0.224797	0.0232	0.0110166270
ss86326290		10	79.229821	0.3675	0.3863	0.534473	0.0121	0.0056132058
ss86314343		10	79.317854	0.8660	5.9390	0.015059	0.0676	0.0156909103
ss86318310		10	79.349778	0.1583	4.4838	0.034572	0.0537	0.0143042589
ss86326999		10	79.422597	0.6254	14.7453	0.000134	0.0744	0.0348765511
ss86338436		10	79.445895	0.3725	14.7947	0.000131	0.0746	0.0348780539
ss86283793		10	79.558719	0.3231	1.6878	0.194323	0.0259	0.0113128503
ss86338920		10	79.584029	0.0824	4.2385	0.039888	0.0700	0.0105803334
ss117966049		10	79.706855	0.4599	8.2123	0.004286	0.0526	0.0261292068
ss61493859	*rs41591080*	10	79.864902	0.5458	8.6878	0.003311	0.0539	0.0267042107
ss117970043		10	79.933252	0.6103	2.9692	0.085308	0.0320	0.0152282751
ss61566847	*rs41668361*	10	80.003074	0.6913	2.7069	0.100366	0.0329	0.0140379348
ss86298072		10	80.065392	0.5552	3.6632	0.056036	0.0354	0.0174886026
ss86322008		10	80.106049	0.6490	0.6095	0.435252	0.0151	0.0068764231
ss86300261		10	80.142362	0.6332	0.4777	0.489706	0.0134	0.0062200066
ss86340095		10	80.215901	0.6354	1.2130	0.271119	0.0210	0.0097107156
ss86294244		10	80.247566	0.6748	0.2341	0.628630	0.0096	0.0041983322
ss61514793	*rs41616349*	10	80.295284	0.1239	0.1291	0.719466	0.0098	0.0021218540
ss105236688		10	80.403647	0.1948	0.8434	0.358735	0.0214	0.0067081367
ss86326876		10	80.440905	0.3431	0.3474	0.555772	0.0117	0.0052942830
ss61491345	*rs41597140*	10	80.499737	0.6354	6.1406	0.013447	0.0471	0.0218076904
		10	80.601154	0.7858	3.6521	0.056410	0.0425	0.0143229866
ss86337460		10	80.634385	0.3181	5.6973	0.017257	0.0469	0.0203418907
	rs29027326	10	80.769773	0.3195	5.6752	0.017474	0.0469	0.0203722060
ss61557261	*rs41652853*	10	80.795997	0.3660	1.4583	0.227616	0.0234	0.0108829278

ss86341654		10	81.115387	0.6440	5.4291	0.020089	0.0454	0.0208119190
ss105236698		10	81.191638	0.6877	1.2068	0.272346	0.0226	0.0097009679
ss86338656		10	81.435879	0.4219	0.7138	0.398486	0.0157	0.0076810052
ss61510592	*rs41605215*	10	81.509011	0.3223	0.1683	0.681729	0.0082	0.0035783976
ss86294684		10	81.723683	0.1941	0.8283	0.363075	0.0214	0.0067036956
ss86325585		10	81.749638	0.1218	3.0436	0.081499	0.0494	0.0105755606
ss86340813		10	81.907315	0.1418	0.0755	0.783513	0.0075	0.0018193537
ss86326360		10	81.949369	0.1175	8.1995	0.004316	0.0817	0.0169470100
ss61557265	*rs41652857*	10	82.049782	0.2794	0.4929	0.482876	0.0146	0.0058595150
ss86287650		10	82.073171	0.4735	0.4793	0.488967	0.0131	0.0065199016
ss61510591	*rs41605214*	10	82.152683	0.5681	0.0946	0.758472	0.0057	0.0028008809
ss61557267	*rs41652859*	10	82.200105	0.2256	1.9801	0.159828	0.0308	0.0107767604
ss61536573	*rs41632698*	10	82.291535	0.7400	0.2381	0.625759	0.0106	0.0040741278
ss105236700		10	82.371367	0.5831	0.0012	0.972465	0.0007	0.0003195670
ss65560669	*rs43641573*	10	82.506615	0.4162	0.1480	0.700565	0.0072	0.0034952083
ss117964109		10	82.562804	0.4155	3.3458	0.067806	0.0330	0.0160509596
ss86337828		10	82.691395	0.8574	0.0233	0.878649	0.0039	0.0009632156
ss86289700		10	82.71958	0.1691	0.1899	0.663114	0.0105	0.0029508474
ss105236704		10	82.750333	0.2672	0.0718	0.788766	0.0054	0.0021062730
ss86326937		10	82.7811	0.1648	0.1162	0.733303	0.0083	0.0022829781
ss86274255		10	82.876726	0.7228	1.6999	0.192734	0.0263	0.0105537116
	rs29020120	10	82.90909	0.7364	2.4799	0.115761	0.0326	0.0126656912
ss65560831	*rs43643134*	10	82.930121	0.6476	0.0012	0.972097	0.0007	0.0003081334
ss86329313		10	82.953539	0.0752	0.9167	0.338667	0.0341	0.0047418960
ss65560924	*rs43643226*	10	82.987613	0.0809	0.0028	0.957921	0.0018	0.0002692223
ss86296832		10	83.008288	0.6877	1.5630	0.211643	0.0253	0.0108727143
ss65560952	*rs43644054*	10	83.03653	0.1426	0.6692	0.413626	0.0220	0.0053733384
ss86303126		10	83.058523	0.7156	0.0368	0.847912	0.0040	0.0016384309
ss86305529		10	83.080685	0.2278	0.0209	0.885038	0.0033	0.0011517812
ss86303997		10	83.10317	0.1404	0.0029	0.957216	0.0014	0.0003453454
ss61504239	*rs41604128*	10	83.352578	0.2772	2.7621	0.096972	0.0346	0.0138615951
ss86341735		10	83.388809	0.4778	1.4813	0.223991	0.0220	0.0109974356
ss86304173		10	83.409989	0.2070	0.1339	0.714529	0.0084	0.0027417804
ss61557826	*rs41655405*	10	83.443719	0.7658	3.2648	0.071215	0.0385	0.0138165941
ss61491621	*rs41589008*	10	83.476843	0.7930	0.1339	0.714529	0.0084	0.0027417804
ss86306300		10	83.523843	0.0946	2.8180	0.093662	0.0503	0.0086188355
ss61510659	*rs41605982*	10	83.562204	0.6497	2.6659	0.102970	0.0323	0.0147006922
ss65561753	*rs43642216*	10	83.642486	0.8804	1.3044	0.253805	0.0310	0.0065216116
ss86321487		10	83.707371	0.7514	0.5850	0.444622	0.0166	0.0062019120
ss86324655		10	83.734763	0.2586	2.1812	0.140158	0.0306	0.0117503880
ss61510664	*rs41605987*	10	83.775089	0.0530	3.5422	0.060244	0.0759	0.0076185588
ss65111948	*rs43202348*	10	83.805892	0.9477	1.0252	0.311643	0.0411	0.0040764142
ss86295994		10	83.836826	0.0924	2.4273	0.119695	0.0471	0.0078995820
ss86313855		10	83.890249	0.9140	6.2465	0.012673	0.0805	0.0126455105
ss64144247	*rs42499023*	10	83.913739	0.0630	0.9126	0.339756	0.0361	0.0042627634
ss64144267	*rs42499933*	10	83.93904	0.9047	6.1630	0.013279	0.0771	0.0132946779
ss86334253		10	83.986714	0.5587	0.0533	0.817413	0.0042	0.0020644933

ss86289303		10	84.012933	0.3238	2.2652	0.132762	0.0295	0.0129312211
ss86340864		10	84.034519	0.4649	2.9891	0.084269	0.0321	0.0159461855
ss86329378		10	84.061902	0.9277	0.9562	0.328486	0.0354	0.0047458327
ss86290190		10	84.166597	0.4721	0.7887	0.374795	0.0163	0.0081438867
ss86330736		10	84.1958	0.8059	0.1898	0.663259	0.0098	0.0030791423
ss86309458		10	84.237784	0.6712	1.3178	0.251374	0.0230	0.0101556095
ss86302941		10	84.311036	0.6705	0.8155	0.366823	0.0181	0.0079765021
ss61529091	*rs41632001*	10	84.349894	0.6734	1.0800	0.299057	0.0207	0.0091162848
ss65562876	*rs43648436*	10	84.45378	0.1361	3.8915	0.048925	0.0514	0.0120827149
ss86296079		10	84.486829	0.5093	0.6391	0.424313	0.0147	0.0073238733
ss65562889	*rs43648449*	10	84.515568	0.8539	0.3489	0.554952	0.0157	0.0039098785
ss61474616	*rs41571111*	10	84.619556	0.7507	2.5300	0.112157	0.0332	0.0124373118
ss61491892	*rs41591956*	10	84.967368	0.6691	2.3113	0.128890	0.0295	0.0130490866
ss86303463		10	85.03067	0.8467	1.5715	0.210413	0.0316	0.0082012518
ss86331387		10	85.213922	0.9191	1.1455	0.284856	0.0353	0.0052466579
ss86339357		10	85.329879	0.6375	1.3327	0.248721	0.0222	0.0102484041
ss86337875		10	85.349986	0.6397	1.8783	0.170971	0.0263	0.0121426756
ss86340123		10	85.40242	0.5408	2.9185	0.088013	0.0320	0.0158933401
ss86278326		10	85.441578	0.7457	1.7486	0.186489	0.0294	0.0111412320
ss86328521		10	85.526676	0.1784	1.2953	0.255473	0.0260	0.0076345331
ss61558415	*rs41656867*	10	85.555355	0.4319	2.1488	0.143130	0.0271	0.0133040078
ss65563239	*rs43642998*	10	85.578309	0.2185	0.0186	0.891693	0.0031	0.0010430731
ss65563244	*rs43643003*	10	85.611199	0.1218	5.5207	0.019072	0.0665	0.0142331351
ss86340259		10	85.636535	0.7844	4.2501	0.039618	0.0468	0.0158187983
ss86295619		10	85.698967	0.8309	9.6190	0.002004	0.0772	0.0216970293
ss86319840		10	85.73309	0.5150	0.5062	0.477012	0.0133	0.0066647497
	rs29016350	10	85.85961	0.8309	4.5011	0.034226	0.0535	0.0150215717
ss86320800		10	85.962874	0.0867	2.0381	0.153849	0.0476	0.0075362692
ss86338988		10	86.1469	0.3868	0.1100	0.740226	0.0062	0.0029360462
ss65563751	*rs43647808*	10	86.203318	0.1089	0.0083	0.927284	0.0027	0.0005245962
ss65559670	*rs43642675*	10	86.227018	0.3574	0.4930	0.482826	0.0134	0.0061744915
ss61491087	*rs41593885*	10	86.283968	0.8804	0.5137	0.473793	0.0203	0.0042782008
ss65563771	*rs43647828*	10	86.342629	0.3868	3.5582	0.059667	0.0351	0.0166328064
ss86301235		10	86.410394	0.2636	5.3085	0.021515	0.0483	0.0187420557
ss65559732	*rs43643837*	10	86.467009	0.3152	12.4242	0.000452	0.0706	0.0304917476
ss86341013		10	86.525096	0.7364	0.1274	0.721297	0.0075	0.0029043906
ss86286750		10	86.570129	0.8976	0.0193	0.889643	0.0043	0.0007894338
ss105236724		10	86.59704	0.4721	9.6897	0.001929	0.0576	0.0287090802
ss65560119	*rs43636524*	10	86.621758	0.8223	9.5221	0.002111	0.0754	0.0220391711
ss86337603		10	86.651132	0.2758	13.6624	0.000236	0.0755	0.0301531264
ss86332407		10	86.692512	0.2457	1.5957	0.206942	0.0271	0.0100634540
ss86340109		10	86.712645	0.7794	0.5393	0.462974	0.0163	0.0056154997
ss86338594		10	86.842558	0.4119	2.4248	0.119887	0.0297	0.0143786192
ss65563930	*rs43650187*	10	86.866841	0.4549	0.0113	0.915236	0.0020	0.0010033695
ss86322005		10	86.889262	0.4011	9.6092	0.002014	0.0581	0.0279335683
ss86335671		10	86.93961	0.2572	0.6794	0.410077	0.0174	0.0066442653
ss65564052	*rs43650909*	10	86.973772	0.2937	5.4323	0.020053	0.0474	0.0196710251

ss61510793	*rs41607013*	10	87.024251	0.1347	0.9340	0.334154	0.0266	0.0061932004
ss86339498		10	87.099116	0.4348	1.7537	0.185848	0.0245	0.0120461073
ss86321529		10	87.17995	0.1956	1.8676	0.172196	0.0317	0.0099750536
ss61558913	*rs41654563*	10	87.219907	0.4585	0.2189	0.640000	0.0089	0.0044144674
ss65564312	*rs43645171*	10	87.258736	0.7858	0.0070	0.933303	0.0019	0.0006374261
ss62838642	*rs42014800*	10	87.29809	0.4606	0.1253	0.723474	0.0068	0.0033554786
ss86330354		10	87.372954	0.8317	15.7447	0.000080	0.0953	0.0266893413
ss86285477		10	87.407822	0.7027	0.2087	0.647947	0.0092	0.0038378733
ss65105249	*rs43191630*	10	87.481635	0.2328	0.0726	0.787648	0.0058	0.0020573426
ss86277867		10	87.50252	0.9119	0.1344	0.713992	0.0119	0.0019054240
ss86297275		10	87.554453	0.3446	5.8239	0.016067	0.0462	0.0208591755
ss86329395		10	87.600067	0.2056	3.7881	0.052022	0.0438	0.0143037403
ss61558932	*rs41654582*	10	87.62231	0.2894	8.0792	0.004609	0.0569	0.0234145535
ss86316791		10	87.797851	0.6726	0.0641	0.800251	0.0049	0.0021525020
ss86307546		10	87.817944	0.7787	0.0516	0.820362	0.0049	0.0016897213
ss65564891	*rs43651034*	10	87.942523	0.3539	0.0013	0.971693	0.0007	0.0003197582
ss65564900	*rs43651043*	10	87.962578	0.3539	0.0013	0.971693	0.0007	0.0003197582
ss65564907	*rs43651050*	10	87.983511	0.5938	4.8120	0.028592	0.0411	0.0198336356
ss65564924	*rs43651067*	10	88.01774	0.5079	2.1162	0.146198	0.0275	0.0137585890
ss86315991		10	88.052728	0.5466	2.0884	0.148876	0.0267	0.0132154408
ss61558935	*rs41654585*	10	88.077609	0.2450	4.0536	0.044461	0.0437	0.0161624411
ss86323608		10	88.110208	0.5072	1.4680	0.226068	0.0229	0.0114378417
ss86338120		10	88.157112	0.1755	3.7514	0.053167	0.0477	0.0138014163
ss86300450		10	88.177926	0.4291	0.5025	0.478653	0.0132	0.0064806108
ss86340922		10	88.203105	0.7299	0.5440	0.461047	0.0154	0.0060793262
ss86331454		10	88.231184	0.5688	2.3386	0.126658	0.0294	0.0143996452
ss86319582		10	88.290172	0.3374	3.5329	0.060582	0.0375	0.0167696824
ss86281727		10	88.318005	0.2543	6.6772	0.009968	0.0545	0.0206523882
ss86329160		10	88.352188	0.7514	7.0715	0.008012	0.0559	0.0208691902
ss65561825	*rs43642888*	10	88.514656	0.7980	5.2885	0.021762	0.0532	0.0171539737
ss61491627	*rs41589014*	10	88.585106	0.5186	0.2632	0.608071	0.0093	0.0046485440
ss86319212		10	88.640385	0.4907	0.0468	0.828870	0.0039	0.0019624043
ss65561948	*rs43643710*	10	88.667968	0.2858	1.5986	0.206521	0.0257	0.0104759469
ss86339890		10	88.6892	0.7801	3.0195	0.082713	0.0383	0.0131324260
ss46526565	*rs41257023*	10	88.728814	0.7092	1.9006	0.168452	0.0278	0.0114796748
ss65562113	*rs43640475*	10	88.753384	0.2765	1.6835	0.194896	0.0265	0.0106085799
ss61558395		10	88.79123	0.7844	4.8642	0.027746	0.0490	0.0165711654
ss117964138		10	88.848592	0.3510	10.8672	0.001029	0.0629	0.0286648587
ss65562222	*rs43641384*	10	88.875895	0.3646	0.0476	0.827354	0.0043	0.0020022506
ss86335611		10	88.981692	0.3596	0.1437	0.704772	0.0075	0.0034315308
ss86316541		10	89.014843	0.7350	0.5513	0.458054	0.0151	0.0058917970
ss86306027		10	89.035785	0.1963	5.8986	0.015405	0.0569	0.0179474845
ss86292429		10	89.068445	0.2493	17.9634	0.000026	0.0916	0.0342715244
ss86301748		10	89.125553	0.2249	4.0312	0.045053	0.0445	0.0155112535
ss86291128		10	89.153666	0.7593	5.5597	0.018654	0.0504	0.0184069624
ss86282625		10	89.183996	0.6655	9.1756	0.002543	0.0581	0.0258814201
ss86297245		10	89.214786	0.6318	4.7895	0.028965	0.0418	0.0194614142

	rs29015733	10	89.404007	0.6662	2.5607	0.110007	0.0310	0.0137992155
ss86315230		10	89.501052	0.0774	3.9933	0.046070	0.0680	0.0097125609
ss65562607	*rs43645367*	10	89.538251	0.7020	0.4310	0.511727	0.0133	0.0055619079
ss65562657	*rs43645417*	10	89.577719	0.1605	6.9108	0.008757	0.0656	0.0176637502
ss63479651	*rs42249704*	10	89.620818	0.6762	0.2052	0.650689	0.0090	0.0039581084
ss86285139		10	89.65567	0.6454	0.4719	0.492344	0.0132	0.0060252381
ss63479471	*rs42249644*	10	89.695557	0.3725	0.6628	0.415864	0.0155	0.0072388789
ss61475618	*rs41575690*	10	89.71928	0.6275	0.6628	0.415864	0.0155	0.0072388789
ss117969927		10	89.787587	0.6970	0.9222	0.337223	0.0190	0.0080053700
ss86287010		10	89.835156	0.1862	2.5152	0.113204	0.0380	0.0115074350
ss64734042	*rs42830999*	10	89.931995	0.6991	0.1164	0.733109	0.0068	0.0028493166
ss86340268		10	89.966395	0.2586	0.0583	0.809274	0.0051	0.0019561610
ss61523812	*rs41623571*	10	90.016629	0.9176	3.1685	0.075506	0.0598	0.0090375076
ss86319941		10	90.144955	0.9105	1.7458	0.186845	0.0427	0.0069667689
ss61515316	*rs41613674*	10	90.185112	0.7027	2.1166	0.146163	0.0302	0.0126305931
ss86283765		10	90.230434	0.4986	0.0016	0.968189	0.0007	0.0003669992
ss86288929		10	90.253057	0.4097	4.0228	0.045275	0.0376	0.0181790842
ss63767031	*rs42345416*	10	90.277721	0.1060	0.0955	0.757439	0.0094	0.0017792696
ss86338125		10	90.302306	0.3761	0.7215	0.395958	0.0160	0.0074892030
ss86341056		10	90.333578	0.2915	2.6213	0.105888	0.0333	0.0137379151
ss86291568		10	90.39238	0.5064	1.2845	0.257454	0.0211	0.0105675500
ss86331873		10	90.43564	0.5344	2.0851	0.149189	0.0279	0.0138919442
ss61515308	*rs41613666*	10	90.462708	0.3610	0.6053	0.436830	0.0147	0.0067856538
ss86310250		10	90.545799	0.1991	8.8010	0.003114	0.0670	0.0213638204
ss61515314	*rs41613672*	10	90.629938	0.5989	4.5696	0.032893	0.0382	0.0183429983
ss86336552		10	90.672399	0.2679	1.3121	0.252405	0.0229	0.0089787985
ss86317394		10	90.715327	0.7822	0.0415	0.838729	0.0046	0.0015521515
ss86331288		10	90.785983	0.5616	7.3994	0.006688	0.0500	0.0246085909
ss61558951	*rs41654601*	10	90.84816	0.3610	0.0965	0.756130	0.0060	0.0027783622
ss86336088		10	90.887327	0.3711	0.3825	0.536466	0.0121	0.0056335761
ss65566177	*rs43645722*	10	90.915797	0.4706	1.1196	0.290361	0.0192	0.0095539083
ss65566208	*rs43646152*	10	90.945939	0.2099	0.5584	0.455173	0.0174	0.0057623752
ss61558955	*rs41654605*	10	90.969576	0.2271	0.0250	0.874368	0.0035	0.0012260306
ss86291540		10	91.068411	0.0372	0.3417	0.559025	0.0283	0.0020304785
ss86274383		10	91.160686	0.3145	0.0515	0.820467	0.0045	0.0019441542
ss86313382		10	91.19333	0.3947	4.7944	0.028884	0.0410	0.0196061582
ss61558938	*rs41654588*	10	91.215058	0.1777	0.0198	0.888256	0.0035	0.0010254597
ss61492132	*rs41594287*	10	91.290323	0.7299	1.5741	0.210033	0.0257	0.0101441209
ss86308908		10	91.31486	0.4713	0.3798	0.537919	0.0118	0.0058992694
ss86312407		10	91.346769	0.4448	0.2151	0.642917	0.0090	0.0044230951
ss86314791		10	91.376701	0.4448	0.2151	0.642917	0.0090	0.0044230951
ss65565626	*rs43646470*	10	91.398979	0.4434	0.3397	0.560187	0.0112	0.0055415137
ss61558941	*rs41654591*	10	91.420639	0.5602	0.6267	0.428836	0.0153	0.0075173954
ss86328098		10	91.477603	0.4835	0.4735	0.491606	0.0136	0.0067707425
ss65565874	*rs43648117*	10	91.503333	0.4642	1.9707	0.160816	0.0277	0.0137683929
ss65565970	*rs43649013*	10	91.556573	0.5322	0.0166	0.897560	0.0026	0.0012720388
ss86325129		10	91.607551	0.0322	0.5463	0.460085	0.0389	0.0024299770

ss117969993		10	91.66982	0.2665	0.0375	0.846600	0.0042	0.0016269510
ss86338264		10	91.692826	0.2400	0.7945	0.373048	0.0192	0.0069912768
ss86297417		10	91.721377	0.8768	0.0503	0.822561	0.0065	0.0014046138
ss86321603		10	91.754982	0.1175	5.9943	0.014598	0.0691	0.0143319680
ss86303775		10	91.803115	0.3438	0.2682	0.604719	0.0100	0.0044940305
ss86298064		10	91.834645	0.5380	0.0166	0.897550	0.0024	0.0011950889
ss105243032		10	91.863621	0.1734	0.0163	0.898588	0.0031	0.0008818984
ss86340023		10	91.901742	0.3646	5.1924	0.022988	0.0443	0.0205191148
ss117964157		10	91.959953	0.5344	0.3622	0.547467	0.0111	0.0055246112
ss86327737		10	91.983651	0.3983	1.7514	0.186129	0.0259	0.0124292039
ss86291479		10	92.064272	0.7034	6.7330	0.009664	0.0524	0.0218532611
ss61469290	*rs41568597*	10	92.088424	0.1848	1.0333	0.309736	0.0236	0.0070977597
ss86290387		10	92.110207	0.9076	3.0007	0.083670	0.0554	0.0092899586
ss86339301		10	92.14281	0.8095	0.5585	0.455121	0.0183	0.0056548023
ss86318223		10	92.191762	0.4520	1.4459	0.229595	0.0225	0.0111243446
ss65566833	*rs43650269*	10	92.21183	0.3202	0.1401	0.708338	0.0074	0.0032248175
ss117969809		10	92.233233	0.5344	0.4926	0.483011	0.0131	0.0065046055
ss86316244		10	92.266701	0.1891	0.3275	0.567323	0.0134	0.0041110750
ss105243039		10	92.299552	0.3496	0.3567	0.550548	0.0115	0.0052458594
ss61492378	*rs41596526*	10	92.362722	0.0509	0.5684	0.451138	0.0324	0.0031248190
ss61492141	*rs41594296*	10	92.398873	0.6239	8.1358	0.004469	0.0544	0.0255405637
ss86339583		10	92.440189	0.8252	8.7443	0.003211	0.0711	0.0205147426
ss65566430	*rs43647371*	10	92.465232	0.5287	2.8079	0.094252	0.0315	0.0157080049
ss86283422		10	92.504834	0.4477	0.0000	0.996842	0.0001	0.0000370754
ss86338248		10	92.570463	0.6504	0.1955	0.658502	0.0087	0.0039597682
ss117970041		10	92.605462	0.6554	0.7987	0.371802	0.0177	0.0079835147
ss86323849		10	92.639737	0.1504	0.0093	0.923228	0.0024	0.0006133698
ss61524410	*rs41620652*	10	92.669369	0.8689	0.2033	0.652241	0.0122	0.0027857931
ss86318864		10	92.726758	0.2242	0.3168	0.573724	0.0121	0.0041961968
ss86328073		10	92.772803	0.4921	10.1043	0.001545	0.0587	0.0293325046
ss64460228	*rs42649278*	10	92.798827	0.6211	5.9780	0.014732	0.0465	0.0218829444
ss86324995		10	92.825052	0.2142	13.7564	0.000225	0.0833	0.0280332799
ss61492387	*rs41597335*	10	92.892687	0.3890	10.6060	0.001182	0.0632	0.0300393293
ss61559553	*rs41657377*	10	92.939956	0.1332	3.6418	0.056756	0.0525	0.0121149092
ss86285467		10	92.965589	0.8675	3.3662	0.066975	0.0508	0.0116783410
ss61559547		10	92.987294	0.4577	14.6372	0.000142	0.0708	0.0351505359
ss61559544	*rs41657368*	10	93.016335	0.5595	12.2338	0.000499	0.0642	0.0316466830
	rs29026927	10	93.049441	0.7744	0.0266	0.870486	0.0036	0.0012697868
ss86336928		10	93.072439	0.1920	0.5517	0.457872	0.0182	0.0056523742
ss86336960		10	93.111289	0.5351	0.3181	0.572938	0.0106	0.0052954222
ss86283629		10	93.209869	0.3188	0.6547	0.418727	0.0162	0.0070481418
ss86338460		10	93.336974	0.6540	0.3242	0.569308	0.0109	0.0049150058
ss65567829	*rs43651752*	10	93.368152	0.4756	0.0665	0.796552	0.0047	0.0023314207
ss86308929		10	93.399875	0.1239	3.1883	0.074604	0.0497	0.0107925168
ss86309484		10	93.427139	0.7235	4.8963	0.027238	0.0471	0.0188450913
ss65567762	*rs43650985*	10	93.464034	0.4191	0.1773	0.673833	0.0079	0.0038377003
ss86297137		10	93.529042	0.7228	0.6795	0.410051	0.0167	0.0067080112

ss86337636		10	93.569226	0.8281	0.0109	0.916761	0.0025	0.0007178755
ss61559543	*rs41657367*	10	93.595703	0.5165	1.2435	0.265175	0.0215	0.0107178783
ss61559541	*rs41657365*	10	93.622263	0.5000	0.3758	0.540063	0.0118	0.0059052071
	rs29021343	10	93.645265	0.9319	8.0712	0.004629	0.1046	0.0132668251
ss105296910		10	93.723136	0.3696	0.0001	0.990795	0.0002	0.0001028765
ss86331693		10	93.749911	0.8130	1.1266	0.288878	0.0250	0.0075930943
ss65568256	*rs43647285*	10	93.838141	0.0759	1.8632	0.172696	0.0464	0.0065158565
ss64833821	*rs42931883*	10	93.881931	0.9391	3.5308	0.060657	0.0717	0.0082015958
ss86310290		10	94.136704	0.4807	0.0091	0.923976	0.0018	0.0009040804
ss63522365	*rs42263103*	10	94.162511	0.1117	6.4076	0.011582	0.0752	0.0149292999
ss63522323	*rs42263089*	10	94.192056	0.2307	5.2568	0.022159	0.0503	0.0178438476
ss61466911	*rs41565055*	10	94.233556	0.1805	3.5565	0.059729	0.0457	0.0135275704
	rs29025325	10	94.288112	0.2779	1.5501	0.213536	0.0258	0.0103446592
ss86326622		10	94.414576	0.7521	2.9629	0.085642	0.0364	0.0135566608
ss63521744	*rs42260897*	10	94.453242	0.5021	3.8955	0.048811	0.0373	0.0186256700
ss61530288	*rs41633366*	10	94.504837	0.3797	1.1127	0.291860	0.0209	0.0098488409
ss86289415		10	94.638	0.1175	7.7823	0.005420	0.0814	0.0168779413
ss63521177	*rs42258408*	10	94.669661	0.0931	3.5311	0.060644	0.0589	0.0099532530
ss86308663		10	94.705385	0.6053	2.5645	0.109743	0.0295	0.0140824867
ss65568275	*rs43647304*	10	94.970504	0.5444	3.7047	0.054668	0.0356	0.0176434346
ss65568398	*rs43648326*	10	95.145725	0.4348	1.5970	0.206756	0.0234	0.0114773409
ss86294679		10	95.171014	0.1884	1.2777	0.258720	0.0258	0.0078997928
ss65568422	*rs43649350*	10	95.204614	0.8037	1.6037	0.205804	0.0285	0.0089998426
ss117964119		10	95.23026	0.8030	0.1347	0.713693	0.0084	0.0026691745
ss61560074	*rs41655599*	10	95.325559	0.1375	6.9056	0.008782	0.0718	0.0170319403
	rs29010022	10	95.372033	0.7421	0.9899	0.320122	0.0209	0.0079993989
ss61570647	*rs41670201*	10	95.408559	0.2543	0.7156	0.397893	0.0179	0.0067927504
ss86331768		10	95.436179	0.4613	0.3095	0.578194	0.0102	0.0050642106
ss86336244		10	95.46036	0.2178	0.4011	0.526741	0.0140	0.0047607222
ss86295317		10	95.626541	0.6884	10.6209	0.001173	0.0640	0.0274510636
ss86294530		10	95.64966	0.6870	0.1660	0.683834	0.0080	0.0034300026
ss117964132		10	95.691959	0.8732	7.3336	0.006934	0.0746	0.0165270913
ss65568949	*rs43654376*	10	95.712417	0.0860	0.9224	0.337191	0.0319	0.0050103056
ss65568991	*rs43654418*	10	95.733848	0.2908	0.0760	0.782864	0.0057	0.0023383040
ss86304976		10	95.835925	0.3825	7.1224	0.007790	0.0512	0.0242019722
ss86320688		10	95.86802	0.1132	1.0760	0.299952	0.0315	0.0063218916
ss61510899	*rs41607819*	10	95.898413	0.6504	8.7697	0.003167	0.0585	0.0265922357
ss65569163	*rs43646591*	10	95.93585	0.9463	3.5098	0.061426	0.0772	0.0078486039
ss61560085	*rs41655610*	10	95.961003	0.3152	1.7088	0.191575	0.0264	0.0113998864
ss65569269	*rs43647597*	10	96.012492	0.3560	4.7652	0.029375	0.0413	0.0189572610
ss117970046		10	96.074574	0.1375	1.4503	0.228885	0.0324	0.0076799550
ss65569319	*rs43648647*	10	96.098495	0.4878	10.0908	0.001556	0.0586	0.0292592364
ss86284361		10	96.126812	0.9427	0.7234	0.395338	0.0347	0.0037448898
ss61524222	*rs41619179*	10	96.272792	0.3095	1.4543	0.228247	0.0242	0.0103499831
ss64681181	*rs42779730*	10	96.353946	0.9026	16.3620	0.000058	0.1236	0.0217396402
ss61551317	*rs41649495*	10	96.424183	0.1332	8.6674	0.003347	0.0786	0.0181527429
ss61528136	*rs41630175*	10	96.474579	0.5752	0.0674	0.795213	0.0048	0.0023468651

ss61475678	*rs41576649*	10	96.508075	0.5115	0.0061	0.937931	0.0014	0.0007218928
ss86339094		10	96.556741	0.2543	6.7369	0.009643	0.0538	0.0203985466
	rs29025348	10	96.582302	0.1712	2.1950	0.138916	0.0355	0.0100797073
ss64020750	*rs42431785*	10	96.659357	0.7385	0.1497	0.698898	0.0079	0.0030554337
ss86341773		10	96.706792	0.3868	0.0051	0.942876	0.0014	0.0006443601
ss61483743	*rs41581008*	10	96.768489	0.6074	10.1690	0.001492	0.0583	0.0278203115
ss105241680		10	96.807326	0.2736	2.8610	0.091201	0.0339	0.0134742356
ss86297583		10	96.912983	0.2858	6.0421	0.014211	0.0487	0.0198761904
ss61527723	*rs41625970*	10	96.954908	0.2650	2.2413	0.134820	0.0315	0.0122696561
ss61477106	*rs41572236*	10	96.988225	0.3309	2.0642	0.151243	0.0277	0.0122686406
ss61492639	*rs41588889*	10	97.115277	0.6318	0.0803	0.777042	0.0055	0.0025450580
ss61560079	*rs41655604*	10	97.143919	0.2120	3.7425	0.053451	0.0428	0.0143112318
ss64021563	*rs42435292*	10	97.25786	0.1669	1.5598	0.212121	0.0305	0.0084946355
ss61560080	*rs41655605*	10	97.279267	0.7042	12.8335	0.000364	0.0702	0.0292550652
ss64986634	*rs43081611*	10	97.308023	0.5337	10.0021	0.001632	0.0582	0.0289795672
ss64842387	*rs42936372*	10	97.369949	0.6741	0.0912	0.762725	0.0061	0.0026697102
ss86325494		10	97.402373	0.8453	5.0328	0.025185	0.0565	0.0147894964
ss117974843		10	97.468389	0.2092	5.0741	0.024596	0.0508	0.0168110977
ss63657345	*rs42299618*	10	97.500099	0.7865	2.8226	0.093393	0.0378	0.0127035569
ss63657414	*rs42300841*	10	97.548922	0.6941	6.2094	0.012939	0.0519	0.0220340246
ss63657552	*rs42300887*	10	97.641437	0.9298	0.0141	0.905607	0.0044	0.0005722518
ss63658221	*rs42303110*	10	97.730007	0.1182	1.2138	0.270960	0.0313	0.0065292801
ss61505767	*rs41608897*	10	98.006211	0.6834	0.6919	0.405801	0.0165	0.0071513427
ss61510952	*rs41609481*	10	98.199153	0.7615	2.6532	0.103796	0.0347	0.0126217555
	rs29021072	10	98.253404	0.3524	2.2135	0.137264	0.0286	0.0130551199
ss86286470		10	98.360284	0.7328	3.6837	0.055354	0.0399	0.0156194578
ss86322224		10	98.390838	0.4312	5.3931	0.020504	0.0426	0.0209005512
ss61477388	*rs41575925*	10	98.433938	0.4305	4.7577	0.029501	0.0400	0.0196172395
ss61492911		10	98.466954	0.1655	0.1106	0.739558	0.0083	0.0023000712
ss86326349		10	98.487976	0.0852	0.1601	0.689145	0.0127	0.0019864239
ss86299241		10	98.522284	0.5523	1.5385	0.215259	0.0237	0.0117178614
ss86315859		10	98.592317	0.6468	3.7796	0.052284	0.0387	0.0176675226
ss117969979		10	98.645007	0.3789	2.1449	0.143497	0.0285	0.0134195303
		10	98.805318	0.5860	1.5607	0.211985	0.0240	0.0116624909
ss86336431		10	98.901861	0.4821	0.2792	0.597370	0.0096	0.0048008164
ss61492906	*rs41590854*	10	98.939889	0.1812	2.5749	0.109026	0.0371	0.0110145072
ss86312014		10	99.175221	0.7077	0.4486	0.503231	0.0140	0.0057833479
ss86322348		10	99.207662	0.0616	4.8802	0.027491	0.0838	0.0096901054
ss61560659	*rs41660376*	10	99.237013	0.2872	1.2048	0.272746	0.0224	0.0091545748
ss61560654	*rs41660371*	10	99.282331	0.0638	1.3496	0.245746	0.0421	0.0050271534
ss61560650	*rs41660367*	10	99.389871	0.1239	2.5463	0.111004	0.0446	0.0096740622
ss86319293		10	99.441991	0.8302	6.7138	0.009767	0.0633	0.0178484121
ss61560648	*rs41660365*	10	99.605138	0.1719	15.8621	0.000075	0.0952	0.0271018688
	rs29018360	10	99.641254	0.3918	3.2504	0.071837	0.0344	0.0163795321
ss86301724		10	99.747559	0.1934	0.1853	0.667017	0.0099	0.0030787623
ss61492652	*rs41588902*	10	99.768906	0.1454	15.4099	0.000095	0.1001	0.0248780352
ss117969989		10	99.805527	0.1289	0.0032	0.955125	0.0016	0.0003538832

ss86312151		10	99.831879	0.8367	0.4893	0.484483	0.0178	0.0048542694
ss117969778		10	99.86905	0.8360	6.3373	0.012046	0.0625	0.0171376553
ss86310828		10	99.916214	0.3102	0.8382	0.360219	0.0181	0.0077499697
	rs29010356	10	99.937681	0.1404	1.1621	0.281398	0.0291	0.0070189018
ss86321605		10	99.983403	0.2228	1.2527	0.263426	0.0252	0.0087257748
ss86276808		10	100.003543	0.1017	4.3882	0.036549	0.0642	0.0117262654
ss86317283		10	100.162787	0.8381	0.0088	0.925376	0.0024	0.0006421831
ss65569360	*rs43648688*	10	100.196039	0.8854	3.6959	0.054953	0.0555	0.0112599841
	rs29027092	10	100.255326	0.1640	0.0024	0.960947	0.0012	0.0003385682
ss61498443	*rs43712043*	10	100.286297	0.3152	4.6038	0.032246	0.0431	0.0186045918
ss86303711		10	100.301949	0.7493	0.2684	0.604599	0.0111	0.0041843147
ss86307610		10	100.336054	0.3517	3.3793	0.066446	0.0351	0.0160273015
ss86291453		10	100.360261	0.2292	4.8048	0.028711	0.0483	0.0170791847
ss65570169	*rs43647091*	10	100.439661	0.5007	0.2789	0.597567	0.0097	0.0048652239
ss117969937		10	100.478954	0.3947	3.1636	0.075732	0.0326	0.0155772033
ss86328652		10	100.591812	0.7206	0.6990	0.403423	0.0173	0.0069581182
ss63373999	*rs42212734*	10	100.624257	0.4563	0.0361	0.849397	0.0035	0.0017328591
ss86330776		10	100.797646	0.4377	0.0133	0.908282	0.0023	0.0011093342
ss61522019	*rs41623012*	10	100.86106	0.2787	3.7665	0.052694	0.0403	0.0162210603
ss64664120	*rs42766898*	10	100.89282	0.6433	0.2258	0.634836	0.0092	0.0042198771
ss86337127		10	100.923319	0.0888	1.7272	0.189202	0.0423	0.0068550060
ss86337032		10	100.971009	0.6325	0.3500	0.554303	0.0115	0.0053273188
ss64982054	*rs43072803*	10	101.053742	0.6325	0.6107	0.434808	0.0151	0.0070368109
ss86337631		10	101.116495	0.6841	1.8323	0.176297	0.0274	0.0118238498
ss62952582	*rs42048867*	10	101.153939	0.0845	0.1119	0.738070	0.0111	0.0017113178
ss63909108	*rs42124630*	10	101.225393	0.1855	0.1558	0.693195	0.0094	0.0028389030
ss86336465		10	101.257707	0.5229	0.0624	0.802819	0.0047	0.0023391418
ss86339465		10	101.293305	0.2822	2.3952	0.122160	0.0331	0.0134026121
ss61469498	*rs41570400*	10	101.34126	0.2894	0.0206	0.886030	0.0030	0.0012194107
ss86297055		10	101.378011	0.4062	2.8327	0.092814	0.0309	0.0149286489
ss64193913	*rs42519066*	10	101.416575	0.3761	0.0452	0.831669	0.0041	0.0019138886
ss61494410	*rs41596710*	10	101.624833	0.7715	2.3197	0.128199	0.0357	0.0125760438
ss64193359	*rs42517389*	10	101.656357	0.2006	1.3422	0.247036	0.0281	0.0089954550
ss86336658		10	101.785501	0.9255	0.1322	0.716256	0.0130	0.0017893895
ss86340824		10	101.885154	0.3016	1.5180	0.218335	0.0245	0.0103063308
ss61504629	*rs41608502*	10	101.924522	0.3138	0.0083	0.927320	0.0018	0.0007793104
ss86293483		10	101.94586	0.1991	0.9164	0.338767	0.0223	0.0071070154
ss65570524	*rs43651346*	10	102.042845	0.9534	0.0072	0.932414	0.0038	0.0003364787
ss64905852	*rs42999157*	10	102.07088	0.6526	0.0154	0.901292	0.0024	0.0010972264
ss86288053		10	102.192005	0.2099	0.3593	0.549106	0.0137	0.0045350089
ss65570637	*rs43651959*	10	102.223176	0.2507	0.2765	0.599170	0.0113	0.0042311265
ss86310544		10	102.247446	0.2235	0.2468	0.619477	0.0111	0.0038592326
ss86324033		10	102.299779	0.8596	3.3984	0.065685	0.0493	0.0118920001
ss86282290		10	102.383091	0.1576	1.2197	0.269802	0.0283	0.0075088380
ss86336102		10	102.406528	0.1297	1.1882	0.276073	0.0295	0.0066568297
	rs29025229	10	102.438585	0.5946	0.4441	0.505361	0.0129	0.0062104487
ss86303286		10	102.465276	0.1175	2.4136	0.120742	0.0434	0.0089960098

ss64531436	*rs42684287*	10	102.506683	0.6461	1.5956	0.206955		0.0251	0.0114637031
ss61514302	*rs41610675*	10	102.536224	0.2149	5.0806	0.024505		0.0496	0.0167337275
ss86337550		10	102.563531	0.2765	0.3369	0.561797		0.0119	0.0047624791
ss105242362		10	102.605776	0.1404	0.7052	0.401323		0.0217	0.0052348996
ss86303918		10	102.636462	0.9506	0.0976	0.754820		0.0128	0.0012043597
ss86339766		10	102.664785	0.1003	0.0777	0.780553		0.0086	0.0015430201
ss86313616		10	102.815899	0.4821	0.0217	0.882993		0.0027	0.0013466767
ss61527613	*rs41625069*	10	102.832815	0.4327	0.1854	0.666909		0.0080	0.0039484358
ss64015860	*rs42438348*	10	102.879046	0.1447	0.7324	0.392414		0.0223	0.0055191488
ss86341531		10	102.907129	0.6340	3.4753	0.062711		0.0366	0.0170082582
	rs29014597	10	102.927416	0.2335	2.4744	0.116167		0.0343	0.0122901746
ss86314089		10	103.01575	0.3890	4.3309	0.037791		0.0399	0.0189654180
ss86286986		10	103.099795	0.5308	0.5263	0.468422		0.0133	0.0066061609
ss86284763		10	103.13218	0.6497	1.3084	0.253083		0.0214	0.0097308986
ss86297548		10	103.334744	0.8324	1.6141	0.204338		0.0315	0.0088029833
ss64469182	*rs42653242*	10	103.431533	0.6225	0.9952	0.318812		0.0192	0.0090230446
ss86307408		10	103.452965	0.8209	1.1285	0.288460		0.0254	0.0074775641
ss86315765		10	103.499301	0.8338	1.5944	0.207128		0.0311	0.0086220563
ss86273956		10	103.593999	0.1812	1.6200	0.203513		0.0301	0.0089360053
ss86290667		10	103.631065	0.6318	0.2891	0.590951		0.0104	0.0048362504
ss86336672		10	103.657962	0.6741	3.4618	0.063225		0.0355	0.0155957563
ss86301214		10	103.700637	0.2665	0.0319	0.858299		0.0037	0.0014372894
ss86331955		10	103.736759	0.3660	3.2509	0.071818		0.0339	0.0157538435
ss86314269		10	103.768639	0.7085	2.2620	0.133037		0.0305	0.0126191138
ss65571295	*rs43649216*	10	103.795346	0.3259	0.0642	0.800001		0.0052	0.0022982427
ss86293851		10	103.826012	0.7042	0.1817	0.670018		0.0090	0.0037479545
ss86304744		10	103.857069	0.7607	0.4725	0.492086		0.0146	0.0053183303
ss86330820		10	103.898867	0.7120	5.3435	0.021090		0.0468	0.0192024979
ss117969990		10	103.936504	0.1734	0.0312	0.859793		0.0043	0.0012283121
ss86328055		10	103.966659	0.5838	1.1404	0.285937		0.0199	0.0096832923
ss86305855		10	104.004851	0.3195	0.4763	0.490311		0.0136	0.0059208186
ss86286212		10	104.033163	0.3381	0.1902	0.662891		0.0084	0.0037587585
ss86286061		10	104.096749	0.0838	0.0724	0.787997		0.0092	0.0014115799
ss86294708		10	104.123602	0.5387	10.8774	0.001023		0.0614	0.0305051103
ss86294721		10	104.193802	0.3252	17.7551	0.000028		0.0818	0.0359056937
ss86288015		10	104.268729	0.5050	6.2933	0.012346		0.0469	0.0234279974
ss61497473	*rs41596899*	10	104.290716	0.5093	4.8289	0.028316		0.0413	0.0206648188
ss117964130		10	104.346553	0.6024	15.1768	0.000107		0.0732	0.0350449264
ss86332579		10	104.387748	0.5251	14.3711	0.000163		0.0677	0.0337761888
ss61543694	*rs41635906*	10	104.428997	0.2113	10.5380	0.001225		0.0705	0.0235063440
ss86287287		10	104.483727	0.6848	0.2224	0.637332		0.0093	0.0040121502
ss86275061		10	104.526696	0.4133	2.1929	0.139103		0.0274	0.0132814855
ss86300818		10	104.556277	0.1977	1.2721	0.259769		0.0254	0.0080592342
ss86341211		10	104.664073	0.2564	4.4053	0.036187		0.0455	0.0173532219
ss61561192	*rs41657604*	10	104.690021	0.4269	10.7375	0.001102		0.0640	0.0313173781
ss86339505		10	104.74093	0.5236	0.3701	0.543128		0.0111	0.0055362460
ss86278818		10	104.777075	0.6117	1.0734	0.300543		0.0194	0.0092157085

ss86337741		10	104.801606	0.1504	0.4701	0.493192	0.0171	0.0043769064
ss86339769		10	104.831424	0.3309	0.0057	0.939979	0.0015	0.0006513600
ss86325563		10	104.86889	0.3059	1.3230	0.250456	0.0228	0.0096757793
ss61521142	*rs41617562*	10	104.889086	0.4319	0.1811	0.670587	0.0077	0.0037818057
ss61521149	*rs41617569*	10	104.917015	0.5072	0.1570	0.692042	0.0073	0.0036608588
ss61563521	*rs41661892*	10	104.951351	0.3087	1.9823	0.159598	0.0280	0.0119350814
ss61493185	*rs41592730*	10	105.001242	0.6941	0.3691	0.543671	0.0119	0.0050593757
ss105241670		10	105.026522	0.1390	0.0198	0.888033	0.0037	0.0008823888
ss86330536		10	105.070157	0.2758	0.0017	0.967474	0.0008	0.0003313161
ss86331713		10	105.100369	0.3926	0.0338	0.854276	0.0034	0.0016024006
ss61493183	*rs41592728*	10	105.126573	0.5279	3.2240	0.073001	0.0329	0.0163746294
ss86340809		10	105.155473	0.5809	0.0001	0.991427	0.0002	0.0000970376
ss61493181	*rs41592726*	10	105.213237	0.1547	3.0539	0.080984	0.0453	0.0118609081
	rs29016931	10	105.258956	0.9069	1.1539	0.283116	0.0343	0.0057976883
ss86302429		10	105.299546	0.7801	0.4003	0.527129	0.0138	0.0047398059
ss86335937		10	105.361376	0.5437	1.1742	0.278920	0.0202	0.0100084858
ss86305708		10	105.451059	0.5050	1.4753	0.224926	0.0226	0.0112961492
ss61529434	*rs41625155*	10	105.58094	0.6017	1.0440	0.307246	0.0191	0.0091444588
ss86340719		10	105.651922	0.4527	7.9240	0.005016	0.0533	0.0264192727
ss86327576		10	105.681005	0.5444	8.2875	0.004114	0.0544	0.0270023998
ss86324886		10	105.712585	0.6662	4.0556	0.044409	0.0394	0.0175251215
ss86340578		10	105.762612	0.9334	3.0569	0.080835	0.0622	0.0077412028
ss61496632	*rs41591285*	10	105.848237	0.4255	13.5993	0.000244	0.0699	0.0341507876
ss38327277	*rs29019387*	10	105.88855	0.2142	2.1408	0.143878	0.0323	0.0108693194
ss61568182	*rs41662084*	10	105.950061	0.4255	8.6503	0.003379	0.0554	0.0270996832
ss86307384		10	106.019273	0.1311	0.2250	0.635396	0.0128	0.0029235701
ss86340925		10	106.090524	0.2092	8.0418	0.004704	0.0655	0.0216740627
ss65571534	*rs43651550*	10	106.125176	0.3789	0.8046	0.370034	0.0169	0.0079346522
ss61556339	*rs41653546*	10	106.203563	0.3983	3.0450	0.081430	0.0326	0.0156189928
ss61525531	*rs41621743*	11	0.0206	0.4097	1.6018	0.206077	0.0230	0.0111027229
ss64586218	*rs42712231*	11	0.16233	0.2149	0.0450	0.832051	0.0048	0.0016164461
ss61515335	*rs41613691*	11	0.202058	0.9040	2.5555	0.110367	0.0513	0.0089056418
ss86329308		11	0.240355	0.6053	0.0608	0.805366	0.0047	0.0022505478
ss86322043		11	0.268271	0.6683	0.3387	0.560784	0.0114	0.0050722159
ss61469631	*rs41571228*	11	0.335035	0.4133	0.4359	0.509323	0.0122	0.0059252805
ss61494533	*rs41597718*	11	0.368451	0.2507	0.0055	0.940813	0.0016	0.0005851500
ss86294195		11	0.390467	0.3238	0.2857	0.593142	0.0104	0.0045492623
ss86313597		11	0.676958	0.1254	0.4895	0.484384	0.0193	0.0042375300
ss86305285		11	0.771677	0.4907	1.6934	0.193586	0.0235	0.0117707709
ss86287648		11	0.846983	0.5301	0.1244	0.724440	0.0068	0.0033711734
ss86336751		11	0.882225	0.5638	0.6845	0.408329	0.0159	0.0078403105
ss64718939	*rs42817664*	11	0.903751	0.2801	2.3501	0.125731	0.0313	0.0126313653
ss117975262		11	0.957816	0.2307	0.0608	0.805380	0.0053	0.0018863914
ss86339903		11	0.981671	0.5867	0.0122	0.912012	0.0021	0.0010180459
ss86295199		11	1.036645	0.2858	0.3109	0.577297	0.0118	0.0048002435
ss86293432		11	1.083222	0.3517	0.4863	0.485831	0.0138	0.0062938739
ss86291167		11	1.125724	0.9234	0.0078	0.929610	0.0030	0.0004293292

ss86335805		11	1.146342	0.5559	0.3205	0.571489	0.0104	0.0051137929
ss86340367		11	1.175664	0.1454	0.5958	0.440449	0.0199	0.0049498578
ss86287660		11	1.196277	0.4778	0.3688	0.543834	0.0109	0.0054167170
ss65572671	*rs43655087*	11	1.231011	0.5401	1.1801	0.277707	0.0207	0.0102603320
ss46526829	*rs41257280*	11	1.253072	0.5867	1.1813	0.277467	0.0202	0.0097926972
ss86324631		11	1.31128	0.5917	1.8211	0.177626	0.0242	0.0116945723
ss86323219		11	1.350151	0.9105	4.5061	0.034128	0.0692	0.0112793710
ss86311825		11	1.380875	0.6232	1.6124	0.204585	0.0240	0.0112771733
ss65572961	*rs43656877*	11	1.407228	0.8044	1.3167	0.251572	0.0266	0.0083638359
ss86314572		11	1.438107	0.8589	8.1416	0.004455	0.0763	0.0185018321
ss64929717	*rs43025943*	11	1.466591	0.8317	11.1417	0.000889	0.0836	0.0234156364
	rs29018814	11	1.491025	0.1726	0.5900	0.442686	0.0180	0.0051464998
ss86297660		11	1.516598	0.2192	10.8686	0.001028	0.0737	0.0252222780
ss86328745		11	1.539115	0.2951	0.7941	0.373163	0.0181	0.0075394976
ss86317638		11	1.592829	0.0788	1.8692	0.172008	0.0471	0.0068431257
ss86313551		11	1.652279	0.1046	1.1523	0.283450	0.0327	0.0061201027
ss86340990		11	1.692828	0.5774	4.0896	0.043529	0.0371	0.0181099381
ss65571996	*rs43654202*	11	1.717017	0.0903	0.3612	0.548022	0.0192	0.0031602790
ss86329897		11	1.763763	0.7722	0.1088	0.741658	0.0072	0.0025309908
	rs29012894	11	1.801184	0.6132	1.4828	0.223750	0.0228	0.0108171686
ss61505330	*rs41604980*	11	1.958312	0.4928	6.6886	0.009905	0.0479	0.0239545814
ss86284709		11	1.985172	0.9219	2.1423	0.143735	0.0497	0.0071496091
ss86333253		11	2.01695	0.8660	1.7716	0.183620	0.0365	0.0084782840
ss86283136		11	2.054633	0.4756	11.2788	0.000827	0.0632	0.0315385836
ss86298860		11	2.119842	0.7321	0.2338	0.628908	0.0101	0.0039801032
ss86290899		11	2.19275	0.2787	4.9036	0.027123	0.0456	0.0183345909
	rs29026446	11	2.235741	0.8825	2.0275	0.154926	0.0401	0.0083142285
ss86291278		11	2.271218	0.8933	1.5741	0.210039	0.0369	0.0070267050
ss86327966		11	2.337096	0.5129	2.9095	0.088507	0.0308	0.0153994630
ss86316211		11	2.382103	0.2908	2.8457	0.092067	0.0329	0.0135549536
ss86336274		11	2.423422	0.7049	9.1400	0.002593	0.0614	0.0255522463
ss65571978	*rs43654184*	11	2.454916	0.2020	6.5841	0.010498	0.0582	0.0187758237
ss86325340		11	2.510378	0.1848	0.0599	0.806702	0.0059	0.0017691300
ss86331080		11	2.542311	0.0681	5.3492	0.021023	0.0825	0.0104591461
ss86321910		11	2.574735	0.2579	2.8213	0.093473	0.0348	0.0133337413
ss117970408		11	2.603117	0.4155	8.0334	0.004725	0.0532	0.0258637706
ss86301289		11	2.641488	0.6497	5.8020	0.016267	0.0473	0.0215142688
ss86318522		11	2.664511	0.3331	1.0440	0.307255	0.0197	0.0087569969
ss61563466	*rs41661838*	11	2.685772	0.2987	2.6453	0.104307	0.0322	0.0134954880
ss86309265		11	2.772561	0.5516	1.5618	0.211818	0.0228	0.0112853551
ss86287385		11	2.857527	0.5401	0.0001	0.992692	0.0002	0.0000856465
ss65576110	*rs43664272*	11	2.896523	0.1927	1.6681	0.196936	0.0299	0.0092889717
ss86336284		11	2.917266	0.6633	0.0104	0.918719	0.0020	0.0009028716
ss86299961		11	2.950999	0.4577	3.9025	0.048609	0.0378	0.0187574330
ss86317960		11	2.971556	0.5451	3.6265	0.057277	0.0363	0.0180240725
ss86334496		11	3.030075	0.5315	3.1548	0.076142	0.0339	0.0168813452
	rs29012894	11	3.076275	0.6103	1.3874	0.239249	0.0221	0.0105250094

ss86297909		11	3.112976	0.0824	0.0023	0.962016	0.0016	0.0002360503
ss86274552		11	3.162947	0.6540	0.3207	0.571355	0.0107	0.0048264721
ss61505549	*rs41607186*	11	3.194197	0.8610	1.2467	0.264578	0.0298	0.0071318271
ss117964169		11	3.219717	0.7872	0.0251	0.874066	0.0034	0.0011329840
ss105238820		11	3.25578	0.1963	0.0072	0.932484	0.0019	0.0005938123
ss65575387	*rs43656077*	11	3.298308	0.8009	0.0136	0.907276	0.0026	0.0008171993
ss86287226		11	3.331606	0.6454	0.0003	0.986917	0.0003	0.0001390923
ss86288875		11	3.358877	0.3331	0.1098	0.740444	0.0062	0.0027617505
ss86273766		11	3.400222	0.3252	7.9841	0.004854	0.0562	0.0246753504
ss64840648	*rs42935030*	11	3.507794	0.8754	0.1004	0.751493	0.0089	0.0019368730
ss64840624	*rs42935006*	11	3.530292	0.8947	0.8576	0.354725	0.0279	0.0052586316
ss65575084	*rs43662878*	11	3.553386	0.8904	1.8328	0.176239	0.0403	0.0078569436
ss61495757	*rs41592424*	11	3.612596	0.4807	4.8649	0.027735	0.0411	0.0205121156
ss86319682		11	3.654279	0.6404	1.8299	0.176582	0.0260	0.0119832457
ss61566343	*rs41663284*	11	3.679855	0.4893	10.2152	0.001456	0.0591	0.0295490513
ss61495741	*rs41592408*	11	3.718518	0.1669	1.0793	0.299210	0.0258	0.0071834821
	rs29011691	11	3.759732	0.6999	4.9151	0.026945	0.0445	0.0186763244
ss86325729		11	3.7857	0.2371	0.6596	0.416971	0.0175	0.0063343509
ss86338549		11	3.826529	0.4491	1.6728	0.196313	0.0246	0.0121714256
ss86320477		11	3.865631	0.5372	4.5751	0.032786	0.0396	0.0197034864
ss86325740		11	3.887405	0.7572	1.0690	0.301533	0.0218	0.0080348070
ss61569567	*rs41668642*	11	3.926168	0.6862	1.9590	0.162064	0.0275	0.0118303044
ss86284294		11	3.962661	0.5100	8.7443	0.003211	0.0546	0.0272701737
ss65576591	*rs43656950*	11	4.04362	0.8560	0.9781	0.323014	0.0254	0.0062614967
ss86299709		11	4.072986	0.8245	0.0196	0.888612	0.0034	0.0009792037
ss105240328		11	4.110774	0.4635	0.9372	0.333335	0.0175	0.0087157053
ss65576342	*rs43654602*	11	4.152322	0.4821	0.0295	0.863635	0.0031	0.0015571388
ss86338698		11	4.201594	0.9097	0.0142	0.905277	0.0038	0.0006265848
ss86287229		11	4.232631	0.6483	19.1285	0.000014	0.0812	0.0370089719
ss86293639		11	4.59233	0.7794	1.0225	0.312285	0.0223	0.0076668720
ss64741199	*rs42835776*	11	4.63788	0.8897	2.1962	0.138805	0.0447	0.0087707104
ss38331259	*rs29025373*	11	4.684785	0.1103	2.1962	0.138805	0.0447	0.0087707104
ss65573262	*rs43653183*	11	4.743546	0.8904	1.8910	0.169527	0.0416	0.0081113158
ss86340481		11	4.761353	0.2557	10.3220	0.001375	0.0662	0.0251927492
ss86287987		11	4.84792	0.5774	6.1658	0.013259	0.0458	0.0223667655
ss105236796		11	4.881649	0.5888	4.1140	0.042910	0.0376	0.0182174304
ss117964174		11	4.911851	0.3116	0.2163	0.642014	0.0093	0.0039966680
ss61564376	*rs41660547*	11	4.950332	0.3446	0.3021	0.582747	0.0106	0.0047681202
ss63570895	*rs42281160*	11	4.99851	0.3446	1.2414	0.265586	0.0213	0.0096324881
ss86299832		11	5.02548	0.2042	0.0879	0.766942	0.0066	0.0021589110
ss63571096	*rs42281227*	11	5.067019	0.2915	10.0368	0.001602	0.0618	0.0255246234
ss86282707		11	5.091274	0.8324	3.8319	0.050684	0.0484	0.0135015145
ss63571507	*rs42283863*	11	5.112304	0.1404	0.1682	0.681853	0.0112	0.0027015782
ss86299996		11	5.154817	0.1132	0.3620	0.547602	0.0179	0.0035865043
	rs29026129	11	5.205393	0.4842	3.2295	0.072758	0.0328	0.0164071162
ss86307142		11	5.23979	0.5086	11.9374	0.000584	0.0619	0.0309270006
ss63571936	*rs42285002*	11	5.273639	0.5451	4.9846	0.025892	0.0409	0.0202937954

ss86337355		11	5.296683	0.5852	4.6947	0.030595	0.0391	0.0189696059
ss86298915		11	5.396347	0.4785	0.6014	0.438297	0.0138	0.0068957218
ss63572305	*rs42274430*	11	5.422469	0.0924	12.7248	0.000386	0.1134	0.0190193709
ss63572398	*rs42275460*	11	5.461016	0.5222	2.4066	0.121277	0.0280	0.0139747582
ss61470389		11	5.484265	0.2658	0.2358	0.627431	0.0101	0.0039439625
ss86323133		11	5.548734	0.9405	1.3338	0.248530	0.0440	0.0049247907
ss86305804		11	5.587819	0.3195	2.5981	0.107445	0.0313	0.0136314864
ss61512142	*rs41610452*	11	5.636509	0.5967	2.0417	0.153491	0.0258	0.0124254001
ss86278343		11	5.660631	0.0831	29.1734	0.000000	0.1746	0.0266002855
ss86338519		11	5.713394	0.1046	1.2006	0.273586	0.0341	0.0063960205
ss86324226		11	5.76767	0.3854	6.8005	0.009308	0.0482	0.0228377289
ss86284588		11	5.803588	0.1705	9.7624	0.001855	0.0749	0.0211966678
ss86340125		11	5.880385	0.1805	0.5496	0.458736	0.0170	0.0050411417
ss65467301	*rs43547057*	11	5.963879	0.1067	16.4782	0.000055	0.1206	0.0229871906
ss86293957		11	5.984654	0.4405	6.9771	0.008441	0.0478	0.0235845060
ss86293617		11	6.016006	0.1877	0.5157	0.472916	0.0166	0.0050725070
ss86283835		11	6.075539	0.4198	4.2649	0.039278	0.0386	0.0187846810
ss61492579	*rs41588330*	11	6.110256	0.2063	0.8488	0.357201	0.0206	0.0067387381
ss65467659	*rs43549615*	11	6.175842	0.2006	0.8728	0.350493	0.0208	0.0066849124
ss86320976		11	6.208887	0.6504	0.3790	0.538357	0.0117	0.0053389864
ss61492580	*rs41588831*	11	6.27938	0.0931	0.0190	0.890342	0.0045	0.0007570986
ss86339643		11	6.300385	0.2285	2.0368	0.153976	0.0308	0.0108550648
ss86291844		11	6.333026	0.9262	0.1427	0.705750	0.0131	0.0017950791
ss86298113		11	6.380896	0.0731	0.1123	0.737688	0.0117	0.0015836007
ss86293363		11	6.45362	0.3474	0.1481	0.700435	0.0075	0.0034037052
ss65577308	*rs43655669*	11	6.515926	0.7357	0.8802	0.348470	0.0194	0.0075334401
ss64932897	*rs43032739*	11	6.553191	0.7579	4.2293	0.040105	0.0428	0.0157127359
ss86311910		11	6.671265	0.4642	4.3707	0.036924	0.0378	0.0187858173
ss86315613		11	6.725193	0.6254	0.0810	0.775968	0.0052	0.0024595074
ss86307738		11	6.846118	0.3761	0.0094	0.922788	0.0018	0.0008425246
	rs29010480	11	6.879569	0.8066	0.9323	0.334611	0.0221	0.0069000443
ss86321402		11	6.902569	0.4577	7.7522	0.005511	0.0500	0.0248069858
ss86338360		11	6.93867	0.7063	1.5695	0.210703	0.0257	0.0106564947
	rs29011405	11	6.982325	0.1511	17.3625	0.000035	0.1090	0.0279654107
ss65579948	*rs43664102*	11	7.051915	0.0716	0.0049	0.944447	0.0026	0.0003403394
ss86278104		11	7.134105	0.5365	3.7362	0.053651	0.0362	0.0180100047
ss86285483		11	7.18943	0.1504	1.5497	0.213599	0.0324	0.0082805693
ss86330665		11	7.231923	0.6168	5.6244	0.017983	0.0445	0.0210330991
ss105298210		11	7.36152	0.1791	1.1747	0.278820	0.0258	0.0075935527
ss65580173	*rs43657533*	11	7.414806	0.2586	1.8655	0.172434	0.0290	0.0111142615
ss86327253		11	7.469584	0.9477	1.0478	0.306360	0.0434	0.0043050901
ss65580253	*rs43657613*	11	7.499766	0.8976	2.5701	0.109353	0.0483	0.0088881248
ss86288388		11	7.520802	0.2729	0.3373	0.561600	0.0119	0.0047196131
ss61470396	*rs41569383*	11	7.544713	0.1017	1.4893	0.222746	0.0366	0.0066874021
ss86288889		11	7.568143	0.5723	2.6605	0.103322	0.0297	0.0145178746
ss86319431		11	7.605292	0.6089	1.4419	0.230242	0.0227	0.0108047239
ss61470401	*rs41569388*	11	7.656112	0.8933	6.2385	0.012730	0.0770	0.0146881162

ss86341521		11	7.704237	0.5867	1.0907	0.296676		0.0190	0.0092195068
ss86321738		11	7.731138	0.3868	1.8171	0.178102		0.0255	0.0120832722
ss86285400		11	7.761851	0.5072	1.5494	0.213636		0.0233	0.0116305577
ss86327207		11	7.783086	0.5007	5.6993	0.017237		0.0441	0.0220648608
ss86305161		11	7.809159	0.5401	0.6387	0.424446		0.0149	0.0074101450
ss61513148	*rs41610542*	11	7.848786	0.7923	3.5771	0.058995		0.0442	0.0145576495
ss65578012	*rs43661073*	11	7.894895	0.1533	4.3846	0.036626		0.0527	0.0136781590
ss65578095	*rs43661856*	11	7.92065	0.6139	4.1666	0.041606		0.0378	0.0179197248
ss61513622		11	7.985968	0.2292	3.2010	0.074026		0.0400	0.0141419651
ss86293996		11	8.008861	0.2579	1.8163	0.178188		0.0291	0.0111532783
ss61471002	*rs41566575*	11	8.035455	0.9284	0.7906	0.374213		0.0323	0.0042932309
ss61514167	*rs41610340*	11	8.121156	0.4456	0.4593	0.498185		0.0122	0.0060439237
	rs29020725	11	8.202167	0.8023	14.4728	0.000155		0.0881	0.0279545492
ss65578468	*rs43657228*	11	8.22957	0.3746	9.2736	0.002412		0.0580	0.0271575027
ss65578489	*rs43657649*	11	8.262881	0.4097	4.6980	0.030535		0.0403	0.0195097252
ss105236867		11	8.375197	0.5050	2.0621	0.151454		0.0270	0.0134838451
ss86339955		11	8.411098	0.4921	1.7844	0.182049		0.0257	0.0128465135
ss117970066		11	8.446008	0.8202	1.0150	0.314049		0.0245	0.0072357346
	rs29018155	11	8.509846	0.1354	1.0661	0.302187		0.0267	0.0062512102
ss105240375		11	8.570825	0.0552	3.2907	0.070105		0.0751	0.0078233763
ss86304536		11	8.628531	0.1411	9.2983	0.002381		0.0833	0.0201969108
ss86311118		11	8.652312	0.8897	5.2971	0.021655		0.0694	0.0136132610
ss86293692		11	8.755196	0.8868	0.3405	0.559707		0.0166	0.0033341173
ss61515733	*rs41616683*	11	8.780769	0.4370	7.1273	0.007769		0.0490	0.0241321784
ss65579058	*rs43660816*	11	8.80241	0.5989	4.4066	0.036159		0.0393	0.0188916239
ss86304469		11	8.826653	0.7364	3.7842	0.052140		0.0402	0.0156197337
ss86313757		11	8.85061	0.2650	4.3175	0.038089		0.0430	0.0167399887
ss61516316	*rs41614732*	11	8.948513	0.9441	2.9070	0.088640		0.0682	0.0072004332
ss86324014		11	8.998918	0.2271	0.2784	0.597924		0.0115	0.0040527990
ss61506347	*rs41605361*	11	9.105655	0.0931	0.6031	0.437648		0.0248	0.0041890792
ss64711061	*rs42803460*	11	9.158648	0.7751	5.2869	0.021782		0.0509	0.0177534715
ss86337800		11	9.180511	0.1282	0.0310	0.860181		0.0050	0.0011107233
ss86329346		11	9.287086	0.7514	6.3787	0.011771		0.0539	0.0201270646
ss86340355		11	9.356784	0.1383	1.6287	0.202310		0.0339	0.0080744749
ss86292007		11	9.401149	0.2701	0.5783	0.447219		0.0161	0.0063479723
ss86318844		11	9.430781	0.3338	0.6657	0.414819		0.0159	0.0070853261
ss61472833	*rs41574552*	11	9.470047	0.1390	0.0024	0.960691		0.0013	0.0003154293
ss86287582		11	9.560886	0.8266	0.4212	0.516540		0.0165	0.0047224036
ss86314231		11	9.60264	0.8854	0.0102	0.919406		0.0030	0.0005988832
ss86284747		11	9.636031	0.2643	4.5946	0.032419		0.0445	0.0173158012
ss61565762	*rs41663009*	11	9.657831	0.2464	2.4306	0.119440		0.0326	0.0121137640
ss64847544	*rs42940368*	11	9.691078	0.2629	3.5781	0.058961		0.0387	0.0149843959
ss65579833	*rs43663287*	11	9.726128	0.1719	9.1608	0.002564		0.0739	0.0210531416
ss65579919	*rs43664073*	11	9.749716	0.5330	2.6039	0.107053		0.0307	0.0152728469
ss86319759		11	9.797446	0.4928	5.3141	0.021447		0.0440	0.0220200060
ss86294152		11	9.82671	0.4011	5.8126	0.016170		0.0471	0.0226396442
ss65580824	*rs43663375*	11	9.900263	0.6726	0.0794	0.778198		0.0056	0.0024654640

ss61522360	*rs41619335*	11	9.936325	0.4642	8.0462	0.004692	0.0537	0.0267204540
ss61474813	*rs41572605*	11	9.973721	0.5480	2.6413	0.104569	0.0302	0.0149537771
ss65580551	*rs43660709*	11	10.01427	0.4957	4.7390	0.029822	0.0406	0.0203214141
ss64537586	*rs42688135*	11	10.088098	0.5437	4.1882	0.041080	0.0372	0.0184370558
ss64537502	*rs42687393*	11	10.121735	0.6798	0.6380	0.424694	0.0162	0.0070400236
ss86323986		11	10.183419	0.2092	0.0002	0.989596	0.0003	0.0000955772
ss86334010		11	10.215898	0.8453	0.3128	0.576165	0.0139	0.0036377728
ss61569466	*rs41667846*	11	10.237051	0.7736	0.2224	0.637353	0.0102	0.0035758992
ss86323612		11	10.274579	0.1569	8.8058	0.003106	0.0776	0.0205238768
ss61466070	*rs41565832*	11	10.372842	0.8711	0.6422	0.423194	0.0227	0.0051011456
ss86316691		11	10.445763	0.6347	1.3630	0.243418	0.0228	0.0105583970
ss61520709	*rs41617432*	11	10.499433	0.6225	0.0899	0.764445	0.0058	0.0027181382
ss65580344	*rs43658503*	11	10.601925	0.8818	0.0037	0.951381	0.0017	0.0003570115
ss86319842		11	10.64945	0.7350	1.3336	0.248558	0.0246	0.0095815460
ss86338865		11	10.694754	0.1999	0.8505	0.356720	0.0209	0.0066716249
ss61521743	*rs41621842*	11	10.740146	0.4083	1.2225	0.269261	0.0213	0.0102716413
ss86280768		11	10.768536	0.4019	2.7938	0.095079	0.0321	0.0154500207
ss86340927		11	10.890327	0.3417	0.1635	0.686047	0.0080	0.0035897051
ss86336612		11	10.942529	0.6289	0.0850	0.770769	0.0057	0.0026553978
ss117970414		11	11.010845	0.7643	1.3810	0.240335	0.0255	0.0092000884
ss117964166		11	11.036102	0.7636	1.5725	0.210269	0.0272	0.0098355203
ss61475893	*rs41578359*	11	11.05675	0.2371	1.8447	0.174845	0.0294	0.0106309599
ss61525261	*rs41619290*	11	11.121631	0.9305	0.0825	0.773978	0.0103	0.0013330487
ss65581933	*rs43663587*	11	11.182798	0.1254	0.0361	0.849460	0.0053	0.0011661152
ss61525264	*rs41619293*	11	11.206103	0.8976	0.0215	0.883431	0.0045	0.0008267645
	rs29018292	11	11.304342	0.2321	0.2925	0.588817	0.0117	0.0041694376
ss61475888	*rs41578354*	11	11.327713	0.1454	1.8648	0.172511	0.0352	0.0087482576
ss61475885	*rs41578351*	11	11.407464	0.5981	2.7618	0.096990	0.0317	0.0152181659
ss61525260	*rs41619289*	11	11.43091	0.5788	4.1284	0.042548	0.0397	0.0193748793
ss117964184		11	11.463441	0.1418	8.6545	0.003371	0.0773	0.0188159169
ss65581449	*rs43660404*	11	11.538038	0.8367	7.9556	0.004930	0.0702	0.0191801442
ss86326388		11	11.68789	0.6211	3.3058	0.069466	0.0375	0.0176525296
ss86316331		11	11.70814	0.4298	0.7779	0.378075	0.0163	0.0080098208
ss61525256	*rs41619285*	11	11.781329	0.8173	4.2766	0.039010	0.0502	0.0149752444
ss46526270	*rs41255409*	11	11.835213	0.5043	3.3179	0.068958	0.0336	0.0167810893
ss86335728		11	11.858188	0.7958	0.0810	0.776028	0.0065	0.0020963828
ss86319466		11	11.878694	0.1297	0.2154	0.642700	0.0125	0.0028106292
ss86340596		11	11.90558	0.4556	3.4393	0.064084	0.0341	0.0168995368
ss86341700		11	12.013615	0.1433	0.3937	0.530549	0.0162	0.0039866827
ss61507189	*rs41603588*	11	12.086205	0.1905	1.9823	0.159597	0.0330	0.0101922855
ss65582270	*rs43660125*	11	12.107854	0.4019	0.0604	0.805953	0.0046	0.0022280823
ss65582278	*rs43661333*	11	12.136258	0.2858	9.4616	0.002180	0.0605	0.0246800346
ss64933469	*rs43024195*	11	12.189238	0.0423	2.3328	0.127129	0.0699	0.0056613702
ss65022581	*rs43115908*	11	12.225327	0.1612	5.0072	0.025558	0.0544	0.0147157134
ss61466366	*rs41568121*	11	12.27232	0.8238	6.6674	0.010023	0.0604	0.0175221424
ss64662394	*rs42759372*	11	12.429309	0.5251	0.3138	0.575520	0.0103	0.0051415835
ss105239540		11	12.46841	0.7443	1.3756	0.241245	0.0250	0.0095034566

ss61493698	*rs41589325*	11	12.667814	0.4205	0.0328	0.856360	0.0034	0.0016330746
ss65584501	*rs43666856*	11	12.699129	0.1898	10.9207	0.001000	0.0778	0.0239374612
ss86337237		11	12.763719	0.6060	6.8434	0.009090	0.0471	0.0225065817
ss86327397		11	12.7843	0.1232	13.9238	0.000206	0.1028	0.0222145961
ss86331115		11	12.860332	0.1884	8.9190	0.002921	0.0702	0.0214682624
ss86332530		11	12.885964	0.3324	6.0407	0.014222	0.0460	0.0204332281
ss86314236		11	12.911567	0.5079	2.3968	0.122042	0.0284	0.0142176966
ss65584299	*rs43664754*	11	12.940481	0.2572	0.0057	0.940003	0.0016	0.0006123777
ss86315793		11	12.974122	0.3918	0.1856	0.666742	0.0081	0.0038726481
ss86325980		11	13.011641	0.0380	1.9629	0.161652	0.0660	0.0048192068
ss61478900	*rs41578974*	11	13.035412	0.0917	0.7914	0.373986	0.0274	0.0045704354
ss86306627		11	13.057503	0.1999	0.6035	0.437523	0.0184	0.0058812797
ss86294264		11	13.170354	0.3947	16.0114	0.000070	0.0750	0.0358326829
ss61493696	*rs41589323*	11	13.241623	0.0996	1.5129	0.219117	0.0383	0.0068711580
ss86306343		11	13.29056	0.2242	7.0644	0.008043	0.0595	0.0206988880
ss86333870		11	13.429317	0.8388	0.0130	0.909131	0.0029	0.0007798007
ss117970081		11	13.451089	0.8453	0.1455	0.703025	0.0098	0.0025543605
ss65583662	*rs43664517*	11	13.519742	0.6898	0.2039	0.651699	0.0092	0.0039395660
ss61493695	*rs41589322*	11	13.54305	0.6082	2.1002	0.147735	0.0283	0.0134800275
ss86310690		11	13.72088	0.5716	9.1601	0.002565	0.0571	0.0279479813
ss105240253		11	13.741419	0.5702	8.3512	0.003974	0.0548	0.0268590396
ss86293906		11	13.780652	0.2178	10.7852	0.001074	0.0745	0.0253935099
ss86300150		11	13.812181	0.6304	0.0423	0.837189	0.0041	0.0018919961
ss61561811	*rs41664418*	11	13.83801	0.7930	13.1976	0.000301	0.0827	0.0271575064
ss65582922	*rs43667475*	11	13.871993	0.2070	13.1976	0.000301	0.0827	0.0271575064
ss61493437	*rs41594968*	11	14.004791	0.3474	0.1680	0.682000	0.0080	0.0036382423
ss86320606		11	14.044311	0.4463	0.2234	0.636600	0.0088	0.0043620828
ss86332761		11	14.065582	0.4183	1.3673	0.242671	0.0220	0.0107176585
ss86289716		11	14.104815	0.3266	1.5655	0.211275	0.0246	0.0108387473
ss86330362		11	14.140314	0.8159	1.6042	0.205734	0.0306	0.0092016086
ss86326813		11	14.164224	0.7285	2.7388	0.098391	0.0341	0.0134752594
ss86290262		11	14.190568	0.2593	0.6661	0.414678	0.0173	0.0066379444
ss86281249		11	14.215076	0.3918	0.1352	0.713163	0.0069	0.0032967854
ss65584752	*rs43668307*	11	14.310833	0.5337	0.0026	0.959092	0.0010	0.0004774576
ss61562315	*rs41659216*	11	14.359911	0.4857	0.0000	0.998456	0.0000	0.0000179371
ss117964197		11	14.380344	0.8711	0.4376	0.508517	0.0176	0.0039552771
ss86291914		11	14.406449	0.8746	0.3481	0.555356	0.0159	0.0034933651
ss86293294		11	14.454079	0.7794	0.0444	0.833089	0.0047	0.0016281588
ss64430160	*rs42631477*	11	14.478191	0.2092	0.0417	0.838261	0.0047	0.0015519144
ss61505876	*rs41600007*	11	14.499391	0.8324	0.1348	0.713577	0.0093	0.0025929297
ss105240266		11	14.543926	0.6110	0.0003	0.987358	0.0003	0.0001475622
ss62026947	*rs41733077*	11	14.666713	0.9262	0.4799	0.488701	0.0243	0.0033207085
ss86304011		11	14.740201	0.1275	0.9658	0.326061	0.0260	0.0057811653
ss61500474	*rs41598926*	11	14.762309	0.9226	0.9502	0.330015	0.0333	0.0047470472
ss105240273		11	14.803394	0.9298	0.2744	0.600544	0.0187	0.0024445815
ss105240941		11	14.841275	0.0924	0.0297	0.863287	0.0055	0.0009176366
ss61529452	*rs41625173*	11	14.870694	0.1196	0.0123	0.911604	0.0031	0.0006595858

ss61562319	*rs41659220*	11	15.115924	0.2987	5.1431	0.023645	0.0452	0.0189201914
ss61562322	*rs41659223*	11	15.42825	0.8231	0.0879	0.766993	0.0073	0.0021381705
ss86275269		11	15.485413	0.2500	15.5419	0.000089	0.0833	0.0312426267
ss86312334		11	15.566451	0.4656	2.0870	0.149011	0.0279	0.0138596633
ss65606428	*rs43686298*	11	15.626965	0.0809	0.0660	0.797307	0.0086	0.0012849215
ss65585142	*rs43672297*	11	15.648428	0.0809	0.0660	0.797307	0.0086	0.0012849215
ss86305356		11	15.689551	0.7135	10.9989	0.000959	0.0677	0.0276862181
ss65585172	*rs43672327*	11	15.733197	0.3639	4.6016	0.032287	0.0418	0.0193647892
	rs29020145	11	15.774261	0.2772	0.9081	0.340945	0.0192	0.0076948306
ss64458200	*rs42649474*	11	15.784436	0.3653	4.1523	0.041955	0.0398	0.0184598127
ss86301589		11	15.812748	0.6368	1.6402	0.200724	0.0246	0.0113584448
ss61512651	*rs41614455*	11	15.889463	0.1547	4.4224	0.035828	0.0525	0.0137403621
ss86320649		11	15.948301	0.7779	5.0488	0.024956	0.0500	0.0172661179
	rs29011156	11	15.996681	0.3840	6.0908	0.013828	0.0458	0.0216491474
ss86336606		11	16.04072	0.7901	5.0500	0.024939	0.0509	0.0168955034
ss65586198	*rs43665044*	11	16.143826	0.3374	15.3541	0.000098	0.0750	0.0335316298
ss86338187		11	16.240343	0.0960	4.3777	0.036774	0.0659	0.0114354153
ss86306158		11	16.264581	0.2414	0.1489	0.699698	0.0083	0.0030262345
ss65585601	*rs43666745*	11	16.478532	0.7937	2.2462	0.134398	0.0329	0.0107681334
ss105240971		11	16.520807	0.2192	1.8013	0.179995	0.0289	0.0098813314
ss86277668		11	16.541093	0.3123	0.6955	0.404574	0.0164	0.0070571460
ss86281340		11	16.663321	0.6862	3.2815	0.070495	0.0369	0.0158932640
ss117966058		11	16.684268	0.6834	4.1795	0.041292	0.0416	0.0179835109
ss65585456	*rs43665600*	11	16.730599	0.8073	0.6277	0.428481	0.0187	0.0058310955
ss86299238		11	16.752108	0.4219	6.4684	0.011196	0.0480	0.0233924539
ss86322692		11	16.777894	0.6805	0.4887	0.484725	0.0138	0.0059822613
ss117970242		11	16.929928	0.4277	3.4881	0.062231	0.0355	0.0173825420
ss65585028	*rs43671283*	11	16.96685	0.1354	0.4931	0.482794	0.0190	0.0044547166
ss65585079	*rs43672234*	11	16.998853	0.1719	2.3554	0.125303	0.0382	0.0108756360
ss86299514		11	17.02601	0.2120	5.6804	0.017422	0.0530	0.0177131980
ss65586331	*rs43666077*	11	17.081616	0.4198	0.7787	0.377850	0.0166	0.0080936021
ss61562909	*rs41665996*	11	17.205374	0.0809	0.3351	0.562883	0.0197	0.0029264244
ss64812286	*rs42907447*	11	17.250028	0.6848	0.8442	0.358524	0.0181	0.0078093626
ss65586503	*rs43669049*	11	17.304232	0.4198	0.7787	0.377850	0.0166	0.0080936021
ss86303850		11	17.335088	0.3832	0.2407	0.623845	0.0093	0.0044175001
ss105237447		11	17.356169	0.1554	1.0998	0.294683	0.0268	0.0070270629
ss61565609	*rs41662356*	11	17.417623	0.2142	1.7515	0.186121	0.0299	0.0100500749
ss64093673	*rs42476612*	11	17.625207	0.8374	1.4734	0.225227	0.0297	0.0080935048
ss86281442		11	17.71043	0.6168	3.8872	0.049050	0.0369	0.0174665453
ss61561738	*rs41664346*	11	17.765302	0.8295	0.3615	0.547871	0.0148	0.0041895627
ss61522227	*rs41617712*	11	17.809083	0.7923	0.0280	0.867115	0.0039	0.0012760285
ss64780117	*rs42870544*	11	17.837842	0.8438	0.4930	0.482838	0.0180	0.0047452266
ss64781766	*rs42876389*	11	17.880399	0.6117	7.3306	0.006945	0.0531	0.0252193440
ss64179891	*rs42513234*	11	17.939584	0.2400	3.8969	0.048769	0.0421	0.0153515104
ss64179981	*rs42513279*	11	17.963973	0.7400	4.8563	0.027872	0.0457	0.0176023660
ss61494216		11	17.987199	0.4327	6.8123	0.009248	0.0498	0.0244307764
ss117970176		11	18.174965	0.7428	1.0088	0.315539	0.0211	0.0080638200

ss61538219	*rs41638807*	11	18.198174	0.9585	0.0101	0.920089	0.0046	0.0003688280
ss62321032	*rs41827589*	11	18.224258	0.6182	5.9223	0.015201	0.0456	0.0215445135
ss64783900	*rs42877704*	11	18.507477	0.4427	19.9004	0.000010	0.0835	0.0412097232
ss63864264	*rs42391598*	11	18.546193	0.6504	11.9149	0.000591	0.0670	0.0304618628
ss61519949	*rs41620276*	11	18.613456	0.5895	6.1421	0.013436	0.0485	0.0234546009
ss86307051		11	18.659193	0.6175	8.2655	0.004164	0.0572	0.0270001106
ss86325685		11	18.723871	0.3675	8.1459	0.004444	0.0553	0.0257142313
ss61529841	*rs41629347*	11	18.750807	0.2479	0.6575	0.417712	0.0173	0.0064492609
ss86273912		11	18.776334	0.5752	0.6067	0.436297	0.0149	0.0072681027
ss117970278		11	18.824843	0.5387	1.2569	0.262629	0.0213	0.0106058551
ss64951223	*rs43045946*	11	18.851207	0.1662	1.2532	0.263337	0.0282	0.0078127621
	rs29015870	11	19.117264	0.6905	0.7818	0.376890	0.0188	0.0080522889
	rs29012734	11	19.241128	0.1762	8.9978	0.002800	0.0703	0.0204109106
ss64801688	*rs42894509*	11	19.267868	0.7264	1.9369	0.164447	0.0273	0.0108682605
ss64295354	*rs42570293*	11	19.349477	0.2615	1.6803	0.195310	0.0261	0.0100855422
ss64294824	*rs42568828*	11	19.376037	0.7314	1.4098	0.235501	0.0239	0.0094102808
ss64294948	*rs42569290*	11	19.40173	0.7085	2.5676	0.109530	0.0317	0.0131113670
ss86315183		11	19.432059	0.2299	1.8128	0.178614	0.0289	0.0102374326
ss61566894	*rs41668406*	11	19.466647	0.3095	2.0415	0.153512	0.0284	0.0121287123
ss117970406		11	19.530715	0.0752	1.6982	0.192950	0.0469	0.0065252006
ss61469823	*rs41572817*	11	19.553268	0.4148	0.1712	0.679210	0.0078	0.0037836205
ss86302619		11	19.635434	0.3596	8.9351	0.002896	0.0580	0.0267299006
ss61474710	*rs41571702*	11	19.657413	0.6511	14.3600	0.000164	0.0731	0.0331885326
ss117970352		11	19.734849	0.3238	3.8082	0.051403	0.0390	0.0170963118
ss86291352		11	19.832528	0.6110	15.3322	0.000099	0.0734	0.0349112058
ss86296498		11	19.866657	0.2350	4.4282	0.035707	0.0453	0.0162705886
ss61505077	*rs41602844*	11	19.933224	0.5917	8.8869	0.002973	0.0580	0.0280287586
ss61562916	*rs41666003*	11	19.969065	0.5466	10.4513	0.001283	0.0624	0.0309497125
ss61505115	*rs41602880*	11	20.223877	0.0745	0.4723	0.492159	0.0245	0.0033847158
ss65590714	*rs43672658*	11	20.417331	0.9148	3.5859	0.058686	0.0634	0.0098859293
	rs29013980	11	20.439308	0.7521	3.0559	0.080887	0.0371	0.0138400048
ss65586896	*rs43673640*	11	20.465096	0.9191	0.3255	0.568497	0.0194	0.0028809495
ss86291630		11	20.500192	0.9083	0.7749	0.379015	0.0276	0.0046017446
ss86333710		11	20.611027	0.3739	12.9496	0.000343	0.0695	0.0325455002
ss86290641		11	20.672763	0.3059	17.9718	0.000025	0.0851	0.0361320149
ss65590769	*rs43672713*	11	20.701937	0.1060	0.2463	0.619883	0.0152	0.0028754029
ss64713508	*rs42811902*	11	20.749555	0.8360	0.6067	0.436315	0.0200	0.0054857843
ss117970174		11	20.901524	0.8410	0.3848	0.535252	0.0158	0.0042179297
ss86313643		11	20.956301	0.8840	2.9379	0.086969	0.0500	0.0102656414
ss86335399		11	20.980236	0.4857	10.0620	0.001580	0.0576	0.0287619104
ss63894228	*rs41915804*	11	21.02266	0.1655	13.1969	0.000301	0.0917	0.0253235669
ss61493938	*rs41592153*	11	21.142789	0.8030	4.0269	0.045166	0.0456	0.0144246264
ss86303163		11	21.166107	0.5179	3.2621	0.071330	0.0320	0.0159624360
ss86326649		11	21.223269	0.2980	14.8829	0.000125	0.0781	0.0326604191
ss62563116	*rs41913692*	11	21.257057	0.6927	5.9694	0.014804	0.0498	0.0212041111
ss86284719		11	21.289388	0.5666	6.2081	0.012948	0.0457	0.0224317134
ss62563503	*rs41914521*	11	21.385252	0.8804	0.0301	0.862383	0.0049	0.0010238323

ss86290855		11	21.451057	0.5752	9.6015	0.002023	0.0586	0.0286143175
ss86334479		11	21.689136	0.7650	0.6607	0.416604	0.0182	0.0065505524
ss63894562	rs41917571	11	21.768763	0.2264	14.1451	0.000183	0.0824	0.0288578219
ss61506882	rs41600091	11	21.817408	0.7335	0.1706	0.679726	0.0088	0.0034417887
ss86330016		11	21.849623	0.8933	2.8968	0.089201	0.0507	0.0096610242
ss65588097	rs43675642	11	21.886472	0.0738	0.0213	0.883899	0.0051	0.0007022538
ss65588077	rs43674522	11	21.907799	0.8890	0.2231	0.636805	0.0138	0.0027307522
ss61562931	rs41657732	11	21.929482	0.9255	0.0170	0.896158	0.0046	0.0006309534
ss61562930	rs41657731	11	21.954298	0.2450	2.4482	0.118111	0.0345	0.0127524242
ss86337729		11	21.982225	0.7178	0.8506	0.356691	0.0195	0.0079003935
ss86337934		11	22.016409	0.7170	0.7241	0.395097	0.0180	0.0073021484
ss61469512	rs41570414	11	22.038596	0.8503	0.0036	0.952026	0.0016	0.0004041890
ss65587928	rs43673273	11	22.15706	0.0451	0.1379	0.710505	0.0165	0.0014253391
ss61493957	rs41592172	11	22.277349	0.1483	1.7109	0.191294	0.0346	0.0087405611
ss65587724	rs43671170	11	22.377727	0.0989	0.0785	0.779366	0.0087	0.0015487822
ss61523007	rs41623475	11	22.40288	0.0989	0.3042	0.581437	0.0173	0.0030753463
ss65587605	rs43669951	11	22.43722	0.8990	0.3620	0.547590	0.0183	0.0033166027
ss105236739		11	22.471023	0.0967	0.8776	0.349182	0.0293	0.0051234119
ss65587543	rs43668789	11	22.502812	0.2077	0.9909	0.319857	0.0230	0.0075572082
ss61493944	rs41592159	11	22.565108	0.0867	1.6937	0.193539	0.0427	0.0067590212
ss65587511	rs43668757	11	22.603198	0.0867	1.6937	0.193539	0.0427	0.0067590212
ss65587419	rs43667365	11	22.626345	0.8016	1.2913	0.256198	0.0266	0.0084612263
ss86296139		11	22.652768	0.7149	3.2462	0.072021	0.0365	0.0148892965
ss86296647		11	22.727079	0.4721	13.5863	0.000246	0.0667	0.0332407074
ss65587173	rs43676217	11	22.749486	0.9097	0.7985	0.371866	0.0291	0.0047784547
ss117964171		11	22.774052	0.6010	10.7067	0.001120	0.0607	0.0290962568
ss86293124		11	22.84477	0.4004	10.3085	0.001385	0.0595	0.0285583135
ss65588306	rs43667552	11	22.897054	0.3933	9.9286	0.001697	0.0582	0.0277845094
ss61563435	rs41660908	11	22.959026	0.4226	7.1499	0.007673	0.0490	0.0239144205
ss61563456		11	23.010441	0.5795	14.1348	0.000184	0.0705	0.0343449722
ss86338985		11	23.058182	0.0759	0.0506	0.822170	0.0077	0.0010859687
ss61563457	rs41660929	11	23.080617	0.4362	11.5907	0.000701	0.0632	0.0310780051
ss65588578	rs43670224	11	23.160824	0.4506	21.1080	0.000005	0.0838	0.0415130093
ss61563461	rs41661833	11	23.187876	0.5931	13.4920	0.000258	0.0668	0.0322372630
ss86328538		11	23.25166	0.2887	8.9837	0.002821	0.0585	0.0240071947
ss86331493		11	23.314222	0.5150	24.5675	0.000001	0.0900	0.0449568435
ss61563470		11	23.365719	0.7042	0.1528	0.696020	0.0080	0.0033212167
ss105236752		11	23.387864	0.1003	1.4319	0.231860	0.0373	0.0067275635
ss86338308		11	23.446196	0.9205	0.6551	0.418576	0.0274	0.0040077062
ss61471671	rs41571820	11	23.489159	0.3997	3.9191	0.048134	0.0380	0.0182429493
ss61568505		11	23.627331	0.5122	1.2815	0.258013	0.0211	0.0105340630
ss86311399		11	23.656239	0.3037	2.6430	0.104461	0.0318	0.0134572637
ss61568507	rs41664598	11	23.688701	0.0573	1.5584	0.212318	0.0502	0.0054187660
ss117970112		11	23.709516	0.0566	3.1054	0.078471	0.0702	0.0074980174
ss61496783	rs41592128	11	23.749758	0.8446	3.8605	0.049832	0.0503	0.0132141315
ss86339494		11	23.77941	0.2973	0.0142	0.905309	0.0025	0.0010274056
ss86336361		11	23.804399	0.6977	0.2247	0.635601	0.0095	0.0040154548

ss61523009	*rs43741350*	11	23.829338	0.1884	3.7205	0.054156	0.0451	0.0137976201
ss86337633		11	23.907607	0.1196	2.7716	0.096401	0.0459	0.0096767232
ss65589373	*rs43667119*	11	23.937302	0.7858	0.0648	0.799175	0.0056	0.0018835693
ss117970411		11	23.974182	0.8481	0.0407	0.840251	0.0052	0.0013343831
ss65589442	*rs43668087*	11	24.019722	0.3388	0.3351	0.562832	0.0114	0.0051238196
ss65589461	*rs43668106*	11	24.046143	0.9341	5.0598	0.024799	0.0832	0.0102375587
ss86291887		11	24.100373	0.8431	1.9269	0.165538	0.0350	0.0092641742
ss65589566	*rs43668411*	11	24.123884	0.6519	0.4251	0.514623	0.0127	0.0057614266
ss64920351	*rs43011998*	11	24.235546	0.2787	1.1313	0.287875	0.0225	0.0090603369
ss61522323	*rs41618505*	11	24.26665	0.2837	0.7015	0.402555	0.0174	0.0070735584
ss61522322	*rs41618504*	11	24.303516	0.6934	0.2115	0.645736	0.0095	0.0040315454
ss65589701	*rs43669744*	11	24.355203	0.3044	1.2587	0.262293	0.0229	0.0096779438
ss65589765	*rs43669808*	11	24.37989	0.2643	0.3554	0.551282	0.0122	0.0047633837
ss64875816	*rs42968596*	11	24.413551	0.9499	0.2488	0.618072	0.0212	0.0020230202
ss86317579		11	24.486469	0.4756	0.1098	0.740425	0.0063	0.0031184102
ss64822694	*rs42918323*	11	24.604572	0.5673	1.9468	0.163382	0.0261	0.0128189230
ss86309580		11	24.645094	0.5079	0.6083	0.435689	0.0144	0.0072112873
ss61471140	*rs41567211*	11	24.817264	0.4864	0.6368	0.425136	0.0147	0.0073507591
ss61513885	*rs41616745*	11	24.845998	0.7299	4.1149	0.042887	0.0425	0.0167704662
ss64512572	*rs42671652*	11	24.892066	0.7378	0.0009	0.976283	0.0006	0.0002415717
ss64287054	*rs42563770*	11	24.927959	0.7923	0.4634	0.496257	0.0153	0.0050409717
ss64286956	*rs42563021*	11	24.949976	0.5287	1.7175	0.190450	0.0241	0.0120251161
ss64286732	*rs42562109*	11	24.970857	0.5573	1.0904	0.296740	0.0193	0.0095328236
ss61524386	*rs41620632*	11	25.011591	0.6941	0.5048	0.477649	0.0145	0.0061487440
ss64146855	*rs42494516*	11	25.076271	0.6884	0.1234	0.725502	0.0069	0.0029679911
ss105237480		11	25.1583	0.0638	1.9701	0.160885	0.0509	0.0060771826
ss86291325		11	25.271734	0.2736	4.7192	0.030165	0.0457	0.0181497827
ss86298186		11	25.304386	0.7092	3.3252	0.068656	0.0373	0.0153941284
ss64147331	*rs42496454*	11	25.333697	0.3832	0.3425	0.558588	0.0115	0.0054356986
ss64759818	*rs42858772*	11	25.354941	0.6168	0.3425	0.558588	0.0115	0.0054356986
ss86282706		11	25.392446	0.3940	0.1024	0.749071	0.0062	0.0029659751
ss65590673	*rs43671617*	11	25.418475	0.3883	0.3001	0.584014	0.0108	0.0051109621
	rs29025725	11	25.489244	0.5860	1.3202	0.250956	0.0226	0.0109629513
ss65590586	*rs43671532*	11	25.534785	0.1232	1.1380	0.286442	0.0285	0.0061628788
ss86337726		11	25.570163	0.5960	3.5855	0.058701	0.0363	0.0174784657
ss61494512	*rs41597697*	11	25.607687	0.5451	0.3439	0.557750	0.0110	0.0054537759
ss86339272		11	25.670631	0.4563	4.2973	0.038540	0.0385	0.0190951429
ss117970300		11	25.735848	0.3130	11.3982	0.000776	0.0678	0.0291640857
ss86324473		11	25.757507	0.6755	14.1868	0.000180	0.0753	0.0330049995
ss117964187		11	25.826469	0.6819	0.0517	0.820135	0.0047	0.0020185756
ss61523013	*rs41623480*	11	25.858551	0.6791	0.0540	0.816308	0.0048	0.0020808144
ss86323548		11	25.878966	0.6218	6.5562	0.010662	0.0495	0.0233005651
ss61493943	*rs41592158*	11	25.92048	0.5487	3.4838	0.062394	0.0352	0.0174574308
ss65587068	*rs43675012*	11	25.942464	0.6597	3.6043	0.058043	0.0372	0.0166911481
ss65587024	*rs43674968*	11	26.03155	0.9262	1.8527	0.173907	0.0478	0.0065399654
ss61493941	*rs41592156*	11	26.080473	0.3446	0.1169	0.732526	0.0068	0.0030564958
ss86309968		11	26.102954	0.6798	2.1069	0.147089	0.0279	0.0121405432

ss61562921	*rs41666008*	11	26.168236	0.6547	1.8323	0.176292	0.0258	0.0116527275
ss86336376		11	26.200238	0.4019	3.4629	0.063180	0.0341	0.0163722992
ss86297456		11	26.358189	0.4291	4.7005	0.030492	0.0403	0.0197325401
ss86307777		11	26.581186	0.0745	0.7596	0.383766	0.0308	0.0042488688
ss86323486		11	26.621224	0.2514	2.9592	0.085836	0.0366	0.0137607095
ss105289365		11	26.644921	0.2536	3.5622	0.059525	0.0399	0.0151192714
ss86324044		11	26.668984	0.7393	1.5581	0.212365	0.0269	0.0103571289
ss86304215		11	26.70398	0.1991	2.8125	0.093983	0.0393	0.0125302471
ss61564615	*rs41662184*	11	26.728628	0.5115	3.7964	0.051766	0.0367	0.0183401995
ss65592095	*rs43674735*	11	26.749387	0.5301	3.9391	0.047568	0.0374	0.0186075681
ss61511380	*rs41611898*	11	26.791429	0.1798	1.9422	0.163875	0.0334	0.0098438792
ss86298031		11	26.829183	0.6003	1.4553	0.228082	0.0228	0.0109362675
ss86319565		11	26.919621	0.3496	2.1079	0.146990	0.0279	0.0126908950
ss61494876	*rs41591463*	11	26.94299	0.6497	2.3190	0.128258	0.0293	0.0133301578
ss86320557		11	26.970506	0.3582	2.0427	0.153388	0.0273	0.0125680334
ss65592457	*rs43671097*	11	26.992349	0.3059	1.7380	0.187831	0.0271	0.0115200916
ss61564620	*rs41662189*	11	27.02234	0.3080	1.0581	0.304007	0.0210	0.0089440344
ss86340427		11	27.150482	0.1390	1.1934	0.275023	0.0297	0.0071149946
ss61481072	*rs41582694*	11	27.335964	0.2958	9.4577	0.002185	0.0604	0.0251856734
ss86274123		11	27.396492	0.1891	6.7088	0.009795	0.0612	0.0187801483
ss86322486		11	27.430933	0.9212	4.7452	0.029715	0.0753	0.0109261546
ss64500222	*rs42667171*	11	27.45383	0.7156	0.8514	0.356476	0.0194	0.0079027990
	rs29014906	11	27.490992	0.6017	3.4285	0.064505	0.0352	0.0168566323
ss62140788	*rs41765671*	11	27.525964	0.5910	3.8605	0.049833	0.0373	0.0180141791
ss61481078	*rs41582700*	11	27.558609	0.8360	7.6583	0.005801	0.0689	0.0188900485
ss61992659	*rs41715426*	11	27.688362	0.7844	7.4234	0.006600	0.0616	0.0208528627
ss61992719	*rs41716446*	11	27.709201	0.8410	5.5136	0.019148	0.0605	0.0161727651
ss61535478	*rs41634610*	11	27.73132	0.1590	5.5136	0.019148	0.0605	0.0161727651
ss117964210		11	27.756097	0.1569	5.5953	0.018282	0.0618	0.0163511265
ss86336189		11	27.778027	0.8417	5.4781	0.019538	0.0603	0.0160796433
ss65585711	*rs43667155*	11	27.816676	0.8259	4.6413	0.031553	0.0513	0.0147563815
ss117970192		11	27.870458	0.2973	7.5893	0.006025	0.0556	0.0232432155
ss61493927	*rs41592142*	11	27.892119	0.6325	10.2007	0.001467	0.0603	0.0280408558
ss61493928	*rs41592143*	11	27.996153	0.8317	4.0453	0.044680	0.0492	0.0137748775
ss86339115		11	28.075619	0.5716	8.6978	0.003293	0.0544	0.0266544409
ss61516964	*rs41621161*	11	28.192599	0.4620	9.4285	0.002220	0.0554	0.0275238863
ss86289220		11	28.240533	0.4585	5.2937	0.021697	0.0407	0.0201877240
ss86331665		11	28.33237	0.3840	21.7519	0.000004	0.0841	0.0397838789
ss86341084		11	28.357295	0.5788	11.6878	0.000666	0.0608	0.0296233842
ss61505112	*rs41602877*	11	28.425268	0.5788	11.6878	0.000666	0.0608	0.0296233842
ss64197239	*rs42526148*	11	28.449704	0.3782	19.4068	0.000012	0.0804	0.0378215893
ss86304002		11	28.483197	0.4162	10.4074	0.001314	0.0577	0.0280164683
ss86338783		11	28.535525	0.1562	7.1649	0.007609	0.0692	0.0182460147
ss64196945	*rs42524901*	11	28.569708	0.3066	4.7472	0.029680	0.0436	0.0185500365
ss86293410		11	28.623016	0.6504	4.1459	0.042114	0.0401	0.0182416738
ss86338365		11	28.66085	0.6375	2.2762	0.131827	0.0282	0.0130149981
ss86323293		11	28.727924	0.7600	8.6422	0.003393	0.0638	0.0232803297

ss61495132	*rs41593715*	11	28.909667	0.3589	4.3779	0.036770		0.0406	0.0187046278
ss86312155		11	29.134031	0.2400	0.7149	0.398109		0.0178	0.0065058380
ss86318354		11	29.187345	0.0645	0.0123	0.911603		0.0042	0.0005125873
ss86312762		11	29.27218	0.1569	0.8549	0.355503		0.0231	0.0061013474
ss63918616	*rs42183769*	11	29.322387	0.9219	0.1554	0.693575		0.0140	0.0020091648
ss63918766	*rs42184844*	11	29.348404	0.8775	8.0795	0.004608		0.0813	0.0174870874
ss86320531		11	29.398943	0.2579	0.1286	0.720043		0.0078	0.0029845778
ss63918984	*rs42228854*	11	29.422786	0.2199	4.6596	0.031221		0.0483	0.0165548387
ss86274738		11	29.447109	0.1497	0.6327	0.426630		0.0205	0.0052208579
ss86306007		11	29.494438	0.4427	7.6270	0.005902		0.0515	0.0254325797
ss61565144	*rs41658403*	11	29.525273	0.5458	6.3121	0.012217		0.0460	0.0228261865
ss86340636		11	29.572318	0.4857	4.7296	0.029984		0.0393	0.0196445099
ss65593185	*rs43677722*	11	29.598026	0.4370	10.2757	0.001410		0.0591	0.0290589641
ss86275358		11	29.767136	0.9362	0.1395	0.708905		0.0139	0.0016571432
ss86320993		11	29.788977	0.3195	0.3010	0.583416		0.0109	0.0047447109
ss86323729		11	29.814967	0.7170	5.9215	0.015208		0.0503	0.0204027402
ss117964217		11	29.840005	0.1411	0.8313	0.362209		0.0243	0.0058857686
ss65592818	*rs43675056*	11	29.951552	0.6361	10.6749	0.001139		0.0621	0.0287539471
ss86331293		11	29.97506	0.3754	4.5022	0.034203		0.0396	0.0185756541
ss86337795		11	30.040632	0.6468	7.3358	0.006926		0.0517	0.0236131984
ss86318545		11	30.070765	0.3481	8.8241	0.003075		0.0571	0.0259134054
ss61565136	*rs41658395*	11	30.130318	0.5337	2.4248	0.119885		0.0288	0.0143270567
ss65589064	*rs43675909*	11	30.21036	0.4620	2.4298	0.119500		0.0284	0.0141363079
ss86335040		11	30.255632	0.0888	0.5161	0.472771		0.0233	0.0037768360
ss86305303		11	30.330448	0.2350	0.0350	0.851625		0.0041	0.0014820957
ss86337872		11	30.36611	0.0838	0.2616	0.609176		0.0171	0.0026311635
ss86327980		11	30.440628	0.3617	4.6794	0.030866		0.0422	0.0194707612
ss86333509		11	30.472205	0.3574	5.9494	0.014971		0.0478	0.0219436769
ss86289343		11	30.510403	0.7572	1.3459	0.246396		0.0245	0.0090236180
ss86339484		11	30.536557	0.3252	3.8374	0.050519		0.0386	0.0169345772
ss86336956		11	30.563055	0.6504	0.0440	0.833905		0.0042	0.0018908281
ss86328445		11	30.60196	0.1605	3.5688	0.059291		0.0476	0.0128268650
ss86302843		11	30.628462	0.1569	4.5342	0.033574		0.0539	0.0142538725
ss86332796		11	30.671402	0.2701	0.9587	0.327845		0.0202	0.0079775039
ss86297003		11	30.799679	0.8266	0.1811	0.670560		0.0105	0.0030000752
ss86327515		11	30.875946	0.1590	5.3234	0.021334		0.0593	0.0158730832
ss105241699		11	30.899695	0.8209	7.0142	0.008270		0.0653	0.0191951778
ss117970195		11	30.921845	0.6705	0.2272	0.633789		0.0094	0.0041379429
ss61564013	*rs41657892*	11	30.988287	0.7701	5.5663	0.018584		0.0519	0.0183829731
ss61494521	*rs41597706*	11	31.019168	0.2292	3.7995	0.051670		0.0429	0.0151750084
ss86337023		11	31.044171	0.5931	6.1859	0.013111		0.0474	0.0228997391
ss86298721		11	31.068997	0.3238	3.4064	0.065368		0.0373	0.0163463285
ss86317275		11	31.117235	0.3360	6.9518	0.008560		0.0520	0.0232147960
ss65590842	*rs43673483*	11	31.206483	0.4384	0.4986	0.480353		0.0134	0.0066102711
ss86273842		11	31.262699	0.2958	4.5066	0.034117		0.0436	0.0181663716
ss61474437	*rs41570140*	11	31.357928	0.3711	1.4400	0.230543		0.0236	0.0110300570
ss86305232		11	31.422206	0.7801	4.6179	0.031984		0.0472	0.0162022899

ss64334698	*rs42583510*	11	31.445192	0.2736	0.3302	0.565746	0.0122	0.0048593334
ss86308694		11	31.468032	0.1827	0.7413	0.389556	0.0204	0.0060918730
ss64335216	*rs42587069*	11	31.488881	0.1819	0.7438	0.388728	0.0204	0.0060874773
ss86326603		11	31.517981	0.8188	0.4347	0.509918	0.0162	0.0048097371
ss86320246		11	31.549658	0.1798	0.8908	0.345576	0.0224	0.0066143117
ss86329844		11	31.665816	0.1705	2.5973	0.107498	0.0398	0.0112702759
ss117970069		11	31.68774	0.4699	0.0425	0.836821	0.0039	0.0019413586
ss64475196	*rs42654631*	11	31.796289	0.1239	9.1024	0.002646	0.0848	0.0184223131
ss117970190		11	31.82033	0.2042	6.4591	0.011254	0.0578	0.0187927271
ss65594314	*rs43673549*	11	31.92322	0.5523	0.6327	0.426653	0.0153	0.0075800925
ss61565181	*rs41659038*	11	31.946339	0.5931	0.0003	0.985577	0.0004	0.0001702783
ss65594299	*rs43673534*	11	31.979912	0.3360	3.0479	0.081281	0.0344	0.0153586153
ss65612518	*rs43691132*	11	32.020469	0.3023	7.3168	0.006998	0.0558	0.0235312002
ss105241011		11	32.045698	0.1934	0.0425	0.836813	0.0048	0.0015118528
ss61499781	*rs41595365*	11	32.084554	0.1368	1.9382	0.164311	0.0377	0.0089010067
ss64889503	*rs42981212*	11	32.157664	0.3037	9.2405	0.002456	0.0629	0.0265889132
ss86313982		11	32.222179	0.2034	5.2527	0.022210	0.0502	0.0162814686
ss61497406	*rs41596833*	11	32.314085	0.2686	6.0530	0.014124	0.0498	0.0195543625
ss86337445		11	32.33711	0.0544	2.8451	0.092100	0.0683	0.0070319959
ss86332346		11	32.46012	0.5337	0.0154	0.901404	0.0024	0.0011811791
ss61565174	*rs41659033*	11	32.569375	0.5630	0.1828	0.669098	0.0080	0.0039119565
ss86301914		11	32.612343	0.7156	1.4440	0.229897	0.0249	0.0101302198
ss86292910		11	32.632669	0.3453	2.7667	0.096697	0.0335	0.0151466563
ss117964221		11	32.663176	0.1261	0.4187	0.517800	0.0183	0.0040236384
ss86305298		11	32.69833	0.8381	4.4766	0.034716	0.0525	0.0142537463
ss86340676		11	32.746096	0.1232	0.5204	0.470927	0.0206	0.0044586804
ss86339235		11	32.782218	0.3252	5.4825	0.019489	0.0450	0.0197562972
ss117970419		11	32.873367	0.4850	5.8907	0.015474	0.0448	0.0224015747
ss63399655	*rs42214386*	11	32.946524	0.1132	2.5603	0.110030	0.0471	0.0094646756
ss63399520	*rs42214341*	11	32.970074	0.9062	0.1102	0.740071	0.0103	0.0017553036
ss86289768		11	32.993631	0.7278	4.0807	0.043759	0.0410	0.0162641938
ss61511555	*rs41613466*	11	33.023301	0.7557	3.5314	0.060633	0.0404	0.0148979495
ss61523897	*rs41624254*	11	33.193745	0.3718	3.4778	0.062619	0.0362	0.0169066054
ss117970306		11	33.276455	0.7758	0.7882	0.374953	0.0196	0.0068020699
ss63398878	*rs42218927*	11	33.301544	0.2600	0.6669	0.414427	0.0170	0.0065530489
ss61495763	*rs41592430*	11	33.333357	0.7844	0.4581	0.498718	0.0156	0.0052635562
ss63398617	*rs42218840*	11	33.381378	0.0408	0.6433	0.422792	0.0373	0.0029184870
ss63398302	*rs42218435*	11	33.511439	0.0530	0.7923	0.373719	0.0364	0.0036593197
ss63398533	*rs42218512*	11	33.532562	0.7443	3.3933	0.065886	0.0397	0.0151243894
ss63398098	*rs42217767*	11	33.619552	0.1884	1.8075	0.179249	0.0318	0.0097168373
ss61505390	*rs41606137*	11	33.691155	0.6139	9.0522	0.002719	0.0583	0.0276511080
ss63397141	*rs42215348*	11	33.801848	0.4971	6.3129	0.012212	0.0467	0.0233394455
ss63397006	*rs42214703*	11	33.877082	0.3388	0.1466	0.701925	0.0074	0.0033087513
ss63396784	*rs42214229*	11	33.914082	0.7514	0.4588	0.498419	0.0143	0.0053252668
ss63396640	*rs42214181*	11	33.936972	0.3066	2.3164	0.128471	0.0300	0.0127635339
ss63396562	*rs42214155*	11	33.961657	0.3044	2.0692	0.150753	0.0283	0.0120047736
ss61564031	*rs41657910*	11	33.989538	0.6232	0.6680	0.414037	0.0155	0.0072782227

ss61564034	*rs41657913*	11	34.105349	0.2486	13.9360	0.000205	0.0817	0.0305333463
ss117970160		11	34.208675	0.7901	3.7689	0.052618	0.0443	0.0146885279
ss86282793		11	34.242513	0.7830	0.2394	0.624761	0.0112	0.0037902690
ss61565699	*rs41662946*	11	34.264733	0.2020	1.9263	0.165606	0.0322	0.0103770008
ss61565703	*rs41662950*	11	34.317238	0.0401	5.5715	0.018530	0.1102	0.0084852462
ss65594704	*rs43675735*	11	34.345131	0.9484	4.4950	0.034348	0.0889	0.0086962574
ss86331315		11	34.430218	0.6511	0.4823	0.487605	0.0140	0.0063405199
	rs29018149	11	34.466963	0.1361	3.6753	0.055633	0.0512	0.0120299120
ss86335997		11	34.496996	0.4964	1.5742	0.210021	0.0239	0.0119735940
ss64192325	*rs42519995*	11	34.654505	0.2600	1.1952	0.274669	0.0233	0.0089702386
ss64192391	*rs42520028*	11	34.733057	0.6948	0.7658	0.381821	0.0176	0.0074606537
ss61517673	*rs41618037*	11	34.84366	0.6583	3.8195	0.051060	0.0384	0.0172906631
ss65099588	*rs43190397*	11	34.892583	0.6619	2.6710	0.102646	0.0311	0.0139218455
ss86293994		11	34.96691	0.3080	11.1760	0.000873	0.0655	0.0279023048
ss64650922	*rs42749058*	11	35.046936	0.0616	3.2230	0.073045	0.0709	0.0081953660
ss64650945	*rs42749081*	11	35.090622	0.9384	3.2230	0.073045	0.0709	0.0081953660
ss64651060	*rs42749396*	11	35.131084	0.9176	1.2816	0.257989	0.0390	0.0058890530
ss64659018	*rs42759270*	11	35.221484	0.6934	2.1734	0.140872	0.0291	0.0123940092
ss61529217	*rs41633025*	11	35.433476	0.7156	8.0700	0.004632	0.0566	0.0230500080
ss61566212	*rs41662254*	11	35.475728	0.8181	0.5121	0.474458	0.0172	0.0051152289
ss64260756	*rs42551362*	11	35.513097	0.8188	0.4032	0.525658	0.0153	0.0045284196
ss61526402	*rs41623096*	11	35.550862	0.9262	0.9475	0.330707	0.0339	0.0046291931
ss63984937	*rs42417896*	11	35.578812	0.0788	1.5966	0.206813	0.0429	0.0062261273
ss61566211	*rs41662253*	11	35.63022	0.5695	0.0351	0.851451	0.0035	0.0017277968
ss64261134	*rs42553151*	11	35.657777	0.7400	7.8449	0.005238	0.0567	0.0218075766
ss63984837	*rs42417846*	11	35.716272	0.4563	3.5238	0.060913	0.0337	0.0167257351
ss61526399	*rs41623094*	11	35.784002	0.4792	5.6093	0.018138	0.0423	0.0211079559
ss65055570	*rs43144817*	11	35.947403	0.4148	9.8920	0.001731	0.0567	0.0275256510
ss64251080	*rs42552262*	11	35.978626	0.5036	6.2923	0.012352	0.0452	0.0225817241
ss64251262	*rs42553353*	11	36.013481	0.5910	7.9063	0.005065	0.0516	0.0249226772
ss64251498	*rs42554769*	11	36.07544	0.4105	0.8460	0.358016	0.0167	0.0080951084
ss86325888		11	36.177279	0.2006	0.0043	0.947770	0.0015	0.0004880691
ss86300968		11	36.263722	0.4162	0.8737	0.350250	0.0169	0.0082267135
ss65149276	*rs43233924*	11	36.291728	0.5781	0.9098	0.340504	0.0172	0.0083973792
ss86326088		11	36.37104	0.7822	0.0804	0.776774	0.0063	0.0021466913
ss65594849	*rs43676478*	11	36.437933	0.2557	1.7761	0.183067	0.0287	0.0109078268
ss61505326	*rs41604976*	11	36.474709	0.2364	5.9860	0.014666	0.0510	0.0184296789
ss86296440		11	36.54997	0.4964	8.4745	0.003717	0.0552	0.0276149045
ss86329476		11	36.574349	0.5824	2.7281	0.099048	0.0316	0.0153534276
ss64289088	*rs42565381*	11	36.672048	0.5888	7.9397	0.004973	0.0527	0.0255182449
ss63911602	*rs42125866*	11	36.712546	0.4405	14.1088	0.000187	0.0706	0.0347783759
	rs29018776	11	36.789741	0.7128	0.0405	0.840637	0.0042	0.0017292066
ss86295377		11	36.843952	0.7156	8.9873	0.002815	0.0609	0.0248077590
ss65595310	*rs43675242*	11	36.915953	0.2228	1.0383	0.308561	0.0228	0.0078844976
ss61466239		11	36.972566	0.5795	5.3575	0.020924	0.0434	0.0211599194
ss61524318	*rs41619768*	11	37.011223	0.0895	1.9818	0.159646	0.0452	0.0073739875
ss61524940	*rs41624768*	11	37.04608	0.1934	8.4566	0.003753	0.0678	0.0211415950

ss86327535		11	37.114302	0.2901	16.1598	0.000065	0.0820	0.0337612511
ss61521840	*rs41622338*	11	37.201656	0.7650	3.4201	0.064830	0.0405	0.0145610473
ss61527626	*rs41625082*	11	37.232754	0.7650	3.4201	0.064830	0.0405	0.0145610473
ss61521842	*rs41622340*	11	37.300075	0.1705	1.9374	0.164401	0.0341	0.0096458152
ss61515544	*rs41615197*	11	37.41235	0.4570	0.8252	0.363972	0.0170	0.0084552009
ss86340220		11	37.464881	0.4556	2.4021	0.121630	0.0289	0.0143375993
ss65594855	*rs43676484*	11	37.502777	0.5967	0.6268	0.428819	0.0152	0.0073013860
ss86286758		11	37.542379	0.4499	2.9818	0.084647	0.0319	0.0157927196
ss86316926		11	37.575808	0.1913	5.5023	0.019271	0.0554	0.0171419992
ss86281685		11	37.596247	0.2751	0.3686	0.543983	0.0124	0.0049535087
ss105238864		11	37.753035	0.5208	6.6341	0.010210	0.0479	0.0238964602
	rs29019692	11	37.837125	0.5387	1.9908	0.158706	0.0263	0.0130820759
ss86339677		11	37.875298	0.6597	6.9506	0.008566	0.0527	0.0236473202
ss86333382		11	38.027813	0.6562	7.5371	0.006200	0.0526	0.0237556427
ss61565722	*rs41662969*	11	38.100979	0.0860	1.4832	0.223692	0.0408	0.0064110108
ss61495728	*rs41592395*	11	38.282146	0.4928	1.6445	0.200139	0.0231	0.0115581149
ss86282918		11	38.377651	0.4377	8.8393	0.003050	0.0557	0.0274109463
ss105238891		11	38.460395	0.2228	10.8990	0.001011	0.0705	0.0244218606
ss86307616		11	38.48508	0.4162	2.6275	0.105478	0.0296	0.0143917944
ss86339500		11	38.523074	0.4155	2.4907	0.114977	0.0287	0.0139572791
ss61566327	*rs41663268*	11	38.544853	0.5817	1.5893	0.207854	0.0231	0.0112239743
ss86297019		11	38.575222	0.3403	14.8648	0.000126	0.0744	0.0333925414
ss105238896		11	38.608787	0.6597	14.8648	0.000126	0.0744	0.0333925414
ss86328535		11	38.662813	0.1225	25.2080	0.000001	0.1375	0.0295634605
ss86327381		11	38.703773	0.5430	11.9307	0.000586	0.0626	0.0310587809
ss86296477		11	38.730069	0.4319	8.1307	0.004481	0.0532	0.0261162445
ss86306845		11	38.763495	0.5587	8.6049	0.003463	0.0549	0.0270481241
	rs29011991	11	38.800905	0.3216	14.9834	0.000119	0.0752	0.0328072313
ss86281946		11	38.822548	0.3352	0.8148	0.367032	0.0179	0.0079810379
ss86313180		11	38.855357	0.2665	0.1722	0.678301	0.0088	0.0034421922
ss61477772	*rs41579594*	11	39.032465	0.0688	1.3143	0.252006	0.0413	0.0052901289
ss105239577		11	39.087803	0.8546	6.4550	0.011280	0.0655	0.0162873629
ss86286447		11	39.201053	0.1583	0.0541	0.816110	0.0059	0.0015722632
ss117964193		11	39.264499	0.1920	2.2600	0.133205	0.0359	0.0111332579
ss86293568		11	39.351812	0.4097	26.2520	0.000000	0.0959	0.0463755580
	rs29015719	11	39.385307	0.6225	11.4264	0.000765	0.0647	0.0304081825
ss86340892		11	39.426976	0.2894	17.7160	0.000029	0.0860	0.0353828372
ss117970355		11	39.528189	0.5938	17.2399	0.000037	0.0774	0.0373589235
ss61564592	*rs41662162*	11	39.606363	0.7407	22.4915	0.000003	0.0946	0.0363312130
ss61562679	*rs41663974*	11	40.028208	0.8345	0.0215	0.883525	0.0036	0.0010047574
ss61494861	*rs41591448*	11	40.098119	0.5645	11.4529	0.000754	0.0621	0.0305255803
ss61499333	*rs43709728*	11	40.12673	0.6698	12.5707	0.000418	0.0697	0.0308525804
ss61494862	*rs41591449*	11	40.164558	0.8968	0.0003	0.986757	0.0005	0.0000913192
ss86339928		11	40.184957	0.3095	9.3298	0.002341	0.0614	0.0262405801
ss86311096		11	40.254856	0.4907	18.3737	0.000021	0.0773	0.0386155139
ss105240280		11	40.276144	0.4792	17.4919	0.000033	0.0749	0.0373914372
ss86335773		11	40.316947	0.5702	0.4833	0.487159	0.0126	0.0061884676

ss117970126		11	40.358107	0.1218	26.1716	0.000000		0.1412	0.0301939301
ss86308768		11	40.384408	0.0903	1.5411	0.214868		0.0397	0.0065267721
ss86335005		11	40.409764	0.2622	1.4177	0.234180		0.0247	0.0095668085
ss86339651		11	40.482282	0.3080	4.0880	0.043571		0.0396	0.0168996139
ss86291327		11	40.567845	0.1476	21.7611	0.000004		0.1198	0.0301365737
ss117970345		11	40.627656	0.4441	0.5499	0.458616		0.0136	0.0067305609
ss86294739		11	40.729584	0.4097	1.7793	0.182666		0.0247	0.0119487663
ss86319422		11	40.775839	0.5953	5.8382	0.015938		0.0450	0.0216807311
ss86339483		11	40.836497	0.5050	6.6032	0.010387		0.0478	0.0238798669
ss61564228	*rs41659401*	11	40.927742	0.0595	0.1888	0.664065		0.0170	0.0019004170
ss86330414		11	40.955947	0.9212	0.0316	0.859006		0.0062	0.0009031253
ss117970339		11	41.108573	0.4585	2.5200	0.112866		0.0295	0.0146435321
ss86340400		11	41.147729	0.3052	1.7015	0.192519		0.0264	0.0111798514
ss105239643		11	41.22918	0.4327	0.0459	0.830507		0.0040	0.0019611234
ss61495279	*rs41595857*	11	41.363787	0.0587	6.8150	0.009234		0.1008	0.0111455679
	rs29013300	11	41.385159	0.5351	0.9548	0.328852		0.0180	0.0089796095
ss61565457	*rs41660809*	11	41.449758	0.2099	0.0913	0.762578		0.0067	0.0022088925
ss86339854		11	41.615596	0.6927	9.0159	0.002772		0.0602	0.0256329894
ss61505340	*rs41604990*	11	41.756742	0.5136	3.6418	0.056757		0.0354	0.0176958496
ss105239650		11	41.778864	0.4871	4.3897	0.036517		0.0387	0.0193435880
ss61565744	*rs41662991*	11	41.810071	0.5229	4.0813	0.043743		0.0378	0.0188772400
	rs29026974	11	41.862726	0.6139	5.7668	0.016593		0.0456	0.0216197973
ss61530386	*rs41624662*	11	42.806299	0.1676	0.4006	0.526980		0.0157	0.0043916412
	rs29010216	11	42.852247	0.3073	2.8565	0.091454		0.0343	0.0145943363
ss61569355	*rs41667139*	11	42.942455	0.5358	2.1051	0.147263		0.0280	0.0139080386
ss61497169	*rs41594599*	11	43.015647	0.7758	1.6590	0.198171		0.0287	0.0099832120
ss86303344		11	43.053345	0.0938	3.3895	0.066040		0.0596	0.0101354732
ss86315261		11	43.117158	0.9126	8.9742	0.002835		0.0992	0.0158166914
ss105298520		11	43.16648	0.0645	0.5100	0.475359		0.0264	0.0031892216
ss86307108		11	43.345557	0.7607	2.0163	0.156061		0.0315	0.0114788583
ss86294547		11	43.385213	0.7135	3.2617	0.071347		0.0374	0.0153028798
ss61500646	*rs41599395*	11	43.478402	0.4277	3.7957	0.051786		0.0373	0.0182694021
ss86297637		11	43.528731	0.7471	2.5806	0.108637		0.0341	0.0128722159
	rs29022274	11	43.572256	0.3968	2.6585	0.103450		0.0308	0.0147224354
ss105238904		11	43.593017	0.6003	2.6964	0.101026		0.0310	0.0148715085
ss61469783	*rs41572779*	11	43.726129	0.3668	5.0638	0.024742		0.0438	0.0203418234
ss61566346	*rs41663287*	11	43.804686	0.4527	1.0050	0.316447		0.0187	0.0092511472
ss61497483	*rs41596909*	11	43.836219	0.5681	4.1688	0.041552		0.0370	0.0181355185
ss61512059	*rs41610069*	11	43.867006	0.5702	6.2392	0.012725		0.0454	0.0222617882
ss86314415		11	44.117829	0.5638	10.6431	0.001159		0.0583	0.0286684242
ss61569935	*rs41671184*	11	44.166371	0.2579	3.6447	0.056658		0.0396	0.0151384352
ss61569934	*rs41671183*	11	44.21853	0.3066	0.0080	0.928766		0.0018	0.0007681623
ss105238922		11	44.26874	0.9219	0.0033	0.954287		0.0019	0.0002786096
ss86297002		11	44.309152	0.3388	0.5326	0.465769		0.0143	0.0063954425
ss86340291		11	44.403089	0.7299	0.0699	0.791497		0.0055	0.0021870012
ss61469786	*rs41572782*	11	44.50015	0.6533	3.7580	0.052958		0.0373	0.0169079716
ss61495750	*rs41592417*	11	44.586083	0.0888	0.8231	0.364582		0.0292	0.0047334277

ss61495760	*rs41592427*	11	44.638631	0.0938	0.5957	0.440467		0.0246	0.0041883181
	rs29016027	11	44.663104	0.2414	1.2015	0.273409		0.0229	0.0083989595
ss86338098		11	44.714003	0.5996	3.4463	0.063816		0.0343	0.0164710114
ss86337988		11	44.787113	0.5996	0.3952	0.529777		0.0115	0.0055321066
ss86321741		11	45.085021	0.0673	0.3984	0.528144		0.0232	0.0029144970
	rs29014004	11	45.173929	0.4527	1.1104	0.292358		0.0190	0.0094164917
ss86312562		11	45.260663	0.1941	7.2647	0.007202		0.0636	0.0198845136
ss61566913	*rs41668424*	11	45.367288	0.9391	0.1673	0.682685		0.0157	0.0017912479
ss61500271	*rs41598426*	11	45.506215	0.1125	0.8242	0.364272		0.0273	0.0054426983
ss46526926	*rs41257374*	11	45.672437	0.7779	5.1359	0.023742		0.0499	0.0172288361
ss86338321		11	45.728768	0.9097	3.6301	0.057156		0.0605	0.0099291098
ss105240325		11	45.805674	0.0888	1.0983	0.294999		0.0329	0.0053308495
ss86311723		11	45.847513	0.7872	4.6238	0.031876		0.0485	0.0162494006
ss86288110		11	45.908683	0.7966	2.7309	0.098874		0.0376	0.0121865962
ss86328458		11	45.950683	0.0523	0.4596	0.498048		0.0276	0.0027322390
ss117970095		11	45.985844	0.0981	0.5566	0.455872		0.0232	0.0041028450
ss86335240		11	46.14228	0.5681	0.8146	0.367085		0.0166	0.0081267005
ss86341662		11	46.246291	0.2328	2.4760	0.116047		0.0339	0.0121137359
ss86332794		11	46.284172	0.2314	2.5051	0.113935		0.0341	0.0121349925
ss86317405		11	46.30423	0.0673	0.0311	0.860080		0.0064	0.0008059263
ss61528542	*rs41626584*	11	46.357352	0.9327	0.0073	0.931902		0.0031	0.0003866337
ss86277089		11	46.387415	0.1368	0.2153	0.642772		0.0125	0.0029551128
ss61528544	*rs41626586*	11	46.408428	0.2414	1.5242	0.217406		0.0264	0.0096670086
ss86341677		11	46.437256	0.2113	3.9800	0.046433		0.0446	0.0148624289
ss61496232	*rs41597485*	11	46.586114	0.1812	0.0524	0.819047		0.0056	0.0016724749
	rs29010501	11	46.636485	0.4570	1.0936	0.296029		0.0191	0.0095033493
ss86340494		11	46.688492	0.7787	3.6794	0.055497		0.0427	0.0147233227
ss61567452	*rs41663167*	11	46.738073	0.5774	0.3032	0.582044		0.0101	0.0049325876
ss86328257		11	46.797971	0.8023	3.6300	0.057160		0.0431	0.0136785767
ss61496223	*rs41597476*	11	46.838255	0.1304	0.4653	0.495377		0.0184	0.0041825700
ss86338256		11	46.884627	0.0809	0.8710	0.351004		0.0323	0.0048080325
ss86338157		11	46.907466	0.8754	1.4113	0.235250		0.0352	0.0076888369
ss86331148		11	46.929471	0.8725	2.2003	0.138434		0.0434	0.0096500582
ss86341272		11	46.96345	0.8811	5.3540	0.020965		0.0640	0.0134138556
ss86293167		11	47.187155	0.1003	8.4488	0.003769		0.0861	0.0155289064
ss86321045		11	47.313225	0.7880	0.0033	0.954059		0.0013	0.0004321617
ss86334206		11	47.426929	0.8660	8.7153	0.003262		0.0807	0.0187183170
ss61496760	*rs41592112*	11	47.53401	0.7837	15.0695	0.000113		0.0849	0.0287979597
ss86285366		11	47.581828	0.3116	7.3727	0.006786		0.0545	0.0233842396
ss86340752		11	47.603218	0.5817	1.1105	0.292333		0.0191	0.0093009056
ss86334830		11	47.659021	0.1655	10.5921	0.001191		0.0779	0.0215105185
ss86307135		11	47.695662	0.4893	0.5058	0.477197		0.0130	0.0064831278
ss61568438	*rs41664535*	11	47.738294	0.4563	1.3715	0.241948		0.0210	0.0104334582
ss86337390		11	47.82961	0.6777	6.7409	0.009622		0.0512	0.0223492448
ss86308689		11	47.851201	0.8288	8.2071	0.004298		0.0681	0.0193394685
ss61477037	*rs41580785*	11	47.896785	0.7715	3.8858	0.049093		0.0427	0.0150649236
ss61505684	*rs41608220*	11	47.931236	0.6605	0.1569	0.692116		0.0078	0.0035133310

ss86341357		11	47.982901	0.2593	0.1241	0.724734	0.0072	0.0027811239
ss86335541		11	48.08055	0.2371	0.0001	0.992685	0.0002	0.0000700230
ss86299227		11	48.145986	0.5852	0.1057	0.745175	0.0062	0.0030055474
ss86274056		11	48.178987	0.4140	0.1406	0.707775	0.0071	0.0034606847
ss86312161		11	48.244598	0.4764	5.6978	0.017252	0.0442	0.0220409818
ss86282487		11	48.3604	0.4327	0.7158	0.397805	0.0160	0.0078400730
ss86308708		11	48.400099	0.4441	0.0345	0.852605	0.0035	0.0017302940
ss86292463		11	48.427055	0.2722	0.0197	0.888335	0.0029	0.0011616347
ss61511756	*rs41615065*	11	48.453359	0.1454	0.6725	0.412446	0.0211	0.0052459548
ss61567467	*rs41663179*	11	48.493774	0.4857	0.0488	0.825287	0.0041	0.0020555257
ss86306497		11	48.53836	0.5215	0.0165	0.897892	0.0024	0.0012057360
ss86298730		11	48.572395	0.4921	1.0482	0.306269	0.0187	0.0093365810
ss86301714		11	48.596258	0.0666	6.0666	0.014017	0.0928	0.0115441271
ss86309730		11	48.632559	0.0781	0.1235	0.725395	0.0124	0.0017915563
ss86340303		11	48.657034	0.1325	4.7490	0.029650	0.0603	0.0138579077
ss61530296	*rs41633374*	11	48.684041	0.0587	1.1605	0.281745	0.0434	0.0048018686
ss86291405		11	48.714415	0.2880	0.0355	0.850615	0.0039	0.0015941599
ss86319093		11	48.802627	0.0702	3.1248	0.077548	0.0660	0.0086173609
ss61512390		11	48.958155	0.8259	1.3878	0.239173	0.0290	0.0083326077
ss61470277	*rs41568666*	11	49.014268	0.5788	3.1700	0.075439	0.0338	0.0164763134
	rs29017556	11	49.094687	0.0788	0.5671	0.451659	0.0261	0.0037827213
ss86302080		11	49.121238	0.3259	0.4574	0.499080	0.0134	0.0058950241
ss86321834		11	49.158147	0.8438	1.6156	0.204132	0.0324	0.0085438716
ss117970392		11	49.194343	0.4054	3.0080	0.083300	0.0325	0.0156919100
ss105297810		11	49.286158	0.3238	0.2270	0.633897	0.0096	0.0042185002
ss86311896		11	49.317563	0.4900	0.7466	0.387839	0.0163	0.0081494052
ss61568430	*rs41663727*	11	49.354616	0.3832	0.9810	0.322286	0.0190	0.0089801560
ss117964207		11	49.480392	0.0838	16.5482	0.000053	0.1334	0.0204817009
ss86330765		11	49.533697	0.5294	0.7150	0.398081	0.0155	0.0077123893
ss46526285	*rs41255424*	11	49.745239	0.5487	0.9796	0.322643	0.0188	0.0093167294
ss86340587		11	49.783295	0.5387	0.9460	0.331073	0.0186	0.0092632581
ss86317608		11	49.807902	0.1483	2.2992	0.129894	0.0391	0.0098786980
ss86284251		11	49.834732	0.8388	0.5593	0.454811	0.0191	0.0051773200
ss46526639	*rs41257095*	11	49.85883	0.0494	0.0867	0.768553	0.0124	0.0011647802
ss86334070		11	49.930284	0.1956	8.9996	0.002797	0.0699	0.0219934415
ss61568411	*rs41663709*	11	50.018805	0.3395	2.0375	0.153914	0.0282	0.0126502794
ss61511911	*rs41616215*	11	50.043098	0.4799	0.1955	0.658491	0.0083	0.0041368485
ss46526817	*rs41257268*	11	50.099082	0.1562	6.7904	0.009361	0.0653	0.0172053896
ss86312353		11	50.163573	0.8675	6.7181	0.009744	0.0695	0.0159726175
ss86313791		11	50.287732	0.2521	7.1788	0.007551	0.0572	0.0215536979
ss86294032		11	50.394779	0.3897	1.0729	0.300651	0.0196	0.0093182130
ss86306702		11	50.445245	0.7400	5.1992	0.022899	0.0486	0.0187100338
ss86298558		11	50.639382	0.1261	0.1470	0.701575	0.0107	0.0023625879
ss86311477		11	50.695005	0.1261	0.1470	0.701575	0.0107	0.0023625879
	rs29027583	11	50.727381	0.7572	0.0083	0.927515	0.0019	0.0007121586
ss61567893	*rs41665691*	11	50.781097	0.3281	3.2108	0.073589	0.0347	0.0153047146
ss61567894	*rs41665692*	11	50.86975	0.4219	1.3642	0.243216	0.0215	0.0105036906

ss86330117		11	50.935477	0.3804	1.2610	0.261842	0.0209	0.0098749232
ss86320792		11	51.169064	0.3890	1.0838	0.298212	0.0194	0.0092391773
ss117970358		11	51.228014	0.4004	1.1618	0.281476	0.0201	0.0096479591
ss86311175		11	51.249966	0.1039	0.6620	0.416116	0.0250	0.0046586428
ss61567928	*rs41665726*	11	51.302884	0.4255	8.2039	0.004306	0.0523	0.0255476662
	rs29018818	11	51.48097	0.3653	3.3085	0.069351	0.0349	0.0161860874
ss86338331		11	51.510467	0.2794	2.5460	0.111028	0.0318	0.0127957579
	rs29018810	11	51.536549	0.8496	3.0346	0.081951	0.0464	0.0118482926
ss86301341		11	51.61309	0.2507	0.0741	0.785604	0.0059	0.0022346540
ss86305670		11	51.656534	0.8574	2.2152	0.137109	0.0402	0.0098229891
ss86281551		11	51.685699	0.1275	0.9760	0.323527	0.0281	0.0062464993
ss64534684	*rs42681694*	11	51.716179	0.3560	0.1306	0.717963	0.0069	0.0031829579
ss64534780	*rs42682542*	11	51.775204	0.3338	2.8474	0.091970	0.0337	0.0150009217
ss86317811		11	51.798976	0.5666	1.0174	0.313498	0.0187	0.0091957468
ss64201384	*rs42530710*	11	51.834115	0.3130	1.4757	0.224853	0.0235	0.0101193074
	rs29020516	11	51.90313	0.5738	0.0087	0.925714	0.0018	0.0008639697
ss61494550	*rs41588041*	11	51.927013	0.5523	0.1248	0.723943	0.0066	0.0032877915
ss86337238		11	51.963605	0.5415	0.5789	0.447018	0.0140	0.0069506624
ss64915199	*rs43011700*	11	52.113063	0.3682	0.3631	0.546961	0.0114	0.0052861609
ss61529966	*rs41630467*	11	52.141652	0.6289	0.4345	0.510003	0.0124	0.0057834966
ss61473063	*rs41566484*	11	52.184675	0.7980	0.3440	0.557705	0.0133	0.0042956496
ss64678062	*rs42775221*	11	52.206771	0.2020	0.4199	0.517188	0.0148	0.0047642664
ss86283350		11	52.228995	0.8739	0.8006	0.371231	0.0249	0.0054776679
ss64678161	*rs42776020*	11	52.250223	0.8696	0.8536	0.355866	0.0251	0.0056891162
ss86337687		11	52.281616	0.7099	2.5434	0.111210	0.0321	0.0132347786
ss65597810	*rs43680229*	11	52.318222	0.5752	0.0425	0.836693	0.0039	0.0019140410
ss65597738	*rs43680157*	11	52.355098	0.1483	0.1550	0.693936	0.0103	0.0026119234
ss86296821		11	52.793421	0.4076	0.0969	0.755717	0.0058	0.0027961374
ss86288145		11	52.96398	0.6827	0.2086	0.647971	0.0089	0.0038571722
ss61505426	*rs41606166*	11	53.138236	0.6211	1.1356	0.286964	0.0200	0.0094332727
ss61511600	*rs41613511*	11	53.308024	0.4126	1.5296	0.216587	0.0223	0.0108226717
ss61530658	*rs41626729*	11	53.396587	0.4370	0.5647	0.452625	0.0139	0.0068458123
ss86328919		11	53.433257	0.4649	2.3394	0.126594	0.0278	0.0138201531
ss86338368		11	53.616723	0.1984	2.5499	0.110752	0.0368	0.0117027974
ss117970361		11	53.659773	0.4226	0.0183	0.892393	0.0025	0.0012316699
ss61564331	*rs41660202*	11	53.766925	0.7708	0.5098	0.475465	0.0154	0.0054559209
ss61494689	*rs41589378*	11	53.838711	0.1827	1.9960	0.158157	0.0336	0.0100283242
ss61505762	*rs41608892*	11	53.92221	0.4069	1.1481	0.284316	0.0195	0.0094356635
ss105237489		11	54.032307	0.4083	1.9861	0.159199	0.0257	0.0124046800
ss105237492		11	54.071621	0.2414	0.1081	0.742460	0.0069	0.0025391604
ss105237493		11	54.230612	0.5315	0.2598	0.610389	0.0094	0.0046711997
	rs29011971	11	54.363221	0.3811	0.0287	0.865491	0.0032	0.0014949102
ss117975021		11	54.437608	0.3503	0.0208	0.885497	0.0028	0.0012528599
ss86331582		11	54.511208	0.3725	0.0336	0.854609	0.0034	0.0016067833
ss86335579		11	54.575531	0.2013	0.0187	0.891166	0.0032	0.0010238985
ss61567552	*rs41663559*	11	54.86941	0.8281	0.0633	0.801412	0.0062	0.0017767363
ss86300974		11	55.68505	0.3173	0.4119	0.521209	0.0124	0.0053908133

ss117975086		11	55.710667	0.4105	0.7296	0.393318	0.0161	0.0077934337
ss86291619		11	55.738317	0.5788	0.0436	0.834745	0.0039	0.0019066338
ss61567280	*rs41661696*	11	55.952234	0.1440	0.9122	0.339866	0.0256	0.0063017237
ss86315899		11	56.003089	0.0953	1.4393	0.230659	0.0383	0.0066085476
	rs29027341	11	56.051148	0.1168	1.0470	0.306554	0.0292	0.0060316035
ss105238947		11	56.076925	0.1461	1.0242	0.311877	0.0267	0.0066650991
ss86275752		11	56.327181	0.9398	0.0836	0.772529	0.0114	0.0012894360
ss61469997	*rs41565994*	11	56.361164	0.3281	0.3645	0.546234	0.0117	0.0051669187
ss117964160		11	56.425698	0.5695	0.2002	0.654672	0.0082	0.0040298228
ss86340567		11	56.487047	0.2364	0.2177	0.640963	0.0099	0.0035911215
ss86331399		11	56.520303	0.5122	0.2970	0.585930	0.0100	0.0050076130
ss86328050		11	56.580026	0.7350	0.8146	0.367072	0.0188	0.0073250156
ss61568973	*rs41668252*	11	56.606373	0.0989	0.3155	0.574532	0.0176	0.0031333554
ss117964219		11	56.697848	0.7142	1.9687	0.161028	0.0295	0.0120635714
ss65598134	*rs43683544*	11	56.743234	0.0781	2.3218	0.128023	0.0517	0.0074503132
ss86294742		11	56.843739	0.1741	2.5829	0.108476	0.0390	0.0112249617
ss64656242	*rs42750132*	11	56.875601	0.6375	0.4639	0.496049	0.0133	0.0061602090
ss61516893	*rs41620193*	11	56.910615	0.1089	4.6179	0.031983	0.0630	0.0122233052
ss61563730	*rs41663399*	11	56.984335	0.5537	3.5006	0.061766	0.0345	0.0170660585
ss61511278	*rs41610799*	11	57.039602	0.4993	0.2988	0.584818	0.0102	0.0051215804
ss86312769		11	57.066019	0.1110	1.0705	0.301184	0.0302	0.0059571281
ss86300833		11	57.150888	0.7242	0.5025	0.478651	0.0149	0.0059709357
ss117970417		11	57.190519	0.2744	0.4695	0.493458	0.0145	0.0057718236
ss61511276	*rs41610797*	11	57.225637	0.1311	1.5013	0.220883	0.0334	0.0076071396
ss86284165		11	57.309102	0.5143	0.0348	0.852137	0.0035	0.0017264973
ss86316979		11	57.333503	0.6597	0.1273	0.721353	0.0070	0.0031210226
ss117970085		11	57.361605	0.2335	0.7798	0.377508	0.0192	0.0068887911
ss61568470	*rs41664564*	11	57.456445	0.5967	0.0557	0.813519	0.0044	0.0021336433
ss61524402	*rs41620644*	11	57.476775	0.3574	0.1924	0.661050	0.0082	0.0037801873
ss61516699	*rs41618000*	11	57.517368	0.6977	1.0107	0.315084	0.0200	0.0084507505
ss86299990		11	57.578888	0.2493	0.3999	0.527331	0.0134	0.0050277740
ss61480075	*rs41584429*	11	57.745885	0.2822	0.7321	0.392511	0.0174	0.0070423320
ss86324524		11	58.61565	0.2715	0.2303	0.631451	0.0101	0.0039995376
ss61568976	*rs41668255*	11	58.647873	0.1160	10.0187	0.001617	0.0912	0.0187041560
ss63453778	*rs42241520*	11	58.724619	0.1196	11.3328	0.000803	0.0960	0.0202263531
ss65598396	*rs43678809*	11	58.777593	0.1196	11.3328	0.000803	0.0960	0.0202263531
ss86341701		11	58.820978	0.2715	0.0087	0.925696	0.0020	0.0007802458
ss63454045	*rs42234610*	11	58.870404	0.8840	10.1727	0.001489	0.0912	0.0187160898
ss63454261	*rs42234782*	11	58.89904	0.1153	10.5835	0.001196	0.0932	0.0190180095
ss86329608		11	58.922823	0.2851	0.0134	0.907847	0.0024	0.0009802255
ss64714052	*rs42808317*	11	58.976746	0.4212	1.7690	0.183941	0.0255	0.0124129738
	rs29010392	11	59.06728	0.5731	3.4262	0.064592	0.0352	0.0172446904
ss86303886		11	59.157409	0.4212	3.9968	0.045974	0.0381	0.0185612396
	rs29014674	11	59.350663	0.3274	4.9371	0.026606	0.0449	0.0197635896
ss61569458	*rs41667839*	11	59.370908	0.2930	1.3769	0.241038	0.0245	0.0101706036
ss64055613	*rs42452733*	11	59.498037	0.2937	1.5200	0.218042	0.0258	0.0107029676
ss64408106	*rs42622730*	11	59.580345	0.1476	6.9461	0.008587	0.0685	0.0172441816

ss64670808	*rs42765176*	11	59.907601	0.1497	1.6146	0.204277		0.0331	0.0084296134
ss64055323	*rs42451303*	11	59.938096	0.1762	0.2220	0.637687		0.0116	0.0033612152
ss38322314	*rs29013422*	11	60.028401	0.5172	0.4560	0.499727		0.0125	0.0062454616
ss86294629		11	60.064245	0.0587	11.1901	0.000866		0.1306	0.0144424584
ss117970076		11	60.102402	0.7615	0.7821	0.376791		0.0190	0.0069042038
ss61534235	*rs41633088*	11	60.128948	0.7486	0.1732	0.677374		0.0088	0.0033252703
ss117970221		11	60.185405	0.8195	0.0732	0.786856		0.0066	0.0019425982
ss105237525		11	60.242004	0.2249	0.0221	0.881752		0.0033	0.0011543689
ss105237526		11	60.264787	0.5709	1.0465	0.306665		0.0190	0.0093110747
ss105273305		11	60.324508	0.7006	0.1333	0.715163		0.0075	0.0031513541
ss61514937		11	60.344955	0.3883	0.5057	0.477262		0.0134	0.0063784234
ss105238192		11	60.429065	0.5423	0.2073	0.649055		0.0087	0.0042963872
ss61514933	*rs41617186*	11	60.47174	0.2163	0.2017	0.653472		0.0102	0.0034741399
ss63758198	*rs42338999*	11	60.503781	0.6218	1.6099	0.204928		0.0246	0.0115927129
ss86339972		11	60.526062	0.2837	0.2270	0.633879		0.0099	0.0040151821
ss63757766	*rs42337856*	11	60.572867	0.5931	0.1605	0.688862		0.0079	0.0037962321
ss61514950	*rs41617203*	11	60.595379	0.2557	0.4154	0.519458		0.0135	0.0051498510
ss105238199		11	60.662786	0.2099	0.3517	0.553338		0.0138	0.0045833892
ss86340431		11	60.747468	0.5458	0.0172	0.895632		0.0025	0.0012180503
ss86322021		11	60.788927	0.7149	0.6028	0.437792		0.0162	0.0065889072
ss61500719	*rs41599474*	11	60.831424	0.3281	0.0212	0.884182		0.0030	0.0013015536
ss64305450	*rs42575596*	11	60.87391	0.8460	0.9443	0.331523		0.0248	0.0064672320
ss86312311		11	60.977321	0.4613	1.5002	0.221051		0.0234	0.0116436279
	rs29016857	11	61.027589	0.2185	7.7787	0.005431		0.0642	0.0219138269
	rs29026371	11	61.053033	0.2278	1.0845	0.298053		0.0234	0.0082344103
	rs29014440	11	61.152082	0.8718	0.9061	0.341490		0.0263	0.0058860748
ss61568981	*rs41668260*	11	61.190539	0.4155	0.6727	0.412402		0.0158	0.0076729315
ss65598576	*rs43681489*	11	61.223697	0.5573	0.4520	0.501587		0.0131	0.0064859912
ss65598617	*rs43681530*	11	61.254258	0.7106	0.7342	0.391830		0.0175	0.0071929244
ss64774230	*rs42867796*	11	61.29872	0.7099	0.8620	0.353508		0.0189	0.0077797407
ss64648069	*rs42738768*	11	61.400945	0.0501	8.7329	0.003231		0.1249	0.0119011531
ss64963565	*rs43050088*	11	61.504579	0.9234	0.9522	0.329497		0.0338	0.0047795978
ss64670153	*rs42769122*	11	61.558407	0.4219	1.4640	0.226712		0.0226	0.0110286184
ss64347059	*rs42594645*	11	61.70688	0.0781	0.6967	0.404177		0.0287	0.0041307405
ss61507476	*rs41606669*	11	61.810053	0.5337	1.7831	0.182209		0.0252	0.0125578455
	rs29015825	11	61.852403	0.1010	0.0099	0.920796		0.0032	0.0005757187
ss65598172	*rs43683582*	11	61.968479	0.4234	4.9606	0.026250		0.0412	0.0201156854
	rs29018191	11	62.088126	0.6261	2.2550	0.133640		0.0296	0.0138584575
ss61569924	*rs41671173*	11	62.209956	0.0544	5.6832	0.017395		0.0975	0.0100405609
ss65598259	*rs43678073*	11	62.262108	0.5100	2.7309	0.098877		0.0311	0.0155612943
ss65598298	*rs43678112*	11	62.286213	0.8467	0.2493	0.617743		0.0129	0.0033581633
ss61532465	*rs41625879*	11	62.332414	0.0731	11.0348	0.000941		0.1186	0.0160668168
ss86338485		11	62.360726	0.8453	0.4096	0.522371		0.0167	0.0043801449
ss86339688		11	62.391236	0.3625	5.6902	0.017327		0.0456	0.0210946962
ss46526432	*rs41256896*	11	62.457186	0.5043	3.9525	0.047192		0.0358	0.0179181114
	rs29022363	11	62.483015	0.5050	4.0832	0.043694		0.0364	0.0181834750
ss105237499		11	62.506278	0.5036	3.8014	0.051612		0.0351	0.0175496397

ss105237500		11	62.558008	0.5036	3.8014	0.051612		0.0351	0.0175496397
ss61569528	*rs41667907*	11	62.607566	0.3130	2.1557	0.142490		0.0291	0.0125063885
ss61569537	*rs41667912*	11	62.631862	0.7593	5.7844	0.016429		0.0509	0.0186074619
ss61497225	*rs41595254*	11	62.661693	0.8610	6.9300	0.008664		0.0683	0.0163347997
ss86309392		11	62.715842	0.0874	0.2082	0.648355		0.0149	0.0023810679
ss61569544	*rs41667919*	11	62.765495	0.1340	0.0520	0.819664		0.0063	0.0014622528
ss61569547	*rs41667922*	11	62.901962	0.5516	7.5187	0.006263		0.0513	0.0253655744
ss86318128		11	62.944112	0.5774	7.6875	0.005709		0.0518	0.0252631316
ss86340248		11	63.038527	0.3159	0.0118	0.913577		0.0022	0.0009450204
ss61530331	*rs41633408*	11	63.10255	0.7450	8.3297	0.004021		0.0610	0.0231646016
ss61520910	*rs41618429*	11	63.122706	0.6039	0.7700	0.380528		0.0168	0.0080313284
ss86309442		11	63.146471	0.3954	0.8353	0.361070		0.0175	0.0083509864
ss86308751		11	63.300765	0.3761	2.3256	0.127717		0.0285	0.0133741827
ss105238955		11	63.556147	0.8682	5.1519	0.023526		0.0638	0.0146006475
ss86309185		11	63.600221	0.3295	1.9385	0.164278		0.0273	0.0120640828
ss117970227		11	63.640435	0.4169	0.7368	0.390988		0.0161	0.0078332463
ss86282878		11	63.699169	0.7794	0.4867	0.485649		0.0158	0.0054254130
ss61512106	*rs41610116*	11	63.809765	0.3087	3.4136	0.065086		0.0377	0.0161025491
	rs29017459	11	63.830819	0.6913	3.4136	0.065086		0.0377	0.0161025491
ss61569590	*rs41668665*	11	63.854488	0.6913	3.4136	0.065086		0.0377	0.0161025491
ss61523029	*rs41623496*	11	63.907092	0.8668	20.6969	0.000006		0.1226	0.0283240059
ss86278244		11	63.962517	0.3037	0.6219	0.430604		0.0159	0.0067146265
ss61470145	*rs41567839*	11	63.983538	0.5294	0.9972	0.318343		0.0186	0.0092670025
ss117970373		11	64.050041	0.3030	0.5327	0.465708		0.0147	0.0062051839
ss61512118	*rs41610128*	11	64.095932	0.4778	1.9914	0.158642		0.0262	0.0130851789
ss61512116	*rs41610126*	11	64.224343	0.8725	2.5539	0.110474		0.0442	0.0098341830
ss61472412	*rs41570847*	11	64.301935	0.2615	8.4996	0.003666		0.0618	0.0238743366
ss86316630		11	64.325726	0.6039	2.9283	0.087485		0.0315	0.0150937533
ss86296238		11	64.366236	0.4162	0.5549	0.456559		0.0140	0.0068271150
	rs29027434	11	64.393473	0.4749	0.9094	0.340616		0.0176	0.0087965116
ss86303547		11	64.422135	0.8016	2.1571	0.142366		0.0346	0.0110159675
ss61505897	*rs41600028*	11	64.458023	0.2937	7.2014	0.007458		0.0544	0.0225741493
ss86296105		11	64.486273	0.7994	1.8544	0.173713		0.0318	0.0101897704
ss86339667		11	64.535799	0.1246	4.8446	0.028061		0.0612	0.0133572548
ss86306783		11	64.565098	0.4370	0.0000	0.999512		0.0000	0.0000056886
ss86336414		11	64.595162	0.4384	5.0739	0.024599		0.0414	0.0204015628
ss86302914		11	64.677706	0.2070	1.2818	0.257956		0.0262	0.0086026472
ss86274502		11	64.6983	0.3933	17.6699	0.000030		0.0790	0.0376956151
ss86285588		11	64.764274	0.7951	2.0662	0.151047		0.0333	0.0108403405
ss61512155	*rs41610465*	11	64.890147	0.8926	0.1782	0.673046		0.0127	0.0024332362
ss61497235	*rs41595264*	11	64.940274	0.1003	6.5396	0.010761		0.0773	0.0139507769
ss61497236	*rs41595265*	11	64.973083	0.7787	0.0389	0.843706		0.0045	0.0015463812
ss86339383		11	65.165954	0.2822	7.3028	0.007053		0.0554	0.0224541272
ss86332482		11	65.201531	0.7729	1.7409	0.187455		0.0284	0.0099803319
ss86333459		11	65.238914	0.3446	0.2308	0.631088		0.0092	0.0041693574
ss46527122	*rs29003479*	11	65.259158	0.9370	4.6630	0.031160		0.0832	0.0098232054
ss61569578	*rs41668653*	11	65.298769	0.3173	0.6757	0.411354		0.0163	0.0070469948

	rs29018917	11	65.336563	0.6612	2.8113	0.094054	0.0326	0.0145985837
ss61507929	*rs41599919*	11	65.363394	0.0652	3.5140	0.061270	0.0713	0.0086848191
ss38326864	*rs29015166*	11	65.405403	0.4491	6.5014	0.010992	0.0473	0.0233956829
ss61512156	*rs41610466*	11	65.518509	0.4205	0.8793	0.348706	0.0170	0.0082645715
ss86313399		11	65.591623	0.3789	0.8278	0.363227	0.0170	0.0080109065
ss86325205		11	65.632004	0.4971	1.0961	0.295490	0.0189	0.0094276059
ss86339997		11	65.671466	0.2106	0.1716	0.678864	0.0093	0.0030917073
ss105238966		11	65.750804	0.0903	6.1642	0.013271	0.0793	0.0130148671
ss86336971		11	65.79211	0.5530	0.7879	0.375052	0.0167	0.0082705283
ss117970367		11	65.840317	0.3940	2.2617	0.133060	0.0282	0.0134650241
ss86333854		11	65.871463	0.6053	2.0329	0.154373	0.0268	0.0127845289
ss61518883	*rs41618633*	11	65.893072	0.3503	3.4620	0.063217	0.0358	0.0163041011
ss86286271		11	65.954389	0.1970	4.4256	0.035761	0.0483	0.0152770280
ss86313491		11	65.993946	0.6468	0.4980	0.480596	0.0138	0.0063008115
ss86337920		11	66.039207	0.1948	6.5574	0.010655	0.0596	0.0186941238
ss86339165		11	66.083731	0.4907	0.2621	0.608830	0.0092	0.0046024827
ss86327068		11	66.137241	0.7908	0.1554	0.693576	0.0089	0.0029519639
ss61512596	*rs41613800*	11	66.203113	0.7034	1.6275	0.202475	0.0247	0.0102890044
ss86319497		11	66.306203	0.7622	1.8466	0.174618	0.0292	0.0105865961
ss86337593		11	66.393769	0.4219	0.2746	0.600452	0.0096	0.0046996603
ss105240352		11	66.531916	0.7958	2.1857	0.139747	0.0331	0.0107554465
ss86312180		11	66.632876	0.5466	3.4303	0.064433	0.0338	0.0167704151
ss86324859		11	66.692326	0.4119	0.0042	0.948175	0.0012	0.0005912358
ss86316875		11	66.854005	0.4019	3.0611	0.080628	0.0335	0.0161060954
ss86308236		11	66.920825	0.5150	0.1162	0.733314	0.0062	0.0030800345
ss61569856	*rs41670407*	11	67.112033	0.1848	11.9643	0.000575	0.0814	0.0245196316
ss86337722		11	67.140801	0.2550	0.6130	0.433934	0.0166	0.0063128528
ss86321096		11	67.321479	0.6261	0.7377	0.390706	0.0170	0.0079375707
ss38326777	*rs29015083*	11	67.393422	0.9491	0.1294	0.719198	0.0152	0.0014691763
ss86284952		11	67.484384	0.3725	0.0884	0.766356	0.0057	0.0026610906
ss86283095		11	67.514622	0.4362	0.9730	0.324278	0.0187	0.0092183926
	rs29022104	11	67.801574	0.2285	2.6727	0.102535	0.0367	0.0129331032
ss61570792	*rs41672342*	11	67.89916	0.5974	0.2849	0.593682	0.0100	0.0047965546
ss86304673		11	67.944329	0.8360	0.2173	0.641281	0.0117	0.0032035581
ss86283147		11	67.979738	0.4792	1.3945	0.238045	0.0217	0.0108541633
ss86285018		11	68.044964	0.9191	0.5908	0.442374	0.0256	0.0038061064
ss86314327		11	68.107197	0.6999	0.3175	0.573280	0.0118	0.0049435619
ss61470393	*rs41569380*	11	68.382981	0.6769	5.2441	0.022320	0.0459	0.0200572382
ss86335650		11	68.409781	0.3660	0.0379	0.845623	0.0038	0.0017619672
ss86292684		11	68.466275	0.2149	5.1126	0.024061	0.0504	0.0169993103
ss86293327		11	68.487251	0.7930	1.1364	0.286780	0.0247	0.0081155500
ss46526343	*rs41255482*	11	68.50815	0.7822	3.3858	0.066188	0.0416	0.0141563292
ss86338191		11	68.724453	0.5014	1.1364	0.286788	0.0197	0.0098576983
ss86339995		11	68.799991	0.1554	2.2024	0.138249	0.0377	0.0099051108
ss117964212		11	68.881873	0.1046	12.9023	0.000351	0.1084	0.0203022147
ss86309251		11	68.975191	0.5444	12.8431	0.000362	0.0656	0.0325625159
ss86311229		11	69.018007	0.5287	12.4568	0.000444	0.0638	0.0318003491

ss86295230		11	69.144294	0.6160	1.2654	0.261018	0.0214	0.0101110580
ss86331930		11	69.215459	0.4169	1.4966	0.221605	0.0229	0.0111338542
ss86338545		11	69.278464	0.1074	9.5318	0.002100	0.0926	0.0177533613
ss86305626		11	69.30534	0.7006	0.9761	0.323518	0.0204	0.0085523437
ss61525495	*rs41621108*	11	69.340896	0.4054	0.0012	0.971974	0.0007	0.0003241931
ss86282767		11	69.404306	0.3417	0.3963	0.529207	0.0122	0.0055048171
ss86325830		11	69.456452	0.6039	0.2942	0.587704	0.0102	0.0048613221
ss86325508		11	69.483664	0.6368	0.4872	0.485417	0.0132	0.0061122117
ss86313211		11	69.517431	0.7206	0.3071	0.579617	0.0116	0.0046685041
ss86336850		11	69.550822	0.3567	1.1419	0.285620	0.0205	0.0093952346
ss46527105	*rs41255648*	11	69.636923	0.3037	0.0515	0.820549	0.0046	0.0019402678
ss86295912		11	69.67123	0.6612	1.0501	0.305842	0.0201	0.0090183109
ss86338983		11	69.718192	0.3395	1.0936	0.296030	0.0205	0.0091912048
ss61470410	*rs41569396*	11	69.770646	0.4262	7.4766	0.006410	0.0521	0.0254676068
ss61470409	*rs41569395*	11	69.797654	0.6189	0.9964	0.318531	0.0192	0.0090728736
ss86279810		11	69.83024	0.2600	0.7405	0.389783	0.0183	0.0070367674
ss61512619	*rs41613823*	11	69.859816	0.1203	5.1711	0.023269	0.0638	0.0134996783
ss38329505	*rs29027118*	11	69.911994	0.2027	0.2910	0.589765	0.0121	0.0039178522
ss61470400	*rs41569387*	11	70.053573	0.4807	3.0346	0.081946	0.0314	0.0156627577
ss86336906		11	70.089956	0.8417	14.0843	0.000189	0.0931	0.0248175028
ss117970364		11	70.246468	0.9205	17.0712	0.000040	0.1409	0.0206228281
ss61562897	*rs41665984*	11	70.267783	0.2042	1.0567	0.304317	0.0233	0.0075710920
ss86340840		11	70.322049	0.8052	9.6521	0.001968	0.0724	0.0227015001
ss105241752		11	70.364979	0.3030	6.4246	0.011473	0.0500	0.0211037786
ss117970383		11	70.562038	0.2163	0.7478	0.387471	0.0195	0.0066213539
ss61470418	*rs41569404*	11	70.584249	0.7830	1.3202	0.250954	0.0258	0.0087812165
ss86323475		11	70.631705	0.3668	17.5299	0.000032	0.0827	0.0383908488
ss86303685		11	70.656665	0.6418	18.0120	0.000025	0.0836	0.0384153905
ss86317221		11	70.697189	0.1626	12.2839	0.000486	0.0861	0.0234382852
ss86336045		11	70.729046	0.4721	17.7167	0.000029	0.0776	0.0386989545
ss86340315		11	70.787792	0.4735	18.4838	0.000020	0.0792	0.0395001612
ss86274010		11	70.905567	0.3195	1.9395	0.164171	0.0270	0.0117322861
ss61513129	*rs41609923*	11	70.939641	0.1726	0.4700	0.493237	0.0172	0.0048999062
ss117970395		11	71.024437	0.3940	2.4988	0.114391	0.0304	0.0145261690
ss86337243		11	71.087025	0.5981	9.8235	0.001795	0.0602	0.0289225951
ss86308685		11	71.136076	0.3546	0.0097	0.921450	0.0020	0.0009006827
ss61470725	*rs41572400*	11	71.17053	0.6340	3.9781	0.046484	0.0386	0.0179174737
ss61497678	*rs43703402*	11	71.196525	0.8188	1.8313	0.176413	0.0320	0.0095005958
ss105241761		11	71.221316	0.1024	2.0642	0.151248	0.0425	0.0078108330
ss86300401		11	71.250973	0.6948	0.0926	0.760988	0.0060	0.0025600069
	rs29026395	11	71.283767	0.8682	4.6565	0.031277	0.0587	0.0134276982
ss105241762		11	71.358035	0.8438	2.1297	0.144920	0.0368	0.0096979151
ss86297868		11	71.396002	0.2951	1.2460	0.264698	0.0223	0.0092866944
ss86286555		11	71.449884	0.2342	0.0213	0.883996	0.0033	0.0011698865
ss86294427		11	71.506935	0.9019	0.3426	0.558518	0.0175	0.0030972618
ss86336577		11	71.544477	0.3159	0.4269	0.513744	0.0131	0.0056703802
ss86337224		11	71.571653	0.7042	0.2801	0.596830	0.0107	0.0044432424

ss86309175		11	71.617269	0.1211	3.1564	0.076066	0.0492	0.0104744672
ss61471600	*rs41571750*	11	71.649377	0.5681	18.6248	0.000018	0.0810	0.0397361476
ss61531764	*rs41629314*	11	71.817569	0.4312	0.6330	0.426527	0.0151	0.0074171942
ss61479048	*rs41579915*	11	71.998049	0.7521	8.2615	0.004173	0.0637	0.0237591312
ss86312075		11	72.047209	0.5759	1.2986	0.254866	0.0217	0.0106158305
ss86332742		11	72.088158	0.4377	0.8552	0.355409	0.0173	0.0085083108
ss86301351		11	72.11689	0.7679	3.4404	0.064043	0.0400	0.0142665399
ss61513608	*rs41613976*	11	72.173199	0.2393	0.2371	0.626480	0.0104	0.0037862587
ss86282783		11	72.281538	0.8331	0.2636	0.607789	0.0126	0.0035027526
ss86321760		11	72.419793	0.2815	0.0041	0.948942	0.0013	0.0005305361
ss86332866		11	72.518505	0.2006	1.2438	0.265122	0.0256	0.0081972334
ss86300406		11	72.57783	0.5251	0.2633	0.607999	0.0095	0.0047616361
ss86331009		11	72.626372	0.5487	3.2034	0.073922	0.0338	0.0167527542
ss117964204		11	72.730718	0.6325	0.0073	0.931978	0.0016	0.0007636642
ss86285471		11	72.757679	0.1311	0.3760	0.539938	0.0171	0.0038902537
ss86330185		11	72.803751	0.2292	0.0847	0.771077	0.0063	0.0022163923
ss61513139	*rs41610533*	11	72.890499	0.8274	0.0787	0.779213	0.0069	0.0019626262
ss38329374	*rs29026988*	11	73.096358	0.6705	14.0558	0.000192	0.0744	0.0328606747
ss86336974		11	73.124049	0.4248	12.9036	0.000351	0.0696	0.0339911413
ss86322411		11	73.17273	0.3847	2.7198	0.099561	0.0317	0.0149844608
ss86336137		11	73.280572	0.3832	15.1399	0.000109	0.0758	0.0358466024
ss86308488		11	73.342248	0.5903	1.4560	0.227981	0.0229	0.0110721988
ss86334761		11	73.454269	0.2314	0.0000	0.998387	0.0000	0.0000155006
ss86297777		11	73.479739	0.4040	6.8545	0.009034	0.0487	0.0234428538
ss86331020		11	73.507198	0.1934	1.6548	0.198739	0.0300	0.0093732990
	rs29022293	11	73.554366	0.4219	2.1343	0.144484	0.0281	0.0137046199
ss117970403		11	73.608905	0.1676	0.0901	0.764113	0.0073	0.0020409804
ss117970123		11	73.913795	0.8532	3.7797	0.052281	0.0508	0.0127314543
ss38327146	*rs29019258*	11	73.974302	0.1117	0.3028	0.582340	0.0159	0.0031652841
ss86296065		11	73.999712	0.0673	8.8995	0.002952	0.1078	0.0135361594
ss61514166	*rs41610339*	11	74.190346	0.1468	0.1581	0.691079	0.0103	0.0025915846
ss61475328		11	74.258955	0.7636	4.1889	0.041065	0.0440	0.0158960654
ss86337849		11	74.537775	0.0587	3.2890	0.070176	0.0720	0.0079616672
ss86340162		11	74.578376	0.1769	4.6545	0.031315	0.0531	0.0154631423
ss86335523		11	74.62616	0.7923	8.3048	0.004076	0.0670	0.0220463708
ss86317647		11	74.657888	0.5122	6.3827	0.011745	0.0463	0.0231564582
ss86335542		11	74.699948	0.6798	6.1551	0.013338	0.0487	0.0212119637
ss86336163		11	74.835007	0.0408	0.5644	0.452753	0.0356	0.0027875050
ss61471291	*rs41569261*	11	74.869822	0.7994	0.2545	0.614059	0.0114	0.0036637536
ss86310199		11	74.911454	0.0946	0.0034	0.953858	0.0018	0.0003047979
ss61465532		11	74.947309	0.7457	1.6724	0.196361	0.0279	0.0105728763
ss86339629		11	74.967675	0.1490	0.0303	0.861958	0.0045	0.0011379772
ss86296994		11	75.003531	0.2228	10.6444	0.001158	0.0737	0.0255365364
ss86337101		11	75.043203	0.6941	10.6137	0.001177	0.0648	0.0275322188
ss86305107		11	75.076327	0.4033	0.3354	0.562702	0.0111	0.0053317823
ss86299633		11	75.120933	0.4004	0.0087	0.925846	0.0017	0.0008315002
ss86315477		11	75.148414	0.1884	9.8685	0.001753	0.0712	0.0217618254

ss86314196		11	75.175001	0.4047	0.1777	0.673512	0.0078	0.0037659279
ss86305908		11	75.198264	0.3746	3.5910	0.058508	0.0359	0.0168386390
ss86315102		11	75.22577	0.3489	5.4334	0.020040	0.0444	0.0201624399
ss86336303		11	75.257053	0.3489	5.4334	0.020040	0.0444	0.0201624399
ss46526046	*rs41255193*	11	75.321344	0.5301	3.0492	0.081219	0.0318	0.0158553105
ss86325909		11	75.411692	0.4470	6.1338	0.013499	0.0446	0.0220686086
ss86338807		11	75.526718	0.6347	2.3270	0.127598	0.0289	0.0134102924
ss86305573		11	75.573344	0.3474	4.8439	0.028072	0.0419	0.0189850855
ss117970203		11	75.896527	0.3847	0.1450	0.703446	0.0076	0.0036099657
ss61471285	*rs41569255*	11	75.966912	0.0272	2.4408	0.118668	0.0889	0.0047085074
ss86273987		11	76.077797	0.2794	4.3640	0.037069	0.0438	0.0176451675
ss86327534		11	76.122799	0.5544	0.0034	0.953262	0.0011	0.0005324905
ss86335929		11	76.156529	0.3331	0.5182	0.471849	0.0147	0.0065222002
ss86332923		11	76.226622	0.1741	0.0737	0.786157	0.0067	0.0019268912
ss117970389		11	76.264191	0.4799	0.1257	0.723030	0.0069	0.0034454315
ss86323653		11	76.286799	0.6433	0.4320	0.511240	0.0133	0.0060987223
ss61514173	*rs41610346*	11	76.309237	0.3610	0.4040	0.525247	0.0129	0.0059380551
ss86338452		11	76.345903	0.2930	0.9166	0.338707	0.0188	0.0078070088
ss86320264		11	76.449085	0.7830	0.2210	0.638399	0.0108	0.0036590496
ss61514168	*rs41610341*	11	76.58192	0.4441	1.3929	0.238314	0.0217	0.0107236305
ss105237565		11	76.698423	0.8030	5.9705	0.014795	0.0558	0.0176485023
	rs29023299	11	76.744039	0.2364	3.6530	0.056380	0.0424	0.0153059987
ss86314247		11	76.834226	0.7944	0.8674	0.352006	0.0218	0.0071337767
ss61526354	*rs41623051*	11	76.957316	0.9377	1.7820	0.182345	0.0518	0.0060521902
ss86300001		11	77.150323	0.2443	5.6573	0.017652	0.0503	0.0185770311
ss86324517		11	77.200036	0.5981	0.9001	0.343074	0.0177	0.0085040445
ss61514694	*rs41615355*	11	77.464588	0.8102	0.0309	0.860433	0.0041	0.0012538195
ss61514693	*rs41615354*	11	77.501148	0.2958	15.6186	0.000085	0.0810	0.0337387451
ss86340313		11	77.533955	0.1160	1.2612	0.261818	0.0331	0.0067823731
ss86337284		11	77.557161	0.6468	8.5926	0.003486	0.0567	0.0259204874
ss86316887		11	77.586117	0.7006	4.3176	0.038085	0.0409	0.0171614696
ss86279986		11	77.666197	0.3503	0.0767	0.781840	0.0054	0.0024733478
ss86289606		11	77.713871	0.6977	10.1670	0.001494	0.0641	0.0270483477
ss86337777		11	77.738782	0.8274	11.6779	0.000669	0.0840	0.0239916854
ss86308955		11	77.762764	0.8087	0.0836	0.772586	0.0069	0.0021218811
ss86341124		11	77.786623	0.1340	7.7655	0.005470	0.0763	0.0177110576
ss86330019		11	77.807112	0.1318	7.6668	0.005774	0.0767	0.0175512323
ss86340942		11	77.829145	0.5093	7.5977	0.005997	0.0513	0.0256646209
ss86284072		11	77.903805	0.1583	0.0257	0.872740	0.0040	0.0010608041
ss86284025		11	77.96693	0.7679	7.1844	0.007528	0.0582	0.0207566188
ss61514702		11	78.179408	0.2564	0.2874	0.592060	0.0111	0.0042430961
ss117970164		11	78.221462	0.9448	3.4689	0.062953	0.0770	0.0080266511
ss86337368		11	78.256748	0.6447	19.5610	0.000011	0.0828	0.0379130776
ss86289943		11	78.305386	0.7729	23.9628	0.000001	0.1049	0.0368131854
ss61514708	*rs41615369*	11	78.410148	0.2521	3.4404	0.064043	0.0389	0.0146804348
ss86341413		11	78.484412	0.4305	4.4721	0.034808	0.0392	0.0192028325
ss86296405		11	78.571065	0.0666	3.9072	0.048473	0.0736	0.0091563401

	rs29023108	11	78.704511	0.9097	7.1044	0.007868	0.0873	0.0143422108
ss61514721	*rs41615380*	11	78.739348	0.5451	0.0180	0.893330	0.0025	0.0012548539
ss61506202	*rs41603420*	11	78.837757	0.6877	0.0927	0.760894	0.0061	0.0026407763
ss86296668		11	78.87184	0.8589	3.9882	0.046209	0.0543	0.0131541025
ss61471516	*rs41570975*	11	78.896956	0.2256	2.9786	0.084816	0.0384	0.0134216443
ss61471528	*rs41570987*	11	78.954849	0.0509	5.3176	0.021404	0.0972	0.0093852844
ss86323667		11	79.119149	0.9033	3.1467	0.076519	0.0564	0.0098460155
ss61515199	*rs41612857*	11	79.139921	0.0938	2.9751	0.084999	0.0554	0.0094223171
ss61515202	*rs41612860*	11	79.285457	0.5129	0.1897	0.663300	0.0084	0.0042140586
ss117970342		11	79.307476	0.4835	1.1166	0.291024	0.0204	0.0102024567
ss105298143		11	79.328409	0.2120	9.2559	0.002436	0.0692	0.0231240683
ss61465621	*rs41567966*	11	79.366328	0.0645	1.3973	0.237574	0.0448	0.0054030197
ss117970370		11	79.443336	0.3825	6.6228	0.010274	0.0491	0.0232159526
ss61515216	*rs41612874*	11	79.537103	0.3524	1.1505	0.283807	0.0217	0.0099183293
ss86310189		11	79.559694	0.2192	9.6174	0.002005	0.0705	0.0241193018
ss86334606		11	79.610835	0.2744	1.0230	0.312171	0.0217	0.0086406665
ss86286719		11	79.700422	0.6991	12.0222	0.000558	0.0713	0.0299895950
ss86319510		11	79.767242	0.3625	5.3047	0.021561	0.0459	0.0212176123
ss86296085		11	79.839244	0.0860	4.1272	0.042580	0.0649	0.0102019682
ss86338701		11	79.875432	0.4871	0.2204	0.638850	0.0090	0.0044742841
ss117964179		11	79.940792	0.2851	4.3576	0.037208	0.0437	0.0177954163
ss86289813		11	79.968686	0.2693	3.5079	0.061497	0.0413	0.0162589739
ss86339948		11	80.090818	0.7213	12.2903	0.000485	0.0751	0.0301841497
ss86278121		11	80.117227	0.5709	2.8722	0.090567	0.0335	0.0164135453
ss86274362		11	80.141588	0.1225	4.6640	0.031142	0.0599	0.0128712162
ss86322704		11	80.251124	0.7672	10.3188	0.001378	0.0714	0.0255092530
ss86291814		11	80.363013	0.1547	0.2058	0.650201	0.0117	0.0030708431
ss86298343		11	80.404051	0.3539	0.2453	0.620571	0.0102	0.0046704723
ss86301250		11	80.436289	0.8087	0.3266	0.567861	0.0136	0.0041928919
ss86303197		11	80.575881	0.7285	3.5168	0.061169	0.0393	0.0155611935
ss86337785		11	80.606407	0.2865	2.7110	0.100110	0.0357	0.0145877100
ss86289301		11	80.662877	0.4341	9.7842	0.001834	0.0577	0.0283293965
ss86335555		11	80.705996	0.2915	2.6890	0.101495	0.0349	0.0144364362
ss117970208		11	80.754474	0.2285	6.1490	0.013385	0.0556	0.0195940856
ss86312514		11	80.795966	0.2307	0.1088	0.741648	0.0073	0.0025827794
ss61515739	*rs41616688*	11	80.830864	0.3883	0.0971	0.755375	0.0062	0.0029226406
ss117970131		11	80.933101	0.4986	0.0128	0.909880	0.0023	0.0011275613
ss86336169		11	80.972661	0.8324	0.0291	0.864658	0.0043	0.0011905709
ss86316283		11	80.995533	0.3832	11.1057	0.000906	0.0643	0.0303876820
ss86337805		11	81.016157	0.6003	12.0245	0.000557	0.0669	0.0321153439
ss86284215		11	81.07337	0.3818	11.5355	0.000722	0.0654	0.0308559974
ss86324016		11	81.118499	0.9319	1.4819	0.223894	0.0450	0.0057094034
ss86296531		11	81.291884	0.3847	14.1908	0.000179	0.0722	0.0341651391
ss86340574		11	81.328792	0.5931	14.2692	0.000172	0.0727	0.0351088255
ss86316503		11	81.349776	0.2235	6.2061	0.012963	0.0568	0.0197309119
ss86337006		11	81.369877	0.1719	0.0103	0.919130	0.0025	0.0007237297
ss86293912		11	81.425348	0.2049	0.0073	0.931799	0.0020	0.0006675136

ss86339346		11	81.449634	0.5817	2.2270	0.136070	0.0292	0.0142170307
ss86315686		11	81.946316	0.5444	6.2480	0.012663	0.0470	0.0232951257
ss117970206		11	82.103452	0.8252	4.9769	0.026006	0.0534	0.0153918286
ss61465681	*rs41568025*	11	82.162856	0.1153	10.8200	0.001055	0.0932	0.0190186490
ss61506276	*rs41604491*	11	82.339004	0.4620	0.7029	0.402108	0.0159	0.0078795635
ss61495003	*rs41592487*	11	82.646768	0.1941	0.1677	0.682253	0.0098	0.0030507782
ss61564949	*rs41664711*	11	82.669609	0.3410	0.5246	0.469135	0.0141	0.0063357808
	rs29011511	11	82.69145	0.7493	1.0288	0.310792	0.0216	0.0081260778
	rs29011508	11	82.715272	0.2364	0.5036	0.478168	0.0154	0.0055728589
ss86314078		11	82.739305	0.2142	0.0949	0.758185	0.0068	0.0022871169
ss86326064		11	82.789903	0.2135	0.1121	0.737911	0.0074	0.0024806577
ss86337419		11	82.854748	0.5838	0.4514	0.501900	0.0131	0.0063453329
ss86325614		11	82.913459	0.2858	2.6487	0.104090	0.0332	0.0135515758
ss105236824		11	82.938261	0.1619	1.4063	0.236079	0.0301	0.0081649351
ss105242521		11	82.998033	0.5158	4.9072	0.027068	0.0408	0.0203694113
ss61515923	*rs41617764*	11	83.502678	0.3302	0.0704	0.790875	0.0053	0.0023243610
ss61515922	*rs41617763*	11	83.588208	0.3968	2.8879	0.089693	0.0331	0.0158528875
ss105298676		11	83.688145	0.6683	6.7305	0.009677	0.0511	0.0226721953
	rs29019237	11	83.712431	0.6583	6.8331	0.009142	0.0505	0.0227040962
ss86282485		11	83.850463	0.6461	0.0103	0.919220	0.0020	0.0008952246
ss86310954		11	83.876376	0.6383	0.2068	0.649398	0.0090	0.0041378545
	rs29013358	11	83.918982	0.3123	2.4103	0.120992	0.0319	0.0136961427
ss86274954		11	83.979735	0.5967	0.0347	0.852268	0.0035	0.0017012438
ss86311232		11	84.270758	0.4319	0.0302	0.862047	0.0031	0.0015364076
ss86290586		11	84.316839	0.7715	0.0176	0.894535	0.0029	0.0010314753
ss86327584		11	84.358919	0.1411	4.3304	0.037802	0.0559	0.0135561382
ss86317308		11	84.460203	0.1984	1.8145	0.178408	0.0322	0.0102547558
ss86327967		11	84.534424	0.2930	0.3429	0.558335	0.0117	0.0048454428
ss61525505	*rs41621118*	11	84.611489	0.5544	1.0171	0.313552	0.0191	0.0094177093
ss61521087	*rs41620105*	11	84.95622	0.1497	0.0594	0.807476	0.0065	0.0016598148
ss61472097	*rs41567637*	11	85.222808	0.0903	2.7391	0.098370	0.0534	0.0087662200
ss61472098	*rs41567638*	11	85.254772	0.5064	4.8798	0.027498	0.0418	0.0209206621
ss61499007		11	85.352174	0.3259	1.0064	0.316106	0.0195	0.0085540277
ss86323559		11	85.374683	0.6748	1.0644	0.302583	0.0200	0.0087818635
ss86324220		11	85.461371	0.1304	0.0023	0.961497	0.0013	0.0002996732
ss86317676		11	85.499845	0.5867	0.0107	0.917632	0.0019	0.0009271603
	rs29014077	11	85.523418	0.8374	11.7146	0.000656	0.0864	0.0235171389
ss86327836		11	85.547736	0.8431	11.3496	0.000796	0.0862	0.0227969136
ss86292486		11	85.599588	0.6870	3.3984	0.065684	0.0360	0.0154965126
ss86318133		11	85.691544	0.0931	6.2461	0.012676	0.0776	0.0131093178
ss86340461		11	85.725138	0.6347	1.0678	0.301795	0.0197	0.0091410067
ss86335727		11	85.811718	0.7514	0.7343	0.391803	0.0177	0.0066002926
ss86328620		11	85.878552	0.3825	1.9861	0.159192	0.0274	0.0129380193
ss86338063		11	86.01953	0.4140	1.0318	0.310097	0.0190	0.0092161809
ss61515002	*rs41611465*	11	86.116625	0.3825	0.1554	0.693555	0.0076	0.0035817877
ss86338970		11	86.175257	0.4019	0.1127	0.737234	0.0063	0.0030073586
ss86294383		11	86.227752	0.3123	2.1941	0.138994	0.0296	0.0127330494

ss86323102		11	86.250898	0.9112	4.6589	0.031234	0.0699	0.0113148092
ss86318115		11	86.326269	0.5659	0.0128	0.909923	0.0021	0.0010548119
ss86331592		11	86.386387	0.8123	3.4506	0.063652	0.0418	0.0127441975
ss86315750		11	86.478142	0.3768	0.7951	0.372882	0.0165	0.0077565664
ss105305755		11	86.513667	0.1468	4.6839	0.030786	0.0551	0.0138040870
	rs29026760	11	86.540548	0.2106	2.1614	0.141965	0.0327	0.0108621935
ss61516345	*rs41614761*	11	86.685991	0.4484	4.6301	0.031758	0.0398	0.0196957252
ss86288158		11	87.183793	0.3138	0.0027	0.958668	0.0010	0.0004331325
ss61530692	*rs41627663*	11	87.371209	0.3739	3.0878	0.079320	0.0325	0.0152022649
ss61530691	*rs41627662*	11	87.515038	0.3883	12.7392	0.000383	0.0699	0.0331944156
ss86335227		11	87.559027	0.3001	1.3192	0.251128	0.0225	0.0094606879
ss86315097		11	87.604665	0.1261	0.0503	0.822648	0.0064	0.0014004645
ss86313744		11	87.631595	0.7607	0.1646	0.685109	0.0088	0.0032211340
ss117970214		11	87.6802	0.8961	0.0072	0.932301	0.0026	0.0004756249
ss86303809		11	87.706913	0.0788	0.3108	0.577369	0.0193	0.0028009456
	rs29012044	11	87.730075	0.6146	3.6914	0.055103	0.0362	0.0171304155
ss61523041	*rs41623508*	11	87.751411	0.3947	0.4007	0.526961	0.0119	0.0056889864
	rs29015690	11	87.885712	0.3524	2.0151	0.156185	0.0279	0.0127443749
ss61549540	*rs41649534*	11	87.924518	0.6060	1.0937	0.296018	0.0199	0.0095180137
ss86314330		11	87.947944	0.1046	2.2284	0.135951	0.0462	0.0086452061
ss86281383		11	88.002622	0.7092	6.8651	0.008981	0.0534	0.0220172057
ss61522157	*rs41617660*	11	88.023316	0.2830	6.2282	0.012804	0.0513	0.0207964966
ss86322192		11	88.060001	0.1153	0.0255	0.873126	0.0048	0.0009843207
ss86339159		11	88.099178	0.6497	2.1112	0.146677	0.0279	0.0126845495
ss86306106		11	88.177694	0.2844	0.0025	0.960226	0.0010	0.0004221084
	rs29017217	11	88.337688	0.3159	5.0375	0.025118	0.0443	0.0191363124
ss86339646		11	88.440906	0.1920	6.7775	0.009428	0.0622	0.0193117930
ss61516765	*rs41618766*	11	88.479672	0.1734	5.0078	0.025549	0.0553	0.0158604695
ss61516757		11	88.606997	0.6146	2.1310	0.144796	0.0282	0.0133526252
ss61523043	*rs41623510*	11	88.640367	0.2629	4.2732	0.039088	0.0414	0.0160447674
ss86293443		11	88.687506	0.2894	0.1456	0.702878	0.0077	0.0031479628
ss86327140		11	88.746532	0.5186	9.5730	0.002054	0.0564	0.0281497916
ss86310267		11	88.769932	0.7980	1.7624	0.184755	0.0305	0.0098449768
ss86338514		11	88.794225	0.6597	0.0074	0.931623	0.0017	0.0007683573
ss86319382		11	89.131027	0.1590	3.4838	0.062392	0.0468	0.0125309861
ss61516768	*rs41618769*	11	89.171145	0.7314	6.4910	0.011056	0.0552	0.0216959567
ss86329983		11	89.230768	0.8395	3.6755	0.055626	0.0478	0.0128687900
	rs29021240	11	89.343247	0.8403	3.3898	0.066025	0.0464	0.0124545166
ss86300557		11	89.386267	0.1605	3.6755	0.055626	0.0478	0.0128687900
ss86334732		11	89.426236	0.2966	0.7195	0.396591	0.0176	0.0073421269
ss86300513		11	89.481529	0.2063	2.9546	0.086076	0.0400	0.0130906585
ss86337761		11	89.521636	0.3524	5.1316	0.023801	0.0441	0.0201188660
ss86337977		11	89.548481	0.7980	3.5647	0.059436	0.0427	0.0137761595
ss86338542		11	89.572604	0.4570	0.5855	0.444436	0.0147	0.0072729051
ss86287224		11	89.634698	0.7350	2.3653	0.124512	0.0334	0.0130112419
ss86277732		11	89.657153	0.6211	0.7623	0.382915	0.0170	0.0079785215
ss86308010		11	89.695127	0.5358	1.8452	0.174780	0.0247	0.0122787303

ss86291936		11	89.733674	0.6318	0.1935	0.660161	0.0085	0.0039395499
ss105305932		11	89.848111	0.5544	2.5828	0.108482	0.0300	0.0147977530
ss86305091		11	89.890707	0.3295	1.1941	0.274890	0.0223	0.0098457068
ss86339029		11	89.9546	0.1884	1.1851	0.276697	0.0256	0.0078398790
ss61517321	*rs41613910*	11	89.976013	0.6361	0.0373	0.846837	0.0037	0.0017271671
	rs29022201	11	90.032675	0.4133	0.1416	0.706797	0.0073	0.0035535446
ss86297166		11	90.061222	0.5874	0.1014	0.750284	0.0059	0.0028561837
ss86338907		11	90.115635	0.3044	5.3304	0.021249	0.0464	0.0196448264
ss86294479		11	90.136929	0.6476	0.4942	0.482291	0.0138	0.0063199263
ss86291960		11	90.177375	0.6683	0.0059	0.938655	0.0015	0.0006633359
ss86320347		11	90.201767	0.0659	1.7626	0.184735	0.0497	0.0061232837
ss86307612		11	90.225484	0.0974	0.2333	0.629276	0.0150	0.0026348515
ss86332138		11	90.252505	0.5122	0.1046	0.746458	0.0059	0.0029273513
ss105242476		11	90.29519	0.8510	0.0296	0.863441	0.0044	0.0011161668
ss86313145		11	90.399339	0.3245	0.0334	0.855043	0.0036	0.0015674289
ss86333311		11	90.43479	0.7156	7.7065	0.005650	0.0576	0.0234519224
ss61472837	*rs41574556*	11	90.465927	0.4384	1.6693	0.196786	0.0249	0.0122827094
ss61465831	*rs41569975*	11	90.487328	0.2643	2.4706	0.116445	0.0335	0.0130328871
ss86303135		11	90.542083	0.1884	2.3858	0.122896	0.0360	0.0110046055
ss61517857	*rs41619120*	11	90.567426	0.1748	0.1609	0.688413	0.0098	0.0028217313
ss86338931		11	90.588405	0.2593	0.1333	0.715124	0.0079	0.0030423107
ss86301212		11	90.618947	0.2034	0.5103	0.475270	0.0167	0.0053964397
ss86286412		11	90.676507	0.3489	0.6348	0.425860	0.0151	0.0068749326
ss86313983		11	90.708825	0.6411	0.7130	0.398755	0.0159	0.0073125061
ss86335991		11	90.730972	0.5165	1.4497	0.228981	0.0214	0.0107129172
ss86338348		11	90.767263	0.3059	4.3244	0.037935	0.0419	0.0177729118
ss105242466		11	90.794233	0.1819	0.2153	0.642771	0.0112	0.0033225327
ss86330643		11	90.823124	0.4062	0.1320	0.716522	0.0067	0.0032319665
ss86341291		11	90.847737	0.6426	5.1342	0.023765	0.0436	0.0200489502
ss86335697		11	90.868063	0.6948	3.3015	0.069648	0.0365	0.0154627550
ss86289284		11	90.889956	0.7794	1.5957	0.206937	0.0282	0.0096925814
ss86287689		11	90.949099	0.2113	1.6789	0.195495	0.0293	0.0097574650
ss86323009		11	91.050646	0.2586	0.9808	0.322350	0.0204	0.0078218603
ss117970218		11	91.163046	0.3532	0.2066	0.649592	0.0090	0.0041219268
ss86291085		11	91.206852	0.2493	2.2395	0.134974	0.0323	0.0121017586
ss86322318		11	91.22829	0.7507	2.2395	0.134974	0.0323	0.0121017586
ss105238263		11	91.328455	0.6139	0.1393	0.709044	0.0071	0.0033644223
ss86315297		11	91.414642	0.2006	0.6497	0.420508	0.0189	0.0060453559
ss86308592		11	91.506184	0.6268	4.8690	0.027669	0.0419	0.0196105930
ss86299166		11	91.539107	0.3818	7.2438	0.007285	0.0515	0.0243055791
ss86316764		11	91.696402	0.1583	1.7712	0.183671	0.0341	0.0090753767
ss86313685		11	91.819727	0.8761	8.1967	0.004323	0.0800	0.0173696362
ss86324769		11	91.877383	0.8625	3.7767	0.052374	0.0528	0.0125257342
ss61517858	*rs41619121*	11	91.933413	0.4921	0.0472	0.828009	0.0040	0.0020231690
ss86279442		11	91.993553	0.2414	0.6509	0.420082	0.0173	0.0063515597
ss86309822		11	92.03474	0.5638	0.0060	0.938481	0.0014	0.0006962247
ss61526717	*rs41625603*	11	92.077933	0.9234	1.3532	0.245116	0.0407	0.0057541873

ss86337614		11	92.137483	0.6390	1.7606	0.184981	0.0252	0.0116361159
ss117970292		11	92.170518	0.6741	1.8641	0.172591	0.0278	0.0122324228
ss86327591		11	92.335772	0.1225	2.2898	0.130678	0.0426	0.0091601546
ss86335712		11	92.390239	0.7944	0.0064	0.936450	0.0018	0.0005932713
	rs29018501	11	92.466054	0.6074	5.4014	0.020408	0.0450	0.0214595090
ss61478336	*rs41575736*	11	92.495514	0.3782	0.0860	0.769399	0.0056	0.0026455499
ss86336682		11	92.520368	0.2006	0.1147	0.734999	0.0076	0.0024319776
ss86289182		11	92.609679	0.3868	0.4623	0.496784	0.0128	0.0060542933
ss86288469		11	92.719286	0.7307	5.4094	0.020315	0.0484	0.0190595128
ss86303795		11	92.821073	0.3403	0.8259	0.363769	0.0171	0.0076551605
	rs29024194	11	92.947758	0.8274	5.7653	0.016607	0.0588	0.0168076227
ss61473289	*rs41568808*	11	92.97902	0.4857	0.0678	0.794690	0.0049	0.0024238244
ss86336880		11	93.023361	0.5372	0.0700	0.791428	0.0049	0.0024602788
ss86309953		11	93.063751	0.2285	9.5848	0.002041	0.0658	0.0232063756
ss61473286		11	93.12896	0.5351	0.0622	0.803094	0.0046	0.0022872480
	rs29014432	11	93.192122	0.8130	4.8215	0.028436	0.0539	0.0163874579
ss86292849		11	93.213401	0.4950	0.5013	0.479181	0.0130	0.0065184494
ss86316004		11	93.26492	0.3990	3.7502	0.053206	0.0365	0.0175207095
ss86322181		11	93.756439	0.2471	0.0004	0.984205	0.0004	0.0001556877
ss86330246		11	93.893436	0.3553	0.0916	0.762231	0.0057	0.0026099794
	rs29016418	11	94.152392	0.7944	0.1477	0.700882	0.0089	0.0028984108
ss86316722		11	94.249574	0.6848	0.3373	0.561596	0.0117	0.0050648694
ss105240379		11	94.292987	0.5723	8.4260	0.003816	0.0558	0.0273301566
ss86324451		11	94.481451	0.3961	0.4912	0.483635	0.0128	0.0061207414
ss61521698		11	94.502041	0.1340	0.1188	0.730395	0.0094	0.0021696573
ss61521696		11	94.543421	0.3066	3.8648	0.049706	0.0382	0.0162473558
ss86336948		11	94.567508	0.6110	0.2924	0.588844	0.0099	0.0046969839
ss86295741		11	94.621163	0.4778	0.0423	0.837161	0.0037	0.0018636366
ss61519001		11	94.722394	0.3789	1.9208	0.166216	0.0263	0.0123606522
	rs29020979	11	94.746275	0.7142	5.4837	0.019476	0.0475	0.0193741572
ss86289799		11	94.839789	0.8395	1.3468	0.246242	0.0274	0.0073890948
ss117970071		11	94.925578	0.7385	1.6395	0.200823	0.0279	0.0107589499
ss117970252		11	95.011306	0.3202	1.3485	0.245947	0.0233	0.0101399773
ss86341115		11	95.044911	0.6583	1.8109	0.178841	0.0263	0.0118156758
ss61529919	*rs41629420*	11	95.069604	0.9105	0.0000	0.997356	0.0001	0.0000178514
ss61525823	*rs41622525*	11	95.107812	0.2113	0.4019	0.526329	0.0143	0.0047537437
ss117970179		11	95.134717	0.0938	0.0739	0.785799	0.0088	0.0015013650
ss86273787		11	95.214926	0.6870	1.5506	0.213463	0.0246	0.0105858376
ss86341446		11	95.240738	0.2701	0.7003	0.402974	0.0177	0.0069619592
ss86311365		11	95.26416	0.5559	0.6476	0.421261	0.0155	0.0076363388
ss61527802	*rs41626844*	11	95.330524	0.3145	0.5322	0.465930	0.0143	0.0061673588
ss117970376		11	95.382284	0.5086	1.0719	0.300879	0.0193	0.0096581457
ss86326186		11	95.55116	0.3066	2.8384	0.092485	0.0343	0.0145824671
ss86288807		11	95.587006	0.5616	3.3321	0.068369	0.0335	0.0165147102
ss86334652		11	95.649109	0.4670	3.5573	0.059700	0.0344	0.0171133151
ss86303746		11	95.680882	0.4893	3.7755	0.052412	0.0354	0.0176705752
ss86338847		11	95.778898	0.6468	3.8439	0.050326	0.0383	0.0174917447

ss86300106		11	95.81532	0.1991	8.9627	0.002853	0.0691	0.0220414253
ss86306390		11	96.003084	0.8840	0.1302	0.718363	0.0106	0.0021669201
ss86287762		11	96.053429	0.0838	2.5165	0.113116	0.0530	0.0081442568
ss86319447		11	96.076503	0.0817	1.8700	0.171912	0.0462	0.0069270621
ss61519585	*rs41616833*	11	96.113656	0.7937	0.2654	0.606579	0.0121	0.0039616695
ss86285167		11	96.173421	0.4434	5.7294	0.016947	0.0437	0.0215605125
ss86291287		11	96.36018	0.4062	14.2288	0.000176	0.0707	0.0341164478
ss86290863		11	96.408168	0.6447	7.5440	0.006177	0.0544	0.0249417275
ss117970118		11	96.473467	0.3990	2.5082	0.113708	0.0304	0.0145943022
ss86336946		11	96.653383	0.7414	5.1039	0.024180	0.0483	0.0185118360
ss61465996	*rs41565459*	11	96.687307	0.8589	1.8581	0.173285	0.0367	0.0088953801
ss86275267		11	96.70776	0.5910	8.3469	0.003984	0.0551	0.0266239789
ss86331711		11	96.733242	0.3653	2.3399	0.126551	0.0298	0.0138186136
ss61474026	*rs41566638*	11	97.241029	0.7085	10.9987	0.000959	0.0668	0.0275800128
ss61474028	*rs41566640*	11	97.269752	0.2923	11.5874	0.000702	0.0685	0.0283417853
ss86313436		11	97.395635	0.1110	0.1078	0.742705	0.0098	0.0019408531
ss86278341		11	97.500533	0.1096	0.1907	0.662457	0.0131	0.0025628375
ss61474029	*rs41566641*	11	97.671701	0.3689	9.1561	0.002570	0.0596	0.0277385111
ss86300336		11	97.719225	0.2292	1.3329	0.248689	0.0254	0.0089756579
ss86337831		11	97.78377	0.8875	2.1621	0.141905	0.0428	0.0085381433
ss86329175		11	97.803776	0.1117	2.3743	0.123799	0.0449	0.0089130400
ss61520112	*rs41611044*	11	97.830382	0.8840	2.2558	0.133567	0.0430	0.0088140765
	rs29022984	11	97.865487	0.8195	2.4456	0.118313	0.0379	0.0112250026
ss86337353		11	97.912669	0.5638	0.1371	0.711296	0.0071	0.0034929022
ss38327008	*rs29015306*	11	97.938855	0.2872	1.9081	0.167618	0.0284	0.0116402540
ss86337584		11	98.062903	0.2815	0.6013	0.438330	0.0160	0.0064889355
ss61506784	*rs41608279*	11	98.251596	0.5774	4.3573	0.037214	0.0389	0.0189908323
	rs29019732	11	98.372858	0.0989	2.0051	0.157214	0.0439	0.0078249525
ss86341567		11	98.435595	0.2493	0.1088	0.741576	0.0069	0.0025852865
ss86314311		11	98.502913	0.1024	0.4972	0.480989	0.0218	0.0040175294
ss86335949		11	98.545605	0.2507	0.3318	0.564768	0.0126	0.0047158061
ss86274584		11	98.603387	0.1977	4.1488	0.042043	0.0471	0.0149485298
ss86337798		11	98.6815	0.6547	2.1753	0.140690	0.0296	0.0134008042
ss86319906		11	98.890769	0.4119	0.0380	0.845431	0.0038	0.0018461439
ss86322688		11	98.923923	0.3933	1.2440	0.265079	0.0217	0.0103586124
ss86307801		11	98.947058	0.7572	0.1481	0.700461	0.0083	0.0030602514
ss86295624		11	98.973736	0.5057	0.3775	0.539135	0.0114	0.0056875578
	rs29019867	11	98.998436	0.8381	1.6212	0.203352	0.0311	0.0084266174
ss61519013	*rs41619651*	11	99.067717	0.3489	0.9898	0.320145	0.0194	0.0088321732
ss61519015	*rs41619653*	11	99.099357	0.8990	1.4781	0.224489	0.0369	0.0067090899
ss86335055		11	99.229749	0.2185	1.8884	0.169824	0.0311	0.0106041415
ss117970140		11	99.265743	0.3947	2.6194	0.106017	0.0318	0.0151844814
ss86333246		11	99.293871	0.0874	7.6886	0.005706	0.0937	0.0149426170
ss61523045	*rs41623512*	11	99.413511	0.6683	0.7212	0.396054	0.0175	0.0077792209
ss86303302		11	99.452165	0.2278	0.1039	0.747241	0.0074	0.0025918429
ss61520122	*rs41611054*	11	99.502416	0.0903	0.3781	0.538845	0.0202	0.0033203480
		11	99.614989	0.4491	0.2064	0.649746	0.0086	0.0042521301

ss86333925		11	99.663235	0.5895	0.2270	0.633924	0.0090	0.0043636260
ss86337415		11	99.711906	0.6748	2.1261	0.145261	0.0283	0.0124399140
ss86290761		11	99.747991	0.5330	1.9711	0.160778	0.0261	0.0129786604
ss86293750		11	99.847987	0.1769	1.5512	0.213378	0.0297	0.0086576561
	rs29017117	11	99.880986	0.2650	0.1594	0.689787	0.0083	0.0032377074
ss86280072		11	99.90543	0.3732	0.6143	0.433422	0.0152	0.0071162115
ss61547408	*rs41645935*	11	100.034815	0.5681	11.7231	0.000653	0.0665	0.0326236509
ss61547411	*rs41645938*	11	100.119137	0.6569	8.6201	0.003434	0.0587	0.0264700554
ss86317225		11	100.200662	0.1705	2.5348	0.111815	0.0397	0.0112174138
ss86337362		11	100.239887	0.5458	6.1608	0.013296	0.0479	0.0237666563
ss86291805		11	100.279631	0.3775	4.2722	0.039110	0.0406	0.0190976583
ss86341284		11	100.331153	0.2600	4.9730	0.026064	0.0478	0.0184003715
ss86297847		11	100.481884	0.3066	0.0000	0.996251	0.0001	0.0000403158
ss86292530		11	100.515387	0.4355	1.5484	0.213792	0.0230	0.0113038136
ss86340452		11	100.578778	0.2708	1.0735	0.300525	0.0224	0.0088530830
ss86292748		11	100.617734	0.6705	0.1741	0.676637	0.0081	0.0035856384
ss86329893		11	100.642125	0.3130	0.3308	0.565351	0.0115	0.0049460295
ss86287661		11	100.671216	0.1526	7.9349	0.004986	0.0730	0.0188869450
ss86273829		11	100.708282	0.9370	25.1316	0.000001	0.1882	0.0222282075
ss86340347		11	100.931764	0.3288	12.9051	0.000351	0.0708	0.0312635007
ss117964202		11	101.050321	0.7822	17.1867	0.000038	0.0916	0.0312226968
ss86330478		11	101.089356	0.1648	4.2556	0.039492	0.0503	0.0138502217
ss61519614	*rs41616861*	11	101.116817	0.1426	17.0642	0.000041	0.1099	0.0268543253
ss61465997	*rs41565460*	11	101.161121	0.1726	4.8049	0.028710	0.0530	0.0151350073
ss117964163		11	101.206486	0.4921	0.7339	0.391902	0.0156	0.0078009949
ss61519612	*rs41616859*	11	101.237154	0.7070	16.1317	0.000066	0.0824	0.0341390576
	rs29014970	11	101.292088	0.0938	4.3646	0.037055	0.0659	0.0112041934
ss86327822		11	101.331975	0.2693	0.9831	0.321789	0.0206	0.0080886702
ss86286639		11	101.35533	0.4871	0.3781	0.538817	0.0116	0.0058065985
ss86338888		11	101.383628	0.5222	0.7147	0.398185	0.0157	0.0078306077
ss86304915		11	101.449293	0.5552	1.5302	0.216498	0.0232	0.0114511505
ss86340633		11	101.473846	0.4441	1.6879	0.194304	0.0243	0.0120091728
ss61520694	*rs41616617*	11	101.622894	0.1426	3.5401	0.060320	0.0489	0.0119637678
ss105241804		11	101.648556	0.2758	2.1681	0.141356	0.0301	0.0120428497
ss86329672		11	101.670096	0.8883	0.0022	0.962360	0.0014	0.0002722020
ss86339918		11	101.728216	0.4112	1.6194	0.203597	0.0241	0.0116906760
ss117964200		11	101.897361	0.3231	0.1017	0.749871	0.0063	0.0027596142
ss86300118		11	101.934983	0.4979	0.3987	0.527963	0.0116	0.0057824488
ss105241809		11	101.956521	0.5014	0.4819	0.487784	0.0127	0.0063651699
ss86338609		11	101.977785	0.7242	6.3787	0.011770	0.0515	0.0205850413
ss86289253		11	102.052938	0.2364	1.9441	0.163673	0.0300	0.0108269307
ss86301220		11	102.083539	0.7543	0.1051	0.745937	0.0070	0.0026062053
ss86333086		11	102.123681	0.3532	5.7488	0.016763	0.0451	0.0206038201
ss86309186		11	102.155582	0.6361	1.5949	0.207055	0.0239	0.0110539617
ss86337885		11	102.196014	0.6347	1.7229	0.189757	0.0248	0.0115224473
ss86304922		11	102.251685	0.4255	0.3271	0.567580	0.0109	0.0053212560
ss86330733		11	102.295264	0.5860	9.3744	0.002285	0.0569	0.0275877896

ss86339737		11	102.321982	0.1153	0.3011	0.583389	0.0157	0.0032059869
ss86275043		11	102.422433	0.5723	1.7023	0.192421	0.0246	0.0120398339
ss61520697	*rs41616620*	11	102.444807	0.0638	0.0154	0.901337	0.0048	0.0005700788
ss86290928		11	102.484428	0.5616	0.3247	0.568998	0.0106	0.0051980083
ss86331914		11	102.586751	0.0766	0.0632	0.801628	0.0090	0.0012714673
ss61474304	*rs41568314*	11	102.68289	0.8216	1.2547	0.263040	0.0280	0.0081922658
ss86292681		11	102.73615	0.0731	0.0020	0.964392	0.0016	0.0002204097
ss86289087		11	102.781211	0.6311	1.2319	0.267428	0.0210	0.0097980140
ss86306577		11	102.812101	0.6311	1.2319	0.267428	0.0210	0.0097980140
ss46526923	*rs41257371*	11	102.944708	0.9026	0.0100	0.920361	0.0032	0.0005679383
ss86338811		11	103.078728	0.0924	0.2797	0.597086	0.0170	0.0028466485
ss61570119	*rs41664884*	11	103.162761	0.7844	1.7578	0.185335	0.0295	0.0099939634
ss86289402		11	103.184367	0.0602	0.0060	0.938101	0.0030	0.0003425573
ss86297860		11	103.212164	0.7973	2.1593	0.142160	0.0340	0.0109861373
ss86307468		11	103.375532	0.2923	0.0198	0.888091	0.0028	0.0011714415
ss86315277		11	103.515296	0.7543	0.7996	0.371532	0.0190	0.0070302772
ss86335293		11	103.552006	0.6110	5.3803	0.020654	0.0434	0.0206176216
ss86284872		11	103.592913	0.6633	0.0405	0.840554	0.0039	0.0017557126
ss86308237		11	103.644699	0.7536	5.0663	0.024707	0.0473	0.0175661686
ss86336082		11	103.69094	0.3152	3.4177	0.064925	0.0370	0.0159684573
ss86325381		11	103.711868	0.5344	0.0444	0.833172	0.0041	0.0020177036
ss86289150		11	103.747007	0.2586	0.1889	0.663943	0.0093	0.0035639223
ss86339025		11	103.767147	0.2156	9.3282	0.002343	0.0669	0.0226347766
ss86309802		11	103.809717	0.1963	0.5062	0.477027	0.0166	0.0052498213
ss61521220	*rs41618140*	11	103.874555	0.4957	2.2878	0.130849	0.0279	0.0139714187
ss86337444		11	103.909654	0.2865	1.6947	0.193416	0.0267	0.0109280304
ss86340850		11	103.951056	0.2342	2.1726	0.140936	0.0325	0.0116548016
ss86284426		11	103.971759	0.0960	8.4426	0.003782	0.0924	0.0160318156
ss86311005		11	104.056769	0.3961	0.0526	0.818611	0.0041	0.0019771298
ss86337181		11	104.158798	0.5466	4.3282	0.037852	0.0386	0.0191572517
ss86290366		11	104.205971	0.8152	2.2003	0.138436	0.0348	0.0104828557
ss117970398		11	104.231701	0.4355	0.0421	0.837440	0.0038	0.0018592708
ss86332609		11	104.259601	0.2543	5.5335	0.018933	0.0501	0.0189835649
ss86331407		11	104.449696	0.7557	5.9759	0.014749	0.0515	0.0190233172
ss86292834		11	104.486949	0.0759	2.7766	0.096104	0.0590	0.0082776910
ss86322612		11	104.507773	0.3574	1.1452	0.284920	0.0210	0.0096355468
ss86338288		11	104.533532	0.8775	10.1922	0.001474	0.0908	0.0195293165
ss61521235	*rs41618154*	11	104.556109	0.1991	2.0572	0.151941	0.0344	0.0109619554
ss86341591		11	104.599272	0.6275	5.8680	0.015673	0.0470	0.0219775743
ss86332463		11	104.621836	0.5709	11.8264	0.000619	0.0644	0.0315440537
ss86328601		11	104.643909	0.1332	8.4961	0.003673	0.0810	0.0187140046
ss86298894		11	104.66866	0.0759	3.2202	0.073168	0.0616	0.0086401066
ss86339716		11	104.735295	0.6218	21.8970	0.000003	0.0857	0.0403281796
ss86325121		11	104.824263	0.7042	2.6568	0.103562	0.0337	0.0140373443
ss86339489		11	104.850907	0.0874	5.5450	0.018811	0.0775	0.0123595383
ss86327727		11	104.891662	0.0924	3.6881	0.055209	0.0636	0.0106705162
ss86341202		11	104.930849	0.5093	4.3518	0.037334	0.0390	0.0194723667

ss86315309		11	104.979929	0.2958	2.0578	0.151877	0.0286	0.0119146292
ss86298316		11	105.014	0.6254	5.5143	0.019140	0.0442	0.0207032114
ss61521756	*rs41621855*	11	105.06944	0.0544	2.0209	0.155600	0.0568	0.0058430471
ss61521758		11	105.096731	0.9434	1.9211	0.166176	0.0545	0.0058189254
	rs29025299	11	105.137568	0.8173	0.4084	0.522993	0.0150	0.0044689512
ss61506973	*rs41601379*	11	105.164138	0.7765	3.1424	0.076719	0.0389	0.0134926542
ss86289852		11	105.234482	0.4234	0.2836	0.594539	0.0099	0.0048244920
ss86288283		11	105.275186	0.7923	3.9863	0.046261	0.0451	0.0148558221
ss86318056		11	105.308465	0.4384	6.7699	0.009468	0.0482	0.0237342910
ss86340155		11	105.336819	0.9284	6.7878	0.009374	0.0964	0.0128276665
ss46526130	*rs41255273*	11	105.373793	0.4491	4.8151	0.028542	0.0410	0.0203126233
ss86317605		11	105.482392	0.5394	4.4776	0.034696	0.0393	0.0195391209
ss86334239		11	105.534909	0.2350	4.9356	0.026630	0.0475	0.0170619801
ss86310654		11	105.56165	0.9334	3.3790	0.066456	0.0686	0.0085356065
ss86335421		11	105.605455	0.6275	2.8520	0.091709	0.0328	0.0153444474
ss86336806		11	105.630829	0.5817	0.1274	0.721210	0.0068	0.0033057724
ss86283465		11	105.667352	0.5602	0.6475	0.421278	0.0156	0.0076761486
ss86306101		11	105.747331	0.5093	12.8267	0.000365	0.0653	0.0326496993
ss86285612		11	105.860127	0.6769	9.3388	0.002329	0.0601	0.0263062038
ss86336779		11	105.880209	0.3109	19.5866	0.000011	0.0866	0.0370903800
ss86305006		11	105.905276	0.6791	9.1874	0.002527	0.0596	0.0259619085
ss86317387		11	105.959798	0.5466	1.4503	0.228897	0.0230	0.0113763091
ss86307487		11	106.007657	0.4835	0.3234	0.569755	0.0106	0.0053017592
ss46526242	*rs41255381*	11	106.0927	0.4821	4.2613	0.039361	0.0390	0.0194971771
ss86329473		11	106.132765	0.0652	3.1262	0.077481	0.0657	0.0080037351
ss86310394		11	106.208942	0.3775	0.7409	0.389668	0.0161	0.0075584400
ss86291437		11	106.233729	0.2321	9.1376	0.002596	0.0651	0.0231958335
ss86335992		11	106.265145	0.1332	0.1219	0.727121	0.0096	0.0022166886
	rs29018427	11	106.320775	0.1905	3.0544	0.080959	0.0407	0.0125461003
ss86299127		11	106.356144	0.1970	4.5648	0.032985	0.0495	0.0156717429
ss86317114		11	106.387274	0.5895	18.9704	0.000015	0.0817	0.0395393740
ss86323698		11	106.458412	0.4592	14.3986	0.000161	0.0705	0.0350220258
ss86313844		11	106.502334	0.3754	6.8860	0.008878	0.0507	0.0237781966
ss86308733		11	106.534689	0.4040	9.1289	0.002608	0.0561	0.0270371496
ss61522368	*rs41619343*	11	106.573154	0.5752	0.0428	0.836207	0.0039	0.0018889665
ss86309395		11	106.597256	0.5981	0.0003	0.985880	0.0003	0.0001645375
ss61466285	*rs41568040*	11	106.623522	0.3897	1.4230	0.233324	0.0224	0.0106520762
ss86337188		11	106.685194	0.6812	11.5577	0.000713	0.0672	0.0291851945
ss86336113		11	106.766452	0.8195	3.0815	0.079628	0.0413	0.0122114203
ss86336729		11	106.819675	0.2636	0.1776	0.673558	0.0087	0.0033897916
ss86302134		11	106.872848	0.1798	3.1768	0.075128	0.0419	0.0123655521
ss86313925		11	107.086181	0.0501	4.3768	0.036792	0.0875	0.0083391417
ss86325226		11	107.154083	0.1855	0.2988	0.584798	0.0132	0.0040025284
ss86316182		11	107.216561	0.3331	3.3096	0.069308	0.0347	0.0153949189
ss86289931		11	107.237844	0.2994	0.5309	0.466465	0.0144	0.0060422064
ss86336319		11	107.267694	0.3653	0.6998	0.403131	0.0159	0.0073591410
ss86313549		11	107.28808	0.1963	7.2453	0.007279	0.0625	0.0197165369

ss86328154		11	107.315638	0.2350	7.8260	0.005292	0.0610	0.0219471309
ss86294022		11	107.336071	0.2350	7.8260	0.005292	0.0610	0.0219471309
ss86329489		11	107.470735	0.0702	0.1090	0.741342	0.0118	0.0015443249
ss86330260		11	107.5736	0.4176	0.7891	0.374678	0.0165	0.0080040148
ss86274027		11	107.594125	0.6662	0.0001	0.992677	0.0002	0.0000792562
ss86338402		11	107.650972	0.1526	1.9631	0.161630	0.0357	0.0092399855
ss86282159		11	107.672844	0.0824	0.4056	0.524417	0.0212	0.0032123594
ss86285290		11	107.685694	0.8216	2.2943	0.130302	0.0363	0.0106518449
ss86308027		11	107.710613	0.7278	3.0820	0.079605	0.0362	0.0143334979
ss86308826		11	107.733547	0.4334	0.0156	0.900508	0.0023	0.0011332141
ss86309223		11	107.860062	0.0838	0.3980	0.528329	0.0209	0.0032122206
ss86337472		11	107.916725	0.5322	0.2762	0.599361	0.0096	0.0047562759
ss86320764		11	107.964527	0.2486	3.1759	0.075167	0.0369	0.0137869700
ss86317574		11	108.019951	0.8861	1.6882	0.194268	0.0364	0.0073408428
ss86337299		11	108.062406	0.1526	7.4526	0.006495	0.0694	0.0179408232
ss86306526		11	108.096693	0.6096	2.0281	0.154861	0.0266	0.0126576120
ss86337417		11	108.119253	0.4764	1.3561	0.244613	0.0218	0.0108773147
ss86335032		11	108.166042	0.1977	8.4850	0.003696	0.0680	0.0215806624
ss86304417		11	108.197801	0.1318	0.0177	0.894125	0.0037	0.0008374562
ss86289481		11	108.22064	0.1325	0.0008	0.977108	0.0008	0.0001834123
ss86321061		11	108.287399	0.1275	0.0048	0.944603	0.0020	0.0004346967
ss86298185		11	108.327984	0.9011	0.1131	0.736717	0.0105	0.0018750491
ss86339570		11	108.421829	0.1189	9.1661	0.002556	0.0850	0.0178175473
ss86321676		11	108.457254	0.2099	0.0741	0.785495	0.0062	0.0020491687
ss86337612		11	108.506371	0.5050	0.1207	0.728406	0.0064	0.0032058300
ss86294388		11	108.55277	0.6705	4.7955	0.028865	0.0426	0.0188157114
ss86283104		11	108.616362	0.4441	3.1333	0.077145	0.0310	0.0153083537
ss86291784		11	108.648402	0.4277	3.1724	0.075326	0.0325	0.0159241141
ss86333031		11	108.669397	0.5623	0.4053	0.524565	0.0114	0.0056090422
ss86337793		11	108.691219	0.6053	0.0067	0.934927	0.0015	0.0007380480
ss86330426		11	108.712347	0.6340	0.2473	0.619119	0.0095	0.0044161667
ss86311725		11	108.735702	0.5573	0.0991	0.752979	0.0060	0.0029567249
	rs29019310	11	108.775233	0.4943	3.2834	0.070413	0.0334	0.0167048919
ss86306277		11	108.870046	0.4413	1.6305	0.202059	0.0239	0.0118077365
ss61470666	*rs41572342*	11	108.91673	0.3073	0.4557	0.499842	0.0141	0.0059817910
ss86288841		11	108.944813	0.4255	0.3925	0.531196	0.0115	0.0056200167
ss86335165		11	108.966377	0.1819	1.4053	0.236249	0.0284	0.0084489698
ss86341598		11	109.067556	0.4212	0.3845	0.535408	0.0117	0.0057277340
ss61524656	*rs41622186*	11	109.101259	0.7034	1.2101	0.271699	0.0221	0.0092141171
ss86312235		11	109.135338	0.4642	0.0368	0.847942	0.0037	0.0018457538
ss86338486		11	109.310439	0.2923	10.4906	0.001257	0.0674	0.0278744465
ss86273705		11	109.346319	0.6218	0.6794	0.410071	0.0158	0.0074393038
ss86335623		11	109.373092	0.7600	1.1860	0.276522	0.0230	0.0083831165
ss86313119		11	109.413576	0.5867	0.0305	0.861486	0.0033	0.0015780965
ss86290265		11	109.454328	0.7672	0.4588	0.498431	0.0145	0.0051928397
ss86303107		11	109.497448	0.5809	2.2564	0.133521	0.0286	0.0139393120
ss86333598		11	109.555306	0.4950	5.9790	0.014724	0.0458	0.0229209492

ss86299253		11	109.575385	0.4728	1.1313	0.287858	0.0196	0.0097717635
ss86297906		11	109.607717	0.6497	1.7058	0.191958	0.0244	0.0111209635
ss86296376		11	109.644736	0.6719	0.7933	0.373409	0.0169	0.0074543941
ss86337667		11	109.67835	0.1769	1.7707	0.183731	0.0325	0.0094778583
ss86289166		11	109.707809	0.2536	5.2487	0.022261	0.0494	0.0186986141
ss86309217		11	109.746307	0.4542	0.0008	0.977229	0.0005	0.0002593762
ss86318536		11	109.771826	0.4226	0.4976	0.480791	0.0128	0.0062297445
ss86309770		11	109.805156	0.1110	1.3137	0.252127	0.0339	0.0066990182
ss86289596		11	109.833816	0.6956	0.2013	0.653825	0.0087	0.0036832838
ss86294777		11	109.867717	0.2056	5.5588	0.018664	0.0543	0.0177281504
ss86320135		11	109.89927	0.6354	0.7205	0.396266	0.0161	0.0074592666
ss86303943		11	109.935639	0.6605	2.2232	0.136401	0.0300	0.0134559405
ss117970295		11	109.95924	0.7772	2.4980	0.114445	0.0344	0.0119009063
ss86293949		11	110.015339	0.3961	6.2297	0.012793	0.0475	0.0227012679
ss86318250		11	110.067997	0.3281	2.4637	0.116960	0.0318	0.0140199548
ss86303633		11	110.100672	0.6841	0.4694	0.493479	0.0144	0.0062133337
ss61553639	*rs43714183*	11	110.120689	0.1282	0.5378	0.463585	0.0202	0.0045110087
ss86329495		11	110.171705	0.6024	9.1344	0.002601	0.0571	0.0273703021
ss61471622	*rs41571772*	12	0.0798	0.2980	1.5763	0.209714	0.0261	0.0109397272
ss86334294		12	0.125974	0.1053	0.8870	0.346618	0.0280	0.0052743225
ss86305079		12	0.175087	0.4642	4.4889	0.034470	0.0391	0.0194444388
ss61524030	*rs41624977*	12	0.219152	0.5924	0.1105	0.739662	0.0063	0.0030227862
ss86294821		12	0.293941	0.8739	4.8277	0.028334	0.0609	0.0134258253
ss63524819	*rs42259618*	12	0.434748	0.4119	14.1132	0.000187	0.0733	0.0355247798
ss117970526		12	0.485143	0.3682	0.2868	0.592441	0.0102	0.0047528689
ss86285936		12	0.549513	0.1827	5.5308	0.018962	0.0559	0.0166824601
ss86289062		12	0.585929	0.6504	1.6203	0.203481	0.0252	0.0114696285
ss86331777		12	0.627754	0.2994	0.9598	0.327569	0.0199	0.0083440256
ss61507825	*rs41609304*	12	0.667854	0.2629	0.1422	0.706212	0.0079	0.0030716770
ss63525884	*rs42261673*	12	0.736585	0.1547	0.0066	0.935466	0.0021	0.0005489845
ss86336657		12	0.763198	0.0974	0.7871	0.375286	0.0279	0.0049118920
ss61531044	*rs41631107*	12	0.785601	0.8990	0.4425	0.506120	0.0207	0.0037559053
ss86274920		12	0.810943	0.5351	0.0008	0.977145	0.0005	0.0002671251
ss61531045	*rs41631108*	12	0.849972	0.1325	1.5492	0.213678	0.0347	0.0079843000
ss86327864		12	0.877503	0.4255	0.0490	0.824827	0.0042	0.0020577287
ss63527021	*rs42258551*	12	0.901957	0.3782	1.2345	0.266926	0.0211	0.0099036375
ss86313149		12	0.930207	0.5294	2.1630	0.141820	0.0268	0.0133593171
ss64857808	*rs42954746*	12	0.978496	0.3023	0.1357	0.712716	0.0074	0.0031179514
ss64857822	*rs42954760*	12	0.999853	0.3016	0.0853	0.770338	0.0059	0.0024758075
ss61526396	*rs41623091*	12	1.021576	0.3037	3.5314	0.060634	0.0377	0.0159576626
	rs29015893	12	1.047841	0.8553	0.1981	0.656375	0.0121	0.0030050928
ss86338344		12	1.073869	0.5150	2.0087	0.156849	0.0259	0.0129534185
ss86308333		12	1.088262	0.8252	0.4109	0.521712	0.0155	0.0044783135
ss86330791		12	1.113231	0.3868	2.2400	0.134933	0.0280	0.0132667924
ss105241832		12	1.136171	0.0802	1.3055	0.253602	0.0401	0.0059137003
ss61566619	*rs41666547*	12	1.190123	0.2407	0.0431	0.835601	0.0045	0.0016329021
ss61495809	*rs41593475*	12	1.315665	0.5158	5.6717	0.017508	0.0439	0.0219090155

ss64283492	*rs42564989*	12	1.347706	0.4921	0.0020	0.964443	0.0008	0.0004148715
ss86294379		12	1.466662	0.4341	4.8388	0.028155	0.0402	0.0197621048
ss61513166	*rs41610553*	12	1.542501	0.9040	2.5073	0.113775	0.0504	0.0087475254
ss65119139	*rs43207519*	12	1.586156	0.3775	0.2075	0.648903	0.0089	0.0041837549
ss86306216		12	1.627266	0.7142	0.3987	0.527972	0.0128	0.0052243715
ss86282998		12	1.691975	0.3883	4.3584	0.037189	0.0406	0.0192964811
	rs29009691	12	1.842479	0.6010	2.1450	0.143491	0.0271	0.0129903255
ss61563254	*rs41660233*	12	1.890849	0.7779	1.2353	0.266766	0.0252	0.0087044882
ss61563259	*rs41660238*	12	1.935374	0.2213	1.0358	0.309144	0.0231	0.0079558479
ss63557061	*rs42268891*	12	1.965024	0.7801	0.9201	0.337787	0.0219	0.0074999362
ss63557106	*rs42268906*	12	1.994965	0.6927	2.0000	0.157742	0.0280	0.0119368839
ss105238307		12	2.062426	0.8252	3.5866	0.058662	0.0471	0.0135804875
ss63558082	*rs42272429*	12	2.102616	0.1275	0.3350	0.562946	0.0157	0.0035002822
ss117970448		12	2.14904	0.8653	0.5649	0.452540	0.0197	0.0045836782
ss63558424	*rs42273843*	12	2.18439	0.1289	0.7089	0.400113	0.0227	0.0050946768
ss86298751		12	2.243002	0.4126	0.8414	0.359323	0.0171	0.0082747621
ss63558757	*rs42274954*	12	2.292361	0.4319	5.4240	0.020147	0.0425	0.0208351172
ss63558862	*rs42274989*	12	2.313868	0.2493	0.6505	0.420200	0.0178	0.0066537262
ss61563264	*rs41660243*	12	2.3603	0.3775	5.8348	0.015969	0.0456	0.0214550595
ss86340176		12	2.425779	0.4398	0.0262	0.871508	0.0030	0.0014604456
ss61563265	*rs41660244*	12	2.453428	0.8352	0.3225	0.570309	0.0142	0.0039184436
ss64929017	*rs43020266*	12	2.494177	0.5480	1.6524	0.199063	0.0236	0.0116794139
ss86335729		12	2.536648	0.7966	0.0240	0.876884	0.0036	0.0011615507
ss61524747	*rs41623276*	12	2.566733	0.5315	0.3907	0.532121	0.0114	0.0056769633
ss64799357	*rs42899898*	12	2.599027	0.6397	6.1056	0.013714	0.0457	0.0210511980
ss64731291	*rs42824654*	12	2.654636	0.4069	0.4932	0.482739	0.0132	0.0063549122
ss64924522	*rs43022471*	12	2.709582	0.6576	0.4318	0.511325	0.0126	0.0056956438
ss86336508		12	2.73496	0.6547	0.3965	0.529101	0.0120	0.0054452542
ss65608412	*rs43686820*	12	2.81024	0.1203	5.4851	0.019461	0.0676	0.0143067656
ss65608468	*rs43687376*	12	2.881725	0.4756	0.0042	0.948078	0.0012	0.0005826705
ss61466589	*rs41571241*	12	3.091974	0.5953	0.6267	0.428821	0.0153	0.0073782899
ss65608534	*rs43688242*	12	3.092594	0.4047	0.6267	0.428821	0.0153	0.0073782899
ss64988664	*rs43081920*	12	3.122823	0.4234	3.1811	0.074931	0.0332	0.0161900304
ss64267842	*rs42558062*	12	3.162977	0.4685	3.4337	0.064304	0.0332	0.0165365166
ss86322338		12	3.201428	0.4871	4.4637	0.034979	0.0386	0.0192623372
ss65608537	*rs43688245*	12	3.351987	0.0509	1.7277	0.189144	0.0540	0.0052158153
ss65028900	*rs43119286*	12	3.468658	0.3381	4.3454	0.037473	0.0402	0.0179722033
ss65609084	*rs43692192*	12	3.503337	0.1748	5.8487	0.015845	0.0584	0.0168455210
ss65608949	*rs43691257*	12	3.525231	0.8367	8.0318	0.004729	0.0703	0.0192019716
ss65608785	*rs43689893*	12	3.557188	0.1039	0.0019	0.965092	0.0013	0.0002492426
ss86296318		12	3.581207	0.2679	2.4737	0.116218	0.0328	0.0128564130
ss105241857		12	3.860852	0.0766	0.6674	0.414255	0.0280	0.0039652635
ss61465740	*rs41568984*	12	4.336751	0.8725	0.5650	0.452518	0.0208	0.0046302130
ss86339942		12	4.496827	0.2951	0.2011	0.653954	0.0091	0.0037700382
ss61507324	*rs41604719*	12	4.525473	0.1146	3.7490	0.053243	0.0572	0.0116079686
ss61466115		12	4.561877	0.4033	2.8524	0.091683	0.0317	0.0152359999
ss61506841	*rs41600051*	12	4.829886	0.8381	3.4048	0.065432	0.0454	0.0123275580

ss61471407	*rs41570076*	12	5.084953	0.2249	0.0113	0.915419	0.0023	0.0008105446
ss61563420	*rs41660893*	12	5.232196	0.6891	0.6571	0.417868	0.0159	0.0068184956
ss86325158		12	5.2563	0.3223	4.9325	0.026678	0.0451	0.0197024208
ss117970488		12	5.379917	0.5888	1.8569	0.173427	0.0255	0.0123514030
ss61466545	*rs41570298*	12	5.442115	0.2414	26.2429	0.000000	0.1058	0.0387497953
ss86302450		12	5.552118	0.5043	0.1526	0.696178	0.0069	0.0034482340
	rs29023094	12	5.587559	0.9377	1.6931	0.193622	0.0512	0.0059803760
ss86292017		12	5.638933	0.3259	4.4596	0.035061	0.0414	0.0181987161
ss105310518		12	5.665748	0.4269	1.8526	0.173924	0.0253	0.0124011696
ss61525742	*rs41622445*	12	5.698639	0.4362	1.5730	0.210194	0.0233	0.0114755189
ss105242566		12	5.739365	0.6748	0.1378	0.710627	0.0073	0.0031963548
ss61507190	*rs41603589*	12	5.763565	0.7851	0.1008	0.750969	0.0069	0.0023229777
ss61518684	*rs41617236*	12	6.281991	0.4434	15.5848	0.000087	0.0706	0.0348585558
ss61526499	*rs43743887*	12	6.351822	0.4069	3.0558	0.080888	0.0339	0.0163544704
ss61479058	*rs41579925*	12	6.387723	0.3410	1.5120	0.219248	0.0243	0.0109059171
ss117970445		12	6.562097	0.1590	10.3092	0.001385	0.0806	0.0215501094
ss61519129	*rs41611182*	12	6.721763	0.2844	0.4618	0.497010	0.0137	0.0055610649
ss61519137	*rs41611190*	12	6.821993	0.4477	0.0500	0.823063	0.0041	0.0020366854
	rs29021206	12	7.24085	0.6282	0.0008	0.977808	0.0005	0.0002484288
ss61527914	*rs41627855*	12	7.332443	0.7199	9.2425	0.002453	0.0621	0.0250457057
ss105306065		12	7.432952	0.5007	8.0034	0.004803	0.0519	0.0259608308
ss61528399	*rs41625444*	12	7.59651	0.6468	0.8825	0.347836	0.0183	0.0083490438
ss64981175	*rs43073761*	12	7.688402	0.6612	1.8181	0.177980	0.0253	0.0113464881
ss65609381	*rs43686689*	12	7.739106	0.9054	0.1399	0.708467	0.0110	0.0018912553
ss65609424	*rs43687532*	12	7.777259	0.8438	6.2685	0.012518	0.0632	0.0166512917
ss65609443	*rs43687551*	12	7.868772	0.4957	0.6018	0.438156	0.0138	0.0069144944
ss65234038	*rs43316446*	12	7.955088	0.3997	4.6937	0.030612	0.0412	0.0197767187
ss65234031	*rs43316439*	12	7.979157	0.6297	2.0412	0.153541	0.0269	0.0125591398
ss86340630		12	8.033488	0.4642	4.1681	0.041570	0.0369	0.0183423742
ss64667324	*rs42769648*	12	8.080803	0.1984	7.0222	0.008233	0.0622	0.0197795209
ss61495012	*rs41592495*	12	8.165769	0.1633	0.0026	0.959499	0.0012	0.0003318523
ss86281236		12	8.199278	0.4850	4.6694	0.031044	0.0399	0.0199529103
ss86337038		12	8.353158	0.1819	0.4116	0.521380	0.0153	0.0045427199
ss61545183	*rs41641537*	12	8.378704	0.8009	0.0063	0.936552	0.0019	0.0006019751
ss86287414		12	8.427468	0.7887	7.0274	0.008210	0.0606	0.0202135191
ss64683613	*rs42782194*	12	8.475144	0.6576	0.0641	0.800202	0.0048	0.0021544217
	rs29018744	12	8.51992	0.0831	6.7853	0.009387	0.0856	0.0130434680
ss86296978		12	8.549944	0.8754	5.4767	0.019554	0.0650	0.0141875151
ss61477797		12	8.704036	0.9119	0.6439	0.422586	0.0268	0.0043133002
ss86336833		12	8.769691	0.8216	1.6839	0.194839	0.0305	0.0089319870
ss86290347		12	8.805327	0.6089	0.2774	0.598558	0.0097	0.0046014993
	rs29016195	12	8.8446	0.4205	1.5584	0.212319	0.0235	0.0114669261
ss63515600	*rs42260066*	12	8.917627	0.4491	11.3393	0.000801	0.0621	0.0307492284
ss86338229		12	8.943443	0.4656	0.0117	0.913957	0.0020	0.0009733721
ss86335485		12	8.964367	0.8926	0.2644	0.607274	0.0152	0.0029108455
ss61466642	*rs41571294*	12	8.992167	0.2837	3.8358	0.050569	0.0403	0.0163719616
ss86322512		12	9.018675	0.2572	5.0379	0.025111	0.0479	0.0183062256

ss61528995	*rs41631006*	12	9.045183	0.6117	5.9184	0.015235	0.0456	0.0216823614
ss86332348		12	9.074901	0.2314	0.5642	0.452822	0.0168	0.0059712716
ss86340521		12	9.187315	0.2872	0.1169	0.732490	0.0071	0.0028965760
ss64423634	*rs42628877*	12	9.231483	0.1175	0.0493	0.824315	0.0065	0.0013511910
ss86319413		12	9.27802	0.4370	4.0378	0.044877	0.0369	0.0181343828
ss86319300		12	9.311551	0.7615	1.4522	0.228583	0.0266	0.0096598386
ss86333927		12	9.340069	0.6203	8.8815	0.002981	0.0573	0.0270072711
ss86337659		12	9.374947	0.3023	4.3821	0.036681	0.0428	0.0180669124
ss63516614	*rs42261504*	12	9.424267	0.3281	0.0169	0.896589	0.0025	0.0010873606
ss117970615		12	9.47589	0.2951	3.8119	0.051290	0.0399	0.0165954356
ss86337750		12	9.49649	0.2830	8.5364	0.003594	0.0611	0.0248028943
ss86285967		12	9.555094	0.1511	3.4729	0.062804	0.0502	0.0128807295
ss61522219	*rs41617704*	12	9.611514	0.4785	15.8673	0.000075	0.0735	0.0366924052
	rs29018848	12	9.713146	0.4463	0.5800	0.446568	0.0139	0.0068683466
ss86313280		12	9.787809	0.2120	0.1117	0.738315	0.0075	0.0024952970
ss86317609		12	9.830243	0.1239	2.0828	0.149415	0.0414	0.0089814070
ss86301711		12	9.961433	0.4986	0.9718	0.324577	0.0186	0.0093038963
ss65609641	*rs43689240*	12	10.022121	0.6633	0.2544	0.614187	0.0101	0.0044914810
ss65610092	*rs43692387*	12	10.051337	0.7794	0.6221	0.430531	0.0176	0.0060537545
ss61508028	*rs41600615*	12	10.079285	0.3080	2.7141	0.099913	0.0327	0.0139420856
ss61479724	*rs41581784*	12	10.10716	0.9377	0.5108	0.475012	0.0274	0.0032038854
ss65610158	*rs43693253*	12	10.127486	0.9499	6.0897	0.013837	0.1046	0.0099661281
ss86338430		12	10.16153	0.9298	0.2675	0.605179	0.0187	0.0024427354
ss86285418		12	10.264902	0.1340	0.8247	0.364115	0.0251	0.0058233388
ss86324552		12	10.287194	0.1576	0.2223	0.637405	0.0118	0.0031378498
ss61533186		12	10.312657	0.2307	16.5164	0.000054	0.0866	0.0307478499
ss117970641		12	10.343017	0.1734	0.5506	0.458337	0.0175	0.0050258992
ss61533188	*rs41630754*	12	10.36402	0.8883	0.6338	0.426234	0.0243	0.0048220910
ss86340914		12	10.388747	0.2715	0.1147	0.734917	0.0071	0.0028209015
ss117964243		12	10.436001	0.7278	0.2393	0.624847	0.0103	0.0040813875
ss117964241		12	10.549086	0.7665	1.1321	0.287692	0.0229	0.0081888319
ss61533193	*rs41630759*	12	10.57107	0.5587	7.1189	0.007805	0.0477	0.0235269445
ss61479754	*rs41581813*	12	10.60336	0.1898	4.0220	0.045298	0.0479	0.0147440366
	rs29012667	12	10.651937	0.4585	16.0017	0.000070	0.0720	0.0357460457
ss64332811	*rs42583682*	12	10.747118	0.8768	0.1731	0.677538	0.0117	0.0025317160
ss86309780		12	10.809728	0.8001	0.2846	0.593871	0.0124	0.0039673165
ss117975273		12	10.887076	0.8832	9.7168	0.001901	0.0878	0.0181139168
ss86330770		12	10.925265	0.6297	0.4246	0.514865	0.0122	0.0056692193
ss86339940		12	11.153571	0.5358	0.1082	0.742335	0.0063	0.0031119721
ss61479107	*rs41580374*	12	11.187495	0.8145	3.9233	0.048014	0.0458	0.0138382529
ss86285065		12	11.219379	0.8023	0.0147	0.903382	0.0028	0.0009004595
ss86339554		12	11.263463	0.4176	0.1391	0.709257	0.0069	0.0033757413
ss61532455	*rs41625869*	12	11.318068	0.8281	0.5451	0.460585	0.0176	0.0049985719
ss86326173		12	11.370359	0.0673	13.8529	0.000214	0.1355	0.0170144966
ss117970508		12	11.455916	0.8503	4.0533	0.044471	0.0506	0.0128807478
ss86295570		12	11.485923	0.1626	4.4177	0.035928	0.0511	0.0139237366
ss86298514		12	11.510628	0.7350	0.3068	0.579801	0.0112	0.0043617400

ss61530943	*rs41630106*	12	11.550585	0.2715	1.2073	0.272246	0.0227	0.0089881805
ss86301947		12	11.708607	0.6067	0.0014	0.970045	0.0007	0.0003461634
ss61533198	*rs41630764*	12	11.872291	0.8968	8.7635	0.003178	0.0916	0.0169504135
ss61533196	*rs41630762*	12	11.911041	0.4219	5.3551	0.020952	0.0434	0.0211927420
ss86337506		12	12.007944	0.2056	0.0157	0.900443	0.0029	0.0009348434
ss61524830	*rs41623859*	12	12.060417	0.1669	0.0601	0.806427	0.0060	0.0016735177
ss61514994	*rs41611457*	12	12.269459	0.5960	0.4939	0.482409	0.0134	0.0064744931
ss86316022		12	12.312888	0.2830	0.6942	0.405018	0.0175	0.0071140040
ss117970644		12	12.358816	0.8245	0.1788	0.672521	0.0104	0.0030197244
	rs29014412	12	12.402597	0.0860	1.2925	0.255984	0.0381	0.0059796951
ss105237575		12	12.424988	0.0824	2.3742	0.123811	0.0509	0.0076958122
ss117970613		12	12.551342	0.2665	0.7946	0.373034	0.0190	0.0074435919
ss86339828		12	12.579529	0.8216	0.0386	0.844263	0.0047	0.0013908157
ss86317551		12	12.6589	0.0587	0.1483	0.700238	0.0153	0.0016973216
ss86302343		12	12.700606	0.6397	3.1706	0.075413	0.0349	0.0160895268
ss105237581		12	12.723234	0.1074	0.0122	0.911913	0.0034	0.0006457309
ss105237585		12	12.770408	0.8703	0.9529	0.329312	0.0264	0.0059656606
ss61568052	*rs41661358*	12	12.828921	0.1963	0.7412	0.389575	0.0195	0.0061611466
ss117970601		12	12.888548	0.9234	0.4173	0.518500	0.0231	0.0032667228
ss63991027	*rs42419976*	12	12.914843	0.2013	0.9590	0.327789	0.0223	0.0071776792
ss86276534		12	12.940297	0.5444	2.6387	0.104742	0.0304	0.0150793204
ss61526577	*rs41624564*	12	12.96593	0.0938	5.5537	0.018718	0.0736	0.0125116158
ss64348539	*rs42602078*	12	12.992685	0.1855	1.0555	0.304596	0.0240	0.0072591515
ss86325333		12	13.063656	0.0802	0.8614	0.353683	0.0313	0.0046175574
ss86309980		12	13.118937	0.4226	0.3716	0.542329	0.0116	0.0056754855
ss64352161	*rs42602465*	12	13.227812	0.0874	0.6540	0.418952	0.0261	0.0041689903
ss64352211	*rs42602490*	12	13.27543	0.1705	0.2432	0.622057	0.0120	0.0033958714
ss86305089		12	13.401683	0.0573	3.9996	0.045901	0.0753	0.0081317066
ss61484368	*rs41578221*	12	13.509341	0.6748	0.1473	0.701234	0.0077	0.0033671313
ss65238321	*rs43323110*	12	13.538105	0.4828	0.0005	0.981401	0.0004	0.0002174254
ss86336289		12	13.658189	0.3360	1.7352	0.188181	0.0257	0.0114603706
ss61479793	*rs41582552*	12	13.820727	0.7063	0.3433	0.558124	0.0121	0.0050141425
ss105237589		12	13.882012	0.7178	1.2900	0.256435	0.0234	0.0094747410
ss86321889		12	13.915348	0.7407	0.6036	0.437463	0.0163	0.0062614264
ss86302730		12	13.989703	0.2156	1.0151	0.314024	0.0228	0.0077176586
ss86299100		12	14.063485	0.8718	0.1519	0.696856	0.0106	0.0023765690
ss61520908	*rs41618427*	12	14.093517	0.1010	0.8889	0.346099	0.0293	0.0053266529
ss86326842		12	14.21323	0.6175	0.2320	0.630206	0.0091	0.0043035370
ss117970430		12	14.28795	0.1067	0.4362	0.509202	0.0198	0.0037831994
ss86325309		12	14.348175	0.1862	1.8816	0.170589	0.0318	0.0096301916
ss61533207	*rs41630773*	12	14.372818	0.5781	0.0731	0.786985	0.0050	0.0024555961
ss61533206	*rs41630772*	12	14.383705	0.2400	3.7639	0.052773	0.0412	0.0150171158
ss117970636		12	14.405372	0.2364	2.9679	0.085379	0.0366	0.0132124075
ss86286540		12	14.431744	0.2221	2.0923	0.148489	0.0322	0.0111240416
ss86318595		12	14.511476	0.5802	0.1510	0.697711	0.0072	0.0035150274
ss86279588		12	14.556717	0.6196	1.4960	0.221703	0.0228	0.0107556148
ss86331618		12	14.65884	0.3073	2.5866	0.108226	0.0332	0.0141180810

ss86325093		12	14.685867	0.7994	1.8242	0.177253	0.0315	0.0100933460
ss86326713		12	14.748724	0.8080	0.4655	0.495303	0.0157	0.0048737312
ss86310060		12	14.768765	0.2887	12.5069	0.000432	0.0722	0.0296465515
ss86305388		12	14.796692	0.2170	8.0329	0.004727	0.0630	0.0214138909
ss86294985		12	14.873556	0.5895	4.0796	0.043787	0.0366	0.0177183973
ss61533279		12	14.906545	0.1254	12.4243	0.000452	0.0967	0.0211957877
ss86289636		12	15.007249	0.4506	6.7672	0.009482	0.0487	0.0241189892
ss86285773		12	15.039762	0.5924	5.7987	0.016297	0.0449	0.0216856890
ss105237607		12	15.070194	0.4907	1.0827	0.298459	0.0194	0.0096773685
	rs29023722	12	15.139028	0.4577	3.2331	0.072599	0.0336	0.0166895969
ss86322090		12	15.190062	0.3746	0.0463	0.829677	0.0039	0.0018458885
ss46526670	*rs41257125*	12	15.227602	0.7593	1.6035	0.205826	0.0268	0.0098059888
ss86294103		12	15.271918	0.1870	0.4823	0.487603	0.0165	0.0050220180
ss61533276	*rs41631638*	12	15.298034	0.4370	0.0286	0.865709	0.0031	0.0015319701
ss86304985		12	15.318457	0.1977	0.3604	0.548491	0.0142	0.0044929918
ss86314727		12	15.377225	0.1948	2.0189	0.155804	0.0331	0.0103844863
ss61533278	*rs41631640*	12	15.413522	0.1182	0.1858	0.666596	0.0125	0.0025990240
ss65610551	*rs43690647*	12	15.4936	0.2529	4.1386	0.042295	0.0426	0.0160978701
ss86320994		12	15.522034	0.1168	0.2814	0.595971	0.0148	0.0030620402
ss86337714		12	15.561929	0.5430	0.0000	0.998684	0.0000	0.0000147416
ss61479812	*rs41582571*	12	15.582075	0.0788	0.8620	0.353512	0.0321	0.0046656618
ss65610767	*rs43692963*	12	15.640853	0.8374	0.0012	0.972317	0.0009	0.0002408045
ss86337787		12	15.674655	0.6862	0.3196	0.572005	0.0112	0.0048177657
ss61479813	*rs41582572*	12	15.708559	0.6117	1.4915	0.222397	0.0228	0.0108425018
ss86320952		12	15.73249	0.3524	3.1475	0.076480	0.0336	0.0153448938
ss61533292	*rs41631654*	12	15.862554	0.6411	2.5516	0.110636	0.0296	0.0136333018
ss61533293	*rs41631655*	12	16.03489	0.2213	3.0028	0.083562	0.0379	0.0130715451
ss86336210		12	16.120571	0.8682	0.0001	0.992079	0.0003	0.0000630920
ss86335587		12	16.149467	0.2579	5.4379	0.019989	0.0486	0.0186139764
ss65611486	*rs43690604*	12	16.195741	0.8782	0.8551	0.355428	0.0265	0.0056615046
ss105238287		12	16.246523	0.5874	0.6408	0.423692	0.0148	0.0071550120
ss86328226		12	16.279671	0.3474	10.1002	0.001548	0.0593	0.0269023659
ss65611569	*rs43691687*	12	16.563156	0.6340	0.1133	0.736476	0.0067	0.0031137003
ss61473202	*rs41567621*	12	16.71605	0.3789	0.3438	0.557832	0.0116	0.0054496749
	rs29015935	12	16.810705	0.1762	0.1244	0.724417	0.0086	0.0024884979
ss86336852		12	17.095048	0.4563	0.6573	0.417784	0.0146	0.0072485802
ss86325874		12	17.419047	0.7056	0.0085	0.926416	0.0018	0.0007605093
ss86327632		12	17.444464	0.5559	2.3326	0.127142	0.0282	0.0139347045
ss61527913	*rs41627854*	12	17.507946	0.3023	0.6117	0.434400	0.0151	0.0063802560
ss61525279	*rs41619308*	12	17.591431	0.1841	0.3276	0.567254	0.0134	0.0040392765
ss86284194		12	17.783235	0.1748	0.1628	0.686716	0.0097	0.0027993904
ss61533306	*rs41631668*	12	17.837259	0.7744	0.2519	0.615893	0.0111	0.0038938650
ss86296716		12	17.886608	0.5766	0.1712	0.679186	0.0076	0.0037150327
ss86297164		12	18.041284	0.2708	2.9591	0.085840	0.0353	0.0139254464
ss86301296		12	18.162095	0.3245	6.8470	0.009072	0.0506	0.0221736299
	rs29026795	12	18.354672	0.8467	1.1123	0.291939	0.0267	0.0069393801
ss86292673		12	18.460882	0.3639	14.2866	0.000170	0.0703	0.0325645668

ss86341088		12	18.536147	0.3854	0.8990	0.343386	0.0178	0.0084157255
ss61476982	*rs41580230*	12	18.627949	0.1254	1.7376	0.187879	0.0376	0.0082538007
ss61562659	*rs41663955*	12	18.697864	0.0731	5.6464	0.017760	0.0843	0.0114187373
ss61533295	*rs41631657*	12	18.724365	0.1232	1.7483	0.186523	0.0382	0.0082574720
ss61479828		12	18.754656	0.8288	0.9265	0.336116	0.0234	0.0066422061
ss86340668		12	19.014662	0.2593	3.9165	0.048207	0.0419	0.0161031262
ss86300929		12	19.153349	0.1497	10.7344	0.001104	0.0835	0.0212469032
ss61470034	*rs41567031*	12	19.236727	0.4090	3.5405	0.060304	0.0352	0.0170296280
ss61479835	*rs41582594*	12	19.345305	0.5688	0.9968	0.318443	0.0185	0.0090584853
ss61523062	*rs41623528*	12	19.418216	0.7514	1.9924	0.158534	0.0301	0.0112354936
ss86300549		12	19.469	0.6153	0.7770	0.378371	0.0166	0.0078799100
ss117975189		12	19.542919	0.2049	1.7558	0.185581	0.0307	0.0100076328
ss86334432		12	19.690024	0.8804	5.6462	0.017762	0.0691	0.0145518704
ss61566001	*rs41659648*	12	19.821835	0.5537	1.4114	0.235221	0.0228	0.0112601616
ss86338757		12	19.855312	0.4678	0.9240	0.336751	0.0179	0.0089241038
ss86310298		12	19.93166	0.4835	1.5846	0.208521	0.0233	0.0116199556
ss86292464		12	19.991313	0.3603	14.3195	0.000168	0.0718	0.0331047758
ss86321997		12	20.145604	0.3854	13.9047	0.000208	0.0695	0.0329282075
ss86340258		12	20.276551	0.2142	11.6586	0.000676	0.0775	0.0260874860
ss86336255		12	20.468378	0.2722	0.9602	0.327485	0.0199	0.0078824675
ss86328557		12	20.498477	0.7450	1.5273	0.216932	0.0257	0.0097575988
ss86336673		12	20.552383	0.3252	1.2692	0.260302	0.0226	0.0099101615
ss86292765		12	20.675711	0.4312	0.4015	0.526530	0.0120	0.0059016492
[truncated: 512,315 more chars]
